# Global, regional, and national incidence and mortality burden of non-COVID-19 lower respiratory infections and aetiologies, 1990–2021: a systematic analysis from the Global Burden of Disease Study 2021

**DOI:** 10.1016/S1473-3099(24)00176-2

**Published:** 2024-09

**Authors:** GBD 2021 Lower Respiratory Infections and Antimicrobial Resistance Collaborators, Rose Grace Bender, Sarah Brooke Sirota, Lucien R Swetschinski, Regina-Mae Villanueva Dominguez, Amanda Novotney, Eve E Wool, Kevin S Ikuta, Avina Vongpradith, Emma Lynn Best Rogowski, Matthew Doxey, Christopher E Troeger, Samuel B Albertson, Jianing Ma, Jiawei He, Kelsey Lynn Maass, Eric A.F.Simões, Meriem Abdoun, Jeza Muhamad Abdul Aziz, Deldar Morad Abdulah, Samir Abu Rumeileh, Hasan Abualruz, Salahdein Aburuz, Abiola Victor Adepoju, Rishan Adha, Wirawan Adikusuma, Saryia Adra, Ali Afraz, Shahin Aghamiri, Antonella Agodi, Amir Mahmoud Ahmadzade, Haroon Ahmed, Ayman Ahmed, Karolina Akinosoglou, Tareq Mohammed Ali AL-Ahdal, Rasmieh Mustafa Al-amer, Mohammed Albashtawy, Mohammad T. AlBataineh, Hediyeh Alemi, Adel Ali Saeed Al-Gheethi, Abid Ali, Syed Shujait Shujait Ali, Jaber S Alqahtani, Mohammad AlQudah, Jaffar A. Al-Tawfiq, Yaser Mohammed Al-Worafi, Karem H Alzoubi, Reza Amani, Prince M Amegbor, Edward Kwabena Ameyaw, John H Amuasi, Abhishek Anil, Philip Emeka Anyanwu, Mosab Arafat, Damelash Areda, Reza Arefnezhad, Kendalem Asmare Atalell, Firayad Ayele, Ahmed Y Azzam, Hassan Babamohamadi, François-Xavier Babin, Yogesh Bahurupi, Stephen Baker, Biswajit Banik, Martina Barchitta, Hiba Jawdat Barqawi, Zarrin Basharat, Pritish Baskaran, Kavita Batra, Ravi Batra, Nebiyou Simegnew Bayileyegn, Apostolos Beloukas, James A Berkley, Kebede A Beyene, Ashish Bhargava, Priyadarshini Bhattacharjee, Julia A Bielicki, Mariah Malak Bilalaga, Veera R Bitra, Colin Stewart Brown, Katrin Burkart, Yasser Bustanji, Sinclair Carr, Yaacoub Chahine, Vijay Kumar Chattu, Fatemeh Chichagi, Hitesh Chopra, Isaac Sunday Chukwu, Eunice Chung, Sriharsha Dadana, Xiaochen Dai, Lalit Dandona, Rakhi Dandona, Isaac Darban, Nihar Ranjan Dash, Mohsen Dashti, Mohadese Dashtkoohi, Denise Myriam Dekker, Ivan Delgado-Enciso, Vinoth Gnana Chellaiyan Devanbu, Kuldeep Dhama, Nancy Diao, Thao Huynh Phuong Do, Klara Georgieva Dokova, Christiane Dolecek, Arkadiusz Marian Dziedzic, Tim Eckmanns, Abdelaziz Ed-Dra, Ferry Efendi, Aziz Eftekharimehrabad, David William Eyre, Ayesha Fahim, Alireza Feizkhah, Timothy William Felton, Nuno Ferreira, Luisa S Flor, Santosh Gaihre, Miglas W Gebregergis, Mesfin Gebrehiwot, Christine Geffers, Urge Gerema, Kazem Ghaffari, Mohamad Goldust, Pouya Goleij, Shi-Yang Guan, Mesay Dechasa Gudeta, Cui Guo, Veer Bala Gupta, Ishita Gupta, Farrokh Habibzadeh, Najah R Hadi, Emily Haeuser, Wase Benti Hailu, Ramtin Hajibeygi, Arvin Haj-Mirzaian, Sebastian Haller, Mohammad Hamiduzzaman, Nasrin Hanifi, Jan Hansel, Md Saquib Hasnain, Johannes Haubold, Nguyen Quoc Hoan, Hong-Han Huynh, Kenneth Chukwuemeka Iregbu, Md. Rabiul Islam, Abdollah Jafarzadeh, Ammar Abdulrahman Jairoun, Mahsa Jalili, Nabi Jomehzadeh, Charity Ehimwenma Joshua, Md. Awal Kabir, Zul Kamal, Kehinde Kazeem Kanmodi, Rami S. Kantar, Arman Karimi Behnagh, Navjot Kaur, Harkiran Kaur, Faham Khamesipour, M Nuruzzaman Khan, Mahammed Ziauddin Khan suheb, Vishnu Khanal, Khaled Khatab, Mahalaqua Nazli Khatib, Grace Kim, Kwanghyun Kim, Aiggan Tamene Tamene Kitila, Somayeh Komaki, Kewal Krishan, Ralf Krumkamp, Md Abdul Kuddus, Maria Dyah Kurniasari, Chandrakant Lahariya, Kaveh Latifinaibin, Nhi Huu Hanh Le, Thao Thi Thu Le, Trang Diep Thanh Le, Seung Won Lee, Alain LEPAPE, Temesgen L. Lerango, Ming-Chieh Li, Amir Ali Mahboobipour, Kashish Malhotra, Tauqeer Hussain Mallhi, Anand Manoharan, Bernardo Alfonso Martinez-Guerra, Alexander G. Mathioudakis, Rita Mattiello, Jürgen May, Barney McManigal, Steven M McPhail, Tesfahun Mekene Meto, Max Alberto Mendez Mendez-Lopez, Sultan Ayoub Meo, Mohsen Merati, Tomislav Mestrovic, Laurette Mhlanga, Le Huu Nhat Minh, Awoke Misganaw, Vinaytosh Mishra, Arup Kumar Misra, Nouh Saad Mohamed, Esmaeil Mohammadi, Mesud Mohammed, Mustapha Mohammed, Ali H Mokdad, Lorenzo Monasta, Catrin E Moore, Rohith Motappa, Vincent Mougin, Parsa Mousavi, Francesk Mulita, Atsedemariam Andualem Mulu, Pirouz Naghavi, Ganesh R Naik, Firzan Nainu, Tapas Sadasivan Nair, Shumaila Nargus, Mohammad Negaresh, Hau Thi Hien Nguyen, Dang H Nguyen, Van Thanh Nguyen, Taxiarchis Konstantinos Nikolouzakis, Efaq Ali Noman, Chisom Adaobi Nri-Ezedi, Ismail A. Odetokun, Patrick Godwin Okwute, Matifan Dereje Olana, Titilope O Olanipekun, Omotola O. Olasupo, Antonio Olivas-Martinez, Michal Ordak, Edgar Ortiz-Brizuela, Amel Ouyahia, Jagadish Rao Padubidri, Anton Pak, Anamika Pandey, Ioannis Pantazopoulos, Pragyan Paramita Parija, Romil R Parikh, Seoyeon Park, Ashwaghosha Parthasarathi, Ava Pashaei, Prince Peprah, Hoang Tran Pham, Dimitri Poddighe, Andrew Pollard, Alfredo Ponce-De-Leon, Peralam Yegneswaran Prakash, Elton Junio Sady Prates, Nguyen Khoi Quan, Pourya Raee, Fakher Rahim, Mosiur Rahman, Masoud Rahmati, Shakthi Kumaran Ramasamy, Shubham Ranjan, Indu Ramachandra Rao, Ahmed Mustafa Rashid, Sayaphet Rattanavong, Nakul Ravikumar, Murali Mohan Rama Krishna Reddy, Elrashdy Moustafa Mohamed Redwan, Robert C Reiner, Luis Felipe Reyes, Tamalee Roberts, Mónica Rodrigues, Victor Daniel Rosenthal, Priyanka Roy, Tilleye Runghien, Umar Saeed, Amene Saghazadeh, Narjes Saheb Sharif-Askari, Fatemeh Saheb Sharif-Askari, Soumya Swaroop Sahoo, Monalisha Sahu, Joseph W Sakshaug, Afeez Abolarinwa Salami, Mohamed A. Saleh, Hossein Salehi omran, Malik Sallam, Sara Samadzadeh, Yoseph Leonardo Samodra, Rama Krishna Sanjeev, Made Ary Sarasmita, Aswini Saravanan, Benn Sartorius, Jennifer Saulam, Austin E Schumacher, Seyed Arsalan Seyedi, Mahan Shafie, Samiah Shahid, Sunder Sham, Muhammad Aaqib Shamim, Mohammad Ali Shamshirgaran, Rajesh P. Shastry, Samendra P Sherchan, Desalegn Shiferaw, Aminu Shittu, Emmanuel Edwar Siddig, Robert Sinto, Aayushi Sood, Reed J D Sorensen, Andy Stergachis, Temenuga Zhekova Stoeva, Chandan Kumar Swain, Lukasz Szarpak, Jacques Lukenze Tamuzi, Mohamad-Hani Temsah, Melkamu B Tessema Tessema, Pugazhenthan Thangaraju, Nghia Minh Tran, Ngoc-Ha Tran, Munkhtuya Tumurkhuu, Sree Sudha Ty, Aniefiok John Udoakang, Inam Ulhaq, Tungki Pratama Umar, Abdurezak Adem Umer, Seyed Mohammad Vahabi, Asokan Govindaraj Vaithinathan, Jef Van den Eynde, Judd L Walson, Muhammad Waqas, Yuhan Xing, Mukesh Kumar Yadav, Galal Yahya, Dong Keon Yon, Abed Zahedi Bialvaei, Fathiah Zakham, Abyalew Mamuye Zeleke, Chunxia Zhai, Zhaofeng Zhang, Haijun Zhang, Magdalena Zielińska, Peng Zheng, Aleksandr Y Aravkin, Theo Vos, Simon I Hay, Jonathan F. Mosser, Stephen S Lim, Mohsen Naghavi, Christopher J L Murray, Hmwe Hmwe Kyu

## Abstract

**Background:**

Lower respiratory infections (LRIs) are a major global contributor to morbidity and mortality. In 2020–21, non-pharmaceutical interventions associated with the COVID-19 pandemic reduced not only the transmission of SARS-CoV-2, but also the transmission of other LRI pathogens. Tracking LRI incidence and mortality, as well as the pathogens responsible, can guide health-system responses and funding priorities to reduce future burden. We present estimates from the Global Burden of Diseases, Injuries, and Risk Factors Study (GBD) 2021 of the burden of non-COVID-19 LRIs and corresponding aetiologies from 1990 to 2021, inclusive of pandemic effects on the incidence and mortality of select respiratory viruses, globally, regionally, and for 204 countries and territories.

**Methods:**

We estimated mortality, incidence, and aetiology attribution for LRI, defined by the GBD as pneumonia or bronchiolitis, not inclusive of COVID-19. We analysed 26 259 site-years of mortality data using the Cause of Death Ensemble model to estimate LRI mortality rates. We analysed all available age-specific and sex-specific data sources, including published literature identified by a systematic review, as well as household surveys, hospital admissions, health insurance claims, and LRI mortality estimates, to generate internally consistent estimates of incidence and prevalence using DisMod-MR 2.1. For aetiology estimation, we analysed multiple causes of death, vital registration, hospital discharge, microbial laboratory, and literature data using a network analysis model to produce the proportion of LRI deaths and episodes attributable to the following pathogens: *Acinetobacter baumannii, Chlamydia* spp, *Enterobacter* spp, *Escherichia coli, fungi, group B streptococcus, Haemophilus influenzae, influenza viruses, Klebsiella pneumoniae, Legionella* spp, *Mycoplasma spp, polymicrobial infections, Pseudomonas aeruginosa*, respiratory syncytial virus (RSV), *Staphylococcus aureus, Streptococcus pneumoniae*, and other viruses (ie, the aggregate of all viruses studied except influenza and RSV), as well as a residual category of other bacterial pathogens.

**Findings:**

Globally, in 2021, we estimated 344 million (95% uncertainty interval [UI] 325–364) incident episodes of LRI, or 4350 episodes (4120–4610) per 100 000 population, and 2·18 million deaths (1·98–2·36), or 27·7 deaths (25·1–29·9) per 100 000. 502 000 deaths (406 000–611 000) were in children younger than 5 years, among which 254 000 deaths (197 000–320 000) occurred in countries with a low Socio-demographic Index. Of the 18 modelled pathogen categories in 2021, *S pneumoniae* was responsible for the highest proportions of LRI episodes and deaths, with an estimated 97·9 million (92·1–104·0) episodes and 505 000 deaths (454 000–555 000) globally. The pathogens responsible for the second and third highest episode counts globally were other viral aetiologies (46·4 million [43·6–49·3] episodes) and *Mycoplasma* spp (25·3 million [23·5–27·2]), while those responsible for the second and third highest death counts were *S aureus* (424 000 [380 000–459 000]) and *K pneumoniae* (176 000 [158 000–194 000]). From 1990 to 2019, the global all-age non-COVID-19 LRI mortality rate declined by 41·7% (35·9–46·9), from 56·5 deaths (51·3–61·9) to 32·9 deaths (29·9–35·4) per 100 000. From 2019 to 2021, during the COVID-19 pandemic and implementation of associated non-pharmaceutical interventions, we estimated a 16·0% (13·1–18·6) decline in the global all-age non-COVID-19 LRI mortality rate, largely accounted for by a 71·8% (63·8–78·9) decline in the number of influenza deaths and a 66·7% (56·6–75·3) decline in the number of RSV deaths.

**Interpretation:**

Substantial progress has been made in reducing LRI mortality, but the burden remains high, especially in low-income and middle-income countries. During the COVID-19 pandemic, with its associated non-pharmaceutical interventions, global incident LRI cases and mortality attributable to influenza and RSV declined substantially. Expanding access to health-care services and vaccines, including *S pneumoniae, H influenzae* type B, and novel RSV vaccines, along with new low-cost interventions against *S aureus*, could mitigate the LRI burden and prevent transmission of LRI-causing pathogens.

**Funding:**

Bill & Melinda Gates Foundation, Wellcome Trust, and Department of Health and Social Care (UK).

**Research in context**

**Evidence before this study**

Lower respiratory infection (LRI) is a common and deadly infectious disease, particularly in children and older adults. Previous iterations of the Global Burden of Diseases, Injuries, and Risk Factors Study (GBD) and estimates from WHO and the Maternal and Child Epidemiology Estimation Group have quantified the LRI burden for select aetiologies in the pre-COVID-19 era. In addition, many studies have estimated the decrease in incidence or mortality due to LRI or select respiratory pathogens during the COVID-19 pandemic, but these studies are generally limited to one or a few surveillance networks, countries, or clinical sites. We searched PubMed with the search terms (“lower respiratory infection*”[Title/Abstract] OR “LRI”[Title/Abstract]) AND (“mortality” OR “incidence”) AND “global*” AND (“etiology” OR “pathogen”) with no language restrictions, for articles published from Jan 1, 2021 to June 16, 2023. We did not identify any studies that evaluated global levels and trends of LRI burden in all ages, attributable to a comprehensive set of aetiologies, across all countries, and inclusive of the COVID-19 pandemic's effects to the year 2021.

**Added value of this study**

This study provides two key improvements on the past GBD study: expanded aetiology estimation and evaluation of COVID-19 pandemic impact. We produced estimates of non-COVID-19 LRI burden attributable to a comprehensive set of 18 different aetiologies (*Acinetobacter baumannii, Chlamydia* spp, *Enterobacter* spp, *Escherichia coli*, fungi, group B streptococcus, *Haemophilus influenzae*, influenza, *Klebsiella pneumoniae, Legionella* spp, *Mycoplasma* spp, polymicrobial infections, *Pseudomonas aeruginosa*, respiratory syncytial virus [RSV], *Staphylococcus aureus, Streptococcus pneumoniae*, and other viruses, as well as a residual category of other bacterial pathogens). 13 of these aetiologies are newly included in the GBD study, significantly expanding our understanding of the diverse causes of LRI. In addition, this research, which models through the year 2021, estimates the reduction in non-COVID-19 LRI incidence and mortality observed during the COVID-19 pandemic period. In addition, we added many new data sources on LRI morbidity and mortality since GBD 2019, which span widely across time and geography, enabling us to revise and improve estimates from previous years. Overall, these enhancements contribute to a more comprehensive and up-to-date understanding of the global burden of LRI, incorporating previously unaccounted for aetiologies and considering the influence of the COVID-19 pandemic on respiratory infections. This information is invaluable for health-care practitioners, policy makers, and researchers in effectively developing targeted interventions to combat LRIs.

**Implications of all the available evidence**

With a comprehensive understanding of the aetiologies of LRI and their impact, health-care authorities can design targeted interventions to address specific pathogens responsible for respiratory infections. These interventions might include vaccination campaigns, improved infection control measures, and early detection and treatment strategies. This study found *S pneumoniae* to be the most common cause of LRI deaths in 2021, followed by *S aureus* and *K pneumoniae*. During the COVID-19 pandemic, following the implementation of non-pharmaceutical interventions such as facemask use and mobility restrictions, we observed a decline in global influenza and RSV infection incidence and mortality. Since 1990, incidence and mortality due to LRI have greatly decreased, especially in children younger than 5 years, while mortality rates in adults, especially those aged 70 years and older, have had a slower rate of decline. Our analysis particularly highlights the decrease in vaccine-preventable aetiologies, *S pneumoniae* and *H influenzae*, and the importance of maintaining and expanding vaccine coverage against these bacteria. We also found high mortality attributable to non-vaccine-preventable aetiologies, including *S aureus*; development of preventive therapies and vaccines for these pathogens should receive further investment and research. Furthermore, as the threat of antimicrobial resistance grows, robust pathogen surveillance, point-of-care pathogen identification, and implementation of strategies to reduce antibiotic overuse become essential. The LRI burden remains highly inequitable, with both deaths and cases highly concentrated in low-income and middle-income countries; thus, all interventions must be financially accessible and distributed to areas with a high burden of LRI.

## Introduction

Lower respiratory infections (LRIs) were the leading infectious cause of death globally in 2019.^1^, ^2^ Gram-positive and Gram-negative bacteria, atypical bacteria, viruses, and fungi can all cause LRI. Mortality rates are highest in adults older than 70 years and in children younger than 5 years, and both incidence and mortality are generally higher in males.^3^, ^4^, ^5^ Risk factors for LRI mortality in all age groups include exposure to tobacco smoke, indoor and outdoor particulate matter, and extreme temperatures.^3^ In children younger than 5 years, wasting is estimated to be responsible for over half of LRI deaths.^3^ Among adults aged 65 years and older, host-level risk factors can include frailty and presence of comorbid conditions such as asthma.^6^, ^7^ Vaccination against *Streptococcus pneumoniae* is protective against pneumococcal pneumonia in both infants and older adults.^7^, ^8^

Among community-acquired bacterial LRIs, *S pneumoniae* remains the most prevalent pathogen in children and adults and across different income-level settings.^9^, ^10^ Historically, *Haemophilus influenzae* was the second-leading cause of childhood pneumonia.^11^ However, with the widespread implementation of *H influenzae* type b (Hib) vaccination, the incidence of *H influenzae* pneumonia has declined substantially over the past decade.^8^, ^12^ *Staphylococcus aureus*, which is not vaccine-preventable, is a noteworthy cause of complicated pneumonia, with substantially higher rates of poor clinical outcomes, including sepsis and death, than *S pneumoniae*.^13^, ^14^ *S aureus* also has the ability to develop resistance to multiple antibiotics, posing further barriers to care.^15^ In school-age children, the atypical bacterium *Mycoplasma pneumoniae* is a leading cause of pneumonia, with one review estimating that it is responsible for 4–39% of cases of paediatric community-acquired pneumonia.^16^, ^17^

Viruses, including influenza and respiratory syncytial virus (RSV), are highly prevalent causes of LRIs, particularly in children.^10^, ^18^ A 2021 global meta-analysis estimated that influenza viruses were responsible for 14·1% of adult LRI hospitalisations, or more than 5 million hospitalisations.^19^ Another global meta-analysis estimated that RSV was responsible for 3·6 million hospitalisations in 2019 among children younger than 5 years.^20^ In addition, viral infections increase patients' risk for superimposed bacterial infections, most commonly by *S pneumoniae* and *S aureus*, causing substantial morbidity and mortality.^21^

Beginning in 2020, the COVID-19 pandemic promoted the adoption of non-pharmaceutical interventions, including stay-at-home orders, school and community closures, and facemask requirements. These measures effectively curbed the incidence of respiratory infections in 2020 and 2021, for both COVID-19 and other respiratory viruses.^22^, ^23^, ^24^, ^25^ RSV and influenza infection incidence declined in response to these non-pharmaceutical interventions, although some locations had outbreaks of these viruses in atypical seasons as non-pharmaceutical interventions were relaxed.^22^, ^23^, ^24^, ^25^

This study presents the results from the Global Burden of Diseases, Injuries, and Risk Factors Study (GBD) 2021, which estimates LRI incidence and mortality, combined with the findings of the Global Research on Antimicrobial Resistance (AMR) project, which estimates LRI pathogen distribution. We aimed to describe the burden and trends of LRIs and the pathogens responsible across 204 countries and territories from 1990 to 2021. Previous GBD studies included estimates of four aetiologies that were not mutually exclusive or collectively exhaustive.^2^, ^26^ In the current study, we provide estimates for a comprehensive set of 18 pathogen categories across all age groups.^27^, ^28^ Additionally, the estimates for 2020 and 2021 account for the reduction of LRIs seen during the COVID-19 pandemic and implementation of non-pharmaceutical interventions.

## Methods

### Overview

This Article was produced as part of the GBD Collaborator Network and in accordance with the GBD protocol. GBD 2021 produced estimates of mortality and morbidity due to LRI by age and sex for 204 countries and territories between 1990 and 2021. The Global Burden of AMR study produced estimates of aetiology-specific fatal and non-fatal burdens of select infectious syndromes, including LRI.^27^ LRI is defined as acute pneumonia or bronchiolitis, not inclusive of COVID-19. ICD codes mapped to LRI in GBD are provided in appendix 1 (pp 17–18) for ICD-9 and ICD-10. The GBD case definition of LRI does not include tuberculosis, pertussis, or COVID-19; although the pathogens that cause these diseases can infect the lower respiratory tract, they are modelled separately due to their individual public health significance and are not included in the GBD category of LRI.

GBD uses a set of modelling tools, described in the sections below, to extrapolate available data out to produce results for the entire global population, by age, sex, and year. Modelling was done at the 1000 draw level, where the point estimate was computed as the mean of 1000 draws, and the 95% uncertainty intervals (UIs) were computed as the 25th and 975th ranked values of 1000 draws. We used the GBD 2021 global population age standard to calculate age-standardised rates, which allow for comparison of rates between locations or years with different age structures.^29^ In the following sections, we summarise key methods from the GBD and Global Burden of AMR studies for the estimation of LRI and its aetiologies. More details on these methods, including a flowchart, are provided in appendix 1 (pp 4–29). Full descriptions of the GBD and Global Burden of AMR studies have been published previously.^2^, ^27^

All metadata for input sources described below are available on the GBD Sources Tool, found on the Global Health Data Exchange (GHDx), which readers can use to identify which sources were used for estimating an outcome in any given location. GBD 2019 complies with the GATHER statement (appendix 1 pp 30–31).^30^ Statistical code used for GBD estimation is publicly available online on the GHDx.

### Mortality estimation

As inputs to the GBD LRI mortality-estimation model, we used a total of 26 259 site-years of data: 23 062 site-years from vital registration, 825 site-years from sample vital registration, 1682 site-years from verbal autopsy, 681 site-years from surveillance sources, and 9 site-years from minimally invasive tissue sampling. Data are processed using a set of standard algorithms accounting for incompleteness, misclassification of the underlying cause of death, garbage coding, and stochastic variability.^2^

We estimated overall LRI mortality using the Cause of Death Ensemble model (CODEm),^31^ which evaluates a wide array of potential models using various combinations of covariates and four model classes. Each model class uses either cause fraction or death rate as the outcome variable, and either a mixed-effects linear model or a spatiotemporal Gaussian process model as the regression method. Models included fixed effects on covariates and age dummies. Random effects are applied at the levels of super-region, region, and age in the spatiotemporal model's mixed-effects structure, and at the levels of super-region, region, country, and age in the mixed-effects linear models. In mixed-effects regression, the random effects are assumed to follow a normal distribution with a mean of zero and a variance–covariance matrix that is to be estimated from the data. Models were evaluated using out-of-sample predictive validity and integrated into one ensemble model. A full list of covariates is provided in appendix 1 (pp 8–9). Final LRI mortality estimates are scaled by a procedure known as CoDCorrect to ensure consistency between the sum of cause-specific mortality and the total envelope of all-cause mortality.^2^

### Morbidity estimation

For LRI morbidity estimation, we used data from published studies identified via a systematic review (appendix 1 p 10), surveillance data, LRI mortality estimates (described above), health insurance claims data, and inpatient data.^2^ To correct for potential systematic bias among different categories of data sources, we used a standardised crosswalking technique to adjust the data to enhance comparability before modelling (appendix 1 pp 10–13). We estimated LRI incidence and prevalence using DisMod-MR 2.1, a compartmental Bayesian meta-regression model that enforces consistency among prevalence, incidence, remission, and mortality.^2^, ^32^ More details on DisMod-MR, including information on priors and a full list of covariates, is provided in appendix 1 (pp 15–17).

### Aetiology estimation

Data used for aetiology estimation originated from multiple cause-of-death vital registration, hospital discharges, microbial laboratory data, and published studies from the literature.^27^ Mortality and morbidity are estimated for the following causes of LRI: *Acinetobacter baumannii, Chlamydia* spp, *Enterobacter* spp, *Escherichia coli*, fungi, group B streptococcus, *H influenzae*, influenza viruses, *Klebsiella pneumoniae, Legionella* spp, *Mycoplasma* spp, polymicrobial infections, *Pseudomonas aeruginosa*, RSV, *S aureus, S pneumoniae*, and other viruses (ie, the aggregate of all viruses except for influenza and RSV), as well as a residual category of other bacterial pathogens. The ICD-9 and ICD-10 codes mapped to each cause are listed in appendix 1 (pp 18–19).

Incidence proportions were estimated using multinomial estimation as part of a network analysis model, which allows for the inclusion of data sources that are considered to be partial observations—ie, which do not contain all pathogen groups modelled in the study.^27^ Proportions were estimated as a function of age group, infection type, Hib and pneumococcal vaccination, and Healthcare Access and Quality (HAQ) Index. These covariates vary across geography and time, creating unique predictions for each age group, location, and year. For data sources that only reported deaths, we used modelled case-fatality rates (CFRs) to retroactively estimate the number of cases. These CFRs for each pathogen were modelled using a Bayesian meta-regression tool, MR-BRT (meta-regression—Bayesian, regularised, trimmed), as a function of age group, pathogen, and HAQ Index, with random effects on data source.^27^, ^33^, ^34^ For *S pneumoniae*, we used a vaccine probe design as an additional input to the incidence proportion model, due to the documented challenge in the microbiological identification of this pathogen.^35^ Modelled CFRs were then used again to compute mortality proportions from case proportions. More details on aetiology estimation can be found in appendix 1 (pp 17–26). Ultimately, all estimated incident LRI cases were distributed to an estimated aetiology, even those with no aetiology detected, following the modelled aetiology distribution patterns by age, location, and year.

### COVID-19 impact adjustment

We developed a multistep modelling process to estimate the reduction of incidence of influenza and RSV in 2020 and 2021. Our source data were reported cases of influenza by country, from notifications reported by countries to WHO's FluNet.^36^ First, we interpolated the number of reported cases of influenza in 2020 and 2021 by month using the RegMod framework, a Poisson model that estimates the underlying rate of infection in each month as a function of a seasonal pattern and an underlying temporal trend.^37^ Second, we calculated an under-reporting ratio in the pre-pandemic reference period, 2017–19, for each location by dividing the interpolated number of reported cases from RegMod by the GBD estimated number of cases of LRI due to influenza. Third, we estimated the pandemic disruption-free counterfactual number of reported cases, meaning the number of reported cases we would have expected during 2020 and 2021 in the hypothetical pandemic-free scenario. We did this by multiplying the under-reporting ratio by the estimated number of cases of LRI due to influenza, for 2020 and 2021, that GBD would have estimated in a pandemic-free scenario. Finally, we calculated a yearly disruption influenza scalar for each location for 2020 and 2021. This scalar was computed by dividing the interpolated number of reported cases from RegMod (result of first step) by the counterfactual disruption-free number of reported cases (result of third step).

These influenza disruption scalars (result of final step) were multiplied by counterfactual incident cases and deaths for both influenza and RSV (result of third step), to estimate adjusted cases and deaths. More details on the adjustments are provided in appendix 1 (pp 26–29).

### Role of the funding source

The funders of the study had no role in study design, data collection, data analysis, data interpretation, or writing of the report.

## Results

### Incidence of LRIs

Globally, in 2019, before the reductions in incidence observed during the COVID-19 pandemic, we estimated 369 million (95% UI 349–391) LRI episodes, for an all-age incidence rate of 4770 episodes (4510–5040) per 100 000 population (table 1).

**Table 1:** Lower respiratory infection incidence counts and rates for all-ages and selected age groups in 1990, 2019, 2020, and 2021, and incidence rate percentage change from 1990 to 2019 and from 2019 to 2021, globally and by SDI quintile and GBD super-region

	**1990**		**2019**		**2020**		**2021**		**Incidence rate change, %**	
	Episode count	Incidence rate per 100 000 population	Episode count	Incidence rate per 100 000 population	Episode count	Incidence rate per 100 000 population	Episode count	Incidence rate per 100 000 population	1990–2019	2019–21
**Global**										
All ages	314 000 000 (294 000 000 to 333 000 000)	5884·6 (5513·2 to 6249·0)	369 000 000 (349 000 000 to 391 000 000)	4766·4 (4507·5 to 5041·7)	342 000 000 (324 000 000 to 360 000 000)	4369·4 (4144·4 to 4608·3)	344 000 000 (325 000 000 to 364 000 000)	4354·2 (4121·1 to 4606·5)	−19·0% (−21·9 to −16·0)	−8·6% (−10·4 to −6·6)
<5 years	101 000 000 (89 800 000 to 114 000 000)	16 302·6 (14 489·0 to 18 341·7)	45 000 000 (40 000 000 to 50 800 000)	6639·0 (5903·8 to 7493·3)	39 800 000 (35 500 000 to 45 000 000)	5940·8 (5296·9 to 6720·0)	37 800 000 (33 500 000 to 43 000 000)	5747·5 (5085·2 to 6537·1)	−59·3% (−60·1 to −58·3)	−13·4% (−15·9 to −10·6)
5–14 years	43 600 000 (35 500 000 to 52 800 000)	3893·0 (3169·1 to 4719·6)	34 000 000 (28 100 000 to 40 700 000)	2560·4 (2117·1 to 3062·5)	32 500 000 (26 800 000 to 38 900 000)	2420·2 (1995·5 to 2900·1)	32 100 000 (26 500 000 to 38 500 000)	2369·3 (1954·0 to 2841·8)	−34·2% (−37·0 to −31·2)	−7·5% (−9·3 to −5·8)
15–49 years	66 700 000 (60 400 000 to 73 300 000)	2460·2 (2229·3 to 2703·7)	94 200 000 (85 700 000 to 103 000 000)	2415·0 (2197·9 to 2641·6)	87 700 000 (79 800 000 to 95 900 000)	2235·5 (2034·2 to 2444·1)	88 800 000 (80 800 000 to 97 100 000)	2249·6 (2046·5 to 2458·9)	−1·8% (−4·0 to 0·6)	−6·8% (−8·6 to −4·8)
50–69 years	56 800 000 (51 400 000 to 62 100 000)	8325·7 (7540·4 to 9112·5)	95 900 000 (87 400 000 to 104 000 000)	6965·5 (6349·8 to 7581·7)	89 500 000 (81 500 000 to 97 100 000)	6352·7 (5787·5 to 6890·2)	91 500 000 (83 300 000 to 99 600 000)	6366·3 (5802·0 to 6936·2)	−16·3% (−19·0 to −13·7)	−8·6% (−10·3 to −6·4)
≥70 years	45 800 000 (41 100 000 to 50 700 000)	22 654·9 (20 326·7 to 25 095·8)	100 000 000 (90 900 000 to 112 000 000)	21 560·2 (19 575·2 to 24 087·4)	92 300 000 (83 800 000 to 102 000 000)	19 279·4 (17 503·3 to 21 229·6)	93 400 000 (84 300 000 to 104 000 000)	18 897·7 (17 055·2 to 21 025·2)	−4·8% (−9·1 to −0·4)	−12·3% (−14·2 to −10·3)
**High SDI**										
All ages	15 700 000 (14 900 000 to 16 600 000)	1783·6 (1689·8 to 1890·0)	17 900 000 (17 000 000 to 18 900 000)	1647·3 (1562·4 to 1738·8)	16 600 000 (15 700 000 to 17 500 000)	1519·6 (1439·4 to 1608·0)	14 800 000 (14 100 000 to 15 700 000)	1354·6 (1285·3 to 1433·2)	−7·6% (−10·1 to −5·3)	−17·8% (−18·9 to −16·5)
<5 years	1 920 000 (1 660 000 to 2 190 000)	3104·5 (2686·7 to 3556·3)	908 000 (775 000 to 1 060 000)	1623·3 (1386·1 to 1900·3)	750 000 (638 000 to 874 000)	1365·2 (1160·7 to 1590·2)	600 000 (513 000 to 702 000)	1114·0 (952·4 to 1302·9)	−47·7% (−50·6 to −45·1)	−31·4% (−32·9 to −29·4)
5–14 years	1 040 000 (820 000to 1 340 000)	841·3 (660·5 to 1080·0)	604 000 (476 000 to 761 000)	511·0 (402·9 to 643·8)	565 000 (447 000 to 720 000)	476·7 (376·7 to 607·5)	533 000 (420 000 to 675 000)	449·5 (354·2 to 569·0)	−39·3% (−41·5 to −36·6)	−12·0% (−13·5 to −10·7)
15–49 years	2 490 000 (2 220 000 to 2 790 000)	539·5 (481·7 to 604·4)	2 120 000 (1 900 000 to 2 340 000)	418·8 (376·0 to 462·7)	1 930 000 (1 740 000 to 2 130 000)	382·9 (344·4 to 422·8)	1 820 000 (1 630 000 to 2 010 000)	363·3 (324·8 to 400·7)	−22·4% (−24·3 to −20·5)	−13·3% (−14·6 to −11·8)
50–69 years	3 860 000 (3 560 000 to 4 170 000)	2354·7 (2173·2 to 2542·5)	4 520 000 (4 180 000 to 4 880 000)	1663·0 (1537·2 to 1796·6)	4 190 000 (3 860 000 to 4 530 000)	1532·8 (1412·3 to 1654·1)	3 860 000 (3 560 000 to 4 160 000)	1399·4 (1290·0 to 1509·6)	−29·4% (−30·9 to −27·5)	−15·9% (−17·1 to −14·3)
≥70 years	6 380 000 (5 880 000 to 6 960 000)	9244·5 (8509·4 to 10 077·5)	9 760 000 (9 050 000 to 10 600 000)	7192·4 (6664·9 to 7805·8)	9 140 000 (8 480 000 to 9 840 000)	6534·2 (6059·1 to 7036·3)	8 000 000 (7 420 000 to 8 680 000)	5578·0 (5172·7 to 6051·3)	−22·2% (−24·4 to −20·1)	−22·4% (−23·7 to −21·1)
**High-middle SDI**										
All ages	37 200 000 (34 900 000 to 39 700 000)	3498·3 (3279·9 to 3730·9)	45 900 000 (43 000 000 to 48 900 000)	3535·0 (3316·4 to 3772·9)	42 400 000 (39 900 000 to 45 200 000)	3261·4 (3065·9 to 3474·3)	40 900 000 (38 500 000 to 43 500 000)	3138·7 (2951·6 to 3339·3)	1·1% (−4·2 to 6·3)	−11·2% (−13·3 to −9·2)
<5 years	11 300 000 (9 950 000 to 12 900 000)	12 155·7 (10 709·8 to 13 896·9)	3 220 000 (2 710 000 to 3 790 000)	4201·3 (3537·7 to 4940·8)	2 720 000 (2 290 000 to 3 200 000)	3682·0 (3096·2 to 4325·9)	2 240 000 (1 880 000 to 2 620 000)	3202·9 (2686·7 to 3735·4)	−65·4% (−68·5 to −62·2)	−23·8% (−26·1 to −21·2)
5–14 years	4 980 000 (4 020 000 to 6 130 000)	2756·3 (2224·9 to 3392·1)	3 730 000 (2 940 000 to 4 640 000)	2412·3 (1902·9 to 3001·3)	3 640 000 (2 870 000 to 4 540 000)	2304·1 (1819·7 to 2877·3)	3 640 000 (2 870 000 to 4 540 000)	2260·8 (1782·5 to 2824·0)	−12·5% (−19·4 to −4·8)	−6·3% (−9·2 to −3·5)
15–49 years	6 710 000 (6 030 000 to 7 440 000)	1188·4 (1068·1 to 1318·7)	8 620 000 (7 700 000 to 9 620 000)	1345·6 (1202·4 to 1502·0)	7 950 000 (7 120 000 to 8 830 000)	1252·6 (1121·2 to 1390·1)	7 620 000 (6 840 000 to 8 460 000)	1211·1 (1085·7 to 1343·0)	13·2% (9·7 to 17·3)	−10·0% (−11·8 to −8·1)
50–69 years	7 460 000 (6 830 000 to 8 080 000)	4284·7 (3925·4 to 4640·2)	12 100 000 (11 000 000 to 13 200 000)	3825·6 (3485·4 to 4174·3)	11 500 000 (10 500 000 to 12 500 000)	3581·3 (3281·3 to 3888·7)	11 100 000 (10 100 000 to 12 100 000)	3417·1 (3094·2 to 3705·9)	−10·7% (−13·8 to −6·9)	−10·7% (−13·3 to −7·7)
≥70 years	6 770 000 (6 140 000 to 7 390 000)	13 149·2 (11 923·5 to 14 362·2)	18 200 000 (16 300 000 to 20 300 000)	16 585·7 (14 816·6 to 18 516·2)	16 600 000 (14 900 000 to 18 300 000)	14 660·6 (13 102·3 to 16 151·7)	16 300 000 (14 600 000 to 18 100 000)	13 866·9 (12 398·2 to 15 377·7)	26·1% (19·2 to 34·3)	−16·4% (−19·6 to −13·1)
**Middle SDI**										
All ages	87 100 000 (81 400 000 to 92 900 000)	5054·4 (4726·3 to 5390·2)	96 700 000 (91 100 000 to 102 000 000)	4012·3 (3778·5 to 4251·6)	89 000 000 (84 200 000 to 94 300 000)	3662·1 (3461·7 to 3879·0)	89 500 000 (84 700 000 to 95 300 000)	3657·2 (3457·2 to 3892·0)	−20·6% (−24·1 to −16·7)	−8·8% (−10·8 to −6·7)
<5 years	29 700 000 (26 300 000 to 33 500 000)	14 797·3 (13 136·4 to 16 713·9)	8 680 000 (7 630 000 to 9 890 000)	4632·6 (4071·5 to 5274·5)	7 590 000 (6 680 000 to 8 680 000)	4148·5 (3650·8 to 4740·1)	6 850 000 (5 990 000 to 7 890 000)	3879·7 (3392·6 to 4469·1)	−68·7% (−69·7 to −67·6)	−16·3% (−18·8 to −13·2)
5–14 years	12 800 000 (10 500 000 to 15 500 000)	3402·6 (2778·2 to 4121·3)	7 370 000 (5 990 000 to 8 910 000)	1930·1 (1568·4 to 2332·2)	6 970 000 (5 660 000 to 8 430 000)	1803·1 (1465·3 to 2183·1)	6 830 000 (5 560 000 to 8 280 000)	1750·7 (1426·0 to 2122·5)	−43·3% (−46·4 to −39·5)	−9·3% (−11·2 to −7·4)
15–49 years	18 900 000 (17 200 000 to 20 800 000)	2074·9 (1885·7 to 2280·5)	24 600 000 (22 300 000 to 27 000 000)	1966·6 (1781·8 to 2159·1)	22 500 000 (20 500 000 to 24 700 000)	1799·4 (1634·5 to 1973·7)	22 600 000 (20 500 000 to 24 900 000)	1803·5 (1633·0 to 1986·2)	−5·2% (−8·0 to −2·8)	−8·3% (−10·5 to −6·1)
50–69 years	14 400 000 (13 000 000 to 15 800 000)	7609·8 (6853·4 to 8360·1)	27 800 000 (25 200 000 to 30 500 000)	6042·7 (5495·9 to 6633·2)	25 900 000 (23 600 000 to 28 300 000)	5468·6 (4987·2 to 5975·0)	26 600 000 (24 200 000 to 29 000 000)	5470·5 (4990·1 to 5976·3)	−20·6% (−23·4 to −17·6)	−9·5% (−11·4 to −7·1)
≥70 years	11 300 000 (10 100 000 to 12 500 000)	24 697·8 (22 037·6 to 27 426·9)	28 300 000 (25 700 000 to 31 600 000)	21 637·0 (19 643·2 to 24 127·6)	26 000 000 (23 700 000 to 28 800 000)	19 223·2 (17 467·8 to 21 269·0)	26 700 000 (24 100 000 to 29 600 000)	18 911·1 (17 100·3 to 21 016·6)	−12·4% (−16·4 to −7·2)	−12·6% (−15·0 to −10·1)
**Low-middle SDI**										
All ages	119 000 000 (111 000 000 to 127 000 000)	10 254·0 (9596·2 to 10 941·0)	137 000 000 (129 000 000 to 145 000 000)	7301·1 (6889·2 to 7748·4)	126 000 000 (119 000 000 to 133 000 000)	6638·3 (6282·2 to 7006·3)	130 000 000 (122 000 000 to 138 000 000)	6742·4 (6355·3 to 7173·5)	−28·8% (−31·5 to −26·0)	−7·7% (−10·3 to −4·6)
<5 years	37 500 000 (33 300 000 to 42 000 000)	21 604·0 (19 187·8 to 24 200·3)	16 700 000 (14 900 000 to 18 700 000)	8530·6 (7610·6 to 9580·6)	14 500 000 (13 000 000 to 16 600 000)	7512·9 (6704·2 to 8555·4)	14 100 000 (12 400 000 to 16 000 000)	7343·5 (6464·9 to 8356·5)	−60·5% (−61·5 to −59·4)	−13·9% (−17·8 to −10·0)
5–14 years	17 000 000 (13 900 000 to 20 800 000)	5694·7 (4647·4 to 6948·8)	13 000 000 (10 700 000 to 15 400 000)	3353·2 (2768·0 to 3977·9)	12 100 000 (10 100 000 to 14 500 000)	3136·1 (2604·9 to 3745·9)	12 000 000 (9 930 000 to 14 400 000)	3099·9 (2556·6 to 3718·7)	−41·1% (−44·2 to −38·0)	−7·6% (−10·6 to −4·4)
15–49 years	26 800 000 (24 200 000 to 29 600 000)	4865·4 (4397·2 to 5362·7)	38 600 000 (35 200 000 to 42 100 000)	3908·7 (3562·9 to 4260·6)	35 800 000 (32 500 000 to 39 100 000)	3573·6 (3242·2 to 3907·2)	36 700 000 (33 300 000 to 40 100 000)	3613·3 (3279·4 to 3947·7)	−19·7% (−21·7 to −17·1)	−7·6% (−10·5 to −4·5)
50–69 years	22 000 000 (19 800 000 to 24 400 000)	19 664·4 (17 647·7 to 21 804·9)	36 300 000 (33 000 000 to 39 700 000)	14 999·1 (13 644·8 to 16 423·3)	33 600 000 (30 500 000 to 36 500 000)	13 488·6 (12 241·0 to 14 682·2)	35 000 000 (31 800 000 to 38 200 000)	13 744·2 (12 491·8 to 14 995·5)	−23·7% (−27·4 to −19·9)	−8·4% (−11·4 to −5·2)
≥70 years	15 800 000 (13 900 000 to 17 800 000)	60 146·0 (52 905·4 to 67 732·4)	32 600 000 (29 400 000 to 36 900 000)	48 877·1 (44 089·3 to 55 283·6)	30 100 000 (27 000 000 to 33 700 000)	43 991·0 (39 501·2 to 49 353·2)	31 700 000 (28 200 000 to 35 900 000)	45 178·2 (40 213·6 to 51 217·1)	−18·7% (−24·0 to −13·3)	−7·6% (−11·3 to −3·5)
**Low SDI**										
All ages	54 600 000 (50 900 000 to 58 200 000)	10 899·1 (10 149·8 to 11 601·2)	71 500 000 (67 300 000 to 75 400 000)	6698·9 (6308·8 to 7070·2)	67 500 000 (63 600 000 to 71 100 000)	6176·0 (5823·9 to 6510·0)	68 600 000 (65 000 000 to 72 600 000)	6143·1 (5812·8 to 6500·9)	−38·5% (−40·4 to −36·5)	−8·3% (−10·2 to −6·3)
<5 years	20 600 000 (18 200 000 to 23 400 000)	22 738·9 (20 015·8 to 25 809·9)	15 500 000 (13 900 000 to 17 400 000)	9564·9 (8563·1 to 10 736·1)	14 200 000 (12 600 000 to 15 900 000)	8642·5 (7675·9 to 9724·6)	14 000 000 (12 500 000 to 15 900 000)	8480·2 (7543·0 to 9585·9)	−57·9% (−59·1 to −56·7)	−11·3% (−14·0 to −8·6)
5–14 years	7 700 000 (6 290 000 to 9 290 000)	5576·6 (4554·1 to 6725·8)	9 320 000 (7 750 000 to 11 100 000)	3263·0 (2713·8 to 3892·8)	9 130 000 (7 650 000 to 10 900 000)	3146·1 (2636·6 to 3757·0)	9 020 000 (7 500 000 to 10 900 000)	3061·6 (2543·7 to 3691·2)	−41·5% (−44·4 to −38·1)	−6·2% (−8·3 to −3·8)
15–49 years	11 800 000 (10 600 000 to 12 900 000)	5317·4 (4806·8 to 5850·9)	20 200 000 (18 300 000 to 22 000 000)	3951·9 (3580·5 to 4303·3)	19 500 000 (17 700 000 to 21 100 000)	3693·8 (3367·2 to 4009·8)	20 000 000 (18 300 000 to 21 900 000)	3686·4 (3364·9 to 4029·7)	−25·7% (−28·1 to −22·8)	−6·7% (−8·7 to −4·5)
50–69 years	9 010 000 (8 030 000 to 9 980 000)	21 438·0 (19 102·1 to 23 759·2)	15 200 000 (13 800 000 to 16 700 000)	17 480·9 (15 878·6 to 19 177·8)	14 300 000 (13 100 000 to 15 600 000)	15 844·2 (14 473·5 to 17 304·6)	14 800 000 (13 500 000 to 16 200 000)	15 955·7 (14 491·9 to 17 459·3)	−18·5% (−22·4 to −14·2)	−8·7% (−11·6 to −6·0)
≥70 years	5 530 000 (4 900 000 to 6 210 000)	59 241·9 (52 529·8 to 66 532·7)	11 200 000 (10 000 000 to 12 700 000)	53 575·8 (47 915·3 to 60 574·5)	10 400 000 (9 300 000 to 11 700 000)	48 762·8 (43 489·0 to 54 900·8)	10 800 000 (9 600 000 to 12 200 000)	49 111·1 (43 765·6 to 55 612·9)	−9·6% (−15·4 to −2·0)	−8·3% (−11·6 to −4·8)
**Central Europe, Eastern Europe, and Central Asia**										
All ages	10 800 000 (10 200 000 to 11 400 000)	2570·4 (2426·7 to 2718·0)	8 010 000 (7 600 000 to 8 450 000)	1913·5 (1816·4 to 2018·3)	7 860 000 (7 490 000 to 8 240 000)	1877·0 (1788·7 to 1969·3)	6 950 000 (6 600 000 to 7 330 000)	1664·0 (1580·1 to 1754·3)	−25·6% (−28·0 to −23·0)	−13·0% (−14·1 to −11·9)
<5 years	3 150 000 (2 800 000 to 3 520 000)	8779·0 (7807·7 to 9795·9)	874 000 (783 000 to 980 000)	3234·3 (2897·7 to 3628·1)	812 000 (719 000 to 908 000)	3078·0 (2726·4 to 3440·6)	607 000 (542 000 to 689 000)	2363·2 (2110·1 to 2679·0)	−63·2% (−65·2 to −61·0)	−26·9% (−28·8 to −24·9)
5–14 years	1 440 000 (1 200 000 to 1 750 000)	2055·0 (1708·4 to 2500·7)	669 000 (557 000 to 801 000)	1249·0 (1040·5 to 1496·1)	666 000 (555 000 to 806 000)	1223·6 (1018·6 to 1479·8)	609 000 (504 000 to 739 000)	1105·0 (914·2 to 1341·5)	−39·2% (−41·5 to −37·0)	−11·5% (−13·9 to −9·0)
15–49 years	1 730 000 (1 600 000 to 1 880 000)	843·2 (775·8 to 914·7)	1 670 000 (1 550 000 to 1 800 000)	836·5 (775·5 to 899·6)	1 660 000 (1 550 000 to 1 790 000)	834·4 (777·3 to 900·3)	1 490 000 (1 390 000 to 1 610 000)	754·8 (701·2 to 815·4)	−0·8% (−4·4 to 3·0)	−9·8% (−11·4 to −8·0)
50–69 years	2 560 000 (2 370 000 to 2 770 000)	3054·6 (2822·0 to 3303·6)	2 500 000 (2 290 000 to 2 700 000)	2499·2 (2292·1 to 2703·0)	2 470 000 (2 290 000 to 2 650 000)	2470·0 (2287·9 to 2655·1)	2 240 000 (2 070 000 to 2 420 000)	2244·8 (2073·3 to 2431·5)	−18·2% (−20·3 to −16·3)	−10·2% (−11·7 to −8·5)
≥70 years	1 930 000 (1 760 000 to 2 120 000)	7626·4 (6970·1 to 8376·3)	2 290 000 (2 110 000 to 2 530 000)	6037·4 (5559·7 to 6670·9)	2 250 000 (2 060 000 to 2 470 000)	5773·6 (5297·5 to 6327·9)	2 010 000 (1 850 000 to 2 210 000)	5062·9 (4662·7 to 5579·0)	−20·8% (−23·0 to −18·7)	−16·1% (−17·6 to −14·6)
**High-income**										
All ages	14 300 000 (13 500 000 to 15 100 000)	1572·4 (1490·5 to 1665·2)	15 900 000 (15 100 000 to 16 800 000)	1465·3 (1388·2 to 1546·9)	14 600 000 (13 900 000 to 15 500 000)	1341·4 (1272·7 to 1418·8)	13 000 000 (12 300 000 to 13 700 000)	1188·6 (1125·1 to 1257·8)	−6·8% (−9·1 to −4·7)	−18·9% (−20·0 to −17·7)
<5 years	1 520 000 (1 320 000 to 1 730 000)	2464·9 (2137·1 to 2814·1)	777 000 (661 000 to 906 000)	1375·3 (1170·6 to 1604·2)	617 000 (521 000 to 726 000)	1114·0 (940·6 to 1310·9)	510 000 (436 000 to 594 000)	939·6 (803·2 to 1093·5)	−44·2% (−47·1 to −41·7)	−31·7% (−33·5 to −29·6)
5–14 years	726 000 (566 000 to 925 000)	576·7 (449·6 to 735·0)	453 000 (358 000 to 570 000)	369·6 (292·5 to 465·3)	414 000 (328 000 to 521 000)	338·4 (268·2 to 425·7)	392 000 (310 000 to 491 000)	320·7 (254·1 to 402·2)	−35·9% (−38·2 to −33·1)	−13·2% (−14·6 to −11·8)
15–49 years	2 200 000 (1 960 000 to 2 470 000)	467·4 (417·2 to 524·1)	1 660 000 (1 490 000 to 1 830 000)	340·4 (306·2 to 375·2)	1 490 000 (1 340 000 to 1 640 000)	306·8 (276·3 to 337·0)	1 420 000 (1 270 000 to 1 560 000)	292·8 (261·8 to 322·8)	−27·2% (−29·0 to −25·0)	−14·0% (−15·2 to −12·6)
50–69 years	3 540 000 (3 270 000 to 3 820 000)	2019·0 (1867·6 to 2179·5)	3 850 000 (3 560 000 to 4 150 000)	1406·3 (1302·5 to 1516·7)	3 530 000 (3 270 000 to 3 790 000)	1282·8 (1188·9 to 1376·1)	3 230 000 (2 970 000 to 3 480 000)	1164·2 (1070·6 to 1255·5)	−30·3% (−32·0 to −28·4)	−17·2% (−18·5 to −16·0)
≥70 years	6 310 000 (5 820 000 to 6 870 000)	8318·1 (7666·1 to 9051·3)	9 180 000 (8 520 000 to 9 950 000)	6284·8 (5830·6 to 6814·7)	8 560 000 (7 980 000 to 9 210 000)	5705·6 (5324·0 to 6143·0)	7 420 000 (6 870 000 to 8 050 000)	4846·6 (4486·3 to 5252·9)	−24·4% (−26·6 to −22·4)	−22·9% (−24·2 to −21·6)
**Latin America and Caribbean**										
All ages	15 800 000 (14 900 000 to 17 000 000)	4052·0 (3806·2 to 4347·7)	15 000 000 (14 200 000 to 15 800 000)	2558·8 (2420·0 to 2702·8)	13 300 000 (12 600 000 to 14 000 000)	2256·3 (2131·9 to 2373·9)	12 900 000 (12 100 000 to 13 700 000)	2165·7 (2044·1 to 2300·0)	−36·9% (−39·4 to −34·2)	−15·4% (−17·6 to −12·6)
<5 years	5 940 000 (5 280 000 to 6 740 000)	11 992·5 (10 660·4 to 13 618·8)	2 390 000 (2 080 000 to 2 740 000)	4912·8 (4271·4 to 5627·1)	1 870 000 (1 620 000 to 2 140 000)	3891·1 (3365·0 to 4461·9)	1 680 000 (1 450 000 to 1 930 000)	3560·6 (3072·6 to 4087·7)	−59·0% (−60·5 to −57·7)	−27·5% (−30·9 to −23·0)
5–14 years	2 360 000 (1 970 000 to 2 850 000)	2491·9 (2074·2 to 3010·9)	1 160 000 (943 000 to 1 430 000)	1211·6 (983·5 to 1495·9)	1 040 000 (854 000 to 1 270 000)	1086·2 (890·3 to 1326·2)	1 000 000 (819 000 to 1 240 000)	1045·7 (853·1 to 1290·9)	−51·4% (−53·8 to −48·8)	−13·7% (−15·8 to −11·3)
15–49 years	2 860 000 (2 630 000 to 3 110 000)	1449·4 (1332·4 to 1578·4)	2 650 000 (2 450 000 to 2 870 000)	859·9 (794·1 to 930·7)	2 400 000 (2 210 000 to 2 600 000)	772·8 (713·0 to 837·7)	2 330 000 (2 150 000 to 2 540 000)	746·6 (688·9 to 814·1)	−40·7% (−42·1 to −39·1)	−13·2% (−15·3 to −10·8)
50–69 years	2 260 000 (2 080 000 to 2 460 000)	5947·3 (5462·6 to 6460·2)	3 630 000 (3 330 000 to 3 910 000)	3645·6 (3346·4 to 3922·1)	3 380 000 (3 100 000 to 3 640 000)	3302·0 (3035·8 to 3559·9)	3 320 000 (3 060 000 to 3 600 000)	3181·4 (2925·2 to 3449·4)	−38·7% (−40·0 to −37·3)	−12·7% (−15·0 to −10·1)
≥70 years	2 400 000 (2 190 000 to 2 620 000)	21 978·6 (20 058·6 to 24 044·7)	5 140 000 (4 710 000 to 5 630 000)	15 764·3 (14 439·0 to 17 253·9)	4 630 000 (4 210 000 to 5 040 000)	13 775·5 (12 531·4 to 14 985·4)	4 530 000 (4 140 000 to 4 990 000)	13 119·2 (11 991·9 to 14 468·1)	−28·3% (−30·1 to −26·2)	−16·8% (−19·4 to −14·0)
**North Africa and Middle East**										
All ages	11 200 000 (10 200 000 to 12 200 000)	3287·9 (3003·6 to 3598·4)	10 500 000 (9 850 000 to 11 200 000)	1731·5 (1625·1 to 1843·1)	10 000 000 (9 370 000 to 10 800 000)	1631·0 (1523·4 to 1757·7)	9 380 000 (8 820 000 to 9 980 000)	1505·4 (1415·1 to 1601·6)	−47·3% (−49·8 to −44·1)	−13·1% (−15·4 to −10·2)
<5 years	5 690 000 (4 890 000 to 6 590 000)	11 105·8 (9544·3 to 12 868·0)	2 270 000 (1 960 000 to 2 640 000)	3558·6 (3073·1 to 4150·2)	1 940 000 (1 640 000 to 2 300 000)	3108·6 (2619·8 to 3682·1)	1 670 000 (1 430 000 to 1 960 000)	2725·0 (2334·8 to 3202·1)	−68·0% (−69·4 to −66·3)	−23·4% (−27·5 to −18·2)
5–14 years	1 640 000 (1 310 000 to 2 020 000)	1840·2 (1464·4 to 2265·8)	1 290 000 (1 020 000 to 1 600 000)	1088·8 (862·9 to 1346·5)	1 240 000 (999 000 to 1 520 000)	1032·4 (829·0 to 1258·8)	1 190 000 (944 000 to 1 460 000)	970·5 (772·4 to 1195·0)	−40·8% (−43·9 to −37·8)	−10·9% (−13·8 to −7·3)
15–49 years	1 660 000 (1 500 000 to 1 840 000)	1035·9 (934·6 to 1150·3)	2 600 000 (2 350 000 to 2 870 000)	797·0 (720·6 to 880·3)	2 510 000 (2 280 000 to 2 790 000)	761·6 (690·0 to 845·4)	2 410 000 (2 190 000 to 2 660 000)	722·0 (655·6 to 795·1)	−23·1% (−25·6 to −20·3)	−9·4% (−11·8 to −6·3)
50–69 years	1 180 000 (1 050 000 to 1 300 000)	3772·2 (3378·9 to 4165·4)	2 210 000 (2 010 000 to 2 410 000)	2793·3 (2538·5 to 3049·8)	2 230 000 (2 020 000 to 2 440 000)	2712·6 (2456·1 to 2975·9)	2 150 000 (1 960 000 to 2 340 000)	2528·9 (2307·4 to 2757·0)	−26·0% (−29·7 to −21·8)	−9·5% (−12·4 to −6·0)
≥70 years	983 000 (870 000to 1 090 000)	13 606·8 (12 046·8 to 15 107·9)	2 140 000 (1 940 000 to 2 360 000)	11 094·4 (10 077·1 to 12 259·5)	2 100 000 (1 890 000 to 2 370 000)	10 609·3 (9538·0 to 11 978·7)	1 960 000 (1 790 000 to 2 180 000)	9653·6 (8803·5 to 10 726·7)	−18·5% (−23·7 to −12·8)	−13·0% (−16·2 to −9·2)
**South Asia**										
All ages	143 000 000 (134 000 000 to 153 000 000)	13 099·4 (12 268·7 to 13 973·1)	180 000 000 (169 000 000 to 192 000 000)	9965·4 (9363·5 to 10 604·1)	165 000 000 (156 000 000 to 174 000 000)	9021·6 (8518·1 to 9543·7)	172 000 000 (161 000 000 to 184 000 000)	9319·4 (8733·8 to 9984·8)	−23·9% (−27·0 to −20·8)	−6·5% (−9·8 to −2·8)
<5 years	38 400 000 (34 100 000 to 42 900 000)	24 450·9 (21 713·4 to 27 340·1)	16 900 000 (15 100 000 to 18 800 000)	10 340·6 (9243·4 to 11 556·0)	15 400 000 (13 600 000 to 17 500 000)	9575·9 (8470·8 to 10 885·7)	15 300 000 (13 400 000 to 17 700 000)	9627·6 (8435·1 to 11 133·9)	−57·7% (−58·9 to −56·3)	−6·9% (−12·7 to −0·7)
5–14 years	20 600 000 (16 800 000 to 25 200 000)	7453·7 (6064·5 to 9107·7)	15 700 000 (12 900 000 to 18 900 000)	4461·8 (3668·6 to 5362·4)	14 600 000 (12 000 000 to 17 700 000)	4170·0 (3413·6 to 5042·5)	14 600 000 (11 900 000 to 17 700 000)	4197·6 (3426·9 to 5077·4)	−40·1% (−43·9 to −36·3)	−5·9% (−9·5 to −2·3)
15–49 years	35 700 000 (32 100 000 to 39 300 000)	6743·8 (6060·6 to 7427·6)	53 200 000 (48 200 000 to 58 200 000)	5436·5 (4925·2 to 5949·9)	48 500 000 (43 900 000 to 53 300 000)	4881·7 (4425·5 to 5366·7)	50 100 000 (45 200 000 to 55 200 000)	4974·0 (4491·6 to 5479·6)	−19·4% (−21·5 to −17·0)	−8·5% (−11·6 to −5·2)
50–69 years	29 000 000 (25 900 000 to 32 200 000)	26 980·0 (24 062·2 to 29 943·6)	50 700 000 (46 000 000 to 55 700 000)	20 611·3 (18 699·2 to 22 650·5)	46 300 000 (42 000 000 to 50 600 000)	18 270·9 (16 562·3 to 19 978·4)	48 900 000 (44 400 000 to 53 700 000)	18 843·4 (17 118·0 to 20 687·0)	−23·6% (−27·3 to −19·8)	−8·6% (−11·6 to −4·8)
≥70 years	19 600 000 (17 200 000 to 22 100 000)	83 238·5 (73 340·4 to 94 207·4)	43 700 000 (39 100 000 to 49 700 000)	63 388·1 (56 714·2 to 71 959·6)	40 200 000 (35 800 000 to 45 200 000)	56 615·1 (50 503·2 to 63 669·3)	43 200 000 (38 200 000 to 49 200 000)	59 004·4 (52 213·3 to 67 237·2)	−23·8% (−28·8 to −18·3)	−6·9% (−11·2 to −2·4)
**Southeast Asia, East Asia, and Oceania**										
All ages	74 600 000 (69 600 000 to 79 900 000)	4418·3 (4117·6 to 4731·3)	75 400 000 (71 000 000 to 80 400 000)	3487·6 (3283·4 to 3716·3)	69 700 000 (65 500 000 to 74 100 000)	3204·6 (3012·0 to 3408·7)	67 300 000 (63 400 000 to 71 400 000)	3080·4 (2900·2 to 3268·2)	−21·1% (−25·7 to −16·4)	−11·7% (−13·0 to −10·1)
<5 years	28 900 000 (25 500 000 to 32 900 000)	16 514·4 (14 541·9 to 18 804·0)	6 830 000 (5 880 000 to 7 850 000)	4564·0 (3928·0 to 5243·7)	5 850 000 (5 010 000 to 6 860 000)	4023·1 (3441·5 to 4717·5)	4 880 000 (4 190 000 to 5 680 000)	3529·1 (3032·2 to 4105·8)	−72·4% (−74·1 to −70·6)	−22·7% (−24·4 to −20·8)
5–14 years	10 700 000 (8 720 000 to 13 000 000)	3253·2 (2656·2 to 3973·7)	6 240 000 (4 970 000 to 7 810 000)	2119·7 (1687·6 to 2649·8)	6 020 000 (4 790 000 to 7 510 000)	2002·8 (1593·2 to 2499·4)	5 940 000 (4 710 000 to 7 370 000)	1936·7 (1535·5 to 2402·1)	−34·8% (−40·2 to −28·5)	−8·6% (−10·5 to −6·7)
15–49 years	12 700 000 (11 400 000 to 14 100 000)	1364·0 (1226·0 to 1514·3)	13 500 000 (12 100 000 to 15 000 000)	1244·5 (1116·4 to 1382·7)	12 500 000 (11 100 000 to 13 800 000)	1160·4 (1034·9 to 1282·4)	11 900 000 (10 600 000 to 13 100 000)	1116·0 (995·9 to 1233·0)	−8·8% (−11·7 to −5·6)	−10·3% (−11·8 to −8·9)
50–69 years	11 900 000 (10 700 000 to 13 000 000)	5720·0 (5163·4 to 6283·5)	20 000 000 (18 200 000 to 21 800 000)	4053·7 (3682·9 to 4420·0)	19 000 000 (17 400 000 to 20 700 000)	3744·9 (3426·5 to 4080·0)	18 600 000 (16 800 000 to 20 200 000)	3571·8 (3236·8 to 3892·7)	−29·1% (−31·3 to −26·2)	−11·9% (−13·6 to −10·0)
≥70 years	10 500 000 (9 370 000 to 11 600 000)	21 063·0 (18 768·0 to 23 319·3)	28 800 000 (25 900 000 to 32 000 000)	20 500·9 (18 415·1 to 22 744·8)	26 300 000 (23 600 000 to 29 000 000)	17 997·9 (16 137·1 to 19 827·2)	26 000 000 (23 400 000 to 28 700 000)	16 906·4 (15 228·1 to 18 662·1)	−2·7% (−8·4 to 4·2)	−17·5% (−19·5 to −15·1)
**Sub-Saharan Africa**										
All ages	43 900 000 (41 000 000 to 46 800 000)	8936·4 (8335·8 to 9525·6)	64 200 000 (60 700 000 to 67 700 000)	5952·8 (5620·3 to 6277·1)	61 400 000 (58 000 000 to 64 600 000)	5546·3 (5245·1 to 5840·1)	62 000 000 (58 500 000 to 65 200 000)	5474·6 (5165·0 to 5753·4)	−33·4% (−35·3 to −31·3)	−8·0% (−9·6 to −6·5)
<5 years	17 500 000 (15 400 000 to 19 800 000)	19 478·0 (17 143·7 to 22 080·6)	15 000 000 (13 400 000 to 16 900 000)	8863·0 (7932·3 to 9960·6)	13 300 000 (11 800 000 to 15 000 000)	7766·1 (6918·0 to 8749·9)	13 200 000 (11 800 000 to 14 800 000)	7642·8 (6825·6 to 8583·4)	−54·5% (−55·8 to −53·1)	−13·8% (−16·0 to −11·4)
5–14 years	6 130 000 (5 060 000 to 7 350 000)	4547·4 (3752·5 to 5452·7)	8 490 000 (7 110 000 to 10 100 000)	2915·6 (2442·3 to 3475·8)	8 470 000 (7 130 000 to 10 000 000)	2849·9 (2399·7 to 3372·7)	8 320 000 (6 920 000 to 9 950 000)	2743·5 (2282·0 to 3281·2)	−35·9% (−38·4 to −32·6)	−5·9% (−8·0 to −3·7)
15–49 years	9 890 000 (8 990 000 to 10 800 000)	4511·4 (4100·7 to 4925·8)	18 900 000 (17 200 000 to 20 600 000)	3673·6 (3345·0 to 3992·1)	18 700 000 (17 100 000 to 20 300 000)	3526·2 (3227·0 to 3824·5)	19 200 000 (17 700 000 to 20 800 000)	3509·4 (3230·1 to 3809·5)	−18·6% (−21·0 to −15·6)	−4·5% (−6·0 to −2·8)
50–69 years	6 350 000 (5 730 000 to 6 990 000)	16 502·6 (14 891·8 to 18 163·4)	13 000 000 (11 800 000 to 14 200 000)	15 344·8 (13 882·5 to 16 730·5)	12 600 000 (11 500 000 to 13 800 000)	14 331·7 (13 087·7 to 15 656·8)	13 000 000 (11 900 000 to 14 200 000)	14 391·6 (13 110·3 to 15 695·7)	−7·0% (−10·5 to −3·8)	−6·2% (−7·8 to −4·6)
≥70 years	4 070 000 (3 620 000 to 4 560 000)	44 066·2 (39 216·4 to 49 342·5)	8 810 000 (7 940 000 to 9 880 000)	46 676·0 (42 070·1 to 52 387·7)	8 290 000 (7 480 000 to 9 300 000)	43 028·9 (38 829·9 to 48 260·3)	8 280 000 (7 500 000 to 9 260 000)	42 264·9 (38 278·7 to 47 260·2)	5·9% (0·7 to 12·1)	−9·5% (−11·2 to −7·6)

Values in parentheses are 95% uncertainty intervals. Count data are presented to three significant figures. GBD=Global Burden of Diseases, Injuries, and Risk Factors Study. SDI=Socio-demographic Index.

**Figure 1:**
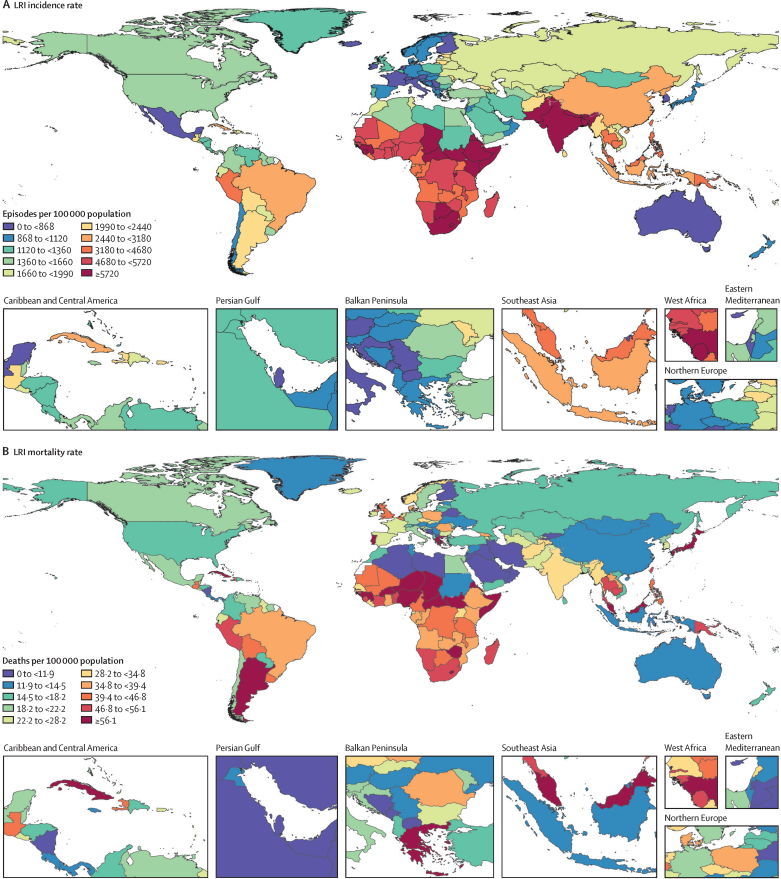
Global maps of LRI incidence and mortality rates across all ages, 2021 Maps show incidence rates (A) and mortality rates (B) per 100 000 population, with colours representing global deciles. LRI=lower respiratory infection.

In 2021, we estimated 344 million (325–364) incident episodes of LRI globally, for an all-age incidence rate of 4350 episodes (4120–4610) per 100 000 (table 1). Across 204 modelled locations, the all-age incidence rate in 2021 ranged from 463 episodes (428–500) per 100 000 in Cyprus to 9980 episodes (9220–10 800) per 100 000 in Nepal (figure 1; appendix 2 p 5). Adults aged 70 years and older had the highest global incidence rate at 18 900 episodes (17 100–21 000) per 100 000, followed by adults aged 50–69 years at 6370 episodes (5800–6940) per 100 000 (table 1). Among children younger than 5 years, we estimated 37·8 million (33·5–43·0) incident episodes of LRI and an incidence rate of 5750 episodes (5090–6540) per 100 000 (table 1), ranging from 413 episodes (335–504) per 100 000 in the Netherlands to 12 190 episodes (10 600–13 900) per 100 000 in Pakistan (appendix 2 p 5).

Since 1990, the all-age global LRI incidence rate decreased 19·0% (95% UI 16·0–21·9), from 5880 (5510–6250) episodes per 100 000 in 1990 to 4770 episodes (4510–5040) per 100 000 in 2019 (table 1). This decline was primarily attributable to reductions in incidence among children younger than 5 years, which decreased 59·3% (58·3–60·1), from 16 300 episodes (14 500–18 300) per 100 000 in 1990 to 6640 episodes (5900–7490) per 100 000 in 2019 (table 1). By contrast, the global incidence rate among adults aged 70 years and older declined at a lower rate from 1990 to 2019, with an overall decrease of 4·8% (0·4–9·1; table 1).

### Mortality of LRIs

Globally in 2019, before reductions in mortality observed during the COVID-19 pandemic, we estimated 2·55 million (95% UI 2·32–2·74) global LRI deaths and an all-age mortality rate of 32·9 deaths (29·9–35·4) per 100 000 population, representing a 41·7% decrease (35·9–46·9) in mortality rate since 1990 (table 2). Among children younger than 5 years, we estimated 693 000 (580 000–822 000) deaths, for a mortality rate of 102·2 deaths (85·5–121·3) per 100 000 in this age group in 2019 (table 2).

**Table 2:** Lower respiratory infection mortality counts and rates for all-ages and selected age groups in 1990, 2019, 2020, and 2021, and mortality rate percentage change from 1990 to 2019 and from 2019 to 2021, globally and by SDI quintile and GBD super-region

	**1990**		**2019**		**2020**		**2021**		**Mortality rate change, %**	
	Death count	Mortality rate per 100 000 population	Death count	Mortality rate per 100 000 population	Death count	Mortality rate per 100 000 population	Death count	Mortality rate per 100 000 population	1990–2019	2019–21
**Global**										
All ages	3 010 000 (2 730 000 to 3 300 000)	56·5 (51·3 to 61·9)	2 550 000 (2 320 000 to 2 740 000)	32·9 (29·9 to 35·4)	2 280 000 (2 080 000 to 2 460 000)	29·1 (26·5 to 31·4)	2 180 000 (1 980 000 to 2 360 000)	27·7 (25·1 to 29·9)	−41·7% (−46·9 to −35·9)	−16·0% (−18·6 to −13·1)
<5 years	1 940 000 (1 690 000 to 2 230 000)	313·7 (272·4 to 359·3)	693 000 (580 000 to 822 000)	102·2 (85·5 to 121·3)	557 000 (455 000 to 665 000)	83·1 (67·9 to 99·3)	502 000 (406 000 to 611 000)	76·2 (61·7 to 92·9)	−67·4% (−72·2 to −61·3)	−25·4% (−30·0 to −20·3)
5–14 years	89 000 (74 200 to 99 900)	7·9 (6·6 to 8·9)	51 900 (45 300 to 58 500)	3·9 (3·4 to 4·4)	46 100 (40 100 to 51 900)	3·4 (3·0 to 3·9)	43 700 (37 600 to 49 400)	3·2 (2·8 to 3·7)	−50·8% (−56·3 to −42·5)	−17·4% (−21·2 to −13·6)
15–49 years	141 000 (130 000 to 150 000)	5·2 (4·8 to 5·5)	174 000 (161 000 to 189 000)	4·5 (4·1 to 4·9)	162 000 (150 000 to 176 000)	4·1 (3·8 to 4·5)	160 000 (147 000 to 175 000)	4·1 (3·7 to 4·4)	−14·2% (−19·6 to −7·3)	−9·0% (−12·1 to −5·7)
50–69 years	243 000 (224 000 to 261 000)	35·6 (32·8 to 38·2)	394 000 (367 000 to 421 000)	28·6 (26·7 to 30·6)	373 000 (345 000 to 400 000)	26·5 (24·5 to 28·4)	367 000 (335 000 to 394 000)	25·5 (23·3 to 27·4)	−19·6% (−24·8 to −13·2)	−10·9% (−14·2 to −7·2)
≥70 years	596 000 (542 000 to 642 000)	295·0 (268·1 to 318·0)	1 240 000 (1 100 000 to 1 330 000)	266·3 (236·5 to 287·2)	1 140 000 (1 020 000 to 1 230 000)	238·5 (212·1 to 256·7)	1 110 000 (978 000 to 1 200 000)	224·6 (197·8 to 243·7)	−9·7% (−14·7 to −4·5)	−15·7% (−18·2 to −12·9)
**High SDI**										
All ages	269 000 (244 000 to 281 000)	30·6 (27·7 to 32·0)	363 000 (308 000 to 393 000)	33·4 (28·4 to 36·1)	332 000 (284 000 to 359 000)	30·4 (26·0 to 32·9)	299 000 (252 000 to 325 000)	27·4 (23·0 to 29·7)	9·2% (2·0 to 13·4)	−18·0% (−19·2 to −17·0)
<5 years	8370 (7650 to 9280)	13·6 (12·4 to 15·0)	1750 (1640 to 1870)	3·1 (2·9 to 3·3)	1350 (1220 to 1470)	2·5 (2·2 to 2·7)	998 (898 to 1080)	1·9 (1·7 to 2·0)	−76·9% (−79·5 to −74·9)	−40·8% (−45·0 to −37·1)
5–14 years	1360 (1270 to 1460)	1·1 (1·0 to 1·2)	469 (448 to 497)	0·4 (0·4 to 0·4)	398 (374 to 428)	0·3 (0·3 to 0·4)	354 (333 to 381)	0·3 (0·3 to 0·3)	−63·9% (−66·5 to −61·1)	−24·9% (−27·2 to −22·8)
15–49 years	10 500 (10 200 to 10 700)	2·3 (2·2 to 2·3)	9230 (8760 to 9810)	1·8 (1·7 to 1·9)	8350 (7840 to 8970)	1·7 (1·6 to 1·8)	7330 (6850 to 7910)	1·5 (1·4 to 1·6)	−19·8% (−24·3 to −14·8)	−20·1% (−22·3 to −17·6)
50–69 years	31 000 (30 200 to 31 600)	18·9 (18·5 to 19·3)	37 500 (36 200 to 38 500)	13·8 (13·3 to 14·2)	34 300 (33 100 to 35 400)	12·5 (12·1 to 12·9)	31 100 (29 900 to 32 200)	11·3 (10·8 to 11·7)	−27·1% (−28·9 to −25·3)	−18·3% (−19·8 to −16·7)
≥70 years	218 000 (192 000 to 230 000)	315·2 (278·4 to 332·6)	314 000 (260 000 to 343 000)	231·4 (191·6 to 252·8)	288 000 (239 000 to 313 000)	205·7 (171·2 to 224·0)	260 000 (213 000 to 284 000)	180·9 (148·2 to 198·0)	−26·6% (−31·8 to −23·6)	−21·8% (−22·9 to −20·8)
**High-middle SDI**										
All ages	248 000 (231 000 to 268 000)	23·3 (21·7 to 25·2)	275 000 (249 000 to 296 000)	21·2 (19·2 to 22·8)	252 000 (226 000 to 272 000)	19·3 (17·4 to 20·9)	242 000 (216 000 to 266 000)	18·5 (16·6 to 20·4)	−9·0% (−17·0 to −0·9)	−12·7% (−17·4 to −7·3)
<5 years	114 000 (101 000 to 131 000)	122·3 (108·3 to 140·9)	9000 (7880 to 10 300)	11·8 (10·3 to 13·4)	7190 (6200 to 8300)	9·7 (8·4 to 11·2)	6000 (5050 to 7020)	8·6 (7·2 to 10·0)	−90·4% (−92·2 to −88·6)	−27·0% (−31·6 to −22·7)
5–14 years	5900 (5370 to 6460)	3·3 (3·0 to 3·6)	1520 (1410 to 1700)	1·0 (0·9 to 1·1)	1280 (1180 to 1440)	0·8 (0·7 to 0·9)	1210 (1110 to 1380)	0·8 (0·7 to 0·9)	−69·9% (−72·9 to −65·9)	−23·5% (−26·5 to −20·8)
15–49 years	17 100 (16 000 to 18 100)	3·0 (2·8 to 3·2)	20 400 (19 600 to 21 300)	3·2 (3·1 to 3·3)	18 400 (17 600 to 19 400)	2·9 (2·8 to 3·1)	17 200 (16 200 to 18 400)	2·7 (2·6 to 2·9)	5·0% (−1·5 to 12·6)	−14·0% (−19·7 to −7·9)
50–69 years	29 700 (27 800 to 31 700)	17·1 (16·0 to 18·2)	48 000 (45 700 to 50 500)	15·2 (14·5 to 16·0)	44 700 (42 300 to 47 400)	13·9 (13·2 to 14·8)	42 300 (39 600 to 45 300)	13·0 (12·1 to 13·9)	−10·7% (−17·1 to −3·7)	−14·8% (−19·9 to −8·8)
≥70 years	81 700 (73 800 to 88 600)	158·7 (143·5 to 172·1)	197 000 (171 000 to 216 000)	178·9 (156·0 to 197·0)	180 000 (156 000 to 198 000)	158·7 (137·8 to 174·3)	175 000 (151 000 to 196 000)	149·1 (128·3 to 167·0)	12·7% (4·3 to 21·4)	−16·7% (−21·6 to −11·0)
**Middle SDI**										
All ages	777 000 (715 000 to 840 000)	45·1 (41·5 to 48·8)	605 000 (555 000 to 647 000)	25·1 (23·0 to 26·8)	548 000 (502 000 to 588 000)	22·5 (20·6 to 24·2)	543 000 (494 000 to 589 000)	22·2 (20·2 to 24·1)	−44·3% (−48·9 to −39·2)	−11·7% (−16·1 to −7·3)
<5 years	509 000 (457 000 to 568 000)	253·9 (227·7 to 283·2)	90 900 (78 300 to 106 000)	48·5 (41·8 to 56·4)	70 200 (59 900 to 82 000)	38·3 (32·7 to 44·8)	60 400 (50 800 to 71 200)	34·2 (28·8 to 40·3)	−80·9% (−83·8 to −77·4)	−29·4% (−34·0 to −24·6)
5–14 years	26 300 (22 100 to 28 600)	7·0 (5·9 to 7·6)	9510 (8610 to 10 700)	2·5 (2·3 to 2·8)	8170 (7440 to 9070)	2·1 (1·9 to 2·3)	7660 (6950 to 8440)	2·0 (1·8 to 2·2)	−64·3% (−68·2 to −57·6)	−21·2% (−24·9 to −17·3)
15–49 years	46 100 (42 500 to 48 900)	5·1 (4·7 to 5·4)	47 000 (44 800 to 50 000)	3·8 (3·6 to 4·0)	42 700 (40 500 to 45 300)	3·4 (3·2 to 3·6)	42 700 (40 000 to 45 900)	3·4 (3·2 to 3·7)	−25·7% (−30·5 to −20·1)	−9·5% (−14·1 to −4·9)
50–69 years	60 400 (55 100 to 65 500)	31·9 (29·1 to 34·6)	111 000 (104 000 to 117 000)	24·1 (22·5 to 25·4)	105 000 (97 800 to 112 000)	22·3 (20·6 to 23·7)	106 000 (97 300 to 114 000)	21·8 (20·0 to 23·4)	−24·5% (−30·5 to −17·2)	−9·5% (−14·7 to −4·5)
≥70 years	135 000 (122 000 to 149 000)	295·5 (266·1 to 326·0)	348 000 (309 000 to 376 000)	265·6 (236·1 to 287·7)	321 000 (284 000 to 349 000)	237·0 (209·5 to 258·0)	326 000 (288 000 to 358 000)	231·5 (204·3 to 254·2)	−10·1% (−17·4 to −1·8)	−12·9% (−17·5 to −7·8)
**Low-middle SDI**										
All ages	954 000 (850 000 to 1 070 000)	82·1 (73·2 to 91·9)	712 000 (641 000 to 777 000)	37·9 (34·2 to 41·4)	619 000 (558 000 to 680 000)	32·6 (29·4 to 35·8)	594 000 (528 000 to 657 000)	30·9 (27·5 to 34·2)	−53·8% (−59·2 to −47·8)	−18·5% (−22·8 to −13·5)
<5 years	719 000 (626 000 to 827 000)	414·3 (360·7 to 476·8)	263 000 (222 000 to 307 000)	134·3 (113·6 to 156·9)	200 000 (168 000 to 238 000)	103·4 (86·7 to 122·8)	180 000 (148 000 to 215 000)	94·0 (77·5 to 112·4)	−67·6% (−73·0 to −61·3)	−30·0% (−35·9 to −23·2)
5–14 years	31 500 (26 000 to 36 500)	10·6 (8·7 to 12·2)	17 300 (15 000 to 19 700)	4·5 (3·9 to 5·1)	15 000 (12 900 to 17 100)	3·9 (3·3 to 4·4)	14 200 (12 100 to 16 300)	3·6 (3·1 to 4·2)	−57·6% (−63·5 to −49·2)	−18·5% (−23·0 to −13·9)
15–49 years	37 200 (33 900 to 42 200)	6·7 (6·1 to 7·7)	50 400 (45 400 to 56 900)	5·1 (4·6 to 5·8)	47 400 (42 600 to 53 600)	4·7 (4·3 to 5·4)	47 200 (41 900 to 53 500)	4·6 (4·1 to 5·3)	−24·4% (−31·0 to −15·8)	−9·0% (−13·8 to −4·1)
50–69 years	68 000 (60 400 to 75 700)	60·8 (54·0 to 67·6)	123 000 (110 000 to 135 000)	50·8 (45·7 to 56·0)	116 000 (103 000 to 129 000)	46·5 (41·4 to 51·8)	114 000 (100 000 to 127 000)	44·8 (39·3 to 50·0)	−16·5% (−25·4 to −4·8)	−11·7% (−17·0 to −5·5)
≥70 years	98 100 (85 900 to 114 000)	374·0 (327·5 to 435·3)	259 000 (232 000 to 286 000)	388·4 (348·6 to 428·5)	241 000 (215 000 to 268 000)	352·6 (314·0 to 391·8)	238 000 (209 000 to 268 000)	340·2 (298·2 to 382·1)	3·8% (−8·4 to 17·7)	−12·4% (−17·3 to −7·2)
**Low SDI**										
All ages	763 000 (644 000 to 891 000)	152·2 (128·5 to 177·7)	591 000 (512 000 to 681 000)	55·4 (48·0 to 63·8)	527 000 (452 000 to 611 000)	48·3 (41·4 to 56·0)	503 000 (430 000 to 582 000)	45·0 (38·5 to 52·1)	−63·6% (−68·8 to −57·2)	−18·9% (−22·5 to −15·1)
<5 years	593 000 (477 000 to 726 000)	653·5 (525·6 to 800·1)	328 000 (262 000 to 403 000)	202·5 (162·0 to 249·0)	277 000 (217 000 to 346 000)	169·3 (132·5 to 211·1)	254 000 (197 000 to 320 000)	153·2 (118·7 to 193·4)	−69·0% (−74·4 to −62·1)	−24·3% (−29·4 to −19·3)
5–14 years	23 800 (18 100 to 28 600)	17·3 (13·1 to 20·7)	23 100 (19 200 to 26 900)	8·1 (6·7 to 9·4)	21 200 (17 500 to 24 600)	7·3 (6·0 to 8·5)	20 300 (16 700 to 23 900)	6·9 (5·7 to 8·1)	−53·2% (−60·5 to −41·8)	−14·7% (−19·2 to −9·7)
15–49 years	29 800 (25 600 to 33 700)	13·5 (11·6 to 15·2)	46 600 (40 300 to 54 000)	9·1 (7·9 to 10·6)	44 900 (38 900 to 52 000)	8·5 (7·4 to 9·9)	45 600 (39 200 to 52 700)	8·4 (7·2 to 9·7)	−32·4% (−40·1 to −22·8)	−7·8% (−12·3 to −3·3)
50–69 years	53 400 (46 100 to 60 500)	127·2 (109·8 to 144·1)	74 800 (65 300 to 85 400)	85·9 (75·0 to 98·1)	72 600 (63 300 to 83 000)	80·4 (70·2 to 92·0)	72 900 (63 200 to 83 500)	78·5 (68·1 to 90·0)	−32·5% (−39·9 to −23·3)	−8·6% (−12·9 to −4·4)
≥70 years	62 800 (54 500 to 72 200)	673·0 (584·5 to 773·7)	119 000 (106 000 to 136 000)	567·4 (504·6 to 647·7)	111 000 (99 300 to 126 000)	521·0 (464·4 to 590·1)	110 000 (97 900 to 126 000)	501·7 (446·2 to 572·5)	−15·7% (−24·7 to −5·3)	−11·6% (−15·9 to −7·4)
**Central Europe, Eastern Europe, and Central Asia**										
All ages	108 000 (104 000 to 112 000)	25·6 (24·7 to 26·7)	102 000 (96 600 to 106 000)	24·3 (23·1 to 25·3)	96 200 (91 200 to 101 000)	23·0 (21·8 to 24·1)	82 800 (77 800 to 87 500)	19·8 (18·6 to 21·0)	−5·3% (−9·8 to −0·9)	−18·4% (−21·5 to −15·2)
<5 years	63 600 (60 000 to 67 600)	177·0 (167·0 to 188·2)	16 200 (13 700 to 19 000)	59·9 (50·8 to 70·2)	14 500 (12 300 to 17 000)	55·1 (46·7 to 64·5)	11 000 (9240 to 13 200)	43·0 (35·9 to 51·3)	−66·2% (−71·1 to −60·2)	−28·3% (−32·0 to −24·5)
5–14 years	2640 (2520 to 2740)	3·8 (3·6 to 3·9)	1460 (1330 to 1610)	2·7 (2·5 to 3·0)	1370 (1240 to 1500)	2·5 (2·3 to 2·8)	1190 (1080 to 1320)	2·2 (2·0 to 2·4)	−27·5% (−33·9 to −20·0)	−20·9% (−23·3 to −18·2)
15–49 years	8800 (8620 to 8980)	4·3 (4·2 to 4·4)	14 600 (14 100 to 15 200)	7·3 (7·0 to 7·6)	13 600 (13 000 to 14 300)	6·9 (6·6 to 7·2)	12 200 (11 300 to 13 300)	6·2 (5·7 to 6·7)	70·7% (64·2 to 78·3)	−15·2% (−21·7 to −8·5)
50–69 years	13 800 (13 500 to 14 100)	16·5 (16·1 to 16·8)	26 200 (25 400 to 27 000)	26·1 (25·4 to 26·9)	24 700 (23 700 to 25 700)	24·7 (23·7 to 25·7)	21 900 (20 600 to 23 500)	22·0 (20·6 to 23·5)	58·9% (53·7 to 64·3)	−15·8% (−21·0 to −10·6)
≥70 years	19 200 (18 100 to 19 900)	75·7 (71·4 to 78·5)	43 200 (39 500 to 45 300)	113·8 (103·9 to 119·2)	42 000 (38 100 to 44 700)	107·9 (97·8 to 114·7)	36 400 (32 900 to 38 600)	91·9 (82·9 to 97·6)	50·4% (43·8 to 56·0)	−19·3% (−21·9 to −16·5)
**High-income**										
All ages	280 000 (252 000 to 293 000)	30·8 (27·7 to 32·2)	400 000 (339 000 to 432 000)	36·8 (31·2 to 39·8)	361 000 (306 000 to 390 000)	33·1 (28·1 to 35·8)	321 000 (267 000 to 348 000)	29·4 (24·5 to 31·9)	19·6% (11·8 to 24·2)	−20·2% (−21·2 to −19·3)
<5 years	6180 (5970 to 6410)	10·0 (9·7 to 10·4)	1640 (1570 to 1720)	2·9 (2·8 to 3·0)	1180 (1070 to 1270)	2·1 (1·9 to 2·3)	855 (760 to 943)	1·6 (1·4 to 1·7)	−71·1% (−72·4 to −69·5)	−45·8% (−51·1 to −40·8)
5–14 years	1040 (976 to 1100)	0·8 (0·8 to 0·9)	439 (427 to 451)	0·4 (0·3 to 0·4)	358 (343 to 375)	0·3 (0·3 to 0·3)	327 (310 to 343)	0·3 (0·3 to 0·3)	−56·6% (−59·2 to −53·6)	−25·2% (−28·3 to −22·5)
15–49 years	9940 (9780 to 10 100)	2·1 (2·1 to 2·1)	8020 (7850 to 8210)	1·6 (1·6 to 1·7)	6950 (6710 to 7190)	1·4 (1·4 to 1·5)	6040 (5860 to 6220)	1·2 (1·2 to 1·3)	−22·1% (−24·1 to −20·1)	−24·3% (−25·7 to −22·8)
50–69 years	31 300 (30 500 to 31 800)	17·8 (17·4 to 18·2)	37 200 (36 000 to 38 200)	13·6 (13·2 to 14·0)	33 700 (32 600 to 34 700)	12·2 (11·8 to 12·6)	30 300 (29 200 to 31 300)	10·9 (10·5 to 11·3)	−23·7% (−25·7 to −21·7)	−19·8% (−20·9 to −18·6)
≥70 years	231 000 (204 000 to 244 000)	304·4 (268·8 to 321·5)	352 000 (292 000 to 384 000)	241·2 (199·9 to 263·2)	318 000 (264 000 to 347 000)	212·3 (176·1 to 231·3)	283 000 (231 000 to 310 000)	184·9 (150·5 to 202·3)	−20·8% (−26·1 to −17·7)	−23·4% (−24·3 to −22·5)
**Latin America and Caribbean**										
All ages	166 000 (158 000 to 174 000)	42·6 (40·6 to 44·7)	215 000 (195 000 to 228 000)	36·7 (33·2 to 39·0)	187 000 (169 000 to 200 000)	31·7 (28·7 to 33·9)	177 000 (157 000 to 194 000)	29·8 (26·5 to 32·6)	−13·9% (−21·0 to −8·2)	−18·9% (−22·5 to −14·8)
<5 years	89 000 (82 600 to 95 800)	179·7 (166·8 to 193·6)	20 100 (16 400 to 23 800)	41·2 (33·8 to 48·8)	14 400 (11 700 to 17 400)	30·0 (24·5 to 36·1)	12 200 (9570 to 15 200)	25·7 (20·2 to 32·1)	−77·0% (−81·2 to −72·7)	−37·6% (−44·2 to −30·5)
5–14 years	4890 (4640 to 5130)	5·2 (4·9 to 5·4)	2250 (2010 to 2500)	2·4 (2·1 to 2·6)	1820 (1630 to 2030)	1·9 (1·7 to 2·1)	1620 (1430 to 1850)	1·7 (1·5 to 1·9)	−54·4% (−59·2 to −49·5)	−28·0% (−33·8 to −22·1)
15–49 years	13 000 (12 600 to 13 400)	6·6 (6·4 to 6·8)	18 000 (17 300 to 18 800)	5·8 (5·6 to 6·1)	16 000 (15 100 to 16 900)	5·1 (4·9 to 5·4)	15 500 (14 500 to 16 900)	5·0 (4·6 to 5·4)	−11·7% (−15·6 to −7·5)	−14·5% (−18·9 to −9·8)
50–69 years	16 000 (15 400 to 16 500)	42·0 (40·5 to 43·5)	38 400 (36 600 to 39 900)	38·5 (36·8 to 40·1)	36 200 (34 400 to 38 300)	35·4 (33·6 to 37·4)	35 400 (32 800 to 38 400)	33·9 (31·4 to 36·7)	−8·2% (−12·4 to −4·1)	−12·1% (−16·7 to −7·1)
≥70 years	43 500 (40 100 to 45 600)	398·9 (367·7 to 417·9)	136 000 (119 000 to 146 000)	417·6 (363·7 to 446·6)	119 000 (103 000 to 128 000)	353·3 (306·4 to 379·4)	112 000 (96 000 to 123 000)	325·1 (278·3 to 356·4)	4·7% (−1·5 to 9·3)	−22·1% (−25·8 to −18·3)
**North Africa and Middle East**										
All ages	181 000 (159 000 to 218 000)	53·3 (46·8 to 64·2)	113 000 (102 000 to 126 000)	18·7 (16·8 to 20·7)	103 000 (91 000 to 116 000)	16·8 (14·8 to 18·9)	92 200 (81 400 to 105 000)	14·8 (13·1 to 16·8)	−64·9% (−70·7 to −60·0)	−20·9% (−24·8 to −16·5)
<5 years	138 000 (118 000 to 176 000)	270·2 (230·0 to 343·4)	32 700 (26 900 to 39 900)	51·4 (42·2 to 62·7)	25 900 (21 000 to 31 300)	41·4 (33·6 to 50·1)	20 200 (16 600 to 24 600)	33·1 (27·1 to 40·3)	−81·0% (−84·8 to −77·0)	−35·6% (−41·6 to −28·8)
5–14 years	7290 (6190 to 8320)	8·2 (6·9 to 9·3)	3610 (3010 to 4380)	3·0 (2·5 to 3·7)	3200 (2660 to 3900)	2·7 (2·2 to 3·2)	2880 (2350 to 3550)	2·4 (1·9 to 2·9)	−62·7% (−70·0 to −55·2)	−22·8% (−27·8 to −17·8)
15–49 years	7660 (6870 to 8970)	4·8 (4·3 to 5·6)	11 800 (10 400 to 13 300)	3·6 (3·2 to 4·1)	11 100 (9600 to 12 800)	3·4 (2·9 to 3·9)	10 500 (8980 to 12 200)	3·1 (2·7 to 3·6)	−24·4% (−32·7 to −15·3)	−13·4% (−18·2 to −8·6)
50–69 years	9840 (8750 to 11 400)	31·5 (28·0 to 36·6)	18 700 (16 600 to 20 800)	23·6 (21·0 to 26·4)	18 300 (16 000 to 20 700)	22·3 (19·5 to 25·3)	17 600 (15 100 to 20 500)	20·7 (17·7 to 24·1)	−25·0% (−34·0 to −15·6)	−12·5% (−18·9 to −6·1)
≥70 years	17 600 (15 300 to 21 400)	243·0 (211·8 to 296·3)	46 600 (40 300 to 51 500)	241·9 (209·1 to 267·6)	44 600 (38 400 to 50 200)	224·8 (193·8 to 253·1)	41 000 (35 300 to 46 200)	201·6 (173·4 to 227·0)	−0·5% (−15·0 to 11·3)	−16·6% (−20·3 to −12·6)
**South Asia**										
All ages	802 000 (696 000 to 902 000)	73·3 (63·7 to 82·5)	609 000 (548 000 to 674 000)	33·7 (30·3 to 37·3)	522 000 (465 000 to 582 000)	28·5 (25·4 to 31·8)	516 000 (451 000 to 584 000)	27·9 (24·4 to 31·6)	−54·1% (−60·3 to −46·5)	−17·1% (−24·2 to −9·2)
<5 years	610 000 (516 000 to 707 000)	388·6 (328·5 to 450·2)	229 000 (191 000 to 273 000)	140·6 (117·4 to 167·3)	167 000 (135 000 to 202 000)	103·6 (84·2 to 125·9)	154 000 (124 000 to 190 000)	97·3 (78·5 to 119·7)	−63·8% (−70·8 to −54·9)	−30·8% (−39·5 to −20·1)
5–14 years	27 900 (21 800 to 33 400)	10·1 (7·9 to 12·1)	13 000 (10 800 to 15 300)	3·7 (3·1 to 4·4)	10 800 (8890 to 12 800)	3·1 (2·5 to 3·6)	10 200 (8360 to 12 100)	2·9 (2·4 to 3·5)	−63·4% (−69·9 to −53·9)	−20·4% (−27·6 to −12·6)
15–49 years	27 200 (24 300 to 32 700)	5·1 (4·6 to 6·2)	31 300 (28 100 to 37 600)	3·2 (2·9 to 3·8)	30 000 (26 500 to 35 800)	3·0 (2·7 to 3·6)	30 800 (26 600 to 37 100)	3·1 (2·6 to 3·7)	−37·7% (−44·2 to −29·7)	−4·5% (−14·4 to 6·2)
50–69 years	58 900 (51 000 to 68 000)	54·8 (47·5 to 63·3)	108 000 (95 200 to 122 000)	44·1 (38·8 to 49·7)	101 000 (88 600 to 116 000)	39·9 (35·0 to 45·9)	101 000 (86 800 to 117 000)	38·9 (33·4 to 45·2)	−19·6% (−29·5 to −6·7)	−11·8% (−21·0 to −1·5)
≥70 years	77 600 (64 200 to 93 800)	330·4 (273·4 to 399·3)	227 000 (199 000 to 257 000)	329·4 (288·5 to 372·3)	213 000 (186 000 to 244 000)	300·7 (262·1 to 344·2)	219 000 (187 000 to 257 000)	299·7 (255·5 to 350·5)	−0·3% (−14·1 to 18·0)	−9·0% (−17·2 to −0·4)
**Southeast Asia, East Asia, and Oceania**										
All ages	734 000 (666 000 to 809 000)	43·5 (39·4 to 47·9)	455 000 (410 000 to 499 000)	21·1 (19·0 to 23·1)	424 000 (378 000 to 469 000)	19·5 (17·4 to 21·6)	431 000 (384 000 to 482 000)	19·7 (17·6 to 22·0)	−51·5% (−56·9 to −45·6)	−6·2% (−13·6 to 2·1)
<5 years	486 000 (426 000 to 557 000)	277·7 (243·2 to 318·4)	57 700 (48 400 to 68 100)	38·5 (32·3 to 45·5)	48 700 (40 900 to 57 400)	33·4 (28·1 to 39·5)	41 700 (34 400 to 49 200)	30·1 (24·9 to 35·6)	−86·1% (−88·5 to −83·2)	−21·8% (−26·1 to −17·0)
5–14 years	23 400 (19 000 to 26 000)	7·1 (5·8 to 7·9)	5790 (5130 to 6860)	2·0 (1·7 to 2·3)	5070 (4530 to 5970)	1·7 (1·5 to 2·0)	4810 (4220 to 5640)	1·6 (1·4 to 1·8)	−72·4% (−76·3 to −64·5)	−20·3% (−24·1 to −15·9)
15–49 years	36 000 (31 700 to 39 600)	3·9 (3·4 to 4·3)	26 200 (24 100 to 29 300)	2·4 (2·2 to 2·7)	23 900 (21 700 to 26 800)	2·2 (2·0 to 2·5)	23 500 (21 000 to 26 200)	2·2 (2·0 to 2·5)	−37·5% (−45·0 to −28·8)	−9·1% (−17·0 to −0·5)
50–69 years	55 200 (48 400 to 61 800)	26·6 (23·3 to 29·8)	72 600 (65 700 to 79 600)	14·7 (13·3 to 16·1)	69 500 (62 700 to 76 500)	13·7 (12·3 to 15·1)	69 800 (62 000 to 77 700)	13·4 (11·9 to 15·0)	−44·7% (−51·9 to −36·3)	−8·6% (−17·3 to 1·4)
≥70 years	134 000 (116 000 to 150 000)	267·5 (232·5 to 300·6)	293 000 (257 000 to 327 000)	208·4 (182·3 to 232·3)	276 000 (239 000 to 309 000)	188·9 (163·6 to 211·5)	292 000 (252 000 to 331 000)	189·5 (163·6 to 214·7)	−22·1% (−30·8 to −12·0)	−9·1% (−17·0 to 0·1)
**Sub-Saharan Africa**										
All ages	742 000 (629 000 to 875 000)	151·1 (127·9 to 178·0)	655 000 (557 000 to 757 000)	60·7 (51·6 to 70·1)	588 000 (494 000 to 686 000)	53·1 (44·6 to 62·0)	563 000 (472 000 to 655 000)	49·7 (41·6 to 57·8)	−59·8% (−65·1 to −53·2)	−18·2% (−21·9 to −14·1)
<5 years	551 000 (443 000 to 683 000)	614·5 (494·0 to 761·5)	335 000 (261 000 to 418 000)	197·9 (153·7 to 246·8)	285 000 (217 000 to 361 000)	166·6 (126·7 to 211·0)	261 000 (197 000 to 334 000)	151·2 (113·9 to 193·4)	−67·8% (−73·4 to −60·8)	−23·6% (−28·6 to −18·3)
5–14 years	21 900 (17 000 to 26 300)	16·2 (12·6 to 19·5)	25 400 (20 700 to 30 100)	8·7 (7·1 to 10·3)	23 500 (18 900 to 27 800)	7·9 (6·4 to 9·4)	22 700 (18 100 to 27 100)	7·5 (6·0 to 8·9)	−46·3% (−54·5 to −33·2)	−14·2% (−19·0 to −8·6)
15–49 years	38 100 (33 000 to 42 300)	17·4 (15·0 to 19·3)	63 800 (55 200 to 73 300)	12·4 (10·7 to 14·2)	60 400 (52 000 to 69 700)	11·4 (9·8 to 13·1)	61 500 (53 200 to 71 000)	11·3 (9·7 to 13·0)	−28·8% (−36·6 to −18·8)	−9·2% (−13·9 to −3·9)
50–69 years	57 700 (50 100 to 65 400)	150·0 (130·3 to 169·8)	92 600 (81 100 to 105 000)	109·2 (95·7 to 123·8)	89 600 (78 200 to 102 000)	102·1 (89·1 to 115·7)	90 600 (78 900 to 103 000)	100·1 (87·2 to 113·7)	−27·2% (−35·0 to −16·6)	−8·4% (−12·6 to −3·9)
≥70 years	73 300 (64 500 to 83 000)	793·6 (698·0 to 898·2)	138 000 (124 000 to 155 000)	732·9 (656·0 to 820·7)	129 000 (115 000 to 143 000)	668·2 (595·9 to 742·1)	127 000 (113 000 to 141 000)	646·4 (576·2 to 718·2)	−7·6% (−16·2 to 2·8)	−11·8% (−15·7 to −7·6)

Values in parentheses are 95% uncertainty intervals. Count data are presented to three significant figures. GBD=Global Burden of Diseases, Injuries, and Risk Factors Study. SDI=Socio-demographic Index.

In 2021, we estimated 2·18 million (1·98–2·36) deaths globally due to LRI and an all-age mortality rate of 27·7 deaths (25·1–29·9) per 100 000 (table 2). The all-age mortality rate ranged from 2·3 deaths (1·8–2·9) per 100 000 in Qatar to 104·0 deaths (81·8–129·2) per 100 000 in Chad (figure 1; appendix 2 p 86). Among children younger than 5 years, we estimated 502 000 deaths (406 000–611 000) due to LRI globally, or 76·2 deaths (61·7–92·9) per 100 000 (table 2), ranging from 0·3 deaths (0·2–0·5) per 100 000 in Andorra to 357·9 deaths (271·4–456·4) per 100 000 in Chad (appendix 2 p 86). Across the aggregated age groups, adults aged 70 years and older had the highest global mortality rate (224·6 deaths [197·8–243·7] per 100 000), followed by children younger than 5 years (table 2).

LRI fatalities in 2021, especially among children, were concentrated in countries with a low Socio-demographic Index (SDI; appendix 2 p 5).^38^ Of 204 modelled countries and territories, 57 had an LRI mortality rate greater than 60 per 100 000 among children younger than 5 years in 2021 (appendix 2 p 86). In 2021, among children younger than 5 years, mortality rates per 100 000 population were 153·2 deaths (118·7–193·4) in low SDI countries, 94·0 (77·5–112·4) in low-middle SDI countries, 34·2 (28·8–40·3) in middle SDI countries, 8·6 (7·2–10·0) in high-middle SDI countries, and 1·9 (1·7–2·0) in high SDI countries (table 2). In total, 254 000 LRI deaths (197 000–320 000) in children younger than 5 years occurred in low SDI countries (table 2). However, although the low SDI quintile had the highest burden in 2021, these countries also showed the greatest improvement in all-age mortality rates over time (table 2).

**Figure 2:**
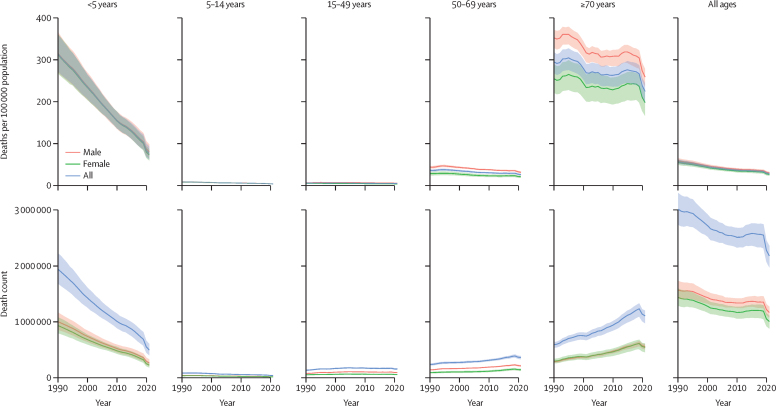
LRI mortality rates and death counts by age and sex, 1990–2021 Upper graphs show mortality rates per 100 000 population. Lower graphs show death counts. Shaded areas represent 95% uncertainty intervals. LRI=lower respiratory infection.

Globally, between 1990 and 2021, the all-age LRI mortality rate decreased by 50·9% (95% UI 45·6–55·9), from 56·5 deaths (51·3–61·9) to 27·7 deaths (25·1–29·9) per 100 000 population (figure 2). For males, it decreased by 49·4% (44·0–54·4), from 58·6 deaths (53·0–64·6) to 29·6 deaths (27·2–32·1) per 100 000. For females, it decreased by 52·7% (46·3–58·6), from 54·4 deaths (48·8–60·5) to 25·7 deaths (22·5–28·3) per 100 000 (figure 2). Analogous to incidence, the decline in mortality was largely attributable to reductions in deaths among children; LRI mortality rate decreased by 75·6% (70·7–79·8) in children younger than 5 years and 59·2% (52·7–64·2) in children aged 5–14 years (figure 2). Adults aged 70 years and older had the smallest decrease in LRI mortality rate, with a 23·8% (18·7–28·7) decline (figure 2). More detailed results on LRI incidence and mortality for additional age groups by sex, country, and year are available online via the GBD Results Tool on the GHDx.

### Aetiologies of LRIs

**Figure 3:**
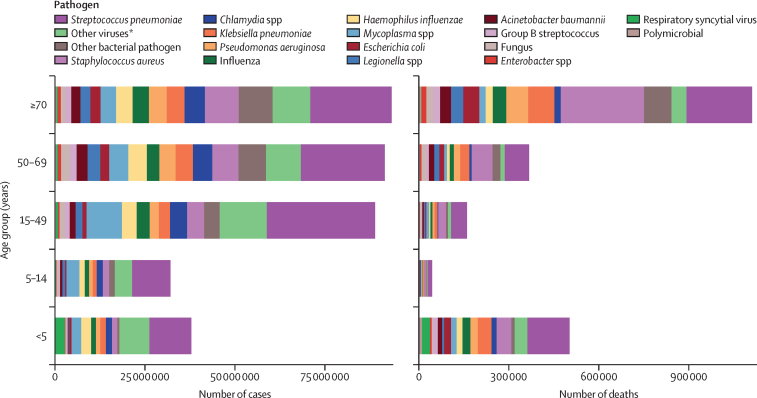
Aetiology distribution of global LRI cases and deaths by age group, 2021 LRI=lower respiratory infection. *“Other viruses” represents the aggregate of all viruses studied except influenza and respiratory syncytial virus.

**Figure 4:**
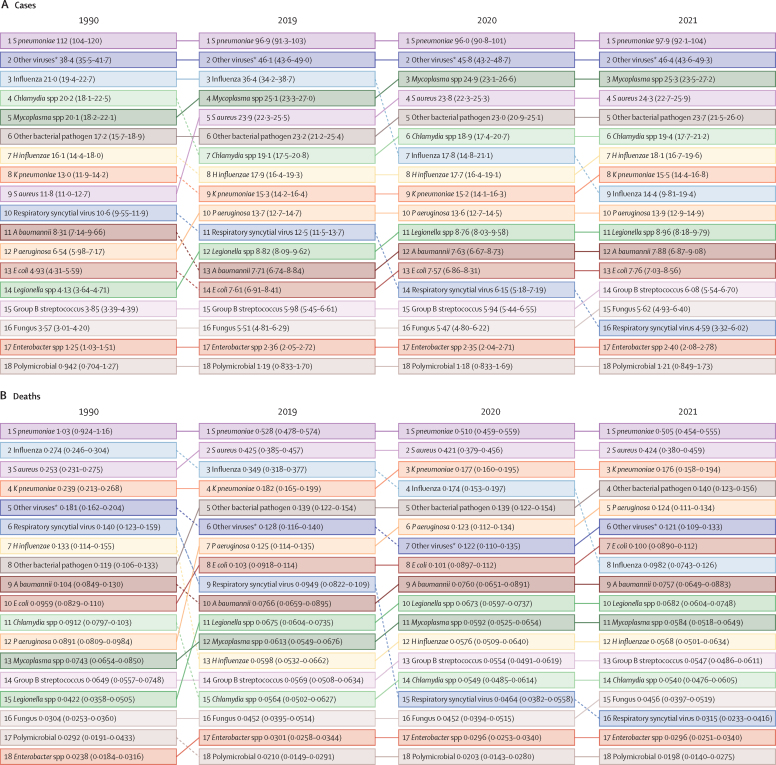
Ranked aetiologies by number of global cases and deaths across all ages, 1990, 2019, 2020, and 2021 Values are estimated millions of cases (A) or deaths (B) caused by each pathogen, with 95% uncertainty intervals in parentheses. Estimates are presented to three significant figures. *A baumannii*=*Acinetobacter baumannii*. *E coli*=*Escherichia coli*. *H influenzae*=*Haemophilus influenzae*. *K pneumoniae*=*Klebsiella pneumoniae*. *P aeruginosa*=*Pseudomonas aeruginosa*. *S aureus=Staphylococcus aureus*. *S pneumoniae=Streptococcus pneumoniae*. *“Other viruses” represents the aggregate of all viruses studied except influenza and respiratory syncytial virus.

In 2021, the pathogen responsible for the largest proportion of LRI incident episodes globally was *S pneumoniae*, which caused an estimated 97·9 million (95% UI 92·1–104·0) episodes (Figure 3, Figure 4; appendix 2 p 2104). This was followed by the categories of other viruses (ie, the aggregate of all viruses studied except influenza and RSV; 46·4 million [43·6–49·3] episodes) and *Mycoplasma* spp (25·3 million [23·5–27·2] episodes; Figure 3, Figure 4; appendix 2 p 2104). Key pathogens varied by age and geography. *S pneumoniae* was responsible for the largest number of episodes in 165 of the 204 modelled countries and territories in 2021, while the category of other viruses was responsible for the largest number of episodes in 39 countries (appendix 2 p 156). For all five studied age subdivisions, *S pneumoniae* caused the most episodes (figure 3; appendix 2 p 2104).

In 2019, before the COVID-19 pandemic and the decline in observed incidence of influenza and RSV, the first and second most common aetiologies were the same as for 2021, but the third most common aetiology was influenza, responsible for 36·4 million (95% UI 34·2–38·7) episodes globally (figure 4). *S pneumoniae* was responsible for the most LRI episodes in all five age groups in 2019, and was followed by the category of other viruses in all age groups except 70 years and older, in which influenza ranked second highest (appendix 2 p 2104). The third most common aetiology was other viruses in people aged 70 years and older, RSV in children younger than 5 years, *Mycoplasma* spp in children aged 5–14 years and people aged 15–49 years, and influenza in people aged 50–69 years (appendix 2 p 1971).

In 2021, the pathogen responsible for the largest proportion of all-age LRI deaths globally was also *S pneumoniae*, which led to an estimated 505 000 deaths (95% UI 454 000–555 000; figure 4; appendix 2 p 2107). This was followed by *S aureus* (424 000 deaths [380 000–459 000]) and *K pneumoniae* (176 000 deaths [158 000–194 000]; figure 4; appendix 2 p 2104). In 2019, before COVID-19 impacted the transmission of influenza and RSV, the first and second most common aetiologies leading to LRI death were the same as in 2021, but the third most common aetiology was influenza, which led to 349 000 deaths (318 000–377 000) globally (figure 4). Across age groups, in both 2019 and 2021, *S pneumoniae* was responsible for the most LRI deaths in people younger than 70 years, whereas *S aureus* caused the most deaths in people aged 70 years and older (figure 3; appendix 2 p 2104). In 2019, the second largest number of deaths came from *S aureus* in all age groups except children younger than 5 years, for whom the second-ranked aetiology was RSV, and people aged 70 years and older, for whom the second-ranked aetiology was *S pneumoniae*. The third most common aetiology leading to death was influenza for all five age groups (appendix 2 p 1971). In 2021, *S pneumoniae* was responsible for the highest number of deaths in 103 of 204 modelled countries and territories, whereas *S aureus* was responsible for the most deaths in the remaining 101 countries. These differences were largely attributable to differences in age structures across countries (appendix 2 p 156).

From 1990 to 2019, *H influenzae* had the largest reduction in global mortality (a 54·8% decrease [95% UI 48·8–60·6] to 59 800 deaths [53 200–66 200]), followed by *S pneumoniae* (48·5% decrease [42·8–53·9]; appendix 2 p 1971). Most of this improvement for both pathogens was in children younger than 5 years, with a 77·4% (72·9–81·2) decline in deaths due to *H influenzae* (from 102 000 [83 800–123 000] to 23 000 [18 600–27 700]) and a 76·1% (71·4–79·7) decline in deaths due to *S pneumoniae* (from 721 000 [621 000–843 000] to 172 000 [142 000–205 000]) during this period (appendix 2 p 1971).

### COVID-19 impact

Following the onset of the COVID-19 pandemic, we estimated that from 2019 to 2021, the number of influenza episodes decreased by 60·3% (95% UI 47·1–72·9) to 14·4 million (9·81–19·4) episodes, and deaths decreased by 71·8% (63·8–78·9) to 98 200 (74 300–126 000; appendix 2 p 1951). Across 18 modelled pathogen categories, influenza fell from being the third leading cause of both LRI episodes and deaths globally in 2019, to the ninth leading cause of episodes and the eighth leading cause of deaths in 2021 (figure 4). The high-income super-region saw the largest decrease in influenza episodes from 2019 to 2021 (91·5% [86·6–94·7]), and south Asia had the smallest decrease in influenza episodes in that same time frame (44·0% [14·6–71·8]; appendix 2 p 1951).

Similarly, since 2019, we estimated that global RSV episodes declined by 63·2% (53·1–72·7) to reach 4·59 million (3·32–6·02) in 2021, with a similar decrease in RSV deaths (66·7% [56·6–75·3]), to 31 500 (23 300–41 600) in 2021 (figure 4; appendix 2 p 1951).

Overall, for non-COVID-19 LRIs from 2019 to 2021, we estimated an 8·6% (6·6–10·4) decline in the overall incidence rate, from 4770 episodes (4510–5040) to 4350 episodes (4120–4610) per 100 000 population (from 369 million [349–391] to 344 million [325–364] total episodes), and a 16·0% (13·1–18·6) decline in mortality rate, from 32·9 deaths (29·9–35·4) to 27·7 deaths (25·1–29·9) per 100 000 (table 2).

## Discussion

This study provides comprehensive global, regional, and national estimates of LRI episodes and deaths attributable to 18 pathogen categories, by age group, from 1990 until 2021. These estimates are inclusive of the reduction in transmission of certain respiratory viruses observed during the COVID-19 pandemic and implementation of non-pharmaceutical interventions. We estimated 344 million (95% UI 325–364) incident episodes of LRIs and 2·18 million (1·98–2·36) deaths worldwide in 2021. *S pneumoniae* was responsible for the highest proportion of both incidence and mortality in all ages, followed by the category of other viral aetiologies and *Mycoplasma* spp for incidence, and *S aureus* and *K pneumoniae* for mortality. Between 2019 and 2021, during the COVID-19 pandemic, we estimated substantial declines in global influenza incidence and RSV incidence.

Although LRIs are ubiquitous across the world, the burden disproportionately falls on people living in poverty.^39^ In 2013, WHO and UNICEF formulated the Global Action Plan for the Prevention and Control of Pneumonia and Diarrhea (GAPPD), with the ambitious goal to end preventable childhood pneumonia and diarrhoea deaths by 2025.^40^ A specific target for 2025 is to reduce mortality from pneumonia in children younger than 5 years to fewer than 3 deaths per 1000 livebirths, roughly equivalent to a mortality rate of less than 60 deaths per 100 000 people per year among children younger than 5 years. As of 2021, we estimated a global LRI mortality rate of 76·2 deaths (61·7–92·9) per 100 000 children in this age group, and that 57 countries and territories, all but one of which were low-income and middle-income countries (LMICs), had a mortality rate over the global benchmark of 60 deaths per 100 000. To reduce mortality, the action plan calls for promotion of exclusive breastfeeding in infants younger than 6 months, reduction of indoor air pollution, expanded access to health care, and ongoing pneumonia case management in LMICs—approaches that have historically driven progress towards reducing LRI child mortality.^41^, ^42^

The WHO and UNICEF GAPPD also calls for increased coverage of pneumococcal conjugate vaccines (PCVs) and Hib vaccines. From 1990 to 2019, *H influenzae* showed the largest decline in global deaths, followed by *S pneumoniae*, both largely attributable to vaccination. Between 2000 and 2015, use of the Hib vaccine prevented an estimated 1·2 million deaths due to *H influenzae* infection globally, and PCV prevented an estimated 250 000 deaths due to pneumococcal infection.^8^ However, global coverage of these vaccines shows substantial room for improvement. According to WHO–UNICEF estimates of national immunisation coverage, global final-dose coverage of PCV among 1-year-olds was 60% and Hib coverage was 76% in 2022, both of which are above 2019 levels, suggesting recovery from pandemic immunisation disruptions.^42^, ^43^, ^44^ Although these global increases are promising, they can mask substantial inequities, and many vulnerable communities remain without access to vaccination.^44^, ^45^, ^46^ Strategies described by the WHO Immunization Agenda 2030 to increase coverage—including focusing on children who have not received any routine immunisations, building trust to avert vaccine hesitancy, and increasing vaccine access across the lifespan—can help reduce pneumonia mortality in areas with the highest burden.^46^, ^47^, ^48^, ^49^

The age groups of children younger than 5 years and adults aged 70 years and older had the highest LRI mortality rates in 1990. Time trends showed a steep decline in mortality in children younger than 5 years between 1990 and 2021, whereas no substantial decrease was observed in adults aged 70 years and older (figure 2). This trend holds true for the more granular age groups of 70–74 years and 75–79 years (appendix 2 p 4). Decline in immune function with ageing, called immunosenescence, promotes susceptibility to LRIs, as do age-related organ system changes and the development of comorbid conditions.^50^, ^51^ Influenza and pneumococcal vaccination remain effective tools to address LRIs in older adults.^7^ Pneumococcal immunisation of infant populations provides some herd protection for older adults.^52^, ^53^ In addition, pneumococcal vaccine administration to adults aged 65 years and older has been shown to be cost-effective^54^ with modest efficacy,^55^, ^56^, ^57^ at least in high-income settings. Because immunosenescence limits the efficacy of some vaccines in older adults, improved vaccine efficacy has emerged as a priority.^51^ Strategies towards more effective vaccines for older adults include higher doses of vaccines, repeated vaccinations, mucosal, subcutaneous, or intradermal administration, and use of more potent adjuvants.^58^ In LMICs, vaccine access for older adults is severely limited.^59^ More research is needed to assess the potential benefits of adult vaccination, understand barriers and challenges, and establish evidence-based guidelines in these settings.^60^, ^61^

RSV, the second-leading cause of LRI deaths in children younger than 5 years in 2019, has historically not been vaccine preventable. The development of affordable RSV vaccines and long-acting, affordable monoclonal antibodies (mAbs) was a priority for WHO's Vaccine Product and Delivery Research Unit and an active area of research.^62^, ^63^ These efforts came to fruition in 2023, when two vaccines for RSV were approved in the EU and the USA.^64^, ^65^, ^66^, ^67^ Both are for use in adults aged 60 years and older, and one is also approved for pregnant women to protect their infants. In addition, a new long-acting mAb injection, nirsevimab, was approved in 2022 in the EU and in 2023 in the USA to prevent RSV hospitalisation in both healthy and high-risk infants.^68^, ^69^ Generally, mAbs, including the long-approved, short-acting, RSV-preventive palivizumab, are too costly for use in most LMICs.^70^ The affordability of long-acting mAbs for LMICs is not yet known; preliminary cost-effectiveness analyses suggest a benefit, but this cost-effectiveness will depend on multiple factors, including pricing.^62^, ^70^, ^71^, ^72^ These long-acting mAbs and RSV vaccines, available for the first time, have the potential to avert unprecedented numbers of RSV cases and deaths in the 2023–24 respiratory infection season and beyond. A 2023 modelling study forecasts that with 60% vaccine coverage, in the USA alone, up to 2·0 million symptomatic RSV respiratory infections could be averted per year in adults older than 60 years, plus another 690 000 infections in the non-vaccinated population through indirect effects.^73^ However, these benefits will only reach locations where patients can access the vaccines. For the full global benefit of these preventives to be realised, equitable distribution is essential. It will be crucial for pharmaceutical companies, non-governmental organisations, and governments to work together to reduce barriers to access in LMICs.^74^

We have quantified the global burden of LRI attributable to *S aureus* in all ages and, for the first time in a comprehensive global study, we have identified the pathogen as the second-leading cause of LRI mortality after *S pneumoniae* in 2021. Although *S aureus* is a less frequent cause of LRI cases than *S pneumoniae*, it has a higher incidence of complications and a higher CFR. In a multisite US study, adult patients with *S aureus* LRI (n=37) had worse outcomes than those with *S pneumoniae* (n=115), including higher rates of intensive care unit (ICU) admission (62·2% *vs* 34·8%), mechanical ventilation (24·3% *vs* 12·2%), and inpatient mortality (10·8% *vs* 4·4%).^13^ Because of this poor prognosis, antistaphylococcal therapy is frequently included in empirical treatment for severe pneumonia.^13^, ^75^ Finding the causative pathogen in a patient with pneumonia can be challenging, and clinicians face the trade-off of balancing sufficiently broad empirical treatment with antibiotic stewardship.^75^, ^76^ In a global meta-analysis of *S aureus* pneumonia, 51% of isolates were meticillin-resistant *S aureus* (MRSA).^77^ The emergence of vancomycin-intermediate and vancomycin-resistant *S aureus* represents an escalating concern, and multidrug-resistant *S aureus* is classified as high-priority on the WHO global priority antimicrobial resistance pathogen list.^78^, ^79^ Antibiotic overuse, a key driver of resistance, remains an important concern across high-income countries and LMICs. Improved point-of-care diagnostics, including targeted PCR testing for MRSA, can prevent antibiotic overuse.^80^, ^81^, ^82^ In addition, although efforts to develop a vaccine against *S aureus* have so far been unsuccessful, ongoing research might generate a new method for prevention or treatment.^83^, ^84^

Our overall estimates of LRI mortality among children younger than 5 years are consistent with findings from a publication by WHO and the Maternal and Child Epidemiology Estimation Group, which estimated 740 000 (95% UI 620 000–840 000) child LRI deaths in 2019.^85^ We estimated 693 000 (580 000–822 000) child LRI deaths in 2019. Our estimates of pathogen distribution are also similar to other global reports. A 2016 study from the Global Initiative for MRSA Pneumonia (GLIMP),^77^ which included data across 54 countries, identified *S aureus* in 188 (6%) of 3193 adults with community-acquired pneumonia, in alignment with the current study. Likewise, a 2021 meta-analysis across eight countries estimated that 18% (95% CI 13–24) of community-acquired pneumonia cases in adults aged 50 years and older were attributable to *S pneumoniae*.^86^ Our estimates for the 50–69 years and 70 years and older global age groups are within the 95% CI of this meta-analysis.

In addition, we have estimated the COVID-19 pandemic-era reduction in influenza and RSV mortality and incidence by country, applied to a comprehensive set of global LRI estimates. From 2019 to 2021, we estimated a 71·8% (95% UI 63·8–78·9) decrease in influenza deaths and a 66·7% (56·6–75·3) decrease in RSV deaths worldwide (appendix 2 p 1951). These reductions were observed following the implementation of non-pharmaceutical interventions such as facemask use and mobility restriction, which have been implicated in the reduction of transmission of respiratory viruses, including influenza and RSV, in 2020 and 2021.^22^, ^87^, ^88^, ^89^, ^90^ However, other respiratory viruses, such as rhinovirus, adenovirus, and respiratory enteroviruses, quickly rebounded within a few months and persisted despite non-pharmaceutical interventions, showing fewer fluctuations in case counts with changing policies compared with influenza and RSV.^22^ Overall, hospitals across the world have reported reductions in admissions for community-acquired pneumonia during the COVID-19 pandemic.^91^, ^92^, ^93^

This study has several limitations. First, we quantified the COVID-19 pandemic-attributable reduction in LRI for influenza and RSV only. New evidence from a global surveillance network including 26 countries shows a decline in incidence of invasive infections attributable to respiratory pathogens, including *S pneumoniae* and *H influenzae*, during the COVID-19 pandemic.^94^ The decline in pneumococcal disease incidence might be primarily attributable to the decline in transmission of co-infecting respiratory viruses, including influenza and RSV, rather than reduced transmissibility or serotype selection of *S pneumoniae* itself.^95^ For the bacterial aetiologies that are predominantly health-care acquired, evidence is mostly limited to single-site studies and mixed, with some studies showing a reduction,^96^, ^97^ others showing an increase,^91^ and others showing no change.^98^ In the post-pandemic period, studies suggest that several of the pathogens that decreased in 2020 rebounded, including RSV, influenza, and pneumococcus.^99^, ^100^, ^101^, ^102^ Other pathogens—namely, *Mycoplasma* spp—showed a decline that persisted for a longer duration after the COVID-19 pandemic, with continued decreased detection observed until the end of 2022, followed by a delayed re-emergence in some countries in mid-2023.^103^, ^104^, ^105^ Due to interruption of established data exchanges caused by the COVID-19 pandemic, we were unable to estimate how bacterial pathogen distributions might have changed between 2019 and 2021. As data become available for more locations, pathogens, and years, we can comprehensively quantify the indirect effects of the pandemic on the incidence of LRI and its aetiologies in future rounds of GBD.

A second limitation is that, when estimating the effect of the COVID-19 pandemic on influenza and RSV incidence, we relied exclusively on case notification data from national and multinational surveillance networks. Our method cannot separate the effects of a true decrease in LRI incidence from the effects of a decrease in health-care-seeking behaviour; we also did not account for potential changes in reporting capability over time. Third, to calculate the reduction in RSV, we applied modelled estimates of COVID-19 pandemic-associated influenza reduction directly to RSV estimates. This decision was based on a meta-analysis of the ratio of the percentage change in influenza to the percentage change in RSV in 2020, relative to the pre-pandemic period, which showed no statistically significant difference in the reduction of the two pathogens. However, empirical studies published since the pandemic have shown that the resurgence patterns of RSV and influenza have differed.^106^, ^107^, ^108^, ^109^, ^110^ Fourth, limited data availability and quality are constraints, particularly in low-income countries, where the LRI burden is highest. Our assessment of LRI mortality in countries lacking vital registration data relies largely on verbal autopsy studies, which have modest sensitivity in accurately identifying deaths due to LRIs.^111^ Covariates and regional trends were leveraged to predict the burden of LRI and corresponding aetiologies for locations with few or no data. In selecting these covariates, some degree of model misspecification is possible due to potential omitted variables that are not captured in the dataset, which could affect the accuracy of our predictive model. Fifth, misclassification might be present in pathogen proportion data if certain pathogens are more difficult to detect than others, or if some pathogens, such as viruses in the population of older adults, are irregularly tested in a laboratory or clinical setting. Sixth, although we used a crosswalking process to adjust for systematic differences in incidence data source categories, this process might not fully account for all forms of bias. Finally, we directly applied LRI aetiology proportions from the Global Burden of AMR study to GBD estimates of LRI cases and deaths, although the two studies use slightly different definitions of LRI. In particular, the Global Burden of AMR study's definition of LRI deaths covers any event for which LRI was present in the causal chain, regardless of the underlying cause of death, whereas the GBD definition only includes instances in which LRI was the underlying cause of death.

In summary, we have shown that, despite declines in incidence during the COVID-19 pandemic, LRIs remain a significant cause of morbidity and mortality worldwide. Increased access to existing vaccines, as well as rollout of novel vaccines and therapies, could reduce the burden of LRIs. Supporting research for low-cost interventions against *S aureus* could accelerate progress in reducing LRI-related mortality and incidence, especially in resource-constrained settings. In addition, the growing threat of antimicrobial resistance highlights the importance of antibiotic stewardship and investment in improved diagnostic technologies to improve the specificity and accuracy of therapy. Finally, all these interventions must come at an affordable cost, so that they can reduce inequities seen in LRI mortality, rather than exacerbate them.

## Data sharing

To download the data used in these analyses, please visit the GHDx GBD 2021 website.

## Declaration of interests

J A Berkley reports support for the present manuscript from research grants from the Bill & Melinda Gates Foundation, Wellcome Trust, the National Institute for Health and Care Research (NIHR), and the Medical Research Council (MRC). C Brown reports other financial support from an ad-hoc one-off market research advisory role (anonymously conducted via market research companies with no direct communication, none specifically related to lower respiratory tract infections), all outside the submitted work. K Krishan reports other non-financial support from the UGC Centre of Advanced Study, CAS II, awarded to the Department of Anthropology, Panjab University (Chandigarh, India) outside the submitted work. M-C Li reports support for the present manuscript from the National Science and Technology Council in Taiwan (112-2410-H-003-031) and other financial or non-financial support as a Technical Editor of the *Journal of the American Heart Association*, Review Editor of *Frontiers in Public Health*, and Editorial Board Member of *BMC Public Health,* outside the submitted work. S A Meo reports grants or contracts from King Saud University (Riyadh, Saudi Arabia; RSP-2024 R47), outside the submitted work. L Monasta reports support for the present manuscript from the Italian Ministry of Health (Ricerca Corrente 34/2017), with payments made to the Institute for Maternal and Child Health IRCCS Burlo Garofolo. C Moore reports participation with Dr Gwen Knight as a member of the advisory board for MRC grants (no payments made), with the WHO Advisory group, and with the REVIVE Advisory group as a member of the steering group; and leadership or fiduciary roles in board, society, committee, or advocacy groups (unpaid) as the co-chair of the Impact and Influence Group of the Microbiology Society, outside the submitted work. A Pollard reports grants or contracts from the Bill & Melinda Gates Foundation, Wellcome Trust, Cepi, MRC, NIHR, AstraZeneca, European Commission, and the Serum Institute of India; royalties or licenses from AstraZeneca; consulting fees from Shionogi; leadership or fiduciary roles in board, society, committee, or advocacy groups (unpaid) as the chair of the Department of Health and Social Care's Joint Committee on Vaccination and Immunisation and as a member of the WHO Strategic Advisory Group of Experts on Immunization until 2022; and receipt of equipment, materials, drugs, medical writing, gifts, or other services from Moderna, outside the submitted work. L F Reyes reports grants or contracts from MSD and Pfizer; consulting fees from GlaxoSmithKline, MSD, and Pfizer; payment or honoraria for lectures, presentations, speakers bureaus, manuscript writing, or educational events from GlaxoSmithKline, MSD, and Pfizer; payment for expert testimony from GlaxoSmithKline, MSD, and Pfizer; and support for attending meetings or travel from GlaxoSmithKline and Pfizer, outside the submitted work. Y L Samodra reports grants or contracts from Taipei Medical University, and leadership or fiduciary roles in board, society, committee, or advocacy groups (paid or unpaid) as the co-founder of Benang Merah Research Center, outside the submitted work. E A F Simôes reports support for the present manuscript from the Bill & Melinda Gates Foundation; grants or contracts from AstraZeneca, Merck & Co, Pfizer, and Icosavax; consulting fees from Merck & Co, Pfizer, GlaxoSmithKline, Sanofi Pasteur, Cidara Therapeutics, Adagio Therapeutics, Nuance Pharmaceuticals, Enanta, and Icosavax; payment or honoraria for lectures, presentations, speakers bureaus, manuscript writing, or educational events from Pfizer and AstraZeneca; support for attending meetings or travel from Pfizer and AstraZeneca; and participation on a data safety monitoring board or advisory board with AbbVie, GlaxoSmithKline, the Bill & Melinda Gates Foundation, and Moderna, outside the submitted work. M Zielińska reports other financial support as an AstraZeneca employee, outside the submitted work.
