# Supplementary material for: Global, regional, and national incidence and mortality burden of non-COVID-19 lower respiratory infections and aetiologies, 1990–2021: a systematic analysis from the Global Burden of Disease Study 2021
Source: Lancet Infect Dis. 2024 Sep;24(9):974–1002. doi: 10.1016/S1473-3099(24)00176-2 (PMC11339187; doi:10.1016/S1473-3099(24)00176-2)
Supplement: Supplementary appendix 2 [file mmc2.pdf]

THE LANCET
Infectious Diseases

Supplementary appendix 2

This appendix formed part of the original submission and has been peer reviewed. We post it as supplied by the authors.

Supplement to: GBD 2021 Lower Respiratory Infections and Antimicrobial Resistance Collaborators. Global, regional, and national incidence and mortality burden of non-COVID-19 lower respiratory infections and aetiologies, 1990–2021: a systematic analysis from the Global Burden of Disease Study 2021. *Lancet Infect Dis* 2024; published online April 15. https://doi.org/10.1016/S1473-3099(24)00176-2.

Supplementary Results Appendix to "Global, regional, and national incidence and mortality burden of non-COVID lower respiratory infections and aetiologies, 1990–2021: a systematic analysis from the Global Burden of Disease Study 2021"

Table of Contents

Appendix Figure 1. Global age pattern of LRI mortality rates per 100 000 (top) and counts (bottom) by SDI quintile, 2021……………………………………………………………………………………………03

Appendix Figure 2 Global LRI mortality rates per 100 000 for 70+, 70-74, and 75-59 age groups, from 1990-2021……………………………………………………………………………………………………04

Appendix Table S1: All-age, and sub age groups LRI incidence counts, rates, and percent change in 1990, 2019, 2020, & 2021 by country and socio-demographic index (SDI)............................05

Appendix Table S2: All-age, and sub age groups LRI mortality counts, rates, and percent change in 1990, 2019, 2020, & 2021 by country and socio-demographic index (SDI)…………………….......86

Appendix Table S3: All-age, and sub age groups LRI etiology counts for deaths and cases for global, seven GBD super-regions, 21 GBD regions, and countries, for 1990 and 2021, and % change……156

Appendix Table S4: All-age, and sub age groups LRI etiology proportions for deaths and cases for global, seven GBD super-regions, 21 GBD regions, and countries, for 1990 and 2021, and % change………1187

Appendix Table S5: All-age Influenza and RSV counts for global, seven GBD super-regions, 21 GBD regions, and countries for 2019, 2020, & 2021 with percent change............................1951

Appendix Table S6: All-age, and sub age groups LRI etiology counts for deaths and cases for 2019 by global, seven GBD super-regions, and 21 GBD regions, with percent change from 1990 to 2019...1971

Appendix Table S7. Aetiology-specific counts for lower respiratory infection deaths and cases in 2021, with percentage change from 1990 – to 2021, for Socio-demographic Index (SDI) quintile, global, seven GBD super-regions, and 21 GBD regions...2104

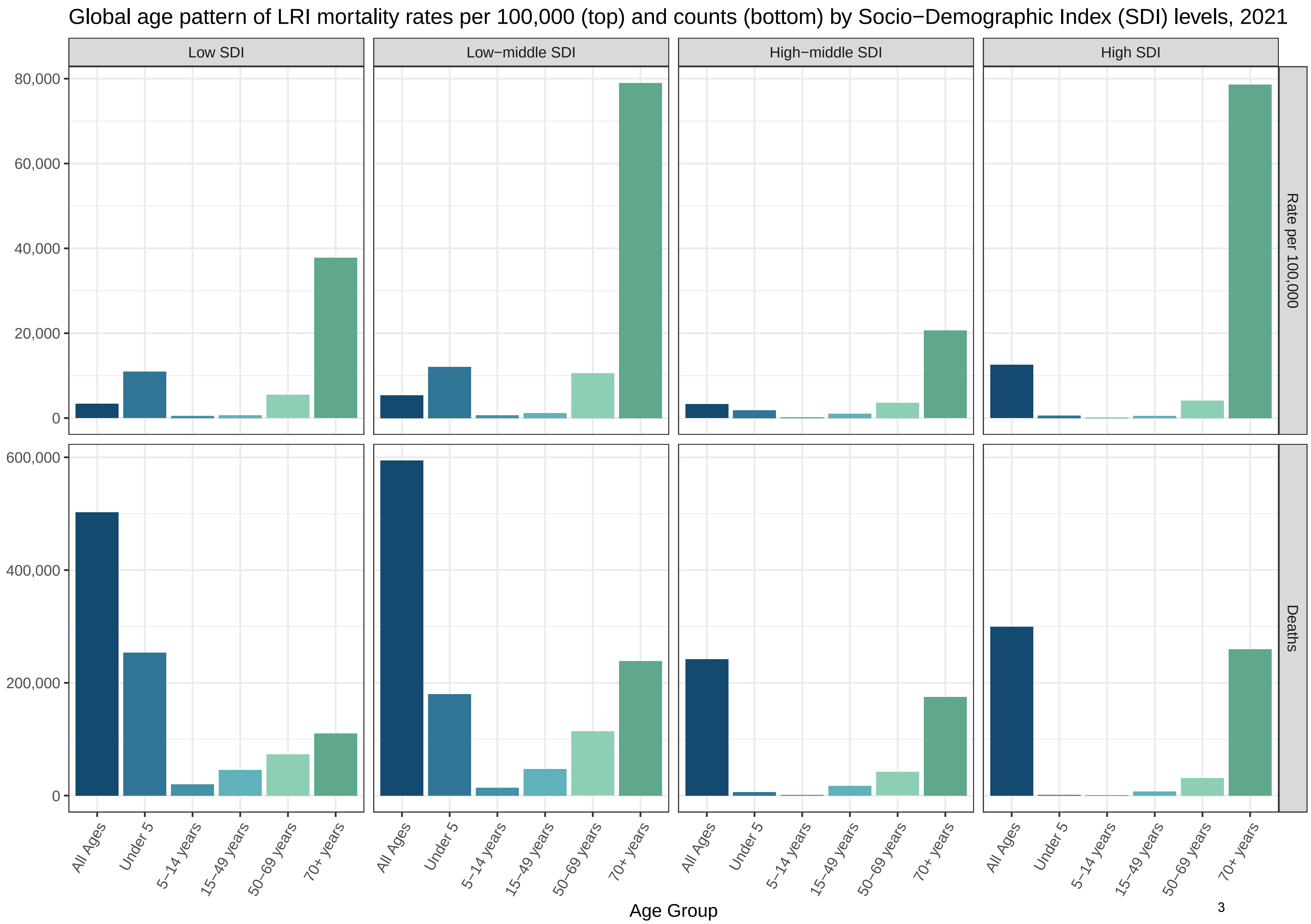

Global age pattern of LRI mortality rates per 100,000 (top) and counts (bottom) by Socio−Demographic Index (SDI) levels, 2021
Low SDI
Low−middle SDI
High−middle SDI
High SDI
Rate per 100,000
Deaths
80,000
60,000
40,000
20,000
0
600,000
400,000
200,000
0
All Ages
Under 5
5−14 years
15−49 years
50−69 years
70+ years
Age Group

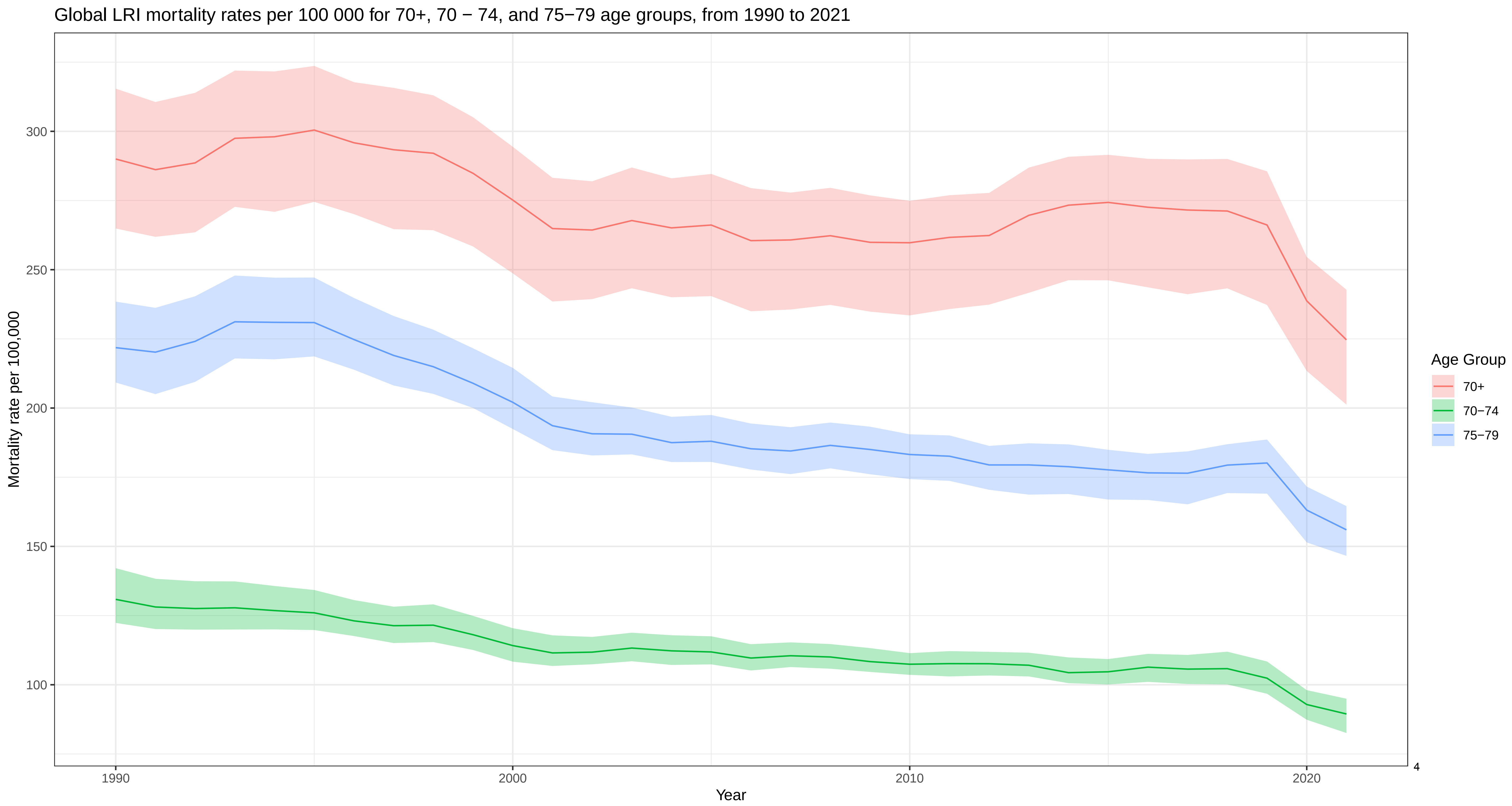

Global LRI mortality rates per 100 000 for 70+, 70 − 74, and 75−79 age groups, from 1990 to 2021
Mortality rate per 100,000
300
250
200
150
100
1990
2000
2010
2020
Year
Age Group
70+
70−74
75−79

Appendix Table S1: All-age, and sub age groups LRI incidence counts, rates, and percent change in 1990, 2019, 2020, & 2021 by country and socio-demographic index (SDI)

Location	Age Group	1990		2019		2020		2021		Percent Incidence Rate Change from 1990-2019 (95% UI)	Percent Incidence Rate Change from 2019-2021 (95% UI)
		Episodes (95% UI)	Rate (95% UI)	Episodes (95% UI)	Rate (95% UI)	Episodes (95% UI)	Rate (95% UI)	Episodes (95% UI)	Rate (95% UI)		
**High SDI**	All Ages	15,700,000 (14,900,000 to 16,600,000)	1,783.6 (1,689.8 to 1,890.0)	17,900,000 (17,000,000 to 18,900,000)	1,647.3 (1,562.4 to 1,738.8)	16,600,000 (15,700,000 to 17,500,000)	1,519.6 (1,439.4 to 1,608.0)	14,800,000 (14,100,000 to 15,700,000)	1,354.6 (1,285.3 to 1,433.2)	-7.6 (-10.1 to -5.3)	-7.8 (-10.0 to -5.9)
	Under 5	1,920,000 (1,660,000 to 2,190,000)	3,104.5 (2,686.7 to 3,556.3)	908,000 (775,000 to 1,060,000)	1,623.3 (1,386.1 to 1,900.3)	750,000 (638,000 to 874,000)	1,365.2 (1,160.7 to 1,590.2)	600,000 (513,000 to 702,000)	1,114.0 (952.4 to 1,302.9)	-47.7 (-50.6 to -45.1)	-15.9 (-19.4 to -12.7)
	5-14 years	1,040,000 (820,000 to 1,340,000)	841.3 (660.5 to 1,080.0)	604,000 (476,000 to 761,000)	511.0 (402.9 to 643.8)	565,000 (447,000 to 720,000)	476.7 (376.7 to 607.5)	533,000 (420,000 to 675,000)	449.5 (354.2 to 569.0)	-39.3 (-41.5 to -36.6)	-6.7 (-8.5 to -4.8)
	15-49 years	2,490,000 (2,220,000 to 2,790,000)	539.5 (481.7 to 604.4)	2,120,000 (1,900,000 to 2,340,000)	418.8 (376.0 to 462.7)	1,930,000 (1,740,000 to 2,130,000)	382.9 (344.4 to 422.8)	1,820,000 (1,630,000 to 2,010,000)	363.3 (324.8 to 400.7)	-22.4 (-24.3 to -20.5)	-8.6 (-10.5 to -6.7)
	50-69 years	3,860,000 (3,560,000 to 4,170,000)	2,354.7 (2,173.2 to 2,542.5)	4,520,000 (4,180,000 to 4,880,000)	1,663.0 (1,537.2 to 1,796.6)	4,190,000 (3,860,000 to 4,530,000)	1,532.8 (1,412.3 to 1,654.1)	3,860,000 (3,560,000 to 4,160,000)	1,399.4 (1,290.0 to 1,509.6)	-29.4 (-30.9 to -27.5)	-7.8 (-10.0 to -6.0)
	70+ years	6,380,000 (5,880,000 to 6,960,000)	9,244.5 (8,509.4 to 10,077.5)	9,760,000 (9,050,000 to 10,600,000)	7,192.4 (6,664.9 to 7,805.8)	9,140,000 (8,480,000 to 9,840,000)	6,534.2 (6,059.1 to 7,036.3)	8,000,000 (7,420,000 to 8,680,000)	5,578.0 (5,172.7 to 6,051.3)	-22.2 (-24.4 to -20.1)	-9.2 (-11.6 to -7.1)
**High-middle SDI**	All Ages	37,200,000 (34,900,000 to 39,700,000)	3,498.3 (3,279.9 to 3,730.9)	45,900,000 (43,000,000 to 48,900,000)	3,535.0 (3,316.4 to 3,772.9)	42,400,000 (39,900,000 to 45,200,000)	3,261.4 (3,065.9 to 3,474.3)	40,900,000 (38,500,000 to 43,500,000)	3,138.7 (2,951.6 to 3,339.3)	1.1 (-4.2 to 6.3)	-7.7 (-10.2 to -5.2)
	Under 5	11,300,000 (9,950,000 to 12,900,000)	12,155.7 (10,709.8 to 13,896.9)	3,220,000 (2,710,000 to 3,790,000)	4,201.3 (3,537.7 to 4,940.8)	2,720,000 (2,290,000 to 3,200,000)	3,682.0 (3,096.2 to 4,325.9)	2,240,000 (1,880,000 to 2,620,000)	3,202.9 (2,686.7 to 3,735.4)	-65.4 (-68.5 to -62.2)	-12.4 (-16.4 to -7.8)
	5-14 years	4,980,000 (4,020,000 to 6,130,000)	2,756.3 (2,224.9 to 3,392.1)	3,730,000 (2,940,000 to 4,640,000)	2,412.3 (1,902.9 to 3,001.3)	3,640,000 (2,870,000 to 4,540,000)	2,304.1 (1,819.7 to 2,877.3)	3,640,000 (2,870,000 to 4,540,000)	2,260.8 (1,782.5 to 2,824.0)	-12.5 (-19.4 to -4.8)	-4.5 (-7.5 to -1.0)
	15-49 years	6,710,000 (6,030,000 to 7,440,000)	1,188.4 (1,068.1 to 1,318.7)	8,620,000 (7,700,000 to 9,620,000)	1,345.6 (1,202.4 to 1,502.0)	7,950,000 (7,120,000 to 8,830,000)	1,252.6 (1,121.2 to 1,390.1)	7,620,000 (6,840,000 to 8,460,000)	1,211.1 (1,085.7 to 1,343.0)	13.2 (9.7 to 17.3)	-6.9 (-9.2 to -4.3)
	50-69 years	7,460,000 (6,830,000 to 8,080,000)	4,284.7 (3,925.4 to 4,640.2)	12,100,000 (11,000,000 to 13,200,000)	3,825.6 (3,485.4 to 4,174.3)	11,500,000 (10,500,000 to 12,500,000)	3,581.3 (3,281.3 to 3,888.7)	11,100,000 (10,100,000 to 12,100,000)	3,417.1 (3,094.2 to 3,705.9)	-10.7 (-13.8 to -6.9)	-6.4 (-9.1 to -3.7)
	70+ years	6,770,000 (6,140,000 to 7,390,000)	13,149.2 (11,923.5 to 14,362.2)	18,200,000 (16,300,000 to 20,300,000)	16,585.7 (14,816.6 to 18,516.2)	16,600,000 (14,900,000 to 18,300,000)	14,660.6 (13,102.3 to 16,151.7)	16,300,000 (14,600,000 to 18,100,000)	13,866.9 (12,398.2 to 15,377.7)	26.1 (19.2 to 34.3)	-11.6 (-14.8 to -7.8)
**Middle SDI**	All Ages	87,100,000 (81,400,000 to 92,900,000)	5,054.4 (4,726.3 to 5,390.2)	96,700,000 (91,100,000 to 102,000,000)	4,012.3 (3,778.5 to 4,251.6)	89,000,000 (84,200,000 to 94,300,000)	3,662.1 (3,461.7 to 3,879.0)	89,500,000 (84,700,000 to 95,300,000)	3,657.2 (3,457.2 to 3,892.0)	-20.6 (-24.1 to -16.7)	-8.7 (-10.2 to -7.3)
	Under 5	29,700,000 (26,300,000 to 33,500,000)	14,797.3 (13,136.4 to 16,713.9)	8,680,000 (7,630,000 to 9,890,000)	4,632.6 (4,071.5 to 5,274.5)	7,590,000 (6,680,000 to 8,680,000)	4,148.5 (3,650.8 to 4,740.1)	6,850,000 (5,990,000 to 7,890,000)	3,879.7 (3,392.6 to 4,469.1)	-68.7 (-69.7 to -67.6)	-10.4 (-12.7 to -8.2)
	5-14 years	12,800,000 (10,500,000 to 15,500,000)	3,402.6 (2,778.2 to 4,121.3)	7,370,000 (5,990,000 to 8,910,000)	1,930.1 (1,568.4 to 2,332.2)	6,970,000 (5,660,000 to 8,430,000)	1,803.1 (1,465.3 to 2,183.1)	6,830,000 (5,560,000 to 8,280,000)	1,750.7 (1,426.0 to 2,122.5)	-43.3 (-46.4 to -39.5)	-6.6 (-8.2 to -5.0)

Location	Age Group	1990		2019		2020		2021		Percent Incidence Rate Change from 1990-2019 (95% UI)	Percent Incidence Rate Change from 2019-2021 (95% UI)
		Episodes (95% UI)	Rate (95% UI)	Episodes (95% UI)	Rate (95% UI)	Episodes (95% UI)	Rate (95% UI)	Episodes (95% UI)	Rate (95% UI)		
	15-49 years	18,900,000 (17,200,000 to 20,800,000)	2,074.9 (1,885.7 to 2,280.5)	24,600,000 (22,300,000 to 27,000,000)	1,966.6 (1,781.8 to 2,159.1)	22,500,000 (20,500,000 to 24,700,000)	1,799.4 (1,634.5 to 1,973.7)	22,600,000 (20,500,000 to 24,900,000)	1,803.5 (1,633.0 to 1,986.2)	-5.2 (-8.0 to -2.8)	-8.5 (-10.0 to -7.0)
	50-69 years	14,400,000 (13,000,000 to 15,800,000)	7,609.8 (6,853.4 to 8,360.1)	27,800,000 (25,200,000 to 30,500,000)	6,042.7 (5,495.9 to 6,633.2)	25,900,000 (23,600,000 to 28,300,000)	5,468.6 (4,987.2 to 5,975.0)	26,600,000 (24,200,000 to 29,000,000)	5,470.5 (4,990.1 to 5,976.3)	-20.6 (-23.4 to -17.6)	-9.5 (-11.2 to -8.0)
	70+ years	11,300,000 (10,100,000 to 12,500,000)	24,697.8 (22,037.6 to 27,426.9)	28,300,000 (25,700,000 to 31,600,000)	21,637.0 (19,643.2 to 24,127.6)	26,000,000 (23,700,000 to 28,800,000)	19,223.2 (17,467.8 to 21,269.0)	26,700,000 (24,100,000 to 29,600,000)	18,911.1 (17,100.3 to 21,016.6)	-12.4 (-16.4 to -7.2)	-11.2 (-13.1 to -9.2)
**Low-middle SDI**	All Ages	119,000,000 (111,000,000 to 127,000,000)	10,254.0 (9,596.2 to 10,941.0)	137,000,000 (129,000,000 to 145,000,000)	7,301.1 (6,889.2 to 7,748.4)	126,000,000 (119,000,000 to 133,000,000)	6,638.3 (6,282.2 to 7,006.3)	130,000,000 (122,000,000 to 138,000,000)	6,742.4 (6,355.3 to 7,173.5)	-28.8 (-31.5 to -26.0)	-9.1 (-10.9 to -7.2)
	Under 5	37,500,000 (33,300,000 to 42,000,000)	21,604.0 (19,187.8 to 24,200.3)	16,700,000 (14,900,000 to 18,700,000)	8,530.6 (7,610.6 to 9,580.6)	14,500,000 (13,000,000 to 16,600,000)	7,512.9 (6,704.2 to 8,555.4)	14,100,000 (12,400,000 to 16,000,000)	7,343.5 (6,464.9 to 8,356.5)	-60.5 (-61.5 to -59.4)	-11.9 (-14.7 to -9.0)
	5-14 years	17,000,000 (13,900,000 to 20,800,000)	5,694.7 (4,647.4 to 6,948.8)	13,000,000 (10,700,000 to 15,400,000)	3,353.2 (2,768.0 to 3,977.9)	12,100,000 (10,100,000 to 14,500,000)	3,136.1 (2,604.9 to 3,745.9)	12,000,000 (9,930,000 to 14,400,000)	3,099.9 (2,556.6 to 3,718.7)	-41.1 (-44.2 to -38.0)	-6.5 (-9.2 to -3.8)
	15-49 years	26,800,000 (24,200,000 to 29,600,000)	4,865.4 (4,397.2 to 5,362.7)	38,600,000 (35,200,000 to 42,100,000)	3,908.7 (3,562.9 to 4,260.6)	35,800,000 (32,500,000 to 39,100,000)	3,573.6 (3,242.2 to 3,907.2)	36,700,000 (33,300,000 to 40,100,000)	3,613.3 (3,279.4 to 3,947.7)	-19.7 (-21.7 to -17.1)	-8.6 (-10.6 to -6.2)
	50-69 years	22,000,000 (19,800,000 to 24,400,000)	19,664.4 (17,647.7 to 21,804.9)	36,300,000 (33,000,000 to 39,700,000)	14,999.1 (13,644.8 to 16,423.3)	33,600,000 (30,500,000 to 36,500,000)	13,488.6 (12,241.0 to 14,682.2)	35,000,000 (31,800,000 to 38,200,000)	13,744.2 (12,491.8 to 14,995.5)	-23.7 (-27.4 to -19.9)	-10.1 (-12.7 to -7.5)
	70+ years	15,800,000 (13,900,000 to 17,800,000)	60,146.0 (52,905.4 to 67,732.4)	32,600,000 (29,400,000 to 36,900,000)	48,877.1 (44,089.3 to 55,283.6)	30,100,000 (27,000,000 to 33,700,000)	43,991.0 (39,501.2 to 49,353.2)	31,700,000 (28,200,000 to 35,900,000)	45,178.2 (40,213.6 to 51,217.1)	-18.7 (-24.0 to -13.3)	-10.0 (-12.7 to -7.0)
**Low SDI**	All Ages	54,600,000 (50,900,000 to 58,200,000)	10,899.1 (10,149.8 to 11,601.2)	71,500,000 (67,300,000 to 75,400,000)	6,698.9 (6,308.8 to 7,070.2)	67,500,000 (63,600,000 to 71,100,000)	6,176.0 (5,823.9 to 6,510.0)	68,600,000 (65,000,000 to 72,600,000)	6,143.1 (5,812.8 to 6,500.9)	-38.5 (-40.4 to -36.5)	-7.8 (-9.6 to -6.2)
	Under 5	20,600,000 (18,200,000 to 23,400,000)	22,738.9 (20,015.8 to 25,809.9)	15,500,000 (13,900,000 to 17,400,000)	9,564.9 (8,563.1 to 10,736.1)	14,200,000 (12,600,000 to 15,900,000)	8,642.5 (7,675.9 to 9,724.6)	14,000,000 (12,500,000 to 15,900,000)	8,480.2 (7,543.0 to 9,585.9)	-57.9 (-59.1 to -56.7)	-9.6 (-11.9 to -7.2)
	5-14 years	7,700,000 (6,290,000 to 9,290,000)	5,576.6 (4,554.1 to 6,725.8)	9,320,000 (7,750,000 to 11,100,000)	3,263.0 (2,713.8 to 3,892.8)	9,130,000 (7,650,000 to 10,900,000)	3,146.1 (2,636.6 to 3,757.0)	9,020,000 (7,500,000 to 10,900,000)	3,061.6 (2,543.7 to 3,691.2)	-41.5 (-44.4 to -38.1)	-3.6 (-5.9 to -1.2)
	15-49 years	11,800,000 (10,600,000 to 12,900,000)	5,317.4 (4,806.8 to 5,850.9)	20,200,000 (18,300,000 to 22,000,000)	3,951.9 (3,580.5 to 4,303.3)	19,500,000 (17,700,000 to 21,100,000)	3,693.8 (3,367.2 to 4,009.8)	20,000,000 (18,300,000 to 21,900,000)	3,686.4 (3,364.9 to 4,029.7)	-25.7 (-28.1 to -22.8)	-6.5 (-8.5 to -4.5)
	50-69 years	9,010,000 (8,030,000 to 9,980,000)	21,438.0 (19,102.1 to 23,759.2)	15,200,000 (13,800,000 to 16,700,000)	17,480.9 (15,878.6 to 19,177.8)	14,300,000 (13,100,000 to 15,600,000)	15,844.2 (14,473.5 to 17,304.6)	14,800,000 (13,500,000 to 16,200,000)	15,955.7 (14,491.9 to 17,459.3)	-18.5 (-22.4 to -14.2)	-9.4 (-11.7 to -7.0)

Location	Age Group	1990		2019		2020		2021		Percent Incidence Rate Change from 1990-2019 (95% UI)	Percent Incidence Rate Change from 2019-2021 (95% UI)
		Episodes (95% UI)	Rate (95% UI)	Episodes (95% UI)	Rate (95% UI)	Episodes (95% UI)	Rate (95% UI)	Episodes (95% UI)	Rate (95% UI)		
	70+ years	5,530,000<br>(4,900,000 to 6,210,000)	59,241.9<br>(52,529.8 to 66,532.7)	11,200,000<br>(10,000,000 to 12,700,000)	53,575.8<br>(47,915.3 to 60,574.5)	10,400,000<br>(9,300,000 to 11,700,000)	48,762.8<br>(43,489.0 to 54,900.8)	10,800,000<br>(9,600,000 to 12,200,000)	49,111.1<br>(43,765.6 to 55,612.9)	-9.6<br>(-15.4 to -2.0)	-9.0<br>(-11.9 to -5.7)
**Armenia**	All Ages	86,700<br>(80,800 to 93,500)	2,535.6<br>(2,362.6 to 2,734.0)	64,600<br>(60,600 to 69,400)	2,136.0<br>(2,002.2 to 2,295.9)	63,100<br>(57,500 to 67,800)	2,096.1<br>(1,908.6 to 2,252.3)	59,300<br>(53,800 to 65,800)	1,981.1<br>(1,795.0 to 2,195.3)	-15.8<br>(-22.4 to -9.2)	-1.9<br>(-9.9 to 4.1)
	Under 5	33,900<br>(30,200 to 37,800)	8,847.8<br>(7,889.3 to 9,872.5)	6,180<br>(5,410 to 7,070)	3,101.9<br>(2,715.4 to 3,545.6)	5,790<br>(4,740 to 6,680)	2,999.3<br>(2,456.6 to 3,464.9)	4,910<br>(3,950 to 6,090)	2,637.4<br>(2,118.9 to 3,272.3)	-64.9<br>(-68.2 to -61.6)	-3.3<br>(-18.3 to 7.0)
	5-14 years	14,900<br>(12,100 to 17,800)	2,251.0<br>(1,826.6 to 2,702.0)	4,380<br>(3,490 to 5,340)	1,091.4<br>(868.2 to 1,328.8)	4,320<br>(3,510 to 5,330)	1,068.1<br>(866.6 to 1,315.8)	4,100<br>(3,260 to 5,020)	1,010.6<br>(801.6 to 1,234.8)	-51.5<br>(-56.9 to -45.4)	-2.1<br>(-12.4 to 8.5)
	15-49 years	14,800<br>(13,000 to 16,800)	866.1<br>(760.0 to 984.6)	11,300<br>(10,100 to 12,600)	766.7<br>(680.4 to 850.4)	11,400<br>(10,200 to 12,800)	780.3<br>(696.3 to 869.7)	10,600<br>(9,370 to 12,000)	726.9<br>(643.5 to 824.9)	-11.5<br>(-19.0 to -3.2)	1.8<br>(-7.4 to 10.7)
	50-69 years	13,000<br>(11,600 to 14,700)	2,367.8<br>(2,115.0 to 2,678.0)	18,900<br>(17,000 to 20,900)	2,659.9<br>(2,386.9 to 2,948.8)	18,800<br>(16,800 to 20,900)	2,654.6<br>(2,375.6 to 2,954.4)	18,000<br>(16,000 to 20,200)	2,559.4<br>(2,284.2 to 2,875.7)	12.3<br>(1.6 to 25.4)	-0.2<br>(-9.2 to 8.4)
	70+ years	10,200<br>(8,940 to 11,600)	8,508.4<br>(7,480.5 to 9,674.1)	23,800<br>(21,600 to 26,600)	10,092.1<br>(9,133.6 to 11,265.2)	22,800<br>(20,000 to 25,600)	9,483.2<br>(8,317.8 to 10,637.9)	21,800<br>(19,000 to 25,000)	8,901.9<br>(7,790.3 to 10,227.9)	18.6<br>(7.4 to 33.2)	-6.0<br>(-14.8 to 2.7)
**Azerbaijan**	All Ages	290,000<br>(271,000 to 313,000)	3,957.4<br>(3,702.0 to 4,271.3)	222,000<br>(208,000 to 236,000)	2,142.9<br>(2,009.3 to 2,276.9)	221,000<br>(205,000 to 234,000)	2,117.5<br>(1,963.8 to 2,246.4)	190,000<br>(178,000 to 203,000)	1,813.4<br>(1,696.8 to 1,933.8)	-45.9<br>(-49.6 to -42.2)	-1.2<br>(-7.5 to 4.2)
	Under 5	161,000<br>(147,000 to 176,000)	17,811.5<br>(16,278.7 to 19,509.5)	43,200<br>(39,100 to 48,500)	5,599.0<br>(5,058.7 to 6,281.8)	41,600<br>(36,700 to 46,600)	5,575.8<br>(4,919.9 to 6,246.1)	29,800<br>(26,500 to 33,400)	4,130.7<br>(3,681.4 to 4,634.5)	-68.6<br>(-70.8 to -65.9)	-0.4<br>(-10.4 to 7.9)
	5-14 years	43,600<br>(35,600 to 53,600)	2,860.7<br>(2,339.9 to 3,517.5)	29,200<br>(24,200 to 34,000)	1,825.6<br>(1,516.2 to 2,129.9)	28,800<br>(23,800 to 33,900)	1,777.1<br>(1,465.0 to 2,086.0)	25,900<br>(21,500 to 30,900)	1,578.7<br>(1,309.2 to 1,883.9)	-36.2<br>(-44.4 to -26.7)	-2.7<br>(-13.6 to 10.7)
	15-49 years	40,000<br>(36,100 to 43,900)	1,090.8<br>(982.8 to 1,195.3)	48,400<br>(43,800 to 53,500)	872.8<br>(788.3 to 964.5)	49,900<br>(44,300 to 55,700)	897.4<br>(797.3 to 1,001.9)	43,000<br>(38,700 to 47,800)	771.4<br>(695.3 to 857.6)	-20.0<br>(-25.9 to -13.2)	2.8<br>(-5.9 to 12.0)
	50-69 years	26,300<br>(23,400 to 29,600)	2,617.0<br>(2,328.5 to 2,952.1)	57,100<br>(50,800 to 63,400)	2,758.7<br>(2,453.9 to 3,059.2)	58,900<br>(52,200 to 65,400)	2,759.0<br>(2,444.2 to 3,064.8)	54,400<br>(48,200 to 61,400)	2,490.5<br>(2,206.6 to 2,809.4)	5.4<br>(-4.7 to 16.4)	0.0<br>(-10.3 to 10.3)
	70+ years	19,100<br>(16,600 to 21,700)	8,489.5<br>(7,384.2 to 9,666.4)	43,900<br>(38,500 to 50,000)	12,072.9<br>(10,573.9 to 13,748.3)	41,700<br>(36,500 to 46,900)	11,236.5<br>(9,830.0 to 12,629.8)	37,300<br>(33,100 to 42,400)	9,744.8<br>(8,636.7 to 11,066.6)	42.2<br>(26.3 to 60.3)	-6.9<br>(-16.1 to 2.4)
**Georgia**	All Ages	145,000<br>(136,000 to 156,000)	2,632.7<br>(2,469.1 to 2,824.8)	96,800<br>(90,200 to 104,000)	2,634.7<br>(2,455.2 to 2,823.5)	89,100<br>(82,000 to 97,600)	2,444.0<br>(2,248.6 to 2,678.6)	80,200<br>(74,800 to 85,700)	2,222.8<br>(2,074.8 to 2,375.9)	0.1<br>(-7.1 to 7.9)	-7.2<br>(-15.3 to 1.8)
	Under 5	42,300<br>(37,500 to 48,500)	9,019.7<br>(7,997.7 to 10,357.2)	5,660<br>(4,970 to 6,470)	2,185.4<br>(1,917.0 to 2,495.9)	4,890<br>(3,860 to 5,970)	1,942.1<br>(1,535.8 to 2,372.1)	3,810<br>(3,320 to 4,350)	1,565.6<br>(1,364.4 to 1,789.6)	-75.8<br>(-78.6 to -72.9)	-11.1<br>(-24.9 to 4.5)
	5-14 years	22,700<br>(19,000 to 27,000)	2,520.8<br>(2,115.8 to 3,002.3)	5,110<br>(4,090 to 6,120)	1,069.5<br>(855.6 to 1,279.8)	4,900<br>(3,980 to 5,940)	1,008.7<br>(818.9 to 1,222.7)	4,580<br>(3,720 to 5,440)	928.6<br>(754.5 to 1,104.0)	-57.6<br>(-64.3 to -50.3)	-5.7<br>(-14.5 to 4.4)

Location	Age Group	1990		2019		2020		2021		Percent Incidence Rate Change from 1990-2019 (95% UI)	Percent Incidence Rate Change from 2019-2021 (95% UI)
		Episodes (95% UI)	Rate (95% UI)	Episodes (95% UI)	Rate (95% UI)	Episodes (95% UI)	Rate (95% UI)	Episodes (95% UI)	Rate (95% UI)		
	15-49 years	26,100 (23,700 to 29,000)	972.5 (883.0 to 1,079.7)	13,300 (12,200 to 14,600)	806.8 (736.0 to 882.5)	12,800 (11,500 to 14,300)	788.2 (708.6 to 879.9)	11,300 (10,400 to 12,500)	708.7 (649.7 to 780.7)	-17.0 (-24.6 to -9.6)	-2.3 (-10.9 to 7.6)
	50-69 years	29,100 (26,200 to 32,100)	2,544.9 (2,297.0 to 2,811.8)	31,600 (28,600 to 34,600)	3,466.1 (3,136.2 to 3,792.7)	29,800 (26,600 to 33,300)	3,289.2 (2,930.6 to 3,674.5)	27,400 (24,700 to 30,200)	3,051.6 (2,744.0 to 3,358.1)	36.2 (24.2 to 48.8)	-5.1 (-14.2 to 5.4)
	70+ years	25,300 (22,800 to 29,000)	7,662.7 (6,914.4 to 8,786.8)	41,100 (37,100 to 46,400)	10,973.5 (9,902.7 to 12,389.3)	36,700 (32,700 to 41,600)	9,757.1 (8,706.9 to 11,052.5)	33,000 (29,500 to 36,800)	8,858.2 (7,914.4 to 9,875.4)	43.2 (25.8 to 59.0)	-11.1 (-20.7 to 0.8)
**Kazakhstan**	All Ages	478,000 (448,000 to 518,000)	2,916.4 (2,734.1 to 3,160.9)	383,000 (360,000 to 408,000)	2,072.6 (1,950.1 to 2,209.2)	365,000 (336,000 to 395,000)	1,948.6 (1,792.7 to 2,107.9)	329,000 (306,000 to 356,000)	1,734.6 (1,615.1 to 1,879.7)	-28.9 (-34.0 to -23.4)	-6.0 (-13.2 to 1.7)
	Under 5	180,000 (159,000 to 206,000)	9,580.8 (8,447.4 to 10,924.3)	56,700 (49,600 to 64,300)	2,982.7 (2,608.7 to 3,384.0)	52,100 (44,000 to 60,900)	2,709.2 (2,285.3 to 3,167.0)	41,600 (36,500 to 47,600)	2,134.6 (1,873.3 to 2,444.0)	-68.9 (-72.8 to -64.8)	-9.2 (-19.9 to 3.4)
	5-14 years	68,800 (56,800 to 82,400)	2,075.7 (1,714.7 to 2,487.2)	36,000 (27,900 to 44,200)	1,100.0 (852.8 to 1,351.2)	35,200 (27,300 to 43,000)	1,041.1 (808.1 to 1,271.7)	33,100 (26,300 to 40,400)	952.8 (756.1 to 1,162.8)	-47.0 (-53.6 to -38.6)	-5.4 (-15.6 to 5.6)
	15-49 years	88,300 (80,100 to 98,000)	1,074.6 (975.3 to 1,192.3)	90,900 (83,300 to 100,000)	983.0 (901.1 to 1,082.6)	89,700 (80,400 to 99,300)	964.2 (864.6 to 1,067.7)	80,500 (72,400 to 89,200)	860.6 (773.4 to 953.3)	-8.5 (-16.6 to -0.1)	-1.9 (-10.3 to 8.5)
	50-69 years	83,000 (75,800 to 90,400)	3,487.6 (3,182.8 to 3,798.4)	119,000 (109,000 to 132,000)	3,650.8 (3,341.9 to 4,063.7)	115,000 (103,000 to 128,000)	3,467.0 (3,107.0 to 3,860.1)	107,000 (95,900 to 121,000)	3,196.7 (2,855.2 to 3,587.5)	4.7 (-4.4 to 15.7)	-5.0 (-14.6 to 4.7)
	70+ years	57,600 (50,300 to 66,800)	9,603.0 (8,386.8 to 11,130.0)	80,000 (72,100 to 89,600)	10,291.0 (9,274.0 to 11,529.7)	72,900 (64,200 to 82,500)	9,117.9 (8,035.9 to 10,319.6)	66,100 (59,800 to 75,200)	8,133.3 (7,350.1 to 9,245.4)	7.2 (-3.7 to 19.2)	-11.4 (-21.6 to -2.7)
**Kyrgyzstan**	All Ages	191,000 (179,000 to 207,000)	4,280.1 (4,006.7 to 4,640.2)	117,000 (109,000 to 125,000)	1,757.0 (1,642.5 to 1,880.2)	113,000 (103,000 to 123,000)	1,665.5 (1,530.3 to 1,819.1)	101,000 (93,600 to 110,000)	1,474.4 (1,363.6 to 1,601.3)	-58.9 (-61.7 to -55.7)	-5.2 (-12.5 to 1.8)
	Under 5	100,000 (90,200 to 114,000)	15,604.2 (14,032.3 to 17,690.5)	33,300 (29,400 to 37,900)	4,101.5 (3,612.8 to 4,660.9)	30,200 (25,700 to 35,000)	3,745.9 (3,187.6 to 4,345.7)	24,000 (20,700 to 28,300)	3,022.0 (2,603.8 to 3,565.0)	-73.7 (-76.7 to -70.7)	-8.7 (-20.9 to 4.7)
	5-14 years	32,200 (27,400 to 38,400)	3,108.7 (2,643.1 to 3,709.6)	20,300 (16,100 to 24,700)	1,465.5 (1,166.5 to 1,788.7)	20,100 (16,000 to 24,800)	1,404.6 (1,117.4 to 1,732.0)	19,200 (15,100 to 23,700)	1,293.7 (1,020.0 to 1,603.5)	-52.9 (-61.0 to -43.3)	-4.2 (-13.6 to 6.0)
	15-49 years	25,400 (22,900 to 28,100)	1,212.7 (1,092.1 to 1,339.3)	26,800 (23,900 to 29,800)	794.6 (708.8 to 883.1)	27,100 (24,100 to 30,400)	795.9 (708.5 to 894.0)	24,500 (21,500 to 27,300)	712.4 (626.4 to 793.4)	-34.5 (-40.7 to -27.0)	0.2 (-7.5 to 8.9)
	50-69 years	18,300 (16,500 to 20,300)	3,340.1 (3,024.0 to 3,711.6)	19,900 (17,900 to 22,300)	2,190.4 (1,964.1 to 2,447.6)	20,000 (18,000 to 22,300)	2,137.4 (1,926.8 to 2,383.9)	19,000 (16,900 to 21,100)	1,978.1 (1,757.7 to 2,204.8)	-34.4 (-39.7 to -28.3)	-2.4 (-11.5 to 7.9)
	70+ years	14,900 (13,200 to 16,500)	10,317.4 (9,114.7 to 11,397.1)	16,600 (14,800 to 19,000)	9,332.7 (8,318.1 to 10,693.0)	15,300 (13,500 to 17,300)	8,254.6 (7,303.1 to 9,371.9)	14,600 (12,900 to 16,400)	7,597.5 (6,742.4 to 8,536.3)	-9.5 (-17.4 to 0.7)	-11.6 (-20.9 to -1.8)
**Mongolia**	All Ages	93,700 (85,700 to 102,000)	4,342.9 (3,970.3 to 4,738.8)	48,400 (45,100 to 51,600)	1,503.0 (1,400.1 to 1,602.0)	49,400 (46,100 to 52,900)	1,507.1 (1,405.0 to 1,611.3)	41,800 (39,000 to 44,800)	1,252.7 (1,170.4 to 1,344.3)	-65.4 (-67.7 to -62.7)	0.3 (-3.7 to 5.1)

Location	Age Group	1990		2019		2020		2021		Percent Incidence Rate Change from 1990-2019 (95% UI)	Percent Incidence Rate Change from 2019-2021 (95% UI)
		Episodes (95% UI)	Rate (95% UI)	Episodes (95% UI)	Rate (95% UI)	Episodes (95% UI)	Rate (95% UI)	Episodes (95% UI)	Rate (95% UI)		
	Under 5	50,900 (44,900 to 57,500)	14,990.6 (13,233.9 to 16,940.7)	12,900 (11,300 to 14,600)	3,236.0 (2,839.6 to 3,673.5)	12,800 (11,300 to 14,800)	3,245.8 (2,848.3 to 3,737.0)	9,130 (8,000 to 10,500)	2,336.8 (2,048.9 to 2,689.4)	-78.4 (-80.7 to -75.9)	0.3 (-7.0 to 7.7)
	5-14 years	15,000 (12,200 to 18,300)	2,681.0 (2,170.0 to 3,272.3)	7,440 (5,850 to 9,110)	1,183.7 (930.6 to 1,449.5)	7,720 (6,050 to 9,450)	1,164.5 (911.7 to 1,425.3)	7,080 (5,760 to 8,660)	1,017.8 (827.1 to 1,244.2)	-55.8 (-61.8 to -49.4)	-1.6 (-11.7 to 9.2)
	15-49 years	13,000 (11,600 to 14,400)	1,267.7 (1,124.8 to 1,403.0)	12,400 (11,300 to 13,800)	742.5 (672.4 to 822.1)	12,800 (11,600 to 14,100)	764.4 (688.0 to 841.4)	11,000 (9,820 to 12,200)	649.4 (582.0 to 724.8)	-41.4 (-46.9 to -35.8)	2.9 (-4.1 to 11.7)
	50-69 years	7,760 (6,790 to 8,650)	4,322.6 (3,783.5 to 4,816.7)	9,520 (8,350 to 10,700)	2,144.2 (1,879.4 to 2,413.0)	10,000 (8,830 to 11,300)	2,165.3 (1,910.7 to 2,452.8)	9,130 (8,020 to 10,300)	1,906.6 (1,674.3 to 2,142.1)	-50.4 (-56.3 to -44.9)	1.0 (-8.3 to 15.6)
	70+ years	7,010 (5,970 to 8,080)	13,640.2 (11,624.6 to 15,722.8)	6,180 (5,410 to 6,960)	7,899.2 (6,917.5 to 8,907.2)	6,060 (5,270 to 6,910)	7,467.4 (6,492.0 to 8,512.9)	5,500 (4,800 to 6,130)	6,532.4 (5,710.5 to 7,287.0)	-42.1 (-47.9 to -35.6)	-5.5 (-14.0 to 4.6)
**Tajikistan**	All Ages	278,000 (257,000 to 302,000)	5,174.7 (4,792.0 to 5,623.9)	241,000 (225,000 to 257,000)	2,482.0 (2,319.0 to 2,642.8)	243,000 (223,000 to 260,000)	2,438.7 (2,246.7 to 2,617.0)	215,000 (201,000 to 229,000)	2,112.5 (1,982.4 to 2,254.0)	-52.0 (-54.9 to -48.7)	-1.7 (-7.5 to 3.3)
	Under 5	152,000 (137,000 to 170,000)	16,069.9 (14,459.8 to 17,988.0)	75,300 (66,800 to 85,000)	5,713.0 (5,072.5 to 6,449.2)	74,200 (64,600 to 83,400)	5,578.0 (4,859.6 to 6,273.3)	59,000 (52,800 to 66,500)	4,409.0 (3,944.3 to 4,966.0)	-64.4 (-67.4 to -61.4)	-2.4 (-10.7 to 5.3)
	5-14 years	43,700 (36,700 to 52,800)	3,171.8 (2,668.1 to 3,832.6)	35,900 (29,300 to 43,100)	1,710.8 (1,395.9 to 2,052.3)	36,500 (30,100 to 43,400)	1,681.5 (1,383.7 to 1,996.2)	34,300 (28,700 to 40,200)	1,525.1 (1,276.5 to 1,791.5)	-46.1 (-52.9 to -38.1)	-1.7 (-12.3 to 11.3)
	15-49 years	41,700 (37,900 to 45,800)	1,728.2 (1,570.9 to 1,901.2)	59,100 (52,800 to 64,600)	1,187.3 (1,062.0 to 1,298.0)	60,600 (54,500 to 66,600)	1,198.1 (1,077.4 to 1,317.7)	54,700 (48,900 to 60,500)	1,063.7 (950.9 to 1,176.8)	-31.3 (-37.8 to -24.2)	0.9 (-7.8 to 8.8)
	50-69 years	22,900 (20,900 to 25,400)	4,512.5 (4,100.9 to 5,000.5)	42,100 (37,700 to 47,100)	3,676.6 (3,288.1 to 4,113.7)	43,500 (39,300 to 49,100)	3,645.9 (3,288.0 to 4,110.2)	41,500 (37,300 to 46,300)	3,355.8 (3,018.2 to 3,747.3)	-18.5 (-25.6 to -9.7)	-0.8 (-10.5 to 8.3)
	70+ years	17,600 (15,200 to 19,900)	13,760.6 (11,885.7 to 15,547.8)	29,000 (25,600 to 32,500)	15,543.5 (13,721.4 to 17,452.7)	27,700 (24,500 to 32,200)	14,324.9 (12,664.4 to 16,629.7)	25,200 (22,100 to 28,700)	12,670.7 (11,115.4 to 14,431.2)	13.0 (0.0 to 28.6)	-7.8 (-17.4 to 1.2)
**Turkmenistan**	All Ages	177,000 (163,000 to 191,000)	4,773.8 (4,416.2 to 5,151.3)	93,000 (86,600 to 99,500)	1,835.7 (1,708.6 to 1,964.0)	93,000 (86,600 to 100,000)	1,817.5 (1,693.5 to 1,954.9)	79,700 (74,200 to 86,100)	1,545.1 (1,439.1 to 1,668.8)	-61.5 (-64.6 to -58.7)	-1.0 (-7.9 to 4.8)
	Under 5	111,000 (100,000 to 123,000)	18,877.2 (17,138.1 to 20,939.8)	30,700 (26,900 to 34,700)	5,666.9 (4,969.4 to 6,403.6)	30,100 (26,500 to 34,500)	5,570.8 (4,902.8 to 6,377.2)	22,700 (20,000 to 25,600)	4,208.0 (3,710.1 to 4,745.7)	-70.0 (-73.2 to -66.3)	-1.7 (-13.2 to 10.8)
	5-14 years	24,500 (20,400 to 29,100)	2,677.4 (2,228.2 to 3,182.1)	14,200 (11,600 to 17,100)	1,475.5 (1,206.1 to 1,772.8)	14,200 (11,500 to 16,800)	1,453.0 (1,181.7 to 1,728.4)	12,900 (10,700 to 15,400)	1,313.8 (1,086.7 to 1,564.3)	-44.9 (-52.9 to -35.9)	-1.5 (-12.1 to 11.4)
	15-49 years	21,300 (19,400 to 23,500)	1,212.6 (1,106.1 to 1,336.5)	22,400 (20,500 to 24,600)	846.9 (772.8 to 929.0)	23,100 (20,900 to 25,200)	868.5 (783.9 to 947.3)	20,500 (18,300 to 22,800)	767.2 (684.8 to 852.9)	-30.2 (-35.1 to -24.1)	2.6 (-5.3 to 10.8)

Location	Age Group	1990		2019		2020		2021		Percent Incidence Rate Change from 1990-2019 (95% UI)	Percent Incidence Rate Change from 2019-2021 (95% UI)
		Episodes (95% UI)	Rate (95% UI)	Episodes (95% UI)	Rate (95% UI)	Episodes (95% UI)	Rate (95% UI)	Episodes (95% UI)	Rate (95% UI)		
	50-69 years	11,300 (10,100 to 12,600)	3,174.1 (2,835.9 to 3,532.5)	14,800 (13,400 to 16,500)	1,940.5 (1,757.0 to 2,166.4)	15,300 (13,700 to 16,800)	1,945.0 (1,741.4 to 2,140.8)	14,200 (12,800 to 15,700)	1,770.5 (1,595.7 to 1,957.4)	-38.9 (-45.4 to -31.3)	0.2 (-8.7 to 9.8)
	70+ years	8,940 (7,970 to 10,100)	10,559.8 (9,417.3 to 11,911.0)	10,900 (9,810 to 12,300)	7,233.0 (6,529.8 to 8,197.0)	10,300 (9,190 to 11,800)	6,747.6 (6,035.7 to 7,769.3)	9,340 (8,400 to 10,500)	5,965.8 (5,361.3 to 6,706.0)	-31.5 (-39.2 to -24.1)	-6.7 (-15.2 to 1.9)
**Uzbekistan**	All Ages	864,000 (804,000 to 938,000)	4,121.2 (3,837.4 to 4,475.7)	706,000 (659,000 to 755,000)	2,132.8 (1,991.3 to 2,282.9)	708,000 (658,000 to 766,000)	2,101.5 (1,955.1 to 2,275.7)	610,000 (570,000 to 652,000)	1,782.6 (1,666.0 to 1,904.6)	-48.2 (-51.7 to -44.9)	-1.5 (-7.8 to 5.0)
	Under 5	471,000 (422,000 to 529,000)	13,964.8 (12,522.9 to 15,688.5)	183,000 (161,000 to 205,000)	4,953.2 (4,351.9 to 5,529.1)	181,000 (158,000 to 207,000)	4,786.1 (4,191.4 to 5,469.6)	134,000 (119,000 to 152,000)	3,503.0 (3,102.9 to 3,951.4)	-64.5 (-68.6 to -60.3)	-3.4 (-13.4 to 7.7)
	5-14 years	144,000 (124,000 to 168,000)	2,784.1 (2,401.9 to 3,247.3)	111,000 (91,700 to 130,000)	1,883.5 (1,557.4 to 2,211.3)	113,000 (94,400 to 136,000)	1,864.9 (1,555.0 to 2,243.9)	102,000 (85,600 to 120,000)	1,623.0 (1,368.5 to 1,918.9)	-32.3 (-40.5 to -23.6)	-1.0 (-13.2 to 14.2)
	15-49 years	128,000 (115,000 to 142,000)	1,303.9 (1,175.7 to 1,450.7)	180,000 (163,000 to 198,000)	1,023.7 (926.9 to 1,127.2)	184,000 (167,000 to 206,000)	1,038.8 (946.0 to 1,163.9)	160,000 (146,000 to 175,000)	898.0 (821.5 to 981.3)	-21.5 (-27.5 to -14.8)	1.5 (-6.5 to 10.4)
	50-69 years	65,300 (59,400 to 73,000)	3,181.1 (2,893.0 to 3,556.1)	144,000 (126,000 to 160,000)	2,864.4 (2,509.1 to 3,183.8)	148,000 (133,000 to 162,000)	2,830.4 (2,545.9 to 3,112.6)	138,000 (124,000 to 155,000)	2,568.7 (2,312.9 to 2,891.8)	-10.0 (-19.4 to -1.7)	-1.2 (-10.8 to 10.0)
	70+ years	55,600 (49,200 to 62,100)	9,752.6 (8,633.9 to 10,884.7)	87,900 (78,400 to 101,000)	9,716.1 (8,670.5 to 11,127.7)	82,000 (71,100 to 93,100)	9,120.8 (7,916.3 to 10,355.5)	76,700 (68,500 to 86,900)	8,039.8 (7,186.4 to 9,114.4)	-0.4 (-9.6 to 10.8)	-6.1 (-18.5 to 4.1)
**Albania**	All Ages	102,000 (93,700 to 111,000)	3,090.9 (2,835.5 to 3,356.6)	32,500 (30,100 to 34,900)	1,199.1 (1,112.6 to 1,287.0)	30,900 (28,400 to 33,400)	1,148.5 (1,058.0 to 1,240.4)	25,600 (23,700 to 27,600)	960.3 (887.9 to 1,034.5)	-61.2 (-64.1 to -57.8)	-4.2 (-11.0 to 2.8)
	Under 5	55,200 (48,300 to 61,900)	13,666.1 (11,965.7 to 15,338.4)	5,520 (4,820 to 6,340)	3,703.9 (3,229.4 to 4,251.6)	4,880 (4,160 to 5,710)	3,346.7 (2,850.8 to 3,915.3)	3,390 (2,960 to 3,890)	2,374.8 (2,077.0 to 2,728.8)	-72.9 (-75.7 to -69.9)	-9.6 (-19.1 to 0.6)
	5-14 years	15,800 (12,800 to 19,000)	2,208.5 (1,789.4 to 2,665.4)	2,780 (2,220 to 3,360)	893.9 (714.8 to 1,080.7)	2,620 (2,060 to 3,220)	858.5 (675.4 to 1,054.9)	2,260 (1,780 to 2,800)	749.7 (591.1 to 929.0)	-59.5 (-64.4 to -53.8)	-4.0 (-11.8 to 4.9)
	15-49 years	9,560 (8,420 to 10,800)	558.7 (491.9 to 630.0)	3,950 (3,480 to 4,490)	303.8 (268.1 to 345.6)	3,710 (3,240 to 4,190)	290.3 (253.3 to 327.8)	3,170 (2,760 to 3,600)	251.4 (218.8 to 285.5)	-45.6 (-50.1 to -40.9)	-4.4 (-10.6 to 2.1)
	50-69 years	9,640 (8,700 to 10,700)	2,557.3 (2,310.2 to 2,850.5)	7,580 (6,780 to 8,480)	1,102.3 (987.1 to 1,233.2)	7,380 (6,480 to 8,280)	1,065.7 (934.7 to 1,194.5)	6,450 (5,700 to 7,180)	929.9 (821.4 to 1,034.8)	-56.9 (-60.8 to -52.1)	-3.3 (-11.8 to 5.7)
	70+ years	12,000 (10,800 to 13,400)	12,048.2 (10,778.4 to 13,445.3)	12,600 (11,200 to 14,300)	4,843.9 (4,293.2 to 5,472.7)	12,300 (10,900 to 14,000)	4,620.8 (4,110.2 to 5,280.1)	10,400 (9,180 to 11,700)	3,819.4 (3,386.3 to 4,328.0)	-59.8 (-63.8 to -54.5)	-4.6 (-14.7 to 5.6)
**Bosnia and Herzegovina**	All Ages	76,400 (71,000 to 82,600)	1,699.4 (1,578.0 to 1,836.2)	38,600 (35,900 to 41,800)	1,138.6 (1,057.6 to 1,233.2)	36,800 (34,000 to 39,700)	1,099.7 (1,015.0 to 1,184.8)	31,900 (29,500 to 34,300)	965.1 (894.6 to 1,039.4)	-33.0 (-37.2 to -28.3)	-3.4 (-9.9 to 3.2)
	Under 5	21,900 (18,600 to 25,100)	6,224.3 (5,299.9 to 7,145.7)	4,440 (3,690 to 5,350)	2,803.4 (2,331.9 to 3,379.3)	4,010 (3,290 to 4,890)	2,587.3 (2,124.3 to 3,152.7)	3,040 (2,500 to 3,690)	2,012.3 (1,657.0 to 2,444.5)	-55.0 (-60.3 to -49.1)	-7.7 (-16.2 to 1.6)

Location	Age Group	1990		2019		2020		2021		Percent Incidence Rate Change from 1990-2019 (95% UI)	Percent Incidence Rate Change from 2019-2021 (95% UI)
		Episodes (95% UI)	Rate (95% UI)	Episodes (95% UI)	Rate (95% UI)	Episodes (95% UI)	Rate (95% UI)	Episodes (95% UI)	Rate (95% UI)		
	5-14 years	12,800 (10,000 to 16,200)	1,717.3 (1,343.6 to 2,177.0)	3,700 (2,850 to 4,760)	1,066.6 (820.5 to 1,370.4)	3,510 (2,680 to 4,440)	1,023.2 (780.8 to 1,294.1)	3,120 (2,370 to 4,040)	917.7 (698.6 to 1,190.8)	-37.9 (-43.8 to -29.8)	-4.1 (-11.5 to 5.2)
	15-49 years	12,400 (10,800 to 14,000)	517.4 (452.7 to 585.1)	4,780 (4,180 to 5,410)	308.9 (270.0 to 349.6)	4,520 (3,900 to 5,100)	297.5 (257.0 to 335.4)	4,000 (3,460 to 4,550)	268.4 (232.1 to 305.3)	-40.3 (-44.3 to -35.8)	-3.7 (-10.1 to 3.3)
	50-69 years	15,900 (14,200 to 17,700)	1,888.5 (1,684.1 to 2,104.0)	9,780 (8,780 to 10,700)	1,024.5 (919.6 to 1,124.5)	9,430 (8,380 to 10,500)	997.9 (886.6 to 1,108.3)	8,420 (7,550 to 9,330)	900.4 (807.7 to 997.6)	-45.8 (-51.0 to -40.3)	-2.6 (-11.7 to 7.3)
	70+ years	13,500 (11,800 to 15,300)	8,060.5 (7,020.4 to 9,125.4)	15,900 (14,100 to 18,100)	4,149.5 (3,668.7 to 4,710.4)	15,400 (13,700 to 17,300)	3,966.8 (3,528.3 to 4,461.9)	13,300 (12,000 to 14,900)	3,424.2 (3,083.7 to 3,826.8)	-48.5 (-54.0 to -42.0)	-4.4 (-13.9 to 6.3)
**Bulgaria**	All Ages	263,000 (248,000 to 281,000)	3,028.5 (2,857.7 to 3,239.2)	96,600 (90,300 to 105,000)	1,392.4 (1,302.2 to 1,507.6)	91,100 (84,400 to 98,000)	1,325.4 (1,228.3 to 1,425.6)	76,600 (71,400 to 82,000)	1,128.4 (1,051.4 to 1,208.1)	-54.0 (-57.5 to -50.6)	-4.8 (-11.6 to 2.7)
	Under 5	54,700 (47,900 to 63,000)	10,187.3 (8,930.6 to 11,729.2)	9,460 (8,270 to 10,900)	3,010.9 (2,630.8 to 3,477.5)	8,420 (7,110 to 9,730)	2,726.0 (2,303.6 to 3,152.2)	6,010 (5,240 to 6,980)	1,986.8 (1,732.2 to 2,307.0)	-70.4 (-73.8 to -66.6)	-9.5 (-18.9 to 1.4)
	5-14 years	29,900 (25,100 to 35,600)	2,492.2 (2,089.2 to 2,972.3)	5,840 (4,570 to 7,220)	863.4 (675.2 to 1,067.6)	5,610 (4,520 to 6,830)	829.6 (668.6 to 1,010.1)	4,890 (3,870 to 6,050)	725.7 (574.3 to 899.0)	-65.4 (-70.6 to -59.6)	-3.9 (-11.9 to 5.5)
	15-49 years	35,800 (32,700 to 38,700)	861.1 (786.7 to 931.4)	12,000 (10,800 to 13,300)	394.2 (354.8 to 439.8)	11,400 (10,300 to 12,600)	381.8 (345.9 to 422.0)	9,900 (8,890 to 10,900)	337.2 (302.7 to 372.2)	-54.2 (-58.0 to -49.9)	-3.1 (-10.5 to 5.7)
	50-69 years	74,800 (69,400 to 80,600)	3,517.7 (3,260.6 to 3,789.2)	28,000 (25,400 to 31,000)	1,486.8 (1,345.5 to 1,646.7)	26,200 (23,300 to 29,000)	1,407.9 (1,250.6 to 1,556.5)	22,900 (20,700 to 25,200)	1,241.2 (1,123.2 to 1,363.7)	-57.7 (-62.0 to -53.4)	-5.3 (-13.7 to 3.5)
	70+ years	67,700 (61,300 to 76,200)	10,259.8 (9,293.6 to 11,549.2)	41,300 (37,300 to 47,200)	4,013.2 (3,625.0 to 4,583.5)	39,400 (34,600 to 44,200)	3,786.6 (3,324.4 to 4,246.0)	32,900 (29,300 to 37,300)	3,194.0 (2,848.6 to 3,626.3)	-60.9 (-65.3 to -56.5)	-5.6 (-14.4 to 4.2)
**Croatia**	All Ages	66,300 (62,100 to 71,700)	1,363.2 (1,277.0 to 1,474.2)	40,700 (37,800 to 43,700)	956.6 (889.2 to 1,028.0)	39,000 (36,100 to 41,900)	921.0 (853.6 to 990.9)	33,400 (31,000 to 36,300)	793.3 (736.8 to 861.7)	-29.8 (-34.2 to -25.5)	-3.7 (-9.9 to 3.4)
	Under 5	10,900 (9,150 to 12,800)	3,602.5 (3,036.5 to 4,265.2)	3,860 (3,170 to 4,690)	2,056.1 (1,691.2 to 2,502.1)	3,460 (2,800 to 4,280)	1,870.1 (1,514.2 to 2,316.4)	2,470 (2,020 to 2,990)	1,353.4 (1,107.5 to 1,639.4)	-42.9 (-48.7 to -36.7)	-9.0 (-18.2 to 0.8)
	5-14 years	7,020 (5,360 to 9,080)	1,022.9 (781.6 to 1,324.8)	2,870 (2,200 to 3,690)	677.3 (519.5 to 870.1)	2,710 (2,070 to 3,470)	645.9 (492.5 to 825.5)	2,370 (1,820 to 3,050)	571.4 (437.8 to 734.3)	-33.8 (-39.0 to -27.8)	-4.6 (-10.8 to 3.2)
	15-49 years	9,410 (8,320 to 10,600)	385.5 (341.0 to 433.1)	4,040 (3,540 to 4,570)	216.8 (190.2 to 245.5)	3,850 (3,380 to 4,360)	208.5 (182.9 to 236.4)	3,400 (2,980 to 3,860)	185.9 (163.0 to 211.0)	-43.8 (-47.7 to -39.7)	-3.8 (-9.6 to 2.3)
	50-69 years	20,200 (18,200 to 22,400)	1,755.5 (1,583.5 to 1,946.4)	9,730 (8,670 to 10,700)	822.5 (733.4 to 906.4)	9,340 (8,400 to 10,400)	794.8 (714.6 to 882.0)	8,250 (7,490 to 9,170)	707.5 (642.0 to 786.2)	-53.1 (-58.1 to -48.6)	-3.4 (-11.9 to 4.7)
	70+ years	18,800 (17,000 to 21,000)	6,635.9 (5,996.1 to 7,392.3)	20,200 (18,100 to 22,400)	3,379.8 (3,022.6 to 3,749.4)	19,600 (17,700 to 21,600)	3,227.7 (2,913.6 to 3,557.3)	16,900 (15,100 to 19,100)	2,747.8 (2,453.9 to 3,103.6)	-49.1 (-53.3 to -43.8)	-4.5 (-12.5 to 4.8)
**Czechia**	All Ages	161,000 (150,000 to 173,000)	1,567.4 (1,456.6 to 1,681.9)	138,000 (127,000 to 148,000)	1,296.0 (1,197.4 to 1,392.3)	133,000 (122,000 to 143,000)	1,245.0 (1,147.3 to 1,342.0)	114,000 (106,000 to 124,000)	1,075.4 (995.7 to 1,167.9)	-17.3 (-22.2 to -11.8)	-3.9 (-10.1 to 2.5)

Location	Age Group	1990		2019		2020		2021		Percent Incidence Rate Change from 1990-2019 (95% UI)	Percent Incidence Rate Change from 2019-2021 (95% UI)
		Episodes (95% UI)	Rate (95% UI)	Episodes (95% UI)	Rate (95% UI)	Episodes (95% UI)	Rate (95% UI)	Episodes (95% UI)	Rate (95% UI)		
	Under 5	25,500 (22,000 to 29,500)	3,930.0 (3,396.9 to 4,550.2)	11,900 (9,930 to 14,200)	2,094.8 (1,741.3 to 2,497.5)	10,800 (8,880 to 13,400)	1,901.8 (1,561.0 to 2,353.4)	7,710 (6,410 to 9,320)	1,371.8 (1,140.5 to 1,658.1)	-46.7 (-51.3 to -41.4)	-9.2 (-19.4 to 0.7)
	5-14 years	16,000 (12,500 to 20,200)	1,028.4 (804.6 to 1,301.8)	8,200 (6,260 to 10,600)	720.0 (550.1 to 928.2)	7,870 (6,080 to 9,980)	685.6 (529.4 to 869.1)	7,010 (5,360 to 8,940)	607.6 (464.1 to 774.7)	-30.0 (-36.4 to -24.1)	-4.8 (-11.5 to 3.1)
	15-49 years	23,000 (20,600 to 25,800)	442.2 (396.6 to 494.9)	14,200 (12,700 to 15,900)	296.8 (264.7 to 330.8)	13,800 (12,100 to 15,300)	289.3 (254.3 to 321.1)	12,100 (10,800 to 13,600)	257.4 (228.7 to 287.3)	-32.9 (-37.7 to -27.5)	-2.5 (-10.2 to 5.3)
	50-69 years	41,500 (37,700 to 45,800)	1,989.5 (1,806.5 to 2,192.4)	34,600 (30,900 to 38,200)	1,297.2 (1,156.4 to 1,431.9)	32,900 (29,400 to 36,600)	1,235.8 (1,105.3 to 1,374.6)	28,800 (25,600 to 32,300)	1,083.7 (962.5 to 1,216.3)	-34.8 (-41.4 to -28.7)	-4.7 (-11.4 to 3.9)
	70+ years	55,300 (49,300 to 62,100)	6,941.5 (6,191.6 to 7,795.5)	69,000 (62,100 to 77,000)	4,694.7 (4,227.9 to 5,241.2)	67,200 (60,500 to 74,400)	4,451.2 (4,003.7 to 4,928.7)	58,700 (52,900 to 66,000)	3,801.2 (3,424.8 to 4,276.6)	-32.4 (-38.6 to -25.8)	-5.2 (-13.7 to 3.3)
**Hungary**	All Ages	163,000 (152,000 to 175,000)	1,572.4 (1,466.6 to 1,681.2)	110,000 (101,000 to 118,000)	1,131.0 (1,047.0 to 1,222.1)	103,000 (94,900 to 112,000)	1,069.2 (983.1 to 1,157.7)	85,400 (79,400 to 92,800)	889.3 (827.4 to 966.9)	-28.1 (-31.7 to -24.3)	-5.5 (-11.6 to 1.8)
	Under 5	35,000 (30,100 to 40,600)	5,658.1 (4,868.8 to 6,553.0)	12,800 (10,700 to 15,400)	2,777.9 (2,328.2 to 3,336.7)	11,400 (9,300 to 13,900)	2,489.0 (2,029.0 to 3,023.7)	7,670 (6,400 to 9,180)	1,688.7 (1,407.8 to 2,020.6)	-50.9 (-55.7 to -45.5)	-10.4 (-20.2 to 1.3)
	5-14 years	21,000 (16,100 to 27,000)	1,389.2 (1,064.4 to 1,784.7)	9,710 (7,390 to 12,600)	1,028.1 (782.1 to 1,333.0)	9,190 (6,900 to 11,900)	977.7 (734.4 to 1,265.4)	7,860 (5,930 to 10,100)	841.1 (634.9 to 1,081.8)	-26.0 (-32.8 to -18.8)	-4.9 (-12.7 to 3.2)
	15-49 years	26,500 (23,700 to 29,600)	521.6 (466.2 to 581.4)	13,400 (11,800 to 15,100)	302.4 (266.2 to 339.3)	12,700 (11,100 to 14,100)	288.4 (253.6 to 321.2)	10,800 (9,560 to 12,300)	248.7 (220.3 to 282.2)	-42.0 (-46.2 to -37.3)	-4.6 (-11.2 to 2.4)
	50-69 years	37,600 (34,300 to 40,900)	1,615.7 (1,473.7 to 1,757.1)	26,600 (24,200 to 29,400)	1,045.5 (951.4 to 1,159.1)	25,100 (22,500 to 27,800)	990.0 (888.5 to 1,097.1)	21,500 (19,800 to 23,900)	853.8 (784.8 to 948.9)	-35.3 (-41.1 to -29.8)	-5.3 (-12.0 to 2.7)
	70+ years	43,300 (38,400 to 47,600)	5,104.5 (4,529.9 to 5,618.2)	47,100 (42,400 to 53,000)	3,611.4 (3,250.2 to 4,065.8)	44,800 (39,600 to 50,500)	3,374.4 (2,983.4 to 3,800.9)	37,500 (33,500 to 42,200)	2,784.7 (2,489.3 to 3,131.0)	-29.2 (-36.5 to -22.2)	-6.6 (-15.2 to 3.4)
**Montenegro**	All Ages	9,590 (8,860 to 10,400)	1,531.2 (1,415.4 to 1,658.2)	6,580 (6,070 to 7,080)	1,056.6 (975.0 to 1,137.7)	6,300 (5,780 to 6,800)	1,014.5 (931.0 to 1,095.3)	5,360 (4,970 to 5,800)	867.9 (804.2 to 937.6)	-31.0 (-35.0 to -26.2)	-4.0 (-10.8 to 2.6)
	Under 5	3,260 (2,820 to 3,750)	6,203.8 (5,375.8 to 7,148.2)	1,060 (888 to 1,250)	2,870.3 (2,394.9 to 3,379.5)	965 (793 to 1,170)	2,630.0 (2,160.8 to 3,187.5)	709 (587 to 839)	1,957.8 (1,622.0 to 2,318.3)	-53.7 (-59.1 to -48.2)	-8.4 (-17.4 to 1.0)
	5-14 years	1,570 (1,220 to 1,990)	1,434.7 (1,114.5 to 1,826.5)	757 (579 to 985)	1,000.3 (765.1 to 1,301.3)	727 (562 to 927)	964.0 (745.2 to 1,228.1)	649 (500 to 856)	862.8 (664.7 to 1,138.0)	-30.3 (-36.9 to -22.9)	-3.6 (-11.7 to 4.6)
	15-49 years	1,420 (1,240 to 1,600)	445.4 (389.7 to 503.2)	917 (804 to 1,040)	310.1 (272.1 to 350.6)	881 (784 to 994)	299.9 (266.7 to 338.3)	781 (690 to 880)	267.3 (236.3 to 301.4)	-30.4 (-35.0 to -25.8)	-3.3 (-9.3 to 3.3)
	50-69 years	1,590 (1,410 to 1,760)	1,397.3 (1,239.7 to 1,552.5)	1,690 (1,520 to 1,860)	1,075.1 (965.8 to 1,182.8)	1,640 (1,450 to 1,810)	1,048.8 (923.6 to 1,156.7)	1,460 (1,310 to 1,620)	935.4 (835.3 to 1,034.1)	-23.1 (-29.9 to -14.7)	-2.5 (-11.7 to 7.6)
	70+ years	1,760 (1,540 to 2,000)	5,495.4 (4,802.2 to 6,255.0)	2,150 (1,900 to 2,440)	3,786.8 (3,353.7 to 4,296.3)	2,080 (1,810 to 2,360)	3,589.4 (3,121.8 to 4,080.5)	1,760 (1,560 to 1,980)	3,029.9 (2,676.1 to 3,407.3)	-31.1 (-38.7 to -21.7)	-5.2 (-14.8 to 3.9)

Location	Age Group	1990		2019		2020		2021		Percent Incidence Rate Change from 1990-2019 (95% UI)	Percent Incidence Rate Change from 2019-2021 (95% UI)
		Episodes (95% UI)	Rate (95% UI)	Episodes (95% UI)	Rate (95% UI)	Episodes (95% UI)	Rate (95% UI)	Episodes (95% UI)	Rate (95% UI)		
**North Macedonia**	All Ages	34,100 (31,500 to 37,100)	1,711.8 (1,580.5 to 1,864.4)	19,000 (17,600 to 20,500)	869.3 (804.5 to 938.9)	18,300 (16,700 to 19,700)	836.6 (764.1 to 902.6)	15,800 (14,700 to 17,100)	724.5 (673.7 to 784.5)	-49.2 (-52.6 to -45.6)	-3.8 (-9.4 to 2.8)
	Under 5	12,600 (11,000 to 14,500)	7,426.1 (6,465.5 to 8,519.1)	2,810 (2,400 to 3,300)	2,635.3 (2,244.0 to 3,087.7)	2,510 (2,100 to 2,980)	2,422.5 (2,019.8 to 2,869.5)	1,860 (1,580 to 2,180)	1,847.8 (1,567.3 to 2,172.0)	-64.5 (-68.5 to -60.7)	-8.1 (-16.8 to 1.8)
	5-14 years	6,240 (4,970 to 7,650)	1,752.0 (1,395.6 to 2,147.2)	2,230 (1,710 to 2,800)	983.7 (755.2 to 1,232.1)	2,160 (1,640 to 2,740)	953.4 (723.1 to 1,209.6)	1,930 (1,500 to 2,460)	851.1 (661.1 to 1,081.6)	-43.9 (-50.3 to -36.9)	-3.1 (-11.4 to 5.4)
	15-49 years	5,040 (4,440 to 5,720)	487.8 (430.5 to 553.9)	3,340 (2,920 to 3,800)	299.9 (261.7 to 341.2)	3,210 (2,810 to 3,630)	289.7 (253.7 to 327.5)	2,880 (2,520 to 3,280)	261.4 (228.8 to 298.4)	-38.5 (-42.7 to -33.8)	-3.4 (-9.0 to 3.0)
	50-69 years	5,090 (4,480 to 5,650)	1,477.0 (1,300.8 to 1,639.9)	5,020 (4,480 to 5,560)	903.4 (805.9 to 1,000.6)	4,820 (4,260 to 5,330)	871.2 (769.5 to 963.0)	4,360 (3,880 to 4,860)	780.4 (694.7 to 870.5)	-38.8 (-44.9 to -32.2)	-3.6 (-11.9 to 6.1)
	70+ years	5,080 (4,450 to 5,720)	5,763.2 (5,054.3 to 6,493.7)	5,590 (4,910 to 6,380)	3,087.9 (2,710.7 to 3,524.9)	5,560 (4,880 to 6,290)	2,911.7 (2,556.2 to 3,297.6)	4,740 (4,180 to 5,410)	2,497.5 (2,202.8 to 2,849.5)	-46.4 (-51.8 to -39.7)	-5.7 (-16.1 to 5.5)
**Poland**	All Ages	538,000 (506,000 to 575,000)	1,409.3 (1,326.8 to 1,505.8)	556,000 (532,000 to 583,000)	1,446.8 (1,383.4 to 1,516.4)	521,000 (482,000 to 564,000)	1,357.6 (1,255.7 to 1,469.6)	458,000 (437,000 to 480,000)	1,197.7 (1,141.6 to 1,256.6)	2.7 (-1.5 to 7.1)	-6.2 (-12.1 to 0.9)
	Under 5	108,000 (94,300 to 123,000)	3,685.5 (3,231.7 to 4,208.0)	86,500 (76,400 to 98,300)	4,449.6 (3,932.1 to 5,059.0)	73,300 (60,200 to 88,800)	3,807.7 (3,127.7 to 4,615.7)	49,000 (42,900 to 55,700)	2,603.5 (2,281.6 to 2,958.6)	20.7 (12.7 to 28.2)	-14.4 (-26.1 to -1.4)
	5-14 years	71,100 (54,800 to 91,000)	1,068.6 (822.4 to 1,366.1)	34,000 (28,800 to 40,700)	858.5 (727.3 to 1,026.9)	32,200 (27,100 to 38,200)	806.5 (680.3 to 957.7)	29,200 (24,400 to 35,300)	730.3 (609.4 to 880.5)	-19.7 (-29.4 to -7.8)	-6.1 (-10.4 to -0.6)
	15-49 years	79,000 (70,800 to 87,800)	416.5 (373.4 to 462.9)	60,200 (55,900 to 64,900)	331.0 (307.1 to 356.5)	57,100 (52,300 to 62,200)	316.2 (289.4 to 344.5)	52,400 (48,400 to 56,700)	292.3 (270.3 to 316.4)	-20.5 (-25.1 to -15.7)	-4.5 (-9.4 to 0.9)
	50-69 years	131,000 (121,000 to 142,000)	1,818.6 (1,673.9 to 1,968.9)	141,000 (132,000 to 151,000)	1,421.9 (1,327.1 to 1,517.1)	134,000 (121,000 to 145,000)	1,356.5 (1,231.7 to 1,468.3)	122,000 (114,000 to 130,000)	1,253.5 (1,163.0 to 1,336.4)	-21.8 (-24.3 to -19.0)	-4.6 (-9.1 to 1.0)
	70+ years	149,000 (136,000 to 164,000)	6,186.1 (5,655.2 to 6,818.5)	234,000 (217,000 to 253,000)	5,327.3 (4,946.6 to 5,753.5)	225,000 (204,000 to 248,000)	4,945.9 (4,484.6 to 5,450.2)	205,000 (191,000 to 223,000)	4,389.4 (4,092.1 to 4,765.2)	-13.9 (-17.9 to -10.2)	-7.2 (-12.7 to -0.6)
**Romania**	All Ages	594,000 (552,000 to 642,000)	2,540.1 (2,361.8 to 2,745.5)	313,000 (292,000 to 339,000)	1,625.4 (1,514.4 to 1,760.6)	306,000 (279,000 to 326,000)	1,601.6 (1,460.0 to 1,707.4)	258,000 (243,000 to 276,000)	1,362.1 (1,281.6 to 1,458.9)	-36.0 (-40.1 to -31.8)	-1.5 (-8.9 to 5.6)
	Under 5	181,000 (154,000 to 217,000)	10,215.7 (8,713.9 to 12,272.4)	34,600 (30,700 to 39,400)	3,594.5 (3,184.1 to 4,088.4)	32,500 (27,200 to 37,200)	3,412.8 (2,852.0 to 3,909.8)	22,700 (20,000 to 26,000)	2,424.4 (2,130.5 to 2,775.9)	-64.8 (-70.6 to -58.7)	-5.1 (-18.5 to 5.8)
	5-14 years	84,100 (69,700 to 101,000)	2,214.6 (1,834.5 to 2,652.7)	20,600 (16,400 to 25,200)	972.3 (777.4 to 1,189.9)	20,100 (16,000 to 24,600)	959.7 (763.1 to 1,172.8)	17,500 (14,200 to 21,300)	846.8 (687.2 to 1,029.3)	-56.1 (-62.3 to -49.2)	-1.3 (-10.7 to 8.0)
	15-49 years	89,500 (82,400 to 97,500)	789.7 (726.7 to 860.3)	42,300 (38,300 to 47,200)	489.0 (443.0 to 545.8)	40,700 (36,600 to 44,800)	480.1 (432.2 to 528.6)	35,400 (31,900 to 38,900)	425.4 (382.4 to 466.9)	-38.1 (-43.3 to -31.8)	-1.8 (-10.5 to 7.8)
	50-69 years	137,000 (123,000 to 149,000)	2,714.9 (2,439.0 to 2,951.4)	95,100 (87,100 to 105,000)	1,869.8 (1,711.3 to 2,064.7)	92,700 (83,800 to 101,000)	1,822.5 (1,645.9 to 1,989.8)	80,400 (73,400 to 87,900)	1,586.4 (1,448.9 to 1,735.9)	-31.1 (-37.9 to -24.3)	-2.5 (-10.9 to 6.1)

Location	Age Group	1990		2019		2020		2021		Percent Incidence Rate Change from 1990-2019 (95% UI)	Percent Incidence Rate Change from 2019-2021 (95% UI)
		Episodes (95% UI)	Rate (95% UI)	Episodes (95% UI)	Rate (95% UI)	Episodes (95% UI)	Rate (95% UI)	Episodes (95% UI)	Rate (95% UI)		
	70+ years	102,000<br>(90,200 to 115,000)	7,177.8<br>(6,324.3 to 8,037.4)	121,000<br>(108,000 to 134,000)	4,925.8<br>(4,405.8 to 5,465.1)	120,000<br>(106,000 to 134,000)	4,797.0<br>(4,234.1 to 5,356.7)	102,000<br>(91,100 to 113,000)	4,024.0<br>(3,598.0 to 4,470.2)	-31.4<br>(-37.5 to -24.5)	-2.6<br>(-12.0 to 8.0)
**Serbia**	All Ages	117,000<br>(109,000 to 126,000)	1,213.5<br>(1,128.0 to 1,308.3)	85,300<br>(79,000 to 91,800)	946.0<br>(876.1 to 1,018.8)	82,300<br>(76,300 to 88,700)	916.6<br>(849.9 to 988.0)	71,500<br>(66,300 to 77,200)	801.6<br>(743.4 to 865.9)	-22.0<br>(-27.8 to -15.6)	-3.1<br>(-9.9 to 3.7)
	Under 5	35,100<br>(30,700 to 40,900)	5,161.9<br>(4,504.1 to 6,004.8)	6,940<br>(5,750 to 8,440)	1,824.8<br>(1,511.9 to 2,218.3)	6,270<br>(5,040 to 7,810)	1,674.8<br>(1,345.1 to 2,085.2)	4,550<br>(3,750 to 5,550)	1,233.2<br>(1,017.0 to 1,505.2)	-64.6<br>(-69.5 to -59.8)	-8.2<br>(-17.2 to 1.4)
	5-14 years	16,600<br>(13,000 to 20,800)	1,115.9<br>(875.9 to 1,400.1)	6,860<br>(5,220 to 8,840)	675.5<br>(513.3 to 870.6)	6,430<br>(4,880 to 8,250)	650.5<br>(493.5 to 834.7)	5,550<br>(4,310 to 7,160)	578.8<br>(449.8 to 746.7)	-39.5<br>(-45.6 to -31.9)	-3.7<br>(-11.9 to 4.9)
	15-49 years	16,900<br>(14,900 to 19,200)	355.1<br>(313.3 to 402.9)	10,100<br>(8,990 to 11,300)	236.9<br>(211.1 to 265.0)	9,830<br>(8,690 to 11,100)	231.4<br>(204.4 to 260.2)	8,850<br>(7,880 to 9,910)	208.6<br>(185.9 to 233.7)	-33.3<br>(-37.4 to -28.7)	-2.3<br>(-8.7 to 4.7)
	50-69 years	26,800<br>(23,900 to 29,800)	1,216.0<br>(1,088.1 to 1,352.4)	23,200<br>(21,000 to 25,600)	1,014.0<br>(917.3 to 1,117.7)	22,800<br>(20,600 to 25,200)	996.5<br>(898.4 to 1,098.9)	20,300<br>(18,100 to 22,400)	892.9<br>(797.0 to 987.7)	-16.6<br>(-24.2 to -5.7)	-1.7<br>(-10.7 to 6.7)
	70+ years	21,400<br>(19,100 to 23,900)	4,316.1<br>(3,839.0 to 4,817.3)	38,200<br>(34,100 to 42,600)	3,555.5<br>(3,174.8 to 3,967.4)	36,900<br>(33,300 to 41,500)	3,440.8<br>(3,106.1 to 3,868.0)	32,300<br>(29,200 to 36,200)	2,985.9<br>(2,698.0 to 3,344.7)	-17.6<br>(-27.9 to -7.1)	-3.2<br>(-12.2 to 6.3)
**Slovakia**	All Ages	122,000<br>(114,000 to 131,000)	2,304.4<br>(2,150.2 to 2,478.6)	79,300<br>(74,100 to 85,700)	1,457.8<br>(1,362.4 to 1,576.5)	69,800<br>(64,700 to 75,500)	1,282.5<br>(1,189.4 to 1,387.5)	58,800<br>(54,900 to 63,500)	1,083.7<br>(1,011.4 to 1,169.4)	-36.7<br>(-40.4 to -32.6)	-12.0<br>(-17.5 to -5.7)
	Under 5	24,000<br>(21,000 to 27,500)	5,871.6<br>(5,139.3 to 6,725.8)	7,050<br>(6,100 to 8,240)	2,460.0<br>(2,128.7 to 2,874.9)	5,630<br>(4,730 to 6,700)	1,957.6<br>(1,647.0 to 2,331.8)	4,050<br>(3,470 to 4,740)	1,416.1<br>(1,211.7 to 1,657.9)	-58.1<br>(-62.3 to -53.4)	-20.4<br>(-28.4 to -10.5)
	5-14 years	15,000<br>(12,000 to 18,300)	1,634.0<br>(1,313.4 to 1,992.4)	4,450<br>(3,460 to 5,670)	788.3<br>(612.7 to 1,003.3)	4,030<br>(3,120 to 5,090)	709.8<br>(550.5 to 897.7)	3,520<br>(2,740 to 4,440)	616.9<br>(480.6 to 778.9)	-51.8<br>(-59.3 to -44.6)	-9.9<br>(-16.9 to -1.2)
	15-49 years	17,100<br>(15,400 to 19,200)	640.6<br>(575.1 to 720.0)	9,200<br>(8,270 to 10,100)	349.5<br>(314.4 to 385.4)	8,230<br>(7,330 to 9,180)	315.3<br>(281.0 to 351.6)	7,090<br>(6,370 to 7,860)	274.1<br>(246.2 to 303.7)	-45.4<br>(-50.2 to -39.9)	-9.8<br>(-16.3 to -1.7)
	50-69 years	29,500<br>(26,600 to 32,600)	3,088.9<br>(2,782.6 to 3,408.3)	23,700<br>(21,400 to 26,000)	1,699.8<br>(1,535.3 to 1,865.3)	21,000<br>(19,200 to 23,100)	1,512.8<br>(1,378.5 to 1,661.4)	18,000<br>(16,100 to 19,900)	1,300.2<br>(1,165.2 to 1,439.2)	-45.0<br>(-50.1 to -40.3)	-11.0<br>(-18.5 to -2.9)
	70+ years	36,100<br>(32,600 to 40,700)	10,926.9<br>(9,860.3 to 12,318.5)	34,900<br>(31,800 to 38,900)	6,200.7<br>(5,657.2 to 6,914.5)	30,800<br>(27,900 to 34,300)	5,285.8<br>(4,778.4 to 5,874.5)	26,200<br>(23,900 to 29,300)	4,352.3<br>(3,973.1 to 4,865.9)	-43.3<br>(-48.2 to -38.4)	-14.8<br>(-22.0 to -7.2)
**Slovenia**	All Ages	33,300<br>(31,000 to 36,000)	1,686.4<br>(1,569.2 to 1,823.3)	25,700<br>(23,700 to 27,900)	1,239.2<br>(1,144.8 to 1,344.4)	25,300<br>(23,100 to 27,700)	1,221.6<br>(1,113.4 to 1,336.0)	21,100<br>(19,500 to 22,900)	1,019.7<br>(943.0 to 1,106.0)	-26.5<br>(-30.7 to -21.7)	-1.4<br>(-8.7 to 4.7)
	Under 5	4,860<br>(4,050 to 5,840)	3,953.7<br>(3,296.2 to 4,748.6)	2,260<br>(1,840 to 2,780)	2,228.7<br>(1,809.4 to 2,731.6)	2,090<br>(1,620 to 2,630)	2,095.4<br>(1,626.2 to 2,633.4)	1,330<br>(1,070 to 1,640)	1,361.7<br>(1,095.4 to 1,680.5)	-43.6<br>(-49.4 to -37.6)	-6.0<br>(-19.1 to 3.3)
	5-14 years	3,090<br>(2,360 to 4,030)	1,064.0<br>(810.5 to 1,387.2)	1,700<br>(1,260 to 2,220)	810.2<br>(599.1 to 1,060.8)	1,670<br>(1,260 to 2,180)	784.6<br>(594.1 to 1,023.8)	1,430<br>(1,080 to 1,870)	668.9<br>(503.9 to 870.4)	-23.9<br>(-30.0 to -17.1)	-3.2<br>(-10.1 to 5.0)
	15-49 years	4,060<br>(3,570 to 4,610)	400.0<br>(352.2 to 454.4)	2,070<br>(1,810 to 2,370)	231.3<br>(201.9 to 264.4)	2,010<br>(1,750 to 2,310)	226.8<br>(197.0 to 260.7)	1,730<br>(1,490 to 1,980)	196.8<br>(170.2 to 225.2)	-42.2<br>(-46.2 to -37.5)	-1.9<br>(-9.2 to 5.8)

Location	Age Group	1990		2019		2020		2021		Percent Incidence Rate Change from 1990-2019 (95% UI)	Percent Incidence Rate Change from 2019-2021 (95% UI)
		Episodes (95% UI)	Rate (95% UI)	Episodes (95% UI)	Rate (95% UI)	Episodes (95% UI)	Rate (95% UI)	Episodes (95% UI)	Rate (95% UI)		
	50-69 years	8,520 (7,660 to 9,500)	2,084.9 (1,874.8 to 2,323.3)	4,990 (4,460 to 5,570)	860.5 (769.6 to 960.3)	4,900 (4,360 to 5,480)	842.9 (749.6 to 942.8)	4,240 (3,830 to 4,690)	732.1 (660.4 to 809.2)	-58.7 (-62.2 to -55.1)	-2.0 (-10.4 to 7.3)
	70+ years	12,700 (11,500 to 14,200)	9,309.4 (8,429.3 to 10,385.4)	14,700 (13,300 to 16,300)	5,122.9 (4,632.9 to 5,699.2)	14,700 (13,100 to 16,300)	5,002.4 (4,456.5 to 5,567.1)	12,400 (11,200 to 13,800)	4,115.8 (3,711.8 to 4,581.9)	-45.0 (-50.0 to -39.7)	-2.4 (-11.1 to 5.2)
**Belarus**	All Ages	297,000 (275,000 to 322,000)	2,839.5 (2,637.2 to 3,080.0)	198,000 (185,000 to 212,000)	2,097.2 (1,953.7 to 2,244.0)	181,000 (167,000 to 196,000)	1,928.1 (1,778.9 to 2,090.6)	178,000 (164,000 to 195,000)	1,909.8 (1,756.9 to 2,096.5)	-26.1 (-30.5 to -21.4)	-8.1 (-13.7 to -1.7)
	Under 5	70,000 (57,200 to 83,100)	8,682.4 (7,097.3 to 10,309.9)	17,400 (14,500 to 21,000)	3,307.7 (2,759.7 to 3,995.0)	13,800 (11,200 to 16,700)	2,779.8 (2,256.7 to 3,361.5)	13,300 (10,700 to 16,500)	2,849.0 (2,286.9 to 3,528.7)	-61.9 (-67.9 to -54.6)	-16.0 (-23.4 to -7.2)
	5-14 years	39,300 (30,200 to 50,900)	2,460.5 (1,886.8 to 3,182.3)	18,000 (13,600 to 23,800)	1,684.3 (1,271.8 to 2,222.9)	17,200 (12,900 to 22,900)	1,570.6 (1,178.6 to 2,088.7)	17,400 (12,900 to 22,900)	1,562.3 (1,161.7 to 2,063.9)	-31.5 (-38.5 to -23.8)	-6.7 (-13.0 to 1.1)
	15-49 years	48,500 (43,100 to 54,100)	962.6 (855.2 to 1,073.2)	36,100 (32,300 to 40,100)	828.9 (743.0 to 921.6)	33,400 (30,100 to 36,600)	776.2 (700.4 to 849.7)	32,800 (29,500 to 36,400)	771.7 (693.7 to 855.7)	-13.9 (-20.4 to -5.8)	-6.4 (-14.4 to 2.7)
	50-69 years	74,500 (67,600 to 81,300)	3,234.4 (2,934.8 to 3,529.5)	72,200 (64,500 to 79,800)	2,811.9 (2,512.3 to 3,109.1)	66,900 (59,900 to 73,300)	2,620.9 (2,347.4 to 2,873.0)	65,500 (58,500 to 73,800)	2,580.6 (2,304.5 to 2,908.3)	-13.1 (-21.3 to -4.1)	-6.8 (-15.6 to 3.2)
	70+ years	64,300 (58,200 to 71,200)	9,239.5 (8,360.0 to 10,228.4)	54,600 (49,500 to 60,800)	5,826.7 (5,288.1 to 6,495.4)	49,900 (44,200 to 56,500)	5,244.4 (4,643.9 to 5,936.5)	49,100 (42,500 to 55,900)	5,145.5 (4,450.8 to 5,855.9)	-36.9 (-43.2 to -29.5)	-10.0 (-18.4 to -1.7)
**Estonia**	All Ages	47,100 (43,400 to 50,700)	3,003.0 (2,770.1 to 3,233.5)	31,400 (29,100 to 33,600)	2,385.3 (2,216.3 to 2,554.8)	27,600 (25,600 to 29,600)	2,101.7 (1,949.4 to 2,252.6)	26,300 (24,300 to 28,200)	2,003.4 (1,855.2 to 2,154.8)	-20.6 (-25.6 to -15.4)	-11.9 (-17.6 to -6.2)
	Under 5	7,870 (6,580 to 9,380)	6,524.6 (5,460.2 to 7,772.9)	2,130 (1,730 to 2,550)	3,014.1 (2,458.1 to 3,617.0)	1,590 (1,320 to 1,940)	2,267.7 (1,881.3 to 2,764.7)	1,420 (1,170 to 1,720)	2,047.0 (1,696.8 to 2,478.5)	-53.8 (-59.9 to -47.8)	-24.8 (-31.0 to -17.2)
	5-14 years	4,830 (3,680 to 6,180)	2,114.7 (1,608.4 to 2,706.0)	2,080 (1,550 to 2,730)	1,424.4 (1,063.0 to 1,867.1)	1,870 (1,430 to 2,430)	1,274.2 (973.3 to 1,659.5)	1,780 (1,380 to 2,340)	1,214.1 (941.7 to 1,591.5)	-32.6 (-39.2 to -25.2)	-10.5 (-17.8 to -2.4)
	15-49 years	8,470 (7,660 to 9,430)	1,115.1 (1,008.2 to 1,241.7)	4,390 (3,880 to 4,920)	753.5 (667.0 to 846.0)	3,900 (3,520 to 4,330)	674.7 (608.6 to 749.8)	3,720 (3,320 to 4,120)	647.6 (578.1 to 716.9)	-32.4 (-37.6 to -26.3)	-10.5 (-17.4 to -2.9)
	50-69 years	15,200 (13,500 to 16,800)	4,450.0 (3,943.2 to 4,912.2)	9,760 (8,750 to 10,900)	2,950.8 (2,645.0 to 3,304.3)	8,770 (7,890 to 9,700)	2,660.7 (2,393.5 to 2,942.2)	8,380 (7,580 to 9,330)	2,543.8 (2,300.4 to 2,832.1)	-33.7 (-39.9 to -26.1)	-9.8 (-17.5 to -1.0)
	70+ years	10,700 (9,450 to 12,300)	9,053.2 (7,961.8 to 10,354.3)	13,000 (11,600 to 14,700)	7,020.6 (6,259.8 to 7,924.9)	11,500 (10,200 to 13,100)	6,067.4 (5,413.5 to 6,918.3)	11,000 (9,710 to 12,600)	5,731.3 (5,074.7 to 6,559.4)	-22.5 (-31.6 to -13.7)	-13.6 (-21.3 to -4.6)
**Latvia**	All Ages	74,200 (68,800 to 79,800)	2,789.6 (2,588.2 to 3,001.5)	46,500 (43,400 to 50,300)	2,431.3 (2,269.3 to 2,629.3)	39,400 (36,600 to 42,800)	2,083.9 (1,933.4 to 2,259.0)	37,100 (34,600 to 39,900)	1,985.4 (1,849.8 to 2,134.0)	-12.8 (-19.0 to -6.3)	-14.3 (-20.2 to -7.7)
	Under 5	11,600 (9,520 to 13,900)	5,701.0 (4,697.4 to 6,881.0)	3,000 (2,440 to 3,650)	2,928.3 (2,380.1 to 3,565.5)	2,020 (1,620 to 2,490)	2,055.3 (1,642.2 to 2,534.8)	1,730 (1,400 to 2,110)	1,844.8 (1,491.4 to 2,250.0)	-48.6 (-55.7 to -41.8)	-29.8 (-37.5 to -19.5)
	5-14 years	6,930 (5,240 to 9,070)	1,892.2 (1,431.9 to 2,476.2)	2,650 (2,000 to 3,400)	1,328.4 (1,002.9 to 1,701.5)	2,340 (1,760 to 3,070)	1,160.6 (869.8 to 1,518.2)	2,270 (1,720 to 2,930)	1,116.3 (846.5 to 1,439.7)	-29.8 (-35.8 to -22.6)	-12.6 (-19.3 to -4.7)

Location	Age Group	1990		2019		2020		2021		Percent Incidence Rate Change from 1990-2019 (95% UI)	Percent Incidence Rate Change from 2019-2021 (95% UI)
		Episodes (95% UI)	Rate (95% UI)	Episodes (95% UI)	Rate (95% UI)	Episodes (95% UI)	Rate (95% UI)	Episodes (95% UI)	Rate (95% UI)		
	15-49 years	13,700<br>(12,500 to 15,300)	1,062.8<br>(970.9 to 1,189.5)	6,800<br>(6,110 to 7,560)	830.8<br>(745.9 to 923.1)	5,810<br>(5,200 to 6,470)	723.3<br>(647.8 to 805.3)	5,460<br>(4,910 to 6,030)	692.4<br>(622.5 to 764.7)	-21.8<br>(-27.8 to -14.7)	-12.9<br>(-20.1 to -6.2)
	50-69 years	23,600<br>(21,600 to 25,900)	3,965.6<br>(3,619.7 to 4,342.9)	15,600<br>(13,800 to 17,500)	3,064.3<br>(2,714.2 to 3,432.2)	13,800<br>(12,400 to 15,300)	2,718.4<br>(2,439.3 to 3,013.6)	13,100<br>(11,700 to 14,700)	2,596.3<br>(2,327.3 to 2,912.4)	-22.7<br>(-29.7 to -13.9)	-11.3<br>(-20.0 to -1.6)
	70+ years	18,400<br>(16,100 to 20,700)	8,848.6<br>(7,753.5 to 9,992.1)	18,400<br>(16,500 to 20,900)	6,558.2<br>(5,886.0 to 7,439.6)	15,500<br>(13,700 to 17,700)	5,480.1<br>(4,835.9 to 6,242.5)	14,600<br>(13,000 to 16,400)	5,187.4<br>(4,602.1 to 5,827.1)	-25.9<br>(-33.7 to -15.4)	-16.4<br>(-24.3 to -6.6)
**Lithuania**	All Ages	92,400<br>(84,700 to 101,000)	2,515.5<br>(2,306.3 to 2,738.2)	70,300<br>(65,700 to 75,600)	2,514.7<br>(2,351.1 to 2,706.8)	65,600<br>(59,900 to 71,600)	2,374.4<br>(2,166.6 to 2,590.7)	64,200<br>(58,800 to 70,700)	2,352.3<br>(2,154.7 to 2,591.5)	0.0<br>(-6.8 to 8.0)	-5.6<br>(-13.0 to 3.7)
	Under 5	16,600<br>(13,800 to 19,800)	5,750.1<br>(4,777.2 to 6,836.4)	4,380<br>(3,630 to 5,190)	3,068.4<br>(2,544.0 to 3,640.6)	3,730<br>(2,940 to 4,670)	2,714.7<br>(2,134.7 to 3,393.0)	3,380<br>(2,640 to 4,290)	2,565.3<br>(2,008.3 to 3,258.2)	-46.6<br>(-52.2 to -40.1)	-11.5<br>(-24.9 to 4.2)
	5-14 years	11,300<br>(8,580 to 14,800)	2,078.7<br>(1,583.6 to 2,730.5)	3,960<br>(3,030 to 5,170)	1,450.2<br>(1,111.8 to 1,895.4)	3,770<br>(2,840 to 4,900)	1,373.7<br>(1,033.7 to 1,784.8)	3,710<br>(2,840 to 4,790)	1,342.0<br>(1,028.4 to 1,735.7)	-30.2<br>(-35.7 to -20.9)	-5.3<br>(-14.1 to 4.6)
	15-49 years	16,100<br>(14,300 to 18,000)	879.0<br>(779.2 to 983.6)	9,410<br>(8,560 to 10,400)	776.6<br>(706.6 to 860.0)	8,700<br>(7,870 to 9,690)	735.7<br>(664.9 to 818.7)	8,340<br>(7,510 to 9,310)	722.0<br>(650.0 to 806.0)	-11.7<br>(-18.8 to -3.2)	-5.3<br>(-13.1 to 4.4)
	50-69 years	24,200<br>(21,700 to 26,500)	3,183.6<br>(2,864.9 to 3,495.8)	22,800<br>(20,600 to 25,200)	2,979.8<br>(2,687.2 to 3,290.2)	21,700<br>(19,200 to 24,200)	2,837.3<br>(2,513.4 to 3,166.5)	21,400<br>(19,200 to 23,500)	2,802.0<br>(2,511.5 to 3,077.1)	-6.4<br>(-14.9 to 3.7)	-4.8<br>(-13.7 to 6.9)
	70+ years	24,300<br>(21,600 to 27,500)	9,570.3<br>(8,512.3 to 10,851.1)	29,700<br>(26,800 to 33,600)	7,414.6<br>(6,699.3 to 8,393.9)	27,700<br>(24,500 to 31,400)	6,877.3<br>(6,095.5 to 7,788.1)	27,400<br>(24,100 to 31,000)	6,812.1<br>(6,002.2 to 7,709.0)	-22.5<br>(-29.4 to -14.1)	-7.2<br>(-17.3 to 5.0)
**Republic of Moldova**	All Ages	156,000<br>(145,000 to 171,000)	3,516.9<br>(3,271.2 to 3,840.6)	94,500<br>(88,300 to 100,000)	2,588.7<br>(2,420.0 to 2,752.5)	92,300<br>(85,400 to 98,900)	2,546.8<br>(2,356.9 to 2,729.2)	80,100<br>(74,700 to 85,400)	2,228.6<br>(2,077.5 to 2,376.6)	-26.4<br>(-31.6 to -20.5)	-1.6<br>(-8.5 to 4.6)
	Under 5	47,300<br>(40,100 to 55,000)	10,987.0<br>(9,302.6 to 12,775.5)	7,610<br>(6,420 to 8,900)	4,549.0<br>(3,834.7 to 5,322.2)	7,030<br>(5,730 to 8,420)	4,372.2<br>(3,562.0 to 5,235.0)	5,070<br>(4,240 to 6,050)	3,284.6<br>(2,747.7 to 3,922.5)	-58.6<br>(-62.9 to -53.8)	-3.9<br>(-17.0 to 9.3)
	5-14 years	22,000<br>(17,600 to 27,000)	2,727.5<br>(2,190.0 to 3,350.4)	5,560<br>(4,470 to 6,910)	1,492.9<br>(1,199.4 to 1,855.1)	5,450<br>(4,280 to 6,850)	1,468.8<br>(1,152.5 to 1,845.0)	4,830<br>(3,840 to 6,030)	1,311.8<br>(1,042.0 to 1,638.5)	-45.3<br>(-50.8 to -38.4)	-1.6<br>(-11.1 to 8.4)
	15-49 years	27,700<br>(25,400 to 30,300)	1,261.4<br>(1,154.6 to 1,380.5)	20,100<br>(18,200 to 22,100)	1,100.9<br>(993.7 to 1,205.9)	19,800<br>(17,900 to 21,800)	1,094.6<br>(987.3 to 1,204.1)	17,500<br>(15,800 to 19,200)	977.4<br>(882.1 to 1,075.1)	-12.7<br>(-20.9 to -5.4)	-0.6<br>(-9.6 to 9.5)
	50-69 years	41,500<br>(38,000 to 45,600)	5,156.4<br>(4,726.6 to 5,669.5)	39,000<br>(35,200 to 43,500)	4,079.1<br>(3,689.0 to 4,555.1)	37,900<br>(33,900 to 41,300)	4,018.9<br>(3,586.4 to 4,374.2)	33,200<br>(29,700 to 36,400)	3,561.6<br>(3,179.8 to 3,906.4)	-20.9<br>(-28.3 to -12.4)	-1.5<br>(-11.5 to 8.5)
	70+ years	17,900<br>(15,700 to 20,700)	8,534.7<br>(7,455.2 to 9,836.9)	22,200<br>(19,700 to 25,300)	6,839.9<br>(6,088.4 to 7,803.7)	22,100<br>(19,400 to 25,000)	6,524.1<br>(5,739.0 to 7,381.1)	19,500<br>(17,200 to 21,800)	5,587.9<br>(4,934.9 to 6,269.2)	-19.9<br>(-29.3 to -10.4)	-4.6<br>(-14.1 to 6.0)
**Russian Federation**	All Ages	3,950,000<br>(3,680,000 to 4,220,000)	2,618.0<br>(2,440.4 to 2,795.2)	3,130,000<br>(2,960,000 to 3,310,000)	2,146.3<br>(2,025.1 to 2,267.2)	3,130,000<br>(2,960,000 to 3,330,000)	2,151.4<br>(2,029.9 to 2,290.6)	2,800,000<br>(2,630,000 to 2,970,000)	1,930.9<br>(1,816.1 to 2,049.8)	-18.0<br>(-21.4 to -14.5)	0.2<br>(-2.8 to 3.4)
	Under 5	893,000<br>(762,000 to 1,040,000)	7,681.8<br>(6,558.7 to 8,922.2)	156,000<br>(131,000 to 184,000)	1,828.2<br>(1,539.0 to 2,153.7)	142,000<br>(117,000 to 167,000)	1,759.7<br>(1,449.5 to 2,076.1)	107,000<br>(89,000 to 127,000)	1,411.3<br>(1,169.2 to 1,672.2)	-76.2<br>(-78.9 to -73.2)	-3.7<br>(-10.0 to 2.1)

Location	Age Group	1990		2019		2020		2021		Percent Incidence Rate Change from 1990-2019 (95% UI)	Percent Incidence Rate Change from 2019-2021 (95% UI)
		Episodes (95% UI)	Rate (95% UI)	Episodes (95% UI)	Rate (95% UI)	Episodes (95% UI)	Rate (95% UI)	Episodes (95% UI)	Rate (95% UI)		
	5-14 years	484,000 (382,000 to 616,000)	2,098.2 (1,657.4 to 2,669.1)	209,000 (164,000 to 265,000)	1,176.8 (925.5 to 1,493.6)	212,000 (168,000 to 266,000)	1,165.6 (925.4 to 1,461.5)	196,000 (154,000 to 249,000)	1,063.2 (832.6 to 1,350.6)	-43.9 (-46.7 to -41.0)	-1.0 (-3.6 to 1.7)
	15-49 years	661,000 (604,000 to 725,000)	890.8 (813.6 to 976.2)	713,000 (657,000 to 772,000)	1,042.0 (959.4 to 1,127.8)	711,000 (655,000 to 775,000)	1,046.3 (964.2 to 1,140.0)	647,000 (596,000 to 702,000)	959.4 (883.3 to 1,041.3)	17.0 (11.3 to 22.9)	0.4 (-2.5 to 3.7)
	50-69 years	1,180,000 (1,090,000 to 1,290,000)	3,655.3 (3,358.8 to 3,984.7)	1,140,000 (1,050,000 to 1,240,000)	3,044.2 (2,789.2 to 3,309.1)	1,140,000 (1,050,000 to 1,240,000)	3,057.4 (2,826.4 to 3,337.3)	1,030,000 (942,000 to 1,120,000)	2,771.4 (2,545.0 to 3,021.8)	-16.7 (-18.9 to -14.0)	0.4 (-2.3 to 3.6)
	70+ years	730,000 (661,000 to 817,000)	7,559.0 (6,844.8 to 8,457.4)	910,000 (830,000 to 1,020,000)	6,687.2 (6,098.7 to 7,482.9)	927,000 (837,000 to 1,020,000)	6,579.5 (5,939.4 to 7,264.7)	820,000 (747,000 to 913,000)	5,731.5 (5,220.1 to 6,379.0)	-11.5 (-14.9 to -7.4)	-1.6 (-5.3 to 2.0)
**Ukraine**	All Ages	1,280,000 (1,190,000 to 1,380,000)	2,426.6 (2,248.9 to 2,608.6)	901,000 (839,000 to 972,000)	2,053.8 (1,911.5 to 2,215.3)	891,000 (832,000 to 952,000)	2,048.1 (1,911.9 to 2,187.5)	789,000 (740,000 to 849,000)	1,832.1 (1,718.1 to 1,970.7)	-15.4 (-21.5 to -10.1)	-0.3 (-5.9 to 6.2)
	Under 5	222,000 (186,000 to 265,000)	5,897.6 (4,923.4 to 7,043.5)	44,300 (36,500 to 52,500)	2,407.3 (1,987.0 to 2,855.7)	40,700 (32,800 to 50,000)	2,386.3 (1,922.6 to 2,927.5)	29,600 (24,200 to 35,300)	1,856.6 (1,519.2 to 2,216.5)	-59.2 (-64.7 to -52.4)	-0.9 (-11.2 to 10.8)
	5-14 years	156,000 (124,000 to 197,000)	2,054.5 (1,628.7 to 2,591.1)	58,900 (45,800 to 74,800)	1,221.8 (950.6 to 1,550.1)	58,500 (45,800 to 74,600)	1,215.9 (950.5 to 1,549.8)	51,500 (40,100 to 65,100)	1,083.1 (843.7 to 1,369.2)	-40.5 (-46.2 to -35.4)	-0.5 (-7.1 to 7.7)
	15-49 years	226,000 (206,000 to 248,000)	904.9 (825.0 to 994.6)	235,000 (212,000 to 257,000)	1,132.4 (1,024.1 to 1,239.2)	230,000 (209,000 to 253,000)	1,125.3 (1,021.8 to 1,238.6)	206,000 (187,000 to 228,000)	1,019.6 (923.9 to 1,125.8)	25.1 (13.9 to 38.9)	-0.6 (-9.9 to 10.9)
	50-69 years	375,000 (340,000 to 409,000)	3,027.0 (2,747.7 to 3,301.5)	323,000 (286,000 to 361,000)	2,749.3 (2,434.4 to 3,072.3)	323,000 (291,000 to 357,000)	2,764.1 (2,485.6 to 3,056.5)	290,000 (264,000 to 326,000)	2,493.1 (2,267.3 to 2,806.9)	-9.2 (-18.6 to 0.4)	0.5 (-7.7 to 11.9)
	70+ years	300,000 (269,000 to 335,000)	7,486.9 (6,717.6 to 8,353.9)	240,000 (216,000 to 273,000)	5,059.9 (4,556.1 to 5,743.7)	238,000 (213,000 to 269,000)	4,938.6 (4,424.1 to 5,571.5)	212,000 (192,000 to 237,000)	4,347.7 (3,929.7 to 4,869.4)	-32.4 (-39.1 to -26.8)	-2.4 (-10.4 to 6.4)
**Australia**	All Ages	157,000 (145,000 to 170,000)	932.7 (858.2 to 1,010.5)	221,000 (208,000 to 236,000)	884.7 (832.0 to 944.6)	183,000 (170,000 to 196,000)	719.9 (672.0 to 773.0)	182,000 (168,000 to 194,000)	705.2 (650.6 to 753.6)	-5.1 (-11.8 to 1.4)	-18.6 (-23.0 to -13.0)
	Under 5	16,700 (14,300 to 19,400)	1,323.3 (1,129.5 to 1,536.9)	11,400 (9,470 to 13,700)	752.2 (623.4 to 900.0)	6,980 (5,790 to 8,430)	461.5 (382.8 to 557.0)	6,570 (5,450 to 7,920)	437.2 (362.4 to 526.5)	-43.2 (-48.0 to -38.7)	-38.6 (-43.0 to -34.2)
	5-14 years	7,220 (5,420 to 9,330)	286.2 (214.7 to 369.6)	7,100 (5,350 to 9,380)	225.7 (170.1 to 298.2)	5,960 (4,550 to 7,760)	186.3 (142.1 to 242.6)	5,890 (4,440 to 7,670)	181.6 (136.9 to 236.3)	-21.1 (-26.0 to -14.8)	-17.4 (-22.7 to -11.0)
	15-49 years	26,000 (22,500 to 29,900)	289.7 (250.3 to 332.6)	26,100 (22,600 to 29,900)	222.6 (193.2 to 254.9)	21,900 (18,700 to 25,500)	184.3 (157.3 to 214.7)	21,500 (18,400 to 25,000)	179.4 (153.3 to 208.1)	-23.2 (-27.9 to -18.4)	-17.2 (-22.0 to -11.8)
	50-69 years	42,300 (37,600 to 47,400)	1,470.1 (1,306.6 to 1,647.8)	52,300 (46,300 to 58,100)	913.0 (808.0 to 1,014.6)	44,700 (39,500 to 49,500)	766.3 (676.9 to 849.5)	44,300 (39,600 to 48,700)	744.6 (664.9 to 817.5)	-37.9 (-44.4 to -31.6)	-16.1 (-23.7 to -7.6)
	70+ years	65,000 (57,700 to 71,900)	5,371.3 (4,770.9 to 5,945.4)	124,000 (113,000 to 137,000)	4,361.0 (3,996.2 to 4,819.1)	103,000 (93,900 to 114,000)	3,485.1 (3,172.4 to 3,841.1)	104,000 (92,200 to 114,000)	3,358.3 (2,988.2 to 3,685.2)	-18.8 (-27.0 to -9.5)	-20.1 (-26.1 to -13.9)
**New Zealand**	All Ages	53,100 (49,500 to 57,300)	1,552.4 (1,447.7 to 1,677.5)	55,600 (51,900 to 59,600)	1,115.2 (1,040.3 to 1,195.6)	46,100 (42,700 to 49,900)	905.3 (838.3 to 979.9)	46,200 (43,100 to 49,600)	893.3 (834.1 to 959.6)	-28.2 (-32.1 to -23.7)	-18.8 (-22.3 to -15.3)

Location	Age Group	1990		2019		2020		2021		Percent Incidence Rate Change from 1990-2019 (95% UI)	Percent Incidence Rate Change from 2019-2021 (95% UI)
		Episodes (95% UI)	Rate (95% UI)	Episodes (95% UI)	Rate (95% UI)	Episodes (95% UI)	Rate (95% UI)	Episodes (95% UI)	Rate (95% UI)		
	Under 5	6,630 (5,770 to 7,540)	2,370.1 (2,061.8 to 2,696.0)	3,700 (3,160 to 4,340)	1,199.8 (1,025.0 to 1,408.6)	2,330 (1,990 to 2,760)	747.8 (636.8 to 885.5)	2,240 (1,910 to 2,630)	715.2 (609.9 to 842.1)	-49.4 (-53.4 to -44.5)	-37.7 (-41.0 to -34.2)
	5-14 years	2,490 (1,840 to 3,240)	477.9 (352.5 to 623.4)	2,640 (2,060 to 3,360)	405.9 (316.1 to 516.4)	2,250 (1,750 to 2,850)	339.7 (264.3 to 430.3)	2,230 (1,730 to 2,860)	333.2 (258.1 to 427.2)	-15.1 (-20.3 to -8.8)	-16.3 (-20.8 to -12.3)
	15-49 years	6,840 (5,910 to 7,840)	379.0 (327.5 to 434.4)	7,300 (6,310 to 8,330)	310.8 (268.7 to 354.5)	6,230 (5,370 to 7,070)	259.5 (223.5 to 294.4)	6,180 (5,350 to 7,000)	253.9 (220.1 to 287.7)	-18.0 (-22.8 to -12.4)	-16.5 (-20.6 to -12.0)
	50-69 years	12,500 (11,200 to 13,700)	2,214.4 (1,983.8 to 2,430.7)	12,500 (11,200 to 14,000)	1,080.4 (963.0 to 1,204.1)	10,800 (9,520 to 12,000)	914.5 (808.4 to 1,019.4)	10,700 (9,680 to 12,000)	898.6 (809.9 to 1,000.0)	-51.2 (-55.8 to -46.0)	-15.4 (-20.5 to -8.9)
	70+ years	24,600 (21,800 to 27,500)	9,934.4 (8,815.5 to 11,118.3)	29,400 (26,500 to 33,100)	5,671.3 (5,110.0 to 6,374.0)	24,500 (21,800 to 27,300)	4,545.3 (4,045.7 to 5,062.4)	24,800 (22,500 to 27,300)	4,431.8 (4,014.3 to 4,874.8)	-42.9 (-47.7 to -37.7)	-19.9 (-24.8 to -14.7)
**Brunei Darussalam**	All Ages	3,690 (3,360 to 4,060)	1,423.1 (1,295.1 to 1,567.7)	4,040 (3,770 to 4,350)	914.5 (853.4 to 983.8)	3,670 (3,390 to 3,950)	821.3 (759.9 to 885.2)	3,390 (3,170 to 3,660)	752.2 (701.8 to 810.1)	-35.7 (-39.2 to -32.2)	-10.2 (-14.4 to -5.8)
	Under 5	1,710 (1,470 to 1,980)	4,950.2 (4,266.5 to 5,751.6)	1,120 (980 to 1,300)	3,620.6 (3,157.1 to 4,200.9)	920 (787 to 1,070)	2,970.7 (2,543.5 to 3,454.9)	766 (667 to 885)	2,479.8 (2,158.7 to 2,866.0)	-26.9 (-32.3 to -21.0)	-18.0 (-24.7 to -11.2)
	5-14 years	788 (611 to 972)	1,404.8 (1,088.9 to 1,733.6)	688 (546 to 841)	1,070.6 (849.8 to 1,308.5)	635 (500 to 770)	993.1 (781.9 to 1,205.4)	594 (472 to 717)	932.2 (741.5 to 1,124.9)	-23.8 (-30.6 to -16.2)	-7.2 (-15.0 to 1.1)
	15-49 years	774 (683 to 875)	528.5 (466.0 to 597.5)	1,160 (1,040 to 1,300)	434.0 (387.2 to 485.2)	1,080 (949 to 1,210)	401.5 (351.5 to 448.9)	1,020 (900 to 1,140)	374.0 (331.3 to 421.1)	-17.9 (-24.0 to -10.4)	-7.5 (-14.6 to -0.4)
	50-69 years	185 (163 to 209)	1,040.4 (916.0 to 1,175.2)	573 (511 to 632)	863.0 (769.0 to 951.7)	552 (490 to 611)	800.9 (711.7 to 887.3)	545 (485 to 609)	763.5 (679.0 to 853.1)	-17.1 (-26.4 to -6.0)	-7.2 (-15.4 to 1.1)
	70+ years	235 (208 to 262)	5,284.5 (4,675.9 to 5,876.6)	492 (446 to 546)	4,115.5 (3,734.1 to 4,571.6)	477 (433 to 536)	3,733.7 (3,389.1 to 4,194.1)	473 (429 to 527)	3,467.5 (3,141.7 to 3,864.3)	-22.1 (-30.4 to -13.3)	-9.3 (-16.8 to -1.2)
**Japan**	All Ages	1,660,000 (1,570,000 to 1,770,000)	1,322.1 (1,245.9 to 1,408.5)	1,570,000 (1,490,000 to 1,660,000)	1,223.6 (1,158.7 to 1,296.0)	1,430,000 (1,330,000 to 1,540,000)	1,118.5 (1,041.6 to 1,202.4)	1,330,000 (1,260,000 to 1,410,000)	1,038.0 (983.0 to 1,104.3)	-7.5 (-11.3 to -3.6)	-8.6 (-12.1 to -4.0)
	Under 5	262,000 (223,000 to 301,000)	3,931.3 (3,353.9 to 4,520.5)	123,000 (104,000 to 145,000)	2,536.8 (2,139.3 to 2,995.1)	94,900 (78,400 to 115,000)	2,010.2 (1,662.3 to 2,444.9)	73,000 (61,300 to 86,500)	1,591.3 (1,337.1 to 1,886.5)	-35.5 (-40.2 to -31.0)	-20.8 (-27.5 to -12.1)
	5-14 years	159,000 (121,000 to 207,000)	968.0 (735.4 to 1,262.0)	79,600 (62,300 to 99,200)	724.6 (567.7 to 903.3)	72,600 (56,600 to 91,500)	664.0 (517.4 to 837.2)	66,900 (52,600 to 83,200)	616.2 (484.3 to 765.9)	-25.1 (-30.6 to -19.2)	-8.4 (-11.3 to -5.0)
	15-49 years	273,000 (236,000 to 310,000)	420.0 (364.2 to 477.4)	127,000 (112,000 to 143,000)	243.4 (214.1 to 274.5)	115,000 (101,000 to 130,000)	223.6 (196.8 to 252.0)	105,000 (92,600 to 120,000)	207.8 (182.7 to 235.9)	-42.0 (-44.2 to -40.1)	-8.2 (-11.1 to -4.4)
	50-69 years	341,000 (312,000 to 372,000)	1,220.8 (1,118.1 to 1,330.1)	193,000 (177,000 to 209,000)	588.7 (537.6 to 635.0)	174,000 (159,000 to 189,000)	533.1 (485.8 to 580.1)	160,000 (146,000 to 172,000)	488.6 (447.2 to 527.6)	-51.8 (-53.7 to -49.9)	-9.5 (-12.3 to -6.1)
	70+ years	629,000 (581,000 to 685,000)	6,367.6 (5,886.8 to 6,939.7)	1,050,000 (975,000 to 1,130,000)	3,807.1 (3,542.3 to 4,113.9)	976,000 (900,000 to 1,060,000)	3,448.5 (3,179.0 to 3,752.4)	921,000 (853,000 to 994,000)	3,185.6 (2,952.3 to 3,437.8)	-40.2 (-42.8 to -37.7)	-9.4 (-12.9 to -5.0)

Location	Age Group	1990		2019		2020		2021		Percent Incidence Rate Change from 1990-2019 (95% UI)	Percent Incidence Rate Change from 2019-2021 (95% UI)
		Episodes (95% UI)	Rate (95% UI)	Episodes (95% UI)	Rate (95% UI)	Episodes (95% UI)	Rate (95% UI)	Episodes (95% UI)	Rate (95% UI)		
**Republic of Korea**	All Ages	532,000<br>(485,000 to 585,000)	1,203.1<br>(1,096.1 to 1,323.1)	408,000<br>(382,000 to 436,000)	787.0<br>(737.1 to 840.3)	381,000<br>(356,000 to 407,000)	737.4<br>(687.5 to 786.8)	358,000<br>(334,000 to 382,000)	693.6<br>(646.7 to 741.7)	-34.6<br>(-40.0 to -28.5)	-6.3<br>(-10.6 to -1.6)
	Under 5	197,000<br>(172,000 to 228,000)	5,950.2<br>(5,174.8 to 6,877.5)	41,500<br>(34,200 to 50,600)	2,277.8<br>(1,879.7 to 2,778.3)	31,500<br>(25,600 to 39,100)	1,876.9<br>(1,526.4 to 2,330.5)	22,900<br>(18,700 to 27,900)	1,480.4<br>(1,208.1 to 1,798.5)	-61.7<br>(-67.4 to -55.6)	-17.6<br>(-23.8 to -9.7)
	5-14 years	129,000<br>(103,000 to 160,000)	1,604.2<br>(1,284.4 to 1,988.1)	38,500<br>(29,500 to 49,300)	835.3<br>(640.1 to 1,069.6)	35,600<br>(27,500 to 45,500)	775.8<br>(600.7 to 993.9)	32,500<br>(25,000 to 41,500)	718.4<br>(552.3 to 916.6)	-47.9<br>(-55.4 to -39.1)	-7.1<br>(-13.8 to -0.6)
	15-49 years	119,000<br>(103,000 to 136,000)	460.7<br>(399.3 to 526.8)	68,700<br>(59,100 to 79,900)	270.8<br>(232.9 to 314.6)	62,600<br>(53,800 to 72,300)	252.0<br>(216.7 to 290.9)	56,900<br>(49,000 to 66,000)	234.4<br>(201.8 to 271.6)	-41.2<br>(-46.0 to -35.6)	-7.0<br>(-13.3 to -1.5)
	50-69 years	45,400<br>(40,500 to 50,200)	789.6<br>(704.4 to 872.8)	79,800<br>(71,400 to 87,200)	537.5<br>(481.5 to 587.6)	78,100<br>(69,700 to 86,000)	512.6<br>(457.7 to 564.5)	77,200<br>(69,900 to 84,800)	492.4<br>(446.2 to 541.5)	-31.9<br>(-37.5 to -25.1)	-4.6<br>(-12.5 to 3.9)
	70+ years	41,200<br>(37,000 to 46,000)	3,242.8<br>(2,911.2 to 3,622.1)	180,000<br>(163,000 to 199,000)	3,450.8<br>(3,139.7 to 3,822.1)	174,000<br>(160,000 to 190,000)	3,220.7<br>(2,962.8 to 3,519.8)	168,000<br>(154,000 to 185,000)	3,035.4<br>(2,773.5 to 3,336.6)	6.4<br>(-2.2 to 16.1)	-6.7<br>(-13.3 to 0.3)
**Singapore**	All Ages	50,500<br>(47,900 to 53,400)	1,657.9<br>(1,571.0 to 1,751.2)	81,400<br>(77,100 to 86,200)	1,435.9<br>(1,360.2 to 1,520.6)	73,000<br>(69,100 to 77,100)	1,281.7<br>(1,213.3 to 1,353.4)	68,600<br>(64,400 to 73,600)	1,198.3<br>(1,124.0 to 1,285.4)	-13.4<br>(-17.9 to -8.9)	-10.7<br>(-15.5 to -5.6)
	Under 5	14,100<br>(12,500 to 15,700)	6,886.3<br>(6,131.9 to 7,677.8)	10,800<br>(9,100 to 12,600)	3,666.9<br>(3,083.5 to 4,277.8)	8,020<br>(6,780 to 9,650)	2,756.1<br>(2,331.8 to 3,315.8)	6,360<br>(5,420 to 7,540)	2,224.0<br>(1,894.6 to 2,638.4)	-46.8<br>(-53.1 to -40.2)	-24.8<br>(-31.8 to -16.3)
	5-14 years	5,910<br>(4,880 to 7,160)	1,327.8<br>(1,095.6 to 1,608.0)	5,400<br>(4,340 to 6,580)	1,095.7<br>(880.6 to 1,336.3)	5,200<br>(4,140 to 6,480)	1,024.4<br>(815.2 to 1,276.2)	5,080<br>(3,990 to 6,300)	966.1<br>(757.7 to 1,197.7)	-17.5<br>(-26.1 to -7.6)	-6.5<br>(-14.3 to 0.8)
	15-49 years	13,200<br>(12,000 to 14,400)	692.6<br>(629.7 to 759.1)	12,000<br>(10,700 to 13,400)	392.6<br>(350.2 to 437.6)	11,100<br>(10,000 to 12,300)	366.4<br>(329.9 to 404.9)	10,500<br>(9,250 to 11,700)	351.5<br>(310.1 to 390.9)	-43.3<br>(-48.2 to -38.8)	-6.7<br>(-13.2 to 0.0)
	50-69 years	7,650<br>(7,010 to 8,270)	1,953.2<br>(1,789.6 to 2,111.7)	19,300<br>(17,600 to 21,100)	1,389.1<br>(1,266.2 to 1,515.8)	17,500<br>(16,200 to 18,900)	1,239.7<br>(1,143.9 to 1,336.9)	16,800<br>(15,100 to 18,400)	1,156.5<br>(1,038.5 to 1,267.8)	-28.9<br>(-34.7 to -22.2)	-10.8<br>(-18.1 to -3.6)
	70+ years	9,730<br>(9,090 to 10,400)	9,300.0<br>(8,685.4 to 9,904.9)	33,800<br>(31,300 to 36,300)	8,052.8<br>(7,469.0 to 8,648.5)	31,200<br>(29,100 to 33,800)	6,890.0<br>(6,428.8 to 7,468.5)	29,900<br>(27,600 to 32,600)	6,197.0<br>(5,717.4 to 6,749.0)	-13.4<br>(-19.0 to -8.4)	-14.4<br>(-20.3 to -7.7)
**Canada**	All Ages	562,000<br>(522,000 to 609,000)	2,060.4<br>(1,915.7 to 2,236.1)	700,000<br>(646,000 to 759,000)	1,901.5<br>(1,754.3 to 2,063.4)	686,000<br>(630,000 to 748,000)	1,846.8<br>(1,695.7 to 2,013.1)	599,000<br>(553,000 to 655,000)	1,599.8<br>(1,475.1 to 1,748.8)	-7.7<br>(-13.9 to -0.4)	-2.9<br>(-9.8 to 4.2)
	Under 5	35,900<br>(30,900 to 41,800)	1,866.9<br>(1,604.7 to 2,169.5)	21,700<br>(18,400 to 25,600)	1,122.2<br>(948.9 to 1,325.2)	19,600<br>(16,200 to 23,800)	1,021.2<br>(843.1 to 1,242.9)	13,500<br>(11,400 to 16,100)	709.7<br>(597.4 to 846.6)	-39.9<br>(-44.1 to -35.4)	-9.0<br>(-19.3 to 3.2)
	5-14 years	17,300<br>(13,000 to 22,100)	451.1<br>(341.0 to 578.7)	14,900<br>(11,200 to 19,200)	354.4<br>(267.3 to 459.2)	14,400<br>(11,000 to 18,900)	340.7<br>(259.1 to 446.5)	12,800<br>(9,860 to 16,500)	300.0<br>(230.8 to 386.7)	-21.4<br>(-26.5 to -15.1)	-3.9<br>(-12.0 to 4.5)
	15-49 years	89,900<br>(78,200 to 103,000)	609.4<br>(530.4 to 698.6)	76,200<br>(66,400 to 87,400)	464.0<br>(404.6 to 532.3)	73,800<br>(63,300 to 84,900)	446.5<br>(382.7 to 513.9)	65,400<br>(56,500 to 74,800)	393.1<br>(339.5 to 449.6)	-23.9<br>(-28.2 to -18.9)	-3.8<br>(-10.7 to 3.7)
	50-69 years	155,000<br>(138,000 to 174,000)	3,266.0<br>(2,913.4 to 3,659.4)	194,000<br>(175,000 to 217,000)	1,989.1<br>(1,790.8 to 2,218.9)	190,000<br>(170,000 to 212,000)	1,940.8<br>(1,734.7 to 2,163.3)	169,000<br>(151,000 to 189,000)	1,714.2<br>(1,533.3 to 1,918.0)	-39.1<br>(-45.0 to -33.1)	-2.4<br>(-11.0 to 6.4)

Location	Age Group	1990		2019		2020		2021		Percent Incidence Rate Change from 1990-2019 (95% UI)	Percent Incidence Rate Change from 2019-2021 (95% UI)
		Episodes (95% UI)	Rate (95% UI)	Episodes (95% UI)	Rate (95% UI)	Episodes (95% UI)	Rate (95% UI)	Episodes (95% UI)	Rate (95% UI)		
	70+ years	263,000 (238,000 to 290,000)	13,100.4 (11,854.7 to 14,434.0)	393,000 (352,000 to 444,000)	8,739.3 (7,835.1 to 9,884.5)	388,000 (346,000 to 432,000)	8,326.8 (7,431.5 to 9,272.7)	339,000 (304,000 to 384,000)	7,035.0 (6,300.1 to 7,970.2)	-33.3 (-38.8 to -25.4)	-4.7 (-13.1 to 4.5)
**Greenland**	All Ages	902 (845 to 972)	1,623.8 (1,520.5 to 1,749.0)	820 (760 to 895)	1,464.9 (1,357.3 to 1,598.6)	807 (744 to 873)	1,440.1 (1,327.1 to 1,557.0)	702 (648 to 764)	1,251.7 (1,154.2 to 1,362.1)	-9.8 (-16.1 to -2.8)	-1.7 (-8.9 to 7.2)
	Under 5	161 (142 to 184)	2,930.8 (2,584.4 to 3,335.2)	48 (41 to 56)	1,166.9 (1,006.9 to 1,363.1)	45 (38 to 52)	1,091.0 (926.8 to 1,272.7)	32 (27 to 37)	782.6 (669.3 to 912.1)	-60.2 (-64.2 to -55.9)	-6.5 (-15.0 to 2.5)
	5-14 years	45 (34 to 58)	519.6 (393.2 to 664.1)	26 (20 to 34)	337.6 (260.9 to 441.2)	25 (19 to 33)	327.5 (250.0 to 426.8)	22 (17 to 29)	285.3 (220.0 to 371.5)	-35.0 (-41.4 to -29.4)	-3.0 (-10.1 to 5.1)
	15-49 years	246 (217 to 278)	735.8 (647.3 to 831.5)	127 (112 to 142)	476.1 (420.7 to 534.4)	122 (106 to 137)	458.9 (401.9 to 515.6)	105 (92 to 118)	396.1 (349.0 to 446.3)	-35.3 (-40.0 to -29.5)	-3.6 (-11.6 to 5.3)
	50-69 years	261 (232 to 293)	3,935.3 (3,504.3 to 4,412.9)	353 (317 to 397)	2,387.8 (2,145.3 to 2,681.1)	353 (316 to 393)	2,360.3 (2,115.6 to 2,625.5)	313 (279 to 348)	2,101.2 (1,877.2 to 2,341.8)	-39.3 (-46.0 to -32.2)	-1.2 (-10.5 to 10.0)
	70+ years	188 (166 to 215)	15,178.3 (13,373.3 to 17,320.9)	267 (236 to 302)	9,432.7 (8,353.7 to 10,683.5)	263 (234 to 299)	9,225.4 (8,197.1 to 10,471.4)	231 (202 to 271)	7,687.5 (6,723.3 to 9,016.4)	-37.9 (-43.6 to -30.6)	-2.2 (-11.7 to 8.7)
**United States of America**	All Ages	5,790,000 (5,460,000 to 6,150,000)	2,276.9 (2,146.9 to 2,421.1)	6,440,000 (6,090,000 to 6,830,000)	1,953.0 (1,845.0 to 2,071.0)	5,970,000 (5,590,000 to 6,410,000)	1,802.3 (1,686.4 to 1,932.2)	5,040,000 (4,740,000 to 5,380,000)	1,515.9 (1,425.5 to 1,617.8)	-14.2 (-16.9 to -11.6)	-7.7 (-12.5 to -4.7)
	Under 5	399,000 (346,000 to 455,000)	2,017.9 (1,750.5 to 2,303.2)	214,000 (189,000 to 241,000)	1,120.6 (987.5 to 1,261.7)	191,000 (162,000 to 222,000)	1,013.5 (859.0 to 1,179.4)	176,000 (153,000 to 202,000)	946.4 (822.1 to 1,087.1)	-44.5 (-46.9 to -41.8)	-9.6 (-18.4 to -4.4)
	5-14 years	165,000 (124,000 to 215,000)	456.9 (341.8 to 594.7)	120,000 (96,900 to 146,000)	289.1 (234.3 to 352.0)	111,000 (89,100 to 137,000)	269.3 (216.5 to 332.8)	109,000 (88,400 to 136,000)	267.7 (216.5 to 332.1)	-36.7 (-43.3 to -28.3)	-6.9 (-12.0 to -3.0)
	15-49 years	915,000 (817,000 to 1,030,000)	681.3 (608.9 to 764.4)	659,000 (598,000 to 721,000)	435.0 (394.8 to 475.8)	582,000 (519,000 to 641,000)	383.5 (342.0 to 422.6)	575,000 (519,000 to 629,000)	378.1 (341.6 to 413.8)	-36.2 (-38.9 to -33.3)	-11.8 (-16.1 to -8.7)
	50-69 years	1,550,000 (1,420,000 to 1,670,000)	3,616.5 (3,317.8 to 3,912.0)	1,840,000 (1,700,000 to 1,970,000)	2,245.4 (2,081.5 to 2,410.8)	1,670,000 (1,540,000 to 1,810,000)	2,032.7 (1,866.1 to 2,204.0)	1,490,000 (1,380,000 to 1,610,000)	1,805.9 (1,666.0 to 1,942.6)	-37.9 (-40.0 to -35.8)	-9.5 (-13.6 to -6.7)
	70+ years	2,760,000 (2,530,000 to 3,060,000)	13,022.3 (11,916.6 to 14,414.8)	3,610,000 (3,340,000 to 3,930,000)	10,010.9 (9,257.5 to 10,873.8)	3,420,000 (3,140,000 to 3,730,000)	9,134.1 (8,401.5 to 9,973.8)	2,690,000 (2,470,000 to 2,920,000)	6,985.9 (6,430.8 to 7,584.6)	-23.1 (-26.6 to -20.2)	-8.8 (-14.0 to -5.4)
**Argentina**	All Ages	680,000 (629,000 to 736,000)	2,052.5 (1,898.3 to 2,223.7)	1,160,000 (1,080,000 to 1,240,000)	2,572.5 (2,403.3 to 2,764.5)	975,000 (901,000 to 1,040,000)	2,152.5 (1,989.9 to 2,307.0)	967,000 (904,000 to 1,030,000)	2,126.0 (1,986.3 to 2,263.8)	25.3 (17.5 to 33.4)	-16.3 (-21.0 to -11.2)
	Under 5	173,000 (145,000 to 203,000)	5,022.8 (4,209.3 to 5,893.1)	108,000 (90,000 to 129,000)	3,229.4 (2,680.9 to 3,835.6)	73,100 (59,600 to 88,800)	2,300.9 (1,876.4 to 2,793.2)	65,900 (54,100 to 80,000)	2,199.0 (1,807.2 to 2,669.6)	-35.7 (-43.2 to -28.2)	-28.8 (-37.0 to -18.7)
	5-14 years	65,300 (50,100 to 83,900)	975.5 (748.4 to 1,251.9)	55,900 (44,100 to 70,400)	786.0 (620.5 to 989.7)	49,400 (38,800 to 62,300)	690.9 (541.6 to 870.6)	48,700 (37,700 to 62,200)	677.2 (524.9 to 865.4)	-19.4 (-26.0 to -9.9)	-12.1 (-18.7 to -4.9)

Location	Age Group	1990		2019		2020		2021		Percent Incidence Rate Change from 1990-2019 (95% UI)	Percent Incidence Rate Change from 2019-2021 (95% UI)
		Episodes (95% UI)	Rate (95% UI)	Episodes (95% UI)	Rate (95% UI)	Episodes (95% UI)	Rate (95% UI)	Episodes (95% UI)	Rate (95% UI)		
	15-49 years	110,000 (97,500 to 123,000)	693.3 (613.7 to 772.6)	187,000 (170,000 to 207,000)	807.8 (734.0 to 893.1)	165,000 (149,000 to 182,000)	703.8 (636.9 to 777.9)	165,000 (149,000 to 183,000)	700.6 (632.9 to 775.9)	16.5 (5.8 to 27.6)	-12.9 (-19.5 to -5.5)
	50-69 years	150,000 (136,000 to 164,000)	2,863.9 (2,594.5 to 3,135.7)	297,000 (272,000 to 331,000)	3,738.9 (3,419.1 to 4,165.3)	259,000 (233,000 to 288,000)	3,208.3 (2,888.9 to 3,562.5)	259,000 (235,000 to 284,000)	3,156.2 (2,864.3 to 3,457.5)	30.6 (21.1 to 44.1)	-14.2 (-20.8 to -5.7)
	70+ years	182,000 (165,000 to 197,000)	9,819.2 (8,935.2 to 10,652.7)	509,000 (456,000 to 557,000)	14,879.3 (13,322.1 to 16,275.3)	429,000 (384,000 to 479,000)	12,249.6 (10,982.2 to 13,702.4)	429,000 (380,000 to 472,000)	12,031.6 (10,676.6 to 13,234.4)	51.5 (36.3 to 65.3)	-17.7 (-24.3 to -10.2)
**Chile**	All Ages	335,000 (315,000 to 357,000)	2,520.7 (2,370.2 to 2,685.7)	234,000 (219,000 to 249,000)	1,270.4 (1,187.7 to 1,352.1)	196,000 (185,000 to 208,000)	1,047.7 (988.9 to 1,114.6)	197,000 (185,000 to 211,000)	1,046.0 (985.8 to 1,123.3)	-49.6 (-52.7 to -46.6)	-17.5 (-22.1 to -11.9)
	Under 5	87,600 (75,500 to 102,000)	6,094.1 (5,255.3 to 7,086.4)	25,000 (20,600 to 30,200)	2,162.6 (1,782.0 to 2,613.1)	15,800 (13,000 to 18,900)	1,408.4 (1,154.0 to 1,684.3)	15,100 (12,300 to 18,300)	1,386.4 (1,133.9 to 1,682.0)	-64.5 (-69.9 to -57.7)	-34.9 (-40.0 to -28.9)
	5-14 years	27,900 (22,300 to 34,800)	1,099.2 (879.5 to 1,372.1)	14,400 (11,000 to 18,600)	574.3 (439.5 to 743.0)	12,500 (9,540 to 16,000)	489.5 (374.7 to 629.8)	12,400 (9,450 to 15,800)	482.0 (368.7 to 614.7)	-47.7 (-53.8 to -40.5)	-14.8 (-20.5 to -6.1)
	15-49 years	58,800 (53,200 to 64,600)	826.6 (747.9 to 908.1)	34,200 (30,400 to 38,300)	364.7 (324.3 to 408.7)	29,400 (26,200 to 32,900)	310.1 (276.2 to 347.6)	29,300 (26,000 to 33,100)	308.5 (273.5 to 348.3)	-55.9 (-59.5 to -51.4)	-15.0 (-20.1 to -8.5)
	50-69 years	71,400 (66,100 to 77,300)	4,294.1 (3,972.3 to 4,648.9)	56,800 (51,700 to 62,200)	1,442.7 (1,312.5 to 1,578.7)	49,900 (45,500 to 54,800)	1,243.6 (1,135.0 to 1,367.7)	50,500 (46,100 to 55,700)	1,237.1 (1,128.4 to 1,364.0)	-66.4 (-69.3 to -63.7)	-13.8 (-20.5 to -5.0)
	70+ years	89,200 (81,400 to 98,700)	16,518.1 (15,058.4 to 18,259.1)	104,000 (93,600 to 113,000)	7,128.7 (6,432.7 to 7,799.5)	88,000 (80,400 to 96,400)	5,826.3 (5,321.6 to 6,383.0)	89,300 (80,700 to 98,600)	5,735.0 (5,179.3 to 6,327.5)	-56.8 (-60.1 to -52.1)	-18.3 (-25.8 to -9.8)
**Uruguay**	All Ages	76,900 (70,900 to 83,900)	2,448.7 (2,257.2 to 2,672.1)	66,300 (61,400 to 71,600)	1,942.1 (1,800.7 to 2,097.2)	53,700 (50,000 to 57,700)	1,573.6 (1,466.5 to 1,693.1)	52,700 (49,000 to 56,200)	1,546.0 (1,437.7 to 1,650.8)	-20.7 (-25.8 to -16.0)	-19.0 (-23.6 to -13.8)
	Under 5	16,700 (14,000 to 19,800)	6,127.9 (5,140.8 to 7,272.0)	6,770 (5,680 to 8,090)	3,127.2 (2,622.7 to 3,737.6)	4,290 (3,520 to 5,160)	2,097.4 (1,722.0 to 2,519.7)	3,920 (3,260 to 4,710)	2,023.3 (1,682.7 to 2,427.0)	-49.0 (-54.3 to -43.3)	-32.9 (-39.3 to -25.3)
	5-14 years	6,050 (4,560 to 7,780)	1,109.0 (835.0 to 1,425.7)	3,790 (2,880 to 4,950)	811.8 (616.4 to 1,059.6)	3,250 (2,440 to 4,240)	696.6 (522.4 to 908.2)	3,190 (2,440 to 4,150)	685.0 (523.9 to 892.3)	-26.8 (-32.9 to -20.4)	-14.2 (-20.9 to -7.4)
	15-49 years	10,800 (9,500 to 12,100)	725.2 (639.6 to 817.7)	8,550 (7,700 to 9,570)	520.2 (468.3 to 582.0)	7,240 (6,480 to 8,080)	440.6 (393.9 to 491.5)	7,140 (6,420 to 7,980)	435.1 (391.0 to 486.2)	-28.3 (-33.5 to -22.3)	-15.3 (-20.8 to -9.4)
	50-69 years	17,100 (15,400 to 19,400)	2,898.1 (2,613.7 to 3,291.5)	14,200 (12,900 to 15,700)	1,983.4 (1,797.8 to 2,186.9)	12,100 (11,000 to 13,400)	1,679.5 (1,517.4 to 1,858.1)	12,000 (11,100 to 13,000)	1,648.9 (1,517.1 to 1,787.2)	-31.6 (-38.4 to -25.3)	-15.3 (-22.3 to -7.3)
	70+ years	26,200 (23,000 to 30,200)	10,691.0 (9,389.5 to 12,313.4)	32,900 (29,100 to 37,200)	8,953.9 (7,902.0 to 10,097.1)	26,700 (24,100 to 29,700)	7,178.6 (6,468.3 to 7,983.6)	26,400 (23,600 to 29,100)	7,018.6 (6,291.0 to 7,746.3)	-16.2 (-25.3 to -8.3)	-19.8 (-26.1 to -12.0)
**Andorra**	All Ages	370 (342 to 401)	680.0 (629.2 to 738.5)	675 (620 to 727)	813.1 (745.9 to 874.8)	639 (595 to 688)	756.9 (705.1 to 815.8)	584 (541 to 636)	682.6 (632.0 to 743.1)	19.6 (11.2 to 29.2)	-6.9 (-12.8 to -0.9)

Location	Age Group	1990		2019		2020		2021		Percent Incidence Rate Change from 1990-2019 (95% UI)	Percent Incidence Rate Change from 2019-2021 (95% UI)
		Episodes (95% UI)	Rate (95% UI)	Episodes (95% UI)	Rate (95% UI)	Episodes (95% UI)	Rate (95% UI)	Episodes (95% UI)	Rate (95% UI)		
	Under 5	38<br>(32 to 45)	1,411.0<br>(1,190.6 to 1,671.3)	22<br>(18 to 27)	818.7<br>(659.1 to 1,015.6)	17<br>(14 to 21)	670.4<br>(537.6 to 832.2)	13<br>(10 to 15)	497.9<br>(400.4 to 608.2)	-42.0<br>(-47.7 to -36.2)	-18.1<br>(-24.5 to -10.8)
	5-14 years	16<br>(12 to 20)	229.6<br>(171.6 to 301.0)	16<br>(11 to 20)	194.6<br>(143.3 to 253.3)	14<br>(10 to 18)	177.9<br>(132.5 to 234.1)	12<br>(9 to 16)	159.5<br>(119.3 to 209.9)	-15.3<br>(-21.1 to -9.2)	-8.6<br>(-15.0 to -1.7)
	15-49 years	76<br>(66 to 90)	235.1<br>(202.9 to 278.1)	91<br>(79 to 104)	222.5<br>(192.8 to 253.8)	85<br>(73 to 98)	206.6<br>(178.2 to 239.1)	77<br>(66 to 89)	186.7<br>(159.2 to 216.5)	-5.4<br>(-12.5 to 2.2)	-7.1<br>(-14.0 to -0.1)
	50-69 years	98<br>(87 to 109)	1,064.8<br>(939.0 to 1,183.3)	175<br>(154 to 194)	762.6<br>(672.1 to 846.7)	170<br>(151 to 190)	714.2<br>(634.7 to 797.3)	162<br>(144 to 181)	653.0<br>(577.5 to 729.3)	-28.4<br>(-34.2 to -20.8)	-6.3<br>(-14.0 to 1.9)
	70+ years	141<br>(124 to 159)	4,410.0<br>(3,866.6 to 4,970.0)	372<br>(333 to 422)	4,327.1<br>(3,871.2 to 4,904.5)	352<br>(316 to 392)	3,915.1<br>(3,507.0 to 4,355.7)	320<br>(286 to 357)	3,430.9<br>(3,067.3 to 3,824.2)	-1.9<br>(-13.1 to 9.7)	-9.5<br>(-17.5 to -1.4)
**Austria**	All Ages	81,700<br>(77,000 to 87,000)	1,051.6<br>(991.3 to 1,120.4)	69,000<br>(64,100 to 74,600)	775.2<br>(720.2 to 837.7)	64,700<br>(60,200 to 69,900)	723.8<br>(673.1 to 781.8)	59,200<br>(54,800 to 63,600)	659.6<br>(610.0 to 707.9)	-26.3<br>(-31.3 to -20.6)	-6.6<br>(-12.6 to -0.6)
	Under 5	7,220<br>(6,240 to 8,480)	1,614.1<br>(1,396.5 to 1,895.8)	4,220<br>(3,440 to 5,180)	969.9<br>(791.2 to 1,189.7)	3,480<br>(2,830 to 4,330)	800.6<br>(651.6 to 996.3)	2,640<br>(2,140 to 3,220)	611.1<br>(494.5 to 744.5)	-39.9<br>(-46.1 to -32.6)	-17.5<br>(-23.6 to -11.0)
	5-14 years	3,200<br>(2,530 to 4,100)	355.3<br>(280.8 to 454.7)	2,120<br>(1,640 to 2,760)	250.2<br>(193.2 to 325.1)	2,000<br>(1,520 to 2,620)	234.4<br>(178.1 to 305.7)	1,870<br>(1,420 to 2,430)	215.9<br>(164.5 to 280.7)	-29.6<br>(-38.3 to -20.9)	-6.3<br>(-12.6 to 0.9)
	15-49 years	13,300<br>(11,800 to 15,000)	331.4<br>(293.6 to 371.9)	8,880<br>(7,650 to 10,300)	220.0<br>(189.6 to 254.6)	8,320<br>(7,160 to 9,540)	206.1<br>(177.3 to 236.4)	7,660<br>(6,540 to 8,870)	189.9<br>(162.1 to 219.9)	-33.6<br>(-39.6 to -26.9)	-6.3<br>(-12.6 to 0.7)
	50-69 years	21,600<br>(19,800 to 23,600)	1,321.6<br>(1,215.5 to 1,445.4)	15,600<br>(13,800 to 17,400)	662.4<br>(584.0 to 736.8)	14,900<br>(13,300 to 16,600)	626.6<br>(559.1 to 695.7)	14,100<br>(12,500 to 15,700)	585.6<br>(517.7 to 652.9)	-49.9<br>(-55.1 to -44.8)	-5.4<br>(-13.4 to 2.6)
	70+ years	36,400<br>(33,300 to 39,900)	4,770.3<br>(4,365.3 to 5,239.6)	38,200<br>(34,800 to 42,100)	3,120.0<br>(2,846.2 to 3,438.2)	36,000<br>(32,200 to 40,100)	2,913.2<br>(2,602.4 to 3,240.4)	33,000<br>(29,600 to 36,400)	2,651.1<br>(2,382.8 to 2,929.0)	-34.6<br>(-40.2 to -27.8)	-6.6<br>(-14.9 to 2.6)
**Belgium**	All Ages	131,000<br>(122,000 to 142,000)	1,314.4<br>(1,225.9 to 1,422.5)	156,000<br>(144,000 to 168,000)	1,368.6<br>(1,263.4 to 1,476.0)	149,000<br>(138,000 to 161,000)	1,305.3<br>(1,204.0 to 1,409.1)	129,000<br>(119,000 to 140,000)	1,126.7<br>(1,038.4 to 1,218.2)	4.1<br>(-2.8 to 11.0)	-4.6<br>(-10.2 to 1.8)
	Under 5	8,260<br>(6,780 to 9,950)	1,385.3<br>(1,137.5 to 1,668.6)	5,490<br>(4,500 to 6,770)	902.0<br>(739.6 to 1,112.5)	4,810<br>(3,830 to 6,000)	801.9<br>(638.3 to 1,000.6)	3,340<br>(2,710 to 4,130)	564.8<br>(458.3 to 697.3)	-34.9<br>(-40.3 to -28.4)	-11.1<br>(-21.4 to -1.1)
	5-14 years	3,500<br>(2,620 to 4,570)	289.6<br>(216.3 to 378.0)	3,100<br>(2,330 to 4,080)	235.6<br>(177.3 to 310.1)	2,930<br>(2,190 to 3,870)	222.1<br>(166.2 to 293.0)	2,580<br>(1,950 to 3,410)	195.2<br>(147.5 to 258.3)	-18.7<br>(-24.4 to -12.3)	-5.7<br>(-12.9 to 1.8)
	15-49 years	14,900<br>(12,800 to 17,100)	301.0<br>(258.9 to 344.0)	12,500<br>(11,000 to 14,400)	250.4<br>(219.2 to 288.0)	11,900<br>(10,300 to 13,700)	238.2<br>(206.5 to 272.9)	10,500<br>(9,080 to 12,100)	209.5<br>(181.9 to 241.6)	-16.8<br>(-22.0 to -10.2)	-4.9<br>(-11.6 to 1.7)
	50-69 years	36,800<br>(33,300 to 40,800)	1,634.9<br>(1,477.3 to 1,809.2)	35,400<br>(31,400 to 39,700)	1,211.9<br>(1,074.9 to 1,359.4)	34,000<br>(30,400 to 37,400)	1,158.0<br>(1,035.9 to 1,274.9)	30,500<br>(27,400 to 33,800)	1,033.1<br>(928.8 to 1,144.6)	-25.9<br>(-33.0 to -17.8)	-4.4<br>(-12.6 to 3.7)
	70+ years	67,600<br>(61,900 to 75,100)	7,039.9<br>(6,445.2 to 7,814.9)	99,700<br>(90,800 to 111,000)	6,390.7<br>(5,817.1 to 7,090.3)	95,700<br>(87,100 to 105,000)	6,042.9<br>(5,498.9 to 6,653.4)	82,300<br>(74,500 to 91,100)	5,119.2<br>(4,631.8 to 5,663.8)	-9.2<br>(-16.7 to -0.9)	-5.4<br>(-12.3 to 2.4)

Location	Age Group	1990		2019		2020		2021		Percent Incidence Rate Change from 1990-2019 (95% UI)	Percent Incidence Rate Change from 2019-2021 (95% UI)
		Episodes (95% UI)	Rate (95% UI)	Episodes (95% UI)	Rate (95% UI)	Episodes (95% UI)	Rate (95% UI)	Episodes (95% UI)	Rate (95% UI)		
**Cyprus**	All Ages	5,760 (5,340 to 6,190)	740.2 (685.9 to 795.5)	7,220 (6,740 to 7,770)	547.2 (510.8 to 589.2)	6,830 (6,360 to 7,360)	510.1 (474.9 to 549.8)	6,290 (5,810 to 6,790)	463.0 (427.7 to 499.8)	-26.1 (-30.7 to -19.5)	-6.8 (-11.8 to -1.1)
	Under 5	743 (625 to 881)	1,162.6 (978.2 to 1,379.0)	536 (435 to 657)	727.0 (589.7 to 890.5)	441 (358 to 550)	591.3 (480.8 to 737.3)	332 (270 to 405)	441.9 (359.7 to 539.9)	-37.5 (-42.7 to -31.6)	-18.7 (-25.2 to -11.4)
	5-14 years	295 (225 to 390)	220.0 (167.6 to 291.3)	239 (180 to 314)	172.1 (129.3 to 225.6)	224 (169 to 296)	158.8 (119.5 to 209.5)	208 (154 to 272)	144.8 (107.1 to 189.1)	-21.8 (-26.9 to -15.6)	-7.7 (-13.6 to -1.4)
	15-49 years	1,030 (894 to 1,180)	254.4 (221.3 to 291.2)	1,250 (1,070 to 1,460)	180.0 (153.6 to 210.3)	1,180 (1,000 to 1,390)	168.3 (142.8 to 198.9)	1,090 (925 to 1,280)	154.3 (131.0 to 181.0)	-29.2 (-34.7 to -23.5)	-6.5 (-12.8 to 0.5)
	50-69 years	1,600 (1,400 to 1,780)	1,278.3 (1,120.2 to 1,427.5)	1,880 (1,690 to 2,080)	653.8 (588.5 to 725.7)	1,780 (1,580 to 1,990)	608.4 (541.6 to 682.5)	1,660 (1,470 to 1,840)	557.1 (494.8 to 616.9)	-48.9 (-53.4 to -42.6)	-6.9 (-14.4 to 1.6)
	70+ years	2,100 (1,840 to 2,360)	4,070.3 (3,568.7 to 4,585.6)	3,320 (3,000 to 3,740)	2,673.0 (2,419.5 to 3,018.6)	3,210 (2,880 to 3,560)	2,466.8 (2,213.1 to 2,734.2)	3,000 (2,660 to 3,330)	2,216.3 (1,965.1 to 2,459.0)	-34.3 (-41.3 to -24.5)	-7.7 (-15.2 to 0.3)
**Denmark**	All Ages	57,300 (52,800 to 61,500)	1,113.0 (1,026.6 to 1,195.4)	67,500 (62,400 to 73,500)	1,163.9 (1,076.2 to 1,267.2)	61,300 (56,400 to 67,100)	1,052.2 (968.1 to 1,151.2)	55,100 (51,100 to 59,600)	941.2 (872.9 to 1,018.0)	4.6 (-2.6 to 13.3)	-9.6 (-16.4 to -2.1)
	Under 5	3,660 (3,040 to 4,340)	1,262.1 (1,049.1 to 1,499.2)	2,640 (2,170 to 3,250)	864.8 (709.3 to 1,062.5)	2,120 (1,660 to 2,670)	686.0 (539.1 to 864.7)	1,610 (1,320 to 1,970)	518.4 (424.3 to 634.6)	-31.5 (-37.8 to -25.1)	-20.7 (-30.8 to -8.4)
	5-14 years	1,510 (1,140 to 1,970)	254.2 (191.6 to 331.8)	1,430 (1,080 to 1,870)	218.7 (166.1 to 286.2)	1,280 (958 to 1,680)	197.7 (148.0 to 259.9)	1,150 (869 to 1,500)	179.4 (135.1 to 233.3)	-14.0 (-20.1 to -7.9)	-9.6 (-17.0 to 0.0)
	15-49 years	7,260 (6,360 to 8,360)	271.8 (237.8 to 312.8)	5,620 (4,910 to 6,450)	219.2 (191.7 to 251.6)	5,140 (4,380 to 5,940)	200.0 (170.4 to 231.4)	4,650 (4,040 to 5,300)	180.8 (157.3 to 206.3)	-19.3 (-25.5 to -13.5)	-8.8 (-15.8 to -1.6)
	50-69 years	13,000 (11,600 to 14,400)	1,261.1 (1,132.2 to 1,399.1)	14,300 (12,800 to 15,700)	978.3 (875.3 to 1,075.5)	13,100 (11,700 to 14,400)	893.0 (802.2 to 987.4)	11,900 (10,600 to 13,100)	808.8 (723.0 to 890.4)	-22.4 (-31.2 to -13.3)	-8.7 (-16.7 to 0.8)
	70+ years	31,800 (28,900 to 34,600)	5,697.5 (5,163.1 to 6,188.7)	43,500 (39,300 to 48,500)	5,308.4 (4,792.0 to 5,908.7)	39,700 (36,000 to 44,200)	4,725.1 (4,277.8 to 5,257.5)	35,800 (32,500 to 39,900)	4,164.6 (3,777.3 to 4,641.8)	-6.8 (-14.3 to 3.0)	-11.0 (-18.4 to -2.0)
**Finland**	All Ages	74,200 (69,400 to 78,700)	1,481.6 (1,384.4 to 1,570.4)	44,600 (41,400 to 48,300)	806.3 (749.4 to 872.9)	40,200 (37,200 to 44,200)	727.3 (671.6 to 798.5)	37,200 (34,800 to 40,000)	672.6 (628.5 to 723.4)	-45.6 (-49.4 to -41.7)	-9.8 (-15.9 to -3.3)
	Under 5	4,540 (3,770 to 5,410)	1,451.0 (1,206.3 to 1,730.5)	2,720 (2,170 to 3,360)	1,058.3 (845.1 to 1,309.5)	2,060 (1,640 to 2,630)	832.2 (661.7 to 1,061.5)	1,610 (1,280 to 1,990)	663.6 (529.1 to 819.6)	-27.1 (-34.3 to -19.5)	-21.4 (-30.7 to -10.9)
	5-14 years	2,000 (1,540 to 2,590)	307.2 (235.4 to 397.2)	1,510 (1,120 to 1,960)	245.6 (182.8 to 320.3)	1,360 (1,020 to 1,740)	223.0 (166.7 to 285.8)	1,250 (940 to 1,600)	206.2 (155.5 to 264.7)	-20.1 (-26.4 to -14.0)	-9.2 (-16.3 to -1.0)
	15-49 years	9,800 (8,730 to 10,900)	379.5 (338.0 to 420.6)	5,090 (4,340 to 5,880)	218.0 (186.0 to 251.8)	4,630 (3,940 to 5,380)	198.3 (168.5 to 230.3)	4,310 (3,670 to 5,020)	184.4 (157.0 to 214.8)	-42.5 (-48.8 to -35.6)	-9.1 (-15.4 to -1.4)
	50-69 years	19,700 (18,100 to 21,800)	1,962.0 (1,797.5 to 2,164.5)	9,890 (8,720 to 11,100)	686.1 (604.6 to 768.4)	8,980 (8,030 to 9,960)	629.5 (562.5 to 698.1)	8,270 (7,400 to 9,210)	585.4 (523.7 to 651.6)	-65.0 (-69.3 to -60.5)	-8.3 (-17.0 to 0.1)

Location	Age Group	1990		2019		2020		2021		Percent Incidence Rate Change from 1990-2019 (95% UI)	Percent Incidence Rate Change from 2019-2021 (95% UI)
		Episodes (95% UI)	Rate (95% UI)	Episodes (95% UI)	Rate (95% UI)	Episodes (95% UI)	Rate (95% UI)	Episodes (95% UI)	Rate (95% UI)		
	70+ years	38,200 (34,800 to 41,200)	8,343.4 (7,617.5 to 9,013.1)	25,400 (23,000 to 28,200)	2,870.1 (2,603.0 to 3,189.0)	23,200 (20,700 to 26,200)	2,542.5 (2,268.4 to 2,871.3)	21,800 (20,000 to 23,900)	2,326.1 (2,135.5 to 2,555.4)	-65.6 (-68.5 to -62.0)	-11.4 (-18.7 to -3.6)
**France**	All Ages	555,000 (519,000 to 594,000)	960.8 (898.1 to 1,028.7)	679,000 (632,000 to 731,000)	1,026.3 (954.8 to 1,104.7)	638,000 (578,000 to 692,000)	963.2 (872.1 to 1,043.3)	562,000 (519,000 to 605,000)	845.8 (782.4 to 912.0)	6.8 (-0.1 to 14.0)	-6.1 (-12.8 to 1.3)
	Under 5	40,100 (33,000 to 48,800)	1,030.8 (847.4 to 1,255.2)	29,100 (23,600 to 35,700)	798.0 (647.4 to 980.9)	24,000 (18,400 to 30,700)	669.1 (514.3 to 856.2)	17,300 (13,900 to 21,500)	489.9 (392.8 to 608.0)	-22.6 (-28.7 to -16.7)	-16.2 (-26.6 to -3.6)
	5-14 years	19,600 (14,800 to 25,400)	250.4 (188.5 to 325.0)	16,900 (12,400 to 22,100)	206.8 (152.0 to 271.5)	15,600 (11,700 to 20,300)	191.4 (144.1 to 249.9)	13,800 (10,400 to 18,000)	171.3 (129.0 to 222.8)	-17.4 (-22.6 to -10.9)	-7.4 (-15.0 to 0.9)
	15-49 years	73,300 (63,500 to 82,500)	251.2 (217.7 to 282.8)	58,800 (50,900 to 67,600)	206.7 (179.2 to 237.8)	54,600 (47,100 to 62,600)	192.5 (166.0 to 220.7)	48,600 (41,400 to 56,000)	171.7 (146.4 to 198.1)	-17.7 (-23.5 to -11.5)	-6.9 (-14.6 to 1.4)
	50-69 years	138,000 (125,000 to 153,000)	1,183.2 (1,067.2 to 1,314.6)	141,000 (126,000 to 156,000)	855.8 (762.1 to 946.9)	133,000 (118,000 to 148,000)	803.5 (712.7 to 892.6)	118,000 (105,000 to 131,000)	714.3 (636.9 to 790.8)	-27.7 (-35.2 to -19.5)	-6.1 (-14.5 to 2.3)
	70+ years	284,000 (261,000 to 310,000)	5,457.0 (5,023.0 to 5,960.5)	433,000 (394,000 to 478,000)	4,599.5 (4,189.8 to 5,073.9)	411,000 (365,000 to 458,000)	4,250.1 (3,767.2 to 4,731.7)	364,000 (328,000 to 400,000)	3,660.2 (3,301.9 to 4,027.4)	-15.7 (-22.4 to -8.0)	-7.6 (-15.5 to 1.7)
**Germany**	All Ages	806,000 (744,000 to 872,000)	1,008.3 (930.6 to 1,090.2)	941,000 (871,000 to 1,020,000)	1,108.0 (1,024.8 to 1,205.4)	876,000 (807,000 to 958,000)	1,027.6 (947.4 to 1,124.6)	800,000 (730,000 to 870,000)	937.0 (855.1 to 1,018.7)	9.9 (2.9 to 19.7)	-7.3 (-13.7 to -0.6)
	Under 5	56,300 (46,300 to 68,900)	1,257.3 (1,033.4 to 1,537.4)	36,600 (29,700 to 45,000)	902.0 (731.4 to 1,107.9)	29,600 (23,800 to 37,100)	728.9 (585.2 to 911.6)	21,900 (17,600 to 27,000)	540.6 (435.6 to 667.0)	-28.3 (-34.5 to -19.8)	-19.2 (-27.1 to -9.8)
	5-14 years	23,700 (17,500 to 30,700)	280.4 (206.9 to 362.4)	17,900 (13,400 to 23,400)	233.5 (175.0 to 304.8)	16,800 (12,500 to 21,800)	216.4 (160.2 to 279.8)	15,700 (12,000 to 20,700)	198.0 (151.0 to 261.6)	-16.7 (-22.6 to -10.9)	-7.3 (-15.9 to -0.5)
	15-49 years	114,000 (99,600 to 130,000)	286.5 (249.6 to 325.7)	84,800 (73,800 to 97,800)	236.7 (205.9 to 272.9)	78,200 (68,000 to 90,500)	218.9 (190.5 to 253.5)	71,000 (61,600 to 82,400)	199.5 (173.0 to 231.5)	-17.4 (-24.0 to -9.4)	-7.5 (-13.6 to -0.5)
	50-69 years	230,000 (207,000 to 255,000)	1,208.7 (1,088.1 to 1,340.4)	231,000 (206,000 to 256,000)	956.8 (853.7 to 1,059.8)	217,000 (191,000 to 250,000)	897.5 (789.9 to 1,031.5)	200,000 (176,000 to 226,000)	825.6 (724.3 to 930.2)	-20.8 (-27.7 to -11.4)	-6.2 (-15.8 to 2.3)
	70+ years	382,000 (340,000 to 421,000)	4,721.9 (4,208.4 to 5,211.6)	571,000 (515,000 to 631,000)	4,308.7 (3,884.2 to 4,764.8)	534,000 (484,000 to 596,000)	3,974.8 (3,603.7 to 4,438.9)	491,000 (437,000 to 549,000)	3,622.1 (3,224.9 to 4,051.7)	-8.8 (-17.1 to 1.8)	-7.7 (-14.8 to 0.5)
**Greece**	All Ages	97,000 (90,600 to 105,000)	933.2 (871.9 to 1,014.4)	124,000 (116,000 to 133,000)	1,198.0 (1,127.4 to 1,284.9)	122,000 (113,000 to 131,000)	1,190.4 (1,106.5 to 1,281.7)	103,000 (95,700 to 111,000)	1,010.1 (940.5 to 1,088.5)	28.4 (21.1 to 35.5)	-0.6 (-7.8 to 6.3)
	Under 5	7,070 (5,840 to 8,500)	1,274.0 (1,052.5 to 1,531.7)	4,210 (3,500 to 5,090)	963.6 (800.8 to 1,164.9)	3,940 (3,080 to 4,920)	914.6 (714.6 to 1,143.7)	2,440 (2,000 to 2,940)	577.2 (472.5 to 695.9)	-24.4 (-30.4 to -17.6)	-5.1 (-19.5 to 6.3)
	5-14 years	3,900 (2,960 to 5,090)	265.4 (201.7 to 346.6)	2,460 (1,900 to 3,170)	244.8 (189.8 to 315.5)	2,360 (1,840 to 3,020)	238.3 (186.5 to 305.4)	1,980 (1,550 to 2,540)	203.6 (159.2 to 261.0)	-7.7 (-15.9 to 0.6)	-2.7 (-10.7 to 6.6)
	15-49 years	13,400 (11,500 to 15,400)	264.6 (228.3 to 304.8)	11,400 (9,980 to 12,700)	254.0 (223.4 to 283.6)	11,000 (9,580 to 12,500)	249.6 (217.9 to 283.9)	9,300 (8,220 to 10,400)	214.9 (189.9 to 241.2)	-4.0 (-11.4 to 3.4)	-1.7 (-9.3 to 5.7)

Location	Age Group	1990		2019		2020		2021		Percent Incidence Rate Change from 1990-2019 (95% UI)	Percent Incidence Rate Change from 2019-2021 (95% UI)
		Episodes (95% UI)	Rate (95% UI)	Episodes (95% UI)	Rate (95% UI)	Episodes (95% UI)	Rate (95% UI)	Episodes (95% UI)	Rate (95% UI)		
	50-69 years	26,900 (24,400 to 29,800)	1,137.7 (1,028.6 to 1,259.7)	23,900 (21,600 to 26,200)	888.5 (804.2 to 976.8)	23,800 (21,500 to 26,200)	877.6 (791.9 to 965.8)	20,600 (18,600 to 22,600)	758.2 (681.8 to 829.8)	-21.9 (-27.4 to -15.2)	-1.2 (-10.3 to 8.1)
	70+ years	45,700 (41,600 to 51,300)	4,821.5 (4,389.3 to 5,417.6)	81,700 (75,700 to 89,600)	4,749.9 (4,398.6 to 5,210.1)	81,000 (73,600 to 89,100)	4,691.3 (4,266.7 to 5,161.7)	68,400 (61,900 to 75,200)	3,952.3 (3,573.1 to 4,345.2)	-1.5 (-9.1 to 6.3)	-1.2 (-9.7 to 7.0)
**Iceland**	All Ages	2,860 (2,670 to 3,080)	1,127.9 (1,051.2 to 1,213.1)	2,970 (2,750 to 3,210)	863.1 (798.4 to 932.3)	2,680 (2,430 to 3,000)	769.8 (699.3 to 863.0)	2,950 (2,670 to 3,280)	842.1 (761.7 to 936.5)	-23.5 (-28.5 to -17.5)	-10.8 (-18.5 to -0.2)
	Under 5	281 (235 to 331)	1,329.7 (1,114.5 to 1,565.0)	184 (150 to 224)	862.9 (704.3 to 1,047.6)	139 (105 to 187)	646.2 (484.7 to 865.8)	168 (127 to 213)	763.6 (577.7 to 968.9)	-35.1 (-41.4 to -29.9)	-25.1 (-36.9 to -6.5)
	5-14 years	106 (80 to 138)	250.7 (188.7 to 326.0)	90 (68 to 116)	195.6 (147.3 to 253.8)	79 (59 to 105)	173.8 (129.1 to 229.2)	84 (62 to 109)	183.5 (135.4 to 240.1)	-22.0 (-27.0 to -16.6)	-11.2 (-18.6 to -2.5)
	15-49 years	390 (344 to 440)	294.3 (259.7 to 332.4)	355 (308 to 407)	217.4 (188.9 to 249.3)	321 (277 to 378)	195.9 (168.7 to 230.7)	346 (297 to 401)	210.0 (180.3 to 243.4)	-26.1 (-31.4 to -20.1)	-9.9 (-16.8 to -0.4)
	50-69 years	652 (583 to 730)	1,639.8 (1,467.1 to 1,836.8)	726 (650 to 808)	917.7 (821.5 to 1,021.0)	670 (593 to 751)	838.7 (741.9 to 940.6)	715 (629 to 811)	890.2 (783.4 to 1,009.9)	-44.0 (-48.8 to -38.3)	-8.6 (-18.1 to 2.6)
	70+ years	1,440 (1,280 to 1,600)	7,855.4 (7,030.3 to 8,770.8)	1,620 (1,440 to 1,810)	4,637.8 (4,115.5 to 5,182.2)	1,460 (1,300 to 1,680)	4,037.7 (3,578.6 to 4,620.5)	1,640 (1,450 to 1,880)	4,331.1 (3,823.5 to 4,972.1)	-41.0 (-46.5 to -34.4)	-12.9 (-21.7 to -1.4)
**Ireland**	All Ages	46,400 (43,100 to 50,000)	1,289.3 (1,197.0 to 1,387.1)	42,800 (39,900 to 45,800)	873.7 (814.1 to 934.6)	40,700 (36,600 to 45,100)	826.8 (743.2 to 916.3)	36,400 (33,800 to 39,400)	737.5 (684.7 to 797.3)	-32.2 (-37.2 to -27.5)	-5.4 (-13.0 to 3.5)
	Under 5	3,970 (3,280 to 4,810)	1,374.1 (1,133.6 to 1,663.3)	2,520 (2,040 to 3,070)	803.7 (651.1 to 979.5)	2,090 (1,560 to 2,740)	684.5 (509.9 to 896.7)	1,480 (1,210 to 1,830)	495.1 (404.2 to 614.5)	-41.5 (-48.2 to -34.4)	-14.8 (-30.3 to 3.3)
	5-14 years	1,840 (1,400 to 2,370)	265.2 (201.2 to 342.2)	1,440 (1,080 to 1,860)	204.7 (154.1 to 264.1)	1,340 (989 to 1,780)	190.4 (140.9 to 254.3)	1,180 (902 to 1,540)	169.5 (129.1 to 220.3)	-22.8 (-29.0 to -15.6)	-7.0 (-16.0 to 2.7)
	15-49 years	5,150 (4,550 to 5,810)	288.9 (255.3 to 326.3)	4,860 (4,160 to 5,630)	208.5 (178.8 to 241.8)	4,550 (3,880 to 5,350)	196.3 (167.1 to 230.7)	4,080 (3,500 to 4,700)	176.9 (151.5 to 203.8)	-27.8 (-33.9 to -21.7)	-5.8 (-14.1 to 3.1)
	50-69 years	11,500 (10,400 to 12,900)	2,025.0 (1,827.6 to 2,257.1)	9,420 (8,460 to 10,500)	875.7 (787.0 to 972.1)	8,990 (7,920 to 10,200)	821.4 (723.9 to 932.6)	8,260 (7,430 to 9,090)	740.1 (665.9 to 814.8)	-56.8 (-61.1 to -51.8)	-6.2 (-14.3 to 4.4)
	70+ years	24,000 (21,900 to 26,000)	8,914.8 (8,167.7 to 9,691.3)	24,600 (22,300 to 26,800)	5,096.6 (4,613.1 to 5,546.6)	23,700 (21,100 to 26,800)	4,731.2 (4,202.0 to 5,345.1)	21,400 (19,500 to 23,900)	4,124.2 (3,753.4 to 4,600.0)	-42.8 (-48.2 to -37.5)	-7.2 (-15.8 to 2.3)
**Israel**	All Ages	37,600 (35,200 to 40,500)	757.8 (709.3 to 817.3)	62,300 (58,000 to 67,200)	670.8 (624.7 to 723.7)	57,600 (53,500 to 62,200)	610.6 (567.2 to 658.8)	52,200 (48,700 to 56,400)	544.2 (507.6 to 587.7)	-11.5 (-16.3 to -6.3)	-9.0 (-14.5 to -3.2)
	Under 5	7,300 (6,080 to 8,650)	1,414.6 (1,177.5 to 1,674.7)	6,760 (5,560 to 8,230)	738.6 (607.3 to 899.1)	5,530 (4,500 to 6,780)	603.3 (490.4 to 739.3)	4,200 (3,420 to 5,120)	457.3 (372.7 to 557.4)	-47.8 (-53.4 to -42.1)	-18.3 (-25.1 to -10.9)
	5-14 years	2,430 (1,840 to 3,160)	239.0 (181.0 to 310.8)	3,200 (2,390 to 4,170)	194.7 (145.2 to 253.1)	2,990 (2,220 to 3,890)	178.1 (132.5 to 232.0)	2,770 (2,090 to 3,640)	161.8 (122.5 to 213.1)	-18.6 (-24.4 to -11.6)	-8.5 (-16.0 to -1.5)

Location	Age Group	1990		2019		2020		2021		Percent Incidence Rate Change from 1990-2019 (95% UI)	Percent Incidence Rate Change from 2019-2021 (95% UI)
		Episodes (95% UI)	Rate (95% UI)	Episodes (95% UI)	Rate (95% UI)	Episodes (95% UI)	Rate (95% UI)	Episodes (95% UI)	Rate (95% UI)		
	15-49 years	5,480 (4,680 to 6,320)	225.8 (192.9 to 260.4)	8,370 (7,220 to 9,620)	191.3 (165.1 to 219.7)	7,840 (6,810 to 8,980)	176.7 (153.4 to 202.5)	7,220 (6,240 to 8,350)	160.7 (139.0 to 185.9)	-15.3 (-20.8 to -9.1)	-7.6 (-12.9 to -0.6)
	50-69 years	8,330 (7,400 to 9,180)	1,195.4 (1,062.0 to 1,317.3)	13,800 (12,300 to 15,200)	864.5 (772.8 to 951.1)	12,900 (11,500 to 14,200)	797.5 (710.2 to 874.8)	12,000 (10,700 to 13,300)	725.6 (648.8 to 804.9)	-27.7 (-34.6 to -20.9)	-7.7 (-16.1 to 1.8)
	70+ years	14,000 (12,700 to 15,600)	4,621.3 (4,188.5 to 5,127.0)	30,200 (27,100 to 33,100)	4,014.7 (3,605.3 to 4,408.2)	28,400 (25,600 to 31,800)	3,594.9 (3,243.9 to 4,026.3)	26,100 (23,700 to 29,100)	3,158.1 (2,870.7 to 3,528.0)	-13.1 (-20.7 to -4.5)	-10.5 (-17.5 to -3.0)
**Italy**	All Ages	474,000 (444,000 to 505,000)	834.9 (781.4 to 888.6)	588,000 (552,000 to 630,000)	974.6 (914.9 to 1,044.3)	536,000 (503,000 to 570,000)	892.8 (837.8 to 949.7)	477,000 (448,000 to 512,000)	797.9 (748.5 to 856.7)	16.7 (11.9 to 22.0)	-8.4 (-11.1 to -5.5)
	Under 5	39,900 (33,900 to 46,400)	1,453.3 (1,236.4 to 1,691.1)	22,700 (19,200 to 26,900)	975.0 (825.2 to 1,154.5)	17,800 (15,000 to 21,300)	791.5 (665.9 to 949.8)	12,700 (10,700 to 15,000)	583.9 (493.9 to 690.8)	-32.9 (-38.2 to -26.8)	-18.8 (-23.5 to -13.4)
	5-14 years	18,600 (14,000 to 24,500)	286.1 (216.1 to 377.6)	12,800 (10,000 to 16,200)	229.4 (179.6 to 289.3)	11,600 (9,010 to 14,600)	209.8 (163.4 to 264.4)	10,200 (8,010 to 12,900)	187.9 (147.5 to 236.8)	-19.8 (-27.1 to -11.2)	-8.6 (-11.1 to -5.9)
	15-49 years	70,200 (60,700 to 80,600)	244.4 (211.4 to 280.8)	45,900 (40,900 to 52,000)	181.1 (161.3 to 204.9)	41,600 (37,000 to 47,400)	166.4 (148.1 to 189.6)	36,700 (32,700 to 41,300)	149.2 (132.8 to 168.0)	-25.9 (-30.0 to -21.4)	-8.1 (-10.7 to -5.2)
	50-69 years	132,000 (120,000 to 145,000)	993.1 (898.3 to 1,087.6)	121,000 (111,000 to 132,000)	726.7 (663.4 to 789.5)	113,000 (103,000 to 123,000)	668.1 (611.2 to 725.5)	104,000 (94,600 to 113,000)	605.8 (553.7 to 658.5)	-26.8 (-29.8 to -23.7)	-8.1 (-10.8 to -4.8)
	70+ years	213,000 (194,000 to 236,000)	3,853.2 (3,510.7 to 4,266.2)	385,000 (354,000 to 422,000)	3,735.9 (3,434.6 to 4,096.6)	352,000 (326,000 to 385,000)	3,381.6 (3,126.8 to 3,697.9)	314,000 (289,000 to 345,000)	2,987.3 (2,743.7 to 3,276.4)	-3.0 (-7.3 to 1.7)	-9.5 (-12.5 to -6.3)
**Luxembourg**	All Ages	3,560 (3,310 to 3,830)	935.3 (868.1 to 1,004.2)	4,540 (4,200 to 4,910)	734.3 (679.2 to 793.8)	4,230 (3,940 to 4,520)	669.5 (624.9 to 716.2)	3,810 (3,540 to 4,090)	590.9 (549.3 to 634.7)	-21.5 (-27.0 to -16.4)	-8.8 (-14.8 to -3.0)
	Under 5	298 (249 to 356)	1,302.5 (1,088.7 to 1,555.1)	266 (216 to 326)	821.9 (667.2 to 1,008.7)	217 (178 to 267)	665.6 (544.9 to 818.7)	162 (132 to 199)	490.5 (398.2 to 600.4)	-36.9 (-43.3 to -28.8)	-19.0 (-25.9 to -11.5)
	5-14 years	107 (81 to 138)	247.6 (188.4 to 319.4)	131 (97 to 171)	197.1 (146.5 to 258.3)	121 (90 to 159)	179.8 (134.2 to 236.5)	109 (83 to 141)	160.3 (121.3 to 206.6)	-20.4 (-26.9 to -14.7)	-8.8 (-15.7 to -1.4)
	15-49 years	534 (465 to 607)	268.5 (233.9 to 305.1)	609 (527 to 703)	198.5 (171.6 to 228.9)	570 (493 to 659)	182.6 (158.0 to 211.2)	520 (444 to 603)	163.8 (140.0 to 190.0)	-26.0 (-32.1 to -19.5)	-8.1 (-14.2 to -1.0)
	50-69 years	972 (860 to 1,070)	1,183.8 (1,048.4 to 1,307.2)	1,060 (941 to 1,180)	705.3 (627.3 to 786.9)	1,020 (911 to 1,130)	656.3 (588.6 to 731.2)	943 (842 to 1,040)	592.2 (528.7 to 651.3)	-40.4 (-46.7 to -34.1)	-7.0 (-14.7 to 2.5)
	70+ years	1,660 (1,490 to 1,870)	4,845.3 (4,355.2 to 5,481.8)	2,480 (2,240 to 2,770)	3,955.8 (3,573.1 to 4,431.5)	2,300 (2,070 to 2,560)	3,569.9 (3,212.8 to 3,969.8)	2,070 (1,830 to 2,310)	3,118.2 (2,756.8 to 3,476.6)	-18.4 (-26.2 to -10.0)	-9.8 (-17.3 to -1.6)
**Malta**	All Ages	3,740 (3,480 to 4,020)	1,007.7 (938.4 to 1,084.2)	5,480 (5,090 to 5,880)	1,246.3 (1,156.7 to 1,337.1)	5,290 (4,900 to 5,750)	1,198.5 (1,111.0 to 1,303.0)	5,020 (4,670 to 5,460)	1,135.9 (1,056.4 to 1,233.7)	23.7 (16.6 to 31.3)	-3.8 (-9.0 to 2.4)
	Under 5	440 (367 to 519)	1,557.1 (1,298.4 to 1,838.7)	230 (191 to 275)	1,043.7 (868.5 to 1,252.0)	194 (159 to 237)	879.6 (719.5 to 1,073.6)	152 (126 to 182)	689.3 (572.0 to 828.1)	-33.0 (-38.9 to -27.0)	-15.7 (-22.4 to -8.5)

Location	Age Group	1990 Episodes (95% UI)	1990 Rate (95% UI)	2019 Episodes (95% UI)	2019 Rate (95% UI)	2020 Episodes (95% UI)	2020 Rate (95% UI)	2021 Episodes (95% UI)	2021 Rate (95% UI)	Percent Incidence Rate Change from 1990-2019 (95% UI)	Percent Incidence Rate Change from 2019-2021 (95% UI)
	5-14 years	160 (122 to 208)	269.8 (206.5 to 351.2)	90 (68 to 118)	219.5 (164.1 to 286.2)	86 (64 to 113)	206.5 (154.7 to 270.9)	81 (60 to 105)	192.0 (141.8 to 250.9)	-18.6 (-24.7 to -13.2)	-5.9 (-12.6 to 1.7)
	15-49 years	605 (528 to 682)	315.6 (275.7 to 355.8)	514 (451 to 578)	261.1 (228.9 to 293.4)	489 (425 to 554)	249.9 (217.0 to 282.7)	457 (398 to 524)	235.1 (204.3 to 269.4)	-17.3 (-24.1 to -11.3)	-4.3 (-11.2 to 2.7)
	50-69 years	1,020 (911 to 1,140)	1,537.1 (1,366.9 to 1,707.1)	1,270 (1,140 to 1,400)	1,120.1 (1,010.9 to 1,238.6)	1,200 (1,080 to 1,330)	1,072.5 (965.9 to 1,186.8)	1,140 (1,020 to 1,260)	1,015.0 (913.1 to 1,129.1)	-27.1 (-33.1 to -20.8)	-4.3 (-11.5 to 5.1)
	70+ years	1,510 (1,360 to 1,700)	6,064.6 (5,477.4 to 6,868.7)	3,380 (3,070 to 3,730)	5,083.6 (4,615.7 to 5,606.9)	3,320 (3,040 to 3,660)	4,774.2 (4,380.4 to 5,278.3)	3,200 (2,910 to 3,570)	4,454.9 (4,050.1 to 4,973.7)	-16.2 (-22.8 to -8.2)	-6.1 (-12.4 to 1.2)
**Monaco**	All Ages	439 (402 to 479)	1,442.6 (1,322.0 to 1,575.4)	527 (482 to 576)	1,399.7 (1,281.4 to 1,528.7)	480 (440 to 527)	1,270.9 (1,165.5 to 1,395.4)	424 (389 to 464)	1,118.9 (1,027.7 to 1,224.3)	-3.0 (-10.4 to 4.5)	-9.2 (-15.3 to -2.1)
	Under 5	16 (13 to 19)	1,364.7 (1,147.8 to 1,629.0)	14 (11 to 17)	861.9 (692.5 to 1,066.4)	11 (9 to 14)	696.7 (561.4 to 872.2)	8 (7 to 10)	513.6 (411.9 to 631.7)	-36.8 (-43.4 to -29.9)	-19.2 (-26.0 to -11.2)
	5-14 years	6 (4 to 7)	242.6 (182.9 to 316.1)	7 (5 to 9)	201.5 (149.7 to 262.2)	6 (5 to 8)	184.7 (138.5 to 241.7)	6 (4 to 7)	167.1 (124.8 to 220.4)	-16.9 (-23.3 to -10.2)	-8.3 (-14.0 to -2.3)
	15-49 years	40 (35 to 46)	291.2 (254.0 to 331.6)	36 (31 to 40)	249.4 (218.8 to 283.0)	32 (28 to 37)	228.3 (200.9 to 260.6)	29 (25 to 32)	203.7 (178.8 to 230.4)	-14.4 (-21.3 to -6.6)	-8.5 (-14.9 to -1.8)
	50-69 years	106 (95 to 118)	1,296.6 (1,161.4 to 1,449.0)	108 (96 to 120)	945.1 (837.0 to 1,053.1)	101 (90 to 112)	873.2 (775.7 to 968.3)	93 (83 to 103)	792.9 (707.5 to 883.0)	-27.1 (-34.4 to -19.9)	-7.6 (-15.0 to 1.2)
	70+ years	271 (241 to 303)	5,475.2 (4,862.0 to 6,123.3)	363 (325 to 409)	5,164.6 (4,625.3 to 5,825.2)	329 (293 to 371)	4,650.6 (4,137.7 to 5,237.6)	288 (258 to 322)	4,048.8 (3,623.6 to 4,529.7)	-5.7 (-14.7 to 4.4)	-10.0 (-17.6 to -1.4)
**Netherlands**	All Ages	123,000 (114,000 to 131,000)	827.2 (761.5 to 880.4)	155,000 (142,000 to 169,000)	902.1 (828.2 to 984.0)	147,000 (132,000 to 162,000)	853.2 (769.8 to 944.5)	127,000 (117,000 to 138,000)	736.8 (682.0 to 801.1)	9.1 (2.5 to 17.5)	-5.4 (-14.4 to 4.0)
	Under 5	8,960 (7,480 to 10,800)	957.3 (798.7 to 1,151.0)	5,870 (4,760 to 7,210)	684.6 (554.8 to 841.2)	5,150 (3,980 to 6,550)	601.2 (464.9 to 764.1)	3,560 (2,880 to 4,340)	413.0 (335.0 to 504.1)	-28.5 (-34.7 to -22.7)	-12.2 (-25.6 to 2.0)
	5-14 years	3,640 (2,720 to 4,760)	203.3 (151.9 to 266.1)	3,170 (2,380 to 4,130)	170.9 (128.4 to 222.6)	2,960 (2,210 to 3,900)	161.5 (120.2 to 212.2)	2,560 (1,910 to 3,300)	140.3 (104.8 to 181.1)	-16.0 (-21.8 to -9.2)	-5.5 (-13.9 to 3.1)
	15-49 years	16,900 (14,400 to 19,600)	208.2 (178.1 to 241.7)	13,500 (11,600 to 15,600)	180.1 (154.3 to 208.0)	12,700 (10,700 to 14,800)	169.7 (143.9 to 198.2)	10,900 (9,430 to 12,600)	146.5 (127.3 to 170.1)	-13.5 (-19.3 to -6.7)	-5.8 (-14.0 to 2.4)
	50-69 years	27,900 (24,800 to 30,400)	993.0 (881.5 to 1,084.5)	35,100 (31,200 to 39,600)	765.6 (680.3 to 861.8)	33,600 (29,400 to 37,500)	726.2 (634.6 to 809.6)	29,800 (26,900 to 32,900)	638.5 (576.5 to 705.7)	-22.9 (-30.1 to -14.9)	-5.2 (-15.0 to 5.4)
	70+ years	66,100 (59,700 to 72,100)	5,150.0 (4,656.8 to 5,618.5)	96,900 (86,800 to 108,000)	4,159.9 (3,726.5 to 4,646.4)	92,200 (81,700 to 103,000)	3,843.6 (3,409.4 to 4,292.9)	80,000 (72,500 to 89,200)	3,258.9 (2,953.2 to 3,632.7)	-19.2 (-25.9 to -11.9)	-7.6 (-17.7 to 3.4)
**Norway**	All Ages	77,800 (73,000 to 83,300)	1,831.9 (1,719.5 to 1,961.1)	59,600 (55,800 to 64,200)	1,114.3 (1,042.9 to 1,200.6)	53,400 (49,200 to 58,400)	992.8 (913.6 to 1,085.7)	48,600 (45,400 to 52,600)	897.5 (838.3 to 970.4)	-39.2 (-41.8 to -36.9)	-10.9 (-15.8 to -4.6)

Location	Age Group	1990		2019		2020		2021		Percent Incidence Rate Change from 1990-2019 (95% UI)	Percent Incidence Rate Change from 2019-2021 (95% UI)
		Episodes (95% UI)	Rate (95% UI)	Episodes (95% UI)	Rate (95% UI)	Episodes (95% UI)	Rate (95% UI)	Episodes (95% UI)	Rate (95% UI)		
	Under 5	3,570<br>(3,060 to 4,210)	1,293.5<br>(1,110.2 to 1,526.3)	2,840<br>(2,340 to 3,460)	975.2<br>(805.8 to 1,191.4)	2,180<br>(1,710 to 2,830)	765.6<br>(598.3 to 993.9)	1,630<br>(1,350 to 1,990)	580.4<br>(479.5 to 708.3)	-24.6<br>(-29.0 to -19.9)	-21.5<br>(-29.8 to -10.5)
	5-14 years	1,740<br>(1,330 to 2,260)	334.0<br>(254.1 to 432.1)	2,050<br>(1,560 to 2,640)	318.9<br>(243.5 to 411.5)	1,850<br>(1,410 to 2,400)	288.0<br>(219.4 to 373.8)	1,670<br>(1,280 to 2,160)	259.6<br>(199.7 to 335.4)	-4.5<br>(-8.3 to -0.9)	-9.7<br>(-14.1 to -4.2)
	15-49 years	6,760<br>(5,900 to 7,690)	311.9<br>(272.2 to 354.9)	5,990<br>(5,160 to 6,870)	240.3<br>(207.2 to 275.6)	5,440<br>(4,610 to 6,390)	218.1<br>(184.9 to 256.0)	4,940<br>(4,250 to 5,720)	197.9<br>(170.2 to 228.9)	-23.0<br>(-26.0 to -20.3)	-9.2<br>(-13.5 to -4.2)
	50-69 years	15,400<br>(14,000 to 16,600)	1,938.0<br>(1,771.5 to 2,096.3)	10,800<br>(9,690 to 11,900)	852.8<br>(762.8 to 937.9)	9,920<br>(8,900 to 11,000)	770.2<br>(691.1 to 850.0)	9,160<br>(8,150 to 10,100)	701.8<br>(625.0 to 770.4)	-56.0<br>(-58.4 to -53.5)	-9.7<br>(-13.9 to -4.5)
	70+ years	50,400<br>(46,600 to 54,900)	10,305.5<br>(9,545.3 to 11,229.0)	37,900<br>(34,700 to 41,700)	5,811.9<br>(5,321.3 to 6,402.9)	34,000<br>(30,700 to 37,700)	5,063.0<br>(4,559.3 to 5,601.1)	31,200<br>(28,600 to 34,200)	4,511.0<br>(4,127.4 to 4,941.6)	-43.6<br>(-46.8 to -40.5)	-12.9<br>(-18.0 to -6.5)
**Portugal**	All Ages	130,000<br>(122,000 to 139,000)	1,285.9<br>(1,204.5 to 1,372.7)	152,000<br>(141,000 to 163,000)	1,420.6<br>(1,323.7 to 1,526.2)	148,000<br>(136,000 to 161,000)	1,386.5<br>(1,276.9 to 1,511.3)	131,000<br>(122,000 to 141,000)	1,233.9<br>(1,151.6 to 1,325.7)	10.5<br>(2.0 to 19.5)	-2.4<br>(-9.7 to 7.5)
	Under 5	12,900<br>(11,000 to 14,900)	2,231.7<br>(1,908.7 to 2,573.0)	3,770<br>(3,160 to 4,530)	873.4<br>(732.8 to 1,049.6)	3,360<br>(2,620 to 4,200)	781.8<br>(609.0 to 976.1)	2,290<br>(1,900 to 2,760)	538.0<br>(445.6 to 647.9)	-60.9<br>(-65.9 to -55.1)	-10.5<br>(-24.5 to 2.4)
	5-14 years	5,590<br>(4,380 to 7,060)	363.8<br>(284.6 to 459.3)	2,170<br>(1,690 to 2,800)	222.3<br>(172.6 to 286.5)	2,040<br>(1,590 to 2,680)	213.3<br>(165.8 to 280.5)	1,780<br>(1,350 to 2,310)	189.7<br>(144.5 to 246.3)	-38.9<br>(-44.5 to -32.4)	-4.0<br>(-12.9 to 3.9)
	15-49 years	20,700<br>(18,600 to 22,900)	414.5<br>(372.3 to 457.1)	12,800<br>(11,400 to 14,000)	272.5<br>(243.4 to 299.3)	12,300<br>(10,900 to 14,000)	264.8<br>(234.2 to 301.6)	10,900<br>(9,560 to 12,200)	237.9<br>(208.8 to 267.4)	-34.3<br>(-39.0 to -28.8)	-2.8<br>(-9.6 to 6.4)
	50-69 years	37,700<br>(34,100 to 41,900)	1,743.7<br>(1,578.8 to 1,941.2)	31,900<br>(29,100 to 35,100)	1,116.1<br>(1,017.4 to 1,226.4)	31,300<br>(28,100 to 34,400)	1,087.7<br>(976.0 to 1,196.7)	28,200<br>(25,800 to 30,900)	975.1<br>(892.5 to 1,067.7)	-36.0<br>(-42.2 to -29.7)	-2.5<br>(-10.6 to 7.2)
	70+ years	53,500<br>(48,200 to 59,300)	6,218.5<br>(5,602.8 to 6,894.0)	101,000<br>(92,300 to 110,000)	5,903.4<br>(5,399.1 to 6,462.8)	98,600<br>(89,400 to 110,000)	5,662.4<br>(5,135.3 to 6,308.0)	87,700<br>(79,900 to 95,900)	4,955.3<br>(4,516.9 to 5,420.7)	-5.1<br>(-14.4 to 5.3)	-4.1<br>(-12.3 to 7.0)
**San Marino**	All Ages	206<br>(191 to 223)	868.0<br>(802.1 to 937.9)	333<br>(305 to 359)	1,019.7<br>(936.6 to 1,101.5)	313<br>(287 to 343)	957.7<br>(879.6 to 1,050.8)	289<br>(265 to 313)	881.4<br>(809.8 to 957.3)	17.5<br>(10.4 to 27.1)	-6.1<br>(-12.6 to 0.8)
	Under 5	15<br>(13 to 18)	1,291.3<br>(1,079.1 to 1,542.3)	10<br>(8 to 13)	800.1<br>(652.0 to 982.0)	8<br>(7 to 10)	661.5<br>(534.4 to 822.9)	6<br>(5 to 8)	507.3<br>(416.5 to 623.2)	-38.0<br>(-43.3 to -31.4)	-17.3<br>(-24.6 to -10.3)
	5-14 years	7<br>(5 to 9)	226.3<br>(170.1 to 293.2)	7<br>(5 to 9)	195.8<br>(146.2 to 258.7)	6<br>(4 to 8)	182.3<br>(136.4 to 236.6)	5<br>(4 to 7)	168.9<br>(126.5 to 221.2)	-13.5<br>(-20.2 to -6.8)	-6.9<br>(-13.9 to 0.1)
	15-49 years	28<br>(24 to 32)	225.7<br>(191.9 to 257.6)	29<br>(24 to 33)	204.6<br>(175.0 to 236.7)	27<br>(23 to 31)	191.4<br>(164.6 to 220.7)	24<br>(21 to 28)	176.3<br>(150.6 to 204.6)	-9.3<br>(-15.4 to -3.1)	-6.4<br>(-12.3 to 0.4)
	50-69 years	48<br>(43 to 54)	979.9<br>(876.0 to 1,102.2)	62<br>(54 to 69)	686.3<br>(603.1 to 765.0)	59<br>(52 to 67)	646.8<br>(570.8 to 725.1)	56<br>(50 to 62)	599.3<br>(533.4 to 663.1)	-30.0<br>(-36.2 to -23.3)	-5.8<br>(-14.0 to 2.7)
	70+ years	108<br>(96 to 120)	4,643.3<br>(4,124.5 to 5,157.1)	225<br>(199 to 252)	4,495.5<br>(3,978.9 to 5,020.7)	213<br>(190 to 241)	4,152.3<br>(3,704.5 to 4,697.5)	197<br>(175 to 221)	3,772.2<br>(3,356.9 to 4,241.2)	-3.2<br>(-12.4 to 7.9)	-7.6<br>(-16.5 to 1.3)

Location	Age Group	1990		2019		2020		2021		Percent Incidence Rate Change from 1990-2019 (95% UI)	Percent Incidence Rate Change from 2019-2021 (95% UI)
		Episodes (95% UI)	Rate (95% UI)	Episodes (95% UI)	Rate (95% UI)	Episodes (95% UI)	Rate (95% UI)	Episodes (95% UI)	Rate (95% UI)		
**Spain**	All Ages	466,000 (439,000 to 501,000)	1,202.6 (1,131.5 to 1,290.9)	512,000 (477,000 to 547,000)	1,112.0 (1,036.7 to 1,189.0)	469,000 (434,000 to 515,000)	1,023.8 (947.1 to 1,125.1)	437,000 (405,000 to 473,000)	958.4 (889.7 to 1,037.6)	-7.5 (-13.8 to -0.9)	-7.9 (-13.5 to -0.1)
	Under 5	35,100 (29,200 to 41,500)	1,684.5 (1,400.3 to 1,994.5)	21,100 (17,300 to 25,600)	1,063.7 (869.4 to 1,285.7)	15,700 (12,500 to 19,600)	820.8 (653.8 to 1,025.5)	12,000 (9,760 to 14,800)	652.7 (530.1 to 802.6)	-36.9 (-43.5 to -30.9)	-22.8 (-31.3 to -13.0)
	5-14 years	18,800 (14,200 to 24,200)	326.1 (246.3 to 420.1)	13,100 (10,000 to 17,200)	274.6 (210.4 to 361.2)	11,800 (8,940 to 15,200)	249.9 (189.9 to 322.5)	10,900 (8,360 to 14,000)	234.4 (180.2 to 302.1)	-15.8 (-23.3 to -8.2)	-9.0 (-16.1 to -0.9)
	15-49 years	73,600 (64,200 to 83,000)	378.7 (330.6 to 427.4)	56,000 (49,000 to 64,100)	270.9 (237.1 to 310.2)	50,700 (43,900 to 57,200)	249.7 (216.1 to 281.3)	46,900 (41,100 to 53,300)	234.7 (205.6 to 266.7)	-28.5 (-35.4 to -22.0)	-7.8 (-14.1 to -1.5)
	50-69 years	130,000 (118,000 to 144,000)	1,610.9 (1,462.1 to 1,778.6)	110,000 (97,600 to 121,000)	921.8 (816.8 to 1,011.1)	104,000 (93,100 to 115,000)	857.9 (768.2 to 950.9)	99,500 (89,600 to 111,000)	808.0 (727.8 to 902.1)	-42.8 (-49.3 to -36.5)	-6.9 (-15.2 to 4.0)
	70+ years	209,000 (192,000 to 230,000)	6,059.8 (5,557.1 to 6,668.3)	311,000 (282,000 to 345,000)	4,692.2 (4,256.6 to 5,202.0)	286,000 (258,000 to 319,000)	4,268.2 (3,841.0 to 4,756.5)	267,000 (240,000 to 296,000)	3,949.8 (3,553.3 to 4,375.7)	-22.6 (-29.4 to -15.2)	-9.0 (-16.5 to 0.9)
**Sweden**	All Ages	148,000 (139,000 to 158,000)	1,728.0 (1,619.4 to 1,835.9)	119,000 (111,000 to 128,000)	1,159.4 (1,081.2 to 1,256.5)	105,000 (98,500 to 113,000)	1,022.9 (956.2 to 1,096.1)	102,000 (93,500 to 111,000)	982.1 (901.0 to 1,070.9)	-32.9 (-37.0 to -29.2)	-11.8 (-16.1 to -7.2)
	Under 5	8,600 (7,370 to 10,100)	1,527.6 (1,308.6 to 1,799.9)	6,580 (5,460 to 8,080)	1,116.6 (926.3 to 1,370.9)	4,980 (4,010 to 6,170)	848.6 (682.7 to 1,052.1)	4,390 (3,440 to 5,580)	753.5 (590.7 to 956.4)	-26.9 (-31.9 to -21.5)	-24.0 (-30.2 to -17.6)
	5-14 years	4,160 (3,200 to 5,360)	424.2 (326.2 to 546.2)	5,240 (4,030 to 6,770)	432.0 (332.3 to 558.3)	4,750 (3,610 to 6,100)	386.9 (293.9 to 497.2)	4,570 (3,480 to 5,900)	369.3 (281.4 to 476.9)	1.8 (-2.7 to 7.4)	-10.4 (-15.7 to -5.7)
	15-49 years	18,000 (15,900 to 20,400)	428.5 (379.4 to 484.3)	15,000 (13,000 to 17,200)	335.4 (290.4 to 383.7)	13,700 (11,800 to 15,600)	303.1 (261.0 to 346.8)	13,200 (11,400 to 15,200)	291.3 (251.7 to 334.8)	-21.7 (-26.7 to -17.1)	-9.6 (-13.9 to -4.8)
	50-69 years	32,700 (30,100 to 35,600)	1,869.5 (1,721.0 to 2,032.6)	22,200 (19,900 to 24,600)	924.7 (830.4 to 1,024.8)	20,100 (18,000 to 22,100)	834.9 (745.1 to 917.9)	19,800 (17,900 to 22,100)	813.1 (736.2 to 907.0)	-50.5 (-54.9 to -46.7)	-9.7 (-15.9 to -2.8)
	70+ years	84,900 (78,100 to 92,200)	7,779.4 (7,155.0 to 8,448.2)	69,500 (62,500 to 77,300)	4,504.6 (4,054.4 to 5,010.3)	61,900 (56,400 to 68,300)	3,935.3 (3,585.5 to 4,347.0)	60,000 (54,000 to 66,600)	3,759.5 (3,388.2 to 4,176.8)	-42.1 (-47.1 to -37.7)	-12.6 (-18.1 to -6.5)
**Switzerland**	All Ages	70,000 (65,100 to 74,600)	1,019.0 (948.1 to 1,086.5)	65,600 (60,200 to 71,300)	747.6 (686.4 to 812.6)	60,400 (56,400 to 65,100)	682.8 (637.3 to 735.3)	55,000 (50,600 to 59,300)	616.7 (567.2 to 664.8)	-26.6 (-32.5 to -20.7)	-8.7 (-15.0 to -1.8)
	Under 5	4,980 (4,250 to 5,970)	1,250.9 (1,066.3 to 1,499.4)	3,040 (2,460 to 3,700)	687.0 (556.4 to 836.9)	2,480 (1,990 to 3,040)	560.0 (450.8 to 688.6)	1,840 (1,500 to 2,260)	416.0 (339.5 to 511.1)	-45.1 (-51.1 to -38.7)	-18.5 (-25.2 to -11.3)
	5-14 years	1,580 (1,190 to 2,100)	208.9 (157.7 to 277.5)	1,470 (1,110 to 1,910)	170.4 (128.0 to 220.5)	1,380 (1,020 to 1,830)	156.8 (116.6 to 208.0)	1,280 (954 to 1,660)	143.3 (107.1 to 186.2)	-18.4 (-24.4 to -11.7)	-8.0 (-15.1 to -0.5)
	15-49 years	8,470 (7,330 to 9,680)	233.4 (202.1 to 267.0)	6,720 (5,730 to 7,920)	168.4 (143.5 to 198.4)	6,240 (5,320 to 7,330)	156.0 (133.1 to 183.2)	5,700 (4,890 to 6,680)	142.2 (122.0 to 166.8)	-27.9 (-33.3 to -22.5)	-7.4 (-13.0 to -1.1)
	50-69 years	16,300 (14,800 to 17,800)	1,176.7 (1,065.1 to 1,283.5)	13,300 (11,800 to 14,800)	585.2 (519.2 to 651.3)	12,600 (11,200 to 13,900)	545.4 (486.3 to 604.7)	11,800 (10,500 to 13,200)	503.0 (449.5 to 564.4)	-50.3 (-55.1 to -44.2)	-6.8 (-15.6 to 2.2)

Location	Age Group	1990		2019		2020		2021		Percent Incidence Rate Change from 1990-2019 (95% UI)	Percent Incidence Rate Change from 2019-2021 (95% UI)
		Episodes (95% UI)	Rate (95% UI)	Episodes (95% UI)	Rate (95% UI)	Episodes (95% UI)	Rate (95% UI)	Episodes (95% UI)	Rate (95% UI)		
	70+ years	38,600 (35,000 to 42,400)	5,538.9 (5,019.3 to 6,076.5)	41,100 (36,400 to 46,500)	3,418.6 (3,031.2 to 3,873.3)	37,800 (34,700 to 41,600)	3,079.4 (2,826.7 to 3,394.1)	34,500 (30,500 to 38,300)	2,762.0 (2,443.8 to 3,069.4)	-38.3 (-44.9 to -31.7)	-9.9 (-17.3 to -1.0)
**United Kingdom**	All Ages	996,000 (937,000 to 1,070,000)	1,737.8 (1,636.1 to 1,863.5)	1,110,000 (1,040,000 to 1,190,000)	1,650.1 (1,552.5 to 1,769.0)	1,020,000 (957,000 to 1,090,000)	1,502.5 (1,416.0 to 1,606.2)	900,000 (845,000 to 965,000)	1,325.9 (1,245.8 to 1,421.7)	-5.0 (-8.4 to -1.7)	-8.9 (-11.3 to -6.3)
	Under 5	52,600 (44,400 to 61,300)	1,368.0 (1,155.6 to 1,594.6)	47,500 (40,200 to 55,600)	1,246.4 (1,056.4 to 1,460.9)	38,000 (32,000 to 45,300)	1,017.8 (857.7 to 1,214.3)	27,800 (23,700 to 32,700)	762.0 (648.1 to 894.2)	-8.9 (-14.0 to -3.1)	-18.3 (-23.0 to -13.3)
	5-14 years	22,900 (17,300 to 29,900)	323.9 (244.0 to 423.0)	19,500 (15,000 to 25,300)	243.5 (187.9 to 316.3)	18,000 (13,800 to 23,400)	223.0 (171.5 to 289.7)	16,200 (12,500 to 21,100)	199.8 (154.2 to 259.6)	-24.8 (-27.1 to -22.5)	-8.4 (-10.4 to -6.0)
	15-49 years	102,000 (90,800 to 114,000)	357.2 (319.2 to 400.2)	92,600 (83,100 to 103,000)	305.0 (273.7 to 340.3)	84,900 (76,200 to 94,900)	279.7 (251.1 to 312.7)	76,200 (68,000 to 85,300)	251.1 (224.1 to 281.2)	-14.6 (-16.4 to -12.8)	-8.3 (-10.4 to -6.0)
	50-69 years	244,000 (225,000 to 265,000)	2,088.9 (1,919.7 to 2,266.9)	242,000 (222,000 to 262,000)	1,492.5 (1,366.6 to 1,617.3)	225,000 (207,000 to 244,000)	1,373.9 (1,261.7 to 1,488.2)	206,000 (189,000 to 224,000)	1,244.9 (1,139.7 to 1,352.8)	-28.6 (-30.7 to -26.3)	-7.9 (-9.9 to -5.6)
	70+ years	574,000 (531,000 to 630,000)	9,229.1 (8,536.2 to 10,119.1)	708,000 (653,000 to 777,000)	7,983.3 (7,361.1 to 8,764.8)	650,000 (599,000 to 709,000)	7,178.1 (6,618.2 to 7,837.5)	573,000 (528,000 to 628,000)	6,263.1 (5,767.6 to 6,866.3)	-13.5 (-17.5 to -9.4)	-10.1 (-12.5 to -7.3)
**Bolivia (Plurinational State of)**	All Ages	424,000 (387,000 to 465,000)	6,640.5 (6,068.8 to 7,283.5)	345,000 (322,000 to 370,000)	2,989.6 (2,790.2 to 3,205.1)	288,000 (268,000 to 310,000)	2,467.8 (2,291.0 to 2,658.2)	279,000 (262,000 to 297,000)	2,365.7 (2,217.1 to 2,515.8)	-55.0 (-58.0 to -51.5)	-17.5 (-22.1 to -12.4)
	Under 5	220,000 (188,000 to 257,000)	21,769.8 (18,569.4 to 25,476.8)	76,800 (65,600 to 89,600)	6,353.0 (5,423.9 to 7,410.6)	55,100 (46,800 to 64,500)	4,583.5 (3,891.2 to 5,369.1)	51,900 (44,500 to 60,600)	4,349.3 (3,723.7 to 5,075.1)	-70.8 (-73.2 to -67.7)	-27.9 (-35.1 to -18.9)
	5-14 years	57,100 (45,700 to 71,500)	3,409.1 (2,729.7 to 4,264.5)	32,300 (26,200 to 39,000)	1,435.0 (1,164.8 to 1,734.5)	28,000 (22,600 to 34,500)	1,233.1 (995.9 to 1,519.2)	27,600 (22,100 to 33,800)	1,202.6 (965.1 to 1,476.7)	-57.9 (-62.9 to -52.2)	-14.1 (-24.6 to -3.9)
	15-49 years	55,800 (50,200 to 61,900)	1,866.2 (1,679.0 to 2,069.0)	62,200 (55,900 to 68,700)	1,020.6 (917.5 to 1,126.1)	54,700 (49,600 to 60,900)	885.8 (802.6 to 985.8)	53,700 (48,600 to 59,700)	858.0 (776.6 to 954.8)	-45.3 (-50.3 to -40.8)	-13.2 (-18.4 to -6.6)
	50-69 years	44,900 (39,700 to 50,700)	8,103.7 (7,158.1 to 9,142.5)	76,100 (68,400 to 85,000)	4,999.5 (4,491.7 to 5,582.2)	67,800 (60,300 to 75,900)	4,339.9 (3,856.3 to 4,857.3)	67,500 (60,400 to 75,100)	4,241.7 (3,797.2 to 4,723.7)	-38.3 (-44.2 to -30.8)	-13.2 (-20.6 to -4.6)
	70+ years	46,000 (40,500 to 52,200)	30,633.0 (26,997.5 to 34,800.9)	97,500 (87,200 to 111,000)	21,234.6 (18,994.1 to 24,101.6)	82,600 (73,900 to 93,700)	17,785.1 (15,910.2 to 20,181.9)	78,400 (69,700 to 88,200)	16,946.5 (15,055.1 to 19,062.5)	-30.7 (-37.4 to -21.2)	-16.2 (-25.2 to -8.6)
**Ecuador**	All Ages	402,000 (373,000 to 434,000)	4,026.0 (3,739.0 to 4,344.8)	395,000 (372,000 to 419,000)	2,259.9 (2,128.7 to 2,395.3)	365,000 (327,000 to 405,000)	2,047.3 (1,835.5 to 2,271.9)	337,000 (313,000 to 368,000)	1,865.3 (1,733.1 to 2,035.5)	-43.9 (-47.1 to -40.0)	-9.4 (-18.1 to 0.9)
	Under 5	163,000 (144,000 to 187,000)	12,136.7 (10,671.0 to 13,865.7)	71,400 (60,900 to 82,900)	4,246.6 (3,619.8 to 4,929.5)	60,500 (48,800 to 75,600)	3,600.4 (2,901.9 to 4,498.1)	50,600 (42,300 to 59,900)	3,046.6 (2,548.9 to 3,604.6)	-65.0 (-68.2 to -61.0)	-15.2 (-29.5 to 1.1)
	5-14 years	60,900 (50,500 to 72,200)	2,415.6 (2,006.1 to 2,866.0)	37,900 (30,700 to 46,300)	1,131.9 (919.0 to 1,383.9)	35,200 (28,500 to 43,400)	1,038.4 (842.8 to 1,282.7)	32,700 (26,500 to 39,800)	959.4 (777.3 to 1,167.0)	-53.1 (-59.6 to -47.2)	-8.3 (-19.4 to 4.5)

Location	Age Group	1990		2019		2020		2021		Percent Incidence Rate Change from 1990-2019 (95% UI)	Percent Incidence Rate Change from 2019-2021 (95% UI)
		Episodes (95% UI)	Rate (95% UI)	Episodes (95% UI)	Rate (95% UI)	Episodes (95% UI)	Rate (95% UI)	Episodes (95% UI)	Rate (95% UI)		
	15-49 years	66,200 (59,900 to 73,000)	1,336.8 (1,209.8 to 1,473.6)	72,500 (66,200 to 79,200)	801.8 (731.8 to 875.9)	68,600 (60,700 to 77,300)	741.4 (656.7 to 835.9)	64,500 (58,400 to 71,100)	686.6 (621.8 to 757.3)	-40.0 (-44.9 to -35.1)	-7.5 (-16.9 to 3.4)
	50-69 years	46,300 (41,900 to 51,100)	5,219.0 (4,720.5 to 5,760.5)	77,800 (69,300 to 86,100)	3,076.2 (2,738.0 to 3,401.1)	74,200 (66,100 to 82,500)	2,844.8 (2,536.7 to 3,164.9)	71,100 (64,600 to 78,600)	2,664.4 (2,422.4 to 2,946.7)	-41.1 (-46.1 to -34.8)	-7.5 (-16.8 to 3.4)
	70+ years	65,000 (58,100 to 71,600)	23,998.9 (21,448.7 to 26,431.2)	136,000 (123,000 to 150,000)	15,176.2 (13,754.1 to 16,757.1)	127,000 (111,000 to 145,000)	13,881.7 (12,147.7 to 15,870.7)	118,000 (105,000 to 132,000)	12,633.6 (11,189.5 to 14,145.4)	-36.8 (-42.0 to -31.4)	-8.5 (-18.7 to 3.2)
**Peru**	All Ages	1,400,000 (1,290,000 to 1,510,000)	6,472.6 (5,959.3 to 6,995.5)	1,280,000 (1,200,000 to 1,370,000)	3,677.1 (3,427.2 to 3,920.7)	1,080,000 (1,000,000 to 1,150,000)	3,012.7 (2,801.2 to 3,213.0)	1,200,000 (1,060,000 to 1,320,000)	3,304.5 (2,933.6 to 3,628.0)	-43.2 (-47.1 to -38.5)	-18.1 (-22.4 to -12.5)
	Under 5	582,000 (502,000 to 680,000)	19,900.4 (17,161.1 to 23,236.0)	175,000 (150,000 to 204,000)	5,430.5 (4,648.4 to 6,316.0)	119,000 (100,000 to 139,000)	3,631.6 (3,057.7 to 4,250.9)	142,000 (110,000 to 176,000)	4,314.0 (3,326.8 to 5,327.1)	-72.7 (-75.6 to -69.4)	-33.1 (-39.5 to -25.8)
	5-14 years	188,000 (154,000 to 230,000)	3,489.6 (2,857.9 to 4,273.6)	95,700 (77,900 to 116,000)	1,590.3 (1,295.6 to 1,928.4)	83,200 (66,300 to 101,000)	1,355.7 (1,080.3 to 1,653.7)	91,400 (74,200 to 112,000)	1,465.8 (1,189.0 to 1,791.7)	-54.4 (-59.9 to -47.8)	-14.8 (-24.3 to -4.2)
	15-49 years	220,000 (197,000 to 240,000)	2,061.4 (1,844.8 to 2,246.4)	236,000 (213,000 to 259,000)	1,270.1 (1,146.5 to 1,394.4)	208,000 (187,000 to 232,000)	1,092.3 (986.0 to 1,218.5)	228,000 (203,000 to 256,000)	1,181.1 (1,050.0 to 1,321.9)	-38.4 (-43.4 to -32.4)	-14.0 (-21.0 to -6.7)
	50-69 years	178,000 (159,000 to 200,000)	8,694.4 (7,795.1 to 9,775.0)	279,000 (246,000 to 308,000)	5,344.8 (4,714.3 to 5,895.2)	247,000 (221,000 to 273,000)	4,594.4 (4,105.5 to 5,080.2)	275,000 (239,000 to 311,000)	4,979.2 (4,323.3 to 5,627.7)	-38.5 (-44.8 to -32.0)	-14.0 (-21.9 to -5.5)
	70+ years	232,000 (209,000 to 260,000)	38,768.3 (34,870.4 to 43,298.7)	497,000 (444,000 to 552,000)	26,918.9 (24,061.0 to 29,926.9)	418,000 (371,000 to 469,000)	22,204.9 (19,685.3 to 24,933.1)	462,000 (396,000 to 526,000)	24,513.3 (21,020.1 to 27,956.4)	-30.6 (-36.1 to -24.9)	-17.5 (-24.3 to -7.9)
**Antigua and Barbuda**	All Ages	1,980 (1,830 to 2,110)	3,278.7 (3,043.9 to 3,509.6)	1,930 (1,800 to 2,050)	2,169.5 (2,028.2 to 2,310.5)	1,790 (1,640 to 1,950)	2,008.4 (1,840.9 to 2,182.9)	1,620 (1,500 to 1,730)	1,806.4 (1,681.4 to 1,939.7)	-33.8 (-36.9 to -30.2)	-7.4 (-14.6 to 1.8)
	Under 5	363 (313 to 422)	5,989.5 (5,161.9 to 6,963.8)	216 (185 to 252)	4,021.0 (3,454.5 to 4,688.0)	181 (148 to 220)	3,401.6 (2,786.5 to 4,143.2)	145 (124 to 170)	2,757.0 (2,360.1 to 3,229.6)	-32.9 (-38.3 to -27.2)	-15.4 (-26.2 to -0.3)
	5-14 years	194 (151 to 251)	1,599.4 (1,246.8 to 2,066.4)	131 (103 to 166)	1,084.9 (856.2 to 1,377.8)	118 (92 to 148)	994.7 (778.0 to 1,248.5)	105 (82 to 133)	900.3 (707.2 to 1,147.3)	-32.2 (-37.6 to -25.3)	-8.3 (-16.5 to 0.7)
	15-49 years	363 (319 to 405)	1,156.7 (1,015.9 to 1,287.9)	371 (335 to 411)	777.8 (702.9 to 860.1)	337 (299 to 378)	709.5 (629.6 to 795.6)	305 (275 to 341)	643.4 (580.1 to 718.6)	-32.8 (-37.9 to -27.3)	-8.8 (-16.6 to 0.0)
	50-69 years	343 (304 to 379)	4,912.1 (4,360.8 to 5,441.1)	552 (498 to 603)	2,976.9 (2,683.4 to 3,250.3)	525 (471 to 584)	2,756.6 (2,471.4 to 3,064.2)	487 (433 to 533)	2,496.0 (2,223.6 to 2,736.6)	-39.4 (-45.3 to -32.9)	-7.4 (-15.3 to 3.2)
	70+ years	712 (631 to 798)	19,562.1 (17,334.0 to 21,922.5)	659 (590 to 730)	12,639.8 (11,318.1 to 14,008.1)	630 (562 to 719)	11,620.2 (10,366.8 to 13,252.8)	573 (500 to 650)	10,207.7 (8,894.2 to 11,578.5)	-35.4 (-40.6 to -29.5)	-8.1 (-17.4 to 2.8)
**Bahamas**	All Ages	6,790 (6,310 to 7,280)	2,644.7 (2,458.4 to 2,835.1)	6,790 (6,370 to 7,220)	1,765.0 (1,656.1 to 1,875.9)	6,050 (5,520 to 6,680)	1,566.2 (1,427.2 to 1,726.9)	5,200 (4,870 to 5,510)	1,339.2 (1,254.5 to 1,421.0)	-33.3 (-37.1 to -29.5)	-11.3 (-17.8 to -2.1)

Location	Age Group	1990		2019		2020		2021		Percent Incidence Rate Change from 1990-2019 (95% UI)	Percent Incidence Rate Change from 2019-2021 (95% UI)
		Episodes (95% UI)	Rate (95% UI)	Episodes (95% UI)	Rate (95% UI)	Episodes (95% UI)	Rate (95% UI)	Episodes (95% UI)	Rate (95% UI)		
	Under 5	1,660 (1,440 to 1,900)	6,488.2 (5,631.5 to 7,466.9)	685 (576 to 804)	3,151.0 (2,650.0 to 3,701.2)	545 (439 to 674)	2,552.3 (2,057.4 to 3,157.4)	414 (348 to 486)	1,964.0 (1,653.0 to 2,309.9)	-51.4 (-56.0 to -47.0)	-19.0 (-30.4 to -4.0)
	5-14 years	881 (689 to 1,130)	1,596.7 (1,249.8 to 2,042.9)	585 (456 to 736)	921.6 (717.9 to 1,159.5)	511 (401 to 640)	824.8 (647.3 to 1,033.3)	435 (348 to 543)	723.9 (578.1 to 903.8)	-42.3 (-46.8 to -37.3)	-10.5 (-17.9 to -1.0)
	15-49 years	1,600 (1,440 to 1,780)	1,120.4 (1,014.4 to 1,249.3)	1,600 (1,460 to 1,760)	772.6 (702.4 to 848.9)	1,420 (1,270 to 1,580)	678.6 (607.0 to 755.5)	1,240 (1,120 to 1,370)	589.1 (535.7 to 651.9)	-31.0 (-36.5 to -24.5)	-12.2 (-19.7 to -3.8)
	50-69 years	1,220 (1,080 to 1,360)	4,750.6 (4,221.0 to 5,312.9)	1,900 (1,690 to 2,100)	2,575.6 (2,293.7 to 2,844.1)	1,750 (1,550 to 1,940)	2,307.5 (2,046.7 to 2,560.4)	1,560 (1,410 to 1,710)	2,005.2 (1,816.3 to 2,201.1)	-45.8 (-51.2 to -40.2)	-10.4 (-19.8 to 0.1)
	70+ years	1,440 (1,280 to 1,650)	17,846.9 (15,779.4 to 20,428.5)	2,020 (1,820 to 2,250)	10,957.8 (9,864.1 to 12,219.9)	1,840 (1,620 to 2,130)	9,622.3 (8,455.1 to 11,146.7)	1,550 (1,410 to 1,720)	8,014.4 (7,269.8 to 8,879.1)	-38.6 (-44.8 to -32.9)	-12.2 (-21.2 to -0.2)
**Barbados**	All Ages	8,890 (8,220 to 9,630)	3,506.4 (3,242.5 to 3,801.3)	9,060 (8,370 to 9,850)	3,050.4 (2,815.4 to 3,315.0)	8,270 (7,520 to 9,150)	2,774.8 (2,522.2 to 3,070.4)	7,280 (6,730 to 7,980)	2,435.0 (2,249.4 to 2,667.4)	-13.0 (-18.7 to -6.1)	-9.0 (-17.1 to 1.3)
	Under 5	1,300 (1,100 to 1,500)	6,611.7 (5,645.0 to 7,652.3)	578 (490 to 676)	4,104.7 (3,476.0 to 4,799.5)	466 (373 to 584)	3,365.0 (2,694.4 to 4,220.4)	354 (296 to 417)	2,599.7 (2,174.3 to 3,060.6)	-37.9 (-43.5 to -32.1)	-18.0 (-30.1 to -1.6)
	5-14 years	751 (586 to 941)	1,754.9 (1,371.0 to 2,199.8)	426 (328 to 541)	1,217.8 (937.9 to 1,548.1)	373 (289 to 476)	1,090.9 (843.6 to 1,389.2)	325 (253 to 416)	970.8 (755.3 to 1,241.6)	-30.6 (-36.5 to -24.3)	-10.4 (-19.3 to -1.2)
	15-49 years	1,530 (1,360 to 1,720)	1,139.4 (1,009.5 to 1,274.3)	1,170 (1,040 to 1,300)	833.1 (745.6 to 925.4)	1,050 (931 to 1,180)	750.3 (665.3 to 844.7)	925 (837 to 1,040)	662.0 (598.7 to 740.4)	-26.9 (-32.1 to -20.8)	-9.9 (-17.4 to 0.1)
	50-69 years	1,610 (1,440 to 1,790)	4,607.9 (4,116.3 to 5,120.6)	2,500 (2,230 to 2,780)	3,221.2 (2,871.8 to 3,575.3)	2,350 (2,080 to 2,630)	2,971.0 (2,631.0 to 3,329.4)	2,110 (1,880 to 2,340)	2,630.8 (2,349.5 to 2,920.6)	-30.1 (-36.9 to -22.7)	-7.8 (-16.9 to 3.8)
	70+ years	3,690 (3,270 to 4,250)	17,277.9 (15,307.1 to 19,894.4)	4,390 (3,860 to 4,960)	14,519.4 (12,785.6 to 16,414.8)	4,040 (3,570 to 4,660)	12,994.4 (11,484.5 to 14,994.4)	3,570 (3,150 to 4,100)	11,135.4 (9,826.4 to 12,796.9)	-16.0 (-24.3 to -6.2)	-10.5 (-20.0 to 0.8)
**Belize**	All Ages	5,840 (5,360 to 6,330)	3,125.8 (2,868.0 to 3,385.2)	7,720 (7,220 to 8,250)	1,871.9 (1,750.1 to 1,999.9)	7,250 (6,650 to 7,890)	1,722.2 (1,580.0 to 1,873.9)	6,640 (6,230 to 7,100)	1,547.4 (1,451.0 to 1,653.8)	-40.1 (-43.9 to -36.0)	-8.0 (-14.0 to -0.3)
	Under 5	2,230 (1,920 to 2,570)	7,550.4 (6,511.5 to 8,703.8)	1,260 (1,080 to 1,470)	3,310.9 (2,841.5 to 3,853.7)	1,090 (907 to 1,300)	2,846.3 (2,376.6 to 3,396.6)	903 (770 to 1,060)	2,369.2 (2,019.6 to 2,776.1)	-56.1 (-60.4 to -51.4)	-14.0 (-24.0 to -0.2)
	5-14 years	909 (719 to 1,150)	1,738.1 (1,373.5 to 2,205.7)	840 (672 to 1,030)	981.7 (785.1 to 1,206.5)	775 (615 to 943)	908.9 (720.7 to 1,106.1)	704 (563 to 870)	828.7 (662.8 to 1,024.1)	-43.5 (-49.0 to -37.2)	-7.4 (-16.2 to 1.7)
	15-49 years	881 (790 to 986)	1,035.3 (927.6 to 1,158.9)	2,000 (1,820 to 2,200)	885.4 (809.0 to 973.1)	1,880 (1,670 to 2,080)	810.5 (721.5 to 899.0)	1,760 (1,580 to 1,950)	740.9 (668.1 to 822.9)	-14.5 (-22.6 to -6.8)	-8.5 (-16.0 to 0.8)
	50-69 years	698 (617 to 772)	4,755.7 (4,205.5 to 5,262.3)	1,710 (1,530 to 1,910)	3,400.4 (3,033.0 to 3,784.1)	1,670 (1,460 to 1,860)	3,164.0 (2,780.1 to 3,541.5)	1,580 (1,410 to 1,730)	2,884.6 (2,570.4 to 3,158.5)	-28.5 (-36.0 to -20.8)	-7.0 (-15.9 to 3.7)

Location	Age Group	1990		2019		2020		2021		Percent Incidence Rate Change from 1990-2019 (95% UI)	Percent Incidence Rate Change from 2019-2021 (95% UI)
		Episodes (95% UI)	Rate (95% UI)	Episodes (95% UI)	Rate (95% UI)	Episodes (95% UI)	Rate (95% UI)	Episodes (95% UI)	Rate (95% UI)		
	70+ years	1,120 (1,020 to 1,250)	21,331.3 (19,268.6 to 23,725.6)	1,910 (1,720 to 2,130)	14,869.9 (13,416.4 to 16,635.3)	1,850 (1,640 to 2,150)	13,772.2 (12,266.4 to 16,039.6)	1,690 (1,540 to 1,920)	12,114.3 (11,021.8 to 13,748.5)	-30.3 (-36.1 to -23.2)	-7.4 (-16.3 to 2.6)
**Bermuda**	All Ages	1,570 (1,450 to 1,690)	2,642.1 (2,443.3 to 2,844.9)	1,460 (1,360 to 1,590)	2,283.0 (2,119.0 to 2,479.9)	1,400 (1,290 to 1,530)	2,191.0 (2,025.3 to 2,392.9)	1,280 (1,190 to 1,390)	2,021.9 (1,877.6 to 2,192.1)	-13.6 (-18.5 to -7.7)	-4.0 (-10.3 to 3.3)
	Under 5	246 (207 to 291)	5,715.8 (4,813.0 to 6,780.3)	91 (76 to 108)	3,384.6 (2,831.8 to 4,017.0)	79 (66 to 94)	3,003.2 (2,501.2 to 3,597.4)	67 (56 to 79)	2,617.2 (2,176.2 to 3,107.4)	-40.8 (-46.1 to -35.6)	-11.3 (-21.1 to 0.3)
	5-14 years	125 (94 to 163)	1,636.7 (1,231.2 to 2,131.5)	73 (55 to 96)	1,171.2 (883.2 to 1,541.3)	66 (50 to 86)	1,087.3 (826.2 to 1,415.6)	60 (45 to 77)	1,014.9 (768.8 to 1,312.2)	-28.4 (-35.0 to -22.4)	-7.2 (-14.0 to 0.8)
	15-49 years	319 (280 to 361)	943.0 (828.6 to 1,066.8)	170 (149 to 196)	612.7 (536.3 to 706.9)	155 (136 to 177)	569.3 (499.5 to 648.9)	141 (123 to 161)	525.7 (459.9 to 599.9)	-35.0 (-39.9 to -28.9)	-7.1 (-13.6 to 0.8)
	50-69 years	371 (331 to 416)	3,650.9 (3,259.0 to 4,088.1)	362 (322 to 400)	1,901.4 (1,690.2 to 2,100.1)	348 (313 to 391)	1,812.2 (1,628.8 to 2,035.1)	319 (285 to 354)	1,656.5 (1,483.0 to 1,840.6)	-47.9 (-52.0 to -42.8)	-4.7 (-12.7 to 4.7)
	70+ years	508 (448 to 585)	14,631.9 (12,908.5 to 16,833.0)	769 (689 to 877)	9,058.2 (8,125.5 to 10,342.0)	752 (677 to 859)	8,612.9 (7,754.7 to 9,839.7)	698 (627 to 778)	7,750.3 (6,954.9 to 8,641.1)	-38.1 (-44.2 to -31.6)	-4.9 (-13.6 to 4.0)
**Cuba**	All Ages	339,000 (313,000 to 364,000)	3,128.7 (2,882.9 to 3,355.6)	378,000 (348,000 to 410,000)	3,331.2 (3,061.9 to 3,608.0)	348,000 (317,000 to 382,000)	3,070.1 (2,794.2 to 3,367.2)	306,000 (280,000 to 332,000)	2,712.2 (2,483.3 to 2,949.7)	6.5 (-0.7 to 14.5)	-7.8 (-15.3 to 1.8)
	Under 5	59,200 (50,400 to 69,400)	6,614.0 (5,633.7 to 7,759.1)	20,000 (16,800 to 23,500)	3,456.0 (2,899.1 to 4,051.0)	15,600 (12,600 to 19,100)	2,768.1 (2,245.5 to 3,398.1)	11,900 (10,200 to 14,100)	2,188.0 (1,866.1 to 2,583.6)	-47.7 (-54.1 to -40.8)	-19.9 (-29.4 to -6.1)
	5-14 years	24,500 (19,200 to 31,000)	1,525.4 (1,192.7 to 1,926.5)	14,900 (11,200 to 19,300)	1,213.4 (908.9 to 1,569.3)	13,400 (9,960 to 17,400)	1,090.3 (810.2 to 1,417.5)	12,000 (9,060 to 15,200)	971.7 (735.3 to 1,233.9)	-20.5 (-28.2 to -12.3)	-10.1 (-17.9 to -0.6)
	15-49 years	62,800 (55,600 to 70,500)	1,021.0 (904.4 to 1,146.2)	43,200 (39,000 to 48,100)	814.8 (735.1 to 906.5)	38,200 (34,100 to 43,300)	735.8 (656.8 to 832.6)	33,300 (30,000 to 36,700)	655.1 (589.5 to 722.3)	-20.2 (-26.8 to -13.8)	-9.7 (-16.9 to -1.2)
	50-69 years	73,900 (66,000 to 82,700)	4,721.1 (4,217.5 to 5,287.3)	104,000 (92,000 to 113,000)	3,406.5 (3,023.0 to 3,728.5)	97,000 (85,600 to 109,000)	3,124.8 (2,757.0 to 3,498.0)	88,700 (79,100 to 97,700)	2,799.9 (2,495.9 to 3,084.7)	-27.8 (-34.4 to -19.1)	-8.3 (-17.5 to 2.6)
	70+ years	119,000 (102,000 to 131,000)	19,007.3 (16,306.2 to 20,899.9)	197,000 (175,000 to 221,000)	16,250.8 (14,461.1 to 18,219.7)	184,000 (164,000 to 206,000)	14,816.2 (13,248.2 to 16,643.3)	160,000 (143,000 to 180,000)	12,865.6 (11,478.1 to 14,532.5)	-14.5 (-22.6 to -3.2)	-8.8 (-18.2 to 2.0)
**Dominica**	All Ages	2,000 (1,860 to 2,150)	2,762.4 (2,571.8 to 2,966.7)	1,460 (1,360 to 1,560)	2,164.4 (2,015.0 to 2,315.1)	1,340 (1,230 to 1,450)	1,992.7 (1,828.9 to 2,157.9)	1,210 (1,120 to 1,300)	1,798.3 (1,664.7 to 1,940.4)	-21.6 (-25.9 to -16.5)	-7.9 (-14.3 to 0.1)
	Under 5	432 (371 to 501)	4,991.8 (4,287.1 to 5,786.4)	143 (124 to 165)	3,768.3 (3,266.5 to 4,352.9)	117 (99 to 141)	3,236.6 (2,742.7 to 3,889.5)	94 (80 to 110)	2,696.8 (2,312.2 to 3,156.4)	-24.5 (-30.1 to -18.2)	-14.1 (-23.8 to -0.7)
	5-14 years	250 (192 to 318)	1,544.6 (1,189.1 to 1,969.9)	115 (90 to 145)	1,065.7 (830.6 to 1,335.3)	103 (82 to 130)	982.0 (774.2 to 1,235.9)	91 (71 to 113)	889.9 (696.5 to 1,111.8)	-31.0 (-37.7 to -25.3)	-7.8 (-16.7 to 2.5)

Location	Age Group	1990		2019		2020		2021		Percent Incidence Rate Change from 1990-2019 (95% UI)	Percent Incidence Rate Change from 2019-2021 (95% UI)
		Episodes (95% UI)	Rate (95% UI)	Episodes (95% UI)	Rate (95% UI)	Episodes (95% UI)	Rate (95% UI)	Episodes (95% UI)	Rate (95% UI)		
	15-49 years	357 (320 to 398)	1,010.4 (904.1 to 1,125.5)	263 (237 to 294)	776.2 (698.4 to 867.0)	238 (212 to 268)	702.9 (625.7 to 791.9)	217 (195 to 242)	642.5 (577.2 to 717.4)	-23.2 (-29.3 to -16.2)	-9.4 (-18.0 to 0.1)
	50-69 years	352 (316 to 389)	4,194.4 (3,763.1 to 4,632.9)	414 (369 to 457)	2,854.9 (2,544.1 to 3,150.1)	394 (351 to 439)	2,656.4 (2,365.1 to 2,955.9)	366 (324 to 405)	2,422.7 (2,145.1 to 2,682.5)	-31.9 (-38.2 to -24.8)	-7.0 (-14.9 to 2.2)
	70+ years	610 (541 to 695)	15,808.0 (14,020.5 to 18,017.4)	525 (463 to 605)	11,863.4 (10,447.5 to 13,660.0)	489 (426 to 563)	10,934.9 (9,519.9 to 12,583.5)	439 (387 to 502)	9,724.4 (8,583.6 to 11,113.2)	-25.0 (-32.4 to -16.4)	-7.8 (-16.9 to 2.3)
**Dominican Republic**	All Ages	237,000 (219,000 to 261,000)	3,312.9 (3,060.3 to 3,646.5)	233,000 (217,000 to 248,000)	2,161.1 (2,011.6 to 2,303.0)	213,000 (194,000 to 232,000)	1,956.6 (1,781.8 to 2,128.7)	191,000 (178,000 to 205,000)	1,738.4 (1,612.8 to 1,858.0)	-34.8 (-39.3 to -30.3)	-9.5 (-16.3 to -0.9)
	Under 5	107,000 (93,000 to 125,000)	10,717.1 (9,337.9 to 12,506.0)	39,300 (33,600 to 45,600)	3,841.8 (3,284.7 to 4,454.5)	31,400 (26,100 to 37,900)	3,046.6 (2,535.8 to 3,680.1)	25,900 (22,000 to 30,200)	2,505.2 (2,133.7 to 2,926.1)	-64.2 (-67.7 to -60.0)	-20.7 (-30.0 to -9.3)
	5-14 years	39,000 (31,300 to 48,000)	2,295.5 (1,839.0 to 2,821.3)	25,500 (20,400 to 31,500)	1,351.3 (1,083.0 to 1,671.2)	23,100 (18,500 to 28,600)	1,218.3 (978.4 to 1,509.2)	21,200 (17,000 to 26,300)	1,114.4 (892.4 to 1,379.8)	-41.1 (-47.5 to -34.5)	-9.8 (-18.0 to -1.0)
	15-49 years	38,900 (34,400 to 44,100)	1,069.8 (945.9 to 1,211.7)	48,600 (43,500 to 54,400)	842.4 (755.0 to 943.9)	44,600 (39,500 to 50,300)	767.5 (678.4 to 864.3)	41,000 (36,500 to 45,700)	698.7 (623.4 to 778.8)	-21.3 (-27.4 to -14.1)	-8.9 (-17.0 to -0.8)
	50-69 years	23,800 (21,400 to 26,800)	3,745.8 (3,374.2 to 4,213.1)	49,200 (43,700 to 55,500)	3,121.3 (2,769.9 to 3,521.0)	47,200 (41,600 to 52,800)	2,911.5 (2,566.0 to 3,257.5)	43,100 (38,500 to 48,400)	2,601.2 (2,324.7 to 2,918.9)	-16.7 (-24.5 to -8.0)	-6.7 (-17.0 to 4.2)
	70+ years	28,500 (25,200 to 32,700)	15,440.5 (13,657.6 to 17,729.7)	70,400 (62,500 to 79,100)	13,292.9 (11,789.1 to 14,927.9)	67,000 (59,200 to 76,700)	12,376.6 (10,937.3 to 14,161.1)	60,300 (52,600 to 68,500)	10,858.0 (9,471.0 to 12,345.2)	-13.9 (-21.6 to -2.9)	-6.9 (-17.6 to 6.4)
**Grenada**	All Ages	3,880 (3,630 to 4,170)	4,459.9 (4,171.5 to 4,793.9)	2,520 (2,350 to 2,700)	2,430.5 (2,270.4 to 2,606.5)	2,320 (2,110 to 2,550)	2,247.4 (2,049.4 to 2,471.0)	2,060 (1,920 to 2,190)	2,005.1 (1,869.8 to 2,137.4)	-45.5 (-48.5 to -41.8)	-7.5 (-13.7 to 1.1)
	Under 5	1,020 (879 to 1,190)	8,490.6 (7,320.0 to 9,888.8)	308 (267 to 357)	4,260.1 (3,690.9 to 4,931.5)	256 (213 to 307)	3,626.1 (3,026.9 to 4,352.2)	203 (175 to 239)	2,961.8 (2,550.4 to 3,486.6)	-49.8 (-54.7 to -45.1)	-14.9 (-25.4 to -0.6)
	5-14 years	426 (341 to 544)	1,992.6 (1,592.3 to 2,540.1)	173 (139 to 214)	1,116.9 (894.0 to 1,380.8)	156 (121 to 192)	1,021.1 (791.9 to 1,262.5)	138 (110 to 173)	925.3 (733.7 to 1,157.6)	-43.9 (-49.4 to -38.1)	-8.6 (-18.3 to 1.6)
	15-49 years	591 (531 to 653)	1,509.4 (1,355.9 to 1,669.6)	530 (480 to 587)	978.4 (885.9 to 1,083.0)	479 (421 to 539)	890.2 (783.5 to 1,001.1)	430 (386 to 475)	805.6 (723.8 to 889.4)	-35.2 (-40.4 to -29.0)	-9.0 (-15.7 to -0.5)
	50-69 years	591 (526 to 673)	6,227.8 (5,541.4 to 7,081.3)	738 (665 to 819)	3,548.5 (3,201.4 to 3,939.2)	702 (628 to 793)	3,310.8 (2,958.8 to 3,739.3)	639 (580 to 708)	2,972.5 (2,695.5 to 3,290.8)	-43.0 (-48.4 to -36.1)	-6.7 (-14.7 to 3.5)
	70+ years	1,250 (1,110 to 1,440)	25,176.2 (22,367.9 to 28,965.5)	767 (677 to 868)	13,286.3 (11,734.7 to 15,033.6)	726 (636 to 827)	12,404.3 (10,861.6 to 14,138.3)	647 (583 to 736)	10,937.4 (9,859.0 to 12,456.1)	-47.2 (-52.7 to -40.4)	-6.6 (-15.5 to 6.7)
**Guyana**	All Ages	21,600 (20,100 to 23,100)	2,764.7 (2,581.5 to 2,961.0)	14,700 (13,800 to 15,700)	1,918.7 (1,797.9 to 2,044.2)	13,100 (11,900 to 14,500)	1,708.9 (1,545.0 to 1,882.3)	11,400 (10,600 to 12,100)	1,484.5 (1,391.1 to 1,579.4)	-30.6 (-33.9 to -26.7)	-10.9 (-18.0 to -1.3)

Location	Age Group	1990		2019		2020		2021		Percent Incidence Rate Change from 1990-2019 (95% UI)	Percent Incidence Rate Change from 2019-2021 (95% UI)
		Episodes (95% UI)	Rate (95% UI)	Episodes (95% UI)	Rate (95% UI)	Episodes (95% UI)	Rate (95% UI)	Episodes (95% UI)	Rate (95% UI)		
	Under 5	7,150 (6,220 to 8,170)	6,337.8 (5,516.4 to 7,241.0)	2,650 (2,290 to 3,060)	3,474.0 (3,001.2 to 4,015.3)	2,120 (1,730 to 2,570)	2,790.7 (2,278.7 to 3,387.1)	1,660 (1,430 to 1,930)	2,231.0 (1,924.5 to 2,595.1)	-45.2 (-49.4 to -40.6)	-19.7 (-30.5 to -5.4)
	5-14 years	2,850 (2,240 to 3,560)	1,574.8 (1,236.2 to 1,964.6)	1,430 (1,140 to 1,780)	1,028.7 (818.1 to 1,281.7)	1,280 (1,020 to 1,610)	919.0 (736.2 to 1,155.7)	1,140 (909 to 1,410)	822.3 (654.4 to 1,018.2)	-34.7 (-40.3 to -27.6)	-10.7 (-20.0 to -0.1)
	15-49 years	4,570 (4,080 to 5,120)	1,134.5 (1,011.3 to 1,270.3)	3,420 (3,070 to 3,800)	847.5 (760.7 to 939.2)	3,070 (2,710 to 3,470)	759.6 (671.4 to 858.9)	2,710 (2,440 to 2,990)	677.0 (611.6 to 747.0)	-25.3 (-31.6 to -17.8)	-10.4 (-19.3 to 1.3)
	50-69 years	3,400 (3,050 to 3,780)	5,211.8 (4,675.6 to 5,795.5)	3,760 (3,370 to 4,180)	3,146.8 (2,819.5 to 3,494.0)	3,500 (3,040 to 3,950)	2,882.9 (2,505.8 to 3,257.2)	3,120 (2,800 to 3,430)	2,548.7 (2,284.2 to 2,800.7)	-39.6 (-44.8 to -34.1)	-8.4 (-17.0 to 3.9)
	70+ years	3,580 (3,220 to 4,070)	20,416.9 (18,359.5 to 23,200.3)	3,440 (3,070 to 3,880)	12,482.9 (11,154.2 to 14,085.2)	3,170 (2,790 to 3,660)	11,149.2 (9,796.8 to 12,875.5)	2,720 (2,400 to 3,050)	9,390.9 (8,280.4 to 10,531.0)	-38.9 (-44.6 to -33.1)	-10.7 (-20.8 to 1.4)
**Haiti**	All Ages	374,000 (339,000 to 407,000)	5,863.4 (5,319.9 to 6,385.1)	337,000 (313,000 to 363,000)	2,720.7 (2,528.4 to 2,932.3)	316,000 (289,000 to 343,000)	2,502.0 (2,288.0 to 2,718.9)	291,000 (270,000 to 313,000)	2,258.7 (2,099.5 to 2,433.5)	-53.6 (-56.0 to -51.0)	-8.0 (-13.8 to -1.6)
	Under 5	202,000 (174,000 to 233,000)	19,023.2 (16,443.1 to 21,967.7)	121,000 (105,000 to 140,000)	7,842.9 (6,804.4 to 9,051.5)	110,000 (93,800 to 127,000)	7,053.0 (6,024.3 to 8,172.0)	98,200 (85,900 to 112,000)	6,253.3 (5,473.7 to 7,119.9)	-58.8 (-62.0 to -55.0)	-10.1 (-18.8 to 0.5)
	5-14 years	46,500 (36,200 to 59,400)	2,814.3 (2,193.6 to 3,592.4)	42,300 (33,800 to 53,800)	1,571.2 (1,255.7 to 1,995.9)	40,200 (32,500 to 50,000)	1,467.0 (1,186.1 to 1,826.1)	37,900 (31,100 to 46,500)	1,360.2 (1,116.8 to 1,671.1)	-44.2 (-51.2 to -36.0)	-6.6 (-16.7 to 5.0)
	15-49 years	48,600 (43,400 to 54,800)	1,657.1 (1,479.9 to 1,868.4)	68,700 (61,400 to 76,500)	1,044.2 (933.2 to 1,162.6)	65,200 (57,400 to 73,100)	970.2 (854.6 to 1,088.5)	62,100 (55,500 to 69,000)	906.5 (810.1 to 1,006.2)	-37.0 (-42.1 to -31.0)	-7.1 (-14.6 to 1.5)
	50-69 years	44,800 (39,500 to 50,600)	7,450.7 (6,572.4 to 8,414.3)	57,700 (51,600 to 64,700)	4,446.7 (3,979.8 to 4,984.7)	56,100 (49,800 to 63,300)	4,186.7 (3,712.7 to 4,721.6)	52,300 (45,900 to 59,100)	3,809.5 (3,344.1 to 4,305.6)	-40.3 (-45.7 to -34.5)	-5.8 (-14.2 to 3.7)
	70+ years	32,400 (28,300 to 36,900)	24,537.9 (21,423.2 to 27,889.7)	47,400 (41,600 to 53,500)	17,220.9 (15,120.4 to 19,453.3)	44,800 (39,400 to 50,700)	16,146.5 (14,182.0 to 18,280.0)	40,100 (35,100 to 45,100)	14,123.7 (12,346.1 to 15,885.7)	-29.8 (-37.2 to -20.9)	-6.2 (-15.3 to 2.9)
**Jamaica**	All Ages	73,300 (68,200 to 79,600)	3,099.6 (2,883.1 to 3,363.0)	52,900 (49,100 to 56,800)	1,890.2 (1,755.7 to 2,030.9)	48,600 (44,700 to 53,200)	1,734.3 (1,597.6 to 1,898.2)	43,000 (40,300 to 46,200)	1,535.7 (1,438.9 to 1,649.1)	-39.0 (-42.7 to -34.8)	-8.2 (-15.0 to 0.0)
	Under 5	21,300 (18,200 to 24,800)	7,618.4 (6,511.8 to 8,883.5)	6,000 (5,090 to 7,060)	3,357.9 (2,848.0 to 3,951.8)	4,970 (4,070 to 6,160)	2,842.6 (2,328.9 to 3,525.7)	3,940 (3,300 to 4,610)	2,300.4 (1,930.8 to 2,694.1)	-55.9 (-61.5 to -49.5)	-15.3 (-25.6 to -1.1)
	5-14 years	11,600 (9,310 to 14,600)	2,093.6 (1,673.9 to 2,629.9)	5,100 (3,870 to 6,450)	1,189.3 (901.3 to 1,504.1)	4,600 (3,560 to 5,900)	1,092.2 (846.0 to 1,400.2)	4,100 (3,140 to 5,200)	994.0 (759.9 to 1,258.8)	-43.2 (-50.4 to -36.2)	-8.2 (-15.7 to 0.4)
	15-49 years	11,400 (10,000 to 13,000)	980.3 (860.7 to 1,114.5)	10,800 (9,570 to 12,100)	703.2 (625.2 to 791.8)	9,820 (8,700 to 11,200)	641.3 (568.4 to 730.2)	8,920 (7,880 to 10,100)	582.6 (514.8 to 661.0)	-28.3 (-33.1 to -22.3)	-8.8 (-16.2 to -0.2)
	50-69 years	9,970 (8,810 to 11,100)	3,990.5 (3,526.8 to 4,442.3)	11,700 (10,400 to 13,000)	2,419.7 (2,158.2 to 2,681.5)	11,200 (9,990 to 12,400)	2,252.3 (2,018.0 to 2,512.4)	10,100 (9,140 to 11,200)	2,012.4 (1,815.8 to 2,225.6)	-39.4 (-44.9 to -33.1)	-6.9 (-16.7 to 2.7)

Location	Age Group	1990		2019		2020		2021		Percent Incidence Rate Change from 1990-2019 (95% UI)	Percent Incidence Rate Change from 2019-2021 (95% UI)
		Episodes (95% UI)	Rate (95% UI)	Episodes (95% UI)	Rate (95% UI)	Episodes (95% UI)	Rate (95% UI)	Episodes (95% UI)	Rate (95% UI)		
	70+ years	19,000 (17,000 to 21,300)	16,365.2 (14,603.7 to 18,331.2)	19,300 (16,600 to 22,000)	10,911.9 (9,378.8 to 12,442.6)	18,000 (15,900 to 20,800)	10,082.6 (8,884.3 to 11,618.9)	15,900 (14,500 to 17,800)	8,745.7 (7,948.3 to 9,809.7)	-33.3 (-39.1 to -24.6)	-7.6 (-17.6 to 4.5)
**Puerto Rico**	All Ages	126,000 (116,000 to 136,000)	3,485.9 (3,220.8 to 3,756.5)	93,900 (87,000 to 102,000)	2,778.4 (2,573.7 to 3,010.1)	85,400 (77,300 to 95,600)	2,559.7 (2,314.6 to 2,864.1)	73,100 (67,700 to 79,700)	2,217.8 (2,054.3 to 2,418.6)	-20.3 (-25.6 to -14.6)	-7.9 (-15.7 to 2.6)
	Under 5	18,800 (16,000 to 22,100)	5,878.1 (5,020.5 to 6,908.5)	3,990 (3,250 to 4,870)	3,237.3 (2,638.2 to 3,949.2)	2,980 (2,370 to 3,780)	2,623.6 (2,083.2 to 3,319.2)	2,080 (1,690 to 2,520)	1,976.8 (1,607.2 to 2,402.1)	-44.9 (-51.3 to -38.4)	-19.0 (-31.2 to -1.4)
	5-14 years	11,100 (8,460 to 14,200)	1,634.5 (1,250.6 to 2,103.8)	4,410 (3,280 to 5,760)	1,176.6 (874.1 to 1,535.2)	3,750 (2,840 to 4,880)	1,049.3 (794.4 to 1,366.4)	3,130 (2,340 to 4,090)	922.2 (690.2 to 1,204.6)	-28.0 (-34.1 to -21.9)	-10.8 (-19.7 to -1.3)
	15-49 years	26,800 (24,300 to 29,600)	1,450.6 (1,315.2 to 1,607.0)	10,800 (9,680 to 12,000)	711.8 (639.6 to 796.2)	9,560 (8,410 to 10,800)	641.8 (564.7 to 723.8)	8,220 (7,380 to 9,120)	560.8 (504.1 to 622.6)	-50.9 (-55.3 to -46.3)	-9.8 (-18.4 to -0.4)
	50-69 years	27,100 (24,500 to 29,900)	4,942.3 (4,475.0 to 5,456.5)	23,100 (20,900 to 25,800)	2,661.4 (2,400.3 to 2,967.8)	21,000 (18,500 to 23,800)	2,427.4 (2,142.4 to 2,748.2)	18,100 (16,200 to 19,900)	2,104.3 (1,886.7 to 2,322.2)	-46.2 (-51.1 to -41.5)	-8.8 (-17.1 to 2.2)
	70+ years	42,200 (36,800 to 47,400)	18,900.7 (16,452.1 to 21,221.1)	51,600 (45,900 to 58,300)	10,324.1 (9,183.9 to 11,664.7)	48,200 (42,100 to 55,800)	9,390.2 (8,217.5 to 10,879.5)	41,600 (37,200 to 47,000)	7,893.8 (7,068.5 to 8,923.1)	-45.4 (-51.0 to -39.5)	-9.0 (-18.9 to 4.3)
**Saint Kitts and Nevis**	All Ages	1,570 (1,460 to 1,700)	3,794.4 (3,523.7 to 4,104.1)	1,100 (1,030 to 1,180)	1,902.2 (1,784.4 to 2,035.3)	1,030 (942 to 1,130)	1,771.9 (1,615.4 to 1,943.7)	928 (872 to 991)	1,582.7 (1,487.6 to 1,689.5)	-49.9 (-53.1 to -46.0)	-6.9 (-14.1 to 1.7)
	Under 5	348 (301 to 407)	7,435.8 (6,425.9 to 8,681.9)	109 (92 to 125)	3,438.9 (2,921.9 to 3,970.2)	90 (74 to 111)	2,899.1 (2,384.4 to 3,559.7)	71 (60 to 82)	2,326.1 (1,979.6 to 2,696.0)	-53.8 (-58.3 to -48.5)	-15.7 (-26.1 to -0.6)
	5-14 years	165 (131 to 205)	1,746.6 (1,390.7 to 2,173.0)	71 (56 to 87)	999.6 (797.3 to 1,234.6)	64 (51 to 80)	925.5 (735.4 to 1,165.8)	57 (45 to 71)	842.5 (668.5 to 1,048.3)	-42.8 (-47.4 to -37.6)	-7.4 (-15.8 to 2.1)
	15-49 years	252 (224 to 279)	1,266.0 (1,125.3 to 1,404.7)	217 (193 to 246)	686.6 (611.3 to 777.4)	198 (175 to 224)	626.9 (553.5 to 708.6)	179 (159 to 202)	566.3 (502.7 to 640.3)	-45.8 (-51.2 to -39.2)	-8.7 (-16.5 to 0.2)
	50-69 years	288 (253 to 320)	6,055.4 (5,331.5 to 6,741.3)	405 (362 to 449)	2,985.6 (2,672.2 to 3,313.1)	397 (351 to 451)	2,829.2 (2,497.2 to 3,214.0)	366 (328 to 407)	2,537.3 (2,273.8 to 2,817.4)	-50.7 (-55.5 to -44.7)	-5.2 (-15.0 to 4.9)
	70+ years	521 (456 to 593)	19,265.9 (16,866.6 to 21,929.1)	302 (274 to 338)	11,628.3 (10,563.1 to 13,001.6)	284 (252 to 322)	10,701.1 (9,501.3 to 12,128.8)	255 (230 to 285)	9,174.0 (8,291.3 to 10,265.7)	-39.6 (-47.0 to -32.6)	-8.0 (-17.0 to 2.5)
**Saint Lucia**	All Ages	4,120 (3,810 to 4,490)	3,020.1 (2,791.9 to 3,285.7)	4,060 (3,810 to 4,300)	2,294.4 (2,153.5 to 2,433.1)	3,730 (3,430 to 4,060)	2,105.0 (1,932.0 to 2,288.7)	3,320 (3,100 to 3,540)	1,869.1 (1,747.8 to 1,992.4)	-24.0 (-29.3 to -18.0)	-8.3 (-14.3 to -0.1)
	Under 5	1,220 (1,040 to 1,420)	6,938.5 (5,917.4 to 8,071.0)	322 (275 to 375)	3,484.9 (2,970.5 to 4,053.1)	264 (217 to 319)	2,922.6 (2,403.1 to 3,528.8)	210 (178 to 246)	2,373.0 (2,018.3 to 2,783.1)	-49.8 (-53.8 to -44.2)	-16.1 (-26.5 to -1.1)
	5-14 years	592 (453 to 750)	1,747.3 (1,337.6 to 2,212.0)	236 (188 to 296)	1,081.8 (860.0 to 1,356.6)	212 (166 to 268)	992.9 (779.4 to 1,256.0)	187 (148 to 232)	895.4 (708.4 to 1,114.7)	-38.1 (-43.3 to -31.8)	-8.2 (-16.1 to 0.4)

Location	Age Group	1990		2019		2020		2021		Percent Incidence Rate Change from 1990-2019 (95% UI)	Percent Incidence Rate Change from 2019-2021 (95% UI)
		Episodes (95% UI)	Rate (95% UI)	Episodes (95% UI)	Rate (95% UI)	Episodes (95% UI)	Rate (95% UI)	Episodes (95% UI)	Rate (95% UI)		
	15-49 years	739<br>(662 to 832)	1,109.4<br>(994.7 to 1,250.0)	769<br>(699 to 847)	821.9<br>(747.0 to 905.1)	694<br>(624 to 779)	745.3<br>(670.7 to 837.2)	617<br>(557 to 687)	667.6<br>(601.7 to 742.5)	-25.9<br>(-32.1 to -19.3)	-9.3<br>(-17.2 to -1.4)
	50-69 years	654<br>(580 to 730)	4,949.5<br>(4,392.9 to 5,522.6)	1,100<br>(994 to 1,220)	2,810.4<br>(2,545.2 to 3,115.6)	1,050<br>(924 to 1,180)	2,597.6<br>(2,279.2 to 2,898.5)	967<br>(856 to 1,080)	2,317.3<br>(2,049.8 to 2,584.2)	-43.2<br>(-48.9 to -37.6)	-7.6<br>(-16.0 to 3.5)
	70+ years	915<br>(803 to 1,060)	17,497.3<br>(15,363.0 to 20,330.9)	1,630<br>(1,450 to 1,800)	12,468.1<br>(11,094.5 to 13,776.1)	1,510<br>(1,350 to 1,710)	11,316.3<br>(10,142.3 to 12,794.3)	1,340<br>(1,200 to 1,490)	9,839.5<br>(8,827.4 to 11,001.0)	-28.7<br>(-36.9 to -20.6)	-9.2<br>(-17.1 to 1.3)
**Saint Vincent and the Grenadines**	All Ages	3,150<br>(2,900 to 3,410)	2,880.2<br>(2,651.6 to 3,116.1)	2,710<br>(2,560 to 2,880)	2,371.6<br>(2,238.4 to 2,527.3)	2,490<br>(2,290 to 2,720)	2,184.8<br>(2,005.5 to 2,381.4)	2,240<br>(2,080 to 2,400)	1,962.2<br>(1,825.5 to 2,105.2)	-17.7<br>(-22.8 to -12.3)	-7.9<br>(-14.7 to 0.0)
	Under 5	895<br>(774 to 1,040)	7,002.8<br>(6,057.6 to 8,145.9)	277<br>(235 to 326)	3,500.7<br>(2,967.6 to 4,119.9)	225<br>(185 to 273)	2,981.9<br>(2,445.2 to 3,615.3)	177<br>(150 to 207)	2,453.6<br>(2,080.4 to 2,878.8)	-50.0<br>(-54.5 to -45.9)	-14.8<br>(-24.7 to -1.0)
	5-14 years	477<br>(364 to 607)	1,683.7<br>(1,285.8 to 2,144.5)	199<br>(156 to 251)	1,101.8<br>(864.0 to 1,388.5)	182<br>(144 to 227)	1,014.9<br>(801.6 to 1,267.2)	164<br>(129 to 208)	923.3<br>(727.9 to 1,170.2)	-34.6<br>(-41.4 to -27.6)	-7.9<br>(-15.4 to 1.9)
	15-49 years	545<br>(487 to 611)	1,021.1<br>(911.2 to 1,143.2)	458<br>(414 to 506)	804.0<br>(726.6 to 888.1)	416<br>(370 to 464)	732.4<br>(652.3 to 817.0)	375<br>(341 to 417)	663.5<br>(604.6 to 738.3)	-21.3<br>(-27.5 to -13.2)	-8.9<br>(-16.6 to 0.2)
	50-69 years	493<br>(444 to 550)	4,634.2<br>(4,171.7 to 5,165.6)	776<br>(701 to 851)	3,311.5<br>(2,991.0 to 3,628.1)	746<br>(663 to 839)	3,092.7<br>(2,745.6 to 3,478.2)	691<br>(612 to 765)	2,800.1<br>(2,477.3 to 3,100.8)	-28.5<br>(-36.3 to -20.8)	-6.6<br>(-16.9 to 4.5)
	70+ years	744<br>(659 to 852)	17,048.1<br>(15,105.1 to 19,526.9)	996<br>(904 to 1,110)	12,938.6<br>(11,739.0 to 14,453.6)	925<br>(819 to 1,050)	11,885.8<br>(10,527.5 to 13,470.0)	832<br>(746 to 937)	10,456.1<br>(9,371.3 to 11,780.0)	-24.1<br>(-31.8 to -15.7)	-8.1<br>(-17.5 to 3.4)
**Suriname**	All Ages	9,900<br>(9,260 to 10,600)	2,559.4<br>(2,393.6 to 2,747.4)	11,000<br>(10,300 to 11,800)	1,922.1<br>(1,794.3 to 2,058.3)	10,300<br>(9,410 to 11,200)	1,778.0<br>(1,629.8 to 1,932.7)	9,250<br>(8,660 to 9,860)	1,596.3<br>(1,495.8 to 1,703.0)	-24.9<br>(-29.5 to -20.2)	-7.5<br>(-15.0 to 0.6)
	Under 5	2,800<br>(2,380 to 3,230)	6,392.8<br>(5,445.2 to 7,381.5)	1,390<br>(1,200 to 1,630)	3,040.9<br>(2,622.8 to 3,561.6)	1,170<br>(984 to 1,410)	2,598.0<br>(2,180.3 to 3,123.0)	952<br>(817 to 1,110)	2,137.8<br>(1,834.0 to 2,492.0)	-52.4<br>(-56.4 to -48.4)	-14.6<br>(-24.4 to -2.2)
	5-14 years	1,300<br>(1,000 to 1,650)	1,497.3<br>(1,159.3 to 1,907.4)	935<br>(738 to 1,200)	940.6<br>(742.7 to 1,205.5)	858<br>(672 to 1,080)	865.5<br>(678.4 to 1,085.3)	775<br>(603 to 968)	785.4<br>(611.0 to 980.1)	-37.2<br>(-43.0 to -31.1)	-8.0<br>(-16.6 to 1.7)
	15-49 years	1,880<br>(1,670 to 2,100)	951.9<br>(846.3 to 1,064.2)	2,120<br>(1,920 to 2,350)	736.1<br>(665.2 to 814.8)	1,930<br>(1,730 to 2,160)	672.1<br>(601.4 to 751.3)	1,750<br>(1,560 to 1,940)	610.4<br>(542.7 to 676.6)	-22.7<br>(-29.2 to -16.3)	-8.7<br>(-17.0 to -0.4)
	50-69 years	1,800<br>(1,590 to 2,010)	3,873.3<br>(3,422.0 to 4,329.2)	2,940<br>(2,620 to 3,230)	2,687.1<br>(2,388.9 to 2,951.7)	2,840<br>(2,510 to 3,200)	2,521.5<br>(2,223.1 to 2,831.9)	2,630<br>(2,330 to 2,920)	2,275.7<br>(2,020.5 to 2,531.0)	-30.6<br>(-37.0 to -23.5)	-6.2<br>(-16.3 to 3.2)
	70+ years	2,120<br>(1,890 to 2,410)	17,347.7<br>(15,463.4 to 19,754.1)	3,650<br>(3,230 to 4,130)	11,528.5<br>(10,191.3 to 13,034.7)	3,450<br>(3,050 to 3,900)	10,651.1<br>(9,395.6 to 12,037.6)	3,140<br>(2,820 to 3,550)	9,429.4<br>(8,480.1 to 10,666.7)	-33.5<br>(-39.6 to -26.4)	-7.6<br>(-17.3 to 2.7)
**Trinidad and Tobago**	All Ages	32,400<br>(30,000 to 35,100)	2,686.6<br>(2,491.3 to 2,909.6)	25,000<br>(23,500 to 26,900)	1,797.3<br>(1,684.7 to 1,929.7)	22,600<br>(20,800 to 24,700)	1,619.1<br>(1,488.9 to 1,771.4)	19,600<br>(18,400 to 20,900)	1,408.9<br>(1,318.2 to 1,499.9)	-33.1<br>(-37.6 to -28.6)	-9.9<br>(-16.7 to -1.2)

Location	Age Group	1990		2019		2020		2021		Percent Incidence Rate Change from 1990-2019 (95% UI)	Percent Incidence Rate Change from 2019-2021 (95% UI)
		Episodes (95% UI)	Rate (95% UI)	Episodes (95% UI)	Rate (95% UI)	Episodes (95% UI)	Rate (95% UI)	Episodes (95% UI)	Rate (95% UI)		
	Under 5	7,340 (6,300 to 8,520)	5,505.7 (4,729.5 to 6,392.5)	2,440 (2,090 to 2,880)	2,839.9 (2,427.6 to 3,344.7)	1,950 (1,610 to 2,380)	2,344.2 (1,936.7 to 2,858.5)	1,500 (1,260 to 1,770)	1,864.6 (1,569.4 to 2,203.1)	-48.4 (-53.9 to -41.8)	-17.5 (-28.2 to -4.8)
	5-14 years	4,780 (3,740 to 5,900)	1,749.2 (1,368.2 to 2,160.0)	1,960 (1,540 to 2,450)	1,013.2 (794.9 to 1,268.8)	1,770 (1,400 to 2,240)	915.4 (724.8 to 1,162.4)	1,560 (1,220 to 1,970)	813.4 (636.0 to 1,027.0)	-42.1 (-48.9 to -35.0)	-9.7 (-17.5 to -0.1)
	15-49 years	7,510 (6,760 to 8,410)	1,214.2 (1,092.3 to 1,359.0)	5,200 (4,670 to 5,800)	749.5 (672.9 to 835.7)	4,630 (4,110 to 5,150)	669.4 (594.2 to 744.3)	4,110 (3,700 to 4,580)	595.7 (535.9 to 663.7)	-38.3 (-44.1 to -32.1)	-10.7 (-18.9 to -1.1)
	50-69 years	5,590 (4,990 to 6,150)	4,217.2 (3,763.3 to 4,638.0)	6,830 (6,040 to 7,640)	2,162.7 (1,910.5 to 2,418.8)	6,250 (5,550 to 7,000)	1,967.8 (1,747.5 to 2,204.0)	5,500 (4,900 to 6,080)	1,726.2 (1,537.3 to 1,908.3)	-48.7 (-53.6 to -43.2)	-9.0 (-19.2 to 2.2)
	70+ years	7,150 (6,390 to 8,020)	15,139.8 (13,529.1 to 17,000.2)	8,610 (7,680 to 9,700)	8,250.8 (7,365.7 to 9,301.4)	7,980 (7,120 to 9,210)	7,327.3 (6,535.2 to 8,460.0)	6,950 (6,280 to 7,750)	6,213.8 (5,615.0 to 6,928.7)	-45.5 (-51.1 to -39.8)	-11.2 (-20.0 to -0.8)
**United States Virgin Islands**	All Ages	2,300 (2,120 to 2,490)	2,172.5 (1,999.9 to 2,351.5)	2,080 (1,910 to 2,250)	2,314.7 (2,115.6 to 2,501.5)	1,890 (1,710 to 2,100)	2,145.8 (1,942.4 to 2,385.2)	1,640 (1,500 to 1,780)	1,903.7 (1,743.7 to 2,073.1)	6.5 (-2.2 to 14.6)	-7.3 (-14.8 to 2.8)
	Under 5	563 (475 to 665)	5,111.4 (4,314.0 to 6,041.4)	131 (109 to 158)	2,963.5 (2,446.5 to 3,572.0)	104 (85 to 131)	2,498.6 (2,032.7 to 3,133.6)	78 (64 to 96)	2,002.7 (1,637.0 to 2,453.0)	-42.0 (-46.2 to -37.1)	-15.7 (-26.2 to -0.9)
	5-14 years	332 (248 to 432)	1,584.4 (1,184.7 to 2,062.9)	115 (86 to 150)	1,098.4 (821.4 to 1,430.4)	100 (75 to 130)	1,002.6 (756.6 to 1,306.7)	85 (63 to 110)	893.2 (663.2 to 1,159.9)	-30.7 (-35.6 to -23.5)	-8.7 (-17.0 to 1.5)
	15-49 years	487 (432 to 546)	887.2 (787.5 to 995.3)	217 (194 to 246)	599.4 (536.9 to 680.2)	188 (165 to 215)	538.6 (473.2 to 616.2)	162 (144 to 181)	478.4 (426.4 to 536.7)	-32.4 (-36.6 to -27.7)	-10.1 (-17.7 to -1.5)
	50-69 years	456 (405 to 505)	2,993.9 (2,655.6 to 3,315.7)	611 (546 to 679)	2,304.1 (2,057.8 to 2,558.6)	555 (494 to 621)	2,116.0 (1,884.1 to 2,369.9)	481 (428 to 531)	1,863.9 (1,659.1 to 2,057.5)	-23.0 (-30.5 to -15.4)	-8.2 (-17.3 to 1.2)
	70+ years	467 (413 to 531)	11,650.2 (10,313.3 to 13,254.9)	1,010 (884 to 1,160)	8,094.5 (7,078.8 to 9,253.7)	941 (823 to 1,090)	7,405.4 (6,472.3 to 8,570.2)	829 (731 to 945)	6,431.2 (5,666.2 to 7,330.7)	-30.5 (-37.4 to -23.1)	-8.5 (-18.3 to 4.3)
**Colombia**	All Ages	967,000 (893,000 to 1,060,000)	2,976.3 (2,749.0 to 3,275.5)	847,000 (790,000 to 911,000)	1,762.7 (1,644.6 to 1,896.7)	747,000 (688,000 to 808,000)	1,530.6 (1,410.3 to 1,655.8)	711,000 (666,000 to 765,000)	1,448.8 (1,358.1 to 1,559.2)	-40.8 (-44.6 to -36.3)	-13.2 (-19.0 to -6.7)
	Under 5	460,000 (396,000 to 534,000)	10,936.8 (9,418.6 to 12,688.8)	146,000 (122,000 to 173,000)	4,183.9 (3,506.4 to 4,963.1)	113,000 (95,100 to 135,000)	3,231.8 (2,725.6 to 3,863.0)	97,400 (82,300 to 116,000)	2,828.3 (2,391.2 to 3,355.7)	-61.7 (-65.7 to -57.0)	-22.8 (-31.2 to -11.8)
	5-14 years	134,000 (106,000 to 164,000)	1,797.7 (1,427.9 to 2,196.7)	68,100 (53,400 to 85,200)	934.5 (732.9 to 1,168.9)	60,200 (47,400 to 76,800)	830.9 (653.3 to 1,059.7)	56,400 (44,700 to 71,700)	786.6 (623.0 to 999.4)	-48.0 (-54.5 to -40.9)	-11.1 (-18.4 to -3.3)
	15-49 years	130,000 (117,000 to 144,000)	768.3 (691.5 to 853.4)	145,000 (131,000 to 161,000)	570.2 (515.2 to 632.0)	131,000 (116,000 to 147,000)	504.5 (446.9 to 565.1)	126,000 (112,000 to 140,000)	482.2 (430.6 to 536.4)	-25.8 (-31.2 to -19.8)	-11.5 (-18.7 to -3.3)
	50-69 years	118,000 (107,000 to 127,000)	3,834.3 (3,487.8 to 4,142.6)	206,000 (185,000 to 232,000)	2,341.1 (2,104.7 to 2,638.8)	188,000 (169,000 to 206,000)	2,094.4 (1,884.4 to 2,298.8)	186,000 (168,000 to 209,000)	2,030.3 (1,837.6 to 2,284.1)	-38.9 (-45.7 to -33.3)	-10.5 (-20.6 to 0.4)

Location	Age Group	1990		2019		2020		2021		Percent Incidence Rate Change from 1990-2019 (95% UI)	Percent Incidence Rate Change from 2019-2021 (95% UI)
		Episodes (95% UI)	Rate (95% UI)	Episodes (95% UI)	Rate (95% UI)	Episodes (95% UI)	Rate (95% UI)	Episodes (95% UI)	Rate (95% UI)		
	70+ years	125,000 (109,000 to 139,000)	14,942.3 (13,046.0 to 16,557.9)	282,000 (254,000 to 314,000)	9,443.3 (8,522.2 to 10,531.8)	255,000 (224,000 to 290,000)	8,212.6 (7,231.9 to 9,352.9)	245,000 (218,000 to 279,000)	7,699.5 (6,855.5 to 8,753.8)	-36.8 (-43.5 to -30.5)	-13.0 (-21.0 to -4.9)
**Costa Rica**	All Ages	59,800 (54,800 to 66,000)	1,965.1 (1,802.8 to 2,169.8)	69,400 (65,200 to 74,200)	1,473.7 (1,384.7 to 1,576.2)	57,200 (53,000 to 61,400)	1,208.1 (1,120.4 to 1,297.0)	55,800 (52,200 to 60,000)	1,175.0 (1,099.6 to 1,262.7)	-25.0 (-29.9 to -20.1)	-18.0 (-22.6 to -13.1)
	Under 5	24,000 (20,300 to 28,500)	5,913.3 (4,992.8 to 7,009.4)	10,700 (8,820 to 12,900)	3,186.6 (2,632.6 to 3,845.0)	7,040 (5,720 to 8,520)	2,180.0 (1,772.9 to 2,638.3)	6,200 (5,150 to 7,470)	2,010.9 (1,670.9 to 2,423.5)	-46.1 (-51.7 to -39.2)	-31.6 (-37.5 to -24.3)
	5-14 years	8,560 (6,500 to 11,100)	1,191.1 (904.3 to 1,548.4)	6,010 (4,540 to 7,720)	846.2 (638.3 to 1,086.1)	5,050 (3,830 to 6,610)	711.5 (539.1 to 930.2)	4,890 (3,730 to 6,450)	689.5 (526.3 to 909.7)	-29.0 (-34.7 to -22.4)	-15.9 (-22.2 to -8.7)
	15-49 years	8,640 (7,590 to 9,810)	560.4 (492.5 to 636.2)	11,500 (10,300 to 12,800)	462.5 (412.6 to 511.9)	9,840 (8,720 to 11,000)	393.8 (348.7 to 440.4)	9,590 (8,450 to 10,800)	383.1 (337.3 to 430.2)	-17.5 (-23.6 to -10.4)	-14.9 (-22.5 to -6.9)
	50-69 years	7,480 (6,700 to 8,330)	2,657.8 (2,381.6 to 2,958.0)	18,000 (16,300 to 19,800)	2,053.0 (1,852.7 to 2,253.3)	15,700 (14,200 to 17,500)	1,756.4 (1,578.8 to 1,947.1)	15,700 (14,100 to 17,300)	1,716.0 (1,542.5 to 1,893.1)	-22.8 (-28.9 to -14.5)	-14.4 (-20.8 to -6.7)
	70+ years	11,100 (9,860 to 12,400)	11,709.3 (10,414.3 to 13,137.2)	23,100 (20,900 to 25,900)	8,005.8 (7,232.5 to 8,971.6)	19,500 (17,200 to 21,600)	6,451.6 (5,685.0 to 7,150.5)	19,400 (17,300 to 21,900)	6,197.9 (5,530.6 to 6,990.9)	-31.6 (-38.1 to -23.5)	-19.4 (-26.2 to -12.9)
**El Salvador**	All Ages	179,000 (165,000 to 196,000)	3,367.2 (3,110.9 to 3,686.2)	159,000 (149,000 to 170,000)	2,485.3 (2,324.0 to 2,659.8)	127,000 (119,000 to 136,000)	1,977.0 (1,851.4 to 2,116.1)	124,000 (117,000 to 133,000)	1,929.8 (1,806.3 to 2,068.5)	-26.2 (-32.3 to -19.5)	-20.5 (-24.5 to -15.9)
	Under 5	82,100 (71,600 to 97,000)	10,666.3 (9,294.6 to 12,602.3)	27,400 (23,000 to 32,500)	4,377.2 (3,677.6 to 5,195.2)	17,100 (14,500 to 20,600)	2,790.7 (2,356.5 to 3,352.5)	16,100 (13,500 to 19,100)	2,681.4 (2,250.0 to 3,186.6)	-59.0 (-64.2 to -53.4)	-36.2 (-41.7 to -28.6)
	5-14 years	24,600 (19,500 to 30,100)	1,771.7 (1,407.8 to 2,168.8)	13,200 (10,400 to 16,800)	1,090.8 (863.2 to 1,385.1)	10,800 (8,560 to 13,700)	891.9 (704.6 to 1,126.1)	10,700 (8,440 to 13,500)	878.9 (692.9 to 1,107.6)	-38.4 (-46.3 to -30.5)	-18.2 (-24.8 to -10.8)
	15-49 years	20,400 (18,500 to 22,700)	816.1 (738.8 to 905.8)	22,900 (20,700 to 25,400)	695.7 (630.2 to 772.3)	19,500 (17,500 to 21,700)	590.9 (528.9 to 657.4)	19,200 (17,300 to 21,500)	579.4 (522.1 to 648.6)	-14.7 (-21.4 to -7.7)	-15.1 (-21.8 to -7.3)
	50-69 years	21,100 (19,100 to 23,500)	4,328.1 (3,919.9 to 4,833.5)	32,400 (29,600 to 36,300)	3,602.9 (3,286.7 to 4,036.1)	28,300 (25,600 to 31,000)	3,070.9 (2,778.1 to 3,371.3)	28,000 (25,400 to 31,000)	2,981.4 (2,705.9 to 3,305.9)	-16.8 (-24.4 to -8.3)	-14.8 (-22.2 to -7.2)
	70+ years	30,400 (27,000 to 34,300)	19,200.6 (17,053.3 to 21,668.8)	63,100 (56,500 to 71,500)	16,909.5 (15,124.2 to 19,146.3)	51,300 (45,600 to 57,500)	13,576.5 (12,051.1 to 15,215.6)	50,500 (45,400 to 56,300)	13,192.9 (11,860.2 to 14,706.1)	-11.9 (-18.8 to -3.1)	-19.7 (-27.1 to -12.6)
**Guatemala**	All Ages	500,000 (457,000 to 543,000)	5,962.1 (5,451.3 to 6,480.3)	383,000 (358,000 to 411,000)	2,479.0 (2,315.2 to 2,656.7)	353,000 (325,000 to 385,000)	2,260.1 (2,081.8 to 2,464.8)	316,000 (294,000 to 340,000)	2,004.6 (1,865.7 to 2,153.3)	-58.4 (-61.5 to -55.9)	-8.8 (-15.6 to -0.1)
	Under 5	234,000 (201,000 to 269,000)	15,250.8 (13,096.4 to 17,531.8)	108,000 (91,800 to 127,000)	6,614.4 (5,620.7 to 7,748.4)	92,700 (76,500 to 112,000)	5,802.1 (4,788.6 to 7,017.7)	71,700 (61,500 to 83,000)	4,599.3 (3,941.2 to 5,324.5)	-56.6 (-61.2 to -50.8)	-12.3 (-24.6 to 3.1)
	5-14 years	97,600 (84,500 to 114,000)	3,860.2 (3,343.4 to 4,523.3)	44,400 (36,000 to 53,100)	1,307.2 (1,060.5 to 1,564.2)	42,000 (34,400 to 51,000)	1,240.9 (1,015.2 to 1,505.6)	37,700 (31,100 to 45,600)	1,118.3 (921.3 to 1,352.2)	-66.1 (-71.8 to -60.3)	-5.1 (-17.2 to 8.6)

Location	Age Group	1990		2019		2020		2021		Percent Incidence Rate Change from 1990-2019 (95% UI)	Percent Incidence Rate Change from 2019-2021 (95% UI)
		Episodes (95% UI)	Rate (95% UI)	Episodes (95% UI)	Rate (95% UI)	Episodes (95% UI)	Rate (95% UI)	Episodes (95% UI)	Rate (95% UI)		
	15-49 years	67,500 (62,300 to 73,600)	1,909.0 (1,761.9 to 2,081.3)	66,000 (60,400 to 72,600)	811.7 (742.7 to 892.2)	63,000 (56,100 to 70,300)	761.4 (678.0 to 849.4)	60,000 (54,200 to 65,800)	713.6 (644.5 to 782.0)	-57.5 (-60.7 to -53.7)	-6.2 (-15.5 to 3.8)
	50-69 years	55,700 (51,000 to 61,700)	8,713.7 (7,973.2 to 9,645.8)	69,200 (63,200 to 75,300)	3,985.7 (3,639.5 to 4,335.0)	65,700 (59,800 to 72,500)	3,675.0 (3,344.4 to 4,054.3)	62,800 (56,800 to 69,700)	3,430.2 (3,103.2 to 3,809.9)	-54.3 (-58.0 to -49.7)	-7.8 (-16.6 to 0.6)
	70+ years	45,100 (40,000 to 49,900)	30,770.6 (27,236.0 to 33,991.6)	95,500 (85,900 to 104,000)	17,191.9 (15,465.5 to 18,791.8)	89,700 (79,900 to 99,300)	15,638.5 (13,938.3 to 17,314.8)	83,800 (76,200 to 92,400)	14,216.0 (12,926.3 to 15,672.1)	-44.1 (-49.7 to -37.3)	-9.0 (-17.2 to 1.3)
**Honduras**	All Ages	171,000 (154,000 to 192,000)	3,631.9 (3,259.3 to 4,077.9)	150,000 (138,000 to 164,000)	1,541.5 (1,415.7 to 1,682.5)	134,000 (120,000 to 147,000)	1,348.6 (1,210.7 to 1,478.4)	129,000 (117,000 to 145,000)	1,277.1 (1,156.2 to 1,434.9)	-57.6 (-60.9 to -54.1)	-12.5 (-19.6 to -4.2)
	Under 5	111,000 (95,400 to 130,000)	13,461.0 (11,588.8 to 15,780.0)	51,100 (43,600 to 59,800)	4,697.8 (4,007.7 to 5,506.4)	42,700 (35,000 to 51,400)	3,913.3 (3,202.3 to 4,707.2)	39,600 (32,900 to 47,900)	3,614.4 (2,999.1 to 4,373.1)	-65.1 (-68.6 to -61.0)	-16.7 (-27.5 to -3.0)
	5-14 years	22,900 (18,100 to 28,400)	1,650.2 (1,302.7 to 2,048.0)	21,600 (16,400 to 27,700)	1,002.8 (761.2 to 1,287.4)	19,800 (15,200 to 25,500)	911.5 (699.7 to 1,177.7)	19,200 (14,700 to 24,500)	879.5 (674.2 to 1,123.9)	-39.2 (-46.7 to -30.5)	-9.1 (-17.8 to 0.3)
	15-49 years	15,900 (14,000 to 17,700)	773.9 (682.2 to 863.1)	24,400 (21,600 to 27,600)	471.3 (417.6 to 532.8)	22,600 (20,000 to 25,800)	427.5 (377.9 to 487.7)	22,400 (19,300 to 25,600)	413.9 (356.6 to 472.7)	-39.1 (-44.4 to -33.7)	-9.3 (-16.4 to -0.2)
	50-69 years	10,600 (9,400 to 11,900)	3,000.4 (2,649.0 to 3,366.8)	25,000 (22,100 to 28,200)	2,422.6 (2,137.3 to 2,728.9)	23,700 (21,100 to 26,300)	2,205.7 (1,963.9 to 2,454.2)	23,500 (20,900 to 26,300)	2,131.2 (1,891.4 to 2,384.6)	-19.3 (-27.4 to -11.1)	-9.0 (-17.5 to 1.9)
	70+ years	10,900 (9,570 to 12,400)	11,173.5 (9,808.9 to 12,719.8)	28,200 (24,800 to 31,800)	9,299.3 (8,163.2 to 10,487.2)	25,200 (22,200 to 29,100)	8,193.6 (7,220.1 to 9,475.0)	24,400 (21,300 to 27,700)	7,743.2 (6,753.6 to 8,797.5)	-16.8 (-25.7 to -8.5)	-11.9 (-22.3 to -0.2)
**Mexico**	All Ages	1,970,000 (1,840,000 to 2,130,000)	2,310.8 (2,154.2 to 2,494.3)	1,330,000 (1,260,000 to 1,410,000)	1,044.4 (985.7 to 1,107.5)	1,270,000 (1,190,000 to 1,360,000)	988.7 (927.0 to 1,058.6)	1,110,000 (1,040,000 to 1,170,000)	856.1 (805.2 to 906.4)	-54.8 (-57.0 to -52.5)	-5.3 (-9.4 to -1.1)
	Under 5	876,000 (779,000 to 989,000)	7,425.5 (6,601.8 to 8,383.6)	250,000 (214,000 to 288,000)	2,371.5 (2,035.9 to 2,732.0)	221,000 (186,000 to 261,000)	2,167.3 (1,818.0 to 2,558.5)	164,000 (140,000 to 192,000)	1,661.2 (1,420.3 to 1,941.0)	-68.1 (-70.3 to -65.8)	-8.6 (-15.2 to -1.6)
	5-14 years	324,000 (262,000 to 396,000)	1,498.5 (1,212.2 to 1,833.0)	124,000 (98,500 to 157,000)	551.7 (438.5 to 700.2)	117,000 (93,300 to 148,000)	524.0 (417.4 to 664.2)	103,000 (81,800 to 131,000)	465.5 (368.8 to 589.5)	-63.2 (-66.7 to -59.3)	-5.0 (-8.5 to -0.8)
	15-49 years	304,000 (276,000 to 335,000)	713.7 (650.0 to 786.5)	278,000 (256,000 to 303,000)	412.7 (379.7 to 449.1)	268,000 (246,000 to 295,000)	394.2 (361.2 to 433.7)	242,000 (222,000 to 266,000)	354.1 (324.8 to 389.1)	-42.2 (-44.0 to -40.1)	-4.5 (-8.2 to -0.7)
	50-69 years	223,000 (203,000 to 243,000)	3,028.8 (2,755.8 to 3,298.6)	315,000 (288,000 to 345,000)	1,522.6 (1,388.2 to 1,665.3)	311,000 (287,000 to 338,000)	1,453.5 (1,341.7 to 1,578.7)	287,000 (261,000 to 310,000)	1,303.6 (1,188.4 to 1,410.4)	-49.7 (-51.0 to -48.2)	-4.5 (-8.0 to -0.4)
	70+ years	247,000 (222,000 to 273,000)	11,872.3 (10,694.3 to 13,125.8)	365,000 (334,000 to 401,000)	5,666.6 (5,177.6 to 6,223.0)	354,000 (324,000 to 392,000)	5,349.7 (4,896.8 to 5,934.5)	310,000 (285,000 to 341,000)	4,592.3 (4,211.7 to 5,049.9)	-52.3 (-53.6 to -51.0)	-5.6 (-9.9 to -1.3)
**Nicaragua**	All Ages	134,000 (121,000 to 149,000)	3,437.8 (3,124.1 to 3,840.9)	100,000 (93,200 to 108,000)	1,539.6 (1,429.9 to 1,657.4)	78,900 (73,400 to 85,100)	1,195.9 (1,112.7 to 1,289.6)	80,300 (74,300 to 86,900)	1,204.7 (1,114.4 to 1,302.4)	-55.2 (-58.2 to -51.6)	-22.3 (-26.1 to -17.7)

Location	Age Group	1990		2019		2020		2021		Percent Incidence Rate Change from 1990-2019 (95% UI)	Percent Incidence Rate Change from 2019-2021 (95% UI)
		Episodes (95% UI)	Rate (95% UI)	Episodes (95% UI)	Rate (95% UI)	Episodes (95% UI)	Rate (95% UI)	Episodes (95% UI)	Rate (95% UI)		
	Under 5	84,200 (72,600 to 98,200)	12,684.4 (10,940.6 to 14,796.8)	31,300 (26,700 to 36,600)	4,715.9 (4,027.0 to 5,520.9)	20,500 (17,400 to 24,100)	3,118.8 (2,646.1 to 3,660.8)	20,600 (17,400 to 24,300)	3,170.1 (2,677.1 to 3,741.9)	-62.8 (-66.6 to -58.9)	-33.9 (-40.4 to -27.2)
	5-14 years	20,400 (16,200 to 25,200)	1,759.2 (1,402.7 to 2,175.9)	13,100 (10,400 to 16,300)	995.2 (790.0 to 1,240.9)	11,000 (8,630 to 13,500)	828.6 (651.8 to 1,018.8)	11,100 (8,900 to 13,800)	834.0 (669.3 to 1,038.1)	-43.4 (-50.3 to -35.9)	-16.7 (-24.0 to -8.3)
	15-49 years	11,600 (10,400 to 13,100)	672.0 (600.5 to 755.1)	16,500 (14,700 to 18,400)	469.4 (419.2 to 525.2)	14,000 (12,400 to 15,600)	394.8 (350.2 to 440.3)	14,300 (12,700 to 16,000)	398.2 (353.0 to 444.4)	-30.2 (-35.1 to -23.8)	-15.9 (-22.0 to -9.7)
	50-69 years	8,280 (7,390 to 9,280)	3,132.7 (2,797.8 to 3,515.3)	17,300 (15,600 to 19,200)	2,176.4 (1,965.7 to 2,415.9)	15,300 (13,800 to 17,000)	1,857.3 (1,672.8 to 2,069.6)	15,800 (14,000 to 17,400)	1,863.2 (1,654.1 to 2,054.1)	-30.5 (-37.3 to -22.9)	-14.7 (-21.3 to -7.0)
	70+ years	9,230 (8,140 to 10,600)	12,538.3 (11,063.0 to 14,373.0)	22,200 (19,800 to 24,800)	9,336.8 (8,346.0 to 10,437.9)	18,100 (16,200 to 20,500)	7,411.9 (6,629.6 to 8,382.0)	18,500 (16,600 to 21,000)	7,427.9 (6,647.8 to 8,408.1)	-25.5 (-32.6 to -17.6)	-20.6 (-27.6 to -13.0)
**Panama**	All Ages	47,200 (43,500 to 51,500)	1,974.6 (1,820.7 to 2,157.7)	67,000 (62,700 to 71,900)	1,613.6 (1,509.2 to 1,731.9)	57,000 (53,000 to 61,700)	1,347.3 (1,250.5 to 1,457.8)	59,000 (53,100 to 66,200)	1,375.4 (1,237.2 to 1,543.5)	-18.3 (-22.5 to -13.1)	-16.5 (-21.5 to -11.0)
	Under 5	17,800 (15,000 to 21,200)	6,221.0 (5,251.0 to 7,415.8)	15,600 (13,200 to 18,500)	4,107.2 (3,482.3 to 4,859.5)	11,400 (9,620 to 13,700)	3,030.1 (2,546.7 to 3,637.1)	11,700 (9,410 to 15,000)	3,164.8 (2,534.3 to 4,028.6)	-34.0 (-41.1 to -27.4)	-26.2 (-34.2 to -16.0)
	5-14 years	6,230 (4,850 to 7,930)	1,136.3 (884.7 to 1,446.3)	6,040 (4,710 to 7,680)	794.9 (619.9 to 1,010.8)	5,380 (4,220 to 6,780)	695.6 (545.5 to 877.3)	5,500 (4,320 to 7,010)	703.4 (551.7 to 895.8)	-30.1 (-36.0 to -23.1)	-12.5 (-20.1 to -4.1)
	15-49 years	7,010 (6,280 to 7,920)	569.0 (509.8 to 642.8)	10,200 (9,080 to 11,300)	484.0 (432.6 to 540.2)	9,060 (8,150 to 10,100)	423.4 (381.1 to 470.1)	9,220 (8,210 to 10,500)	425.7 (379.2 to 484.5)	-14.9 (-22.3 to -7.8)	-12.5 (-19.4 to -3.5)
	50-69 years	6,840 (6,080 to 7,580)	2,822.0 (2,508.8 to 3,128.6)	13,800 (12,500 to 15,300)	2,063.3 (1,866.0 to 2,284.1)	12,500 (11,000 to 14,000)	1,803.8 (1,597.4 to 2,033.8)	13,000 (11,500 to 14,500)	1,817.2 (1,607.4 to 2,027.2)	-26.9 (-33.4 to -19.4)	-12.6 (-21.0 to -4.9)
	70+ years	9,330 (8,170 to 10,600)	11,559.0 (10,128.5 to 13,195.3)	21,400 (19,000 to 24,300)	8,750.9 (7,771.2 to 9,958.2)	18,700 (16,400 to 21,300)	7,353.7 (6,434.7 to 8,370.5)	19,600 (17,100 to 22,500)	7,512.4 (6,560.8 to 8,607.9)	-24.3 (-31.6 to -15.9)	-16.0 (-24.6 to -6.7)
**Venezuela (Bolivarian Republic of)**	All Ages	451,000 (412,000 to 496,000)	2,400.2 (2,188.9 to 2,637.0)	480,000 (451,000 to 515,000)	1,698.0 (1,595.2 to 1,823.4)	388,000 (363,000 to 414,000)	1,436.6 (1,344.2 to 1,533.8)	348,000 (326,000 to 372,000)	1,305.3 (1,223.0 to 1,396.5)	-29.3 (-34.5 to -23.4)	-15.4 (-19.9 to -10.8)
	Under 5	210,000 (177,000 to 247,000)	8,291.3 (6,981.8 to 9,778.2)	104,000 (89,000 to 120,000)	4,132.5 (3,534.0 to 4,777.4)	71,300 (60,100 to 82,900)	3,115.4 (2,624.5 to 3,620.9)	59,500 (51,000 to 70,000)	2,725.1 (2,334.3 to 3,202.0)	-50.2 (-56.7 to -44.3)	-24.6 (-31.3 to -16.9)
	5-14 years	62,000 (48,500 to 78,000)	1,359.6 (1,062.8 to 1,708.4)	42,100 (33,000 to 52,600)	883.8 (693.3 to 1,106.2)	34,500 (27,500 to 42,900)	759.5 (606.7 to 944.7)	31,400 (24,900 to 39,400)	707.9 (561.8 to 886.8)	-35.0 (-41.7 to -27.6)	-14.1 (-22.1 to -6.2)
	15-49 years	66,300 (60,100 to 73,400)	691.1 (626.8 to 765.8)	82,900 (75,700 to 91,400)	577.0 (526.7 to 635.7)	66,800 (60,400 to 72,900)	496.5 (449.3 to 542.3)	60,200 (54,600 to 66,500)	457.7 (415.0 to 505.5)	-16.5 (-23.8 to -9.5)	-13.9 (-20.3 to -6.8)
	50-69 years	51,800 (46,300 to 56,900)	3,139.3 (2,805.9 to 3,450.4)	114,000 (103,000 to 126,000)	2,249.5 (2,033.5 to 2,478.0)	101,000 (89,800 to 111,000)	1,943.0 (1,736.1 to 2,144.7)	92,300 (84,100 to 100,000)	1,757.7 (1,601.3 to 1,912.2)	-28.3 (-35.4 to -19.5)	-13.6 (-21.5 to -6.3)

Location	Age Group	1990		2019		2020		2021		Percent Incidence Rate Change from 1990-2019 (95% UI)	Percent Incidence Rate Change from 2019-2021 (95% UI)
		Episodes (95% UI)	Rate (95% UI)	Episodes (95% UI)	Rate (95% UI)	Episodes (95% UI)	Rate (95% UI)	Episodes (95% UI)	Rate (95% UI)		
	70+ years	61,500 (54,800 to 67,800)	12,955.0 (11,535.4 to 14,278.2)	136,000 (123,000 to 156,000)	9,012.4 (8,148.6 to 10,351.3)	115,000 (105,000 to 126,000)	7,351.5 (6,693.3 to 8,068.3)	104,000 (93,100 to 118,000)	6,485.7 (5,795.9 to 7,353.5)	-30.4 (-36.9 to -22.7)	-18.4 (-26.3 to -11.6)
**Brazil**	All Ages	7,640,000 (7,190,000 to 8,190,000)	5,144.7 (4,842.3 to 5,514.5)	7,990,000 (7,560,000 to 8,480,000)	3,688.1 (3,486.0 to 3,910.5)	7,120,000 (6,670,000 to 7,600,000)	3,253.5 (3,050.7 to 3,474.8)	6,990,000 (6,530,000 to 7,520,000)	3,169.9 (2,964.9 to 3,412.2)	-28.3 (-31.4 to -25.3)	-11.8 (-15.7 to -7.0)
	Under 5	2,350,000 (2,080,000 to 2,670,000)	14,274.8 (12,642.0 to 16,228.1)	1,090,000 (948,000 to 1,250,000)	6,541.3 (5,664.3 to 7,497.2)	843,000 (718,000 to 987,000)	5,053.3 (4,305.3 to 5,915.8)	784,000 (666,000 to 925,000)	4,736.1 (4,022.3 to 5,584.4)	-54.2 (-55.9 to -52.3)	-22.7 (-28.8 to -14.4)
	5-14 years	1,180,000 (960,000 to 1,430,000)	3,313.3 (2,704.6 to 4,017.2)	538,000 (434,000 to 663,000)	1,720.7 (1,389.3 to 2,120.3)	481,000 (387,000 to 589,000)	1,529.0 (1,231.0 to 1,874.9)	470,000 (384,000 to 582,000)	1,487.1 (1,212.6 to 1,841.5)	-48.1 (-51.0 to -45.0)	-11.1 (-14.6 to -7.0)
	15-49 years	1,640,000 (1,500,000 to 1,790,000)	2,142.0 (1,956.5 to 2,339.8)	1,390,000 (1,280,000 to 1,510,000)	1,202.0 (1,108.5 to 1,305.4)	1,250,000 (1,140,000 to 1,370,000)	1,076.0 (988.3 to 1,183.5)	1,220,000 (1,120,000 to 1,340,000)	1,048.8 (963.5 to 1,159.0)	-43.9 (-45.7 to -41.9)	-10.5 (-14.2 to -6.4)
	50-69 years	1,270,000 (1,160,000 to 1,380,000)	8,082.8 (7,402.3 to 8,766.4)	2,080,000 (1,910,000 to 2,240,000)	5,147.2 (4,725.6 to 5,548.1)	1,930,000 (1,770,000 to 2,100,000)	4,675.8 (4,279.7 to 5,074.5)	1,910,000 (1,750,000 to 2,090,000)	4,543.8 (4,164.3 to 4,953.6)	-36.3 (-38.7 to -33.7)	-9.2 (-12.7 to -4.7)
	70+ years	1,210,000 (1,090,000 to 1,340,000)	28,362.1 (25,622.3 to 31,452.2)	2,900,000 (2,660,000 to 3,170,000)	22,135.2 (20,291.3 to 24,224.3)	2,620,000 (2,360,000 to 2,890,000)	19,272.9 (17,367.1 to 21,296.5)	2,600,000 (2,370,000 to 2,930,000)	18,487.6 (16,827.2 to 20,801.6)	-22.0 (-25.1 to -18.2)	-12.9 (-17.0 to -8.0)
**Paraguay**	All Ages	165,000 (152,000 to 180,000)	4,089.2 (3,754.9 to 4,442.6)	146,000 (137,000 to 156,000)	2,087.6 (1,961.7 to 2,229.4)	125,000 (117,000 to 133,000)	1,759.1 (1,644.9 to 1,875.9)	126,000 (118,000 to 134,000)	1,753.7 (1,639.9 to 1,874.1)	-48.9 (-52.2 to -45.3)	-15.7 (-20.1 to -11.3)
	Under 5	72,700 (62,100 to 85,700)	11,790.4 (10,061.7 to 13,891.0)	21,700 (18,300 to 25,400)	3,285.7 (2,772.3 to 3,850.3)	15,200 (12,800 to 17,800)	2,321.8 (1,951.9 to 2,716.6)	14,800 (12,500 to 17,700)	2,282.9 (1,916.3 to 2,720.8)	-72.1 (-75.4 to -68.4)	-29.3 (-36.0 to -22.0)
	5-14 years	27,700 (22,400 to 34,100)	2,632.6 (2,130.2 to 3,236.0)	16,400 (12,900 to 20,900)	1,210.2 (948.7 to 1,539.3)	14,200 (11,200 to 17,800)	1,044.2 (821.7 to 1,308.9)	14,100 (11,000 to 17,900)	1,036.8 (809.9 to 1,320.5)	-54.0 (-59.4 to -47.9)	-13.7 (-21.5 to -5.8)
	15-49 years	23,800 (21,200 to 26,600)	1,259.5 (1,123.5 to 1,406.9)	31,500 (28,500 to 35,100)	842.1 (761.2 to 937.3)	27,900 (25,000 to 31,300)	732.9 (656.8 to 823.5)	28,300 (25,400 to 31,500)	733.5 (659.2 to 816.3)	-33.1 (-38.7 to -26.8)	-13.0 (-18.9 to -6.8)
	50-69 years	18,200 (16,200 to 20,100)	4,953.7 (4,413.9 to 5,493.6)	32,800 (29,500 to 35,900)	3,439.2 (3,092.6 to 3,761.1)	30,100 (27,200 to 33,100)	3,070.9 (2,771.1 to 3,370.8)	30,400 (27,200 to 33,300)	3,043.3 (2,716.6 to 3,333.0)	-30.6 (-36.7 to -23.4)	-10.7 (-17.2 to -3.1)
	70+ years	22,900 (20,300 to 26,000)	19,737.2 (17,530.9 to 22,408.5)	43,700 (39,600 to 48,600)	15,331.7 (13,899.4 to 17,051.8)	37,300 (32,600 to 41,900)	12,770.4 (11,172.3 to 14,357.1)	38,100 (33,900 to 42,500)	12,604.5 (11,218.4 to 14,078.4)	-22.3 (-29.1 to -15.1)	-16.7 (-24.7 to -9.4)
**Afghanistan**	All Ages	575,000 (525,000 to 637,000)	5,780.7 (5,278.1 to 6,406.3)	724,000 (670,000 to 790,000)	2,474.1 (2,288.5 to 2,697.5)	703,000 (630,000 to 782,000)	2,325.2 (2,085.6 to 2,585.6)	669,000 (613,000 to 735,000)	2,142.5 (1,964.8 to 2,354.7)	-57.2 (-60.0 to -54.1)	-6.0 (-13.5 to 2.3)
	Under 5	336,000 (292,000 to 393,000)	19,614.4 (17,057.8 to 22,906.3)	344,000 (301,000 to 399,000)	6,645.0 (5,816.8 to 7,718.5)	325,000 (274,000 to 382,000)	6,111.0 (5,146.9 to 7,174.8)	303,000 (260,000 to 354,000)	5,530.7 (4,751.0 to 6,456.4)	-66.1 (-69.0 to -62.4)	-8.0 (-17.9 to 3.5)

Location	Age Group	1990		2019		2020		2021		Percent Incidence Rate Change from 1990-2019 (95% UI)	Percent Incidence Rate Change from 2019-2021 (95% UI)
		Episodes (95% UI)	Rate (95% UI)	Episodes (95% UI)	Rate (95% UI)	Episodes (95% UI)	Rate (95% UI)	Episodes (95% UI)	Rate (95% UI)		
	5-14 years	65,300<br>(51,700 to 80,500)	2,515.9<br>(1,991.9 to 3,101.8)	123,000<br>(99,500 to 151,000)	1,490.3<br>(1,205.1 to 1,826.5)	122,000<br>(97,000 to 148,000)	1,437.7<br>(1,144.5 to 1,749.6)	120,000<br>(95,800 to 147,000)	1,373.0<br>(1,098.7 to 1,685.6)	-40.8<br>(-48.0 to -32.4)	-3.5<br>(-15.6 to 7.1)
	15-49 years	56,200<br>(50,400 to 63,100)	1,405.5<br>(1,259.6 to 1,577.4)	141,000<br>(126,000 to 156,000)	1,012.6<br>(908.7 to 1,119.1)	139,000<br>(125,000 to 155,000)	969.7<br>(872.2 to 1,077.9)	136,000<br>(122,000 to 151,000)	919.6<br>(828.5 to 1,022.4)	-28.0<br>(-35.1 to -20.4)	-4.2<br>(-12.0 to 4.7)
	50-69 years	63,500<br>(55,600 to 71,400)	4,789.7<br>(4,195.3 to 5,385.9)	55,000<br>(48,800 to 61,800)	3,671.7<br>(3,257.0 to 4,125.8)	58,000<br>(51,000 to 65,300)	3,473.7<br>(3,057.9 to 3,914.7)	57,600<br>(50,900 to 65,200)	3,132.6<br>(2,770.4 to 3,544.8)	-23.3<br>(-31.6 to -13.2)	-5.4<br>(-15.6 to 5.5)
	70+ years	53,600<br>(47,100 to 60,800)	17,254.5<br>(15,162.1 to 19,572.1)	61,400<br>(54,300 to 69,700)	14,291.9<br>(12,623.9 to 16,223.7)	58,500<br>(51,300 to 66,400)	14,179.3<br>(12,428.0 to 16,108.9)	52,600<br>(46,600 to 59,600)	12,997.6<br>(11,521.6 to 14,741.6)	-17.2<br>(-25.7 to -6.1)	-0.8<br>(-11.7 to 10.3)
**Algeria**	All Ages	750,000<br>(687,000 to 823,000)	2,966.2<br>(2,716.6 to 3,254.6)	751,000<br>(699,000 to 803,000)	1,749.4<br>(1,629.1 to 1,870.9)	719,000<br>(662,000 to 788,000)	1,649.1<br>(1,518.4 to 1,807.8)	669,000<br>(622,000 to 721,000)	1,512.5<br>(1,406.8 to 1,631.7)	-41.0<br>(-44.7 to -36.4)	-5.7<br>(-12.4 to 2.2)
	Under 5	315,000<br>(263,000 to 372,000)	8,429.6<br>(7,046.2 to 9,964.4)	135,000<br>(114,000 to 160,000)	2,789.5<br>(2,369.0 to 3,308.7)	116,000<br>(95,100 to 141,000)	2,419.2<br>(1,987.6 to 2,950.6)	96,400<br>(81,000 to 116,000)	2,049.8<br>(1,722.2 to 2,465.3)	-66.9<br>(-69.8 to -63.4)	-13.3<br>(-24.1 to 1.5)
	5-14 years	137,000<br>(110,000 to 169,000)	1,962.8<br>(1,579.4 to 2,413.8)	88,000<br>(67,700 to 113,000)	1,096.6<br>(844.2 to 1,406.0)	86,000<br>(67,000 to 107,000)	1,033.6<br>(804.5 to 1,284.2)	83,000<br>(64,500 to 106,000)	965.6<br>(750.8 to 1,235.2)	-44.1<br>(-50.5 to -36.7)	-5.7<br>(-13.9 to 4.8)
	15-49 years	135,000<br>(119,000 to 152,000)	1,144.8<br>(1,008.5 to 1,287.3)	188,000<br>(169,000 to 209,000)	840.6<br>(757.1 to 933.4)	179,000<br>(159,000 to 202,000)	795.4<br>(705.9 to 898.8)	170,000<br>(151,000 to 191,000)	751.3<br>(668.3 to 845.3)	-26.6<br>(-32.7 to -19.7)	-5.4<br>(-13.8 to 4.8)
	50-69 years	86,500<br>(76,900 to 97,800)	3,987.5<br>(3,545.7 to 4,507.9)	167,000<br>(147,000 to 187,000)	2,739.1<br>(2,412.3 to 3,073.9)	166,000<br>(148,000 to 189,000)	2,639.9<br>(2,342.6 to 2,991.9)	160,000<br>(141,000 to 178,000)	2,455.9<br>(2,163.9 to 2,733.1)	-31.3<br>(-38.6 to -23.6)	-3.6<br>(-12.1 to 6.5)
	70+ years	76,700<br>(66,100 to 87,700)	12,320.3<br>(10,609.5 to 14,077.1)	173,000<br>(151,000 to 195,000)	10,707.7<br>(9,359.2 to 12,060.0)	172,000<br>(152,000 to 197,000)	10,195.7<br>(8,998.4 to 11,688.8)	159,000<br>(141,000 to 181,000)	9,176.9<br>(8,137.5 to 10,440.3)	-13.1<br>(-22.9 to -1.7)	-4.8<br>(-14.9 to 7.2)
**Bahrain**	All Ages	8,000<br>(7,240 to 8,850)	1,580.3<br>(1,430.2 to 1,747.4)	16,300<br>(14,900 to 17,700)	1,087.7<br>(997.2 to 1,182.0)	15,300<br>(13,800 to 17,100)	1,013.4<br>(914.0 to 1,126.4)	14,200<br>(13,000 to 15,400)	927.1<br>(848.5 to 1,007.8)	-31.2<br>(-35.0 to -26.8)	-6.8<br>(-14.2 to 2.2)
	Under 5	2,700<br>(2,280 to 3,190)	4,388.6<br>(3,710.9 to 5,200.0)	2,470<br>(2,010 to 2,990)	2,501.4<br>(2,036.4 to 3,019.7)	2,060<br>(1,590 to 2,570)	2,132.3<br>(1,652.5 to 2,666.8)	1,650<br>(1,320 to 2,030)	1,759.7<br>(1,412.9 to 2,161.9)	-43.0<br>(-49.0 to -37.4)	-14.8<br>(-27.5 to 1.9)
	5-14 years	1,430<br>(1,060 to 1,910)	1,405.3<br>(1,038.3 to 1,871.3)	2,180<br>(1,630 to 2,820)	1,088.6<br>(815.4 to 1,406.0)	2,040<br>(1,540 to 2,630)	1,010.0<br>(762.2 to 1,304.6)	1,880<br>(1,410 to 2,400)	924.7<br>(696.6 to 1,183.3)	-22.5<br>(-28.4 to -16.0)	-7.2<br>(-15.9 to 3.0)
	15-49 years	2,180<br>(1,860 to 2,540)	723.0<br>(615.9 to 842.2)	6,040<br>(5,250 to 6,910)	621.8<br>(540.4 to 710.6)	5,650<br>(4,880 to 6,580)	577.6<br>(499.0 to 672.7)	5,250<br>(4,550 to 6,010)	536.5<br>(464.9 to 613.4)	-14.0<br>(-21.2 to -6.6)	-7.1<br>(-15.5 to 1.6)
	50-69 years	1,070<br>(949 to 1,200)	2,991.2<br>(2,649.7 to 3,362.2)	3,750<br>(3,340 to 4,170)	1,847.1<br>(1,647.9 to 2,053.8)	3,720<br>(3,260 to 4,230)	1,739.4<br>(1,522.2 to 1,975.8)	3,640<br>(3,210 to 4,080)	1,592.3<br>(1,406.2 to 1,784.1)	-38.3<br>(-43.9 to -31.2)	-5.8<br>(-15.9 to 4.8)
	70+ years	625<br>(546 to 722)	10,928.4<br>(9,548.6 to 12,613.3)	1,810<br>(1,590 to 2,040)	8,986.7<br>(7,901.3 to 10,130.1)	1,870<br>(1,630 to 2,180)	8,086.5<br>(7,033.6 to 9,410.0)	1,760<br>(1,570 to 2,000)	6,999.3<br>(6,216.4 to 7,954.0)	-17.8<br>(-27.0 to -8.8)	-10.0<br>(-19.8 to 1.6)

Location	Age Group	1990		2019		2020		2021		Percent Incidence Rate Change from 1990-2019 (95% UI)	Percent Incidence Rate Change from 2019-2021 (95% UI)
		Episodes (95% UI)	Rate (95% UI)	Episodes (95% UI)	Rate (95% UI)	Episodes (95% UI)	Rate (95% UI)	Episodes (95% UI)	Rate (95% UI)		
**Egypt**	All Ages	2,190,000 (1,980,000 to 2,410,000)	3,957.3 (3,574.4 to 4,359.2)	1,930,000 (1,790,000 to 2,090,000)	1,887.0 (1,746.0 to 2,043.9)	1,830,000 (1,690,000 to 1,990,000)	1,762.2 (1,628.4 to 1,915.8)	1,670,000 (1,540,000 to 1,810,000)	1,583.2 (1,458.4 to 1,718.0)	-52.3 (-55.4 to -49.0)	-6.6 (-12.7 to -0.8)
	Under 5	1,260,000 (1,070,000 to 1,460,000)	14,711.5 (12,566.6 to 17,084.7)	592,000 (498,000 to 704,000)	4,324.1 (3,637.3 to 5,136.4)	481,000 (405,000 to 569,000)	3,594.1 (3,023.6 to 4,255.6)	394,000 (327,000 to 472,000)	3,022.3 (2,510.8 to 3,619.5)	-70.6 (-73.5 to -67.6)	-16.9 (-28.6 to -7.7)
	5-14 years	283,000 (225,000 to 349,000)	2,071.2 (1,647.5 to 2,557.7)	256,000 (197,000 to 324,000)	1,138.9 (876.6 to 1,444.1)	251,000 (196,000 to 313,000)	1,082.2 (847.0 to 1,351.7)	239,000 (183,000 to 298,000)	1,002.4 (768.9 to 1,251.5)	-45.0 (-52.1 to -37.8)	-5.0 (-13.5 to 5.3)
	15-49 years	302,000 (269,000 to 338,000)	1,125.1 (1,001.3 to 1,260.6)	440,000 (389,000 to 498,000)	852.2 (754.2 to 965.4)	440,000 (389,000 to 497,000)	837.8 (740.2 to 945.8)	420,000 (377,000 to 470,000)	785.5 (704.5 to 879.6)	-24.3 (-29.4 to -18.7)	-1.7 (-10.4 to 7.6)
	50-69 years	209,000 (187,000 to 236,000)	3,913.3 (3,505.7 to 4,434.5)	405,000 (364,000 to 456,000)	3,243.5 (2,918.7 to 3,653.6)	414,000 (372,000 to 465,000)	3,234.6 (2,908.8 to 3,638.4)	390,000 (345,000 to 437,000)	2,991.0 (2,639.0 to 3,347.1)	-17.1 (-25.0 to -8.4)	-0.3 (-8.6 to 9.2)
	70+ years	141,000 (121,000 to 161,000)	14,360.8 (12,342.5 to 16,335.2)	238,000 (209,000 to 270,000)	11,390.5 (9,986.7 to 12,884.9)	248,000 (217,000 to 288,000)	11,359.5 (9,930.4 to 13,196.9)	229,000 (200,000 to 260,000)	10,095.2 (8,797.6 to 11,442.6)	-20.7 (-30.5 to -9.9)	-0.3 (-10.6 to 10.8)
**Iran (Islamic Republic of)**	All Ages	1,450,000 (1,320,000 to 1,580,000)	2,532.1 (2,311.4 to 2,768.8)	1,170,000 (1,100,000 to 1,240,000)	1,381.7 (1,302.3 to 1,464.1)	1,160,000 (1,070,000 to 1,250,000)	1,365.9 (1,261.0 to 1,467.8)	1,160,000 (1,080,000 to 1,250,000)	1,355.5 (1,268.3 to 1,458.9)	-45.4 (-49.1 to -41.2)	-1.1 (-6.4 to 2.5)
	Under 5	653,000 (558,000 to 761,000)	7,442.6 (6,356.3 to 8,671.5)	101,000 (85,400 to 118,000)	1,449.4 (1,231.1 to 1,695.8)	89,900 (73,900 to 107,000)	1,368.0 (1,124.3 to 1,623.8)	82,800 (68,800 to 98,600)	1,344.5 (1,118.3 to 1,601.4)	-80.5 (-82.0 to -78.9)	-5.6 (-16.2 to 0.7)
	5-14 years	264,000 (212,000 to 323,000)	1,588.9 (1,278.6 to 1,943.5)	111,000 (87,900 to 138,000)	827.2 (656.7 to 1,030.7)	111,000 (86,600 to 136,000)	806.9 (631.0 to 989.0)	111,000 (87,300 to 138,000)	793.2 (622.2 to 987.1)	-47.9 (-50.8 to -44.8)	-2.5 (-6.9 to 0.9)
	15-49 years	230,000 (205,000 to 256,000)	901.0 (802.1 to 1,004.3)	319,000 (286,000 to 354,000)	671.5 (603.5 to 745.2)	313,000 (279,000 to 349,000)	658.8 (588.6 to 736.2)	311,000 (281,000 to 346,000)	657.0 (593.0 to 730.0)	-25.5 (-28.9 to -21.9)	-1.9 (-6.3 to 1.6)
	50-69 years	182,000 (162,000 to 201,000)	3,485.5 (3,115.1 to 3,851.3)	292,000 (266,000 to 319,000)	2,226.5 (2,025.8 to 2,434.9)	299,000 (273,000 to 328,000)	2,197.4 (2,000.2 to 2,406.3)	306,000 (279,000 to 334,000)	2,165.0 (1,974.5 to 2,364.4)	-36.1 (-39.7 to -31.8)	-1.3 (-6.0 to 2.4)
	70+ years	117,000 (105,000 to 129,000)	12,074.7 (10,838.7 to 13,327.9)	344,000 (314,000 to 381,000)	9,878.7 (9,029.3 to 10,947.4)	348,000 (313,000 to 387,000)	9,702.5 (8,746.1 to 10,810.0)	346,000 (311,000 to 387,000)	9,468.4 (8,527.1 to 10,588.2)	-18.2 (-23.4 to -11.6)	-1.8 (-7.8 to 2.8)
**Iraq**	All Ages	632,000 (570,000 to 699,000)	3,430.4 (3,094.5 to 3,796.7)	581,000 (538,000 to 632,000)	1,455.6 (1,347.0 to 1,583.7)	553,000 (504,000 to 605,000)	1,363.5 (1,240.6 to 1,489.5)	518,000 (480,000 to 566,000)	1,256.4 (1,163.4 to 1,371.9)	-57.6 (-60.4 to -54.4)	-6.3 (-12.6 to 0.2)
	Under 5	344,000 (290,000 to 402,000)	10,934.9 (9,215.0 to 12,781.6)	159,000 (133,000 to 186,000)	3,550.0 (2,984.3 to 4,169.5)	139,000 (114,000 to 167,000)	3,181.0 (2,609.8 to 3,806.9)	120,000 (101,000 to 146,000)	2,803.8 (2,341.5 to 3,393.9)	-67.5 (-70.4 to -64.2)	-10.4 (-21.3 to 1.5)
	5-14 years	112,000 (89,200 to 140,000)	2,194.2 (1,751.0 to 2,743.4)	99,700 (77,500 to 127,000)	1,100.0 (855.2 to 1,398.2)	95,900 (75,200 to 122,000)	1,050.5 (824.0 to 1,340.1)	91,200 (70,900 to 114,000)	995.2 (772.9 to 1,242.4)	-49.9 (-55.5 to -43.3)	-4.5 (-12.5 to 4.8)
	15-49 years	83,300 (73,800 to 94,000)	984.0 (871.9 to 1,110.0)	148,000 (131,000 to 167,000)	698.5 (618.5 to 788.1)	144,000 (127,000 to 163,000)	664.9 (587.2 to 752.6)	140,000 (124,000 to 159,000)	632.9 (558.9 to 718.4)	-29.0 (-35.0 to -22.4)	-4.8 (-12.4 to 2.0)

Location	Age Group	1990		2019		2020		2021		Percent Incidence Rate Change from 1990-2019 (95% UI)	Percent Incidence Rate Change from 2019-2021 (95% UI)
		Episodes (95% UI)	Rate (95% UI)	Episodes (95% UI)	Rate (95% UI)	Episodes (95% UI)	Rate (95% UI)	Episodes (95% UI)	Rate (95% UI)		
	50-69 years	44,000 (38,100 to 49,900)	3,344.1 (2,898.1 to 3,795.2)	94,300 (83,800 to 106,000)	2,234.4 (1,985.2 to 2,522.1)	95,200 (84,100 to 106,000)	2,154.9 (1,902.2 to 2,389.8)	92,600 (83,200 to 104,000)	2,005.0 (1,802.2 to 2,246.1)	-33.2 (-40.9 to -25.1)	-3.6 (-13.5 to 7.3)
	70+ years	49,100 (43,600 to 55,500)	12,280.6 (10,903.1 to 13,901.8)	80,400 (71,600 to 92,400)	8,461.0 (7,536.0 to 9,732.3)	78,800 (69,000 to 88,400)	8,100.8 (7,097.6 to 9,093.1)	73,500 (64,600 to 83,500)	7,412.1 (6,514.1 to 8,423.8)	-31.1 (-39.3 to -21.6)	-4.3 (-14.7 to 6.2)
**Jordan**	All Ages	85,600 (77,900 to 94,400)	2,291.0 (2,085.9 to 2,527.3)	138,000 (128,000 to 149,000)	1,194.7 (1,113.2 to 1,292.3)	136,000 (121,000 to 149,000)	1,140.6 (1,018.2 to 1,250.5)	128,000 (118,000 to 140,000)	1,040.3 (958.8 to 1,133.3)	-47.9 (-51.4 to -43.9)	-4.5 (-12.6 to 3.7)
	Under 5	39,900 (34,300 to 46,900)	6,646.8 (5,711.3 to 7,819.6)	29,800 (25,400 to 34,900)	2,688.5 (2,294.6 to 3,147.9)	26,900 (21,300 to 33,300)	2,438.2 (1,934.7 to 3,020.6)	22,200 (18,500 to 26,700)	2,026.3 (1,683.8 to 2,430.9)	-59.6 (-63.5 to -55.6)	-9.3 (-24.8 to 5.3)
	5-14 years	13,600 (10,700 to 17,200)	1,313.4 (1,035.8 to 1,662.8)	19,400 (15,000 to 25,000)	782.5 (605.4 to 1,008.9)	18,900 (14,800 to 23,700)	750.9 (589.0 to 942.0)	17,700 (13,700 to 22,400)	698.1 (540.7 to 883.4)	-40.4 (-46.2 to -34.4)	-4.0 (-13.3 to 5.3)
	15-49 years	16,900 (14,900 to 19,000)	946.4 (834.5 to 1,067.6)	40,400 (36,100 to 45,300)	638.1 (570.2 to 715.0)	40,100 (35,000 to 45,600)	609.0 (530.9 to 691.3)	39,100 (34,600 to 44,100)	571.2 (506.3 to 644.8)	-32.6 (-38.2 to -25.7)	-4.6 (-12.4 to 4.0)
	50-69 years	8,820 (7,800 to 9,910)	3,247.9 (2,873.2 to 3,649.6)	25,700 (22,800 to 28,800)	1,968.2 (1,746.1 to 2,206.3)	27,400 (24,100 to 30,500)	1,918.7 (1,691.1 to 2,141.1)	27,400 (24,600 to 30,300)	1,777.1 (1,592.3 to 1,966.4)	-39.4 (-45.4 to -32.4)	-2.5 (-12.5 to 7.3)
	70+ years	6,440 (5,710 to 7,270)	13,129.7 (11,635.8 to 14,822.6)	22,200 (19,500 to 25,500)	7,983.7 (7,022.5 to 9,183.9)	22,800 (19,400 to 26,200)	7,805.0 (6,651.6 to 8,992.9)	21,800 (19,300 to 24,700)	7,091.5 (6,278.1 to 8,058.2)	-39.2 (-45.7 to -31.8)	-2.2 (-13.8 to 10.7)
**Kuwait**	All Ages	34,300 (31,300 to 37,200)	1,993.2 (1,820.7 to 2,167.0)	70,100 (65,500 to 74,900)	1,597.5 (1,494.4 to 1,708.2)	67,000 (60,900 to 74,200)	1,481.0 (1,346.4 to 1,640.7)	62,800 (57,900 to 67,900)	1,351.5 (1,244.6 to 1,459.7)	-19.8 (-24.9 to -13.8)	-7.3 (-15.5 to 1.5)
	Under 5	12,200 (10,100 to 14,400)	5,989.4 (4,953.6 to 7,063.3)	8,570 (7,230 to 10,300)	3,069.8 (2,590.8 to 3,689.4)	7,060 (5,610 to 8,850)	2,592.9 (2,058.1 to 3,250.1)	5,630 (4,600 to 6,800)	2,125.0 (1,735.1 to 2,567.2)	-48.7 (-54.4 to -41.2)	-15.5 (-29.7 to 1.4)
	5-14 years	5,070 (4,020 to 6,450)	1,448.0 (1,148.8 to 1,842.5)	5,960 (4,640 to 7,450)	1,058.3 (823.5 to 1,321.8)	5,610 (4,400 to 6,900)	978.9 (767.5 to 1,204.2)	5,210 (4,140 to 6,600)	898.2 (713.0 to 1,136.6)	-26.9 (-33.3 to -19.5)	-7.5 (-16.9 to 2.7)
	15-49 years	9,890 (8,710 to 11,200)	965.3 (850.4 to 1,090.3)	22,700 (19,900 to 25,700)	785.7 (689.2 to 889.5)	21,700 (19,000 to 24,900)	732.5 (638.6 to 837.9)	20,600 (18,200 to 23,300)	677.0 (598.7 to 765.7)	-18.6 (-25.9 to -11.2)	-6.8 (-15.8 to 2.7)
	50-69 years	3,920 (3,470 to 4,420)	3,277.5 (2,900.9 to 3,691.0)	15,800 (14,100 to 17,400)	2,822.8 (2,512.1 to 3,104.4)	16,700 (14,700 to 18,700)	2,723.5 (2,403.7 to 3,057.9)	16,400 (14,700 to 18,200)	2,482.3 (2,226.4 to 2,756.0)	-13.9 (-21.3 to -5.1)	-3.5 (-13.2 to 7.5)
	70+ years	3,130 (2,800 to 3,530)	15,671.5 (14,050.0 to 17,700.6)	17,000 (15,100 to 19,000)	17,906.9 (15,899.1 to 20,031.9)	15,900 (13,900 to 18,500)	16,725.7 (14,679.1 to 19,547.3)	15,000 (13,300 to 16,800)	15,182.2 (13,516.3 to 17,029.9)	14.3 (4.4 to 27.3)	-6.6 (-17.0 to 5.0)
**Lebanon**	All Ages	65,400 (59,900 to 71,200)	2,187.3 (2,003.2 to 2,379.3)	102,000 (94,600 to 110,000)	1,836.0 (1,705.0 to 1,974.0)	97,200 (88,400 to 107,000)	1,751.1 (1,593.1 to 1,933.6)	89,200 (82,700 to 96,600)	1,610.0 (1,492.9 to 1,743.9)	-16.1 (-21.4 to -10.3)	-4.6 (-12.5 to 4.3)
	Under 5	19,100 (16,200 to 22,900)	4,896.0 (4,146.4 to 5,869.9)	11,100 (9,090 to 13,500)	2,493.0 (2,039.2 to 3,030.1)	9,080 (7,100 to 11,300)	2,144.3 (1,676.1 to 2,664.3)	7,350 (5,870 to 8,930)	1,810.6 (1,446.5 to 2,200.7)	-49.1 (-53.7 to -44.1)	-14.0 (-24.7 to 0.4)

Location	Age Group	1990		2019		2020		2021		Percent Incidence Rate Change from 1990-2019 (95% UI)	Percent Incidence Rate Change from 2019-2021 (95% UI)
		Episodes (95% UI)	Rate (95% UI)	Episodes (95% UI)	Rate (95% UI)	Episodes (95% UI)	Rate (95% UI)	Episodes (95% UI)	Rate (95% UI)		
	5-14 years	8,560 (6,470 to 11,100)	1,305.1 (986.5 to 1,697.2)	8,800 (6,610 to 11,300)	1,009.1 (758.4 to 1,300.6)	8,340 (6,270 to 10,600)	952.9 (716.2 to 1,210.2)	7,720 (5,820 to 10,000)	885.5 (667.9 to 1,148.2)	-22.7 (-28.7 to -15.5)	-5.6 (-13.8 to 4.6)
	15-49 years	12,000 (10,500 to 13,600)	835.5 (733.3 to 948.9)	19,600 (17,200 to 22,300)	645.4 (566.8 to 735.2)	18,500 (16,200 to 21,000)	611.0 (535.3 to 693.8)	17,400 (15,400 to 19,600)	577.4 (511.2 to 651.9)	-22.8 (-28.2 to -16.7)	-5.3 (-13.3 to 3.2)
	50-69 years	12,800 (11,200 to 14,300)	3,097.5 (2,727.2 to 3,459.7)	18,700 (16,500 to 20,900)	2,265.6 (2,002.7 to 2,524.5)	18,500 (16,300 to 20,800)	2,181.4 (1,918.1 to 2,457.4)	17,400 (15,600 to 19,300)	2,014.3 (1,801.8 to 2,231.7)	-26.9 (-34.5 to -18.4)	-3.7 (-13.5 to 8.2)
	70+ years	13,000 (11,400 to 15,000)	13,606.7 (11,885.6 to 15,697.5)	43,700 (39,000 to 48,900)	11,699.1 (10,431.4 to 13,105.2)	42,800 (37,800 to 49,000)	11,271.1 (9,958.8 to 12,905.0)	39,300 (35,100 to 44,700)	10,227.2 (9,128.4 to 11,619.2)	-14.0 (-22.7 to -6.0)	-3.7 (-14.0 to 9.0)
**Libya**	All Ages	106,000 (96,400 to 117,000)	2,514.8 (2,286.1 to 2,771.5)	102,000 (95,100 to 110,000)	1,505.6 (1,398.3 to 1,611.5)	99,600 (91,300 to 109,000)	1,456.5 (1,335.0 to 1,597.4)	93,400 (86,700 to 101,000)	1,359.5 (1,261.7 to 1,464.5)	-40.1 (-45.1 to -35.0)	-3.3 (-10.0 to 4.9)
	Under 5	48,500 (40,500 to 57,300)	7,619.2 (6,356.9 to 9,003.6)	12,200 (10,200 to 14,700)	2,685.0 (2,236.3 to 3,222.1)	10,500 (8,540 to 12,900)	2,394.9 (1,946.4 to 2,947.0)	8,750 (7,200 to 10,700)	2,068.9 (1,702.5 to 2,519.1)	-64.8 (-68.0 to -60.9)	-10.8 (-22.4 to 4.7)
	5-14 years	16,600 (13,100 to 20,800)	1,413.1 (1,113.3 to 1,774.9)	10,500 (8,040 to 13,200)	935.5 (718.6 to 1,175.5)	9,830 (7,660 to 12,100)	897.8 (699.8 to 1,109.4)	9,040 (7,030 to 11,300)	846.4 (658.0 to 1,060.6)	-33.8 (-40.4 to -26.7)	-4.0 (-11.7 to 4.4)
	15-49 years	17,800 (15,800 to 20,000)	901.6 (799.9 to 1,012.0)	32,900 (29,200 to 36,600)	802.5 (711.5 to 894.0)	31,700 (28,300 to 35,800)	771.7 (688.8 to 871.3)	30,100 (26,800 to 33,900)	731.3 (650.9 to 822.3)	-11.0 (-18.8 to -2.8)	-3.8 (-11.8 to 5.8)
	50-69 years	11,000 (9,740 to 12,400)	3,268.7 (2,881.5 to 3,671.0)	23,600 (20,700 to 26,700)	2,583.2 (2,265.5 to 2,922.4)	24,500 (21,700 to 27,800)	2,511.8 (2,219.9 to 2,848.9)	24,100 (21,200 to 27,300)	2,325.0 (2,043.5 to 2,629.3)	-21.0 (-30.7 to -12.3)	-2.8 (-11.9 to 9.6)
	70+ years	12,000 (10,600 to 13,400)	13,791.4 (12,169.2 to 15,445.0)	23,200 (20,400 to 26,200)	10,856.6 (9,551.4 to 12,261.5)	23,000 (19,900 to 26,300)	10,668.1 (9,241.2 to 12,179.5)	21,300 (18,800 to 24,500)	9,740.2 (8,576.8 to 11,162.2)	-21.3 (-30.7 to -11.1)	-1.7 (-12.7 to 10.7)
**Morocco**	All Ages	852,000 (777,000 to 941,000)	3,361.0 (3,065.0 to 3,712.7)	750,000 (695,000 to 804,000)	2,044.3 (1,894.4 to 2,191.5)	707,000 (645,000 to 777,000)	1,913.7 (1,744.2 to 2,102.0)	651,000 (598,000 to 700,000)	1,751.7 (1,608.8 to 1,883.0)	-39.2 (-43.9 to -34.3)	-6.4 (-13.9 to 2.5)
	Under 5	421,000 (362,000 to 497,000)	11,825.7 (10,166.4 to 13,965.8)	128,000 (110,000 to 152,000)	3,730.9 (3,215.5 to 4,452.3)	108,000 (86,700 to 133,000)	3,244.0 (2,597.0 to 3,991.3)	89,800 (74,500 to 108,000)	2,761.4 (2,292.9 to 3,307.1)	-68.5 (-71.3 to -64.9)	-13.1 (-25.0 to 1.0)
	5-14 years	109,000 (85,400 to 138,000)	1,746.2 (1,372.0 to 2,217.6)	71,300 (54,300 to 89,900)	1,089.1 (829.9 to 1,373.7)	67,100 (52,000 to 83,300)	1,024.5 (794.5 to 1,272.5)	62,500 (48,300 to 77,400)	954.8 (737.7 to 1,183.5)	-37.6 (-44.6 to -30.4)	-5.9 (-14.9 to 5.9)
	15-49 years	128,000 (115,000 to 144,000)	1,029.2 (923.7 to 1,154.9)	161,000 (144,000 to 179,000)	837.7 (749.6 to 934.4)	152,000 (134,000 to 172,000)	786.2 (694.3 to 890.3)	143,000 (128,000 to 160,000)	736.5 (658.9 to 825.5)	-18.6 (-25.1 to -11.6)	-6.1 (-15.5 to 3.4)
	50-69 years	93,500 (82,700 to 105,000)	3,867.1 (3,420.4 to 4,327.3)	192,000 (168,000 to 213,000)	3,206.8 (2,815.9 to 3,564.9)	191,000 (169,000 to 217,000)	3,092.7 (2,736.3 to 3,518.0)	181,000 (161,000 to 201,000)	2,874.6 (2,552.1 to 3,194.8)	-17.1 (-26.6 to -7.3)	-3.6 (-12.9 to 8.2)
	70+ years	101,000 (88,300 to 115,000)	14,233.3 (12,450.5 to 16,220.3)	199,000 (175,000 to 227,000)	12,458.3 (10,968.3 to 14,206.8)	189,000 (163,000 to 219,000)	11,763.7 (10,131.3 to 13,617.9)	175,000 (155,000 to 198,000)	10,567.4 (9,367.6 to 12,002.7)	-12.5 (-23.8 to -2.3)	-5.6 (-16.9 to 7.6)

Location	Age Group	1990		2019		2020		2021		Percent Incidence Rate Change from 1990-2019 (95% UI)	Percent Incidence Rate Change from 2019-2021 (95% UI)
		Episodes (95% UI)	Rate (95% UI)	Episodes (95% UI)	Rate (95% UI)	Episodes (95% UI)	Rate (95% UI)	Episodes (95% UI)	Rate (95% UI)		
**Oman**	All Ages	58,000 (52,400 to 64,400)	2,924.3 (2,642.7 to 3,245.6)	57,000 (52,800 to 61,500)	1,319.7 (1,223.2 to 1,423.9)	49,900 (46,600 to 53,600)	1,105.1 (1,031.8 to 1,186.9)	51,500 (47,100 to 55,900)	1,094.0 (1,001.6 to 1,187.9)	-54.9 (-57.9 to -51.8)	-16.3 (-20.0 to -12.4)
	Under 5	28,000 (23,500 to 33,200)	8,543.8 (7,174.7 to 10,130.9)	11,300 (9,620 to 13,500)	2,699.6 (2,294.5 to 3,231.1)	8,100 (6,800 to 9,800)	1,916.2 (1,606.8 to 2,318.3)	8,130 (6,620 to 9,850)	1,917.9 (1,561.3 to 2,322.1)	-68.4 (-71.4 to -65.1)	-29.0 (-35.6 to -21.3)
	5-14 years	9,130 (7,280 to 11,300)	1,781.1 (1,420.4 to 2,213.5)	7,740 (6,110 to 9,790)	1,079.3 (851.6 to 1,365.1)	7,080 (5,430 to 8,920)	932.8 (715.1 to 1,175.8)	7,420 (5,830 to 9,440)	929.0 (729.6 to 1,181.4)	-39.4 (-45.6 to -32.4)	-13.6 (-20.7 to -5.0)
	15-49 years	10,300 (9,080 to 11,500)	1,030.5 (913.0 to 1,154.5)	18,100 (16,000 to 20,600)	657.5 (580.8 to 747.7)	16,500 (14,300 to 18,600)	571.9 (496.3 to 646.3)	17,100 (15,000 to 19,600)	571.7 (499.6 to 655.5)	-36.2 (-41.5 to -30.2)	-13.0 (-19.6 to -5.0)
	50-69 years	5,410 (4,860 to 6,070)	4,497.4 (4,042.7 to 5,051.6)	10,300 (9,200 to 11,300)	2,817.9 (2,529.1 to 3,114.7)	9,600 (8,580 to 10,600)	2,466.7 (2,205.2 to 2,724.6)	10,000 (9,030 to 11,100)	2,404.2 (2,160.9 to 2,660.2)	-37.3 (-44.1 to -30.1)	-12.5 (-19.2 to -5.7)
	70+ years	5,240 (4,610 to 5,930)	18,171.9 (15,968.4 to 20,554.5)	9,550 (8,520 to 10,900)	15,458.8 (13,804.1 to 17,646.2)	8,650 (7,630 to 9,850)	13,094.2 (11,544.9 to 14,906.2)	8,740 (7,650 to 9,940)	12,663.1 (11,077.1 to 14,392.1)	-14.9 (-25.2 to -6.3)	-15.3 (-22.8 to -7.4)
**Palestine**	All Ages	79,000 (71,700 to 87,500)	3,857.8 (3,505.1 to 4,275.6)	86,400 (79,200 to 93,400)	1,738.0 (1,592.7 to 1,879.4)	79,500 (71,800 to 88,200)	1,573.6 (1,419.8 to 1,745.4)	73,100 (67,600 to 79,500)	1,423.9 (1,315.5 to 1,548.9)	-54.9 (-57.8 to -51.7)	-9.5 (-16.4 to -1.0)
	Under 5	38,600 (32,400 to 45,800)	9,905.4 (8,320.3 to 11,738.4)	20,300 (17,000 to 24,300)	3,235.5 (2,702.8 to 3,864.8)	16,000 (12,900 to 19,800)	2,574.4 (2,073.3 to 3,189.5)	13,300 (11,000 to 16,000)	2,169.5 (1,790.2 to 2,603.0)	-67.3 (-70.4 to -62.7)	-20.4 (-31.2 to -6.1)
	5-14 years	13,000 (10,400 to 16,000)	2,246.2 (1,797.1 to 2,758.1)	16,800 (13,200 to 20,900)	1,354.3 (1,070.3 to 1,692.6)	15,500 (12,300 to 18,700)	1,238.8 (988.1 to 1,502.1)	14,400 (11,300 to 18,100)	1,144.9 (904.4 to 1,440.5)	-39.7 (-45.7 to -33.5)	-8.5 (-18.3 to 2.2)
	15-49 years	12,600 (11,200 to 14,000)	1,424.0 (1,265.9 to 1,583.4)	24,000 (21,300 to 26,500)	944.8 (837.5 to 1,044.5)	23,000 (20,100 to 26,200)	885.0 (774.8 to 1,008.4)	21,800 (19,400 to 24,300)	819.7 (731.3 to 915.7)	-33.6 (-37.9 to -27.9)	-6.3 (-14.7 to 3.8)
	50-69 years	7,260 (6,350 to 8,220)	4,861.8 (4,251.9 to 5,508.0)	13,900 (12,400 to 15,500)	2,963.5 (2,634.8 to 3,306.9)	13,900 (12,200 to 15,700)	2,837.6 (2,488.8 to 3,200.4)	13,400 (11,900 to 14,900)	2,625.4 (2,343.0 to 2,923.2)	-39.0 (-45.5 to -31.2)	-4.2 (-14.0 to 7.6)
	70+ years	7,450 (6,550 to 8,460)	17,675.0 (15,523.6 to 20,067.7)	11,400 (9,880 to 12,900)	11,691.8 (10,131.4 to 13,183.8)	11,200 (9,730 to 13,100)	11,146.3 (9,651.4 to 12,959.8)	10,300 (9,210 to 11,700)	9,949.8 (8,874.4 to 11,306.2)	-33.9 (-41.7 to -24.5)	-4.7 (-15.7 to 8.3)
**Qatar**	All Ages	7,120 (6,520 to 7,880)	1,600.5 (1,466.5 to 1,771.7)	26,100 (23,800 to 28,400)	923.8 (840.2 to 1,005.0)	23,400 (21,300 to 25,600)	805.4 (735.2 to 882.5)	21,900 (20,100 to 23,900)	736.8 (674.6 to 801.9)	-42.3 (-46.4 to -38.4)	-12.8 (-18.4 to -6.8)
	Under 5	2,600 (2,210 to 3,100)	5,119.6 (4,349.0 to 6,109.9)	4,130 (3,440 to 4,880)	2,358.8 (1,967.6 to 2,789.8)	3,210 (2,580 to 3,820)	1,784.0 (1,433.3 to 2,126.6)	2,720 (2,290 to 3,230)	1,475.6 (1,241.5 to 1,754.4)	-53.9 (-57.8 to -49.5)	-24.4 (-32.0 to -15.3)
	5-14 years	1,140 (881 to 1,450)	1,532.7 (1,186.8 to 1,953.0)	2,840 (2,200 to 3,620)	1,034.8 (798.4 to 1,315.5)	2,650 (2,020 to 3,370)	906.4 (690.9 to 1,150.8)	2,560 (1,990 to 3,300)	828.4 (641.4 to 1,065.6)	-32.5 (-38.2 to -26.5)	-12.4 (-19.4 to -3.4)
	15-49 years	2,480 (2,170 to 2,860)	852.4 (743.8 to 981.3)	13,000 (11,400 to 14,900)	616.2 (537.1 to 703.0)	11,800 (10,300 to 13,400)	547.2 (475.9 to 622.0)	11,100 (9,660 to 12,800)	508.3 (440.7 to 583.2)	-27.7 (-34.3 to -20.2)	-11.2 (-19.0 to -2.1)

Location	Age Group	1990		2019		2020		2021		Percent Incidence Rate Change from 1990-2019 (95% UI)	Percent Incidence Rate Change from 2019-2021 (95% UI)
		Episodes (95% UI)	Rate (95% UI)	Episodes (95% UI)	Rate (95% UI)	Episodes (95% UI)	Rate (95% UI)	Episodes (95% UI)	Rate (95% UI)		
	50-69 years	637 (549 to 739)	2,457.4 (2,119.4 to 2,851.2)	4,260 (3,780 to 4,780)	1,736.4 (1,540.3 to 1,949.3)	4,040 (3,570 to 4,560)	1,564.1 (1,381.5 to 1,766.6)	3,920 (3,480 to 4,380)	1,436.6 (1,275.0 to 1,609.0)	-29.3 (-36.8 to -19.8)	-9.9 (-18.5 to -0.9)
	70+ years	259 (227 to 292)	10,744.2 (9,394.0 to 12,114.9)	1,850 (1,670 to 2,080)	11,406.3 (10,319.7 to 12,804.4)	1,690 (1,490 to 1,920)	10,150.1 (8,966.5 to 11,548.9)	1,590 (1,420 to 1,770)	9,044.8 (8,100.8 to 10,071.2)	6.2 (-4.5 to 19.1)	-11.0 (-19.2 to -2.4)
**Saudi Arabia**	All Ages	365,000 (334,000 to 401,000)	2,300.8 (2,104.0 to 2,528.0)	541,000 (502,000 to 583,000)	1,494.0 (1,387.7 to 1,611.5)	517,000 (473,000 to 573,000)	1,399.2 (1,278.7 to 1,550.7)	488,000 (451,000 to 529,000)	1,294.5 (1,195.5 to 1,403.8)	-35.1 (-39.8 to -28.9)	-6.3 (-14.8 to 2.5)
	Under 5	145,000 (123,000 to 173,000)	6,005.9 (5,067.7 to 7,172.6)	53,500 (43,500 to 65,500)	2,118.2 (1,722.8 to 2,593.3)	44,900 (35,100 to 56,300)	1,809.1 (1,414.9 to 2,270.0)	36,400 (28,800 to 45,500)	1,496.5 (1,184.4 to 1,868.4)	-64.7 (-68.8 to -60.0)	-14.6 (-27.4 to 0.5)
	5-14 years	61,200 (47,100 to 79,100)	1,479.8 (1,139.1 to 1,913.8)	51,400 (38,700 to 67,000)	1,015.0 (763.6 to 1,321.8)	48,400 (36,400 to 61,100)	948.6 (712.7 to 1,197.3)	44,800 (33,400 to 57,700)	871.9 (651.3 to 1,123.8)	-31.4 (-39.4 to -23.0)	-6.5 (-15.5 to 2.8)
	15-49 years	76,300 (68,300 to 85,400)	951.9 (852.0 to 1,065.1)	238,000 (212,000 to 266,000)	979.4 (871.5 to 1,094.4)	228,000 (202,000 to 259,000)	919.8 (814.4 to 1,042.4)	218,000 (195,000 to 246,000)	863.2 (772.0 to 972.6)	2.9 (-6.1 to 12.9)	-6.1 (-16.1 to 3.9)
	50-69 years	44,100 (39,100 to 49,700)	4,258.4 (3,777.1 to 4,803.7)	140,000 (123,000 to 157,000)	3,664.3 (3,230.1 to 4,102.0)	139,000 (123,000 to 159,000)	3,434.4 (3,039.6 to 3,937.4)	137,000 (120,000 to 154,000)	3,162.4 (2,788.9 to 3,560.0)	-14.0 (-23.2 to -4.4)	-6.3 (-16.8 to 6.3)
	70+ years	38,000 (33,300 to 43,100)	15,305.5 (13,419.5 to 17,364.5)	57,800 (50,300 to 65,400)	12,184.2 (10,598.1 to 13,781.3)	56,600 (48,100 to 66,500)	11,176.2 (9,488.7 to 13,120.6)	51,900 (45,300 to 59,600)	9,998.9 (8,725.9 to 11,486.5)	-20.4 (-31.1 to -9.6)	-8.3 (-18.8 to 5.7)
**Sudan**	All Ages	727,000 (651,000 to 820,000)	3,631.5 (3,250.7 to 4,094.4)	668,000 (619,000 to 726,000)	1,595.1 (1,478.0 to 1,733.1)	617,000 (562,000 to 682,000)	1,446.0 (1,316.5 to 1,597.7)	574,000 (529,000 to 621,000)	1,322.6 (1,218.0 to 1,431.0)	-56.1 (-59.2 to -52.8)	-9.3 (-15.5 to -1.9)
	Under 5	417,000 (352,000 to 496,000)	11,970.3 (10,121.1 to 14,240.4)	206,000 (175,000 to 244,000)	3,580.8 (3,051.1 to 4,245.6)	169,000 (139,000 to 204,000)	2,959.1 (2,441.8 to 3,578.7)	147,000 (123,000 to 175,000)	2,600.6 (2,187.7 to 3,099.7)	-70.1 (-73.1 to -66.9)	-17.4 (-27.6 to -5.6)
	5-14 years	83,600 (65,200 to 104,000)	1,545.2 (1,205.6 to 1,925.2)	113,000 (90,600 to 142,000)	1,054.3 (843.1 to 1,324.5)	106,000 (84,800 to 133,000)	978.6 (780.9 to 1,220.5)	101,000 (80,500 to 126,000)	922.8 (735.4 to 1,150.0)	-31.8 (-39.9 to -23.1)	-7.2 (-15.9 to 3.7)
	15-49 years	99,900 (88,800 to 111,000)	1,096.6 (974.0 to 1,223.0)	162,000 (144,000 to 182,000)	762.3 (678.6 to 855.3)	157,000 (138,000 to 179,000)	720.6 (634.9 to 819.3)	153,000 (135,000 to 171,000)	682.1 (605.3 to 763.1)	-30.5 (-36.1 to -24.4)	-5.5 (-13.8 to 2.6)
	50-69 years	63,800 (56,400 to 72,100)	4,126.9 (3,649.6 to 4,666.8)	92,800 (82,100 to 104,000)	2,775.4 (2,456.4 to 3,111.9)	93,400 (81,900 to 105,000)	2,683.1 (2,353.3 to 3,027.3)	90,100 (78,600 to 100,000)	2,492.4 (2,173.6 to 2,779.9)	-32.7 (-38.9 to -25.2)	-3.3 (-12.7 to 6.7)
	70+ years	63,100 (54,700 to 71,400)	13,386.5 (11,607.8 to 15,156.8)	94,700 (84,100 to 108,000)	11,292.0 (10,025.0 to 12,865.2)	91,900 (80,300 to 105,000)	10,789.3 (9,431.4 to 12,333.9)	84,000 (74,000 to 94,900)	9,807.7 (8,645.2 to 11,082.7)	-15.6 (-26.1 to -5.4)	-4.5 (-14.1 to 7.4)
**Syrian Arab Republic**	All Ages	382,000 (342,000 to 423,000)	3,003.3 (2,685.3 to 3,326.0)	263,000 (242,000 to 282,000)	1,880.7 (1,731.9 to 2,017.4)	250,000 (229,000 to 273,000)	1,789.8 (1,635.6 to 1,953.3)	231,000 (216,000 to 248,000)	1,647.7 (1,538.2 to 1,770.7)	-37.4 (-42.6 to -31.4)	-4.8 (-11.3 to 2.7)
	Under 5	201,000 (168,000 to 240,000)	9,303.5 (7,786.1 to 11,118.9)	48,800 (40,700 to 59,300)	4,744.8 (3,960.0 to 5,772.1)	42,900 (34,500 to 52,700)	4,215.8 (3,389.6 to 5,186.1)	36,900 (30,100 to 45,500)	3,675.7 (2,993.9 to 4,531.5)	-49.0 (-55.2 to -42.7)	-11.2 (-23.4 to 3.1)

Location	Age Group	1990		2019		2020		2021		Percent Incidence Rate Change from 1990-2019 (95% UI)	Percent Incidence Rate Change from 2019-2021 (95% UI)
		Episodes (95% UI)	Rate (95% UI)	Episodes (95% UI)	Rate (95% UI)	Episodes (95% UI)	Rate (95% UI)	Episodes (95% UI)	Rate (95% UI)		
	5-14 years	72,200 (57,100 to 88,500)	1,917.1 (1,517.6 to 2,351.1)	37,300 (30,200 to 44,400)	1,247.2 (1,011.8 to 1,484.4)	33,400 (27,900 to 40,000)	1,185.1 (989.9 to 1,419.7)	29,500 (24,400 to 35,600)	1,111.3 (918.7 to 1,339.0)	-34.9 (-42.5 to -25.9)	-5.0 (-13.9 to 7.1)
	15-49 years	54,300 (48,000 to 61,000)	968.2 (856.6 to 1,088.0)	57,400 (51,100 to 64,300)	834.4 (743.2 to 935.3)	55,000 (49,000 to 61,400)	786.9 (702.0 to 878.8)	52,300 (46,900 to 58,400)	736.5 (660.6 to 822.5)	-13.8 (-21.2 to -6.3)	-5.7 (-12.9 to 2.1)
	50-69 years	30,200 (26,600 to 33,800)	3,114.5 (2,746.1 to 3,487.5)	67,100 (59,200 to 75,000)	2,666.5 (2,352.4 to 2,980.7)	67,100 (59,400 to 76,000)	2,582.6 (2,286.8 to 2,922.6)	64,100 (57,100 to 72,300)	2,393.9 (2,133.5 to 2,698.6)	-14.4 (-22.9 to -4.7)	-3.1 (-12.8 to 7.0)
	70+ years	24,600 (21,800 to 27,800)	11,064.3 (9,800.0 to 12,477.2)	52,100 (45,500 to 58,700)	9,453.0 (8,258.3 to 10,652.9)	52,000 (45,000 to 59,800)	9,111.7 (7,877.5 to 10,480.9)	48,300 (42,400 to 55,200)	8,181.0 (7,180.9 to 9,351.5)	-14.6 (-23.7 to -3.4)	-3.6 (-16.1 to 8.3)
**Tunisia**	All Ages	253,000 (232,000 to 279,000)	3,035.0 (2,779.1 to 3,341.2)	246,000 (228,000 to 265,000)	2,101.6 (1,946.0 to 2,264.9)	239,000 (219,000 to 261,000)	2,029.5 (1,862.5 to 2,215.2)	226,000 (209,000 to 243,000)	1,904.3 (1,761.7 to 2,053.3)	-30.8 (-36.5 to -25.2)	-3.4 (-10.1 to 4.3)
	Under 5	107,000 (90,400 to 127,000)	10,045.9 (8,474.8 to 11,946.6)	28,400 (23,500 to 34,200)	3,016.1 (2,496.2 to 3,628.4)	24,600 (20,100 to 30,200)	2,682.8 (2,184.2 to 3,290.5)	21,000 (17,300 to 25,100)	2,350.2 (1,940.7 to 2,814.3)	-70.0 (-73.0 to -66.4)	-11.1 (-21.7 to 2.4)
	5-14 years	39,700 (31,300 to 48,800)	1,945.8 (1,534.5 to 2,395.8)	21,400 (16,900 to 26,900)	1,167.0 (922.3 to 1,464.6)	20,800 (16,300 to 26,000)	1,119.6 (875.7 to 1,400.3)	19,900 (15,600 to 25,300)	1,059.9 (830.2 to 1,350.1)	-40.0 (-47.6 to -31.8)	-4.1 (-13.3 to 5.7)
	15-49 years	41,400 (36,000 to 46,900)	1,010.9 (878.4 to 1,144.1)	52,600 (46,300 to 59,300)	870.7 (766.3 to 981.9)	50,200 (44,500 to 56,100)	830.7 (737.3 to 928.7)	48,200 (43,200 to 54,600)	797.4 (714.8 to 903.8)	-13.9 (-21.0 to -6.4)	-4.6 (-12.3 to 5.2)
	50-69 years	33,300 (29,700 to 37,500)	3,672.3 (3,281.2 to 4,139.2)	64,200 (56,900 to 71,200)	2,880.3 (2,551.3 to 3,192.1)	64,400 (56,700 to 72,800)	2,814.9 (2,479.5 to 3,186.4)	62,100 (53,900 to 69,100)	2,659.1 (2,309.2 to 2,958.5)	-21.6 (-28.9 to -13.7)	-2.3 (-12.7 to 7.0)
	70+ years	31,900 (27,800 to 35,900)	13,181.8 (11,503.6 to 14,835.2)	79,300 (70,600 to 90,300)	12,112.1 (10,782.0 to 13,801.7)	79,200 (69,200 to 91,000)	11,651.0 (10,178.3 to 13,390.1)	74,400 (65,800 to 85,400)	10,682.8 (9,451.4 to 12,266.0)	-8.1 (-17.9 to 3.0)	-3.8 (-14.6 to 9.0)
**Türkiye**	All Ages	1,850,000 (1,690,000 to 2,030,000)	3,223.5 (2,947.1 to 3,527.9)	1,540,000 (1,440,000 to 1,650,000)	1,866.0 (1,737.6 to 1,992.3)	1,460,000 (1,330,000 to 1,610,000)	1,755.0 (1,599.8 to 1,930.8)	1,330,000 (1,240,000 to 1,440,000)	1,593.4 (1,479.2 to 1,720.0)	-42.1 (-46.8 to -36.7)	-5.9 (-13.8 to 3.9)
	Under 5	877,000 (746,000 to 1,020,000)	12,607.7 (10,722.6 to 14,653.8)	162,000 (138,000 to 192,000)	2,690.5 (2,295.1 to 3,193.7)	132,000 (105,000 to 165,000)	2,284.3 (1,810.6 to 2,842.2)	106,000 (86,600 to 127,000)	1,904.5 (1,559.5 to 2,294.2)	-78.7 (-80.6 to -76.2)	-15.1 (-27.8 to 2.5)
	5-14 years	252,000 (201,000 to 311,000)	1,865.0 (1,483.2 to 2,298.8)	124,000 (95,700 to 155,000)	962.4 (744.5 to 1,207.9)	117,000 (90,300 to 148,000)	901.0 (698.2 to 1,144.7)	108,000 (83,900 to 140,000)	832.1 (646.8 to 1,081.0)	-48.4 (-55.1 to -40.7)	-6.4 (-16.0 to 4.9)
	15-49 years	294,000 (258,000 to 332,000)	1,015.0 (891.5 to 1,146.2)	315,000 (276,000 to 356,000)	721.6 (631.7 to 814.8)	296,000 (259,000 to 340,000)	674.6 (591.2 to 776.6)	274,000 (239,000 to 309,000)	623.6 (544.1 to 702.9)	-28.9 (-34.8 to -21.3)	-6.5 (-14.4 to 1.5)
	50-69 years	227,000 (199,000 to 255,000)	3,494.2 (3,061.2 to 3,930.4)	404,000 (360,000 to 446,000)	2,640.3 (2,352.3 to 2,916.6)	400,000 (352,000 to 447,000)	2,547.1 (2,244.2 to 2,846.3)	375,000 (334,000 to 413,000)	2,330.5 (2,077.8 to 2,571.4)	-24.4 (-32.8 to -13.7)	-3.5 (-13.2 to 8.4)
	70+ years	202,000 (178,000 to 228,000)	13,467.4 (11,867.6 to 15,184.2)	537,000 (479,000 to 603,000)	11,245.5 (10,020.8 to 12,618.4)	516,000 (453,000 to 594,000)	10,445.8 (9,163.5 to 12,028.6)	470,000 (424,000 to 534,000)	9,245.7 (8,347.3 to 10,495.1)	-16.5 (-26.4 to -5.9)	-7.1 (-17.6 to 4.9)

Location	Age Group	1990		2019		2020		2021		Percent Incidence Rate Change from 1990-2019 (95% UI)	Percent Incidence Rate Change from 2019-2021 (95% UI)
		Episodes (95% UI)	Rate (95% UI)	Episodes (95% UI)	Rate (95% UI)	Episodes (95% UI)	Rate (95% UI)	Episodes (95% UI)	Rate (95% UI)		
**United Arab Emirates**	All Ages	31,500 (28,600 to 34,600)	1,685.1 (1,529.4 to 1,848.8)	99,200 (90,100 to 110,000)	1,044.1 (947.6 to 1,152.6)	95,600 (85,500 to 107,000)	999.4 (892.8 to 1,115.1)	91,500 (83,200 to 100,000)	949.9 (864.2 to 1,043.3)	-38.0 (-42.9 to -33.1)	-4.3 (-12.4 to 5.3)
	Under 5	10,700 (8,890 to 12,800)	4,677.8 (3,898.2 to 5,605.8)	11,300 (9,040 to 14,100)	2,386.1 (1,904.8 to 2,977.2)	9,440 (7,240 to 12,000)	2,071.7 (1,588.6 to 2,624.0)	7,570 (5,970 to 9,410)	1,748.9 (1,379.1 to 2,175.2)	-49.0 (-54.9 to -43.4)	-13.2 (-24.5 to 3.3)
	5-14 years	5,420 (4,100 to 7,060)	1,499.3 (1,135.7 to 1,954.3)	9,110 (6,760 to 12,200)	1,077.5 (799.8 to 1,438.3)	8,830 (6,510 to 11,600)	1,007.6 (743.6 to 1,319.7)	8,300 (6,140 to 10,900)	915.6 (677.5 to 1,198.8)	-28.1 (-35.5 to -21.2)	-6.5 (-15.7 to 3.8)
	15-49 years	9,130 (7,970 to 10,400)	774.2 (676.4 to 882.0)	49,000 (42,600 to 56,300)	694.7 (602.9 to 797.1)	45,700 (39,500 to 52,200)	656.1 (567.1 to 749.6)	42,500 (36,800 to 48,700)	618.6 (535.7 to 709.2)	-10.3 (-18.7 to -0.9)	-5.5 (-15.4 to 5.5)
	50-69 years	3,300 (2,960 to 3,720)	3,638.3 (3,264.0 to 4,107.9)	25,100 (22,400 to 28,500)	2,302.1 (2,049.9 to 2,612.9)	26,700 (23,500 to 30,400)	2,166.3 (1,909.9 to 2,465.6)	28,000 (24,600 to 31,500)	2,026.9 (1,785.1 to 2,281.5)	-36.7 (-43.5 to -30.1)	-5.9 (-16.7 to 6.6)
	70+ years	3,020 (2,680 to 3,410)	24,826.7 (22,015.7 to 27,987.7)	4,650 (4,040 to 5,270)	13,388.6 (11,609.3 to 15,168.5)	5,000 (4,230 to 5,720)	12,187.4 (10,306.7 to 13,932.2)	5,190 (4,500 to 5,970)	10,750.0 (9,331.8 to 12,372.3)	-46.1 (-51.5 to -39.5)	-9.0 (-22.1 to 5.2)
**Yemen**	All Ages	647,000 (575,000 to 732,000)	4,744.1 (4,214.6 to 5,369.6)	625,000 (577,000 to 678,000)	1,937.7 (1,787.5 to 2,100.6)	596,000 (541,000 to 661,000)	1,808.5 (1,641.7 to 2,005.9)	557,000 (514,000 to 605,000)	1,655.0 (1,528.5 to 1,798.0)	-59.2 (-62.5 to -55.5)	-6.7 (-13.4 to 1.4)
	Under 5	413,000 (350,000 to 491,000)	14,886.7 (12,591.8 to 17,666.7)	198,000 (169,000 to 233,000)	4,138.6 (3,538.7 to 4,890.8)	176,000 (145,000 to 213,000)	3,710.5 (3,072.7 to 4,498.5)	154,000 (130,000 to 183,000)	3,275.8 (2,774.9 to 3,889.1)	-72.2 (-75.1 to -68.9)	-10.3 (-20.3 to 1.6)
	5-14 years	89,100 (69,900 to 112,000)	2,062.9 (1,618.9 to 2,586.4)	110,000 (87,500 to 138,000)	1,243.1 (984.1 to 1,556.6)	106,000 (84,800 to 131,000)	1,180.5 (941.2 to 1,452.8)	101,000 (79,500 to 126,000)	1,110.1 (874.6 to 1,382.8)	-39.7 (-45.6 to -32.4)	-5.0 (-15.0 to 5.3)
	15-49 years	65,600 (57,600 to 74,700)	1,204.7 (1,059.2 to 1,373.5)	146,000 (130,000 to 164,000)	931.2 (832.1 to 1,046.5)	144,000 (126,000 to 162,000)	887.9 (781.0 to 1,000.8)	141,000 (126,000 to 158,000)	841.7 (753.2 to 947.0)	-22.7 (-29.4 to -15.2)	-4.6 (-13.1 to 4.8)
	50-69 years	46,300 (40,400 to 52,600)	5,120.5 (4,467.0 to 5,826.0)	91,000 (80,800 to 102,000)	3,847.3 (3,418.0 to 4,324.5)	91,800 (79,700 to 106,000)	3,739.5 (3,249.0 to 4,299.0)	88,200 (78,000 to 99,700)	3,479.1 (3,075.1 to 3,931.2)	-24.9 (-32.2 to -15.8)	-2.8 (-12.9 to 8.3)
	70+ years	32,600 (28,500 to 36,800)	16,719.4 (14,623.9 to 18,848.8)	80,400 (70,400 to 90,400)	14,047.3 (12,299.0 to 15,802.9)	78,800 (68,400 to 90,800)	13,504.5 (11,724.8 to 15,567.5)	73,000 (63,600 to 83,000)	12,278.1 (10,696.4 to 13,953.4)	-16.0 (-25.2 to -6.4)	-3.9 (-14.3 to 7.5)
**Bangladesh**	All Ages	15,900,000 (14,800,000 to 17,200,000)	14,605.8 (13,550.7 to 15,799.0)	11,400,000 (10,600,000 to 12,200,000)	7,001.8 (6,563.5 to 7,495.8)	10,300,000 (9,590,000 to 11,100,000)	6,313.1 (5,864.2 to 6,812.5)	10,600,000 (9,900,000 to 11,400,000)	6,449.6 (6,015.5 to 6,915.9)	-52.1 (-55.2 to -48.8)	-9.8 (-15.4 to -3.6)
	Under 5	6,070,000 (5,400,000 to 6,860,000)	32,050.8 (28,516.4 to 36,239.2)	1,270,000 (1,120,000 to 1,450,000)	8,387.3 (7,421.7 to 9,603.5)	966,000 (831,000 to 1,130,000)	6,553.4 (5,637.1 to 7,649.3)	928,000 (798,000 to 1,080,000)	6,459.2 (5,555.6 to 7,526.9)	-73.8 (-76.2 to -71.4)	-21.9 (-28.9 to -12.6)
	5-14 years	2,620,000 (2,110,000 to 3,250,000)	8,752.3 (7,034.2 to 10,828.4)	1,300,000 (1,090,000 to 1,550,000)	4,035.1 (3,390.6 to 4,820.9)	1,170,000 (959,000 to 1,430,000)	3,681.4 (3,017.9 to 4,492.3)	1,150,000 (949,000 to 1,370,000)	3,673.6 (3,023.4 to 4,371.8)	-53.9 (-60.4 to -46.2)	-8.8 (-19.7 to 1.4)

Location	Age Group	1990		2019		2020		2021		Percent Incidence Rate Change from 1990-2019 (95% UI)	Percent Incidence Rate Change from 2019-2021 (95% UI)
		Episodes (95% UI)	Rate (95% UI)	Episodes (95% UI)	Rate (95% UI)	Episodes (95% UI)	Rate (95% UI)	Episodes (95% UI)	Rate (95% UI)		
	15-49 years	3,600,000 (3,230,000 to 4,020,000)	7,209.6 (6,463.2 to 8,036.1)	3,220,000 (2,850,000 to 3,620,000)	3,741.8 (3,312.5 to 4,212.7)	2,960,000 (2,620,000 to 3,280,000)	3,399.0 (3,010.9 to 3,770.3)	3,030,000 (2,690,000 to 3,440,000)	3,441.1 (3,059.3 to 3,907.9)	-48.1 (-52.4 to -43.4)	-9.2 (-16.1 to -1.0)
	50-69 years	2,000,000 (1,740,000 to 2,260,000)	25,138.5 (21,887.7 to 28,378.1)	2,850,000 (2,520,000 to 3,180,000)	12,919.4 (11,426.0 to 14,422.9)	2,640,000 (2,330,000 to 2,970,000)	11,598.2 (10,217.2 to 13,025.7)	2,780,000 (2,470,000 to 3,120,000)	11,825.0 (10,506.7 to 13,242.2)	-48.6 (-53.8 to -43.0)	-10.2 (-19.4 to -1.0)
	70+ years	1,640,000 (1,430,000 to 1,870,000)	72,217.9 (63,004.5 to 82,370.6)	2,720,000 (2,400,000 to 3,060,000)	39,848.4 (35,241.1 to 44,841.0)	2,580,000 (2,260,000 to 2,960,000)	36,218.2 (31,657.6 to 41,521.6)	2,730,000 (2,420,000 to 3,100,000)	37,000.0 (32,788.0 to 42,092.1)	-44.8 (-50.6 to -37.5)	-9.1 (-18.7 to 3.2)
**Bhutan**	All Ages	66,100 (60,800 to 72,000)	10,499.0 (9,656.5 to 11,429.8)	46,100 (42,900 to 49,600)	6,071.2 (5,653.8 to 6,531.0)	42,700 (39,700 to 45,800)	5,629.0 (5,229.3 to 6,044.9)	44,200 (40,600 to 47,700)	5,834.9 (5,368.5 to 6,308.2)	-42.2 (-46.1 to -37.8)	-7.3 (-12.4 to -1.7)
	Under 5	26,500 (22,900 to 30,800)	27,733.4 (24,009.4 to 32,227.7)	5,380 (4,680 to 6,290)	8,424.2 (7,333.5 to 9,855.2)	4,490 (3,870 to 5,210)	7,200.9 (6,208.9 to 8,356.9)	4,390 (3,780 to 5,190)	7,189.1 (6,202.1 to 8,505.5)	-69.6 (-72.4 to -66.3)	-14.5 (-22.3 to -5.1)
	5-14 years	8,420 (6,500 to 10,700)	5,051.8 (3,900.1 to 6,415.0)	3,650 (2,910 to 4,610)	2,802.1 (2,235.8 to 3,539.6)	3,320 (2,630 to 4,120)	2,592.3 (2,054.8 to 3,215.4)	3,300 (2,600 to 4,160)	2,620.0 (2,060.9 to 3,294.0)	-44.5 (-51.2 to -37.3)	-7.5 (-15.0 to 0.6)
	15-49 years	16,100 (14,100 to 18,200)	5,174.1 (4,535.0 to 5,845.6)	13,500 (11,800 to 15,400)	3,066.7 (2,692.7 to 3,499.8)	12,500 (10,900 to 14,100)	2,847.0 (2,491.9 to 3,202.9)	12,700 (11,300 to 14,200)	2,904.3 (2,581.4 to 3,249.8)	-40.7 (-45.8 to -35.8)	-7.2 (-14.6 to 0.8)
	50-69 years	9,740 (8,390 to 11,200)	20,758.0 (17,886.5 to 23,898.7)	11,100 (9,840 to 12,400)	11,689.0 (10,334.6 to 13,063.2)	10,600 (9,280 to 12,000)	10,800.4 (9,502.9 to 12,256.1)	11,100 (9,840 to 12,600)	11,129.2 (9,843.6 to 12,584.9)	-43.7 (-49.3 to -37.1)	-7.6 (-16.3 to 2.5)
	70+ years	5,370 (4,620 to 6,270)	59,887.7 (51,540.1 to 69,915.1)	12,500 (10,800 to 14,400)	40,925.6 (35,460.3 to 47,181.2)	11,800 (10,300 to 13,400)	37,887.4 (33,066.1 to 43,064.8)	12,600 (11,000 to 14,600)	39,217.3 (33,989.5 to 45,376.9)	-31.7 (-39.8 to -22.8)	-7.4 (-15.9 to 2.7)
**India**	All Ages	108,000,000 (101,000,000 to 116,000,000)	12,709.7 (11,875.4 to 13,574.3)	145,000,000 (137,000,000 to 155,000,000)	10,470.5 (9,837.8 to 11,146.8)	133,000,000 (125,000,000 to 141,000,000)	9,477.1 (8,939.0 to 10,043.0)	139,000,000 (130,000,000 to 151,000,000)	9,853.6 (9,184.6 to 10,650.7)	-17.6 (-21.4 to -13.9)	-9.5 (-11.8 to -7.0)
	Under 5	25,100,000 (22,200,000 to 28,100,000)	21,634.6 (19,101.1 to 24,202.9)	10,700,000 (9,630,000 to 12,000,000)	9,313.2 (8,352.7 to 10,440.9)	10,400,000 (9,130,000 to 11,800,000)	9,139.1 (8,057.8 to 10,390.0)	10,400,000 (9,030,000 to 12,100,000)	9,327.0 (8,114.3 to 10,910.7)	-57.0 (-58.5 to -55.4)	-1.9 (-6.6 to 3.7)
	5-14 years	15,600,000 (12,700,000 to 19,000,000)	7,409.4 (6,027.4 to 9,020.5)	11,000,000 (9,000,000 to 13,200,000)	4,246.9 (3,468.1 to 5,097.3)	10,300,000 (8,380,000 to 12,500,000)	4,003.3 (3,254.3 to 4,836.7)	10,300,000 (8,410,000 to 12,600,000)	4,050.9 (3,296.7 to 4,950.5)	-42.7 (-47.1 to -38.6)	-5.7 (-8.9 to -2.2)
	15-49 years	28,200,000 (25,300,000 to 31,100,000)	6,702.1 (6,013.5 to 7,404.1)	43,200,000 (39,300,000 to 47,400,000)	5,684.3 (5,170.9 to 6,241.5)	39,100,000 (35,300,000 to 42,900,000)	5,073.3 (4,588.6 to 5,573.8)	40,400,000 (36,400,000 to 44,600,000)	5,182.4 (4,666.7 to 5,725.7)	-15.2 (-17.9 to -12.3)	-10.7 (-13.0 to -8.4)
	50-69 years	24,200,000 (21,600,000 to 26,800,000)	27,419.1 (24,485.7 to 30,395.3)	43,200,000 (39,100,000 to 47,600,000)	21,817.7 (19,750.1 to 24,041.6)	39,300,000 (35,600,000 to 42,900,000)	19,236.8 (17,430.2 to 21,024.7)	41,500,000 (37,600,000 to 45,700,000)	19,883.0 (18,015.4 to 21,899.0)	-20.4 (-24.8 to -16.2)	-11.8 (-14.4 to -9.3)

Location	Age Group	1990		2019		2020		2021		Percent Incidence Rate Change from 1990-2019 (95% UI)	Percent Incidence Rate Change from 2019-2021 (95% UI)
		Episodes (95% UI)	Rate (95% UI)	Episodes (95% UI)	Rate (95% UI)	Episodes (95% UI)	Rate (95% UI)	Episodes (95% UI)	Rate (95% UI)		
	70+ years	15,400,000 (13,500,000 to 17,400,000)	85,233.1 (74,977.5 to 96,467.1)	37,200,000 (33,200,000 to 42,600,000)	66,275.4 (59,129.0 to 75,822.3)	33,900,000 (30,300,000 to 38,400,000)	58,837.9 (52,498.9 to 66,533.0)	36,700,000 (32,300,000 to 42,100,000)	61,648.2 (54,239.7 to 70,706.9)	-22.2 (-27.7 to -16.2)	-11.2 (-14.4 to -7.5)
**Nepal**	All Ages	3,470,000 (3,210,000 to 3,750,000)	17,827.4 (16,468.4 to 19,266.3)	3,370,000 (3,140,000 to 3,600,000)	11,033.9 (10,272.7 to 11,807.1)	3,190,000 (2,930,000 to 3,470,000)	10,330.1 (9,500.8 to 11,256.2)	3,110,000 (2,870,000 to 3,360,000)	9,984.1 (9,220.9 to 10,797.5)	-38.1 (-42.2 to -33.6)	-6.4 (-12.6 to -0.1)
	Under 5	1,430,000 (1,250,000 to 1,630,000)	43,297.2 (37,964.7 to 49,425.9)	420,000 (371,000 to 476,000)	13,591.1 (11,995.6 to 15,390.1)	365,000 (314,000 to 423,000)	11,765.9 (10,138.0 to 13,625.8)	330,000 (286,000 to 380,000)	10,619.8 (9,190.9 to 12,240.2)	-68.6 (-71.4 to -65.8)	-13.4 (-22.5 to -3.7)
	5-14 years	459,000 (364,000 to 563,000)	8,943.5 (7,089.4 to 10,971.1)	360,000 (284,000 to 438,000)	5,812.8 (4,582.7 to 7,072.6)	339,000 (269,000 to 418,000)	5,507.5 (4,367.7 to 6,803.6)	324,000 (258,000 to 396,000)	5,290.5 (4,222.3 to 6,463.9)	-35.0 (-42.1 to -26.6)	-5.3 (-14.4 to 5.6)
	15-49 years	726,000 (645,000 to 815,000)	8,159.0 (7,243.4 to 9,158.1)	942,000 (833,000 to 1,050,000)	5,813.3 (5,139.3 to 6,486.3)	900,000 (792,000 to 1,000,000)	5,478.3 (4,819.2 to 6,099.9)	880,000 (786,000 to 981,000)	5,287.4 (4,722.9 to 5,890.6)	-28.7 (-35.1 to -21.5)	-5.8 (-13.0 to 2.1)
	50-69 years	538,000 (466,000 to 612,000)	30,261.3 (26,213.1 to 34,428.8)	877,000 (782,000 to 987,000)	22,332.1 (19,931.7 to 25,136.6)	828,000 (734,000 to 937,000)	20,633.3 (18,277.7 to 23,336.6)	832,000 (738,000 to 945,000)	20,334.7 (18,046.7 to 23,087.0)	-26.2 (-35.0 to -17.7)	-7.6 (-16.0 to 2.4)
	70+ years	322,000 (273,000 to 369,000)	88,191.0 (74,784.9 to 101,284.8)	768,000 (668,000 to 883,000)	69,715.3 (60,575.8 to 80,109.7)	754,000 (651,000 to 873,000)	66,110.8 (57,103.8 to 76,593.5)	742,000 (651,000 to 854,000)	63,701.7 (55,880.9 to 73,338.7)	-20.9 (-30.9 to -11.4)	-5.2 (-15.0 to 6.8)
**Pakistan**	All Ages	15,300,000 (14,300,000 to 16,300,000)	13,798.1 (12,823.7 to 14,702.7)	20,000,000 (18,600,000 to 21,200,000)	8,853.9 (8,266.3 to 9,411.6)	18,500,000 (17,300,000 to 19,700,000)	8,007.3 (7,501.5 to 8,542.7)	18,900,000 (17,700,000 to 20,200,000)	8,041.1 (7,502.2 to 8,587.2)	-35.8 (-38.7 to -33.1)	-9.6 (-13.3 to -5.6)
	Under 5	5,730,000 (5,050,000 to 6,490,000)	31,011.7 (27,331.4 to 35,163.1)	4,420,000 (3,920,000 to 4,990,000)	15,039.5 (13,346.4 to 16,983.2)	3,710,000 (3,270,000 to 4,220,000)	12,529.8 (11,039.4 to 14,254.5)	3,620,000 (3,150,000 to 4,130,000)	12,185.9 (10,603.1 to 13,879.3)	-51.5 (-54.3 to -48.5)	-16.7 (-22.2 to -10.8)
	5-14 years	1,930,000 (1,530,000 to 2,380,000)	6,256.1 (4,957.6 to 7,726.8)	3,010,000 (2,440,000 to 3,650,000)	5,605.8 (4,555.1 to 6,812.5)	2,790,000 (2,290,000 to 3,390,000)	5,092.1 (4,182.4 to 6,204.3)	2,810,000 (2,280,000 to 3,410,000)	5,048.1 (4,095.5 to 6,117.6)	-10.4 (-17.4 to -2.7)	-9.2 (-15.2 to -2.5)
	15-49 years	3,160,000 (2,780,000 to 3,540,000)	6,383.0 (5,622.1 to 7,168.4)	5,830,000 (5,190,000 to 6,480,000)	5,027.1 (4,471.6 to 5,588.7)	5,530,000 (4,950,000 to 6,080,000)	4,651.3 (4,162.7 to 5,113.8)	5,750,000 (5,180,000 to 6,340,000)	4,711.9 (4,243.0 to 5,194.8)	-21.2 (-25.9 to -16.5)	-7.5 (-12.5 to -2.1)
	50-69 years	2,300,000 (2,010,000 to 2,600,000)	23,901.7 (20,882.2 to 27,082.7)	3,680,000 (3,250,000 to 4,120,000)	17,115.6 (15,112.6 to 19,155.6)	3,540,000 (3,160,000 to 3,980,000)	15,859.3 (14,173.2 to 17,837.9)	3,770,000 (3,330,000 to 4,250,000)	16,360.0 (14,435.1 to 18,419.3)	-28.4 (-33.9 to -21.7)	-7.3 (-12.8 to -0.6)
	70+ years	2,230,000 (1,930,000 to 2,540,000)	78,835.7 (68,266.9 to 89,828.3)	3,020,000 (2,640,000 to 3,450,000)	61,766.8 (53,992.1 to 70,653.8)	2,900,000 (2,530,000 to 3,310,000)	58,018.1 (50,631.8 to 66,314.2)	2,990,000 (2,620,000 to 3,400,000)	58,964.3 (51,705.8 to 67,228.7)	-21.7 (-28.8 to -13.4)	-6.1 (-13.4 to 1.1)
**China**	All Ages	50,900,000 (47,500,000 to 54,600,000)	4,329.7 (4,040.2 to 4,640.8)	50,000,000 (46,600,000 to 53,600,000)	3,534.5 (3,297.9 to 3,788.8)	46,100,000 (42,900,000 to 49,400,000)	3,244.7 (3,019.2 to 3,478.5)	44,700,000 (41,800,000 to 47,800,000)	3,142.1 (2,936.6 to 3,358.6)	-18.4 (-23.6 to -13.6)	-8.2 (-11.1 to -5.1)

Location	Age Group	1990		2019		2020		2021		Percent Incidence Rate Change from 1990-2019 (95% UI)	Percent Incidence Rate Change from 2019-2021 (95% UI)
		Episodes (95% UI)	Rate (95% UI)	Episodes (95% UI)	Rate (95% UI)	Episodes (95% UI)	Rate (95% UI)	Episodes (95% UI)	Rate (95% UI)		
	Under 5	18,600,000 (16,400,000 to 21,100,000)	16,638.3 (14,661.9 to 18,916.4)	3,550,000 (2,970,000 to 4,160,000)	4,035.0 (3,374.6 to 4,726.4)	2,960,000 (2,450,000 to 3,570,000)	3,511.9 (2,909.9 to 4,233.3)	2,400,000 (2,000,000 to 2,830,000)	3,088.8 (2,571.9 to 3,649.7)	-75.7 (-78.1 to -73.2)	-13.0 (-18.7 to -5.8)
	5-14 years	7,190,000 (5,880,000 to 8,820,000)	3,482.7 (2,844.3 to 4,269.1)	4,110,000 (3,220,000 to 5,120,000)	2,420.8 (1,899.0 to 3,017.2)	4,020,000 (3,170,000 to 5,050,000)	2,289.7 (1,802.6 to 2,871.1)	4,050,000 (3,190,000 to 5,060,000)	2,226.4 (1,750.4 to 2,781.5)	-30.5 (-37.3 to -22.4)	-5.4 (-8.7 to -2.0)
	15-49 years	8,460,000 (7,540,000 to 9,410,000)	1,268.3 (1,131.1 to 1,411.8)	7,950,000 (6,980,000 to 9,010,000)	1,159.2 (1,017.8 to 1,314.1)	7,230,000 (6,330,000 to 8,170,000)	1,072.2 (938.5 to 1,212.8)	6,900,000 (6,040,000 to 7,820,000)	1,040.9 (910.9 to 1,178.6)	-8.6 (-12.5 to -4.0)	-7.5 (-10.3 to -4.3)
	50-69 years	8,710,000 (7,840,000 to 9,610,000)	5,665.6 (5,098.6 to 6,251.1)	12,900,000 (11,700,000 to 14,200,000)	3,564.1 (3,216.9 to 3,913.6)	12,300,000 (11,200,000 to 13,500,000)	3,299.4 (2,994.6 to 3,633.1)	12,000,000 (10,800,000 to 13,100,000)	3,149.9 (2,830.7 to 3,445.5)	-37.1 (-39.3 to -34.3)	-7.4 (-10.4 to -4.3)
	70+ years	7,970,000 (7,070,000 to 8,850,000)	21,247.8 (18,849.8 to 23,580.1)	21,500,000 (19,100,000 to 23,900,000)	19,774.4 (17,607.9 to 22,001.2)	19,600,000 (17,400,000 to 21,800,000)	17,299.9 (15,424.8 to 19,241.9)	19,400,000 (17,400,000 to 21,500,000)	16,231.1 (14,550.4 to 17,980.8)	-6.9 (-13.5 to -0.1)	-12.5 (-15.8 to -8.8)
**Democratic People's Republic of Korea**	All Ages	1,080,000 (1,000,000 to 1,170,000)	5,251.1 (4,856.0 to 5,682.3)	887,000 (819,000 to 963,000)	3,374.4 (3,117.1 to 3,665.7)	823,000 (763,000 to 895,000)	3,125.0 (2,897.9 to 3,395.9)	798,000 (742,000 to 864,000)	3,024.3 (2,811.4 to 3,274.8)	-35.7 (-40.5 to -29.9)	-7.4 (-12.8 to -1.0)
	Under 5	452,000 (391,000 to 524,000)	19,355.7 (16,757.1 to 22,411.9)	79,900 (68,200 to 94,000)	5,028.7 (4,295.9 to 5,918.9)	68,900 (58,400 to 81,600)	4,438.3 (3,760.7 to 5,259.9)	58,600 (49,700 to 69,600)	3,874.6 (3,283.8 to 4,599.6)	-74.0 (-76.4 to -71.4)	-11.7 (-19.9 to -3.2)
	5-14 years	133,000 (106,000 to 166,000)	3,682.4 (2,928.8 to 4,586.7)	67,000 (52,600 to 83,500)	1,987.4 (1,560.2 to 2,476.6)	62,000 (48,500 to 78,400)	1,871.1 (1,462.4 to 2,366.3)	58,000 (45,000 to 71,900)	1,778.1 (1,379.0 to 2,204.3)	-46.0 (-52.1 to -39.6)	-5.9 (-14.4 to 4.4)
	15-49 years	155,000 (138,000 to 175,000)	1,440.5 (1,281.4 to 1,630.4)	151,000 (135,000 to 171,000)	1,082.9 (962.8 to 1,220.1)	139,000 (123,000 to 156,000)	1,000.1 (884.7 to 1,119.6)	132,000 (117,000 to 147,000)	956.3 (843.5 to 1,065.0)	-24.8 (-30.5 to -18.0)	-7.7 (-14.6 to -0.4)
	50-69 years	191,000 (169,000 to 214,000)	5,883.3 (5,210.3 to 6,588.7)	253,000 (224,000 to 289,000)	4,531.0 (4,000.8 to 5,161.4)	247,000 (220,000 to 280,000)	4,236.6 (3,779.2 to 4,801.1)	245,000 (218,000 to 277,000)	4,064.6 (3,614.1 to 4,589.3)	-23.0 (-30.8 to -13.8)	-6.5 (-15.0 to 3.0)
	70+ years	150,000 (131,000 to 170,000)	22,976.3 (20,033.8 to 25,966.1)	335,000 (298,000 to 382,000)	19,227.0 (17,119.8 to 21,905.4)	306,000 (272,000 to 343,000)	17,646.8 (15,692.2 to 19,775.4)	304,000 (270,000 to 345,000)	17,364.2 (15,416.7 to 19,689.8)	-16.3 (-25.7 to -5.8)	-8.2 (-16.4 to 2.4)
**Taiwan (Province of China)**	All Ages	816,000 (749,000 to 888,000)	4,002.5 (3,673.3 to 4,355.4)	1,270,000 (1,190,000 to 1,360,000)	5,369.3 (5,016.2 to 5,771.2)	1,130,000 (1,030,000 to 1,230,000)	4,785.3 (4,358.0 to 5,219.5)	1,070,000 (1,000,000 to 1,160,000)	4,536.8 (4,241.8 to 4,887.1)	34.1 (23.3 to 46.1)	-10.9 (-17.4 to -4.1)
	Under 5	154,000 (128,000 to 183,000)	9,577.3 (7,945.1 to 11,420.8)	52,100 (42,500 to 62,400)	5,298.9 (4,329.0 to 6,351.3)	39,300 (31,500 to 49,000)	4,192.3 (3,360.9 to 5,226.5)	31,100 (25,500 to 38,400)	3,483.4 (2,854.9 to 4,300.9)	-44.7 (-52.0 to -38.1)	-20.9 (-30.8 to -10.0)
	5-14 years	114,000 (87,300 to 146,000)	2,916.3 (2,237.5 to 3,743.9)	51,000 (39,600 to 65,300)	2,491.6 (1,937.2 to 3,192.4)	46,500 (35,600 to 60,900)	2,270.7 (1,739.0 to 2,971.5)	43,400 (33,300 to 55,300)	2,112.4 (1,621.9 to 2,691.3)	-14.6 (-21.4 to -6.5)	-8.9 (-17.1 to -0.1)
	15-49 years	135,000 (120,000 to 151,000)	1,200.9 (1,062.4 to 1,342.4)	129,000 (114,000 to 142,000)	1,100.8 (979.1 to 1,213.3)	113,000 (101,000 to 126,000)	981.6 (873.2 to 1,096.1)	106,000 (95,500 to 119,000)	935.1 (839.5 to 1,044.1)	-8.3 (-16.4 to -0.7)	-10.8 (-19.1 to -1.4)
	50-69 years	215,000 (192,000 to 237,000)	7,416.3 (6,636.2 to 8,196.6)	375,000 (338,000 to 417,000)	5,624.2 (5,070.8 to 6,255.1)	342,000 (298,000 to 381,000)	5,063.0 (4,416.1 to 5,634.9)	324,000 (294,000 to 359,000)	4,769.0 (4,334.1 to 5,279.1)	-24.2 (-31.5 to -15.5)	-10.0 (-19.5 to -1.6)

Location	Age Group	1990		2019		2020		2021		Percent Incidence Rate Change from 1990-2019 (95% UI)	Percent Incidence Rate Change from 2019-2021 (95% UI)
		Episodes (95% UI)	Rate (95% UI)	Episodes (95% UI)	Rate (95% UI)	Episodes (95% UI)	Rate (95% UI)	Episodes (95% UI)	Rate (95% UI)		
	70+ years	199,000 (173,000 to 220,000)	27,225.7 (23,710.0 to 30,223.7)	663,000 (599,000 to 731,000)	29,403.3 (26,584.1 to 32,459.9)	590,000 (523,000 to 662,000)	24,869.8 (22,023.1 to 27,872.2)	568,000 (517,000 to 633,000)	22,474.4 (20,458.2 to 25,047.6)	8.0 (-2.4 to 20.6)	-15.4 (-23.4 to -6.9)
**American Samoa**	All Ages	1,680 (1,560 to 1,820)	3,462.7 (3,212.9 to 3,750.1)	1,420 (1,320 to 1,510)	2,766.2 (2,582.4 to 2,956.1)	1,300 (1,210 to 1,390)	2,582.5 (2,402.7 to 2,753.9)	1,220 (1,140 to 1,300)	2,452.8 (2,295.5 to 2,612.4)	-20.1 (-25.0 to -14.9)	-6.6 (-11.4 to -1.6)
	Under 5	632 (547 to 736)	8,288.2 (7,181.9 to 9,663.1)	192 (165 to 225)	4,563.3 (3,909.8 to 5,335.5)	162 (137 to 188)	4,096.8 (3,474.2 to 4,768.8)	136 (117 to 159)	3,669.6 (3,157.9 to 4,280.2)	-44.9 (-49.9 to -40.4)	-10.2 (-17.0 to -1.9)
	5-14 years	227 (181 to 282)	1,987.1 (1,583.1 to 2,474.8)	159 (126 to 195)	1,421.0 (1,122.8 to 1,745.9)	144 (114 to 183)	1,331.4 (1,047.5 to 1,690.0)	130 (104 to 160)	1,244.8 (989.4 to 1,529.9)	-28.5 (-34.3 to -21.8)	-6.3 (-13.4 to 2.0)
	15-49 years	404 (362 to 449)	1,658.6 (1,487.6 to 1,845.9)	350 (317 to 390)	1,423.6 (1,289.8 to 1,588.0)	323 (295 to 358)	1,332.1 (1,214.7 to 1,474.5)	303 (272 to 332)	1,261.7 (1,136.1 to 1,383.9)	-14.2 (-21.8 to -6.1)	-6.4 (-13.4 to 1.3)
	50-69 years	251 (225 to 280)	5,892.1 (5,285.1 to 6,574.1)	385 (343 to 426)	4,251.8 (3,780.1 to 4,695.9)	367 (324 to 406)	3,961.3 (3,503.4 to 4,384.5)	355 (318 to 394)	3,788.4 (3,385.4 to 4,202.7)	-27.8 (-34.9 to -20.1)	-6.8 (-14.1 to 0.7)
	70+ years	167 (146 to 190)	18,995.4 (16,628.4 to 21,643.1)	329 (289 to 374)	15,511.0 (13,667.4 to 17,645.6)	307 (270 to 349)	14,303.8 (12,569.3 to 16,260.2)	296 (261 to 335)	13,365.6 (11,780.2 to 15,149.8)	-18.3 (-27.1 to -9.1)	-7.8 (-16.1 to 4.4)
**Cook Islands**	All Ages	1,120 (1,040 to 1,200)	5,901.3 (5,495.0 to 6,338.4)	761 (709 to 824)	4,282.0 (3,993.4 to 4,637.1)	703 (651 to 761)	3,958.3 (3,669.0 to 4,286.6)	664 (618 to 716)	3,738.7 (3,481.5 to 4,031.6)	-27.4 (-33.0 to -21.4)	-7.6 (-13.3 to -1.4)
	Under 5	321 (276 to 376)	14,309.1 (12,307.5 to 16,772.1)	45 (38 to 54)	3,914.5 (3,263.1 to 4,667.7)	38 (31 to 45)	3,301.2 (2,747.2 to 3,914.7)	31 (26 to 38)	2,779.7 (2,291.9 to 3,336.1)	-72.6 (-76.0 to -69.1)	-15.7 (-22.6 to -8.7)
	5-14 years	149 (120 to 182)	3,421.7 (2,747.6 to 4,182.2)	42 (33 to 54)	1,497.6 (1,182.1 to 1,913.1)	38 (30 to 47)	1,374.0 (1,079.6 to 1,715.1)	34 (27 to 43)	1,276.1 (1,009.4 to 1,621.6)	-56.2 (-62.6 to -48.9)	-8.3 (-14.9 to -1.3)
	15-49 years	262 (236 to 291)	2,758.8 (2,484.4 to 3,068.2)	142 (129 to 157)	1,738.1 (1,577.4 to 1,926.2)	129 (116 to 143)	1,598.3 (1,434.7 to 1,764.5)	119 (108 to 131)	1,481.5 (1,343.5 to 1,625.6)	-37.0 (-41.9 to -31.1)	-8.0 (-15.0 to -0.1)
	50-69 years	211 (190 to 236)	9,428.6 (8,459.3 to 10,537.3)	238 (212 to 267)	5,597.1 (4,998.9 to 6,289.9)	219 (196 to 245)	5,113.6 (4,583.4 to 5,725.7)	207 (186 to 231)	4,778.1 (4,275.4 to 5,329.4)	-40.6 (-46.5 to -33.8)	-8.6 (-16.3 to 0.8)
	70+ years	174 (154 to 195)	29,535.0 (26,113.1 to 33,094.2)	294 (262 to 333)	20,973.8 (18,696.4 to 23,795.4)	279 (248 to 314)	18,705.5 (16,630.5 to 21,083.7)	272 (241 to 312)	17,434.2 (15,504.1 to 20,036.8)	-29.0 (-36.6 to -20.4)	-10.8 (-19.2 to -0.8)
**Fiji**	All Ages	28,500 (26,300 to 30,600)	3,753.1 (3,472.8 to 4,034.5)	25,700 (24,100 to 27,500)	2,808.6 (2,631.0 to 2,999.5)	22,900 (21,500 to 24,600)	2,490.5 (2,331.4 to 2,672.3)	20,900 (19,500 to 22,300)	2,256.6 (2,104.9 to 2,412.6)	-25.2 (-29.2 to -21.2)	-11.3 (-15.8 to -6.5)
	Under 5	7,940 (6,850 to 9,220)	8,427.5 (7,267.4 to 9,783.8)	5,250 (4,500 to 6,210)	5,622.9 (4,821.4 to 6,647.9)	4,300 (3,700 to 5,010)	4,658.3 (4,013.7 to 5,424.7)	3,530 (3,020 to 4,100)	3,876.9 (3,319.3 to 4,503.3)	-33.3 (-39.0 to -27.1)	-17.2 (-25.0 to -7.5)
	5-14 years	4,820 (3,880 to 5,880)	2,573.9 (2,071.0 to 3,142.7)	3,220 (2,580 to 3,940)	1,811.8 (1,452.6 to 2,216.8)	2,930 (2,400 to 3,580)	1,630.9 (1,333.4 to 1,988.1)	2,700 (2,210 to 3,260)	1,488.7 (1,217.2 to 1,794.9)	-29.6 (-38.0 to -21.5)	-10.0 (-18.8 to 1.5)

Location	Age Group	1990		2019		2020		2021		Percent Incidence Rate Change from 1990-2019 (95% UI)	Percent Incidence Rate Change from 2019-2021 (95% UI)
		Episodes (95% UI)	Rate (95% UI)	Episodes (95% UI)	Rate (95% UI)	Episodes (95% UI)	Rate (95% UI)	Episodes (95% UI)	Rate (95% UI)		
	15-49 years	8,540 (7,720 to 9,410)	2,160.0 (1,953.1 to 2,380.7)	6,830 (6,240 to 7,520)	1,465.9 (1,339.1 to 1,613.9)	6,180 (5,540 to 6,820)	1,323.8 (1,186.5 to 1,462.4)	5,720 (5,160 to 6,340)	1,221.6 (1,101.9 to 1,355.7)	-32.1 (-38.3 to -25.8)	-9.7 (-17.1 to -1.4)
	50-69 years	4,470 (3,950 to 5,030)	6,542.2 (5,782.3 to 7,366.2)	6,270 (5,610 to 7,060)	4,207.1 (3,764.8 to 4,739.3)	5,740 (5,120 to 6,390)	3,814.5 (3,399.1 to 4,245.3)	5,380 (4,770 to 6,000)	3,542.4 (3,142.9 to 3,949.3)	-35.7 (-41.8 to -28.5)	-9.3 (-17.6 to -0.4)
	70+ years	2,700 (2,370 to 3,090)	19,972.3 (17,516.8 to 22,832.3)	4,170 (3,640 to 4,750)	13,658.4 (11,908.3 to 15,547.1)	3,780 (3,340 to 4,300)	11,978.8 (10,568.6 to 13,607.2)	3,540 (3,080 to 3,990)	10,986.0 (9,562.7 to 12,412.1)	-31.6 (-39.7 to -21.3)	-12.3 (-21.0 to -2.4)
**Guam**	All Ages	3,970 (3,690 to 4,280)	2,900.4 (2,694.0 to 3,126.6)	4,820 (4,480 to 5,140)	3,003.1 (2,790.9 to 3,202.6)	4,500 (4,200 to 4,800)	2,815.2 (2,628.3 to 2,999.0)	4,270 (3,980 to 4,570)	2,681.4 (2,502.8 to 2,868.5)	3.5 (-3.2 to 10.2)	-6.3 (-11.5 to -0.7)
	Under 5	1,090 (944 to 1,260)	6,712.7 (5,837.8 to 7,791.2)	634 (546 to 734)	4,717.8 (4,060.8 to 5,462.2)	554 (481 to 642)	4,219.9 (3,665.2 to 4,892.9)	483 (418 to 558)	3,780.4 (3,267.5 to 4,370.2)	-29.7 (-35.6 to -23.3)	-10.6 (-17.6 to -2.4)
	5-14 years	516 (407 to 633)	2,020.5 (1,593.9 to 2,479.4)	359 (280 to 455)	1,451.7 (1,133.7 to 1,839.6)	330 (260 to 413)	1,362.1 (1,070.5 to 1,704.1)	304 (240 to 379)	1,279.1 (1,009.1 to 1,592.0)	-28.2 (-33.9 to -21.7)	-6.2 (-14.6 to 2.9)
	15-49 years	1,070 (953 to 1,200)	1,378.5 (1,232.7 to 1,546.4)	904 (814 to 1,010)	1,183.8 (1,066.5 to 1,320.3)	836 (761 to 929)	1,107.4 (1,008.5 to 1,230.3)	784 (713 to 868)	1,050.5 (954.6 to 1,161.8)	-14.1 (-21.5 to -5.4)	-6.5 (-13.0 to 1.0)
	50-69 years	799 (706 to 890)	5,331.2 (4,711.3 to 5,934.9)	1,260 (1,130 to 1,390)	3,636.2 (3,261.6 to 4,014.4)	1,200 (1,080 to 1,340)	3,396.5 (3,051.2 to 3,787.8)	1,160 (1,030 to 1,300)	3,250.0 (2,883.5 to 3,641.4)	-31.8 (-37.9 to -24.0)	-6.6 (-14.5 to 1.9)
	70+ years	501 (439 to 576)	18,007.9 (15,760.5 to 20,703.5)	1,660 (1,460 to 1,880)	14,658.2 (12,873.9 to 16,543.6)	1,580 (1,400 to 1,780)	13,433.3 (11,852.1 to 15,072.5)	1,540 (1,360 to 1,720)	12,556.1 (11,113.1 to 14,065.3)	-18.6 (-27.9 to -7.1)	-8.4 (-16.7 to 0.8)
**Kiribati**	All Ages	3,460 (3,200 to 3,750)	4,651.2 (4,297.2 to 5,035.4)	3,360 (3,140 to 3,590)	2,842.0 (2,656.0 to 3,041.1)	3,090 (2,890 to 3,300)	2,582.6 (2,411.6 to 2,759.1)	2,880 (2,700 to 3,070)	2,379.8 (2,227.0 to 2,536.9)	-38.9 (-42.3 to -35.5)	-9.1 (-13.6 to -3.9)
	Under 5	1,320 (1,130 to 1,530)	11,213.8 (9,578.1 to 13,008.3)	701 (602 to 820)	4,889.5 (4,202.3 to 5,723.4)	607 (515 to 710)	4,240.5 (3,598.1 to 4,960.8)	523 (444 to 611)	3,660.1 (3,104.0 to 4,274.7)	-56.4 (-60.3 to -52.5)	-13.3 (-20.3 to -4.8)
	5-14 years	452 (359 to 565)	2,546.0 (2,019.0 to 3,179.5)	464 (375 to 567)	1,709.5 (1,381.3 to 2,090.9)	431 (346 to 528)	1,568.2 (1,261.5 to 1,922.4)	401 (328 to 481)	1,447.8 (1,184.4 to 1,735.8)	-32.9 (-39.7 to -24.5)	-8.3 (-16.8 to 1.8)
	15-49 years	850 (768 to 947)	2,317.1 (2,093.4 to 2,582.3)	1,050 (941 to 1,160)	1,748.6 (1,561.7 to 1,916.3)	989 (899 to 1,090)	1,618.3 (1,469.9 to 1,777.1)	936 (838 to 1,040)	1,508.5 (1,349.7 to 1,673.8)	-24.5 (-32.0 to -17.0)	-7.4 (-14.6 to 1.2)
	50-69 years	529 (470 to 593)	7,830.0 (6,957.5 to 8,766.0)	733 (661 to 825)	5,205.7 (4,689.5 to 5,856.5)	693 (615 to 769)	4,825.3 (4,287.0 to 5,355.5)	666 (593 to 747)	4,566.4 (4,067.6 to 5,120.2)	-33.5 (-39.8 to -26.3)	-7.3 (-16.1 to 2.5)
	70+ years	309 (270 to 354)	21,438.2 (18,690.4 to 24,552.1)	407 (356 to 463)	17,135.7 (14,994.4 to 19,467.7)	371 (327 to 427)	15,383.9 (13,569.7 to 17,715.4)	356 (318 to 400)	14,326.3 (12,765.8 to 16,086.0)	-20.1 (-29.5 to -9.5)	-10.2 (-18.0 to -0.4)
**Marshall Islands**	All Ages	2,160 (1,990 to 2,360)	4,761.6 (4,380.1 to 5,194.3)	1,820 (1,700 to 1,940)	3,251.0 (3,041.7 to 3,462.0)	1,670 (1,560 to 1,780)	2,969.6 (2,785.2 to 3,181.6)	1,540 (1,430 to 1,650)	2,736.0 (2,548.8 to 2,924.3)	-31.7 (-35.7 to -26.8)	-8.7 (-13.4 to -3.9)

Location	Age Group	1990		2019		2020		2021		Percent Incidence Rate Change from 1990-2019 (95% UI)	Percent Incidence Rate Change from 2019-2021 (95% UI)
		Episodes (95% UI)	Rate (95% UI)	Episodes (95% UI)	Rate (95% UI)	Episodes (95% UI)	Rate (95% UI)	Episodes (95% UI)	Rate (95% UI)		
	Under 5	861 (740 to 1,010)	11,581.5 (9,947.9 to 13,523.2)	390 (333 to 458)	6,663.1 (5,690.0 to 7,828.2)	333 (288 to 385)	5,774.2 (5,002.6 to 6,688.6)	281 (244 to 328)	4,959.4 (4,300.6 to 5,775.9)	-42.5 (-46.9 to -36.9)	-13.3 (-21.0 to -5.8)
	5-14 years	396 (320 to 483)	2,733.0 (2,205.5 to 3,326.9)	232 (189 to 283)	1,913.3 (1,557.4 to 2,334.8)	210 (171 to 255)	1,758.4 (1,434.4 to 2,130.0)	192 (158 to 232)	1,626.9 (1,343.5 to 1,966.8)	-30.0 (-37.6 to -21.2)	-8.1 (-18.1 to 1.8)
	15-49 years	493 (439 to 543)	2,456.6 (2,187.0 to 2,707.4)	602 (536 to 662)	2,002.2 (1,782.9 to 2,199.2)	562 (509 to 619)	1,855.5 (1,679.0 to 2,043.3)	526 (476 to 581)	1,724.2 (1,562.0 to 1,904.1)	-18.5 (-26.6 to -11.5)	-7.3 (-15.0 to 0.8)
	50-69 years	230 (204 to 260)	8,591.0 (7,632.7 to 9,709.4)	409 (366 to 456)	5,954.4 (5,319.6 to 6,633.7)	386 (347 to 432)	5,487.4 (4,931.6 to 6,148.8)	369 (331 to 413)	5,145.9 (4,614.5 to 5,752.4)	-30.7 (-37.2 to -22.7)	-7.8 (-15.3 to 1.0)
	70+ years	182 (158 to 210)	24,811.6 (21,500.6 to 28,605.9)	185 (161 to 209)	18,320.9 (15,923.8 to 20,709.8)	175 (156 to 202)	16,403.6 (14,604.8 to 18,861.5)	172 (151 to 197)	14,938.4 (13,133.2 to 17,169.2)	-26.2 (-34.9 to -15.7)	-10.5 (-18.7 to -1.4)
**Micronesia (Federated States of)**	All Ages	5,620 (5,200 to 6,080)	5,431.4 (5,021.7 to 5,874.5)	3,420 (3,190 to 3,670)	3,329.6 (3,102.0 to 3,568.2)	3,120 (2,900 to 3,350)	3,033.3 (2,827.7 to 3,260.7)	2,890 (2,700 to 3,100)	2,816.9 (2,637.0 to 3,022.5)	-38.7 (-42.6 to -34.8)	-8.9 (-13.8 to -4.2)
	Under 5	2,140 (1,820 to 2,490)	13,656.9 (11,638.8 to 15,917.1)	543 (460 to 640)	5,572.2 (4,721.5 to 6,564.2)	456 (390 to 530)	4,751.7 (4,059.7 to 5,518.8)	387 (331 to 451)	4,085.1 (3,495.8 to 4,759.9)	-59.2 (-62.7 to -55.2)	-14.7 (-21.7 to -7.0)
	5-14 years	930 (740 to 1,140)	3,073.4 (2,445.5 to 3,750.1)	371 (298 to 455)	1,690.3 (1,358.7 to 2,071.8)	334 (267 to 414)	1,552.5 (1,237.7 to 1,920.6)	305 (246 to 370)	1,443.8 (1,165.2 to 1,753.2)	-45.0 (-50.4 to -38.7)	-8.2 (-16.1 to 0.2)
	15-49 years	1,240 (1,110 to 1,380)	2,620.6 (2,336.9 to 2,917.4)	1,060 (967 to 1,170)	1,981.0 (1,801.8 to 2,184.9)	982 (891 to 1,090)	1,826.3 (1,656.6 to 2,022.8)	913 (824 to 1,010)	1,695.2 (1,529.6 to 1,880.9)	-24.4 (-30.4 to -18.1)	-7.8 (-14.0 to -0.5)
	50-69 years	714 (633 to 809)	9,079.3 (8,042.7 to 10,289.0)	953 (849 to 1,070)	6,320.0 (5,624.6 to 7,094.3)	903 (803 to 1,010)	5,851.3 (5,199.6 to 6,544.5)	856 (764 to 968)	5,489.1 (4,900.6 to 6,203.7)	-30.4 (-37.2 to -22.3)	-7.4 (-16.0 to 1.4)
	70+ years	597 (525 to 682)	25,413.0 (22,363.2 to 29,053.2)	493 (434 to 553)	20,930.2 (18,418.4 to 23,508.1)	439 (386 to 502)	18,634.6 (16,391.0 to 21,279.4)	429 (377 to 481)	16,963.6 (14,923.7 to 19,027.4)	-17.6 (-25.7 to -8.6)	-11.0 (-19.6 to -1.7)
**Nauru**	All Ages	505 (462 to 553)	4,949.1 (4,529.5 to 5,414.8)	362 (336 to 390)	3,326.5 (3,086.4 to 3,576.2)	327 (302 to 351)	2,985.5 (2,760.1 to 3,202.0)	299 (278 to 322)	2,714.5 (2,525.1 to 2,917.4)	-32.8 (-36.2 to -29.0)	-10.3 (-14.7 to -5.7)
	Under 5	212 (181 to 247)	12,989.3 (11,071.9 to 15,102.7)	105 (90 to 122)	7,445.8 (6,393.4 to 8,593.2)	89 (76 to 103)	6,348.8 (5,417.2 to 7,302.4)	75 (65 to 86)	5,378.7 (4,640.3 to 6,142.3)	-42.7 (-47.4 to -36.8)	-14.7 (-22.4 to -6.5)
	5-14 years	70 (56 to 86)	2,722.8 (2,156.7 to 3,343.2)	53 (42 to 63)	2,041.8 (1,638.2 to 2,440.1)	48 (40 to 59)	1,875.9 (1,542.6 to 2,287.1)	45 (37 to 54)	1,734.2 (1,418.3 to 2,097.4)	-25.0 (-34.3 to -14.1)	-8.1 (-18.4 to 1.7)
	15-49 years	120 (107 to 134)	2,415.7 (2,157.8 to 2,698.2)	105 (93 to 117)	1,863.3 (1,656.4 to 2,070.9)	98 (88 to 108)	1,724.2 (1,553.6 to 1,901.0)	92 (83 to 102)	1,605.9 (1,449.7 to 1,772.4)	-22.9 (-29.3 to -14.9)	-7.5 (-14.5 to 0.6)
	50-69 years	67 (59 to 76)	7,793.6 (6,915.9 to 8,805.5)	60 (54 to 67)	5,723.9 (5,125.4 to 6,357.2)	56 (50 to 62)	5,243.2 (4,700.5 to 5,833.1)	53 (48 to 59)	4,930.5 (4,421.3 to 5,498.9)	-26.6 (-33.6 to -19.4)	-8.4 (-15.9 to 0.4)

Location	Age Group	1990		2019		2020		2021		Percent Incidence Rate Change from 1990-2019 (95% UI)	Percent Incidence Rate Change from 2019-2021 (95% UI)
		Episodes (95% UI)	Rate (95% UI)	Episodes (95% UI)	Rate (95% UI)	Episodes (95% UI)	Rate (95% UI)	Episodes (95% UI)	Rate (95% UI)		
	70+ years	35 (31 to 41)	22,460.7 (19,357.6 to 26,010.2)	39 (35 to 45)	18,307.0 (16,205.7 to 20,910.3)	36 (32 to 41)	16,257.0 (14,420.1 to 18,556.6)	34 (30 to 39)	15,050.6 (13,181.8 to 17,219.1)	-18.5 (-28.8 to -8.8)	-11.2 (-19.8 to -1.1)
**Niue**	All Ages	128 (119 to 137)	5,533.7 (5,150.7 to 5,954.8)	67 (62 to 72)	4,003.2 (3,735.9 to 4,308.3)	59 (55 to 63)	3,511.1 (3,277.3 to 3,759.0)	53 (49 to 57)	3,179.5 (2,957.2 to 3,422.8)	-27.7 (-32.0 to -23.3)	-12.3 (-17.3 to -7.1)
	Under 5	28 (24 to 32)	10,905.3 (9,290.9 to 12,647.4)	8 (6 to 9)	6,333.1 (5,411.7 to 7,419.7)	6 (5 to 7)	5,110.9 (4,354.6 to 6,046.6)	5 (4 to 6)	4,136.5 (3,550.1 to 4,802.8)	-41.9 (-47.0 to -36.9)	-19.3 (-26.7 to -10.4)
	5-14 years	14 (12 to 18)	2,589.4 (2,086.2 to 3,182.3)	5 (4 to 6)	1,772.3 (1,451.3 to 2,173.4)	4 (4 to 5)	1,593.6 (1,282.9 to 1,951.6)	4 (3 to 5)	1,446.8 (1,187.1 to 1,783.4)	-31.6 (-37.9 to -23.8)	-10.1 (-19.3 to -0.5)
	15-49 years	24 (21 to 26)	2,288.9 (2,039.4 to 2,537.6)	12 (11 to 13)	1,568.0 (1,419.1 to 1,724.2)	11 (10 to 12)	1,403.1 (1,264.1 to 1,536.6)	10 (9 to 11)	1,287.3 (1,156.7 to 1,421.8)	-31.5 (-36.6 to -25.6)	-10.5 (-17.4 to -2.7)
	50-69 years	24 (21 to 27)	7,398.0 (6,504.8 to 8,337.3)	19 (17 to 21)	5,069.9 (4,464.1 to 5,714.7)	18 (16 to 20)	4,605.0 (4,100.7 to 5,148.0)	16 (15 to 18)	4,266.7 (3,779.5 to 4,742.3)	-31.5 (-38.4 to -24.0)	-9.2 (-17.1 to 0.5)
	70+ years	38 (33 to 43)	25,903.1 (22,417.8 to 29,062.3)	23 (20 to 26)	19,031.1 (16,784.1 to 21,444.2)	20 (17 to 22)	16,618.0 (14,661.2 to 18,712.0)	18 (16 to 20)	15,127.7 (13,423.7 to 17,247.9)	-26.5 (-34.4 to -18.7)	-12.7 (-20.4 to -2.6)
**Northern Mariana Islands**	All Ages	1,320 (1,220 to 1,430)	2,931.5 (2,708.1 to 3,170.9)	1,390 (1,290 to 1,480)	2,831.7 (2,639.2 to 3,016.5)	1,290 (1,210 to 1,400)	2,655.7 (2,481.4 to 2,861.8)	1,220 (1,140 to 1,300)	2,515.0 (2,348.4 to 2,682.9)	-3.4 (-9.9 to 3.6)	-6.2 (-11.1 to -1.2)
	Under 5	394 (336 to 454)	8,285.1 (7,080.5 to 9,546.7)	205 (174 to 236)	6,041.0 (5,136.6 to 6,962.8)	178 (151 to 211)	5,383.8 (4,567.9 to 6,394.4)	152 (128 to 178)	4,748.9 (3,975.9 to 5,555.7)	-27.1 (-33.6 to -20.4)	-10.9 (-19.0 to -2.7)
	5-14 years	138 (107 to 176)	1,863.2 (1,438.6 to 2,377.0)	123 (97 to 153)	1,533.0 (1,205.8 to 1,905.3)	115 (90 to 148)	1,424.2 (1,120.0 to 1,826.6)	107 (84 to 135)	1,325.5 (1,040.3 to 1,672.3)	-17.7 (-23.9 to -10.3)	-7.1 (-15.0 to 1.7)
	15-49 years	509 (448 to 565)	1,754.6 (1,542.0 to 1,944.8)	331 (296 to 368)	1,356.2 (1,212.1 to 1,508.4)	303 (276 to 336)	1,263.0 (1,150.6 to 1,399.6)	281 (254 to 312)	1,184.4 (1,071.2 to 1,315.1)	-22.7 (-29.0 to -14.7)	-6.9 (-13.4 to 0.3)
	50-69 years	193 (171 to 218)	5,584.1 (4,952.0 to 6,312.0)	490 (435 to 543)	4,232.7 (3,757.4 to 4,689.4)	449 (400 to 503)	3,903.3 (3,471.3 to 4,371.8)	420 (379 to 463)	3,691.0 (3,326.2 to 4,061.2)	-24.2 (-31.4 to -16.3)	-7.8 (-15.4 to 1.9)
	70+ years	89 (78 to 103)	18,518.3 (16,161.4 to 21,339.8)	238 (210 to 266)	15,218.8 (13,477.4 to 17,048.7)	249 (222 to 282)	13,389.8 (11,912.3 to 15,125.6)	259 (230 to 294)	12,235.0 (10,879.5 to 13,858.5)	-17.8 (-28.6 to -6.5)	-12.0 (-20.0 to -3.3)
**Palau**	All Ages	714 (665 to 767)	4,701.3 (4,374.4 to 5,051.7)	658 (614 to 709)	3,642.3 (3,401.4 to 3,925.8)	596 (554 to 643)	3,293.4 (3,063.1 to 3,555.3)	551 (511 to 594)	3,044.3 (2,822.5 to 3,284.6)	-22.5 (-27.3 to -16.9)	-9.6 (-14.5 to -3.4)
	Under 5	188 (161 to 222)	12,758.0 (10,899.3 to 15,028.6)	60 (52 to 70)	5,897.5 (5,035.5 to 6,881.5)	47 (40 to 56)	4,811.7 (4,112.1 to 5,659.9)	37 (32 to 44)	3,940.3 (3,387.1 to 4,644.9)	-53.8 (-58.0 to -49.0)	-18.4 (-25.9 to -8.9)
	5-14 years	84 (68 to 103)	2,722.8 (2,209.6 to 3,344.3)	41 (33 to 50)	1,699.4 (1,385.7 to 2,078.0)	36 (30 to 44)	1,535.3 (1,258.5 to 1,880.9)	32 (26 to 39)	1,393.7 (1,130.1 to 1,679.3)	-37.6 (-43.7 to -31.2)	-9.7 (-18.7 to 0.3)

Location	Age Group	1990		2019		2020		2021		Percent Incidence Rate Change from 1990-2019 (95% UI)	Percent Incidence Rate Change from 2019-2021 (95% UI)
		Episodes (95% UI)	Rate (95% UI)	Episodes (95% UI)	Rate (95% UI)	Episodes (95% UI)	Rate (95% UI)	Episodes (95% UI)	Rate (95% UI)		
	15-49 years	206 (186 to 229)	2,402.7 (2,169.6 to 2,676.7)	180 (163 to 199)	1,939.8 (1,753.4 to 2,150.9)	161 (145 to 178)	1,759.0 (1,579.7 to 1,936.4)	147 (131 to 163)	1,616.5 (1,447.7 to 1,801.8)	-19.3 (-27.5 to -10.8)	-9.3 (-16.2 to -1.8)
	50-69 years	127 (113 to 143)	8,003.4 (7,091.9 to 8,983.8)	235 (206 to 261)	5,142.3 (4,500.6 to 5,716.9)	218 (195 to 249)	4,634.3 (4,148.5 to 5,297.2)	210 (187 to 236)	4,324.2 (3,844.9 to 4,854.3)	-35.7 (-42.3 to -29.8)	-9.9 (-18.0 to 0.3)
	70+ years	109 (93 to 123)	22,753.4 (19,370.9 to 25,787.4)	142 (125 to 163)	17,694.1 (15,545.2 to 20,287.4)	133 (118 to 151)	15,164.9 (13,455.7 to 17,184.3)	125 (109 to 141)	13,675.3 (11,908.3 to 15,414.6)	-22.2 (-30.4 to -12.0)	-14.3 (-22.6 to -5.0)
**Papua New Guinea**	All Ages	253,000 (233,000 to 276,000)	6,162.8 (5,673.5 to 6,737.4)	400,000 (370,000 to 433,000)	4,051.0 (3,745.0 to 4,381.3)	376,000 (348,000 to 406,000)	3,694.2 (3,424.6 to 3,991.7)	355,000 (326,000 to 382,000)	3,390.5 (3,117.4 to 3,652.5)	-34.3 (-37.5 to -31.2)	-8.8 (-13.3 to -4.1)
	Under 5	129,000 (114,000 to 148,000)	19,951.2 (17,560.3 to 22,835.0)	162,000 (141,000 to 185,000)	11,198.3 (9,768.0 to 12,836.1)	147,000 (129,000 to 168,000)	9,924.6 (8,669.7 to 11,360.2)	133,000 (117,000 to 153,000)	8,774.8 (7,664.5 to 10,079.6)	-43.9 (-48.0 to -39.5)	-11.4 (-17.7 to -3.2)
	5-14 years	30,900 (24,300 to 38,500)	2,935.1 (2,310.7 to 3,659.7)	48,000 (38,300 to 59,300)	2,140.4 (1,705.9 to 2,640.4)	46,000 (36,200 to 56,900)	1,984.8 (1,559.1 to 2,453.8)	44,200 (34,800 to 55,300)	1,845.1 (1,451.7 to 2,307.9)	-27.1 (-33.2 to -18.6)	-7.3 (-16.2 to 1.0)
	15-49 years	43,400 (38,100 to 48,900)	2,182.5 (1,919.0 to 2,462.3)	87,300 (76,300 to 99,100)	1,717.0 (1,500.5 to 1,948.5)	83,800 (74,700 to 93,000)	1,607.7 (1,432.6 to 1,783.8)	80,800 (71,000 to 90,600)	1,513.6 (1,329.3 to 1,695.8)	-21.3 (-27.0 to -15.4)	-6.4 (-13.0 to 0.7)
	50-69 years	32,800 (28,900 to 37,400)	9,234.4 (8,143.0 to 10,538.5)	63,200 (55,400 to 71,800)	6,696.0 (5,869.0 to 7,601.7)	61,800 (54,400 to 70,300)	6,236.0 (5,489.1 to 7,101.4)	60,700 (53,700 to 68,900)	5,891.9 (5,215.1 to 6,683.5)	-27.5 (-34.1 to -19.4)	-6.9 (-15.5 to 1.7)
	70+ years	16,600 (14,200 to 19,200)	27,589.2 (23,599.5 to 31,945.0)	40,100 (35,200 to 45,700)	24,212.5 (21,221.3 to 27,558.1)	37,100 (32,100 to 42,200)	22,002.3 (19,054.8 to 25,063.2)	35,500 (30,800 to 40,300)	20,379.0 (17,717.6 to 23,142.5)	-12.2 (-22.4 to 0.1)	-9.1 (-18.4 to 1.8)
**Samoa**	All Ages	8,750 (8,100 to 9,450)	5,178.1 (4,793.8 to 5,591.6)	7,080 (6,590 to 7,580)	3,394.8 (3,163.2 to 3,636.3)	6,580 (6,170 to 7,040)	3,121.6 (2,924.8 to 3,335.5)	6,160 (5,810 to 6,540)	2,882.4 (2,718.7 to 3,061.1)	-34.4 (-37.7 to -30.5)	-8.0 (-12.0 to -3.5)
	Under 5	3,030 (2,600 to 3,520)	11,903.4 (10,207.1 to 13,801.6)	1,470 (1,260 to 1,690)	5,097.0 (4,391.2 to 5,887.8)	1,300 (1,110 to 1,510)	4,472.1 (3,828.0 to 5,208.7)	1,140 (989 to 1,320)	3,892.4 (3,382.4 to 4,496.1)	-57.2 (-60.5 to -53.8)	-12.3 (-19.1 to -4.5)
	5-14 years	1,390 (1,110 to 1,720)	3,034.7 (2,431.6 to 3,751.8)	875 (712 to 1,080)	1,767.7 (1,438.8 to 2,184.3)	825 (665 to 1,030)	1,647.7 (1,328.2 to 2,055.3)	777 (623 to 941)	1,533.0 (1,229.1 to 1,855.5)	-41.8 (-47.6 to -35.1)	-6.8 (-14.9 to 2.7)
	15-49 years	1,830 (1,650 to 2,030)	2,336.7 (2,111.1 to 2,591.5)	1,770 (1,590 to 1,980)	1,804.3 (1,620.0 to 2,016.8)	1,670 (1,520 to 1,840)	1,685.9 (1,527.7 to 1,851.2)	1,570 (1,410 to 1,750)	1,567.2 (1,406.3 to 1,743.0)	-22.8 (-29.7 to -16.5)	-6.6 (-12.9 to 2.2)
	50-69 years	1,390 (1,240 to 1,560)	8,953.9 (7,985.0 to 10,056.0)	1,500 (1,330 to 1,680)	5,865.5 (5,199.0 to 6,565.0)	1,430 (1,260 to 1,600)	5,489.2 (4,850.2 to 6,149.2)	1,370 (1,240 to 1,530)	5,153.6 (4,683.3 to 5,755.1)	-34.5 (-41.0 to -27.0)	-6.4 (-15.2 to 2.7)
	70+ years	1,100 (959 to 1,250)	29,071.5 (25,295.8 to 32,934.3)	1,470 (1,300 to 1,680)	22,389.0 (19,886.0 to 25,700.1)	1,360 (1,220 to 1,510)	20,426.7 (18,394.0 to 22,622.4)	1,300 (1,170 to 1,470)	19,046.9 (17,095.0 to 21,499.3)	-23.0 (-30.6 to -14.8)	-8.8 (-16.3 to -0.3)

Location	Age Group	1990		2019		2020		2021		Percent Incidence Rate Change from 1990-2019 (95% UI)	Percent Incidence Rate Change from 2019-2021 (95% UI)
		Episodes (95% UI)	Rate (95% UI)	Episodes (95% UI)	Rate (95% UI)	Episodes (95% UI)	Rate (95% UI)	Episodes (95% UI)	Rate (95% UI)		
**Solomon Islands**	All Ages	26,600 (24,700 to 28,900)	7,851.3 (7,275.3 to 8,522.9)	34,800 (32,700 to 37,200)	5,298.4 (4,976.1 to 5,661.0)	31,900 (29,900 to 34,200)	4,760.4 (4,457.8 to 5,101.8)	29,700 (27,800 to 31,800)	4,340.7 (4,063.1 to 4,649.1)	-32.5 (-36.2 to -28.8)	-10.2 (-15.1 to -4.7)
	Under 5	10,500 (9,020 to 12,100)	17,537.0 (15,128.7 to 20,343.6)	8,580 (7,410 to 9,880)	9,177.3 (7,924.2 to 10,562.9)	7,330 (6,360 to 8,490)	7,761.6 (6,734.5 to 8,995.1)	6,220 (5,440 to 7,200)	6,528.6 (5,703.1 to 7,552.5)	-47.7 (-52.3 to -42.5)	-15.4 (-23.1 to -7.9)
	5-14 years	3,330 (2,670 to 4,090)	3,465.8 (2,781.8 to 4,254.2)	4,060 (3,280 to 4,910)	2,530.7 (2,042.3 to 3,058.4)	3,760 (3,040 to 4,650)	2,310.8 (1,872.4 to 2,861.4)	3,500 (2,830 to 4,260)	2,124.3 (1,718.5 to 2,583.8)	-27.0 (-34.9 to -18.2)	-8.7 (-18.1 to 2.5)
	15-49 years	6,140 (5,590 to 6,780)	4,049.4 (3,690.6 to 4,476.8)	10,700 (9,720 to 11,800)	3,243.2 (2,941.1 to 3,568.4)	10,000 (9,060 to 10,900)	2,969.7 (2,684.6 to 3,243.7)	9,520 (8,620 to 10,500)	2,765.5 (2,503.0 to 3,063.0)	-19.9 (-26.2 to -13.2)	-8.4 (-15.7 to -1.4)
	50-69 years	4,520 (4,060 to 5,040)	16,666.4 (14,958.7 to 18,584.0)	6,880 (6,220 to 7,590)	11,352.4 (10,268.0 to 12,528.0)	6,540 (5,940 to 7,190)	10,360.5 (9,402.3 to 11,387.6)	6,400 (5,740 to 7,140)	9,715.6 (8,713.7 to 10,831.3)	-31.9 (-38.3 to -24.9)	-8.7 (-17.2 to -1.0)
	70+ years	2,170 (1,920 to 2,470)	46,261.7 (40,876.0 to 52,603.6)	4,610 (4,110 to 5,280)	36,366.1 (32,451.6 to 41,691.7)	4,270 (3,790 to 4,780)	33,027.4 (29,297.2 to 36,953.0)	4,020 (3,640 to 4,590)	30,353.5 (27,477.6 to 34,663.0)	-21.4 (-29.4 to -10.5)	-9.2 (-17.5 to 0.7)
**Tokelau**	All Ages	86 (79 to 93)	5,375.9 (4,976.7 to 5,806.5)	47 (44 to 50)	3,477.5 (3,231.5 to 3,721.8)	44 (41 to 47)	3,200.5 (2,987.1 to 3,445.6)	41 (38 to 45)	2,999.2 (2,772.8 to 3,252.1)	-35.3 (-39.6 to -30.6)	-8.0 (-12.5 to -3.3)
	Under 5	25 (22 to 30)	12,846.4 (11,066.5 to 15,234.0)	5 (4 to 6)	4,668.2 (3,933.8 to 5,437.1)	4 (4 to 5)	4,186.8 (3,529.9 to 4,978.3)	4 (3 to 4)	3,735.6 (3,169.0 to 4,375.0)	-63.7 (-66.7 to -59.7)	-10.3 (-18.6 to -1.6)
	5-14 years	10 (8 to 12)	2,443.4 (1,933.9 to 3,001.3)	4 (3 to 5)	1,498.2 (1,171.0 to 1,891.5)	4 (3 to 5)	1,390.8 (1,077.6 to 1,757.4)	4 (3 to 5)	1,292.8 (1,022.9 to 1,629.7)	-38.7 (-45.3 to -30.7)	-7.2 (-14.3 to 0.1)
	15-49 years	15 (14 to 17)	2,165.9 (1,941.7 to 2,422.8)	9 (9 to 11)	1,470.2 (1,319.7 to 1,630.6)	9 (8 to 10)	1,382.6 (1,243.9 to 1,540.8)	9 (8 to 10)	1,311.6 (1,181.7 to 1,463.0)	-32.1 (-37.1 to -26.3)	-6.0 (-13.1 to 1.8)
	50-69 years	18 (16 to 20)	8,039.5 (7,052.0 to 9,085.2)	11 (10 to 13)	5,039.7 (4,533.5 to 5,632.9)	11 (10 to 12)	4,650.9 (4,141.5 to 5,185.2)	10 (9 to 11)	4,315.4 (3,810.1 to 4,814.5)	-37.3 (-43.6 to -30.4)	-7.7 (-16.4 to 0.1)
	70+ years	18 (16 to 20)	23,904.3 (20,658.9 to 27,127.9)	17 (15 to 19)	19,677.0 (17,404.0 to 22,296.5)	16 (14 to 18)	17,957.9 (15,977.2 to 20,369.5)	15 (13 to 17)	16,400.1 (14,559.9 to 18,483.0)	-17.7 (-27.7 to -6.5)	-8.7 (-17.0 to -0.6)
**Tonga**	All Ages	4,360 (4,040 to 4,680)	4,410.6 (4,090.1 to 4,738.8)	3,610 (3,390 to 3,870)	3,407.2 (3,197.4 to 3,656.9)	3,260 (3,050 to 3,480)	3,076.6 (2,879.3 to 3,280.5)	2,990 (2,800 to 3,200)	2,814.7 (2,633.6 to 3,010.7)	-22.7 (-26.8 to -18.3)	-9.7 (-14.7 to -5.1)
	Under 5	1,420 (1,230 to 1,660)	9,304.3 (8,072.6 to 10,885.6)	707 (611 to 821)	4,819.0 (4,164.6 to 5,602.2)	593 (508 to 694)	4,079.7 (3,495.1 to 4,776.9)	497 (429 to 572)	3,452.2 (2,977.4 to 3,970.1)	-48.2 (-52.0 to -44.2)	-15.3 (-22.2 to -8.6)
	5-14 years	602 (491 to 745)	2,269.5 (1,851.8 to 2,809.2)	406 (327 to 496)	1,653.7 (1,331.1 to 2,020.5)	375 (302 to 463)	1,525.9 (1,229.1 to 1,885.7)	346 (274 to 423)	1,404.3 (1,114.7 to 1,717.0)	-27.1 (-33.6 to -20.3)	-7.7 (-15.4 to 0.6)

Location	Age Group	1990		2019		2020		2021		Percent Incidence Rate Change from 1990-2019 (95% UI)	Percent Incidence Rate Change from 2019-2021 (95% UI)
		Episodes (95% UI)	Rate (95% UI)	Episodes (95% UI)	Rate (95% UI)	Episodes (95% UI)	Rate (95% UI)	Episodes (95% UI)	Rate (95% UI)		
	15-49 years	854 (765 to 950)	1,929.5 (1,727.7 to 2,144.9)	778 (708 to 858)	1,582.6 (1,440.4 to 1,744.6)	718 (649 to 796)	1,460.9 (1,319.4 to 1,618.1)	664 (608 to 726)	1,348.7 (1,236.6 to 1,474.7)	-18.0 (-24.0 to -10.7)	-7.7 (-16.0 to -0.4)
	50-69 years	801 (706 to 900)	7,873.1 (6,938.9 to 8,849.5)	735 (656 to 817)	5,567.8 (4,966.9 to 6,186.3)	691 (621 to 784)	5,136.2 (4,614.4 to 5,822.3)	653 (585 to 732)	4,769.0 (4,269.7 to 5,345.9)	-29.3 (-36.6 to -22.6)	-7.8 (-16.5 to 1.2)
	70+ years	682 (594 to 768)	26,240.0 (22,862.7 to 29,573.6)	982 (876 to 1,120)	22,755.3 (20,294.7 to 25,891.1)	886 (776 to 991)	20,400.4 (17,865.1 to 22,810.3)	832 (738 to 939)	18,995.2 (16,852.9 to 21,444.1)	-13.3 (-22.3 to -2.8)	-10.3 (-18.2 to -1.1)
**Tuvalu**	All Ages	649 (596 to 702)	6,825.2 (6,269.6 to 7,383.4)	406 (380 to 433)	3,365.8 (3,148.9 to 3,590.3)	378 (353 to 405)	3,096.7 (2,888.2 to 3,320.1)	358 (337 to 384)	2,896.4 (2,723.1 to 3,106.6)	-50.7 (-53.8 to -47.1)	-8.0 (-12.9 to -3.2)
	Under 5	260 (225 to 300)	17,228.9 (14,920.1 to 19,889.0)	54 (47 to 63)	4,307.8 (3,729.3 to 5,012.7)	48 (41 to 56)	3,781.2 (3,230.0 to 4,412.6)	42 (36 to 49)	3,297.7 (2,816.4 to 3,782.3)	-75.0 (-77.0 to -72.5)	-12.2 (-18.9 to -4.4)
	5-14 years	69 (55 to 84)	3,495.6 (2,816.6 to 4,282.2)	38 (31 to 45)	1,547.6 (1,256.8 to 1,853.4)	35 (28 to 43)	1,431.9 (1,158.3 to 1,751.1)	33 (26 to 40)	1,340.9 (1,082.3 to 1,616.3)	-55.7 (-60.4 to -50.1)	-7.5 (-16.1 to 1.3)
	15-49 years	129 (117 to 143)	2,861.9 (2,579.7 to 3,171.4)	112 (101 to 124)	1,859.4 (1,665.9 to 2,060.5)	107 (96 to 118)	1,739.3 (1,566.0 to 1,919.3)	102 (91 to 113)	1,629.2 (1,449.6 to 1,816.2)	-35.0 (-40.9 to -28.7)	-6.5 (-14.5 to 2.3)
	50-69 years	122 (108 to 135)	9,751.4 (8,617.6 to 10,829.6)	108 (97 to 121)	5,856.4 (5,283.9 to 6,551.7)	102 (92 to 114)	5,456.1 (4,895.8 to 6,076.5)	97 (88 to 107)	5,149.8 (4,659.3 to 5,696.3)	-39.9 (-45.5 to -34.2)	-6.8 (-15.2 to 2.0)
	70+ years	69 (60 to 80)	25,700.9 (22,385.1 to 29,477.7)	94 (82 to 107)	19,490.4 (17,067.7 to 22,186.5)	86 (77 to 98)	17,658.6 (15,824.9 to 20,105.1)	84 (74 to 95)	16,580.9 (14,572.5 to 18,676.8)	-24.2 (-33.0 to -14.8)	-9.4 (-19.0 to 0.6)
**Vanuatu**	All Ages	7,320 (6,730 to 7,940)	4,810.7 (4,422.7 to 5,217.6)	10,100 (9,420 to 10,800)	3,360.3 (3,140.4 to 3,596.1)	9,510 (8,890 to 10,200)	3,102.2 (2,900.7 to 3,316.3)	9,030 (8,400 to 9,680)	2,885.9 (2,684.4 to 3,092.5)	-30.1 (-34.0 to -25.9)	-7.7 (-12.7 to -3.0)
	Under 5	3,160 (2,680 to 3,680)	11,681.6 (9,929.9 to 13,611.3)	2,770 (2,390 to 3,210)	6,643.0 (5,746.3 to 7,700.3)	2,490 (2,140 to 2,890)	5,945.0 (5,118.4 to 6,896.8)	2,220 (1,940 to 2,570)	5,274.6 (4,611.1 to 6,090.8)	-43.1 (-47.8 to -37.9)	-10.5 (-17.8 to -1.9)
	5-14 years	1,030 (819 to 1,280)	2,511.9 (1,994.8 to 3,114.7)	1,400 (1,140 to 1,720)	1,933.7 (1,575.3 to 2,382.0)	1,330 (1,070 to 1,660)	1,813.1 (1,459.2 to 2,262.1)	1,260 (1,010 to 1,530)	1,689.3 (1,351.9 to 2,060.7)	-23.0 (-31.6 to -12.9)	-6.2 (-15.6 to 4.4)
	15-49 years	1,620 (1,440 to 1,820)	2,302.9 (2,043.6 to 2,589.0)	2,830 (2,540 to 3,110)	1,903.4 (1,713.3 to 2,093.6)	2,730 (2,480 to 3,010)	1,789.7 (1,626.0 to 1,976.9)	2,640 (2,380 to 2,910)	1,692.4 (1,524.8 to 1,865.0)	-17.3 (-24.1 to -10.0)	-6.0 (-13.0 to 3.6)
	50-69 years	969 (870 to 1,100)	8,345.0 (7,497.8 to 9,462.9)	1,880 (1,650 to 2,130)	6,020.2 (5,281.6 to 6,818.0)	1,830 (1,620 to 2,060)	5,617.5 (4,971.0 to 6,324.2)	1,810 (1,590 to 2,020)	5,356.1 (4,716.5 to 5,982.2)	-27.9 (-35.8 to -20.1)	-6.7 (-15.5 to 2.5)
	70+ years	547 (471 to 636)	23,778.6 (20,466.7 to 27,623.4)	1,210 (1,040 to 1,380)	19,122.5 (16,332.5 to 21,844.9)	1,140 (1,010 to 1,290)	17,518.0 (15,485.9 to 19,884.3)	1,100 (968 to 1,260)	16,585.3 (14,542.6 to 18,897.9)	-19.6 (-29.8 to -8.3)	-8.4 (-18.4 to 2.5)
**Cambodia**	All Ages	934,000 (856,000 to 1,020,000)	9,089.4 (8,328.0 to 9,952.3)	675,000 (632,000 to 716,000)	4,066.2 (3,809.8 to 4,313.5)	614,000 (572,000 to 661,000)	3,645.5 (3,396.1 to 3,926.1)	566,000 (530,000 to 605,000)	3,319.2 (3,108.4 to 3,552.2)	-55.3 (-58.5 to -51.5)	-10.3 (-16.2 to -3.5)

Location	Age Group	1990		2019		2020		2021		Percent Incidence Rate Change from 1990-2019 (95% UI)	Percent Incidence Rate Change from 2019-2021 (95% UI)
		Episodes (95% UI)	Rate (95% UI)	Episodes (95% UI)	Rate (95% UI)	Episodes (95% UI)	Rate (95% UI)	Episodes (95% UI)	Rate (95% UI)		
	Under 5	534,000 (462,000 to 612,000)	29,283.3 (25,333.0 to 33,542.1)	158,000 (138,000 to 182,000)	8,997.9 (7,837.8 to 10,335.2)	131,000 (111,000 to 154,000)	7,457.4 (6,312.1 to 8,783.8)	108,000 (93,500 to 124,000)	6,157.8 (5,347.7 to 7,097.3)	-69.3 (-72.6 to -65.8)	-17.1 (-26.7 to -5.4)
	5-14 years	126,000 (101,000 to 156,000)	4,452.6 (3,568.7 to 5,489.5)	61,900 (49,900 to 75,800)	1,867.8 (1,506.1 to 2,288.1)	57,200 (46,200 to 69,000)	1,710.1 (1,382.8 to 2,064.6)	53,900 (42,700 to 66,600)	1,599.9 (1,268.5 to 1,976.4)	-58.1 (-63.0 to -50.8)	-8.4 (-19.0 to 4.5)
	15-49 years	125,000 (112,000 to 140,000)	2,731.3 (2,438.4 to 3,042.8)	152,000 (138,000 to 167,000)	1,728.2 (1,572.3 to 1,908.0)	143,000 (129,000 to 158,000)	1,606.4 (1,450.1 to 1,773.5)	135,000 (121,000 to 149,000)	1,495.1 (1,345.8 to 1,656.8)	-36.7 (-43.1 to -30.2)	-7.0 (-15.4 to 2.1)
	50-69 years	86,600 (76,800 to 96,400)	10,407.2 (9,236.7 to 11,586.4)	163,000 (147,000 to 179,000)	7,267.4 (6,520.7 to 7,981.0)	153,000 (138,000 to 171,000)	6,639.5 (5,960.3 to 7,397.1)	147,000 (132,000 to 165,000)	6,221.8 (5,588.4 to 6,975.3)	-30.2 (-37.2 to -22.6)	-8.6 (-16.5 to 0.2)
	70+ years	61,000 (54,000 to 68,900)	32,715.6 (28,982.8 to 36,941.2)	140,000 (126,000 to 159,000)	27,595.1 (24,891.2 to 31,507.2)	129,000 (115,000 to 146,000)	24,501.1 (21,721.2 to 27,701.3)	122,000 (108,000 to 140,000)	22,345.1 (19,780.4 to 25,613.3)	-15.7 (-24.5 to -6.5)	-11.2 (-20.7 to -1.6)
**Indonesia**	All Ages	8,280,000 (7,630,000 to 9,010,000)	4,478.5 (4,124.8 to 4,869.2)	8,550,000 (8,010,000 to 9,120,000)	3,134.6 (2,937.1 to 3,344.8)	8,230,000 (7,680,000 to 8,820,000)	2,983.5 (2,783.0 to 3,198.0)	7,670,000 (7,170,000 to 8,190,000)	2,749.8 (2,571.5 to 2,937.3)	-30.0 (-34.7 to -25.2)	-4.8 (-8.5 to -1.7)
	Under 5	4,090,000 (3,560,000 to 4,730,000)	18,343.4 (15,934.2 to 21,203.6)	1,160,000 (1,020,000 to 1,330,000)	5,212.6 (4,570.4 to 5,966.5)	1,100,000 (952,000 to 1,280,000)	5,008.7 (4,317.6 to 5,798.5)	907,000 (794,000 to 1,040,000)	4,141.9 (3,623.0 to 4,759.7)	-71.6 (-73.1 to -69.7)	-3.9 (-10.1 to 1.5)
	5-14 years	1,140,000 (904,000 to 1,390,000)	2,500.8 (1,990.6 to 3,068.2)	883,000 (690,000 to 1,110,000)	1,923.1 (1,502.2 to 2,416.5)	846,000 (657,000 to 1,070,000)	1,852.2 (1,438.5 to 2,333.5)	786,000 (609,000 to 984,000)	1,732.0 (1,342.8 to 2,169.0)	-23.1 (-29.8 to -14.9)	-3.7 (-6.9 to -0.5)
	15-49 years	1,390,000 (1,250,000 to 1,550,000)	1,471.6 (1,327.6 to 1,636.0)	2,030,000 (1,810,000 to 2,270,000)	1,348.9 (1,208.2 to 1,510.7)	1,980,000 (1,770,000 to 2,220,000)	1,300.8 (1,163.8 to 1,464.2)	1,850,000 (1,660,000 to 2,060,000)	1,206.8 (1,080.1 to 1,346.1)	-8.3 (-12.4 to -4.5)	-3.6 (-7.2 to -0.5)
	50-69 years	967,000 (861,000 to 1,080,000)	5,111.9 (4,550.0 to 5,689.5)	2,400,000 (2,190,000 to 2,630,000)	5,295.9 (4,819.8 to 5,802.0)	2,340,000 (2,130,000 to 2,560,000)	4,972.7 (4,527.1 to 5,436.6)	2,250,000 (2,050,000 to 2,470,000)	4,635.6 (4,223.2 to 5,081.9)	3.6 (-2.7 to 11.3)	-6.1 (-9.7 to -2.4)
	70+ years	696,000 (619,000 to 775,000)	18,283.7 (16,247.7 to 20,353.4)	2,080,000 (1,870,000 to 2,310,000)	23,235.6 (20,916.4 to 25,880.5)	1,970,000 (1,760,000 to 2,200,000)	21,113.6 (18,913.1 to 23,612.9)	1,870,000 (1,680,000 to 2,100,000)	19,398.9 (17,377.6 to 21,761.0)	27.1 (16.7 to 39.4)	-9.1 (-13.7 to -4.5)
**Lao People's Democratic Republic**	All Ages	300,000 (273,000 to 330,000)	7,184.9 (6,545.8 to 7,915.7)	191,000 (177,000 to 205,000)	2,653.6 (2,467.4 to 2,855.6)	171,000 (160,000 to 183,000)	2,353.3 (2,201.2 to 2,516.9)	168,000 (157,000 to 180,000)	2,274.9 (2,123.6 to 2,436.4)	-63.1 (-66.1 to -60.0)	-11.3 (-16.0 to -6.3)
	Under 5	168,000 (146,000 to 195,000)	23,643.2 (20,503.1 to 27,495.4)	46,500 (40,700 to 53,600)	5,648.1 (4,940.7 to 6,511.8)	39,700 (34,700 to 46,200)	4,794.9 (4,197.2 to 5,589.5)	36,900 (31,700 to 42,400)	4,446.6 (3,824.7 to 5,106.7)	-76.1 (-78.3 to -73.7)	-15.1 (-23.4 to -6.5)
	5-14 years	42,900 (34,400 to 53,500)	3,789.1 (3,039.6 to 4,723.2)	19,300 (15,300 to 24,100)	1,330.4 (1,054.3 to 1,663.5)	17,800 (14,300 to 21,900)	1,218.5 (980.4 to 1,501.3)	17,300 (14,100 to 21,600)	1,180.4 (959.1 to 1,470.6)	-64.9 (-68.9 to -59.4)	-8.4 (-18.3 to 3.3)
	15-49 years	39,300 (35,300 to 44,000)	2,112.2 (1,898.8 to 2,365.0)	46,700 (41,800 to 51,900)	1,197.9 (1,073.6 to 1,330.9)	43,300 (39,000 to 47,700)	1,094.8 (987.1 to 1,206.3)	42,600 (38,100 to 46,700)	1,065.5 (952.9 to 1,167.9)	-43.3 (-48.5 to -37.4)	-8.6 (-16.7 to -0.5)

Location	Age Group	1990		2019		2020		2021		Percent Incidence Rate Change from 1990-2019 (95% UI)	Percent Incidence Rate Change from 2019-2021 (95% UI)
		Episodes (95% UI)	Rate (95% UI)	Episodes (95% UI)	Rate (95% UI)	Episodes (95% UI)	Rate (95% UI)	Episodes (95% UI)	Rate (95% UI)		
	50-69 years	29,800 (26,200 to 33,900)	7,851.8 (6,906.6 to 8,941.1)	41,600 (36,900 to 47,300)	4,993.9 (4,427.1 to 5,680.5)	38,100 (34,400 to 42,400)	4,441.0 (4,005.7 to 4,940.0)	38,400 (34,400 to 42,800)	4,331.2 (3,879.8 to 4,831.8)	-36.4 (-43.3 to -27.0)	-11.1 (-19.9 to -0.5)
	70+ years	19,600 (16,800 to 22,500)	22,034.9 (18,984.5 to 25,402.6)	36,500 (32,200 to 41,600)	20,453.1 (18,060.1 to 23,307.4)	32,500 (28,800 to 36,900)	17,393.1 (15,389.2 to 19,744.9)	32,600 (29,000 to 37,200)	16,927.4 (15,067.2 to 19,301.1)	-7.2 (-18.0 to 3.6)	-15.0 (-23.2 to -6.0)
**Malaysia**	All Ages	713,000 (660,000 to 770,000)	4,038.2 (3,734.9 to 4,360.8)	1,260,000 (1,170,000 to 1,360,000)	4,030.9 (3,754.9 to 4,346.4)	1,210,000 (1,120,000 to 1,310,000)	3,821.0 (3,554.5 to 4,142.1)	1,140,000 (1,060,000 to 1,230,000)	3,592.8 (3,341.8 to 3,855.5)	-0.2 (-7.7 to 8.0)	-5.2 (-11.1 to 2.3)
	Under 5	218,000 (185,000 to 262,000)	9,163.2 (7,784.1 to 11,008.1)	84,300 (69,400 to 101,000)	3,373.6 (2,775.8 to 4,045.5)	76,700 (63,400 to 92,200)	3,089.1 (2,551.0 to 3,714.3)	63,900 (52,600 to 76,800)	2,599.2 (2,138.1 to 3,122.1)	-63.2 (-67.5 to -58.8)	-8.4 (-17.7 to 1.6)
	5-14 years	104,000 (82,300 to 128,000)	2,487.0 (1,962.8 to 3,062.9)	75,400 (58,800 to 94,700)	1,458.3 (1,136.5 to 1,832.2)	71,800 (56,300 to 89,700)	1,390.9 (1,090.7 to 1,737.1)	66,900 (53,500 to 83,300)	1,298.7 (1,037.9 to 1,615.0)	-41.4 (-48.4 to -34.6)	-4.6 (-12.7 to 4.5)
	15-49 years	164,000 (147,000 to 183,000)	1,822.9 (1,635.3 to 2,030.9)	315,000 (281,000 to 349,000)	1,804.6 (1,613.1 to 2,001.3)	305,000 (277,000 to 345,000)	1,734.8 (1,571.4 to 1,959.0)	288,000 (258,000 to 320,000)	1,623.7 (1,455.2 to 1,801.3)	-1.0 (-9.5 to 8.3)	-3.9 (-12.2 to 5.8)
	50-69 years	113,000 (99,900 to 125,000)	6,827.2 (6,053.4 to 7,595.6)	397,000 (356,000 to 444,000)	8,183.4 (7,343.2 to 9,156.2)	382,000 (346,000 to 430,000)	7,714.7 (6,977.2 to 8,691.0)	363,000 (325,000 to 403,000)	7,218.3 (6,452.5 to 8,005.3)	19.9 (7.5 to 33.7)	-5.7 (-14.1 to 4.7)
	70+ years	114,000 (101,000 to 129,000)	26,206.8 (23,133.5 to 29,649.7)	388,000 (344,000 to 433,000)	30,251.8 (26,801.1 to 33,754.3)	369,000 (327,000 to 423,000)	27,453.6 (24,330.7 to 31,438.3)	360,000 (319,000 to 410,000)	25,490.5 (22,535.6 to 28,976.3)	15.4 (1.7 to 29.1)	-9.2 (-18.2 to 2.0)
**Maldives**	All Ages	7,140 (6,460 to 7,920)	3,213.6 (2,907.4 to 3,561.4)	7,690 (7,120 to 8,300)	1,577.2 (1,460.2 to 1,701.0)	7,170 (6,690 to 7,700)	1,425.7 (1,330.3 to 1,530.7)	6,950 (6,480 to 7,440)	1,343.6 (1,252.7 to 1,438.2)	-50.9 (-54.7 to -45.8)	-9.6 (-14.7 to -4.2)
	Under 5	3,470 (2,940 to 4,090)	8,308.7 (7,040.2 to 9,788.4)	902 (767 to 1,060)	2,717.0 (2,309.5 to 3,180.4)	739 (623 to 872)	2,273.8 (1,916.3 to 2,683.1)	620 (524 to 725)	1,950.8 (1,649.7 to 2,282.2)	-67.3 (-70.7 to -63.6)	-16.3 (-23.3 to -8.3)
	5-14 years	1,110 (880 to 1,400)	1,755.6 (1,391.2 to 2,220.4)	791 (602 to 1,020)	1,176.9 (896.4 to 1,515.3)	733 (558 to 959)	1,079.4 (821.5 to 1,411.5)	697 (534 to 902)	1,018.5 (780.2 to 1,318.1)	-33.0 (-40.1 to -25.9)	-8.3 (-14.7 to -0.3)
	15-49 years	1,230 (1,100 to 1,380)	1,296.2 (1,152.3 to 1,454.2)	2,780 (2,430 to 3,200)	875.6 (766.0 to 1,008.0)	2,700 (2,350 to 3,090)	822.6 (715.4 to 940.3)	2,660 (2,320 to 3,040)	785.6 (686.2 to 898.3)	-32.5 (-39.2 to -25.1)	-6.0 (-14.9 to 2.6)
	50-69 years	904 (794 to 1,000)	4,761.7 (4,182.1 to 5,282.8)	1,550 (1,380 to 1,750)	2,727.9 (2,423.1 to 3,073.2)	1,490 (1,330 to 1,650)	2,447.4 (2,186.6 to 2,706.9)	1,510 (1,360 to 1,680)	2,323.3 (2,089.4 to 2,582.5)	-42.7 (-48.3 to -35.3)	-10.3 (-18.0 to -0.9)
	70+ years	422 (370 to 481)	14,057.8 (12,333.9 to 16,026.5)	1,670 (1,450 to 1,890)	12,802.4 (11,122.1 to 14,489.0)	1,510 (1,320 to 1,700)	11,314.6 (9,929.2 to 12,792.6)	1,470 (1,300 to 1,640)	10,776.4 (9,591.7 to 12,077.7)	-8.9 (-22.2 to 5.7)	-11.6 (-20.1 to -0.9)
**Mauritius**	All Ages	38,800 (36,200 to 41,800)	3,542.5 (3,302.1 to 3,817.1)	35,000 (32,700 to 37,400)	2,745.7 (2,567.6 to 2,937.5)	31,600 (29,400 to 34,100)	2,481.2 (2,309.9 to 2,678.1)	29,800 (27,800 to 32,000)	2,344.1 (2,187.3 to 2,513.5)	-22.5 (-27.5 to -16.6)	-9.6 (-15.0 to -3.8)
	Under 5	8,880 (7,570 to 10,600)	8,423.5 (7,180.9 to 10,034.8)	2,780 (2,290 to 3,320)	4,288.3 (3,543.7 to 5,130.4)	2,240 (1,890 to 2,690)	3,475.3 (2,927.2 to 4,168.5)	1,860 (1,550 to 2,230)	2,900.2 (2,404.5 to 3,462.3)	-49.1 (-55.6 to -40.5)	-19.0 (-26.9 to -10.6)

Location	Age Group	1990		2019		2020		2021		Percent Incidence Rate Change from 1990-2019 (95% UI)	Percent Incidence Rate Change from 2019-2021 (95% UI)
		Episodes (95% UI)	Rate (95% UI)	Episodes (95% UI)	Rate (95% UI)	Episodes (95% UI)	Rate (95% UI)	Episodes (95% UI)	Rate (95% UI)		
	5-14 years	5,130 (3,980 to 6,490)	2,283.8 (1,773.4 to 2,888.2)	2,070 (1,610 to 2,660)	1,362.5 (1,061.8 to 1,752.4)	1,830 (1,420 to 2,340)	1,242.3 (967.7 to 1,590.1)	1,680 (1,290 to 2,190)	1,175.7 (904.0 to 1,531.0)	-40.3 (-47.8 to -31.8)	-8.8 (-15.7 to -1.1)
	15-49 years	9,730 (8,620 to 10,700)	1,607.3 (1,424.1 to 1,772.7)	7,020 (6,320 to 7,740)	1,086.3 (978.1 to 1,198.3)	6,380 (5,770 to 7,070)	991.4 (897.5 to 1,099.7)	6,000 (5,380 to 6,680)	937.3 (840.4 to 1,043.6)	-32.4 (-38.5 to -26.1)	-8.7 (-15.6 to -0.6)
	50-69 years	8,220 (7,270 to 9,080)	6,444.5 (5,696.8 to 7,117.8)	11,000 (9,870 to 12,100)	3,424.3 (3,071.6 to 3,757.0)	10,100 (9,090 to 11,400)	3,124.2 (2,803.6 to 3,501.4)	9,480 (8,410 to 10,500)	2,920.1 (2,591.0 to 3,229.3)	-46.9 (-52.2 to -39.9)	-8.8 (-18.5 to 2.6)
	70+ years	6,860 (6,190 to 7,660)	20,825.6 (18,803.8 to 23,252.6)	12,100 (10,700 to 13,900)	13,505.5 (11,970.4 to 15,477.5)	11,000 (9,820 to 12,500)	11,674.1 (10,404.5 to 13,247.6)	10,800 (9,570 to 12,200)	10,767.7 (9,540.3 to 12,168.0)	-35.1 (-41.8 to -27.6)	-13.6 (-21.3 to -4.5)
**Myanmar**	All Ages	2,110,000 (1,930,000 to 2,290,000)	5,205.7 (4,779.2 to 5,656.5)	1,480,000 (1,390,000 to 1,570,000)	2,668.0 (2,507.9 to 2,830.9)	1,340,000 (1,250,000 to 1,420,000)	2,393.4 (2,229.4 to 2,549.6)	1,280,000 (1,200,000 to 1,350,000)	2,260.5 (2,124.3 to 2,398.0)	-48.7 (-52.4 to -44.8)	-10.3 (-15.3 to -4.7)
	Under 5	1,000,000 (867,000 to 1,160,000)	19,834.4 (17,190.3 to 23,090.1)	314,000 (270,000 to 365,000)	6,031.1 (5,186.1 to 7,002.3)	270,000 (234,000 to 320,000)	5,174.7 (4,482.1 to 6,121.5)	239,000 (208,000 to 275,000)	4,579.3 (3,970.9 to 5,266.5)	-69.6 (-72.3 to -65.9)	-14.2 (-22.6 to -5.6)
	5-14 years	268,000 (212,000 to 338,000)	2,751.2 (2,179.4 to 3,471.0)	117,000 (95,700 to 145,000)	1,136.2 (928.6 to 1,408.2)	109,000 (87,000 to 132,000)	1,049.1 (841.6 to 1,276.9)	104,000 (83,600 to 129,000)	1,001.7 (804.3 to 1,237.7)	-58.7 (-63.5 to -52.8)	-7.7 (-16.6 to 2.2)
	15-49 years	350,000 (307,000 to 389,000)	1,715.5 (1,502.6 to 1,907.5)	298,000 (272,000 to 328,000)	1,030.4 (939.4 to 1,131.2)	279,000 (255,000 to 309,000)	955.1 (872.4 to 1,058.2)	266,000 (241,000 to 295,000)	905.7 (821.1 to 1,003.6)	-39.9 (-45.5 to -33.7)	-7.3 (-13.9 to -0.2)
	50-69 years	287,000 (252,000 to 321,000)	6,734.7 (5,903.4 to 7,527.0)	375,000 (333,000 to 415,000)	4,317.8 (3,831.1 to 4,769.1)	351,000 (312,000 to 392,000)	3,918.4 (3,485.9 to 4,377.2)	343,000 (311,000 to 375,000)	3,743.2 (3,395.4 to 4,084.4)	-35.9 (-42.6 to -28.5)	-9.3 (-17.0 to -0.5)
	70+ years	200,000 (174,000 to 229,000)	20,405.1 (17,762.9 to 23,413.4)	370,000 (331,000 to 414,000)	17,462.1 (15,627.9 to 19,538.1)	328,000 (291,000 to 373,000)	15,211.9 (13,461.9 to 17,263.1)	322,000 (287,000 to 370,000)	14,428.0 (12,837.5 to 16,574.7)	-14.4 (-23.9 to -2.7)	-12.9 (-21.0 to -4.0)
**Philippines**	All Ages	4,080,000 (3,790,000 to 4,410,000)	6,474.4 (6,013.9 to 7,001.2)	4,700,000 (4,460,000 to 4,970,000)	4,255.1 (4,036.0 to 4,502.7)	4,260,000 (4,020,000 to 4,510,000)	3,804.5 (3,593.4 to 4,026.7)	4,220,000 (4,010,000 to 4,470,000)	3,725.7 (3,540.0 to 3,949.1)	-34.3 (-37.8 to -30.6)	-10.6 (-13.1 to -7.6)
	Under 5	1,610,000 (1,420,000 to 1,830,000)	17,419.9 (15,326.3 to 19,781.7)	645,000 (566,000 to 737,000)	5,645.2 (4,960.2 to 6,450.9)	527,000 (456,000 to 609,000)	4,662.7 (4,035.0 to 5,383.9)	487,000 (426,000 to 555,000)	4,345.5 (3,797.0 to 4,950.4)	-67.6 (-68.6 to -66.7)	-17.4 (-21.6 to -11.9)
	5-14 years	865,000 (718,000 to 1,030,000)	5,417.6 (4,496.3 to 6,460.8)	469,000 (395,000 to 555,000)	2,074.0 (1,749.4 to 2,457.1)	426,000 (361,000 to 510,000)	1,873.2 (1,588.0 to 2,243.5)	418,000 (355,000 to 492,000)	1,833.3 (1,559.0 to 2,158.6)	-61.7 (-63.6 to -59.5)	-9.7 (-12.2 to -6.5)
	15-49 years	817,000 (744,000 to 891,000)	2,625.5 (2,391.2 to 2,864.6)	1,190,000 (1,090,000 to 1,300,000)	2,046.3 (1,869.6 to 2,221.9)	1,110,000 (1,020,000 to 1,210,000)	1,873.3 (1,728.3 to 2,042.8)	1,100,000 (1,010,000 to 1,200,000)	1,829.5 (1,684.2 to 1,994.1)	-22.1 (-25.2 to -19.1)	-8.5 (-11.1 to -5.6)
	50-69 years	448,000 (404,000 to 492,000)	8,311.9 (7,499.4 to 9,135.0)	1,220,000 (1,110,000 to 1,330,000)	8,279.8 (7,565.6 to 9,007.6)	1,130,000 (1,040,000 to 1,240,000)	7,466.2 (6,856.9 to 8,136.1)	1,140,000 (1,040,000 to 1,240,000)	7,312.9 (6,675.8 to 7,936.0)	-0.4 (-4.2 to 4.5)	-9.8 (-12.4 to -7.0)

Location	Age Group	1990		2019		2020		2021		Percent Incidence Rate Change from 1990-2019 (95% UI)	Percent Incidence Rate Change from 2019-2021 (95% UI)
		Episodes (95% UI)	Rate (95% UI)	Episodes (95% UI)	Rate (95% UI)	Episodes (95% UI)	Rate (95% UI)	Episodes (95% UI)	Rate (95% UI)		
	70+ years	338,000 (304,000 to 374,000)	26,103.3 (23,433.9 to 28,868.5)	1,170,000 (1,080,000 to 1,290,000)	34,920.0 (32,007.0 to 38,382.6)	1,060,000 (965,000 to 1,170,000)	30,223.0 (27,435.1 to 33,257.4)	1,080,000 (985,000 to 1,190,000)	29,723.4 (27,225.6 to 32,973.8)	33.8 (26.7 to 41.4)	-13.5 (-16.1 to -10.1)
**Seychelles**	All Ages	3,430 (3,210 to 3,670)	4,709.5 (4,409.9 to 5,041.8)	4,610 (4,300 to 4,980)	4,458.4 (4,155.5 to 4,809.2)	4,200 (3,930 to 4,540)	4,023.2 (3,764.7 to 4,347.2)	4,060 (3,800 to 4,390)	3,853.5 (3,599.5 to 4,166.9)	-5.3 (-11.4 to 1.2)	-9.8 (-15.2 to -3.7)
	Under 5	665 (565 to 783)	8,257.1 (7,009.9 to 9,710.7)	383 (328 to 446)	4,816.3 (4,117.7 to 5,605.7)	320 (273 to 378)	4,043.8 (3,441.3 to 4,771.4)	275 (234 to 322)	3,489.7 (2,965.9 to 4,092.3)	-41.7 (-47.0 to -36.3)	-16.0 (-23.0 to -8.1)
	5-14 years	330 (260 to 414)	2,104.3 (1,659.4 to 2,639.2)	241 (192 to 299)	1,573.0 (1,255.2 to 1,954.6)	222 (173 to 280)	1,441.5 (1,122.1 to 1,816.4)	212 (171 to 261)	1,368.0 (1,100.7 to 1,679.1)	-25.2 (-31.8 to -17.9)	-8.4 (-16.8 to 2.0)
	15-49 years	774 (697 to 860)	2,089.0 (1,881.9 to 2,320.6)	1,090 (982 to 1,200)	2,011.7 (1,813.9 to 2,221.0)	1,010 (920 to 1,120)	1,872.5 (1,699.6 to 2,074.9)	963 (861 to 1,070)	1,778.1 (1,590.1 to 1,979.6)	-3.7 (-13.5 to 6.5)	-6.9 (-15.2 to 2.5)
	50-69 years	737 (658 to 816)	8,489.5 (7,582.8 to 9,408.5)	1,510 (1,370 to 1,680)	7,205.4 (6,551.0 to 8,032.4)	1,400 (1,270 to 1,560)	6,477.3 (5,849.3 to 7,184.2)	1,390 (1,250 to 1,550)	6,213.6 (5,580.1 to 6,929.8)	-15.1 (-23.3 to -5.6)	-10.1 (-18.5 to -0.5)
	70+ years	926 (829 to 1,060)	27,069.8 (24,227.0 to 30,937.6)	1,400 (1,230 to 1,590)	26,930.1 (23,694.5 to 30,684.6)	1,240 (1,120 to 1,400)	23,278.7 (20,959.2 to 26,237.8)	1,220 (1,080 to 1,400)	22,144.3 (19,564.5 to 25,315.9)	-0.5 (-10.5 to 9.7)	-13.6 (-22.3 to -5.1)
**Sri Lanka**	All Ages	552,000 (508,000 to 597,000)	3,225.0 (2,965.1 to 3,482.3)	582,000 (536,000 to 621,000)	2,643.7 (2,433.8 to 2,823.1)	508,000 (473,000 to 545,000)	2,294.2 (2,134.6 to 2,461.8)	500,000 (465,000 to 538,000)	2,245.1 (2,088.1 to 2,417.4)	-18.0 (-23.1 to -12.1)	-13.2 (-18.2 to -7.7)
	Under 5	138,000 (118,000 to 163,000)	7,796.8 (6,665.3 to 9,212.6)	53,800 (44,700 to 64,300)	3,298.7 (2,738.9 to 3,941.1)	40,100 (33,300 to 47,400)	2,509.5 (2,082.3 to 2,969.3)	35,600 (29,600 to 42,100)	2,276.9 (1,892.8 to 2,691.1)	-57.7 (-62.8 to -52.5)	-23.9 (-30.3 to -16.7)
	5-14 years	79,200 (62,300 to 100,000)	2,103.4 (1,655.4 to 2,658.4)	44,700 (35,200 to 55,900)	1,242.7 (979.3 to 1,552.8)	39,300 (31,000 to 49,700)	1,100.9 (868.1 to 1,390.9)	37,600 (29,700 to 47,100)	1,064.0 (840.5 to 1,332.1)	-40.9 (-48.0 to -32.9)	-11.4 (-18.1 to -3.7)
	15-49 years	139,000 (126,000 to 156,000)	1,518.1 (1,367.0 to 1,702.7)	121,000 (109,000 to 135,000)	1,111.7 (1,001.6 to 1,232.2)	108,000 (97,900 to 121,000)	987.7 (892.2 to 1,101.4)	105,000 (94,800 to 118,000)	955.4 (859.4 to 1,066.3)	-26.8 (-32.7 to -20.3)	-11.2 (-18.0 to -4.6)
	50-69 years	110,000 (97,800 to 125,000)	5,740.5 (5,096.1 to 6,537.2)	187,000 (166,000 to 209,000)	4,156.9 (3,688.4 to 4,644.1)	165,000 (147,000 to 183,000)	3,607.2 (3,211.1 to 4,005.7)	162,000 (144,000 to 180,000)	3,498.4 (3,119.8 to 3,892.3)	-27.6 (-35.5 to -18.8)	-13.2 (-23.3 to -4.1)
	70+ years	85,800 (75,600 to 97,500)	17,404.2 (15,322.7 to 19,771.7)	175,000 (156,000 to 197,000)	12,872.5 (11,463.8 to 14,463.6)	156,000 (137,000 to 177,000)	10,822.2 (9,506.5 to 12,288.8)	160,000 (141,000 to 180,000)	10,499.7 (9,287.6 to 11,807.2)	-26.0 (-32.9 to -17.9)	-15.9 (-23.0 to -6.7)
**Thailand**	All Ages	1,860,000 (1,720,000 to 2,000,000)	3,271.2 (3,024.5 to 3,532.0)	3,110,000 (2,860,000 to 3,370,000)	4,657.5 (4,284.4 to 5,044.4)	2,830,000 (2,620,000 to 3,040,000)	4,239.3 (3,923.3 to 4,559.5)	2,810,000 (2,590,000 to 3,070,000)	4,220.6 (3,877.2 to 4,602.0)	42.4 (29.6 to 57.5)	-9.0 (-14.6 to -3.3)
	Under 5	546,000 (461,000 to 633,000)	10,503.2 (8,867.6 to 12,162.9)	136,000 (113,000 to 162,000)	4,546.3 (3,753.8 to 5,390.3)	113,000 (92,400 to 135,000)	3,869.6 (3,177.6 to 4,638.0)	102,000 (84,200 to 121,000)	3,600.8 (2,978.0 to 4,295.8)	-56.7 (-61.3 to -52.1)	-14.9 (-22.3 to -6.0)
	5-14 years	264,000 (208,000 to 331,000)	2,261.4 (1,787.4 to 2,838.1)	102,000 (80,800 to 130,000)	1,404.2 (1,114.3 to 1,795.8)	91,500 (72,500 to 115,000)	1,288.4 (1,021.3 to 1,622.3)	86,800 (67,500 to 107,000)	1,250.8 (972.4 to 1,540.4)	-37.9 (-44.6 to -30.3)	-8.2 (-15.6 to 0.1)

Location	Age Group	1990		2019		2020		2021		Percent Incidence Rate Change from 1990-2019 (95% UI)	Percent Incidence Rate Change from 2019-2021 (95% UI)
		Episodes (95% UI)	Rate (95% UI)	Episodes (95% UI)	Rate (95% UI)	Episodes (95% UI)	Rate (95% UI)	Episodes (95% UI)	Rate (95% UI)		
	15-49 years	424,000 (375,000 to 477,000)	1,338.4 (1,185.6 to 1,506.1)	494,000 (444,000 to 553,000)	1,504.6 (1,350.6 to 1,683.9)	449,000 (403,000 to 495,000)	1,387.1 (1,244.2 to 1,529.1)	427,000 (381,000 to 471,000)	1,337.9 (1,193.4 to 1,474.9)	12.4 (0.7 to 24.9)	-7.8 (-14.7 to -0.8)
	50-69 years	334,000 (293,000 to 375,000)	4,965.0 (4,365.8 to 5,581.5)	999,000 (892,000 to 1,110,000)	5,575.4 (4,975.9 to 6,219.3)	924,000 (830,000 to 1,010,000)	5,024.1 (4,511.9 to 5,505.8)	917,000 (828,000 to 1,030,000)	4,886.8 (4,411.4 to 5,465.3)	12.3 (-1.1 to 27.1)	-9.9 (-16.8 to -2.2)
	70+ years	290,000 (248,000 to 329,000)	18,949.6 (16,256.6 to 21,526.6)	1,380,000 (1,230,000 to 1,550,000)	24,074.5 (21,450.0 to 27,154.5)	1,250,000 (1,110,000 to 1,410,000)	21,009.9 (18,697.5 to 23,656.1)	1,280,000 (1,130,000 to 1,460,000)	20,570.8 (18,162.6 to 23,469.0)	27.0 (9.6 to 47.0)	-12.7 (-19.9 to -4.3)
**Timor-Leste**	All Ages	54,300 (49,400 to 59,700)	6,953.3 (6,320.3 to 7,643.2)	42,900 (39,900 to 46,300)	3,197.9 (2,973.0 to 3,451.9)	39,700 (37,100 to 42,400)	2,898.9 (2,706.9 to 3,093.0)	38,100 (35,700 to 40,600)	2,727.4 (2,553.0 to 2,908.0)	-54.0 (-57.5 to -49.8)	-9.3 (-13.9 to -4.7)
	Under 5	33,600 (29,300 to 38,800)	23,971.3 (20,939.6 to 27,704.0)	10,800 (9,200 to 12,700)	6,072.2 (5,190.6 to 7,172.2)	9,670 (8,260 to 11,100)	5,349.4 (4,564.7 to 6,164.6)	8,930 (7,530 to 10,600)	4,830.0 (4,073.8 to 5,709.2)	-74.7 (-77.2 to -71.5)	-11.9 (-19.0 to -3.3)
	5-14 years	6,740 (5,180 to 8,410)	3,501.7 (2,689.8 to 4,367.2)	4,740 (3,860 to 5,740)	1,432.8 (1,167.6 to 1,735.7)	4,440 (3,610 to 5,490)	1,333.2 (1,084.5 to 1,648.6)	4,330 (3,480 to 5,310)	1,288.4 (1,035.3 to 1,581.8)	-59.1 (-64.1 to -52.8)	-7.0 (-16.9 to 2.4)
	15-49 years	7,630 (6,760 to 8,520)	1,994.2 (1,764.6 to 2,226.0)	8,430 (7,570 to 9,290)	1,287.8 (1,156.2 to 1,419.3)	8,050 (7,200 to 9,070)	1,197.0 (1,070.2 to 1,349.3)	7,870 (7,060 to 8,740)	1,140.9 (1,023.5 to 1,266.6)	-35.4 (-40.4 to -29.7)	-7.1 (-13.7 to 0.6)
	50-69 years	4,120 (3,600 to 4,680)	7,203.7 (6,297.1 to 8,182.3)	9,500 (8,360 to 10,600)	6,840.6 (6,016.7 to 7,669.3)	8,410 (7,430 to 9,380)	6,061.2 (5,357.6 to 6,763.2)	8,130 (7,280 to 9,070)	5,715.9 (5,120.1 to 6,375.5)	-5.0 (-15.3 to 6.7)	-11.4 (-19.8 to -1.6)
	70+ years	2,250 (1,970 to 2,530)	26,351.2 (23,128.7 to 29,669.9)	9,450 (8,410 to 10,800)	23,899.2 (21,255.8 to 27,382.2)	9,120 (8,050 to 10,300)	20,610.6 (18,180.5 to 23,369.6)	8,870 (7,900 to 10,100)	19,610.5 (17,461.4 to 22,338.7)	-9.3 (-19.2 to 1.4)	-13.8 (-22.1 to -4.4)
**Viet Nam**	All Ages	2,480,000 (2,260,000 to 2,730,000)	3,633.4 (3,306.7 to 3,996.8)	2,080,000 (1,950,000 to 2,230,000)	2,117.3 (1,982.2 to 2,262.4)	1,930,000 (1,790,000 to 2,080,000)	1,940.8 (1,802.8 to 2,092.8)	1,810,000 (1,690,000 to 1,930,000)	1,804.5 (1,690.0 to 1,926.0)	-41.7 (-45.8 to -37.2)	-8.3 (-13.3 to -2.3)
	Under 5	1,150,000 (996,000 to 1,370,000)	12,258.0 (10,590.0 to 14,553.2)	343,000 (297,000 to 399,000)	4,099.8 (3,544.4 to 4,760.3)	291,000 (247,000 to 346,000)	3,524.2 (2,988.0 to 4,181.2)	241,000 (208,000 to 279,000)	2,957.6 (2,558.2 to 3,431.4)	-66.6 (-69.9 to -63.1)	-14.0 (-23.1 to -3.4)
	5-14 years	286,000 (216,000 to 365,000)	1,669.6 (1,264.8 to 2,136.8)	170,000 (132,000 to 218,000)	1,058.7 (821.9 to 1,362.5)	161,000 (125,000 to 205,000)	985.5 (766.2 to 1,254.8)	152,000 (117,000 to 195,000)	916.7 (702.8 to 1,171.9)	-36.6 (-42.8 to -30.4)	-6.9 (-14.9 to 2.6)
	15-49 years	372,000 (329,000 to 420,000)	1,133.5 (1,002.9 to 1,282.0)	466,000 (416,000 to 518,000)	894.7 (798.7 to 993.4)	440,000 (395,000 to 489,000)	844.9 (757.7 to 937.8)	411,000 (368,000 to 454,000)	788.8 (707.4 to 871.8)	-21.1 (-26.9 to -13.1)	-5.6 (-12.9 to 2.2)
	50-69 years	319,000 (281,000 to 363,000)	4,636.9 (4,081.6 to 5,262.6)	558,000 (496,000 to 621,000)	3,146.6 (2,800.1 to 3,505.0)	541,000 (477,000 to 604,000)	2,931.5 (2,582.5 to 3,275.4)	525,000 (473,000 to 586,000)	2,767.3 (2,496.5 to 3,091.9)	-32.1 (-38.8 to -23.9)	-6.8 (-15.4 to 2.7)
	70+ years	349,000 (308,000 to 393,000)	17,110.0 (15,088.9 to 19,281.3)	548,000 (486,000 to 613,000)	12,987.4 (11,523.4 to 14,533.3)	495,000 (435,000 to 559,000)	11,700.0 (10,280.4 to 13,195.0)	481,000 (419,000 to 546,000)	10,761.0 (9,388.1 to 12,221.1)	-24.1 (-32.8 to -14.5)	-9.9 (-18.4 to -1.5)

Location	Age Group	1990		2019		2020		2021		Percent Incidence Rate Change from 1990-2019 (95% UI)	Percent Incidence Rate Change from 2019-2021 (95% UI)
		Episodes (95% UI)	Rate (95% UI)	Episodes (95% UI)	Rate (95% UI)	Episodes (95% UI)	Rate (95% UI)	Episodes (95% UI)	Rate (95% UI)		
**Angola**	All Ages	798,000 (731,000 to 860,000)	7,761.4 (7,109.0 to 8,366.7)	1,170,000 (1,100,000 to 1,260,000)	3,828.7 (3,581.4 to 4,105.7)	1,180,000 (1,080,000 to 1,270,000)	3,724.9 (3,412.6 to 4,009.6)	1,180,000 (1,090,000 to 1,270,000)	3,597.0 (3,325.3 to 3,897.3)	-50.7 (-53.5 to -46.9)	-2.7 (-8.2 to 3.2)
	Under 5	355,000 (307,000 to 407,000)	18,166.4 (15,746.6 to 20,856.8)	310,000 (274,000 to 358,000)	5,665.5 (5,004.0 to 6,538.7)	301,000 (258,000 to 352,000)	5,408.1 (4,640.3 to 6,325.7)	294,000 (252,000 to 341,000)	5,217.9 (4,478.3 to 6,050.5)	-68.8 (-71.4 to -65.6)	-4.5 (-14.0 to 4.4)
	5-14 years	103,000 (83,000 to 127,000)	3,728.4 (3,004.6 to 4,583.8)	174,000 (142,000 to 211,000)	1,934.6 (1,572.9 to 2,347.2)	182,000 (147,000 to 218,000)	1,952.0 (1,575.0 to 2,344.9)	178,000 (142,000 to 216,000)	1,846.7 (1,475.6 to 2,250.5)	-48.1 (-54.2 to -39.9)	0.9 (-8.8 to 12.3)
	15-49 years	175,000 (156,000 to 195,000)	3,755.2 (3,359.8 to 4,185.7)	342,000 (307,000 to 379,000)	2,520.8 (2,261.8 to 2,797.5)	349,000 (314,000 to 384,000)	2,474.2 (2,225.6 to 2,721.2)	352,000 (317,000 to 393,000)	2,401.1 (2,162.4 to 2,680.4)	-32.9 (-39.1 to -25.8)	-1.9 (-10.6 to 7.9)
	50-69 years	116,000 (102,000 to 130,000)	14,841.6 (13,093.4 to 16,673.5)	234,000 (208,000 to 262,000)	10,450.3 (9,294.2 to 11,661.4)	234,000 (207,000 to 258,000)	10,053.5 (8,889.1 to 11,121.0)	238,000 (210,000 to 270,000)	9,918.2 (8,758.9 to 11,256.7)	-29.6 (-37.4 to -20.5)	-3.8 (-13.0 to 5.8)
	70+ years	49,600 (43,500 to 56,700)	38,661.6 (33,942.7 to 44,176.7)	113,000 (98,200 to 128,000)	30,540.9 (26,624.5 to 34,764.7)	115,000 (100,000 to 131,000)	29,743.4 (25,866.3 to 33,908.9)	115,000 (100,000 to 134,000)	28,703.3 (25,028.5 to 33,431.3)	-21.0 (-31.4 to -10.5)	-2.6 (-13.6 to 10.5)
**Central African Republic**	All Ages	230,000 (212,000 to 248,000)	8,408.2 (7,771.2 to 9,094.3)	314,000 (295,000 to 337,000)	5,949.5 (5,583.6 to 6,378.1)	315,000 (290,000 to 339,000)	5,842.1 (5,372.3 to 6,289.8)	314,000 (291,000 to 338,000)	5,721.4 (5,304.5 to 6,166.1)	-29.2 (-32.7 to -25.9)	-1.8 (-7.3 to 3.5)
	Under 5	96,300 (83,600 to 111,000)	19,118.4 (16,602.4 to 22,135.6)	91,100 (79,500 to 103,000)	11,055.1 (9,642.7 to 12,497.4)	89,300 (77,400 to 104,000)	10,706.4 (9,286.2 to 12,426.5)	87,500 (75,100 to 101,000)	10,425.2 (8,957.6 to 12,097.0)	-42.2 (-46.2 to -37.6)	-3.2 (-12.8 to 5.4)
	5-14 years	27,600 (22,200 to 34,100)	3,838.1 (3,083.9 to 4,746.8)	40,800 (33,700 to 49,300)	2,906.0 (2,397.2 to 3,507.4)	42,500 (34,100 to 51,300)	2,977.7 (2,388.7 to 3,593.0)	40,900 (32,900 to 50,000)	2,832.9 (2,274.0 to 3,459.0)	-24.3 (-33.2 to -14.3)	2.5 (-9.8 to 15.0)
	15-49 years	49,900 (44,100 to 55,700)	4,015.5 (3,554.4 to 4,489.9)	90,300 (81,900 to 100,000)	3,546.8 (3,216.5 to 3,933.6)	92,300 (82,400 to 102,000)	3,535.3 (3,157.9 to 3,913.7)	93,000 (83,700 to 105,000)	3,490.3 (3,139.7 to 3,932.4)	-11.7 (-18.8 to -3.6)	-0.3 (-8.4 to 8.0)
	50-69 years	39,900 (35,500 to 44,800)	17,391.8 (15,503.1 to 19,549.2)	67,300 (59,500 to 76,500)	15,089.9 (13,359.2 to 17,164.3)	66,700 (59,000 to 74,700)	14,552.9 (12,861.6 to 16,297.8)	68,400 (60,400 to 77,500)	14,542.0 (12,827.1 to 16,477.7)	-13.2 (-21.6 to -4.0)	-3.6 (-12.1 to 8.4)
	70+ years	16,000 (13,800 to 18,200)	43,028.1 (37,218.5 to 49,032.6)	24,900 (21,600 to 28,900)	39,150.1 (33,921.5 to 45,497.2)	24,300 (21,100 to 28,000)	37,746.0 (32,711.2 to 43,487.8)	23,900 (20,800 to 27,500)	37,464.9 (32,616.4 to 43,033.1)	-9.0 (-19.1 to 2.6)	-3.6 (-14.0 to 8.3)
**Congo**	All Ages	152,000 (141,000 to 164,000)	6,308.9 (5,875.2 to 6,818.6)	206,000 (193,000 to 218,000)	3,954.1 (3,701.7 to 4,193.4)	206,000 (189,000 to 220,000)	3,878.2 (3,573.9 to 4,158.5)	206,000 (191,000 to 222,000)	3,823.0 (3,539.1 to 4,112.1)	-37.3 (-40.7 to -33.3)	-1.9 (-7.9 to 4.8)
	Under 5	48,700 (41,300 to 57,200)	12,348.4 (10,466.2 to 14,511.6)	29,700 (25,800 to 34,300)	4,521.1 (3,922.6 to 5,218.7)	27,900 (23,700 to 32,800)	4,328.8 (3,677.0 to 5,082.8)	26,500 (22,300 to 30,900)	4,183.6 (3,516.6 to 4,889.0)	-63.4 (-66.6 to -60.2)	-4.3 (-14.1 to 5.6)
	5-14 years	19,700 (15,800 to 23,900)	2,995.0 (2,404.6 to 3,634.6)	22,900 (19,000 to 27,400)	1,803.8 (1,495.5 to 2,159.9)	23,600 (19,000 to 28,200)	1,837.1 (1,474.5 to 2,195.4)	22,600 (18,500 to 27,400)	1,743.2 (1,423.4 to 2,115.1)	-39.8 (-47.4 to -31.7)	1.8 (-8.7 to 13.1)

Location	Age Group	1990		2019		2020		2021		Percent Incidence Rate Change from 1990-2019 (95% UI)	Percent Incidence Rate Change from 2019-2021 (95% UI)
		Episodes (95% UI)	Rate (95% UI)	Episodes (95% UI)	Rate (95% UI)	Episodes (95% UI)	Rate (95% UI)	Episodes (95% UI)	Rate (95% UI)		
	15-49 years	38,000 (34,100 to 42,400)	3,435.7 (3,086.0 to 3,837.2)	69,700 (62,800 to 77,100)	2,594.8 (2,341.2 to 2,871.9)	70,100 (62,700 to 77,700)	2,548.5 (2,280.0 to 2,823.1)	71,300 (64,300 to 79,200)	2,530.0 (2,283.4 to 2,811.7)	-24.5 (-31.1 to -17.9)	-1.8 (-9.3 to 6.3)
	50-69 years	29,200 (25,900 to 32,800)	14,649.6 (12,970.9 to 16,475.4)	53,400 (47,200 to 59,200)	10,705.2 (9,464.8 to 11,869.1)	54,200 (47,300 to 61,000)	10,321.4 (8,998.2 to 11,602.4)	56,200 (49,700 to 63,300)	10,245.9 (9,061.9 to 11,547.8)	-26.9 (-34.8 to -18.7)	-3.6 (-13.0 to 7.5)
	70+ years	15,900 (13,800 to 18,200)	36,451.8 (31,626.4 to 41,706.0)	30,100 (26,600 to 34,700)	32,007.7 (28,198.8 to 36,879.1)	29,700 (25,800 to 34,200)	31,728.3 (27,570.3 to 36,535.3)	29,600 (25,700 to 35,000)	30,771.3 (26,769.4 to 36,418.1)	-12.2 (-22.5 to 0.5)	-0.9 (-12.7 to 10.0)
**Democratic Republic of the Congo**	All Ages	3,300,000 (3,040,000 to 3,590,000)	8,647.8 (7,960.4 to 9,411.0)	4,200,000 (3,920,000 to 4,520,000)	4,888.2 (4,561.1 to 5,267.5)	4,210,000 (3,850,000 to 4,520,000)	4,785.3 (4,379.2 to 5,143.1)	4,210,000 (3,870,000 to 4,580,000)	4,679.0 (4,304.6 to 5,085.3)	-43.5 (-47.0 to -40.0)	-2.1 (-8.2 to 3.7)
	Under 5	1,350,000 (1,150,000 to 1,570,000)	18,459.2 (15,774.2 to 21,487.7)	824,000 (711,000 to 957,000)	6,088.3 (5,252.7 to 7,070.7)	787,000 (665,000 to 930,000)	5,805.9 (4,900.7 to 6,857.0)	761,000 (645,000 to 901,000)	5,612.3 (4,756.0 to 6,644.9)	-67.0 (-70.0 to -63.8)	-4.6 (-13.7 to 3.5)
	5-14 years	440,000 (349,000 to 542,000)	4,227.9 (3,354.8 to 5,211.6)	581,000 (470,000 to 699,000)	2,475.6 (2,000.8 to 2,979.5)	603,000 (482,000 to 740,000)	2,516.6 (2,010.6 to 3,088.3)	586,000 (468,000 to 730,000)	2,399.0 (1,913.0 to 2,987.0)	-41.4 (-47.8 to -33.6)	1.7 (-8.8 to 13.2)
	15-49 years	710,000 (628,000 to 796,000)	4,231.2 (3,741.2 to 4,742.3)	1,340,000 (1,190,000 to 1,500,000)	3,285.8 (2,933.6 to 3,689.4)	1,370,000 (1,220,000 to 1,530,000)	3,261.1 (2,908.9 to 3,654.8)	1,380,000 (1,240,000 to 1,550,000)	3,194.8 (2,853.7 to 3,568.3)	-22.3 (-29.0 to -14.8)	-0.8 (-8.9 to 7.9)
	50-69 years	537,000 (468,000 to 608,000)	17,364.5 (15,139.7 to 19,660.0)	969,000 (855,000 to 1,100,000)	13,970.9 (12,336.0 to 15,905.7)	973,000 (859,000 to 1,100,000)	13,472.2 (11,893.3 to 15,283.8)	1,000,000 (881,000 to 1,130,000)	13,482.0 (11,847.3 to 15,168.3)	-19.5 (-27.9 to -10.6)	-3.6 (-13.1 to 6.4)
	70+ years	265,000 (229,000 to 302,000)	46,843.5 (40,406.2 to 53,421.2)	486,000 (425,000 to 555,000)	39,335.2 (34,420.3 to 44,962.2)	477,000 (413,000 to 549,000)	38,742.3 (33,526.0 to 44,597.6)	478,000 (411,000 to 552,000)	37,788.8 (32,540.0 to 43,617.1)	-16.0 (-25.0 to -4.6)	-1.5 (-12.7 to 11.1)
**Equatorial Guinea**	All Ages	32,600 (30,100 to 35,000)	7,702.4 (7,114.8 to 8,280.5)	41,800 (38,900 to 44,500)	2,920.0 (2,716.2 to 3,109.3)	42,600 (39,400 to 45,900)	2,892.3 (2,677.7 to 3,115.2)	42,900 (39,700 to 46,300)	2,837.9 (2,627.9 to 3,059.3)	-62.1 (-64.3 to -59.6)	-0.9 (-6.9 to 4.9)
	Under 5	12,700 (10,900 to 14,700)	15,461.0 (13,207.8 to 17,860.3)	6,460 (5,530 to 7,480)	3,389.2 (2,902.8 to 3,929.9)	6,200 (5,320 to 7,250)	3,281.5 (2,811.8 to 3,833.5)	6,020 (5,140 to 7,050)	3,211.7 (2,742.3 to 3,763.2)	-78.1 (-80.0 to -75.8)	-3.2 (-10.8 to 4.0)
	5-14 years	4,210 (3,410 to 5,150)	3,674.7 (2,973.4 to 4,493.1)	6,050 (4,840 to 7,360)	1,569.7 (1,254.1 to 1,907.9)	6,290 (5,100 to 7,660)	1,602.3 (1,300.5 to 1,951.3)	6,080 (4,970 to 7,430)	1,528.4 (1,250.7 to 1,869.2)	-57.3 (-62.0 to -51.2)	2.1 (-7.9 to 14.0)
	15-49 years	6,740 (6,030 to 7,510)	3,729.5 (3,337.4 to 4,155.2)	14,900 (13,400 to 16,600)	1,994.6 (1,786.1 to 2,218.8)	15,500 (13,800 to 17,500)	1,989.5 (1,768.1 to 2,242.5)	16,100 (14,400 to 17,900)	1,979.3 (1,769.7 to 2,208.8)	-46.5 (-51.2 to -42.1)	-0.3 (-8.7 to 8.2)
	50-69 years	5,900 (5,190 to 6,590)	15,694.3 (13,808.6 to 17,532.7)	8,430 (7,380 to 9,340)	9,466.7 (8,292.8 to 10,487.3)	8,700 (7,770 to 9,640)	9,308.2 (8,307.3 to 10,312.4)	8,880 (7,800 to 9,900)	9,167.6 (8,050.0 to 10,213.3)	-39.7 (-45.6 to -33.6)	-1.7 (-10.0 to 7.8)

Location	Age Group	1990		2019		2020		2021		Percent Incidence Rate Change from 1990-2019 (95% UI)	Percent Incidence Rate Change from 2019-2021 (95% UI)
		Episodes (95% UI)	Rate (95% UI)	Episodes (95% UI)	Rate (95% UI)	Episodes (95% UI)	Rate (95% UI)	Episodes (95% UI)	Rate (95% UI)		
	70+ years	2,990 (2,580 to 3,470)	39,889.7 (34,317.5 to 46,237.2)	5,950 (5,290 to 6,820)	31,650.9 (28,116.9 to 36,237.9)	5,900 (5,130 to 6,780)	31,728.1 (27,592.5 to 36,499.0)	5,880 (5,100 to 6,770)	30,731.2 (26,632.3 to 35,380.5)	-20.7 (-29.3 to -11.1)	0.2 (-10.1 to 11.4)
**Gabon**	All Ages	71,600 (66,700 to 76,900)	7,275.2 (6,778.0 to 7,815.1)	78,000 (73,300 to 82,500)	4,418.4 (4,153.0 to 4,675.0)	77,700 (71,700 to 83,000)	4,340.2 (4,001.2 to 4,634.5)	77,500 (71,900 to 83,400)	4,268.2 (3,958.6 to 4,593.5)	-39.3 (-42.7 to -35.8)	-1.8 (-8.1 to 3.9)
	Under 5	20,900 (18,000 to 24,400)	13,403.1 (11,546.3 to 15,619.5)	10,900 (9,290 to 12,500)	4,951.8 (4,233.0 to 5,679.7)	10,300 (8,800 to 12,100)	4,763.7 (4,064.2 to 5,583.9)	9,830 (8,360 to 11,500)	4,603.3 (3,915.9 to 5,387.2)	-63.1 (-65.9 to -59.8)	-3.8 (-13.1 to 4.7)
	5-14 years	8,610 (7,010 to 10,600)	3,425.7 (2,790.0 to 4,206.3)	7,950 (6,450 to 9,910)	1,899.1 (1,540.9 to 2,366.6)	8,160 (6,530 to 9,780)	1,931.0 (1,545.7 to 2,313.5)	7,830 (6,330 to 9,510)	1,839.6 (1,488.2 to 2,234.3)	-44.6 (-50.7 to -37.3)	1.7 (-10.8 to 15.3)
	15-49 years	15,800 (14,200 to 17,900)	3,542.2 (3,169.1 to 4,003.7)	22,900 (20,500 to 25,400)	2,571.5 (2,305.6 to 2,858.0)	23,200 (20,800 to 25,900)	2,556.0 (2,286.4 to 2,851.7)	23,300 (21,000 to 26,000)	2,512.7 (2,265.6 to 2,805.8)	-27.4 (-33.2 to -20.4)	-0.6 (-9.8 to 7.2)
	50-69 years	14,900 (13,000 to 17,000)	14,767.3 (12,965.3 to 16,868.3)	22,800 (20,200 to 25,000)	11,523.5 (10,202.4 to 12,640.8)	23,100 (20,300 to 25,800)	11,265.0 (9,899.2 to 12,567.4)	23,300 (20,600 to 26,200)	11,147.7 (9,859.4 to 12,548.1)	-22.0 (-29.8 to -12.5)	-2.2 (-11.9 to 8.0)
	70+ years	11,300 (10,100 to 12,800)	40,089.8 (35,646.7 to 45,529.4)	13,500 (11,900 to 15,400)	34,000.2 (30,063.0 to 38,668.8)	12,900 (11,300 to 15,200)	33,458.2 (29,186.8 to 39,231.9)	13,300 (11,500 to 15,300)	32,721.3 (28,409.5 to 37,706.3)	-15.2 (-23.7 to -3.9)	-1.6 (-11.4 to 9.9)
**Burundi**	All Ages	598,000 (557,000 to 646,000)	10,762.6 (10,030.7 to 11,628.1)	811,000 (759,000 to 864,000)	6,572.7 (6,153.2 to 7,000.0)	758,000 (705,000 to 811,000)	5,921.9 (5,508.0 to 6,332.6)	786,000 (728,000 to 847,000)	5,943.7 (5,506.9 to 6,404.1)	-38.9 (-42.3 to -35.1)	-9.9 (-14.9 to -4.5)
	Under 5	204,000 (178,000 to 234,000)	18,970.1 (16,533.8 to 21,759.2)	167,000 (147,000 to 192,000)	8,072.8 (7,093.5 to 9,290.7)	144,000 (124,000 to 167,000)	6,799.4 (5,846.4 to 7,860.6)	149,000 (129,000 to 173,000)	6,886.0 (5,955.9 to 8,028.6)	-57.4 (-61.0 to -52.7)	-15.8 (-21.9 to -9.0)
	5-14 years	98,900 (80,000 to 121,000)	6,394.4 (5,174.7 to 7,809.8)	141,000 (116,000 to 172,000)	4,072.0 (3,343.8 to 4,951.7)	138,000 (114,000 to 165,000)	3,843.3 (3,163.7 to 4,609.0)	141,000 (115,000 to 171,000)	3,809.5 (3,119.6 to 4,630.0)	-36.3 (-43.4 to -28.0)	-5.6 (-16.9 to 6.9)
	15-49 years	144,000 (129,000 to 160,000)	5,933.0 (5,307.9 to 6,591.1)	253,000 (228,000 to 278,000)	4,409.2 (3,965.1 to 4,844.9)	246,000 (222,000 to 273,000)	4,099.7 (3,696.9 to 4,547.1)	256,000 (230,000 to 284,000)	4,100.7 (3,682.0 to 4,542.0)	-25.7 (-32.4 to -17.8)	-7.0 (-15.6 to 1.9)
	50-69 years	88,700 (78,000 to 100,000)	22,529.3 (19,805.4 to 25,443.1)	165,000 (144,000 to 184,000)	18,476.8 (16,211.4 to 20,671.4)	151,000 (133,000 to 172,000)	16,520.5 (14,537.0 to 18,809.5)	160,000 (141,000 to 179,000)	16,944.5 (14,921.8 to 18,957.8)	-18.0 (-27.7 to -8.1)	-10.6 (-19.4 to 0.4)
	70+ years	62,300 (53,400 to 71,100)	55,042.9 (47,218.3 to 62,856.5)	84,700 (74,400 to 97,200)	51,535.0 (45,273.1 to 59,089.5)	78,800 (68,700 to 90,400)	45,005.2 (39,286.1 to 51,683.7)	80,600 (69,400 to 93,700)	44,735.0 (38,517.2 to 52,040.2)	-6.4 (-17.7 to 7.4)	-12.7 (-22.4 to -3.9)
**Comoros**	All Ages	45,000 (41,400 to 48,700)	9,735.0 (8,954.0 to 10,527.9)	46,900 (43,800 to 50,000)	6,454.2 (6,031.2 to 6,874.5)	44,600 (41,500 to 47,500)	6,056.6 (5,634.8 to 6,450.1)	46,300 (43,400 to 49,300)	6,223.3 (5,831.2 to 6,618.7)	-33.7 (-37.9 to -28.9)	-6.2 (-10.9 to -1.3)

Location	Age Group	1990		2019		2020		2021		Percent Incidence Rate Change from 1990-2019 (95% UI)	Percent Incidence Rate Change from 2019-2021 (95% UI)
		Episodes (95% UI)	Rate (95% UI)	Episodes (95% UI)	Rate (95% UI)	Episodes (95% UI)	Rate (95% UI)	Episodes (95% UI)	Rate (95% UI)		
	Under 5	15,900 (13,800 to 18,500)	19,300.2 (16,778.3 to 22,394.1)	5,930 (5,160 to 6,760)	7,181.8 (6,253.4 to 8,195.4)	5,240 (4,550 to 6,020)	6,401.9 (5,560.5 to 7,352.9)	5,340 (4,620 to 6,150)	6,572.5 (5,687.1 to 7,566.1)	-62.8 (-66.0 to -59.4)	-10.9 (-17.4 to -3.4)
	5-14 years	7,140 (5,670 to 8,730)	5,480.1 (4,354.9 to 6,704.6)	5,020 (4,130 to 6,060)	3,161.3 (2,595.7 to 3,815.9)	4,920 (3,980 to 5,910)	3,097.0 (2,506.8 to 3,720.1)	4,900 (4,020 to 5,950)	3,082.7 (2,533.2 to 3,745.3)	-42.3 (-49.2 to -33.8)	-2.0 (-11.9 to 8.9)
	15-49 years	11,000 (9,740 to 12,300)	5,351.6 (4,755.6 to 6,024.4)	14,800 (13,200 to 16,500)	3,904.3 (3,481.2 to 4,350.8)	14,400 (13,100 to 16,000)	3,733.9 (3,385.9 to 4,150.9)	15,000 (13,600 to 16,700)	3,830.9 (3,459.5 to 4,252.0)	-27.0 (-34.0 to -19.9)	-4.4 (-12.1 to 3.1)
	50-69 years	7,120 (6,300 to 8,000)	19,275.7 (17,053.7 to 21,658.4)	11,900 (10,600 to 13,300)	14,250.0 (12,767.1 to 15,976.8)	11,400 (10,100 to 12,800)	13,193.6 (11,667.8 to 14,792.2)	12,300 (10,900 to 14,100)	13,699.4 (12,123.8 to 15,664.9)	-26.1 (-34.1 to -17.7)	-7.4 (-16.2 to 1.9)
	70+ years	3,900 (3,410 to 4,490)	48,200.6 (42,116.0 to 55,394.1)	9,270 (8,080 to 10,600)	41,707.9 (36,349.5 to 47,737.0)	8,540 (7,480 to 9,890)	38,319.2 (33,571.3 to 44,358.1)	8,770 (7,690 to 10,100)	38,667.6 (33,882.0 to 44,537.8)	-13.5 (-24.7 to -1.5)	-8.1 (-17.9 to 3.2)
**Djibouti**	All Ages	28,400 (26,200 to 30,600)	6,856.1 (6,312.7 to 7,383.5)	70,200 (65,700 to 74,800)	5,869.1 (5,493.6 to 6,254.4)	66,800 (62,200 to 71,200)	5,439.3 (5,064.7 to 5,799.8)	69,700 (65,300 to 74,200)	5,537.3 (5,191.3 to 5,893.6)	-14.4 (-19.0 to -9.0)	-7.3 (-12.5 to -2.4)
	Under 5	10,500 (8,940 to 12,000)	16,194.3 (13,841.1 to 18,617.3)	12,200 (10,600 to 14,200)	8,460.0 (7,312.5 to 9,814.2)	10,800 (9,380 to 12,500)	7,408.3 (6,457.1 to 8,582.1)	10,900 (9,480 to 12,600)	7,513.2 (6,505.3 to 8,662.9)	-47.8 (-51.7 to -42.8)	-12.4 (-19.3 to -4.4)
	5-14 years	4,450 (3,580 to 5,390)	4,065.6 (3,268.1 to 4,924.7)	8,980 (7,400 to 11,000)	3,534.9 (2,916.0 to 4,318.8)	8,900 (7,250 to 10,500)	3,412.3 (2,779.7 to 4,034.3)	9,020 (7,400 to 11,100)	3,374.2 (2,765.6 to 4,135.5)	-13.1 (-22.8 to -0.7)	-3.5 (-13.1 to 8.2)
	15-49 years	7,800 (6,970 to 8,690)	3,748.5 (3,352.4 to 4,175.7)	23,500 (21,200 to 26,500)	3,574.2 (3,233.5 to 4,035.8)	22,600 (20,600 to 25,000)	3,360.7 (3,052.9 to 3,717.2)	23,900 (21,600 to 26,300)	3,458.3 (3,132.5 to 3,810.7)	-4.6 (-13.6 to 3.5)	-6.0 (-13.3 to 2.4)
	50-69 years	4,030 (3,530 to 4,540)	14,445.9 (12,659.7 to 16,296.0)	17,200 (15,100 to 19,200)	14,296.7 (12,560.4 to 15,945.1)	16,600 (14,500 to 18,600)	13,079.0 (11,455.2 to 14,691.3)	17,700 (15,800 to 19,500)	13,331.0 (11,906.0 to 14,691.6)	-1.0 (-10.5 to 10.5)	-8.5 (-18.5 to 2.3)
	70+ years	1,660 (1,440 to 1,880)	39,045.9 (33,782.7 to 44,225.9)	8,300 (7,260 to 9,420)	40,838.2 (35,697.9 to 46,336.5)	7,920 (6,880 to 9,020)	37,040.0 (32,158.6 to 42,166.4)	8,110 (7,090 to 9,410)	37,015.5 (32,392.5 to 42,972.4)	4.6 (-8.0 to 17.7)	-9.3 (-18.9 to 0.6)
**Eritrea**	All Ages	365,000 (332,000 to 398,000)	10,720.4 (9,760.7 to 11,689.0)	460,000 (427,000 to 495,000)	7,281.0 (6,766.0 to 7,839.2)	429,000 (396,000 to 461,000)	6,645.2 (6,140.2 to 7,134.5)	444,000 (410,000 to 478,000)	6,726.4 (6,220.9 to 7,247.0)	-32.1 (-36.1 to -27.7)	-8.7 (-13.2 to -3.8)
	Under 5	140,000 (121,000 to 164,000)	22,487.8 (19,343.6 to 26,273.0)	90,000 (77,700 to 104,000)	10,016.5 (8,642.7 to 11,618.9)	77,600 (66,700 to 89,200)	8,541.8 (7,335.0 to 9,819.9)	79,400 (69,100 to 92,100)	8,652.0 (7,526.8 to 10,037.0)	-55.5 (-59.2 to -51.5)	-14.7 (-21.9 to -5.5)
	5-14 years	59,800 (47,500 to 74,200)	6,170.2 (4,905.4 to 7,663.9)	61,100 (49,400 to 74,600)	3,948.7 (3,194.0 to 4,821.9)	59,400 (48,000 to 71,900)	3,771.9 (3,048.1 to 4,563.2)	59,400 (47,700 to 73,500)	3,699.5 (2,966.1 to 4,573.0)	-36.0 (-42.5 to -29.4)	-4.5 (-12.7 to 5.0)
	15-49 years	96,300 (84,900 to 109,000)	6,249.1 (5,510.5 to 7,040.0)	167,000 (148,000 to 188,000)	5,103.8 (4,536.4 to 5,753.0)	159,000 (141,000 to 179,000)	4,768.4 (4,211.6 to 5,340.1)	165,000 (147,000 to 186,000)	4,832.5 (4,293.0 to 5,431.7)	-18.3 (-24.3 to -11.3)	-6.6 (-13.6 to 1.3)

Location	Age Group	1990		2019		2020		2021		Percent Incidence Rate Change from 1990-2019 (95% UI)	Percent Incidence Rate Change from 2019-2021 (95% UI)
		Episodes (95% UI)	Rate (95% UI)	Episodes (95% UI)	Rate (95% UI)	Episodes (95% UI)	Rate (95% UI)	Episodes (95% UI)	Rate (95% UI)		
	50-69 years	54,300 (46,300 to 63,000)	22,235.2 (18,938.6 to 25,784.2)	99,000 (86,100 to 113,000)	19,153.3 (16,664.4 to 21,910.3)	92,900 (81,600 to 105,000)	17,271.0 (15,173.0 to 19,609.2)	98,500 (85,200 to 113,000)	17,640.4 (15,259.4 to 20,310.9)	-13.9 (-21.8 to -4.8)	-9.8 (-18.1 to -1.1)
	70+ years	14,500 (12,300 to 16,700)	51,462.6 (43,643.7 to 59,389.7)	43,100 (37,200 to 49,900)	49,283.6 (42,534.7 to 57,089.0)	39,600 (33,800 to 46,300)	44,241.4 (37,763.9 to 51,717.6)	41,000 (34,800 to 47,700)	44,686.6 (37,951.1 to 51,991.9)	-4.2 (-16.5 to 8.6)	-10.2 (-20.3 to 1.8)
**Ethiopia**	All Ages	6,270,000 (5,840,000 to 6,730,000)	12,394.2 (11,543.2 to 13,314.6)	6,610,000 (6,210,000 to 7,010,000)	6,326.3 (5,944.8 to 6,704.7)	6,380,000 (5,980,000 to 6,750,000)	5,976.2 (5,606.9 to 6,328.1)	6,620,000 (6,230,000 to 7,020,000)	6,078.9 (5,719.5 to 6,440.4)	-49.0 (-51.1 to -46.8)	-5.5 (-8.2 to -2.1)
	Under 5	2,400,000 (2,110,000 to 2,750,000)	24,943.7 (21,927.4 to 28,516.1)	1,380,000 (1,220,000 to 1,580,000)	8,887.9 (7,818.1 to 10,126.6)	1,250,000 (1,090,000 to 1,420,000)	7,910.1 (6,924.3 to 8,998.2)	1,300,000 (1,140,000 to 1,490,000)	8,159.3 (7,163.0 to 9,326.4)	-64.4 (-65.9 to -62.7)	-11.0 (-15.1 to -5.8)
	5-14 years	974,000 (804,000 to 1,170,000)	6,615.0 (5,457.8 to 7,955.3)	927,000 (772,000 to 1,110,000)	3,323.3 (2,769.3 to 3,971.9)	931,000 (773,000 to 1,110,000)	3,310.9 (2,746.7 to 3,953.7)	938,000 (771,000 to 1,120,000)	3,304.0 (2,716.3 to 3,936.7)	-49.8 (-53.6 to -45.1)	-0.4 (-5.7 to 5.0)
	15-49 years	1,490,000 (1,340,000 to 1,620,000)	6,847.2 (6,155.2 to 7,456.6)	1,970,000 (1,800,000 to 2,160,000)	3,791.4 (3,457.1 to 4,149.0)	1,980,000 (1,810,000 to 2,180,000)	3,694.3 (3,382.6 to 4,074.2)	2,080,000 (1,900,000 to 2,270,000)	3,785.4 (3,449.8 to 4,126.3)	-44.6 (-47.4 to -41.2)	-2.6 (-6.4 to 1.7)
	50-69 years	953,000 (845,000 to 1,050,000)	25,609.8 (22,715.1 to 28,319.6)	1,210,000 (1,100,000 to 1,330,000)	17,007.7 (15,459.2 to 18,714.0)	1,180,000 (1,060,000 to 1,290,000)	15,962.1 (14,433.6 to 17,516.9)	1,230,000 (1,120,000 to 1,360,000)	16,289.7 (14,817.6 to 17,999.6)	-33.6 (-37.8 to -28.5)	-6.1 (-10.2 to -1.7)
	70+ years	450,000 (393,000 to 505,000)	58,178.1 (50,879.5 to 65,352.8)	1,120,000 (1,000,000 to 1,270,000)	58,035.3 (51,785.3 to 65,650.8)	1,050,000 (926,000 to 1,180,000)	54,087.7 (47,873.3 to 60,887.1)	1,060,000 (958,000 to 1,200,000)	53,942.7 (48,598.9 to 60,709.4)	-0.2 (-11.3 to 10.8)	-6.8 (-12.1 to -1.7)
**Kenya**	All Ages	2,220,000 (2,060,000 to 2,380,000)	9,587.1 (8,913.2 to 10,266.2)	4,200,000 (3,950,000 to 4,450,000)	8,644.6 (8,133.7 to 9,160.7)	3,970,000 (3,740,000 to 4,200,000)	8,033.7 (7,574.4 to 8,508.5)	4,080,000 (3,850,000 to 4,340,000)	8,149.9 (7,687.2 to 8,671.0)	-9.8 (-12.4 to -6.7)	-7.1 (-9.9 to -4.0)
	Under 5	776,000 (679,000 to 879,000)	18,085.1 (15,815.7 to 20,481.0)	840,000 (739,000 to 957,000)	13,678.3 (12,025.0 to 15,578.7)	539,000 (474,000 to 611,000)	8,907.4 (7,835.3 to 10,101.7)	540,000 (475,000 to 612,000)	9,080.4 (7,976.2 to 10,293.4)	-24.4 (-26.6 to -21.8)	-34.9 (-38.4 to -30.9)
	5-14 years	352,000 (285,000 to 428,000)	5,113.9 (4,137.2 to 6,218.4)	558,000 (462,000 to 667,000)	4,396.0 (3,640.0 to 5,253.1)	522,000 (434,000 to 617,000)	4,103.9 (3,412.4 to 4,847.6)	512,000 (421,000 to 606,000)	4,030.3 (3,312.2 to 4,767.9)	-14.0 (-18.1 to -8.7)	-6.6 (-11.0 to -2.4)
	15-49 years	515,000 (460,000 to 571,000)	5,072.0 (4,529.4 to 5,621.5)	1,230,000 (1,120,000 to 1,350,000)	4,956.2 (4,510.1 to 5,437.9)	1,290,000 (1,170,000 to 1,410,000)	5,062.7 (4,591.5 to 5,522.9)	1,350,000 (1,220,000 to 1,470,000)	5,146.0 (4,684.7 to 5,632.6)	-2.3 (-6.6 to 2.0)	2.1 (-0.9 to 5.6)
	50-69 years	321,000 (283,000 to 358,000)	22,137.2 (19,524.9 to 24,646.2)	944,000 (853,000 to 1,040,000)	23,422.5 (21,166.9 to 25,789.6)	981,000 (889,000 to 1,080,000)	23,469.1 (21,264.4 to 25,910.1)	1,030,000 (938,000 to 1,150,000)	23,921.4 (21,719.4 to 26,577.1)	5.8 (1.0 to 10.8)	0.2 (-2.9 to 3.6)
	70+ years	255,000 (226,000 to 285,000)	68,754.6 (60,926.0 to 76,852.5)	628,000 (565,000 to 701,000)	71,104.7 (64,004.0 to 79,377.8)	632,000 (570,000 to 709,000)	69,506.6 (62,660.0 to 77,944.1)	649,000 (579,000 to 730,000)	69,502.7 (62,085.4 to 78,215.3)	3.4 (-2.9 to 10.0)	-2.2 (-5.6 to 1.9)

Location	Age Group	1990		2019		2020		2021		Percent Incidence Rate Change from 1990-2019 (95% UI)	Percent Incidence Rate Change from 2019-2021 (95% UI)
		Episodes (95% UI)	Rate (95% UI)	Episodes (95% UI)	Rate (95% UI)	Episodes (95% UI)	Rate (95% UI)	Episodes (95% UI)	Rate (95% UI)		
**Madagascar**	All Ages	1,300,000 (1,200,000 to 1,400,000)	10,933.0 (10,122.2 to 11,746.6)	1,760,000 (1,640,000 to 1,890,000)	6,449.0 (6,008.5 to 6,914.6)	1,620,000 (1,510,000 to 1,740,000)	5,796.6 (5,404.1 to 6,219.4)	1,630,000 (1,500,000 to 1,770,000)	5,720.5 (5,250.7 to 6,214.1)	-41.0 (-44.4 to -37.4)	-10.1 (-15.5 to -5.4)
	Under 5	448,000 (397,000 to 507,000)	20,789.5 (18,412.4 to 23,507.3)	335,000 (292,000 to 383,000)	8,142.4 (7,093.6 to 9,302.8)	280,000 (243,000 to 319,000)	6,824.4 (5,929.0 to 7,757.9)	275,000 (234,000 to 321,000)	6,726.1 (5,714.1 to 7,848.1)	-60.8 (-64.4 to -57.0)	-16.2 (-23.0 to -6.8)
	5-14 years	250,000 (205,000 to 301,000)	7,588.7 (6,219.7 to 9,125.7)	276,000 (227,000 to 333,000)	3,733.9 (3,066.3 to 4,497.3)	267,000 (216,000 to 324,000)	3,544.8 (2,870.2 to 4,298.8)	259,000 (214,000 to 314,000)	3,392.9 (2,798.8 to 4,105.1)	-50.8 (-56.7 to -44.0)	-5.1 (-15.6 to 5.5)
	15-49 years	327,000 (295,000 to 361,000)	6,165.5 (5,568.3 to 6,807.3)	622,000 (557,000 to 695,000)	4,682.2 (4,198.9 to 5,237.0)	593,000 (533,000 to 656,000)	4,330.2 (3,892.1 to 4,792.7)	605,000 (543,000 to 678,000)	4,281.4 (3,842.7 to 4,800.6)	-24.1 (-29.7 to -16.9)	-7.5 (-15.9 to 0.7)
	50-69 years	176,000 (154,000 to 197,000)	19,001.7 (16,620.5 to 21,243.8)	376,000 (329,000 to 423,000)	17,291.8 (15,120.4 to 19,436.8)	346,000 (298,000 to 392,000)	15,272.3 (13,164.7 to 17,323.7)	360,000 (315,000 to 412,000)	15,377.8 (13,469.2 to 17,597.9)	-9.0 (-18.3 to 0.9)	-11.7 (-20.6 to -1.3)
	70+ years	99,500 (87,000 to 114,000)	46,371.9 (40,537.4 to 53,159.1)	152,000 (135,000 to 176,000)	43,781.5 (38,760.4 to 50,588.2)	134,000 (117,000 to 154,000)	37,795.2 (33,036.7 to 43,236.6)	135,000 (116,000 to 157,000)	37,052.6 (31,774.4 to 43,283.2)	-5.6 (-14.8 to 5.7)	-13.7 (-22.8 to -4.4)
**Malawi**	All Ages	952,000 (876,000 to 1,030,000)	9,711.5 (8,936.3 to 10,538.7)	1,040,000 (965,000 to 1,110,000)	5,582.9 (5,200.3 to 5,974.5)	939,000 (876,000 to 1,010,000)	4,935.3 (4,608.2 to 5,291.0)	959,000 (886,000 to 1,030,000)	4,930.5 (4,554.4 to 5,278.0)	-42.5 (-45.8 to -38.8)	-11.6 (-16.2 to -6.3)
	Under 5	397,000 (341,000 to 459,000)	20,895.6 (17,959.9 to 24,175.1)	239,000 (206,000 to 275,000)	8,749.1 (7,546.2 to 10,066.2)	192,000 (166,000 to 223,000)	7,045.6 (6,072.2 to 8,163.9)	195,000 (169,000 to 225,000)	7,166.1 (6,209.7 to 8,251.0)	-58.1 (-62.0 to -54.4)	-19.5 (-26.3 to -11.9)
	5-14 years	145,000 (117,000 to 178,000)	5,473.2 (4,423.4 to 6,714.2)	173,000 (140,000 to 208,000)	3,265.4 (2,637.7 to 3,922.8)	164,000 (135,000 to 199,000)	3,059.1 (2,523.5 to 3,709.1)	162,000 (132,000 to 195,000)	2,994.6 (2,449.3 to 3,606.1)	-40.3 (-46.0 to -33.8)	-6.3 (-15.8 to 4.5)
	15-49 years	200,000 (178,000 to 224,000)	4,545.3 (4,045.4 to 5,098.6)	304,000 (273,000 to 339,000)	3,379.4 (3,031.6 to 3,773.5)	292,000 (263,000 to 324,000)	3,129.1 (2,812.0 to 3,473.4)	301,000 (270,000 to 337,000)	3,110.8 (2,790.6 to 3,484.8)	-25.7 (-32.8 to -18.3)	-7.4 (-14.6 to 2.0)
	50-69 years	131,000 (115,000 to 149,000)	18,779.8 (16,426.2 to 21,363.9)	191,000 (171,000 to 211,000)	15,362.4 (13,798.3 to 17,017.4)	173,000 (155,000 to 193,000)	13,479.9 (12,066.9 to 14,972.6)	183,000 (162,000 to 202,000)	13,705.4 (12,186.3 to 15,172.8)	-18.2 (-26.7 to -7.3)	-12.3 (-20.6 to -4.1)
	70+ years	79,100 (69,400 to 90,900)	50,870.1 (44,593.3 to 58,458.3)	130,000 (114,000 to 148,000)	43,498.7 (38,383.7 to 49,841.4)	117,000 (101,000 to 133,000)	37,654.7 (32,653.6 to 42,877.0)	118,000 (104,000 to 137,000)	38,013.4 (33,357.3 to 43,993.2)	-14.5 (-25.1 to -2.2)	-13.4 (-21.7 to -4.0)
**Mozambique**	All Ages	1,130,000 (1,040,000 to 1,220,000)	8,456.1 (7,806.3 to 9,125.0)	1,500,000 (1,410,000 to 1,610,000)	5,102.9 (4,775.4 to 5,449.9)	1,390,000 (1,300,000 to 1,490,000)	4,597.9 (4,278.9 to 4,907.6)	1,430,000 (1,330,000 to 1,530,000)	4,603.8 (4,271.8 to 4,933.6)	-39.7 (-43.2 to -35.7)	-9.9 (-14.0 to -5.2)
	Under 5	419,000 (357,000 to 485,000)	17,402.7 (14,807.1 to 20,146.0)	305,000 (267,000 to 349,000)	6,019.4 (5,276.6 to 6,878.9)	263,000 (231,000 to 305,000)	5,135.0 (4,504.7 to 5,954.1)	269,000 (233,000 to 310,000)	5,189.7 (4,497.6 to 5,980.4)	-65.4 (-68.6 to -61.7)	-14.7 (-21.1 to -7.0)

Location	Age Group	1990		2019		2020		2021		Percent Incidence Rate Change from 1990-2019 (95% UI)	Percent Incidence Rate Change from 2019-2021 (95% UI)
		Episodes (95% UI)	Rate (95% UI)	Episodes (95% UI)	Rate (95% UI)	Episodes (95% UI)	Rate (95% UI)	Episodes (95% UI)	Rate (95% UI)		
	5-14 years	171,000 (138,000 to 213,000)	4,511.6 (3,639.4 to 5,611.5)	256,000 (209,000 to 311,000)	2,955.5 (2,405.5 to 3,586.1)	249,000 (202,000 to 299,000)	2,798.2 (2,276.2 to 3,371.0)	251,000 (206,000 to 303,000)	2,757.8 (2,262.7 to 3,333.2)	-34.5 (-40.9 to -26.3)	-5.3 (-16.0 to 5.5)
	15-49 years	232,000 (208,000 to 258,000)	4,017.0 (3,602.5 to 4,468.0)	451,000 (406,000 to 498,000)	3,381.4 (3,041.8 to 3,732.1)	432,000 (391,000 to 479,000)	3,127.9 (2,832.3 to 3,470.1)	449,000 (406,000 to 502,000)	3,145.4 (2,844.5 to 3,517.9)	-15.8 (-22.6 to -8.3)	-7.5 (-15.6 to 0.6)
	50-69 years	192,000 (172,000 to 214,000)	16,927.2 (15,171.7 to 18,880.2)	316,000 (282,000 to 356,000)	15,855.6 (14,120.5 to 17,849.6)	291,000 (257,000 to 322,000)	14,117.9 (12,474.4 to 15,611.5)	302,000 (264,000 to 337,000)	14,261.2 (12,498.0 to 15,945.5)	-6.3 (-15.3 to 2.8)	-11.0 (-18.8 to -3.5)
	70+ years	116,000 (100,000 to 132,000)	47,538.1 (41,204.0 to 54,320.3)	176,000 (157,000 to 204,000)	42,357.4 (37,655.8 to 48,971.9)	159,000 (137,000 to 182,000)	37,512.3 (32,422.6 to 43,160.4)	161,000 (137,000 to 185,000)	37,441.7 (31,967.3 to 43,147.4)	-10.9 (-21.1 to 0.3)	-11.4 (-20.4 to -2.0)
**Rwanda**	All Ages	870,000 (797,000 to 939,000)	12,101.3 (11,084.2 to 13,055.1)	682,000 (640,000 to 726,000)	5,354.2 (5,031.3 to 5,702.9)	620,000 (574,000 to 660,000)	4,765.7 (4,417.7 to 5,078.4)	631,000 (589,000 to 673,000)	4,755.6 (4,437.3 to 5,070.2)	-55.8 (-58.4 to -52.7)	-11.0 (-16.2 to -6.0)
	Under 5	340,000 (293,000 to 402,000)	25,254.9 (21,735.1 to 29,791.6)	133,000 (115,000 to 153,000)	7,695.5 (6,664.5 to 8,853.6)	110,000 (95,300 to 127,000)	6,345.6 (5,481.8 to 7,321.5)	108,000 (91,600 to 126,000)	6,160.3 (5,239.0 to 7,194.0)	-69.5 (-72.6 to -66.5)	-17.5 (-24.7 to -9.3)
	5-14 years	145,000 (116,000 to 179,000)	7,073.1 (5,695.0 to 8,763.5)	90,300 (74,700 to 107,000)	2,852.3 (2,361.5 to 3,392.7)	84,900 (69,400 to 101,000)	2,657.0 (2,173.2 to 3,175.6)	83,600 (68,200 to 99,700)	2,595.3 (2,117.5 to 3,093.8)	-59.7 (-64.0 to -54.7)	-6.8 (-15.6 to 3.6)
	15-49 years	201,000 (180,000 to 226,000)	6,358.2 (5,688.1 to 7,146.4)	214,000 (193,000 to 237,000)	3,314.4 (2,991.0 to 3,668.9)	203,000 (182,000 to 224,000)	3,043.1 (2,735.4 to 3,359.4)	210,000 (188,000 to 233,000)	3,057.9 (2,743.5 to 3,393.8)	-47.9 (-52.7 to -43.1)	-8.2 (-15.7 to 0.9)
	50-69 years	124,000 (109,000 to 142,000)	23,419.4 (20,512.2 to 26,738.1)	155,000 (136,000 to 174,000)	13,515.4 (11,915.4 to 15,190.5)	141,000 (125,000 to 157,000)	12,028.7 (10,616.9 to 13,306.6)	147,000 (129,000 to 165,000)	12,215.4 (10,703.0 to 13,699.3)	-42.3 (-48.6 to -35.1)	-11.0 (-19.2 to -1.6)
	70+ years	60,100 (52,000 to 68,900)	54,463.2 (47,063.2 to 62,408.4)	89,300 (78,300 to 103,000)	39,411.1 (34,547.4 to 45,416.6)	80,300 (69,200 to 93,300)	34,054.9 (29,312.9 to 39,552.0)	83,500 (73,500 to 95,000)	33,789.1 (29,763.0 to 38,430.3)	-27.6 (-36.3 to -17.2)	-13.6 (-22.9 to -4.1)
**Somalia**	All Ages	776,000 (713,000 to 847,000)	9,779.6 (8,980.2 to 10,664.3)	1,400,000 (1,310,000 to 1,500,000)	6,986.3 (6,545.1 to 7,487.0)	1,360,000 (1,260,000 to 1,470,000)	6,549.7 (6,040.9 to 7,066.6)	1,410,000 (1,310,000 to 1,520,000)	6,540.3 (6,061.1 to 7,045.7)	-28.6 (-32.0 to -24.7)	-6.2 (-10.8 to -1.7)
	Under 5	339,000 (294,000 to 394,000)	21,943.9 (19,048.4 to 25,503.8)	449,000 (389,000 to 514,000)	11,640.4 (10,105.1 to 13,328.9)	423,000 (367,000 to 485,000)	10,597.3 (9,192.5 to 12,153.2)	440,000 (378,000 to 505,000)	10,657.9 (9,156.5 to 12,237.7)	-47.0 (-50.8 to -42.6)	-9.0 (-15.3 to -2.1)
	5-14 years	115,000 (91,300 to 142,000)	4,889.9 (3,886.1 to 6,023.2)	223,000 (180,000 to 269,000)	3,867.5 (3,114.6 to 4,665.6)	229,000 (180,000 to 279,000)	3,822.1 (3,010.8 to 4,672.9)	233,000 (185,000 to 282,000)	3,756.0 (2,977.8 to 4,545.9)	-20.9 (-30.2 to -7.9)	-1.2 (-11.8 to 11.9)
	15-49 years	192,000 (170,000 to 216,000)	5,492.5 (4,876.0 to 6,186.3)	429,000 (388,000 to 473,000)	4,711.2 (4,255.2 to 5,184.9)	435,000 (391,000 to 484,000)	4,555.9 (4,101.7 to 5,075.2)	455,000 (408,000 to 512,000)	4,564.2 (4,091.9 to 5,134.9)	-14.2 (-21.1 to -6.0)	-3.3 (-11.7 to 5.8)

Location	Age Group	1990		2019		2020		2021		Percent Incidence Rate Change from 1990-2019 (95% UI)	Percent Incidence Rate Change from 2019-2021 (95% UI)
		Episodes (95% UI)	Rate (95% UI)	Episodes (95% UI)	Rate (95% UI)	Episodes (95% UI)	Rate (95% UI)	Episodes (95% UI)	Rate (95% UI)		
	50-69 years	94,600 (82,800 to 106,000)	19,735.8 (17,282.8 to 22,127.3)	213,000 (191,000 to 242,000)	19,447.4 (17,419.8 to 22,089.9)	198,000 (174,000 to 223,000)	17,826.7 (15,721.1 to 20,069.5)	205,000 (178,000 to 231,000)	18,229.8 (15,810.1 to 20,519.2)	-1.5 (-11.3 to 9.4)	-8.3 (-16.1 to 1.6)
	70+ years	35,900 (31,200 to 40,500)	51,189.8 (44,459.7 to 57,691.5)	84,000 (72,600 to 97,400)	46,477.8 (40,134.7 to 53,897.3)	79,000 (69,200 to 91,400)	42,040.1 (36,823.8 to 48,649.0)	80,600 (69,900 to 93,900)	42,299.2 (36,668.3 to 49,270.5)	-9.2 (-18.9 to 1.9)	-9.5 (-19.4 to 2.1)
**South Sudan**	All Ages	533,000 (495,000 to 576,000)	9,067.8 (8,424.2 to 9,792.8)	564,000 (529,000 to 606,000)	6,197.7 (5,805.4 to 6,655.2)	544,000 (508,000 to 583,000)	5,784.5 (5,394.1 to 6,188.5)	556,000 (520,000 to 592,000)	5,751.1 (5,377.8 to 6,117.4)	-31.7 (-34.7 to -27.9)	-6.7 (-11.5 to -1.8)
	Under 5	215,000 (187,000 to 249,000)	21,152.5 (18,349.2 to 24,511.7)	159,000 (139,000 to 183,000)	11,047.6 (9,655.0 to 12,686.8)	152,000 (134,000 to 175,000)	10,081.7 (8,930.8 to 11,611.8)	157,000 (137,000 to 180,000)	10,066.6 (8,798.3 to 11,551.1)	-47.8 (-51.6 to -43.1)	-8.7 (-15.4 to -1.7)
	5-14 years	68,300 (56,000 to 84,700)	4,252.8 (3,487.9 to 5,271.4)	79,400 (64,400 to 96,600)	2,956.0 (2,396.8 to 3,592.3)	78,800 (64,500 to 96,500)	2,899.1 (2,373.8 to 3,549.4)	77,700 (63,000 to 94,800)	2,844.6 (2,304.3 to 3,467.7)	-30.5 (-37.5 to -21.4)	-1.9 (-12.3 to 9.1)
	15-49 years	112,000 (98,700 to 126,000)	4,147.3 (3,657.0 to 4,658.7)	151,000 (135,000 to 167,000)	3,676.6 (3,305.3 to 4,069.9)	150,000 (135,000 to 167,000)	3,491.2 (3,143.7 to 3,887.7)	154,000 (138,000 to 171,000)	3,453.0 (3,098.8 to 3,851.4)	-11.3 (-19.1 to -1.4)	-5.0 (-12.5 to 1.7)
	50-69 years	78,000 (68,600 to 88,100)	18,246.0 (16,036.2 to 20,593.9)	109,000 (97,700 to 121,000)	14,762.0 (13,241.1 to 16,426.8)	105,000 (90,900 to 116,000)	13,683.2 (11,885.8 to 15,236.9)	109,000 (96,200 to 122,000)	13,833.4 (12,187.8 to 15,463.2)	-19.1 (-27.5 to -9.2)	-7.3 (-16.7 to 2.0)
	70+ years	59,300 (51,700 to 67,900)	47,227.2 (41,174.1 to 53,995.9)	66,000 (58,100 to 75,300)	46,604.0 (41,039.7 to 53,185.3)	59,900 (51,900 to 69,300)	42,362.9 (36,716.4 to 48,982.6)	58,400 (51,100 to 68,900)	41,971.1 (36,733.7 to 49,472.1)	-1.3 (-14.2 to 11.5)	-9.1 (-18.2 to 0.4)
**Uganda**	All Ages	1,510,000 (1,390,000 to 1,630,000)	8,707.2 (8,037.6 to 9,449.4)	2,050,000 (1,900,000 to 2,200,000)	4,958.3 (4,602.0 to 5,326.5)	1,940,000 (1,770,000 to 2,120,000)	4,577.7 (4,189.6 to 5,003.0)	2,010,000 (1,850,000 to 2,220,000)	4,644.5 (4,271.2 to 5,128.7)	-43.1 (-46.3 to -39.4)	-7.7 (-13.1 to -1.1)
	Under 5	630,000 (546,000 to 728,000)	17,529.9 (15,210.0 to 20,268.8)	483,000 (423,000 to 551,000)	6,690.0 (5,856.3 to 7,629.6)	418,000 (358,000 to 492,000)	5,743.5 (4,918.2 to 6,757.8)	432,000 (364,000 to 523,000)	5,898.0 (4,980.3 to 7,151.7)	-61.8 (-65.3 to -57.8)	-14.1 (-23.3 to -4.2)
	5-14 years	223,000 (181,000 to 273,000)	4,616.6 (3,740.4 to 5,646.1)	366,000 (301,000 to 443,000)	3,037.8 (2,499.0 to 3,671.7)	363,000 (296,000 to 434,000)	2,950.2 (2,410.5 to 3,529.3)	371,000 (297,000 to 444,000)	2,967.2 (2,376.3 to 3,550.1)	-34.2 (-42.5 to -24.0)	-2.9 (-12.9 to 7.0)
	15-49 years	298,000 (262,000 to 332,000)	4,005.5 (3,523.5 to 4,461.6)	587,000 (527,000 to 656,000)	3,115.5 (2,796.0 to 3,477.4)	580,000 (520,000 to 642,000)	2,980.1 (2,668.9 to 3,295.0)	604,000 (542,000 to 679,000)	3,004.7 (2,697.9 to 3,378.3)	-22.2 (-28.6 to -13.9)	-4.3 (-12.1 to 4.9)
	50-69 years	209,000 (183,000 to 238,000)	18,182.7 (15,913.7 to 20,744.3)	359,000 (323,000 to 400,000)	14,076.0 (12,638.5 to 15,671.2)	349,000 (312,000 to 387,000)	12,991.8 (11,611.9 to 14,432.1)	370,000 (327,000 to 415,000)	13,309.0 (11,790.0 to 14,942.6)	-22.6 (-30.3 to -13.1)	-7.7 (-16.7 to 1.5)
	70+ years	147,000 (129,000 to 169,000)	50,101.2 (44,037.6 to 57,853.1)	250,000 (220,000 to 285,000)	42,327.9 (37,259.5 to 48,096.2)	227,000 (194,000 to 262,000)	38,805.5 (33,149.2 to 44,782.8)	235,000 (205,000 to 274,000)	39,099.5 (34,057.5 to 45,521.7)	-15.5 (-23.9 to -5.0)	-8.3 (-17.8 to 1.5)

Location	Age Group	1990		2019		2020		2021		Percent Incidence Rate Change from 1990-2019 (95% UI)	Percent Incidence Rate Change from 2019-2021 (95% UI)
		Episodes (95% UI)	Rate (95% UI)	Episodes (95% UI)	Rate (95% UI)	Episodes (95% UI)	Rate (95% UI)	Episodes (95% UI)	Rate (95% UI)		
**United Republic of Tanzania**	All Ages	2,320,000 (2,160,000 to 2,480,000)	8,969.7 (8,349.3 to 9,616.1)	3,220,000 (3,020,000 to 3,440,000)	5,759.7 (5,398.4 to 6,156.5)	2,960,000 (2,760,000 to 3,180,000)	5,172.1 (4,834.7 to 5,553.3)	3,090,000 (2,880,000 to 3,310,000)	5,279.7 (4,925.2 to 5,667.6)	-35.8 (-39.3 to -32.1)	-10.2 (-15.0 to -5.5)
	Under 5	970,000 (850,000 to 1,110,000)	20,171.8 (17,685.1 to 23,050.9)	786,000 (688,000 to 900,000)	8,956.1 (7,833.5 to 10,250.6)	655,000 (571,000 to 759,000)	7,422.9 (6,472.8 to 8,607.5)	683,000 (593,000 to 796,000)	7,721.9 (6,699.5 to 8,997.6)	-55.6 (-58.9 to -51.5)	-17.1 (-25.0 to -9.2)
	5-14 years	346,000 (280,000 to 412,000)	4,754.9 (3,846.8 to 5,664.2)	490,000 (399,000 to 590,000)	3,264.2 (2,658.6 to 3,929.4)	479,000 (397,000 to 573,000)	3,130.3 (2,596.4 to 3,746.7)	485,000 (396,000 to 578,000)	3,117.0 (2,544.9 to 3,715.0)	-31.4 (-38.0 to -23.3)	-4.1 (-13.4 to 7.6)
	15-49 years	440,000 (396,000 to 486,000)	3,888.6 (3,504.6 to 4,300.9)	865,000 (774,000 to 957,000)	3,250.5 (2,909.8 to 3,596.1)	828,000 (751,000 to 914,000)	3,022.4 (2,739.9 to 3,336.5)	873,000 (798,000 to 966,000)	3,095.2 (2,830.2 to 3,424.5)	-16.4 (-23.2 to -9.2)	-7.0 (-14.1 to 0.5)
	50-69 years	331,000 (292,000 to 367,000)	16,836.4 (14,856.8 to 18,666.8)	618,000 (550,000 to 682,000)	14,072.1 (12,518.6 to 15,537.5)	582,000 (519,000 to 657,000)	12,690.8 (11,320.9 to 14,330.1)	619,000 (548,000 to 684,000)	13,059.2 (11,572.0 to 14,448.3)	-16.4 (-23.3 to -7.3)	-9.8 (-18.8 to -1.6)
	70+ years	231,000 (202,000 to 266,000)	47,450.9 (41,511.0 to 54,597.2)	459,000 (408,000 to 525,000)	42,369.5 (37,690.5 to 48,441.9)	414,000 (366,000 to 470,000)	38,090.5 (33,664.2 to 43,201.7)	427,000 (378,000 to 498,000)	38,404.6 (34,019.0 to 44,838.9)	-10.7 (-19.2 to -1.1)	-10.1 (-17.9 to -0.8)
**Zambia**	All Ages	685,000 (626,000 to 743,000)	8,632.7 (7,892.5 to 9,358.8)	801,000 (751,000 to 855,000)	4,329.4 (4,056.5 to 4,617.6)	758,000 (703,000 to 822,000)	3,983.0 (3,696.8 to 4,322.0)	770,000 (715,000 to 836,000)	3,943.5 (3,662.9 to 4,282.5)	-49.8 (-52.9 to -46.7)	-8.0 (-14.3 to -1.1)
	Under 5	309,000 (268,000 to 358,000)	20,458.6 (17,729.9 to 23,749.8)	183,000 (159,000 to 212,000)	6,220.6 (5,408.5 to 7,212.5)	157,000 (135,000 to 186,000)	5,354.5 (4,587.7 to 6,316.6)	156,000 (132,000 to 183,000)	5,318.8 (4,499.8 to 6,239.7)	-69.6 (-72.5 to -66.2)	-13.9 (-23.6 to -2.2)
	5-14 years	99,100 (80,700 to 120,000)	4,414.5 (3,593.6 to 5,327.2)	126,000 (102,000 to 152,000)	2,468.2 (2,002.4 to 2,971.4)	126,000 (102,000 to 152,000)	2,414.8 (1,944.8 to 2,914.7)	125,000 (101,000 to 150,000)	2,338.7 (1,881.8 to 2,814.9)	-44.1 (-50.7 to -36.7)	-2.2 (-13.4 to 12.3)
	15-49 years	139,000 (126,000 to 154,000)	3,934.2 (3,552.5 to 4,353.3)	255,000 (229,000 to 279,000)	2,833.9 (2,547.7 to 3,102.8)	251,000 (224,000 to 279,000)	2,686.8 (2,404.0 to 2,988.4)	259,000 (233,000 to 288,000)	2,683.6 (2,410.1 to 2,983.1)	-28.0 (-34.1 to -21.6)	-5.2 (-13.4 to 4.3)
	50-69 years	85,200 (75,000 to 97,100)	15,979.5 (14,067.8 to 18,212.3)	145,000 (129,000 to 161,000)	12,131.9 (10,801.0 to 13,484.9)	139,000 (123,000 to 156,000)	11,065.4 (9,738.8 to 12,387.7)	146,000 (130,000 to 165,000)	11,152.8 (9,879.8 to 12,553.4)	-24.1 (-31.5 to -15.9)	-8.8 (-18.4 to 1.4)
	70+ years	53,100 (45,600 to 60,700)	45,809.8 (39,334.1 to 52,362.9)	92,200 (81,700 to 107,000)	34,463.4 (30,503.9 to 40,033.1)	84,300 (73,800 to 98,000)	31,204.9 (27,297.6 to 36,248.6)	83,200 (71,200 to 96,000)	30,931.7 (26,463.2 to 35,658.0)	-24.8 (-33.0 to -16.2)	-9.5 (-19.6 to 2.3)
**Botswana**	All Ages	107,000 (99,700 to 115,000)	8,125.9 (7,554.3 to 8,727.9)	152,000 (142,000 to 164,000)	6,546.8 (6,115.6 to 7,059.8)	135,000 (125,000 to 145,000)	5,715.3 (5,281.9 to 6,145.8)	138,000 (128,000 to 149,000)	5,775.5 (5,364.3 to 6,221.8)	-19.4 (-24.4 to -14.6)	-12.7 (-18.1 to -7.4)
	Under 5	30,200 (26,300 to 34,800)	14,185.4 (12,351.4 to 16,336.7)	24,400 (20,900 to 28,300)	10,318.0 (8,863.3 to 11,999.1)	18,400 (15,800 to 21,300)	7,808.8 (6,688.8 to 9,037.6)	18,400 (15,800 to 21,500)	7,798.3 (6,687.9 to 9,144.9)	-27.3 (-33.2 to -19.5)	-24.3 (-31.3 to -17.0)
	5-14 years	17,200 (13,900 to 20,900)	4,560.6 (3,686.3 to 5,538.9)	14,800 (12,200 to 18,000)	3,227.8 (2,656.1 to 3,919.7)	13,200 (10,800 to 16,200)	2,855.6 (2,343.6 to 3,518.4)	13,100 (10,500 to 16,300)	2,833.2 (2,273.0 to 3,525.3)	-29.2 (-38.3 to -18.9)	-11.5 (-21.7 to -1.2)

Location	Age Group	1990		2019		2020		2021		Percent Incidence Rate Change from 1990-2019 (95% UI)	Percent Incidence Rate Change from 2019-2021 (95% UI)
		Episodes (95% UI)	Rate (95% UI)	Episodes (95% UI)	Rate (95% UI)	Episodes (95% UI)	Rate (95% UI)	Episodes (95% UI)	Rate (95% UI)		
	15-49 years	33,800 (30,000 to 38,100)	5,613.6 (4,974.9 to 6,328.4)	58,600 (52,700 to 66,400)	4,447.9 (3,996.0 to 5,037.0)	53,800 (47,600 to 60,400)	4,017.1 (3,554.2 to 4,507.7)	55,000 (48,700 to 61,900)	4,045.9 (3,579.8 to 4,554.8)	-20.8 (-30.4 to -10.0)	-9.7 (-17.6 to -0.7)
	50-69 years	17,100 (15,000 to 19,300)	16,559.9 (14,508.8 to 18,758.4)	36,000 (31,800 to 41,000)	13,949.0 (12,311.2 to 15,866.4)	33,400 (29,100 to 37,900)	12,400.3 (10,831.9 to 14,068.6)	35,100 (31,100 to 39,300)	12,693.3 (11,237.3 to 14,203.6)	-15.8 (-25.6 to -5.6)	-11.1 (-20.0 to -0.4)
	70+ years	8,860 (7,580 to 10,100)	38,566.2 (33,008.0 to 44,184.0)	18,600 (16,400 to 21,000)	33,018.7 (29,127.5 to 37,267.5)	16,300 (14,300 to 18,600)	28,422.5 (24,922.0 to 32,380.7)	16,600 (14,800 to 18,900)	28,355.0 (25,291.0 to 32,318.8)	-14.4 (-24.3 to -2.3)	-13.9 (-21.8 to -4.3)
**Eswatini**	All Ages	55,400 (50,900 to 60,000)	6,870.8 (6,307.6 to 7,442.4)	61,500 (57,000 to 65,900)	5,382.2 (4,991.8 to 5,768.3)	53,500 (49,700 to 57,200)	4,651.2 (4,321.3 to 4,975.6)	53,900 (50,000 to 58,100)	4,661.8 (4,326.0 to 5,032.5)	-21.7 (-26.5 to -16.3)	-13.6 (-18.1 to -8.2)
	Under 5	21,000 (18,000 to 24,500)	14,412.3 (12,356.8 to 16,876.5)	12,800 (11,200 to 14,800)	8,872.5 (7,744.3 to 10,281.2)	9,280 (8,070 to 10,700)	6,516.2 (5,666.5 to 7,514.3)	9,260 (8,060 to 10,700)	6,592.8 (5,743.3 to 7,610.4)	-38.4 (-43.8 to -32.5)	-26.6 (-32.3 to -20.7)
	5-14 years	9,230 (7,360 to 11,200)	3,841.3 (3,064.0 to 4,656.1)	7,930 (6,430 to 9,800)	2,914.1 (2,361.2 to 3,602.0)	6,950 (5,610 to 8,370)	2,553.8 (2,060.0 to 3,075.1)	6,890 (5,660 to 8,440)	2,529.5 (2,079.7 to 3,100.9)	-24.1 (-33.5 to -13.7)	-12.4 (-21.6 to -2.9)
	15-49 years	14,800 (13,300 to 16,600)	4,150.8 (3,722.2 to 4,663.1)	22,200 (19,600 to 25,000)	3,679.7 (3,258.1 to 4,143.9)	20,500 (18,200 to 22,800)	3,355.0 (2,992.2 to 3,735.6)	20,700 (18,300 to 23,400)	3,358.2 (2,961.4 to 3,789.2)	-11.3 (-18.7 to -2.3)	-8.8 (-15.9 to 1.2)
	50-69 years	6,780 (5,950 to 7,810)	12,876.1 (11,284.0 to 14,819.6)	12,600 (11,300 to 14,300)	12,602.1 (11,228.4 to 14,256.8)	11,500 (10,100 to 13,000)	11,258.7 (9,917.1 to 12,685.8)	11,800 (10,500 to 13,500)	11,438.8 (10,131.0 to 13,012.2)	-2.1 (-13.7 to 11.8)	-10.7 (-20.9 to -0.8)
	70+ years	3,650 (3,170 to 4,200)	30,860.3 (26,790.6 to 35,495.5)	5,900 (5,170 to 6,770)	26,103.3 (22,901.7 to 29,975.4)	5,260 (4,630 to 6,040)	22,896.3 (20,141.8 to 26,286.8)	5,170 (4,440 to 6,040)	22,986.8 (19,769.9 to 26,882.4)	-15.4 (-26.4 to -5.4)	-12.3 (-21.6 to -0.7)
**Lesotho**	All Ages	110,000 (102,000 to 119,000)	7,185.3 (6,631.2 to 7,737.7)	125,000 (117,000 to 134,000)	6,689.1 (6,244.3 to 7,164.0)	109,000 (102,000 to 117,000)	5,840.3 (5,427.6 to 6,243.2)	110,000 (102,000 to 119,000)	5,860.6 (5,430.2 to 6,328.2)	-6.9 (-12.5 to -1.1)	-12.7 (-17.3 to -7.8)
	Under 5	32,800 (28,200 to 38,800)	13,316.5 (11,441.5 to 15,765.1)	20,600 (18,100 to 23,800)	9,830.0 (8,607.9 to 11,350.4)	15,300 (13,300 to 17,600)	7,393.4 (6,456.1 to 8,525.1)	15,200 (13,100 to 17,500)	7,443.0 (6,451.4 to 8,608.7)	-26.2 (-32.5 to -19.7)	-24.8 (-30.6 to -17.7)
	5-14 years	17,800 (14,400 to 21,900)	4,090.9 (3,308.7 to 5,013.4)	14,500 (12,000 to 17,700)	3,352.9 (2,773.9 to 4,097.2)	12,700 (10,300 to 15,200)	2,944.3 (2,392.9 to 3,539.4)	12,500 (10,100 to 15,100)	2,938.1 (2,372.7 to 3,549.1)	-18.0 (-27.6 to -6.3)	-12.2 (-21.3 to -2.3)
	15-49 years	27,900 (24,800 to 31,000)	4,208.8 (3,742.1 to 4,673.5)	43,600 (39,200 to 48,600)	4,439.2 (3,994.5 to 4,948.3)	40,000 (36,000 to 44,400)	4,030.8 (3,621.7 to 4,466.7)	40,900 (36,800 to 45,900)	4,076.8 (3,665.7 to 4,568.2)	5.5 (-4.8 to 16.8)	-9.2 (-16.6 to -0.5)
	50-69 years	17,900 (15,900 to 20,000)	12,457.9 (11,082.3 to 13,937.6)	31,300 (28,000 to 35,300)	15,914.9 (14,220.8 to 17,938.0)	28,000 (24,500 to 31,900)	14,355.2 (12,555.0 to 16,382.4)	27,800 (24,400 to 31,800)	14,510.2 (12,756.6 to 16,597.7)	27.7 (15.1 to 41.7)	-9.8 (-19.0 to 0.1)

Location	Age Group	1990		2019		2020		2021		Percent Incidence Rate Change from 1990-2019 (95% UI)	Percent Incidence Rate Change from 2019-2021 (95% UI)
		Episodes (95% UI)	Rate (95% UI)	Episodes (95% UI)	Rate (95% UI)	Episodes (95% UI)	Rate (95% UI)	Episodes (95% UI)	Rate (95% UI)		
	70+ years	13,700 (11,900 to 15,700)	31,823.6 (27,681.4 to 36,571.8)	15,000 (13,200 to 17,200)	31,162.1 (27,311.8 to 35,658.4)	13,500 (11,800 to 15,500)	27,535.6 (24,024.0 to 31,623.3)	13,400 (11,700 to 15,500)	27,911.7 (24,434.1 to 32,137.8)	-2.1 (-13.6 to 11.6)	-11.6 (-22.7 to 0.1)
**Namibia**	All Ages	114,000 (105,000 to 123,000)	8,102.6 (7,513.8 to 8,725.9)	136,000 (127,000 to 146,000)	5,718.0 (5,346.6 to 6,133.0)	123,000 (114,000 to 130,000)	5,092.1 (4,753.8 to 5,400.1)	127,000 (118,000 to 137,000)	5,231.0 (4,864.4 to 5,616.6)	-29.4 (-33.6 to -24.9)	-10.9 (-15.6 to -5.8)
	Under 5	33,700 (28,900 to 39,400)	14,885.6 (12,752.3 to 17,414.0)	21,000 (18,400 to 24,600)	7,455.0 (6,522.9 to 8,722.6)	16,100 (13,900 to 18,800)	5,762.4 (4,953.9 to 6,696.6)	16,400 (14,200 to 19,200)	5,905.5 (5,085.8 to 6,880.7)	-49.9 (-54.5 to -45.0)	-22.7 (-28.6 to -17.1)
	5-14 years	16,100 (12,900 to 19,700)	4,308.2 (3,439.7 to 5,252.1)	14,800 (12,100 to 17,500)	2,735.1 (2,239.6 to 3,236.8)	13,500 (10,900 to 16,500)	2,477.1 (1,996.2 to 3,026.6)	13,600 (11,100 to 16,300)	2,490.6 (2,021.5 to 2,984.6)	-36.5 (-43.5 to -28.3)	-9.4 (-17.9 to 0.4)
	15-49 years	35,100 (31,200 to 38,800)	5,336.3 (4,732.9 to 5,890.8)	48,100 (43,100 to 54,300)	3,831.0 (3,434.5 to 4,327.7)	44,800 (40,300 to 49,900)	3,522.1 (3,164.8 to 3,924.5)	46,400 (41,800 to 51,700)	3,592.4 (3,241.9 to 4,003.5)	-28.2 (-34.7 to -20.1)	-8.1 (-15.8 to 0.6)
	50-69 years	19,200 (16,900 to 22,000)	16,230.7 (14,244.0 to 18,596.2)	33,100 (29,500 to 37,700)	13,744.4 (12,253.8 to 15,652.7)	31,000 (27,600 to 34,600)	12,460.5 (11,100.9 to 13,915.7)	33,100 (29,200 to 37,400)	12,986.4 (11,442.9 to 14,643.0)	-15.3 (-23.6 to -4.3)	-9.3 (-19.0 to 0.8)
	70+ years	9,600 (8,290 to 11,000)	36,141.5 (31,216.8 to 41,297.8)	18,900 (16,700 to 21,900)	32,478.5 (28,612.7 to 37,488.8)	17,000 (15,000 to 19,500)	28,681.0 (25,238.1 to 32,820.7)	17,600 (15,200 to 20,300)	29,398.1 (25,337.3 to 33,901.5)	-10.1 (-20.9 to 3.6)	-11.7 (-20.8 to -0.9)
**South Africa**	All Ages	3,330,000 (3,140,000 to 3,530,000)	9,001.3 (8,481.9 to 9,548.2)	4,920,000 (4,630,000 to 5,210,000)	8,785.6 (8,272.2 to 9,307.2)	4,390,000 (4,120,000 to 4,660,000)	7,762.9 (7,287.2 to 8,251.0)	4,480,000 (4,190,000 to 4,760,000)	7,888.0 (7,368.4 to 8,379.1)	-2.4 (-7.1 to 2.4)	-11.6 (-14.3 to -8.9)
	Under 5	748,000 (657,000 to 849,000)	15,328.7 (13,474.0 to 17,403.5)	456,000 (405,000 to 517,000)	8,910.3 (7,905.3 to 10,100.2)	335,000 (298,000 to 382,000)	6,648.4 (5,901.0 to 7,571.7)	334,000 (293,000 to 382,000)	6,722.7 (5,904.8 to 7,694.0)	-41.9 (-44.4 to -39.0)	-25.4 (-28.3 to -21.7)
	5-14 years	465,000 (390,000 to 555,000)	5,326.7 (4,469.8 to 6,351.1)	317,000 (262,000 to 377,000)	3,111.0 (2,579.2 to 3,708.2)	281,000 (234,000 to 335,000)	2,747.3 (2,287.5 to 3,279.3)	280,000 (229,000 to 338,000)	2,734.8 (2,235.5 to 3,295.8)	-41.6 (-45.7 to -37.5)	-11.7 (-15.7 to -7.7)
	15-49 years	1,340,000 (1,230,000 to 1,460,000)	7,094.8 (6,516.0 to 7,737.5)	1,930,000 (1,760,000 to 2,110,000)	6,298.3 (5,733.3 to 6,896.0)	1,780,000 (1,620,000 to 1,940,000)	5,765.5 (5,248.8 to 6,280.1)	1,820,000 (1,670,000 to 2,000,000)	5,856.8 (5,353.0 to 6,419.9)	-11.2 (-17.2 to -5.5)	-8.5 (-11.9 to -4.6)
	50-69 years	422,000 (379,000 to 469,000)	11,988.9 (10,783.4 to 13,330.5)	1,300,000 (1,180,000 to 1,430,000)	16,340.3 (14,835.0 to 17,910.7)	1,210,000 (1,090,000 to 1,330,000)	14,789.5 (13,356.4 to 16,371.0)	1,250,000 (1,130,000 to 1,370,000)	15,037.3 (13,615.4 to 16,544.9)	36.3 (28.2 to 44.2)	-9.5 (-13.4 to -5.2)
	70+ years	358,000 (319,000 to 401,000)	35,306.3 (31,464.3 to 39,482.3)	919,000 (825,000 to 1,030,000)	42,823.8 (38,448.5 to 48,135.6)	783,000 (706,000 to 883,000)	35,780.6 (32,253.6 to 40,331.4)	801,000 (714,000 to 903,000)	36,196.5 (32,275.9 to 40,827.9)	21.3 (11.6 to 32.7)	-16.4 (-20.2 to -12.1)
**Zimbabwe**	All Ages	627,000 (583,000 to 671,000)	6,058.6 (5,640.0 to 6,491.2)	907,000 (852,000 to 962,000)	5,992.8 (5,626.4 to 6,354.4)	795,000 (743,000 to 846,000)	5,171.4 (4,833.9 to 5,500.8)	811,000 (752,000 to 867,000)	5,200.0 (4,824.1 to 5,561.6)	-1.1 (-7.1 to 4.6)	-13.7 (-17.9 to -9.6)

Location	Age Group	1990		2019		2020		2021		Percent Incidence Rate Change from 1990-2019 (95% UI)	Percent Incidence Rate Change from 2019-2021 (95% UI)
		Episodes (95% UI)	Rate (95% UI)	Episodes (95% UI)	Rate (95% UI)	Episodes (95% UI)	Rate (95% UI)	Episodes (95% UI)	Rate (95% UI)		
	Under 5	211,000 (183,000 to 243,000)	11,947.4 (10,358.6 to 13,779.3)	206,000 (181,000 to 238,000)	9,308.0 (8,177.0 to 10,758.5)	158,000 (138,000 to 182,000)	7,149.5 (6,232.7 to 8,208.6)	158,000 (138,000 to 183,000)	7,146.2 (6,263.1 to 8,261.6)	-22.1 (-27.8 to -16.6)	-23.2 (-30.2 to -16.6)
	5-14 years	88,200 (71,700 to 109,000)	2,889.6 (2,348.6 to 3,568.5)	105,000 (85,600 to 128,000)	2,648.8 (2,159.3 to 3,234.1)	93,600 (76,900 to 115,000)	2,325.4 (1,910.0 to 2,851.0)	94,700 (76,900 to 115,000)	2,317.6 (1,882.3 to 2,811.2)	-8.3 (-18.4 to 4.3)	-12.2 (-19.8 to -2.5)
	15-49 years	164,000 (147,000 to 182,000)	3,552.9 (3,194.1 to 3,956.9)	294,000 (265,000 to 328,000)	3,939.9 (3,546.5 to 4,390.6)	269,000 (243,000 to 301,000)	3,541.2 (3,187.7 to 3,950.0)	276,000 (248,000 to 307,000)	3,561.7 (3,206.9 to 3,967.1)	10.9 (-0.4 to 23.2)	-10.1 (-18.9 to -2.4)
	50-69 years	104,000 (92,900 to 117,000)	14,078.4 (12,601.3 to 15,848.9)	209,000 (186,000 to 235,000)	16,987.0 (15,129.9 to 19,099.3)	193,000 (172,000 to 217,000)	15,263.4 (13,599.9 to 17,149.7)	200,000 (176,000 to 226,000)	15,528.7 (13,625.5 to 17,531.4)	20.7 (8.4 to 33.9)	-10.1 (-18.9 to 0.3)
	70+ years	60,100 (53,200 to 69,600)	33,538.7 (29,687.0 to 38,788.8)	92,900 (81,900 to 108,000)	35,512.9 (31,309.2 to 41,261.1)	80,700 (69,800 to 93,300)	30,542.4 (26,423.1 to 35,324.4)	82,100 (71,600 to 94,000)	30,930.1 (26,968.8 to 35,417.0)	5.9 (-4.5 to 19.2)	-14.0 (-23.5 to -4.7)
**Benin**	All Ages	362,000 (335,000 to 391,000)	7,464.1 (6,910.6 to 8,054.2)	697,000 (652,000 to 744,000)	5,494.7 (5,147.1 to 5,867.9)	670,000 (629,000 to 719,000)	5,120.7 (4,803.3 to 5,491.0)	666,000 (623,000 to 712,000)	4,932.5 (4,615.5 to 5,277.6)	-26.4 (-30.1 to -22.2)	-6.8 (-11.9 to -1.0)
	Under 5	156,000 (136,000 to 179,000)	15,803.9 (13,777.6 to 18,149.8)	187,000 (165,000 to 211,000)	8,327.1 (7,380.3 to 9,412.5)	171,000 (149,000 to 196,000)	7,455.9 (6,506.1 to 8,539.6)	167,000 (145,000 to 194,000)	7,127.7 (6,203.4 to 8,265.1)	-47.3 (-51.7 to -42.9)	-10.5 (-17.6 to -1.9)
	5-14 years	47,600 (38,600 to 58,700)	3,316.8 (2,692.5 to 4,088.1)	93,600 (75,300 to 114,000)	2,655.9 (2,135.1 to 3,235.4)	94,900 (76,800 to 115,000)	2,612.7 (2,114.9 to 3,162.5)	91,400 (73,600 to 112,000)	2,445.3 (1,969.4 to 2,983.3)	-19.9 (-28.9 to -9.7)	-1.6 (-10.8 to 10.2)
	15-49 years	66,900 (59,700 to 74,900)	3,338.2 (2,982.6 to 3,736.7)	192,000 (172,000 to 212,000)	3,292.1 (2,947.3 to 3,633.3)	190,000 (172,000 to 212,000)	3,151.0 (2,851.4 to 3,506.0)	192,000 (174,000 to 212,000)	3,076.4 (2,779.9 to 3,385.7)	-1.4 (-9.2 to 6.8)	-4.3 (-12.6 to 4.8)
	50-69 years	47,700 (42,300 to 53,600)	14,757.4 (13,083.9 to 16,600.8)	126,000 (110,000 to 142,000)	14,599.9 (12,781.9 to 16,431.0)	123,000 (110,000 to 136,000)	13,595.7 (12,062.2 to 15,001.1)	126,000 (111,000 to 141,000)	13,349.0 (11,763.6 to 14,907.8)	-1.1 (-10.4 to 9.5)	-6.9 (-15.5 to 3.1)
	70+ years	44,000 (38,700 to 50,600)	42,861.3 (37,703.7 to 49,323.8)	98,400 (86,700 to 115,000)	44,880.0 (39,538.9 to 52,296.8)	90,700 (79,600 to 105,000)	41,615.0 (36,538.5 to 47,990.3)	89,300 (78,100 to 102,000)	39,883.9 (34,885.3 to 45,712.2)	4.7 (-7.5 to 19.3)	-7.3 (-15.9 to 4.2)
**Burkina Faso**	All Ages	746,000 (695,000 to 804,000)	7,834.3 (7,294.1 to 8,439.7)	1,320,000 (1,230,000 to 1,410,000)	6,131.3 (5,707.7 to 6,523.0)	1,260,000 (1,170,000 to 1,350,000)	5,689.7 (5,276.2 to 6,091.5)	1,240,000 (1,150,000 to 1,330,000)	5,464.0 (5,068.8 to 5,860.9)	-21.7 (-25.8 to -17.5)	-7.2 (-12.5 to -0.8)
	Under 5	339,000 (300,000 to 381,000)	18,056.1 (15,968.2 to 20,328.8)	415,000 (366,000 to 467,000)	10,577.6 (9,335.6 to 11,904.2)	374,000 (324,000 to 430,000)	9,331.0 (8,083.4 to 10,728.5)	367,000 (319,000 to 421,000)	8,936.8 (7,759.1 to 10,257.6)	-41.4 (-45.9 to -35.9)	-11.8 (-20.3 to -2.2)
	5-14 years	84,100 (68,100 to 106,000)	2,957.6 (2,394.7 to 3,727.8)	167,000 (137,000 to 203,000)	2,789.1 (2,293.3 to 3,404.8)	168,000 (136,000 to 201,000)	2,740.0 (2,215.8 to 3,292.9)	161,000 (132,000 to 195,000)	2,568.3 (2,103.8 to 3,111.3)	-5.7 (-16.8 to 6.7)	-1.8 (-11.1 to 11.2)
	15-49 years	122,000 (109,000 to 136,000)	3,172.9 (2,851.5 to 3,540.0)	311,000 (278,000 to 349,000)	3,214.9 (2,869.2 to 3,598.1)	309,000 (278,000 to 345,000)	3,086.9 (2,778.6 to 3,453.8)	312,000 (282,000 to 347,000)	3,025.5 (2,734.5 to 3,368.5)	1.3 (-7.2 to 9.6)	-4.0 (-11.9 to 4.0)

Location	Age Group	1990		2019		2020		2021		Percent Incidence Rate Change from 1990-2019 (95% UI)	Percent Incidence Rate Change from 2019-2021 (95% UI)
		Episodes (95% UI)	Rate (95% UI)	Episodes (95% UI)	Rate (95% UI)	Episodes (95% UI)	Rate (95% UI)	Episodes (95% UI)	Rate (95% UI)		
	50-69 years	116,000 (104,000 to 130,000)	14,943.0 (13,321.8 to 16,662.0)	240,000 (212,000 to 265,000)	15,269.1 (13,502.4 to 16,898.5)	230,000 (204,000 to 254,000)	14,246.9 (12,647.6 to 15,738.2)	230,000 (202,000 to 255,000)	13,880.1 (12,174.4 to 15,395.7)	2.2 (-7.6 to 13.1)	-6.7 (-15.7 to 4.0)
	70+ years	85,500 (74,400 to 99,100)	43,553.9 (37,899.9 to 50,500.2)	190,000 (167,000 to 219,000)	46,413.3 (40,779.2 to 53,545.9)	180,000 (160,000 to 207,000)	43,105.6 (38,235.0 to 49,443.9)	174,000 (151,000 to 200,000)	41,222.7 (35,822.4 to 47,376.4)	6.6 (-4.9 to 23.0)	-7.1 (-16.9 to 4.1)
**Cabo Verde**	All Ages	24,500 (22,800 to 26,100)	6,936.8 (6,455.8 to 7,366.1)	32,500 (30,500 to 34,400)	5,878.4 (5,530.6 to 6,234.9)	31,000 (29,000 to 33,100)	5,582.8 (5,222.4 to 5,949.7)	30,900 (28,900 to 33,000)	5,523.9 (5,161.1 to 5,906.5)	-15.3 (-20.9 to -9.6)	-5.0 (-10.5 to 1.1)
	Under 5	7,210 (6,260 to 8,410)	12,115.6 (10,521.3 to 14,136.8)	2,010 (1,760 to 2,290)	4,316.8 (3,776.0 to 4,912.7)	1,740 (1,500 to 2,000)	3,843.0 (3,309.0 to 4,420.3)	1,620 (1,400 to 1,870)	3,675.7 (3,173.4 to 4,247.8)	-64.4 (-67.4 to -61.2)	-11.0 (-18.5 to -2.3)
	5-14 years	2,740 (2,190 to 3,390)	2,799.8 (2,235.4 to 3,466.1)	2,000 (1,590 to 2,430)	1,972.6 (1,569.0 to 2,392.4)	1,980 (1,590 to 2,450)	1,971.4 (1,582.4 to 2,443.5)	1,830 (1,460 to 2,270)	1,841.8 (1,471.1 to 2,288.5)	-29.5 (-36.5 to -21.9)	-0.1 (-8.5 to 9.2)
	15-49 years	4,920 (4,350 to 5,500)	3,340.0 (2,954.7 to 3,730.3)	9,310 (8,320 to 10,300)	3,027.0 (2,704.1 to 3,343.4)	9,070 (8,180 to 10,100)	2,918.3 (2,631.6 to 3,255.1)	9,120 (8,140 to 10,200)	2,906.0 (2,592.0 to 3,235.1)	-9.4 (-17.2 to 0.0)	-3.6 (-11.9 to 4.1)
	50-69 years	4,030 (3,550 to 4,470)	11,790.2 (10,394.1 to 13,087.9)	9,690 (8,720 to 10,800)	12,775.8 (11,504.6 to 14,292.3)	9,720 (8,750 to 10,700)	12,253.0 (11,036.2 to 13,512.6)	9,950 (8,920 to 11,100)	12,176.3 (10,919.6 to 13,618.2)	8.4 (-2.9 to 22.2)	-4.1 (-12.0 to 6.2)
	70+ years	5,640 (5,010 to 6,370)	37,739.7 (33,478.4 to 42,584.4)	9,450 (8,430 to 10,600)	45,594.3 (40,662.8 to 51,227.8)	8,530 (7,670 to 9,790)	42,419.9 (38,147.0 to 48,701.5)	8,380 (7,460 to 9,370)	40,897.7 (36,419.2 to 45,758.6)	20.8 (8.8 to 34.7)	-7.0 (-14.9 to 2.6)
**Cameroon**	All Ages	677,000 (634,000 to 727,000)	6,488.9 (6,072.9 to 6,963.8)	1,540,000 (1,440,000 to 1,640,000)	5,129.8 (4,800.4 to 5,459.8)	1,450,000 (1,350,000 to 1,560,000)	4,701.6 (4,377.5 to 5,035.8)	1,460,000 (1,350,000 to 1,580,000)	4,597.7 (4,262.5 to 4,958.6)	-20.9 (-25.2 to -17.1)	-8.3 (-13.2 to -2.2)
	Under 5	270,000 (237,000 to 305,000)	13,452.9 (11,788.8 to 15,192.9)	385,000 (340,000 to 441,000)	8,052.6 (7,116.8 to 9,223.9)	333,000 (290,000 to 383,000)	6,887.9 (6,013.2 to 7,939.9)	328,000 (283,000 to 374,000)	6,739.4 (5,819.8 to 7,682.3)	-40.1 (-44.2 to -35.1)	-14.5 (-21.9 to -4.9)
	5-14 years	82,600 (66,800 to 101,000)	2,871.3 (2,323.3 to 3,520.1)	207,000 (169,000 to 250,000)	2,545.3 (2,073.1 to 3,067.0)	207,000 (165,000 to 248,000)	2,465.2 (1,972.3 to 2,962.8)	201,000 (162,000 to 248,000)	2,341.9 (1,881.2 to 2,877.4)	-11.4 (-20.6 to -0.1)	-3.1 (-13.6 to 9.6)
	15-49 years	138,000 (125,000 to 153,000)	3,046.7 (2,754.6 to 3,376.6)	441,000 (397,000 to 494,000)	3,071.0 (2,765.9 to 3,440.2)	431,000 (386,000 to 478,000)	2,899.0 (2,591.4 to 3,215.0)	445,000 (396,000 to 495,000)	2,884.9 (2,569.4 to 3,208.0)	0.8 (-6.9 to 9.9)	-5.6 (-13.3 to 4.2)
	50-69 years	113,000 (99,700 to 126,000)	13,362.2 (11,825.5 to 14,973.4)	302,000 (268,000 to 337,000)	13,653.5 (12,106.6 to 15,266.6)	292,000 (257,000 to 323,000)	12,666.6 (11,176.5 to 14,011.6)	300,000 (265,000 to 334,000)	12,585.6 (11,113.2 to 14,012.5)	2.2 (-8.6 to 12.6)	-7.2 (-16.4 to 2.7)
	70+ years	74,200 (65,200 to 84,600)	40,299.4 (35,418.7 to 45,971.3)	203,000 (177,000 to 234,000)	41,988.5 (36,732.7 to 48,613.1)	190,000 (169,000 to 221,000)	38,584.6 (34,275.2 to 44,999.8)	187,000 (163,000 to 218,000)	37,291.6 (32,620.8 to 43,639.5)	4.2 (-7.4 to 16.0)	-8.1 (-17.9 to 3.3)

Location	Age Group	1990		2019		2020		2021		Percent Incidence Rate Change from 1990-2019 (95% UI)	Percent Incidence Rate Change from 2019-2021 (95% UI)
		Episodes (95% UI)	Rate (95% UI)	Episodes (95% UI)	Rate (95% UI)	Episodes (95% UI)	Rate (95% UI)	Episodes (95% UI)	Rate (95% UI)		
**Chad**	All Ages	534,000 (496,000 to 577,000)	8,868.4 (8,231.5 to 9,569.4)	1,190,000 (1,110,000 to 1,280,000)	7,240.2 (6,746.4 to 7,770.8)	1,180,000 (1,090,000 to 1,270,000)	6,907.8 (6,400.3 to 7,417.2)	1,170,000 (1,090,000 to 1,250,000)	6,602.2 (6,149.8 to 7,056.4)	-18.4 (-22.5 to -13.6)	-4.6 (-9.7 to 0.3)
	Under 5	244,000 (215,000 to 281,000)	19,665.7 (17,281.7 to 22,615.4)	458,000 (405,000 to 521,000)	13,518.2 (11,958.1 to 15,362.6)	441,000 (384,000 to 503,000)	12,562.6 (10,931.3 to 14,338.6)	432,000 (378,000 to 488,000)	11,889.7 (10,384.3 to 13,427.1)	-31.3 (-36.9 to -24.6)	-7.1 (-14.7 to 1.2)
	5-14 years	57,900 (46,600 to 70,600)	3,440.6 (2,767.6 to 4,193.3)	171,000 (140,000 to 206,000)	3,409.3 (2,794.5 to 4,106.1)	179,000 (146,000 to 215,000)	3,455.8 (2,807.7 to 4,135.6)	175,000 (139,000 to 220,000)	3,252.1 (2,577.9 to 4,098.6)	-0.9 (-12.6 to 15.3)	1.4 (-9.7 to 14.0)
	15-49 years	88,000 (78,500 to 97,700)	3,533.0 (3,151.2 to 3,919.9)	262,000 (236,000 to 290,000)	3,828.5 (3,450.7 to 4,239.8)	267,000 (241,000 to 296,000)	3,747.4 (3,379.8 to 4,152.6)	272,000 (248,000 to 299,000)	3,668.6 (3,339.0 to 4,035.0)	8.4 (-2.5 to 18.9)	-2.1 (-9.0 to 6.8)
	50-69 years	74,000 (64,900 to 82,800)	16,148.9 (14,161.9 to 18,069.7)	177,000 (157,000 to 198,000)	17,546.7 (15,570.0 to 19,656.2)	176,000 (158,000 to 200,000)	16,797.0 (15,087.7 to 19,056.9)	177,000 (159,000 to 200,000)	16,441.6 (14,723.4 to 18,487.3)	8.7 (-2.4 to 21.5)	-4.3 (-13.0 to 7.2)
	70+ years	70,100 (61,100 to 80,100)	46,532.3 (40,547.2 to 53,192.2)	126,000 (110,000 to 145,000)	52,890.2 (46,280.2 to 61,144.1)	118,000 (103,000 to 135,000)	50,349.4 (43,606.5 to 57,350.7)	115,000 (100,000 to 131,000)	48,296.9 (41,982.0 to 55,000.5)	13.7 (-0.2 to 28.9)	-4.8 (-13.8 to 5.7)
**Côte d'Ivoire**	All Ages	764,000 (711,000 to 824,000)	6,262.5 (5,825.1 to 6,756.9)	1,460,000 (1,370,000 to 1,560,000)	5,492.7 (5,133.0 to 5,874.6)	1,450,000 (1,340,000 to 1,560,000)	5,326.0 (4,932.3 to 5,742.6)	1,340,000 (1,260,000 to 1,430,000)	4,812.0 (4,507.5 to 5,118.2)	-12.3 (-16.7 to -7.2)	-3.0 (-8.8 to 2.4)
	Under 5	341,000 (303,000 to 387,000)	14,741.5 (13,093.6 to 16,756.9)	392,000 (345,000 to 447,000)	9,201.4 (8,105.2 to 10,483.7)	373,000 (326,000 to 426,000)	8,661.2 (7,560.8 to 9,889.4)	323,000 (284,000 to 369,000)	7,423.2 (6,513.9 to 8,469.5)	-37.6 (-42.3 to -32.9)	-5.9 (-14.2 to 1.9)
	5-14 years	99,900 (81,500 to 124,000)	2,945.7 (2,402.9 to 3,653.7)	191,000 (157,000 to 234,000)	2,784.9 (2,287.5 to 3,411.0)	198,000 (161,000 to 240,000)	2,804.3 (2,292.6 to 3,409.4)	180,000 (145,000 to 218,000)	2,490.8 (2,005.9 to 3,025.4)	-5.5 (-16.8 to 7.5)	0.7 (-10.3 to 11.8)
	15-49 years	165,000 (148,000 to 184,000)	2,960.5 (2,665.2 to 3,302.0)	416,000 (370,000 to 461,000)	3,181.8 (2,829.9 to 3,529.1)	420,000 (378,000 to 467,000)	3,137.5 (2,824.5 to 3,491.5)	401,000 (356,000 to 443,000)	2,927.8 (2,603.2 to 3,231.9)	7.5 (-1.2 to 16.8)	-1.4 (-9.5 to 8.1)
	50-69 years	106,000 (94,600 to 119,000)	13,325.7 (11,863.8 to 14,957.7)	287,000 (256,000 to 321,000)	14,168.5 (12,626.2 to 15,845.4)	285,000 (254,000 to 318,000)	13,568.0 (12,117.7 to 15,127.1)	277,000 (247,000 to 310,000)	12,707.8 (11,340.9 to 14,243.7)	6.3 (-4.1 to 19.2)	-4.2 (-14.1 to 5.6)
	70+ years	52,400 (45,800 to 59,200)	39,186.7 (34,227.9 to 44,275.9)	176,000 (156,000 to 204,000)	44,342.8 (39,414.3 to 51,389.4)	175,000 (153,000 to 198,000)	42,642.0 (37,288.1 to 48,047.5)	160,000 (140,000 to 180,000)	38,334.3 (33,680.1 to 43,219.3)	13.2 (0.1 to 26.7)	-3.8 (-13.7 to 6.8)
**Gambia**	All Ages	73,900 (68,000 to 80,000)	7,524.0 (6,931.9 to 8,148.1)	123,000 (115,000 to 130,000)	5,389.9 (5,054.3 to 5,710.8)	117,000 (109,000 to 125,000)	4,992.6 (4,667.8 to 5,350.5)	114,000 (106,000 to 122,000)	4,745.9 (4,446.2 to 5,080.9)	-28.4 (-32.9 to -23.1)	-7.4 (-12.6 to -1.5)
	Under 5	30,900 (26,900 to 35,400)	16,644.4 (14,471.7 to 19,067.1)	21,400 (18,900 to 24,300)	6,071.2 (5,352.9 to 6,906.1)	18,900 (16,300 to 21,700)	5,339.6 (4,604.2 to 6,110.0)	17,900 (15,600 to 20,800)	4,998.5 (4,358.2 to 5,828.4)	-63.5 (-66.5 to -60.0)	-12.0 (-19.9 to -3.5)
	5-14 years	9,630 (7,750 to 11,900)	3,493.5 (2,813.6 to 4,324.9)	14,500 (12,000 to 17,500)	2,373.9 (1,956.5 to 2,863.4)	14,600 (11,900 to 17,600)	2,331.5 (1,910.9 to 2,821.8)	13,600 (11,200 to 16,300)	2,143.5 (1,762.4 to 2,566.8)	-32.0 (-39.4 to -23.0)	-1.8 (-12.1 to 8.6)

Location	Age Group	1990		2019		2020		2021		Percent Incidence Rate Change from 1990-2019 (95% UI)	Percent Incidence Rate Change from 2019-2021 (95% UI)
		Episodes (95% UI)	Rate (95% UI)	Episodes (95% UI)	Rate (95% UI)	Episodes (95% UI)	Rate (95% UI)	Episodes (95% UI)	Rate (95% UI)		
	15-49 years	15,700 (13,900 to 17,500)	3,533.9 (3,132.6 to 3,944.2)	38,000 (34,600 to 41,700)	3,411.8 (3,110.6 to 3,751.6)	37,100 (33,100 to 41,500)	3,225.9 (2,878.1 to 3,608.9)	37,400 (33,700 to 41,500)	3,150.7 (2,841.9 to 3,501.4)	-3.5 (-13.4 to 5.8)	-5.4 (-13.5 to 3.0)
	50-69 years	10,200 (9,060 to 11,700)	16,499.0 (14,700.9 to 18,972.1)	26,400 (23,700 to 29,300)	16,567.0 (14,882.2 to 18,385.7)	25,100 (22,700 to 27,900)	15,268.6 (13,784.9 to 16,995.0)	25,200 (22,100 to 28,300)	14,832.5 (13,031.7 to 16,641.8)	0.4 (-10.7 to 13.7)	-7.8 (-16.9 to 1.9)
	70+ years	7,490 (6,500 to 8,530)	48,533.6 (42,155.6 to 55,294.6)	22,700 (20,100 to 25,800)	50,350.3 (44,557.8 to 57,383.8)	21,100 (18,700 to 24,500)	46,258.1 (41,044.3 to 53,733.8)	19,600 (17,100 to 22,300)	43,644.0 (38,209.2 to 49,707.5)	3.7 (-7.5 to 16.0)	-8.1 (-17.6 to 3.9)
**Ghana**	All Ages	1,020,000 (945,000 to 1,100,000)	6,803.8 (6,308.9 to 7,334.0)	1,880,000 (1,760,000 to 1,990,000)	5,756.6 (5,402.3 to 6,093.4)	1,830,000 (1,700,000 to 1,970,000)	5,470.1 (5,090.0 to 5,877.3)	1,830,000 (1,710,000 to 1,960,000)	5,351.8 (5,005.0 to 5,714.1)	-15.4 (-21.0 to -9.4)	-5.0 (-10.3 to 1.2)
	Under 5	378,000 (326,000 to 438,000)	14,350.0 (12,385.7 to 16,644.4)	304,000 (270,000 to 344,000)	6,656.2 (5,898.3 to 7,527.1)	269,000 (231,000 to 307,000)	5,840.4 (5,007.6 to 6,649.5)	262,000 (229,000 to 303,000)	5,655.5 (4,940.7 to 6,528.7)	-53.6 (-57.8 to -49.2)	-12.3 (-20.3 to -3.6)
	5-14 years	127,000 (102,000 to 156,000)	3,102.1 (2,489.0 to 3,817.8)	193,000 (157,000 to 236,000)	2,462.3 (2,003.0 to 3,003.3)	197,000 (161,000 to 236,000)	2,445.9 (2,000.9 to 2,927.6)	190,000 (152,000 to 228,000)	2,300.2 (1,844.8 to 2,764.6)	-20.6 (-28.7 to -10.8)	-0.7 (-11.1 to 11.1)
	15-49 years	233,000 (207,000 to 262,000)	3,409.0 (3,026.1 to 3,839.0)	631,000 (575,000 to 691,000)	3,798.8 (3,460.3 to 4,164.6)	629,000 (564,000 to 700,000)	3,686.9 (3,308.1 to 4,104.3)	643,000 (580,000 to 709,000)	3,667.8 (3,309.9 to 4,045.0)	11.4 (0.2 to 23.2)	-2.9 (-10.5 to 5.1)
	50-69 years	173,000 (154,000 to 197,000)	14,825.3 (13,221.8 to 16,894.9)	453,000 (401,000 to 507,000)	15,407.8 (13,642.2 to 17,233.5)	448,000 (400,000 to 502,000)	14,723.3 (13,139.6 to 16,496.3)	457,000 (406,000 to 513,000)	14,525.2 (12,914.5 to 16,291.1)	3.9 (-6.8 to 16.5)	-4.4 (-13.9 to 5.7)
	70+ years	108,000 (94,400 to 125,000)	42,745.9 (37,248.2 to 49,132.6)	296,000 (263,000 to 337,000)	44,898.6 (39,851.5 to 51,102.6)	286,000 (251,000 to 325,000)	42,052.1 (36,928.2 to 47,775.6)	281,000 (247,000 to 320,000)	40,783.8 (35,917.8 to 46,418.2)	5.0 (-7.5 to 18.4)	-6.3 (-16.1 to 3.4)
**Guinea**	All Ages	606,000 (563,000 to 652,000)	10,111.0 (9,389.4 to 10,880.1)	936,000 (876,000 to 993,000)	7,346.9 (6,873.1 to 7,798.0)	913,000 (858,000 to 971,000)	6,979.3 (6,556.7 to 7,424.2)	907,000 (846,000 to 965,000)	6,753.3 (6,296.3 to 7,182.3)	-27.3 (-31.8 to -23.4)	-5.0 (-10.1 to 0.2)
	Under 5	266,000 (233,000 to 306,000)	22,949.2 (20,102.8 to 26,357.5)	232,000 (203,000 to 266,000)	10,613.7 (9,270.7 to 12,176.4)	217,000 (191,000 to 246,000)	9,800.4 (8,614.8 to 11,096.6)	214,000 (187,000 to 243,000)	9,499.0 (8,307.1 to 10,794.2)	-53.8 (-57.7 to -49.6)	-7.7 (-16.0 to 0.5)
	5-14 years	63,100 (50,500 to 78,500)	3,964.1 (3,173.3 to 4,930.8)	127,000 (104,000 to 155,000)	3,526.7 (2,894.8 to 4,301.4)	131,000 (108,000 to 160,000)	3,537.5 (2,915.8 to 4,331.7)	127,000 (105,000 to 152,000)	3,340.1 (2,760.9 to 4,017.5)	-11.0 (-21.6 to 2.7)	0.3 (-9.7 to 11.9)
	15-49 years	101,000 (89,800 to 111,000)	3,999.3 (3,566.5 to 4,428.5)	247,000 (223,000 to 274,000)	4,298.4 (3,874.4 to 4,763.0)	248,000 (224,000 to 275,000)	4,183.5 (3,776.0 to 4,635.7)	253,000 (227,000 to 279,000)	4,126.2 (3,709.9 to 4,551.6)	7.5 (-1.3 to 17.0)	-2.7 (-11.4 to 5.5)
	50-69 years	95,200 (84,500 to 107,000)	17,040.4 (15,129.4 to 19,159.6)	181,000 (162,000 to 202,000)	18,986.9 (17,006.8 to 21,193.2)	174,000 (154,000 to 198,000)	17,921.3 (15,878.0 to 20,378.9)	177,000 (156,000 to 199,000)	17,849.6 (15,700.7 to 20,063.7)	11.4 (-0.5 to 24.1)	-5.6 (-16.0 to 3.4)

Location	Age Group	1990		2019		2020		2021		Percent Incidence Rate Change from 1990-2019 (95% UI)	Percent Incidence Rate Change from 2019-2021 (95% UI)
		Episodes (95% UI)	Rate (95% UI)	Episodes (95% UI)	Rate (95% UI)	Episodes (95% UI)	Rate (95% UI)	Episodes (95% UI)	Rate (95% UI)		
	70+ years	80,700 (70,800 to 91,100)	48,690.4 (42,727.0 to 54,963.7)	150,000 (131,000 to 172,000)	57,433.1 (50,477.9 to 65,976.1)	143,000 (125,000 to 161,000)	54,320.1 (47,470.1 to 61,448.1)	136,000 (119,000 to 156,000)	52,466.2 (45,900.1 to 60,048.5)	18.0 (5.0 to 31.4)	-5.4 (-14.7 to 4.5)
Guinea-Bissau	All Ages	83,000 (76,700 to 89,200)	8,238.6 (7,613.2 to 8,861.0)	110,000 (103,000 to 118,000)	5,580.7 (5,218.8 to 5,968.9)	106,000 (98,400 to 113,000)	5,253.0 (4,875.9 to 5,620.6)	105,000 (98,800 to 113,000)	5,107.2 (4,788.5 to 5,464.4)	-32.3 (-36.2 to -28.0)	-5.9 (-11.0 to -0.7)
	Under 5	34,600 (30,000 to 40,000)	18,400.0 (15,938.3 to 21,247.1)	24,200 (21,300 to 27,700)	7,369.9 (6,496.8 to 8,451.6)	21,700 (19,100 to 24,600)	6,575.2 (5,787.4 to 7,447.5)	21,100 (18,600 to 23,800)	6,350.0 (5,585.1 to 7,155.9)	-59.9 (-62.8 to -56.4)	-10.8 (-18.2 to -3.7)
	5-14 years	11,200 (9,050 to 13,800)	3,814.2 (3,075.4 to 4,687.6)	14,100 (11,300 to 17,100)	2,613.2 (2,099.3 to 3,165.3)	14,400 (11,600 to 17,300)	2,598.3 (2,094.1 to 3,135.8)	13,800 (11,300 to 16,500)	2,437.6 (1,998.3 to 2,918.3)	-31.5 (-38.9 to -21.7)	-0.6 (-11.3 to 10.4)
	15-49 years	17,300 (15,400 to 19,500)	3,970.7 (3,518.9 to 4,471.2)	36,900 (32,900 to 40,900)	3,895.3 (3,470.7 to 4,317.7)	36,800 (33,100 to 40,700)	3,777.0 (3,400.8 to 4,185.4)	37,100 (33,500 to 41,300)	3,712.8 (3,345.3 to 4,128.3)	-1.9 (-11.5 to 8.6)	-3.0 (-11.7 to 5.9)
	50-69 years	12,400 (11,100 to 13,800)	17,362.0 (15,486.7 to 19,376.3)	22,900 (20,000 to 25,600)	17,201.1 (15,018.9 to 19,274.8)	22,000 (19,500 to 24,800)	16,070.6 (14,224.3 to 18,090.9)	22,400 (20,000 to 25,000)	15,997.1 (14,253.2 to 17,800.2)	-0.9 (-11.9 to 10.6)	-6.6 (-15.9 to 4.8)
	70+ years	7,390 (6,440 to 8,430)	45,166.2 (39,399.3 to 51,563.2)	12,100 (10,400 to 14,000)	47,382.9 (40,979.3 to 54,998.4)	11,200 (9,760 to 12,900)	44,282.0 (38,731.4 to 51,288.5)	10,900 (9,690 to 12,400)	43,077.4 (38,166.6 to 48,979.7)	4.9 (-7.4 to 20.1)	-6.5 (-16.4 to 3.9)
Liberia	All Ages	225,000 (207,000 to 244,000)	9,132.9 (8,414.4 to 9,936.3)	255,000 (238,000 to 269,000)	4,897.2 (4,584.9 to 5,173.0)	252,000 (236,000 to 270,000)	4,731.0 (4,438.3 to 5,074.6)	255,000 (239,000 to 273,000)	4,678.7 (4,384.4 to 4,994.0)	-46.4 (-49.9 to -42.5)	-3.4 (-8.3 to 1.6)
	Under 5	100,000 (87,100 to 116,000)	21,628.8 (18,744.8 to 25,011.2)	44,100 (38,400 to 50,500)	5,762.2 (5,016.4 to 6,593.9)	41,100 (35,500 to 46,700)	5,371.4 (4,635.4 to 6,103.9)	40,400 (35,100 to 46,200)	5,265.5 (4,576.4 to 6,022.3)	-73.4 (-76.0 to -70.2)	-6.8 (-13.7 to 0.7)
	5-14 years	27,800 (22,600 to 33,800)	4,177.1 (3,399.9 to 5,078.1)	37,400 (30,800 to 44,500)	2,706.9 (2,234.7 to 3,226.9)	38,300 (31,700 to 45,300)	2,731.0 (2,262.5 to 3,229.3)	36,800 (30,800 to 44,600)	2,593.9 (2,171.3 to 3,138.9)	-35.2 (-42.5 to -26.0)	0.9 (-9.5 to 11.7)
	15-49 years	39,300 (34,900 to 43,700)	3,631.0 (3,222.4 to 4,035.0)	89,400 (81,100 to 98,600)	3,418.5 (3,102.0 to 3,771.7)	90,500 (81,100 to 99,600)	3,343.4 (2,999.2 to 3,682.5)	94,200 (84,500 to 104,000)	3,359.5 (3,013.8 to 3,713.5)	-5.9 (-14.0 to 2.7)	-2.2 (-10.1 to 6.1)
	50-69 years	30,500 (27,000 to 34,100)	16,235.4 (14,347.6 to 18,135.8)	49,900 (44,600 to 55,500)	13,802.4 (12,332.7 to 15,371.7)	50,000 (44,800 to 55,900)	13,291.9 (11,897.4 to 14,856.0)	52,000 (46,000 to 58,500)	13,318.9 (11,785.1 to 14,987.6)	-15.0 (-23.0 to -6.1)	-3.7 (-11.8 to 5.8)
	70+ years	26,600 (23,200 to 31,000)	44,747.3 (39,053.0 to 52,039.5)	34,000 (30,100 to 38,500)	42,761.6 (37,821.5 to 48,451.6)	32,100 (28,100 to 36,800)	41,322.1 (36,160.2 to 47,331.2)	32,100 (28,500 to 37,000)	40,263.1 (35,752.1 to 46,366.8)	-4.4 (-14.1 to 5.7)	-3.4 (-12.8 to 7.5)
Mali	All Ages	488,000 (452,000 to 522,000)	5,629.3 (5,217.5 to 6,029.2)	904,000 (840,000 to 971,000)	4,034.3 (3,747.9 to 4,332.9)	873,000 (806,000 to 940,000)	3,756.6 (3,468.0 to 4,040.8)	873,000 (807,000 to 940,000)	3,620.2 (3,346.9 to 3,900.5)	-28.3 (-31.9 to -24.5)	-6.9 (-12.0 to -1.2)

Location	Age Group	1990		2019		2020		2021		Percent Incidence Rate Change from 1990-2019 (95% UI)	Percent Incidence Rate Change from 2019-2021 (95% UI)
		Episodes (95% UI)	Rate (95% UI)	Episodes (95% UI)	Rate (95% UI)	Episodes (95% UI)	Rate (95% UI)	Episodes (95% UI)	Rate (95% UI)		
	Under 5	227,000 (201,000 to 259,000)	13,113.6 (11,635.3 to 14,966.7)	343,000 (304,000 to 392,000)	7,975.1 (7,075.9 to 9,112.7)	318,000 (275,000 to 367,000)	7,163.2 (6,199.9 to 8,273.0)	322,000 (281,000 to 368,000)	7,029.3 (6,130.1 to 8,029.8)	-39.2 (-44.2 to -33.0)	-10.2 (-18.1 to -1.5)
	5-14 years	52,800 (41,700 to 65,400)	2,197.6 (1,737.3 to 2,722.9)	109,000 (86,300 to 138,000)	1,674.7 (1,325.2 to 2,124.3)	112,000 (90,200 to 139,000)	1,658.7 (1,336.3 to 2,066.8)	109,000 (85,800 to 133,000)	1,553.4 (1,227.0 to 1,905.2)	-23.8 (-31.5 to -15.6)	-1.0 (-10.9 to 10.0)
	15-49 years	80,700 (73,300 to 89,300)	2,231.6 (2,024.7 to 2,469.0)	188,000 (169,000 to 209,000)	1,942.4 (1,747.3 to 2,159.8)	190,000 (169,000 to 212,000)	1,880.8 (1,676.2 to 2,105.0)	192,000 (172,000 to 213,000)	1,823.4 (1,636.4 to 2,027.0)	-13.0 (-19.3 to -6.2)	-3.2 (-10.6 to 5.3)
	50-69 years	81,200 (70,900 to 90,800)	10,792.1 (9,424.1 to 12,070.0)	156,000 (139,000 to 172,000)	10,004.3 (8,926.7 to 11,059.3)	151,000 (136,000 to 170,000)	9,404.9 (8,418.4 to 10,534.6)	154,000 (136,000 to 174,000)	9,283.6 (8,191.2 to 10,497.0)	-7.3 (-17.0 to 4.4)	-6.0 (-14.8 to 4.3)
	70+ years	46,200 (40,200 to 53,700)	28,433.7 (24,748.8 to 33,055.7)	108,000 (95,000 to 125,000)	29,798.5 (26,118.4 to 34,499.4)	102,000 (89,600 to 117,000)	27,753.3 (24,266.3 to 31,771.7)	96,800 (85,200 to 111,000)	26,207.9 (23,073.4 to 30,006.2)	4.8 (-9.1 to 18.8)	-6.9 (-17.9 to 5.0)
**Mauritania**	All Ages	147,000 (138,000 to 158,000)	7,176.6 (6,716.7 to 7,713.9)	229,000 (215,000 to 243,000)	5,431.7 (5,101.2 to 5,779.0)	219,000 (205,000 to 234,000)	5,087.3 (4,757.8 to 5,438.6)	217,000 (203,000 to 233,000)	4,928.3 (4,620.2 to 5,294.1)	-24.3 (-28.3 to -20.3)	-6.3 (-11.3 to -0.4)
	Under 5	53,500 (46,200 to 61,800)	14,432.6 (12,441.9 to 16,656.6)	47,600 (42,100 to 54,000)	7,244.6 (6,406.0 to 8,224.9)	42,700 (36,800 to 48,800)	6,498.8 (5,602.1 to 7,424.3)	41,000 (35,800 to 46,800)	6,244.7 (5,441.9 to 7,126.8)	-49.8 (-53.2 to -46.0)	-10.3 (-18.0 to -2.2)
	5-14 years	17,200 (13,800 to 21,200)	3,115.5 (2,490.4 to 3,833.9)	26,500 (21,300 to 31,800)	2,300.2 (1,852.2 to 2,762.5)	26,900 (21,800 to 32,800)	2,289.1 (1,851.5 to 2,794.7)	25,600 (20,800 to 30,900)	2,139.0 (1,741.2 to 2,584.8)	-26.2 (-33.8 to -18.3)	-0.5 (-10.5 to 11.7)
	15-49 years	30,600 (27,700 to 34,100)	3,359.8 (3,033.5 to 3,742.1)	57,500 (52,000 to 63,700)	2,963.4 (2,680.5 to 3,284.4)	56,600 (51,300 to 62,800)	2,835.0 (2,567.3 to 3,147.0)	57,400 (51,700 to 63,300)	2,793.5 (2,514.6 to 3,079.6)	-11.8 (-18.3 to -4.2)	-4.3 (-11.2 to 3.7)
	50-69 years	25,100 (22,200 to 28,300)	15,071.0 (13,337.9 to 16,996.0)	51,300 (45,500 to 56,800)	14,104.6 (12,511.9 to 15,622.3)	49,900 (43,800 to 56,100)	13,285.7 (11,645.2 to 14,933.4)	50,400 (44,900 to 56,400)	13,079.9 (11,671.4 to 14,659.6)	-6.4 (-15.0 to 3.8)	-5.8 (-14.4 to 4.1)
	70+ years	21,000 (18,300 to 24,200)	40,199.0 (35,027.9 to 46,302.9)	45,900 (40,200 to 52,600)	46,427.2 (40,673.8 to 53,188.0)	42,800 (38,000 to 49,100)	43,170.6 (38,288.5 to 49,566.8)	42,200 (37,300 to 48,400)	41,500.5 (36,607.6 to 47,564.4)	15.5 (4.4 to 31.5)	-7.0 (-16.8 to 3.1)
**Niger**	All Ages	753,000 (690,000 to 822,000)	9,370.0 (8,585.3 to 10,231.9)	1,450,000 (1,340,000 to 1,550,000)	6,232.7 (5,795.5 to 6,699.5)	1,420,000 (1,320,000 to 1,540,000)	5,904.0 (5,475.1 to 6,374.1)	1,420,000 (1,320,000 to 1,530,000)	5,676.8 (5,265.1 to 6,107.1)	-33.5 (-37.4 to -29.7)	-5.3 (-10.1 to 0.9)
	Under 5	391,000 (341,000 to 447,000)	23,180.9 (20,223.9 to 26,493.5)	504,000 (436,000 to 584,000)	10,631.9 (9,195.6 to 12,319.0)	477,000 (408,000 to 551,000)	9,704.2 (8,315.5 to 11,215.1)	476,000 (418,000 to 544,000)	9,343.0 (8,193.0 to 10,683.1)	-54.1 (-58.5 to -50.1)	-8.7 (-16.8 to 0.1)
	5-14 years	95,200 (76,300 to 116,000)	4,005.1 (3,213.2 to 4,900.3)	208,000 (167,000 to 256,000)	2,903.8 (2,322.5 to 3,570.2)	216,000 (178,000 to 270,000)	2,905.8 (2,400.3 to 3,632.5)	208,000 (170,000 to 253,000)	2,707.1 (2,212.8 to 3,304.7)	-27.5 (-35.3 to -18.8)	0.1 (-10.1 to 11.4)
	15-49 years	130,000 (116,000 to 144,000)	3,884.7 (3,467.5 to 4,291.9)	333,000 (300,000 to 373,000)	3,510.0 (3,163.1 to 3,930.7)	337,000 (304,000 to 370,000)	3,397.0 (3,066.7 to 3,736.2)	343,000 (311,000 to 379,000)	3,305.7 (3,002.6 to 3,657.9)	-9.6 (-17.0 to -1.3)	-3.2 (-11.5 to 6.1)

Location	Age Group	1990		2019		2020		2021		Percent Incidence Rate Change from 1990-2019 (95% UI)	Percent Incidence Rate Change from 2019-2021 (95% UI)
		Episodes (95% UI)	Rate (95% UI)	Episodes (95% UI)	Rate (95% UI)	Episodes (95% UI)	Rate (95% UI)	Episodes (95% UI)	Rate (95% UI)		
	50-69 years	87,900 (78,100 to 98,200)	16,973.5 (15,064.5 to 18,960.6)	246,000 (221,000 to 276,000)	16,499.1 (14,834.1 to 18,528.9)	236,000 (206,000 to 265,000)	15,560.0 (13,575.1 to 17,496.5)	242,000 (217,000 to 273,000)	15,483.4 (13,873.5 to 17,460.7)	-2.8 (-12.3 to 9.3)	-5.7 (-14.5 to 4.5)
	70+ years	48,400 (42,200 to 54,800)	47,062.7 (41,056.1 to 53,256.5)	155,000 (138,000 to 178,000)	49,789.0 (44,120.7 to 57,119.8)	158,000 (137,000 to 181,000)	46,729.3 (40,454.0 to 53,658.8)	153,000 (131,000 to 177,000)	44,907.9 (38,350.1 to 52,032.2)	5.8 (-6.5 to 19.3)	-6.1 (-14.9 to 4.2)
**Nigeria**	All Ages	7,680,000 (7,120,000 to 8,240,000)	8,532.4 (7,911.2 to 9,147.9)	12,700,000 (11,900,000 to 13,400,000)	5,799.5 (5,446.8 to 6,136.7)	12,400,000 (11,600,000 to 13,200,000)	5,500.0 (5,166.6 to 5,855.4)	12,300,000 (11,500,000 to 13,000,000)	5,319.1 (4,994.3 to 5,631.1)	-32.0 (-34.2 to -29.8)	-5.2 (-7.5 to -2.5)
	Under 5	3,550,000 (3,140,000 to 4,010,000)	22,308.0 (19,763.0 to 25,208.8)	3,670,000 (3,280,000 to 4,110,000)	10,135.4 (9,053.2 to 11,358.3)	3,390,000 (3,000,000 to 3,810,000)	9,254.0 (8,178.1 to 10,385.5)	3,310,000 (2,960,000 to 3,700,000)	8,926.0 (7,965.9 to 9,966.7)	-54.6 (-55.8 to -53.3)	-8.7 (-12.4 to -4.7)
	5-14 years	920,000 (741,000 to 1,120,000)	3,959.3 (3,190.6 to 4,831.8)	1,600,000 (1,330,000 to 1,930,000)	2,609.6 (2,165.0 to 3,132.6)	1,650,000 (1,370,000 to 1,970,000)	2,616.7 (2,166.5 to 3,128.0)	1,580,000 (1,310,000 to 1,910,000)	2,453.9 (2,031.3 to 2,956.7)	-34.1 (-37.1 to -30.9)	0.3 (-2.5 to 3.2)
	15-49 years	1,410,000 (1,270,000 to 1,570,000)	3,441.2 (3,097.9 to 3,826.3)	3,290,000 (2,990,000 to 3,640,000)	3,262.5 (2,956.7 to 3,605.0)	3,320,000 (3,010,000 to 3,660,000)	3,181.0 (2,887.2 to 3,507.5)	3,350,000 (3,040,000 to 3,690,000)	3,107.0 (2,815.7 to 3,425.0)	-5.2 (-8.8 to -1.3)	-2.5 (-4.7 to 0.0)
	50-69 years	1,030,000 (918,000 to 1,150,000)	13,198.7 (11,763.7 to 14,760.7)	2,380,000 (2,130,000 to 2,630,000)	14,225.5 (12,766.9 to 15,714.6)	2,310,000 (2,080,000 to 2,550,000)	13,350.5 (11,988.2 to 14,754.4)	2,380,000 (2,140,000 to 2,640,000)	13,268.3 (11,918.8 to 14,667.0)	7.8 (2.9 to 12.6)	-6.2 (-8.1 to -3.9)
	70+ years	775,000 (682,000 to 870,000)	37,011.8 (32,563.1 to 41,545.6)	1,750,000 (1,570,000 to 1,950,000)	49,552.0 (44,562.2 to 55,236.0)	1,700,000 (1,530,000 to 1,900,000)	46,262.1 (41,642.6 to 51,515.1)	1,670,000 (1,490,000 to 1,850,000)	44,481.5 (39,918.9 to 49,473.5)	33.9 (26.7 to 42.8)	-6.6 (-9.0 to -3.8)
**Sao Tome and Principe**	All Ages	10,600 (9,880 to 11,500)	8,764.0 (8,142.5 to 9,482.1)	13,900 (13,000 to 14,800)	6,625.0 (6,180.4 to 7,049.8)	13,300 (12,300 to 14,300)	6,245.0 (5,766.7 to 6,688.8)	13,200 (12,400 to 14,200)	6,112.1 (5,709.0 to 6,534.9)	-24.4 (-29.3 to -19.4)	-5.7 (-11.5 to 0.1)
	Under 5	3,670 (3,190 to 4,200)	17,900.9 (15,554.6 to 20,514.6)	1,530 (1,330 to 1,730)	5,896.6 (5,138.1 to 6,684.8)	1,270 (1,100 to 1,460)	4,999.7 (4,309.8 to 5,762.4)	1,190 (1,020 to 1,380)	4,766.4 (4,102.8 to 5,519.3)	-67.1 (-69.7 to -64.0)	-15.2 (-22.4 to -6.1)
	5-14 years	1,640 (1,340 to 2,000)	4,534.2 (3,695.7 to 5,523.3)	1,460 (1,180 to 1,780)	2,752.3 (2,224.1 to 3,352.6)	1,440 (1,180 to 1,740)	2,707.9 (2,229.3 to 3,275.5)	1,330 (1,080 to 1,600)	2,513.5 (2,049.1 to 3,032.3)	-39.3 (-46.1 to -30.3)	-1.6 (-11.8 to 10.3)
	15-49 years	2,020 (1,800 to 2,260)	4,046.6 (3,609.6 to 4,527.4)	5,120 (4,580 to 5,620)	4,773.1 (4,274.6 to 5,241.6)	5,020 (4,480 to 5,580)	4,563.4 (4,072.2 to 5,076.5)	5,090 (4,600 to 5,590)	4,506.5 (4,078.3 to 4,952.5)	18.0 (6.0 to 30.3)	-4.4 (-12.8 to 5.4)
	50-69 years	1,840 (1,630 to 2,080)	16,266.2 (14,396.6 to 18,406.8)	3,500 (3,090 to 3,940)	17,931.9 (15,821.9 to 20,163.7)	3,440 (3,040 to 3,890)	16,844.2 (14,872.6 to 19,027.4)	3,530 (3,100 to 4,060)	16,579.6 (14,528.1 to 19,044.1)	10.2 (0.0 to 23.0)	-6.1 (-15.9 to 4.3)
	70+ years	1,460 (1,290 to 1,670)	44,211.9 (38,942.5 to 50,448.7)	2,320 (2,020 to 2,630)	51,804.6 (45,244.6 to 58,907.7)	2,160 (1,900 to 2,480)	47,925.0 (42,130.5 to 55,067.2)	2,100 (1,860 to 2,400)	45,832.9 (40,518.0 to 52,274.1)	17.2 (7.1 to 30.3)	-7.5 (-17.0 to 2.3)

Location	Age Group	1990		2019		2020		2021		Percent Incidence Rate Change from 1990-2019 (95% UI)	Percent Incidence Rate Change from 2019-2021 (95% UI)
		Episodes (95% UI)	Rate (95% UI)	Episodes (95% UI)	Rate (95% UI)	Episodes (95% UI)	Rate (95% UI)	Episodes (95% UI)	Rate (95% UI)		
**Senegal**	All Ages	518,000 (478,000 to 560,000)	6,783.4 (6,264.5 to 7,343.2)	811,000 (761,000 to 863,000)	5,313.0 (4,981.7 to 5,649.1)	757,000 (702,000 to 810,000)	4,859.0 (4,511.2 to 5,203.7)	751,000 (699,000 to 809,000)	4,736.6 (4,408.7 to 5,102.4)	-21.7 (-26.3 to -16.6)	-8.5 (-14.0 to -2.8)
	Under 5	220,000 (191,000 to 253,000)	14,999.6 (13,056.3 to 17,260.0)	167,000 (147,000 to 189,000)	7,367.7 (6,481.7 to 8,342.8)	140,000 (121,000 to 164,000)	6,189.6 (5,327.2 to 7,218.3)	134,000 (116,000 to 158,000)	5,920.4 (5,098.3 to 6,955.1)	-50.9 (-55.1 to -45.8)	-16.0 (-24.6 to -6.8)
	5-14 years	70,000 (56,200 to 85,500)	3,201.2 (2,570.1 to 3,910.0)	98,500 (78,800 to 120,000)	2,473.9 (1,978.8 to 3,010.2)	96,300 (77,600 to 117,000)	2,384.7 (1,920.5 to 2,889.4)	92,000 (73,800 to 112,000)	2,251.0 (1,805.9 to 2,737.7)	-22.7 (-30.5 to -12.9)	-3.6 (-15.5 to 8.7)
	15-49 years	102,000 (91,200 to 113,000)	3,110.4 (2,795.0 to 3,458.6)	230,000 (207,000 to 258,000)	3,136.1 (2,819.6 to 3,515.0)	222,000 (200,000 to 245,000)	2,942.3 (2,659.0 to 3,251.7)	226,000 (203,000 to 253,000)	2,925.1 (2,627.9 to 3,265.9)	0.8 (-7.4 to 9.5)	-6.2 (-13.6 to 2.1)
	50-69 years	71,200 (62,300 to 79,100)	12,595.6 (11,030.9 to 13,989.6)	176,000 (157,000 to 198,000)	13,102.2 (11,687.9 to 14,775.0)	167,000 (148,000 to 189,000)	12,121.0 (10,790.8 to 13,723.1)	170,000 (151,000 to 190,000)	12,041.3 (10,726.6 to 13,469.2)	4.0 (-5.4 to 16.1)	-7.5 (-17.7 to 2.0)
	70+ years	55,300 (48,600 to 62,900)	36,544.4 (32,112.7 to 41,597.6)	140,000 (122,000 to 158,000)	40,987.1 (35,769.6 to 46,169.7)	132,000 (114,000 to 152,000)	37,299.4 (32,340.1 to 43,029.9)	129,000 (112,000 to 152,000)	36,363.1 (31,569.9 to 42,849.8)	12.2 (-1.3 to 27.5)	-9.0 (-19.3 to 1.3)
**Sierra Leone**	All Ages	391,000 (363,000 to 419,000)	9,414.5 (8,737.9 to 10,089.0)	588,000 (552,000 to 628,000)	7,018.0 (6,587.9 to 7,497.6)	567,000 (524,000 to 608,000)	6,575.3 (6,073.4 to 7,046.4)	564,000 (528,000 to 603,000)	6,357.5 (5,950.6 to 6,802.9)	-25.5 (-29.5 to -21.5)	-6.3 (-11.5 to -0.2)
	Under 5	162,000 (142,000 to 185,000)	20,978.4 (18,312.0 to 23,950.1)	134,000 (119,000 to 151,000)	10,258.3 (9,122.3 to 11,583.3)	121,000 (106,000 to 138,000)	9,146.4 (8,032.7 to 10,437.7)	117,000 (102,000 to 134,000)	8,740.8 (7,607.2 to 9,956.3)	-51.1 (-54.8 to -46.6)	-10.8 (-17.9 to -2.6)
	5-14 years	42,400 (33,300 to 52,000)	4,079.3 (3,202.1 to 5,008.9)	77,400 (63,700 to 93,400)	3,637.7 (2,991.3 to 4,385.6)	78,800 (63,700 to 94,300)	3,614.1 (2,923.1 to 4,325.7)	75,000 (60,800 to 90,900)	3,358.8 (2,720.7 to 4,068.1)	-10.8 (-21.6 to 0.4)	-0.6 (-10.5 to 12.2)
	15-49 years	72,000 (64,200 to 79,900)	3,788.5 (3,381.1 to 4,204.3)	174,000 (156,000 to 191,000)	4,171.5 (3,738.6 to 4,591.2)	172,000 (155,000 to 189,000)	4,003.1 (3,591.1 to 4,402.7)	175,000 (158,000 to 193,000)	3,937.8 (3,559.5 to 4,349.1)	10.1 (-0.9 to 21.1)	-4.0 (-11.9 to 5.6)
	50-69 years	58,200 (51,700 to 65,300)	17,595.0 (15,635.0 to 19,729.7)	114,000 (101,000 to 128,000)	18,237.1 (16,178.8 to 20,394.9)	109,000 (96,300 to 123,000)	16,775.0 (14,835.2 to 18,888.4)	112,000 (99,500 to 125,000)	16,626.9 (14,778.6 to 18,561.6)	3.6 (-7.0 to 15.1)	-8.0 (-17.9 to 2.5)
	70+ years	56,000 (48,800 to 63,900)	51,804.1 (45,196.5 to 59,126.2)	89,600 (79,500 to 101,000)	54,772.8 (48,622.0 to 61,990.3)	86,200 (76,700 to 97,700)	51,077.8 (45,471.4 to 57,931.8)	84,400 (74,600 to 94,200)	49,192.9 (43,524.6 to 54,938.7)	5.7 (-4.8 to 18.1)	-6.7 (-17.1 to 4.5)
**Togo**	All Ages	271,000 (252,000 to 291,000)	7,434.0 (6,903.8 to 7,987.4)	463,000 (434,000 to 493,000)	5,771.2 (5,414.9 to 6,151.4)	450,000 (415,000 to 484,000)	5,484.3 (5,061.7 to 5,898.4)	459,000 (423,000 to 495,000)	5,486.6 (5,052.8 to 5,915.8)	-22.4 (-26.5 to -17.6)	-5.0 (-11.5 to 2.5)
	Under 5	119,000 (104,000 to 135,000)	17,341.4 (15,142.9 to 19,725.6)	99,800 (87,400 to 113,000)	8,437.1 (7,395.7 to 9,595.3)	89,500 (76,200 to 105,000)	7,591.7 (6,461.5 to 8,879.2)	89,500 (76,600 to 104,000)	7,634.0 (6,531.9 to 8,833.0)	-51.3 (-55.0 to -46.5)	-10.0 (-19.6 to 1.8)
	5-14 years	38,000 (30,300 to 46,300)	3,532.6 (2,812.2 to 4,299.3)	55,500 (44,900 to 67,000)	2,690.6 (2,178.1 to 3,249.3)	56,600 (45,900 to 67,800)	2,695.0 (2,186.7 to 3,229.0)	54,900 (45,200 to 65,700)	2,567.9 (2,114.7 to 3,074.4)	-23.8 (-32.3 to -13.8)	0.2 (-11.4 to 13.1)

Location	Age Group	1990		2019		2020		2021		Percent Incidence Rate Change from 1990-2019 (95% UI)	Percent Incidence Rate Change from 2019-2021 (95% UI)
		Episodes (95% UI)	Rate (95% UI)	Episodes (95% UI)	Rate (95% UI)	Episodes (95% UI)	Rate (95% UI)	Episodes (95% UI)	Rate (95% UI)		
	15-49 years	56,300 (50,800 to 62,600)	3,502.6 (3,159.0 to 3,889.8)	143,000 (129,000 to 158,000)	3,623.5 (3,262.0 to 4,008.1)	141,000 (126,000 to 156,000)	3,486.8 (3,101.8 to 3,844.4)	146,000 (130,000 to 165,000)	3,518.1 (3,139.0 to 3,973.4)	3.5 (-6.6 to 13.6)	-3.8 (-13.1 to 5.3)
	50-69 years	34,600 (30,500 to 38,900)	15,345.4 (13,542.5 to 17,264.0)	107,000 (95,000 to 119,000)	15,224.1 (13,517.5 to 16,930.9)	105,000 (90,400 to 116,000)	14,325.8 (12,366.3 to 15,868.2)	109,000 (96,600 to 125,000)	14,379.5 (12,695.8 to 16,420.5)	-0.8 (-10.4 to 10.2)	-5.9 (-17.2 to 4.4)
	70+ years	23,200 (20,200 to 26,500)	45,680.6 (39,850.0 to 52,177.1)	57,800 (51,500 to 65,500)	43,503.0 (38,770.5 to 49,329.6)	57,600 (50,300 to 66,900)	41,173.4 (35,923.6 to 47,833.9)	59,300 (51,800 to 70,200)	41,008.9 (35,869.2 to 48,588.0)	-4.8 (-14.3 to 7.6)	-5.4 (-15.6 to 5.7)

Appendix Table S2: All-age, and sub age groups LRI mortality counts, rates, and percent change in 1990, 2019, 2020, & 2021 by country and socio-demographic index (SDI)

Country	Age Group	1990 Deaths (95% UI)	1990 Mortality Rate per 100,000 (95% UI)	2019 Deaths (95% UI)	2019 Mortality Rate per 100,000 (95% UI)	2020 Deaths (95% UI)	2020 Mortality Rate per 100,000 (95% UI)	2021 Deaths (95% UI)	2021 Mortality Rate per 100,000 (95% UI)	Percent Mortality Rate Change from 1990 - 2019 (95% UI)	Percent Mortality Rate Change from 2019 - 2021 (95% UI)
**High SDI**	All Ages	269,000<br>(244,000 to 281,000)	30.6<br>(27.7 to 32.0)	363,000<br>(308,000 to 393,000)	33.4<br>(28.4 to 36.1)	332,000<br>(284,000 to 359,000)	30.4<br>(26.0 to 32.9)	299,000<br>(252,000 to 325,000)	27.4<br>(23.0 to 29.7)	-10.5<br>(-17.2 to -6.7)	-18.0<br>(-19.2 to -17.0)
	Under 5	8,370<br>(7,650 to 9,280)	13.6<br>(12.4 to 15.0)	1,750<br>(1,640 to 1,870)	3.1<br>(2.9 to 3.3)	1,350<br>(1,220 to 1,470)	2.5<br>(2.2 to 2.7)	998<br>(898 to 1,080)	1.9<br>(1.7 to 2.0)	-86.3<br>(-88.2 to -84.9)	-40.8<br>(-45.0 to -37.1)
	5-14 years	1,360<br>(1,270 to 1,460)	1.1<br>(1.0 to 1.2)	469<br>(448 to 497)	0.4<br>(0.4 to 0.4)	398<br>(374 to 428)	0.3<br>(0.3 to 0.4)	354<br>(333 to 381)	0.3<br>(0.3 to 0.3)	-72.9<br>(-75.0 to -70.5)	-24.9<br>(-27.2 to -22.8)
	15-49 years	10,500<br>(10,200 to 10,700)	2.3<br>(2.2 to 2.3)	9,230<br>(8,760 to 9,810)	1.8<br>(1.7 to 1.9)	8,350<br>(7,840 to 8,970)	1.7<br>(1.6 to 1.8)	7,330<br>(6,850 to 7,910)	1.5<br>(1.4 to 1.6)	-35.9<br>(-40.3 to -30.6)	-20.1<br>(-22.3 to -17.6)
	50-69 years	31,000<br>(30,200 to 31,600)	18.9<br>(18.5 to 19.3)	37,500<br>(36,200 to 38,500)	13.8<br>(13.3 to 14.2)	34,300<br>(33,100 to 35,400)	12.5<br>(12.1 to 12.9)	31,100<br>(29,900 to 32,200)	11.3<br>(10.8 to 11.7)	-40.4<br>(-42.3 to -38.6)	-18.3<br>(-19.8 to -16.7)
	70+ years	218,000<br>(192,000 to 230,000)	315.2<br>(278.4 to 332.6)	314,000<br>(260,000 to 343,000)	231.4<br>(191.6 to 252.8)	288,000<br>(239,000 to 313,000)	205.7<br>(171.2 to 224.0)	260,000<br>(213,000 to 284,000)	180.9<br>(148.2 to 198.0)	-42.6<br>(-47.0 to -40.0)	-21.8<br>(-22.9 to -20.8)
**High-middle SDI**	All Ages	248,000<br>(231,000 to 268,000)	23.3<br>(21.7 to 25.2)	275,000<br>(249,000 to 296,000)	21.2<br>(19.2 to 22.8)	252,000<br>(226,000 to 272,000)	19.3<br>(17.4 to 20.9)	242,000<br>(216,000 to 266,000)	18.5<br>(16.6 to 20.4)	-20.5<br>(-28.4 to -12.3)	-12.7<br>(-17.4 to -7.3)
	Under 5	114,000<br>(101,000 to 131,000)	122.3<br>(108.3 to 140.9)	9,000<br>(7,880 to 10,300)	11.8<br>(10.3 to 13.4)	7,190<br>(6,200 to 8,300)	9.7<br>(8.4 to 11.2)	6,000<br>(5,050 to 7,020)	8.6<br>(7.2 to 10.0)	-93.0<br>(-94.6 to -91.5)	-27.0<br>(-31.6 to -22.7)
	5-14 years	5,900<br>(5,370 to 6,460)	3.3<br>(3.0 to 3.6)	1,520<br>(1,410 to 1,700)	1.0<br>(0.9 to 1.1)	1,280<br>(1,180 to 1,440)	0.8<br>(0.7 to 0.9)	1,210<br>(1,110 to 1,380)	0.8<br>(0.7 to 0.9)	-76.9<br>(-79.5 to -73.5)	-23.5<br>(-26.5 to -20.8)
	15-49 years	17,100<br>(16,000 to 18,100)	3.0<br>(2.8 to 3.2)	20,400<br>(19,600 to 21,300)	3.2<br>(3.1 to 3.3)	18,400<br>(17,600 to 19,400)	2.9<br>(2.8 to 3.1)	17,200<br>(16,200 to 18,400)	2.7<br>(2.6 to 2.9)	-9.6<br>(-17.0 to -1.0)	-14.0<br>(-19.7 to -7.9)
	50-69 years	29,700<br>(27,800 to 31,700)	17.1<br>(16.0 to 18.2)	48,000<br>(45,700 to 50,500)	15.2<br>(14.5 to 16.0)	44,700<br>(42,300 to 47,400)	13.9<br>(13.2 to 14.8)	42,300<br>(39,600 to 45,300)	13.0<br>(12.1 to 13.9)	-23.9<br>(-30.5 to -17.2)	-14.8<br>(-19.9 to -8.8)
	70+ years	81,700<br>(73,800 to 88,600)	158.7<br>(143.5 to 172.1)	197,000<br>(171,000 to 216,000)	178.9<br>(156.0 to 197.0)	180,000<br>(156,000 to 198,000)	158.7<br>(137.8 to 174.3)	175,000<br>(151,000 to 196,000)	149.1<br>(128.3 to 167.0)	-6.1<br>(-14.6 to 2.6)	-16.7<br>(-21.6 to -11.0)
**Middle SDI**	All Ages	777,000<br>(715,000 to 840,000)	45.1<br>(41.5 to 48.8)	605,000<br>(555,000 to 647,000)	25.1<br>(23.0 to 26.8)	548,000<br>(502,000 to 588,000)	22.5<br>(20.6 to 24.2)	543,000<br>(494,000 to 589,000)	22.2<br>(20.2 to 24.1)	-50.8<br>(-55.8 to -45.7)	-11.7<br>(-16.1 to -7.3)
	Under 5	509,000<br>(457,000 to 568,000)	253.9<br>(227.7 to 283.2)	90,900<br>(78,300 to 106,000)	48.5<br>(41.8 to 56.4)	70,200<br>(59,900 to 82,000)	38.3<br>(32.7 to 44.8)	60,400<br>(50,800 to 71,200)	34.2<br>(28.8 to 40.3)	-86.5<br>(-88.8 to -83.8)	-29.4<br>(-34.0 to -24.6)
	5-14 years	26,300<br>(22,100 to 28,600)	7.0<br>(5.9 to 7.6)	9,510<br>(8,610 to 10,700)	2.5<br>(2.3 to 2.8)	8,170<br>(7,440 to 9,070)	2.1<br>(1.9 to 2.3)	7,660<br>(6,950 to 8,440)	2.0<br>(1.8 to 2.2)	-71.9<br>(-75.1 to -67.4)	-21.2<br>(-24.9 to -17.3)
	15-49 years	46,100<br>(42,500 to 48,900)	5.1<br>(4.7 to 5.4)	47,000<br>(44,800 to 50,000)	3.8<br>(3.6 to 4.0)	42,700<br>(40,500 to 45,300)	3.4<br>(3.2 to 3.6)	42,700<br>(40,000 to 45,900)	3.4<br>(3.2 to 3.7)	-32.8<br>(-38.0 to -26.8)	-9.5<br>(-14.1 to -4.9)

	50-69 years	60,400 (55,100 to 65,500)	31.9 (29.1 to 34.6)	111,000 (104,000 to 117,000)	24.1 (22.5 to 25.4)	105,000 (97,800 to 112,000)	22.3 (20.6 to 23.7)	106,000 (97,300 to 114,000)	21.8 (20.0 to 23.4)	-31.7 (-38.0 to -24.0)	-9.5 (-14.7 to -4.5)
	70+ years	135,000 (122,000 to 149,000)	295.5 (266.1 to 326.0)	348,000 (309,000 to 376,000)	265.6 (236.1 to 287.7)	321,000 (284,000 to 349,000)	237.0 (209.5 to 258.0)	326,000 (288,000 to 358,000)	231.5 (204.3 to 254.2)	-21.7 (-29.3 to -13.3)	-12.9 (-17.5 to -7.8)
**Low-middle SDI**	All Ages	954,000 (850,000 to 1,070,000)	82.1 (73.2 to 91.9)	712,000 (641,000 to 777,000)	37.9 (34.2 to 41.4)	619,000 (558,000 to 680,000)	32.6 (29.4 to 35.8)	594,000 (528,000 to 657,000)	30.9 (27.5 to 34.2)	-62.3 (-67.4 to -56.8)	-18.5 (-22.8 to -13.5)
	Under 5	719,000 (626,000 to 827,000)	414.3 (360.7 to 476.8)	263,000 (222,000 to 307,000)	134.3 (113.6 to 156.9)	200,000 (168,000 to 238,000)	103.4 (86.7 to 122.8)	180,000 (148,000 to 215,000)	94.0 (77.5 to 112.4)	-77.3 (-81.4 to -72.2)	-30.0 (-35.9 to -23.2)
	5-14 years	31,500 (26,000 to 36,500)	10.6 (8.7 to 12.2)	17,300 (15,000 to 19,700)	4.5 (3.9 to 5.1)	15,000 (12,900 to 17,100)	3.9 (3.3 to 4.4)	14,200 (12,100 to 16,300)	3.6 (3.1 to 4.2)	-65.5 (-70.4 to -59.0)	-18.5 (-23.0 to -13.9)
	15-49 years	37,200 (33,900 to 42,200)	6.7 (6.1 to 7.7)	50,400 (45,400 to 56,900)	5.1 (4.6 to 5.8)	47,400 (42,600 to 53,600)	4.7 (4.3 to 5.4)	47,200 (41,900 to 53,500)	4.6 (4.1 to 5.3)	-31.2 (-37.9 to -22.6)	-9.0 (-13.8 to -4.1)
	50-69 years	68,000 (60,400 to 75,700)	60.8 (54.0 to 67.6)	123,000 (110,000 to 135,000)	50.8 (45.7 to 56.0)	116,000 (103,000 to 129,000)	46.5 (41.4 to 51.8)	114,000 (100,000 to 127,000)	44.8 (39.3 to 50.0)	-26.3 (-35.0 to -15.0)	-11.7 (-17.0 to -5.5)
	70+ years	98,100 (85,900 to 114,000)	374.0 (327.5 to 435.3)	259,000 (232,000 to 286,000)	388.4 (348.6 to 428.5)	241,000 (215,000 to 268,000)	352.6 (314.0 to 391.8)	238,000 (209,000 to 268,000)	340.2 (298.2 to 382.1)	-9.0 (-20.3 to 4.3)	-12.4 (-17.3 to -7.2)
**Low SDI**	All Ages	763,000 (644,000 to 891,000)	152.2 (128.5 to 177.7)	591,000 (512,000 to 681,000)	55.4 (48.0 to 63.8)	527,000 (452,000 to 611,000)	48.3 (41.4 to 56.0)	503,000 (430,000 to 582,000)	45.0 (38.5 to 52.1)	-70.5 (-74.7 to -65.0)	-18.9 (-22.5 to -15.1)
	Under 5	593,000 (477,000 to 726,000)	653.5 (525.6 to 800.1)	328,000 (262,000 to 403,000)	202.5 (162.0 to 249.0)	277,000 (217,000 to 346,000)	169.3 (132.5 to 211.1)	254,000 (197,000 to 320,000)	153.2 (118.7 to 193.4)	-76.6 (-81.0 to -70.9)	-24.3 (-29.4 to -19.3)
	5-14 years	23,800 (18,100 to 28,600)	17.3 (13.1 to 20.7)	23,100 (19,200 to 26,900)	8.1 (6.7 to 9.4)	21,200 (17,500 to 24,600)	7.3 (6.0 to 8.5)	20,300 (16,700 to 23,900)	6.9 (5.7 to 8.1)	-60.0 (-66.9 to -50.0)	-14.7 (-19.2 to -9.7)
	15-49 years	29,800 (25,600 to 33,700)	13.5 (11.6 to 15.2)	46,600 (40,300 to 54,000)	9.1 (7.9 to 10.6)	44,900 (38,900 to 52,000)	8.5 (7.4 to 9.9)	45,600 (39,200 to 52,700)	8.4 (7.2 to 9.7)	-37.6 (-45.4 to -28.6)	-7.8 (-12.3 to -3.3)
	50-69 years	53,400 (46,100 to 60,500)	127.2 (109.8 to 144.1)	74,800 (65,300 to 85,400)	85.9 (75.0 to 98.1)	72,600 (63,300 to 83,000)	80.4 (70.2 to 92.0)	72,900 (63,200 to 83,500)	78.5 (68.1 to 90.0)	-38.3 (-45.5 to -29.2)	-8.6 (-12.9 to -4.4)
	70+ years	62,800 (54,500 to 72,200)	673.0 (584.5 to 773.7)	119,000 (106,000 to 136,000)	567.4 (504.6 to 647.7)	111,000 (99,300 to 126,000)	521.0 (464.4 to 590.1)	110,000 (97,900 to 126,000)	501.7 (446.2 to 572.5)	-25.5 (-34.3 to -15.3)	-11.6 (-15.9 to -7.4)
**Armenia**	All Ages	1,140 (1,030 to 1,270)	33.4 (30.1 to 37.2)	966 (876 to 1,040)	31.9 (29.0 to 34.3)	930 (818 to 1,020)	30.9 (27.2 to 34.0)	836 (719 to 964)	27.9 (24.0 to 32.2)	-16.4 (-30.1 to -0.7)	-12.6 (-21.9 to -1.0)
	Under 5	920 (810 to 1,050)	240.2 (211.4 to 275.0)	112 (91 to 138)	56.3 (45.9 to 69.1)	98 (77 to 123)	50.8 (40.0 to 63.8)	77 (60 to 99)	41.5 (32.3 to 53.3)	-82.7 (-87.0 to -77.4)	-26.3 (-36.8 to -14.8)
	5-14 years	32 (29 to 35)	4.8 (4.4 to 5.3)	11 (10 to 13)	2.9 (2.5 to 3.3)	8 (6 to 9)	1.9 (1.6 to 2.1)	6 (5 to 7)	1.4 (1.2 to 1.7)	-70.6 (-76.1 to -64.2)	-50.3 (-56.4 to -42.4)
	15-49 years	59 (54 to 64)	3.5 (3.2 to 3.8)	41 (38 to 44)	2.8 (2.5 to 3.0)	39 (34 to 43)	2.7 (2.3 to 3.0)	37 (31 to 43)	2.5 (2.1 to 2.9)	-27.6 (-40.6 to -13.5)	-9.1 (-21.2 to 3.9)

	50-69 years	70 (65 to 76)	12.8 (11.7 to 13.8)	149 (134 to 168)	21.0 (18.9 to 23.7)	144 (124 to 167)	20.4 (17.6 to 23.6)	135 (112 to 163)	19.2 (15.9 to 23.2)	50.2 (21.3 to 87.4)	-8.7 (-20.8 to 5.0)
	70+ years	61 (54 to 67)	50.8 (45.3 to 56.2)	652 (575 to 708)	276.4 (243.5 to 299.7)	641 (551 to 713)	266.8 (229.3 to 296.4)	582 (494 to 676)	237.9 (201.9 to 276.6)	368.1 (297.7 to 449.5)	-13.9 (-23.5 to -2.1)
**Azerbaijan**	All Ages	8,250 (7,190 to 9,280)	112.6 (98.1 to 126.7)	2,660 (2,220 to 3,130)	25.7 (21.4 to 30.3)	2,430 (2,030 to 2,860)	23.3 (19.4 to 27.4)	1,830 (1,540 to 2,170)	17.4 (14.6 to 20.6)	-84.6 (-87.6 to -80.6)	-32.2 (-37.9 to -26.2)
	Under 5	7,430 (6,410 to 8,450)	822.0 (709.1 to 934.6)	1,590 (1,250 to 1,990)	205.8 (161.9 to 258.0)	1,400 (1,110 to 1,740)	187.1 (148.1 to 233.0)	966 (764 to 1,220)	134.0 (106.0 to 169.2)	-83.7 (-87.6 to -78.6)	-34.9 (-41.7 to -27.0)
	5-14 years	233 (188 to 273)	15.3 (12.4 to 17.9)	131 (106 to 157)	8.2 (6.6 to 9.8)	136 (109 to 163)	8.4 (6.7 to 10.1)	112 (90 to 133)	6.8 (5.5 to 8.1)	-55.4 (-66.4 to -42.8)	-16.9 (-24.3 to -10.0)
	15-49 years	226 (185 to 274)	6.1 (5.0 to 7.5)	209 (151 to 276)	3.8 (2.7 to 5.0)	181 (128 to 244)	3.3 (2.3 to 4.4)	156 (106 to 214)	2.8 (1.9 to 3.8)	-54.5 (-70.0 to -33.8)	-25.5 (-36.7 to -14.5)
	50-69 years	191 (155 to 234)	19.1 (15.4 to 23.3)	309 (242 to 389)	14.9 (11.7 to 18.8)	304 (236 to 387)	14.2 (11.1 to 18.2)	270 (204 to 346)	12.3 (9.3 to 15.8)	-35.3 (-52.9 to -9.9)	-17.2 (-28.5 to -4.7)
	70+ years	173 (136 to 222)	77.1 (60.7 to 99.0)	421 (297 to 584)	115.6 (81.5 to 160.4)	410 (288 to 570)	110.3 (77.5 to 153.4)	323 (228 to 446)	84.3 (59.4 to 116.3)	9.4 (-28.2 to 60.3)	-27.1 (-36.1 to -16.6)
**Georgia**	All Ages	1,970 (1,790 to 2,170)	35.6 (32.4 to 39.3)	1,670 (1,490 to 1,850)	45.4 (40.5 to 50.3)	1,590 (1,400 to 1,810)	43.7 (38.3 to 49.7)	1,320 (1,140 to 1,510)	36.6 (31.6 to 41.7)	2.7 (-13.3 to 20.1)	-19.5 (-25.9 to -11.5)
	Under 5	1,330 (1,170 to 1,530)	284.5 (249.0 to 325.8)	61 (49 to 76)	23.4 (18.8 to 29.3)	48 (37 to 61)	19.0 (14.6 to 24.3)	36 (28 to 46)	14.7 (11.4 to 19.0)	-94.8 (-96.0 to -93.2)	-37.0 (-43.1 to -31.5)
	5-14 years	95 (87 to 105)	10.5 (9.6 to 11.6)	10 (9 to 12)	2.1 (1.8 to 2.5)	9 (8 to 10)	1.8 (1.6 to 2.1)	6 (5 to 6)	1.1 (0.9 to 1.3)	-89.3 (-91.2 to -87.3)	-47.0 (-52.9 to -42.0)
	15-49 years	165 (144 to 188)	6.2 (5.4 to 7.0)	105 (90 to 121)	6.4 (5.4 to 7.3)	86 (72 to 103)	5.3 (4.4 to 6.3)	77 (64 to 90)	4.8 (4.0 to 5.6)	-22.2 (-36.6 to -5.6)	-24.6 (-31.3 to -18.1)
	50-69 years	180 (165 to 196)	15.8 (14.4 to 17.2)	400 (361 to 439)	43.9 (39.7 to 48.2)	383 (340 to 428)	42.2 (37.5 to 47.3)	338 (293 to 387)	37.6 (32.6 to 43.1)	138.4 (100.9 to 180.3)	-14.2 (-22.0 to -5.6)
	70+ years	193 (168 to 221)	58.5 (50.9 to 67.0)	1,090 (942 to 1,240)	291.5 (251.2 to 331.8)	1,070 (908 to 1,240)	284.3 (241.4 to 330.9)	862 (726 to 1,010)	231.2 (194.8 to 270.6)	295.2 (227.5 to 376.8)	-20.7 (-27.6 to -12.1)
**Kazakhstan**	All Ages	6,270 (5,780 to 6,840)	38.2 (35.3 to 41.7)	4,100 (3,660 to 4,580)	22.2 (19.8 to 24.8)	3,780 (3,250 to 4,300)	20.2 (17.4 to 23.0)	3,240 (2,780 to 3,700)	17.1 (14.7 to 19.5)	-55.3 (-61.7 to -48.0)	-23.1 (-30.1 to -15.7)
	Under 5	4,860 (4,380 to 5,410)	258.3 (232.6 to 287.3)	606 (497 to 742)	31.9 (26.1 to 39.0)	513 (419 to 635)	26.7 (21.8 to 33.0)	387 (315 to 477)	19.9 (16.2 to 24.5)	-92.3 (-93.9 to -90.4)	-37.7 (-43.5 to -31.4)
	5-14 years	189 (172 to 210)	5.7 (5.2 to 6.3)	75 (65 to 86)	2.3 (2.0 to 2.6)	56 (49 to 64)	1.7 (1.5 to 1.9)	46 (40 to 52)	1.3 (1.2 to 1.5)	-76.8 (-80.2 to -72.8)	-42.3 (-47.2 to -37.5)
	15-49 years	392 (363 to 424)	4.8 (4.4 to 5.2)	689 (616 to 773)	7.4 (6.7 to 8.4)	593 (515 to 671)	6.4 (5.5 to 7.2)	537 (470 to 607)	5.7 (5.0 to 6.5)	20.4 (3.5 to 41.1)	-22.9 (-31.2 to -14.0)

	50-69 years	439<br>(386 to 497)	18.4<br>(16.2 to 20.9)	1,240<br>(1,070 to 1,440)	37.9<br>(32.7 to 44.1)	1,170<br>(975 to 1,360)	35.2<br>(29.4 to 41.2)	1,040<br>(860 to 1,230)	31.1<br>(25.6 to 36.7)	68.5<br>(35.5 to 110.8)	-18.1<br>(-28.2 to -8.0)
	70+ years	385<br>(337 to 430)	64.1<br>(56.1 to 71.7)	1,500<br>(1,280 to 1,710)	192.5<br>(164.9 to 220.6)	1,450<br>(1,200 to 1,700)	181.5<br>(150.5 to 213.3)	1,230<br>(1,010 to 1,440)	150.8<br>(123.9 to 176.7)	135.1<br>(89.9 to 185.3)	-21.7<br>(-28.9 to -13.9)
**Kyrgyzstan**	All Ages	3,680<br>(3,330 to 4,050)	82.5<br>(74.6 to 90.8)	879<br>(814 to 948)	13.2<br>(12.2 to 14.3)	807<br>(723 to 890)	11.9<br>(10.7 to 13.2)	693<br>(606 to 783)	10.1<br>(8.8 to 11.4)	-87.7<br>(-89.7 to -85.7)	-23.5<br>(-30.6 to -16.1)
	Under 5	3,180<br>(2,830 to 3,550)	495.1<br>(440.4 to 551.6)	454<br>(404 to 507)	55.9<br>(49.7 to 62.4)	393<br>(338 to 448)	48.8<br>(42.0 to 55.6)	315<br>(262 to 370)	39.6<br>(33.0 to 46.6)	-92.0<br>(-93.6 to -90.2)	-29.1<br>(-37.8 to -19.4)
	5-14 years	115<br>(102 to 128)	11.1<br>(9.8 to 12.4)	53<br>(46 to 60)	3.8<br>(3.4 to 4.3)	51<br>(43 to 58)	3.5<br>(3.0 to 4.1)	45<br>(38 to 52)	3.0<br>(2.6 to 3.5)	-72.7<br>(-77.5 to -67.1)	-21.0<br>(-27.6 to -13.6)
	15-49 years	141<br>(129 to 154)	6.7<br>(6.2 to 7.3)	140<br>(123 to 160)	4.2<br>(3.6 to 4.7)	137<br>(116 to 161)	4.0<br>(3.4 to 4.7)	128<br>(104 to 156)	3.7<br>(3.0 to 4.5)	-44.5<br>(-55.9 to -30.7)	-10.0<br>(-21.3 to 2.6)
	50-69 years	115<br>(102 to 129)	21.0<br>(18.6 to 23.6)	127<br>(110 to 149)	14.0<br>(12.0 to 16.4)	126<br>(104 to 153)	13.5<br>(11.1 to 16.4)	118<br>(92 to 146)	12.3<br>(9.6 to 15.2)	-41.6<br>(-54.9 to -24.9)	-12.3<br>(-24.8 to 1.5)
	70+ years	128<br>(112 to 142)	88.3<br>(77.2 to 98.4)	104<br>(90 to 121)	58.6<br>(50.4 to 68.2)	100<br>(83 to 120)	54.3<br>(44.9 to 64.6)	88<br>(71 to 107)	45.8<br>(37.0 to 55.8)	-48.2<br>(-58.4 to -35.1)	-21.9<br>(-30.8 to -12.3)
**Mongolia**	All Ages	3,030<br>(2,580 to 3,480)	140.4<br>(119.7 to 161.3)	572<br>(469 to 694)	17.8<br>(14.6 to 21.5)	523<br>(429 to 625)	15.9<br>(13.1 to 19.0)	420<br>(344 to 510)	12.6<br>(10.3 to 15.3)	-91.0<br>(-93.0 to -88.7)	-29.2<br>(-36.4 to -21.0)
	Under 5	2,770<br>(2,320 to 3,230)	815.5<br>(684.2 to 952.0)	313<br>(228 to 422)	78.8<br>(57.3 to 106.1)	259<br>(184 to 349)	65.7<br>(46.6 to 88.3)	204<br>(143 to 278)	52.2<br>(36.7 to 71.2)	-93.6<br>(-95.6 to -91.0)	-33.8<br>(-44.7 to -22.4)
	5-14 years	60<br>(46 to 74)	10.6<br>(8.1 to 13.3)	24<br>(18 to 30)	3.8<br>(2.9 to 4.8)	33<br>(26 to 42)	5.0<br>(3.8 to 6.3)	24<br>(19 to 30)	3.4<br>(2.7 to 4.3)	-67.6<br>(-76.8 to -54.0)	-9.5<br>(-22.4 to 7.2)
	15-49 years	65<br>(52 to 79)	6.3<br>(5.1 to 7.7)	95<br>(76 to 118)	5.7<br>(4.5 to 7.1)	92<br>(76 to 113)	5.5<br>(4.5 to 6.7)	73<br>(58 to 90)	4.3<br>(3.4 to 5.3)	-32.1<br>(-45.8 to -15.2)	-24.4<br>(-34.5 to -12.7)
	50-69 years	66<br>(51 to 82)	36.7<br>(28.7 to 45.7)	86<br>(67 to 109)	19.4<br>(15.1 to 24.5)	83<br>(66 to 102)	17.9<br>(14.4 to 22.1)	70<br>(55 to 88)	14.7<br>(11.5 to 18.3)	-59.9<br>(-70.9 to -46.0)	-24.2<br>(-35.3 to -11.6)
	70+ years	70<br>(53 to 88)	135.3<br>(102.9 to 171.1)	53<br>(42 to 67)	68.3<br>(53.8 to 86.2)	55<br>(44 to 67)	67.3<br>(53.7 to 83.1)	49<br>(38 to 61)	58.1<br>(44.9 to 72.8)	-57.1<br>(-68.5 to -40.0)	-14.9<br>(-26.4 to -2.6)
**Tajikistan**	All Ages	7,510<br>(6,640 to 8,420)	139.9<br>(123.7 to 156.7)	4,590<br>(3,470 to 5,760)	47.2<br>(35.6 to 59.2)	4,220<br>(3,230 to 5,300)	42.5<br>(32.5 to 53.3)	3,480<br>(2,580 to 4,450)	34.2<br>(25.4 to 43.8)	-75.6<br>(-82.1 to -68.1)	-27.5<br>(-34.4 to -21.0)
	Under 5	6,520<br>(5,690 to 7,400)	689.1<br>(602.0 to 783.0)	3,430<br>(2,450 to 4,520)	260.6<br>(186.1 to 342.7)	3,070<br>(2,190 to 4,050)	231.0<br>(164.6 to 304.3)	2,430<br>(1,640 to 3,320)	181.4<br>(122.5 to 248.3)	-73.7<br>(-82.3 to -63.5)	-30.4<br>(-38.5 to -22.6)
	5-14 years	237<br>(203 to 272)	17.2<br>(14.7 to 19.8)	238<br>(180 to 301)	11.3<br>(8.5 to 14.3)	225<br>(171 to 285)	10.4<br>(7.9 to 13.1)	210<br>(157 to 269)	9.4<br>(7.0 to 12.0)	-45.6<br>(-59.9 to -30.6)	-17.2<br>(-24.8 to -9.0)
	15-49 years	273<br>(236 to 319)	11.3<br>(9.8 to 13.2)	331<br>(255 to 432)	6.7<br>(5.1 to 8.7)	330<br>(252 to 424)	6.5<br>(5.0 to 8.4)	303<br>(229 to 394)	5.9<br>(4.5 to 7.7)	-47.8<br>(-61.0 to -31.2)	-11.3<br>(-21.1 to -0.4)

	50-69 years	225<br>(191 to 264)	44.3<br>(37.6 to 51.9)	280<br>(218 to 347)	24.4<br>(19.1 to 30.3)	290<br>(221 to 364)	24.3<br>(18.5 to 30.5)	269<br>(205 to 338)	21.8<br>(16.6 to 27.4)	-50.8<br>(-64.2 to -34.9)	-10.9<br>(-22.6 to 1.2)
	70+ years	264<br>(217 to 313)	206.0<br>(169.7 to 244.4)	309<br>(237 to 396)	165.9<br>(127.2 to 212.3)	308<br>(237 to 401)	158.9<br>(122.4 to 206.9)	265<br>(203 to 341)	133.3<br>(102.3 to 171.4)	-35.3<br>(-52.9 to -12.2)	-19.7<br>(-28.8 to -9.5)
**Turkmenistan**	All Ages	4,200<br>(3,750 to 4,720)	113.7<br>(101.5 to 127.6)	1,640<br>(1,390 to 1,930)	32.5<br>(27.5 to 38.1)	1,530<br>(1,300 to 1,770)	30.0<br>(25.5 to 34.7)	1,210<br>(1,040 to 1,410)	23.5<br>(20.2 to 27.3)	-79.3<br>(-83.0 to -75.2)	-27.7<br>(-32.4 to -22.5)
	Under 5	3,770<br>(3,340 to 4,280)	644.5<br>(570.0 to 731.2)	1,160<br>(929 to 1,410)	213.4<br>(171.7 to 261.1)	1,050<br>(845 to 1,280)	194.0<br>(156.3 to 235.8)	781<br>(621 to 953)	144.7<br>(115.1 to 176.6)	-77.5<br>(-82.5 to -71.4)	-32.2<br>(-37.6 to -26.6)
	5-14 years	111<br>(99 to 126)	12.2<br>(10.8 to 13.8)	80<br>(66 to 96)	8.3<br>(6.8 to 9.9)	77<br>(64 to 91)	7.9<br>(6.6 to 9.3)	68<br>(57 to 81)	6.9<br>(5.8 to 8.2)	-43.1<br>(-54.0 to -30.0)	-17.0<br>(-23.0 to -10.5)
	15-49 years	123<br>(112 to 134)	7.0<br>(6.4 to 7.6)	176<br>(139 to 219)	6.6<br>(5.2 to 8.3)	172<br>(135 to 218)	6.5<br>(5.1 to 8.2)	153<br>(117 to 202)	5.7<br>(4.4 to 7.6)	-18.1<br>(-38.7 to 8.7)	-13.6<br>(-23.7 to -1.9)
	50-69 years	104<br>(96 to 113)	29.4<br>(27.1 to 31.7)	127<br>(101 to 158)	16.7<br>(13.2 to 20.7)	129<br>(99 to 162)	16.4<br>(12.7 to 20.7)	117<br>(88 to 151)	14.6<br>(11.0 to 18.8)	-50.2<br>(-62.7 to -35.6)	-12.4<br>(-23.5 to 0.1)
	70+ years	92<br>(81 to 103)	108.8<br>(95.8 to 121.8)	106<br>(86 to 130)	70.8<br>(57.0 to 86.6)	105<br>(83 to 131)	68.9<br>(54.8 to 86.1)	92<br>(72 to 116)	58.6<br>(45.9 to 74.1)	-46.1<br>(-59.2 to -30.2)	-17.1<br>(-25.1 to -8.6)
**Uzbekistan**	All Ages	18,600<br>(17,200 to 20,200)	89.0<br>(82.1 to 96.5)	9,260<br>(7,980 to 10,700)	28.0<br>(24.1 to 32.4)	8,740<br>(7,540 to 10,100)	26.0<br>(22.4 to 30.0)	6,980<br>(6,000 to 8,050)	20.4<br>(17.5 to 23.5)	-77.1<br>(-80.7 to -73.1)	-27.2<br>(-31.6 to -22.6)
	Under 5	16,600<br>(15,200 to 18,200)	493.6<br>(451.8 to 539.3)	6,650<br>(5,460 to 8,030)	179.9<br>(147.6 to 217.2)	6,160<br>(5,060 to 7,470)	163.3<br>(133.9 to 197.8)	4,700<br>(3,820 to 5,770)	122.6<br>(99.7 to 150.5)	-75.2<br>(-80.2 to -69.4)	-31.9<br>(-37.5 to -26.0)
	5-14 years	548<br>(499 to 598)	10.6<br>(9.6 to 11.5)	476<br>(415 to 550)	8.1<br>(7.1 to 9.3)	462<br>(404 to 533)	7.6<br>(6.6 to 8.8)	408<br>(355 to 470)	6.5<br>(5.7 to 7.5)	-38.4<br>(-47.8 to -27.3)	-19.4<br>(-24.0 to -14.7)
	15-49 years	627<br>(592 to 661)	6.4<br>(6.1 to 6.8)	839<br>(779 to 897)	4.8<br>(4.4 to 5.1)	823<br>(730 to 916)	4.6<br>(4.1 to 5.2)	720<br>(617 to 837)	4.0<br>(3.5 to 4.7)	-37.0<br>(-46.7 to -25.7)	-15.4<br>(-25.2 to -5.1)
	50-69 years	438<br>(417 to 460)	21.4<br>(20.3 to 22.4)	693<br>(627 to 765)	13.8<br>(12.5 to 15.2)	711<br>(613 to 822)	13.6<br>(11.7 to 15.7)	646<br>(532 to 771)	12.1<br>(9.9 to 14.4)	-43.5<br>(-54.0 to -32.6)	-12.6<br>(-24.3 to -0.7)
	70+ years	380<br>(346 to 410)	66.7<br>(60.6 to 71.9)	594<br>(535 to 655)	65.7<br>(59.1 to 72.4)	577<br>(501 to 658)	64.3<br>(55.7 to 73.2)	500<br>(421 to 584)	52.5<br>(44.1 to 61.2)	-21.3<br>(-33.5 to -7.8)	-20.1<br>(-28.2 to -11.9)
**Albania**	All Ages	1,670<br>(1,450 to 1,870)	50.5<br>(43.9 to 56.5)	558<br>(455 to 717)	20.6<br>(16.8 to 26.5)	522<br>(420 to 671)	19.4<br>(15.6 to 25.0)	438<br>(347 to 570)	16.4<br>(13.0 to 21.4)	-67.5<br>(-75.3 to -55.4)	-20.3<br>(-30.8 to -7.5)
	Under 5	1,070<br>(902 to 1,240)	264.4<br>(223.5 to 308.4)	51<br>(35 to 70)	34.2<br>(23.7 to 47.1)	43<br>(30 to 60)	29.7<br>(20.4 to 40.9)	30<br>(20 to 41)	20.7<br>(14.1 to 28.8)	-92.2<br>(-94.8 to -88.5)	-39.5<br>(-47.2 to -31.7)
	5-14 years	48<br>(40 to 57)	6.7<br>(5.6 to 8.0)	7<br>(6 to 10)	2.4<br>(1.8 to 3.2)	3<br>(2 to 5)	1.1<br>(0.8 to 1.6)	3<br>(2 to 4)	0.9<br>(0.6 to 1.2)	-86.5<br>(-90.8 to -80.8)	-62.0<br>(-67.2 to -56.2)
	15-49 years	75<br>(66 to 87)	4.4<br>(3.8 to 5.1)	36<br>(30 to 44)	2.8<br>(2.3 to 3.4)	27<br>(22 to 34)	2.1<br>(1.7 to 2.7)	23<br>(18 to 29)	1.8<br>(1.4 to 2.3)	-59.1<br>(-69.0 to -47.2)	-36.0<br>(-46.1 to -24.6)

	50-69 years	103 (85 to 122)	27.3 (22.6 to 32.5)	78 (60 to 100)	11.3 (8.7 to 14.5)	69 (52 to 91)	10.0 (7.5 to 13.1)	61 (44 to 81)	8.7 (6.3 to 11.7)	-68.0 (-77.5 to -55.6)	-22.9 (-36.1 to -6.2)
	70+ years	375 (288 to 433)	375.3 (288.6 to 434.2)	385 (306 to 510)	147.5 (117.1 to 195.2)	378 (296 to 504)	142.3 (111.5 to 189.5)	322 (249 to 434)	118.9 (91.8 to 159.9)	-68.3 (-77.0 to -51.4)	-19.4 (-30.5 to -6.1)
**Bosnia and Herzegovina**	All Ages	424 (368 to 481)	9.4 (8.2 to 10.7)	465 (385 to 564)	13.7 (11.4 to 16.6)	447 (367 to 548)	13.3 (11.0 to 16.3)	359 (266 to 454)	10.9 (8.0 to 13.8)	15.4 (-15.4 to 46.4)	-20.8 (-35.5 to -8.4)
	Under 5	49 (37 to 61)	13.9 (10.4 to 17.4)	9 (7 to 12)	5.9 (4.4 to 7.8)	7 (5 to 10)	4.7 (3.4 to 6.2)	5 (3 to 6)	3.0 (2.1 to 4.0)	-78.5 (-85.6 to -67.6)	-49.7 (-61.1 to -36.1)
	5-14 years	11 (9 to 13)	1.4 (1.1 to 1.8)	2 (2 to 3)	0.6 (0.5 to 0.7)	2 (2 to 2)	0.6 (0.5 to 0.7)	2 (1 to 2)	0.5 (0.4 to 0.6)	-67.4 (-76.2 to -56.7)	-22.5 (-33.1 to -8.4)
	15-49 years	50 (42 to 60)	2.1 (1.7 to 2.5)	27 (21 to 33)	1.7 (1.4 to 2.1)	22 (17 to 27)	1.4 (1.1 to 1.8)	17 (11 to 23)	1.2 (0.8 to 1.5)	-44.5 (-64.0 to -22.8)	-32.7 (-52.6 to -17.2)
	50-69 years	104 (86 to 123)	12.4 (10.3 to 14.7)	95 (76 to 117)	9.9 (8.0 to 12.2)	90 (72 to 111)	9.5 (7.6 to 11.8)	74 (52 to 96)	7.9 (5.6 to 10.3)	-36.2 (-56.2 to -13.9)	-20.5 (-38.5 to -3.8)
	70+ years	210 (171 to 248)	125.1 (102.1 to 148.1)	332 (270 to 412)	86.7 (70.5 to 107.5)	326 (262 to 404)	84.2 (67.8 to 104.3)	262 (196 to 339)	67.3 (50.5 to 87.3)	-46.2 (-60.9 to -28.4)	-22.3 (-35.6 to -10.3)
**Bulgaria**	All Ages	3,780 (3,590 to 3,980)	43.6 (41.3 to 45.8)	1,970 (1,840 to 2,130)	28.5 (26.5 to 30.7)	1,860 (1,700 to 2,050)	27.0 (24.7 to 29.9)	1,540 (1,350 to 1,730)	22.7 (19.9 to 25.5)	-47.9 (-54.6 to -40.9)	-20.2 (-28.1 to -12.6)
	Under 5	500 (465 to 537)	93.2 (86.7 to 100.0)	71 (62 to 81)	22.7 (19.9 to 25.7)	58 (50 to 66)	18.7 (16.2 to 21.3)	42 (35 to 50)	14.0 (11.4 to 16.6)	-84.9 (-87.8 to -82.0)	-38.2 (-46.8 to -29.0)
	5-14 years	60 (53 to 65)	5.0 (4.5 to 5.4)	17 (15 to 18)	2.5 (2.2 to 2.7)	13 (11 to 14)	1.9 (1.6 to 2.1)	11 (10 to 13)	1.7 (1.4 to 1.9)	-66.7 (-71.5 to -60.4)	-32.6 (-39.9 to -25.3)
	15-49 years	332 (302 to 362)	8.0 (7.3 to 8.7)	197 (180 to 215)	6.5 (5.9 to 7.1)	175 (156 to 194)	5.9 (5.2 to 6.5)	146 (124 to 169)	5.0 (4.2 to 5.7)	-37.7 (-48.8 to -26.1)	-23.6 (-33.9 to -13.8)
	50-69 years	989 (896 to 1,080)	46.5 (42.1 to 51.0)	493 (454 to 535)	26.2 (24.1 to 28.4)	470 (426 to 519)	25.2 (22.9 to 27.8)	398 (339 to 459)	21.6 (18.4 to 24.9)	-53.6 (-61.3 to -44.9)	-17.5 (-28.9 to -6.9)
	70+ years	1,900 (1,770 to 2,040)	288.2 (267.9 to 309.0)	1,200 (1,100 to 1,300)	116.2 (106.4 to 126.0)	1,140 (1,030 to 1,270)	109.7 (99.0 to 122.1)	943 (831 to 1,060)	91.6 (80.7 to 102.5)	-68.2 (-72.1 to -63.7)	-21.2 (-28.1 to -14.2)
**Croatia**	All Ages	729 (683 to 767)	15.0 (14.0 to 15.8)	956 (871 to 1,030)	22.5 (20.5 to 24.2)	924 (834 to 1,010)	21.8 (19.7 to 23.9)	809 (712 to 887)	19.2 (16.9 to 21.1)	28.2 (13.4 to 41.6)	-14.5 (-21.3 to -8.6)
	Under 5	53 (47 to 59)	17.5 (15.5 to 19.7)	15 (13 to 17)	7.8 (6.7 to 9.1)	12 (10 to 15)	6.7 (5.5 to 8.0)	9 (6 to 11)	4.8 (3.6 to 6.1)	-72.7 (-79.8 to -64.1)	-38.9 (-50.8 to -27.0)
	5-14 years	9 (8 to 10)	1.3 (1.1 to 1.5)	3 (2 to 3)	0.6 (0.5 to 0.7)	2 (1 to 2)	0.4 (0.4 to 0.5)	2 (1 to 2)	0.4 (0.3 to 0.5)	-70.1 (-76.6 to -62.6)	-34.9 (-41.1 to -28.1)
	15-49 years	69 (64 to 75)	2.8 (2.6 to 3.1)	35 (33 to 37)	1.9 (1.8 to 2.0)	29 (27 to 32)	1.6 (1.5 to 1.7)	26 (23 to 29)	1.4 (1.2 to 1.6)	-50.1 (-57.2 to -42.4)	-25.1 (-33.7 to -17.2)

	50-69 years	159 (147 to 170)	13.8 (12.8 to 14.8)	139 (127 to 151)	11.8 (10.8 to 12.7)	128 (115 to 142)	10.9 (9.8 to 12.1)	112 (97 to 127)	9.6 (8.3 to 10.9)	-30.3 (-41.2 to -18.8)	-18.4 (-27.8 to -10.1)
	70+ years	439 (402 to 471)	155.0 (141.9 to 166.3)	764 (685 to 831)	127.9 (114.7 to 139.2)	752 (671 to 828)	123.7 (110.3 to 136.2)	661 (578 to 726)	107.4 (94.0 to 118.2)	-30.7 (-38.4 to -23.1)	-16.0 (-22.3 to -10.3)
**Czechia**	All Ages	2,250 (2,110 to 2,400)	21.9 (20.5 to 23.3)	4,260 (3,820 to 4,600)	40.0 (35.9 to 43.2)	4,190 (3,700 to 4,600)	39.4 (34.8 to 43.2)	3,480 (3,030 to 3,850)	32.7 (28.5 to 36.2)	49.7 (29.5 to 67.9)	-18.2 (-24.2 to -13.1)
	Under 5	173 (159 to 189)	26.7 (24.5 to 29.1)	54 (48 to 59)	9.4 (8.4 to 10.4)	40 (35 to 46)	7.1 (6.2 to 8.0)	28 (22 to 34)	4.9 (3.9 to 6.0)	-81.7 (-85.6 to -77.5)	-47.8 (-56.9 to -38.5)
	5-14 years	27 (25 to 29)	1.7 (1.6 to 1.9)	12 (11 to 13)	1.1 (1.0 to 1.2)	9 (8 to 10)	0.8 (0.7 to 0.9)	8 (7 to 9)	0.7 (0.6 to 0.8)	-58.2 (-63.9 to -52.2)	-32.7 (-38.5 to -26.4)
	15-49 years	192 (179 to 207)	3.7 (3.4 to 4.0)	175 (162 to 189)	3.6 (3.4 to 3.9)	149 (134 to 165)	3.1 (2.8 to 3.5)	129 (111 to 147)	2.7 (2.3 to 3.1)	-25.7 (-37.3 to -13.6)	-24.8 (-33.8 to -17.1)
	50-69 years	406 (385 to 429)	19.5 (18.5 to 20.6)	644 (599 to 691)	24.1 (22.4 to 25.9)	578 (524 to 631)	21.7 (19.7 to 23.7)	526 (454 to 596)	19.8 (17.1 to 22.5)	1.9 (-13.2 to 16.9)	-17.8 (-26.6 to -9.5)
	70+ years	1,450 (1,330 to 1,580)	182.2 (166.7 to 197.7)	3,370 (2,960 to 3,680)	229.7 (201.7 to 250.4)	3,410 (2,980 to 3,770)	226.0 (197.7 to 249.7)	2,790 (2,390 to 3,100)	180.5 (154.6 to 200.6)	-0.9 (-14.8 to 12.1)	-21.4 (-26.8 to -16.9)
**Hungary**	All Ages	1,130 (1,070 to 1,180)	10.8 (10.3 to 11.4)	1,570 (1,440 to 1,700)	16.2 (14.8 to 17.6)	1,480 (1,340 to 1,640)	15.4 (13.8 to 17.0)	1,160 (1,030 to 1,290)	12.1 (10.7 to 13.4)	11.8 (-0.5 to 24.0)	-25.2 (-31.0 to -20.7)
	Under 5	129 (116 to 141)	20.8 (18.8 to 22.8)	24 (21 to 29)	5.3 (4.5 to 6.3)	20 (16 to 24)	4.3 (3.5 to 5.3)	12 (9 to 16)	2.7 (2.0 to 3.5)	-87.2 (-90.6 to -82.9)	-50.1 (-59.8 to -38.9)
	5-14 years	12 (11 to 13)	0.8 (0.7 to 0.9)	5 (5 to 6)	0.6 (0.5 to 0.6)	4 (3 to 4)	0.4 (0.3 to 0.4)	2 (2 to 3)	0.2 (0.2 to 0.3)	-69.1 (-74.4 to -63.4)	-57.1 (-63.2 to -49.8)
	15-49 years	147 (135 to 158)	2.9 (2.7 to 3.1)	86 (79 to 94)	1.9 (1.8 to 2.1)	76 (69 to 84)	1.7 (1.6 to 1.9)	66 (56 to 74)	1.5 (1.3 to 1.7)	-47.6 (-56.3 to -39.2)	-22.2 (-31.7 to -14.3)
	50-69 years	259 (245 to 274)	11.1 (10.5 to 11.8)	316 (294 to 339)	12.4 (11.6 to 13.3)	296 (269 to 322)	11.7 (10.6 to 12.7)	252 (224 to 280)	10.0 (8.9 to 11.1)	-10.0 (-20.5 to 1.2)	-19.5 (-27.0 to -12.8)
	70+ years	582 (530 to 629)	68.6 (62.5 to 74.2)	1,140 (1,010 to 1,260)	87.4 (77.7 to 96.2)	1,090 (954 to 1,220)	81.8 (71.8 to 92.2)	832 (722 to 930)	61.8 (53.6 to 69.0)	-9.9 (-21.1 to 2.7)	-29.3 (-34.5 to -25.3)
**Montenegro**	All Ages	54 (47 to 62)	8.6 (7.4 to 9.9)	72 (60 to 85)	11.6 (9.7 to 13.6)	72 (61 to 85)	11.6 (9.9 to 13.7)	58 (48 to 69)	9.4 (7.8 to 11.2)	9.3 (-12.3 to 35.4)	-19.0 (-25.4 to -12.3)
	Under 5	13 (9 to 17)	23.9 (17.5 to 33.1)	1 (1 to 2)	3.6 (2.3 to 5.1)	1 (1 to 1)	2.7 (1.7 to 3.8)	1 (0 to 1)	1.8 (1.1 to 2.6)	-92.5 (-96.0 to -87.5)	-50.4 (-60.1 to -37.4)
	5-14 years	1 (1 to 2)	1.2 (0.9 to 1.4)	0 (0 to 1)	0.5 (0.4 to 0.7)	0 (0 to 0)	0.2 (0.2 to 0.3)	0 (0 to 0)	0.3 (0.2 to 0.4)	-72.4 (-81.1 to -60.1)	-40.7 (-50.1 to -29.4)
	15-49 years	6 (5 to 7)	1.8 (1.5 to 2.1)	5 (4 to 6)	1.6 (1.3 to 1.9)	4 (3 to 5)	1.4 (1.1 to 1.7)	3 (3 to 4)	1.1 (0.9 to 1.4)	-36.8 (-52.1 to -17.1)	-29.2 (-36.6 to -21.3)

	50-69 years	10 (8 to 13)	9.1 (7.2 to 11.5)	15 (12 to 20)	9.7 (7.6 to 12.6)	15 (12 to 19)	9.5 (7.3 to 12.2)	12 (9 to 16)	7.6 (5.7 to 9.9)	-16.8 (-42.6 to 19.7)	-22.1 (-31.3 to -12.0)
	70+ years	24 (20 to 28)	74.9 (62.6 to 89.0)	51 (42 to 61)	89.1 (73.5 to 107.3)	52 (43 to 63)	89.7 (74.0 to 108.0)	42 (35 to 51)	72.3 (59.4 to 87.5)	-3.4 (-23.6 to 21.4)	-18.8 (-25.1 to -12.2)
**North Macedonia**	All Ages	291 (250 to 342)	14.6 (12.6 to 17.2)	179 (153 to 209)	8.2 (7.0 to 9.6)	163 (137 to 194)	7.5 (6.3 to 8.9)	137 (108 to 168)	6.3 (5.0 to 7.7)	-57.0 (-67.3 to -45.3)	-23.5 (-34.4 to -11.8)
	Under 5	164 (131 to 211)	96.6 (76.9 to 123.8)	6 (4 to 8)	5.5 (3.9 to 7.1)	5 (3 to 6)	4.7 (3.2 to 6.2)	3 (2 to 5)	3.3 (2.2 to 4.5)	-96.5 (-98.1 to -94.8)	-39.5 (-51.1 to -25.5)
	5-14 years	5 (4 to 6)	1.5 (1.2 to 1.8)	2 (1 to 2)	0.7 (0.5 to 0.9)	1 (1 to 2)	0.6 (0.4 to 0.7)	1 (1 to 1)	0.4 (0.3 to 0.5)	-73.6 (-81.1 to -63.2)	-41.2 (-52.5 to -28.1)
	15-49 years	17 (15 to 20)	1.7 (1.4 to 2.0)	12 (9 to 14)	1.0 (0.8 to 1.3)	10 (8 to 12)	0.9 (0.7 to 1.1)	9 (6 to 11)	0.8 (0.6 to 1.0)	-52.7 (-66.2 to -35.3)	-24.4 (-37.3 to -9.8)
	50-69 years	31 (25 to 38)	9.1 (7.4 to 10.9)	38 (30 to 47)	6.9 (5.5 to 8.5)	36 (28 to 46)	6.5 (5.0 to 8.3)	32 (23 to 43)	5.7 (4.2 to 7.6)	-37.2 (-56.6 to -12.3)	-17.8 (-33.5 to -0.1)
	70+ years	73 (61 to 85)	82.4 (68.7 to 96.2)	122 (102 to 144)	67.3 (56.4 to 79.8)	112 (92 to 134)	58.5 (48.2 to 70.5)	92 (73 to 114)	48.4 (38.5 to 59.9)	-41.3 (-55.0 to -24.6)	-28.1 (-37.5 to -18.0)
**Poland**	All Ages	5,880 (5,620 to 6,110)	15.4 (14.7 to 16.0)	17,500 (16,000 to 18,400)	45.6 (41.7 to 47.8)	16,600 (14,900 to 18,400)	43.3 (38.8 to 47.8)	14,700 (13,100 to 16,200)	38.6 (34.2 to 42.3)	150.2 (126.9 to 173.9)	-15.4 (-22.0 to -9.0)
	Under 5	559 (520 to 599)	19.1 (17.8 to 20.5)	96 (89 to 105)	5.0 (4.6 to 5.4)	76 (62 to 91)	3.9 (3.2 to 4.7)	56 (43 to 69)	3.0 (2.3 to 3.7)	-84.6 (-88.1 to -80.7)	-40.5 (-52.0 to -28.6)
	5-14 years	66 (64 to 69)	1.0 (1.0 to 1.0)	38 (36 to 39)	1.0 (0.9 to 1.0)	29 (26 to 31)	0.7 (0.7 to 0.8)	25 (23 to 26)	0.6 (0.6 to 0.7)	-37.8 (-43.2 to -32.9)	-34.6 (-40.1 to -30.6)
	15-49 years	555 (537 to 573)	2.9 (2.8 to 3.0)	958 (930 to 987)	5.3 (5.1 to 5.4)	854 (782 to 930)	4.7 (4.3 to 5.1)	772 (695 to 851)	4.3 (3.9 to 4.7)	47.4 (30.9 to 62.6)	-18.1 (-26.5 to -10.1)
	50-69 years	1,240 (1,190 to 1,280)	17.1 (16.5 to 17.8)	3,840 (3,700 to 4,000)	38.7 (37.2 to 40.2)	3,520 (3,230 to 3,830)	35.7 (32.8 to 38.9)	3,150 (2,810 to 3,480)	32.3 (28.8 to 35.7)	88.3 (67.0 to 109.8)	-16.5 (-25.4 to -8.1)
	70+ years	3,470 (3,230 to 3,650)	144.1 (134.3 to 151.6)	12,600 (11,200 to 13,300)	286.4 (254.0 to 303.7)	12,100 (10,600 to 13,500)	267.3 (234.1 to 297.8)	10,700 (9,360 to 11,900)	230.0 (200.4 to 253.8)	59.7 (44.8 to 74.9)	-19.7 (-25.7 to -13.8)
**Romania**	All Ages	8,710 (8,370 to 9,040)	37.3 (35.8 to 38.6)	8,120 (7,570 to 8,660)	42.2 (39.3 to 44.9)	7,860 (7,100 to 8,550)	41.1 (37.1 to 44.7)	6,780 (6,060 to 7,520)	35.8 (32.0 to 39.7)	-3.9 (-13.9 to 6.8)	-15.1 (-22.0 to -7.6)
	Under 5	3,860 (3,620 to 4,090)	218.4 (204.5 to 230.9)	334 (304 to 366)	34.6 (31.6 to 38.0)	295 (253 to 335)	31.0 (26.6 to 35.2)	200 (171 to 231)	21.4 (18.2 to 24.6)	-90.2 (-91.8 to -88.5)	-38.4 (-44.6 to -32.7)
	5-14 years	203 (184 to 219)	5.3 (4.9 to 5.8)	44 (41 to 48)	2.1 (1.9 to 2.3)	39 (35 to 43)	1.9 (1.7 to 2.1)	33 (30 to 37)	1.6 (1.4 to 1.8)	-69.9 (-73.7 to -65.5)	-23.6 (-28.8 to -18.5)
	15-49 years	878 (819 to 934)	7.7 (7.2 to 8.2)	791 (729 to 855)	9.1 (8.4 to 9.9)	706 (636 to 775)	8.3 (7.5 to 9.1)	604 (531 to 675)	7.3 (6.4 to 8.1)	-6.4 (-18.3 to 6.6)	-20.6 (-28.8 to -13.2)

	50-69 years	1,470 (1,340 to 1,590)	29.1 (26.6 to 31.5)	2,410 (2,180 to 2,690)	47.4 (42.8 to 52.9)	2,340 (2,050 to 2,650)	46.1 (40.3 to 52.1)	2,060 (1,770 to 2,380)	40.7 (35.0 to 47.1)	39.8 (17.6 to 64.2)	-14.2 (-23.9 to -4.8)
	70+ years	2,300 (2,100 to 2,500)	160.9 (147.2 to 175.1)	4,540 (4,100 to 4,950)	185.5 (167.4 to 202.4)	4,470 (3,960 to 5,000)	178.5 (158.2 to 199.5)	3,880 (3,440 to 4,370)	153.3 (136.0 to 172.7)	-4.7 (-16.2 to 8.5)	-17.4 (-23.5 to -9.9)
**Serbia**	All Ages	1,180 (1,030 to 1,360)	12.2 (10.7 to 14.2)	1,390 (1,180 to 1,620)	15.4 (13.0 to 18.0)	1,350 (1,120 to 1,640)	15.0 (12.4 to 18.3)	1,150 (945 to 1,410)	12.9 (10.6 to 15.8)	5.5 (-17.5 to 33.8)	-16.4 (-26.6 to -4.9)
	Under 5	308 (237 to 435)	45.3 (34.8 to 64.0)	17 (13 to 21)	4.4 (3.3 to 5.5)	14 (10 to 17)	3.6 (2.7 to 4.6)	10 (7 to 13)	2.6 (1.9 to 3.4)	-94.3 (-96.7 to -91.5)	-40.6 (-52.3 to -25.4)
	5-14 years	27 (20 to 36)	1.8 (1.4 to 2.4)	4 (3 to 5)	0.4 (0.3 to 0.5)	3 (2 to 4)	0.3 (0.2 to 0.4)	2 (2 to 3)	0.3 (0.2 to 0.4)	-85.8 (-91.1 to -77.7)	-32.9 (-45.6 to -19.8)
	15-49 years	104 (84 to 129)	2.2 (1.8 to 2.7)	66 (54 to 80)	1.6 (1.3 to 1.9)	58 (46 to 74)	1.4 (1.1 to 1.7)	49 (38 to 64)	1.2 (0.9 to 1.5)	-46.9 (-62.4 to -26.8)	-25.3 (-36.3 to -15.1)
	50-69 years	227 (182 to 281)	10.3 (8.3 to 12.8)	278 (223 to 341)	12.1 (9.7 to 14.9)	280 (217 to 351)	12.2 (9.5 to 15.3)	230 (175 to 294)	10.1 (7.7 to 13.0)	-1.9 (-32.1 to 37.7)	-16.5 (-29.7 to -2.5)
	70+ years	508 (413 to 630)	102.3 (83.2 to 126.9)	1,020 (845 to 1,240)	95.4 (78.7 to 115.1)	992 (801 to 1,230)	92.5 (74.7 to 114.8)	857 (683 to 1,060)	79.2 (63.2 to 98.3)	-22.6 (-41.7 to 3.4)	-16.9 (-26.9 to -5.1)
**Slovakia**	All Ages	2,400 (2,050 to 2,690)	45.4 (38.8 to 50.9)	1,840 (1,590 to 2,130)	33.8 (29.2 to 39.1)	1,700 (1,450 to 2,000)	31.3 (26.6 to 36.7)	1,460 (1,220 to 1,740)	27.0 (22.5 to 32.0)	-40.6 (-51.9 to -26.4)	-20.2 (-27.3 to -13.9)
	Under 5	234 (207 to 263)	57.3 (50.5 to 64.2)	58 (47 to 68)	20.1 (16.5 to 23.9)	45 (36 to 54)	15.6 (12.6 to 18.6)	34 (26 to 42)	11.9 (9.0 to 14.8)	-79.3 (-84.6 to -73.3)	-40.8 (-50.1 to -30.9)
	5-14 years	32 (27 to 37)	3.5 (2.9 to 4.0)	9 (7 to 10)	1.5 (1.2 to 1.8)	7 (6 to 9)	1.3 (1.0 to 1.5)	5 (4 to 6)	0.9 (0.7 to 1.1)	-73.9 (-80.4 to -66.1)	-41.2 (-48.6 to -33.7)
	15-49 years	213 (182 to 249)	8.0 (6.8 to 9.3)	112 (95 to 135)	4.3 (3.6 to 5.1)	93 (77 to 114)	3.6 (3.0 to 4.4)	80 (65 to 100)	3.1 (2.5 to 3.9)	-61.1 (-70.6 to -50.0)	-27.3 (-36.4 to -19.6)
	50-69 years	523 (431 to 618)	54.7 (45.1 to 64.7)	364 (290 to 447)	26.1 (20.8 to 32.1)	324 (255 to 404)	23.3 (18.3 to 29.1)	292 (221 to 373)	21.1 (16.0 to 26.9)	-61.4 (-72.0 to -47.2)	-19.3 (-29.8 to -9.3)
	70+ years	1,400 (1,120 to 1,650)	422.4 (337.3 to 498.2)	1,300 (1,090 to 1,520)	230.3 (194.2 to 271.0)	1,230 (1,030 to 1,470)	211.3 (177.2 to 252.3)	1,050 (865 to 1,250)	175.0 (143.9 to 207.2)	-58.6 (-67.1 to -46.2)	-24.0 (-30.2 to -17.6)
**Slovenia**	All Ages	493 (457 to 526)	25.0 (23.1 to 26.6)	557 (486 to 613)	26.8 (23.5 to 29.5)	564 (487 to 627)	27.2 (23.5 to 30.3)	439 (376 to 495)	21.2 (18.2 to 23.9)	-15.1 (-25.9 to -4.0)	-21.0 (-27.5 to -14.7)
	Under 5	31 (27 to 34)	25.0 (22.3 to 27.6)	8 (7 to 9)	7.9 (6.8 to 9.1)	7 (6 to 8)	6.7 (5.6 to 7.8)	4 (4 to 5)	4.5 (3.7 to 5.3)	-82.0 (-85.3 to -78.3)	-43.4 (-48.8 to -37.4)
	5-14 years	5 (5 to 6)	1.8 (1.6 to 2.0)	2 (2 to 2)	1.0 (0.9 to 1.1)	1 (1 to 2)	0.6 (0.6 to 0.7)	1 (1 to 1)	0.6 (0.5 to 0.7)	-68.5 (-73.8 to -61.8)	-44.2 (-49.4 to -37.9)
	15-49 years	41 (37 to 45)	4.0 (3.7 to 4.5)	25 (23 to 28)	2.8 (2.6 to 3.1)	21 (19 to 24)	2.4 (2.1 to 2.7)	18 (15 to 21)	2.0 (1.7 to 2.4)	-49.2 (-58.5 to -39.3)	-27.8 (-38.2 to -19.0)

	50-69 years	89 (81 to 98)	21.8 (19.8 to 24.1)	80 (70 to 90)	13.8 (12.1 to 15.6)	69 (60 to 80)	11.9 (10.3 to 13.7)	61 (50 to 72)	10.5 (8.7 to 12.4)	-51.9 (-60.6 to -42.5)	-23.8 (-32.9 to -14.9)
	70+ years	327 (295 to 353)	238.4 (215.7 to 258.1)	441 (373 to 489)	154.0 (130.4 to 170.7)	465 (397 to 520)	158.8 (135.7 to 177.6)	354 (298 to 400)	117.9 (99.3 to 133.2)	-50.5 (-57.1 to -43.6)	-23.4 (-29.3 to -17.8)
**Belarus**	All Ages	871 (803 to 937)	8.3 (7.7 to 9.0)	976 (883 to 1,080)	10.3 (9.3 to 11.4)	884 (761 to 1,030)	9.4 (8.1 to 11.0)	889 (712 to 1,100)	9.5 (7.6 to 11.8)	14.3 (-7.1 to 40.2)	-7.7 (-24.4 to 10.7)
	Under 5	323 (273 to 380)	40.1 (33.9 to 47.1)	32 (25 to 40)	6.1 (4.8 to 7.7)	21 (16 to 28)	4.3 (3.2 to 5.7)	20 (14 to 26)	4.2 (3.1 to 5.6)	-89.5 (-92.5 to -85.2)	-31.2 (-43.5 to -16.7)
	5-14 years	14 (12 to 16)	0.9 (0.8 to 1.0)	4 (3 to 5)	0.4 (0.3 to 0.4)	4 (3 to 4)	0.3 (0.3 to 0.4)	3 (3 to 4)	0.3 (0.3 to 0.4)	-66.2 (-72.1 to -58.0)	-18.2 (-28.0 to -7.5)
	15-49 years	137 (121 to 154)	2.7 (2.4 to 3.1)	278 (240 to 320)	6.4 (5.5 to 7.3)	257 (212 to 312)	6.0 (4.9 to 7.2)	267 (208 to 342)	6.3 (4.9 to 8.0)	131.1 (76.9 to 206.5)	-1.5 (-19.9 to 19.2)
	50-69 years	212 (192 to 235)	9.2 (8.3 to 10.2)	480 (426 to 541)	18.7 (16.6 to 21.1)	439 (367 to 521)	17.2 (14.4 to 20.4)	435 (335 to 551)	17.1 (13.2 to 21.7)	85.8 (42.8 to 139.1)	-8.3 (-26.9 to 12.1)
	70+ years	184 (163 to 203)	26.5 (23.5 to 29.2)	182 (163 to 203)	19.5 (17.4 to 21.6)	163 (141 to 187)	17.2 (14.8 to 19.6)	163 (133 to 197)	17.1 (14.0 to 20.7)	-35.4 (-47.5 to -20.9)	-12.2 (-25.4 to 2.2)
**Estonia**	All Ages	162 (153 to 171)	10.3 (9.8 to 10.9)	214 (194 to 233)	16.3 (14.7 to 17.7)	189 (168 to 207)	14.4 (12.8 to 15.8)	181 (157 to 204)	13.8 (12.0 to 15.5)	33.8 (16.2 to 51.3)	-14.9 (-22.6 to -6.3)
	Under 5	37 (34 to 41)	31.0 (27.9 to 34.4)	4 (3 to 5)	5.5 (4.7 to 6.5)	3 (2 to 3)	3.8 (3.2 to 4.5)	2 (2 to 3)	3.3 (2.8 to 4.0)	-89.3 (-91.4 to -86.9)	-39.9 (-46.1 to -34.1)
	5-14 years	3 (3 to 3)	1.2 (1.1 to 1.3)	1 (1 to 1)	0.6 (0.5 to 0.7)	1 (0 to 1)	0.4 (0.3 to 0.4)	0 (0 to 0)	0.2 (0.2 to 0.3)	-81.3 (-84.4 to -77.8)	-58.4 (-66.0 to -50.9)
	15-49 years	36 (33 to 39)	4.8 (4.4 to 5.2)	20 (18 to 22)	3.4 (3.1 to 3.8)	18 (16 to 20)	3.1 (2.7 to 3.4)	17 (15 to 20)	3.0 (2.6 to 3.5)	-36.3 (-46.6 to -25.2)	-11.2 (-21.3 to -1.0)
	50-69 years	53 (48 to 57)	15.4 (14.2 to 16.8)	58 (53 to 64)	17.6 (15.9 to 19.3)	55 (49 to 60)	16.5 (14.7 to 18.3)	49 (42 to 55)	14.8 (12.7 to 16.8)	-3.8 (-19.0 to 12.5)	-15.4 (-25.2 to -5.6)
	70+ years	33 (30 to 37)	27.9 (25.1 to 30.9)	131 (114 to 146)	70.8 (61.3 to 79.1)	113 (96 to 128)	59.8 (51.0 to 67.4)	112 (95 to 128)	58.7 (49.6 to 67.0)	110.6 (78.3 to 142.9)	-17.0 (-24.1 to -8.4)
**Latvia**	All Ages	279 (265 to 293)	10.5 (10.0 to 11.0)	351 (324 to 377)	18.3 (16.9 to 19.7)	294 (265 to 326)	15.5 (14.0 to 17.2)	278 (247 to 308)	14.9 (13.2 to 16.5)	41.6 (25.9 to 58.8)	-19.0 (-26.0 to -11.7)
	Under 5	52 (48 to 56)	25.5 (23.5 to 27.5)	8 (7 to 9)	7.7 (6.7 to 8.7)	5 (4 to 6)	5.3 (4.4 to 6.2)	4 (3 to 5)	4.4 (3.6 to 5.3)	-82.6 (-85.9 to -79.1)	-42.4 (-49.1 to -34.9)
	5-14 years	4 (4 to 5)	1.2 (1.1 to 1.3)	1 (1 to 1)	0.5 (0.5 to 0.6)	1 (1 to 1)	0.3 (0.3 to 0.4)	0 (0 to 0)	0.2 (0.1 to 0.2)	-86.0 (-88.6 to -82.6)	-67.4 (-71.6 to -63.3)
	15-49 years	62 (58 to 68)	4.9 (4.5 to 5.3)	49 (44 to 54)	6.0 (5.3 to 6.6)	39 (34 to 45)	4.9 (4.3 to 5.6)	40 (34 to 46)	5.1 (4.4 to 5.8)	4.6 (-11.2 to 22.5)	-14.6 (-23.2 to -5.5)

	50-69 years	93<br>(85 to 100)	15.5<br>(14.3 to 16.8)	110<br>(101 to 120)	21.6<br>(19.8 to 23.5)	94<br>(84 to 105)	18.5<br>(16.7 to 20.7)	92<br>(80 to 104)	18.3<br>(15.8 to 20.7)	17.8<br>(-0.1 to 35.3)	-15.3<br>(-24.3 to -6.5)
	70+ years	68<br>(62 to 73)	32.7<br>(29.9 to 35.4)	183<br>(163 to 200)	65.2<br>(58.2 to 71.1)	155<br>(136 to 174)	54.8<br>(48.0 to 61.6)	141<br>(122 to 158)	50.2<br>(43.4 to 56.0)	53.4<br>(36.0 to 73.0)	-23.0<br>(-29.0 to -16.4)
**Lithuania**	All Ages	239<br>(226 to 254)	6.5<br>(6.2 to 6.9)	470<br>(434 to 504)	16.8<br>(15.5 to 18.0)	437<br>(391 to 487)	15.8<br>(14.2 to 17.6)	419<br>(366 to 477)	15.4<br>(13.4 to 17.5)	135.9<br>(103.1 to 170.3)	-8.8<br>(-18.4 to 2.6)
	Under 5	57<br>(51 to 62)	19.6<br>(17.7 to 21.5)	9<br>(8 to 10)	6.3<br>(5.5 to 7.2)	6<br>(5 to 8)	4.7<br>(3.9 to 5.8)	6<br>(5 to 7)	4.2<br>(3.5 to 5.3)	-78.4<br>(-82.6 to -72.8)	-32.9<br>(-42.5 to -19.4)
	5-14 years	5<br>(4 to 5)	0.9<br>(0.8 to 1.0)	1<br>(1 to 1)	0.4<br>(0.4 to 0.5)	1<br>(1 to 1)	0.3<br>(0.3 to 0.4)	1<br>(1 to 1)	0.3<br>(0.3 to 0.4)	-61.1<br>(-68.0 to -52.3)	-20.1<br>(-28.7 to -10.4)
	15-49 years	62<br>(58 to 66)	3.4<br>(3.2 to 3.6)	65<br>(60 to 71)	5.4<br>(4.9 to 5.8)	60<br>(53 to 66)	5.0<br>(4.5 to 5.6)	57<br>(50 to 65)	4.9<br>(4.3 to 5.6)	45.8<br>(24.2 to 69.3)	-8.0<br>(-18.6 to 3.7)
	50-69 years	71<br>(64 to 79)	9.3<br>(8.4 to 10.4)	159<br>(143 to 176)	20.8<br>(18.7 to 23.0)	152<br>(132 to 172)	19.8<br>(17.3 to 22.5)	144<br>(121 to 166)	18.8<br>(15.9 to 21.8)	102.2<br>(65.6 to 140.9)	-9.4<br>(-20.3 to 2.3)
	70+ years	45<br>(41 to 49)	17.8<br>(16.3 to 19.4)	236<br>(210 to 255)	59.0<br>(52.5 to 63.7)	219<br>(192 to 247)	54.3<br>(47.6 to 61.4)	212<br>(181 to 244)	52.7<br>(45.2 to 60.7)	196.5<br>(156.6 to 245.3)	-10.5<br>(-19.8 to 0.6)
**Republic of Moldova**	All Ages	1,060<br>(987 to 1,150)	23.9<br>(22.2 to 25.9)	868<br>(777 to 962)	23.8<br>(21.3 to 26.4)	817<br>(743 to 891)	22.5<br>(20.5 to 24.6)	717<br>(636 to 807)	20.0<br>(17.7 to 22.5)	-16.5<br>(-28.1 to -4.1)	-16.0<br>(-23.5 to -7.8)
	Under 5	572<br>(501 to 656)	132.7<br>(116.2 to 152.3)	60<br>(47 to 77)	36.0<br>(28.1 to 46.2)	52<br>(40 to 67)	32.2<br>(24.9 to 41.7)	36<br>(27 to 48)	23.3<br>(17.4 to 31.3)	-82.5<br>(-87.2 to -76.0)	-35.5<br>(-42.8 to -28.0)
	5-14 years	19<br>(16 to 21)	2.3<br>(2.0 to 2.6)	8<br>(6 to 9)	2.0<br>(1.7 to 2.4)	6<br>(5 to 7)	1.5<br>(1.3 to 1.8)	5<br>(4 to 5)	1.2<br>(1.0 to 1.4)	-46.6<br>(-56.1 to -33.1)	-39.2<br>(-44.4 to -33.7)
	15-49 years	164<br>(152 to 178)	7.5<br>(6.9 to 8.1)	215<br>(190 to 240)	11.7<br>(10.4 to 13.1)	191<br>(173 to 210)	10.6<br>(9.5 to 11.6)	171<br>(152 to 193)	9.6<br>(8.5 to 10.8)	27.9<br>(10.2 to 47.7)	-18.5<br>(-26.7 to -9.4)
	50-69 years	215<br>(198 to 232)	26.7<br>(24.6 to 28.9)	413<br>(362 to 468)	43.2<br>(37.9 to 49.0)	394<br>(352 to 441)	41.7<br>(37.3 to 46.7)	346<br>(301 to 397)	37.1<br>(32.2 to 42.5)	39.1<br>(18.7 to 62.4)	-14.1<br>(-22.9 to -4.1)
	70+ years	94<br>(86 to 102)	44.8<br>(40.8 to 48.7)	172<br>(152 to 192)	53.2<br>(47.0 to 59.4)	174<br>(152 to 194)	51.5<br>(45.0 to 57.4)	160<br>(140 to 183)	45.9<br>(40.1 to 52.4)	2.4<br>(-11.9 to 19.5)	-13.8<br>(-21.2 to -5.7)
**Russian Federation**	All Ages	16,700<br>(16,400 to 17,000)	11.1<br>(10.9 to 11.2)	26,100<br>(25,200 to 26,700)	17.9<br>(17.2 to 18.3)	24,900<br>(23,600 to 26,200)	17.1<br>(16.2 to 18.0)	22,100<br>(20,100 to 24,000)	15.3<br>(13.9 to 16.6)	37.9<br>(25.5 to 49.8)	-14.5<br>(-21.9 to -7.5)
	Under 5	6,460<br>(6,250 to 6,660)	55.5<br>(53.8 to 57.3)	727<br>(683 to 774)	8.5<br>(8.0 to 9.1)	634<br>(581 to 691)	7.9<br>(7.2 to 8.6)	518<br>(453 to 573)	6.8<br>(5.9 to 7.5)	-87.7<br>(-89.3 to -86.4)	-20.1<br>(-27.6 to -13.4)
	5-14 years	368<br>(361 to 375)	1.6<br>(1.6 to 1.6)	158<br>(155 to 162)	0.9<br>(0.9 to 0.9)	145<br>(139 to 151)	0.8<br>(0.8 to 0.8)	126<br>(118 to 133)	0.7<br>(0.6 to 0.7)	-57.2<br>(-60.0 to -54.5)	-23.3<br>(-27.8 to -19.2)
	15-49 years	2,620<br>(2,570 to 2,670)	3.5<br>(3.5 to 3.6)	6,260<br>(6,170 to 6,360)	9.2<br>(9.0 to 9.3)	5,990<br>(5,690 to 6,270)	8.8<br>(8.4 to 9.2)	5,400<br>(4,920 to 5,850)	8.0<br>(7.3 to 8.7)	127.3<br>(106.8 to 146.7)	-12.5<br>(-20.0 to -5.6)

	50-69 years	4,390<br>(4,300 to 4,480)	13.5<br>(13.3 to 13.8)	10,100<br>(9,890 to 10,300)	26.8<br>(26.3 to 27.4)	9,560<br>(9,030 to 10,100)	25.6<br>(24.2 to 27.0)	8,450<br>(7,550 to 9,350)	22.8<br>(20.4 to 25.2)	68.6<br>(49.9 to 86.3)	-15.0<br>(-24.1 to -6.3)
	70+ years	2,900<br>(2,750 to 3,000)	30.0<br>(28.5 to 31.0)	8,840<br>(8,120 to 9,220)	64.9<br>(59.6 to 67.7)	8,600<br>(7,880 to 9,160)	61.0<br>(55.9 to 65.0)	7,630<br>(6,900 to 8,320)	53.4<br>(48.3 to 58.1)	77.7<br>(62.9 to 92.4)	-17.8<br>(-24.1 to -11.9)
**Ukraine**	All Ages	4,450<br>(4,180 to 4,750)	8.4<br>(7.9 to 9.0)	6,300<br>(5,220 to 7,530)	14.4<br>(11.9 to 17.2)	5,830<br>(4,780 to 7,090)	13.4<br>(11.0 to 16.3)	5,160<br>(3,560 to 7,020)	12.0<br>(8.3 to 16.3)	42.0<br>(-3.2 to 93.5)	-16.6<br>(-45.8 to 17.6)
	Under 5	1,360<br>(1,170 to 1,570)	36.1<br>(31.2 to 41.6)	206<br>(177 to 236)	11.2<br>(9.6 to 12.8)	176<br>(147 to 206)	10.3<br>(8.6 to 12.1)	121<br>(95 to 149)	7.6<br>(5.9 to 9.3)	-78.9<br>(-84.2 to -72.5)	-32.0<br>(-47.7 to -20.6)
	5-14 years	87<br>(78 to 97)	1.1<br>(1.0 to 1.3)	41<br>(37 to 46)	0.9<br>(0.8 to 1.0)	39<br>(34 to 45)	0.8<br>(0.7 to 0.9)	32<br>(28 to 37)	0.7<br>(0.6 to 0.8)	-40.6<br>(-51.2 to -29.1)	-20.3<br>(-30.9 to -9.6)
	15-49 years	929<br>(853 to 1,010)	3.7<br>(3.4 to 4.0)	2,520<br>(2,030 to 3,060)	12.2<br>(9.8 to 14.8)	2,370<br>(1,890 to 2,960)	11.6<br>(9.2 to 14.5)	2,130<br>(1,420 to 2,900)	10.5<br>(7.0 to 14.3)	183.2<br>(86.0 to 287.7)	-13.4<br>(-45.6 to 23.1)
	50-69 years	1,250<br>(1,130 to 1,380)	10.1<br>(9.1 to 11.1)	2,520<br>(1,990 to 3,070)	21.4<br>(17.0 to 26.1)	2,300<br>(1,830 to 2,910)	19.7<br>(15.6 to 24.9)	2,050<br>(1,340 to 2,910)	17.7<br>(11.5 to 25.0)	74.9<br>(14.2 to 153.5)	-17.6<br>(-48.7 to 20.8)
	70+ years	822<br>(761 to 879)	20.5<br>(19.0 to 21.9)	1,020<br>(862 to 1,170)	21.4<br>(18.2 to 24.6)	937<br>(792 to 1,090)	19.4<br>(16.4 to 22.6)	823<br>(624 to 1,070)	16.9<br>(12.8 to 21.9)	-17.7<br>(-37.7 to 5.6)	-21.2<br>(-41.7 to 3.6)
**Australia**	All Ages	1,830<br>(1,660 to 1,960)	10.9<br>(9.9 to 11.6)	4,520<br>(3,720 to 5,060)	18.1<br>(14.9 to 20.3)	3,360<br>(2,740 to 3,770)	13.2<br>(10.8 to 14.8)	3,530<br>(2,890 to 3,950)	13.7<br>(11.2 to 15.3)	25.9<br>(9.9 to 39.3)	-24.3<br>(-26.4 to -22.3)
	Under 5	73<br>(67 to 80)	5.8<br>(5.3 to 6.4)	33<br>(30 to 37)	2.2<br>(2.0 to 2.4)	20<br>(17 to 22)	1.3<br>(1.1 to 1.5)	13<br>(10 to 16)	0.9<br>(0.7 to 1.1)	-85.3<br>(-88.5 to -81.3)	-60.9<br>(-68.3 to -53.0)
	5-14 years	7<br>(7 to 8)	0.3<br>(0.3 to 0.3)	5<br>(4 to 5)	0.1<br>(0.1 to 0.2)	3<br>(3 to 3)	0.1<br>(0.1 to 0.1)	3<br>(3 to 4)	0.1<br>(0.1 to 0.1)	-64.3<br>(-69.2 to -58.6)	-28.3<br>(-33.5 to -22.9)
	15-49 years	85<br>(79 to 91)	0.9<br>(0.9 to 1.0)	85<br>(77 to 93)	0.7<br>(0.7 to 0.8)	63<br>(57 to 69)	0.5<br>(0.5 to 0.6)	63<br>(57 to 69)	0.5<br>(0.5 to 0.6)	-45.0<br>(-51.6 to -38.1)	-27.9<br>(-30.5 to -25.4)
	50-69 years	235<br>(214 to 255)	8.2<br>(7.5 to 8.9)	276<br>(250 to 302)	4.8<br>(4.4 to 5.3)	217<br>(197 to 238)	3.7<br>(3.4 to 4.1)	221<br>(201 to 243)	3.7<br>(3.4 to 4.1)	-54.3<br>(-59.8 to -48.2)	-22.9<br>(-25.3 to -20.1)
	70+ years	1,430<br>(1,260 to 1,550)	118.4<br>(104.2 to 128.4)	4,120<br>(3,340 to 4,640)	145.0<br>(117.6 to 163.4)	3,050<br>(2,450 to 3,450)	103.2<br>(82.8 to 116.5)	3,230<br>(2,590 to 3,640)	104.8<br>(84.1 to 118.0)	-11.5<br>(-23.1 to -1.7)	-27.7<br>(-29.9 to -25.7)
**New Zealand**	All Ages	959<br>(846 to 1,040)	28.1<br>(24.7 to 30.4)	1,000<br>(820 to 1,120)	20.1<br>(16.4 to 22.4)	746<br>(605 to 840)	14.6<br>(11.9 to 16.5)	802<br>(649 to 898)	15.5<br>(12.6 to 17.4)	-44.7<br>(-50.8 to -38.8)	-22.7<br>(-25.0 to -20.5)
	Under 5	41<br>(37 to 45)	14.6<br>(13.3 to 16.0)	11<br>(10 to 13)	3.6<br>(3.3 to 4.1)	6<br>(5 to 7)	1.9<br>(1.6 to 2.1)	7<br>(5 to 8)	2.1<br>(1.7 to 2.4)	-85.8<br>(-88.2 to -83.1)	-42.9<br>(-50.3 to -34.7)
	5-14 years	3<br>(3 to 3)	0.6<br>(0.5 to 0.6)	1<br>(1 to 1)	0.1<br>(0.1 to 0.2)	1<br>(1 to 1)	0.1<br>(0.1 to 0.1)	1<br>(1 to 1)	0.1<br>(0.1 to 0.1)	-83.6<br>(-85.8 to -80.9)	-36.0<br>(-41.7 to -30.4)
	15-49 years	20<br>(18 to 21)	1.1<br>(1.0 to 1.2)	10<br>(9 to 10)	0.4<br>(0.4 to 0.4)	7<br>(7 to 8)	0.3<br>(0.3 to 0.3)	7<br>(7 to 8)	0.3<br>(0.3 to 0.3)	-72.5<br>(-75.3 to -69.3)	-26.6<br>(-29.3 to -23.7)

	50-69 years	65<br>(60 to 71)	11.5<br>(10.5 to 12.5)	43<br>(39 to 46)	3.7<br>(3.4 to 4.0)	35<br>(32 to 39)	3.0<br>(2.7 to 3.3)	35<br>(32 to 39)	3.0<br>(2.7 to 3.2)	-74.3<br>(-77.3 to -71.0)	-19.5<br>(-22.6 to -16.2)
	70+ years	830<br>(715 to 909)	335.6<br>(289.1 to 367.3)	937<br>(758 to 1,050)	180.4<br>(146.0 to 202.4)	696<br>(558 to 787)	129.1<br>(103.4 to 146.0)	752<br>(602 to 846)	134.4<br>(107.7 to 151.2)	-59.9<br>(-64.4 to -55.5)	-25.5<br>(-27.7 to -23.2)
**Brunei Darussalam**	All Ages	45<br>(40 to 51)	17.4<br>(15.4 to 19.8)	69<br>(60 to 78)	15.6<br>(13.6 to 17.7)	61<br>(52 to 71)	13.6<br>(11.6 to 15.9)	57<br>(49 to 66)	12.7<br>(10.9 to 14.6)	-26.8<br>(-38.2 to -15.1)	-18.2<br>(-25.7 to -11.5)
	Under 5	5<br>(4 to 6)	13.8<br>(10.9 to 17.1)	3<br>(2 to 3)	8.3<br>(6.6 to 10.6)	2<br>(2 to 3)	6.6<br>(5.0 to 8.6)	2<br>(1 to 2)	5.3<br>(3.8 to 7.2)	-61.6<br>(-72.3 to -45.5)	-36.4<br>(-46.1 to -25.8)
	5-14 years	1<br>(1 to 2)	2.5<br>(2.0 to 3.1)	1<br>(1 to 1)	1.2<br>(0.9 to 1.5)	0<br>(0 to 1)	0.6<br>(0.5 to 0.8)	0<br>(0 to 0)	0.6<br>(0.4 to 0.7)	-77.6<br>(-83.7 to -70.7)	-53.6<br>(-60.4 to -45.2)
	15-49 years	6<br>(5 to 7)	4.3<br>(3.6 to 5.1)	7<br>(6 to 8)	2.5<br>(2.1 to 2.9)	6<br>(5 to 8)	2.4<br>(1.9 to 2.9)	6<br>(5 to 7)	2.0<br>(1.7 to 2.4)	-53.1<br>(-62.2 to -40.8)	-17.9<br>(-26.9 to -10.2)
	50-69 years	7<br>(6 to 9)	41.0<br>(32.8 to 50.4)	15<br>(12 to 18)	22.2<br>(18.3 to 26.7)	14<br>(11 to 17)	19.9<br>(16.1 to 24.5)	13<br>(10 to 16)	18.2<br>(14.6 to 22.2)	-55.5<br>(-65.6 to -42.5)	-17.9<br>(-29.9 to -7.5)
	70+ years	25<br>(21 to 30)	570.5<br>(481.3 to 671.3)	44<br>(38 to 51)	368.7<br>(314.9 to 426.8)	38<br>(32 to 45)	297.4<br>(248.5 to 353.8)	37<br>(31 to 43)	270.9<br>(227.1 to 318.5)	-52.5<br>(-61.6 to -42.4)	-26.5<br>(-32.9 to -20.5)
**Japan**	All Ages	61,000<br>(54,600 to 64,300)	48.5<br>(43.4 to 51.1)	93,900<br>(75,100 to 104,000)	73.1<br>(58.5 to 81.0)	83,300<br>(67,000 to 93,100)	65.0<br>(52.3 to 72.7)	79,600<br>(63,300 to 88,400)	62.3<br>(49.6 to 69.3)	28.5<br>(12.5 to 37.6)	-14.8<br>(-16.0 to -13.6)
	Under 5	433<br>(411 to 454)	6.5<br>(6.2 to 6.8)	104<br>(97 to 111)	2.1<br>(2.0 to 2.3)	68<br>(60 to 78)	1.4<br>(1.3 to 1.6)	49<br>(44 to 55)	1.1<br>(0.9 to 1.2)	-83.5<br>(-85.7 to -81.1)	-50.1<br>(-55.4 to -44.9)
	5-14 years	148<br>(144 to 153)	0.9<br>(0.9 to 0.9)	38<br>(36 to 40)	0.3<br>(0.3 to 0.4)	30<br>(28 to 32)	0.3<br>(0.3 to 0.3)	29<br>(28 to 30)	0.3<br>(0.3 to 0.3)	-70.3<br>(-71.8 to -68.7)	-22.3<br>(-24.8 to -19.8)
	15-49 years	1,170<br>(1,150 to 1,200)	1.8<br>(1.8 to 1.8)	491<br>(478 to 504)	0.9<br>(0.9 to 1.0)	445<br>(426 to 468)	0.9<br>(0.8 to 0.9)	385<br>(373 to 397)	0.8<br>(0.7 to 0.8)	-58.0<br>(-59.6 to -56.3)	-19.2<br>(-20.7 to -17.9)
	50-69 years	6,970<br>(6,710 to 7,210)	25.0<br>(24.0 to 25.8)	4,730<br>(4,510 to 4,920)	14.4<br>(13.7 to 15.0)	4,060<br>(3,860 to 4,280)	12.4<br>(11.8 to 13.1)	3,620<br>(3,440 to 3,780)	11.1<br>(10.5 to 11.6)	-55.6<br>(-57.7 to -53.4)	-23.1<br>(-24.5 to -21.6)
	70+ years	52,300<br>(46,200 to 55,300)	529.4<br>(467.6 to 560.1)	88,500<br>(70,000 to 98,500)	321.5<br>(254.3 to 358.0)	78,700<br>(62,400 to 88,300)	278.1<br>(220.4 to 312.2)	75,500<br>(59,300 to 84,300)	261.2<br>(205.3 to 291.5)	-50.7<br>(-56.7 to -47.3)	-18.8<br>(-19.9 to -17.7)
**Republic of Korea**	All Ages	5,520<br>(4,870 to 6,530)	12.5<br>(11.0 to 14.8)	15,700<br>(12,500 to 18,700)	30.3<br>(24.1 to 36.0)	14,600<br>(11,700 to 17,400)	28.2<br>(22.6 to 33.6)	14,100<br>(11,100 to 16,700)	27.4<br>(21.6 to 32.5)	119.5<br>(68.7 to 162.1)	-9.7<br>(-13.8 to -5.8)
	Under 5	538<br>(413 to 682)	16.2<br>(12.4 to 20.5)	23<br>(18 to 27)	1.2<br>(1.0 to 1.5)	16<br>(13 to 20)	1.0<br>(0.8 to 1.2)	10<br>(7 to 13)	0.7<br>(0.5 to 0.9)	-96.0<br>(-97.3 to -94.0)	-47.3<br>(-59.0 to -34.9)
	5-14 years	247<br>(185 to 311)	3.1<br>(2.3 to 3.9)	12<br>(9 to 15)	0.3<br>(0.2 to 0.3)	10<br>(8 to 13)	0.2<br>(0.2 to 0.3)	9<br>(7 to 11)	0.2<br>(0.2 to 0.3)	-93.5<br>(-95.4 to -90.1)	-20.9<br>(-28.0 to -13.3)
	15-49 years	548<br>(468 to 615)	2.1<br>(1.8 to 2.4)	206<br>(175 to 249)	0.8<br>(0.7 to 1.0)	183<br>(155 to 221)	0.7<br>(0.6 to 0.9)	162<br>(135 to 195)	0.7<br>(0.6 to 0.8)	-68.6<br>(-74.4 to -60.8)	-18.1<br>(-23.5 to -12.9)

	50-69 years	954 (815 to 1,140)	16.6 (14.2 to 19.8)	1,350 (1,120 to 1,610)	9.1 (7.5 to 10.9)	1,230 (1,010 to 1,500)	8.1 (6.6 to 9.8)	1,200 (974 to 1,460)	7.6 (6.2 to 9.3)	-53.9 (-65.9 to -40.6)	-15.7 (-22.6 to -8.5)
	70+ years	3,230 (2,700 to 4,040)	254.4 (212.5 to 317.9)	14,100 (10,900 to 17,000)	271.6 (208.9 to 326.6)	13,200 (10,300 to 15,800)	244.1 (190.8 to 293.7)	12,700 (9,830 to 15,300)	229.9 (177.4 to 276.5)	-9.6 (-33.3 to 11.2)	-15.4 (-19.5 to -11.5)
**Singapore**	All Ages	1,330 (1,240 to 1,400)	43.5 (40.7 to 45.8)	3,480 (2,960 to 3,790)	61.4 (52.3 to 66.9)	3,190 (2,690 to 3,500)	56.0 (47.2 to 61.5)	3,100 (2,620 to 3,390)	54.1 (45.7 to 59.1)	24.4 (10.4 to 34.9)	-11.9 (-14.1 to -9.7)
	Under 5	59 (53 to 65)	29.0 (26.2 to 31.7)	12 (10 to 13)	4.0 (3.4 to 4.6)	7 (6 to 9)	2.6 (2.2 to 3.0)	7 (5 to 8)	2.3 (1.9 to 2.7)	-92.1 (-93.7 to -90.5)	-42.2 (-49.8 to -34.8)
	5-14 years	18 (17 to 20)	4.1 (3.8 to 4.4)	6 (5 to 7)	1.2 (1.1 to 1.3)	5 (4 to 5)	0.9 (0.8 to 1.1)	4 (4 to 5)	0.8 (0.7 to 0.9)	-80.8 (-83.2 to -78.0)	-34.7 (-39.9 to -29.0)
	15-49 years	112 (105 to 119)	5.9 (5.5 to 6.2)	53 (49 to 57)	1.7 (1.6 to 1.8)	49 (45 to 53)	1.6 (1.5 to 1.7)	42 (39 to 46)	1.4 (1.3 to 1.5)	-75.9 (-78.1 to -73.3)	-17.5 (-20.3 to -14.4)
	50-69 years	238 (219 to 258)	60.8 (56.0 to 65.8)	440 (403 to 482)	31.6 (29.0 to 34.6)	404 (367 to 444)	28.5 (25.9 to 31.4)	364 (327 to 401)	25.1 (22.5 to 27.7)	-58.7 (-63.4 to -53.2)	-20.7 (-23.9 to -17.3)
	70+ years	898 (823 to 955)	858.3 (786.4 to 912.9)	2,970 (2,470 to 3,260)	708.3 (587.8 to 776.9)	2,730 (2,260 to 3,010)	602.9 (499.3 to 666.1)	2,680 (2,210 to 2,950)	555.4 (457.7 to 610.7)	-35.3 (-42.3 to -29.7)	-21.6 (-23.5 to -19.6)
**Canada**	All Ages	6,750 (6,020 to 7,210)	24.8 (22.1 to 26.5)	8,920 (7,570 to 9,800)	24.2 (20.6 to 26.6)	8,690 (7,400 to 9,720)	23.4 (19.9 to 26.2)	6,970 (5,850 to 7,740)	18.6 (15.6 to 20.7)	-24.9 (-31.6 to -18.7)	-23.3 (-25.4 to -20.9)
	Under 5	95 (88 to 104)	5.0 (4.6 to 5.4)	34 (31 to 37)	1.7 (1.6 to 1.9)	29 (24 to 35)	1.5 (1.2 to 1.8)	16 (13 to 19)	0.8 (0.7 to 1.0)	-83.1 (-86.4 to -79.3)	-52.0 (-60.2 to -43.3)
	5-14 years	22 (20 to 24)	0.6 (0.5 to 0.6)	8 (7 to 9)	0.2 (0.2 to 0.2)	8 (7 to 8)	0.2 (0.2 to 0.2)	7 (6 to 7)	0.2 (0.1 to 0.2)	-73.0 (-76.6 to -68.8)	-20.1 (-26.1 to -13.7)
	15-49 years	244 (229 to 260)	1.7 (1.6 to 1.8)	204 (188 to 223)	1.2 (1.1 to 1.4)	200 (181 to 221)	1.2 (1.1 to 1.3)	170 (156 to 187)	1.0 (0.9 to 1.1)	-38.2 (-44.7 to -31.3)	-17.6 (-20.5 to -14.3)
	50-69 years	733 (661 to 809)	15.4 (13.9 to 17.0)	920 (836 to 1,010)	9.4 (8.6 to 10.4)	810 (727 to 903)	8.3 (7.4 to 9.2)	716 (649 to 790)	7.3 (6.6 to 8.0)	-52.9 (-59.0 to -46.0)	-22.9 (-26.0 to -19.5)
	70+ years	5,650 (4,970 to 6,090)	281.1 (247.1 to 302.6)	7,750 (6,400 to 8,570)	172.5 (142.4 to 190.7)	7,640 (6,350 to 8,590)	164.0 (136.4 to 184.5)	6,060 (4,960 to 6,770)	125.7 (103.0 to 140.5)	-55.3 (-59.4 to -51.6)	-27.1 (-29.2 to -24.8)
**Greenland**	All Ages	12 (10 to 13)	21.2 (17.9 to 24.2)	10 (8 to 11)	17.0 (14.5 to 19.9)	10 (8 to 12)	17.8 (15.0 to 21.1)	7 (6 to 9)	12.7 (10.4 to 15.8)	-40.2 (-52.2 to -23.5)	-25.3 (-32.4 to -14.9)
	Under 5	2 (2 to 3)	43.9 (33.6 to 57.6)	0 (0 to 0)	7.9 (6.0 to 10.2)	0 (0 to 0)	8.3 (6.3 to 10.6)	0 (0 to 0)	4.9 (3.7 to 6.4)	-88.8 (-92.7 to -83.8)	-37.6 (-45.5 to -29.6)
	5-14 years	0 (0 to 0)	1.5 (1.2 to 2.0)	0 (0 to 0)	0.4 (0.3 to 0.6)	0 (0 to 0)	0.5 (0.3 to 0.6)	0 (0 to 0)	0.4 (0.3 to 0.5)	-76.3 (-83.6 to -66.1)	-15.0 (-25.6 to -3.0)
	15-49 years	2 (1 to 2)	5.1 (4.1 to 6.2)	1 (0 to 1)	2.3 (1.8 to 3.0)	1 (0 to 1)	2.3 (1.8 to 3.1)	0 (0 to 1)	1.8 (1.3 to 2.5)	-64.2 (-75.9 to -49.7)	-22.3 (-34.1 to -13.8)

	50-69 years	3<br>(2 to 3)	40.7<br>(33.3 to 48.6)	3<br>(2 to 3)	18.8<br>(15.0 to 22.9)	3<br>(2 to 4)	19.6<br>(15.7 to 23.8)	2<br>(2 to 3)	15.2<br>(12.1 to 19.4)	-62.5<br>(-72.3 to -50.4)	-18.9<br>(-26.8 to -8.7)
	70+ years	5<br>(4 to 6)	388.5<br>(307.0 to 458.3)	6<br>(5 to 7)	203.1<br>(162.9 to 246.6)	6<br>(5 to 8)	212.8<br>(170.0 to 263.7)	4<br>(3 to 6)	137.5<br>(107.1 to 183.0)	-64.6<br>(-73.2 to -52.0)	-32.3<br>(-40.0 to -18.9)
**United States of America**	All Ages	70,900<br>(62,600 to 75,200)	27.9<br>(24.6 to 29.6)	65,200<br>(55,600 to 70,400)	19.8<br>(16.9 to 21.3)	63,200<br>(53,200 to 69,700)	19.1<br>(16.0 to 21.0)	53,900<br>(45,900 to 58,600)	16.2<br>(13.8 to 17.6)	-42.0<br>(-44.5 to -39.2)	-18.1<br>(-20.2 to -15.6)
	Under 5	1,360<br>(1,300 to 1,410)	6.9<br>(6.6 to 7.2)	559<br>(533 to 584)	2.9<br>(2.8 to 3.1)	480<br>(423 to 531)	2.5<br>(2.2 to 2.8)	318<br>(274 to 364)	1.7<br>(1.5 to 2.0)	-75.1<br>(-78.7 to -71.3)	-41.5<br>(-49.9 to -33.5)
	5-14 years	203<br>(198 to 210)	0.6<br>(0.5 to 0.6)	157<br>(151 to 162)	0.4<br>(0.4 to 0.4)	144<br>(134 to 154)	0.4<br>(0.3 to 0.4)	117<br>(109 to 125)	0.3<br>(0.3 to 0.3)	-48.9<br>(-52.5 to -45.2)	-24.2<br>(-28.4 to -20.2)
	15-49 years	3,660<br>(3,560 to 3,760)	2.7<br>(2.7 to 2.8)	2,980<br>(2,890 to 3,060)	2.0<br>(1.9 to 2.0)	2,810<br>(2,630 to 2,970)	1.9<br>(1.7 to 2.0)	2,320<br>(2,200 to 2,440)	1.5<br>(1.4 to 1.6)	-44.1<br>(-47.2 to -41.2)	-22.3<br>(-25.0 to -19.5)
	50-69 years	9,240<br>(8,940 to 9,500)	21.6<br>(20.9 to 22.2)	12,200<br>(11,700 to 12,600)	14.9<br>(14.3 to 15.4)	11,800<br>(11,100 to 12,400)	14.3<br>(13.5 to 15.1)	10,300<br>(9,850 to 10,800)	12.5<br>(11.9 to 13.1)	-42.2<br>(-45.0 to -39.2)	-15.9<br>(-18.1 to -13.7)
	70+ years	56,500<br>(48,400 to 60,500)	266.3<br>(228.1 to 285.4)	49,400<br>(40,100 to 54,300)	136.8<br>(111.2 to 150.3)	48,000<br>(38,400 to 53,700)	128.3<br>(102.7 to 143.6)	40,800<br>(32,900 to 45,000)	106.0<br>(85.4 to 117.0)	-60.2<br>(-62.4 to -58.2)	-22.5<br>(-24.6 to -20.1)
**Argentina**	All Ages	8,200<br>(7,730 to 8,590)	24.8<br>(23.4 to 25.9)	41,600<br>(38,000 to 44,200)	92.5<br>(84.5 to 98.2)	33,600<br>(30,300 to 36,100)	74.1<br>(66.9 to 79.8)	30,200<br>(27,100 to 32,600)	66.4<br>(59.5 to 71.7)	168.0<br>(147.0 to 187.4)	-28.2<br>(-30.7 to -25.7)
	Under 5	1,340<br>(1,250 to 1,450)	39.1<br>(36.4 to 42.1)	425<br>(389 to 463)	12.6<br>(11.6 to 13.8)	254<br>(217 to 294)	8.0<br>(6.8 to 9.2)	211<br>(169 to 262)	7.1<br>(5.7 to 8.8)	-81.9<br>(-85.6 to -77.3)	-44.2<br>(-54.6 to -32.8)
	5-14 years	85<br>(78 to 92)	1.3<br>(1.2 to 1.4)	102<br>(93 to 110)	1.4<br>(1.3 to 1.6)	74<br>(66 to 82)	1.0<br>(0.9 to 1.2)	73<br>(65 to 82)	1.0<br>(0.9 to 1.1)	-19.6<br>(-30.4 to -7.8)	-29.0<br>(-34.9 to -22.4)
	15-49 years	526<br>(495 to 558)	3.3<br>(3.1 to 3.5)	1,690<br>(1,580 to 1,800)	7.3<br>(6.8 to 7.8)	1,320<br>(1,220 to 1,420)	5.6<br>(5.2 to 6.1)	1,210<br>(1,120 to 1,320)	5.2<br>(4.8 to 5.6)	55.9<br>(40.8 to 71.1)	-29.3<br>(-32.1 to -26.4)
	50-69 years	1,360<br>(1,260 to 1,460)	26.0<br>(24.1 to 28.0)	6,050<br>(5,630 to 6,510)	76.2<br>(70.9 to 81.9)	5,300<br>(4,900 to 5,720)	65.6<br>(60.7 to 70.9)	4,800<br>(4,410 to 5,210)	58.6<br>(53.8 to 63.5)	125.3<br>(102.2 to 151.3)	-23.2<br>(-26.4 to -20.1)
	70+ years	4,890<br>(4,450 to 5,210)	264.5<br>(240.6 to 281.7)	33,400<br>(29,800 to 35,700)	974.7<br>(870.4 to 1,041.6)	26,600<br>(23,500 to 28,900)	761.0<br>(670.5 to 827.0)	23,900<br>(21,000 to 26,000)	670.9<br>(588.1 to 729.9)	153.7<br>(132.4 to 173.3)	-31.2<br>(-33.8 to -28.7)
**Chile**	All Ages	6,370<br>(5,960 to 6,700)	47.9<br>(44.9 to 50.5)	5,130<br>(4,540 to 5,530)	27.9<br>(24.7 to 30.0)	3,880<br>(3,360 to 4,200)	20.8<br>(18.0 to 22.5)	3,960<br>(3,420 to 4,310)	21.1<br>(18.2 to 22.9)	-56.0<br>(-60.2 to -52.4)	-24.3<br>(-26.6 to -22.2)
	Under 5	779<br>(730 to 828)	54.2<br>(50.8 to 57.7)	65<br>(58 to 72)	5.6<br>(5.1 to 6.2)	35<br>(31 to 39)	3.1<br>(2.7 to 3.4)	30<br>(26 to 35)	2.8<br>(2.4 to 3.2)	-94.9<br>(-95.6 to -94.0)	-50.6<br>(-56.8 to -44.1)
	5-14 years	42<br>(39 to 45)	1.6<br>(1.5 to 1.8)	18<br>(16 to 20)	0.7<br>(0.7 to 0.8)	12<br>(11 to 13)	0.5<br>(0.4 to 0.5)	13<br>(12 to 14)	0.5<br>(0.5 to 0.6)	-69.1<br>(-72.9 to -65.2)	-29.9<br>(-33.8 to -25.9)
	15-49 years	411<br>(384 to 440)	5.8<br>(5.4 to 6.2)	255<br>(238 to 274)	2.7<br>(2.5 to 2.9)	193<br>(179 to 207)	2.0<br>(1.9 to 2.2)	199<br>(185 to 213)	2.1<br>(1.9 to 2.2)	-63.9<br>(-67.2 to -60.3)	-23.3<br>(-25.6 to -20.7)

	50-69 years	1,050<br>(954 to 1,140)	62.9<br>(57.3 to 68.5)	633<br>(577 to 700)	16.1<br>(14.6 to 17.8)	511<br>(464 to 567)	12.8<br>(11.6 to 14.1)	521<br>(471 to 579)	12.8<br>(11.5 to 14.2)	-79.7<br>(-82.3 to -76.6)	-20.7<br>(-23.9 to -17.2)
	70+ years	4,090<br>(3,710 to 4,360)	756.7<br>(686.6 to 806.3)	4,160<br>(3,580 to 4,520)	286.0<br>(246.0 to 310.7)	3,130<br>(2,630 to 3,430)	207.3<br>(174.1 to 226.9)	3,200<br>(2,680 to 3,520)	205.4<br>(172.1 to 225.9)	-72.9<br>(-75.6 to -70.5)	-28.2<br>(-30.7 to -26.0)
**Uruguay**	All Ages	879<br>(813 to 934)	28.0<br>(25.9 to 29.7)	1,750<br>(1,540 to 1,890)	51.3<br>(45.1 to 55.4)	1,330<br>(1,140 to 1,450)	39.1<br>(33.6 to 42.6)	1,410<br>(1,220 to 1,530)	41.4<br>(35.8 to 45.0)	47.8<br>(35.1 to 60.1)	-19.4<br>(-21.8 to -16.9)
	Under 5	91<br>(83 to 99)	33.5<br>(30.5 to 36.4)	18<br>(16 to 21)	8.5<br>(7.4 to 9.7)	13<br>(10 to 15)	6.2<br>(5.1 to 7.5)	11<br>(8 to 14)	5.5<br>(4.3 to 7.0)	-83.5<br>(-87.4 to -78.8)	-35.4<br>(-47.4 to -22.0)
	5-14 years	4<br>(3 to 4)	0.7<br>(0.6 to 0.8)	3<br>(3 to 4)	0.7<br>(0.6 to 0.8)	1<br>(1 to 2)	0.3<br>(0.2 to 0.3)	3<br>(2 to 3)	0.6<br>(0.5 to 0.6)	-19.9<br>(-35.2 to -3.5)	-17.9<br>(-25.3 to -9.4)
	15-49 years	35<br>(33 to 38)	2.4<br>(2.2 to 2.5)	60<br>(56 to 64)	3.7<br>(3.4 to 3.9)	46<br>(43 to 50)	2.8<br>(2.6 to 3.0)	47<br>(44 to 51)	2.9<br>(2.7 to 3.1)	22.0<br>(10.7 to 34.3)	-21.0<br>(-23.8 to -18.3)
	50-69 years	130<br>(120 to 140)	22.0<br>(20.3 to 23.7)	179<br>(165 to 195)	25.0<br>(23.0 to 27.2)	144<br>(132 to 157)	19.9<br>(18.2 to 21.8)	153<br>(140 to 168)	21.0<br>(19.2 to 23.0)	-4.5<br>(-14.8 to 7.4)	-16.1<br>(-19.6 to -12.6)
	70+ years	619<br>(557 to 667)	252.1<br>(227.0 to 271.7)	1,490<br>(1,280 to 1,620)	404.8<br>(348.4 to 441.4)	1,130<br>(947 to 1,240)	302.8<br>(254.1 to 333.1)	1,200<br>(1,010 to 1,310)	317.9<br>(268.9 to 349.2)	26.1<br>(14.8 to 38.0)	-21.4<br>(-23.9 to -19.0)
**Andorra**	All Ages	5<br>(4 to 7)	9.6<br>(7.1 to 12.7)	14<br>(10 to 18)	17.1<br>(12.6 to 22.0)	11<br>(8 to 15)	13.1<br>(9.3 to 17.3)	10<br>(7 to 13)	11.6<br>(7.9 to 15.5)	20.9<br>(-20.2 to 75.4)	-32.3<br>(-42.6 to -24.9)
	Under 5	0<br>(0 to 0)	7.3<br>(4.9 to 11.0)	0<br>(0 to 0)	0.9<br>(0.7 to 1.3)	0<br>(0 to 0)	0.7<br>(0.5 to 1.0)	0<br>(0 to 0)	0.3<br>(0.2 to 0.5)	-95.4<br>(-97.7 to -91.8)	-64.3<br>(-76.5 to -51.7)
	5-14 years	0<br>(0 to 0)	0.3<br>(0.2 to 0.4)	0<br>(0 to 0)	0.1<br>(0.1 to 0.1)	0<br>(0 to 0)	0.1<br>(0.1 to 0.1)	0<br>(0 to 0)	0.1<br>(0.1 to 0.1)	-77.1<br>(-85.6 to -64.7)	-28.4<br>(-35.4 to -21.3)
	15-49 years	0<br>(0 to 0)	1.0<br>(0.7 to 1.3)	0<br>(0 to 0)	0.8<br>(0.6 to 1.0)	0<br>(0 to 0)	0.5<br>(0.3 to 0.8)	0<br>(0 to 0)	0.5<br>(0.3 to 0.7)	-51.5<br>(-72.5 to -23.0)	-39.1<br>(-58.1 to -27.9)
	50-69 years	1<br>(1 to 1)	8.3<br>(5.6 to 12.2)	1<br>(1 to 2)	4.8<br>(3.4 to 6.6)	1<br>(1 to 1)	3.5<br>(2.2 to 5.1)	1<br>(0 to 1)	3.1<br>(1.9 to 4.5)	-63.0<br>(-80.0 to -37.4)	-35.8<br>(-52.4 to -24.1)
	70+ years	4<br>(3 to 5)	122.3<br>(88.5 to 161.8)	13<br>(9 to 16)	148.6<br>(107.5 to 189.9)	10<br>(7 to 13)	111.0<br>(78.4 to 146.4)	9<br>(6 to 12)	96.0<br>(64.7 to 127.9)	-21.5<br>(-47.2 to 13.4)	-35.4<br>(-44.4 to -28.4)
**Austria**	All Ages	1,290<br>(1,170 to 1,380)	16.7<br>(15.1 to 17.8)	1,480<br>(1,250 to 1,610)	16.6<br>(14.0 to 18.1)	1,390<br>(1,190 to 1,520)	15.6<br>(13.3 to 17.0)	1,150<br>(963 to 1,270)	12.8<br>(10.7 to 14.2)	-23.1<br>(-30.8 to -16.4)	-22.8<br>(-25.0 to -20.8)
	Under 5	16<br>(14 to 17)	3.5<br>(3.2 to 3.9)	4<br>(3 to 4)	0.8<br>(0.7 to 1.0)	3<br>(2 to 3)	0.6<br>(0.5 to 0.7)	2<br>(2 to 2)	0.4<br>(0.4 to 0.5)	-87.5<br>(-89.6 to -85.2)	-47.7<br>(-54.8 to -40.4)
	5-14 years	2<br>(2 to 2)	0.2<br>(0.2 to 0.3)	1<br>(1 to 1)	0.1<br>(0.1 to 0.1)	1<br>(1 to 1)	0.1<br>(0.1 to 0.1)	1<br>(1 to 1)	0.1<br>(0.1 to 0.1)	-72.2<br>(-76.2 to -68.0)	-43.9<br>(-49.0 to -38.6)
	15-49 years	44<br>(40 to 47)	1.1<br>(1.0 to 1.2)	20<br>(18 to 22)	0.5<br>(0.5 to 0.5)	16<br>(14 to 18)	0.4<br>(0.4 to 0.4)	14<br>(13 to 15)	0.3<br>(0.3 to 0.4)	-68.6<br>(-72.1 to -64.6)	-30.7<br>(-33.5 to -27.9)

	50-69 years	136 (126 to 148)	8.3 (7.7 to 9.1)	116 (106 to 127)	4.9 (4.5 to 5.4)	102 (93 to 113)	4.3 (3.9 to 4.7)	91 (82 to 99)	3.8 (3.4 to 4.1)	-54.8 (-59.8 to -48.8)	-23.4 (-26.4 to -20.2)
	70+ years	1,100 (979 to 1,180)	143.8 (128.4 to 154.3)	1,340 (1,110 to 1,470)	109.2 (91.0 to 120.1)	1,270 (1,060 to 1,400)	102.7 (86.2 to 113.0)	1,040 (858 to 1,160)	83.9 (68.9 to 93.5)	-41.6 (-47.6 to -36.3)	-23.2 (-25.6 to -21.1)
**Belgium**	All Ages	3,420 (3,040 to 3,670)	34.3 (30.5 to 36.8)	6,510 (5,340 to 7,170)	57.1 (46.8 to 62.8)	6,150 (5,090 to 6,850)	53.8 (44.5 to 59.9)	4,730 (3,810 to 5,310)	41.2 (33.2 to 46.3)	20.1 (6.3 to 31.6)	-27.8 (-30.2 to -24.8)
	Under 5	40 (37 to 44)	6.8 (6.2 to 7.4)	14 (12 to 16)	2.3 (2.0 to 2.6)	10 (9 to 12)	1.7 (1.5 to 2.1)	7 (6 to 9)	1.3 (1.0 to 1.6)	-81.4 (-85.7 to -76.4)	-45.3 (-55.0 to -35.1)
	5-14 years	7 (6 to 8)	0.6 (0.5 to 0.7)	3 (3 to 3)	0.2 (0.2 to 0.2)	3 (2 to 3)	0.2 (0.2 to 0.3)	3 (3 to 3)	0.2 (0.2 to 0.3)	-62.0 (-68.4 to -54.5)	4.2 (-3.5 to 12.1)
	15-49 years	88 (83 to 94)	1.8 (1.7 to 1.9)	64 (59 to 69)	1.3 (1.2 to 1.4)	50 (45 to 55)	1.0 (0.9 to 1.1)	50 (46 to 55)	1.0 (0.9 to 1.1)	-43.9 (-49.5 to -37.3)	-21.1 (-24.4 to -17.5)
	50-69 years	373 (341 to 407)	16.5 (15.1 to 18.1)	427 (391 to 467)	14.6 (13.4 to 16.0)	361 (326 to 396)	12.3 (11.1 to 13.5)	346 (313 to 381)	11.7 (10.6 to 12.9)	-29.2 (-37.8 to -19.8)	-19.9 (-23.3 to -16.3)
	70+ years	2,920 (2,540 to 3,150)	303.5 (264.8 to 328.1)	6,010 (4,850 to 6,650)	385.0 (310.7 to 426.2)	5,730 (4,660 to 6,410)	361.7 (294.1 to 404.6)	4,320 (3,410 to 4,880)	268.7 (212.0 to 303.3)	-11.5 (-21.9 to -2.8)	-30.2 (-32.7 to -27.2)
**Cyprus**	All Ages	145 (121 to 169)	18.6 (15.5 to 21.7)	179 (150 to 213)	13.5 (11.4 to 16.1)	158 (131 to 190)	11.8 (9.8 to 14.2)	139 (113 to 169)	10.2 (8.3 to 12.5)	-45.1 (-56.7 to -31.4)	-24.4 (-30.8 to -17.5)
	Under 5	4 (3 to 7)	7.0 (4.9 to 10.5)	0 (0 to 1)	0.7 (0.5 to 0.9)	0 (0 to 1)	0.5 (0.4 to 0.7)	0 (0 to 0)	0.4 (0.3 to 0.5)	-94.6 (-97.2 to -91.0)	-43.1 (-51.5 to -34.2)
	5-14 years	0 (0 to 1)	0.3 (0.2 to 0.4)	0 (0 to 0)	0.2 (0.2 to 0.3)	0 (0 to 0)	0.1 (0.1 to 0.1)	0 (0 to 0)	0.1 (0.0 to 0.1)	-78.3 (-85.3 to -69.2)	-70.9 (-76.8 to -64.7)
	15-49 years	4 (3 to 4)	0.9 (0.8 to 1.0)	3 (3 to 4)	0.5 (0.4 to 0.6)	3 (2 to 3)	0.4 (0.3 to 0.5)	2 (2 to 3)	0.3 (0.3 to 0.4)	-61.5 (-69.4 to -51.2)	-28.3 (-35.2 to -20.6)
	50-69 years	13 (10 to 15)	10.1 (8.1 to 12.3)	14 (11 to 17)	4.8 (3.8 to 5.9)	12 (9 to 15)	4.0 (3.1 to 5.1)	10 (8 to 13)	3.5 (2.7 to 4.4)	-65.2 (-74.1 to -53.1)	-26.7 (-34.4 to -18.5)
	70+ years	124 (101 to 148)	240.4 (196.5 to 287.0)	161 (134 to 194)	129.6 (107.6 to 156.0)	143 (117 to 173)	109.6 (90.2 to 133.2)	126 (101 to 153)	92.9 (74.8 to 113.3)	-61.4 (-70.2 to -50.7)	-28.3 (-34.5 to -21.6)
**Denmark**	All Ages	1,790 (1,600 to 1,910)	34.8 (31.2 to 37.1)	2,560 (2,160 to 2,800)	44.1 (37.2 to 48.3)	2,250 (1,900 to 2,510)	38.7 (32.6 to 43.1)	2,070 (1,730 to 2,280)	35.4 (29.6 to 39.0)	1.7 (-6.9 to 10.4)	-19.7 (-22.2 to -17.0)
	Under 5	18 (16 to 20)	6.3 (5.6 to 7.0)	4 (4 to 5)	1.5 (1.3 to 1.7)	3 (3 to 4)	1.1 (0.9 to 1.4)	3 (2 to 3)	0.9 (0.7 to 1.1)	-85.8 (-88.9 to -82.4)	-39.3 (-48.3 to -29.4)
	5-14 years	3 (3 to 4)	0.6 (0.5 to 0.6)	1 (1 to 1)	0.2 (0.2 to 0.2)	1 (1 to 1)	0.2 (0.2 to 0.2)	1 (1 to 1)	0.1 (0.1 to 0.2)	-74.0 (-78.1 to -68.8)	-25.1 (-31.5 to -17.9)
	15-49 years	46 (43 to 50)	1.7 (1.6 to 1.9)	22 (20 to 24)	0.9 (0.8 to 0.9)	20 (18 to 22)	0.8 (0.7 to 0.9)	16 (15 to 18)	0.6 (0.6 to 0.7)	-63.5 (-67.5 to -58.4)	-27.5 (-30.7 to -24.1)

	50-69 years	147<br>(134 to 162)	14.3<br>(13.0 to 15.8)	172<br>(153 to 194)	11.8<br>(10.5 to 13.3)	149<br>(131 to 171)	10.2<br>(9.0 to 11.7)	131<br>(115 to 149)	8.9<br>(7.8 to 10.2)	-37.7<br>(-46.8 to -26.5)	-24.2<br>(-27.7 to -20.6)
	70+ years	1,580<br>(1,390 to 1,690)	282.0<br>(248.8 to 302.3)	2,360<br>(1,980 to 2,590)	287.7<br>(240.9 to 315.9)	2,080<br>(1,730 to 2,330)	247.4<br>(205.4 to 277.2)	1,920<br>(1,580 to 2,120)	223.6<br>(184.1 to 246.9)	-20.7<br>(-28.0 to -13.8)	-22.3<br>(-24.7 to -19.6)
**Finland**	All Ages	2,330<br>(2,080 to 2,500)	46.5<br>(41.6 to 50.0)	775<br>(635 to 860)	14.0<br>(11.5 to 15.6)	691<br>(569 to 776)	12.5<br>(10.3 to 14.0)	644<br>(520 to 720)	11.6<br>(9.4 to 13.0)	-75.0<br>(-77.5 to -72.6)	-17.0<br>(-19.7 to -14.0)
	Under 5	13<br>(12 to 15)	4.3<br>(3.8 to 4.7)	3<br>(3 to 4)	1.2<br>(1.1 to 1.4)	2<br>(2 to 3)	0.9<br>(0.8 to 1.1)	2<br>(2 to 2)	0.8<br>(0.6 to 0.9)	-82.3<br>(-85.5 to -78.5)	-39.2<br>(-47.6 to -29.6)
	5-14 years	3<br>(3 to 4)	0.5<br>(0.5 to 0.6)	1<br>(1 to 1)	0.2<br>(0.2 to 0.2)	1<br>(1 to 1)	0.1<br>(0.1 to 0.2)	1<br>(1 to 1)	0.2<br>(0.2 to 0.2)	-61.7<br>(-67.3 to -55.1)	5.2<br>(-5.3 to 17.6)
	15-49 years	70<br>(66 to 75)	2.7<br>(2.5 to 2.9)	22<br>(20 to 23)	0.9<br>(0.9 to 1.0)	19<br>(18 to 21)	0.8<br>(0.8 to 0.9)	16<br>(15 to 17)	0.7<br>(0.6 to 0.7)	-75.1<br>(-77.4 to -72.5)	-26.8<br>(-29.5 to -23.6)
	50-69 years	221<br>(203 to 240)	22.0<br>(20.2 to 23.9)	74<br>(68 to 81)	5.1<br>(4.7 to 5.6)	65<br>(59 to 71)	4.5<br>(4.1 to 5.0)	60<br>(55 to 66)	4.2<br>(3.9 to 4.6)	-80.8<br>(-83.0 to -78.4)	-17.8<br>(-21.4 to -14.2)
	70+ years	2,020<br>(1,770 to 2,180)	442.0<br>(386.6 to 477.7)	675<br>(539 to 757)	76.3<br>(60.9 to 85.6)	603<br>(484 to 684)	66.1<br>(53.0 to 74.9)	566<br>(447 to 638)	60.4<br>(47.7 to 68.1)	-86.3<br>(-87.8 to -85.0)	-20.9<br>(-23.5 to -18.0)
**France**	All Ages	18,200<br>(16,200 to 19,500)	31.5<br>(28.0 to 33.7)	23,200<br>(19,100 to 25,600)	35.0<br>(28.9 to 38.7)	21,000<br>(17,700 to 23,600)	31.7<br>(26.7 to 35.6)	17,700<br>(14,400 to 19,600)	26.6<br>(21.7 to 29.5)	-15.3<br>(-24.3 to -7.9)	-24.0<br>(-26.2 to -21.7)
	Under 5	160<br>(148 to 173)	4.1<br>(3.8 to 4.4)	59<br>(53 to 67)	1.6<br>(1.5 to 1.8)	44<br>(36 to 53)	1.2<br>(1.0 to 1.5)	31<br>(25 to 36)	0.9<br>(0.7 to 1.0)	-79.0<br>(-82.8 to -74.9)	-46.9<br>(-54.8 to -39.7)
	5-14 years	30<br>(27 to 32)	0.4<br>(0.3 to 0.4)	13<br>(11 to 14)	0.2<br>(0.1 to 0.2)	12<br>(10 to 13)	0.1<br>(0.1 to 0.2)	10<br>(9 to 11)	0.1<br>(0.1 to 0.1)	-68.2<br>(-72.4 to -63.9)	-21.7<br>(-25.8 to -17.6)
	15-49 years	424<br>(395 to 455)	1.5<br>(1.4 to 1.6)	219<br>(203 to 237)	0.8<br>(0.7 to 0.8)	193<br>(175 to 215)	0.7<br>(0.6 to 0.8)	156<br>(144 to 170)	0.6<br>(0.5 to 0.6)	-62.1<br>(-66.0 to -57.8)	-28.5<br>(-31.0 to -25.7)
	50-69 years	1,400<br>(1,280 to 1,540)	12.0<br>(11.0 to 13.2)	1,230<br>(1,110 to 1,360)	7.4<br>(6.7 to 8.2)	1,100<br>(977 to 1,230)	6.6<br>(5.9 to 7.4)	945<br>(849 to 1,050)	5.7<br>(5.1 to 6.4)	-52.4<br>(-58.1 to -45.7)	-23.1<br>(-26.3 to -20.1)
	70+ years	16,200<br>(14,200 to 17,400)	310.6<br>(272.5 to 334.3)	21,700<br>(17,700 to 24,000)	230.2<br>(188.0 to 255.4)	19,600<br>(16,400 to 22,200)	202.9<br>(169.4 to 229.3)	16,500<br>(13,300 to 18,500)	166.5<br>(133.9 to 185.9)	-46.4<br>(-52.2 to -41.5)	-27.6<br>(-29.9 to -25.4)
**Germany**	All Ages	17,600<br>(15,800 to 18,800)	22.0<br>(19.8 to 23.5)	23,100<br>(19,400 to 25,400)	27.2<br>(22.9 to 29.9)	21,300<br>(17,800 to 23,500)	25.0<br>(20.9 to 27.6)	18,600<br>(15,300 to 20,500)	21.8<br>(17.9 to 24.0)	-1.0<br>(-10.3 to 7.8)	-20.0<br>(-22.0 to -18.1)
	Under 5	227<br>(212 to 243)	5.1<br>(4.7 to 5.4)	38<br>(35 to 42)	0.9<br>(0.9 to 1.0)	27<br>(24 to 31)	0.7<br>(0.6 to 0.8)	20<br>(17 to 22)	0.5<br>(0.4 to 0.6)	-90.3<br>(-91.8 to -88.9)	-48.4<br>(-53.9 to -43.4)
	5-14 years	50<br>(46 to 54)	0.6<br>(0.5 to 0.6)	13<br>(12 to 14)	0.2<br>(0.2 to 0.2)	10<br>(9 to 11)	0.1<br>(0.1 to 0.1)	9<br>(8 to 10)	0.1<br>(0.1 to 0.1)	-80.3<br>(-82.7 to -77.4)	-29.8<br>(-34.0 to -25.8)
	15-49 years	608<br>(571 to 645)	1.5<br>(1.4 to 1.6)	318<br>(294 to 343)	0.9<br>(0.8 to 1.0)	273<br>(250 to 299)	0.8<br>(0.7 to 0.8)	234<br>(216 to 253)	0.7<br>(0.6 to 0.7)	-56.8<br>(-61.2 to -52.4)	-25.8<br>(-28.2 to -23.6)

	50-69 years	2,050 (1,900 to 2,210)	10.8 (10.0 to 11.6)	2,310 (2,120 to 2,510)	9.6 (8.8 to 10.4)	2,100 (1,920 to 2,300)	8.7 (7.9 to 9.5)	1,890 (1,720 to 2,070)	7.8 (7.1 to 8.5)	-27.8 (-35.9 to -18.6)	-18.4 (-21.5 to -15.4)
	70+ years	14,600 (13,000 to 15,800)	180.9 (160.2 to 195.8)	20,400 (16,700 to 22,700)	154.1 (125.8 to 171.1)	18,900 (15,300 to 21,100)	141.0 (114.1 to 156.9)	16,400 (13,300 to 18,300)	121.1 (98.0 to 134.8)	-33.1 (-40.1 to -26.5)	-21.4 (-23.6 to -19.5)
**Greece**	All Ages	1,920 (1,750 to 2,060)	18.5 (16.8 to 19.8)	6,930 (5,840 to 7,610)	67.2 (56.6 to 73.7)	6,840 (5,810 to 7,590)	66.7 (56.6 to 74.0)	5,730 (4,770 to 6,330)	56.3 (46.9 to 62.2)	204.1 (170.7 to 231.9)	-16.1 (-19.1 to -13.3)
	Under 5	22 (20 to 24)	4.0 (3.7 to 4.3)	12 (11 to 14)	2.8 (2.5 to 3.2)	10 (8 to 12)	2.3 (1.8 to 2.8)	6 (4 to 7)	1.3 (1.1 to 1.6)	-66.6 (-73.9 to -58.4)	-53.3 (-62.6 to -44.1)
	5-14 years	6 (6 to 7)	0.4 (0.4 to 0.5)	2 (2 to 2)	0.2 (0.2 to 0.2)	1 (1 to 2)	0.1 (0.1 to 0.2)	1 (1 to 1)	0.1 (0.1 to 0.1)	-70.0 (-74.5 to -64.9)	-36.6 (-42.4 to -30.4)
	15-49 years	64 (61 to 68)	1.3 (1.2 to 1.4)	58 (53 to 62)	1.3 (1.2 to 1.4)	53 (49 to 59)	1.2 (1.1 to 1.3)	44 (40 to 48)	1.0 (0.9 to 1.1)	-20.2 (-28.3 to -11.4)	-20.8 (-23.8 to -18.1)
	50-69 years	243 (222 to 267)	10.3 (9.4 to 11.3)	313 (284 to 342)	11.7 (10.6 to 12.7)	315 (283 to 348)	11.6 (10.4 to 12.8)	271 (245 to 296)	10.0 (9.0 to 10.9)	-3.1 (-15.4 to 10.4)	-14.6 (-18.0 to -11.2)
	70+ years	1,590 (1,420 to 1,710)	167.7 (150.3 to 180.5)	6,550 (5,470 to 7,210)	380.6 (318.1 to 419.0)	6,460 (5,430 to 7,180)	374.3 (314.6 to 416.0)	5,410 (4,450 to 5,980)	312.5 (257.1 to 345.7)	86.4 (66.1 to 103.5)	-17.9 (-20.9 to -15.0)
**Iceland**	All Ages	95 (82 to 104)	37.5 (32.3 to 40.8)	104 (84 to 117)	30.1 (24.5 to 34.0)	83 (67 to 97)	23.9 (19.3 to 27.9)	94 (77 to 110)	26.8 (21.8 to 31.3)	-28.5 (-37.0 to -18.9)	-10.9 (-19.2 to -3.2)
	Under 5	1 (1 to 1)	5.3 (4.6 to 6.1)	0 (0 to 0)	1.6 (1.3 to 2.0)	0 (0 to 0)	1.3 (1.0 to 1.6)	0 (0 to 0)	1.4 (1.1 to 1.8)	-74.0 (-81.0 to -65.2)	-14.8 (-28.5 to 0.8)
	5-14 years	0 (0 to 0)	0.8 (0.7 to 0.9)	0 (0 to 0)	0.1 (0.1 to 0.1)	0 (0 to 0)	0.3 (0.2 to 0.4)	0 (0 to 0)	0.3 (0.2 to 0.3)	-66.1 (-72.8 to -57.5)	170.4 (116.6 to 244.1)
	15-49 years	2 (2 to 2)	1.7 (1.6 to 1.9)	2 (1 to 2)	1.0 (0.9 to 1.1)	1 (1 to 2)	0.9 (0.8 to 1.0)	2 (1 to 2)	1.0 (0.9 to 1.1)	-42.3 (-50.0 to -32.7)	-1.1 (-10.6 to 8.1)
	50-69 years	7 (6 to 7)	17.1 (15.6 to 18.7)	6 (5 to 7)	7.4 (6.6 to 8.4)	5 (5 to 6)	6.9 (6.0 to 7.8)	6 (5 to 6)	7.1 (6.1 to 8.1)	-58.7 (-65.2 to -51.4)	-5.0 (-13.4 to 3.8)
	70+ years	85 (72 to 93)	463.9 (392.1 to 508.1)	96 (77 to 109)	274.6 (221.2 to 312.4)	76 (60 to 89)	208.3 (165.5 to 244.7)	86 (69 to 101)	228.0 (182.6 to 267.7)	-50.8 (-56.7 to -43.8)	-17.0 (-24.8 to -9.6)
**Ireland**	All Ages	1,860 (1,710 to 1,970)	51.7 (47.4 to 54.8)	1,620 (1,350 to 1,790)	32.9 (27.6 to 36.4)	1,420 (1,160 to 1,640)	28.9 (23.6 to 33.2)	1,150 (930 to 1,290)	23.3 (18.8 to 26.0)	-55.0 (-61.1 to -50.4)	-29.4 (-32.6 to -26.1)
	Under 5	24 (22 to 26)	8.3 (7.6 to 9.2)	8 (7 to 9)	2.5 (2.1 to 2.8)	5 (4 to 7)	1.8 (1.4 to 2.2)	4 (3 to 5)	1.3 (1.1 to 1.5)	-84.4 (-87.3 to -81.0)	-46.9 (-54.1 to -38.0)
	5-14 years	6 (6 to 7)	0.9 (0.8 to 1.0)	2 (2 to 3)	0.3 (0.3 to 0.4)	2 (2 to 3)	0.3 (0.2 to 0.4)	1 (1 to 1)	0.2 (0.1 to 0.2)	-81.5 (-84.1 to -78.2)	-48.4 (-55.1 to -41.1)
	15-49 years	44 (41 to 46)	2.4 (2.3 to 2.6)	33 (30 to 36)	1.4 (1.3 to 1.5)	26 (23 to 30)	1.1 (1.0 to 1.3)	22 (20 to 24)	1.0 (0.9 to 1.0)	-60.8 (-64.8 to -56.7)	-32.3 (-35.5 to -28.5)

	50-69 years	165<br>(153 to 180)	29.0<br>(26.8 to 31.6)	98<br>(89 to 107)	9.1<br>(8.3 to 10.0)	84<br>(76 to 95)	7.7<br>(6.9 to 8.7)	70<br>(63 to 77)	6.3<br>(5.7 to 6.9)	-78.4<br>(-80.8 to -75.6)	-31.4<br>(-34.9 to -27.7)
	70+ years	1,620<br>(1,470 to 1,730)	604.5<br>(548.5 to 642.4)	1,480<br>(1,220 to 1,640)	305.4<br>(252.6 to 339.4)	1,300<br>(1,050 to 1,510)	260.1<br>(209.2 to 301.0)	1,050<br>(838 to 1,190)	202.4<br>(161.3 to 228.5)	-66.5<br>(-71.2 to -62.8)	-33.7<br>(-36.9 to -30.6)
**Israel**	All Ages	680<br>(616 to 723)	13.7<br>(12.4 to 14.6)	1,710<br>(1,410 to 1,880)	18.4<br>(15.2 to 20.2)	1,530<br>(1,270 to 1,680)	16.2<br>(13.5 to 17.8)	1,330<br>(1,090 to 1,480)	13.9<br>(11.3 to 15.5)	1.5<br>(-9.9 to 10.7)	-24.3<br>(-27.0 to -21.7)
	Under 5	34<br>(30 to 37)	6.5<br>(5.9 to 7.2)	12<br>(11 to 14)	1.4<br>(1.2 to 1.5)	8<br>(7 to 9)	0.9<br>(0.7 to 1.0)	4<br>(3 to 5)	0.5<br>(0.3 to 0.6)	-93.0<br>(-94.8 to -91.0)	-66.7<br>(-74.3 to -59.1)
	5-14 years	4<br>(4 to 5)	0.4<br>(0.4 to 0.5)	2<br>(2 to 2)	0.1<br>(0.1 to 0.2)	2<br>(2 to 2)	0.1<br>(0.1 to 0.1)	2<br>(2 to 2)	0.1<br>(0.1 to 0.1)	-74.4<br>(-78.9 to -68.5)	-18.5<br>(-29.1 to -6.2)
	15-49 years	23<br>(22 to 24)	0.9<br>(0.9 to 1.0)	29<br>(27 to 31)	0.7<br>(0.6 to 0.7)	23<br>(21 to 25)	0.5<br>(0.5 to 0.6)	19<br>(18 to 21)	0.4<br>(0.4 to 0.5)	-54.8<br>(-58.9 to -50.2)	-34.6<br>(-37.8 to -31.4)
	50-69 years	69<br>(63 to 75)	9.9<br>(9.1 to 10.8)	127<br>(114 to 140)	8.0<br>(7.2 to 8.8)	110<br>(99 to 122)	6.8<br>(6.1 to 7.5)	95<br>(85 to 106)	5.8<br>(5.2 to 6.4)	-41.8<br>(-48.7 to -33.6)	-27.5<br>(-31.2 to -23.7)
	70+ years	550<br>(486 to 591)	180.8<br>(159.7 to 194.4)	1,540<br>(1,250 to 1,700)	204.5<br>(166.9 to 226.7)	1,380<br>(1,130 to 1,540)	175.4<br>(143.2 to 194.7)	1,210<br>(973 to 1,360)	147.0<br>(117.8 to 164.9)	-18.7<br>(-28.3 to -10.8)	-28.1<br>(-30.6 to -25.5)
**Italy**	All Ages	7,680<br>(6,770 to 8,160)	13.5<br>(11.9 to 14.4)	14,400<br>(11,600 to 16,000)	23.9<br>(19.3 to 26.5)	13,400<br>(11,000 to 14,800)	22.3<br>(18.3 to 24.7)	11,300<br>(9,080 to 12,500)	18.9<br>(15.2 to 21.0)	39.6<br>(25.8 to 49.6)	-21.1<br>(-22.5 to -19.7)
	Under 5	198<br>(188 to 208)	7.2<br>(6.8 to 7.6)	27<br>(24 to 30)	1.2<br>(1.0 to 1.3)	21<br>(17 to 25)	0.9<br>(0.8 to 1.1)	15<br>(12 to 18)	0.7<br>(0.5 to 0.8)	-90.3<br>(-92.5 to -88.4)	-40.2<br>(-52.1 to -29.4)
	5-14 years	29<br>(28 to 30)	0.4<br>(0.4 to 0.5)	9<br>(8 to 9)	0.2<br>(0.1 to 0.2)	7<br>(6 to 8)	0.1<br>(0.1 to 0.1)	7<br>(6 to 7)	0.1<br>(0.1 to 0.1)	-72.1<br>(-74.4 to -69.6)	-18.1<br>(-21.7 to -14.4)
	15-49 years	292<br>(281 to 303)	1.0<br>(1.0 to 1.1)	183<br>(175 to 191)	0.7<br>(0.7 to 0.8)	135<br>(128 to 143)	0.5<br>(0.5 to 0.6)	125<br>(118 to 131)	0.5<br>(0.5 to 0.5)	-50.2<br>(-53.3 to -47.1)	-29.7<br>(-31.4 to -28.1)
	50-69 years	796<br>(758 to 835)	6.0<br>(5.7 to 6.3)	770<br>(726 to 812)	4.6<br>(4.3 to 4.9)	657<br>(621 to 697)	3.9<br>(3.7 to 4.1)	619<br>(582 to 657)	3.6<br>(3.4 to 3.8)	-39.4<br>(-43.7 to -35.0)	-21.3<br>(-23.0 to -19.6)
	70+ years	6,360<br>(5,440 to 6,840)	114.8<br>(98.3 to 123.4)	13,400<br>(10,700 to 15,000)	130.5<br>(103.6 to 145.4)	12,600<br>(10,200 to 14,000)	120.6<br>(97.7 to 134.2)	10,500<br>(8,350 to 11,800)	100.0<br>(79.4 to 111.7)	-12.9<br>(-20.7 to -6.8)	-23.3<br>(-24.7 to -22.0)
**Luxembourg**	All Ages	84<br>(77 to 89)	22.0<br>(20.3 to 23.4)	130<br>(113 to 143)	21.1<br>(18.3 to 23.2)	115<br>(100 to 128)	18.2<br>(15.9 to 20.2)	95<br>(82 to 106)	14.7<br>(12.7 to 16.4)	-33.0<br>(-40.5 to -25.2)	-30.2<br>(-33.5 to -26.9)
	Under 5	1<br>(1 to 1)	4.7<br>(4.2 to 5.4)	0<br>(0 to 0)	1.2<br>(1.0 to 1.4)	0<br>(0 to 0)	1.0<br>(0.8 to 1.2)	0<br>(0 to 0)	0.7<br>(0.6 to 0.9)	-85.2<br>(-88.4 to -81.2)	-42.5<br>(-49.3 to -35.9)
	5-14 years	0<br>(0 to 0)	0.5<br>(0.4 to 0.5)	0<br>(0 to 0)	0.1<br>(0.0 to 0.1)	0<br>(0 to 0)	0.1<br>(0.1 to 0.1)	0<br>(0 to 0)	0.1<br>(0.1 to 0.1)	-82.0<br>(-85.6 to -77.5)	48.5<br>(28.9 to 71.6)
	15-49 years	3<br>(3 to 3)	1.5<br>(1.4 to 1.6)	2<br>(1 to 2)	0.5<br>(0.5 to 0.6)	1<br>(1 to 1)	0.4<br>(0.4 to 0.5)	1<br>(1 to 1)	0.4<br>(0.3 to 0.4)	-76.3<br>(-79.4 to -72.8)	-29.7<br>(-34.9 to -24.2)

	50-69 years	10<br>(9 to 11)	11.8<br>(10.7 to 12.9)	9<br>(8 to 10)	5.8<br>(5.1 to 6.5)	7<br>(6 to 8)	4.7<br>(4.1 to 5.3)	7<br>(6 to 8)	4.3<br>(3.7 to 4.9)	-63.7<br>(-69.1 to -57.4)	-26.4<br>(-31.6 to -20.8)
	70+ years	70<br>(64 to 75)	204.2<br>(186.4 to 219.1)	119<br>(103 to 132)	190.9<br>(164.1 to 211.0)	106<br>(92 to 118)	164.8<br>(142.9 to 182.7)	86<br>(74 to 97)	130.1<br>(110.9 to 145.9)	-36.3<br>(-43.7 to -28.6)	-31.8<br>(-34.9 to -28.7)
**Malta**	All Ages	86<br>(78 to 92)	23.2<br>(21.1 to 24.8)	176<br>(145 to 197)	39.9<br>(33.1 to 44.9)	158<br>(129 to 178)	35.8<br>(29.3 to 40.3)	155<br>(125 to 176)	35.0<br>(28.2 to 39.8)	51.0<br>(30.3 to 69.1)	-12.3<br>(-15.5 to -9.1)
	Under 5	2<br>(2 to 2)	6.8<br>(5.9 to 7.8)	1<br>(1 to 1)	3.4<br>(2.8 to 4.1)	1<br>(0 to 1)	2.3<br>(1.8 to 2.8)	0<br>(0 to 0)	1.7<br>(1.3 to 2.2)	-74.4<br>(-80.8 to -66.5)	-48.8<br>(-55.1 to -42.1)
	5-14 years	0<br>(0 to 0)	0.6<br>(0.5 to 0.7)	0<br>(0 to 0)	0.3<br>(0.3 to 0.4)	0<br>(0 to 0)	0.5<br>(0.4 to 0.6)	0<br>(0 to 0)	0.5<br>(0.4 to 0.6)	-15.4<br>(-31.3 to 7.9)	53.9<br>(35.9 to 75.6)
	15-49 years	3<br>(3 to 3)	1.6<br>(1.5 to 1.7)	2<br>(2 to 3)	1.3<br>(1.1 to 1.4)	2<br>(2 to 3)	1.1<br>(1.0 to 1.3)	2<br>(2 to 2)	1.0<br>(0.8 to 1.1)	-40.3<br>(-49.6 to -30.5)	-23.1<br>(-27.7 to -18.6)
	50-69 years	11<br>(10 to 13)	17.2<br>(15.7 to 18.8)	11<br>(10 to 12)	9.7<br>(8.7 to 10.8)	9<br>(8 to 10)	8.0<br>(7.1 to 8.9)	9<br>(8 to 10)	8.2<br>(7.2 to 9.1)	-52.6<br>(-59.0 to -45.1)	-16.2<br>(-20.7 to -11.4)
	70+ years	69<br>(62 to 75)	278.0<br>(248.7 to 301.0)	161<br>(131 to 182)	242.4<br>(196.9 to 274.3)	146<br>(118 to 165)	210.2<br>(169.6 to 237.3)	143<br>(114 to 163)	199.4<br>(158.5 to 227.6)	-28.3<br>(-38.0 to -19.6)	-17.7<br>(-20.8 to -14.7)
**Monaco**	All Ages	16<br>(12 to 19)	52.0<br>(38.9 to 64.0)	25<br>(19 to 30)	66.1<br>(50.2 to 80.6)	22<br>(17 to 27)	58.7<br>(45.1 to 71.8)	19<br>(15 to 24)	50.8<br>(39.3 to 62.4)	-2.2<br>(-23.3 to 31.7)	-23.1<br>(-27.4 to -18.5)
	Under 5	0<br>(0 to 0)	6.0<br>(4.0 to 8.6)	0<br>(0 to 0)	1.3<br>(0.9 to 1.9)	0<br>(0 to 0)	2.6<br>(1.9 to 3.3)	0<br>(0 to 0)	2.3<br>(1.8 to 3.0)	-61.0<br>(-75.0 to -39.7)	80.2<br>(28.4 to 152.6)
	5-14 years	0<br>(0 to 0)	0.6<br>(0.4 to 0.7)	0<br>(0 to 0)	0.2<br>(0.2 to 0.3)	0<br>(0 to 0)	0.4<br>(0.3 to 0.5)	0<br>(0 to 0)	0.3<br>(0.2 to 0.4)	-41.1<br>(-58.7 to -13.3)	39.5<br>(14.7 to 67.9)
	15-49 years	0<br>(0 to 0)	2.2<br>(1.7 to 2.8)	0<br>(0 to 0)	1.9<br>(1.4 to 2.6)	0<br>(0 to 0)	1.7<br>(1.2 to 2.3)	0<br>(0 to 0)	1.5<br>(1.0 to 2.0)	-32.4<br>(-55.9 to -1.9)	-23.1<br>(-32.0 to -14.9)
	50-69 years	1<br>(1 to 2)	14.7<br>(10.7 to 19.8)	1<br>(1 to 1)	10.1<br>(7.7 to 13.0)	1<br>(1 to 1)	9.2<br>(6.8 to 11.9)	1<br>(1 to 1)	8.3<br>(6.3 to 10.9)	-43.3<br>(-62.6 to -16.5)	-17.2<br>(-24.8 to -9.8)
	70+ years	14<br>(11 to 18)	287.0<br>(213.1 to 356.6)	23<br>(18 to 29)	333.5<br>(250.6 to 409.6)	21<br>(16 to 26)	294.0<br>(224.2 to 360.3)	18<br>(14 to 22)	253.0<br>(193.2 to 313.2)	-11.9<br>(-31.0 to 18.7)	-24.1<br>(-28.7 to -19.6)
**Netherlands**	All Ages	3,850<br>(3,360 to 4,160)	25.8<br>(22.5 to 27.9)	6,360<br>(5,260 to 7,020)	37.1<br>(30.7 to 41.0)	6,150<br>(5,180 to 6,950)	35.8<br>(30.2 to 40.5)	5,310<br>(4,430 to 5,850)	30.8<br>(25.7 to 34.0)	19.5<br>(10.1 to 27.7)	-16.9<br>(-19.2 to -14.2)
	Under 5	51<br>(46 to 56)	5.5<br>(5.0 to 6.0)	17<br>(15 to 18)	1.9<br>(1.7 to 2.1)	14<br>(11 to 16)	1.6<br>(1.3 to 1.9)	10<br>(8 to 11)	1.1<br>(1.0 to 1.3)	-79.2<br>(-82.8 to -75.1)	-41.1<br>(-47.8 to -33.6)
	5-14 years	10<br>(9 to 11)	0.6<br>(0.5 to 0.6)	4<br>(3 to 4)	0.2<br>(0.2 to 0.2)	4<br>(3 to 4)	0.2<br>(0.2 to 0.2)	3<br>(3 to 4)	0.2<br>(0.2 to 0.2)	-67.5<br>(-72.2 to -62.0)	-3.9<br>(-10.9 to 3.1)
	15-49 years	95<br>(90 to 101)	1.2<br>(1.1 to 1.2)	62<br>(58 to 67)	0.8<br>(0.8 to 0.9)	52<br>(47 to 58)	0.7<br>(0.6 to 0.8)	42<br>(39 to 45)	0.6<br>(0.5 to 0.6)	-51.5<br>(-56.0 to -46.8)	-31.0<br>(-33.4 to -28.2)

	50-69 years	245<br>(226 to 268)	8.7<br>(8.1 to 9.5)	387<br>(353 to 423)	8.4<br>(7.7 to 9.2)	339<br>(303 to 376)	7.3<br>(6.5 to 8.1)	297<br>(269 to 327)	6.4<br>(5.8 to 7.0)	-27.1<br>(-35.9 to -17.4)	-24.5<br>(-27.4 to -21.2)
	70+ years	3,450<br>(2,970 to 3,750)	268.8<br>(231.5 to 292.4)	5,890<br>(4,810 to 6,530)	252.9<br>(206.5 to 280.3)	5,740<br>(4,760 to 6,510)	239.4<br>(198.5 to 271.6)	4,950<br>(4,090 to 5,490)	201.7<br>(166.6 to 223.4)	-25.0<br>(-31.0 to -19.6)	-20.3<br>(-22.5 to -17.5)
**Norway**	All Ages	2,880<br>(2,510 to 3,070)	67.7<br>(59.0 to 72.3)	2,020<br>(1,650 to 2,230)	37.9<br>(30.9 to 41.7)	1,760<br>(1,460 to 1,980)	32.6<br>(27.0 to 36.7)	1,750<br>(1,440 to 1,940)	32.4<br>(26.5 to 35.8)	-52.2<br>(-55.9 to -49.3)	-14.5<br>(-16.5 to -12.2)
	Under 5	13<br>(12 to 13)	4.5<br>(4.2 to 4.9)	2<br>(2 to 2)	0.7<br>(0.6 to 0.8)	1<br>(1 to 2)	0.5<br>(0.4 to 0.5)	1<br>(1 to 1)	0.4<br>(0.4 to 0.5)	-90.3<br>(-91.9 to -88.6)	-39.9<br>(-47.2 to -31.7)
	5-14 years	2<br>(2 to 2)	0.4<br>(0.4 to 0.5)	1<br>(1 to 1)	0.1<br>(0.1 to 0.1)	1<br>(1 to 1)	0.1<br>(0.1 to 0.1)	0<br>(0 to 0)	0.1<br>(0.1 to 0.1)	-83.8<br>(-85.2 to -82.0)	-23.0<br>(-27.9 to -17.8)
	15-49 years	27<br>(26 to 28)	1.2<br>(1.2 to 1.3)	10<br>(9 to 10)	0.4<br>(0.4 to 0.4)	9<br>(8 to 10)	0.4<br>(0.3 to 0.4)	8<br>(8 to 8)	0.3<br>(0.3 to 0.3)	-74.5<br>(-76.1 to -72.8)	-20.1<br>(-22.7 to -17.4)
	50-69 years	137<br>(130 to 143)	17.2<br>(16.5 to 18.1)	71<br>(66 to 75)	5.6<br>(5.2 to 5.9)	61<br>(57 to 66)	4.8<br>(4.4 to 5.1)	59<br>(55 to 63)	4.5<br>(4.2 to 4.8)	-73.7<br>(-75.6 to -71.7)	-18.6<br>(-21.2 to -15.6)
	70+ years	2,700<br>(2,330 to 2,890)	551.9<br>(477.2 to 591.7)	1,940<br>(1,580 to 2,140)	297.9<br>(241.8 to 328.9)	1,680<br>(1,390 to 1,900)	250.4<br>(206.0 to 282.5)	1,690<br>(1,370 to 1,870)	243.5<br>(198.0 to 270.4)	-55.9<br>(-59.4 to -53.2)	-18.3<br>(-20.2 to -16.1)
**Portugal**	All Ages	3,170<br>(2,950 to 3,370)	31.3<br>(29.1 to 33.3)	7,250<br>(6,010 to 7,980)	67.9<br>(56.3 to 74.8)	6,980<br>(5,810 to 7,880)	65.6<br>(54.6 to 74.1)	6,000<br>(4,900 to 6,670)	56.5<br>(46.2 to 62.8)	80.7<br>(56.1 to 99.1)	-16.8<br>(-19.4 to -14.0)
	Under 5	96<br>(88 to 104)	16.6<br>(15.1 to 18.0)	13<br>(11 to 14)	3.0<br>(2.6 to 3.3)	9<br>(7 to 11)	2.1<br>(1.6 to 2.5)	5<br>(4 to 7)	1.3<br>(1.0 to 1.5)	-92.3<br>(-93.8 to -90.5)	-56.4<br>(-63.5 to -49.0)
	5-14 years	23<br>(20 to 25)	1.5<br>(1.3 to 1.6)	4<br>(3 to 4)	0.4<br>(0.3 to 0.4)	3<br>(2 to 3)	0.3<br>(0.3 to 0.3)	3<br>(3 to 4)	0.3<br>(0.3 to 0.4)	-76.8<br>(-80.5 to -72.3)	-7.7<br>(-16.9 to 1.0)
	15-49 years	163<br>(151 to 176)	3.3<br>(3.0 to 3.5)	87<br>(79 to 96)	1.9<br>(1.7 to 2.0)	76<br>(67 to 86)	1.6<br>(1.5 to 1.8)	64<br>(58 to 71)	1.4<br>(1.3 to 1.6)	-57.1<br>(-62.2 to -51.3)	-24.6<br>(-27.7 to -21.2)
	50-69 years	457<br>(423 to 495)	21.1<br>(19.6 to 22.9)	431<br>(395 to 471)	15.1<br>(13.8 to 16.5)	392<br>(353 to 432)	13.6<br>(12.3 to 15.0)	352<br>(322 to 385)	12.2<br>(11.1 to 13.3)	-42.5<br>(-49.0 to -35.0)	-19.3<br>(-22.7 to -15.5)
	70+ years	2,430<br>(2,210 to 2,630)	283.0<br>(257.6 to 305.6)	6,710<br>(5,500 to 7,440)	392.6<br>(321.9 to 434.9)	6,500<br>(5,360 to 7,370)	373.7<br>(307.7 to 423.2)	5,570<br>(4,500 to 6,220)	314.8<br>(254.2 to 351.8)	11.2<br>(-4.2 to 23.8)	-19.8<br>(-22.4 to -17.1)
**San Marino**	All Ages	5<br>(4 to 5)	19.0<br>(15.4 to 22.1)	9<br>(7 to 11)	26.9<br>(20.6 to 32.9)	5<br>(3 to 6)	14.6<br>(10.1 to 19.0)	4<br>(3 to 6)	12.4<br>(7.8 to 16.9)	-35.0<br>(-54.8 to -11.3)	-54.0<br>(-63.9 to -43.9)
	Under 5	0<br>(0 to 0)	5.6<br>(4.0 to 7.9)	0<br>(0 to 0)	0.9<br>(0.6 to 1.3)	0<br>(0 to 0)	0.7<br>(0.5 to 1.0)	0<br>(0 to 0)	0.4<br>(0.2 to 0.7)	-92.5<br>(-95.9 to -87.0)	-54.3<br>(-67.2 to -41.5)
	5-14 years	0<br>(0 to 0)	0.3<br>(0.2 to 0.4)	0<br>(0 to 0)	0.1<br>(0.1 to 0.1)	0<br>(0 to 0)	0.1<br>(0.1 to 0.1)	0<br>(0 to 0)	0.1<br>(0.0 to 0.1)	-77.2<br>(-85.1 to -65.0)	-27.1<br>(-36.2 to -17.8)
	15-49 years	0<br>(0 to 0)	0.7<br>(0.6 to 0.9)	0<br>(0 to 0)	0.5<br>(0.4 to 0.7)	0<br>(0 to 0)	0.3<br>(0.2 to 0.5)	0<br>(0 to 0)	0.2<br>(0.1 to 0.4)	-65.5<br>(-83.0 to -42.9)	-50.7<br>(-70.5 to -31.9)

	50-69 years	0<br>(0 to 0)	6.7<br>(5.2 to 8.5)	0<br>(0 to 0)	3.6<br>(2.7 to 4.6)	0<br>(0 to 0)	2.3<br>(1.4 to 3.3)	0<br>(0 to 0)	1.9<br>(1.0 to 2.9)	-71.8<br>(-86.0 to -54.1)	-47.2<br>(-67.7 to -27.7)
	70+ years	4<br>(3 to 5)	172.2<br>(138.7 to 202.5)	8<br>(6 to 10)	166.6<br>(127.3 to 204.7)	4<br>(3 to 6)	87.6<br>(60.1 to 115.4)	4<br>(2 to 5)	73.5<br>(47.0 to 100.9)	-57.3<br>(-70.3 to -42.0)	-55.9<br>(-65.3 to -46.3)
**Spain**	All Ages	8,700<br>(7,780 to 9,310)	22.4<br>(20.1 to 24.0)	14,400<br>(11,800 to 16,000)	31.3<br>(25.6 to 34.8)	13,200<br>(11,000 to 14,800)	28.9<br>(24.1 to 32.3)	11,300<br>(9,130 to 12,700)	24.8<br>(20.0 to 27.8)	10.8<br>(-2.2 to 21.3)	-20.7<br>(-23.1 to -18.2)
	Under 5	111<br>(103 to 120)	5.4<br>(5.0 to 5.8)	23<br>(21 to 25)	1.2<br>(1.0 to 1.3)	15<br>(13 to 18)	0.8<br>(0.7 to 0.9)	12<br>(10 to 13)	0.6<br>(0.5 to 0.7)	-88.2<br>(-90.1 to -86.1)	-45.2<br>(-51.3 to -38.8)
	5-14 years	32<br>(29 to 35)	0.6<br>(0.5 to 0.6)	8<br>(7 to 9)	0.2<br>(0.2 to 0.2)	6<br>(6 to 7)	0.1<br>(0.1 to 0.2)	7<br>(6 to 8)	0.2<br>(0.1 to 0.2)	-72.5<br>(-76.5 to -67.7)	-10.9<br>(-16.2 to -5.1)
	15-49 years	385<br>(362 to 409)	2.0<br>(1.9 to 2.1)	175<br>(159 to 191)	0.8<br>(0.8 to 0.9)	135<br>(122 to 149)	0.7<br>(0.6 to 0.7)	128<br>(117 to 142)	0.6<br>(0.6 to 0.7)	-67.6<br>(-71.2 to -63.9)	-24.0<br>(-26.7 to -20.7)
	50-69 years	958<br>(886 to 1,040)	11.9<br>(11.0 to 12.8)	822<br>(753 to 898)	6.9<br>(6.3 to 7.5)	695<br>(633 to 763)	5.7<br>(5.2 to 6.3)	686<br>(627 to 753)	5.6<br>(5.1 to 6.1)	-53.0<br>(-58.3 to -47.2)	-19.0<br>(-22.0 to -15.9)
	70+ years	7,210<br>(6,320 to 7,790)	209.3<br>(183.3 to 225.9)	13,400<br>(10,800 to 15,000)	202.0<br>(162.6 to 225.7)	12,400<br>(10,200 to 13,900)	184.4<br>(151.8 to 206.9)	10,500<br>(8,300 to 11,800)	154.9<br>(122.7 to 174.4)	-26.0<br>(-34.6 to -18.7)	-23.3<br>(-25.7 to -20.7)
**Sweden**	All Ages	4,060<br>(3,590 to 4,360)	47.3<br>(41.8 to 50.7)	2,540<br>(2,100 to 2,810)	24.8<br>(20.6 to 27.5)	2,250<br>(1,870 to 2,480)	21.8<br>(18.1 to 24.1)	2,050<br>(1,660 to 2,370)	19.8<br>(16.0 to 22.8)	-58.1<br>(-63.9 to -52.4)	-20.1<br>(-28.1 to -11.7)
	Under 5	24<br>(22 to 26)	4.3<br>(4.0 to 4.7)	5<br>(4 to 5)	0.8<br>(0.7 to 0.9)	4<br>(3 to 4)	0.6<br>(0.5 to 0.7)	3<br>(2 to 3)	0.5<br>(0.4 to 0.6)	-89.0<br>(-91.2 to -86.3)	-42.2<br>(-51.9 to -29.0)
	5-14 years	3<br>(3 to 3)	0.3<br>(0.3 to 0.4)	1<br>(1 to 1)	0.1<br>(0.1 to 0.1)	1<br>(1 to 1)	0.1<br>(0.1 to 0.1)	1<br>(1 to 1)	0.1<br>(0.1 to 0.1)	-79.5<br>(-82.8 to -75.5)	-16.6<br>(-26.1 to -4.5)
	15-49 years	79<br>(74 to 83)	1.9<br>(1.8 to 2.0)	25<br>(23 to 27)	0.6<br>(0.5 to 0.6)	19<br>(18 to 21)	0.4<br>(0.4 to 0.5)	17<br>(14 to 20)	0.4<br>(0.3 to 0.4)	-79.9<br>(-83.0 to -76.4)	-32.6<br>(-41.7 to -23.5)
	50-69 years	240<br>(225 to 258)	13.7<br>(12.8 to 14.7)	160<br>(147 to 173)	6.7<br>(6.1 to 7.2)	130<br>(119 to 141)	5.4<br>(4.9 to 5.9)	117<br>(99 to 137)	4.8<br>(4.1 to 5.7)	-64.9<br>(-70.5 to -57.7)	-27.6<br>(-37.4 to -17.5)
	70+ years	3,710<br>(3,250 to 4,000)	340.1<br>(297.5 to 366.5)	2,340<br>(1,910 to 2,610)	152.0<br>(123.9 to 169.4)	2,090<br>(1,720 to 2,330)	133.2<br>(109.3 to 148.1)	1,920<br>(1,540 to 2,220)	120.1<br>(96.3 to 138.9)	-64.7<br>(-69.6 to -60.0)	-20.9<br>(-28.6 to -12.6)
**Switzerland**	All Ages	2,550<br>(2,230 to 2,750)	37.2<br>(32.4 to 40.0)	1,970<br>(1,550 to 2,210)	22.4<br>(17.7 to 25.1)	1,910<br>(1,550 to 2,130)	21.6<br>(17.6 to 24.0)	1,590<br>(1,240 to 1,790)	17.8<br>(13.9 to 20.1)	-52.0<br>(-57.6 to -47.7)	-20.5<br>(-22.7 to -18.3)
	Under 5	23<br>(20 to 25)	5.7<br>(5.1 to 6.3)	5<br>(4 to 5)	1.1<br>(0.9 to 1.2)	4<br>(3 to 4)	0.9<br>(0.7 to 1.0)	3<br>(2 to 3)	0.6<br>(0.5 to 0.8)	-88.7<br>(-91.1 to -86.0)	-39.8<br>(-49.1 to -30.4)
	5-14 years	3<br>(3 to 3)	0.4<br>(0.4 to 0.4)	1<br>(1 to 1)	0.1<br>(0.1 to 0.2)	1<br>(1 to 1)	0.1<br>(0.1 to 0.1)	1<br>(1 to 1)	0.1<br>(0.1 to 0.1)	-71.8<br>(-75.8 to -67.3)	-23.8<br>(-29.8 to -17.7)
	15-49 years	46<br>(43 to 50)	1.3<br>(1.2 to 1.4)	20<br>(18 to 22)	0.5<br>(0.5 to 0.5)	15<br>(14 to 17)	0.4<br>(0.4 to 0.4)	14<br>(13 to 15)	0.3<br>(0.3 to 0.4)	-73.0<br>(-75.8 to -70.0)	-30.5<br>(-33.1 to -27.9)

	50-69 years	177<br>(162 to 193)	12.8<br>(11.7 to 14.0)	96<br>(87 to 106)	4.2<br>(3.8 to 4.7)	79<br>(71 to 88)	3.4<br>(3.1 to 3.8)	78<br>(71 to 86)	3.4<br>(3.0 to 3.7)	-73.7<br>(-77.1 to -70.2)	-20.6<br>(-23.8 to -17.1)
	70+ years	2,300<br>(1,990 to 2,500)	330.2<br>(285.2 to 357.6)	1,850<br>(1,440 to 2,080)	153.8<br>(119.7 to 173.1)	1,810<br>(1,460 to 2,030)	147.8<br>(118.9 to 165.3)	1,500<br>(1,150 to 1,690)	119.9<br>(92.3 to 135.7)	-63.7<br>(-68.0 to -60.4)	-22.0<br>(-24.2 to -19.9)
**United Kingdom**	All Ages	33,200<br>(29,900 to 34,900)	58.0<br>(52.2 to 60.9)	40,700<br>(34,800 to 43,600)	60.6<br>(51.7 to 64.9)	35,600<br>(30,800 to 38,200)	52.7<br>(45.6 to 56.6)	30,100<br>(25,200 to 32,500)	44.4<br>(37.2 to 47.9)	-23.5<br>(-28.5 to -20.3)	-26.7<br>(-28.2 to -25.3)
	Under 5	287<br>(276 to 299)	7.5<br>(7.2 to 7.8)	107<br>(101 to 112)	2.8<br>(2.7 to 2.9)	63<br>(57 to 69)	1.7<br>(1.5 to 1.8)	54<br>(45 to 62)	1.5<br>(1.2 to 1.7)	-80.3<br>(-83.7 to -77.2)	-47.6<br>(-56.7 to -39.4)
	5-14 years	40<br>(39 to 41)	0.6<br>(0.6 to 0.6)	23<br>(23 to 24)	0.3<br>(0.3 to 0.3)	14<br>(13 to 15)	0.2<br>(0.2 to 0.2)	15<br>(14 to 16)	0.2<br>(0.2 to 0.2)	-66.7<br>(-68.8 to -64.8)	-34.9<br>(-39.0 to -31.2)
	15-49 years	604<br>(595 to 612)	2.1<br>(2.1 to 2.2)	633<br>(624 to 643)	2.1<br>(2.1 to 2.1)	504<br>(488 to 524)	1.7<br>(1.6 to 1.7)	447<br>(435 to 459)	1.5<br>(1.4 to 1.5)	-30.6<br>(-32.4 to -28.6)	-29.4<br>(-31.2 to -27.8)
	50-69 years	2,400<br>(2,340 to 2,460)	20.6<br>(20.0 to 21.1)	2,770<br>(2,690 to 2,820)	17.1<br>(16.6 to 17.4)	2,360<br>(2,290 to 2,440)	14.4<br>(14.0 to 14.9)	2,160<br>(2,090 to 2,230)	13.0<br>(12.6 to 13.4)	-36.7<br>(-38.9 to -34.3)	-23.7<br>(-25.5 to -22.0)
	70+ years	29,900<br>(26,500 to 31,500)	480.6<br>(426.5 to 506.3)	37,200<br>(31,400 to 40,000)	419.3<br>(353.4 to 451.5)	32,600<br>(27,900 to 35,300)	360.7<br>(308.0 to 389.7)	27,400<br>(22,700 to 29,800)	299.8<br>(247.7 to 325.7)	-37.6<br>(-42.0 to -34.9)	-28.5<br>(-29.9 to -27.0)
**Bolivia (Plurinational State of)**	All Ages	9,160<br>(7,370 to 11,100)	143.6<br>(115.5 to 173.7)	6,300<br>(5,120 to 7,830)	54.6<br>(44.3 to 67.9)	5,070<br>(4,100 to 6,340)	43.4<br>(35.1 to 54.3)	4,680<br>(3,750 to 5,870)	39.7<br>(31.8 to 49.7)	-72.4<br>(-79.3 to -63.0)	-27.3<br>(-31.7 to -22.1)
	Under 5	6,470<br>(4,770 to 8,270)	640.6<br>(472.3 to 818.6)	1,230<br>(902 to 1,590)	101.7<br>(74.7 to 131.5)	841<br>(624 to 1,090)	70.0<br>(52.0 to 90.9)	747<br>(551 to 975)	62.5<br>(46.2 to 81.6)	-90.2<br>(-92.8 to -86.6)	-38.5<br>(-45.8 to -30.8)
	5-14 years	248<br>(191 to 312)	14.8<br>(11.4 to 18.6)	110<br>(81 to 147)	4.9<br>(3.6 to 6.5)	87<br>(64 to 116)	3.8<br>(2.8 to 5.1)	81<br>(60 to 112)	3.6<br>(2.6 to 4.9)	-76.0<br>(-83.7 to -64.1)	-27.6<br>(-35.8 to -18.8)
	15-49 years	374<br>(301 to 454)	12.5<br>(10.1 to 15.2)	397<br>(280 to 547)	6.5<br>(4.6 to 9.0)	344<br>(247 to 467)	5.6<br>(4.0 to 7.6)	343<br>(246 to 466)	5.5<br>(3.9 to 7.4)	-56.2<br>(-70.1 to -37.8)	-15.8<br>(-23.8 to -6.6)
	50-69 years	614<br>(488 to 760)	110.7<br>(88.1 to 136.9)	1,000<br>(721 to 1,340)	65.7<br>(47.3 to 88.2)	891<br>(633 to 1,190)	57.0<br>(40.5 to 76.3)	877<br>(625 to 1,180)	55.1<br>(39.3 to 74.2)	-50.2<br>(-65.9 to -28.1)	-16.1<br>(-23.8 to -6.9)
	70+ years	1,460<br>(1,240 to 1,730)	970.6<br>(824.9 to 1,149.6)	3,560<br>(2,880 to 4,430)	775.0<br>(626.6 to 964.1)	2,910<br>(2,360 to 3,660)	626.3<br>(507.1 to 786.9)	2,630<br>(2,120 to 3,300)	568.6<br>(457.5 to 712.6)	-41.4<br>(-54.3 to -25.4)	-26.6<br>(-31.6 to -20.6)
**Ecuador**	All Ages	4,530<br>(4,280 to 4,750)	45.4<br>(42.9 to 47.6)	5,930<br>(5,340 to 6,540)	33.9<br>(30.6 to 37.4)	5,040<br>(4,170 to 5,990)	28.2<br>(23.4 to 33.6)	4,530<br>(3,790 to 5,390)	25.1<br>(21.0 to 29.8)	-44.8<br>(-53.6 to -34.2)	-26.1<br>(-35.3 to -15.0)
	Under 5	1,990<br>(1,810 to 2,160)	147.7<br>(134.6 to 160.8)	669<br>(512 to 853)	39.8<br>(30.5 to 50.7)	493<br>(361 to 669)	29.3<br>(21.5 to 39.8)	383<br>(266 to 532)	23.0<br>(16.0 to 32.0)	-84.4<br>(-89.3 to -78.0)	-42.1<br>(-54.8 to -27.1)
	5-14 years	162<br>(147 to 178)	6.4<br>(5.8 to 7.1)	120<br>(104 to 138)	3.6<br>(3.1 to 4.1)	83<br>(70 to 98)	2.4<br>(2.1 to 2.9)	72<br>(62 to 85)	2.1<br>(1.8 to 2.5)	-67.1<br>(-72.5 to -60.2)	-40.9<br>(-47.4 to -33.4)
	15-49 years	289<br>(273 to 306)	5.8<br>(5.5 to 6.2)	447<br>(396 to 502)	4.9<br>(4.4 to 5.5)	361<br>(288 to 446)	3.9<br>(3.1 to 4.8)	338<br>(269 to 428)	3.6<br>(2.9 to 4.6)	-38.2<br>(-51.2 to -21.3)	-27.1<br>(-39.7 to -13.0)

	50-69 years	345 (322 to 370)	38.8 (36.3 to 41.7)	784 (672 to 900)	31.0 (26.6 to 35.6)	751 (581 to 945)	28.8 (22.3 to 36.2)	710 (540 to 924)	26.6 (20.2 to 34.6)	-31.4 (-49.4 to -9.3)	-14.0 (-30.5 to 7.9)
	70+ years	1,740 (1,600 to 1,840)	644.0 (591.8 to 680.3)	3,910 (3,460 to 4,300)	437.2 (387.3 to 480.5)	3,350 (2,760 to 4,000)	366.9 (302.5 to 438.3)	3,020 (2,540 to 3,610)	323.5 (272.2 to 386.4)	-49.8 (-57.8 to -40.0)	-26.0 (-35.7 to -14.8)
**Peru**	All Ages	20,000 (18,000 to 22,200)	92.7 (83.4 to 102.7)	25,000 (21,000 to 28,700)	71.6 (60.4 to 82.3)	18,900 (15,700 to 22,400)	53.1 (43.9 to 62.7)	20,300 (15,900 to 25,700)	56.1 (43.7 to 70.8)	-39.5 (-52.6 to -23.7)	-21.7 (-35.9 to -6.8)
	Under 5	11,000 (9,380 to 12,900)	374.8 (320.7 to 441.0)	1,650 (1,210 to 2,230)	51.1 (37.5 to 69.2)	974 (680 to 1,340)	29.7 (20.8 to 41.0)	1,060 (647 to 1,550)	32.2 (19.6 to 47.1)	-91.4 (-94.9 to -86.9)	-36.9 (-58.6 to -13.7)
	5-14 years	575 (478 to 677)	10.7 (8.9 to 12.6)	298 (243 to 367)	5.0 (4.0 to 6.1)	261 (208 to 326)	4.2 (3.4 to 5.3)	237 (186 to 301)	3.8 (3.0 to 4.8)	-64.4 (-73.2 to -52.8)	-23.2 (-37.1 to -8.1)
	15-49 years	1,210 (1,030 to 1,400)	11.3 (9.7 to 13.1)	1,610 (1,320 to 1,940)	8.7 (7.1 to 10.5)	1,220 (999 to 1,480)	6.4 (5.3 to 7.8)	1,370 (1,050 to 1,770)	7.1 (5.4 to 9.1)	-37.4 (-52.9 to -16.4)	-18.3 (-35.7 to 1.3)
	50-69 years	1,620 (1,360 to 1,910)	79.2 (66.8 to 93.7)	3,260 (2,600 to 4,030)	62.5 (49.8 to 77.2)	2,810 (2,200 to 3,520)	52.2 (40.9 to 65.4)	3,090 (2,300 to 4,120)	56.0 (41.7 to 74.7)	-29.3 (-49.1 to -1.2)	-10.3 (-29.6 to 11.4)
	70+ years	5,680 (4,850 to 6,560)	946.6 (809.3 to 1,094.5)	18,100 (15,000 to 21,100)	983.2 (813.8 to 1,142.8)	13,700 (11,300 to 16,300)	726.7 (597.8 to 865.4)	14,600 (11,300 to 18,500)	773.8 (599.3 to 983.3)	-18.3 (-35.8 to 5.0)	-21.3 (-36.0 to -6.1)
**Antigua and Barbuda**	All Ages	22 (20 to 23)	35.8 (32.9 to 38.5)	31 (28 to 34)	35.1 (31.8 to 38.7)	28 (25 to 31)	31.2 (27.9 to 34.9)	25 (23 to 27)	27.8 (25.8 to 29.9)	-22.2 (-29.0 to -14.1)	-20.7 (-29.3 to -10.9)
	Under 5	1 (1 to 2)	21.9 (17.8 to 26.5)	1 (1 to 2)	23.0 (18.5 to 28.4)	1 (1 to 1)	19.3 (15.6 to 23.7)	1 (1 to 1)	15.5 (12.9 to 18.3)	-29.1 (-44.6 to -8.0)	-32.4 (-43.1 to -23.1)
	5-14 years	0 (0 to 0)	1.0 (0.8 to 1.1)	0 (0 to 0)	1.1 (0.9 to 1.3)	0 (0 to 0)	0.8 (0.6 to 1.0)	0 (0 to 0)	0.8 (0.7 to 0.9)	-21.7 (-35.9 to -3.8)	-29.7 (-38.0 to -21.1)
	15-49 years	1 (1 to 1)	3.4 (3.1 to 3.7)	2 (2 to 2)	3.5 (3.1 to 4.0)	2 (1 to 2)	3.4 (3.0 to 3.9)	1 (1 to 1)	2.2 (2.0 to 2.4)	-36.0 (-43.8 to -26.9)	-38.3 (-44.4 to -30.4)
	50-69 years	2 (2 to 3)	35.2 (31.6 to 38.9)	5 (5 to 6)	29.4 (26.0 to 33.5)	5 (4 to 5)	24.4 (21.2 to 28.2)	5 (4 to 5)	25.0 (22.9 to 27.5)	-28.8 (-38.2 to -18.2)	-14.7 (-23.4 to -5.5)
	70+ years	17 (15 to 18)	455.9 (413.0 to 495.1)	23 (20 to 25)	435.4 (390.1 to 479.4)	20 (18 to 23)	376.6 (334.5 to 422.0)	18 (16 to 20)	321.5 (291.2 to 353.8)	-29.5 (-37.1 to -20.5)	-26.2 (-34.8 to -15.5)
**Bahamas**	All Ages	62 (57 to 67)	24.1 (22.2 to 26.0)	99 (84 to 116)	25.8 (21.8 to 30.3)	91 (75 to 108)	23.4 (19.3 to 27.9)	80 (66 to 97)	20.6 (16.9 to 24.9)	-14.5 (-30.3 to 4.5)	-20.2 (-25.4 to -15.5)
	Under 5	12 (10 to 14)	46.7 (38.3 to 56.3)	4 (3 to 6)	20.2 (14.3 to 27.9)	3 (2 to 5)	16.3 (11.4 to 22.7)	3 (2 to 4)	13.0 (9.3 to 17.8)	-72.1 (-80.6 to -59.7)	-35.4 (-42.5 to -27.7)
	5-14 years	1 (1 to 1)	1.5 (1.3 to 1.8)	1 (0 to 1)	0.9 (0.7 to 1.0)	1 (0 to 1)	0.9 (0.7 to 1.1)	0 (0 to 1)	0.8 (0.6 to 0.9)	-51.1 (-62.4 to -36.6)	-11.3 (-19.8 to -2.3)
	15-49 years	9 (8 to 10)	6.4 (5.8 to 7.0)	11 (9 to 14)	5.4 (4.3 to 6.7)	10 (8 to 13)	4.9 (3.9 to 6.1)	9 (7 to 12)	4.4 (3.4 to 5.6)	-30.9 (-46.8 to -10.2)	-18.7 (-25.8 to -12.1)

	50-69 years	12<br>(11 to 13)	46.4<br>(41.5 to 51.2)	20<br>(16 to 24)	27.0<br>(21.9 to 32.7)	19<br>(15 to 24)	25.1<br>(19.9 to 31.2)	18<br>(14 to 23)	22.9<br>(18.1 to 29.0)	-50.6<br>(-61.9 to -36.1)	-15.0<br>(-22.2 to -8.0)
	70+ years	28<br>(25 to 31)	347.4<br>(311.1 to 380.8)	63<br>(54 to 73)	343.4<br>(293.2 to 393.8)	57<br>(48 to 67)	299.4<br>(250.9 to 349.9)	50<br>(42 to 58)	256.1<br>(214.7 to 301.8)	-26.3<br>(-39.3 to -11.1)	-25.4<br>(-29.8 to -21.2)
**Barbados**	All Ages	84<br>(78 to 89)	33.1<br>(30.6 to 35.1)	160<br>(136 to 184)	53.9<br>(45.6 to 62.0)	139<br>(116 to 164)	46.8<br>(38.9 to 55.1)	124<br>(100 to 147)	41.5<br>(33.5 to 49.3)	25.2<br>(2.0 to 49.9)	-23.1<br>(-30.0 to -16.7)
	Under 5	5<br>(4 to 6)	24.9<br>(20.4 to 29.6)	2<br>(2 to 3)	15.1<br>(11.1 to 20.0)	2<br>(1 to 2)	12.5<br>(8.9 to 17.3)	1<br>(1 to 2)	9.9<br>(6.8 to 14.0)	-60.0<br>(-72.4 to -44.0)	-34.1<br>(-44.0 to -23.8)
	5-14 years	1<br>(1 to 1)	1.4<br>(1.2 to 1.6)	0<br>(0 to 0)	0.8<br>(0.7 to 1.0)	0<br>(0 to 0)	0.6<br>(0.4 to 0.8)	0<br>(0 to 0)	0.5<br>(0.4 to 0.7)	-61.4<br>(-74.6 to -45.7)	-35.4<br>(-52.4 to -21.8)
	15-49 years	5<br>(4 to 5)	3.4<br>(3.1 to 3.6)	5<br>(4 to 6)	3.2<br>(2.6 to 4.0)	4<br>(3 to 5)	2.8<br>(2.2 to 3.5)	3<br>(2 to 4)	2.3<br>(1.8 to 3.0)	-31.1<br>(-47.7 to -8.8)	-28.6<br>(-37.1 to -20.0)
	50-69 years	10<br>(9 to 10)	27.2<br>(24.8 to 29.7)	20<br>(16 to 25)	26.1<br>(20.8 to 31.5)	18<br>(14 to 23)	23.3<br>(18.0 to 28.6)	16<br>(12 to 21)	20.0<br>(14.9 to 25.6)	-26.4<br>(-46.1 to -4.7)	-23.3<br>(-32.0 to -15.0)
	70+ years	64<br>(59 to 69)	301.5<br>(275.4 to 323.2)	133<br>(112 to 153)	439.7<br>(370.7 to 505.3)	115<br>(96 to 135)	370.9<br>(309.4 to 435.9)	103<br>(84 to 122)	321.9<br>(261.5 to 379.9)	6.8<br>(-11.9 to 26.8)	-26.8<br>(-33.1 to -20.9)
**Belize**	All Ages	63<br>(59 to 69)	34.0<br>(31.3 to 36.9)	110<br>(100 to 120)	26.7<br>(24.2 to 29.1)	97<br>(87 to 108)	23.0<br>(20.7 to 25.7)	89<br>(79 to 100)	20.8<br>(18.3 to 23.2)	-38.8<br>(-46.5 to -30.7)	-22.2<br>(-27.5 to -17.1)
	Under 5	31<br>(28 to 36)	106.1<br>(93.8 to 120.8)	14<br>(11 to 17)	35.6<br>(29.1 to 43.4)	11<br>(9 to 14)	30.0<br>(24.1 to 37.1)	9<br>(7 to 11)	23.9<br>(19.1 to 29.7)	-77.5<br>(-82.7 to -71.3)	-33.0<br>(-39.1 to -26.6)
	5-14 years	2<br>(1 to 2)	3.2<br>(2.8 to 3.6)	2<br>(1 to 2)	1.8<br>(1.6 to 2.1)	1<br>(1 to 1)	1.2<br>(1.0 to 1.4)	1<br>(1 to 1)	1.0<br>(0.8 to 1.1)	-70.0<br>(-74.9 to -64.0)	-48.2<br>(-51.7 to -44.4)
	15-49 years	3<br>(3 to 3)	3.7<br>(3.4 to 4.1)	14<br>(12 to 15)	6.1<br>(5.4 to 6.8)	11<br>(10 to 13)	4.9<br>(4.3 to 5.5)	10<br>(9 to 12)	4.4<br>(3.9 to 5.1)	18.2<br>(-0.1 to 38.0)	-27.2<br>(-33.4 to -20.9)
	50-69 years	5<br>(5 to 5)	33.9<br>(31.0 to 37.0)	18<br>(16 to 20)	36.0<br>(32.0 to 40.3)	17<br>(15 to 19)	32.8<br>(29.2 to 36.8)	17<br>(14 to 19)	30.6<br>(26.4 to 35.0)	-9.9<br>(-24.0 to 5.3)	-15.0<br>(-22.2 to -7.5)
	70+ years	22<br>(20 to 24)	423.5<br>(373.2 to 460.0)	63<br>(55 to 70)	492.2<br>(431.9 to 542.4)	56<br>(49 to 63)	417.4<br>(364.7 to 470.9)	52<br>(45 to 59)	372.4<br>(322.8 to 419.9)	-12.1<br>(-22.6 to -1.7)	-24.3<br>(-30.0 to -19.4)
**Bermuda**	All Ages	12<br>(11 to 12)	19.5<br>(17.9 to 20.8)	19<br>(16 to 23)	30.2<br>(25.6 to 36.0)	18<br>(15 to 21)	28.4<br>(23.9 to 33.6)	18<br>(15 to 22)	28.3<br>(23.5 to 34.6)	45.2<br>(23.8 to 78.0)	-6.3<br>(-10.6 to -0.7)
	Under 5	1<br>(0 to 1)	11.9<br>(9.8 to 14.2)	0<br>(0 to 0)	4.3<br>(3.0 to 5.6)	0<br>(0 to 0)	3.7<br>(2.5 to 5.0)	0<br>(0 to 0)	3.5<br>(2.4 to 4.4)	-70.8<br>(-79.5 to -60.4)	-18.6<br>(-27.7 to -7.3)
	5-14 years	0<br>(0 to 0)	0.4<br>(0.3 to 0.4)	0<br>(0 to 0)	0.2<br>(0.2 to 0.3)	0<br>(0 to 0)	0.2<br>(0.2 to 0.3)	0<br>(0 to 0)	0.2<br>(0.2 to 0.2)	-46.9<br>(-59.8 to -32.9)	-20.7<br>(-26.5 to -14.5)
	15-49 years	1<br>(1 to 1)	1.9<br>(1.7 to 2.1)	0<br>(0 to 0)	1.3<br>(1.1 to 1.6)	0<br>(0 to 0)	1.2<br>(1.0 to 1.4)	0<br>(0 to 0)	1.2<br>(1.0 to 1.5)	-37.0<br>(-50.5 to -20.4)	-11.2<br>(-17.6 to -4.4)

	50-69 years	2<br>(2 to 2)	16.9<br>(15.2 to 18.6)	2<br>(2 to 2)	9.6<br>(8.0 to 11.4)	2<br>(1 to 2)	8.7<br>(7.2 to 10.5)	2<br>(1 to 2)	8.8<br>(7.1 to 11.0)	-47.8<br>(-58.5 to -34.3)	-8.3<br>(-14.6 to -1.2)
	70+ years	9<br>(8 to 9)	249.8<br>(226.9 to 268.5)	17<br>(14 to 20)	201.0<br>(167.9 to 240.0)	16<br>(13 to 19)	183.7<br>(153.4 to 217.9)	16<br>(13 to 19)	176.1<br>(145.6 to 215.4)	-29.5<br>(-39.6 to -13.9)	-12.3<br>(-16.4 to -6.9)
**Cuba**	All Ages	3,210<br>(3,020 to 3,360)	29.6<br>(27.9 to 31.0)	8,130<br>(7,430 to 8,610)	71.5<br>(65.4 to 75.8)	7,710<br>(6,940 to 8,500)	68.1<br>(61.2 to 75.0)	6,930<br>(5,980 to 7,800)	61.5<br>(53.0 to 69.2)	107.6<br>(83.3 to 132.4)	-14.0<br>(-22.9 to -4.8)
	Under 5	237<br>(221 to 254)	26.5<br>(24.7 to 28.4)	109<br>(98 to 122)	18.8<br>(16.9 to 21.0)	76<br>(63 to 91)	13.4<br>(11.3 to 16.3)	47<br>(38 to 57)	8.7<br>(7.1 to 10.5)	-67.3<br>(-73.6 to -60.4)	-53.9<br>(-61.7 to -45.0)
	5-14 years	22<br>(20 to 24)	1.3<br>(1.2 to 1.5)	14<br>(13 to 16)	1.2<br>(1.0 to 1.3)	11<br>(9 to 12)	0.9<br>(0.8 to 1.0)	9<br>(7 to 10)	0.7<br>(0.6 to 0.8)	-48.5<br>(-56.8 to -39.5)	-40.9<br>(-49.1 to -32.0)
	15-49 years	185<br>(175 to 195)	3.0<br>(2.9 to 3.2)	211<br>(197 to 226)	4.0<br>(3.7 to 4.3)	186<br>(168 to 205)	3.6<br>(3.2 to 4.0)	164<br>(141 to 187)	3.2<br>(2.8 to 3.7)	7.2<br>(-8.2 to 23.1)	-19.2<br>(-29.6 to -8.7)
	50-69 years	386<br>(359 to 417)	24.7<br>(23.0 to 26.6)	1,160<br>(1,060 to 1,250)	38.0<br>(34.9 to 41.1)	1,080<br>(972 to 1,210)	34.9<br>(31.3 to 38.9)	996<br>(854 to 1,150)	31.4<br>(26.9 to 36.3)	27.2<br>(6.0 to 48.8)	-17.3<br>(-28.0 to -5.9)
	70+ years	2,380<br>(2,200 to 2,530)	380.9<br>(351.4 to 404.2)	6,640<br>(5,980 to 7,100)	548.1<br>(494.2 to 586.7)	6,360<br>(5,620 to 7,070)	512.8<br>(453.2 to 570.1)	5,720<br>(4,900 to 6,440)	460.5<br>(394.3 to 518.2)	20.9<br>(6.2 to 36.3)	-16.0<br>(-24.6 to -7.2)
**Dominica**	All Ages	24<br>(21 to 26)	32.5<br>(29.0 to 36.6)	25<br>(21 to 29)	36.4<br>(30.9 to 42.4)	25<br>(21 to 29)	36.8<br>(31.2 to 43.4)	20<br>(17 to 23)	29.3<br>(24.6 to 34.8)	-9.7<br>(-23.6 to 8.2)	-19.3<br>(-23.3 to -14.6)
	Under 5	3<br>(2 to 3)	29.8<br>(23.2 to 37.1)	2<br>(1 to 2)	41.6<br>(29.8 to 56.4)	2<br>(1 to 2)	42.2<br>(29.5 to 58.6)	1<br>(1 to 2)	32.5<br>(22.1 to 46.5)	9.3<br>(-27.9 to 63.4)	-21.7<br>(-33.0 to -8.7)
	5-14 years	0<br>(0 to 0)	1.4<br>(1.1 to 1.7)	0<br>(0 to 0)	1.8<br>(1.4 to 2.3)	0<br>(0 to 0)	1.8<br>(1.4 to 2.3)	0<br>(0 to 0)	1.6<br>(1.2 to 2.0)	17.8<br>(-14.8 to 61.3)	-13.0<br>(-20.7 to -4.2)
	15-49 years	1<br>(1 to 1)	3.3<br>(2.9 to 3.8)	1<br>(1 to 2)	3.6<br>(2.8 to 4.4)	1<br>(1 to 2)	3.6<br>(2.8 to 4.5)	1<br>(1 to 1)	3.1<br>(2.4 to 3.9)	-6.5<br>(-28.7 to 23.8)	-14.9<br>(-21.0 to -8.2)
	50-69 years	3<br>(3 to 4)	38.4<br>(32.6 to 45.2)	4<br>(3 to 5)	29.0<br>(23.2 to 36.1)	4<br>(3 to 5)	29.2<br>(23.1 to 36.6)	4<br>(3 to 5)	25.6<br>(19.9 to 32.5)	-33.3<br>(-50.8 to -10.9)	-11.8<br>(-18.5 to -3.9)
	70+ years	16<br>(14 to 19)	424.4<br>(374.1 to 484.1)	17<br>(14 to 20)	391.9<br>(327.0 to 462.1)	18<br>(15 to 21)	391.7<br>(326.7 to 459.8)	13<br>(11 to 16)	299.0<br>(248.5 to 352.9)	-29.6<br>(-41.0 to -14.7)	-23.7<br>(-28.2 to -18.9)
**Dominican Republic**	All Ages	1,900<br>(1,660 to 2,190)	26.6<br>(23.2 to 30.6)	1,890<br>(1,560 to 2,210)	17.5<br>(14.5 to 20.5)	1,820<br>(1,490 to 2,150)	16.7<br>(13.7 to 19.7)	1,610<br>(1,300 to 2,060)	14.6<br>(11.8 to 18.7)	-45.0<br>(-57.4 to -29.9)	-16.3<br>(-31.0 to 3.7)
	Under 5	1,240<br>(1,010 to 1,480)	124.2<br>(101.5 to 148.1)	236<br>(149 to 328)	23.0<br>(14.6 to 32.1)	192<br>(121 to 269)	18.7<br>(11.7 to 26.1)	158<br>(98 to 222)	15.3<br>(9.5 to 21.4)	-87.7<br>(-92.5 to -82.1)	-33.7<br>(-45.8 to -20.5)
	5-14 years	55<br>(43 to 68)	3.2<br>(2.6 to 4.0)	25<br>(18 to 33)	1.3<br>(1.0 to 1.7)	36<br>(26 to 47)	1.9<br>(1.4 to 2.5)	28<br>(20 to 38)	1.5<br>(1.1 to 2.0)	-54.3<br>(-68.2 to -35.9)	11.6<br>(-6.8 to 32.8)
	15-49 years	114<br>(97 to 134)	3.1<br>(2.7 to 3.7)	227<br>(182 to 281)	3.9<br>(3.2 to 4.9)	168<br>(136 to 205)	2.9<br>(2.3 to 3.5)	155<br>(124 to 194)	2.7<br>(2.1 to 3.3)	-15.7<br>(-34.6 to 10.6)	-32.8<br>(-46.0 to -16.0)

	50-69 years	112<br>(93 to 138)	17.7<br>(14.7 to 21.7)	291<br>(222 to 372)	18.4<br>(14.1 to 23.6)	301<br>(232 to 383)	18.6<br>(14.3 to 23.6)	265<br>(199 to 356)	16.0<br>(12.0 to 21.5)	-9.4<br>(-35.7 to 28.7)	-13.3<br>(-31.8 to 15.2)
	70+ years	387<br>(327 to 474)	209.7<br>(177.4 to 257.0)	1,110<br>(868 to 1,340)	209.3<br>(163.9 to 252.0)	1,120<br>(881 to 1,380)	206.5<br>(162.6 to 255.6)	1,010<br>(759 to 1,330)	181.4<br>(136.8 to 239.8)	-13.5<br>(-34.7 to 16.5)	-13.3<br>(-30.3 to 11.1)
**Grenada**	All Ages	51<br>(46 to 55)	58.2<br>(52.7 to 63.7)	45<br>(41 to 48)	43.0<br>(39.3 to 46.5)	45<br>(41 to 49)	43.4<br>(39.4 to 47.5)	36<br>(31 to 40)	34.8<br>(30.2 to 38.9)	-40.2<br>(-48.9 to -32.3)	-19.1<br>(-28.3 to -14.8)
	Under 5	7<br>(6 to 9)	59.2<br>(48.2 to 72.1)	2<br>(2 to 2)	27.3<br>(21.3 to 34.4)	2<br>(1 to 2)	26.3<br>(20.4 to 33.4)	1<br>(1 to 2)	17.3<br>(13.3 to 22.1)	-70.8<br>(-77.9 to -62.3)	-36.6<br>(-43.0 to -29.3)
	5-14 years	1<br>(1 to 1)	2.8<br>(2.4 to 3.3)	0<br>(0 to 0)	1.9<br>(1.7 to 2.2)	0<br>(0 to 0)	1.9<br>(1.6 to 2.2)	0<br>(0 to 0)	1.5<br>(1.3 to 1.7)	-47.6<br>(-58.0 to -34.3)	-23.2<br>(-28.6 to -18.0)
	15-49 years	3<br>(3 to 3)	7.9<br>(7.0 to 8.8)	3<br>(3 to 3)	5.6<br>(4.9 to 6.3)	3<br>(3 to 3)	5.5<br>(4.8 to 6.4)	2<br>(2 to 3)	4.6<br>(3.9 to 5.5)	-41.1<br>(-52.6 to -28.5)	-16.6<br>(-26.2 to -9.0)
	50-69 years	6<br>(5 to 7)	63.7<br>(56.2 to 72.5)	8<br>(7 to 10)	40.8<br>(35.9 to 45.9)	9<br>(7 to 10)	40.6<br>(35.3 to 46.1)	8<br>(6 to 9)	35.1<br>(29.2 to 41.0)	-44.9<br>(-55.6 to -32.8)	-14.0<br>(-25.4 to -7.4)
	70+ years	34<br>(30 to 37)	679.6<br>(606.0 to 744.6)	31<br>(28 to 33)	533.0<br>(478.7 to 577.6)	31<br>(28 to 34)	531.3<br>(475.8 to 582.2)	24<br>(21 to 27)	410.7<br>(352.2 to 457.7)	-39.6<br>(-48.6 to -31.3)	-22.9<br>(-31.9 to -18.9)
**Guyana**	All Ages	251<br>(227 to 275)	32.1<br>(29.1 to 35.2)	261<br>(214 to 311)	34.1<br>(28.0 to 40.6)	233<br>(189 to 283)	30.3<br>(24.6 to 36.8)	197<br>(154 to 244)	25.7<br>(20.2 to 31.9)	-20.0<br>(-38.7 to 0.5)	-24.5<br>(-31.8 to -17.1)
	Under 5	97<br>(81 to 115)	86.1<br>(71.4 to 102.4)	39<br>(30 to 51)	51.4<br>(39.1 to 66.5)	31<br>(23 to 41)	40.9<br>(30.3 to 53.5)	24<br>(17 to 32)	31.7<br>(23.0 to 42.3)	-63.2<br>(-75.3 to -46.6)	-38.4<br>(-46.8 to -29.9)
	5-14 years	6<br>(5 to 7)	3.4<br>(2.8 to 3.9)	4<br>(3 to 5)	2.8<br>(2.3 to 3.3)	3<br>(3 to 4)	2.5<br>(2.0 to 3.0)	3<br>(2 to 3)	2.1<br>(1.7 to 2.5)	-38.5<br>(-52.3 to -20.1)	-26.1<br>(-33.4 to -19.3)
	15-49 years	26<br>(22 to 29)	6.4<br>(5.5 to 7.3)	34<br>(26 to 43)	8.4<br>(6.4 to 10.7)	31<br>(23 to 39)	7.6<br>(5.8 to 9.8)	27<br>(20 to 35)	6.7<br>(5.0 to 8.8)	3.7<br>(-25.6 to 39.1)	-20.5<br>(-30.3 to -9.9)
	50-69 years	35<br>(31 to 40)	54.2<br>(47.9 to 61.6)	59<br>(46 to 74)	49.6<br>(38.7 to 62.1)	55<br>(42 to 71)	45.3<br>(34.9 to 58.1)	50<br>(36 to 65)	40.4<br>(29.7 to 53.0)	-25.4<br>(-46.2 to 1.2)	-18.4<br>(-28.9 to -7.5)
	70+ years	86<br>(78 to 94)	490.5<br>(442.7 to 534.7)	125<br>(103 to 146)	452.8<br>(375.5 to 528.1)	113<br>(94 to 133)	395.5<br>(330.1 to 465.7)	94<br>(76 to 113)	324.4<br>(261.5 to 389.9)	-33.9<br>(-47.1 to -18.9)	-28.4<br>(-35.1 to -21.5)
**Haiti**	All Ages	8,430<br>(6,960 to 10,000)	132.1<br>(109.1 to 156.7)	6,280<br>(4,820 to 7,970)	50.7<br>(38.9 to 64.3)	5,760<br>(4,370 to 7,290)	45.6<br>(34.6 to 57.7)	5,200<br>(3,930 to 6,670)	40.4<br>(30.5 to 51.9)	-69.4<br>(-77.0 to -59.7)	-20.3<br>(-26.9 to -13.0)
	Under 5	6,500<br>(5,140 to 7,990)	612.5<br>(485.1 to 753.3)	3,250<br>(2,300 to 4,360)	211.0<br>(148.9 to 282.5)	2,880<br>(2,050 to 3,880)	185.1<br>(131.5 to 249.6)	2,530<br>(1,720 to 3,390)	161.2<br>(109.8 to 216.1)	-73.7<br>(-82.3 to -62.8)	-23.6<br>(-34.4 to -11.4)
	5-14 years	179<br>(116 to 252)	10.9<br>(7.0 to 15.2)	169<br>(110 to 232)	6.3<br>(4.1 to 8.6)	158<br>(102 to 216)	5.8<br>(3.7 to 7.9)	148<br>(96 to 201)	5.3<br>(3.4 to 7.2)	-51.0<br>(-65.8 to -28.9)	-15.2<br>(-27.1 to 1.6)
	15-49 years	365<br>(234 to 513)	12.4<br>(8.0 to 17.5)	572<br>(367 to 822)	8.7<br>(5.6 to 12.5)	545<br>(344 to 781)	8.1<br>(5.1 to 11.6)	519<br>(327 to 738)	7.6<br>(4.8 to 10.8)	-39.2<br>(-59.2 to -14.1)	-13.0<br>(-20.2 to -5.4)

	50-69 years	545 (352 to 796)	90.7 (58.6 to 132.4)	802 (525 to 1,150)	61.8 (40.5 to 88.9)	789 (506 to 1,140)	58.8 (37.8 to 84.7)	752 (491 to 1,070)	54.9 (35.8 to 78.2)	-39.5 (-60.5 to -11.5)	-11.3 (-18.7 to -3.2)
	70+ years	845 (578 to 1,160)	639.2 (436.8 to 880.7)	1,490 (1,010 to 2,080)	541.1 (368.4 to 757.7)	1,390 (947 to 1,940)	501.2 (341.3 to 700.1)	1,250 (847 to 1,740)	440.2 (297.9 to 613.4)	-31.1 (-49.7 to -6.3)	-18.7 (-25.4 to -11.9)
**Jamaica**	All Ages	408 (379 to 434)	17.3 (16.0 to 18.3)	476 (388 to 570)	17.0 (13.9 to 20.4)	425 (332 to 521)	15.2 (11.8 to 18.6)	395 (310 to 488)	14.1 (11.1 to 17.4)	-18.2 (-33.8 to 0.5)	-17.0 (-26.6 to -6.3)
	Under 5	78 (68 to 89)	27.8 (24.4 to 32.0)	21 (15 to 27)	11.5 (8.6 to 15.0)	17 (12 to 23)	9.7 (7.1 to 13.0)	13 (9 to 18)	7.6 (5.2 to 10.4)	-72.8 (-81.0 to -62.9)	-34.4 (-42.2 to -25.7)
	5-14 years	9 (8 to 10)	1.6 (1.4 to 1.8)	3 (2 to 3)	0.6 (0.5 to 0.7)	2 (1 to 3)	0.5 (0.3 to 0.6)	2 (2 to 2)	0.5 (0.4 to 0.6)	-70.1 (-76.2 to -62.1)	-20.2 (-27.1 to -13.1)
	15-49 years	16 (14 to 17)	1.3 (1.2 to 1.4)	26 (20 to 33)	1.7 (1.3 to 2.1)	24 (18 to 31)	1.5 (1.1 to 2.0)	22 (17 to 30)	1.5 (1.1 to 1.9)	10.0 (-19.0 to 48.1)	-14.1 (-28.1 to 2.1)
	50-69 years	39 (36 to 43)	15.7 (14.4 to 17.2)	67 (52 to 86)	13.9 (10.7 to 17.7)	63 (47 to 81)	12.7 (9.5 to 16.3)	61 (44 to 80)	12.1 (8.8 to 16.0)	-22.8 (-44.2 to 2.9)	-13.3 (-28.7 to 4.4)
	70+ years	267 (241 to 288)	229.9 (207.2 to 247.3)	359 (292 to 427)	202.7 (164.8 to 240.8)	320 (247 to 386)	178.7 (138.0 to 216.1)	297 (235 to 362)	163.4 (129.4 to 199.1)	-28.9 (-42.1 to -13.7)	-19.4 (-28.2 to -10.1)
**Puerto Rico**	All Ages	1,200 (1,120 to 1,260)	33.3 (31.0 to 35.0)	1,110 (957 to 1,200)	32.7 (28.3 to 35.5)	1,000 (856 to 1,130)	30.0 (25.7 to 34.0)	912 (744 to 1,070)	27.7 (22.6 to 32.4)	-16.8 (-30.5 to -3.0)	-15.4 (-27.4 to -3.6)
	Under 5	56 (51 to 61)	17.6 (16.1 to 19.2)	8 (7 to 9)	6.3 (5.6 to 7.3)	6 (5 to 7)	5.4 (4.6 to 6.5)	4 (3 to 5)	4.0 (3.3 to 4.9)	-77.1 (-81.7 to -71.6)	-36.4 (-47.3 to -23.2)
	5-14 years	5 (4 to 5)	0.7 (0.6 to 0.8)	1 (1 to 1)	0.2 (0.2 to 0.3)	0 (0 to 1)	0.1 (0.1 to 0.2)	0 (0 to 0)	0.1 (0.1 to 0.1)	-84.7 (-87.2 to -81.8)	-52.2 (-57.5 to -46.3)
	15-49 years	97 (89 to 105)	5.3 (4.9 to 5.7)	35 (32 to 38)	2.3 (2.1 to 2.5)	31 (28 to 35)	2.1 (1.9 to 2.4)	26 (21 to 31)	1.8 (1.5 to 2.1)	-66.4 (-72.6 to -59.8)	-23.7 (-36.2 to -10.9)
	50-69 years	169 (155 to 185)	30.9 (28.3 to 33.7)	151 (137 to 166)	17.4 (15.8 to 19.1)	138 (122 to 157)	15.9 (14.1 to 18.1)	122 (99 to 147)	14.2 (11.5 to 17.1)	-54.1 (-63.6 to -43.6)	-18.5 (-32.9 to -3.7)
	70+ years	875 (800 to 930)	391.4 (358.0 to 416.2)	912 (767 to 1,000)	182.3 (153.4 to 199.9)	827 (690 to 948)	161.3 (134.5 to 184.9)	760 (612 to 885)	144.3 (116.1 to 168.1)	-63.1 (-69.3 to -57.2)	-20.8 (-31.8 to -10.3)
**Saint Kitts and Nevis**	All Ages	19 (18 to 20)	46.2 (43.1 to 49.3)	19 (17 to 22)	33.1 (29.2 to 37.2)	19 (17 to 22)	33.2 (28.8 to 37.6)	17 (14 to 19)	28.3 (24.3 to 32.4)	-38.8 (-47.7 to -28.9)	-14.5 (-19.8 to -10.4)
	Under 5	2 (2 to 3)	48.8 (43.3 to 55.2)	1 (1 to 1)	30.1 (23.7 to 37.3)	1 (1 to 1)	27.8 (21.6 to 35.2)	1 (0 to 1)	21.2 (16.0 to 27.5)	-56.5 (-67.9 to -42.1)	-29.5 (-36.4 to -22.0)
	5-14 years	0 (0 to 0)	2.1 (1.9 to 2.4)	0 (0 to 0)	1.7 (1.5 to 2.1)	0 (0 to 0)	1.7 (1.5 to 2.0)	0 (0 to 0)	1.4 (1.2 to 1.7)	-33.8 (-45.4 to -13.6)	-18.8 (-24.1 to -12.0)
	15-49 years	1 (1 to 1)	5.7 (5.2 to 6.3)	1 (1 to 1)	3.4 (2.7 to 4.3)	1 (1 to 1)	3.4 (2.6 to 4.4)	1 (1 to 1)	2.9 (2.3 to 3.8)	-49.1 (-61.1 to -33.2)	-14.0 (-20.9 to -5.9)

	50-69 years	3<br>(2 to 3)	55.2<br>(50.0 to 60.9)	5<br>(4 to 6)	35.1<br>(28.9 to 42.0)	5<br>(4 to 6)	35.6<br>(28.9 to 43.1)	5<br>(4 to 5)	31.4<br>(25.1 to 38.1)	-43.0<br>(-55.2 to -29.4)	-10.3<br>(-17.2 to -4.0)
	70+ years	13<br>(12 to 14)	477.8<br>(441.8 to 512.9)	12<br>(11 to 14)	473.3<br>(422.5 to 524.4)	12<br>(11 to 14)	463.3<br>(410.8 to 517.6)	10<br>(9 to 12)	373.9<br>(327.1 to 418.5)	-21.7<br>(-31.5 to -11.0)	-21.0<br>(-25.9 to -17.5)
**Saint Lucia**	All Ages	34<br>(32 to 36)	24.7<br>(23.2 to 26.4)	55<br>(48 to 62)	30.9<br>(26.9 to 35.2)	54<br>(46 to 62)	30.2<br>(26.0 to 34.8)	52<br>(43 to 60)	29.0<br>(24.0 to 34.0)	17.4<br>(-2.4 to 37.7)	-6.1<br>(-16.5 to -0.3)
	Under 5	6<br>(5 to 7)	32.6<br>(26.8 to 39.1)	2<br>(1 to 2)	18.8<br>(13.4 to 25.5)	1<br>(1 to 2)	15.5<br>(11.1 to 21.6)	1<br>(1 to 2)	12.8<br>(8.8 to 18.1)	-60.6<br>(-74.1 to -41.5)	-31.5<br>(-39.4 to -23.6)
	5-14 years	0<br>(0 to 0)	1.2<br>(1.0 to 1.3)	0<br>(0 to 0)	1.0<br>(0.9 to 1.2)	0<br>(0 to 0)	1.0<br>(0.8 to 1.2)	0<br>(0 to 0)	1.0<br>(0.8 to 1.2)	-15.3<br>(-31.4 to 4.7)	-3.6<br>(-11.3 to 3.7)
	15-49 years	3<br>(3 to 3)	4.1<br>(3.8 to 4.4)	3<br>(3 to 4)	3.7<br>(3.1 to 4.3)	3<br>(3 to 4)	3.4<br>(2.9 to 4.1)	3<br>(2 to 3)	3.1<br>(2.6 to 3.8)	-22.9<br>(-37.9 to -5.6)	-14.7<br>(-23.8 to -8.1)
	50-69 years	4<br>(4 to 5)	34.0<br>(31.0 to 37.2)	8<br>(7 to 9)	20.0<br>(16.9 to 23.7)	8<br>(6 to 9)	19.1<br>(15.8 to 22.9)	7<br>(6 to 9)	17.9<br>(14.1 to 22.0)	-47.3<br>(-58.8 to -34.2)	-10.6<br>(-23.7 to -2.6)
	70+ years	20<br>(19 to 22)	390.8<br>(363.2 to 419.1)	41<br>(36 to 47)	316.8<br>(274.3 to 358.1)	41<br>(35 to 47)	307.5<br>(265.5 to 350.6)	40<br>(33 to 46)	293.1<br>(243.3 to 339.8)	-25.0<br>(-37.2 to -12.6)	-7.5<br>(-17.3 to -1.7)
**Saint Vincent and the Grenadines**	All Ages	27<br>(25 to 29)	24.4<br>(22.5 to 26.3)	45<br>(41 to 49)	39.1<br>(35.7 to 42.6)	44<br>(40 to 49)	39.0<br>(35.1 to 43.1)	37<br>(33 to 42)	32.6<br>(28.9 to 36.5)	33.4<br>(18.0 to 52.1)	-16.6<br>(-20.9 to -12.4)
	Under 5	5<br>(4 to 6)	40.8<br>(32.5 to 50.1)	2<br>(1 to 2)	19.3<br>(15.0 to 24.4)	1<br>(1 to 2)	17.8<br>(13.5 to 22.9)	1<br>(1 to 1)	12.8<br>(9.3 to 17.2)	-68.7<br>(-78.3 to -53.8)	-33.9<br>(-42.2 to -24.5)
	5-14 years	1<br>(0 to 1)	1.8<br>(1.5 to 2.1)	0<br>(0 to 0)	1.9<br>(1.6 to 2.3)	0<br>(0 to 0)	1.9<br>(1.6 to 2.3)	0<br>(0 to 0)	1.6<br>(1.3 to 1.9)	-11.1<br>(-29.0 to 12.0)	-18.1<br>(-23.8 to -11.7)
	15-49 years	2<br>(2 to 2)	3.8<br>(3.4 to 4.1)	3<br>(3 to 3)	5.0<br>(4.4 to 5.6)	3<br>(2 to 3)	4.9<br>(4.3 to 5.6)	2<br>(2 to 3)	4.3<br>(3.7 to 5.0)	13.0<br>(-5.3 to 34.1)	-14.3<br>(-20.2 to -8.3)
	50-69 years	3<br>(3 to 3)	29.3<br>(26.3 to 32.5)	7<br>(6 to 8)	30.0<br>(26.8 to 33.6)	7<br>(6 to 8)	30.0<br>(26.2 to 34.5)	7<br>(6 to 8)	26.6<br>(22.7 to 30.9)	-9.3<br>(-24.4 to 8.1)	-11.5<br>(-17.7 to -5.4)
	70+ years	16<br>(14 to 17)	363.8<br>(330.4 to 394.9)	33<br>(30 to 36)	426.4<br>(386.8 to 466.6)	33<br>(29 to 36)	420.7<br>(377.9 to 464.2)	27<br>(24 to 30)	339.1<br>(303.3 to 379.2)	-6.8<br>(-17.6 to 5.1)	-20.5<br>(-24.2 to -16.8)
**Suriname**	All Ages	111<br>(98 to 123)	28.7<br>(25.3 to 31.7)	166<br>(144 to 188)	28.9<br>(25.1 to 32.8)	152<br>(129 to 176)	26.3<br>(22.4 to 30.5)	124<br>(90 to 157)	21.4<br>(15.6 to 27.2)	-25.4<br>(-46.0 to -2.9)	-26.0<br>(-41.1 to -10.9)
	Under 5	37<br>(29 to 46)	84.8<br>(65.6 to 104.5)	18<br>(13 to 24)	39.0<br>(27.4 to 52.8)	14<br>(10 to 20)	31.6<br>(22.3 to 43.6)	11<br>(8 to 16)	25.5<br>(17.6 to 35.8)	-69.9<br>(-80.3 to -53.8)	-34.6<br>(-45.6 to -22.9)
	5-14 years	2<br>(1 to 3)	2.5<br>(1.5 to 3.2)	2<br>(1 to 2)	1.6<br>(1.3 to 2.1)	1<br>(1 to 2)	1.4<br>(1.1 to 1.9)	1<br>(1 to 2)	1.3<br>(1.0 to 1.7)	-47.3<br>(-63.0 to -14.0)	-19.3<br>(-28.7 to -7.9)
	15-49 years	9<br>(7 to 11)	4.7<br>(3.8 to 5.3)	14<br>(12 to 16)	4.8<br>(4.1 to 5.5)	13<br>(11 to 15)	4.4<br>(3.7 to 5.2)	11<br>(8 to 13)	3.7<br>(2.9 to 4.7)	-20.8<br>(-40.2 to 7.0)	-22.3<br>(-35.2 to -9.3)

	50-69 years	16 (13 to 18)	33.6 (28.7 to 39.1)	33 (28 to 39)	30.2 (25.2 to 35.6)	32 (26 to 38)	28.0 (22.9 to 34.1)	27 (20 to 36)	23.6 (17.1 to 30.8)	-29.7 (-50.2 to -5.6)	-21.8 (-38.7 to -3.9)
	70+ years	47 (40 to 54)	382.3 (329.5 to 438.4)	100 (83 to 116)	315.1 (263.3 to 366.0)	92 (75 to 109)	282.9 (231.5 to 335.5)	73 (50 to 97)	220.6 (150.6 to 291.4)	-42.3 (-59.7 to -23.2)	-30.0 (-47.0 to -13.0)
**Trinidad and Tobago**	All Ages	301 (286 to 316)	25.0 (23.8 to 26.2)	298 (258 to 338)	21.4 (18.5 to 24.3)	285 (238 to 339)	20.4 (17.1 to 24.3)	255 (198 to 316)	18.3 (14.2 to 22.7)	-26.9 (-42.9 to -10.0)	-14.4 (-28.9 to 1.0)
	Under 5	45 (37 to 53)	33.6 (28.1 to 39.7)	14 (11 to 18)	16.4 (12.4 to 20.9)	11 (8 to 15)	13.3 (10.0 to 17.6)	9 (6 to 12)	10.7 (7.7 to 14.9)	-68.1 (-78.6 to -53.2)	-34.7 (-43.5 to -24.6)
	5-14 years	6 (5 to 6)	2.0 (1.8 to 2.3)	2 (2 to 3)	1.1 (0.9 to 1.3)	2 (1 to 2)	0.9 (0.7 to 1.1)	1 (1 to 2)	0.7 (0.6 to 0.9)	-63.9 (-71.8 to -54.5)	-32.9 (-39.8 to -25.7)
	15-49 years	25 (23 to 27)	4.0 (3.7 to 4.3)	28 (23 to 33)	4.0 (3.3 to 4.8)	25 (20 to 32)	3.6 (2.9 to 4.6)	23 (17 to 30)	3.3 (2.4 to 4.3)	-18.0 (-39.7 to 9.4)	-17.4 (-34.1 to 1.5)
	50-69 years	42 (39 to 46)	31.7 (29.3 to 34.4)	60 (50 to 71)	19.0 (15.7 to 22.5)	57 (45 to 72)	18.1 (14.1 to 22.6)	53 (39 to 70)	16.6 (12.2 to 21.8)	-47.6 (-62.0 to -30.9)	-12.4 (-30.6 to 8.7)
	70+ years	184 (171 to 195)	389.9 (363.3 to 412.3)	194 (166 to 221)	185.9 (159.5 to 211.5)	189 (158 to 224)	173.7 (145.5 to 205.2)	169 (132 to 206)	151.2 (117.7 to 184.0)	-61.2 (-69.6 to -52.7)	-18.7 (-31.5 to -5.3)
**United States Virgin Islands**	All Ages	15 (13 to 17)	14.1 (12.1 to 16.2)	22 (17 to 27)	23.9 (19.1 to 29.5)	17 (13 to 21)	19.0 (15.0 to 23.8)	14 (11 to 18)	16.8 (13.3 to 21.0)	18.7 (-8.4 to 53.1)	-29.7 (-35.0 to -24.6)
	Under 5	2 (1 to 3)	18.0 (13.0 to 23.4)	0 (0 to 0)	4.8 (3.1 to 6.9)	0 (0 to 0)	4.0 (2.6 to 5.7)	0 (0 to 0)	3.0 (2.0 to 4.2)	-83.1 (-90.0 to -72.6)	-36.5 (-55.3 to -12.5)
	5-14 years	0 (0 to 0)	1.0 (0.7 to 1.3)	0 (0 to 0)	0.3 (0.2 to 0.4)	0 (0 to 0)	0.1 (0.1 to 0.2)	0 (0 to 0)	0.1 (0.0 to 0.1)	-92.5 (-96.0 to -87.2)	-72.8 (-80.8 to -63.5)
	15-49 years	2 (1 to 2)	3.2 (2.7 to 3.8)	1 (1 to 1)	3.2 (2.4 to 4.0)	1 (1 to 1)	3.0 (2.2 to 3.8)	1 (0 to 1)	2.0 (1.5 to 2.5)	-38.8 (-55.8 to -18.1)	-38.4 (-45.4 to -30.6)
	50-69 years	3 (2 to 3)	18.7 (14.8 to 22.9)	3 (2 to 4)	11.8 (8.8 to 15.8)	2 (2 to 3)	8.2 (6.1 to 11.0)	2 (1 to 3)	7.3 (5.3 to 9.8)	-61.1 (-72.4 to -45.3)	-38.7 (-44.5 to -32.5)
	70+ years	8 (7 to 10)	204.6 (169.1 to 242.0)	17 (14 to 21)	135.9 (108.1 to 168.2)	13 (10 to 17)	105.2 (82.4 to 131.5)	12 (9 to 15)	91.1 (71.4 to 114.6)	-55.5 (-66.3 to -41.2)	-33.0 (-38.5 to -27.7)
**Colombia**	All Ages	7,690 (7,060 to 8,320)	23.7 (21.7 to 25.6)	8,790 (7,910 to 9,440)	18.3 (16.5 to 19.6)	7,740 (6,850 to 8,500)	15.9 (14.0 to 17.4)	7,320 (6,150 to 8,560)	14.9 (12.5 to 17.4)	-36.9 (-47.6 to -25.0)	-18.4 (-29.0 to -7.7)
	Under 5	4,060 (3,520 to 4,650)	96.6 (83.5 to 110.5)	847 (619 to 1,120)	24.3 (17.7 to 32.2)	539 (386 to 732)	15.4 (11.1 to 21.0)	421 (283 to 609)	12.2 (8.2 to 17.7)	-87.3 (-91.6 to -81.3)	-49.7 (-61.1 to -37.4)
	5-14 years	298 (265 to 332)	4.0 (3.6 to 4.4)	159 (135 to 182)	2.2 (1.9 to 2.5)	115 (100 to 133)	1.6 (1.4 to 1.8)	102 (87 to 120)	1.4 (1.2 to 1.7)	-64.5 (-70.7 to -57.0)	-34.7 (-42.9 to -26.2)
	15-49 years	634 (602 to 668)	3.7 (3.6 to 4.0)	886 (840 to 933)	3.5 (3.3 to 3.7)	743 (690 to 800)	2.9 (2.7 to 3.1)	697 (595 to 812)	2.7 (2.3 to 3.1)	-28.8 (-40.1 to -15.8)	-23.1 (-34.3 to -11.6)

	50-69 years	735<br>(686 to 785)	23.9<br>(22.3 to 25.5)	1,590<br>(1,470 to 1,710)	18.1<br>(16.7 to 19.5)	1,570<br>(1,410 to 1,760)	17.5<br>(15.7 to 19.6)	1,540<br>(1,250 to 1,850)	16.9<br>(13.7 to 20.2)	-29.3<br>(-42.8 to -13.7)	-6.5<br>(-22.1 to 11.6)
	70+ years	1,960<br>(1,770 to 2,090)	234.0<br>(211.6 to 250.0)	5,310<br>(4,560 to 5,800)	178.0<br>(152.9 to 194.4)	4,770<br>(4,030 to 5,330)	153.7<br>(130.1 to 172.0)	4,560<br>(3,730 to 5,290)	143.0<br>(117.0 to 166.0)	-38.9<br>(-47.8 to -28.7)	-19.7<br>(-29.4 to -9.0)
**Costa Rica**	All Ages	448<br>(418 to 475)	14.7<br>(13.7 to 15.6)	856<br>(769 to 915)	18.2<br>(16.3 to 19.4)	666<br>(590 to 720)	14.1<br>(12.5 to 15.2)	671<br>(587 to 743)	14.1<br>(12.4 to 15.6)	-4.1<br>(-14.7 to 6.8)	-22.3<br>(-28.7 to -16.8)
	Under 5	150<br>(135 to 166)	37.0<br>(33.3 to 40.9)	51<br>(46 to 58)	15.4<br>(13.7 to 17.2)	30<br>(26 to 34)	9.2<br>(8.0 to 10.5)	26<br>(21 to 31)	8.3<br>(6.7 to 10.0)	-77.6<br>(-82.4 to -71.9)	-46.1<br>(-55.2 to -36.1)
	5-14 years	9<br>(8 to 10)	1.3<br>(1.2 to 1.4)	6<br>(5 to 6)	0.8<br>(0.7 to 0.9)	5<br>(4 to 5)	0.6<br>(0.6 to 0.7)	4<br>(4 to 5)	0.6<br>(0.5 to 0.6)	-54.5<br>(-60.4 to -47.4)	-29.4<br>(-34.8 to -24.0)
	15-49 years	33<br>(31 to 36)	2.2<br>(2.0 to 2.3)	68<br>(64 to 74)	2.7<br>(2.6 to 3.0)	55<br>(51 to 60)	2.2<br>(2.0 to 2.4)	61<br>(55 to 67)	2.5<br>(2.2 to 2.7)	13.5<br>(-0.2 to 27.4)	-10.6<br>(-18.6 to -4.1)
	50-69 years	48<br>(44 to 52)	16.9<br>(15.6 to 18.3)	153<br>(141 to 166)	17.4<br>(16.1 to 18.9)	132<br>(120 to 144)	14.7<br>(13.4 to 16.0)	139<br>(121 to 159)	15.3<br>(13.2 to 17.4)	-9.6<br>(-22.1 to 5.2)	-12.3<br>(-22.5 to -3.7)
	70+ years	208<br>(184 to 225)	219.2<br>(193.8 to 237.5)	577<br>(494 to 627)	199.7<br>(171.0 to 217.0)	445<br>(377 to 491)	147.3<br>(125.0 to 162.6)	440<br>(367 to 492)	140.3<br>(116.9 to 156.9)	-36.0<br>(-42.7 to -28.9)	-29.7<br>(-35.1 to -25.2)
**El Salvador**	All Ages	2,140<br>(1,920 to 2,400)	40.3<br>(36.1 to 45.2)	2,090<br>(1,830 to 2,340)	32.6<br>(28.6 to 36.6)	1,720<br>(1,450 to 2,020)	26.7<br>(22.6 to 31.5)	1,650<br>(1,330 to 2,020)	25.6<br>(20.6 to 31.3)	-36.3<br>(-49.9 to -20.2)	-21.5<br>(-33.3 to -8.9)
	Under 5	1,080<br>(889 to 1,280)	140.3<br>(115.4 to 166.9)	162<br>(116 to 216)	25.9<br>(18.6 to 34.6)	101<br>(69 to 143)	16.5<br>(11.2 to 23.3)	89<br>(58 to 125)	14.9<br>(9.7 to 20.8)	-89.4<br>(-93.2 to -84.5)	-42.5<br>(-51.4 to -32.9)
	5-14 years	69<br>(54 to 88)	5.0<br>(3.9 to 6.4)	29<br>(23 to 37)	2.4<br>(1.9 to 3.0)	21<br>(16 to 26)	1.7<br>(1.3 to 2.2)	21<br>(16 to 28)	1.8<br>(1.3 to 2.3)	-64.7<br>(-74.4 to -51.9)	-26.8<br>(-35.5 to -17.8)
	15-49 years	150<br>(133 to 176)	6.0<br>(5.3 to 7.0)	197<br>(171 to 227)	6.0<br>(5.2 to 6.9)	162<br>(133 to 193)	4.9<br>(4.0 to 5.8)	164<br>(128 to 207)	4.9<br>(3.9 to 6.3)	-17.7<br>(-37.3 to 8.0)	-17.2<br>(-32.9 to -0.2)
	50-69 years	183<br>(156 to 215)	37.6<br>(32.1 to 44.1)	346<br>(289 to 409)	38.5<br>(32.2 to 45.4)	305<br>(246 to 373)	33.1<br>(26.7 to 40.5)	292<br>(221 to 373)	31.2<br>(23.6 to 39.8)	-17.1<br>(-39.9 to 11.4)	-18.9<br>(-34.9 to -0.2)
	70+ years	653<br>(545 to 761)	412.2<br>(343.9 to 480.3)	1,360<br>(1,120 to 1,570)	363.0<br>(300.4 to 420.7)	1,130<br>(913 to 1,350)	298.6<br>(241.4 to 356.0)	1,090<br>(850 to 1,340)	283.7<br>(222.1 to 349.2)	-31.2<br>(-46.4 to -14.7)	-21.8<br>(-33.8 to -9.3)
**Guatemala**	All Ages	10,300<br>(9,760 to 11,000)	123.1<br>(116.4 to 130.7)	9,370<br>(8,730 to 10,000)	60.7<br>(56.5 to 64.9)	7,660<br>(6,890 to 8,420)	49.0<br>(44.1 to 53.9)	6,900<br>(5,950 to 7,970)	43.7<br>(37.8 to 50.6)	-64.5<br>(-69.5 to -58.8)	-27.9<br>(-35.3 to -20.1)
	Under 5	5,170<br>(4,670 to 5,770)	337.3<br>(304.2 to 376.3)	2,750<br>(2,260 to 3,250)	168.2<br>(138.5 to 199.2)	2,100<br>(1,720 to 2,550)	131.3<br>(107.4 to 159.7)	1,720<br>(1,330 to 2,200)	110.1<br>(85.6 to 141.3)	-67.3<br>(-74.9 to -57.6)	-34.5<br>(-44.4 to -23.3)
	5-14 years	724<br>(664 to 792)	28.7<br>(26.3 to 31.4)	226<br>(197 to 256)	6.6<br>(5.8 to 7.5)	178<br>(155 to 202)	5.3<br>(4.6 to 6.0)	152<br>(130 to 177)	4.5<br>(3.9 to 5.2)	-84.3<br>(-86.9 to -81.3)	-32.4<br>(-39.1 to -24.1)
	15-49 years	1,070<br>(1,020 to 1,120)	30.3<br>(28.9 to 31.7)	917<br>(868 to 969)	11.3<br>(10.7 to 11.9)	753<br>(688 to 827)	9.1<br>(8.3 to 10.0)	700<br>(605 to 803)	8.3<br>(7.2 to 9.5)	-72.6<br>(-76.5 to -68.2)	-26.2<br>(-35.4 to -16.4)

	50-69 years	1,210 (1,150 to 1,280)	189.9 (179.6 to 200.6)	1,230 (1,150 to 1,310)	70.9 (66.3 to 75.7)	1,200 (1,080 to 1,320)	67.1 (60.2 to 73.9)	1,140 (955 to 1,340)	62.1 (52.2 to 73.4)	-67.3 (-72.7 to -61.3)	-12.4 (-25.8 to 2.4)
	70+ years	2,140 (2,010 to 2,240)	1,456.0 (1,373.0 to 1,526.4)	4,250 (3,900 to 4,500)	765.5 (702.5 to 810.1)	3,430 (3,020 to 3,790)	598.6 (527.2 to 661.3)	3,190 (2,760 to 3,600)	541.2 (468.6 to 610.1)	-62.8 (-67.2 to -58.1)	-29.3 (-36.4 to -21.8)
**Honduras**	All Ages	1,950 (1,690 to 2,240)	41.4 (35.8 to 47.6)	1,940 (1,610 to 2,310)	19.9 (16.5 to 23.7)	1,730 (1,420 to 2,090)	17.4 (14.3 to 21.0)	1,640 (1,320 to 2,000)	16.2 (13.1 to 19.8)	-60.9 (-69.0 to -50.9)	-18.9 (-26.1 to -9.3)
	Under 5	1,420 (1,180 to 1,680)	172.0 (143.2 to 204.2)	398 (281 to 531)	36.6 (25.8 to 48.8)	313 (217 to 421)	28.7 (19.9 to 38.5)	277 (186 to 382)	25.3 (17.0 to 34.8)	-85.3 (-90.5 to -78.9)	-30.9 (-41.5 to -18.3)
	5-14 years	96 (74 to 121)	6.9 (5.3 to 8.7)	30 (18 to 43)	1.4 (0.8 to 2.0)	26 (15 to 38)	1.2 (0.7 to 1.7)	24 (14 to 35)	1.1 (0.6 to 1.6)	-84.5 (-91.4 to -75.6)	-22.4 (-34.2 to -9.1)
	15-49 years	122 (103 to 142)	6.0 (5.0 to 6.9)	174 (114 to 251)	3.4 (2.2 to 4.9)	164 (105 to 232)	3.1 (2.0 to 4.4)	163 (104 to 236)	3.0 (1.9 to 4.4)	-49.6 (-68.5 to -26.1)	-10.6 (-25.2 to 7.3)
	50-69 years	106 (86 to 127)	29.8 (24.1 to 35.7)	395 (301 to 525)	38.3 (29.2 to 50.8)	377 (285 to 508)	35.2 (26.6 to 47.4)	369 (277 to 491)	33.4 (25.1 to 44.5)	12.0 (-17.8 to 52.8)	-12.7 (-21.2 to -3.0)
	70+ years	209 (176 to 247)	214.5 (180.6 to 253.3)	945 (759 to 1,180)	311.6 (250.3 to 388.9)	849 (669 to 1,060)	276.3 (217.8 to 345.0)	803 (629 to 1,020)	254.7 (199.5 to 322.8)	18.7 (-8.9 to 53.9)	-18.3 (-26.2 to -8.5)
**Mexico**	All Ages	31,800 (29,800 to 34,200)	37.3 (35.0 to 40.0)	31,700 (30,100 to 33,000)	24.9 (23.6 to 25.8)	29,600 (26,900 to 32,600)	23.0 (20.9 to 25.3)	26,400 (23,100 to 30,000)	20.4 (17.9 to 23.2)	-45.2 (-52.4 to -36.8)	-17.9 (-27.3 to -7.8)
	Under 5	19,800 (18,000 to 22,100)	167.9 (152.5 to 187.1)	3,050 (2,410 to 3,760)	28.9 (22.9 to 35.7)	2,380 (1,770 to 3,090)	23.3 (17.3 to 30.2)	1,800 (1,300 to 2,420)	18.2 (13.2 to 24.5)	-89.1 (-92.3 to -85.0)	-37.0 (-47.3 to -25.2)
	5-14 years	960 (897 to 1,030)	4.4 (4.1 to 4.8)	303 (271 to 340)	1.4 (1.2 to 1.5)	236 (210 to 268)	1.1 (0.9 to 1.2)	204 (180 to 230)	0.9 (0.8 to 1.0)	-79.3 (-82.0 to -76.1)	-32.0 (-38.4 to -25.4)
	15-49 years	1,910 (1,860 to 1,970)	4.5 (4.4 to 4.6)	4,090 (3,960 to 4,220)	6.1 (5.9 to 6.3)	3,940 (3,600 to 4,370)	5.8 (5.3 to 6.4)	3,550 (3,070 to 4,050)	5.2 (4.5 to 5.9)	15.2 (-0.4 to 31.8)	-14.6 (-26.0 to -2.8)
	50-69 years	2,170 (2,110 to 2,250)	29.5 (28.6 to 30.5)	7,250 (6,990 to 7,500)	35.0 (33.7 to 36.2)	7,280 (6,530 to 8,120)	34.0 (30.5 to 37.9)	6,760 (5,700 to 7,900)	30.7 (25.9 to 35.9)	4.2 (-12.3 to 22.2)	-12.1 (-25.7 to 1.9)
	70+ years	6,960 (6,600 to 7,220)	335.1 (317.6 to 347.6)	17,000 (15,700 to 17,800)	264.2 (243.8 to 275.8)	15,700 (14,100 to 17,400)	238.1 (213.1 to 262.5)	14,100 (12,300 to 15,900)	208.4 (181.5 to 235.5)	-37.8 (-44.9 to -30.2)	-21.1 (-29.4 to -12.3)
**Nicaragua**	All Ages	1,680 (1,440 to 1,930)	43.1 (37.1 to 49.7)	888 (772 to 1,030)	13.6 (11.8 to 15.7)	616 (527 to 722)	9.3 (8.0 to 10.9)	599 (505 to 717)	9.0 (7.6 to 10.7)	-79.2 (-83.0 to -74.0)	-34.1 (-40.7 to -26.0)
	Under 5	1,360 (1,140 to 1,600)	204.3 (171.6 to 241.0)	299 (219 to 395)	45.1 (33.0 to 59.4)	183 (129 to 248)	27.8 (19.6 to 37.7)	168 (116 to 231)	25.8 (17.8 to 35.6)	-87.4 (-91.5 to -81.7)	-42.7 (-50.0 to -35.1)
	5-14 years	51 (41 to 62)	4.4 (3.5 to 5.4)	23 (18 to 28)	1.7 (1.4 to 2.1)	12 (9 to 15)	0.9 (0.7 to 1.1)	10 (8 to 12)	0.7 (0.6 to 0.9)	-83.2 (-87.6 to -77.6)	-57.1 (-63.5 to -50.5)
	15-49 years	61 (53 to 68)	3.5 (3.1 to 3.9)	100 (89 to 113)	2.9 (2.5 to 3.2)	71 (62 to 81)	2.0 (1.7 to 2.3)	73 (59 to 89)	2.0 (1.7 to 2.5)	-42.7 (-53.4 to -29.6)	-29.2 (-38.7 to -17.4)

	50-69 years	58<br>(50 to 65)	21.8<br>(19.0 to 24.8)	131<br>(114 to 151)	16.4<br>(14.4 to 18.9)	108<br>(91 to 126)	13.1<br>(11.1 to 15.4)	111<br>(86 to 139)	13.1<br>(10.2 to 16.4)	-40.2<br>(-54.2 to -21.8)	-20.4<br>(-33.9 to -3.4)
	70+ years	150<br>(126 to 172)	204.4<br>(171.1 to 233.7)	336<br>(278 to 410)	141.2<br>(117.2 to 172.3)	243<br>(200 to 303)	99.3<br>(81.6 to 123.9)	238<br>(190 to 302)	95.3<br>(76.2 to 121.0)	-53.4<br>(-62.7 to -41.9)	-32.5<br>(-40.8 to -22.4)
**Panama**	All Ages	433<br>(400 to 465)	18.1<br>(16.7 to 19.5)	712<br>(635 to 766)	17.1<br>(15.3 to 18.4)	594<br>(522 to 656)	14.0<br>(12.3 to 15.5)	600<br>(480 to 719)	14.0<br>(11.2 to 16.8)	-22.9<br>(-36.9 to -8.6)	-18.5<br>(-32.6 to -4.6)
	Under 5	154<br>(131 to 179)	54.1<br>(46.0 to 62.7)	135<br>(110 to 164)	35.6<br>(28.8 to 43.2)	94<br>(73 to 115)	24.8<br>(19.5 to 30.5)	92<br>(69 to 118)	24.7<br>(18.7 to 31.8)	-54.3<br>(-66.5 to -38.8)	-30.5<br>(-42.1 to -17.3)
	5-14 years	14<br>(12 to 16)	2.5<br>(2.2 to 2.8)	9<br>(8 to 11)	1.2<br>(1.1 to 1.4)	8<br>(7 to 10)	1.1<br>(0.9 to 1.2)	8<br>(7 to 9)	1.0<br>(0.9 to 1.2)	-59.9<br>(-67.0 to -51.0)	-19.3<br>(-27.9 to -9.4)
	15-49 years	38<br>(35 to 40)	3.1<br>(2.9 to 3.3)	52<br>(49 to 56)	2.5<br>(2.3 to 2.6)	46<br>(42 to 50)	2.2<br>(2.0 to 2.3)	47<br>(38 to 57)	2.2<br>(1.7 to 2.6)	-29.3<br>(-43.3 to -12.7)	-13.0<br>(-29.9 to 4.1)
	50-69 years	54<br>(50 to 59)	22.4<br>(20.7 to 24.3)	90<br>(83 to 97)	13.5<br>(12.4 to 14.5)	86<br>(76 to 95)	12.4<br>(11.0 to 13.8)	86<br>(65 to 107)	12.1<br>(9.2 to 15.0)	-46.0<br>(-58.9 to -31.4)	-10.1<br>(-30.7 to 9.4)
	70+ years	173<br>(154 to 187)	214.4<br>(191.0 to 232.1)	425<br>(362 to 466)	173.9<br>(148.1 to 190.5)	360<br>(305 to 401)	141.4<br>(119.6 to 157.7)	367<br>(286 to 446)	140.6<br>(109.7 to 170.9)	-34.4<br>(-47.2 to -21.5)	-19.2<br>(-34.2 to -4.2)
**Venezuela (Bolivarian Republic of)**	All Ages	4,200<br>(4,000 to 4,380)	22.3<br>(21.3 to 23.3)	6,840<br>(5,570 to 8,230)	24.2<br>(19.7 to 29.1)	5,830<br>(4,700 to 7,240)	21.6<br>(17.4 to 26.8)	5,580<br>(4,360 to 7,020)	21.0<br>(16.4 to 26.4)	-6.2<br>(-27.1 to 17.5)	-13.5<br>(-26.8 to 2.3)
	Under 5	1,790<br>(1,680 to 1,920)	70.8<br>(66.2 to 75.9)	1,260<br>(959 to 1,630)	50.1<br>(38.1 to 64.6)	727<br>(541 to 979)	31.8<br>(23.6 to 42.7)	563<br>(408 to 770)	25.8<br>(18.7 to 35.3)	-63.6<br>(-74.0 to -50.1)	-48.6<br>(-58.4 to -37.3)
	5-14 years	132<br>(122 to 142)	2.9<br>(2.7 to 3.1)	120<br>(102 to 141)	2.5<br>(2.1 to 3.0)	96<br>(80 to 116)	2.1<br>(1.8 to 2.5)	89<br>(74 to 109)	2.0<br>(1.7 to 2.4)	-30.5<br>(-43.5 to -14.1)	-20.2<br>(-30.0 to -9.4)
	15-49 years	461<br>(438 to 485)	4.8<br>(4.6 to 5.1)	706<br>(565 to 871)	4.9<br>(3.9 to 6.1)	610<br>(479 to 778)	4.5<br>(3.6 to 5.8)	590<br>(449 to 759)	4.5<br>(3.4 to 5.8)	-6.6<br>(-29.5 to 20.5)	-8.7<br>(-25.3 to 12.0)
	50-69 years	483<br>(447 to 519)	29.3<br>(27.1 to 31.5)	1,150<br>(901 to 1,460)	22.6<br>(17.7 to 28.6)	1,100<br>(834 to 1,430)	21.2<br>(16.1 to 27.7)	1,130<br>(827 to 1,480)	21.4<br>(15.8 to 28.2)	-26.8<br>(-46.9 to -3.1)	-5.2<br>(-23.8 to 16.6)
	70+ years	1,330<br>(1,190 to 1,430)	280.8<br>(251.0 to 300.5)	3,600<br>(2,910 to 4,300)	238.5<br>(192.4 to 284.8)	3,300<br>(2,670 to 4,030)	210.7<br>(170.5 to 257.5)	3,210<br>(2,530 to 3,980)	200.0<br>(157.3 to 247.5)	-28.8<br>(-42.8 to -12.0)	-16.1<br>(-28.1 to -2.5)
**Brazil**	All Ages	54,100<br>(50,800 to 57,500)	36.5<br>(34.2 to 38.7)	92,800<br>(81,800 to 98,400)	42.8<br>(37.7 to 45.4)	82,100<br>(72,400 to 88,800)	37.5<br>(33.1 to 40.6)	78,000<br>(68,300 to 85,100)	35.4<br>(31.0 to 38.6)	-2.9<br>(-14.0 to 7.3)	-17.3<br>(-21.3 to -11.7)
	Under 5	25,400<br>(22,600 to 28,600)	154.2<br>(137.2 to 173.6)	3,550<br>(2,830 to 4,400)	21.2<br>(16.9 to 26.3)	2,180<br>(1,690 to 2,780)	13.0<br>(10.1 to 16.6)	1,840<br>(1,400 to 2,340)	11.1<br>(8.5 to 14.1)	-92.8<br>(-94.8 to -90.5)	-47.8<br>(-58.5 to -34.5)
	5-14 years	1,210<br>(1,090 to 1,320)	3.4<br>(3.1 to 3.7)	566<br>(484 to 643)	1.8<br>(1.6 to 2.1)	433<br>(365 to 497)	1.4<br>(1.2 to 1.6)	399<br>(333 to 462)	1.3<br>(1.1 to 1.5)	-63.1<br>(-68.4 to -57.2)	-30.3<br>(-37.1 to -22.2)
	15-49 years	5,690<br>(5,500 to 5,890)	7.4<br>(7.2 to 7.7)	6,960<br>(6,750 to 7,170)	6.0<br>(5.8 to 6.2)	6,280<br>(5,950 to 6,620)	5.4<br>(5.1 to 5.7)	6,320<br>(5,970 to 6,740)	5.5<br>(5.1 to 5.8)	-26.6<br>(-31.3 to -21.4)	-9.6<br>(-13.9 to -3.9)

	50-69 years	6,800 (6,540 to 7,070)	43.4 (41.7 to 45.1)	17,900 (17,200 to 18,500)	44.3 (42.5 to 45.8)	16,700 (15,900 to 17,600)	40.4 (38.4 to 42.6)	16,400 (15,400 to 17,400)	38.8 (36.6 to 41.3)	-10.4 (-15.9 to -3.6)	-12.4 (-16.5 to -7.1)
	70+ years	15,000 (13,600 to 15,900)	354.0 (319.8 to 373.8)	63,900 (53,600 to 68,900)	487.4 (409.0 to 526.2)	56,500 (47,400 to 62,100)	416.6 (349.6 to 457.9)	53,100 (44,100 to 58,900)	377.7 (313.3 to 418.7)	6.7 (-3.7 to 15.4)	-22.5 (-26.4 to -17.1)
**Paraguay**	All Ages	954 (820 to 1,100)	23.6 (20.3 to 27.2)	1,730 (1,420 to 2,090)	24.8 (20.3 to 29.8)	1,310 (1,060 to 1,580)	18.5 (14.9 to 22.3)	1,250 (961 to 1,580)	17.5 (13.4 to 22.0)	-25.9 (-44.6 to -4.6)	-29.4 (-43.2 to -12.8)
	Under 5	500 (392 to 619)	81.0 (63.5 to 100.3)	169 (115 to 237)	25.5 (17.4 to 35.9)	90 (58 to 133)	13.7 (8.8 to 20.2)	83 (51 to 126)	12.8 (7.8 to 19.4)	-84.2 (-91.2 to -74.5)	-50.0 (-62.3 to -35.1)
	5-14 years	34 (27 to 42)	3.3 (2.5 to 4.0)	24 (19 to 30)	1.8 (1.4 to 2.2)	34 (27 to 43)	2.5 (2.0 to 3.2)	19 (15 to 25)	1.4 (1.1 to 1.8)	-56.7 (-68.3 to -40.3)	-21.0 (-32.1 to -7.4)
	15-49 years	64 (53 to 75)	3.4 (2.8 to 4.0)	153 (120 to 192)	4.1 (3.2 to 5.1)	117 (92 to 144)	3.1 (2.4 to 3.8)	116 (86 to 151)	3.0 (2.2 to 3.9)	-10.4 (-35.8 to 23.2)	-26.3 (-44.7 to -2.9)
	50-69 years	79 (64 to 95)	21.4 (17.6 to 25.9)	284 (216 to 359)	29.7 (22.6 to 37.6)	244 (186 to 312)	24.8 (18.9 to 31.8)	244 (174 to 328)	24.4 (17.4 to 32.8)	13.6 (-21.1 to 60.6)	-17.9 (-39.7 to 11.3)
	70+ years	278 (226 to 341)	239.6 (194.6 to 294.3)	1,100 (874 to 1,360)	387.5 (306.6 to 476.8)	825 (645 to 1,000)	282.5 (220.8 to 344.1)	791 (598 to 1,010)	262.2 (198.2 to 333.5)	9.4 (-17.0 to 43.2)	-32.3 (-45.4 to -17.2)
**Afghanistan**	All Ages	17,200 (13,700 to 21,700)	173.0 (138.0 to 218.2)	13,200 (10,600 to 16,400)	45.1 (36.3 to 56.0)	11,400 (8,940 to 14,500)	37.8 (29.6 to 47.9)	9,600 (7,690 to 11,800)	30.7 (24.6 to 37.8)	-82.2 (-86.1 to -77.2)	-31.9 (-39.7 to -23.3)
	Under 5	13,600 (10,300 to 18,100)	790.8 (601.2 to 1,056.7)	8,480 (6,250 to 11,200)	163.8 (120.8 to 216.2)	7,050 (5,060 to 9,560)	132.3 (95.1 to 179.5)	5,600 (4,010 to 7,680)	102.2 (73.1 to 140.2)	-87.1 (-90.9 to -81.5)	-37.6 (-47.7 to -26.0)
	5-14 years	380 (233 to 528)	14.7 (9.0 to 20.4)	652 (488 to 849)	7.9 (5.9 to 10.3)	593 (440 to 769)	7.0 (5.2 to 9.1)	529 (392 to 688)	6.1 (4.5 to 7.9)	-58.6 (-71.2 to -38.4)	-23.2 (-34.8 to -8.9)
	15-49 years	495 (370 to 649)	12.4 (9.3 to 16.2)	1,310 (964 to 1,750)	9.4 (6.9 to 12.6)	1,230 (911 to 1,650)	8.6 (6.4 to 11.5)	1,150 (844 to 1,520)	7.8 (5.7 to 10.3)	-37.1 (-54.2 to -11.4)	-17.1 (-24.3 to -9.6)
	50-69 years	1,120 (817 to 1,450)	84.1 (61.6 to 109.2)	820 (592 to 1,090)	54.7 (39.5 to 72.8)	807 (577 to 1,070)	48.3 (34.6 to 64.3)	770 (563 to 1,030)	41.9 (30.6 to 56.2)	-50.2 (-64.6 to -27.8)	-23.5 (-30.8 to -16.6)
	70+ years	1,650 (1,260 to 2,020)	531.8 (406.8 to 649.3)	1,960 (1,460 to 2,430)	455.6 (339.5 to 564.4)	1,750 (1,290 to 2,170)	423.5 (312.0 to 526.7)	1,550 (1,160 to 1,910)	382.3 (288.0 to 471.2)	-28.1 (-44.9 to -6.5)	-16.1 (-22.3 to -8.8)
**Algeria**	All Ages	6,470 (5,270 to 8,130)	25.6 (20.9 to 32.2)	5,770 (4,800 to 6,960)	13.5 (11.2 to 16.2)	5,280 (4,290 to 6,490)	12.1 (9.8 to 14.9)	4,830 (3,940 to 5,960)	10.9 (8.9 to 13.5)	-57.3 (-69.2 to -44.3)	-18.9 (-25.0 to -12.5)
	Under 5	4,120 (2,970 to 5,920)	110.2 (79.4 to 158.4)	957 (717 to 1,270)	19.8 (14.9 to 26.3)	721 (519 to 998)	15.1 (10.8 to 20.9)	554 (391 to 756)	11.8 (8.3 to 16.1)	-89.3 (-93.1 to -83.5)	-40.6 (-49.2 to -31.6)
	5-14 years	388 (289 to 573)	5.6 (4.1 to 8.2)	147 (108 to 219)	1.8 (1.3 to 2.7)	134 (99 to 203)	1.6 (1.2 to 2.4)	128 (92 to 194)	1.5 (1.1 to 2.3)	-73.2 (-80.6 to -63.5)	-18.7 (-25.9 to -10.9)
	15-49 years	421 (327 to 595)	3.6 (2.8 to 5.1)	560 (436 to 752)	2.5 (2.0 to 3.4)	526 (401 to 712)	2.3 (1.8 to 3.2)	500 (384 to 685)	2.2 (1.7 to 3.0)	-38.3 (-52.7 to -18.5)	-11.8 (-19.4 to -3.9)

	50-69 years	398 (298 to 528)	18.4 (13.7 to 24.3)	807 (608 to 1,050)	13.3 (10.0 to 17.3)	788 (580 to 1,040)	12.5 (9.2 to 16.4)	762 (560 to 1,000)	11.7 (8.6 to 15.4)	-36.4 (-55.1 to -9.9)	-11.9 (-21.3 to -1.8)
	70+ years	1,140 (895 to 1,480)	183.0 (143.7 to 237.8)	3,300 (2,620 to 4,140)	204.1 (161.7 to 255.9)	3,120 (2,430 to 3,970)	185.1 (144.4 to 235.5)	2,880 (2,270 to 3,650)	166.6 (131.4 to 210.9)	-9.0 (-30.9 to 19.1)	-18.4 (-25.1 to -11.1)
**Bahrain**	All Ages	63 (57 to 72)	12.5 (11.2 to 14.1)	95 (82 to 110)	6.4 (5.5 to 7.4)	89 (74 to 108)	5.9 (4.9 to 7.1)	83 (68 to 100)	5.4 (4.5 to 6.5)	-56.8 (-65.0 to -46.2)	-15.1 (-23.0 to -6.2)
	Under 5	28 (24 to 35)	46.0 (38.4 to 57.6)	10 (9 to 12)	10.5 (8.8 to 12.4)	7 (5 to 9)	6.9 (5.1 to 9.3)	4 (3 to 6)	4.7 (3.4 to 6.1)	-89.8 (-92.9 to -85.9)	-55.4 (-64.6 to -45.7)
	5-14 years	2 (2 to 3)	2.1 (1.6 to 2.6)	3 (2 to 3)	1.3 (1.0 to 1.6)	2 (2 to 3)	1.1 (0.8 to 1.4)	2 (1 to 2)	0.9 (0.7 to 1.2)	-54.6 (-67.5 to -36.4)	-26.5 (-34.1 to -18.7)
	15-49 years	7 (6 to 8)	2.3 (2.0 to 2.8)	18 (15 to 21)	1.8 (1.5 to 2.1)	16 (13 to 20)	1.6 (1.3 to 2.0)	15 (12 to 18)	1.5 (1.2 to 1.9)	-35.7 (-50.1 to -16.5)	-16.6 (-25.1 to -7.1)
	50-69 years	9 (8 to 12)	26.5 (21.6 to 32.4)	21 (17 to 27)	10.6 (8.3 to 13.4)	21 (16 to 27)	9.7 (7.5 to 12.6)	21 (16 to 27)	9.1 (6.9 to 12.0)	-65.8 (-75.4 to -52.8)	-14.4 (-24.7 to -3.8)
	70+ years	17 (14 to 20)	288.7 (241.4 to 347.0)	43 (36 to 52)	215.4 (178.9 to 260.5)	43 (35 to 54)	185.9 (151.2 to 231.8)	41 (33 to 51)	163.0 (131.8 to 202.0)	-43.6 (-56.1 to -26.7)	-24.4 (-31.7 to -15.9)
**Egypt**	All Ages	53,300 (45,600 to 62,800)	96.4 (82.5 to 113.4)	27,000 (24,100 to 30,100)	26.4 (23.5 to 29.4)	24,600 (21,200 to 28,300)	23.7 (20.3 to 27.2)	21,500 (17,700 to 26,000)	20.3 (16.7 to 24.6)	-78.9 (-83.7 to -73.2)	-22.9 (-32.5 to -13.2)
	Under 5	42,200 (34,900 to 51,400)	494.2 (409.5 to 602.3)	10,400 (7,990 to 13,200)	75.8 (58.3 to 96.1)	8,020 (6,070 to 10,100)	59.9 (45.4 to 75.1)	6,020 (4,570 to 7,720)	46.2 (35.1 to 59.2)	-90.7 (-93.5 to -87.0)	-39.1 (-48.7 to -27.9)
	5-14 years	2,870 (2,110 to 3,510)	21.0 (15.4 to 25.7)	854 (693 to 1,050)	3.8 (3.1 to 4.7)	778 (617 to 953)	3.4 (2.7 to 4.1)	697 (546 to 864)	2.9 (2.3 to 3.6)	-86.1 (-90.2 to -79.2)	-23.1 (-32.1 to -13.2)
	15-49 years	2,100 (1,850 to 2,310)	7.8 (6.9 to 8.6)	2,420 (2,170 to 2,650)	4.7 (4.2 to 5.1)	2,420 (2,070 to 2,800)	4.6 (3.9 to 5.3)	2,250 (1,820 to 2,760)	4.2 (3.4 to 5.2)	-46.0 (-57.0 to -32.7)	-9.9 (-25.4 to 7.3)
	50-69 years	2,780 (2,400 to 3,200)	52.2 (45.0 to 60.1)	5,590 (4,840 to 6,350)	44.8 (38.8 to 50.9)	5,630 (4,700 to 6,780)	44.0 (36.8 to 53.0)	5,300 (4,110 to 6,830)	40.6 (31.5 to 52.3)	-22.1 (-40.6 to 2.9)	-9.3 (-26.3 to 10.9)
	70+ years	3,430 (2,860 to 4,810)	349.0 (291.0 to 489.1)	7,740 (6,850 to 8,860)	369.9 (327.4 to 423.7)	7,780 (6,670 to 9,140)	356.4 (305.3 to 418.6)	7,220 (5,970 to 8,730)	317.8 (262.9 to 384.5)	-8.9 (-32.0 to 17.1)	-14.1 (-25.4 to -2.1)
**Iran (Islamic Republic of)**	All Ages	13,600 (11,700 to 17,100)	23.7 (20.5 to 30.0)	9,730 (8,520 to 10,500)	11.5 (10.1 to 12.5)	8,850 (7,640 to 9,750)	10.4 (9.0 to 11.5)	8,600 (7,380 to 9,540)	10.1 (8.6 to 11.2)	-57.6 (-67.2 to -49.1)	-12.7 (-19.4 to -6.3)
	Under 5	9,680 (7,860 to 13,300)	110.4 (89.6 to 151.1)	610 (428 to 746)	8.8 (6.2 to 10.7)	331 (228 to 415)	5.0 (3.5 to 6.3)	184 (115 to 254)	3.0 (1.9 to 4.1)	-97.3 (-98.7 to -96.0)	-66.1 (-77.1 to -55.0)
	5-14 years	644 (545 to 860)	3.9 (3.3 to 5.2)	221 (184 to 273)	1.7 (1.4 to 2.0)	216 (179 to 264)	1.6 (1.3 to 1.9)	206 (170 to 253)	1.5 (1.2 to 1.8)	-62.1 (-69.0 to -53.9)	-11.2 (-18.8 to -4.3)
	15-49 years	711 (643 to 831)	2.8 (2.5 to 3.3)	1,160 (1,080 to 1,260)	2.4 (2.3 to 2.7)	1,040 (948 to 1,140)	2.2 (2.0 to 2.4)	1,040 (937 to 1,140)	2.2 (2.0 to 2.4)	-21.1 (-31.7 to -10.3)	-10.2 (-17.5 to -3.0)

	50-69 years	983<br>(886 to 1,090)	18.8<br>(17.0 to 20.9)	1,680<br>(1,510 to 1,810)	12.8<br>(11.5 to 13.8)	1,560<br>(1,400 to 1,700)	11.5<br>(10.2 to 12.5)	1,600<br>(1,420 to 1,750)	11.3<br>(10.1 to 12.4)	-40.1<br>(-47.4 to -32.6)	-11.8<br>(-18.6 to -5.1)
	70+ years	1,530<br>(1,310 to 1,710)	157.9<br>(134.9 to 176.5)	6,060<br>(5,060 to 6,700)	174.0<br>(145.3 to 192.5)	5,700<br>(4,690 to 6,400)	159.1<br>(130.8 to 178.7)	5,570<br>(4,620 to 6,290)	152.5<br>(126.4 to 172.3)	-3.4<br>(-14.8 to 7.6)	-12.4<br>(-19.2 to -5.9)
**Iraq**	All Ages	5,830<br>(4,760 to 7,280)	31.6<br>(25.9 to 39.5)	3,660<br>(2,980 to 4,480)	9.2<br>(7.5 to 11.2)	3,260<br>(2,650 to 3,940)	8.0<br>(6.5 to 9.7)	2,910<br>(2,360 to 3,560)	7.1<br>(5.7 to 8.6)	-77.7<br>(-84.1 to -70.5)	-23.0<br>(-29.0 to -15.9)
	Under 5	4,530<br>(3,540 to 5,880)	144.1<br>(112.8 to 187.1)	1,570<br>(1,170 to 2,050)	35.1<br>(26.1 to 45.8)	1,220<br>(909 to 1,630)	27.8<br>(20.7 to 37.1)	1,010<br>(731 to 1,390)	23.5<br>(17.0 to 32.3)	-83.7<br>(-89.5 to -75.7)	-33.1<br>(-42.9 to -22.7)
	5-14 years	373<br>(279 to 479)	7.3<br>(5.5 to 9.4)	244<br>(189 to 315)	2.7<br>(2.1 to 3.5)	156<br>(115 to 206)	1.7<br>(1.3 to 2.3)	139<br>(100 to 186)	1.5<br>(1.1 to 2.0)	-79.3<br>(-87.1 to -69.7)	-43.7<br>(-54.1 to -33.6)
	15-49 years	275<br>(222 to 341)	3.2<br>(2.6 to 4.0)	470<br>(350 to 637)	2.2<br>(1.6 to 3.0)	420<br>(313 to 583)	1.9<br>(1.4 to 2.7)	394<br>(297 to 554)	1.8<br>(1.3 to 2.5)	-45.2<br>(-60.1 to -19.6)	-19.7<br>(-26.6 to -10.6)
	50-69 years	239<br>(186 to 310)	18.2<br>(14.2 to 23.5)	521<br>(369 to 707)	12.3<br>(8.7 to 16.7)	538<br>(375 to 725)	12.2<br>(8.5 to 16.4)	528<br>(369 to 701)	11.4<br>(8.0 to 15.2)	-37.0<br>(-57.4 to -7.9)	-7.3<br>(-16.6 to 3.0)
	70+ years	414<br>(325 to 566)	103.6<br>(81.3 to 141.7)	860<br>(663 to 1,170)	90.6<br>(69.8 to 123.2)	926<br>(710 to 1,240)	95.2<br>(73.0 to 127.8)	843<br>(660 to 1,140)	85.0<br>(66.5 to 114.9)	-18.0<br>(-38.1 to 6.1)	-6.1<br>(-15.7 to 2.6)
**Jordan**	All Ages	824<br>(717 to 948)	22.1<br>(19.2 to 25.4)	903<br>(760 to 1,060)	7.8<br>(6.6 to 9.2)	871<br>(704 to 1,060)	7.3<br>(5.9 to 8.9)	812<br>(663 to 997)	6.6<br>(5.4 to 8.1)	-70.1<br>(-76.8 to -61.9)	-16.0<br>(-23.6 to -7.6)
	Under 5	533<br>(440 to 640)	88.8<br>(73.3 to 106.6)	247<br>(198 to 307)	22.4<br>(17.9 to 27.7)	203<br>(156 to 260)	18.4<br>(14.2 to 23.6)	165<br>(128 to 213)	15.0<br>(11.7 to 19.4)	-83.1<br>(-87.7 to -76.0)	-32.9<br>(-41.0 to -23.2)
	5-14 years	48<br>(38 to 60)	4.6<br>(3.7 to 5.8)	52<br>(41 to 65)	2.1<br>(1.7 to 2.6)	47<br>(37 to 60)	1.9<br>(1.5 to 2.4)	42<br>(34 to 53)	1.7<br>(1.3 to 2.1)	-64.0<br>(-74.0 to -50.4)	-19.6<br>(-27.1 to -11.4)
	15-49 years	70<br>(57 to 85)	3.9<br>(3.2 to 4.8)	130<br>(104 to 159)	2.0<br>(1.6 to 2.5)	129<br>(100 to 162)	2.0<br>(1.5 to 2.5)	125<br>(96 to 157)	1.8<br>(1.4 to 2.3)	-53.5<br>(-66.0 to -36.2)	-11.1<br>(-19.5 to -2.2)
	50-69 years	61<br>(47 to 79)	22.3<br>(17.2 to 29.1)	134<br>(103 to 172)	10.3<br>(7.9 to 13.2)	142<br>(106 to 186)	10.0<br>(7.4 to 13.1)	143<br>(105 to 192)	9.3<br>(6.8 to 12.5)	-58.5<br>(-71.6 to -39.9)	-9.8<br>(-20.6 to 0.5)
	70+ years	112<br>(90 to 139)	228.9<br>(184.5 to 284.4)	339<br>(269 to 417)	122.1<br>(96.8 to 150.2)	351<br>(269 to 444)	120.2<br>(92.1 to 152.1)	337<br>(264 to 424)	109.8<br>(86.0 to 138.1)	-52.0<br>(-63.9 to -37.4)	-10.0<br>(-18.6 to -0.7)
**Kuwait**	All Ages	177<br>(165 to 190)	10.3<br>(9.6 to 11.0)	631<br>(560 to 678)	14.4<br>(12.8 to 15.5)	620<br>(542 to 706)	13.7<br>(12.0 to 15.6)	553<br>(457 to 654)	11.9<br>(9.8 to 14.1)	15.4<br>(-5.5 to 38.1)	-17.4<br>(-29.0 to -4.6)
	Under 5	72<br>(63 to 82)	35.1<br>(30.8 to 40.2)	71<br>(63 to 80)	25.6<br>(22.5 to 28.8)	52<br>(44 to 63)	19.2<br>(16.2 to 23.0)	45<br>(36 to 56)	16.9<br>(13.5 to 21.1)	-51.8<br>(-62.7 to -38.4)	-33.9<br>(-44.3 to -22.0)
	5-14 years	14<br>(12 to 15)	3.9<br>(3.4 to 4.4)	17<br>(15 to 19)	3.0<br>(2.7 to 3.3)	15<br>(13 to 17)	2.6<br>(2.3 to 3.0)	13<br>(11 to 15)	2.2<br>(1.9 to 2.6)	-43.6<br>(-54.1 to -30.9)	-26.6<br>(-35.6 to -16.0)
	15-49 years	31<br>(28 to 35)	3.0<br>(2.7 to 3.4)	75<br>(70 to 81)	2.6<br>(2.4 to 2.8)	76<br>(67 to 86)	2.6<br>(2.3 to 2.9)	68<br>(57 to 83)	2.2<br>(1.9 to 2.7)	-26.5<br>(-41.0 to -7.9)	-14.3<br>(-27.7 to 2.4)

	50-69 years	23<br>(21 to 26)	19.4<br>(17.2 to 21.7)	88<br>(81 to 96)	15.8<br>(14.5 to 17.1)	97<br>(85 to 110)	15.9<br>(13.9 to 18.0)	93<br>(75 to 114)	14.1<br>(11.4 to 17.3)	-27.1<br>(-43.3 to -7.3)	-10.5<br>(-26.0 to 7.8)
	70+ years	37<br>(32 to 42)	186.7<br>(159.0 to 209.2)	379<br>(313 to 421)	398.6<br>(329.0 to 443.1)	379<br>(313 to 441)	399.8<br>(330.2 to 465.3)	334<br>(260 to 397)	338.3<br>(263.1 to 401.9)	81.3<br>(50.1 to 115.2)	-15.1<br>(-26.7 to -2.7)
**Lebanon**	All Ages	808<br>(654 to 1,020)	27.0<br>(21.9 to 34.0)	1,970<br>(1,620 to 2,310)	35.5<br>(29.1 to 41.6)	1,920<br>(1,560 to 2,270)	34.6<br>(28.2 to 40.9)	1,830<br>(1,480 to 2,160)	33.1<br>(26.6 to 39.1)	22.5<br>(-8.6 to 56.5)	-6.9<br>(-13.0 to 0.3)
	Under 5	388<br>(281 to 540)	99.5<br>(72.1 to 138.7)	91<br>(64 to 128)	20.4<br>(14.4 to 28.8)	71<br>(49 to 101)	16.7<br>(11.5 to 23.8)	60<br>(40 to 87)	14.7<br>(9.9 to 21.3)	-85.2<br>(-91.3 to -75.5)	-28.1<br>(-38.1 to -17.0)
	5-14 years	33<br>(25 to 42)	5.0<br>(3.8 to 6.4)	17<br>(13 to 21)	1.9<br>(1.5 to 2.4)	15<br>(12 to 18)	1.7<br>(1.3 to 2.1)	13<br>(10 to 16)	1.5<br>(1.2 to 1.9)	-69.7<br>(-77.8 to -58.6)	-21.3<br>(-29.7 to -12.3)
	15-49 years	66<br>(51 to 94)	4.6<br>(3.5 to 6.5)	95<br>(80 to 113)	3.1<br>(2.6 to 3.7)	91<br>(74 to 109)	3.0<br>(2.5 to 3.6)	87<br>(71 to 106)	2.9<br>(2.4 to 3.5)	-36.5<br>(-56.5 to -13.6)	-7.9<br>(-16.2 to 1.2)
	50-69 years	93<br>(68 to 144)	22.7<br>(16.6 to 35.0)	182<br>(148 to 221)	22.0<br>(17.9 to 26.7)	177<br>(141 to 222)	20.8<br>(16.6 to 26.2)	168<br>(132 to 215)	19.5<br>(15.2 to 24.9)	-14.0<br>(-46.8 to 28.9)	-11.4<br>(-19.9 to -1.1)
	70+ years	228<br>(176 to 366)	238.8<br>(184.1 to 383.2)	1,590<br>(1,260 to 1,880)	425.0<br>(337.8 to 504.5)	1,570<br>(1,250 to 1,870)	413.5<br>(330.5 to 492.0)	1,500<br>(1,180 to 1,790)	391.3<br>(308.2 to 465.9)	63.9<br>(-3.7 to 125.0)	-7.9<br>(-14.2 to -1.0)
**Libya**	All Ages	717<br>(584 to 897)	17.0<br>(13.9 to 21.3)	973<br>(772 to 1,290)	14.3<br>(11.4 to 18.9)	896<br>(695 to 1,210)	13.1<br>(10.2 to 17.7)	816<br>(633 to 1,100)	11.9<br>(9.2 to 16.0)	-30.1<br>(-47.2 to -1.1)	-17.0<br>(-23.6 to -9.6)
	Under 5	369<br>(274 to 503)	58.0<br>(43.1 to 78.9)	121<br>(82 to 181)	26.5<br>(18.0 to 39.7)	89<br>(57 to 134)	20.3<br>(13.0 to 30.5)	64<br>(43 to 92)	15.0<br>(10.2 to 21.8)	-74.1<br>(-84.2 to -58.8)	-43.4<br>(-52.6 to -32.3)
	5-14 years	30<br>(22 to 45)	2.6<br>(1.9 to 3.8)	25<br>(19 to 36)	2.3<br>(1.7 to 3.2)	22<br>(17 to 31)	2.0<br>(1.5 to 2.8)	19<br>(15 to 27)	1.8<br>(1.4 to 2.6)	-29.5<br>(-48.2 to -3.2)	-19.5<br>(-27.0 to -10.9)
	15-49 years	63<br>(49 to 88)	3.2<br>(2.5 to 4.4)	169<br>(128 to 226)	4.1<br>(3.1 to 5.5)	157<br>(116 to 211)	3.8<br>(2.8 to 5.1)	146<br>(109 to 199)	3.5<br>(2.7 to 4.8)	10.9<br>(-18.7 to 58.1)	-14.1<br>(-21.5 to -5.6)
	50-69 years	72<br>(52 to 99)	21.2<br>(15.5 to 29.2)	192<br>(142 to 271)	21.1<br>(15.6 to 29.7)	193<br>(139 to 274)	19.8<br>(14.3 to 28.1)	191<br>(135 to 274)	18.4<br>(13.0 to 26.4)	-13.1<br>(-41.8 to 37.1)	-12.5<br>(-21.3 to -3.1)
	70+ years	182<br>(135 to 237)	209.6<br>(155.0 to 272.5)	466<br>(361 to 637)	218.2<br>(169.0 to 298.1)	434<br>(335 to 599)	201.5<br>(155.3 to 277.8)	396<br>(304 to 551)	180.9<br>(138.6 to 251.6)	-13.7<br>(-34.8 to 26.1)	-17.1<br>(-24.1 to -9.3)
**Morocco**	All Ages	12,700<br>(10,500 to 15,500)	50.1<br>(41.3 to 61.0)	6,840<br>(5,290 to 8,490)	18.6<br>(14.4 to 23.1)	6,250<br>(4,820 to 7,700)	16.9<br>(13.0 to 20.8)	5,710<br>(4,480 to 7,050)	15.4<br>(12.1 to 19.0)	-69.3<br>(-76.4 to -60.8)	-17.6<br>(-23.6 to -10.4)
	Under 5	9,740<br>(7,720 to 12,200)	273.6<br>(216.7 to 341.5)	1,140<br>(739 to 1,590)	33.3<br>(21.6 to 46.4)	867<br>(536 to 1,220)	26.0<br>(16.0 to 36.6)	671<br>(412 to 962)	20.6<br>(12.7 to 29.6)	-92.5<br>(-95.6 to -88.3)	-38.0<br>(-46.3 to -29.2)
	5-14 years	319<br>(211 to 502)	5.1<br>(3.4 to 8.1)	117<br>(71 to 188)	1.8<br>(1.1 to 2.9)	101<br>(61 to 160)	1.5<br>(0.9 to 2.4)	89<br>(53 to 144)	1.4<br>(0.8 to 2.2)	-73.4<br>(-82.9 to -61.1)	-23.8<br>(-33.0 to -14.1)
	15-49 years	492<br>(376 to 701)	4.0<br>(3.0 to 5.6)	558<br>(405 to 854)	2.9<br>(2.1 to 4.5)	519<br>(372 to 778)	2.7<br>(1.9 to 4.0)	486<br>(350 to 751)	2.5<br>(1.8 to 3.9)	-36.7<br>(-54.6 to -2.0)	-14.0<br>(-23.2 to -2.6)

	50-69 years	625<br>(490 to 816)	25.9<br>(20.3 to 33.8)	1,390<br>(951 to 1,830)	23.2<br>(15.9 to 30.6)	1,360<br>(937 to 1,780)	22.1<br>(15.2 to 28.9)	1,300<br>(921 to 1,690)	20.6<br>(14.6 to 26.8)	-20.3<br>(-45.6 to 6.1)	-11.3<br>(-19.6 to -1.1)
	70+ years	1,520<br>(1,200 to 1,880)	213.8<br>(168.4 to 265.1)	3,640<br>(2,780 to 4,550)	227.5<br>(174.0 to 284.7)	3,400<br>(2,590 to 4,220)	211.4<br>(161.0 to 261.8)	3,160<br>(2,430 to 3,890)	191.3<br>(147.0 to 235.4)	-10.5<br>(-30.9 to 13.1)	-15.9<br>(-23.2 to -6.5)
**Oman**	All Ages	495<br>(394 to 662)	25.0<br>(19.9 to 33.4)	466<br>(392 to 548)	10.8<br>(9.1 to 12.7)	375<br>(313 to 444)	8.3<br>(6.9 to 9.8)	351<br>(287 to 423)	7.5<br>(6.1 to 9.0)	-70.1<br>(-78.6 to -59.7)	-30.8<br>(-36.5 to -24.4)
	Under 5	279<br>(204 to 431)	85.1<br>(62.2 to 131.6)	85<br>(69 to 102)	20.2<br>(16.5 to 24.4)	56<br>(45 to 68)	13.3<br>(10.6 to 16.2)	49<br>(38 to 62)	11.5<br>(8.9 to 14.5)	-86.5<br>(-91.7 to -79.6)	-43.2<br>(-50.3 to -35.0)
	5-14 years	22<br>(17 to 29)	4.4<br>(3.3 to 5.7)	16<br>(13 to 19)	2.2<br>(1.8 to 2.7)	9<br>(7 to 10)	1.1<br>(0.9 to 1.4)	8<br>(7 to 10)	1.0<br>(0.8 to 1.3)	-75.9<br>(-82.8 to -66.1)	-51.9<br>(-57.1 to -45.3)
	15-49 years	42<br>(31 to 55)	4.2<br>(3.1 to 5.6)	80<br>(65 to 97)	2.9<br>(2.4 to 3.5)	53<br>(43 to 65)	1.9<br>(1.5 to 2.3)	56<br>(45 to 68)	1.9<br>(1.5 to 2.3)	-55.2<br>(-68.6 to -35.2)	-35.6<br>(-41.8 to -28.4)
	50-69 years	54<br>(39 to 73)	44.8<br>(32.6 to 61.1)	91<br>(70 to 117)	25.1<br>(19.3 to 32.1)	71<br>(54 to 92)	18.3<br>(13.9 to 23.7)	74<br>(56 to 96)	17.8<br>(13.4 to 22.9)	-60.3<br>(-73.3 to -42.4)	-29.4<br>(-37.4 to -21.4)
	70+ years	99<br>(76 to 126)	342.8<br>(264.5 to 437.4)	194<br>(156 to 236)	314.5<br>(252.3 to 381.6)	186<br>(149 to 226)	281.6<br>(226.2 to 341.6)	164<br>(128 to 202)	237.7<br>(185.0 to 292.0)	-30.7<br>(-50.2 to -4.6)	-24.4<br>(-30.6 to -16.9)
**Palestine**	All Ages	626<br>(518 to 749)	30.6<br>(25.3 to 36.6)	559<br>(488 to 633)	11.2<br>(9.8 to 12.7)	502<br>(420 to 589)	9.9<br>(8.3 to 11.7)	462<br>(391 to 534)	9.0<br>(7.6 to 10.4)	-70.5<br>(-76.3 to -63.1)	-20.0<br>(-25.5 to -13.8)
	Under 5	382<br>(291 to 498)	98.0<br>(74.6 to 127.7)	134<br>(100 to 175)	21.4<br>(16.0 to 27.9)	103<br>(74 to 139)	16.6<br>(11.9 to 22.4)	82<br>(58 to 115)	13.4<br>(9.5 to 18.8)	-86.4<br>(-91.8 to -78.0)	-37.5<br>(-45.9 to -28.8)
	5-14 years	30<br>(23 to 38)	5.2<br>(4.0 to 6.5)	34<br>(28 to 41)	2.8<br>(2.2 to 3.3)	33<br>(27 to 40)	2.7<br>(2.1 to 3.2)	25<br>(20 to 30)	2.0<br>(1.6 to 2.4)	-61.7<br>(-71.3 to -48.2)	-28.3<br>(-35.6 to -21.2)
	15-49 years	34<br>(26 to 44)	3.8<br>(2.9 to 5.0)	64<br>(55 to 74)	2.5<br>(2.2 to 2.9)	59<br>(48 to 71)	2.3<br>(1.9 to 2.7)	56<br>(46 to 67)	2.1<br>(1.7 to 2.5)	-44.0<br>(-59.7 to -24.5)	-16.5<br>(-23.3 to -9.6)
	50-69 years	46<br>(34 to 61)	30.6<br>(22.5 to 40.5)	95<br>(79 to 113)	20.3<br>(16.9 to 24.0)	91<br>(73 to 113)	18.7<br>(14.8 to 23.0)	90<br>(72 to 110)	17.7<br>(14.1 to 21.6)	-42.1<br>(-58.9 to -16.8)	-12.6<br>(-21.1 to -4.4)
	70+ years	134<br>(107 to 167)	318.5<br>(253.8 to 395.8)	231<br>(189 to 271)	236.7<br>(194.0 to 277.3)	216<br>(170 to 259)	214.0<br>(169.1 to 257.4)	209<br>(166 to 249)	201.4<br>(160.3 to 240.3)	-36.8<br>(-51.7 to -14.4)	-14.9<br>(-21.5 to -7.7)
**Qatar**	All Ages	36<br>(29 to 44)	8.0<br>(6.6 to 9.8)	100<br>(81 to 124)	3.5<br>(2.9 to 4.4)	80<br>(64 to 99)	2.8<br>(2.2 to 3.4)	69<br>(55 to 86)	2.3<br>(1.8 to 2.9)	-71.3<br>(-77.8 to -62.5)	-34.8<br>(-40.1 to -29.6)
	Under 5	15<br>(11 to 21)	30.1<br>(21.5 to 40.4)	13<br>(9 to 17)	7.2<br>(5.4 to 9.5)	9<br>(7 to 12)	5.2<br>(3.9 to 6.9)	7<br>(5 to 9)	3.6<br>(2.6 to 4.9)	-88.1<br>(-92.5 to -81.5)	-50.3<br>(-57.6 to -43.1)
	5-14 years	2<br>(1 to 2)	2.3<br>(1.7 to 2.9)	3<br>(2 to 4)	1.0<br>(0.8 to 1.3)	2<br>(2 to 3)	0.7<br>(0.5 to 0.9)	2<br>(1 to 2)	0.6<br>(0.4 to 0.7)	-74.5<br>(-81.9 to -63.9)	-44.6<br>(-51.1 to -38.2)
	15-49 years	6<br>(5 to 8)	2.2<br>(1.7 to 2.7)	34<br>(26 to 44)	1.6<br>(1.2 to 2.1)	23<br>(18 to 30)	1.1<br>(0.8 to 1.4)	21<br>(16 to 28)	1.0<br>(0.7 to 1.3)	-55.5<br>(-68.8 to -36.8)	-40.5<br>(-46.5 to -33.8)

	50-69 years	5 (4 to 6)	19.0 (14.7 to 24.5)	22 (16 to 29)	9.1 (6.6 to 12.0)	18 (13 to 24)	6.9 (4.9 to 9.3)	17 (12 to 23)	6.1 (4.3 to 8.4)	-67.9 (-78.8 to -50.5)	-32.4 (-40.6 to -24.0)
	70+ years	8 (6 to 10)	311.6 (251.6 to 396.2)	28 (22 to 35)	171.8 (134.0 to 216.4)	27 (21 to 34)	163.6 (127.1 to 205.7)	22 (17 to 28)	127.4 (99.3 to 159.2)	-59.1 (-70.0 to -44.9)	-25.8 (-32.1 to -19.7)
**Saudi Arabia**	All Ages	4,300 (3,420 to 5,270)	27.1 (21.6 to 33.2)	4,590 (3,760 to 5,580)	12.7 (10.4 to 15.4)	4,300 (3,440 to 5,360)	11.6 (9.3 to 14.5)	4,030 (3,180 to 5,080)	10.7 (8.4 to 13.5)	-60.5 (-71.5 to -46.9)	-15.7 (-22.1 to -9.1)
	Under 5	2,090 (1,550 to 2,910)	86.5 (64.3 to 120.5)	152 (102 to 206)	6.0 (4.1 to 8.1)	103 (68 to 145)	4.1 (2.7 to 5.8)	78 (49 to 111)	3.2 (2.0 to 4.6)	-96.3 (-98.1 to -94.0)	-47.0 (-57.2 to -36.7)
	5-14 years	189 (137 to 264)	4.6 (3.3 to 6.4)	52 (38 to 69)	1.0 (0.7 to 1.4)	45 (32 to 61)	0.9 (0.6 to 1.2)	40 (29 to 55)	0.8 (0.6 to 1.1)	-82.9 (-88.9 to -75.2)	-23.2 (-30.7 to -15.4)
	15-49 years	505 (367 to 684)	6.3 (4.6 to 8.5)	1,630 (1,210 to 2,130)	6.7 (5.0 to 8.7)	1,540 (1,120 to 2,050)	6.2 (4.5 to 8.3)	1,440 (1,040 to 1,930)	5.7 (4.1 to 7.6)	-9.7 (-42.2 to 40.0)	-15.1 (-23.7 to -5.8)
	50-69 years	550 (398 to 736)	53.1 (38.4 to 71.1)	1,450 (1,160 to 1,780)	37.8 (30.3 to 46.6)	1,390 (1,090 to 1,740)	34.4 (26.9 to 43.0)	1,360 (1,050 to 1,730)	31.5 (24.4 to 40.0)	-40.7 (-59.6 to -12.8)	-16.7 (-23.6 to -9.8)
	70+ years	960 (746 to 1,200)	386.8 (300.6 to 485.0)	1,310 (1,100 to 1,560)	276.6 (231.3 to 328.9)	1,220 (1,000 to 1,510)	241.7 (197.9 to 298.4)	1,110 (912 to 1,360)	214.5 (175.7 to 261.5)	-44.6 (-57.7 to -25.8)	-22.4 (-28.2 to -16.2)
**Sudan**	All Ages	21,000 (16,300 to 29,100)	105.0 (81.5 to 145.2)	7,630 (5,770 to 9,810)	18.2 (13.8 to 23.4)	6,720 (5,050 to 8,640)	15.7 (11.8 to 20.2)	5,900 (4,390 to 7,690)	13.6 (10.1 to 17.7)	-87.1 (-91.7 to -81.5)	-25.4 (-32.1 to -19.2)
	Under 5	18,200 (13,500 to 26,700)	524.1 (388.2 to 767.0)	3,660 (2,620 to 5,140)	63.6 (45.5 to 89.5)	2,970 (2,060 to 4,160)	52.1 (36.1 to 73.0)	2,400 (1,670 to 3,350)	42.5 (29.6 to 59.4)	-91.9 (-94.6 to -88.3)	-33.2 (-41.8 to -24.0)
	5-14 years	464 (273 to 686)	8.6 (5.0 to 12.7)	444 (276 to 674)	4.1 (2.6 to 6.3)	399 (248 to 608)	3.7 (2.3 to 5.6)	358 (221 to 542)	3.3 (2.0 to 4.9)	-61.9 (-74.7 to -41.5)	-20.8 (-30.5 to -10.6)
	15-49 years	565 (384 to 800)	6.2 (4.2 to 8.8)	875 (522 to 1,270)	4.1 (2.5 to 6.0)	828 (496 to 1,210)	3.8 (2.3 to 5.5)	782 (462 to 1,140)	3.5 (2.1 to 5.1)	-43.6 (-64.2 to -15.3)	-15.2 (-24.8 to -6.2)
	50-69 years	604 (409 to 864)	39.1 (26.5 to 55.9)	810 (525 to 1,180)	24.2 (15.7 to 35.4)	793 (518 to 1,180)	22.8 (14.9 to 34.0)	774 (500 to 1,130)	21.4 (13.8 to 31.3)	-45.3 (-62.6 to -19.0)	-11.6 (-19.7 to -3.5)
	70+ years	1,140 (783 to 1,560)	242.5 (166.2 to 332.1)	1,850 (1,300 to 2,500)	220.4 (155.6 to 297.9)	1,740 (1,210 to 2,400)	203.8 (142.2 to 281.5)	1,590 (1,120 to 2,190)	185.8 (130.5 to 256.1)	-23.4 (-42.2 to 1.7)	-15.7 (-22.9 to -8.0)
**Syrian Arab Republic**	All Ages	3,440 (2,710 to 4,410)	27.1 (21.3 to 34.7)	2,330 (1,820 to 2,930)	16.7 (13.0 to 21.0)	2,120 (1,640 to 2,700)	15.2 (11.7 to 19.3)	1,940 (1,490 to 2,460)	13.8 (10.7 to 17.5)	-48.9 (-63.5 to -29.8)	-17.2 (-26.4 to -7.0)
	Under 5	2,270 (1,640 to 3,180)	105.1 (75.9 to 147.3)	328 (234 to 442)	31.9 (22.8 to 43.0)	213 (142 to 303)	21.0 (13.9 to 29.8)	134 (88 to 183)	13.3 (8.8 to 18.2)	-87.3 (-92.2 to -80.2)	-58.3 (-68.7 to -45.3)
	5-14 years	300 (232 to 382)	8.0 (6.2 to 10.2)	136 (100 to 171)	4.5 (3.3 to 5.7)	114 (82 to 144)	4.0 (2.9 to 5.1)	95 (70 to 120)	3.6 (2.6 to 4.5)	-55.2 (-67.7 to -37.1)	-21.5 (-30.0 to -12.3)
	15-49 years	245 (192 to 309)	4.4 (3.4 to 5.5)	263 (190 to 357)	3.8 (2.8 to 5.2)	247 (178 to 331)	3.5 (2.6 to 4.7)	232 (167 to 312)	3.3 (2.4 to 4.4)	-25.3 (-48.8 to 13.1)	-14.5 (-26.5 to -0.9)

	50-69 years	220 (163 to 294)	22.7 (16.8 to 30.3)	574 (397 to 800)	22.8 (15.8 to 31.8)	561 (395 to 774)	21.6 (15.2 to 29.8)	543 (377 to 766)	20.3 (14.1 to 28.6)	-10.6 (-42.2 to 42.2)	-11.1 (-24.0 to 4.7)
	70+ years	411 (325 to 553)	184.5 (145.9 to 248.4)	1,030 (781 to 1,320)	187.0 (141.8 to 239.7)	988 (761 to 1,290)	173.0 (133.4 to 225.8)	935 (717 to 1,200)	158.3 (121.5 to 203.3)	-14.2 (-37.3 to 17.1)	-15.3 (-24.8 to -5.2)
**Tunisia**	All Ages	2,350 (1,790 to 3,310)	28.1 (21.5 to 39.7)	1,880 (1,370 to 2,460)	16.0 (11.7 to 21.0)	1,770 (1,270 to 2,360)	15.0 (10.8 to 20.1)	1,660 (1,210 to 2,180)	14.0 (10.2 to 18.4)	-50.2 (-68.4 to -27.4)	-12.8 (-19.6 to -4.7)
	Under 5	1,590 (1,060 to 2,580)	149.0 (99.1 to 241.8)	174 (107 to 255)	18.5 (11.3 to 27.1)	134 (81 to 200)	14.5 (8.8 to 21.8)	105 (63 to 157)	11.8 (7.1 to 17.6)	-92.1 (-95.1 to -87.8)	-36.1 (-43.8 to -27.8)
	5-14 years	81 (61 to 118)	4.0 (3.0 to 5.8)	24 (17 to 38)	1.3 (0.9 to 2.0)	22 (15 to 34)	1.2 (0.8 to 1.8)	19 (13 to 29)	1.0 (0.7 to 1.6)	-74.3 (-83.7 to -63.8)	-23.1 (-31.5 to -15.3)
	15-49 years	98 (76 to 138)	2.4 (1.9 to 3.4)	140 (99 to 191)	2.3 (1.6 to 3.2)	130 (90 to 180)	2.2 (1.5 to 3.0)	124 (87 to 170)	2.0 (1.4 to 2.8)	-14.3 (-39.0 to 19.1)	-11.7 (-20.0 to -1.0)
	50-69 years	160 (124 to 211)	17.7 (13.7 to 23.3)	317 (209 to 453)	14.2 (9.4 to 20.3)	307 (204 to 439)	13.4 (8.9 to 19.2)	301 (205 to 427)	12.9 (8.8 to 18.3)	-27.2 (-51.7 to 8.1)	-9.4 (-20.5 to 3.3)
	70+ years	419 (334 to 548)	173.5 (138.2 to 226.7)	1,220 (879 to 1,620)	186.8 (134.3 to 247.9)	1,170 (836 to 1,580)	172.9 (123.0 to 233.2)	1,110 (796 to 1,450)	159.2 (114.4 to 208.9)	-8.2 (-33.9 to 24.7)	-14.8 (-22.2 to -7.0)
**Türkiye**	All Ages	23,700 (19,300 to 30,700)	41.3 (33.6 to 53.3)	17,600 (14,400 to 20,700)	21.3 (17.4 to 25.0)	16,400 (13,100 to 20,100)	19.8 (15.8 to 24.1)	14,600 (11,400 to 17,700)	17.4 (13.7 to 21.1)	-57.8 (-69.2 to -44.8)	-18.1 (-26.1 to -9.6)
	Under 5	16,900 (12,700 to 23,500)	243.7 (182.8 to 338.5)	875 (649 to 1,180)	14.5 (10.8 to 19.6)	610 (434 to 826)	10.5 (7.5 to 14.3)	448 (313 to 611)	8.1 (5.6 to 11.0)	-96.7 (-98.2 to -94.9)	-44.5 (-53.3 to -35.0)
	5-14 years	787 (593 to 1,010)	5.8 (4.4 to 7.4)	217 (178 to 263)	1.7 (1.4 to 2.0)	185 (149 to 228)	1.4 (1.2 to 1.8)	160 (130 to 196)	1.2 (1.0 to 1.5)	-78.7 (-84.7 to -69.8)	-26.7 (-34.2 to -17.9)
	15-49 years	1,150 (950 to 1,390)	4.0 (3.3 to 4.8)	1,000 (836 to 1,180)	2.3 (1.9 to 2.7)	911 (732 to 1,120)	2.1 (1.7 to 2.5)	842 (671 to 1,040)	1.9 (1.5 to 2.4)	-51.5 (-63.7 to -36.9)	-16.3 (-26.0 to -6.5)
	50-69 years	1,340 (1,070 to 1,760)	20.6 (16.5 to 27.1)	2,940 (2,330 to 3,630)	19.2 (15.2 to 23.7)	2,810 (2,170 to 3,570)	17.9 (13.8 to 22.7)	2,640 (2,020 to 3,360)	16.4 (12.6 to 20.9)	-20.1 (-46.5 to 13.3)	-14.3 (-25.8 to -1.4)
	70+ years	3,510 (2,860 to 4,480)	234.3 (190.6 to 298.4)	12,600 (9,970 to 15,000)	262.9 (208.7 to 314.9)	11,900 (9,300 to 14,800)	241.4 (188.3 to 299.1)	10,500 (8,050 to 12,900)	206.1 (158.3 to 253.6)	-12.0 (-38.4 to 15.5)	-21.6 (-29.1 to -13.6)
**United Arab Emirates**	All Ages	240 (196 to 295)	12.9 (10.5 to 15.8)	521 (422 to 641)	5.5 (4.4 to 6.7)	394 (313 to 499)	4.1 (3.3 to 5.2)	362 (284 to 456)	3.8 (3.0 to 4.7)	-70.7 (-76.0 to -64.7)	-31.4 (-47.4 to -14.0)
	Under 5	97 (72 to 135)	42.7 (31.6 to 59.2)	38 (30 to 48)	8.0 (6.3 to 10.1)	30 (23 to 37)	6.5 (5.0 to 8.2)	21 (16 to 27)	4.8 (3.6 to 6.3)	-88.6 (-92.4 to -83.1)	-39.7 (-49.0 to -30.7)
	5-14 years	13 (10 to 18)	3.6 (2.8 to 5.0)	9 (7 to 13)	1.1 (0.8 to 1.5)	7 (5 to 10)	0.8 (0.6 to 1.1)	6 (5 to 9)	0.7 (0.5 to 1.0)	-81.4 (-86.7 to -75.3)	-37.3 (-48.0 to -27.4)
	15-49 years	38 (28 to 51)	3.2 (2.4 to 4.3)	133 (102 to 176)	1.9 (1.4 to 2.5)	106 (79 to 141)	1.5 (1.1 to 2.0)	88 (65 to 116)	1.3 (1.0 to 1.7)	-59.7 (-68.4 to -49.2)	-32.0 (-49.9 to -10.5)

	50-69 years	34 (25 to 46)	37.8 (27.7 to 50.7)	141 (103 to 194)	13.0 (9.4 to 17.8)	106 (77 to 148)	8.6 (6.2 to 12.0)	109 (78 to 149)	7.9 (5.6 to 10.8)	-79.1 (-84.5 to -71.8)	-39.1 (-56.1 to -18.3)
	70+ years	58 (46 to 73)	478.8 (375.0 to 597.1)	199 (158 to 247)	572.9 (453.3 to 711.7)	145 (109 to 187)	354.3 (266.9 to 455.1)	138 (105 to 176)	285.9 (216.5 to 365.3)	-40.3 (-53.8 to -21.8)	-50.1 (-61.1 to -39.1)
**Yemen**	All Ages	12,800 (9,780 to 18,100)	93.6 (71.7 to 132.5)	6,790 (5,070 to 8,790)	21.0 (15.7 to 27.2)	6,140 (4,530 to 8,030)	18.6 (13.8 to 24.4)	5,500 (4,120 to 7,200)	16.4 (12.2 to 21.4)	-82.5 (-88.6 to -74.8)	-22.2 (-30.0 to -14.1)
	Under 5	11,300 (8,360 to 16,600)	405.4 (301.1 to 599.3)	3,580 (2,570 to 4,860)	74.9 (53.8 to 101.7)	3,020 (2,140 to 4,140)	63.8 (45.2 to 87.4)	2,530 (1,810 to 3,390)	53.9 (38.5 to 72.2)	-86.7 (-91.5 to -80.5)	-28.0 (-38.3 to -15.6)
	5-14 years	294 (186 to 432)	6.8 (4.3 to 10.0)	328 (195 to 516)	3.7 (2.2 to 5.8)	305 (184 to 482)	3.4 (2.0 to 5.3)	282 (169 to 443)	3.1 (1.9 to 4.9)	-54.6 (-71.4 to -33.3)	-16.0 (-26.6 to -3.0)
	15-49 years	252 (170 to 367)	4.6 (3.1 to 6.7)	583 (379 to 873)	3.7 (2.4 to 5.6)	572 (374 to 857)	3.5 (2.3 to 5.3)	551 (356 to 838)	3.3 (2.1 to 5.0)	-28.8 (-52.2 to 9.7)	-11.5 (-19.3 to -2.4)
	50-69 years	420 (272 to 627)	46.5 (30.1 to 69.4)	788 (488 to 1,220)	33.3 (20.6 to 51.8)	785 (486 to 1,220)	32.0 (19.8 to 49.9)	769 (473 to 1,200)	30.3 (18.7 to 47.4)	-34.9 (-56.9 to 1.5)	-9.1 (-17.4 to 0.4)
	70+ years	533 (349 to 792)	273.0 (179.0 to 405.9)	1,510 (999 to 2,230)	264.3 (174.6 to 389.5)	1,460 (958 to 2,170)	250.0 (164.2 to 372.3)	1,370 (898 to 2,020)	230.4 (151.0 to 339.0)	-15.6 (-40.7 to 16.9)	-12.8 (-20.1 to -3.8)
**Bangladesh**	All Ages	119,000 (103,000 to 134,000)	108.9 (94.6 to 122.8)	38,500 (31,600 to 46,900)	23.8 (19.5 to 28.9)	33,300 (27,100 to 41,000)	20.4 (16.6 to 25.1)	32,600 (26,400 to 40,700)	19.8 (16.0 to 24.7)	-81.8 (-85.8 to -76.3)	-16.5 (-23.4 to -8.5)
	Under 5	99,500 (84,200 to 115,000)	525.5 (445.0 to 605.2)	14,400 (10,800 to 18,500)	94.9 (71.3 to 122.3)	10,600 (7,810 to 13,900)	72.3 (53.0 to 94.1)	9,510 (6,880 to 12,500)	66.2 (47.9 to 86.8)	-87.4 (-91.2 to -82.7)	-30.2 (-40.2 to -19.1)
	5-14 years	4,640 (3,230 to 6,030)	15.5 (10.8 to 20.1)	1,980 (1,500 to 2,490)	6.2 (4.7 to 7.8)	1,720 (1,310 to 2,190)	5.4 (4.1 to 6.9)	1,620 (1,230 to 2,080)	5.2 (3.9 to 6.6)	-66.7 (-76.5 to -49.5)	-16.2 (-24.2 to -6.9)
	15-49 years	3,070 (2,570 to 3,600)	6.2 (5.1 to 7.2)	2,130 (1,650 to 2,750)	2.5 (1.9 to 3.2)	1,960 (1,500 to 2,540)	2.2 (1.7 to 2.9)	1,960 (1,500 to 2,530)	2.2 (1.7 to 2.9)	-63.7 (-73.6 to -50.7)	-10.0 (-17.7 to -1.6)
	50-69 years	4,470 (3,670 to 5,460)	56.2 (46.1 to 68.6)	5,410 (3,850 to 7,360)	24.5 (17.4 to 33.4)	5,110 (3,610 to 7,020)	22.4 (15.8 to 30.8)	5,250 (3,650 to 7,290)	22.3 (15.5 to 31.0)	-60.3 (-73.3 to -44.1)	-9.0 (-19.1 to 1.6)
	70+ years	7,200 (5,780 to 9,010)	316.4 (254.2 to 396.0)	14,700 (11,200 to 19,300)	215.0 (164.9 to 282.8)	13,800 (10,400 to 18,100)	194.2 (146.0 to 254.6)	14,300 (10,800 to 18,800)	194.2 (146.8 to 254.9)	-38.6 (-56.4 to -14.5)	-9.7 (-17.9 to -0.5)
**Bhutan**	All Ages	442 (279 to 593)	70.2 (44.3 to 94.2)	154 (109 to 198)	20.2 (14.4 to 26.0)	152 (107 to 198)	20.1 (14.1 to 26.1)	137 (97 to 179)	18.1 (12.8 to 23.6)	-74.2 (-82.9 to -57.4)	-10.5 (-18.3 to -2.0)
	Under 5	388 (233 to 528)	406.4 (244.1 to 553.1)	48 (32 to 67)	74.6 (49.4 to 105.2)	44 (29 to 62)	70.6 (46.2 to 99.3)	35 (22 to 48)	56.8 (36.2 to 79.3)	-86.0 (-92.1 to -74.1)	-23.8 (-33.5 to -11.8)
	5-14 years	8 (2 to 16)	4.9 (1.3 to 9.4)	3 (2 to 5)	2.5 (1.4 to 3.6)	3 (2 to 5)	2.4 (1.4 to 3.5)	3 (2 to 4)	2.2 (1.3 to 3.2)	-55.3 (-76.2 to 79.7)	-12.0 (-20.7 to -2.0)
	15-49 years	10 (6 to 15)	3.4 (2.0 to 4.9)	8 (4 to 12)	1.7 (1.0 to 2.7)	8 (4 to 12)	1.7 (1.0 to 2.7)	7 (4 to 11)	1.6 (0.9 to 2.5)	-51.9 (-69.5 to -26.0)	-6.5 (-15.2 to 3.6)

	50-69 years	16 (10 to 23)	34.0 (20.8 to 49.2)	20 (11 to 31)	21.3 (11.9 to 32.1)	21 (12 to 31)	21.2 (11.9 to 32.2)	20 (11 to 30)	19.6 (11.1 to 29.7)	-42.3 (-63.4 to -14.2)	-7.7 (-17.0 to 2.0)
	70+ years	20 (13 to 28)	220.2 (147.0 to 312.6)	75 (47 to 105)	245.7 (155.1 to 344.3)	77 (48 to 107)	246.1 (154.7 to 344.1)	73 (46 to 101)	225.8 (142.8 to 314.7)	2.6 (-26.0 to 41.1)	-8.1 (-15.1 to -0.4)
**India**	All Ages	575,000 (489,000 to 661,000)	67.4 (57.4 to 77.5)	484,000 (432,000 to 540,000)	34.9 (31.1 to 38.9)	422,000 (374,000 to 474,000)	30.1 (26.7 to 33.8)	421,000 (364,000 to 482,000)	29.7 (25.7 to 34.1)	-55.9 (-63.7 to -46.3)	-14.7 (-23.3 to -5.0)
	Under 5	417,000 (337,000 to 500,000)	358.3 (289.5 to 429.7)	153,000 (123,000 to 188,000)	132.5 (107.0 to 162.6)	113,000 (88,400 to 141,000)	99.3 (78.0 to 124.6)	105,000 (81,000 to 134,000)	94.1 (72.7 to 120.0)	-73.7 (-80.7 to -64.6)	-29.0 (-41.3 to -12.8)
	5-14 years	21,000 (16,300 to 25,500)	10.0 (7.7 to 12.1)	8,090 (6,540 to 9,960)	3.1 (2.5 to 3.8)	6,440 (5,230 to 8,040)	2.5 (2.0 to 3.1)	6,110 (4,860 to 7,480)	2.4 (1.9 to 2.9)	-76.1 (-81.1 to -69.8)	-23.2 (-33.0 to -12.0)
	15-49 years	22,300 (19,600 to 27,400)	5.3 (4.7 to 6.5)	25,900 (23,200 to 30,900)	3.4 (3.1 to 4.1)	24,900 (22,000 to 29,800)	3.2 (2.9 to 3.9)	25,700 (22,200 to 31,400)	3.3 (2.8 to 4.0)	-37.7 (-46.2 to -26.9)	-3.1 (-14.0 to 9.9)
	50-69 years	51,200 (43,900 to 59,300)	58.1 (49.8 to 67.3)	97,200 (85,400 to 109,000)	49.0 (43.1 to 55.1)	90,500 (78,900 to 104,000)	44.3 (38.6 to 50.9)	90,200 (76,800 to 105,000)	43.2 (36.8 to 50.3)	-25.7 (-36.9 to -11.6)	-12.0 (-21.6 to -0.1)
	70+ years	63,900 (51,800 to 77,800)	354.9 (287.6 to 431.8)	200,000 (174,000 to 226,000)	356.5 (310.4 to 402.0)	188,000 (163,000 to 214,000)	325.9 (282.1 to 371.2)	194,000 (164,000 to 228,000)	325.2 (274.5 to 382.9)	-8.4 (-24.1 to 11.3)	-8.8 (-17.6 to 1.1)
**Nepal**	All Ages	29,100 (24,600 to 33,800)	149.6 (126.1 to 173.5)	8,090 (6,140 to 10,300)	26.5 (20.1 to 33.7)	7,470 (5,720 to 9,410)	24.2 (18.6 to 30.5)	6,910 (5,200 to 8,870)	22.2 (16.7 to 28.5)	-85.2 (-89.1 to -80.2)	-16.2 (-23.3 to -9.0)
	Under 5	25,800 (21,100 to 30,700)	784.6 (641.9 to 931.8)	3,170 (2,240 to 4,280)	102.6 (72.6 to 138.3)	2,710 (1,960 to 3,700)	87.3 (63.1 to 119.3)	2,340 (1,670 to 3,220)	75.5 (53.6 to 103.6)	-90.4 (-93.5 to -86.1)	-26.4 (-37.0 to -14.2)
	5-14 years	518 (334 to 769)	10.1 (6.5 to 15.0)	181 (122 to 252)	2.9 (2.0 to 4.1)	164 (112 to 231)	2.7 (1.8 to 3.8)	150 (104 to 210)	2.5 (1.7 to 3.4)	-75.7 (-86.4 to -58.0)	-15.9 (-25.5 to -5.6)
	15-49 years	535 (365 to 762)	6.0 (4.1 to 8.6)	437 (293 to 613)	2.7 (1.8 to 3.8)	417 (275 to 590)	2.5 (1.7 to 3.6)	399 (260 to 567)	2.4 (1.6 to 3.4)	-60.1 (-78.6 to -31.7)	-10.9 (-19.4 to -1.8)
	50-69 years	964 (664 to 1,380)	54.2 (37.3 to 77.3)	1,240 (792 to 1,770)	31.5 (20.2 to 45.2)	1,200 (769 to 1,740)	29.8 (19.2 to 43.3)	1,160 (741 to 1,680)	28.3 (18.1 to 41.1)	-47.8 (-72.8 to -5.2)	-10.0 (-18.5 to -0.4)
	70+ years	1,280 (913 to 1,780)	349.8 (250.3 to 488.6)	3,070 (2,030 to 4,370)	278.3 (183.9 to 396.2)	2,980 (1,970 to 4,260)	261.8 (173.1 to 373.3)	2,860 (1,880 to 4,100)	245.6 (161.8 to 351.9)	-29.8 (-61.5 to 20.8)	-11.7 (-20.0 to -2.5)
**Pakistan**	All Ages	78,500 (66,000 to 93,700)	70.7 (59.4 to 84.3)	78,100 (62,500 to 95,000)	34.6 (27.7 to 42.1)	58,600 (45,500 to 72,700)	25.4 (19.7 to 31.5)	55,400 (42,800 to 68,900)	23.5 (18.2 to 29.3)	-66.7 (-75.0 to -56.4)	-32.1 (-41.2 to -21.6)
	Under 5	68,000 (55,600 to 83,100)	368.5 (301.3 to 449.9)	58,800 (44,600 to 74,500)	199.9 (151.7 to 253.5)	40,700 (30,300 to 53,500)	137.5 (102.3 to 180.7)	37,600 (27,800 to 50,000)	126.4 (93.5 to 168.2)	-65.7 (-75.8 to -52.3)	-36.8 (-46.6 to -25.2)
	5-14 years	1,660 (1,080 to 2,330)	5.4 (3.5 to 7.6)	2,710 (1,870 to 3,680)	5.1 (3.5 to 6.9)	2,440 (1,690 to 3,250)	4.5 (3.1 to 5.9)	2,340 (1,600 to 3,180)	4.2 (2.9 to 5.7)	-22.3 (-43.1 to 8.7)	-16.9 (-28.0 to -5.2)
	15-49 years	1,350 (1,010 to 1,770)	2.7 (2.0 to 3.6)	2,890 (1,980 to 4,040)	2.5 (1.7 to 3.5)	2,690 (1,770 to 3,810)	2.3 (1.5 to 3.2)	2,710 (1,810 to 3,850)	2.2 (1.5 to 3.2)	-18.4 (-39.9 to 11.7)	-10.7 (-33.9 to 20.0)

	50-69 years	2,310<br>(1,690 to 3,200)	24.0<br>(17.5 to 33.3)	4,470<br>(2,910 to 6,600)	20.8<br>(13.5 to 30.7)	4,270<br>(2,720 to 6,420)	19.2<br>(12.2 to 28.8)	4,340<br>(2,720 to 6,450)	18.8<br>(11.8 to 28.0)	-21.7<br>(-44.1 to 9.6)	-9.6<br>(-34.9 to 26.1)
	70+ years	5,200<br>(3,690 to 7,280)	184.1<br>(130.6 to 257.6)	9,230<br>(6,370 to 12,800)	189.0<br>(130.4 to 261.5)	8,560<br>(5,760 to 11,900)	171.2<br>(115.2 to 238.4)	8,410<br>(5,740 to 11,800)	166.2<br>(113.4 to 233.3)	-9.7<br>(-29.9 to 21.2)	-12.1<br>(-30.6 to 11.6)
**China**	All Ages	475,000<br>(415,000 to 531,000)	40.4<br>(35.3 to 45.1)	199,000<br>(170,000 to 235,000)	14.0<br>(12.0 to 16.6)	188,000<br>(156,000 to 225,000)	13.3<br>(11.0 to 15.8)	207,000<br>(172,000 to 248,000)	14.5<br>(12.1 to 17.4)	-64.0<br>(-70.9 to -55.0)	3.6<br>(-12.6 to 22.6)
	Under 5	314,000<br>(267,000 to 368,000)	280.7<br>(238.9 to 328.9)	13,000<br>(10,600 to 15,500)	14.7<br>(12.1 to 17.6)	11,000<br>(8,740 to 13,400)	13.0<br>(10.4 to 15.9)	10,100<br>(7,790 to 12,600)	13.0<br>(10.0 to 16.2)	-95.4<br>(-96.6 to -93.8)	-11.5<br>(-21.1 to -2.4)
	5-14 years	12,700<br>(10,100 to 14,500)	6.1<br>(4.9 to 7.0)	1,590<br>(1,380 to 1,880)	0.9<br>(0.8 to 1.1)	1,470<br>(1,270 to 1,750)	0.8<br>(0.7 to 1.0)	1,550<br>(1,310 to 1,880)	0.9<br>(0.7 to 1.0)	-86.1<br>(-88.7 to -81.4)	-9.0<br>(-15.2 to -2.2)
	15-49 years	20,300<br>(16,700 to 23,300)	3.0<br>(2.5 to 3.5)	7,000<br>(5,810 to 8,460)	1.0<br>(0.8 to 1.2)	6,370<br>(5,160 to 7,760)	0.9<br>(0.8 to 1.2)	6,610<br>(5,380 to 8,050)	1.0<br>(0.8 to 1.2)	-67.3<br>(-74.5 to -56.7)	-2.4<br>(-23.8 to 25.5)
	50-69 years	33,400<br>(27,300 to 38,700)	21.7<br>(17.8 to 25.2)	22,400<br>(18,200 to 27,600)	6.2<br>(5.0 to 7.6)	21,500<br>(17,000 to 26,700)	5.8<br>(4.6 to 7.2)	23,000<br>(18,300 to 28,500)	6.0<br>(4.8 to 7.5)	-72.2<br>(-78.9 to -62.3)	-2.3<br>(-25.1 to 27.8)
	70+ years	94,600<br>(76,900 to 106,000)	252.1<br>(204.8 to 282.6)	155,000<br>(130,000 to 186,000)	142.4<br>(120.0 to 171.4)	148,000<br>(121,000 to 178,000)	131.0<br>(107.4 to 157.8)	166,000<br>(136,000 to 201,000)	138.8<br>(113.6 to 168.3)	-44.9<br>(-55.7 to -28.7)	-2.5<br>(-17.0 to 14.7)
**Democratic People's Republic of Korea**	All Ages	6,790<br>(5,360 to 8,490)	33.0<br>(26.0 to 41.2)	6,510<br>(5,080 to 8,290)	24.8<br>(19.3 to 31.6)	6,000<br>(4,620 to 7,750)	22.8<br>(17.5 to 29.4)	5,810<br>(4,400 to 7,560)	22.0<br>(16.7 to 28.6)	-33.2<br>(-50.7 to -8.8)	-11.1<br>(-16.8 to -4.9)
	Under 5	3,510<br>(2,640 to 4,580)	150.4<br>(113.0 to 195.8)	513<br>(318 to 725)	32.3<br>(20.0 to 45.6)	400<br>(241 to 574)	25.8<br>(15.5 to 37.0)	336<br>(196 to 485)	22.2<br>(13.0 to 32.1)	-85.3<br>(-91.7 to -77.4)	-31.3<br>(-39.3 to -23.4)
	5-14 years	165<br>(113 to 225)	4.6<br>(3.1 to 6.2)	56<br>(37 to 79)	1.7<br>(1.1 to 2.3)	48<br>(31 to 69)	1.5<br>(0.9 to 2.1)	43<br>(28 to 62)	1.3<br>(0.9 to 1.9)	-70.9<br>(-82.3 to -52.7)	-20.3<br>(-28.5 to -11.5)
	15-49 years	440<br>(308 to 621)	4.1<br>(2.9 to 5.8)	464<br>(332 to 650)	3.3<br>(2.4 to 4.7)	426<br>(303 to 603)	3.1<br>(2.2 to 4.3)	405<br>(290 to 573)	2.9<br>(2.1 to 4.1)	-28.4<br>(-54.4 to 11.8)	-11.6<br>(-19.6 to -3.4)
	50-69 years	740<br>(532 to 991)	22.8<br>(16.4 to 30.5)	1,100<br>(813 to 1,450)	19.6<br>(14.5 to 25.9)	1,070<br>(783 to 1,420)	18.3<br>(13.4 to 24.3)	1,060<br>(776 to 1,420)	17.6<br>(12.9 to 23.5)	-22.9<br>(-47.1 to 14.4)	-10.3<br>(-17.3 to -2.3)
	70+ years	1,930<br>(1,420 to 2,460)	294.4<br>(216.1 to 374.9)	4,380<br>(3,290 to 5,660)	251.1<br>(189.0 to 324.9)	4,060<br>(3,040 to 5,310)	234.0<br>(175.2 to 306.1)	3,960<br>(2,910 to 5,170)	226.5<br>(166.5 to 295.7)	-23.0<br>(-41.1 to 2.9)	-9.8<br>(-16.6 to -2.6)
**Taiwan (Province of China)**	All Ages	4,300<br>(4,040 to 4,510)	21.1<br>(19.8 to 22.1)	13,800<br>(12,100 to 14,700)	58.3<br>(51.3 to 62.3)	11,800<br>(10,000 to 13,100)	50.1<br>(42.5 to 55.2)	11,700<br>(9,980 to 13,000)	49.6<br>(42.2 to 54.8)	135.3<br>(105.3 to 161.2)	-14.9<br>(-21.5 to -9.3)
	Under 5	421<br>(396 to 450)	26.2<br>(24.6 to 28.0)	88<br>(79 to 99)	9.0<br>(8.0 to 10.1)	62<br>(53 to 72)	6.6<br>(5.7 to 7.6)	53<br>(45 to 62)	6.0<br>(5.0 to 7.0)	-77.2<br>(-81.2 to -73.2)	-33.5<br>(-42.6 to -24.3)
	5-14 years	83<br>(76 to 90)	2.1<br>(2.0 to 2.3)	13<br>(12 to 15)	0.7<br>(0.6 to 0.7)	10<br>(9 to 12)	0.5<br>(0.4 to 0.6)	9<br>(8 to 11)	0.5<br>(0.4 to 0.5)	-78.7<br>(-82.5 to -75.1)	-31.4<br>(-40.5 to -25.0)
	15-49 years	322<br>(304 to 340)	2.9<br>(2.7 to 3.0)	288<br>(265 to 312)	2.5<br>(2.3 to 2.7)	234<br>(213 to 259)	2.0<br>(1.8 to 2.2)	218<br>(194 to 242)	1.9<br>(1.7 to 2.1)	-32.9<br>(-41.1 to -24.7)	-22.1<br>(-27.4 to -16.6)

	50-69 years	918 (843 to 997)	31.7 (29.1 to 34.4)	1,660 (1,520 to 1,810)	24.9 (22.9 to 27.1)	1,480 (1,340 to 1,630)	21.8 (19.8 to 24.1)	1,410 (1,250 to 1,580)	20.7 (18.4 to 23.3)	-34.6 (-43.2 to -25.4)	-16.7 (-23.6 to -10.2)
	70+ years	2,550 (2,340 to 2,730)	350.1 (320.5 to 374.8)	11,700 (10,200 to 12,600)	520.6 (452.9 to 560.6)	10,100 (8,360 to 11,200)	423.7 (352.1 to 470.7)	10,000 (8,340 to 11,200)	397.2 (330.2 to 443.4)	13.5 (-0.9 to 26.6)	-23.7 (-29.7 to -18.5)
**American Samoa**	All Ages	13 (11 to 14)	25.8 (22.8 to 29.2)	13 (11 to 15)	25.3 (21.4 to 30.1)	14 (12 to 16)	27.1 (23.3 to 31.5)	13 (11 to 16)	25.9 (21.7 to 31.3)	0.5 (-17.4 to 23.8)	2.5 (-2.6 to 7.9)
	Under 5	6 (5 to 7)	72.3 (59.2 to 86.0)	2 (1 to 2)	35.7 (26.6 to 46.2)	2 (1 to 2)	38.1 (27.9 to 49.7)	1 (1 to 2)	34.0 (24.0 to 45.3)	-53.0 (-67.0 to -34.9)	-5.0 (-16.3 to 7.4)
	5-14 years	0 (0 to 0)	3.3 (2.6 to 4.1)	0 (0 to 0)	2.2 (1.7 to 2.8)	0 (0 to 0)	2.2 (1.3 to 2.9)	0 (0 to 0)	2.1 (1.5 to 2.7)	-36.1 (-54.6 to -12.9)	-4.3 (-13.3 to 5.3)
	15-49 years	2 (2 to 3)	8.6 (7.0 to 10.7)	2 (2 to 3)	8.4 (6.6 to 10.6)	2 (2 to 3)	9.1 (7.2 to 11.2)	2 (2 to 3)	8.4 (6.4 to 10.7)	-2.6 (-29.7 to 33.4)	-0.3 (-7.6 to 7.5)
	50-69 years	2 (2 to 2)	42.6 (35.5 to 50.4)	3 (2 to 4)	33.4 (27.6 to 40.3)	3 (3 to 4)	35.6 (28.8 to 43.4)	3 (3 to 4)	33.4 (27.0 to 41.6)	-21.5 (-40.5 to 1.5)	0.0 (-6.5 to 6.9)
	70+ years	3 (2 to 3)	309.4 (258.2 to 363.5)	6 (5 to 7)	287.4 (239.1 to 343.7)	6 (5 to 8)	300.6 (250.6 to 362.6)	6 (5 to 8)	283.3 (233.6 to 340.1)	-8.4 (-26.3 to 13.1)	-1.5 (-6.7 to 4.2)
**Cook Islands**	All Ages	10 (9 to 12)	53.6 (46.4 to 61.2)	9 (7 to 10)	48.8 (40.7 to 57.7)	9 (8 to 11)	50.7 (42.3 to 60.3)	9 (8 to 11)	51.6 (42.8 to 61.8)	-3.7 (-20.5 to 16.5)	5.8 (0.6 to 10.8)
	Under 5	3 (2 to 4)	126.9 (99.9 to 158.6)	0 (0 to 0)	11.2 (7.5 to 15.4)	0 (0 to 0)	26.9 (18.8 to 32.5)	0 (0 to 0)	26.9 (18.6 to 32.7)	-78.8 (-86.5 to -70.9)	139.4 (88.1 to 216.4)
	5-14 years	0 (0 to 0)	6.5 (5.0 to 8.2)	0 (0 to 0)	1.3 (1.0 to 1.7)	0 (0 to 0)	2.0 (1.5 to 2.4)	0 (0 to 0)	2.0 (1.5 to 2.5)	-69.1 (-78.5 to -57.4)	50.4 (23.3 to 88.0)
	15-49 years	1 (1 to 2)	14.7 (11.5 to 18.8)	1 (1 to 1)	8.6 (6.7 to 11.2)	1 (1 to 1)	8.5 (6.5 to 11.1)	1 (1 to 1)	8.3 (6.3 to 11.0)	-43.5 (-61.1 to -16.4)	-3.5 (-10.8 to 5.5)
	50-69 years	2 (2 to 2)	82.0 (67.6 to 98.2)	2 (2 to 2)	43.8 (35.6 to 53.0)	2 (1 to 2)	42.5 (34.2 to 52.1)	2 (1 to 2)	41.7 (33.2 to 51.6)	-49.1 (-62.3 to -32.5)	-4.8 (-12.3 to 2.8)
	70+ years	4 (3 to 4)	642.4 (540.8 to 753.4)	6 (5 to 7)	423.2 (346.4 to 502.0)	6 (5 to 7)	411.2 (334.8 to 491.9)	6 (5 to 8)	405.8 (327.4 to 491.9)	-36.8 (-49.6 to -21.3)	-4.1 (-9.3 to 1.1)
**Fiji**	All Ages	206 (175 to 242)	27.2 (23.1 to 31.9)	225 (180 to 280)	24.6 (19.7 to 30.5)	200 (157 to 251)	21.7 (17.0 to 27.3)	181 (139 to 229)	19.5 (15.0 to 24.8)	-28.3 (-45.7 to -4.8)	-20.6 (-27.4 to -13.9)
	Under 5	75 (57 to 98)	79.3 (60.6 to 103.5)	55 (40 to 73)	58.5 (42.9 to 78.5)	44 (31 to 59)	47.2 (34.0 to 64.3)	36 (26 to 51)	40.0 (28.1 to 56.0)	-49.5 (-66.5 to -23.0)	-31.5 (-39.3 to -23.2)
	5-14 years	11 (8 to 14)	5.8 (4.5 to 7.3)	9 (7 to 12)	5.2 (4.0 to 6.6)	8 (6 to 11)	4.6 (3.6 to 5.9)	7 (5 to 9)	3.9 (2.9 to 5.0)	-33.6 (-54.6 to -4.8)	-25.2 (-36.9 to -15.5)
	15-49 years	46 (39 to 55)	11.8 (9.8 to 13.9)	37 (29 to 47)	7.9 (6.2 to 10.1)	33 (25 to 43)	7.1 (5.4 to 9.2)	31 (23 to 40)	6.6 (4.9 to 8.5)	-44.1 (-59.8 to -23.0)	-17.1 (-25.9 to -7.8)

	50-69 years	35<br>(28 to 43)	50.8<br>(41.3 to 62.9)	52<br>(38 to 67)	34.6<br>(25.7 to 45.1)	48<br>(35 to 64)	31.7<br>(23.2 to 42.3)	45<br>(32 to 61)	29.7<br>(21.4 to 40.1)	-41.6<br>(-59.7 to -17.4)	-14.3<br>(-24.4 to -3.8)
	70+ years	40<br>(33 to 48)	293.4<br>(244.0 to 356.6)	73<br>(58 to 93)	239.3<br>(190.4 to 303.0)	67<br>(53 to 86)	211.5<br>(167.3 to 270.8)	61<br>(48 to 80)	190.6<br>(148.0 to 247.5)	-35.0<br>(-50.8 to -15.6)	-20.3<br>(-27.9 to -13.1)
**Guam**	All Ages	21<br>(19 to 24)	15.7<br>(13.9 to 17.4)	31<br>(27 to 35)	19.1<br>(16.5 to 21.8)	23<br>(20 to 26)	14.2<br>(12.2 to 16.2)	22<br>(18 to 25)	13.6<br>(11.4 to 15.6)	-13.5<br>(-27.7 to 2.6)	-29.0<br>(-33.8 to -24.2)
	Under 5	7<br>(6 to 8)	40.3<br>(34.1 to 47.4)	5<br>(4 to 7)	39.5<br>(32.3 to 48.6)	4<br>(3 to 5)	31.6<br>(24.8 to 39.7)	4<br>(3 to 5)	27.7<br>(20.7 to 36.4)	-31.2<br>(-49.6 to -7.1)	-29.8<br>(-40.9 to -19.9)
	5-14 years	1<br>(1 to 1)	2.9<br>(2.4 to 3.6)	1<br>(0 to 1)	2.3<br>(1.8 to 2.7)	0<br>(0 to 0)	0.8<br>(0.6 to 1.1)	0<br>(0 to 0)	0.7<br>(0.5 to 1.0)	-75.0<br>(-84.0 to -62.0)	-67.5<br>(-76.4 to -56.8)
	15-49 years	4<br>(3 to 4)	4.7<br>(3.9 to 5.4)	4<br>(4 to 5)	5.5<br>(4.8 to 6.3)	4<br>(3 to 4)	4.7<br>(4.0 to 5.4)	3<br>(3 to 4)	4.0<br>(3.5 to 4.7)	-13.2<br>(-29.8 to 7.7)	-26.3<br>(-32.0 to -20.1)
	50-69 years	4<br>(4 to 5)	28.7<br>(23.7 to 34.5)	7<br>(6 to 8)	19.9<br>(16.7 to 23.9)	6<br>(5 to 7)	17.1<br>(14.5 to 20.3)	6<br>(5 to 7)	16.5<br>(13.6 to 19.9)	-42.4<br>(-56.2 to -26.0)	-16.9<br>(-24.8 to -8.5)
	70+ years	6<br>(5 to 7)	225.1<br>(185.2 to 266.7)	14<br>(11 to 16)	120.6<br>(94.8 to 144.6)	9<br>(7 to 11)	74.4<br>(55.8 to 90.7)	9<br>(7 to 11)	73.0<br>(53.8 to 89.4)	-67.6<br>(-75.7 to -58.7)	-39.5<br>(-45.7 to -33.7)
**Kiribati**	All Ages	38<br>(31 to 46)	51.4<br>(42.0 to 62.3)	29<br>(23 to 36)	24.3<br>(19.3 to 30.7)	29<br>(23 to 36)	24.0<br>(19.4 to 30.4)	29<br>(23 to 36)	23.7<br>(19.0 to 30.0)	-53.9<br>(-63.9 to -42.1)	-2.3<br>(-10.8 to 7.0)
	Under 5	25<br>(19 to 33)	216.0<br>(159.1 to 279.2)	9<br>(6 to 12)	63.6<br>(44.1 to 87.2)	9<br>(6 to 12)	62.2<br>(42.1 to 87.2)	9<br>(6 to 12)	59.6<br>(40.6 to 82.6)	-72.4<br>(-81.6 to -59.8)	-6.3<br>(-24.9 to 17.5)
	5-14 years	1<br>(1 to 2)	7.0<br>(5.2 to 9.1)	1<br>(1 to 2)	4.6<br>(3.3 to 6.1)	1<br>(1 to 2)	4.6<br>(3.2 to 6.1)	1<br>(1 to 2)	4.5<br>(3.2 to 6.0)	-35.2<br>(-55.2 to -7.4)	-1.3<br>(-20.9 to 23.2)
	15-49 years	4<br>(3 to 5)	11.4<br>(9.2 to 14.2)	6<br>(5 to 8)	10.4<br>(7.8 to 13.7)	6<br>(5 to 8)	10.4<br>(7.7 to 13.5)	6<br>(5 to 9)	10.3<br>(7.6 to 13.8)	-9.7<br>(-35.2 to 26.5)	-0.9<br>(-8.3 to 6.9)
	50-69 years	4<br>(3 to 4)	52.0<br>(41.2 to 64.4)	6<br>(4 to 8)	40.8<br>(31.1 to 54.2)	6<br>(4 to 8)	41.1<br>(31.1 to 55.2)	6<br>(5 to 8)	41.3<br>(31.1 to 55.3)	-20.5<br>(-41.2 to 9.1)	1.2<br>(-6.0 to 9.0)
	70+ years	4<br>(3 to 5)	268.4<br>(210.6 to 344.7)	6<br>(5 to 8)	264.9<br>(208.6 to 338.0)	6<br>(5 to 8)	264.6<br>(206.4 to 337.2)	7<br>(5 to 8)	262.1<br>(204.3 to 333.9)	-2.3<br>(-24.9 to 27.0)	-1.1<br>(-6.9 to 5.5)
**Marshall Islands**	All Ages	21<br>(18 to 24)	45.5<br>(38.7 to 52.3)	19<br>(15 to 24)	33.7<br>(26.1 to 43.3)	19<br>(15 to 25)	34.0<br>(26.2 to 43.9)	19<br>(14 to 25)	33.6<br>(25.7 to 43.6)	-26.1<br>(-44.4 to -3.6)	-0.3<br>(-7.0 to 7.1)
	Under 5	10<br>(8 to 13)	141.0<br>(108.8 to 173.3)	5<br>(3 to 7)	81.5<br>(55.8 to 111.4)	5<br>(3 to 7)	82.4<br>(56.4 to 112.8)	4<br>(3 to 6)	78.0<br>(51.8 to 105.6)	-44.7<br>(-63.2 to -20.0)	-4.3<br>(-22.9 to 17.6)
	5-14 years	1<br>(1 to 1)	5.3<br>(3.9 to 6.7)	1<br>(0 to 1)	4.7<br>(3.5 to 6.1)	1<br>(0 to 1)	4.8<br>(3.5 to 6.2)	1<br>(0 to 1)	4.6<br>(3.4 to 6.1)	-12.4<br>(-38.6 to 24.7)	-1.6<br>(-14.8 to 16.4)
	15-49 years	4<br>(3 to 4)	18.4<br>(15.7 to 21.6)	5<br>(4 to 7)	17.4<br>(13.0 to 23.1)	5<br>(4 to 7)	17.2<br>(12.9 to 23.0)	5<br>(4 to 7)	17.0<br>(12.8 to 22.8)	-7.6<br>(-32.0 to 22.4)	-2.1<br>(-8.0 to 3.9)

	50-69 years	2<br>(2 to 3)	91.5<br>(74.6 to 111.3)	5<br>(3 to 6)	67.4<br>(49.6 to 90.3)	5<br>(3 to 6)	66.8<br>(49.1 to 89.9)	5<br>(3 to 6)	66.1<br>(48.5 to 90.5)	-27.8<br>(-48.8 to -1.5)	-2.0<br>(-8.4 to 5.2)
	70+ years	3<br>(3 to 4)	445.5<br>(364.3 to 543.1)	4<br>(3 to 5)	362.7<br>(276.9 to 452.5)	4<br>(3 to 5)	358.1<br>(271.5 to 447.5)	4<br>(3 to 5)	351.1<br>(266.7 to 443.2)	-21.2<br>(-39.9 to 4.1)	-3.2<br>(-9.0 to 3.1)
**Micronesia (Federated States of)**	All Ages	64<br>(53 to 77)	62.1<br>(51.0 to 73.9)	35<br>(27 to 43)	33.6<br>(26.6 to 42.2)	34<br>(27 to 44)	33.3<br>(26.5 to 42.8)	34<br>(27 to 43)	33.3<br>(26.2 to 42.2)	-46.4<br>(-58.3 to -28.9)	-0.8<br>(-6.5 to 5.7)
	Under 5	33<br>(25 to 41)	211.2<br>(162.6 to 263.1)	6<br>(4 to 7)	57.0<br>(39.9 to 75.6)	5<br>(3 to 7)	52.2<br>(35.5 to 69.0)	5<br>(3 to 6)	50.0<br>(34.3 to 66.6)	-76.3<br>(-84.3 to -66.2)	-12.2<br>(-29.7 to 10.9)
	5-14 years	3<br>(2 to 3)	8.5<br>(6.4 to 10.9)	1<br>(1 to 1)	3.9<br>(2.9 to 5.1)	1<br>(1 to 1)	3.7<br>(2.8 to 4.8)	1<br>(1 to 1)	3.6<br>(2.7 to 4.8)	-57.9<br>(-70.5 to -41.8)	-7.7<br>(-20.7 to 8.4)
	15-49 years	9<br>(7 to 12)	19.6<br>(14.4 to 25.2)	8<br>(6 to 11)	15.0<br>(10.9 to 20.4)	8<br>(6 to 11)	14.9<br>(10.7 to 20.2)	8<br>(6 to 11)	14.8<br>(10.7 to 20.3)	-24.5<br>(-47.1 to 10.2)	-1.3<br>(-9.1 to 6.6)
	50-69 years	7<br>(6 to 10)	93.0<br>(71.0 to 121.9)	9<br>(7 to 13)	61.9<br>(45.4 to 83.6)	10<br>(7 to 13)	62.5<br>(46.1 to 85.5)	10<br>(7 to 13)	62.4<br>(46.0 to 86.2)	-32.9<br>(-53.0 to -0.4)	0.9<br>(-6.8 to 9.6)
	70+ years	12<br>(10 to 15)	512.8<br>(415.7 to 631.6)	11<br>(9 to 13)	454.6<br>(361.8 to 558.0)	11<br>(8 to 13)	453.1<br>(360.1 to 559.5)	11<br>(9 to 14)	432.9<br>(345.6 to 536.3)	-15.6<br>(-34.8 to 8.9)	-4.8<br>(-10.2 to 0.9)
**Nauru**	All Ages	6<br>(5 to 7)	59.3<br>(48.9 to 69.7)	5<br>(4 to 6)	45.9<br>(35.8 to 58.8)	5<br>(4 to 6)	44.7<br>(34.5 to 57.5)	5<br>(4 to 6)	43.6<br>(33.4 to 56.7)	-26.4<br>(-44.7 to -2.5)	-5.0<br>(-9.7 to 0.3)
	Under 5	3<br>(2 to 4)	190.1<br>(147.1 to 237.5)	2<br>(1 to 3)	132.8<br>(94.9 to 178.3)	2<br>(1 to 2)	125.8<br>(88.4 to 171.5)	2<br>(1 to 2)	119.8<br>(83.2 to 165.8)	-37.0<br>(-56.6 to -6.0)	-9.8<br>(-18.2 to -0.7)
	5-14 years	0<br>(0 to 0)	8.0<br>(6.1 to 10.1)	0<br>(0 to 0)	6.5<br>(4.8 to 8.6)	0<br>(0 to 0)	6.3<br>(4.6 to 8.3)	0<br>(0 to 0)	6.1<br>(4.5 to 8.1)	-23.7<br>(-46.6 to 6.2)	-5.9<br>(-13.6 to 2.5)
	15-49 years	1<br>(1 to 1)	23.0<br>(17.9 to 29.1)	1<br>(1 to 2)	19.9<br>(15.0 to 26.9)	1<br>(1 to 2)	19.7<br>(14.6 to 26.7)	1<br>(1 to 2)	19.5<br>(14.4 to 26.6)	-15.4<br>(-38.3 to 17.1)	-2.4<br>(-8.5 to 4.0)
	50-69 years	1<br>(1 to 1)	101.4<br>(76.1 to 130.9)	1<br>(1 to 1)	82.7<br>(61.0 to 113.2)	1<br>(1 to 1)	81.7<br>(59.9 to 111.5)	1<br>(1 to 1)	80.8<br>(59.2 to 109.9)	-20.3<br>(-45.3 to 17.7)	-2.3<br>(-9.9 to 5.3)
	70+ years	1<br>(1 to 1)	458.6<br>(364.3 to 574.1)	1<br>(1 to 1)	450.3<br>(342.5 to 566.0)	1<br>(1 to 1)	445.2<br>(334.5 to 563.7)	1<br>(1 to 1)	438.5<br>(329.8 to 564.9)	-4.4<br>(-29.8 to 26.5)	-2.6<br>(-8.7 to 3.9)
**Niue**	All Ages	1<br>(1 to 2)	62.5<br>(51.9 to 75.4)	1<br>(1 to 1)	51.5<br>(42.1 to 61.9)	1<br>(1 to 1)	64.4<br>(55.9 to 74.7)	1<br>(1 to 1)	64.1<br>(55.8 to 73.8)	2.5<br>(-15.7 to 24.4)	24.4<br>(15.6 to 35.3)
	Under 5	0<br>(0 to 0)	106.0<br>(82.9 to 133.0)	0<br>(0 to 0)	82.0<br>(58.5 to 112.4)	0<br>(0 to 0)	224.6<br>(192.6 to 257.4)	0<br>(0 to 0)	225.9<br>(193.6 to 259.8)	113.1<br>(63.2 to 183.1)	175.4<br>(110.6 to 277.3)
	5-14 years	0<br>(0 to 0)	6.1<br>(4.6 to 7.9)	0<br>(0 to 0)	4.4<br>(3.3 to 5.9)	0<br>(0 to 0)	16.1<br>(13.2 to 19.8)	0<br>(0 to 0)	16.3<br>(13.4 to 19.9)	167.2<br>(95.8 to 265.8)	267.7<br>(182.0 to 344.4)
	15-49 years	0<br>(0 to 0)	11.7<br>(8.6 to 16.3)	0<br>(0 to 0)	8.4<br>(6.0 to 12.2)	0<br>(0 to 0)	10.6<br>(8.7 to 13.6)	0<br>(0 to 0)	10.5<br>(8.6 to 13.5)	-9.7<br>(-37.4 to 30.5)	25.4<br>(5.6 to 53.7)

	50-69 years	0 (0 to 0)	57.4 (43.7 to 72.7)	0 (0 to 0)	41.9 (31.2 to 53.8)	0 (0 to 0)	42.4 (31.7 to 54.6)	0 (0 to 0)	42.6 (31.9 to 55.4)	-25.7 (-47.6 to 3.9)	1.8 (-5.7 to 9.7)
	70+ years	1 (1 to 1)	566.5 (461.4 to 686.1)	1 (0 to 1)	434.1 (348.8 to 524.7)	1 (0 to 1)	438.5 (355.3 to 531.9)	1 (0 to 1)	434.5 (351.9 to 526.8)	-23.3 (-39.3 to -4.5)	0.1 (-4.9 to 5.5)
**Northern Mariana Islands**	All Ages	7 (5 to 9)	15.5 (12.1 to 19.8)	10 (8 to 12)	19.7 (16.1 to 23.6)	8 (7 to 11)	17.4 (13.8 to 21.6)	9 (8 to 10)	18.5 (16.1 to 20.7)	19.3 (-7.8 to 52.9)	-6.0 (-21.4 to 10.5)
	Under 5	2 (1 to 2)	31.8 (23.8 to 41.9)	1 (0 to 1)	17.1 (13.0 to 22.2)	0 (0 to 1)	14.0 (10.5 to 18.5)	0 (0 to 0)	11.6 (8.7 to 14.8)	-63.5 (-72.3 to -51.1)	-32.3 (-42.8 to -22.5)
	5-14 years	0 (0 to 0)	1.9 (1.4 to 2.6)	0 (0 to 0)	1.4 (1.1 to 1.9)	0 (0 to 0)	1.2 (0.5 to 1.8)	0 (0 to 0)	1.2 (1.0 to 1.6)	-37.0 (-55.1 to -9.6)	-14.2 (-27.3 to -1.7)
	15-49 years	3 (2 to 3)	8.9 (5.9 to 12.1)	1 (1 to 2)	6.0 (4.6 to 8.2)	1 (1 to 1)	4.6 (3.6 to 5.9)	1 (1 to 2)	5.5 (4.4 to 6.6)	-38.2 (-57.3 to -8.6)	-9.5 (-22.3 to 6.9)
	50-69 years	1 (1 to 2)	40.0 (31.4 to 51.5)	3 (3 to 4)	30.1 (23.5 to 37.4)	4 (3 to 5)	30.9 (23.1 to 40.4)	3 (2 to 4)	26.1 (21.4 to 31.0)	-34.8 (-52.7 to -13.4)	-13.3 (-27.7 to 4.2)
	70+ years	1 (1 to 2)	292.1 (240.9 to 356.3)	4 (3 to 5)	255.7 (213.6 to 299.5)	3 (3 to 4)	174.2 (136.1 to 218.3)	4 (3 to 5)	200.3 (162.9 to 237.3)	-31.4 (-47.0 to -14.5)	-21.7 (-35.1 to -6.5)
**Palau**	All Ages	6 (5 to 8)	39.7 (32.2 to 49.4)	6 (5 to 7)	34.0 (28.0 to 40.9)	6 (5 to 8)	34.4 (28.1 to 41.8)	6 (5 to 8)	34.9 (28.2 to 43.0)	-12.3 (-32.1 to 13.6)	2.5 (-3.0 to 8.6)
	Under 5	2 (1 to 3)	134.8 (98.6 to 177.4)	1 (0 to 1)	51.3 (38.4 to 66.1)	0 (0 to 1)	50.0 (38.1 to 64.5)	0 (0 to 1)	49.0 (37.2 to 63.3)	-63.7 (-74.8 to -46.9)	-4.6 (-15.0 to 6.5)
	5-14 years	0 (0 to 0)	5.0 (3.6 to 6.5)	0 (0 to 0)	2.1 (1.6 to 2.6)	0 (0 to 0)	2.0 (1.6 to 2.6)	0 (0 to 0)	2.0 (1.6 to 2.6)	-59.5 (-70.4 to -44.3)	-2.4 (-10.8 to 6.9)
	15-49 years	1 (1 to 1)	13.3 (9.7 to 17.3)	1 (1 to 2)	13.3 (10.7 to 16.5)	1 (1 to 2)	13.3 (10.6 to 16.5)	1 (1 to 2)	13.3 (10.5 to 16.6)	0.1 (-30.6 to 41.2)	-0.2 (-7.0 to 6.7)
	50-69 years	1 (1 to 1)	61.0 (46.7 to 78.8)	2 (1 to 2)	37.0 (27.8 to 48.2)	2 (1 to 2)	36.6 (27.4 to 47.9)	2 (1 to 2)	36.9 (27.5 to 48.2)	-39.5 (-57.4 to -13.8)	-0.4 (-8.1 to 8.4)
	70+ years	2 (1 to 2)	373.9 (298.7 to 465.2)	3 (2 to 3)	329.5 (263.2 to 409.8)	3 (2 to 3)	312.1 (248.1 to 387.6)	3 (2 to 3)	307.6 (243.0 to 381.3)	-17.7 (-37.2 to 5.6)	-6.6 (-12.5 to -0.6)
**Papua New Guinea**	All Ages	4,440 (3,650 to 5,500)	108.2 (88.9 to 134.1)	6,580 (5,270 to 8,180)	66.6 (53.3 to 82.7)	5,950 (4,730 to 7,370)	58.5 (46.4 to 72.4)	5,460 (4,360 to 6,770)	52.2 (41.7 to 64.7)	-51.8 (-62.7 to -37.0)	-21.6 (-29.5 to -12.6)
	Under 5	3,790 (2,980 to 4,840)	585.5 (460.7 to 746.6)	5,220 (4,030 to 6,810)	361.6 (279.3 to 471.5)	4,650 (3,520 to 6,110)	313.7 (237.1 to 411.8)	4,220 (3,230 to 5,490)	277.4 (212.2 to 360.9)	-52.6 (-65.1 to -36.1)	-23.3 (-32.8 to -12.1)
	5-14 years	56 (37 to 90)	5.3 (3.5 to 8.5)	86 (54 to 170)	3.8 (2.4 to 7.6)	83 (53 to 162)	3.6 (2.3 to 7.0)	78 (49 to 150)	3.3 (2.1 to 6.3)	-38.6 (-61.9 to -4.9)	-15.1 (-28.5 to 2.6)
	15-49 years	154 (108 to 240)	7.8 (5.4 to 12.1)	334 (234 to 633)	6.6 (4.6 to 12.4)	313 (219 to 590)	6.0 (4.2 to 11.3)	299 (207 to 562)	5.6 (3.9 to 10.5)	-27.8 (-52.3 to 10.6)	-14.7 (-21.8 to -7.5)

	50-69 years	232<br>(163 to 329)	65.4<br>(46.0 to 92.5)	418<br>(306 to 613)	44.2<br>(32.4 to 65.0)	412<br>(301 to 623)	41.6<br>(30.4 to 62.8)	403<br>(286 to 609)	39.1<br>(27.8 to 59.1)	-40.1<br>(-60.4 to -11.0)	-11.5<br>(-21.4 to -3.0)
	70+ years	206<br>(151 to 301)	343.5<br>(252.0 to 501.9)	522<br>(400 to 742)	314.9<br>(241.2 to 447.4)	491<br>(371 to 698)	291.3<br>(220.0 to 414.0)	460<br>(340 to 660)	264.6<br>(195.3 to 379.2)	-23.0<br>(-46.0 to 9.2)	-16.0<br>(-25.1 to -8.2)
**Samoa**	All Ages	74<br>(61 to 89)	43.6<br>(36.0 to 52.7)	67<br>(56 to 80)	32.1<br>(26.7 to 38.5)	67<br>(56 to 81)	31.8<br>(26.4 to 38.5)	67<br>(55 to 82)	31.3<br>(26.0 to 38.3)	-28.2<br>(-37.9 to -15.9)	-2.4<br>(-9.8 to 5.7)
	Under 5	35<br>(25 to 47)	138.7<br>(97.6 to 185.1)	14<br>(10 to 20)	48.8<br>(33.4 to 67.9)	14<br>(9 to 19)	47.6<br>(31.8 to 66.0)	13<br>(9 to 19)	45.3<br>(29.2 to 64.3)	-67.4<br>(-79.5 to -49.1)	-7.2<br>(-32.1 to 27.9)
	5-14 years	2<br>(2 to 3)	5.4<br>(4.0 to 7.1)	1<br>(1 to 2)	2.8<br>(2.0 to 3.8)	1<br>(1 to 2)	2.8<br>(2.0 to 3.7)	1<br>(1 to 2)	2.7<br>(1.9 to 3.6)	-50.7<br>(-65.5 to -28.2)	-5.4<br>(-23.3 to 17.6)
	15-49 years	8<br>(6 to 11)	10.4<br>(8.0 to 13.7)	10<br>(7 to 13)	10.3<br>(7.4 to 13.6)	10<br>(7 to 13)	10.2<br>(7.4 to 13.6)	10<br>(7 to 14)	10.0<br>(7.2 to 13.6)	-3.7<br>(-33.8 to 36.4)	-2.3<br>(-11.6 to 6.4)
	50-69 years	10<br>(7 to 12)	61.3<br>(47.3 to 78.9)	12<br>(10 to 16)	48.5<br>(38.4 to 61.8)	13<br>(10 to 16)	48.3<br>(37.9 to 61.7)	13<br>(10 to 16)	48.1<br>(38.0 to 62.1)	-21.6<br>(-42.2 to 6.8)	-1.0<br>(-8.0 to 6.6)
	70+ years	18<br>(15 to 23)	479.2<br>(392.5 to 598.6)	29<br>(24 to 35)	441.9<br>(364.0 to 538.4)	29<br>(24 to 36)	437.8<br>(360.3 to 533.4)	29<br>(24 to 36)	431.3<br>(355.3 to 524.8)	-10.0<br>(-26.6 to 12.7)	-2.4<br>(-7.6 to 3.7)
**Solomon Islands**	All Ages	351<br>(271 to 433)	103.4<br>(79.9 to 127.6)	416<br>(338 to 505)	63.3<br>(51.4 to 76.8)	417<br>(341 to 508)	62.2<br>(50.9 to 75.7)	418<br>(340 to 509)	61.1<br>(49.8 to 74.5)	-40.9<br>(-51.8 to -24.3)	-3.5<br>(-9.6 to 2.0)
	Under 5	183<br>(140 to 236)	306.2<br>(235.0 to 395.7)	112<br>(86 to 143)	120.2<br>(91.5 to 153.3)	106<br>(79 to 136)	112.2<br>(84.1 to 144.4)	104<br>(78 to 134)	109.0<br>(81.3 to 141.1)	-64.4<br>(-74.8 to -50.8)	-9.3<br>(-20.4 to 3.9)
	5-14 years	9<br>(7 to 12)	9.5<br>(6.8 to 12.7)	11<br>(8 to 14)	6.6<br>(5.0 to 8.9)	10<br>(8 to 14)	6.2<br>(4.7 to 8.5)	10<br>(8 to 14)	6.2<br>(4.6 to 8.4)	-35.3<br>(-55.8 to -2.8)	-6.7<br>(-18.2 to 7.1)
	15-49 years	45<br>(26 to 62)	29.6<br>(17.4 to 40.6)	87<br>(62 to 118)	26.4<br>(18.8 to 35.6)	89<br>(64 to 121)	26.3<br>(18.9 to 35.7)	89<br>(64 to 121)	25.8<br>(18.7 to 35.1)	-12.7<br>(-41.6 to 68.5)	-2.3<br>(-9.4 to 5.2)
	50-69 years	60<br>(36 to 82)	222.5<br>(131.5 to 303.1)	90<br>(67 to 123)	148.2<br>(110.4 to 203.9)	93<br>(70 to 126)	147.5<br>(110.1 to 200.1)	94<br>(70 to 127)	142.7<br>(106.8 to 192.1)	-35.9<br>(-55.9 to 9.1)	-3.7<br>(-11.6 to 4.6)
	70+ years	54<br>(39 to 66)	1,141.2<br>(833.1 to 1,414.9)	116<br>(91 to 141)	918.3<br>(714.3 to 1,113.6)	119<br>(93 to 145)	918.9<br>(717.9 to 1,121.1)	120<br>(94 to 148)	909.2<br>(709.1 to 1,113.1)	-20.3<br>(-37.6 to 1.9)	-1.0<br>(-6.3 to 4.8)
**Tokelau**	All Ages	1<br>(1 to 1)	55.9<br>(45.8 to 67.8)	1<br>(0 to 1)	42.3<br>(33.6 to 52.8)	1<br>(1 to 1)	61.4<br>(50.5 to 72.9)	1<br>(1 to 1)	60.5<br>(49.4 to 71.5)	8.2<br>(-12.5 to 32.2)	42.9<br>(27.5 to 60.6)
	Under 5	0<br>(0 to 0)	129.5<br>(95.0 to 169.4)	0<br>(0 to 0)	30.6<br>(20.0 to 43.9)	0<br>(0 to 0)	237.3<br>(162.3 to 287.7)	0<br>(0 to 0)	240.4<br>(164.9 to 290.0)	85.5<br>(29.0 to 159.5)	686.4<br>(454.5 to 1,023.3)
	5-14 years	0<br>(0 to 0)	5.9<br>(4.3 to 7.8)	0<br>(0 to 0)	2.4<br>(1.6 to 3.3)	0<br>(0 to 0)	14.3<br>(10.4 to 18.9)	0<br>(0 to 0)	14.1<br>(10.2 to 18.5)	140.7<br>(69.0 to 241.8)	493.6<br>(359.7 to 666.7)
	15-49 years	0<br>(0 to 0)	13.0<br>(9.1 to 18.1)	0<br>(0 to 0)	8.6<br>(6.1 to 12.7)	0<br>(0 to 0)	12.2<br>(10.2 to 15.3)	0<br>(0 to 0)	12.1<br>(10.2 to 15.1)	-7.1<br>(-32.9 to 30.5)	40.3<br>(13.9 to 79.4)

	50-69 years	0 (0 to 0)	70.9 (51.7 to 92.5)	0 (0 to 0)	43.1 (31.8 to 56.1)	0 (0 to 0)	42.7 (31.2 to 55.3)	0 (0 to 0)	41.2 (30.2 to 53.2)	-41.9 (-58.2 to -17.5)	-4.5 (-11.7 to 4.5)
	70+ years	0 (0 to 0)	489.0 (391.9 to 598.5)	0 (0 to 0)	443.5 (351.4 to 544.3)	0 (0 to 0)	434.2 (343.9 to 534.3)	0 (0 to 0)	409.2 (325.8 to 499.0)	-16.3 (-33.5 to 4.3)	-7.7 (-12.8 to -1.8)
**Tonga**	All Ages	39 (34 to 46)	39.8 (34.2 to 46.1)	40 (33 to 49)	38.1 (30.7 to 46.4)	40 (32 to 49)	38.0 (30.4 to 46.5)	40 (32 to 49)	37.8 (30.2 to 46.1)	-5.2 (-26.8 to 20.2)	-0.9 (-5.7 to 4.3)
	Under 5	16 (13 to 21)	107.8 (83.4 to 136.4)	7 (5 to 9)	47.0 (34.9 to 62.2)	7 (5 to 9)	46.1 (33.4 to 61.5)	6 (5 to 9)	44.5 (31.7 to 59.3)	-58.7 (-71.6 to -40.0)	-5.4 (-15.1 to 4.8)
	5-14 years	1 (1 to 1)	3.6 (2.6 to 4.5)	1 (0 to 1)	2.7 (2.0 to 3.6)	1 (0 to 1)	2.7 (2.0 to 3.6)	1 (0 to 1)	2.6 (1.9 to 3.5)	-26.8 (-49.7 to 5.9)	-3.5 (-11.5 to 5.4)
	15-49 years	4 (3 to 4)	8.1 (6.4 to 10.0)	4 (3 to 5)	8.1 (6.1 to 10.9)	4 (3 to 5)	8.0 (6.1 to 10.9)	4 (3 to 5)	8.0 (6.0 to 10.8)	-1.2 (-31.2 to 40.1)	-1.2 (-8.0 to 6.5)
	50-69 years	6 (5 to 7)	58.1 (46.1 to 72.0)	6 (5 to 8)	46.3 (35.1 to 58.9)	6 (5 to 8)	45.9 (34.6 to 58.6)	6 (5 to 8)	45.5 (34.0 to 58.3)	-21.7 (-45.0 to 8.1)	-1.8 (-9.4 to 5.6)
	70+ years	12 (10 to 15)	480.9 (390.2 to 578.7)	23 (18 to 28)	525.8 (415.2 to 637.5)	23 (18 to 28)	525.4 (417.4 to 642.1)	23 (18 to 28)	523.5 (412.8 to 646.8)	8.9 (-15.9 to 40.0)	-0.4 (-5.8 to 5.4)
**Tuvalu**	All Ages	11 (9 to 14)	117.9 (96.5 to 145.1)	5 (4 to 6)	40.1 (33.6 to 48.8)	5 (4 to 6)	39.4 (32.8 to 47.6)	5 (4 to 6)	38.8 (32.4 to 47.0)	-67.1 (-74.4 to -58.6)	-3.5 (-8.2 to 2.3)
	Under 5	8 (6 to 10)	507.7 (392.7 to 666.5)	1 (1 to 1)	77.1 (57.1 to 100.5)	1 (1 to 1)	72.1 (52.5 to 95.5)	1 (1 to 1)	67.8 (49.4 to 91.0)	-86.6 (-91.2 to -80.7)	-12.0 (-22.1 to -0.1)
	5-14 years	0 (0 to 0)	11.8 (8.9 to 15.6)	0 (0 to 0)	4.2 (3.2 to 5.5)	0 (0 to 0)	4.1 (3.1 to 5.3)	0 (0 to 0)	3.9 (3.0 to 5.1)	-66.6 (-77.6 to -51.4)	-7.3 (-16.4 to 3.9)
	15-49 years	1 (1 to 1)	19.9 (15.7 to 24.6)	1 (1 to 1)	12.7 (9.9 to 16.1)	1 (1 to 1)	12.6 (9.8 to 15.8)	1 (1 to 1)	12.4 (9.7 to 15.7)	-37.5 (-51.3 to -19.0)	-2.4 (-9.7 to 5.5)
	50-69 years	1 (1 to 2)	95.1 (75.8 to 121.3)	1 (1 to 1)	57.3 (46.1 to 71.3)	1 (1 to 1)	57.1 (45.7 to 71.5)	1 (1 to 1)	56.5 (45.4 to 70.8)	-40.6 (-54.2 to -23.0)	-1.4 (-9.0 to 6.9)
	70+ years	1 (1 to 2)	456.6 (369.2 to 568.7)	2 (2 to 2)	404.0 (335.1 to 493.8)	2 (2 to 2)	399.3 (331.2 to 490.5)	2 (2 to 2)	390.2 (321.6 to 477.1)	-14.5 (-31.2 to 7.4)	-3.4 (-8.7 to 2.1)
**Vanuatu**	All Ages	75 (61 to 92)	49.5 (39.9 to 60.1)	103 (85 to 122)	34.2 (28.3 to 40.5)	103 (85 to 124)	33.7 (27.8 to 40.3)	88 (73 to 107)	28.2 (23.4 to 34.0)	-43.0 (-53.7 to -28.7)	-17.5 (-23.3 to -11.1)
	Under 5	44 (32 to 57)	161.5 (118.7 to 212.1)	38 (28 to 50)	90.4 (67.4 to 119.4)	36 (26 to 49)	86.6 (62.8 to 115.9)	28 (20 to 38)	66.6 (47.4 to 89.0)	-58.8 (-71.1 to -39.3)	-26.4 (-37.1 to -11.9)
	5-14 years	3 (2 to 3)	6.3 (4.5 to 8.4)	4 (3 to 5)	4.8 (3.7 to 6.2)	3 (3 to 5)	4.7 (3.6 to 6.2)	3 (2 to 4)	3.8 (2.8 to 4.9)	-39.7 (-58.1 to -15.3)	-22.1 (-32.7 to -9.4)
	15-49 years	11 (8 to 14)	15.5 (11.2 to 20.5)	20 (16 to 26)	13.7 (10.6 to 17.3)	21 (16 to 27)	13.6 (10.5 to 17.5)	19 (15 to 25)	12.3 (9.4 to 15.7)	-21.1 (-46.0 to 12.9)	-10.4 (-17.5 to -3.4)

	50-69 years	9 (7 to 12)	76.5 (58.0 to 101.8)	18 (14 to 23)	58.0 (45.8 to 73.7)	19 (15 to 24)	58.0 (45.4 to 73.8)	18 (14 to 23)	52.6 (41.1 to 68.5)	-31.2 (-51.5 to -2.0)	-9.3 (-16.2 to -2.0)
	70+ years	9 (7 to 12)	406.6 (316.4 to 520.0)	23 (19 to 28)	364.4 (297.3 to 446.1)	24 (20 to 29)	367.8 (300.8 to 449.6)	21 (17 to 25)	309.4 (249.5 to 379.0)	-23.9 (-41.4 to -2.3)	-15.1 (-20.4 to -9.5)
**Cambodia**	All Ages	22,800 (19,600 to 26,900)	222.4 (190.6 to 261.7)	11,700 (9,380 to 14,200)	70.2 (56.5 to 85.8)	11,700 (9,380 to 14,300)	69.7 (55.7 to 85.1)	9,560 (7,570 to 11,700)	56.1 (44.4 to 68.9)	-74.8 (-80.2 to -68.9)	-20.1 (-24.9 to -15.7)
	Under 5	18,300 (15,200 to 22,400)	1,004.8 (831.6 to 1,225.4)	3,260 (2,540 to 4,140)	185.2 (144.4 to 235.2)	3,090 (2,400 to 3,880)	176.1 (136.6 to 220.8)	2,220 (1,710 to 2,790)	126.9 (97.7 to 159.4)	-87.4 (-90.5 to -83.6)	-31.5 (-37.7 to -25.1)
	5-14 years	574 (449 to 733)	20.2 (15.8 to 25.8)	236 (178 to 305)	7.1 (5.4 to 9.2)	233 (177 to 301)	7.0 (5.3 to 9.0)	187 (138 to 244)	5.6 (4.1 to 7.3)	-72.5 (-81.7 to -59.6)	-22.1 (-30.4 to -13.1)
	15-49 years	826 (678 to 987)	18.0 (14.8 to 21.5)	1,010 (735 to 1,400)	11.5 (8.4 to 16.0)	1,020 (746 to 1,420)	11.5 (8.4 to 16.0)	879 (644 to 1,240)	9.8 (7.1 to 13.8)	-45.7 (-61.1 to -21.8)	-15.3 (-22.7 to -7.3)
	50-69 years	1,250 (987 to 1,540)	150.3 (118.7 to 185.1)	2,260 (1,670 to 2,890)	100.5 (74.3 to 128.7)	2,320 (1,720 to 3,040)	100.4 (74.6 to 131.5)	2,070 (1,520 to 2,720)	87.4 (64.4 to 115.0)	-41.8 (-59.7 to -17.5)	-13.0 (-20.0 to -6.4)
	70+ years	1,860 (1,490 to 2,260)	995.8 (798.2 to 1,214.0)	4,890 (3,750 to 5,920)	966.8 (740.5 to 1,169.2)	5,060 (3,880 to 6,100)	959.6 (735.3 to 1,156.2)	4,210 (3,200 to 5,100)	769.0 (584.0 to 932.9)	-22.8 (-42.2 to 1.9)	-20.5 (-25.2 to -15.4)
**Indonesia**	All Ages	70,100 (60,400 to 82,000)	37.9 (32.7 to 44.3)	46,400 (39,500 to 52,200)	17.0 (14.5 to 19.2)	44,900 (37,800 to 51,200)	16.3 (13.7 to 18.6)	40,200 (33,200 to 46,300)	14.4 (11.9 to 16.6)	-62.0 (-70.1 to -52.2)	-15.2 (-29.0 to 1.2)
	Under 5	49,900 (41,500 to 61,000)	223.7 (185.9 to 273.3)	11,300 (8,630 to 14,300)	50.8 (38.9 to 64.6)	9,950 (7,600 to 12,800)	45.1 (34.5 to 58.1)	8,020 (6,160 to 10,300)	36.6 (28.1 to 47.0)	-83.6 (-88.3 to -77.3)	-27.9 (-37.1 to -16.7)
	5-14 years	2,270 (1,650 to 2,900)	5.0 (3.6 to 6.4)	776 (629 to 936)	1.7 (1.4 to 2.0)	710 (577 to 858)	1.6 (1.3 to 1.9)	602 (484 to 728)	1.3 (1.1 to 1.6)	-73.4 (-80.1 to -62.2)	-21.4 (-33.2 to -7.4)
	15-49 years	3,910 (3,140 to 4,820)	4.1 (3.3 to 5.1)	3,420 (2,830 to 4,250)	2.3 (1.9 to 2.8)	3,320 (2,660 to 4,200)	2.2 (1.8 to 2.8)	3,010 (2,370 to 3,950)	2.0 (1.5 to 2.6)	-52.4 (-66.3 to -32.5)	-13.8 (-35.5 to 20.4)
	50-69 years	6,010 (4,560 to 7,530)	31.8 (24.1 to 39.8)	10,200 (8,080 to 12,100)	22.5 (17.8 to 26.7)	10,300 (8,160 to 12,400)	22.0 (17.4 to 26.3)	9,890 (7,450 to 12,000)	20.3 (15.3 to 24.7)	-36.0 (-55.8 to -6.6)	-9.6 (-31.2 to 20.0)
	70+ years	8,030 (5,670 to 10,400)	210.9 (148.8 to 273.1)	20,700 (16,500 to 23,700)	231.5 (184.2 to 264.7)	20,600 (16,300 to 23,800)	221.1 (175.4 to 255.0)	18,700 (14,500 to 21,700)	194.0 (150.1 to 224.5)	-8.0 (-36.8 to 40.1)	-16.2 (-32.5 to 2.9)
**Lao People's Democratic Republic**	All Ages	8,990 (7,160 to 11,300)	215.6 (171.7 to 271.7)	3,370 (2,620 to 4,140)	47.0 (36.6 to 57.7)	3,340 (2,580 to 4,190)	45.9 (35.4 to 57.5)	2,800 (2,160 to 3,540)	38.0 (29.3 to 48.1)	-82.4 (-87.0 to -76.4)	-19.1 (-25.4 to -12.9)
	Under 5	7,470 (5,700 to 9,740)	1,050.5 (801.9 to 1,370.8)	1,450 (1,040 to 1,930)	176.1 (125.9 to 234.6)	1,380 (958 to 1,890)	166.9 (115.7 to 227.9)	1,060 (736 to 1,460)	127.8 (88.7 to 175.4)	-87.8 (-91.6 to -82.9)	-27.4 (-36.9 to -16.8)
	5-14 years	292 (202 to 394)	25.8 (17.8 to 34.8)	96 (72 to 127)	6.6 (4.9 to 8.7)	94 (70 to 125)	6.4 (4.8 to 8.6)	82 (59 to 108)	5.6 (4.0 to 7.4)	-78.4 (-85.8 to -66.6)	-16.1 (-27.6 to -2.6)
	15-49 years	292 (217 to 369)	15.7 (11.7 to 19.9)	274 (202 to 366)	7.0 (5.2 to 9.4)	275 (201 to 371)	7.0 (5.1 to 9.4)	244 (178 to 332)	6.1 (4.5 to 8.3)	-61.2 (-74.1 to -44.4)	-13.4 (-20.3 to -5.5)

	50-69 years	420<br>(308 to 551)	110.7<br>(81.3 to 145.2)	460<br>(344 to 596)	55.3<br>(41.4 to 71.7)	469<br>(349 to 610)	54.6<br>(40.7 to 71.1)	429<br>(317 to 560)	48.4<br>(35.8 to 63.2)	-56.3<br>(-71.0 to -34.1)	-12.5<br>(-20.2 to -4.4)
	70+ years	519<br>(396 to 656)	584.3<br>(446.5 to 739.4)	1,090<br>(832 to 1,350)	611.4<br>(466.0 to 757.8)	1,130<br>(854 to 1,400)	601.9<br>(456.1 to 748.3)	988<br>(747 to 1,230)	513.1<br>(387.7 to 637.6)	-12.2<br>(-36.9 to 21.6)	-16.1<br>(-22.5 to -9.2)
**Malaysia**	All Ages	5,650<br>(5,020 to 6,770)	32.0<br>(28.4 to 38.3)	19,100<br>(15,900 to 21,100)	61.3<br>(50.8 to 67.7)	16,600<br>(13,700 to 18,500)	52.5<br>(43.3 to 58.7)	17,900<br>(15,000 to 20,200)	56.3<br>(47.1 to 63.6)	76.0<br>(36.1 to 109.3)	-8.1<br>(-13.9 to -2.2)
	Under 5	1,050<br>(807 to 1,320)	44.1<br>(33.9 to 55.2)	356<br>(287 to 438)	14.2<br>(11.5 to 17.5)	281<br>(221 to 354)	11.3<br>(8.9 to 14.3)	221<br>(167 to 285)	9.0<br>(6.8 to 11.6)	-79.6<br>(-85.7 to -70.5)	-37.0<br>(-47.6 to -24.9)
	5-14 years	227<br>(180 to 277)	5.4<br>(4.3 to 6.6)	137<br>(114 to 166)	2.7<br>(2.2 to 3.2)	86<br>(70 to 108)	1.7<br>(1.3 to 2.1)	95<br>(75 to 118)	1.8<br>(1.5 to 2.3)	-66.2<br>(-73.9 to -54.9)	-30.8<br>(-39.5 to -21.0)
	15-49 years	714<br>(623 to 832)	7.9<br>(6.9 to 9.2)	1,740<br>(1,480 to 1,970)	10.0<br>(8.5 to 11.3)	1,530<br>(1,300 to 1,760)	8.7<br>(7.4 to 10.0)	1,560<br>(1,340 to 1,790)	8.8<br>(7.6 to 10.1)	11.1<br>(-12.4 to 35.2)	-11.8<br>(-17.1 to -6.2)
	50-69 years	1,220<br>(1,030 to 1,480)	74.1<br>(62.5 to 89.7)	5,390<br>(4,240 to 6,240)	111.3<br>(87.5 to 128.8)	4,960<br>(3,910 to 5,790)	100.2<br>(79.0 to 117.0)	4,940<br>(3,870 to 5,800)	98.1<br>(76.9 to 115.1)	32.3<br>(-3.2 to 71.0)	-11.9<br>(-16.9 to -6.1)
	70+ years	2,440<br>(2,010 to 3,170)	560.1<br>(461.9 to 728.8)	11,500<br>(9,550 to 13,100)	896.7<br>(744.5 to 1,017.8)	9,690<br>(7,900 to 11,100)	720.5<br>(587.1 to 822.0)	11,100<br>(9,060 to 12,800)	784.6<br>(640.6 to 907.9)	40.1<br>(1.7 to 75.7)	-12.5<br>(-20.6 to -5.1)
**Maldives**	All Ages	88<br>(76 to 104)	39.6<br>(34.1 to 46.8)	49<br>(42 to 56)	10.0<br>(8.5 to 11.4)	43<br>(37 to 50)	8.6<br>(7.3 to 10.0)	43<br>(35 to 51)	8.2<br>(6.8 to 9.8)	-79.2<br>(-83.7 to -73.9)	-17.9<br>(-26.2 to -10.9)
	Under 5	60<br>(48 to 75)	143.5<br>(115.7 to 179.7)	6<br>(5 to 8)	19.0<br>(14.2 to 24.8)	5<br>(4 to 6)	14.6<br>(10.9 to 19.1)	4<br>(3 to 5)	12.8<br>(9.5 to 17.1)	-91.1<br>(-93.8 to -86.8)	-32.5<br>(-40.8 to -22.2)
	5-14 years	6<br>(4 to 8)	9.8<br>(7.1 to 12.5)	1<br>(1 to 2)	1.9<br>(1.4 to 2.4)	1<br>(1 to 1)	1.6<br>(1.2 to 2.1)	1<br>(1 to 1)	1.5<br>(1.1 to 1.9)	-84.8<br>(-89.4 to -78.0)	-20.9<br>(-28.6 to -13.0)
	15-49 years	5<br>(4 to 6)	5.3<br>(4.3 to 6.2)	5<br>(4 to 6)	1.5<br>(1.3 to 1.8)	4<br>(4 to 5)	1.3<br>(1.1 to 1.6)	4<br>(3 to 5)	1.3<br>(1.0 to 1.6)	-75.3<br>(-81.7 to -66.6)	-14.4<br>(-28.3 to -4.3)
	50-69 years	8<br>(6 to 10)	41.7<br>(33.5 to 50.2)	6<br>(5 to 7)	10.0<br>(8.1 to 12.3)	5<br>(4 to 7)	8.6<br>(6.8 to 10.7)	6<br>(4 to 7)	8.6<br>(6.5 to 11.1)	-79.4<br>(-85.1 to -71.8)	-14.7<br>(-29.7 to -3.2)
	70+ years	9<br>(7 to 11)	297.7<br>(232.9 to 362.4)	31<br>(25 to 36)	235.7<br>(191.8 to 275.9)	28<br>(22 to 33)	208.5<br>(168.2 to 247.0)	27<br>(22 to 33)	202.1<br>(161.4 to 244.3)	-32.1<br>(-48.6 to -9.9)	-14.3<br>(-23.3 to -6.7)
**Mauritius**	All Ages	317<br>(301 to 332)	28.9<br>(27.4 to 30.3)	406<br>(371 to 430)	31.9<br>(29.2 to 33.8)	356<br>(323 to 382)	27.9<br>(25.4 to 30.0)	353<br>(321 to 378)	27.8<br>(25.3 to 29.7)	-4.1<br>(-12.1 to 3.2)	-12.9<br>(-19.0 to -7.8)
	Under 5	46<br>(42 to 51)	44.0<br>(39.9 to 48.2)	22<br>(19 to 25)	33.9<br>(29.7 to 38.5)	16<br>(14 to 19)	24.9<br>(21.0 to 29.3)	12<br>(10 to 14)	19.3<br>(15.8 to 22.5)	-56.2<br>(-64.9 to -47.0)	-43.2<br>(-52.4 to -33.0)
	5-14 years	10<br>(9 to 11)	4.3<br>(3.8 to 4.8)	4<br>(4 to 5)	2.7<br>(2.4 to 3.0)	2<br>(2 to 3)	1.6<br>(1.4 to 1.9)	2<br>(2 to 3)	1.6<br>(1.3 to 1.9)	-61.7<br>(-69.2 to -52.9)	-39.0<br>(-50.2 to -28.1)
	15-49 years	32<br>(30 to 34)	5.3<br>(4.9 to 5.6)	36<br>(33 to 39)	5.5<br>(5.1 to 6.0)	33<br>(30 to 36)	5.1<br>(4.6 to 5.6)	29<br>(26 to 31)	4.5<br>(4.1 to 4.9)	-14.4<br>(-24.1 to -4.2)	-18.5<br>(-24.6 to -14.3)

	50-69 years	67<br>(62 to 73)	52.3<br>(48.3 to 56.9)	94<br>(87 to 103)	29.4<br>(27.1 to 31.9)	85<br>(78 to 92)	26.1<br>(23.9 to 28.4)	83<br>(76 to 90)	25.6<br>(23.3 to 27.6)	-51.1<br>(-56.9 to -44.9)	-13.0<br>(-19.0 to -8.5)
	70+ years	163<br>(149 to 175)	494.1<br>(452.2 to 530.4)	250<br>(220 to 270)	278.8<br>(245.2 to 301.2)	220<br>(192 to 240)	232.6<br>(203.0 to 254.1)	227<br>(195 to 249)	226.0<br>(194.9 to 248.8)	-54.3<br>(-59.5 to -49.4)	-18.9<br>(-26.1 to -13.1)
**Myanmar**	All Ages	51,700<br>(40,700 to 64,600)	127.9<br>(100.7 to 159.9)	23,000<br>(18,900 to 27,500)	41.7<br>(34.2 to 49.7)	20,700<br>(17,100 to 24,700)	37.1<br>(30.6 to 44.3)	19,000<br>(15,400 to 23,000)	33.7<br>(27.3 to 40.7)	-73.6<br>(-79.6 to -65.3)	-19.1<br>(-24.2 to -13.1)
	Under 5	40,100<br>(29,800 to 52,200)	795.2<br>(590.0 to 1,035.5)	8,760<br>(6,600 to 11,400)	167.9<br>(126.5 to 218.8)	7,300<br>(5,450 to 9,520)	139.8<br>(104.2 to 182.3)	6,250<br>(4,620 to 8,200)	119.5<br>(88.5 to 156.8)	-85.0<br>(-89.4 to -78.2)	-28.8<br>(-36.8 to -20.0)
	5-14 years	1,600<br>(1,090 to 2,190)	16.4<br>(11.2 to 22.5)	587<br>(464 to 740)	5.7<br>(4.5 to 7.2)	537<br>(424 to 672)	5.2<br>(4.1 to 6.5)	486<br>(379 to 619)	4.7<br>(3.7 to 6.0)	-71.4<br>(-80.5 to -58.1)	-17.9<br>(-28.3 to -6.9)
	15-49 years	2,260<br>(1,730 to 2,940)	11.1<br>(8.5 to 14.4)	1,640<br>(1,240 to 2,120)	5.7<br>(4.3 to 7.3)	1,500<br>(1,120 to 1,980)	5.1<br>(3.8 to 6.8)	1,410<br>(1,060 to 1,880)	4.8<br>(3.6 to 6.4)	-56.5<br>(-70.5 to -37.7)	-15.0<br>(-21.9 to -7.2)
	50-69 years	3,460<br>(2,520 to 4,610)	81.2<br>(59.2 to 108.1)	3,870<br>(2,920 to 5,080)	44.5<br>(33.6 to 58.5)	3,720<br>(2,790 to 4,940)	41.5<br>(31.1 to 55.1)	3,600<br>(2,640 to 4,780)	39.2<br>(28.8 to 52.1)	-51.7<br>(-67.7 to -26.0)	-11.9<br>(-20.3 to -3.1)
	70+ years	4,300<br>(3,300 to 5,600)	440.1<br>(337.1 to 572.3)	8,200<br>(6,350 to 10,100)	386.9<br>(299.7 to 475.5)	7,640<br>(5,910 to 9,470)	353.9<br>(273.7 to 438.7)	7,280<br>(5,610 to 9,060)	325.8<br>(251.2 to 405.3)	-26.0<br>(-46.8 to 5.5)	-15.8<br>(-21.4 to -8.6)
**Philippines**	All Ages	40,500<br>(36,300 to 46,200)	64.3<br>(57.7 to 73.3)	60,000<br>(52,000 to 65,600)	54.3<br>(47.0 to 59.4)	52,100<br>(44,600 to 57,200)	46.6<br>(39.8 to 51.1)	51,400<br>(43,100 to 59,500)	45.4<br>(38.0 to 52.6)	-29.4<br>(-44.0 to -15.2)	-16.5<br>(-27.7 to -4.5)
	Under 5	23,600<br>(19,800 to 28,800)	254.9<br>(214.3 to 311.8)	8,800<br>(6,900 to 11,200)	77.1<br>(60.4 to 97.9)	6,390<br>(5,040 to 8,040)	56.5<br>(44.5 to 71.1)	5,720<br>(4,470 to 7,170)	51.0<br>(39.9 to 63.9)	-80.0<br>(-85.7 to -73.4)	-33.8<br>(-45.0 to -20.6)
	5-14 years	4,320<br>(3,460 to 5,040)	27.1<br>(21.6 to 31.6)	1,730<br>(1,460 to 2,220)	7.7<br>(6.5 to 9.8)	1,400<br>(1,200 to 1,760)	6.2<br>(5.3 to 7.8)	1,290<br>(1,060 to 1,650)	5.7<br>(4.7 to 7.3)	-79.1<br>(-84.1 to -69.4)	-26.2<br>(-34.9 to -16.6)
	15-49 years	3,880<br>(3,540 to 4,210)	12.5<br>(11.4 to 13.5)	5,600<br>(5,110 to 6,210)	9.6<br>(8.8 to 10.6)	4,890<br>(4,440 to 5,410)	8.3<br>(7.5 to 9.1)	5,000<br>(4,100 to 6,010)	8.3<br>(6.8 to 10.0)	-33.3<br>(-45.8 to -18.3)	-13.3<br>(-29.3 to 4.5)
	50-69 years	2,920<br>(2,590 to 3,410)	54.2<br>(48.1 to 63.2)	11,800<br>(10,600 to 13,100)	80.0<br>(72.2 to 89.0)	10,800<br>(9,620 to 12,000)	71.0<br>(63.4 to 79.2)	10,800<br>(8,720 to 13,200)	69.5<br>(55.9 to 84.8)	28.2<br>(-0.7 to 63.4)	-13.2<br>(-30.7 to 7.1)
	70+ years	5,800<br>(5,170 to 7,480)	447.4<br>(398.6 to 577.0)	32,100<br>(25,000 to 35,600)	954.3<br>(742.7 to 1,059.4)	28,700<br>(22,500 to 32,100)	816.3<br>(638.6 to 912.0)	28,500<br>(21,700 to 33,500)	788.4<br>(600.7 to 926.2)	76.2<br>(15.4 to 115.8)	-17.4<br>(-29.2 to -5.1)
**Seychelles**	All Ages	50<br>(45 to 55)	68.2<br>(61.1 to 75.6)	73<br>(64 to 81)	70.1<br>(62.0 to 78.1)	63<br>(56 to 71)	60.7<br>(53.3 to 68.2)	55<br>(48 to 63)	52.6<br>(45.3 to 59.9)	-22.8<br>(-33.6 to -10.5)	-25.0<br>(-30.0 to -19.6)
	Under 5	5<br>(4 to 5)	55.9<br>(47.2 to 65.0)	4<br>(3 to 5)	52.2<br>(42.9 to 63.5)	4<br>(3 to 5)	49.6<br>(40.0 to 61.0)	3<br>(2 to 4)	36.0<br>(27.9 to 45.7)	-35.5<br>(-52.0 to -14.3)	-30.9<br>(-38.6 to -23.1)
	5-14 years	1<br>(1 to 1)	5.2<br>(4.3 to 6.3)	1<br>(1 to 1)	5.1<br>(4.2 to 6.2)	0<br>(0 to 0)	1.0<br>(0.7 to 1.4)	0<br>(0 to 0)	1.3<br>(0.9 to 1.8)	-74.5<br>(-81.7 to -64.8)	-74.0<br>(-80.5 to -66.7)
	15-49 years	5<br>(4 to 5)	12.5<br>(11.0 to 14.1)	6<br>(5 to 7)	11.8<br>(10.1 to 13.8)	7<br>(6 to 8)	12.3<br>(10.6 to 14.4)	5<br>(4 to 5)	8.3<br>(6.8 to 10.0)	-33.2<br>(-46.2 to -17.5)	-29.3<br>(-36.4 to -21.4)

	50-69 years	10 (8 to 11)	110.3 (92.6 to 132.3)	16 (13 to 19)	75.7 (63.6 to 90.0)	15 (13 to 18)	69.8 (58.0 to 83.0)	12 (10 to 14)	53.1 (43.4 to 63.9)	-51.9 (-63.0 to -37.1)	-29.9 (-36.4 to -22.8)
	70+ years	30 (26 to 35)	881.4 (750.4 to 1,011.2)	45 (38 to 52)	876.5 (737.7 to 1,003.6)	38 (31 to 43)	701.6 (585.8 to 807.4)	36 (30 to 42)	652.3 (540.8 to 763.7)	-26.0 (-38.5 to -10.3)	-25.6 (-31.2 to -19.6)
**Sri Lanka**	All Ages	3,630 (3,240 to 4,110)	21.2 (18.9 to 24.0)	4,070 (3,120 to 5,340)	18.5 (14.2 to 24.2)	3,220 (1,840 to 4,600)	14.6 (8.3 to 20.8)	3,450 (2,300 to 4,660)	15.5 (10.3 to 20.9)	-27.0 (-50.8 to -0.8)	-16.3 (-40.5 to 3.8)
	Under 5	573 (486 to 667)	32.4 (27.5 to 37.7)	156 (115 to 203)	9.5 (7.0 to 12.4)	111 (81 to 151)	6.9 (5.1 to 9.4)	86 (59 to 123)	5.5 (3.8 to 7.8)	-83.1 (-88.6 to -75.3)	-42.5 (-54.1 to -28.7)
	5-14 years	189 (149 to 234)	5.0 (3.9 to 6.2)	54 (40 to 70)	1.5 (1.1 to 2.0)	14 (0 to 32)	0.4 (0.0 to 0.9)	38 (27 to 50)	1.1 (0.8 to 1.4)	-78.7 (-85.0 to -69.5)	-28.4 (-40.5 to -16.0)
	15-49 years	611 (534 to 697)	6.7 (5.8 to 7.6)	423 (302 to 581)	3.9 (2.8 to 5.3)	335 (190 to 478)	3.1 (1.7 to 4.4)	349 (226 to 482)	3.2 (2.0 to 4.4)	-52.5 (-69.0 to -33.2)	-18.4 (-42.5 to 3.9)
	50-69 years	763 (629 to 913)	39.8 (32.8 to 47.6)	1,090 (770 to 1,540)	24.3 (17.1 to 34.2)	901 (527 to 1,320)	19.7 (11.5 to 28.8)	914 (533 to 1,320)	19.8 (11.5 to 28.6)	-50.3 (-71.6 to -25.3)	-18.7 (-48.5 to 6.3)
	70+ years	1,500 (1,260 to 1,840)	303.3 (256.0 to 373.2)	2,340 (1,760 to 3,150)	172.3 (129.5 to 231.8)	1,860 (984 to 2,760)	129.7 (68.4 to 191.9)	2,060 (1,380 to 2,860)	135.5 (90.4 to 187.7)	-55.3 (-70.1 to -37.8)	-21.4 (-42.6 to -4.2)
**Thailand**	All Ages	9,930 (8,180 to 14,200)	17.5 (14.4 to 25.1)	38,000 (29,400 to 44,400)	56.9 (44.0 to 66.5)	36,000 (27,500 to 43,100)	54.0 (41.3 to 64.6)	36,400 (26,600 to 45,700)	54.6 (39.9 to 68.5)	212.0 (89.3 to 328.7)	-4.1 (-19.6 to 14.5)
	Under 5	3,620 (2,790 to 4,680)	69.6 (53.7 to 90.0)	612 (516 to 727)	20.4 (17.2 to 24.2)	479 (391 to 584)	16.5 (13.5 to 20.1)	423 (334 to 527)	15.0 (11.8 to 18.6)	-78.5 (-84.9 to -69.8)	-26.7 (-35.9 to -17.6)
	5-14 years	465 (331 to 792)	4.0 (2.8 to 6.8)	174 (145 to 205)	2.4 (2.0 to 2.8)	155 (128 to 185)	2.2 (1.8 to 2.6)	139 (113 to 169)	2.0 (1.6 to 2.4)	-49.6 (-69.0 to -24.8)	-16.1 (-24.6 to -7.3)
	15-49 years	939 (687 to 1,540)	3.0 (2.2 to 4.9)	2,300 (1,910 to 2,690)	7.0 (5.8 to 8.2)	2,070 (1,680 to 2,500)	6.4 (5.2 to 7.7)	1,950 (1,490 to 2,490)	6.1 (4.7 to 7.8)	106.0 (14.6 to 211.5)	-12.6 (-29.0 to 7.7)
	50-69 years	1,460 (1,090 to 2,430)	21.7 (16.3 to 36.2)	7,250 (5,670 to 8,780)	40.5 (31.7 to 49.0)	6,920 (5,210 to 8,860)	37.6 (28.3 to 48.2)	6,860 (4,940 to 9,140)	36.6 (26.3 to 48.7)	68.2 (-12.1 to 166.9)	-9.7 (-28.6 to 14.5)
	70+ years	3,450 (2,530 to 6,310)	225.5 (165.3 to 412.7)	27,600 (20,900 to 32,900)	483.2 (365.7 to 575.1)	26,400 (19,600 to 32,100)	443.0 (329.1 to 539.0)	27,000 (19,700 to 34,300)	433.7 (315.5 to 550.4)	92.3 (-4.6 to 189.0)	-10.2 (-24.3 to 6.5)
**Timor-Leste**	All Ages	1,360 (1,140 to 1,680)	174.4 (145.9 to 214.4)	674 (549 to 810)	50.2 (41.0 to 60.4)	688 (557 to 832)	50.2 (40.7 to 60.8)	595 (484 to 718)	42.6 (34.6 to 51.3)	-75.6 (-81.9 to -68.8)	-15.3 (-21.4 to -9.3)
	Under 5	1,220 (995 to 1,520)	867.5 (710.6 to 1,088.7)	296 (223 to 385)	167.0 (125.7 to 216.9)	295 (224 to 384)	163.4 (124.1 to 212.6)	243 (178 to 315)	131.5 (96.3 to 170.6)	-84.8 (-89.0 to -79.8)	-21.3 (-32.7 to -9.9)
	5-14 years	38 (26 to 49)	19.6 (13.6 to 25.4)	20 (14 to 27)	6.0 (4.3 to 8.2)	20 (14 to 26)	5.9 (4.3 to 7.9)	17 (12 to 23)	5.1 (3.7 to 6.9)	-74.0 (-82.5 to -59.8)	-15.0 (-26.1 to -1.1)
	15-49 years	31 (22 to 43)	8.2 (5.7 to 11.3)	37 (25 to 52)	5.7 (3.8 to 8.0)	38 (26 to 54)	5.7 (3.8 to 8.1)	35 (24 to 50)	5.1 (3.4 to 7.2)	-38.1 (-58.5 to -9.1)	-10.3 (-17.0 to -3.5)

	50-69 years	34 (23 to 46)	58.8 (40.3 to 81.1)	90 (62 to 125)	64.5 (45.0 to 90.0)	85 (59 to 118)	61.2 (42.4 to 85.1)	77 (53 to 107)	54.0 (37.4 to 75.3)	-8.2 (-37.9 to 31.2)	-16.3 (-23.1 to -9.3)
	70+ years	45 (33 to 58)	525.8 (382.6 to 685.5)	231 (172 to 302)	583.9 (434.2 to 764.7)	249 (185 to 329)	562.8 (417.7 to 743.4)	223 (165 to 299)	492.5 (363.8 to 661.0)	-6.3 (-33.5 to 31.2)	-15.7 (-21.8 to -8.9)
**Viet Nam**	All Ages	27,000 (23,200 to 31,800)	39.5 (34.0 to 46.7)	21,400 (17,800 to 25,300)	21.8 (18.1 to 25.7)	19,900 (16,500 to 24,100)	20.1 (16.6 to 24.2)	18,200 (14,900 to 22,200)	18.2 (14.9 to 22.1)	-54.0 (-64.1 to -42.8)	-16.5 (-21.1 to -12.1)
	Under 5	17,500 (14,400 to 21,400)	186.5 (152.9 to 227.7)	3,300 (2,490 to 4,300)	39.4 (29.7 to 51.3)	2,730 (2,020 to 3,600)	33.0 (24.4 to 43.5)	2,210 (1,620 to 2,900)	27.1 (19.8 to 35.7)	-85.5 (-89.7 to -79.5)	-31.3 (-39.2 to -22.6)
	5-14 years	348 (248 to 483)	2.0 (1.5 to 2.8)	185 (134 to 257)	1.2 (0.8 to 1.6)	171 (123 to 239)	1.0 (0.8 to 1.5)	152 (108 to 213)	0.9 (0.6 to 1.3)	-55.0 (-70.1 to -33.1)	-21.0 (-30.6 to -10.3)
	15-49 years	1,040 (752 to 1,440)	3.2 (2.3 to 4.4)	1,400 (1,040 to 1,890)	2.7 (2.0 to 3.6)	1,310 (970 to 1,780)	2.5 (1.9 to 3.4)	1,210 (887 to 1,660)	2.3 (1.7 to 3.2)	-27.0 (-50.1 to 10.2)	-13.3 (-20.9 to -5.0)
	50-69 years	2,140 (1,510 to 3,050)	31.0 (22.0 to 44.3)	4,170 (3,090 to 5,430)	23.5 (17.4 to 30.6)	4,180 (3,130 to 5,470)	22.7 (16.9 to 29.6)	3,960 (2,930 to 5,270)	20.9 (15.4 to 27.8)	-32.5 (-56.8 to 4.1)	-11.2 (-19.0 to -2.6)
	70+ years	5,900 (4,380 to 8,440)	289.3 (215.0 to 413.9)	12,400 (9,900 to 15,300)	293.6 (234.8 to 362.1)	11,500 (9,210 to 14,500)	272.7 (217.6 to 341.6)	10,700 (8,510 to 13,400)	239.4 (190.5 to 298.8)	-17.3 (-40.7 to 17.5)	-18.5 (-23.2 to -13.8)
**Angola**	All Ages	17,300 (13,800 to 21,300)	168.8 (134.1 to 206.9)	13,400 (10,400 to 16,600)	43.6 (34.1 to 54.0)	12,800 (9,940 to 16,000)	40.3 (31.4 to 50.5)	12,300 (9,480 to 15,600)	37.7 (29.0 to 47.8)	-77.7 (-83.4 to -70.0)	-13.7 (-22.3 to -4.2)
	Under 5	13,800 (10,200 to 17,400)	704.4 (524.4 to 889.3)	5,750 (4,020 to 7,980)	105.1 (73.5 to 145.7)	5,030 (3,460 to 7,120)	90.4 (62.2 to 128.1)	4,530 (3,100 to 6,280)	80.4 (55.0 to 111.5)	-88.6 (-92.4 to -82.8)	-23.5 (-37.1 to -8.3)
	5-14 years	400 (295 to 526)	14.5 (10.7 to 19.0)	559 (405 to 752)	6.2 (4.5 to 8.4)	534 (373 to 726)	5.7 (4.0 to 7.8)	522 (367 to 713)	5.4 (3.8 to 7.4)	-62.5 (-74.9 to -44.1)	-12.6 (-27.5 to 3.9)
	15-49 years	756 (555 to 979)	16.2 (11.9 to 21.0)	1,620 (1,180 to 2,110)	12.0 (8.7 to 15.5)	1,640 (1,200 to 2,160)	11.7 (8.5 to 15.3)	1,680 (1,200 to 2,220)	11.4 (8.2 to 15.1)	-29.7 (-53.0 to 3.4)	-4.4 (-13.8 to 5.4)
	50-69 years	1,360 (1,030 to 1,780)	174.3 (131.7 to 228.5)	2,670 (1,980 to 3,530)	118.9 (88.2 to 157.3)	2,720 (2,000 to 3,560)	117.2 (86.0 to 153.1)	2,760 (2,000 to 3,600)	115.0 (83.7 to 150.2)	-34.0 (-56.1 to -4.6)	-3.3 (-12.6 to 7.0)
	70+ years	1,080 (841 to 1,340)	839.8 (655.6 to 1,046.3)	2,770 (2,170 to 3,450)	749.4 (587.5 to 934.8)	2,840 (2,240 to 3,560)	732.6 (578.5 to 918.1)	2,840 (2,230 to 3,550)	706.9 (555.5 to 884.5)	-15.8 (-37.8 to 14.6)	-5.7 (-15.0 to 4.0)
**Central African Republic**	All Ages	5,620 (4,290 to 7,110)	206.0 (157.1 to 260.5)	5,010 (3,730 to 6,530)	94.7 (70.6 to 123.5)	4,830 (3,590 to 6,220)	89.5 (66.6 to 115.4)	4,610 (3,480 to 5,940)	84.1 (63.5 to 108.2)	-59.2 (-69.4 to -44.1)	-11.2 (-19.4 to -2.3)
	Under 5	4,130 (2,790 to 5,570)	820.7 (553.9 to 1,105.3)	2,540 (1,700 to 3,560)	308.1 (206.2 to 431.3)	2,380 (1,580 to 3,370)	285.9 (189.8 to 404.6)	2,210 (1,420 to 3,150)	263.9 (169.4 to 375.1)	-67.8 (-77.6 to -53.2)	-14.3 (-26.6 to -0.4)
	5-14 years	121 (87 to 159)	16.8 (12.2 to 22.1)	168 (117 to 226)	11.9 (8.3 to 16.1)	166 (117 to 225)	11.6 (8.2 to 15.7)	161 (112 to 218)	11.1 (7.8 to 15.1)	-33.8 (-54.5 to -4.2)	-6.6 (-18.9 to 7.7)
	15-49 years	315 (234 to 399)	25.4 (18.8 to 32.1)	614 (411 to 863)	24.1 (16.2 to 33.9)	611 (413 to 854)	23.4 (15.8 to 32.7)	605 (412 to 845)	22.7 (15.5 to 31.7)	-10.6 (-37.0 to 24.0)	-5.9 (-14.2 to 3.1)

	50-69 years	641<br>(485 to 818)	279.8<br>(211.4 to 356.9)	1,010<br>(719 to 1,390)	225.9<br>(161.3 to 312.1)	1,010<br>(712 to 1,370)	219.7<br>(155.2 to 299.4)	1,010<br>(723 to 1,370)	213.9<br>(153.7 to 290.3)	-23.6<br>(-44.5 to 4.2)	-5.3<br>(-12.6 to 3.3)
	70+ years	413<br>(310 to 522)	1,111.5<br>(833.9 to 1,404.2)	678<br>(494 to 901)	1,066.1<br>(777.2 to 1,416.9)	659<br>(481 to 872)	1,022.0<br>(745.9 to 1,353.2)	627<br>(455 to 843)	982.9<br>(713.5 to 1,320.7)	-11.6<br>(-31.5 to 12.9)	-7.8<br>(-15.9 to 0.5)
**Congo**	All Ages	2,040<br>(1,570 to 2,540)	85.1<br>(65.4 to 105.7)	2,070<br>(1,550 to 2,650)	39.7<br>(29.8 to 51.0)	2,030<br>(1,510 to 2,620)	38.2<br>(28.5 to 49.4)	1,980<br>(1,480 to 2,560)	36.8<br>(27.5 to 47.4)	-56.8<br>(-67.3 to -43.9)	-7.4<br>(-15.6 to 1.8)
	Under 5	1,010<br>(677 to 1,370)	256.3<br>(171.5 to 347.1)	324<br>(222 to 449)	49.3<br>(33.8 to 68.4)	285<br>(189 to 399)	44.2<br>(29.4 to 61.8)	255<br>(166 to 356)	40.3<br>(26.2 to 56.2)	-84.3<br>(-88.9 to -76.8)	-18.3<br>(-34.3 to 1.0)
	5-14 years	53<br>(36 to 73)	8.0<br>(5.5 to 11.1)	59<br>(42 to 80)	4.6<br>(3.3 to 6.3)	56<br>(39 to 77)	4.4<br>(3.0 to 6.0)	53<br>(37 to 74)	4.1<br>(2.9 to 5.7)	-48.8<br>(-64.7 to -24.9)	-10.9<br>(-24.0 to 4.5)
	15-49 years	196<br>(135 to 265)	17.7<br>(12.2 to 24.0)	368<br>(244 to 542)	13.7<br>(9.1 to 20.2)	366<br>(239 to 546)	13.3<br>(8.7 to 19.8)	365<br>(239 to 538)	12.9<br>(8.5 to 19.1)	-27.1<br>(-51.6 to 14.3)	-5.6<br>(-15.3 to 5.9)
	50-69 years	398<br>(293 to 510)	199.9<br>(147.2 to 255.6)	597<br>(406 to 815)	119.8<br>(81.4 to 163.5)	619<br>(424 to 850)	117.7<br>(80.6 to 161.7)	626<br>(432 to 855)	114.2<br>(78.8 to 156.1)	-42.9<br>(-58.9 to -22.1)	-4.6<br>(-12.8 to 6.2)
	70+ years	386<br>(297 to 481)	885.5<br>(681.8 to 1,103.3)	721<br>(539 to 892)	765.7<br>(572.7 to 946.7)	701<br>(533 to 870)	748.5<br>(569.4 to 930.1)	685<br>(520 to 851)	712.8<br>(540.3 to 884.7)	-19.5<br>(-37.7 to 3.5)	-6.9<br>(-15.4 to 1.6)
**Democratic Republic of the Congo**	All Ages	53,300<br>(39,600 to 65,400)	139.7<br>(103.9 to 171.6)	45,100<br>(34,300 to 60,500)	52.5<br>(39.9 to 70.4)	43,400<br>(33,400 to 58,700)	49.3<br>(38.0 to 66.8)	41,700<br>(31,600 to 56,800)	46.3<br>(35.1 to 63.1)	-66.9<br>(-76.2 to -53.7)	-11.9<br>(-19.9 to -3.8)
	Under 5	37,900<br>(25,800 to 51,700)	520.1<br>(353.7 to 709.2)	13,600<br>(9,270 to 19,500)	100.5<br>(68.5 to 143.8)	11,900<br>(8,180 to 17,100)	87.5<br>(60.3 to 126.0)	10,200<br>(7,010 to 15,000)	75.6<br>(51.7 to 110.4)	-85.5<br>(-89.7 to -79.7)	-24.8<br>(-35.2 to -14.4)
	5-14 years	1,410<br>(938 to 2,080)	13.6<br>(9.0 to 20.0)	1,850<br>(1,240 to 2,710)	7.9<br>(5.3 to 11.5)	1,740<br>(1,190 to 2,520)	7.3<br>(5.0 to 10.5)	1,630<br>(1,120 to 2,380)	6.7<br>(4.6 to 9.7)	-50.9<br>(-64.3 to -31.8)	-15.2<br>(-27.0 to -1.4)
	15-49 years	2,690<br>(1,660 to 4,080)	16.0<br>(9.9 to 24.3)	6,020<br>(3,950 to 8,950)	14.8<br>(9.7 to 22.0)	6,070<br>(3,990 to 9,090)	14.5<br>(9.5 to 21.7)	6,130<br>(4,040 to 9,150)	14.2<br>(9.3 to 21.1)	-11.6<br>(-37.0 to 27.9)	-4.3<br>(-13.4 to 4.8)
	50-69 years	5,860<br>(3,690 to 8,880)	189.7<br>(119.3 to 287.3)	11,200<br>(7,390 to 17,000)	161.6<br>(106.6 to 245.0)	11,600<br>(7,580 to 17,600)	160.5<br>(105.0 to 244.0)	11,700<br>(7,690 to 17,700)	157.3<br>(103.4 to 237.6)	-17.1<br>(-41.6 to 15.2)	-2.7<br>(-11.6 to 6.9)
	70+ years	5,390<br>(3,470 to 7,770)	951.9<br>(613.0 to 1,372.4)	12,400<br>(8,400 to 18,000)	1,004.4<br>(680.0 to 1,454.8)	12,100<br>(8,290 to 17,500)	986.3<br>(673.6 to 1,423.5)	12,000<br>(8,130 to 17,200)	945.3<br>(642.9 to 1,359.2)	-0.7<br>(-26.3 to 34.4)	-5.9<br>(-15.5 to 3.9)
**Equatorial Guinea**	All Ages	674<br>(521 to 847)	159.4<br>(123.2 to 200.2)	421<br>(293 to 578)	29.4<br>(20.5 to 40.4)	411<br>(286 to 569)	27.9<br>(19.4 to 38.6)	403<br>(284 to 557)	26.7<br>(18.8 to 36.9)	-83.3<br>(-88.3 to -76.0)	-9.2<br>(-17.5 to -0.9)
	Under 5	466<br>(320 to 618)	566.4<br>(389.6 to 751.2)	101<br>(63 to 167)	52.9<br>(33.0 to 87.5)	89<br>(53 to 151)	47.2<br>(28.3 to 79.9)	82<br>(48 to 140)	43.6<br>(25.7 to 74.5)	-92.3<br>(-95.3 to -87.1)	-17.4<br>(-30.0 to -4.3)
	5-14 years	16<br>(11 to 22)	14.1<br>(9.8 to 18.9)	18<br>(9 to 37)	4.7<br>(2.4 to 9.7)	18<br>(9 to 39)	4.6<br>(2.4 to 10.1)	18<br>(9 to 40)	4.6<br>(2.2 to 10.1)	-67.2<br>(-84.3 to -24.2)	-1.7<br>(-21.2 to 25.3)
	15-49 years	37<br>(27 to 49)	20.2<br>(14.7 to 27.0)	70<br>(42 to 106)	9.4<br>(5.6 to 14.2)	72<br>(44 to 110)	9.3<br>(5.6 to 14.1)	74<br>(46 to 111)	9.1<br>(5.7 to 13.7)	-54.8<br>(-71.7 to -26.7)	-2.8<br>(-14.0 to 8.8)

	50-69 years	82 (61 to 105)	218.6 (161.7 to 280.5)	89 (56 to 128)	99.5 (62.8 to 144.2)	92 (60 to 134)	98.8 (64.0 to 143.2)	93 (62 to 132)	95.7 (63.7 to 136.1)	-56.2 (-72.1 to -34.4)	-3.8 (-13.9 to 7.6)
	70+ years	73 (57 to 91)	975.2 (760.7 to 1,218.2)	143 (107 to 189)	761.1 (569.5 to 1,007.3)	139 (104 to 182)	750.5 (558.1 to 980.4)	137 (100 to 177)	713.1 (523.6 to 922.6)	-26.9 (-47.7 to 2.0)	-6.3 (-15.4 to 3.9)
**Gabon**	All Ages	828 (656 to 1,000)	84.2 (66.7 to 102.1)	745 (529 to 984)	42.2 (29.9 to 55.8)	716 (506 to 947)	40.0 (28.2 to 52.9)	688 (491 to 925)	37.9 (27.0 to 51.0)	-55.0 (-66.2 to -41.1)	-10.2 (-17.6 to -2.5)
	Under 5	337 (239 to 457)	215.9 (153.0 to 292.9)	117 (75 to 179)	53.5 (34.3 to 81.8)	98 (61 to 150)	45.5 (28.3 to 69.1)	85 (52 to 133)	39.9 (24.5 to 62.2)	-81.5 (-88.2 to -72.4)	-25.3 (-36.0 to -13.1)
	5-14 years	17 (11 to 24)	6.7 (4.5 to 9.4)	18 (11 to 27)	4.2 (2.6 to 6.5)	17 (10 to 26)	4.0 (2.4 to 6.1)	16 (10 to 24)	3.7 (2.3 to 5.6)	-44.4 (-62.7 to -17.1)	-12.1 (-29.1 to 2.8)
	15-49 years	61 (44 to 82)	13.6 (9.8 to 18.3)	93 (58 to 140)	10.5 (6.5 to 15.7)	91 (56 to 140)	10.0 (6.2 to 15.5)	90 (55 to 140)	9.7 (6.0 to 15.1)	-28.7 (-53.1 to 7.5)	-7.3 (-17.4 to 3.8)
	50-69 years	159 (117 to 209)	158.3 (116.4 to 207.3)	214 (139 to 304)	108.1 (70.2 to 154.0)	219 (142 to 310)	106.8 (69.3 to 151.2)	214 (140 to 309)	102.4 (67.0 to 147.7)	-35.3 (-53.9 to -9.5)	-5.2 (-13.6 to 4.3)
	70+ years	254 (194 to 317)	900.7 (686.7 to 1,121.8)	304 (224 to 387)	763.7 (562.8 to 975.0)	290 (214 to 373)	750.4 (552.5 to 964.1)	283 (207 to 368)	699.1 (511.1 to 908.4)	-22.4 (-40.0 to 0.3)	-8.5 (-16.6 to -0.2)
**Burundi**	All Ages	8,370 (6,450 to 10,200)	150.8 (116.1 to 183.0)	5,960 (4,760 to 7,380)	48.3 (38.6 to 59.8)	5,290 (4,250 to 6,520)	41.3 (33.2 to 50.9)	5,360 (4,320 to 6,650)	40.5 (32.7 to 50.3)	-73.1 (-78.9 to -65.9)	-16.1 (-21.8 to -9.7)
	Under 5	5,260 (3,570 to 6,880)	489.5 (332.0 to 640.1)	2,160 (1,400 to 3,180)	104.5 (67.6 to 153.4)	1,740 (1,100 to 2,610)	82.3 (51.7 to 123.0)	1,680 (1,040 to 2,500)	77.6 (48.4 to 115.9)	-84.1 (-89.1 to -77.5)	-25.7 (-33.5 to -17.0)
	5-14 years	391 (262 to 519)	25.3 (16.9 to 33.6)	416 (310 to 551)	12.0 (8.9 to 15.9)	367 (271 to 491)	10.2 (7.6 to 13.7)	356 (262 to 476)	9.6 (7.1 to 12.9)	-61.9 (-72.4 to -45.6)	-19.6 (-27.7 to -10.8)
	15-49 years	608 (449 to 819)	25.1 (18.5 to 33.8)	828 (611 to 1,080)	14.4 (10.6 to 18.9)	782 (575 to 1,020)	13.0 (9.6 to 17.0)	826 (609 to 1,080)	13.2 (9.7 to 17.3)	-47.3 (-62.9 to -28.0)	-8.3 (-15.9 to -0.1)
	50-69 years	880 (655 to 1,140)	223.5 (166.4 to 290.9)	1,110 (825 to 1,480)	124.3 (92.7 to 166.7)	1,040 (767 to 1,390)	113.5 (83.8 to 151.9)	1,090 (806 to 1,440)	115.4 (85.5 to 152.8)	-48.4 (-63.6 to -26.6)	-7.2 (-14.7 to 1.2)
	70+ years	1,230 (963 to 1,550)	1,087.7 (850.7 to 1,368.2)	1,450 (1,110 to 1,880)	879.6 (674.7 to 1,146.3)	1,360 (1,040 to 1,760)	777.3 (594.1 to 1,007.2)	1,420 (1,080 to 1,850)	785.2 (597.2 to 1,026.9)	-27.8 (-46.7 to -3.0)	-10.7 (-17.6 to -2.7)
**Comoros**	All Ages	630 (489 to 777)	136.1 (105.7 to 168.0)	403 (323 to 499)	55.4 (44.4 to 68.7)	376 (298 to 465)	51.0 (40.5 to 63.2)	379 (298 to 473)	50.9 (40.1 to 63.6)	-62.6 (-71.3 to -48.4)	-8.2 (-14.6 to -1.8)
	Under 5	456 (343 to 583)	553.8 (416.2 to 707.5)	115 (86 to 153)	139.6 (103.8 to 185.3)	100 (74 to 132)	121.8 (90.5 to 160.7)	96 (71 to 127)	117.7 (87.9 to 156.2)	-78.7 (-85.3 to -68.6)	-15.7 (-26.5 to -3.1)
	5-14 years	21 (11 to 30)	16.3 (8.6 to 23.0)	14 (10 to 19)	8.7 (6.2 to 11.7)	13 (9 to 17)	8.3 (6.0 to 11.0)	13 (9 to 17)	8.1 (5.8 to 10.8)	-50.4 (-66.7 to 2.7)	-7.1 (-18.3 to 5.4)
	15-49 years	34 (18 to 48)	16.7 (8.9 to 23.6)	44 (30 to 59)	11.4 (8.0 to 15.6)	43 (30 to 59)	11.2 (7.8 to 15.3)	44 (31 to 59)	11.2 (7.8 to 15.1)	-32.9 (-57.2 to 35.9)	-2.3 (-10.0 to 5.6)

	50-69 years	54 (39 to 71)	146.5 (104.8 to 191.5)	75 (51 to 102)	89.8 (61.0 to 122.2)	75 (51 to 102)	86.1 (59.0 to 117.9)	78 (53 to 107)	86.4 (59.3 to 118.8)	-41.0 (-60.7 to -14.8)	-3.7 (-11.6 to 5.5)
	70+ years	64 (48 to 81)	785.7 (589.2 to 993.9)	156 (115 to 205)	700.8 (516.1 to 920.8)	145 (107 to 193)	649.5 (481.5 to 864.1)	149 (108 to 198)	656.5 (475.5 to 874.3)	-16.4 (-37.5 to 12.4)	-6.3 (-13.7 to 2.0)
**Djibouti**	All Ages	304 (236 to 373)	73.3 (57.0 to 90.0)	482 (356 to 653)	40.3 (29.8 to 54.6)	434 (321 to 590)	35.4 (26.2 to 48.1)	438 (320 to 595)	34.8 (25.4 to 47.3)	-52.6 (-66.0 to -33.1)	-13.7 (-21.9 to -4.6)
	Under 5	195 (140 to 257)	301.2 (216.2 to 397.7)	129 (84 to 186)	89.3 (58.3 to 128.9)	100 (67 to 142)	69.0 (46.2 to 97.5)	92 (60 to 133)	62.8 (41.3 to 91.5)	-79.1 (-86.4 to -69.6)	-29.6 (-46.3 to -8.2)
	5-14 years	16 (11 to 21)	14.2 (10.4 to 18.8)	20 (14 to 29)	8.0 (5.6 to 11.5)	18 (13 to 26)	7.0 (4.9 to 10.0)	18 (12 to 26)	6.7 (4.5 to 9.8)	-52.9 (-68.8 to -30.2)	-16.3 (-32.7 to 3.2)
	15-49 years	27 (19 to 36)	12.9 (9.1 to 17.5)	75 (47 to 114)	11.4 (7.2 to 17.3)	70 (45 to 107)	10.3 (6.7 to 15.9)	72 (47 to 109)	10.4 (6.8 to 15.8)	-19.2 (-49.6 to 33.8)	-8.5 (-17.8 to 1.7)
	50-69 years	35 (25 to 47)	123.9 (89.1 to 169.6)	117 (76 to 172)	97.6 (63.4 to 142.9)	113 (74 to 168)	89.6 (58.8 to 133.1)	119 (79 to 174)	89.8 (59.5 to 130.7)	-27.5 (-52.6 to 11.1)	-8.1 (-17.1 to 1.8)
	70+ years	32 (25 to 41)	755.8 (591.8 to 969.0)	140 (103 to 190)	690.3 (508.3 to 933.0)	132 (97 to 180)	618.5 (454.3 to 842.9)	137 (101 to 186)	625.1 (460.8 to 850.6)	-17.3 (-40.0 to 14.5)	-9.4 (-17.6 to 0.0)
**Eritrea**	All Ages	4,890 (3,660 to 6,200)	143.6 (107.6 to 182.1)	4,310 (2,770 to 6,500)	68.2 (43.8 to 103.0)	3,890 (2,490 to 5,850)	60.2 (38.5 to 90.6)	3,970 (2,510 to 5,910)	60.2 (38.0 to 89.6)	-58.1 (-71.1 to -41.8)	-11.7 (-18.0 to -4.7)
	Under 5	3,140 (2,330 to 4,000)	503.7 (373.6 to 641.3)	1,350 (945 to 1,860)	150.5 (105.1 to 207.3)	1,100 (762 to 1,520)	121.4 (83.9 to 166.9)	1,090 (744 to 1,500)	118.3 (81.1 to 163.2)	-76.5 (-84.4 to -64.2)	-21.4 (-31.4 to -9.5)
	5-14 years	228 (125 to 367)	23.5 (12.9 to 37.9)	198 (111 to 336)	12.8 (7.2 to 21.7)	181 (102 to 307)	11.5 (6.4 to 19.4)	183 (104 to 303)	11.4 (6.5 to 18.9)	-51.5 (-66.2 to -29.3)	-10.6 (-19.1 to -0.5)
	15-49 years	515 (296 to 829)	33.4 (19.2 to 53.8)	792 (390 to 1,400)	24.3 (11.9 to 43.0)	743 (371 to 1,310)	22.2 (11.1 to 39.1)	777 (388 to 1,360)	22.7 (11.3 to 39.8)	-32.1 (-53.1 to -3.8)	-6.4 (-14.4 to 2.1)
	50-69 years	682 (405 to 1,060)	279.0 (165.7 to 433.3)	955 (532 to 1,590)	184.8 (103.0 to 308.5)	918 (510 to 1,520)	170.7 (94.9 to 281.9)	957 (538 to 1,570)	171.4 (96.3 to 280.9)	-38.5 (-53.9 to -17.9)	-7.3 (-13.3 to 0.2)
	70+ years	325 (197 to 506)	1,155.6 (697.9 to 1,796.5)	1,010 (624 to 1,550)	1,155.0 (713.5 to 1,777.8)	941 (586 to 1,440)	1,051.6 (654.6 to 1,611.1)	971 (604 to 1,490)	1,057.6 (657.4 to 1,620.3)	-8.5 (-27.7 to 16.7)	-8.4 (-14.3 to -1.2)
**Ethiopia**	All Ages	96,000 (76,900 to 117,000)	189.9 (152.1 to 232.0)	42,900 (37,900 to 48,800)	41.0 (36.3 to 46.7)	38,100 (33,300 to 43,300)	35.7 (31.2 to 40.5)	38,200 (33,500 to 43,800)	35.1 (30.8 to 40.2)	-81.5 (-85.5 to -76.5)	-14.5 (-24.5 to -2.1)
	Under 5	68,500 (50,600 to 88,500)	710.4 (524.7 to 917.9)	17,300 (13,400 to 21,800)	111.0 (86.2 to 140.0)	13,900 (10,800 to 17,800)	88.5 (68.8 to 113.3)	13,500 (9,970 to 17,300)	84.5 (62.4 to 108.5)	-88.1 (-91.5 to -83.0)	-23.9 (-34.8 to -11.4)
	5-14 years	3,280 (2,290 to 4,310)	22.3 (15.5 to 29.3)	1,970 (1,570 to 2,460)	7.1 (5.6 to 8.8)	1,800 (1,410 to 2,220)	6.4 (5.0 to 7.9)	1,780 (1,390 to 2,230)	6.3 (4.9 to 7.9)	-71.8 (-80.1 to -57.5)	-11.3 (-27.3 to 8.4)
	15-49 years	5,750 (4,550 to 6,920)	26.5 (21.0 to 31.9)	4,390 (3,660 to 5,140)	8.4 (7.0 to 9.9)	4,190 (3,390 to 5,070)	7.8 (6.3 to 9.5)	4,380 (3,500 to 5,320)	8.0 (6.4 to 9.7)	-70.0 (-77.4 to -59.3)	-5.6 (-25.4 to 19.0)

	50-69 years	9,550 (7,500 to 11,800)	256.6 (201.5 to 317.3)	5,580 (4,710 to 6,640)	78.8 (66.4 to 93.8)	5,490 (4,450 to 6,720)	74.4 (60.4 to 91.2)	5,660 (4,580 to 6,940)	74.7 (60.4 to 91.5)	-70.9 (-78.3 to -59.4)	-5.1 (-23.6 to 19.3)
	70+ years	8,950 (6,930 to 11,400)	1,158.4 (896.6 to 1,471.9)	13,700 (11,800 to 15,600)	706.4 (608.0 to 806.3)	12,700 (10,700 to 14,800)	656.5 (556.0 to 764.0)	12,900 (10,800 to 15,300)	654.4 (549.1 to 774.0)	-43.5 (-58.2 to -23.3)	-7.4 (-19.4 to 7.4)
**Kenya**	All Ages	19,900 (17,200 to 24,700)	85.9 (74.2 to 106.8)	20,800 (17,000 to 25,000)	42.8 (35.0 to 51.5)	18,100 (14,300 to 22,000)	36.7 (29.0 to 44.6)	18,000 (14,500 to 22,200)	35.9 (28.9 to 44.3)	-58.1 (-68.3 to -46.9)	-16.0 (-29.2 to -0.7)
	Under 5	13,700 (11,200 to 18,300)	319.1 (260.7 to 426.9)	5,470 (4,250 to 6,900)	89.1 (69.2 to 112.4)	4,040 (3,120 to 5,120)	66.8 (51.6 to 84.7)	3,750 (2,880 to 4,800)	63.1 (48.4 to 80.7)	-80.2 (-86.3 to -73.5)	-29.2 (-36.7 to -21.8)
	5-14 years	611 (465 to 773)	8.9 (6.8 to 11.2)	714 (562 to 917)	5.6 (4.4 to 7.2)	622 (473 to 811)	4.9 (3.7 to 6.4)	604 (465 to 780)	4.8 (3.7 to 6.1)	-46.5 (-58.9 to -29.4)	-15.6 (-28.3 to 1.1)
	15-49 years	1,010 (757 to 1,280)	9.9 (7.5 to 12.6)	2,640 (2,010 to 3,380)	10.6 (8.1 to 13.6)	2,390 (1,730 to 3,090)	9.4 (6.8 to 12.1)	2,430 (1,850 to 3,140)	9.3 (7.1 to 12.0)	-6.3 (-27.4 to 23.8)	-12.4 (-31.3 to 12.4)
	50-69 years	1,550 (1,130 to 2,020)	107.0 (77.7 to 138.9)	4,410 (3,310 to 5,540)	109.5 (82.2 to 137.6)	4,160 (3,070 to 5,290)	99.6 (73.5 to 126.5)	4,260 (3,210 to 5,460)	98.7 (74.3 to 126.3)	-7.8 (-28.1 to 20.4)	-9.9 (-28.7 to 14.5)
	70+ years	3,000 (2,270 to 3,820)	811.1 (611.5 to 1,032.2)	7,580 (5,760 to 9,630)	858.0 (652.3 to 1,090.5)	6,890 (5,060 to 8,770)	757.6 (555.9 to 964.0)	6,940 (5,210 to 8,860)	744.2 (558.4 to 949.2)	-8.2 (-26.2 to 15.8)	-13.3 (-29.9 to 7.2)
**Madagascar**	All Ages	17,200 (15,300 to 19,200)	144.2 (128.7 to 161.2)	16,200 (13,200 to 19,600)	59.3 (48.2 to 71.6)	14,100 (11,400 to 16,900)	50.4 (40.8 to 60.4)	13,600 (11,000 to 16,400)	47.6 (38.5 to 57.5)	-67.0 (-73.6 to -59.4)	-19.8 (-26.9 to -11.9)
	Under 5	11,700 (9,920 to 13,500)	542.1 (460.3 to 626.6)	7,300 (5,420 to 9,700)	177.4 (131.8 to 235.7)	5,860 (4,260 to 7,760)	142.6 (103.6 to 189.1)	5,320 (3,820 to 7,080)	130.1 (93.3 to 173.3)	-76.0 (-82.8 to -67.3)	-26.7 (-36.5 to -15.6)
	5-14 years	871 (673 to 1,070)	26.4 (20.4 to 32.5)	1,100 (819 to 1,400)	14.8 (11.1 to 18.9)	993 (746 to 1,270)	13.2 (9.9 to 16.8)	950 (710 to 1,230)	12.4 (9.3 to 16.1)	-52.9 (-65.6 to -36.8)	-16.1 (-28.1 to -2.5)
	15-49 years	1,240 (1,050 to 1,430)	23.5 (19.8 to 26.9)	2,550 (1,930 to 3,300)	19.2 (14.6 to 24.9)	2,360 (1,800 to 3,020)	17.2 (13.1 to 22.0)	2,400 (1,800 to 3,110)	17.0 (12.7 to 22.0)	-27.5 (-46.9 to -2.2)	-11.6 (-19.6 to -2.6)
	50-69 years	1,550 (1,330 to 1,820)	167.5 (144.0 to 196.4)	2,720 (1,960 to 3,580)	124.8 (90.3 to 164.4)	2,580 (1,870 to 3,370)	114.1 (82.7 to 148.9)	2,640 (1,920 to 3,450)	112.9 (82.1 to 147.5)	-32.6 (-53.5 to -8.0)	-9.5 (-17.7 to 0.7)
	70+ years	1,810 (1,560 to 2,100)	844.0 (725.9 to 979.2)	2,550 (1,940 to 3,220)	732.4 (557.4 to 926.2)	2,280 (1,720 to 2,870)	642.8 (484.8 to 807.1)	2,280 (1,700 to 2,890)	626.6 (467.2 to 793.9)	-25.8 (-45.2 to -2.3)	-14.4 (-22.2 to -4.7)
**Malawi**	All Ages	16,600 (12,700 to 21,000)	169.2 (129.6 to 214.3)	9,770 (7,900 to 11,800)	52.6 (42.5 to 63.7)	8,390 (6,730 to 10,200)	44.1 (35.4 to 53.6)	8,220 (6,580 to 10,000)	42.3 (33.8 to 51.4)	-75.0 (-80.2 to -67.5)	-19.7 (-25.7 to -13.0)
	Under 5	12,800 (9,190 to 17,400)	676.4 (483.8 to 915.0)	3,990 (2,880 to 5,260)	146.2 (105.2 to 192.6)	3,110 (2,140 to 4,170)	114.1 (78.4 to 152.9)	2,900 (1,950 to 3,880)	106.3 (71.6 to 142.6)	-84.3 (-88.5 to -77.9)	-27.2 (-36.7 to -18.3)
	5-14 years	560 (385 to 742)	21.1 (14.5 to 28.0)	473 (339 to 635)	8.9 (6.4 to 12.0)	401 (283 to 541)	7.5 (5.3 to 10.1)	386 (270 to 518)	7.2 (5.0 to 9.6)	-66.1 (-76.6 to -49.8)	-19.9 (-31.7 to -7.6)
	15-49 years	709 (546 to 874)	16.1 (12.4 to 19.9)	1,260 (990 to 1,570)	14.0 (11.0 to 17.4)	1,160 (906 to 1,460)	12.4 (9.7 to 15.6)	1,190 (923 to 1,500)	12.3 (9.5 to 15.4)	-23.6 (-43.7 to 3.7)	-12.1 (-20.6 to -3.3)

	50-69 years	1,120 (869 to 1,380)	159.7 (124.4 to 197.2)	1,630 (1,280 to 2,050)	131.3 (102.9 to 164.9)	1,520 (1,200 to 1,890)	118.0 (93.1 to 147.2)	1,560 (1,220 to 1,950)	116.9 (91.6 to 146.1)	-26.8 (-46.4 to 2.0)	-10.9 (-18.2 to -2.5)
	70+ years	1,360 (1,060 to 1,680)	877.3 (684.8 to 1,080.4)	2,410 (1,930 to 2,920)	808.9 (649.3 to 979.5)	2,200 (1,760 to 2,660)	709.8 (566.9 to 855.6)	2,190 (1,760 to 2,660)	703.5 (564.8 to 854.6)	-19.8 (-38.7 to 7.0)	-13.0 (-20.1 to -4.2)
**Mozambique**	All Ages	17,800 (13,700 to 24,700)	133.4 (102.7 to 185.2)	14,300 (11,300 to 17,400)	48.4 (38.3 to 59.1)	12,400 (9,880 to 15,000)	40.9 (32.6 to 49.7)	12,100 (9,590 to 14,900)	39.1 (30.9 to 48.0)	-70.7 (-78.0 to -61.7)	-19.3 (-25.2 to -12.5)
	Under 5	13,300 (9,450 to 19,900)	553.0 (392.2 to 826.6)	5,600 (3,990 to 7,680)	110.6 (78.7 to 151.6)	4,480 (3,160 to 6,200)	87.4 (61.7 to 121.1)	4,220 (3,010 to 5,850)	81.5 (58.2 to 112.9)	-85.3 (-90.2 to -77.4)	-26.3 (-35.0 to -15.9)
	5-14 years	570 (401 to 775)	15.0 (10.6 to 20.4)	757 (518 to 1,050)	8.7 (6.0 to 12.1)	655 (435 to 920)	7.4 (4.9 to 10.4)	649 (428 to 917)	7.1 (4.7 to 10.1)	-52.4 (-68.9 to -25.9)	-18.2 (-29.0 to -7.4)
	15-49 years	731 (568 to 922)	12.6 (9.8 to 15.9)	1,960 (1,400 to 2,650)	14.7 (10.5 to 19.8)	1,800 (1,270 to 2,450)	13.1 (9.2 to 17.7)	1,840 (1,280 to 2,510)	12.9 (9.0 to 17.6)	2.3 (-30.5 to 42.9)	-12.2 (-21.7 to -2.2)
	50-69 years	1,440 (1,140 to 1,750)	127.3 (100.4 to 154.6)	2,740 (1,980 to 3,570)	137.3 (99.5 to 179.0)	2,560 (1,850 to 3,330)	124.4 (89.8 to 161.7)	2,570 (1,850 to 3,360)	121.6 (87.4 to 158.9)	-4.5 (-32.5 to 31.9)	-11.4 (-18.8 to -3.0)
	70+ years	1,760 (1,460 to 2,100)	722.8 (600.1 to 864.3)	3,220 (2,480 to 3,990)	773.3 (595.0 to 957.4)	2,890 (2,210 to 3,540)	682.7 (522.2 to 836.5)	2,860 (2,180 to 3,560)	664.1 (506.0 to 829.3)	-8.1 (-32.3 to 21.3)	-14.1 (-21.5 to -5.5)
**Rwanda**	All Ages	13,100 (10,000 to 16,400)	182.4 (139.5 to 227.8)	5,200 (4,020 to 6,600)	40.8 (31.6 to 51.9)	4,620 (3,600 to 5,810)	35.5 (27.7 to 44.7)	4,660 (3,590 to 5,900)	35.1 (27.1 to 44.5)	-80.7 (-85.9 to -73.8)	-13.9 (-21.4 to -5.7)
	Under 5	9,460 (6,740 to 12,400)	701.9 (500.5 to 917.4)	1,810 (1,300 to 2,390)	104.8 (75.0 to 138.3)	1,470 (1,060 to 1,900)	84.5 (61.2 to 109.0)	1,380 (1,000 to 1,810)	79.2 (57.3 to 103.3)	-88.7 (-92.5 to -83.3)	-24.4 (-36.7 to -11.9)
	5-14 years	536 (371 to 745)	26.2 (18.1 to 36.4)	222 (160 to 312)	7.0 (5.1 to 9.8)	197 (143 to 271)	6.2 (4.5 to 8.5)	191 (139 to 260)	5.9 (4.3 to 8.1)	-77.3 (-84.4 to -66.5)	-15.4 (-25.7 to -4.0)
	15-49 years	824 (616 to 1,060)	26.1 (19.5 to 33.7)	634 (441 to 881)	9.8 (6.8 to 13.6)	591 (410 to 828)	8.9 (6.2 to 12.4)	617 (424 to 861)	9.0 (6.2 to 12.6)	-65.5 (-76.6 to -50.4)	-8.2 (-17.0 to 1.1)
	50-69 years	1,180 (910 to 1,490)	222.2 (171.6 to 280.3)	1,010 (699 to 1,400)	88.5 (61.1 to 122.5)	966 (664 to 1,340)	82.1 (56.4 to 114.1)	999 (679 to 1,380)	83.2 (56.5 to 114.7)	-62.6 (-74.6 to -47.7)	-6.1 (-15.8 to 3.8)
	70+ years	1,110 (883 to 1,370)	1,006.8 (800.1 to 1,237.7)	1,520 (1,080 to 2,040)	668.2 (474.6 to 897.5)	1,400 (986 to 1,890)	592.9 (418.1 to 799.6)	1,470 (1,030 to 1,990)	596.2 (417.4 to 804.7)	-40.8 (-56.2 to -19.6)	-10.8 (-19.7 to -1.0)
**Somalia**	All Ages	13,000 (9,600 to 16,900)	164.0 (121.0 to 213.3)	16,400 (12,000 to 21,400)	81.8 (60.1 to 106.9)	14,600 (10,900 to 19,100)	70.4 (52.5 to 91.8)	14,500 (10,600 to 19,000)	66.9 (49.3 to 88.0)	-59.2 (-70.2 to -45.5)	-18.2 (-27.4 to -8.2)
	Under 5	9,960 (6,710 to 13,700)	644.8 (434.4 to 885.5)	9,970 (6,670 to 14,200)	258.7 (173.1 to 368.7)	8,520 (5,770 to 12,100)	213.6 (144.6 to 303.6)	8,280 (5,430 to 12,000)	200.6 (131.5 to 290.4)	-68.9 (-79.6 to -55.9)	-22.5 (-36.8 to -8.5)
	5-14 years	348 (225 to 499)	14.8 (9.6 to 21.2)	662 (416 to 971)	11.5 (7.2 to 16.8)	616 (387 to 915)	10.3 (6.5 to 15.3)	582 (357 to 856)	9.4 (5.8 to 13.8)	-36.6 (-57.9 to -6.5)	-18.2 (-33.6 to -2.6)
	15-49 years	873 (621 to 1,210)	25.0 (17.8 to 34.5)	1,800 (1,220 to 2,570)	19.8 (13.4 to 28.2)	1,780 (1,220 to 2,530)	18.7 (12.8 to 26.5)	1,870 (1,300 to 2,640)	18.8 (13.0 to 26.4)	-24.8 (-48.8 to 12.4)	-5.1 (-13.0 to 3.7)

	50-69 years	1,040 (739 to 1,380)	217.8 (154.1 to 289.0)	2,200 (1,600 to 2,910)	200.7 (146.3 to 265.9)	2,090 (1,520 to 2,780)	188.7 (137.3 to 250.7)	2,100 (1,530 to 2,790)	186.4 (136.2 to 248.3)	-14.4 (-39.6 to 21.2)	-7.1 (-14.0 to 0.4)
	70+ years	789 (604 to 1,010)	1,123.8 (860.0 to 1,434.3)	1,740 (1,300 to 2,280)	960.3 (721.5 to 1,263.0)	1,630 (1,230 to 2,150)	868.7 (655.2 to 1,142.1)	1,630 (1,240 to 2,140)	856.9 (648.9 to 1,124.4)	-23.8 (-41.9 to 1.5)	-10.8 (-18.2 to -2.7)
**South Sudan**	All Ages	9,380 (7,500 to 11,900)	159.5 (127.5 to 203.3)	8,630 (6,360 to 12,100)	94.8 (69.8 to 133.1)	7,680 (5,700 to 11,000)	81.6 (60.5 to 116.5)	7,740 (5,690 to 10,900)	80.0 (58.8 to 113.1)	-49.9 (-61.7 to -34.6)	-15.6 (-23.0 to -7.8)
	Under 5	6,940 (5,200 to 9,510)	682.4 (511.3 to 934.2)	5,370 (3,670 to 8,430)	372.1 (254.8 to 584.5)	4,550 (3,110 to 7,460)	302.6 (207.0 to 496.3)	4,570 (3,110 to 7,400)	292.5 (199.2 to 473.6)	-57.1 (-69.0 to -40.1)	-21.4 (-30.4 to -11.5)
	5-14 years	230 (164 to 297)	14.3 (10.2 to 18.5)	424 (299 to 587)	15.8 (11.1 to 21.8)	398 (277 to 540)	14.6 (10.2 to 19.9)	386 (275 to 523)	14.1 (10.1 to 19.1)	-1.2 (-31.8 to 42.8)	-10.4 (-22.1 to 1.7)
	15-49 years	384 (274 to 510)	14.2 (10.1 to 18.9)	612 (421 to 864)	14.9 (10.3 to 21.1)	610 (430 to 860)	14.2 (10.0 to 20.1)	640 (452 to 896)	14.4 (10.2 to 20.1)	1.1 (-30.3 to 52.1)	-3.6 (-12.3 to 6.5)
	50-69 years	733 (535 to 979)	171.4 (125.1 to 228.9)	942 (664 to 1,330)	127.7 (90.0 to 180.1)	941 (668 to 1,330)	123.1 (87.4 to 174.3)	987 (696 to 1,370)	125.0 (88.2 to 173.3)	-27.1 (-49.7 to 8.9)	-2.1 (-10.8 to 7.5)
	70+ years	1,080 (843 to 1,390)	863.4 (671.1 to 1,107.6)	1,280 (969 to 1,680)	906.6 (684.4 to 1,182.8)	1,180 (885 to 1,540)	833.6 (626.1 to 1,087.7)	1,160 (857 to 1,520)	829.4 (615.3 to 1,093.7)	-3.9 (-29.2 to 28.3)	-8.5 (-15.5 to -0.8)
**Uganda**	All Ages	16,000 (12,300 to 22,600)	92.8 (71.3 to 130.7)	15,300 (12,100 to 18,700)	37.2 (29.4 to 45.3)	13,900 (10,900 to 17,000)	32.8 (25.9 to 40.1)	13,800 (10,800 to 17,000)	31.8 (24.9 to 39.3)	-65.7 (-74.8 to -53.9)	-14.4 (-22.2 to -4.0)
	Under 5	11,200 (7,610 to 17,100)	312.3 (212.0 to 475.9)	5,970 (3,910 to 8,420)	82.7 (54.1 to 116.6)	5,100 (3,340 to 7,280)	70.0 (45.9 to 100.1)	4,840 (3,100 to 6,990)	66.1 (42.3 to 95.6)	-78.8 (-85.7 to -69.3)	-20.0 (-32.1 to -4.9)
	5-14 years	530 (350 to 797)	11.0 (7.3 to 16.5)	950 (697 to 1,250)	7.9 (5.8 to 10.4)	934 (695 to 1,200)	7.6 (5.7 to 9.8)	917 (673 to 1,200)	7.3 (5.4 to 9.6)	-33.3 (-55.6 to 4.6)	-7.1 (-20.5 to 9.3)
	15-49 years	780 (533 to 1,080)	10.5 (7.2 to 14.5)	1,920 (1,360 to 2,530)	10.2 (7.2 to 13.4)	1,790 (1,280 to 2,390)	9.2 (6.6 to 12.3)	1,850 (1,310 to 2,460)	9.2 (6.5 to 12.2)	-12.2 (-43.8 to 37.1)	-9.3 (-18.8 to 1.9)
	50-69 years	1,380 (1,000 to 1,870)	120.3 (87.2 to 162.9)	2,300 (1,660 to 3,060)	90.3 (65.0 to 119.7)	2,260 (1,640 to 3,000)	84.3 (60.9 to 111.7)	2,320 (1,640 to 3,150)	83.7 (59.2 to 113.4)	-30.5 (-54.0 to 3.1)	-7.3 (-17.0 to 3.2)
	70+ years	2,140 (1,640 to 2,750)	731.3 (561.0 to 939.3)	4,210 (3,350 to 5,320)	711.2 (565.4 to 899.1)	3,810 (3,000 to 4,780)	650.7 (512.8 to 816.6)	3,860 (3,020 to 4,930)	641.2 (501.8 to 819.9)	-12.3 (-36.6 to 21.6)	-9.8 (-18.6 to 1.4)
**United Republic of Tanzania**	All Ages	44,300 (36,200 to 53,800)	171.5 (140.2 to 208.4)	28,400 (23,300 to 33,700)	50.8 (41.7 to 60.4)	24,400 (20,300 to 29,000)	42.7 (35.5 to 50.7)	24,400 (20,200 to 29,100)	41.7 (34.6 to 49.8)	-75.7 (-80.7 to -69.1)	-17.9 (-24.6 to -10.0)
	Under 5	33,500 (25,800 to 42,300)	697.1 (536.2 to 879.8)	13,400 (10,100 to 17,400)	152.7 (115.1 to 197.7)	10,500 (7,600 to 13,900)	119.4 (86.2 to 158.2)	10,100 (7,220 to 13,400)	113.8 (81.6 to 152.0)	-83.7 (-88.0 to -77.4)	-25.5 (-34.7 to -14.9)
	5-14 years	1,400 (1,040 to 1,790)	19.2 (14.4 to 24.6)	1,390 (1,050 to 1,810)	9.3 (7.0 to 12.1)	1,240 (925 to 1,620)	8.1 (6.1 to 10.6)	1,240 (949 to 1,640)	8.0 (6.1 to 10.5)	-58.3 (-70.2 to -40.6)	-13.8 (-25.4 to -1.5)
	15-49 years	1,680 (1,330 to 2,030)	14.8 (11.8 to 18.0)	2,610 (1,920 to 3,370)	9.8 (7.2 to 12.7)	2,430 (1,800 to 3,140)	8.9 (6.6 to 11.4)	2,540 (1,890 to 3,290)	9.0 (6.7 to 11.7)	-39.4 (-57.2 to -14.9)	-8.1 (-17.2 to 2.5)

	50-69 years	3,060 (2,520 to 3,750)	155.8 (128.0 to 190.6)	3,740 (2,780 to 4,880)	85.1 (63.3 to 111.0)	3,610 (2,680 to 4,740)	78.6 (58.6 to 103.4)	3,750 (2,810 to 4,900)	79.2 (59.3 to 103.3)	-49.2 (-64.6 to -30.0)	-7.0 (-15.7 to 2.6)
	70+ years	4,660 (3,850 to 5,510)	957.4 (790.6 to 1,131.7)	7,240 (5,950 to 8,940)	668.8 (549.1 to 825.3)	6,590 (5,380 to 8,210)	606.0 (494.7 to 754.9)	6,790 (5,530 to 8,500)	611.2 (497.7 to 765.0)	-36.2 (-51.2 to -15.7)	-8.6 (-16.2 to -0.3)
**Zambia**	All Ages	12,500 (10,200 to 15,700)	157.8 (128.3 to 197.5)	8,330 (6,470 to 10,500)	45.0 (35.0 to 56.9)	7,520 (5,890 to 9,300)	39.5 (31.0 to 48.9)	7,140 (5,520 to 8,890)	36.6 (28.3 to 45.5)	-76.8 (-83.1 to -68.7)	-18.7 (-25.7 to -11.2)
	Under 5	9,830 (7,640 to 12,800)	651.6 (506.5 to 847.3)	3,490 (2,500 to 4,660)	118.6 (84.9 to 158.2)	2,880 (2,030 to 3,860)	98.2 (69.0 to 131.3)	2,540 (1,780 to 3,430)	86.8 (60.8 to 117.4)	-86.7 (-90.9 to -80.8)	-26.8 (-36.2 to -16.6)
	5-14 years	310 (229 to 403)	13.8 (10.2 to 17.9)	434 (311 to 586)	8.5 (6.1 to 11.5)	403 (290 to 541)	7.7 (5.6 to 10.3)	387 (280 to 516)	7.2 (5.2 to 9.7)	-47.5 (-63.1 to -24.4)	-14.8 (-25.1 to -3.7)
	15-49 years	587 (471 to 706)	16.6 (13.3 to 20.0)	1,250 (872 to 1,680)	13.9 (9.7 to 18.7)	1,190 (841 to 1,600)	12.8 (9.0 to 17.2)	1,220 (847 to 1,660)	12.6 (8.8 to 17.2)	-24.2 (-50.4 to 8.3)	-9.2 (-19.4 to 1.9)
	50-69 years	774 (625 to 977)	145.2 (117.3 to 183.2)	1,270 (928 to 1,670)	106.4 (77.6 to 140.0)	1,260 (924 to 1,630)	99.9 (73.4 to 129.7)	1,290 (944 to 1,690)	98.1 (71.9 to 128.8)	-32.4 (-53.9 to -5.5)	-7.8 (-16.8 to 2.4)
	70+ years	1,020 (836 to 1,200)	878.8 (722.1 to 1,040.2)	1,880 (1,490 to 2,380)	704.1 (556.3 to 890.5)	1,780 (1,420 to 2,220)	658.1 (527.2 to 820.2)	1,700 (1,370 to 2,120)	633.4 (510.2 to 788.2)	-27.9 (-44.7 to -7.3)	-10.0 (-19.1 to 0.8)
**Botswana**	All Ages	926 (714 to 1,170)	70.2 (54.1 to 89.0)	1,140 (881 to 1,430)	48.8 (37.8 to 61.4)	977 (739 to 1,240)	41.3 (31.3 to 52.5)	971 (727 to 1,260)	40.6 (30.4 to 52.6)	-42.2 (-54.7 to -26.4)	-16.9 (-25.0 to -8.4)
	Under 5	333 (249 to 418)	156.2 (116.9 to 196.1)	250 (175 to 348)	105.9 (74.1 to 147.6)	188 (127 to 263)	79.5 (53.9 to 111.7)	186 (125 to 263)	79.2 (53.0 to 111.9)	-49.3 (-66.0 to -24.2)	-25.3 (-36.1 to -13.0)
	5-14 years	36 (24 to 50)	9.5 (6.3 to 13.3)	32 (24 to 43)	7.1 (5.3 to 9.3)	28 (21 to 37)	6.0 (4.5 to 8.0)	27 (20 to 36)	5.8 (4.3 to 7.7)	-38.9 (-55.9 to -13.0)	-18.1 (-27.5 to -8.0)
	15-49 years	171 (105 to 255)	28.3 (17.4 to 42.4)	225 (148 to 308)	17.1 (11.2 to 23.4)	197 (125 to 274)	14.7 (9.3 to 20.5)	200 (126 to 281)	14.7 (9.3 to 20.7)	-48.1 (-67.1 to -18.1)	-14.0 (-28.3 to -0.4)
	50-69 years	202 (139 to 280)	196.1 (135.2 to 271.8)	271 (192 to 362)	104.9 (74.5 to 140.4)	250 (174 to 341)	92.8 (64.8 to 126.7)	254 (176 to 350)	91.7 (63.6 to 126.5)	-53.2 (-69.1 to -31.2)	-12.6 (-24.9 to -0.3)
	70+ years	184 (132 to 247)	802.8 (574.9 to 1,077.0)	358 (272 to 468)	636.0 (484.4 to 832.9)	314 (236 to 413)	548.6 (412.5 to 720.2)	304 (227 to 402)	519.1 (387.0 to 686.9)	-35.3 (-51.6 to -7.1)	-18.4 (-26.3 to -9.7)
**Eswatini**	All Ages	642 (547 to 745)	79.6 (67.8 to 92.4)	770 (562 to 1,010)	67.5 (49.2 to 88.7)	637 (464 to 847)	55.4 (40.3 to 73.7)	612 (442 to 827)	53.0 (38.3 to 71.6)	-33.5 (-52.9 to -9.2)	-21.5 (-27.4 to -15.5)
	Under 5	363 (290 to 451)	249.7 (199.3 to 310.0)	213 (152 to 280)	147.4 (105.0 to 193.6)	165 (115 to 223)	115.5 (80.4 to 156.3)	156 (108 to 213)	110.8 (77.2 to 151.4)	-55.6 (-69.0 to -36.8)	-24.8 (-34.8 to -14.0)
	5-14 years	23 (17 to 30)	9.7 (7.2 to 12.6)	24 (17 to 31)	8.7 (6.2 to 11.5)	20 (15 to 26)	7.4 (5.4 to 9.7)	19 (14 to 25)	7.0 (5.1 to 9.1)	-28.3 (-50.5 to 0.7)	-19.9 (-31.2 to -4.4)
	15-49 years	81 (62 to 103)	22.6 (17.4 to 28.8)	189 (115 to 270)	31.5 (19.2 to 44.8)	161 (99 to 234)	26.4 (16.3 to 38.3)	161 (98 to 234)	26.1 (15.9 to 37.9)	15.5 (-30.8 to 74.8)	-16.9 (-26.0 to -7.6)

	50-69 years	90 (67 to 115)	170.0 (127.5 to 219.2)	189 (128 to 276)	188.3 (127.9 to 275.4)	164 (113 to 239)	160.4 (110.2 to 233.9)	161 (109 to 236)	155.4 (105.0 to 227.8)	-8.6 (-39.5 to 37.6)	-17.5 (-24.1 to -9.2)
	70+ years	85 (66 to 107)	722.5 (558.5 to 905.6)	155 (118 to 203)	687.7 (521.6 to 898.7)	127 (97 to 167)	550.5 (423.2 to 727.1)	115 (88 to 154)	513.5 (390.5 to 683.9)	-28.9 (-47.7 to -4.0)	-25.3 (-31.4 to -19.0)
**Lesotho**	All Ages	1,300 (1,080 to 1,540)	84.9 (70.8 to 100.2)	1,820 (1,490 to 2,200)	97.5 (79.5 to 117.4)	1,520 (1,240 to 1,830)	81.2 (66.5 to 97.5)	1,470 (1,210 to 1,760)	78.5 (64.4 to 94.0)	-7.5 (-28.2 to 20.5)	-19.5 (-24.4 to -13.6)
	Under 5	623 (510 to 753)	253.0 (206.9 to 305.9)	483 (355 to 634)	230.2 (169.0 to 302.3)	403 (287 to 541)	194.8 (138.6 to 261.9)	404 (287 to 542)	198.5 (140.8 to 266.1)	-21.5 (-44.6 to 10.6)	-13.7 (-23.6 to -3.0)
	5-14 years	38 (28 to 51)	8.7 (6.3 to 11.8)	53 (40 to 71)	12.3 (9.1 to 16.4)	44 (33 to 60)	10.3 (7.6 to 13.9)	43 (33 to 58)	10.1 (7.6 to 13.6)	15.8 (-21.7 to 81.5)	-17.5 (-26.3 to -7.9)
	15-49 years	126 (87 to 166)	18.9 (13.1 to 25.0)	358 (258 to 479)	36.5 (26.3 to 48.8)	299 (208 to 404)	30.1 (20.9 to 40.6)	301 (211 to 402)	30.0 (21.1 to 40.0)	58.5 (4.4 to 154.7)	-17.7 (-25.8 to -9.8)
	50-69 years	219 (167 to 286)	152.2 (116.3 to 199.4)	502 (368 to 656)	255.2 (187.1 to 333.4)	422 (309 to 554)	216.7 (158.8 to 284.3)	401 (293 to 531)	209.5 (153.0 to 277.4)	37.7 (-7.5 to 103.4)	-17.9 (-24.8 to -10.1)
	70+ years	296 (228 to 382)	690.1 (532.3 to 891.1)	427 (342 to 527)	886.6 (709.2 to 1,093.9)	353 (282 to 442)	720.6 (574.7 to 902.6)	322 (255 to 401)	670.1 (530.5 to 834.8)	-2.9 (-29.8 to 36.2)	-24.4 (-30.4 to -18.4)
**Namibia**	All Ages	1,030 (818 to 1,280)	73.6 (58.3 to 91.1)	1,400 (1,010 to 1,870)	58.7 (42.6 to 78.8)	1,180 (838 to 1,580)	48.9 (34.8 to 65.9)	1,160 (833 to 1,570)	47.8 (34.2 to 64.6)	-35.0 (-51.5 to -14.1)	-18.6 (-24.5 to -11.5)
	Under 5	398 (306 to 499)	176.0 (135.3 to 220.2)	224 (154 to 312)	79.3 (54.7 to 110.6)	171 (112 to 243)	61.0 (39.9 to 86.7)	166 (109 to 235)	59.5 (39.0 to 84.3)	-66.2 (-77.1 to -49.9)	-25.0 (-35.6 to -11.1)
	5-14 years	33 (22 to 46)	8.7 (5.9 to 12.3)	37 (25 to 53)	6.9 (4.7 to 9.9)	32 (22 to 46)	5.9 (4.1 to 8.5)	31 (21 to 45)	5.7 (3.9 to 8.2)	-35.1 (-56.1 to 0.5)	-17.8 (-26.8 to -2.0)
	15-49 years	154 (106 to 214)	23.4 (16.1 to 32.5)	280 (175 to 425)	22.4 (14.0 to 33.9)	244 (149 to 368)	19.2 (11.7 to 28.9)	246 (151 to 376)	19.1 (11.7 to 29.1)	-18.7 (-46.5 to 27.5)	-14.7 (-24.3 to -3.4)
	50-69 years	239 (172 to 319)	201.8 (145.1 to 269.1)	387 (267 to 541)	160.7 (110.7 to 224.6)	349 (237 to 493)	140.3 (95.1 to 197.9)	353 (237 to 497)	138.2 (93.0 to 194.9)	-31.5 (-52.6 to -2.9)	-14.0 (-21.2 to -6.3)
	70+ years	208 (148 to 284)	784.8 (555.6 to 1,068.5)	467 (349 to 606)	801.4 (599.2 to 1,039.4)	380 (284 to 493)	639.4 (478.5 to 831.0)	366 (274 to 476)	612.0 (457.8 to 794.3)	-22.0 (-41.0 to 5.6)	-23.6 (-29.3 to -17.0)
**South Africa**	All Ages	27,900 (25,300 to 31,100)	75.4 (68.4 to 84.1)	34,300 (31,700 to 37,200)	61.2 (56.5 to 66.4)	28,500 (25,900 to 30,900)	50.4 (45.9 to 54.8)	28,300 (25,800 to 31,100)	49.8 (45.4 to 54.7)	-33.9 (-41.7 to -26.1)	-18.6 (-23.8 to -13.0)
	Under 5	13,600 (11,500 to 16,500)	278.5 (236.3 to 337.7)	5,880 (4,690 to 7,160)	114.9 (91.6 to 139.8)	3,630 (2,870 to 4,440)	71.9 (56.9 to 88.0)	3,540 (2,760 to 4,400)	71.3 (55.6 to 88.6)	-74.4 (-80.7 to -66.8)	-37.9 (-44.7 to -30.6)
	5-14 years	865 (733 to 998)	9.9 (8.4 to 11.4)	732 (623 to 845)	7.2 (6.1 to 8.3)	641 (545 to 742)	6.3 (5.3 to 7.3)	651 (549 to 759)	6.4 (5.4 to 7.4)	-35.8 (-46.7 to -23.9)	-11.7 (-19.1 to -3.2)
	15-49 years	6,030 (5,400 to 6,640)	32.0 (28.6 to 35.2)	6,690 (6,100 to 7,340)	21.8 (19.9 to 24.0)	5,950 (5,310 to 6,600)	19.2 (17.2 to 21.4)	6,270 (5,530 to 7,040)	20.1 (17.7 to 22.6)	-37.0 (-45.5 to -27.9)	-7.9 (-18.4 to 3.5)

	50-69 years	2,740<br>(2,300 to 3,260)	77.9<br>(65.2 to 92.6)	8,710<br>(8,000 to 9,460)	109.3<br>(100.4 to 118.6)	7,940<br>(7,200 to 8,700)	97.4<br>(88.3 to 106.7)	7,960<br>(7,200 to 8,850)	96.1<br>(86.9 to 106.8)	23.3<br>(4.6 to 47.1)	-12.0<br>(-19.5 to -4.9)
	70+ years	4,680<br>(3,910 to 5,740)	460.8<br>(385.0 to 565.5)	12,300<br>(10,900 to 13,500)	571.2<br>(508.9 to 628.3)	10,300<br>(9,060 to 11,400)	470.7<br>(414.0 to 520.2)	9,890<br>(8,680 to 11,000)	447.0<br>(392.4 to 497.5)	-3.0<br>(-17.8 to 13.6)	-21.7<br>(-26.0 to -17.3)
**Zimbabwe**	All Ages	8,120<br>(7,130 to 9,040)	78.5<br>(68.9 to 87.4)	13,800<br>(11,000 to 16,700)	91.1<br>(72.7 to 110.6)	11,400<br>(8,980 to 14,100)	74.0<br>(58.4 to 91.6)	11,500<br>(9,020 to 14,300)	74.0<br>(57.9 to 92.0)	-5.7<br>(-26.3 to 18.2)	-18.8<br>(-24.8 to -12.7)
	Under 5	4,430<br>(3,730 to 5,160)	251.1<br>(211.4 to 292.7)	4,820<br>(3,780 to 5,980)	217.4<br>(170.4 to 269.8)	3,610<br>(2,780 to 4,580)	163.1<br>(125.8 to 206.8)	3,820<br>(2,950 to 4,850)	173.1<br>(133.5 to 219.6)	-31.1<br>(-48.7 to -8.6)	-20.4<br>(-28.6 to -11.1)
	5-14 years	137<br>(96 to 181)	4.5<br>(3.1 to 5.9)	330<br>(231 to 446)	8.3<br>(5.8 to 11.3)	278<br>(191 to 384)	6.9<br>(4.7 to 9.5)	313<br>(234 to 411)	7.7<br>(5.7 to 10.1)	71.1<br>(18.2 to 147.7)	-7.9<br>(-20.7 to 8.8)
	15-49 years	701<br>(571 to 859)	15.2<br>(12.4 to 18.6)	2,530<br>(1,850 to 3,370)	33.8<br>(24.8 to 45.1)	2,190<br>(1,570 to 2,950)	28.8<br>(20.6 to 38.8)	2,240<br>(1,600 to 3,060)	29.0<br>(20.7 to 39.5)	90.5<br>(32.7 to 173.5)	-14.3<br>(-22.4 to -6.3)
	50-69 years	1,350<br>(1,060 to 1,660)	182.8<br>(144.4 to 224.9)	3,340<br>(2,430 to 4,280)	272.0<br>(197.4 to 348.4)	3,030<br>(2,200 to 3,900)	239.0<br>(174.0 to 308.1)	3,040<br>(2,220 to 3,940)	235.8<br>(171.7 to 305.5)	29.0<br>(-9.4 to 82.9)	-13.3<br>(-20.0 to -6.6)
	70+ years	1,500<br>(1,190 to 1,820)	837.3<br>(661.6 to 1,015.5)	2,780<br>(2,120 to 3,380)	1,062.9<br>(810.2 to 1,292.7)	2,270<br>(1,740 to 2,790)	860.6<br>(657.2 to 1,055.9)	2,120<br>(1,590 to 2,620)	798.3<br>(599.4 to 986.6)	-4.7<br>(-27.2 to 24.9)	-24.9<br>(-30.3 to -19.0)
**Benin**	All Ages	8,060<br>(6,600 to 9,730)	166.1<br>(136.0 to 200.6)	7,800<br>(6,020 to 9,970)	61.5<br>(47.5 to 78.6)	7,290<br>(5,580 to 9,470)	55.7<br>(42.6 to 72.3)	6,810<br>(5,200 to 8,860)	50.5<br>(38.5 to 65.7)	-69.6<br>(-76.2 to -61.2)	-17.9<br>(-24.7 to -9.7)
	Under 5	5,930<br>(4,540 to 7,590)	601.3<br>(460.0 to 769.3)	4,300<br>(3,000 to 6,080)	191.6<br>(134.0 to 271.2)	3,900<br>(2,700 to 5,640)	169.9<br>(117.7 to 246.0)	3,480<br>(2,400 to 5,010)	148.8<br>(102.5 to 213.8)	-75.3<br>(-82.7 to -64.3)	-22.4<br>(-31.6 to -10.8)
	5-14 years	227<br>(158 to 309)	15.8<br>(11.0 to 21.5)	286<br>(204 to 405)	8.1<br>(5.8 to 11.5)	288<br>(204 to 401)	7.9<br>(5.6 to 11.0)	276<br>(195 to 388)	7.4<br>(5.2 to 10.4)	-53.2<br>(-69.6 to -28.5)	-9.1<br>(-20.4 to 6.1)
	15-49 years	292<br>(232 to 361)	14.6<br>(11.6 to 18.0)	600<br>(427 to 809)	10.3<br>(7.3 to 13.9)	587<br>(415 to 786)	9.7<br>(6.9 to 13.0)	583<br>(407 to 784)	9.3<br>(6.5 to 12.5)	-36.0<br>(-57.2 to -7.6)	-9.4<br>(-17.8 to -0.6)
	50-69 years	540<br>(433 to 661)	167.3<br>(134.1 to 204.7)	888<br>(644 to 1,170)	103.0<br>(74.6 to 135.6)	902<br>(646 to 1,190)	99.3<br>(71.2 to 131.2)	896<br>(643 to 1,200)	95.0<br>(68.1 to 127.1)	-43.2<br>(-61.3 to -18.1)	-7.7<br>(-15.5 to 0.9)
	70+ years	1,060<br>(882 to 1,260)	1,037.1<br>(859.5 to 1,227.0)	1,730<br>(1,390 to 2,080)	787.1<br>(633.5 to 949.5)	1,620<br>(1,280 to 1,960)	743.5<br>(589.2 to 900.2)	1,570<br>(1,250 to 1,910)	703.3<br>(556.8 to 853.7)	-32.2<br>(-48.6 to -10.9)	-10.6<br>(-16.7 to -3.6)
**Burkina Faso**	All Ages	19,300<br>(15,300 to 23,300)	202.8<br>(160.9 to 244.8)	19,900<br>(15,800 to 24,500)	92.4<br>(73.1 to 113.7)	17,700<br>(13,900 to 21,600)	80.0<br>(62.8 to 97.6)	16,700<br>(13,300 to 20,500)	73.2<br>(58.2 to 90.0)	-63.9<br>(-70.9 to -55.0)	-20.8<br>(-26.9 to -13.8)
	Under 5	14,800<br>(11,200 to 18,700)	790.3<br>(597.4 to 997.1)	12,400<br>(8,990 to 16,300)	316.6<br>(229.4 to 416.9)	10,600<br>(7,710 to 14,100)	265.3<br>(192.1 to 350.3)	9,780<br>(7,000 to 13,200)	238.3<br>(170.4 to 320.3)	-69.9<br>(-77.6 to -59.7)	-24.7<br>(-32.2 to -16.4)
	5-14 years	411<br>(283 to 560)	14.5<br>(10.0 to 19.7)	690<br>(494 to 888)	11.6<br>(8.3 to 14.9)	611<br>(442 to 799)	10.0<br>(7.2 to 13.1)	586<br>(421 to 764)	9.4<br>(6.7 to 12.2)	-35.4<br>(-54.9 to -4.7)	-19.0<br>(-26.8 to -10.0)
	15-49 years	564<br>(431 to 695)	14.7<br>(11.3 to 18.1)	1,120<br>(836 to 1,420)	11.6<br>(8.6 to 14.7)	1,060<br>(775 to 1,370)	10.6<br>(7.8 to 13.7)	1,040<br>(763 to 1,360)	10.1<br>(7.4 to 13.2)	-31.3<br>(-50.9 to -6.2)	-12.7<br>(-20.9 to -4.4)

	50-69 years	1,360 (1,080 to 1,700)	174.6 (138.6 to 218.2)	1,960 (1,480 to 2,450)	124.7 (94.1 to 156.4)	1,860 (1,380 to 2,360)	115.5 (85.8 to 146.3)	1,840 (1,360 to 2,380)	110.9 (82.0 to 143.5)	-36.5 (-54.7 to -12.5)	-11.0 (-18.7 to -3.0)
	70+ years	2,150 (1,760 to 2,580)	1,097.2 (899.3 to 1,315.2)	3,740 (3,050 to 4,500)	915.3 (745.8 to 1,100.9)	3,540 (2,890 to 4,310)	847.7 (691.9 to 1,030.7)	3,400 (2,740 to 4,130)	807.6 (651.5 to 981.6)	-26.4 (-42.5 to -3.3)	-11.8 (-18.6 to -5.1)
**Cabo Verde**	All Ages	185 (157 to 220)	52.4 (44.3 to 62.1)	296 (250 to 344)	53.6 (45.3 to 62.2)	274 (228 to 321)	49.3 (41.0 to 57.8)	262 (214 to 312)	46.8 (38.3 to 55.8)	-10.7 (-29.3 to 11.3)	-12.8 (-18.1 to -7.2)
	Under 5	67 (48 to 91)	113.4 (81.1 to 152.7)	11 (8 to 15)	23.9 (16.4 to 32.3)	9 (6 to 12)	19.8 (13.4 to 27.5)	8 (5 to 11)	17.8 (11.6 to 25.3)	-84.3 (-91.1 to -73.5)	-25.6 (-35.3 to -14.2)
	5-14 years	5 (3 to 6)	4.7 (2.9 to 6.5)	3 (2 to 4)	3.0 (2.4 to 3.7)	3 (2 to 3)	2.8 (2.2 to 3.4)	3 (2 to 3)	2.6 (2.1 to 3.2)	-44.4 (-62.3 to -6.9)	-14.5 (-21.9 to -6.1)
	15-49 years	16 (13 to 20)	11.0 (9.1 to 13.3)	27 (21 to 35)	8.8 (6.8 to 11.4)	26 (20 to 34)	8.4 (6.4 to 11.0)	26 (20 to 34)	8.3 (6.2 to 11.0)	-24.9 (-44.6 to 5.3)	-6.3 (-14.1 to 2.1)
	50-69 years	21 (16 to 27)	61.8 (48.1 to 79.9)	59 (45 to 75)	78.1 (59.8 to 99.3)	60 (46 to 77)	76.3 (58.1 to 96.9)	61 (46 to 78)	74.6 (55.9 to 95.2)	20.6 (-14.5 to 72.5)	-4.5 (-13.3 to 4.0)
	70+ years	76 (62 to 97)	508.4 (413.1 to 650.3)	196 (165 to 228)	943.8 (793.8 to 1,102.1)	176 (145 to 207)	875.2 (722.8 to 1,032.0)	164 (134 to 196)	802.4 (655.1 to 955.3)	57.8 (20.4 to 103.8)	-15.0 (-20.6 to -9.2)
**Cameroon**	All Ages	12,600 (9,950 to 16,000)	120.9 (95.4 to 153.7)	17,800 (13,400 to 23,200)	59.2 (44.6 to 77.5)	15,800 (11,900 to 20,600)	51.0 (38.5 to 66.6)	14,900 (11,200 to 19,400)	46.8 (35.3 to 61.1)	-61.3 (-69.8 to -50.0)	-20.9 (-28.0 to -12.3)
	Under 5	8,690 (6,470 to 11,500)	433.2 (322.6 to 575.1)	8,880 (6,390 to 11,700)	185.7 (133.6 to 244.8)	7,400 (5,130 to 9,990)	153.3 (106.2 to 207.0)	6,610 (4,710 to 8,810)	135.9 (96.8 to 181.1)	-68.6 (-77.0 to -56.7)	-26.8 (-35.7 to -15.8)
	5-14 years	336 (222 to 475)	11.7 (7.7 to 16.5)	659 (446 to 929)	8.1 (5.5 to 11.4)	603 (405 to 862)	7.2 (4.8 to 10.3)	565 (376 to 805)	6.6 (4.4 to 9.4)	-43.8 (-61.5 to -17.4)	-18.8 (-29.6 to -6.8)
	15-49 years	658 (484 to 863)	14.5 (10.7 to 19.1)	1,820 (1,190 to 2,650)	12.7 (8.3 to 18.5)	1,720 (1,140 to 2,480)	11.6 (7.7 to 16.6)	1,740 (1,140 to 2,530)	11.3 (7.4 to 16.4)	-22.5 (-46.8 to 9.9)	-11.1 (-19.6 to -1.3)
	50-69 years	1,210 (898 to 1,570)	143.1 (106.6 to 186.1)	2,580 (1,680 to 3,850)	116.9 (76.2 to 174.2)	2,490 (1,640 to 3,730)	108.1 (71.4 to 161.8)	2,500 (1,640 to 3,740)	105.0 (69.0 to 156.7)	-26.6 (-50.4 to 8.1)	-10.2 (-18.4 to -1.3)
	70+ years	1,720 (1,320 to 2,140)	937.1 (715.7 to 1,164.4)	3,810 (2,820 to 5,120)	789.3 (584.8 to 1,062.1)	3,540 (2,600 to 4,760)	718.6 (527.8 to 966.6)	3,470 (2,550 to 4,680)	693.0 (508.8 to 936.4)	-26.0 (-42.9 to -2.7)	-12.2 (-19.5 to -3.9)
**Chad**	All Ages	11,700 (9,640 to 14,000)	194.2 (160.0 to 231.5)	20,000 (15,800 to 24,600)	121.3 (95.9 to 149.5)	18,900 (15,100 to 23,400)	110.3 (88.2 to 136.9)	18,500 (14,500 to 22,900)	104.0 (81.8 to 129.2)	-46.4 (-59.4 to -29.9)	-14.2 (-21.8 to -6.2)
	Under 5	8,720 (6,890 to 10,800)	701.8 (554.1 to 869.0)	14,300 (10,900 to 18,100)	422.5 (322.1 to 534.9)	13,400 (10,300 to 16,900)	380.4 (292.4 to 482.4)	13,000 (9,870 to 16,600)	357.9 (271.4 to 456.4)	-49.0 (-64.3 to -29.1)	-15.3 (-24.9 to -5.3)
	5-14 years	278 (169 to 407)	16.5 (10.0 to 24.2)	699 (496 to 930)	13.9 (9.9 to 18.6)	662 (469 to 871)	12.7 (9.0 to 16.8)	657 (460 to 861)	12.2 (8.5 to 16.0)	-26.2 (-52.5 to 17.5)	-12.4 (-25.2 to 5.0)
	15-49 years	380 (261 to 519)	15.2 (10.5 to 20.8)	1,000 (734 to 1,330)	14.7 (10.7 to 19.5)	1,000 (735 to 1,320)	14.1 (10.3 to 18.5)	1,020 (760 to 1,350)	13.8 (10.3 to 18.2)	-9.7 (-40.4 to 37.3)	-6.2 (-14.0 to 1.8)

	50-69 years	804<br>(572 to 1,100)	175.4<br>(124.9 to 240.1)	1,610<br>(1,170 to 2,100)	159.4<br>(116.6 to 208.8)	1,620<br>(1,190 to 2,110)	155.0<br>(113.8 to 201.4)	1,630<br>(1,190 to 2,130)	150.8<br>(110.5 to 197.5)	-14.1<br>(-42.4 to 27.0)	-5.4<br>(-12.9 to 2.6)
	70+ years	1,520<br>(1,100 to 1,980)	1,006.2<br>(733.9 to 1,316.3)	2,360<br>(1,890 to 2,910)	994.1<br>(794.8 to 1,222.3)	2,220<br>(1,800 to 2,730)	945.9<br>(764.1 to 1,159.1)	2,150<br>(1,740 to 2,660)	901.9<br>(730.5 to 1,118.3)	-10.4<br>(-34.5 to 26.7)	-9.3<br>(-15.6 to -2.5)
**Côte d'Ivoire**	All Ages	15,400<br>(12,100 to 19,800)	126.3<br>(99.3 to 162.3)	17,200<br>(13,000 to 22,200)	64.8<br>(48.8 to 83.3)	16,100<br>(12,300 to 20,700)	59.3<br>(45.3 to 76.0)	14,000<br>(10,600 to 18,000)	50.3<br>(38.2 to 64.5)	-60.2<br>(-68.5 to -50.4)	-22.4<br>(-27.6 to -16.5)
	Under 5	11,700<br>(8,790 to 15,600)	504.7<br>(380.5 to 676.3)	9,440<br>(6,790 to 12,800)	221.5<br>(159.3 to 301.1)	8,400<br>(5,930 to 11,500)	194.7<br>(137.6 to 265.6)	6,850<br>(4,840 to 9,310)	157.3<br>(111.0 to 213.7)	-68.8<br>(-76.7 to -59.1)	-29.0<br>(-35.8 to -21.4)
	5-14 years	409<br>(288 to 544)	12.1<br>(8.5 to 16.0)	581<br>(399 to 822)	8.5<br>(5.8 to 12.0)	551<br>(380 to 784)	7.8<br>(5.4 to 11.1)	505<br>(349 to 710)	7.0<br>(4.8 to 9.8)	-42.0<br>(-61.2 to -12.6)	-17.3<br>(-26.9 to -7.5)
	15-49 years	823<br>(635 to 1,040)	14.8<br>(11.4 to 18.7)	1,670<br>(1,150 to 2,360)	12.8<br>(8.8 to 18.0)	1,660<br>(1,120 to 2,380)	12.4<br>(8.4 to 17.8)	1,540<br>(1,040 to 2,200)	11.2<br>(7.6 to 16.0)	-24.0<br>(-48.5 to 10.3)	-12.2<br>(-18.3 to -5.4)
	50-69 years	1,250<br>(968 to 1,560)	156.4<br>(121.4 to 196.2)	2,320<br>(1,580 to 3,270)	114.3<br>(78.0 to 161.2)	2,310<br>(1,580 to 3,290)	110.2<br>(75.4 to 156.9)	2,210<br>(1,500 to 3,130)	101.3<br>(68.8 to 143.7)	-35.2<br>(-56.0 to -4.7)	-11.4<br>(-18.4 to -4.3)
	70+ years	1,270<br>(1,060 to 1,520)	949.7<br>(790.1 to 1,136.0)	3,230<br>(2,530 to 4,100)	815.1<br>(638.6 to 1,036.2)	3,220<br>(2,540 to 4,100)	783.5<br>(617.8 to 996.9)	2,910<br>(2,280 to 3,750)	698.8<br>(546.7 to 898.7)	-26.4<br>(-43.5 to -1.5)	-14.3<br>(-19.5 to -8.3)
**Gambia**	All Ages	1,160<br>(965 to 1,400)	117.8<br>(98.3 to 142.8)	1,420<br>(1,150 to 1,690)	62.2<br>(50.4 to 74.2)	1,280<br>(1,050 to 1,540)	54.9<br>(44.8 to 65.6)	1,220<br>(975 to 1,480)	51.0<br>(40.7 to 61.8)	-56.7<br>(-66.6 to -45.1)	-18.0<br>(-23.9 to -10.8)
	Under 5	751<br>(588 to 979)	404.6<br>(316.7 to 527.2)	340<br>(255 to 445)	96.6<br>(72.3 to 126.4)	280<br>(210 to 365)	78.9<br>(59.3 to 102.7)	250<br>(188 to 331)	69.9<br>(52.6 to 92.7)	-82.7<br>(-88.1 to -75.4)	-27.6<br>(-35.5 to -19.0)
	5-14 years	39<br>(27 to 51)	14.0<br>(9.8 to 18.6)	55<br>(41 to 73)	9.0<br>(6.8 to 11.9)	49<br>(37 to 64)	7.9<br>(5.9 to 10.3)	47<br>(35 to 61)	7.4<br>(5.5 to 9.6)	-47.5<br>(-63.4 to -24.3)	-18.3<br>(-27.6 to -8.4)
	15-49 years	79<br>(57 to 103)	17.9<br>(12.9 to 23.3)	201<br>(147 to 265)	18.0<br>(13.2 to 23.8)	188<br>(136 to 249)	16.3<br>(11.9 to 21.7)	186<br>(133 to 251)	15.7<br>(11.2 to 21.1)	-12.1<br>(-41.8 to 30.3)	-12.8<br>(-20.7 to -4.6)
	50-69 years	117<br>(86 to 153)	189.6<br>(139.9 to 248.8)	279<br>(202 to 367)	174.9<br>(126.7 to 229.8)	264<br>(190 to 347)	160.6<br>(115.4 to 211.2)	264<br>(188 to 346)	155.7<br>(110.9 to 203.5)	-17.9<br>(-46.1 to 17.9)	-10.9<br>(-18.7 to -3.0)
	70+ years	170<br>(138 to 210)	1,102.2<br>(897.4 to 1,358.0)	544<br>(421 to 670)	1,206.6<br>(935.0 to 1,487.6)	502<br>(380 to 624)	1,102.6<br>(833.0 to 1,368.5)	472<br>(354 to 584)	1,053.2<br>(789.9 to 1,302.5)	-4.4<br>(-30.8 to 27.4)	-12.7<br>(-20.1 to -4.2)
**Ghana**	All Ages	12,100<br>(9,990 to 14,600)	81.0<br>(66.7 to 97.3)	15,700<br>(12,600 to 19,000)	48.0<br>(38.5 to 58.4)	14,300<br>(11,400 to 17,300)	42.8<br>(34.0 to 51.7)	13,500<br>(10,600 to 16,600)	39.4<br>(31.0 to 48.4)	-51.4<br>(-62.8 to -37.4)	-18.0<br>(-24.2 to -11.4)
	Under 5	6,940<br>(5,200 to 9,070)	263.3<br>(197.3 to 344.4)	3,840<br>(2,610 to 5,320)	84.1<br>(57.1 to 116.3)	3,150<br>(2,060 to 4,400)	68.3<br>(44.6 to 95.5)	2,700<br>(1,710 to 3,860)	58.1<br>(36.9 to 83.1)	-77.9<br>(-84.6 to -68.2)	-30.9<br>(-39.3 to -22.1)
	5-14 years	363<br>(258 to 483)	8.9<br>(6.3 to 11.8)	413<br>(303 to 551)	5.3<br>(3.9 to 7.0)	383<br>(282 to 525)	4.8<br>(3.5 to 6.5)	360<br>(264 to 484)	4.4<br>(3.2 to 5.9)	-50.9<br>(-68.0 to -23.6)	-17.2<br>(-26.1 to -7.5)
	15-49 years	922<br>(675 to 1,220)	13.5<br>(9.9 to 17.8)	2,520<br>(1,890 to 3,290)	15.2<br>(11.4 to 19.8)	2,360<br>(1,720 to 3,100)	13.8<br>(10.1 to 18.2)	2,280<br>(1,660 to 3,080)	13.0<br>(9.5 to 17.6)	-3.7<br>(-38.5 to 48.1)	-14.4<br>(-22.5 to -5.3)

	50-69 years	1,770 (1,320 to 2,330)	152.3 (113.0 to 200.1)	3,670 (2,710 to 4,730)	124.8 (92.0 to 160.9)	3,510 (2,580 to 4,600)	115.3 (84.9 to 151.2)	3,450 (2,500 to 4,520)	109.7 (79.3 to 143.8)	-28.0 (-53.1 to 7.6)	-12.1 (-20.6 to -2.9)
	70+ years	2,130 (1,670 to 2,710)	841.4 (657.8 to 1,069.7)	5,220 (4,170 to 6,430)	790.7 (632.2 to 974.2)	4,920 (3,900 to 6,150)	723.7 (573.4 to 903.9)	4,700 (3,680 to 5,890)	682.6 (535.2 to 855.1)	-18.9 (-43.3 to 12.8)	-13.7 (-21.6 to -5.5)
**Guinea**	All Ages	15,500 (12,300 to 19,400)	259.2 (204.7 to 323.7)	12,300 (9,530 to 15,400)	96.9 (74.8 to 121.1)	11,100 (8,540 to 13,900)	85.1 (65.3 to 106.4)	10,300 (7,900 to 13,000)	76.5 (58.8 to 96.9)	-70.5 (-77.5 to -60.6)	-21.1 (-27.4 to -14.4)
	Under 5	12,200 (9,020 to 16,100)	1,051.8 (777.5 to 1,386.4)	6,620 (4,520 to 8,960)	302.8 (207.1 to 410.1)	5,690 (3,820 to 7,750)	256.6 (172.0 to 349.4)	5,060 (3,400 to 7,010)	224.8 (150.9 to 311.5)	-78.6 (-85.0 to -69.6)	-25.8 (-35.5 to -15.1)
	5-14 years	324 (208 to 456)	20.3 (13.1 to 28.7)	449 (320 to 607)	12.5 (8.9 to 16.9)	418 (298 to 566)	11.3 (8.1 to 15.3)	390 (277 to 528)	10.3 (7.3 to 13.9)	-49.5 (-65.0 to -21.9)	-17.6 (-30.0 to -2.7)
	15-49 years	405 (285 to 545)	16.1 (11.3 to 21.6)	904 (650 to 1,260)	15.7 (11.3 to 22.0)	885 (638 to 1,230)	14.9 (10.7 to 20.7)	885 (639 to 1,230)	14.4 (10.4 to 20.0)	-10.1 (-36.9 to 30.8)	-8.3 (-15.5 to -0.7)
	50-69 years	952 (687 to 1,230)	170.3 (122.9 to 220.6)	1,520 (1,080 to 2,080)	159.4 (113.6 to 218.8)	1,460 (1,050 to 2,010)	150.4 (107.9 to 207.0)	1,440 (1,040 to 1,990)	144.7 (104.2 to 200.5)	-15.1 (-40.7 to 21.2)	-9.2 (-16.6 to -1.5)
	70+ years	1,650 (1,200 to 2,090)	995.6 (723.6 to 1,260.1)	2,860 (2,220 to 3,690)	1,099.6 (853.0 to 1,418.4)	2,680 (2,110 to 3,430)	1,022.2 (802.6 to 1,305.5)	2,500 (1,970 to 3,190)	963.9 (757.9 to 1,229.0)	-3.2 (-26.8 to 29.4)	-12.3 (-18.3 to -6.4)
**Guinea-Bissau**	All Ages	1,740 (1,360 to 2,150)	172.4 (135.6 to 213.5)	1,180 (961 to 1,410)	59.6 (48.7 to 71.3)	1,080 (884 to 1,280)	53.5 (43.8 to 63.3)	1,020 (828 to 1,210)	49.3 (40.1 to 58.8)	-71.4 (-77.6 to -63.7)	-17.3 (-24.0 to -10.4)
	Under 5	1,140 (832 to 1,480)	606.7 (442.5 to 789.6)	352 (241 to 485)	107.3 (73.4 to 147.9)	298 (207 to 407)	90.2 (62.6 to 123.0)	261 (184 to 362)	78.5 (55.3 to 108.9)	-87.1 (-91.3 to -81.3)	-26.8 (-37.5 to -14.9)
	5-14 years	59 (39 to 81)	20.2 (13.3 to 27.6)	52 (35 to 88)	9.6 (6.5 to 16.3)	49 (34 to 84)	8.9 (6.1 to 15.2)	46 (32 to 77)	8.2 (5.6 to 13.7)	-59.6 (-72.5 to -33.1)	-15.4 (-26.8 to -1.9)
	15-49 years	126 (98 to 159)	28.8 (22.4 to 36.4)	218 (165 to 286)	23.0 (17.5 to 30.2)	210 (157 to 274)	21.5 (16.1 to 28.2)	208 (153 to 273)	20.8 (15.3 to 27.3)	-27.8 (-49.3 to 0.4)	-9.7 (-17.6 to -0.4)
	50-69 years	205 (155 to 261)	286.5 (217.5 to 365.0)	272 (207 to 341)	204.7 (155.7 to 256.6)	265 (198 to 331)	193.2 (144.5 to 241.3)	259 (194 to 325)	184.4 (138.3 to 232.0)	-35.6 (-54.6 to -9.9)	-9.9 (-17.6 to -2.0)
	70+ years	205 (166 to 249)	1,254.7 (1,012.5 to 1,524.8)	283 (223 to 343)	1,111.6 (877.8 to 1,348.6)	259 (203 to 316)	1,026.4 (807.0 to 1,254.5)	245 (193 to 299)	964.7 (760.8 to 1,177.5)	-23.1 (-41.8 to 1.5)	-13.2 (-20.4 to -5.9)
**Liberia**	All Ages	4,970 (3,810 to 6,450)	201.8 (154.8 to 262.0)	2,130 (1,610 to 2,810)	40.9 (31.0 to 54.0)	1,960 (1,500 to 2,570)	36.8 (28.1 to 48.2)	1,880 (1,400 to 2,490)	34.4 (25.6 to 45.7)	-83.0 (-87.5 to -77.0)	-16.0 (-23.4 to -8.3)
	Under 5	4,040 (2,960 to 5,440)	869.2 (637.8 to 1,170.3)	763 (507 to 1,100)	99.6 (66.2 to 143.6)	641 (413 to 932)	83.7 (54.0 to 121.7)	567 (359 to 834)	74.0 (46.8 to 108.8)	-91.5 (-94.2 to -87.5)	-25.7 (-36.4 to -12.5)
	5-14 years	134 (90 to 184)	20.1 (13.5 to 27.6)	137 (96 to 185)	9.9 (7.0 to 13.4)	128 (91 to 173)	9.1 (6.5 to 12.3)	121 (84 to 161)	8.5 (5.9 to 11.3)	-57.8 (-72.2 to -35.0)	-14.2 (-23.8 to -3.4)
	15-49 years	126 (95 to 161)	11.6 (8.8 to 14.8)	302 (213 to 417)	11.6 (8.1 to 15.9)	302 (215 to 419)	11.2 (7.9 to 15.5)	310 (215 to 430)	11.1 (7.7 to 15.3)	-5.0 (-36.5 to 43.3)	-4.4 (-12.0 to 3.6)

	50-69 years	235<br>(183 to 294)	124.9<br>(97.5 to 156.6)	344<br>(240 to 483)	95.2<br>(66.5 to 133.8)	350<br>(249 to 482)	92.9<br>(66.1 to 128.1)	353<br>(249 to 480)	90.4<br>(63.8 to 123.1)	-27.6<br>(-50.5 to 3.5)	-5.0<br>(-13.3 to 3.9)
	70+ years	434<br>(360 to 521)	729.8<br>(604.6 to 875.5)	583<br>(451 to 754)	733.2<br>(567.2 to 948.2)	540<br>(425 to 691)	693.9<br>(546.1 to 888.3)	526<br>(408 to 676)	659.1<br>(512.0 to 847.5)	-9.7<br>(-31.5 to 21.1)	-10.1<br>(-16.5 to -3.1)
**Mali**	All Ages	9,770<br>(7,840 to 12,100)	112.8<br>(90.5 to 139.6)	12,600<br>(9,970 to 15,600)	56.3<br>(44.5 to 69.4)	11,300<br>(8,920 to 14,000)	48.5<br>(38.3 to 60.3)	10,300<br>(8,120 to 12,900)	42.7<br>(33.7 to 53.4)	-62.1<br>(-70.3 to -51.4)	-24.1<br>(-30.5 to -17.4)
	Under 5	6,960<br>(5,200 to 9,150)	402.6<br>(300.5 to 529.0)	8,000<br>(5,880 to 10,400)	186.2<br>(136.8 to 243.0)	6,910<br>(4,930 to 9,140)	155.7<br>(111.0 to 205.9)	6,100<br>(4,400 to 8,150)	133.1<br>(96.1 to 177.8)	-66.9<br>(-75.7 to -53.6)	-28.5<br>(-36.6 to -19.8)
	5-14 years	248<br>(160 to 350)	10.3<br>(6.7 to 14.6)	414<br>(289 to 587)	6.4<br>(4.4 to 9.0)	391<br>(274 to 545)	5.8<br>(4.1 to 8.1)	352<br>(243 to 493)	5.0<br>(3.5 to 7.1)	-51.3<br>(-68.7 to -23.1)	-20.7<br>(-30.7 to -10.7)
	15-49 years	519<br>(411 to 623)	14.3<br>(11.4 to 17.2)	896<br>(652 to 1,190)	9.3<br>(6.7 to 12.3)	866<br>(618 to 1,170)	8.6<br>(6.1 to 11.6)	866<br>(607 to 1,170)	8.2<br>(5.8 to 11.2)	-42.5<br>(-60.0 to -23.0)	-10.9<br>(-18.3 to -3.2)
	50-69 years	947<br>(753 to 1,170)	125.9<br>(100.1 to 155.4)	1,290<br>(936 to 1,700)	82.7<br>(60.0 to 108.9)	1,250<br>(895 to 1,660)	77.5<br>(55.5 to 103.1)	1,240<br>(885 to 1,650)	74.8<br>(53.5 to 99.8)	-40.6<br>(-58.8 to -17.7)	-9.6<br>(-17.1 to -1.7)
	70+ years	1,090<br>(902 to 1,300)	672.1<br>(555.1 to 798.4)	2,010<br>(1,530 to 2,530)	552.5<br>(420.5 to 694.4)	1,870<br>(1,400 to 2,380)	505.7<br>(378.7 to 644.9)	1,730<br>(1,290 to 2,220)	469.5<br>(350.1 to 600.1)	-30.1<br>(-49.1 to -6.7)	-15.0<br>(-21.7 to -7.5)
**Mauritania**	All Ages	2,100<br>(1,770 to 2,430)	102.1<br>(86.3 to 118.2)	1,920<br>(1,520 to 2,390)	45.7<br>(36.2 to 56.7)	1,770<br>(1,410 to 2,170)	41.2<br>(32.7 to 50.5)	1,740<br>(1,390 to 2,120)	39.5<br>(31.6 to 48.2)	-61.3<br>(-69.8 to -50.2)	-13.7<br>(-19.6 to -6.8)
	Under 5	1,160<br>(903 to 1,420)	312.8<br>(243.4 to 381.7)	593<br>(446 to 768)	90.3<br>(67.9 to 117.0)	492<br>(369 to 633)	74.8<br>(56.1 to 96.3)	452<br>(342 to 586)	68.7<br>(52.1 to 89.1)	-78.0<br>(-84.4 to -70.5)	-23.9<br>(-34.7 to -11.2)
	5-14 years	57<br>(41 to 74)	10.2<br>(7.5 to 13.5)	59<br>(43 to 79)	5.1<br>(3.8 to 6.9)	54<br>(40 to 71)	4.6<br>(3.4 to 6.0)	52<br>(39 to 68)	4.4<br>(3.3 to 5.7)	-57.4<br>(-69.8 to -37.4)	-15.0<br>(-26.9 to -1.8)
	15-49 years	124<br>(99 to 152)	13.6<br>(10.8 to 16.7)	154<br>(113 to 208)	7.9<br>(5.8 to 10.7)	148<br>(112 to 198)	7.4<br>(5.6 to 9.9)	152<br>(113 to 203)	7.4<br>(5.5 to 9.9)	-45.8<br>(-60.7 to -21.7)	-7.0<br>(-14.8 to 2.3)
	50-69 years	271<br>(210 to 337)	163.0<br>(126.4 to 202.4)	339<br>(237 to 466)	93.2<br>(65.3 to 128.2)	338<br>(238 to 463)	90.0<br>(63.3 to 123.3)	346<br>(243 to 472)	89.8<br>(63.0 to 122.6)	-44.9<br>(-62.0 to -20.0)	-3.6<br>(-11.5 to 5.9)
	70+ years	485<br>(385 to 592)	930.4<br>(737.4 to 1,135.2)	780<br>(591 to 1,020)	789.4<br>(598.0 to 1,028.3)	741<br>(558 to 959)	747.1<br>(562.8 to 967.3)	734<br>(552 to 959)	720.7<br>(541.9 to 942.5)	-22.5<br>(-42.5 to 2.5)	-8.7<br>(-15.7 to -1.2)
**Niger**	All Ages	26,000<br>(19,800 to 33,000)	324.1<br>(246.6 to 411.3)	19,000<br>(14,200 to 25,500)	82.1<br>(61.0 to 110.0)	17,900<br>(13,500 to 23,500)	74.1<br>(56.0 to 97.4)	16,800<br>(12,500 to 22,200)	67.1<br>(50.0 to 88.6)	-79.3<br>(-84.5 to -72.5)	-18.2<br>(-25.0 to -10.5)
	Under 5	23,000<br>(16,800 to 30,000)	1,362.5<br>(994.4 to 1,778.1)	13,100<br>(9,200 to 18,200)	277.1<br>(194.2 to 383.4)	12,000<br>(8,380 to 16,400)	245.0<br>(170.5 to 334.9)	11,100<br>(7,670 to 15,200)	217.0<br>(150.5 to 298.5)	-84.1<br>(-88.6 to -77.9)	-21.7<br>(-29.8 to -12.3)
	5-14 years	582<br>(365 to 804)	24.5<br>(15.4 to 33.8)	633<br>(408 to 910)	8.8<br>(5.7 to 12.7)	632<br>(436 to 882)	8.5<br>(5.9 to 11.9)	592<br>(404 to 830)	7.7<br>(5.3 to 10.8)	-68.4<br>(-79.4 to -51.1)	-12.3<br>(-23.5 to 1.2)
	15-49 years	539<br>(410 to 698)	16.1<br>(12.2 to 20.8)	970<br>(683 to 1,340)	10.2<br>(7.2 to 14.2)	962<br>(691 to 1,320)	9.7<br>(7.0 to 13.3)	972<br>(691 to 1,350)	9.4<br>(6.7 to 13.1)	-41.8<br>(-61.4 to -15.7)	-8.4<br>(-16.1 to -0.2)

	50-69 years	918 (694 to 1,170)	177.3 (133.9 to 225.1)	1,780 (1,290 to 2,410)	119.8 (86.6 to 161.7)	1,700 (1,220 to 2,260)	112.1 (80.7 to 149.3)	1,720 (1,230 to 2,330)	109.9 (78.6 to 149.2)	-38.0 (-58.7 to -8.3)	-8.3 (-16.0 to -0.2)
	70+ years	1,010 (761 to 1,280)	983.7 (740.6 to 1,240.4)	2,530 (1,970 to 3,250)	811.9 (632.5 to 1,042.6)	2,530 (1,980 to 3,260)	747.8 (586.7 to 963.6)	2,470 (1,920 to 3,180)	725.1 (564.3 to 931.9)	-26.3 (-45.2 to 2.8)	-10.7 (-16.8 to -4.1)
**Nigeria**	All Ages	170,000 (138,000 to 205,000)	188.4 (153.4 to 227.5)	172,000 (133,000 to 215,000)	78.4 (60.6 to 98.4)	153,000 (114,000 to 200,000)	68.1 (50.5 to 89.0)	142,000 (105,000 to 186,000)	61.4 (45.4 to 80.7)	-67.4 (-74.3 to -59.5)	-21.7 (-29.9 to -12.0)
	Under 5	141,000 (112,000 to 173,000)	889.2 (706.7 to 1,090.5)	128,000 (92,900 to 167,000)	353.2 (256.5 to 461.0)	111,000 (76,700 to 151,000)	303.1 (209.2 to 410.8)	100,000 (69,600 to 139,000)	270.0 (187.6 to 375.1)	-69.6 (-77.3 to -60.8)	-23.5 (-32.3 to -13.8)
	5-14 years	4,720 (3,100 to 6,070)	20.3 (13.3 to 26.1)	6,000 (4,050 to 7,980)	9.7 (6.6 to 13.0)	5,640 (3,620 to 7,610)	8.9 (5.7 to 12.1)	5,430 (3,490 to 7,380)	8.4 (5.4 to 11.4)	-58.5 (-68.9 to -44.6)	-13.6 (-27.5 to 4.3)
	15-49 years	4,530 (3,330 to 5,810)	11.1 (8.1 to 14.2)	6,980 (4,850 to 9,420)	6.9 (4.8 to 9.3)	6,750 (4,360 to 9,340)	6.5 (4.2 to 8.9)	6,810 (4,400 to 9,730)	6.3 (4.1 to 9.0)	-42.9 (-60.6 to -19.0)	-8.7 (-35.3 to 27.8)
	50-69 years	7,250 (5,580 to 9,160)	92.9 (71.5 to 117.4)	10,700 (7,940 to 14,000)	64.3 (47.5 to 83.6)	10,400 (7,500 to 13,700)	60.1 (43.3 to 79.4)	10,500 (7,610 to 14,200)	58.7 (42.4 to 79.3)	-36.8 (-56.5 to -11.4)	-8.7 (-33.9 to 26.5)
	70+ years	11,800 (9,640 to 14,600)	564.3 (460.1 to 696.0)	20,100 (16,800 to 23,800)	568.9 (475.8 to 674.4)	19,400 (15,900 to 23,100)	525.8 (432.3 to 628.5)	18,900 (15,600 to 22,700)	505.6 (415.9 to 606.4)	-10.4 (-27.9 to 10.3)	-11.1 (-23.3 to 3.5)
**Sao Tome and Principe**	All Ages	156 (133 to 178)	128.6 (109.4 to 146.8)	111 (92 to 133)	52.8 (44.0 to 63.4)	104 (86 to 125)	48.6 (40.3 to 58.5)	100 (83 to 121)	46.1 (38.3 to 55.9)	-64.1 (-70.9 to -55.1)	-12.6 (-17.7 to -6.3)
	Under 5	83 (65 to 101)	407.1 (316.4 to 491.1)	11 (8 to 16)	43.8 (29.7 to 61.1)	9 (6 to 13)	36.2 (24.0 to 52.0)	8 (5 to 11)	30.9 (20.2 to 44.8)	-92.4 (-95.3 to -88.5)	-29.3 (-38.5 to -18.1)
	5-14 years	10 (7 to 12)	26.9 (20.1 to 34.0)	3 (2 to 4)	6.3 (4.4 to 8.2)	3 (2 to 4)	5.5 (3.8 to 7.4)	3 (2 to 4)	4.9 (3.4 to 6.6)	-81.6 (-88.2 to -72.7)	-20.9 (-30.3 to -10.4)
	15-49 years	9 (6 to 11)	17.3 (11.3 to 22.3)	18 (13 to 26)	17.1 (12.0 to 24.0)	18 (12 to 25)	16.0 (11.1 to 22.8)	18 (12 to 25)	15.5 (10.5 to 22.1)	-10.3 (-43.4 to 52.3)	-9.2 (-19.3 to 2.1)
	50-69 years	19 (15 to 22)	165.5 (135.2 to 198.0)	28 (22 to 35)	143.0 (112.6 to 179.7)	28 (22 to 35)	135.5 (106.7 to 169.5)	28 (22 to 35)	130.8 (104.4 to 164.3)	-20.9 (-38.7 to 4.1)	-8.5 (-15.0 to -1.3)
	70+ years	35 (30 to 42)	1,074.3 (909.7 to 1,261.0)	50 (42 to 61)	1,119.7 (943.6 to 1,353.1)	46 (38 to 56)	1,027.5 (853.0 to 1,242.2)	44 (37 to 53)	964.3 (807.0 to 1,163.4)	-10.2 (-26.1 to 9.9)	-13.9 (-19.7 to -7.2)
**Senegal**	All Ages	8,530 (7,050 to 10,100)	111.8 (92.4 to 132.0)	6,610 (5,420 to 7,980)	43.3 (35.5 to 52.3)	5,790 (4,710 to 7,050)	37.2 (30.3 to 45.3)	5,510 (4,430 to 6,810)	34.7 (27.9 to 43.0)	-68.9 (-75.9 to -59.9)	-19.7 (-26.9 to -11.3)
	Under 5	6,290 (5,010 to 7,760)	429.1 (341.8 to 529.6)	2,620 (1,940 to 3,420)	115.7 (85.6 to 151.4)	2,060 (1,510 to 2,770)	90.9 (66.7 to 122.2)	1,810 (1,310 to 2,420)	79.8 (57.5 to 106.4)	-81.4 (-86.9 to -73.7)	-31.0 (-40.7 to -19.1)
	5-14 years	284 (207 to 371)	13.0 (9.5 to 17.0)	224 (171 to 293)	5.6 (4.3 to 7.4)	198 (151 to 261)	4.9 (3.7 to 6.5)	188 (141 to 246)	4.6 (3.4 to 6.0)	-64.6 (-75.4 to -47.9)	-18.4 (-29.0 to -6.0)
	15-49 years	398 (310 to 480)	12.2 (9.5 to 14.7)	689 (527 to 908)	9.4 (7.2 to 12.4)	637 (488 to 837)	8.4 (6.5 to 11.1)	632 (483 to 841)	8.2 (6.2 to 10.9)	-33.0 (-52.2 to -5.4)	-13.0 (-20.9 to -3.9)

	50-69 years	588<br>(473 to 714)	103.9<br>(83.7 to 126.3)	1,050<br>(771 to 1,390)	78.2<br>(57.5 to 103.4)	990<br>(726 to 1,310)	72.0<br>(52.8 to 95.2)	1,000<br>(726 to 1,340)	71.0<br>(51.5 to 95.0)	-31.7<br>(-52.4 to -4.4)	-9.2<br>(-17.9 to 0.7)
	70+ years	975<br>(800 to 1,170)	644.5<br>(529.3 to 774.7)	2,030<br>(1,550 to 2,620)	594.3<br>(453.9 to 767.2)	1,900<br>(1,430 to 2,450)	538.9<br>(405.9 to 695.0)	1,880<br>(1,400 to 2,430)	529.9<br>(394.5 to 685.3)	-17.8<br>(-39.2 to 8.6)	-10.8<br>(-19.2 to -1.0)
**Sierra Leone**	All Ages	9,090<br>(6,950 to 11,500)	218.9<br>(167.4 to 277.3)	5,460<br>(4,200 to 7,080)	65.1<br>(50.1 to 84.4)	4,970<br>(3,740 to 6,420)	57.7<br>(43.4 to 74.4)	4,720<br>(3,580 to 6,150)	53.2<br>(40.3 to 69.4)	-75.7<br>(-81.5 to -67.9)	-18.3<br>(-24.6 to -11.1)
	Under 5	6,960<br>(5,010 to 9,250)	899.7<br>(647.7 to 1,195.4)	2,300<br>(1,630 to 3,200)	176.8<br>(125.4 to 245.9)	1,950<br>(1,360 to 2,780)	147.6<br>(102.7 to 210.3)	1,740<br>(1,200 to 2,480)	129.8<br>(89.6 to 184.7)	-85.6<br>(-89.8 to -79.6)	-26.6<br>(-35.4 to -16.6)
	5-14 years	207<br>(140 to 281)	19.9<br>(13.5 to 27.0)	266<br>(191 to 366)	12.5<br>(9.0 to 17.2)	247<br>(172 to 339)	11.3<br>(7.9 to 15.5)	235<br>(164 to 321)	10.5<br>(7.3 to 14.4)	-47.2<br>(-63.4 to -19.5)	-15.9<br>(-24.0 to -6.8)
	15-49 years	270<br>(192 to 354)	14.2<br>(10.1 to 18.6)	581<br>(413 to 792)	14.0<br>(9.9 to 19.0)	563<br>(395 to 769)	13.1<br>(9.2 to 17.9)	564<br>(393 to 772)	12.7<br>(8.8 to 17.4)	-10.6<br>(-39.5 to 26.4)	-9.2<br>(-17.0 to -0.6)
	50-69 years	539<br>(400 to 695)	162.8<br>(121.0 to 210.1)	825<br>(607 to 1,080)	131.9<br>(97.1 to 172.1)	800<br>(576 to 1,050)	123.3<br>(88.7 to 161.5)	804<br>(573 to 1,070)	119.4<br>(85.2 to 159.6)	-26.7<br>(-48.5 to 6.2)	-9.5<br>(-17.5 to -1.2)
	70+ years	1,110<br>(921 to 1,340)	1,029.5<br>(852.5 to 1,245.4)	1,490<br>(1,180 to 1,870)	908.6<br>(720.9 to 1,145.4)	1,410<br>(1,110 to 1,800)	836.8<br>(658.2 to 1,069.8)	1,370<br>(1,070 to 1,730)	801.2<br>(623.1 to 1,009.3)	-22.2<br>(-40.7 to 2.0)	-11.8<br>(-18.5 to -4.4)
**Togo**	All Ages	4,060<br>(3,370 to 4,840)	111.4<br>(92.5 to 132.6)	4,780<br>(3,700 to 5,990)	59.5<br>(46.1 to 74.7)	4,390<br>(3,380 to 5,470)	53.5<br>(41.2 to 66.7)	4,360<br>(3,370 to 5,450)	52.1<br>(40.2 to 65.1)	-53.3<br>(-64.0 to -40.1)	-12.6<br>(-19.2 to -5.0)
	Under 5	2,810<br>(2,190 to 3,580)	409.1<br>(318.8 to 522.0)	1,710<br>(1,240 to 2,240)	144.6<br>(105.0 to 189.0)	1,420<br>(1,010 to 1,880)	120.4<br>(86.0 to 159.3)	1,300<br>(926 to 1,730)	111.1<br>(79.0 to 147.7)	-72.8<br>(-80.7 to -62.2)	-23.1<br>(-32.8 to -12.7)
	5-14 years	146<br>(104 to 191)	13.6<br>(9.7 to 17.8)	159<br>(114 to 210)	7.7<br>(5.5 to 10.2)	146<br>(107 to 194)	7.0<br>(5.1 to 9.2)	141<br>(101 to 186)	6.6<br>(4.8 to 8.7)	-51.5<br>(-65.6 to -28.3)	-14.3<br>(-24.8 to -2.6)
	15-49 years	254<br>(195 to 324)	15.8<br>(12.2 to 20.1)	650<br>(465 to 861)	16.5<br>(11.8 to 21.8)	619<br>(440 to 805)	15.3<br>(10.9 to 19.9)	628<br>(451 to 821)	15.1<br>(10.9 to 19.8)	-4.3<br>(-33.8 to 33.8)	-8.5<br>(-16.7 to 0.8)
	50-69 years	357<br>(275 to 454)	158.5<br>(121.8 to 201.3)	1,050<br>(742 to 1,390)	148.8<br>(105.5 to 198.3)	1,020<br>(737 to 1,350)	139.0<br>(100.8 to 184.5)	1,050<br>(769 to 1,390)	138.3<br>(101.1 to 182.3)	-12.7<br>(-38.1 to 27.3)	-7.0<br>(-15.7 to 2.8)
	70+ years	497<br>(401 to 609)	981.1<br>(790.2 to 1,201.1)	1,210<br>(932 to 1,570)	911.2<br>(701.7 to 1,179.4)	1,190<br>(941 to 1,520)	850.1<br>(672.5 to 1,086.1)	1,230<br>(971 to 1,560)	853.2<br>(671.9 to 1,078.5)	-13.0<br>(-31.9 to 13.4)	-6.4<br>(-15.3 to 3.0)

Appendix Table S3: All-age, and sub age groups LRI etiology counts for deaths and cases for global, seven GBD super-regions, 21 GBD regions, and countries, for 1990 and 2021, and % change

Location	Age Group	Aetiology	Episodes			Deaths		
			1990	2021	Percent Change	1990	2021	Percent Change
**Global**	All Ages	*Acinetobacter baumannii*	8310000 (7140000 to 9660000)	7880000 (6870000 to 9080000)	-5.0 (-10.9 to 1.4)	104000 (84900 to 130000)	75700 (64900 to 88300)	-27.0 (-37.3 to -16.4)
		Chlamydia spp.	20200000 (18100000 to 22500000)	19400000 (17700000 to 21200000)	-3.6 (-9.0 to 1.9)	91200 (79700 to 103000)	54000 (47600 to 60500)	-40.6 (-47.8 to -32.6)
		Enterobacter spp.	1250000 (1030000 to 1510000)	2400000 (2080000 to 2780000)	92.8 (77.5 to 108.2)	23800 (18400 to 31600)	29600 (25100 to 34000)	25.8 (0.5 to 50.9)
		Escherichia coli	4930000 (4310000 to 5590000)	7760000 (7030000 to 8560000)	57.5 (48.2 to 68.0)	95900 (82900 to 110000)	100000 (89000 to 112000)	4.9 (-7.1 to 18.4)
		Fungus	3570000 (3010000 to 4200000)	5620000 (4930000 to 6400000)	57.8 (47.6 to 69.2)	30400 (25300 to 36000)	45600 (39700 to 51900)	50.5 (34.5 to 66.4)
		Group B Streptococcus	3850000 (3390000 to 4390000)	6080000 (5540000 to 6700000)	58.2 (47.8 to 69.3)	64900 (55700 to 74800)	54700 (48600 to 61100)	-15.4 (-26.0 to -3.2)
		Haemophilus influenzae	16100000 (14400000 to 18000000)	18100000 (16700000 to 19600000)	12.5 (5.5 to 19.4)	133000 (114000 to 155000)	56800 (50100 to 63400)	-57.1 (-62.8 to -51.0)
		Influenza	21000000 (19400000 to 22700000)	14400000 (9810000 to 19400000)	-31.2 (-52.8 to -7.9)	274000 (246000 to 304000)	98200 (74300 to 126000)	-64.1 (-72.8 to -54.0)
		Klebsiella pneumoniae	13000000 (11900000 to 14200000)	15500000 (14400000 to 16800000)	19.8 (13.0 to 26.6)	239000 (213000 to 268000)	176000 (158000 to 194000)	-26.4 (-34.1 to -16.7)
		Legionella spp.	4130000 (3640000 to 4710000)	8960000 (8180000 to 9790000)	117.1 (103.3 to 130.6)	42200 (35800 to 50500)	68200 (60400 to 74800)	62.5 (37.9 to 84.4)
		Mycoplasma spp.	20100000 (18200000 to 22100000)	25300000 (23500000 to 27200000)	26.1 (20.4 to 32.0)	74300 (65400 to 85000)	58400 (51800 to 64900)	-21.3 (-30.1 to -11.5)
		Polymicrobial	942000 (704000 to 1270000)	1210000 (849000 to 1730000)	28.5 (15.0 to 42.1)	29200 (19100 to 43300)	19800 (14000 to 27500)	-31.1 (-45.9 to -14.2)
		Pseudomonas aeruginosa	6540000 (5980000 to 7170000)	13900000 (12900000 to 14900000)	112.5 (101.7 to 123.6)	89100 (80900 to 98400)	124000 (111000 to 134000)	38.9 (24.5 to 53.9)
		Respiratory syncytial virus	10600000 (9550000 to 11900000)	4590000 (3320000 to 6020000)	-56.7 (-67.9 to -44.8)	140000 (123000 to 159000)	31500 (23300 to 41600)	-77.4 (-83.0 to -70.7)
		Staphylococcus aureus	11800000 (11000000 to 12700000)	24300000 (22700000 to 25900000)	105.7 (96.3 to 115.4)	253000 (231000 to 275000)	424000 (380000 to 459000)	67.7 (52.6 to 82.5)
		Streptococcus pneumoniae	112000000 (104000000 to 120000000)	97900000 (92100000 to 104000000)	-12.5 (-16.8 to -8.0)	1030000 (924000 to 1160000)	505000 (454000 to 555000)	-50.7 (-56.4 to -44.6)
		Other viruses	38400000 (35500000 to 41700000)	46400000 (43600000 to 49300000)	21.0 (14.9 to 27.4)	181000 (162000 to 204000)	121000 (109000 to 133000)	-33.1 (-40.9 to -24.3)
		Other bacterial pathogens	17200000 (15700000 to 18900000)	23700000 (21500000 to 26000000)	37.7 (30.1 to 45.5)	119000 (106000 to 133000)	140000 (123000 to 156000)	17.8 (6.6 to 28.5)
	Under 5	*Acinetobacter baumannii*	496000 (360000 to 679000)	169000 (125000 to 232000)	-65.9 (-69.1 to -62.4)	46900 (29600 to 72900)	14000 (8460 to 22400)	-70.1 (-75.4 to -62.1)
		Chlamydia spp.	4500000 (3830000 to 5290000)	1750000 (1510000 to 2010000)	-61.1 (-64.2 to -57.8)	61600 (51300 to 71800)	17900 (14500 to 21900)	-70.8 (-76.0 to -64.1)

		Enterobacter spp.	249000 (190000 to 329000)	161000 (126000 to 207000)	-35.2 (-41.5 to -28.2)	14200 (9380 to 21600)	5970 (3920 to 8860)	-57.8 (-65.4 to -46.9)
		Escherichia coli	1120000 (923000 to 1360000)	693000 (589000 to 820000)	-37.9 (-43.6 to -31.9)	59000 (47800 to 72800)	23600 (18300 to 29700)	-59.9 (-67.3 to -50.5)
		Fungus	220000 (150000 to 311000)	117000 (81600 to 160000)	-46.7 (-52.4 to -40.8)	6320 (4160 to 9170)	2260 (1470 to 3290)	-64.0 (-71.5 to -55.0)
		Group B Streptococcus	879000 (719000 to 1050000)	516000 (436000 to 603000)	-41.1 (-46.1 to -35.2)	47300 (38200 to 56800)	18000 (14300 to 22300)	-61.9 (-68.7 to -53.1)
		Haemophilus influenzae	6550000 (5560000 to 7700000)	2780000 (2420000 to 3210000)	-57.5 (-61.0 to -53.6)	102000 (83800 to 123000)	19000 (14800 to 23800)	-81.3 (-84.9 to -76.8)
		Influenza	5830000 (5110000 to 6670000)	1410000 (1060000 to 1820000)	-75.9 (-81.2 to -70.0)	167000 (142000 to 196000)	27600 (19900 to 36800)	-83.4 (-87.7 to -78.2)
		Klebsiella pneumoniae	4220000 (3630000 to 4890000)	1580000 (1370000 to 1820000)	-62.4 (-65.3 to -59.6)	165000 (139000 to 193000)	46400 (36700 to 57300)	-71.8 (-76.8 to -65.1)
		Legionella spp.	224000 (158000 to 307000)	139000 (99900 to 189000)	-37.7 (-43.6 to -31.2)	14600 (9270 to 22000)	6090 (4000 to 9050)	-57.8 (-65.8 to -48.3)
		Mycoplasma spp.	4030000 (3470000 to 4640000)	2620000 (2280000 to 3010000)	-35.0 (-39.8 to -29.9)	46700 (39000 to 56700)	18500 (14700 to 22900)	-60.2 (-67.5 to -50.9)
		Polymicrobial	167000 (110000 to 246000)	83900 (55000 to 124000)	-49.7 (-54.5 to -44.9)	22900 (13200 to 36800)	9320 (5110 to 15300)	-59.2 (-66.5 to -50.3)
		Pseudomonas aeruginosa	1390000 (1190000 to 1620000)	1110000 (969000 to 1270000)	-20.4 (-27.0 to -13.8)	49300 (41800 to 57600)	23100 (18600 to 28200)	-53.0 (-61.2 to -41.6)
		Respiratory syncytial virus	8580000 (7580000 to 9760000)	2740000 (2040000 to 3530000)	-68.1 (-75.3 to -60.6)	133000 (117000 to 152000)	27800 (20300 to 37000)	-79.1 (-84.4 to -72.8)
		Staphylococcus aureus	2150000 (1880000 to 2470000)	1440000 (1250000 to 1650000)	-33.1 (-36.4 to -29.4)	111000 (94500 to 130000)	47900 (38400 to 59400)	-56.7 (-64.3 to -46.8)
		Streptococcus pneumoniae	40400000 (35700000 to 45800000)	11600000 (10200000 to 13300000)	-71.3 (-72.8 to -69.8)	721000 (621000 to 843000)	139000 (110000 to 171000)	-80.6 (-84.1 to -76.2)
		Other viruses	18700000 (16400000 to 21300000)	8350000 (7320000 to 9560000)	-55.4 (-57.6 to -53.0)	141000 (123000 to 162000)	43200 (34800 to 52600)	-69.3 (-74.7 to -62.4)
		Other bacterial pathogens	1340000 (1070000 to 1650000)	607000 (497000 to 738000)	-54.7 (-58.8 to -49.9)	35700 (27600 to 45600)	11600 (8570 to 15600)	-67.5 (-73.3 to -59.5)
	5-14 years	*Acinetobacter baumannii*	1140000 (883000 to 1450000)	581000 (464000 to 732000)	-49.0 (-52.4 to -45.6)	4260 (3310 to 5190)	1660 (1340 to 2000)	-60.9 (-66.3 to -53.6)
		Chlamydia spp.	2930000 (2300000 to 3700000)	1740000 (1390000 to 2140000)	-40.6 (-44.0 to -37.0)	2700 (2180 to 3230)	1180 (964 to 1390)	-56.2 (-62.2 to -48.6)
		Enterobacter spp.	141000 (106000 to 188000)	155000 (121000 to 197000)	10.2 (0.8 to 19.0)	496 (375 to 623)	376 (303 to 460)	-23.7 (-34.3 to -10.6)
		Escherichia coli	484000 (377000 to 608000)	444000 (354000 to 545000)	-8.1 (-13.9 to -2.3)	1760 (1420 to 2120)	1170 (976 to 1400)	-33.1 (-41.7 to -21.5)
		Fungus	589000 (446000 to 763000)	510000 (405000 to 643000)	-13.2 (-19.3 to -6.9)	2110 (1620 to 2620)	1310 (1050 to 1600)	-37.8 (-46.2 to -27.1)
		Group B Streptococcus	530000 (407000 to 671000)	515000 (412000 to 639000)	-2.7 (-9.7 to 4.6)	1370 (1080 to 1650)	934 (777 to 1100)	-31.4 (-40.7 to -19.7)
		Haemophilus influenzae	1840000 (1480000 to 2290000)	1500000 (1220000 to 1840000)	-18.6 (-23.1 to -13.9)	2550 (2100 to 2980)	1020 (859 to 1190)	-59.7 (-64.7 to -53.4)
		Influenza	2790000 (2260000 to 3400000)	1280000 (881000 to 1760000)	-54.1 (-65.6 to -40.9)	7560 (6310 to 8570)	2500 (1870 to 3190)	-66.9 (-75.3 to -57.3)

		Klebsiella pneumoniae	1440000 (1160000 to 1790000)	1110000 (906000 to 1350000)	-22.7 (-26.5 to -18.5)	5200 (4280 to 5960)	2790 (2370 to 3210)	-46.1 (-52.9 to -37.3)
		Legionella spp.	625000 (496000 to 780000)	706000 (571000 to 859000)	13.1 (5.4 to 21.4)	1540 (1250 to 1820)	1100 (923 to 1270)	-28.7 (-38.1 to -17.5)
		Mycoplasma spp.	4340000 (3440000 to 5350000)	3530000 (2850000 to 4270000)	-18.5 (-23.0 to -13.9)	4160 (3430 to 4740)	2300 (1960 to 2650)	-44.5 (-51.3 to -35.7)
		Polymicrobial	121000 (82800 to 173000)	91200 (60300 to 131000)	-24.5 (-31.4 to -17.7)	446 (311 to 634)	255 (175 to 361)	-42.8 (-51.0 to -32.4)
		Pseudomonas aeruginosa	758000 (599000 to 928000)	923000 (742000 to 1130000)	22.0 (15.3 to 28.9)	1890 (1570 to 2180)	1480 (1290 to 1700)	-21.2 (-31.0 to -8.4)
		Respiratory syncytial virus	456000 (362000 to 561000)	234000 (159000 to 328000)	-48.5 (-62.4 to -33.5)	559 (464 to 642)	185 (138 to 239)	-66.7 (-75.0 to -57.1)
		Staphylococcus aureus	1500000 (1220000 to 1850000)	1730000 (1410000 to 2080000)	15.3 (9.9 to 21.1)	7670 (6370 to 8670)	6080 (5190 to 6880)	-20.6 (-30.4 to -7.9)
		Streptococcus pneumoniae	16900000 (13800000 to 20500000)	10600000 (8700000 to 12700000)	-37.2 (-40.3 to -34.2)	36200 (30200 to 40700)	14700 (12500 to 16600)	-59.4 (-64.4 to -53.0)
		Other viruses	4720000 (3820000 to 5740000)	4830000 (3960000 to 5820000)	2.3 (-2.3 to 7.3)	3940 (3320 to 4420)	2450 (2110 to 2770)	-37.6 (-44.9 to -28.4)
		Other bacterial pathogens	2240000 (1790000 to 2780000)	1580000 (1280000 to 1950000)	-29.1 (-33.2 to -24.9)	4550 (3740 to 5250)	2290 (1900 to 2650)	-49.6 (-56.2 to -41.0)
	15-49 years	*Acinetobacter baumannii*	1790000 (1490000 to 2170000)	1620000 (1380000 to 1920000)	-9.5 (-14.9 to -3.8)	5970 (5050 to 7070)	5120 (4320 to 6060)	-14.1 (-22.9 to -4.2)
		Chlamydia spp.	4530000 (3890000 to 5240000)	4820000 (4190000 to 5490000)	6.7 (1.5 to 11.9)	3970 (3440 to 4550)	3890 (3370 to 4450)	-1.9 (-11.0 to 9.1)
		Enterobacter spp.	216000 (173000 to 265000)	419000 (351000 to 495000)	94.8 (81.1 to 108.9)	801 (658 to 971)	1460 (1230 to 1730)	82.8 (63.2 to 103.4)
		Escherichia coli	743000 (631000 to 874000)	1200000 (1060000 to 1370000)	62.3 (53.6 to 71.7)	2710 (2350 to 3100)	4240 (3710 to 4840)	56.9 (42.3 to 74.4)
		Fungus	903000 (732000 to 1090000)	1390000 (1170000 to 1630000)	54.4 (45.0 to 64.4)	3240 (2700 to 3820)	4680 (4000 to 5440)	44.7 (29.8 to 60.2)
		Group B Streptococcus	811000 (690000 to 976000)	1400000 (1220000 to 1610000)	72.7 (62.1 to 83.7)	2150 (1860 to 2470)	3470 (3050 to 3970)	61.9 (46.0 to 79.9)
		Haemophilus influenzae	2810000 (2430000 to 3200000)	4110000 (3650000 to 4620000)	46.6 (39.8 to 54.1)	4110 (3660 to 4570)	3850 (3450 to 4320)	-6.3 (-13.8 to 1.9)
		Influenza	4260000 (3780000 to 4790000)	3640000 (2490000 to 4980000)	-14.5 (-42.2 to 15.7)	12100 (11000 to 13100)	7780 (6110 to 9690)	-35.4 (-50.5 to -19.3)
		Klebsiella pneumoniae	2200000 (1940000 to 2480000)	3070000 (2750000 to 3420000)	39.4 (33.5 to 45.6)	7980 (7190 to 8790)	9880 (8860 to 11000)	23.9 (13.5 to 35.7)
		Legionella spp.	953000 (811000 to 1110000)	1900000 (1660000 to 2140000)	99.4 (87.2 to 110.9)	2730 (2390 to 3110)	4670 (4190 to 5220)	71.0 (56.4 to 87.7)
		Mycoplasma spp.	6630000 (5840000 to 7410000)	9710000 (8710000 to 10800000)	46.7 (40.5 to 53.8)	6650 (5990 to 7330)	8580 (7700 to 9510)	29.2 (18.2 to 41.1)
		Polymicrobial	187000 (132000 to 257000)	249000 (170000 to 351000)	32.8 (20.8 to 44.1)	654 (467 to 923)	853 (585 to 1230)	30.2 (16.1 to 46.7)
		Pseudomonas aeruginosa	1150000 (1010000 to 1310000)	2480000 (2200000 to 2780000)	114.8 (105.5 to 125.1)	3260 (2950 to 3580)	6200 (5670 to 6830)	90.4 (75.3 to 107.0)
		Respiratory syncytial virus	696000 (609000 to 786000)	675000 (447000 to 943000)	-2.8 (-36.2 to 32.1)	1010 (912 to 1110)	623 (482 to 783)	-38.2 (-52.4 to -22.7)

		Staphylococcus aureus	2280000 (2040000 to 2550000)	4650000 (4180000 to 5140000)	103.6 (96.3 to 111.0)	12500 (11600 to 13500)	23900 (21800 to 26300)	91.4 (76.8 to 108.7)
		Streptococcus pneumoniae	25900000 (23400000 to 28600000)	30000000 (27200000 to 33000000)	16.0 (11.8 to 20.6)	57000 (52800 to 61000)	52600 (48200 to 57900)	-7.6 (-14.8 to 0.6)
		Other viruses	7200000 (6480000 to 8000000)	13100000 (11800000 to 14400000)	81.6 (75.5 to 88.3)	6960 (6450 to 7450)	10200 (9450 to 11200)	47.2 (36.4 to 58.6)
		Other bacterial pathogens	3440000 (3000000 to 3910000)	4380000 (3850000 to 4980000)	27.6 (21.6 to 34.3)	6940 (6220 to 7720)	7980 (7010 to 8990)	15.1 (4.6 to 26.9)
	50-69 years	*Acinetobacter baumannii*	2930000 (2430000 to 3500000)	3020000 (2570000 to 3550000)	3.1 (-3.6 to 9.7)	18600 (15800 to 22100)	18400 (15700 to 21400)	-0.5 (-10.2 to 11.1)
		Chlamydia spp.	4490000 (3880000 to 5150000)	5440000 (4820000 to 6120000)	21.2 (14.3 to 27.9)	7290 (6340 to 8320)	8380 (7410 to 9420)	15.0 (4.2 to 27.8)
		Enterobacter spp.	370000 (297000 to 462000)	846000 (723000 to 1010000)	129.3 (112.3 to 147.5)	2490 (2040 to 3010)	5530 (4750 to 6360)	123.3 (101.1 to 148.1)
		Escherichia coli	1370000 (1170000 to 1580000)	2520000 (2220000 to 2840000)	84.3 (73.7 to 96.2)	9120 (7920 to 10400)	16600 (14800 to 18500)	82.9 (66.0 to 101.6)
		Fungus	1320000 (1100000 to 1600000)	2370000 (2050000 to 2750000)	79.3 (67.7 to 91.8)	8680 (7280 to 10300)	15100 (13100 to 17300)	74.5 (58.1 to 93.1)
		Group B Streptococcus	970000 (819000 to 1140000)	1940000 (1690000 to 2210000)	100.2 (87.0 to 113.0)	4670 (4050 to 5400)	9050 (8000 to 10100)	94.3 (75.2 to 114.7)
		Haemophilus influenzae	2910000 (2540000 to 3340000)	5150000 (4620000 to 5760000)	77.1 (68.2 to 87.2)	7680 (6880 to 8570)	9150 (8330 to 10100)	19.2 (9.6 to 29.4)
		Influenza	3780000 (3370000 to 4230000)	3520000 (2240000 to 4930000)	-6.6 (-40.0 to 28.0)	19400 (17500 to 21200)	14600 (10800 to 19100)	-24.8 (-45.0 to -1.3)
		Klebsiella pneumoniae	2850000 (2520000 to 3190000)	4820000 (4350000 to 5310000)	69.7 (61.5 to 78.5)	18800 (17000 to 20700)	30200 (27300 to 33100)	60.9 (48.0 to 76.2)
		Legionella spp.	1460000 (1260000 to 1690000)	3510000 (3120000 to 3910000)	140.2 (126.1 to 155.0)	7520 (6610 to 8490)	16100 (14600 to 17500)	114.3 (96.7 to 134.3)
		Mycoplasma spp.	3130000 (2750000 to 3520000)	5220000 (4690000 to 5770000)	67.1 (58.7 to 76.2)	5680 (5060 to 6320)	8590 (7740 to 9390)	51.4 (39.0 to 65.6)
		Polymicrobial	261000 (184000 to 365000)	394000 (269000 to 572000)	50.7 (35.9 to 65.3)	1690 (1220 to 2380)	2560 (1750 to 3770)	51.3 (33.6 to 72.1)
		Pseudomonas aeruginosa	1760000 (1560000 to 1970000)	4460000 (4040000 to 4920000)	154.0 (142.1 to 167.1)	8920 (8120 to 9800)	20700 (19000 to 22300)	131.9 (114.3 to 152.3)
		Respiratory syncytial virus	490000 (430000 to 552000)	507000 (327000 to 721000)	3.6 (-33.5 to 41.7)	1270 (1140 to 1410)	896 (639 to 1200)	-29.5 (-48.7 to -6.6)
		Staphylococcus aureus	2970000 (2660000 to 3270000)	7160000 (6500000 to 7840000)	141.5 (131.6 to 152.6)	29300 (27200 to 31600)	69200 (63900 to 74500)	136.1 (119.3 to 155.7)
		Streptococcus pneumoniae	16300000 (14700000 to 17900000)	23200000 (21100000 to 25300000)	42.5 (36.6 to 49.1)	65100 (60100 to 70100)	80200 (73000 to 86700)	23.3 (13.3 to 35.3)
		Other viruses	4230000 (3810000 to 4650000)	9680000 (8780000 to 10600000)	128.9 (120.4 to 138.4)	7330 (6840 to 7860)	14500 (13400 to 15600)	98.4 (84.4 to 113.5)
		Other bacterial pathogens	5210000 (4620000 to 5880000)	7700000 (6830000 to 8670000)	47.9 (39.5 to 56.5)	19200 (17200 to 21300)	26800 (23500 to 29800)	39.2 (26.9 to 52.9)
	70+ years	*Acinetobacter baumannii*	1940000 (1620000 to 2340000)	2490000 (2110000 to 2940000)	28.5 (18.1 to 40.4)	28700 (24200 to 34100)	36500 (31000 to 42500)	27.5 (15.8 to 41.3)
		Chlamydia spp.	3710000 (3190000 to 4300000)	5660000 (4950000 to 6480000)	52.8 (41.8 to 65.2)	15700 (13700 to 17800)	22700 (19700 to 25100)	44.9 (32.6 to 58.6)

		Enterobacter spp.	274000 (220000 to 338000)	822000 (695000 to 963000)	201.3 (174.9 to 229.2)	5750 (4820 to 6810)	16200 (13600 to 18500)	182.8 (158.1 to 209.5)
		Escherichia coli	1220000 (1030000 to 1410000)	2900000 (2540000 to 3320000)	138.5 (123.1 to 155.3)	23300 (20400 to 26200)	54600 (47900 to 60300)	135.0 (115.1 to 153.9)
		Fungus	534000 (436000 to 641000)	1230000 (1040000 to 1440000)	131.3 (113.5 to 149.4)	10000 (8590 to 11700)	22200 (19000 to 25600)	121.9 (102.2 to 141.5)
		Group B Streptococcus	658000 (561000 to 766000)	1710000 (1500000 to 1960000)	160.4 (141.3 to 180.5)	9480 (8190 to 10800)	23300 (20200 to 26000)	146.1 (123.8 to 168.1)
		Haemophilus influenzae	1990000 (1730000 to 2270000)	4550000 (4030000 to 5110000)	128.6 (114.1 to 144.3)	16400 (14600 to 18000)	23800 (20700 to 26100)	45.3 (35.1 to 55.6)
		Influenza	4320000 (3850000 to 4820000)	4580000 (2930000 to 6370000)	6.1 (-29.7 to 44.9)	68000 (61500 to 74300)	45800 (34200 to 60300)	-32.6 (-50.2 to -12.2)
		Klebsiella pneumoniae	2270000 (2020000 to 2570000)	4940000 (4400000 to 5550000)	118.1 (103.9 to 132.5)	42900 (38600 to 46900)	86600 (75900 to 94700)	102.1 (87.4 to 116.6)
		Legionella spp.	870000 (754000 to 1010000)	2710000 (2410000 to 3050000)	212.4 (191.9 to 234.4)	15800 (14000 to 17600)	40300 (34700 to 44400)	154.5 (135.5 to 174.7)
		Mycoplasma spp.	1990000 (1750000 to 2240000)	4260000 (3790000 to 4820000)	114.5 (100.9 to 128.3)	11100 (9840 to 12200)	20400 (17700 to 22400)	83.8 (71.2 to 97.4)
		Polymicrobial	207000 (143000 to 297000)	397000 (261000 to 597000)	91.1 (69.7 to 113.4)	3480 (2400 to 5220)	6820 (4380 to 10600)	95.2 (73.5 to 119.1)
		Pseudomonas aeruginosa	1480000 (1320000 to 1670000)	4920000 (4430000 to 5530000)	232.7 (214.2 to 251.7)	25800 (23200 to 27900)	72100 (62300 to 78800)	180.1 (160.2 to 198.9)
		Respiratory syncytial virus	387000 (341000 to 442000)	436000 (288000 to 609000)	12.7 (-24.5 to 51.5)	3630 (3200 to 4020)	1990 (1500 to 2630)	-45.0 (-59.4 to -28.9)
		Staphylococcus aureus	2940000 (2620000 to 3270000)	9370000 (8420000 to 10500000)	218.6 (201.5 to 237.1)	92400 (83300 to 99500)	277000 (241000 to 301000)	199.4 (178.9 to 218.0)
		Streptococcus pneumoniae	12400000 (11100000 to 13800000)	22500000 (20100000 to 25200000)	81.3 (71.0 to 92.8)	149000 (136000 to 161000)	219000 (194000 to 239000)	46.6 (36.9 to 56.6)
		Other viruses	3550000 (3210000 to 3940000)	10500000 (9430000 to 11700000)	195.4 (182.1 to 209.5)	21700 (19600 to 23200)	50200 (43800 to 54700)	131.1 (117.1 to 144.3)
		Other bacterial pathogens	5000000 (4370000 to 5650000)	9440000 (8200000 to 10700000)	88.7 (75.8 to 103.0)	52700 (46400 to 58900)	91700 (78200 to 103000)	73.9 (60.4 to 87.1)
**Central Europe, Eastern Europe, and Central Asia**	All Ages	*Acinetobacter baumannii*	116000 (101000 to 130000)	71500 (63300 to 80000)	-38.1 (-41.0 to -34.7)	2250 (1770 to 2890)	1600 (1400 to 1840)	-28.2 (-39.4 to -15.8)
		Chlamydia spp.	435000 (401000 to 473000)	266000 (249000 to 284000)	-38.9 (-41.6 to -35.9)	2330 (2170 to 2500)	1460 (1340 to 1570)	-37.5 (-42.8 to -32.2)
		Enterobacter spp.	50100 (43700 to 56800)	57400 (51900 to 63200)	14.7 (7.5 to 21.8)	955 (775 to 1200)	1240 (1110 to 1390)	31.1 (7.9 to 53.5)
		Escherichia coli	156000 (141000 to 171000)	152000 (142000 to 163000)	-2.3 (-7.1 to 2.6)	3190 (2910 to 3500)	3440 (3190 to 3690)	8.2 (-0.8 to 17.4)
		Fungus	112000 (98700 to 126000)	108000 (98300 to 119000)	-3.7 (-8.9 to 1.5)	1160 (1020 to 1290)	1890 (1720 to 2060)	63.4 (49.6 to 77.9)
		Group B Streptococcus	130000 (118000 to 145000)	127000 (118000 to 137000)	-2.3 (-7.6 to 3.4)	2270 (2090 to 2490)	2070 (1920 to 2240)	-8.6 (-16.9 to 0.3)
		Haemophilus influenzae	585000 (543000 to 634000)	412000 (389000 to 438000)	-29.6 (-33.1 to -26.0)	4810 (4400 to 5270)	2230 (2080 to 2380)	-53.6 (-58.0 to -49.1)
		Influenza	749000 (702000 to 797000)	83100 (51900 to 131000)	-88.9 (-93.0 to -82.4)	9690 (9190 to 10200)	970 (604 to 1490)	-90.0 (-93.8 to -84.3)

		Klebsiella pneumoniae	382000 (358000 to 409000)	288000 (273000 to 306000)	-24.5 (-27.5 to -21.3)	7430 (6990 to 7910)	6160 (5770 to 6540)	-17.1 (-23.5 to -10.4)
		Legionella spp.	232000 (210000 to 255000)	275000 (256000 to 296000)	18.8 (12.9 to 25.1)	2110 (1880 to 2380)	3720 (3460 to 3990)	76.9 (58.1 to 94.9)
		Mycoplasma spp.	677000 (626000 to 734000)	517000 (487000 to 549000)	-23.6 (-26.7 to -20.5)	2660 (2490 to 2850)	2450 (2290 to 2630)	-7.6 (-15.4 to -0.1)
		Polymicrobial	22000 (15200 to 32200)	18000 (11700 to 28200)	-18.6 (-27.0 to -10.9)	1100 (686 to 1670)	629 (427 to 897)	-41.8 (-54.4 to -25.1)
		Pseudomonas aeruginosa	326000 (303000 to 351000)	392000 (369000 to 416000)	20.3 (15.7 to 25.3)	3960 (3730 to 4220)	5990 (5570 to 6360)	51.4 (39.5 to 63.2)
		Respiratory syncytial virus	482000 (436000 to 534000)	33800 (22100 to 50500)	-93.0 (-95.4 to -89.6)	6260 (5890 to 6680)	301 (177 to 454)	-95.2 (-97.2 to -92.6)
		Staphylococcus aureus	488000 (460000 to 517000)	593000 (560000 to 629000)	21.5 (17.8 to 25.3)	10200 (9740 to 10600)	18000 (16800 to 19100)	76.8 (66.3 to 88.3)
		Streptococcus pneumoniae	3550000 (3340000 to 3770000)	1840000 (1740000 to 1940000)	-48.2 (-50.3 to -46.2)	35300 (33900 to 37000)	19800 (18500 to 21000)	-44.1 (-48.2 to -40.0)
		Other viruses	1800000 (1680000 to 1930000)	1290000 (1220000 to 1360000)	-28.3 (-31.7 to -25.1)	8220 (7830 to 8650)	5910 (5530 to 6290)	-28.1 (-33.5 to -22.5)
		Other bacterial pathogens	531000 (480000 to 583000)	435000 (391000 to 482000)	-18.0 (-21.1 to -14.8)	4010 (3640 to 4450)	4970 (4430 to 5530)	23.9 (13.7 to 33.5)
	Under 5	*Acinetobacter baumannii*	6690 (4820 to 9110)	1570 (1120 to 2190)	-76.5 (-79.0 to -74.0)	1100 (641 to 1710)	288 (162 to 486)	-73.9 (-78.7 to -67.6)
		Chlamydia spp.	81000 (70600 to 94000)	18800 (16500 to 21500)	-76.8 (-79.2 to -74.2)	1430 (1290 to 1570)	336 (275 to 408)	-76.5 (-80.7 to -71.5)
		Enterobacter spp.	7580 (5870 to 9580)	3070 (2430 to 3880)	-59.4 (-64.0 to -54.5)	531 (365 to 767)	181 (121 to 265)	-65.8 (-72.0 to -58.4)
		Escherichia coli	27100 (22900 to 32000)	10700 (9270 to 12300)	-60.4 (-65.0 to -55.8)	1790 (1560 to 2080)	583 (473 to 721)	-67.5 (-73.1 to -60.7)
		Fungus	5280 (3740 to 7320)	1730 (1250 to 2340)	-67.1 (-71.2 to -62.7)	169 (120 to 229)	47.7 (33.1 to 65.8)	-71.7 (-77.3 to -64.8)
		Group B Streptococcus	23300 (19500 to 27500)	8660 (7410 to 10100)	-62.7 (-66.4 to -58.6)	1520 (1350 to 1730)	475 (388 to 576)	-68.7 (-74.0 to -62.2)
		Haemophilus influenzae	199000 (173000 to 229000)	49300 (43400 to 56100)	-75.2 (-77.7 to -72.7)	3380 (3000 to 3810)	381 (316 to 456)	-88.7 (-90.7 to -86.3)
		Influenza	168000 (149000 to 189000)	5500 (3510 to 8110)	-96.7 (-97.9 to -95.3)	5210 (4790 to 5650)	145 (76 to 237)	-97.2 (-98.5 to -95.3)
		Klebsiella pneumoniae	101000 (88800 to 115000)	22600 (20100 to 25700)	-77.5 (-79.4 to -75.6)	4570 (4160 to 4980)	1000 (824 to 1200)	-78.0 (-81.8 to -73.4)
		Legionella spp.	8610 (6170 to 11900)	3300 (2410 to 4540)	-61.6 (-65.4 to -57.4)	617 (444 to 835)	208 (150 to 278)	-66.1 (-72.5 to -59.3)
		Mycoplasma spp.	115000 (100000 to 133000)	48500 (42800 to 54900)	-57.7 (-61.7 to -53.5)	1370 (1230 to 1540)	453 (375 to 544)	-66.9 (-72.8 to -59.8)
		Polymicrobial	4040 (2550 to 6080)	1370 (827 to 2120)	-66.1 (-69.4 to -62.2)	908 (507 to 1470)	306 (160 to 511)	-66.3 (-72.2 to -59.0)
		Pseudomonas aeruginosa	52500 (45600 to 60700)	27200 (24200 to 30700)	-48.2 (-53.2 to -43.0)	2010 (1830 to 2220)	780 (648 to 924)	-61.2 (-68.0 to -53.0)
		Respiratory syncytial virus	369000 (329000 to 416000)	18000 (11600 to 26700)	-95.1 (-96.8 to -93.0)	5900 (5540 to 6330)	228 (123 to 371)	-96.1 (-97.9 to -93.7)

		Staphylococcus aureus	67900 (59900 to 76700)	28100 (24900 to 31900)	-58.6 (-61.4 to -55.7)	3950 (3640 to 4260)	1370 (1140 to 1630)	-65.3 (-71.2 to -58.2)
		Streptococcus pneumoniae	1110000 (988000 to 1250000)	158000 (141000 to 180000)	-85.7 (-86.6 to -84.9)	22000 (20700 to 23600)	2680 (2190 to 3260)	-87.8 (-90.2 to -85.1)
		Other viruses	774000 (683000 to 870000)	192000 (171000 to 218000)	-75.1 (-76.7 to -73.4)	6060 (5690 to 6470)	1320 (1110 to 1570)	-78.2 (-81.8 to -74.0)
		Other bacterial pathogens	30800 (24600 to 37900)	8460 (6880 to 10200)	-72.5 (-75.5 to -68.9)	980 (763 to 1260)	252 (181 to 346)	-74.3 (-79.1 to -68.7)
	5-14 years	*Acinetobacter baumannii*	13700 (10900 to 17000)	4820 (3930 to 5900)	-64.7 (-66.9 to -62.4)	56.1 (48.4 to 64.6)	24.6 (20.8 to 29.3)	-56.1 (-61.6 to -49.5)
		Chlamydia spp.	58000 (46900 to 72000)	21400 (17700 to 26000)	-63.0 (-65.0 to -60.8)	54.1 (48.9 to 60.1)	22.6 (19.7 to 25.9)	-58.2 (-62.9 to -52.8)
		Enterobacter spp.	5000 (3920 to 6330)	3290 (2620 to 4100)	-34.1 (-38.8 to -29.7)	16.1 (13.8 to 18.7)	11.9 (10.2 to 13.8)	-26.0 (-34.6 to -16.5)
		Escherichia coli	13300 (10700 to 16400)	7510 (6160 to 9010)	-43.4 (-46.8 to -39.8)	46.3 (41.8 to 51.1)	29.7 (26.3 to 33.7)	-35.8 (-42.9 to -28.2)
		Fungus	16000 (12700 to 20000)	8590 (6970 to 10500)	-46.4 (-49.6 to -42.9)	55.1 (48.2 to 62.3)	33.6 (29 to 38.7)	-39.0 (-45.7 to -31.6)
		Group B Streptococcus	16500 (13200 to 20300)	9710 (7950 to 11800)	-40.9 (-44.7 to -37.1)	39.8 (35.5 to 45.1)	26.6 (23.3 to 30.6)	-33.1 (-40.5 to -24.7)
		Haemophilus influenzae	65500 (54000 to 80100)	31200 (25600 to 38000)	-52.4 (-54.5 to -50.2)	83.1 (76.8 to 89.7)	29.1 (26.3 to 32.4)	-65.0 (-68.2 to -61.5)
		Influenza	93600 (77000 to 113000)	6620 (4080 to 10200)	-92.9 (-95.4 to -89.4)	235 (222 to 249)	16.9 (9.61 to 26.7)	-92.8 (-95.9 to -88.5)
		Klebsiella pneumoniae	39100 (32300 to 48200)	18600 (15500 to 22400)	-52.5 (-54.5 to -50.3)	136 (127 to 144)	72.3 (64.9 to 80.9)	-46.6 (-51.8 to -40.6)
		Legionella spp.	31100 (25200 to 38600)	19200 (15800 to 23300)	-38.0 (-41.7 to -34.2)	66.7 (59.7 to 74.5)	43 (38.5 to 47.9)	-35.4 (-42.2 to -27.8)
		Mycoplasma spp.	146000 (120000 to 179000)	68400 (56000 to 83000)	-53.2 (-55.2 to -51.0)	130 (120 to 139)	65.4 (58.8 to 73.1)	-49.5 (-54.4 to -43.7)
		Polymicrobial	2360 (1510 to 3560)	1160 (713 to 1770)	-50.9 (-54.8 to -47.1)	8.84 (5.92 to 13.1)	5.11 (3.29 to 7.72)	-42.3 (-49.3 to -34.5)
		Pseudomonas aeruginosa	34600 (28000 to 42100)	23500 (19200 to 28600)	-31.9 (-35.0 to -28.4)	75.9 (70.6 to 82)	56.3 (50.8 to 62.5)	-25.7 (-32.8 to -17.6)
		Respiratory syncytial virus	24000 (19400 to 29600)	1830 (1120 to 2890)	-92.4 (-95.1 to -88.5)	25.3 (23.3 to 27.5)	1.88 (1.08 to 2.94)	-92.6 (-95.7 to -88.4)
		Staphylococcus aureus	55200 (45200 to 67200)	37900 (31300 to 45800)	-31.2 (-34.0 to -28.6)	258 (244 to 271)	199 (180 to 221)	-22.9 (-30.4 to -14.7)
		Streptococcus pneumoniae	534000 (441000 to 650000)	191000 (157000 to 231000)	-64.3 (-65.7 to -62.9)	1070 (1020 to 1110)	400 (362 to 445)	-62.4 (-65.9 to -58.3)
		Other viruses	233000 (191000 to 286000)	130000 (107000 to 158000)	-44.3 (-46.5 to -42.2)	169 (161 to 177)	96.8 (88.4 to 107)	-42.7 (-47.7 to -37.0)
		Other bacterial pathogens	58200 (47400 to 71500)	25000 (20200 to 30400)	-57.1 (-59.2 to -54.9)	115 (104 to 126)	54.3 (47.1 to 61.9)	-52.5 (-57.4 to -46.8)
	15-49 years	*Acinetobacter baumannii*	15900 (13500 to 18600)	10600 (9130 to 12200)	-33.2 (-37.0 to -28.9)	152 (132 to 174)	154 (133 to 178)	0.9 (-9.2 to 10.8)
		Chlamydia spp.	68500 (60900 to 77200)	50400 (45200 to 56000)	-26.4 (-30.2 to -22.3)	163 (149 to 179)	189 (168 to 210)	15.9 (6.3 to 26.1)

		Enterobacter spp.	6030 (5130 to 7030)	7770 (6800 to 8820)	29.0 (20.8 to 37.1)	54.6 (47.5 to 62.4)	114 (99.7 to 129)	109.6 (89.3 to 131.4)
		Escherichia coli	15900 (14100 to 18000)	17600 (16000 to 19500)	10.7 (4.8 to 17.0)	149 (136 to 163)	262 (238 to 289)	76.1 (60.0 to 91.2)
		Fungus	19200 (16600 to 22000)	20100 (17900 to 22600)	5.1 (-0.8 to 11.7)	177 (157 to 198)	298 (262 to 333)	68.1 (53.5 to 83.6)
		Group B Streptococcus	19800 (17400 to 22800)	22900 (20500 to 25600)	15.7 (8.6 to 22.6)	132 (117 to 147)	245 (219 to 274)	86.6 (68.9 to 104.9)
		Haemophilus influenzae	79100 (71100 to 87200)	77100 (70500 to 84400)	-2.5 (-6.6 to 2.2)	282 (265 to 299)	348 (320 to 378)	23.5 (13.8 to 33.7)
		Influenza	113000 (103000 to 124000)	16200 (9990 to 25600)	-85.6 (-91.2 to -77.2)	788 (754 to 820)	169 (94.3 to 279)	-78.6 (-88.0 to -64.4)
		Klebsiella pneumoniae	46800 (42500 to 51300)	44100 (40400 to 47900)	-5.7 (-9.4 to -1.3)	436 (414 to 458)	662 (608 to 719)	52.1 (40.3 to 65.5)
		Legionella spp.	38000 (33400 to 42800)	47700 (43400 to 52300)	25.8 (18.7 to 33.6)	243 (220 to 267)	512 (468 to 562)	110.8 (91.7 to 129.9)
		Mycoplasma spp.	176000 (160000 to 194000)	167000 (153000 to 181000)	-5.3 (-9.5 to -0.8)	435 (411 to 461)	675 (617 to 732)	55.1 (42.0 to 67.9)
		Polymicrobial	2790 (1800 to 4170)	2650 (1650 to 4160)	-5.3 (-12.5 to 2.3)	26.6 (17.5 to 39.9)	39.2 (24.4 to 61.9)	46.8 (31.9 to 62.2)
		Pseudomonas aeruginosa	42100 (38000 to 46800)	56800 (51800 to 61900)	35.0 (29.1 to 41.4)	272 (255 to 289)	611 (560 to 664)	125.0 (106.8 to 144.6)
		Respiratory syncytial virus	29300 (26200 to 32600)	4620 (2820 to 7490)	-84.2 (-90.5 to -74.6)	93.3 (87 to 100)	22.7 (12.6 to 38.3)	-75.6 (-86.4 to -59.1)
		Staphylococcus aureus	66700 (61100 to 73000)	89700 (82800 to 97200)	34.5 (29.2 to 39.8)	882 (850 to 913)	1930 (1790 to 2090)	119.0 (102.3 to 136.1)
		Streptococcus pneumoniae	642000 (590000 to 696000)	473000 (439000 to 511000)	-26.3 (-28.9 to -23.2)	3530 (3450 to 3600)	4320 (3980 to 4690)	22.2 (12.6 to 32.5)
		Other viruses	285000 (262000 to 310000)	324000 (300000 to 351000)	14.0 (9.6 to 18.6)	616 (598 to 636)	1190 (1090 to 1280)	92.5 (77.4 to 108.3)
		Other bacterial pathogens	69500 (60900 to 78600)	59700 (52500 to 67500)	-14.1 (-18.0 to -9.6)	365 (331 to 399)	504 (444 to 566)	38.2 (27.1 to 49.9)
	50-69 years	*Acinetobacter baumannii*	46800 (40400 to 53900)	29900 (25900 to 34100)	-36.0 (-38.9 to -32.8)	426 (378 to 483)	458 (403 to 524)	7.6 (-1.1 to 16.4)
		Chlamydia spp.	124000 (112000 to 139000)	88200 (79800 to 97500)	-29.0 (-32.0 to -25.8)	279 (254 to 303)	338 (307 to 370)	21.4 (12.5 to 30.7)
		Enterobacter spp.	18700 (16200 to 21700)	23300 (20700 to 26300)	24.9 (17.2 to 32.2)	159 (138 to 180)	363 (326 to 404)	128.8 (108.2 to 150.2)
		Escherichia coli	53600 (47500 to 60000)	56800 (51600 to 62700)	6.1 (1.4 to 10.9)	472 (433 to 514)	889 (819 to 964)	88.5 (74.3 to 103.3)
		Fungus	51400 (45000 to 58400)	52300 (46700 to 58700)	1.8 (-3.5 to 7.3)	446 (398 to 497)	811 (726 to 898)	81.7 (67.9 to 97.2)
		Group B Streptococcus	43000 (37800 to 49000)	47900 (43000 to 53400)	11.3 (5.5 to 16.9)	269 (243 to 300)	540 (492 to 590)	101.2 (84.5 to 119.0)
		Haemophilus influenzae	148000 (135000 to 162000)	144000 (132000 to 157000)	-2.7 (-6.3 to 1.0)	497 (471 to 526)	650 (602 to 698)	30.9 (21.3 to 41.3)
		Influenza	181000 (167000 to 197000)	25100 (15100 to 40800)	-86.1 (-91.7 to -77.4)	1190 (1130 to 1240)	243 (137 to 399)	-79.6 (-88.4 to -66.0)

		Klebsiella pneumoniae	110000 (102000 to 120000)	104000 (96300 to 114000)	-5.3 (-8.2 to -2.2)	966 (922 to 1010)	1630 (1520 to 1750)	69.2 (57.6 to 81.3)
		Legionella spp.	102000 (90800 to 114000)	126000 (114000 to 138000)	22.9 (16.6 to 29.4)	613 (559 to 670)	1380 (1280 to 1500)	126.0 (108.1 to 145.2)
		Mycoplasma spp.	151000 (137000 to 165000)	135000 (124000 to 148000)	-10.1 (-13.5 to -6.7)	350 (327 to 373)	559 (516 to 603)	59.9 (47.7 to 72.2)
		Polymicrobial	7130 (4700 to 10900)	6540 (4110 to 10300)	-8.7 (-15.4 to -1.6)	64 (42.1 to 96.7)	102 (63.9 to 164)	58.7 (43.4 to 74.9)
		Pseudomonas aeruginosa	114000 (103000 to 125000)	148000 (135000 to 161000)	30.0 (24.9 to 35.3)	688 (646 to 730)	1670 (1550 to 1790)	143.1 (126.7 to 161.6)
		Respiratory syncytial virus	35900 (32100 to 39900)	5380 (3130 to 8950)	-85.0 (-91.0 to -75.3)	107 (98 to 117)	24 (13.3 to 40.4)	-77.6 (-87.5 to -62.6)
		Staphylococcus aureus	156000 (144000 to 169000)	204000 (187000 to 222000)	30.7 (26.8 to 34.8)	1940 (1870 to 2010)	4670 (4360 to 4980)	140.7 (124.7 to 157.1)
		Streptococcus pneumoniae	734000 (676000 to 792000)	543000 (500000 to 591000)	-26.1 (-28.1 to -24.0)	3800 (3700 to 3890)	4960 (4610 to 5310)	30.5 (21.7 to 40.1)
		Other viruses	293000 (271000 to 317000)	338000 (312000 to 368000)	15.3 (11.8 to 18.5)	595 (575 to 616)	1260 (1180 to 1350)	112.6 (98.7 to 128.0)
		Other bacterial pathogens	192000 (171000 to 215000)	161000 (141000 to 182000)	-16.4 (-19.8 to -13.3)	952 (867 to 1040)	1390 (1230 to 1560)	46.4 (35.9 to 57.6)
	70+ years	*Acinetobacter baumannii*	32500 (27700 to 37900)	24600 (21300 to 28300)	-24.3 (-28.3 to -20.1)	514 (449 to 583)	676 (580 to 765)	31.5 (21.4 to 41.7)
		Chlamydia spp.	103000 (91400 to 116000)	87100 (78400 to 97200)	-15.7 (-19.7 to -11.5)	409 (369 to 444)	572 (511 to 622)	40.0 (30.0 to 50.5)
		Enterobacter spp.	12800 (10800 to 15000)	19900 (17500 to 22700)	56.1 (45.6 to 67.0)	195 (168 to 225)	571 (496 to 635)	193.2 (165.4 to 221.8)
		Escherichia coli	46000 (40500 to 52600)	59600 (53900 to 66600)	29.6 (23.0 to 36.1)	723 (658 to 792)	1680 (1510 to 1810)	132.2 (114.5 to 150.1)
		Fungus	20000 (17200 to 23200)	25000 (22100 to 28300)	24.8 (17.6 to 32.6)	310 (273 to 350)	700 (620 to 779)	125.8 (108.8 to 144.9)
		Group B Streptococcus	27700 (24400 to 31500)	38000 (34300 to 42200)	37.7 (29.4 to 45.3)	309 (276 to 345)	783 (699 to 852)	153.6 (132.9 to 176.0)
		Haemophilus influenzae	93600 (84400 to 104000)	110000 (101000 to 122000)	17.9 (13.2 to 23.0)	564 (523 to 601)	818 (743 to 874)	44.9 (36.4 to 54.4)
		Influenza	193000 (175000 to 213000)	29700 (17600 to 48100)	-84.6 (-90.6 to -75.1)	2280 (2130 to 2400)	397 (233 to 623)	-82.6 (-89.7 to -72.4)
		Klebsiella pneumoniae	85000 (77100 to 93800)	98600 (90700 to 109000)	16.1 (11.7 to 20.2)	1330 (1240 to 1400)	2780 (2520 to 2980)	109.8 (96.9 to 122.0)
		Legionella spp.	51800 (45600 to 58600)	79100 (72100 to 87700)	53.0 (44.2 to 61.8)	565 (509 to 622)	1570 (1410 to 1690)	177.8 (155.5 to 200.8)
		Mycoplasma spp.	89300 (80300 to 100000)	97600 (89000 to 109000)	9.3 (4.6 to 14.3)	372 (340 to 401)	702 (635 to 756)	88.7 (75.9 to 101.4)
		Polymicrobial	5700 (3650 to 8940)	6310 (3830 to 10200)	10.2 (1.0 to 19.5)	90.1 (57.9 to 140)	177 (107 to 292)	95.3 (75.7 to 116.9)
		Pseudomonas aeruginosa	83200 (75500 to 93100)	137000 (126000 to 151000)	64.4 (57.7 to 72.2)	914 (848 to 974)	2870 (2580 to 3080)	214.6 (192.6 to 237.5)
		Respiratory syncytial virus	23700 (20900 to 26900)	3970 (2330 to 6420)	-83.3 (-89.9 to -72.4)	129 (117 to 141)	24 (13.8 to 38.7)	-81.4 (-89.2 to -70.4)

		Staphylococcus aureus	142000 (129000 to 156000)	233000 (214000 to 256000)	64.0 (58.7 to 69.7)	3170 (2970 to 3320)	9870 (8890 to 10500)	211.0 (192.8 to 229.5)
		Streptococcus pneumoniae	529000 (482000 to 584000)	472000 (435000 to 521000)	-10.7 (-13.6 to -7.6)	4900 (4610 to 5100)	7410 (6710 to 7870)	51.1 (43.0 to 59.4)
		Other viruses	210000 (190000 to 232000)	302000 (278000 to 333000)	43.7 (39.0 to 48.7)	776 (726 to 812)	2040 (1840 to 2170)	162.6 (148.2 to 178.2)
		Other bacterial pathogens	180000 (158000 to 205000)	181000 (160000 to 206000)	0.7 (-3.5 to 5.2)	1600 (1440 to 1760)	2770 (2400 to 3090)	72.5 (61.0 to 84.2)
Central Asia	All Ages	*Acinetobacter baumannii*	27100 (23600 to 30800)	23700 (20900 to 26800)	-12.2 (-18.8 to -5.6)	1150 (777 to 1680)	549 (419 to 744)	-51.8 (-60.6 to -41.3)
		Chlamydia spp.	110000 (100000 to 120000)	77200 (71500 to 83200)	-29.4 (-34.2 to -24.5)	1330 (1200 to 1450)	511 (442 to 596)	-61.5 (-67.0 to -55.3)
		Enterobacter spp.	9450 (8000 to 11000)	13800 (12300 to 15500)	46.1 (33.6 to 59.7)	466 (330 to 659)	311 (247 to 394)	-32.3 (-45.6 to -17.3)
		Escherichia coli	32300 (28900 to 36100)	39900 (36900 to 43400)	23.7 (14.7 to 33.2)	1610 (1410 to 1850)	971 (842 to 1120)	-39.4 (-47.8 to -29.6)
		Fungus	20400 (17600 to 23300)	28200 (25200 to 31700)	38.7 (28.2 to 49.3)	299 (247 to 356)	348 (298 to 400)	16.9 (0.8 to 35.4)
		Group B Streptococcus	28400 (25400 to 31700)	34000 (31500 to 37100)	20.1 (10.4 to 30.0)	1320 (1170 to 1500)	682 (587 to 794)	-48.3 (-55.8 to -39.2)
		Haemophilus influenzae	146000 (133000 to 160000)	102000 (95200 to 110000)	-29.8 (-34.7 to -25.0)	2770 (2450 to 3130)	559 (483 to 644)	-79.8 (-82.9 to -76.2)
		Influenza	165000 (154000 to 178000)	25300 (15000 to 39700)	-84.7 (-90.9 to -76.1)	4600 (4230 to 4980)	357 (197 to 576)	-92.2 (-95.6 to -87.4)
		Klebsiella pneumoniae	92300 (85500 to 101000)	75800 (71200 to 80800)	-17.8 (-22.7 to -12.6)	3980 (3630 to 4340)	1690 (1470 to 1930)	-57.6 (-63.4 to -51.1)
		Legionella spp.	33200 (29800 to 36900)	50900 (46900 to 55100)	53.3 (42.2 to 63.9)	669 (525 to 852)	593 (512 to 687)	-10.5 (-26.4 to 8.0)
		Mycoplasma spp.	158000 (145000 to 172000)	144000 (134000 to 154000)	-8.9 (-14.1 to -3.6)	1310 (1180 to 1460)	727 (636 to 832)	-44.3 (-52.4 to -35.4)
		Polymicrobial	5550 (4050 to 7670)	5580 (3850 to 8140)	0.3 (-15.7 to 14.4)	749 (433 to 1200)	341 (201 to 537)	-54.2 (-62.4 to -44.4)
		Pseudomonas aeruginosa	57600 (53100 to 62400)	84500 (79100 to 90200)	46.9 (38.0 to 55.9)	1750 (1590 to 1930)	1310 (1150 to 1480)	-25.2 (-35.4 to -13.7)
		Respiratory syncytial virus	157000 (144000 to 173000)	14900 (8500 to 23800)	-90.5 (-94.6 to -85.1)	4340 (4030 to 4710)	223 (116 to 373)	-94.9 (-97.3 to -91.4)
		Staphylococcus aureus	89700 (84200 to 95600)	140000 (132000 to 149000)	56.2 (49.3 to 63.8)	3790 (3500 to 4080)	3490 (3110 to 3880)	-7.8 (-18.9 to 3.8)
		Streptococcus pneumoniae	924000 (867000 to 986000)	443000 (418000 to 468000)	-52.0 (-54.5 to -49.7)	18700 (17500 to 20100)	4730 (4130 to 5430)	-74.7 (-78.4 to -70.7)
		Other viruses	453000 (421000 to 488000)	304000 (286000 to 323000)	-32.8 (-36.6 to -28.9)	4610 (4290 to 4960)	1690 (1460 to 1970)	-63.4 (-68.7 to -57.3)
		Other bacterial pathogens	94500 (86200 to 104000)	99500 (90000 to 110000)	5.3 (-0.6 to 11.8)	1230 (1040 to 1460)	923 (802 to 1060)	-24.5 (-35.4 to -12.8)
	Under 5	*Acinetobacter baumannii*	3970 (2840 to 5480)	1160 (827 to 1620)	-70.6 (-73.6 to -67.3)	910 (532 to 1420)	272 (153 to 461)	-70.2 (-75.8 to -62.8)
		Chlamydia spp.	40800 (35900 to 46500)	12500 (10900 to 14200)	-69.4 (-72.8 to -65.5)	1160 (1040 to 1280)	314 (253 to 384)	-72.8 (-77.9 to -66.6)

		Enterobacter spp.	3340 (2600 to 4240)	1820 (1440 to 2270)	-45.4 (-52.2 to -38.2)	407 (271 to 596)	164 (109 to 243)	-59.4 (-67.3 to -49.7)
		Escherichia coli	12600 (10800 to 14700)	6770 (5900 to 7750)	-46.2 (-52.6 to -39.3)	1400 (1210 to 1640)	536 (429 to 669)	-61.8 (-68.8 to -53.3)
		Fungus	2350 (1660 to 3270)	1070 (761 to 1430)	-54.3 (-60.4 to -47.3)	126 (88.9 to 172)	43 (29.7 to 59.9)	-65.8 (-72.9 to -56.8)
		Group B Streptococcus	10900 (9330 to 12800)	5370 (4600 to 6220)	-50.8 (-56.2 to -44.6)	1200 (1050 to 1380)	439 (354 to 538)	-63.4 (-69.8 to -55.1)
		Haemophilus influenzae	84100 (74100 to 95500)	26800 (23600 to 30100)	-68.1 (-71.5 to -64.3)	2540 (2220 to 2900)	325 (264 to 398)	-87.2 (-89.7 to -84.1)
		Influenza	71700 (64700 to 79900)	3670 (2070 to 5920)	-94.9 (-97.1 to -91.8)	3920 (3560 to 4290)	134 (66.7 to 226)	-96.6 (-98.3 to -94.1)
		Klebsiella pneumoniae	45700 (40900 to 51600)	13600 (12100 to 15300)	-70.3 (-73.0 to -67.3)	3520 (3180 to 3870)	917 (742 to 1120)	-73.9 (-78.8 to -68.0)
		Legionella spp.	3710 (2690 to 4960)	1790 (1340 to 2440)	-51.5 (-56.7 to -45.3)	473 (334 to 649)	189 (135 to 255)	-59.9 (-68.1 to -50.8)
		Mycoplasma spp.	49400 (43700 to 55700)	28500 (25300 to 32000)	-42.2 (-48.2 to -35.9)	1050 (927 to 1190)	408 (335 to 498)	-60.9 (-68.4 to -51.7)
		Polymicrobial	2270 (1430 to 3480)	920 (562 to 1400)	-59.4 (-63.6 to -55.0)	716 (400 to 1160)	282 (147 to 472)	-60.6 (-67.9 to -51.0)
		Pseudomonas aeruginosa	21100 (18700 to 23900)	14900 (13300 to 16700)	-29.5 (-37.1 to -21.6)	1500 (1350 to 1680)	696 (571 to 838)	-53.5 (-62.0 to -42.9)
		Respiratory syncytial virus	140000 (128000 to 156000)	11100 (6270 to 17800)	-92.1 (-95.6 to -87.5)	4290 (3980 to 4650)	207 (107 to 350)	-95.2 (-97.5 to -91.8)
		Staphylococcus aureus	28200 (25300 to 31400)	16200 (14400 to 18200)	-42.5 (-46.7 to -37.9)	2920 (2660 to 3190)	1230 (1010 to 1490)	-57.9 (-65.6 to -48.6)
		Streptococcus pneumoniae	476000 (433000 to 527000)	85500 (76400 to 95700)	-82.0 (-83.3 to -80.7)	16300 (15100 to 17600)	2370 (1900 to 2920)	-85.4 (-88.6 to -81.9)
		Other viruses	292000 (265000 to 322000)	92500 (83000 to 104000)	-68.3 (-70.6 to -65.8)	4270 (3960 to 4620)	1140 (937 to 1380)	-73.3 (-78.3 to -67.5)
		Other bacterial pathogens	14200 (11400 to 17200)	5210 (4240 to 6320)	-63.2 (-67.7 to -58.0)	765 (595 to 990)	231 (165 to 319)	-69.8 (-75.6 to -62.7)
	5-14 years	*Acinetobacter baumannii*	5360 (4400 to 6480)	2850 (2340 to 3450)	-46.8 (-51.3 to -41.9)	40.2 (34.6 to 46.7)	21.8 (18.2 to 26.3)	-45.7 (-53.7 to -36.7)
		Chlamydia spp.	19600 (16400 to 23500)	10500 (8760 to 12400)	-46.5 (-50.9 to -41.8)	36.2 (32.3 to 40.7)	18.8 (16.1 to 22.1)	-48.0 (-54.8 to -40.0)
		Enterobacter spp.	1400 (1140 to 1750)	1440 (1170 to 1750)	2.8 (-6.6 to 12.5)	9.75 (8.22 to 11.5)	9.37 (7.84 to 11.1)	-3.6 (-17.3 to 12.3)
		Escherichia coli	4060 (3380 to 4910)	3550 (2970 to 4200)	-12.5 (-20.4 to -4.4)	29.4 (26.3 to 33)	24.1 (20.9 to 28)	-17.7 (-28.2 to -6.0)
		Fungus	4910 (4020 to 5980)	4020 (3330 to 4850)	-18.0 (-25.0 to -10.5)	35 (30.3 to 40.1)	27.3 (23 to 32.2)	-21.9 (-32.4 to -9.6)
		Group B Streptococcus	4830 (4020 to 5830)	4410 (3680 to 5210)	-8.7 (-17.1 to 0.0)	24.7 (21.8 to 28.3)	21.3 (18.3 to 25)	-13.5 (-24.9 to -0.2)
		Haemophilus influenzae	18400 (15500 to 21700)	12200 (10200 to 14400)	-33.2 (-38.1 to -27.9)	50.3 (45.9 to 55.5)	21.4 (18.8 to 24.6)	-57.4 (-62.7 to -51.3)
		Influenza	26800 (22800 to 31600)	3170 (1760 to 5030)	-88.1 (-93.3 to -80.8)	144 (133 to 155)	14.1 (7.42 to 23.8)	-90.2 (-94.9 to -83.0)

		Klebsiella pneumoniae	12000 (10200 to 14200)	8430 (7160 to 9810)	-29.7 (-34.8 to -24.3)	86.1 (79.2 to 93.3)	58.3 (51.1 to 66.8)	-32.2 (-40.4 to -22.6)
		Legionella spp.	7720 (6410 to 9190)	6900 (5790 to 8030)	-10.5 (-18.9 to -1.7)	37.7 (33.3 to 42.8)	31.3 (27.2 to 36)	-16.8 (-28.0 to -3.2)
		Mycoplasma spp.	41700 (35200 to 49500)	28100 (23700 to 32600)	-32.7 (-37.5 to -27.2)	79.3 (72.6 to 85.8)	50.8 (44.4 to 58.4)	-35.9 (-44.1 to -27.0)
		Polymicrobial	803 (535 to 1170)	611 (384 to 922)	-24.1 (-31.5 to -16.5)	5.92 (4.04 to 8.62)	4.33 (2.8 to 6.46)	-27.0 (-37.3 to -15.0)
		Pseudomonas aeruginosa	8860 (7430 to 10400)	9180 (7730 to 10600)	3.6 (-4.3 to 12.7)	43.7 (40 to 47.9)	42.5 (37.1 to 48.6)	-2.7 (-14.6 to 11.8)
		Respiratory syncytial virus	5830 (4910 to 6880)	733 (406 to 1170)	-87.4 (-92.8 to -79.8)	14.1 (12.9 to 15.6)	1.48 (0.77 to 2.46)	-89.5 (-94.5 to -82.5)
		Staphylococcus aureus	15300 (13100 to 18000)	16500 (14100 to 19000)	7.9 (0.2 to 15.8)	155 (144 to 166)	156 (137 to 178)	0.8 (-11.4 to 14.7)
		Streptococcus pneumoniae	156000 (133000 to 181000)	74000 (62500 to 85500)	-52.5 (-55.8 to -49.3)	660 (622 to 700)	307 (271 to 351)	-53.4 (-59.3 to -47.2)
		Other viruses	58100 (49700 to 67400)	44900 (38300 to 52200)	-22.7 (-28.2 to -16.6)	95.8 (89.5 to 102)	69.8 (61.6 to 79.7)	-27.0 (-35.8 to -17.3)
		Other bacterial pathogens	18100 (15200 to 21400)	11300 (9340 to 13300)	-37.8 (-42.7 to -32.3)	73.3 (66 to 81.3)	43.7 (37.2 to 50.7)	-40.3 (-47.9 to -31.5)
	15-49 years	*Acinetobacter baumannii*	5130 (4390 to 6040)	4750 (4110 to 5480)	-7.4 (-14.5 to 0.1)	50 (43.3 to 57.4)	46 (39.1 to 54.1)	-7.8 (-19.7 to 5.6)
		Chlamydia spp.	18900 (16700 to 21400)	17800 (15900 to 19800)	-5.9 (-12.4 to 0.7)	45.5 (41.1 to 51.1)	42.5 (36.8 to 49)	-6.6 (-17.7 to 5.6)
		Enterobacter spp.	1360 (1150 to 1610)	2490 (2160 to 2850)	82.5 (68.1 to 98.8)	12.5 (10.6 to 14.6)	22.8 (19.4 to 26.9)	83.3 (59.7 to 107.9)
		Escherichia coli	3940 (3450 to 4500)	6110 (5500 to 6830)	55.4 (44.3 to 67.5)	37.3 (33.5 to 41.7)	57.9 (50.5 to 65.8)	55.2 (36.4 to 75.8)
		Fungus	4760 (4050 to 5580)	6900 (6050 to 7820)	45.1 (34.6 to 56.8)	44.5 (38.5 to 50.9)	64.4 (54.7 to 74.2)	45.0 (28.3 to 64.4)
		Group B Streptococcus	4690 (4090 to 5500)	7570 (6790 to 8500)	61.8 (48.5 to 75.6)	31.5 (27.8 to 35.9)	51 (44.2 to 58.7)	62.1 (41.5 to 84.4)
		Haemophilus influenzae	17900 (16000 to 19800)	21200 (19200 to 23300)	18.7 (11.7 to 24.9)	64.5 (59.3 to 69.5)	51.9 (45.9 to 58.2)	-19.4 (-28.7 to -9.5)
		Influenza	26000 (23800 to 28500)	5670 (3280 to 9040)	-78.2 (-87.6 to -65.4)	184 (172 to 194)	39.6 (21.1 to 66.7)	-78.5 (-88.5 to -63.6)
		Klebsiella pneumoniae	11600 (10500 to 12800)	14400 (13100 to 15700)	23.9 (17.2 to 31.2)	109 (102 to 118)	135 (120 to 152)	23.8 (9.6 to 39.2)
		Legionella spp.	7560 (6610 to 8580)	12000 (10900 to 13200)	59.0 (46.5 to 72.1)	48.9 (43.3 to 55.1)	78.1 (69 to 87.7)	60.3 (40.2 to 83.4)
		Mycoplasma spp.	40600 (36500 to 44700)	48200 (44100 to 52800)	19.0 (12.0 to 26.6)	101 (93.5 to 110)	121 (107 to 136)	19.2 (5.5 to 34.3)
		Polymicrobial	774 (519 to 1140)	1040 (667 to 1570)	34.0 (21.9 to 47.0)	7.45 (5.08 to 10.7)	9.96 (6.42 to 15.2)	33.3 (15.3 to 53.2)
		Pseudomonas aeruginosa	8660 (7770 to 9680)	15900 (14500 to 17400)	83.7 (71.8 to 95.7)	56.5 (52.3 to 61)	105 (93.2 to 117)	85.5 (64.0 to 110.9)
		Respiratory syncytial virus	5710 (5120 to 6390)	1320 (763 to 2080)	-76.8 (-86.7 to -63.6)	18.3 (16.8 to 19.9)	4.2 (2.26 to 6.97)	-77.1 (-87.8 to -61.7)

		Staphylococcus aureus	14900 (13600 to 16200)	28500 (26300 to 31000)	91.6 (82.1 to 102.5)	199 (187 to 211)	383 (341 to 428)	92.7 (71.4 to 115.8)
		Streptococcus pneumoniae	151000 (139000 to 165000)	126000 (116000 to 136000)	-17.0 (-21.1 to -12.7)	843 (804 to 886)	699 (624 to 780)	-17.0 (-26.2 to -7.3)
		Other viruses	56800 (52300 to 61700)	77600 (71900 to 84400)	36.7 (29.5 to 43.8)	124 (118 to 131)	171 (152 to 190)	37.6 (22.6 to 53.4)
		Other bacterial pathogens	17500 (15400 to 19700)	19200 (17100 to 21600)	9.8 (2.9 to 16.9)	93 (84.2 to 102)	102 (87.9 to 117)	9.5 (-3.0 to 24.2)
	50-69 years	*Acinetobacter baumannii*	7310 (6300 to 8500)	9210 (7940 to 10600)	26.1 (17.0 to 35.7)	81.8 (71.6 to 93.7)	98.2 (84.1 to 115)	20.2 (4.8 to 36.9)
		Chlamydia spp.	16200 (14400 to 18200)	20800 (18500 to 23000)	28.2 (19.3 to 37.9)	44.4 (40 to 48.8)	56.1 (48.3 to 63.9)	26.4 (10.6 to 44.8)
		Enterobacter spp.	1960 (1660 to 2310)	4960 (4310 to 5770)	153.0 (133.0 to 173.8)	20.2 (17.1 to 23.8)	52.5 (44.7 to 61)	160.4 (123.4 to 198.6)
		Escherichia coli	6190 (5430 to 6980)	13200 (11800 to 14700)	113.0 (98.3 to 130.4)	66.4 (59.5 to 73.9)	143 (125 to 163)	115.8 (87.4 to 146.6)
		Fungus	5960 (5140 to 6880)	12000 (10500 to 13700)	101.4 (86.5 to 116.2)	62.9 (55 to 71.8)	128 (109 to 148)	103.3 (78.2 to 132.9)
		Group B Streptococcus	4750 (4140 to 5420)	10500 (9270 to 11800)	121.5 (105.1 to 140.5)	36 (31.8 to 40.7)	81.1 (69.6 to 92.4)	125.8 (94.9 to 158.5)
		Haemophilus influenzae	15500 (14100 to 17100)	26700 (24300 to 29500)	72.0 (62.6 to 82.8)	63 (58.3 to 68.3)	75.9 (66.5 to 85.9)	20.7 (5.9 to 38.0)
		Influenza	19400 (17800 to 21300)	6260 (3630 to 9800)	-67.7 (-81.1 to -49.3)	154 (144 to 164)	51.6 (27.3 to 84.9)	-66.5 (-82.2 to -44.1)
		Klebsiella pneumoniae	12800 (11700 to 14000)	23200 (21100 to 25400)	81.3 (72.0 to 91.7)	136 (127 to 146)	249 (220 to 280)	82.5 (60.4 to 107.2)
		Legionella spp.	9370 (8250 to 10600)	20600 (18600 to 22800)	120.3 (103.9 to 138.9)	67.6 (60.3 to 75.7)	156 (136 to 176)	131.5 (99.9 to 167.1)
		Mycoplasma spp.	16100 (14600 to 17900)	25100 (22800 to 27700)	56.1 (47.0 to 66.4)	45.4 (41.6 to 49.2)	72.1 (63.7 to 81.8)	59.2 (38.7 to 81.9)
		Polymicrobial	933 (636 to 1380)	1750 (1140 to 2700)	86.8 (69.8 to 104.1)	10.2 (7 to 14.9)	19 (12.1 to 29.3)	84.7 (59.1 to 114.6)
		Pseudomonas aeruginosa	10800 (9800 to 11900)	26700 (24200 to 29400)	147.6 (132.3 to 164.1)	78.6 (72.5 to 84.9)	203 (178 to 229)	158.4 (125.3 to 194.4)
		Respiratory syncytial virus	3220 (2860 to 3600)	1050 (601 to 1640)	-67.4 (-80.9 to -48.7)	11.5 (10.4 to 12.7)	3.97 (2.13 to 6.52)	-65.5 (-81.3 to -42.3)
		Staphylococcus aureus	16100 (14800 to 17600)	43200 (39300 to 47300)	167.4 (154.0 to 182.6)	242 (229 to 258)	669 (586 to 753)	176.2 (142.0 to 213.4)
		Streptococcus pneumoniae	80600 (74700 to 87400)	95000 (86600 to 104000)	18.0 (12.4 to 24.7)	506 (481 to 531)	602 (533 to 676)	19.2 (5.3 to 34.6)
		Other viruses	27000 (24800 to 29500)	53900 (49100 to 59000)	99.8 (90.4 to 111.5)	65.8 (62.2 to 69.7)	137 (121 to 154)	109.1 (84.0 to 137.6)
		Other bacterial pathogens	22700 (20300 to 25500)	34400 (30300 to 38900)	51.6 (42.1 to 62.5)	137 (124 to 149)	208 (179 to 241)	52.4 (33.1 to 73.7)
	70+ years	*Acinetobacter baumannii*	5300 (4470 to 6250)	5760 (5010 to 6640)	8.9 (1.2 to 17.8)	71.1 (61.7 to 82.7)	111 (93.9 to 129)	56.5 (38.5 to 76.2)
		Chlamydia spp.	14000 (12200 to 16000)	15700 (14100 to 17700)	12.5 (4.4 to 21.0)	45.8 (40.4 to 51)	79.3 (68 to 90)	73.2 (52.7 to 94.7)

		Enterobacter spp.	1390 (1140 to 1670)	3080 (2630 to 3580)	123.2 (104.6 to 142.5)	17 (14.1 to 20.2)	61.9 (52.2 to 72.1)	266.2 (217.7 to 316.4)
		Escherichia coli	5500 (4760 to 6400)	10300 (9210 to 11500)	87.3 (73.0 to 101.0)	70.4 (62.2 to 78.9)	211 (183 to 237)	200.0 (164.9 to 239.2)
		Fungus	2400 (2020 to 2850)	4240 (3680 to 4890)	77.0 (62.7 to 91.6)	30.3 (26 to 34.9)	85.3 (72.8 to 99.5)	182.0 (151.1 to 218.8)
		Group B Streptococcus	3150 (2720 to 3650)	6140 (5500 to 6910)	95.1 (78.3 to 111.3)	28.5 (24.8 to 32.3)	89.7 (77.4 to 102)	215.3 (177.3 to 257.0)
		Haemophilus influenzae	10100 (8940 to 11500)	15400 (14000 to 17200)	52.6 (43.7 to 61.9)	48.9 (44.2 to 53.6)	84.3 (73.8 to 94.5)	72.5 (53.1 to 93.3)
		Influenza	21400 (19000 to 23900)	6530 (3830 to 10100)	-69.4 (-81.3 to -53.6)	203 (185 to 219)	118 (65.6 to 189)	-41.6 (-67.6 to -7.2)
		Klebsiella pneumoniae	10200 (9130 to 11500)	16300 (14800 to 18100)	59.5 (50.3 to 68.9)	130 (118 to 140)	328 (286 to 366)	152.8 (125.4 to 181.6)
		Legionella spp.	4880 (4210 to 5630)	9560 (8620 to 10700)	96.4 (80.6 to 113.0)	41.8 (36.7 to 47.3)	139 (120 to 157)	234.2 (193.7 to 280.7)
		Mycoplasma spp.	9870 (8770 to 11300)	13600 (12400 to 15300)	38.4 (29.1 to 47.6)	33.1 (29.7 to 36.2)	74.3 (64.8 to 83.3)	125.0 (99.6 to 152.4)
		Polymicrobial	775 (507 to 1170)	1270 (807 to 1960)	63.0 (47.5 to 78.5)	10.1 (6.83 to 15.2)	25.6 (16 to 40.3)	151.0 (118.0 to 188.6)
		Pseudomonas aeruginosa	8130 (7240 to 9230)	17800 (16300 to 19800)	119.8 (105.3 to 134.5)	70.5 (63.8 to 76.8)	259 (228 to 289)	268.7 (227.2 to 312.4)
		Respiratory syncytial virus	2180 (1900 to 2500)	686 (404 to 1040)	-68.5 (-80.7 to -52.1)	9.29 (8.28 to 10.3)	5.81 (3.16 to 9.22)	-37.4 (-65.8 to 0.5)
		Staphylococcus aureus	15100 (13600 to 16900)	35700 (32600 to 39500)	136.0 (122.3 to 149.1)	270 (246 to 291)	1050 (924 to 1170)	288.9 (246.3 to 333.0)
		Streptococcus pneumoniae	60000 (54200 to 67100)	62700 (57500 to 69200)	4.7 (-1.2 to 10.4)	449 (412 to 479)	752 (664 to 835)	67.7 (50.5 to 86.9)
		Other viruses	19900 (17900 to 22400)	35400 (32200 to 39200)	78.2 (67.5 to 87.9)	57.6 (52.6 to 61.7)	173 (152 to 194)	201.5 (169.6 to 236.7)
		Other bacterial pathogens	22000 (19300 to 25100)	29400 (26000 to 33400)	33.6 (24.8 to 42.3)	160 (142 to 176)	338 (288 to 386)	112.0 (88.4 to 136.8)
Armenia	All Ages	*Acinetobacter baumannii*	905 (789 to 1030)	639 (539 to 752)	-29.4 (-39.5 to -18.8)	23.5 (16 to 34.2)	16.1 (13.8 to 18.7)	-29.4 (-51.7 to -0.2)
		Chlamydia spp.	3520 (3200 to 3900)	2240 (1960 to 2570)	-36.2 (-45.1 to -26.5)	25.6 (22.7 to 28.9)	13.7 (12.1 to 15.4)	-46.2 (-54.5 to -37.4)
		Enterobacter spp.	350 (300 to 403)	530 (450 to 619)	52.1 (30.0 to 76.1)	10.5 (7.47 to 14.6)	12.3 (10.6 to 14.2)	21.1 (-15.6 to 65.5)
		Escherichia coli	1130 (1020 to 1260)	1460 (1270 to 1690)	29.4 (11.2 to 49.7)	33.9 (29.5 to 39.6)	37.8 (33.1 to 42.2)	12.1 (-6.4 to 31.6)
		Fungus	781 (681 to 895)	952 (821 to 1100)	22.2 (5.6 to 40.9)	7.48 (6.36 to 8.68)	17.7 (15.4 to 20.6)	137.4 (98.9 to 180.7)
		Group B Streptococcus	990 (890 to 1110)	1140 (995 to 1310)	15.3 (-0.4 to 32.6)	27.9 (24.3 to 32)	18.8 (16.5 to 21)	-32.5 (-43.6 to -19.4)
		Haemophilus influenzae	4730 (4330 to 5220)	3280 (2870 to 3780)	-30.5 (-39.7 to -20.0)	56.7 (48.8 to 65.5)	18.5 (16.4 to 20.5)	-67.2 (-72.5 to -61.1)
		Influenza	5690 (5280 to 6190)	4180 (1400 to 7510)	-26.5 (-75.3 to 31.9)	95.7 (85.5 to 107)	75 (22.9 to 148)	-21.5 (-76.5 to 57.1)

		Klebsiella pneumoniae	2990 (2750 to 3280)	2510 (2200 to 2860)	-16.1 (-28.1 to -3.5)	78.9 (70 to 89.4)	60.1 (52.9 to 66.8)	-23.6 (-35.3 to -11.0)
		Legionella spp.	1420 (1260 to 1590)	2170 (1900 to 2480)	52.7 (31.4 to 75.1)	17.2 (13.9 to 21.1)	31 (27.1 to 35)	82.3 (41.6 to 120.6)
		Mycoplasma spp.	5500 (5030 to 6060)	3900 (3410 to 4450)	-29.1 (-37.8 to -19.2)	27.1 (24.2 to 30.9)	18.4 (16.3 to 20.4)	-32.0 (-42.2 to -21.5)
		Polymicrobial	186 (132 to 260)	175 (111 to 269)	-6.6 (-26.4 to 13.7)	16.7 (9.48 to 27.2)	5.4 (3.67 to 8)	-66.2 (-79.5 to -44.8)
		Pseudomonas aeruginosa	2200 (2010 to 2390)	3330 (2900 to 3810)	51.6 (31.0 to 74.6)	40.3 (36 to 45.7)	54.8 (48.5 to 61)	36.2 (15.7 to 58.4)
		Respiratory syncytial virus	4690 (4230 to 5210)	1450 (499 to 2560)	-69.0 (-88.9 to -45.2)	94.3 (82.8 to 108)	13.2 (4.2 to 25.5)	-86.0 (-95.7 to -72.6)
		Staphylococcus aureus	3360 (3120 to 3620)	5570 (4890 to 6370)	65.6 (44.3 to 88.7)	86.3 (78.3 to 95.4)	193 (170 to 215)	124.5 (94.4 to 157.6)
		Streptococcus pneumoniae	29600 (27500 to 32100)	12600 (11100 to 14300)	-57.5 (-62.9 to -51.2)	372 (333 to 419)	150 (133 to 166)	-59.5 (-65.6 to -52.9)
		Other viruses	15000 (13800 to 16300)	9420 (8200 to 10800)	-37.1 (-45.8 to -27.5)	99.7 (88.6 to 113)	45.1 (40.2 to 49.9)	-54.6 (-61.5 to -47.4)
		Other bacterial pathogens	3620 (3240 to 4010)	3800 (3200 to 4480)	5.1 (-8.6 to 20.4)	28.6 (24.6 to 34)	54.7 (45.8 to 63.8)	92.5 (55.8 to 130.5)
	Under 5	*Acinetobacter baumannii*	87.2 (60.4 to 121)	10.1 (6.36 to 15.7)	-88.5 (-91.7 to -84.6)	17.1 (9.51 to 27.2)	1.08 (0.6 to 1.84)	-93.6 (-95.1 to -91.7)
		Chlamydia spp.	927 (805 to 1070)	125 (91.4 to 164)	-86.5 (-90.0 to -82.2)	20.7 (18 to 24.1)	1.49 (1.18 to 1.89)	-92.8 (-94.4 to -90.7)
		Enterobacter spp.	87.1 (67.5 to 111)	23.5 (16 to 32.7)	-73.0 (-80.1 to -64.4)	8.5 (5.58 to 12.8)	1.04 (0.72 to 1.5)	-87.6 (-90.9 to -83.1)
		Escherichia coli	309 (264 to 361)	82.1 (60.5 to 108)	-73.4 (-80.2 to -64.7)	27.4 (23.1 to 33.1)	3.3 (2.61 to 4.22)	-87.9 (-90.8 to -84.1)
		Fungus	56.7 (40.1 to 77.5)	12.4 (8.1 to 18.1)	-78.0 (-83.8 to -70.7)	2.2 (1.55 to 3.05)	0.33 (0.22 to 0.48)	-85.1 (-89.0 to -80.3)
		Group B Streptococcus	276 (235 to 324)	64.9 (46.6 to 86.4)	-76.5 (-82.6 to -69.0)	24.2 (20.5 to 28.1)	2.41 (1.92 to 3.02)	-90.0 (-92.3 to -87.2)
		Haemophilus influenzae	2170 (1890 to 2520)	357 (262 to 465)	-83.5 (-87.7 to -78.2)	49.6 (41.7 to 58.2)	2.67 (2.09 to 3.44)	-94.6 (-95.9 to -92.9)
		Influenza	1820 (1610 to 2060)	220 (78.6 to 394)	-87.9 (-95.6 to -78.5)	74.4 (64 to 85.7)	5.07 (1.6 to 9.85)	-93.2 (-97.9 to -86.6)
		Klebsiella pneumoniae	1110 (968 to 1270)	151 (111 to 198)	-86.4 (-89.9 to -82.3)	64.8 (55.7 to 75)	5.41 (4.24 to 6.91)	-91.6 (-93.5 to -89.2)
		Legionella spp.	104 (74.9 to 140)	25.6 (17.1 to 37.5)	-75.2 (-81.8 to -67.4)	10.4 (7.42 to 14.3)	1.13 (0.82 to 1.52)	-89.0 (-91.8 to -85.7)
		Mycoplasma spp.	1250 (1090 to 1450)	375 (275 to 495)	-70.0 (-77.9 to -60.5)	19.7 (16.9 to 23.4)	3.03 (2.37 to 3.92)	-84.5 (-88.2 to -79.9)
		Polymicrobial	59.7 (36.7 to 92.9)	11 (6.1 to 18.2)	-81.7 (-86.6 to -75.5)	15.7 (8.52 to 26.1)	1.45 (0.74 to 2.41)	-90.8 (-93.0 to -87.9)
		Pseudomonas aeruginosa	578 (504 to 659)	215 (158 to 283)	-62.7 (-72.4 to -50.7)	31.8 (27.4 to 36.9)	5.31 (4.23 to 6.72)	-83.2 (-86.9 to -78.0)
		Respiratory syncytial virus	3960 (3520 to 4440)	787 (286 to 1400)	-80.1 (-92.6 to -64.9)	92.6 (81.1 to 107)	8.98 (2.83 to 17.5)	-90.3 (-97.0 to -80.9)

		Staphylococcus aureus	734 (649 to 831)	213 (156 to 279)	-71.0 (-78.2 to -62.3)	58 (50.3 to 66.4)	9.46 (7.47 to 12.1)	-83.6 (-87.5 to -79.0)
		Streptococcus pneumoniae	11900 (10600 to 13400)	882 (648 to 1160)	-92.6 (-94.4 to -90.3)	300 (261 to 347)	14.4 (11.3 to 18.4)	-95.2 (-96.3 to -93.7)
		Other viruses	8100 (7220 to 9070)	1300 (951 to 1700)	-84.0 (-87.9 to -79.0)	88.8 (77.6 to 102)	9.31 (7.34 to 11.8)	-89.5 (-92.0 to -86.4)
		Other bacterial pathogens	341 (271 to 415)	59.8 (41.4 to 82.4)	-82.4 (-87.1 to -76.4)	14.2 (10.6 to 19.2)	1.36 (0.97 to 1.82)	-90.4 (-92.7 to -87.5)
	5-14 years	*Acinetobacter baumannii*	156 (120 to 198)	27.9 (21.5 to 35.4)	-82.1 (-84.8 to -78.9)	0.63 (0.54 to 0.74)	0.07 (0.06 to 0.09)	-88.4 (-90.4 to -86.1)
		Chlamydia spp.	638 (504 to 788)	135 (104 to 169)	-78.8 (-81.9 to -75.2)	0.64 (0.55 to 0.72)	0.09 (0.07 to 0.1)	-86.2 (-88.6 to -83.6)
		Enterobacter spp.	51.5 (39.5 to 65.5)	23.6 (17.9 to 29.9)	-54.1 (-60.9 to -45.9)	0.2 (0.16 to 0.23)	0.06 (0.05 to 0.07)	-70.2 (-75.5 to -64.3)
		Escherichia coli	141 (110 to 174)	53.4 (41.3 to 65.9)	-62.2 (-68.2 to -55.5)	0.56 (0.49 to 0.64)	0.14 (0.12 to 0.16)	-75.5 (-79.7 to -70.4)
		Fungus	171 (134 to 215)	59.3 (46.1 to 74.8)	-65.2 (-70.4 to -59.1)	0.66 (0.56 to 0.77)	0.15 (0.13 to 0.18)	-77.4 (-81.3 to -73.2)
		Group B Streptococcus	173 (136 to 216)	68.1 (52.7 to 84.6)	-60.4 (-66.5 to -53.7)	0.48 (0.41 to 0.56)	0.12 (0.1 to 0.14)	-74.3 (-78.9 to -69.6)
		Haemophilus influenzae	674 (545 to 819)	205 (161 to 254)	-69.6 (-73.8 to -64.4)	1.01 (0.9 to 1.13)	0.14 (0.12 to 0.16)	-86.6 (-88.9 to -84.2)
		Influenza	971 (784 to 1180)	250 (79.4 to 473)	-74.1 (-91.7 to -53.1)	2.85 (2.55 to 3.16)	0.47 (0.14 to 0.94)	-83.5 (-95.2 to -66.2)
		Klebsiella pneumoniae	417 (335 to 509)	123 (97.5 to 152)	-70.5 (-74.6 to -65.7)	1.63 (1.46 to 1.81)	0.31 (0.27 to 0.36)	-80.8 (-84.0 to -77.3)
		Legionella spp.	304 (242 to 374)	128 (100 to 160)	-57.6 (-64.1 to -50.2)	0.82 (0.7 to 0.95)	0.22 (0.19 to 0.26)	-72.5 (-77.5 to -67.3)
		Mycoplasma spp.	1520 (1220 to 1850)	441 (343 to 546)	-70.8 (-74.8 to -66.3)	1.57 (1.4 to 1.76)	0.3 (0.25 to 0.34)	-81.1 (-84.3 to -77.5)
		Polymicrobial	26 (16.7 to 38.8)	7.99 (4.78 to 12.4)	-69.4 (-74.4 to -63.5)	0.1 (0.07 to 0.16)	0.02 (0.01 to 0.03)	-80.2 (-83.8 to -75.8)
		Pseudomonas aeruginosa	343 (275 to 414)	162 (128 to 200)	-52.6 (-59.3 to -45.0)	0.93 (0.83 to 1.05)	0.28 (0.25 to 0.33)	-69.3 (-74.5 to -63.6)
		Respiratory syncytial virus	232 (185 to 282)	64.9 (21.1 to 125)	-71.9 (-90.8 to -48.8)	0.31 (0.28 to 0.35)	0.06 (0.02 to 0.11)	-82.1 (-94.7 to -63.6)
		Staphylococcus aureus	565 (456 to 681)	275 (217 to 337)	-51.4 (-58.2 to -43.7)	3.14 (2.83 to 3.48)	0.99 (0.86 to 1.13)	-68.5 (-73.6 to -62.7)
		Streptococcus pneumoniae	5580 (4500 to 6700)	1090 (863 to 1350)	-80.4 (-83.0 to -77.3)	12.9 (11.7 to 14.2)	1.64 (1.41 to 1.88)	-87.3 (-89.3 to -85.0)
		Other viruses	2280 (1840 to 2740)	824 (652 to 1010)	-63.8 (-68.8 to -58.1)	2.07 (1.87 to 2.29)	0.48 (0.42 to 0.56)	-76.5 (-80.3 to -72.3)
		Other bacterial pathogens	623 (496 to 764)	162 (125 to 205)	-73.9 (-77.6 to -69.5)	1.37 (1.19 to 1.56)	0.23 (0.19 to 0.27)	-83.1 (-86.0 to -80.0)
	15-49 years	*Acinetobacter baumannii*	156 (129 to 187)	72 (60 to 87.2)	-53.6 (-60.0 to -46.9)	1.17 (0.99 to 1.37)	0.46 (0.39 to 0.55)	-60.4 (-67.1 to -52.7)
		Chlamydia spp.	635 (541 to 741)	349 (299 to 412)	-44.9 (-52.3 to -37.1)	1.18 (1.04 to 1.33)	0.56 (0.47 to 0.64)	-53.0 (-61.1 to -44.6)

		Enterobacter spp.	51.2 (41.8 to 61.6)	60.7 (50.8 to 72.8)	19.1 (2.5 to 38.6)	0.36 (0.31 to 0.43)	0.37 (0.31 to 0.44)	1.7 (-15.3 to 21.8)
		Escherichia coli	141 (119 to 164)	138 (118 to 161)	-1.9 (-14.9 to 12.3)	1.04 (0.9 to 1.16)	0.86 (0.75 to 1)	-16.3 (-29.9 to -0.1)
		Fungus	170 (142 to 203)	153 (129 to 182)	-9.7 (-22.1 to 4.1)	1.23 (1.05 to 1.42)	0.95 (0.81 to 1.1)	-22.9 (-36.0 to -8.7)
		Group B Streptococcus	172 (146 to 204)	176 (150 to 207)	2.7 (-11.0 to 17.5)	0.9 (0.77 to 1.03)	0.78 (0.67 to 0.91)	-12.4 (-27.1 to 4.0)
		Haemophilus influenzae	670 (576 to 776)	528 (456 to 616)	-21.1 (-30.9 to -10.3)	1.88 (1.69 to 2.09)	0.86 (0.75 to 0.98)	-54.2 (-61.7 to -45.7)
		Influenza	966 (837 to 1100)	646 (217 to 1180)	-33.1 (-77.6 to 20.7)	5.3 (4.81 to 5.78)	2.96 (0.93 to 5.82)	-44.1 (-83.2 to 12.1)
		Klebsiella pneumoniae	414 (357 to 472)	317 (274 to 367)	-23.4 (-32.8 to -13.3)	3.03 (2.73 to 3.32)	1.98 (1.73 to 2.27)	-34.6 (-45.0 to -22.6)
		Legionella spp.	302 (254 to 356)	331 (286 to 386)	9.9 (-4.6 to 27.3)	1.52 (1.33 to 1.74)	1.42 (1.23 to 1.63)	-6.2 (-22.3 to 12.0)
		Mycoplasma spp.	1510 (1310 to 1730)	1140 (988 to 1310)	-24.4 (-33.6 to -14.0)	2.93 (2.63 to 3.24)	1.88 (1.63 to 2.15)	-35.5 (-46.0 to -23.3)
		Polymicrobial	25.9 (16.8 to 38.6)	20.6 (12.9 to 31.8)	-20.6 (-32.6 to -6.2)	0.19 (0.13 to 0.29)	0.13 (0.08 to 0.21)	-32.3 (-44.4 to -18.5)
		Pseudomonas aeruginosa	341 (293 to 395)	418 (363 to 487)	23.0 (7.5 to 40.5)	1.73 (1.55 to 1.92)	1.81 (1.57 to 2.07)	5.0 (-12.4 to 25.1)
		Respiratory syncytial virus	231 (197 to 266)	168 (56.4 to 306)	-27.3 (-75.6 to 32.9)	0.58 (0.51 to 0.64)	0.35 (0.11 to 0.7)	-39.2 (-81.5 to 21.3)
		Staphylococcus aureus	562 (491 to 640)	708 (613 to 817)	26.2 (10.9 to 43.0)	5.85 (5.32 to 6.38)	6.28 (5.48 to 7.17)	7.7 (-9.6 to 27.0)
		Streptococcus pneumoniae	5550 (4860 to 6300)	2820 (2450 to 3260)	-49.1 (-55.1 to -42.2)	24 (22 to 26.1)	10.4 (9.07 to 11.8)	-56.5 (-63.4 to -48.5)
		Other viruses	2270 (1980 to 2580)	2120 (1840 to 2450)	-6.2 (-17.5 to 6.0)	3.86 (3.52 to 4.2)	3.08 (2.69 to 3.53)	-19.9 (-32.5 to -5.1)
		Other bacterial pathogens	620 (521 to 726)	418 (352 to 499)	-32.4 (-40.7 to -22.9)	2.55 (2.25 to 2.88)	1.47 (1.22 to 1.73)	-42.2 (-51.6 to -31.3)
	50-69 years	*Acinetobacter baumannii*	294 (247 to 350)	248 (207 to 298)	-15.2 (-28.0 to -1.6)	2.62 (2.25 to 3.02)	2.95 (2.43 to 3.62)	13.2 (-7.9 to 39.7)
		Chlamydia spp.	708 (615 to 821)	696 (602 to 810)	-1.5 (-16.4 to 15.1)	1.57 (1.39 to 1.76)	2.06 (1.7 to 2.45)	31.5 (7.4 to 62.2)
		Enterobacter spp.	93.8 (78.1 to 113)	201 (170 to 238)	115.5 (82.6 to 154.5)	0.79 (0.67 to 0.93)	2.27 (1.87 to 2.75)	187.6 (133.6 to 256.5)
		Escherichia coli	285 (246 to 331)	497 (428 to 576)	75.0 (49.0 to 105.2)	2.49 (2.19 to 2.8)	5.79 (4.88 to 6.93)	133.6 (91.3 to 187.9)
		Fungus	273 (231 to 320)	446 (378 to 523)	63.8 (39.6 to 92.1)	2.36 (2.03 to 2.71)	5.14 (4.27 to 6.3)	118.7 (78.2 to 169.2)
		Group B Streptococcus	222 (188 to 263)	408 (348 to 477)	84.2 (55.2 to 115.7)	1.38 (1.19 to 1.57)	3.38 (2.81 to 4)	145.9 (101.7 to 203.0)
		Haemophilus influenzae	741 (649 to 849)	1090 (939 to 1250)	47.0 (25.7 to 70.8)	2.47 (2.21 to 2.74)	3.29 (2.77 to 3.9)	33.4 (9.7 to 67.0)
		Influenza	920 (810 to 1040)	1130 (369 to 2060)	23.0 (-59.7 to 124.7)	6 (5.4 to 6.57)	9.64 (3.03 to 19.7)	61.1 (-51.6 to 230.8)

		Klebsiella pneumoniae	587 (519 to 663)	854 (741 to 980)	45.9 (25.6 to 70.4)	5.1 (4.62 to 5.58)	9.91 (8.33 to 11.8)	94.7 (60.8 to 139.5)
		Legionella spp.	470 (401 to 547)	930 (803 to 1080)	98.5 (68.0 to 132.5)	2.81 (2.48 to 3.18)	7.42 (6.22 to 8.88)	165.0 (115.8 to 227.4)
		Mycoplasma spp.	764 (669 to 871)	996 (860 to 1150)	30.6 (12.3 to 52.5)	1.76 (1.58 to 1.97)	3.07 (2.59 to 3.65)	74.4 (43.6 to 116.2)
		Polymicrobial	40.7 (26.6 to 60)	58.9 (36.4 to 93.4)	44.4 (20.1 to 70.7)	0.36 (0.24 to 0.55)	0.7 (0.43 to 1.11)	92.8 (56.2 to 137.6)
		Pseudomonas aeruginosa	534 (468 to 612)	1160 (1000 to 1340)	118.4 (85.8 to 155.4)	3.23 (2.88 to 3.56)	9.37 (7.92 to 11.2)	191.6 (138.5 to 259.0)
		Respiratory syncytial virus	163 (139 to 186)	210 (68.1 to 388)	29.7 (-57.5 to 138.6)	0.48 (0.42 to 0.55)	0.82 (0.25 to 1.67)	69.8 (-49.5 to 246.3)
		Staphylococcus aureus	776 (683 to 878)	1780 (1540 to 2030)	129.4 (97.1 to 167.4)	9.58 (8.72 to 10.5)	29.3 (24.6 to 34.8)	206.2 (152.3 to 277.5)
		Streptococcus pneumoniae	3780 (3370 to 4270)	3600 (3120 to 4130)	-4.5 (-17.9 to 11.2)	19.4 (17.8 to 21.1)	24.7 (20.8 to 29.5)	27.5 (5.0 to 56.8)
		Other viruses	1350 (1200 to 1520)	2410 (2100 to 2760)	78.4 (53.9 to 106.6)	2.73 (2.49 to 2.99)	6.5 (5.5 to 7.72)	138.2 (95.2 to 194.1)
		Other bacterial pathogens	1030 (901 to 1200)	1260 (1050 to 1480)	21.9 (4.5 to 41.4)	5.07 (4.49 to 5.72)	8.25 (6.78 to 10)	63.1 (32.8 to 101.4)
	70+ years	*Acinetobacter baumannii*	212 (177 to 255)	280 (231 to 346)	32.4 (10.6 to 55.3)	2.05 (1.72 to 2.39)	11.5 (9.68 to 13.5)	462.5 (385.8 to 552.1)
		Chlamydia spp.	611 (521 to 714)	937 (790 to 1120)	53.8 (29.7 to 81.9)	1.46 (1.26 to 1.65)	9.53 (8.11 to 10.8)	553.7 (469.3 to 645.9)
		Enterobacter spp.	66.1 (53.9 to 80.5)	221 (180 to 268)	236.4 (180.3 to 303.5)	0.6 (0.5 to 0.72)	8.6 (7.27 to 9.98)	1329.6 (1132.4 to 1553.0)
		Escherichia coli	253 (216 to 297)	689 (581 to 823)	173.3 (128.0 to 220.3)	2.39 (2.06 to 2.73)	27.7 (23.8 to 31.2)	1061.3 (907.8 to 1228.9)
		Fungus	110 (91.4 to 133)	281 (235 to 341)	155.8 (114.0 to 205.2)	1.03 (0.86 to 1.2)	11.1 (9.49 to 12.9)	986.9 (835.9 to 1155.7)
		Group B Streptococcus	148 (126 to 174)	423 (357 to 506)	187.5 (140.0 to 241.5)	0.99 (0.84 to 1.15)	12.1 (10.3 to 13.7)	1122.1 (957.9 to 1303.6)
		Haemophilus influenzae	483 (416 to 560)	1110 (943 to 1310)	129.6 (94.8 to 170.0)	1.74 (1.51 to 1.95)	11.5 (10 to 12.9)	563.1 (476.0 to 659.3)
		Influenza	1010 (882 to 1160)	1930 (650 to 3470)	91.2 (-36.3 to 247.6)	7.14 (6.32 to 7.97)	56.8 (17.3 to 113)	698.2 (143.1 to 1461.2)
		Klebsiella pneumoniae	468 (405 to 543)	1060 (910 to 1260)	127.8 (93.0 to 168.9)	4.4 (3.87 to 4.87)	42.5 (36.7 to 47.5)	868.0 (741.8 to 1001.7)
		Legionella spp.	245 (205 to 288)	755 (637 to 903)	210.0 (160.9 to 266.5)	1.58 (1.37 to 1.81)	20.8 (17.8 to 23.4)	1217.2 (1045.9 to 1416.5)
		Mycoplasma spp.	468 (404 to 541)	951 (808 to 1130)	103.9 (73.0 to 138.6)	1.17 (1.02 to 1.32)	10.1 (8.66 to 11.3)	766.9 (660.2 to 891.9)
		Polymicrobial	33.8 (21.9 to 52.1)	76.3 (46.4 to 123)	125.5 (84.6 to 168.9)	0.32 (0.21 to 0.49)	3.11 (1.88 to 4.96)	858.1 (725.0 to 1010.5)
		Pseudomonas aeruginosa	403 (349 to 469)	1370 (1160 to 1620)	241.0 (188.3 to 302.7)	2.63 (2.31 to 2.96)	38 (32.9 to 42.4)	1349.0 (1167.0 to 1557.7)
		Respiratory syncytial virus	110 (94.1 to 130)	222 (74.1 to 400)	101.6 (-32.5 to 263.3)	0.36 (0.31 to 0.41)	2.98 (0.88 to 5.93)	741.7 (158.5 to 1553.2)

		Staphylococcus aureus	727 (637 to 830)	2600 (2200 to 3080)	258.1 (204.5 to 321.2)	9.7 (8.57 to 10.8)	147 (127 to 165)	1421.8 (1229.5 to 1628.7)
		Streptococcus pneumoniae	2820 (2480 to 3220)	4190 (3560 to 4970)	49.1 (26.7 to 75.1)	15.6 (13.9 to 17.3)	99 (86.2 to 110)	533.8 (457.9 to 623.1)
		Other viruses	996 (875 to 1150)	2770 (2360 to 3280)	178.5 (136.3 to 226.6)	2.18 (1.92 to 2.42)	25.7 (22.2 to 28.7)	1083.6 (932.4 to 1249.3)
		Other bacterial pathogens	1000 (858 to 1170)	1900 (1590 to 2300)	90.4 (60.3 to 125.1)	5.37 (4.65 to 6.12)	43.4 (36.1 to 50.7)	710.5 (610.4 to 829.3)
Azerbaijan	All Ages	*Acinetobacter baumannii*	3070 (2670 to 3550)	2480 (2190 to 2800)	-19.1 (-26.7 to -10.9)	196 (122 to 303)	54.1 (38.1 to 78.1)	-71.9 (-78.2 to -62.9)
		Chlamydia spp.	12500 (11400 to 13900)	9170 (8400 to 10000)	-26.8 (-32.7 to -20.1)	218 (186 to 252)	52.7 (43.8 to 63.3)	-75.7 (-80.7 to -69.3)
		Enterobacter spp.	1030 (862 to 1210)	1550 (1360 to 1750)	51.4 (34.9 to 69.1)	75.9 (50.2 to 116)	30.7 (23.1 to 42.2)	-58.6 (-68.2 to -44.2)
		Escherichia coli	3590 (3200 to 4030)	4690 (4270 to 5140)	31.0 (19.3 to 43.1)	260 (216 to 314)	97 (79.9 to 117)	-62.5 (-70.6 to -51.6)
		Fungus	2110 (1790 to 2450)	3250 (2850 to 3660)	54.7 (40.9 to 70.6)	38.1 (30.1 to 47.6)	30 (23.7 to 38)	-20.4 (-39.4 to 2.2)
		Group B Streptococcus	3210 (2860 to 3600)	3770 (3440 to 4170)	17.5 (6.7 to 28.8)	225 (187 to 271)	70.8 (58.7 to 86.8)	-68.3 (-75.1 to -59.1)
		Haemophilus influenzae	16600 (14900 to 18500)	12500 (11600 to 13600)	-24.2 (-31.1 to -17.2)	432 (357 to 509)	62.2 (50.7 to 75.4)	-85.5 (-88.6 to -81.3)
		Influenza	18100 (16700 to 19700)	1350 (311 to 3210)	-92.5 (-98.3 to -82.1)	693 (594 to 794)	16.2 (3.29 to 40.5)	-97.7 (-99.5 to -94.1)
		Klebsiella pneumoniae	10400 (9590 to 11500)	8490 (7880 to 9140)	-18.6 (-25.8 to -12.0)	614 (524 to 700)	159 (132 to 190)	-74.0 (-79.1 to -67.2)
		Legionella spp.	3260 (2880 to 3660)	6080 (5530 to 6690)	86.7 (71.0 to 104.3)	104 (76.9 to 143)	58.3 (47.6 to 71.4)	-43.2 (-57.9 to -26.0)
		Mycoplasma spp.	17100 (15600 to 18900)	16100 (14900 to 17400)	-6.1 (-13.2 to 0.9)	199 (168 to 235)	69.9 (56.8 to 84.8)	-64.7 (-72.4 to -55.2)
		Polymicrobial	692 (504 to 967)	640 (443 to 942)	-7.3 (-28.2 to 14.3)	134 (75.1 to 216)	41.1 (23.1 to 66.9)	-69.0 (-76.6 to -58.1)
		Pseudomonas aeruginosa	6080 (5550 to 6660)	9150 (8490 to 9930)	50.7 (38.7 to 62.7)	275 (233 to 322)	123 (102 to 147)	-55.1 (-64.3 to -43.3)
		Respiratory syncytial virus	18900 (17300 to 20700)	676 (156 to 1610)	-96.4 (-99.2 to -91.4)	698 (602 to 802)	13 (2.74 to 31.6)	-98.1 (-99.6 to -95.4)
		Staphylococcus aureus	9400 (8740 to 10100)	15600 (14500 to 16800)	65.8 (53.0 to 79.2)	551 (479 to 623)	305 (253 to 362)	-44.4 (-55.6 to -30.4)
		Streptococcus pneumoniae	103000 (96300 to 112000)	49500 (46400 to 52900)	-52.2 (-55.7 to -48.5)	2680 (2320 to 3030)	395 (325 to 471)	-85.2 (-88.3 to -81.6)
		Other viruses	50500 (46800 to 54800)	33100 (31100 to 35500)	-34.4 (-39.3 to -29.7)	677 (580 to 770)	162 (132 to 197)	-76.0 (-81.1 to -69.5)
		Other bacterial pathogens	9800 (8860 to 10900)	12400 (11000 to 13700)	26.2 (15.6 to 38.3)	178 (142 to 225)	87.8 (70.9 to 111)	-50.2 (-61.4 to -36.8)
	Under 5	*Acinetobacter baumannii*	591 (415 to 832)	99.3 (67.8 to 142)	-83.2 (-85.2 to -80.9)	167 (91.9 to 273)	31.9 (17.1 to 54.7)	-80.9 (-85.8 to -73.6)
		Chlamydia spp.	5420 (4770 to 6140)	1160 (1010 to 1330)	-78.6 (-80.9 to -76.0)	198 (166 to 231)	34.4 (27.2 to 44.3)	-82.5 (-86.8 to -76.8)

		Enterobacter spp.	437 (339 to 559)	165 (131 to 208)	-62.1 (-67.2 to -56.6)	69.4 (44.2 to 109)	18.5 (11.7 to 29.3)	-73.0 (-79.7 to -63.6)
		Escherichia coli	1650 (1420 to 1910)	633 (545 to 732)	-61.5 (-65.9 to -56.3)	238 (194 to 289)	58.8 (45.8 to 75.5)	-75.1 (-81.2 to -66.7)
		Fungus	289 (205 to 398)	95 (67.9 to 130)	-67.0 (-71.5 to -61.9)	18.3 (12.7 to 25.7)	3.61 (2.33 to 5.22)	-80.1 (-86.0 to -73.1)
		Group B Streptococcus	1490 (1280 to 1720)	488 (422 to 560)	-67.1 (-70.8 to -63.2)	212 (174 to 257)	50.2 (39.4 to 64.8)	-76.1 (-81.8 to -67.8)
		Haemophilus influenzae	10500 (9240 to 12000)	2820 (2460 to 3170)	-73.1 (-76.2 to -69.7)	406 (333 to 484)	39.2 (29.6 to 50.1)	-90.3 (-92.9 to -86.7)
		Influenza	8940 (8090 to 9960)	172 (40.4 to 412)	-98.1 (-99.6 to -95.4)	617 (522 to 716)	7.73 (1.67 to 18.9)	-98.7 (-99.7 to -96.9)
		Klebsiella pneumoniae	5780 (5160 to 6520)	1220 (1070 to 1390)	-78.9 (-81.3 to -76.5)	562 (474 to 646)	92.8 (71.6 to 119)	-83.4 (-87.3 to -78.2)
		Legionella spp.	507 (367 to 675)	176 (131 to 236)	-65.2 (-70.0 to -59.9)	83.5 (56.5 to 121)	23.2 (16.3 to 33)	-71.8 (-79.0 to -61.0)
		Mycoplasma spp.	6210 (5490 to 6990)	2710 (2400 to 3080)	-56.3 (-61.0 to -50.7)	169 (139 to 203)	41.2 (31.5 to 52.7)	-75.4 (-81.8 to -67.2)
		Polymicrobial	354 (218 to 542)	90.3 (55.6 to 141)	-74.4 (-77.6 to -70.5)	130 (71.3 to 212)	36 (18 to 61.2)	-72.2 (-79.5 to -61.2)
		Pseudomonas aeruginosa	2660 (2350 to 3010)	1320 (1160 to 1500)	-50.3 (-55.4 to -44.7)	248 (208 to 295)	73.7 (58.2 to 94.7)	-70.1 (-77.5 to -60.3)
		Respiratory syncytial virus	17300 (15800 to 19000)	489 (113 to 1160)	-97.2 (-99.3 to -93.2)	692 (596 to 797)	12.5 (2.63 to 30.2)	-98.2 (-99.6 to -95.6)
		Staphylococcus aureus	3490 (3140 to 3870)	1440 (1270 to 1630)	-58.7 (-62.3 to -54.5)	456 (388 to 523)	120 (93.5 to 152)	-73.6 (-80.2 to -65.1)
		Streptococcus pneumoniae	58500 (53200 to 64400)	7430 (6590 to 8440)	-87.3 (-88.4 to -86.1)	2400 (2070 to 2730)	184 (137 to 236)	-92.3 (-94.4 to -89.8)
		Other viruses	35000 (31800 to 38400)	8780 (7780 to 9910)	-74.9 (-77.0 to -72.6)	639 (545 to 730)	114 (88.7 to 147)	-82.0 (-86.5 to -76.4)
		Other bacterial pathogens	1810 (1470 to 2180)	495 (399 to 604)	-72.6 (-75.9 to -68.7)	126 (92.8 to 171)	24.8 (16.6 to 35.6)	-80.3 (-85.3 to -73.7)
	5-14 years	*Acinetobacter baumannii*	612 (480 to 779)	245 (199 to 302)	-59.7 (-65.2 to -53.5)	6.06 (4.79 to 7.46)	1.99 (1.52 to 2.46)	-66.9 (-75.4 to -57.9)
		Chlamydia spp.	2180 (1750 to 2720)	1130 (925 to 1390)	-47.8 (-54.8 to -39.7)	5.35 (4.24 to 6.39)	2.27 (1.8 to 2.75)	-57.1 (-68.2 to -45.0)
		Enterobacter spp.	149 (114 to 194)	148 (119 to 182)	-0.1 (-14.0 to 16.5)	1.4 (1.09 to 1.74)	1.13 (0.88 to 1.41)	-18.0 (-39.2 to 6.5)
		Escherichia coli	440 (350 to 553)	379 (311 to 454)	-13.4 (-25.0 to 0.4)	4.27 (3.48 to 5.19)	3.01 (2.4 to 3.64)	-29.0 (-46.6 to -9.5)
		Fungus	532 (415 to 681)	417 (337 to 514)	-21.4 (-32.1 to -9.0)	5.1 (4.02 to 6.26)	3.26 (2.55 to 4)	-35.5 (-52.2 to -17.3)
		Group B Streptococcus	518 (408 to 648)	451 (367 to 548)	-12.5 (-24.6 to 1.7)	3.57 (2.81 to 4.38)	2.54 (2 to 3.07)	-28.2 (-46.3 to -8.2)
		Haemophilus influenzae	1940 (1570 to 2420)	1460 (1210 to 1740)	-24.5 (-34.4 to -13.0)	7.2 (5.84 to 8.52)	3.15 (2.5 to 3.77)	-55.9 (-67.1 to -43.4)
		Influenza	2850 (2310 to 3490)	168 (38.3 to 412)	-94.1 (-98.6 to -85.7)	20.7 (16.7 to 24.2)	1.03 (0.23 to 2.56)	-95.0 (-98.9 to -87.5)

		Klebsiella pneumoniae	1300 (1060 to 1620)	876 (723 to 1040)	-32.3 (-40.7 to -22.4)	12.5 (10.2 to 14.9)	6.91 (5.53 to 8.31)	-44.4 (-58.4 to -28.7)
		Legionella spp.	792 (631 to 996)	760 (624 to 911)	-3.5 (-16.4 to 11.4)	5.27 (4.11 to 6.41)	4.13 (3.28 to 5.01)	-20.8 (-40.2 to 2.2)
		Mycoplasma spp.	4440 (3610 to 5490)	3070 (2520 to 3660)	-30.7 (-39.8 to -19.9)	11.4 (9.19 to 13.4)	6.43 (5.05 to 7.74)	-43.1 (-57.0 to -27.3)
		Polymicrobial	89.1 (57.9 to 132)	62.3 (38.8 to 95.9)	-30.1 (-40.4 to -18.4)	0.88 (0.58 to 1.31)	0.5 (0.31 to 0.78)	-42.6 (-57.7 to -26.4)
		Pseudomonas aeruginosa	917 (741 to 1130)	927 (761 to 1100)	1.5 (-11.5 to 16.9)	6.15 (4.96 to 7.26)	5.08 (4.11 to 6.14)	-16.7 (-37.4 to 6.0)
		Respiratory syncytial virus	594 (482 to 729)	36.2 (7.87 to 86.9)	-93.9 (-98.5 to -85.0)	1.96 (1.58 to 2.3)	0.1 (0.02 to 0.25)	-94.8 (-98.9 to -87.0)
		Staphylococcus aureus	1610 (1320 to 1980)	1690 (1400 to 2000)	4.9 (-8.4 to 20.6)	22.2 (17.8 to 26.2)	18.9 (15.1 to 22.6)	-13.9 (-35.8 to 10.3)
		Streptococcus pneumoniae	16700 (13700 to 20600)	7920 (6570 to 9390)	-52.4 (-58.3 to -45.0)	95.3 (77.1 to 112)	37 (29.8 to 44.2)	-60.9 (-70.6 to -50.0)
		Other viruses	5960 (4890 to 7330)	4910 (4070 to 5840)	-17.4 (-27.8 to -5.0)	13.4 (10.9 to 15.7)	9.01 (7.27 to 10.8)	-32.2 (-49.2 to -13.3)
		Other bacterial pathogens	1970 (1590 to 2450)	1230 (1000 to 1490)	-37.2 (-45.5 to -27.6)	10.7 (8.6 to 12.8)	5.48 (4.32 to 6.62)	-48.4 (-61.4 to -34.3)
	15-49 years	*Acinetobacter baumannii*	562 (473 to 663)	407 (347 to 478)	-27.4 (-34.5 to -19.6)	5.86 (4.63 to 7.41)	2.77 (1.82 to 3.87)	-52.3 (-68.9 to -32.1)
		Chlamydia spp.	2000 (1720 to 2290)	1880 (1630 to 2120)	-5.8 (-14.7 to 5.2)	5.17 (4.16 to 6.31)	3.17 (2.12 to 4.36)	-38.2 (-60.1 to -11.1)
		Enterobacter spp.	137 (113 to 164)	245 (209 to 285)	80.2 (62.3 to 101.6)	1.35 (1.04 to 1.72)	1.58 (1.06 to 2.22)	18.3 (-24.1 to 68.6)
		Escherichia coli	404 (348 to 468)	629 (556 to 722)	56.1 (41.5 to 73.1)	4.13 (3.34 to 5.12)	4.19 (2.82 to 5.78)	2.4 (-33.5 to 46.6)
		Fungus	489 (406 to 578)	692 (590 to 807)	41.8 (28.6 to 58.3)	4.93 (3.88 to 6.28)	4.54 (3.04 to 6.26)	-7.0 (-39.8 to 32.6)
		Group B Streptococcus	476 (411 to 558)	749 (654 to 865)	57.8 (41.9 to 75.8)	3.45 (2.7 to 4.33)	3.54 (2.4 to 4.89)	3.6 (-33.1 to 48.3)
		Haemophilus influenzae	1790 (1560 to 2010)	2430 (2160 to 2710)	36.1 (24.5 to 50.6)	6.97 (5.69 to 8.56)	4.38 (2.96 to 6.05)	-36.5 (-58.7 to -9.9)
		Influenza	2620 (2330 to 2930)	280 (64.6 to 677)	-89.3 (-97.7 to -74.2)	20 (16.4 to 24.4)	1.46 (0.3 to 3.81)	-92.6 (-98.5 to -79.9)
		Klebsiella pneumoniae	1190 (1060 to 1330)	1450 (1300 to 1630)	22.1 (11.9 to 34.9)	12.1 (9.83 to 14.7)	9.62 (6.56 to 13.2)	-19.9 (-47.9 to 14.8)
		Legionella spp.	728 (622 to 841)	1260 (1120 to 1430)	74.0 (57.9 to 94.6)	5.09 (4.08 to 6.28)	5.75 (3.9 to 7.88)	14.2 (-26.1 to 65.5)
		Mycoplasma spp.	4080 (3610 to 4560)	5090 (4530 to 5700)	25.0 (14.4 to 38.2)	11 (8.99 to 13.5)	8.96 (6.03 to 12.3)	-17.9 (-46.4 to 18.1)
		Polymicrobial	81.8 (54.4 to 118)	103 (66.4 to 156)	26.1 (11.9 to 43.1)	0.85 (0.56 to 1.24)	0.7 (0.4 to 1.17)	-17.2 (-45.6 to 18.8)
		Pseudomonas aeruginosa	843 (743 to 947)	1540 (1370 to 1740)	83.0 (67.8 to 102.3)	5.95 (4.79 to 7.23)	7.07 (4.8 to 9.78)	20.1 (-22.0 to 73.3)
		Respiratory syncytial virus	545 (480 to 613)	60.2 (13.2 to 144)	-88.9 (-97.5 to -73.5)	1.9 (1.55 to 2.34)	0.14 (0.03 to 0.37)	-92.4 (-98.5 to -79.2)

		Staphylococcus aureus	1480 (1330 to 1650)	2800 (2510 to 3130)	89.1 (74.1 to 108.3)	21.4 (17.4 to 26.1)	26.3 (17.9 to 36.1)	24.2 (-19.3 to 78.2)
		Streptococcus pneumoniae	15300 (13800 to 16800)	13200 (11900 to 14600)	-14.1 (-20.7 to -5.3)	92.2 (75.4 to 112)	51.5 (35.2 to 70.3)	-43.6 (-63.1 to -19.2)
		Other viruses	5480 (4930 to 6060)	8150 (7340 to 9030)	49.0 (37.3 to 64.6)	12.9 (10.6 to 15.6)	12.5 (8.53 to 17.1)	-2.2 (-36.3 to 40.0)
		Other bacterial pathogens	1810 (1580 to 2070)	2050 (1780 to 2350)	13.3 (3.5 to 25.7)	10.3 (8.34 to 12.7)	7.63 (5.11 to 10.7)	-25.5 (-51.5 to 7.4)
	50-69 years	*Acinetobacter baumannii*	780 (650 to 935)	1040 (886 to 1220)	34.1 (17.2 to 52.9)	9.34 (7.31 to 12)	8.29 (6.14 to 11)	-10.3 (-36.0 to 22.9)
		Chlamydia spp.	1640 (1410 to 1910)	2810 (2460 to 3220)	71.7 (49.0 to 95.7)	4.88 (3.88 to 6.05)	5.55 (4.14 to 7.34)	14.9 (-17.7 to 57.8)
		Enterobacter spp.	184 (150 to 223)	606 (514 to 716)	230.2 (186.3 to 281.6)	2.09 (1.62 to 2.68)	4.56 (3.36 to 6)	120.8 (59.3 to 211.3)
		Escherichia coli	602 (514 to 708)	1690 (1480 to 1940)	182.3 (149.4 to 223.4)	7.06 (5.57 to 8.83)	13.2 (9.98 to 17.4)	88.9 (36.5 to 158.6)
		Fungus	579 (483 to 687)	1500 (1270 to 1750)	160.2 (127.8 to 197.0)	6.7 (5.24 to 8.44)	11.5 (8.58 to 15.3)	74.0 (25.8 to 139.8)
		Group B Streptococcus	454 (384 to 530)	1300 (1120 to 1510)	187.5 (151.1 to 226.3)	3.78 (2.93 to 4.79)	7.19 (5.42 to 9.33)	92.4 (37.9 to 168.8)
		Haemophilus influenzae	1450 (1260 to 1680)	3680 (3240 to 4210)	153.5 (123.7 to 189.6)	6.51 (5.2 to 8.01)	7.77 (5.88 to 10.1)	20.5 (-13.2 to 66.1)
		Influenza	1840 (1620 to 2090)	366 (85 to 885)	-80.0 (-95.5 to -51.5)	16.1 (12.9 to 19.8)	2.22 (0.44 to 5.76)	-86.1 (-97.2 to -63.5)
		Klebsiella pneumoniae	1240 (1100 to 1410)	2870 (2530 to 3230)	131.3 (103.1 to 161.5)	14.5 (11.7 to 17.7)	22.2 (16.8 to 28.9)	54.7 (11.3 to 114.2)
		Legionella spp.	832 (717 to 969)	2640 (2300 to 3000)	217.9 (177.1 to 262.9)	6.68 (5.24 to 8.27)	14.1 (10.7 to 18.3)	112.7 (54.8 to 195.0)
		Mycoplasma spp.	1520 (1320 to 1750)	3340 (2940 to 3770)	120.1 (91.8 to 149.7)	4.72 (3.81 to 5.75)	6.88 (5.18 to 8.99)	47.2 (6.2 to 103.3)
		Polymicrobial	94.5 (63.1 to 136)	220 (143 to 336)	132.1 (100.1 to 169.8)	1.12 (0.73 to 1.66)	1.74 (1.01 to 2.8)	55.3 (10.7 to 114.0)
		Pseudomonas aeruginosa	972 (844 to 1110)	3190 (2800 to 3630)	228.9 (189.3 to 273.4)	7.87 (6.36 to 9.58)	17.1 (12.9 to 22.3)	120.0 (59.2 to 201.0)
		Respiratory syncytial virus	283 (245 to 326)	56.5 (12.9 to 137)	-80.0 (-95.4 to -51.6)	1.13 (0.91 to 1.4)	0.16 (0.03 to 0.41)	-86.0 (-97.2 to -63.4)
		Staphylococcus aureus	1500 (1330 to 1700)	5230 (4610 to 5920)	248.0 (207.0 to 293.6)	24.9 (20.3 to 30.4)	57.5 (43.9 to 74.3)	132.8 (69.1 to 221.5)
		Streptococcus pneumoniae	7680 (6820 to 8670)	12500 (11100 to 14100)	63.4 (43.7 to 85.2)	53 (43 to 64.4)	57.4 (43.5 to 73.8)	9.3 (-20.8 to 50.4)
		Other viruses	2400 (2120 to 2710)	6780 (6000 to 7620)	182.8 (148.8 to 220.7)	6.51 (5.27 to 7.94)	12.2 (9.28 to 15.6)	89.1 (37.3 to 159.6)
		Other bacterial pathogens	2220 (1930 to 2550)	4580 (3920 to 5290)	106.7 (81.2 to 135.6)	14.6 (11.7 to 17.9)	20 (15 to 26.6)	38.4 (0.2 to 91.0)
	70+ years	*Acinetobacter baumannii*	524 (440 to 628)	681 (577 to 802)	30.6 (13.5 to 48.3)	7.63 (5.7 to 10.2)	9.16 (6.35 to 13.1)	22.1 (-21.2 to 77.6)
		Chlamydia spp.	1310 (1110 to 1530)	2190 (1890 to 2510)	67.1 (45.9 to 88.9)	4.75 (3.59 to 6.16)	7.3 (5.08 to 10.2)	56.3 (1.6 to 125.2)

		Enterobacter spp.	121 (97.1 to 146)	385 (322 to 455)	221.3 (177.6 to 264.2)	1.66 (1.24 to 2.24)	4.91 (3.37 to 6.95)	200.5 (93.6 to 339.5)
		Escherichia coli	496 (420 to 586)	1360 (1170 to 1560)	174.8 (138.9 to 211.7)	7.08 (5.41 to 9.35)	17.9 (12.5 to 24.6)	157.0 (66.1 to 271.1)
		Fungus	217 (177 to 260)	547 (462 to 637)	153.2 (119.0 to 187.1)	3.05 (2.27 to 4)	7.1 (4.84 to 10)	136.8 (52.5 to 243.4)
		Group B Streptococcus	280 (236 to 331)	780 (670 to 908)	179.8 (143.3 to 217.7)	2.83 (2.16 to 3.71)	7.29 (5 to 10.2)	161.7 (69.2 to 279.2)
		Haemophilus influenzae	880 (758 to 1020)	2160 (1890 to 2460)	146.7 (116.0 to 177.8)	4.79 (3.69 to 6.19)	7.73 (5.42 to 10.8)	64.0 (6.3 to 137.5)
		Influenza	1870 (1620 to 2150)	364 (82.2 to 894)	-80.6 (-95.6 to -52.5)	20 (15.6 to 25.7)	3.73 (0.72 to 9.75)	-81.0 (-96.5 to -49.2)
		Klebsiella pneumoniae	920 (793 to 1060)	2070 (1830 to 2340)	125.1 (96.7 to 153.9)	13.1 (10.1 to 16.9)	27.1 (19.1 to 37.3)	110.5 (35.3 to 204.7)
		Legionella spp.	402 (338 to 471)	1240 (1080 to 1430)	209.4 (169.6 to 249.8)	3.93 (3.01 to 5.13)	11.2 (7.8 to 15.5)	189.4 (86.5 to 317.4)
		Mycoplasma spp.	864 (740 to 1000)	1850 (1610 to 2110)	114.2 (86.7 to 143.1)	3.26 (2.52 to 4.25)	6.43 (4.48 to 8.81)	100.3 (30.4 to 188.9)
		Polymicrobial	72.8 (48 to 108)	165 (104 to 255)	125.9 (92.4 to 158.7)	1.05 (0.65 to 1.65)	2.2 (1.22 to 3.66)	111.3 (34.0 to 209.5)
		Pseudomonas aeruginosa	680 (587 to 787)	2170 (1920 to 2460)	220.1 (180.1 to 261.2)	6.7 (5.16 to 8.72)	19.7 (14 to 27.4)	199.4 (96.1 to 335.0)
		Respiratory syncytial virus	178 (152 to 208)	34.6 (7.78 to 84.8)	-80.5 (-95.7 to -52.7)	0.87 (0.67 to 1.11)	0.16 (0.03 to 0.43)	-81.0 (-96.5 to -49.6)
		Staphylococcus aureus	1310 (1140 to 1490)	4420 (3900 to 5040)	238.7 (196.8 to 282.3)	26.4 (20.6 to 33.9)	82.3 (57.5 to 114)	216.7 (106.4 to 357.7)
		Streptococcus pneumoniae	5310 (4610 to 6050)	8420 (7460 to 9580)	59.0 (39.1 to 78.6)	44.6 (35.1 to 57.4)	65.2 (46.1 to 90)	48.7 (-3.8 to 115.7)
		Other viruses	1640 (1430 to 1870)	4500 (3960 to 5120)	175.2 (141.8 to 208.6)	5.42 (4.24 to 6.98)	13.7 (9.61 to 19)	157.4 (67.2 to 272.9)
		Other bacterial pathogens	2000 (1710 to 2310)	4010 (3420 to 4630)	101.2 (75.2 to 128.2)	16.1 (12.3 to 20.9)	29.9 (20.6 to 41.4)	88.4 (22.6 to 175.0)
Georgia	All Ages	*Acinetobacter baumannii*	1460 (1280 to 1660)	1250 (1100 to 1420)	-14.1 (-21.1 to -6.8)	35.5 (26.1 to 48.4)	36.1 (30.4 to 42.6)	4.1 (-27.4 to 42.0)
		Chlamydia spp.	5650 (5170 to 6200)	3820 (3490 to 4150)	-32.3 (-37.7 to -26.9)	38.4 (34.6 to 42.9)	26.7 (22.8 to 31.1)	-30.3 (-41.7 to -17.5)
		Enterobacter spp.	656 (571 to 747)	759 (662 to 856)	15.9 (6.0 to 25.9)	17.6 (13.4 to 23.4)	20.3 (17.1 to 24)	17.6 (-14.9 to 53.0)
		Escherichia coli	2020 (1820 to 2240)	2220 (2020 to 2440)	10.0 (1.3 to 18.7)	54.7 (48.6 to 62.3)	64.4 (55 to 74.9)	18.0 (-2.7 to 40.6)
		Fungus	1450 (1270 to 1640)	1460 (1300 to 1630)	1.0 (-7.1 to 8.9)	17.3 (15.1 to 19.7)	35 (29.5 to 41.6)	102.3 (71.8 to 137.6)
		Group B Streptococcus	1730 (1550 to 1940)	1660 (1510 to 1820)	-3.9 (-12.4 to 4.0)	42.6 (37.8 to 49)	31.5 (27 to 36.4)	-25.7 (-38.3 to -12.1)
		Haemophilus influenzae	7790 (7180 to 8480)	4740 (4380 to 5120)	-39.2 (-44.4 to -34.6)	91.4 (80.4 to 104)	32.4 (27.9 to 37)	-64.5 (-70.4 to -57.8)
		Influenza	9980 (9270 to 10800)	134 (31.5 to 446)	-98.7 (-99.7 to -95.5)	168 (152 to 186)	2.79 (0.62 to 9.11)	-98.3 (-99.6 to -94.5)

		Klebsiella pneumoniae	4960 (4630 to 5370)	3950 (3650 to 4270)	-20.3 (-26.3 to -14.7)	127 (115 to 142)	109 (93.6 to 125)	-14.5 (-28.5 to 0.8)
		Legionella spp.	3040 (2730 to 3400)	3020 (2760 to 3290)	-0.4 (-8.7 to 8.0)	35.6 (30.8 to 41.4)	53.3 (45.7 to 61.3)	50.2 (22.8 to 79.5)
		Mycoplasma spp.	9350 (8650 to 10200)	5460 (5090 to 5850)	-41.5 (-46.0 to -37.3)	48.5 (43.9 to 53.9)	31.8 (27.6 to 36)	-34.3 (-44.4 to -23.6)
		Polymicrobial	300 (209 to 435)	279 (180 to 429)	-7.5 (-22.1 to 5.0)	24.1 (13.9 to 37.8)	8.65 (5.73 to 13)	-62.2 (-78.9 to -34.8)
		Pseudomonas aeruginosa	4310 (3950 to 4670)	4590 (4230 to 4970)	6.6 (-1.6 to 14.6)	72.9 (65.4 to 81.9)	87 (75 to 100)	19.7 (0.8 to 42.2)
		Respiratory syncytial virus	6830 (6190 to 7610)	34.9 (8.12 to 115)	-99.5 (-99.9 to -98.3)	147 (128 to 168)	0.27 (0.06 to 0.86)	-99.8 (-100.0 to -99.4)
		Staphylococcus aureus	6430 (5990 to 6930)	7830 (7240 to 8490)	21.9 (13.2 to 30.1)	169 (156 to 183)	314 (269 to 363)	86.4 (58.6 to 116.6)
		Streptococcus pneumoniae	47600 (44600 to 51300)	20300 (18900 to 21600)	-57.4 (-60.8 to -54.6)	644 (581 to 713)	292 (254 to 333)	-54.5 (-61.7 to -47.0)
		Other viruses	24900 (23200 to 27000)	12400 (11600 to 13200)	-50.2 (-54.2 to -46.7)	175 (156 to 197)	68.2 (59.6 to 77.1)	-60.9 (-67.1 to -54.2)
		Other bacterial pathogens	6890 (6180 to 7720)	6320 (5650 to 7070)	-8.1 (-15.3 to -0.7)	58.4 (51.2 to 66.8)	105 (87.9 to 123)	79.6 (47.5 to 116.1)
	Under 5	*Acinetobacter baumannii*	87 (60.8 to 122)	8.79 (6.35 to 12)	-89.8 (-91.1 to -88.3)	20.6 (11.5 to 33.2)	0.74 (0.42 to 1.27)	-96.4 (-97.3 to -95.3)
		Chlamydia spp.	999 (865 to 1160)	127 (109 to 148)	-87.3 (-89.0 to -85.3)	26 (22.3 to 30.2)	0.96 (0.75 to 1.24)	-96.3 (-97.2 to -95.2)
		Enterobacter spp.	106 (82.5 to 134)	19.7 (15.4 to 25.3)	-81.3 (-84.0 to -78.1)	12 (8 to 17.9)	0.57 (0.39 to 0.83)	-95.2 (-96.4 to -93.6)
		Escherichia coli	357 (305 to 426)	72.4 (61.2 to 84.7)	-79.7 (-82.6 to -76.7)	36.9 (31.2 to 43.9)	1.83 (1.4 to 2.39)	-95.0 (-96.2 to -93.5)
		Fungus	66.3 (46.5 to 91.9)	12 (8.41 to 16.3)	-81.9 (-84.8 to -78.7)	3.16 (2.21 to 4.33)	0.17 (0.11 to 0.25)	-94.6 (-96.0 to -92.8)
		Group B Streptococcus	322 (273 to 378)	55.6 (46.5 to 66.4)	-82.7 (-84.9 to -80.3)	32.1 (27.5 to 38.1)	1.41 (1.1 to 1.82)	-95.6 (-96.6 to -94.3)
		Haemophilus influenzae	2660 (2300 to 3100)	338 (290 to 388)	-87.2 (-89.1 to -85.3)	70.8 (60.2 to 82.9)	1.5 (1.16 to 1.96)	-97.9 (-98.4 to -97.2)
		Influenza	2210 (1940 to 2550)	4.71 (1.06 to 15.6)	-99.8 (-100.0 to -99.3)	106 (91.2 to 122)	0.06 (0.01 to 0.2)	-99.9 (-100.0 to -99.8)
		Klebsiella pneumoniae	1290 (1120 to 1480)	151 (130 to 176)	-88.2 (-89.7 to -86.7)	88.5 (76.2 to 103)	3.2 (2.45 to 4.14)	-96.4 (-97.2 to -95.3)
		Legionella spp.	132 (96.2 to 178)	19.8 (14 to 27.5)	-84.9 (-87.0 to -82.5)	14.8 (10.9 to 20)	0.64 (0.44 to 0.89)	-95.7 (-96.7 to -94.3)
		Mycoplasma spp.	1510 (1310 to 1780)	336 (288 to 390)	-77.8 (-80.8 to -74.5)	27.3 (23.4 to 31.9)	1.58 (1.21 to 2.07)	-94.2 (-95.7 to -92.4)
		Polymicrobial	67.5 (40.7 to 106)	7.21 (4.46 to 10.8)	-89.3 (-91.0 to -87.4)	21.6 (11.6 to 34.8)	0.87 (0.45 to 1.47)	-95.9 (-96.9 to -94.6)
		Pseudomonas aeruginosa	744 (642 to 864)	174 (150 to 201)	-76.5 (-79.7 to -73.1)	46.7 (39.8 to 54.6)	2.65 (2.05 to 3.41)	-94.3 (-95.7 to -92.5)
		Respiratory syncytial virus	5290 (4680 to 6050)	14.9 (3.38 to 49.9)	-99.7 (-99.9 to -99.1)	141 (123 to 162)	0.1 (0.02 to 0.34)	-99.9 (-100.0 to -99.8)

		Staphylococcus aureus	912 (800 to 1060)	185 (160 to 213)	-79.7 (-82.1 to -77.1)	85.4 (73.8 to 97.8)	4.75 (3.63 to 6.13)	-94.4 (-95.8 to -92.7)
		Streptococcus pneumoniae	14400 (12700 to 16600)	1020 (879 to 1170)	-92.9 (-93.7 to -92.0)	440 (380 to 505)	9.15 (6.99 to 12)	-97.9 (-98.4 to -97.3)
		Other viruses	10800 (9530 to 12300)	1210 (1050 to 1380)	-88.8 (-90.0 to -87.3)	141 (122 to 162)	4.9 (3.76 to 6.4)	-96.5 (-97.4 to -95.5)
		Other bacterial pathogens	391 (309 to 484)	58.4 (46.6 to 72.2)	-85.0 (-87.2 to -82.4)	19 (14.1 to 25.8)	0.8 (0.56 to 1.11)	-95.8 (-96.8 to -94.4)
	5-14 years	*Acinetobacter baumannii*	192 (154 to 238)	41.6 (32.9 to 51.7)	-78.2 (-81.9 to -74.1)	1.51 (1.28 to 1.77)	0.1 (0.07 to 0.12)	-93.7 (-94.9 to -92.5)
		Chlamydia spp.	867 (712 to 1050)	181 (143 to 221)	-79.0 (-82.4 to -75.0)	1.7 (1.5 to 1.94)	0.1 (0.08 to 0.12)	-93.9 (-95.1 to -92.8)
		Enterobacter spp.	79.1 (63.5 to 99.7)	25.9 (20.2 to 32.5)	-67.1 (-72.5 to -60.8)	0.59 (0.5 to 0.7)	0.06 (0.04 to 0.07)	-90.5 (-92.2 to -88.7)
		Escherichia coli	206 (168 to 251)	62 (49.3 to 74.9)	-69.8 (-74.8 to -64.0)	1.59 (1.4 to 1.79)	0.14 (0.11 to 0.16)	-91.2 (-92.9 to -89.6)
		Fungus	248 (201 to 305)	70.6 (56.1 to 86.8)	-71.5 (-76.0 to -65.8)	1.89 (1.61 to 2.21)	0.16 (0.12 to 0.19)	-91.7 (-93.3 to -90.2)
		Group B Streptococcus	258 (211 to 318)	78.1 (61.3 to 95.5)	-69.6 (-74.5 to -63.9)	1.42 (1.23 to 1.65)	0.12 (0.1 to 0.15)	-91.2 (-92.8 to -89.6)
		Haemophilus influenzae	1040 (867 to 1250)	240 (191 to 288)	-76.8 (-80.6 to -72.7)	3.06 (2.73 to 3.42)	0.14 (0.12 to 0.17)	-95.2 (-96.1 to -94.4)
		Influenza	1480 (1220 to 1780)	6.24 (1.37 to 20.2)	-99.6 (-99.9 to -98.6)	8.52 (7.74 to 9.42)	0.01 (0 to 0.03)	-99.9 (-100.0 to -99.6)
		Klebsiella pneumoniae	605 (503 to 726)	152 (121 to 183)	-74.9 (-78.8 to -70.2)	4.65 (4.21 to 5.19)	0.34 (0.27 to 0.4)	-92.7 (-94.0 to -91.4)
		Legionella spp.	505 (416 to 617)	138 (111 to 168)	-72.6 (-77.1 to -67.3)	2.67 (2.34 to 3.07)	0.21 (0.17 to 0.25)	-92.1 (-93.6 to -90.6)
		Mycoplasma spp.	2300 (1920 to 2770)	529 (420 to 641)	-77.0 (-80.7 to -72.8)	4.71 (4.23 to 5.27)	0.31 (0.25 to 0.37)	-93.3 (-94.6 to -92.1)
		Polymicrobial	35.3 (22.4 to 54.7)	10 (6.15 to 15.5)	-71.6 (-76.2 to -66.1)	0.28 (0.18 to 0.42)	0.02 (0.01 to 0.04)	-91.8 (-93.3 to -90.2)
		Pseudomonas aeruginosa	558 (461 to 667)	174 (139 to 211)	-68.8 (-73.8 to -62.8)	2.98 (2.67 to 3.35)	0.27 (0.22 to 0.32)	-90.9 (-92.6 to -89.3)
		Respiratory syncytial virus	390 (324 to 473)	1.55 (0.34 to 5.03)	-99.6 (-99.9 to -98.6)	1.03 (0.92 to 1.15)	0 (0 to 0)	-99.9 (-100.0 to -99.6)
		Staphylococcus aureus	877 (735 to 1050)	298 (241 to 358)	-66.0 (-71.4 to -59.7)	9.59 (8.71 to 10.6)	0.94 (0.76 to 1.1)	-90.1 (-91.9 to -88.4)
		Streptococcus pneumoniae	8370 (7030 to 9950)	1440 (1170 to 1720)	-82.7 (-85.5 to -79.6)	38.1 (34.8 to 41.9)	1.91 (1.54 to 2.23)	-95.0 (-95.9 to -94.1)
		Other viruses	3790 (3170 to 4510)	920 (747 to 1110)	-75.6 (-79.5 to -71.3)	6.77 (6.16 to 7.48)	0.48 (0.38 to 0.56)	-92.9 (-94.2 to -91.7)
		Other bacterial pathogens	897 (729 to 1090)	204 (161 to 251)	-77.2 (-81.0 to -72.9)	3.89 (3.41 to 4.4)	0.26 (0.2 to 0.3)	-93.4 (-94.6 to -92.2)
	15-49 years	*Acinetobacter baumannii*	221 (185 to 260)	103 (89.2 to 120)	-53.1 (-57.6 to -47.6)	2.63 (2.19 to 3.15)	1.31 (1.06 to 1.62)	-49.9 (-59.7 to -39.3)
		Chlamydia spp.	996 (875 to 1150)	449 (400 to 506)	-54.8 (-59.0 to -50.1)	2.95 (2.52 to 3.43)	1.42 (1.16 to 1.68)	-51.8 (-61.1 to -40.9)

		Enterobacter spp.	91 (76.8 to 108)	64.2 (55.1 to 74.2)	-29.2 (-36.4 to -21.3)	1.03 (0.86 to 1.23)	0.77 (0.63 to 0.94)	-24.4 (-39.7 to -7.9)
		Escherichia coli	237 (207 to 274)	154 (137 to 172)	-35.0 (-41.2 to -27.9)	2.77 (2.36 to 3.21)	1.91 (1.59 to 2.27)	-30.6 (-44.4 to -14.8)
		Fungus	285 (244 to 332)	175 (152 to 200)	-38.6 (-44.7 to -31.5)	3.29 (2.76 to 3.91)	2.15 (1.75 to 2.59)	-34.4 (-47.6 to -19.8)
		Group B Streptococcus	296 (257 to 345)	194 (171 to 220)	-34.5 (-41.0 to -27.0)	2.46 (2.07 to 2.92)	1.71 (1.4 to 2.04)	-30.1 (-44.1 to -14.7)
		Haemophilus influenzae	1190 (1060 to 1350)	594 (540 to 665)	-50.1 (-54.5 to -45.0)	5.32 (4.65 to 6.13)	2 (1.66 to 2.35)	-62.3 (-69.6 to -54.2)
		Influenza	1700 (1530 to 1890)	15.5 (3.57 to 51)	-99.1 (-99.8 to -97.0)	14.8 (12.9 to 16.8)	0.14 (0.03 to 0.49)	-99.0 (-99.8 to -96.8)
		Klebsiella pneumoniae	695 (621 to 782)	376 (343 to 416)	-45.9 (-50.8 to -40.5)	8.09 (7.01 to 9.22)	4.65 (3.88 to 5.5)	-42.2 (-53.3 to -30.5)
		Legionella spp.	581 (507 to 664)	342 (307 to 379)	-41.0 (-46.8 to -34.5)	4.65 (3.94 to 5.41)	2.91 (2.43 to 3.47)	-37.0 (-50.1 to -22.1)
		Mycoplasma spp.	2650 (2370 to 2970)	1310 (1190 to 1450)	-50.4 (-55.0 to -44.9)	8.19 (7.01 to 9.39)	4.31 (3.58 to 5.11)	-47.1 (-57.3 to -35.5)
		Polymicrobial	40.6 (25.6 to 60.5)	24.9 (15.8 to 38.4)	-38.7 (-45.1 to -32.0)	0.48 (0.31 to 0.72)	0.31 (0.19 to 0.49)	-34.6 (-47.6 to -20.1)
		Pseudomonas aeruginosa	642 (568 to 726)	431 (390 to 480)	-32.7 (-39.1 to -25.6)	5.18 (4.47 to 5.97)	3.71 (3.07 to 4.41)	-28.2 (-42.3 to -13.4)
		Respiratory syncytial virus	449 (395 to 507)	3.85 (0.87 to 12.9)	-99.1 (-99.8 to -97.2)	1.79 (1.54 to 2.07)	0.02 (0 to 0.06)	-99.1 (-99.8 to -96.9)
		Staphylococcus aureus	1010 (908 to 1130)	738 (672 to 814)	-26.7 (-33.3 to -19.2)	16.7 (14.5 to 19.1)	13 (10.9 to 15.4)	-21.8 (-37.1 to -5.4)
		Streptococcus pneumoniae	9620 (8700 to 10700)	3580 (3270 to 3940)	-62.8 (-66.0 to -59.0)	66.2 (57.7 to 75.2)	26.2 (21.9 to 30.9)	-60.2 (-67.7 to -52.0)
		Other viruses	4350 (3930 to 4840)	2280 (2080 to 2510)	-47.5 (-52.2 to -42.0)	11.8 (10.3 to 13.4)	6.57 (5.52 to 7.77)	-44.0 (-54.6 to -32.5)
		Other bacterial pathogens	1030 (898 to 1190)	505 (445 to 579)	-50.9 (-55.6 to -45.8)	6.76 (5.81 to 7.91)	3.53 (2.86 to 4.26)	-47.6 (-57.9 to -36.1)
	50-69 years	*Acinetobacter baumannii*	530 (446 to 620)	510 (439 to 586)	-3.6 (-12.5 to 5.6)	5.47 (4.7 to 6.32)	10.1 (8.54 to 12)	85.4 (54.9 to 119.6)
		Chlamydia spp.	1420 (1250 to 1620)	1290 (1150 to 1430)	-8.8 (-16.6 to -0.6)	3.63 (3.22 to 4.07)	6.35 (5.42 to 7.39)	75.3 (47.3 to 108.6)
		Enterobacter spp.	212 (179 to 247)	306 (264 to 350)	44.3 (31.3 to 57.4)	2.08 (1.77 to 2.41)	5.74 (4.82 to 6.75)	177.3 (131.4 to 228.7)
		Escherichia coli	610 (531 to 691)	798 (707 to 895)	31.1 (20.3 to 43.4)	6.18 (5.5 to 7)	15.5 (13.4 to 18)	152.1 (111.4 to 200.3)
		Fungus	585 (506 to 675)	733 (643 to 828)	25.5 (14.5 to 37.4)	5.84 (5.07 to 6.69)	14 (11.8 to 16.7)	141.1 (102.4 to 184.9)
		Group B Streptococcus	489 (420 to 567)	647 (568 to 725)	32.6 (20.5 to 46.0)	3.52 (3.05 to 3.98)	8.94 (7.57 to 10.4)	154.8 (113.8 to 201.0)
		Haemophilus influenzae	1680 (1500 to 1890)	1750 (1570 to 1940)	4.2 (-3.8 to 13.0)	6.49 (5.84 to 7.18)	9.18 (7.86 to 10.5)	41.7 (19.0 to 66.6)
		Influenza	2060 (1850 to 2290)	39 (9.06 to 128)	-98.1 (-99.6 to -93.7)	15.6 (14.1 to 17)	0.56 (0.12 to 1.82)	-96.4 (-99.2 to -88.2)

		Klebsiella pneumoniae	1250 (1120 to 1390)	1440 (1290 to 1590)	15.0 (6.3 to 24.7)	12.6 (11.5 to 13.9)	27.9 (24 to 32.2)	121.1 (85.9 to 160.5)
		Legionella spp.	1150 (999 to 1320)	1380 (1240 to 1540)	20.2 (9.5 to 31.2)	7.97 (6.98 to 9.03)	18.4 (15.7 to 21.2)	131.0 (92.6 to 174.6)
		Mycoplasma spp.	1710 (1530 to 1920)	1670 (1480 to 1850)	-2.5 (-10.5 to 5.8)	4.57 (4.12 to 5.1)	8.55 (7.38 to 9.87)	87.4 (57.9 to 120.7)
		Polymicrobial	81.2 (53.1 to 123)	102 (64.8 to 156)	25.5 (14.5 to 38.3)	0.83 (0.55 to 1.28)	2.01 (1.28 to 3.11)	141.1 (102.4 to 183.2)
		Pseudomonas aeruginosa	1280 (1140 to 1430)	1730 (1550 to 1910)	35.3 (24.3 to 46.9)	8.96 (8.08 to 9.95)	23.2 (20 to 26.8)	160.0 (117.1 to 208.5)
		Respiratory syncytial virus	403 (353 to 460)	6.98 (1.6 to 23)	-98.3 (-99.6 to -94.2)	1.39 (1.23 to 1.56)	0.05 (0.01 to 0.15)	-96.7 (-99.3 to -89.2)
		Staphylococcus aureus	1770 (1600 to 1960)	2650 (2390 to 2910)	49.9 (38.6 to 62.0)	25.3 (23.1 to 27.7)	72.9 (62.8 to 83.9)	188.2 (141.5 to 239.7)
		Streptococcus pneumoniae	8340 (7540 to 9240)	6510 (5860 to 7170)	-22.0 (-27.8 to -15.5)	49.7 (45.5 to 54.3)	74.4 (64.5 to 85.4)	50.0 (26.0 to 76.4)
		Other viruses	3300 (2980 to 3660)	3680 (3300 to 4050)	11.4 (2.9 to 20.9)	7.74 (7.05 to 8.43)	16.5 (14.3 to 19)	114.2 (80.3 to 152.4)
		Other bacterial pathogens	2190 (1910 to 2520)	2190 (1920 to 2470)	-0.1 (-8.3 to 9.3)	12.4 (11 to 14)	23.8 (20.1 to 28.1)	92.0 (61.4 to 126.0)
	70+ years	*Acinetobacter baumannii*	429 (359 to 507)	587 (505 to 678)	37.3 (23.2 to 51.7)	5.3 (4.43 to 6.35)	23.9 (19.5 to 28.6)	352.2 (269.9 to 450.5)
		Chlamydia spp.	1370 (1190 to 1590)	1770 (1560 to 2020)	29.8 (16.8 to 43.0)	4.2 (3.55 to 4.96)	17.9 (14.8 to 21.2)	327.6 (248.8 to 421.9)
		Enterobacter spp.	168 (140 to 201)	343 (294 to 400)	105.4 (84.1 to 126.1)	1.96 (1.62 to 2.36)	13.2 (10.8 to 15.9)	576.5 (453.2 to 721.4)
		Escherichia coli	607 (526 to 705)	1130 (999 to 1290)	86.7 (68.5 to 105.1)	7.35 (6.27 to 8.63)	45 (37.5 to 53)	514.9 (401.1 to 644.1)
		Fungus	264 (223 to 315)	470 (404 to 541)	78.6 (60.3 to 95.7)	3.16 (2.65 to 3.77)	18.5 (15 to 22.6)	488.3 (384.1 to 616.2)
		Group B Streptococcus	364 (314 to 422)	684 (605 to 772)	88.7 (68.3 to 107.4)	3.13 (2.65 to 3.69)	19.4 (16 to 23)	521.7 (409.0 to 655.2)
		Haemophilus influenzae	1230 (1090 to 1400)	1820 (1620 to 2040)	48.4 (34.4 to 62.0)	5.67 (4.88 to 6.56)	19.5 (16.4 to 23)	245.8 (185.4 to 320.6)
		Influenza	2540 (2270 to 2900)	68.3 (15.8 to 229)	-97.3 (-99.4 to -90.9)	22.9 (20 to 26.4)	2.01 (0.44 to 6.52)	-91.2 (-98.1 to -71.0)
		Klebsiella pneumoniae	1120 (1000 to 1290)	1830 (1640 to 2050)	63.8 (48.9 to 78.3)	13.5 (11.7 to 15.5)	72.5 (60.9 to 85.5)	439.4 (346.2 to 554.0)
		Legionella spp.	670 (579 to 783)	1140 (1020 to 1290)	71.1 (53.2 to 88.7)	5.56 (4.67 to 6.54)	31.2 (25.9 to 36.9)	463.5 (358.6 to 589.9)
		Mycoplasma spp.	1170 (1030 to 1350)	1620 (1450 to 1830)	38.8 (25.1 to 51.7)	3.75 (3.22 to 4.35)	17.1 (14.3 to 20)	357.2 (275.8 to 450.4)
		Polymicrobial	75.4 (48.1 to 120)	135 (83.3 to 214)	78.6 (59.5 to 98.8)	0.93 (0.59 to 1.44)	5.43 (3.4 to 8.48)	488.2 (380.3 to 620.5)
		Pseudomonas aeruginosa	1080 (955 to 1250)	2080 (1850 to 2340)	92.6 (73.7 to 110.8)	9.04 (7.83 to 10.4)	57.1 (48.3 to 67.1)	534.3 (420.7 to 667.6)
		Respiratory syncytial virus	306 (266 to 356)	7.54 (1.74 to 25.2)	-97.5 (-99.4 to -91.7)	1.26 (1.07 to 1.48)	0.1 (0.02 to 0.33)	-91.9 (-98.3 to -73.5)

		Staphylococcus aureus	1860 (1660 to 2130)	3960 (3530 to 4420)	113.4 (94.3 to 131.7)	31.8 (27.6 to 36.6)	223 (187 to 261)	603.0 (479.5 to 750.5)
		Streptococcus pneumoniae	6950 (6230 to 7980)	7710 (6890 to 8640)	11.1 (1.2 to 20.6)	49.6 (43.1 to 56.8)	181 (152 to 212)	266.0 (201.9 to 341.3)
		Other viruses	2720 (2440 to 3130)	4310 (3830 to 4830)	58.6 (44.5 to 72.4)	7.63 (6.62 to 8.72)	39.7 (33.2 to 46.6)	422.5 (330.6 to 532.1)
		Other bacterial pathogens	2380 (2060 to 2770)	3370 (2940 to 3870)	42.1 (27.7 to 55.3)	16.3 (13.8 to 19)	76.2 (62.7 to 90.8)	368.4 (285.3 to 467.5)
Kazakhstan	All Ages	*Acinetobacter baumannii*	5380 (4680 to 6130)	3560 (3130 to 4080)	-33.6 (-39.8 to -26.5)	127 (91.4 to 182)	69.9 (58.1 to 83.8)	-43.7 (-58.6 to -24.6)
		Chlamydia spp.	20000 (18100 to 22100)	13500 (12300 to 14800)	-32.6 (-38.8 to -25.5)	139 (123 to 155)	61.2 (52.6 to 69.9)	-55.7 (-62.7 to -48.0)
		Enterobacter spp.	1960 (1670 to 2260)	3130 (2770 to 3530)	60.1 (43.4 to 78.9)	54.7 (40.6 to 75.4)	54.2 (45.6 to 63.6)	1.2 (-25.6 to 34.0)
		Escherichia coli	6410 (5770 to 7140)	8610 (7840 to 9570)	34.7 (22.1 to 50.2)	181 (158 to 207)	159 (136 to 182)	-11.7 (-26.2 to 4.8)
		Fungus	4520 (3920 to 5150)	6010 (5370 to 6830)	33.3 (20.5 to 47.3)	45.9 (39 to 53.8)	89.8 (74.3 to 107)	96.4 (60.5 to 140.3)
		Group B Streptococcus	5490 (4900 to 6170)	7080 (6420 to 7880)	29.2 (16.4 to 43.8)	144 (125 to 165)	90.5 (77.9 to 103)	-36.8 (-47.4 to -25.0)
		Haemophilus influenzae	26000 (23700 to 28900)	20900 (19100 to 22900)	-19.8 (-27.6 to -11.5)	305 (268 to 348)	82.3 (71.2 to 94.3)	-72.9 (-77.3 to -68.1)
		Influenza	31600 (29200 to 34200)	6710 (2730 to 12800)	-78.7 (-91.6 to -59.5)	530 (482 to 585)	81.2 (30 to 162)	-84.6 (-94.4 to -69.8)
		Klebsiella pneumoniae	16900 (15600 to 18400)	14400 (13300 to 15900)	-14.3 (-22.0 to -6.1)	434 (393 to 481)	249 (213 to 284)	-42.6 (-51.2 to -33.3)
		Legionella spp.	7950 (7080 to 8870)	12400 (11300 to 13600)	55.8 (40.0 to 72.1)	92.1 (76.2 to 112)	139 (116 to 161)	52.1 (19.4 to 92.4)
		Mycoplasma spp.	30200 (27700 to 33200)	26700 (24500 to 29100)	-11.5 (-18.8 to -3.6)	151 (136 to 168)	96.8 (85 to 109)	-35.8 (-45.4 to -25.5)
		Polymicrobial	1010 (715 to 1420)	1090 (731 to 1640)	7.2 (-6.9 to 21.7)	81.2 (47 to 128)	30 (20.4 to 42.4)	-61.9 (-73.3 to -46.2)
		Pseudomonas aeruginosa	12100 (11200 to 13300)	18900 (17300 to 20700)	55.4 (42.5 to 69.6)	213 (192 to 239)	224 (193 to 256)	5.2 (-11.5 to 24.6)
		Respiratory syncytial virus	24500 (21900 to 27600)	3120 (1320 to 5820)	-87.2 (-94.8 to -76.2)	481 (429 to 538)	18.5 (7.14 to 36.4)	-96.2 (-98.6 to -92.5)
		Staphylococcus aureus	18900 (17500 to 20400)	32000 (29600 to 35000)	69.9 (57.4 to 83.8)	483 (443 to 526)	772 (657 to 889)	60.3 (36.0 to 87.8)
		Streptococcus pneumoniae	164000 (153000 to 178000)	71700 (66100 to 78100)	-56.2 (-59.9 to -52.3)	2100 (1940 to 2290)	616 (533 to 703)	-70.7 (-74.8 to -66.0)
		Other viruses	80400 (74400 to 88200)	58800 (54200 to 64000)	-26.9 (-33.7 to -19.8)	539 (490 to 592)	203 (179 to 229)	-62.2 (-67.4 to -56.7)
		Other bacterial pathogens	20900 (18700 to 23100)	20400 (18100 to 23100)	-2.5 (-11.3 to 7.3)	166 (143 to 194)	205 (171 to 242)	24.0 (-0.4 to 49.5)
	Under 5	*Acinetobacter baumannii*	430 (306 to 602)	119 (77.8 to 181)	-72.4 (-77.8 to -65.9)	84.3 (48.4 to 137)	10.2 (5.45 to 17.3)	-87.9 (-90.6 to -84.4)
		Chlamydia spp.	4940 (4220 to 5820)	1360 (1150 to 1610)	-72.3 (-77.2 to -66.7)	107 (92.6 to 123)	11.3 (9.11 to 14.1)	-89.4 (-91.7 to -86.8)

		Enterobacter spp.	447 (343 to 571)	257 (197 to 330)	-42.3 (-53.0 to -30.8)	42.4 (28.7 to 62.5)	7.51 (4.93 to 11.2)	-82.2 (-86.0 to -77.3)
		Escherichia coli	1620 (1360 to 1950)	922 (776 to 1090)	-42.9 (-53.4 to -32.3)	139 (118 to 164)	22.9 (18.3 to 28.7)	-83.5 (-87.2 to -79.1)
		Fungus	310 (216 to 432)	133 (94.4 to 182)	-56.7 (-65.4 to -46.4)	12.4 (8.69 to 16.5)	1.6 (1.09 to 2.3)	-87.0 (-90.2 to -83.2)
		Group B Streptococcus	1400 (1170 to 1680)	716 (597 to 863)	-48.8 (-57.9 to -39.0)	120 (102 to 141)	18.6 (15.2 to 23.2)	-84.4 (-87.8 to -80.3)
		Haemophilus influenzae	11500 (9870 to 13500)	3710 (3150 to 4320)	-67.6 (-73.1 to -61.4)	260 (224 to 301)	13.5 (10.7 to 16.9)	-94.8 (-95.9 to -93.4)
		Influenza	9740 (8440 to 11100)	656 (279 to 1200)	-93.2 (-97.1 to -87.4)	396 (351 to 449)	7.77 (3 to 14.8)	-98.0 (-99.3 to -96.1)
		Klebsiella pneumoniae	5970 (5140 to 6890)	1580 (1350 to 1840)	-73.4 (-77.8 to -68.6)	344 (305 to 389)	34.1 (27.3 to 42.5)	-90.0 (-92.1 to -87.5)
		Legionella spp.	506 (367 to 691)	272 (199 to 369)	-45.9 (-55.1 to -36.1)	50.5 (36 to 70)	9.1 (6.58 to 12.5)	-81.8 (-86.0 to -76.8)
		Mycoplasma spp.	6670 (5740 to 7860)	3810 (3280 to 4460)	-42.6 (-52.4 to -32.3)	104 (89.7 to 120)	16.5 (13.2 to 20.8)	-84.1 (-87.6 to -80.0)
		Polymicrobial	268 (169 to 405)	132 (75.4 to 206)	-51.0 (-59.3 to -41.5)	74.9 (40.8 to 120)	13.5 (6.91 to 22.9)	-81.9 (-86.1 to -76.9)
		Pseudomonas aeruginosa	2990 (2540 to 3470)	2190 (1890 to 2550)	-26.4 (-39.5 to -12.4)	160 (140 to 185)	32.4 (26.3 to 40.3)	-79.7 (-83.9 to -74.9)
		Respiratory syncytial virus	20500 (18000 to 23400)	2090 (892 to 3840)	-89.7 (-95.7 to -81.0)	471 (419 to 527)	13.6 (5.24 to 26.3)	-97.1 (-98.9 to -94.3)
		Staphylococcus aureus	3910 (3400 to 4500)	2290 (1970 to 2670)	-41.1 (-49.8 to -31.3)	305 (272 to 344)	54.4 (43.8 to 67.8)	-82.1 (-85.9 to -77.6)
		Streptococcus pneumoniae	64500 (56400 to 74000)	8200 (7060 to 9550)	-87.3 (-89.2 to -85.2)	1650 (1480 to 1830)	62.6 (50 to 78.3)	-96.2 (-97.0 to -95.3)
		Other viruses	42800 (37500 to 48900)	12500 (10800 to 14500)	-70.7 (-75.1 to -65.7)	471 (423 to 526)	48.5 (39.4 to 60.6)	-89.7 (-91.9 to -87.1)
		Other bacterial pathogens	1830 (1440 to 2270)	633 (498 to 796)	-65.3 (-71.8 to -58.0)	74.3 (56.3 to 99.2)	8.96 (6.1 to 13)	-87.9 (-90.5 to -84.7)
	5-14 years	*Acinetobacter baumannii*	759 (591 to 948)	253 (198 to 321)	-66.6 (-71.8 to -61.1)	3.9 (3.28 to 4.57)	0.65 (0.54 to 0.77)	-83.3 (-86.1 to -80.1)
		Chlamydia spp.	3030 (2430 to 3740)	1250 (977 to 1540)	-58.7 (-64.5 to -51.7)	3.86 (3.36 to 4.39)	0.79 (0.68 to 0.93)	-79.4 (-82.7 to -75.6)
		Enterobacter spp.	238 (183 to 300)	220 (169 to 277)	-7.3 (-22.1 to 9.9)	1.16 (0.96 to 1.37)	0.53 (0.45 to 0.64)	-53.7 (-61.4 to -44.7)
		Escherichia coli	661 (524 to 805)	519 (409 to 637)	-21.3 (-33.0 to -7.9)	3.33 (2.91 to 3.81)	1.3 (1.12 to 1.51)	-60.7 (-66.9 to -53.9)
		Fungus	798 (639 to 993)	554 (434 to 702)	-30.5 (-40.9 to -18.1)	3.97 (3.38 to 4.6)	1.37 (1.15 to 1.62)	-65.3 (-70.7 to -58.7)
		Group B Streptococcus	802 (642 to 987)	634 (500 to 800)	-20.7 (-32.9 to -7.6)	2.87 (2.46 to 3.3)	1.13 (0.97 to 1.32)	-60.4 (-66.5 to -53.0)
		Haemophilus influenzae	3110 (2510 to 3770)	1840 (1460 to 2280)	-40.7 (-48.9 to -30.7)	5.98 (5.28 to 6.74)	1.19 (1.03 to 1.37)	-80.0 (-83.1 to -76.5)
		Influenza	4490 (3660 to 5440)	625 (244 to 1210)	-86.0 (-94.5 to -73.2)	16.9 (15.3 to 18.7)	1.13 (0.44 to 2.2)	-93.3 (-97.4 to -86.7)

		Klebsiella pneumoniae	1950 (1580 to 2360)	1090 (861 to 1330)	-44.1 (-51.6 to -35.0)	9.75 (8.65 to 10.9)	2.71 (2.37 to 3.11)	-72.1 (-76.3 to -67.2)
		Legionella spp.	1380 (1100 to 1680)	1120 (885 to 1380)	-18.8 (-30.8 to -5.2)	4.76 (4.12 to 5.48)	1.92 (1.66 to 2.21)	-59.5 (-66.2 to -51.9)
		Mycoplasma spp.	7010 (5650 to 8470)	3960 (3100 to 4880)	-43.5 (-51.1 to -34.2)	9.33 (8.29 to 10.4)	2.62 (2.28 to 3)	-71.8 (-76.3 to -66.9)
		Polymicrobial	124 (79.2 to 183)	77.6 (47.6 to 120)	-37.3 (-46.8 to -26.1)	0.63 (0.41 to 0.93)	0.2 (0.12 to 0.3)	-68.7 (-74.1 to -62.2)
		Pseudomonas aeruginosa	1560 (1260 to 1890)	1440 (1130 to 1760)	-8.0 (-20.5 to 7.1)	5.43 (4.82 to 6.11)	2.49 (2.16 to 2.85)	-54.1 (-61.4 to -45.9)
		Respiratory syncytial virus	1050 (844 to 1280)	147 (57.5 to 286)	-86.0 (-94.5 to -73.3)	1.8 (1.58 to 2.02)	0.12 (0.05 to 0.24)	-93.2 (-97.4 to -86.6)
		Staphylococcus aureus	2610 (2130 to 3120)	2560 (2020 to 3140)	-1.7 (-14.6 to 14.0)	18.5 (16.7 to 20.5)	9.07 (7.94 to 10.4)	-51.0 (-58.5 to -42.4)
		Streptococcus pneumoniae	25900 (21500 to 31100)	8640 (6830 to 10600)	-66.6 (-70.9 to -61.1)	76.8 (69.3 to 84.8)	12.8 (11.2 to 14.6)	-83.3 (-85.9 to -80.6)
		Other viruses	10400 (8500 to 12400)	6750 (5330 to 8260)	-34.8 (-43.2 to -24.2)	12.1 (10.9 to 13.3)	3.92 (3.43 to 4.47)	-67.5 (-72.5 to -62.0)
		Other bacterial pathogens	2920 (2330 to 3530)	1480 (1160 to 1870)	-49.3 (-56.7 to -40.5)	8.24 (7.2 to 9.29)	2.08 (1.76 to 2.43)	-74.7 (-78.7 to -70.3)
	15-49 years	*Acinetobacter baumannii*	974 (825 to 1160)	614 (522 to 718)	-36.8 (-44.6 to -29.0)	8.07 (6.83 to 9.37)	7.56 (6.36 to 8.89)	-6.0 (-20.3 to 10.7)
		Chlamydia spp.	3890 (3390 to 4440)	3030 (2640 to 3420)	-21.9 (-30.2 to -11.9)	7.99 (7.07 to 9.09)	9.26 (7.86 to 10.7)	16.2 (-1.7 to 36.5)
		Enterobacter spp.	305 (255 to 363)	533 (455 to 613)	75.3 (53.5 to 98.6)	2.39 (2 to 2.8)	6.22 (5.26 to 7.24)	160.8 (119.2 to 209.5)
		Escherichia coli	849 (736 to 972)	1260 (1110 to 1430)	48.8 (33.2 to 67.1)	6.89 (6.04 to 7.78)	15.2 (13.2 to 17.5)	121.4 (87.4 to 159.2)
		Fungus	1030 (872 to 1200)	1350 (1160 to 1550)	31.5 (16.5 to 48.6)	8.21 (7.08 to 9.46)	16 (13.5 to 18.6)	95.6 (65.7 to 132.2)
		Group B Streptococcus	1030 (897 to 1210)	1540 (1340 to 1760)	50.1 (31.5 to 71.1)	5.93 (5.17 to 6.76)	13.2 (11.2 to 15.3)	123.3 (88.3 to 164.5)
		Haemophilus influenzae	3990 (3560 to 4490)	4480 (3980 to 5010)	12.2 (0.3 to 24.1)	12.4 (11.2 to 13.7)	13.9 (12 to 15.8)	12.6 (-3.9 to 32.0)
		Influenza	5770 (5200 to 6480)	1520 (623 to 2940)	-73.5 (-89.3 to -49.0)	35 (31.8 to 38.2)	13.3 (4.9 to 26.5)	-61.9 (-86.3 to -23.6)
		Klebsiella pneumoniae	2500 (2240 to 2800)	2640 (2360 to 2950)	5.8 (-4.6 to 17.6)	20.2 (18.4 to 22.1)	31.7 (27.5 to 35.9)	57.3 (35.0 to 83.2)
		Legionella spp.	1770 (1540 to 2040)	2720 (2420 to 3040)	53.7 (35.9 to 73.0)	9.85 (8.58 to 11.2)	22.4 (19.4 to 25.6)	128.6 (94.3 to 171.3)
		Mycoplasma spp.	9000 (8070 to 10100)	9610 (8600 to 10700)	6.9 (-4.3 to 19.3)	19.3 (17.5 to 21.2)	30.6 (26.8 to 34.7)	59.0 (34.7 to 86.5)
		Polymicrobial	159 (104 to 234)	189 (117 to 294)	18.7 (3.1 to 35.4)	1.31 (0.87 to 1.93)	2.31 (1.45 to 3.59)	76.5 (46.8 to 109.8)
		Pseudomonas aeruginosa	2010 (1780 to 2260)	3490 (3110 to 3890)	74.2 (55.3 to 94.0)	11.2 (10.2 to 12.4)	29.1 (25.2 to 33)	159.0 (121.0 to 202.1)
		Respiratory syncytial virus	1350 (1190 to 1520)	357 (148 to 697)	-73.4 (-89.3 to -48.8)	3.72 (3.36 to 4.11)	1.42 (0.52 to 2.88)	-61.7 (-86.4 to -24.0)

		Staphylococcus aureus	3350 (3000 to 3710)	6210 (5560 to 6910)	85.9 (68.4 to 106.0)	38.4 (35.1 to 41.9)	106 (92.5 to 120)	176.5 (137.1 to 220.7)
		Streptococcus pneumoniae	33300 (30200 to 36900)	21000 (18800 to 23400)	-36.8 (-42.8 to -30.3)	159 (147 to 172)	149 (131 to 168)	-6.0 (-19.6 to 9.6)
		Other viruses	13300 (12000 to 14800)	16400 (14700 to 18200)	23.3 (11.2 to 36.1)	25 (23.1 to 27.2)	45.7 (40.1 to 51.8)	83.4 (57.3 to 113.8)
		Other bacterial pathogens	3750 (3280 to 4280)	3590 (3130 to 4120)	-4.1 (-14.3 to 7.5)	17 (15.1 to 19.1)	24.3 (20.7 to 28.2)	42.8 (21.1 to 67.2)
	50-69 years	*Acinetobacter baumannii*	1960 (1670 to 2270)	1630 (1380 to 1900)	-16.6 (-26.4 to -5.1)	17.1 (14.2 to 20.4)	24.8 (20 to 30.3)	46.1 (15.4 to 83.9)
		Chlamydia spp.	4610 (4050 to 5140)	4620 (4030 to 5240)	0.2 (-11.8 to 13.7)	10 (8.46 to 11.7)	17.5 (14.1 to 21)	75.7 (39.1 to 119.4)
		Enterobacter spp.	595 (497 to 701)	1350 (1150 to 1580)	127.7 (100.4 to 160.0)	4.92 (4.03 to 5.94)	19.5 (15.9 to 23.6)	299.1 (214.5 to 404.2)
		Escherichia coli	1830 (1610 to 2070)	3440 (3040 to 3910)	88.8 (67.6 to 115.9)	15.6 (13.3 to 18.2)	51.5 (42.1 to 61.3)	230.8 (161.9 to 314.0)
		Fungus	1760 (1510 to 2020)	3000 (2590 to 3460)	71.5 (51.7 to 94.8)	14.8 (12.4 to 17.6)	44.3 (36 to 53.8)	200.5 (138.3 to 281.8)
		Group B Streptococcus	1420 (1230 to 1620)	2730 (2370 to 3150)	92.7 (68.6 to 117.6)	8.63 (7.21 to 10.2)	29 (23.5 to 34.5)	237.8 (167.7 to 326.6)
		Haemophilus influenzae	4700 (4230 to 5210)	7100 (6320 to 8060)	51.2 (35.6 to 69.5)	15.4 (13.4 to 17.6)	27.3 (22.2 to 32.6)	78.6 (42.9 to 125.0)
		Influenza	5850 (5290 to 6430)	2070 (848 to 4010)	-64.6 (-85.8 to -31.9)	37.4 (32.5 to 43)	22.4 (8.29 to 45.2)	-39.7 (-78.9 to 23.8)
		Klebsiella pneumoniae	3770 (3420 to 4120)	5550 (4950 to 6270)	47.4 (32.5 to 64.9)	32.1 (27.9 to 36.7)	82.5 (67.7 to 98.2)	158.3 (106.5 to 219.6)
		Legionella spp.	2930 (2580 to 3310)	5810 (5150 to 6600)	98.8 (73.9 to 125.4)	17.2 (14.5 to 20.1)	59.5 (48.8 to 70.9)	248.4 (178.8 to 343.9)
		Mycoplasma spp.	4860 (4340 to 5400)	6240 (5530 to 7050)	28.7 (14.1 to 44.9)	11 (9.42 to 12.7)	24.7 (20.4 to 29.3)	125.6 (79.7 to 182.0)
		Polymicrobial	265 (178 to 398)	414 (261 to 646)	55.5 (34.4 to 78.4)	2.3 (1.53 to 3.42)	6.27 (3.85 to 9.92)	172.5 (114.7 to 241.9)
		Pseudomonas aeruginosa	3350 (3020 to 3710)	7310 (6470 to 8210)	118.5 (94.8 to 146.3)	19.8 (17.1 to 22.6)	75.4 (62.2 to 89.8)	282.9 (205.5 to 376.7)
		Respiratory syncytial virus	1010 (891 to 1130)	340 (136 to 659)	-66.3 (-86.8 to -36.1)	2.95 (2.52 to 3.42)	1.68 (0.62 to 3.39)	-42.8 (-79.8 to 17.9)
		Staphylococcus aureus	4910 (4460 to 5350)	11900 (10700 to 13400)	143.5 (118.8 to 172.6)	59.4 (51.7 to 67.8)	252 (207 to 299)	326.6 (241.5 to 433.5)
		Streptococcus pneumoniae	24100 (22000 to 26200)	21100 (18800 to 23800)	-12.3 (-21.2 to -1.6)	121 (106 to 138)	186 (153 to 220)	53.7 (23.3 to 91.6)
		Other viruses	8440 (7680 to 9220)	14500 (12900 to 16300)	72.0 (54.9 to 92.5)	16.7 (14.6 to 19)	50.2 (41.2 to 59.8)	201.4 (140.8 to 277.0)
		Other bacterial pathogens	6660 (5920 to 7490)	8230 (7110 to 9580)	23.7 (9.8 to 40.2)	32 (27.2 to 36.7)	69.1 (56.5 to 83.6)	117.0 (73.3 to 171.0)
	70+ years	*Acinetobacter baumannii*	1260 (1030 to 1510)	949 (816 to 1120)	-24.3 (-34.3 to -13.3)	13.5 (11.3 to 15.8)	26.6 (21.4 to 32)	97.5 (58.3 to 140.9)
		Chlamydia spp.	3540 (3030 to 4240)	3210 (2840 to 3660)	-9.0 (-21.2 to 4.6)	9.46 (8.11 to 10.8)	22.4 (18.2 to 26.7)	137.4 (90.0 to 188.9)

		Enterobacter spp.	373 (303 to 461)	768 (652 to 895)	106.8 (77.5 to 135.8)	3.81 (3.1 to 4.56)	20.4 (16.5 to 24.5)	439.4 (328.1 to 562.9)
		Escherichia coli	1440 (1220 to 1740)	2470 (2160 to 2820)	71.4 (47.8 to 95.1)	15.2 (13 to 17.7)	67.9 (55.6 to 80)	347.2 (259.7 to 449.7)
		Fungus	630 (521 to 764)	977 (841 to 1140)	55.7 (34.2 to 78.6)	6.55 (5.5 to 7.76)	26.5 (21.6 to 32.2)	306.1 (228.4 to 399.0)
		Group B Streptococcus	839 (710 to 995)	1460 (1280 to 1690)	75.0 (50.0 to 100.0)	6.28 (5.3 to 7.31)	28.6 (23.3 to 33.9)	356.6 (267.0 to 459.4)
		Haemophilus influenzae	2730 (2360 to 3200)	3730 (3320 to 4300)	37.3 (20.0 to 55.5)	11 (9.51 to 12.5)	26.4 (21.6 to 31.3)	141.4 (94.7 to 193.1)
		Influenza	5730 (4980 to 6700)	1840 (724 to 3510)	-67.9 (-86.9 to -39.7)	45.1 (39.2 to 51)	36.6 (13.3 to 74.3)	-18.6 (-71.3 to 65.4)
		Klebsiella pneumoniae	2670 (2330 to 3140)	3570 (3190 to 4080)	33.9 (17.2 to 51.2)	28.1 (24.5 to 31.6)	97.6 (80 to 115)	249.1 (183.3 to 321.8)
		Legionella spp.	1360 (1150 to 1620)	2440 (2150 to 2790)	80.5 (54.8 to 106.7)	9.81 (8.3 to 11.4)	46 (37.5 to 54.5)	371.0 (277.9 to 483.1)
		Mycoplasma spp.	2650 (2290 to 3110)	3080 (2740 to 3550)	16.9 (1.7 to 32.3)	7.37 (6.33 to 8.42)	22.4 (18.3 to 26.6)	204.9 (144.8 to 267.9)
		Polymicrobial	196 (126 to 300)	277 (173 to 442)	41.2 (19.5 to 63.7)	2.09 (1.37 to 3.15)	7.72 (4.71 to 12.1)	268.4 (191.4 to 357.9)
		Pseudomonas aeruginosa	2240 (1950 to 2650)	4440 (3960 to 5060)	98.4 (72.5 to 124.1)	16.3 (14.2 to 18.6)	84.3 (69.4 to 99)	417.6 (318.8 to 526.7)
		Respiratory syncytial virus	611 (522 to 725)	186 (74.2 to 359)	-69.5 (-87.6 to -43.1)	2.19 (1.88 to 2.53)	1.69 (0.62 to 3.44)	-22.7 (-72.8 to 54.7)
		Staphylococcus aureus	4090 (3560 to 4780)	9020 (8060 to 10300)	121.1 (93.2 to 149.3)	61 (53 to 68.6)	351 (288 to 412)	476.6 (365.8 to 597.1)
		Streptococcus pneumoniae	16000 (14000 to 18600)	12700 (11400 to 14400)	-20.3 (-30.2 to -9.9)	99.1 (86.6 to 111)	205 (168 to 241)	107.8 (68.2 to 150.4)
		Other viruses	5530 (4810 to 6460)	8610 (7730 to 9810)	56.2 (36.8 to 77.4)	13.5 (11.7 to 15.2)	54.8 (45 to 64.5)	307.4 (229.0 to 394.0)
		Other bacterial pathogens	5740 (4890 to 6800)	6430 (5580 to 7490)	12.3 (-1.7 to 27.2)	34.3 (29.1 to 39.2)	100 (82.5 to 120)	193.4 (136.8 to 253.6)
Kyrgyzstan	All Ages	*Acinetobacter baumannii*	1990 (1740 to 2300)	1270 (1110 to 1460)	-36.0 (-42.1 to -29.2)	92 (59.8 to 139)	14.5 (11.8 to 17.9)	-83.7 (-88.5 to -77.3)
		Chlamydia spp.	8100 (7350 to 9000)	4360 (3940 to 4800)	-46.1 (-51.8 to -40.5)	98.1 (86.8 to 111)	14.3 (12.5 to 16.2)	-85.3 (-87.8 to -82.8)
		Enterobacter spp.	680 (576 to 799)	742 (643 to 848)	9.4 (-3.2 to 21.8)	35.3 (23.5 to 52.1)	9 (7.56 to 10.6)	-73.6 (-81.8 to -63.8)
		Escherichia coli	2360 (2110 to 2650)	2160 (1930 to 2370)	-8.2 (-17.7 to 1.6)	119 (102 to 141)	28.7 (24.8 to 32.6)	-75.8 (-80.2 to -71.1)
		Fungus	1440 (1250 to 1670)	1520 (1330 to 1710)	5.1 (-5.6 to 15.8)	19.2 (15.8 to 22.8)	13.6 (11.3 to 16.4)	-28.9 (-41.2 to -13.7)
		Group B Streptococcus	2070 (1850 to 2320)	1900 (1690 to 2120)	-8.1 (-17.8 to 2.5)	103 (88.6 to 121)	18.4 (16.2 to 20.7)	-82.2 (-85.4 to -78.6)
		Haemophilus influenzae	10800 (9720 to 12000)	5920 (5310 to 6600)	-45.0 (-51.4 to -38.8)	191 (163 to 224)	20.9 (18.1 to 23.7)	-89.0 (-91.0 to -86.8)
		Influenza	12100 (11200 to 13200)	2650 (808 to 5670)	-78.0 (-93.1 to -52.8)	310 (276 to 346)	23.5 (7.08 to 53.4)	-92.4 (-97.7 to -82.5)

		Klebsiella pneumoniae	6810 (6280 to 7480)	4280 (3860 to 4680)	-37.1 (-43.4 to -30.7)	271 (239 to 304)	53.9 (47.5 to 60.7)	-80.1 (-83.2 to -76.6)
		Legionella spp.	2270 (2010 to 2560)	2660 (2370 to 2920)	17.4 (5.5 to 29.4)	51.4 (39 to 67.7)	18 (15.2 to 21.4)	-64.3 (-73.7 to -53.6)
		Mycoplasma spp.	11400 (10400 to 12600)	8810 (7910 to 9790)	-22.9 (-30.7 to -13.9)	90.2 (78.6 to 105)	28.2 (25 to 31.5)	-68.6 (-73.9 to -62.9)
		Polymicrobial	416 (301 to 574)	300 (204 to 438)	-28.0 (-41.4 to -15.5)	62.9 (35.2 to 102)	6.22 (4.13 to 9.01)	-89.8 (-92.5 to -86.2)
		Pseudomonas aeruginosa	4090 (3750 to 4490)	4750 (4310 to 5190)	16.2 (4.8 to 27.6)	128 (113 to 145)	42.4 (37.2 to 48)	-66.7 (-72.1 to -60.4)
		Respiratory syncytial virus	11900 (10800 to 13400)	2010 (632 to 4200)	-83.1 (-94.6 to -64.5)	311 (276 to 349)	16.5 (4.89 to 37.7)	-94.7 (-98.4 to -87.8)
		Staphylococcus aureus	6400 (5970 to 6910)	7630 (6940 to 8290)	19.3 (8.8 to 29.9)	256 (230 to 285)	119 (103 to 135)	-53.5 (-60.7 to -45.6)
		Streptococcus pneumoniae	68100 (63600 to 74300)	26300 (23800 to 28800)	-61.4 (-65.3 to -57.1)	1160 (1040 to 1300)	176 (155 to 198)	-84.8 (-87.1 to -82.3)
		Other viruses	33400 (30700 to 36800)	18700 (16800 to 20700)	-44.0 (-50.3 to -37.5)	297 (265 to 332)	61.2 (53.7 to 68.8)	-79.3 (-82.5 to -75.7)
		Other bacterial pathogens	6740 (6120 to 7480)	5300 (4670 to 5960)	-21.4 (-28.5 to -14.2)	86.6 (70.8 to 108)	29.8 (24.7 to 35.6)	-65.3 (-73.2 to -57.0)
	Under 5	*Acinetobacter baumannii*	313 (226 to 434)	68.8 (48.8 to 97.9)	-77.9 (-81.9 to -73.7)	74.9 (42.6 to 121)	4.12 (2.57 to 6.43)	-94.4 (-95.6 to -93.0)
		Chlamydia spp.	3170 (2760 to 3650)	830 (676 to 1000)	-73.8 (-78.6 to -68.7)	86.1 (75 to 99.1)	6.99 (5.91 to 8.18)	-91.9 (-93.4 to -90.3)
		Enterobacter spp.	260 (201 to 327)	126 (96.1 to 165)	-51.2 (-60.4 to -41.5)	31.3 (19.5 to 48.5)	3.97 (2.99 to 5.26)	-86.9 (-90.6 to -81.9)
		Escherichia coli	980 (835 to 1150)	477 (384 to 585)	-51.1 (-60.5 to -41.0)	105 (87.7 to 126)	14.3 (11.9 to 17.1)	-86.3 (-89.1 to -83.0)
		Fungus	181 (127 to 252)	76.1 (53.8 to 105)	-57.6 (-66.5 to -48.3)	7.4 (5.1 to 10.3)	1.78 (1.23 to 2.47)	-75.8 (-81.7 to -68.5)
		Group B Streptococcus	855 (722 to 1010)	366 (294 to 449)	-57.1 (-64.5 to -48.8)	95.2 (80.2 to 113)	9.38 (7.83 to 11.1)	-90.1 (-92.0 to -87.9)
		Haemophilus influenzae	6490 (5640 to 7540)	1930 (1590 to 2330)	-70.2 (-75.6 to -64.5)	175 (148 to 208)	12.4 (10.2 to 14.7)	-92.9 (-94.5 to -91.1)
		Influenza	5540 (4930 to 6330)	508 (160 to 1070)	-90.8 (-97.0 to -80.8)	263 (230 to 300)	10.1 (3.01 to 23.1)	-96.1 (-98.8 to -91.2)
		Klebsiella pneumoniae	3530 (3110 to 4090)	942 (776 to 1120)	-73.3 (-78.0 to -68.4)	239 (209 to 272)	26.2 (21.6 to 30.4)	-89.0 (-91.3 to -86.6)
		Legionella spp.	290 (212 to 391)	121 (85.3 to 170)	-58.3 (-66.1 to -49.9)	38.4 (26.3 to 54.5)	3.58 (2.66 to 4.67)	-90.5 (-92.8 to -87.6)
		Mycoplasma spp.	3820 (3330 to 4400)	2130 (1760 to 2550)	-43.9 (-53.5 to -32.8)	72.5 (61.4 to 85.9)	14.5 (11.9 to 17.3)	-79.8 (-84.2 to -74.9)
		Polymicrobial	182 (112 to 277)	53.8 (32.7 to 80.6)	-70.3 (-75.7 to -64.7)	60.5 (33 to 99.3)	4.12 (2.35 to 6.44)	-93.1 (-94.6 to -91.4)
		Pseudomonas aeruginosa	1630 (1430 to 1890)	1100 (908 to 1320)	-32.6 (-44.7 to -19.9)	111 (96.6 to 127)	21.3 (17.8 to 24.8)	-80.7 (-84.5 to -76.7)
		Respiratory syncytial virus	10800 (9640 to 12200)	1610 (510 to 3370)	-85.0 (-95.2 to -68.6)	308 (272 to 345)	15.4 (4.55 to 35.4)	-95.0 (-98.5 to -88.5)

		Staphylococcus aureus	2180 (1930 to 2480)	1150 (959 to 1380)	-47.0 (-56.0 to -37.3)	196 (172 to 224)	42.1 (34.8 to 49.9)	-78.5 (-82.8 to -73.6)
		Streptococcus pneumoniae	36700 (32700 to 41700)	5540 (4590 to 6630)	-84.9 (-87.4 to -82.2)	990 (873 to 1130)	79 (65.2 to 93.8)	-92.0 (-93.7 to -90.0)
		Other viruses	22300 (20000 to 25400)	6590 (5500 to 7870)	-70.5 (-75.4 to -65.2)	273 (243 to 309)	39.2 (32.6 to 46.2)	-85.6 (-88.6 to -82.4)
		Other bacterial pathogens	1100 (884 to 1350)	369 (282 to 461)	-66.3 (-72.8 to -59.2)	54.5 (39.9 to 75.7)	6.33 (4.85 to 8.07)	-88.2 (-91.1 to -84.8)
	5-14 years	*Acinetobacter baumannii*	430 (350 to 530)	210 (160 to 266)	-51.1 (-60.4 to -40.2)	2.86 (2.36 to 3.39)	0.9 (0.73 to 1.07)	-68.2 (-74.1 to -61.3)
		Chlamydia spp.	1570 (1290 to 1920)	798 (611 to 1000)	-48.9 (-58.5 to -37.8)	2.58 (2.2 to 2.98)	0.85 (0.7 to 1.01)	-66.8 (-72.8 to -59.8)
		Enterobacter spp.	110 (87.9 to 139)	111 (84.3 to 144)	1.2 (-18.8 to 24.3)	0.69 (0.56 to 0.83)	0.45 (0.36 to 0.54)	-34.2 (-46.3 to -20.5)
		Escherichia coli	322 (264 to 393)	270 (210 to 336)	-15.9 (-32.4 to 2.5)	2.09 (1.78 to 2.44)	1.14 (0.94 to 1.33)	-45.3 (-55.3 to -34.5)
		Fungus	389 (313 to 476)	309 (237 to 391)	-20.2 (-35.2 to -2.5)	2.5 (2.1 to 2.94)	1.29 (1.06 to 1.54)	-48.2 (-57.7 to -37.8)
		Group B Streptococcus	381 (308 to 469)	340 (260 to 429)	-10.6 (-27.5 to 8.9)	1.76 (1.49 to 2.08)	1.02 (0.84 to 1.21)	-41.9 (-52.2 to -29.4)
		Haemophilus influenzae	1440 (1190 to 1730)	933 (718 to 1170)	-35.1 (-46.8 to -20.6)	3.57 (3.1 to 4.07)	1.02 (0.86 to 1.19)	-71.2 (-76.3 to -65.5)
		Influenza	2100 (1780 to 2500)	487 (140 to 1080)	-76.8 (-92.9 to -48.2)	10.2 (9.05 to 11.5)	1.58 (0.46 to 3.6)	-84.5 (-95.5 to -65.2)
		Klebsiella pneumoniae	949 (798 to 1140)	651 (506 to 811)	-31.3 (-43.4 to -16.8)	6.14 (5.36 to 6.9)	2.73 (2.29 to 3.17)	-55.3 (-63.4 to -46.2)
		Legionella spp.	596 (492 to 725)	534 (409 to 671)	-10.2 (-27.3 to 9.4)	2.66 (2.24 to 3.09)	1.54 (1.27 to 1.81)	-41.6 (-52.3 to -28.9)
		Mycoplasma spp.	3280 (2740 to 3910)	2150 (1660 to 2670)	-34.3 (-46.1 to -20.1)	5.63 (4.94 to 6.42)	2.4 (2.03 to 2.77)	-57.3 (-64.9 to -48.7)
		Polymicrobial	64.1 (42.3 to 94.5)	46.3 (28.3 to 70.2)	-27.7 (-41.9 to -10.8)	0.42 (0.28 to 0.62)	0.2 (0.13 to 0.3)	-53.0 (-62.3 to -43.0)
		Pseudomonas aeruginosa	688 (571 to 818)	712 (553 to 886)	3.8 (-15.3 to 26.8)	3.09 (2.71 to 3.52)	2.08 (1.74 to 2.41)	-32.5 (-44.5 to -19.0)
		Respiratory syncytial virus	448 (370 to 541)	113 (33 to 252)	-74.7 (-92.4 to -43.5)	0.99 (0.86 to 1.13)	0.17 (0.05 to 0.38)	-83.1 (-95.1 to -62.1)
		Staphylococcus aureus	1200 (1020 to 1430)	1270 (994 to 1580)	6.4 (-12.7 to 29.1)	11 (9.67 to 12.3)	7.59 (6.42 to 8.76)	-30.8 (-43.1 to -17.2)
		Streptococcus pneumoniae	12300 (10400 to 14700)	5830 (4560 to 7250)	-52.4 (-61.0 to -42.3)	47 (41.6 to 52.3)	14.5 (12.3 to 16.6)	-69.1 (-74.6 to -63.0)
		Other viruses	4490 (3800 to 5370)	3520 (2750 to 4370)	-21.3 (-35.4 to -4.5)	6.75 (5.98 to 7.59)	3.44 (2.92 to 3.97)	-48.8 (-57.9 to -38.8)
		Other bacterial pathogens	1440 (1180 to 1730)	861 (665 to 1090)	-39.9 (-50.9 to -27.0)	5.23 (4.52 to 6.01)	2.04 (1.69 to 2.4)	-60.9 (-67.8 to -53.2)
	15-49 years	*Acinetobacter baumannii*	340 (289 to 405)	269 (226 to 318)	-20.8 (-30.3 to -10.4)	3.49 (2.99 to 4.07)	2.59 (2.06 to 3.27)	-25.8 (-41.0 to -6.7)
		Chlamydia spp.	1240 (1070 to 1420)	1020 (879 to 1180)	-17.3 (-26.6 to -7.2)	3.16 (2.76 to 3.61)	2.44 (1.93 to 3.05)	-22.5 (-38.4 to -2.3)

		Enterobacter spp.	86.9 (71.9 to 105)	142 (119 to 167)	63.7 (42.5 to 85.6)	0.85 (0.7 to 1.01)	1.29 (1 to 1.61)	53.5 (20.4 to 89.9)
		Escherichia coli	254 (218 to 294)	345 (298 to 393)	36.1 (19.3 to 52.1)	2.56 (2.24 to 2.91)	3.26 (2.61 to 4.02)	27.6 (0.9 to 59.9)
		Fungus	307 (259 to 363)	396 (338 to 463)	29.0 (12.4 to 45.3)	3.05 (2.6 to 3.55)	3.68 (2.91 to 4.58)	21.0 (-3.4 to 50.6)
		Group B Streptococcus	301 (257 to 353)	434 (374 to 506)	44.7 (26.4 to 63.5)	2.15 (1.84 to 2.48)	2.91 (2.36 to 3.62)	35.7 (7.2 to 69.4)
		Haemophilus influenzae	1140 (996 to 1290)	1190 (1040 to 1360)	5.0 (-6.9 to 16.3)	4.37 (3.9 to 4.9)	2.93 (2.38 to 3.6)	-32.8 (-46.7 to -16.8)
		Influenza	1660 (1480 to 1860)	623 (192 to 1340)	-62.5 (-88.5 to -21.2)	12.5 (11.3 to 13.8)	4.5 (1.3 to 10.3)	-63.9 (-89.7 to -17.7)
		Klebsiella pneumoniae	750 (671 to 842)	832 (731 to 943)	11.2 (-1.6 to 23.6)	7.51 (6.79 to 8.29)	7.81 (6.34 to 9.47)	4.2 (-16.9 to 29.3)
		Legionella spp.	471 (405 to 546)	682 (588 to 778)	45.3 (27.3 to 63.8)	3.25 (2.82 to 3.74)	4.41 (3.54 to 5.39)	36.2 (7.1 to 68.7)
		Mycoplasma spp.	2590 (2300 to 2890)	2750 (2400 to 3110)	6.4 (-6.3 to 18.2)	6.9 (6.19 to 7.66)	6.86 (5.58 to 8.34)	-0.3 (-20.8 to 24.3)
		Polymicrobial	50.6 (33.5 to 73.9)	59.3 (37.9 to 90.3)	17.0 (1.0 to 33.4)	0.52 (0.35 to 0.74)	0.57 (0.35 to 0.89)	9.7 (-13.4 to 37.4)
		Pseudomonas aeruginosa	543 (481 to 611)	911 (796 to 1030)	68.0 (48.7 to 87.2)	3.78 (3.39 to 4.21)	5.94 (4.85 to 7.21)	57.5 (24.3 to 97.6)
		Respiratory syncytial virus	354 (311 to 400)	145 (43.2 to 311)	-59.1 (-87.7 to -12.7)	1.22 (1.08 to 1.37)	0.48 (0.14 to 1.1)	-60.6 (-88.8 to -10.4)
		Staphylococcus aureus	945 (844 to 1050)	1630 (1430 to 1830)	72.2 (52.7 to 90.4)	13.5 (12.2 to 14.8)	21.7 (17.6 to 26.4)	61.4 (28.6 to 99.3)
		Streptococcus pneumoniae	9700 (8740 to 10700)	7450 (6550 to 8370)	-23.1 (-31.8 to -14.8)	57.5 (52.5 to 62.7)	41.4 (33.7 to 50.1)	-27.9 (-42.8 to -10.6)
		Other viruses	3540 (3160 to 3930)	4510 (3960 to 5060)	27.4 (13.1 to 41.6)	8.26 (7.56 to 9.04)	9.84 (8.02 to 11.9)	19.4 (-5.0 to 48.4)
		Other bacterial pathogens	1130 (986 to 1290)	1100 (942 to 1270)	-2.7 (-14.1 to 8.3)	6.4 (5.65 to 7.19)	5.83 (4.63 to 7.26)	-8.7 (-27.6 to 14.4)
	50-69 years	*Acinetobacter baumannii*	518 (435 to 614)	421 (351 to 497)	-18.7 (-27.9 to -8.6)	5.35 (4.51 to 6.38)	4.11 (3.24 to 5.27)	-22.8 (-40.2 to -0.3)
		Chlamydia spp.	1110 (974 to 1270)	917 (800 to 1040)	-17.6 (-26.9 to -7.0)	2.86 (2.43 to 3.3)	2.23 (1.74 to 2.8)	-21.8 (-39.9 to 0.3)
		Enterobacter spp.	129 (106 to 154)	213 (181 to 251)	66.0 (45.8 to 87.3)	1.26 (1.03 to 1.53)	1.97 (1.5 to 2.52)	57.6 (20.8 to 101.2)
		Escherichia coli	415 (362 to 477)	565 (492 to 645)	36.4 (20.9 to 53.2)	4.2 (3.59 to 4.85)	5.42 (4.23 to 6.79)	29.5 (-0.9 to 66.0)
		Fungus	399 (338 to 468)	523 (447 to 604)	31.3 (16.4 to 47.5)	3.99 (3.35 to 4.76)	4.95 (3.83 to 6.22)	24.6 (-4.5 to 60.2)
		Group B Streptococcus	315 (269 to 369)	456 (393 to 525)	45.2 (28.7 to 62.9)	2.26 (1.92 to 2.66)	3.11 (2.46 to 3.91)	37.9 (5.0 to 79.1)
		Haemophilus influenzae	1020 (895 to 1150)	1130 (991 to 1270)	11.0 (-0.6 to 23.7)	3.93 (3.43 to 4.55)	2.81 (2.21 to 3.52)	-28.1 (-45.1 to -8.4)
		Influenza	1280 (1140 to 1430)	490 (151 to 1060)	-61.6 (-88.3 to -18.5)	9.67 (8.44 to 10.9)	3.6 (1.04 to 8.32)	-62.6 (-89.3 to -15.0)

		Klebsiella pneumoniae	858 (765 to 955)	1030 (904 to 1160)	20.0 (7.8 to 33.2)	8.64 (7.56 to 9.76)	9.81 (7.71 to 12.3)	14.0 (-11.7 to 45.2)
		Legionella spp.	591 (512 to 680)	870 (759 to 984)	47.4 (30.0 to 64.9)	4.1 (3.5 to 4.81)	5.71 (4.5 to 7.15)	40.0 (6.7 to 79.3)
		Mycoplasma spp.	1060 (942 to 1190)	1100 (960 to 1250)	3.5 (-7.3 to 15.7)	2.84 (2.47 to 3.25)	2.78 (2.2 to 3.52)	-1.8 (-24.4 to 25.8)
		Polymicrobial	64.2 (43.1 to 95.1)	76.8 (49.8 to 117)	19.4 (4.6 to 35.6)	0.66 (0.44 to 0.96)	0.75 (0.45 to 1.2)	13.4 (-13.1 to 44.9)
		Pseudomonas aeruginosa	688 (609 to 772)	1160 (1010 to 1310)	68.3 (50.0 to 87.9)	4.81 (4.21 to 5.53)	7.66 (6.07 to 9.68)	59.8 (22.6 to 105.1)
		Respiratory syncytial virus	202 (175 to 230)	82.4 (25.5 to 178)	-59.0 (-87.5 to -12.2)	0.7 (0.6 to 0.81)	0.28 (0.08 to 0.62)	-60.1 (-88.7 to -10.0)
		Staphylococcus aureus	1050 (943 to 1180)	1850 (1620 to 2070)	75.5 (57.9 to 95.2)	15.1 (13.3 to 17.2)	25 (19.8 to 31.3)	66.7 (28.7 to 112.3)
		Streptococcus pneumoniae	5330 (4810 to 5930)	4270 (3760 to 4770)	-19.9 (-27.9 to -10.9)	31.8 (28 to 35.9)	24.1 (19 to 30.1)	-24.0 (-41.7 to -3.0)
		Other viruses	1710 (1530 to 1900)	2330 (2060 to 2610)	36.8 (23.2 to 52.3)	4 (3.54 to 4.55)	5.17 (4.13 to 6.48)	29.9 (-0.2 to 66.0)
		Other bacterial pathogens	1530 (1330 to 1750)	1510 (1300 to 1740)	-1.4 (-11.7 to 10.5)	8.68 (7.5 to 10)	8.11 (6.31 to 10.4)	-6.2 (-27.6 to 19.4)
	70+ years	*Acinetobacter baumannii*	392 (328 to 462)	305 (256 to 361)	-22.0 (-31.2 to -12.1)	5.38 (4.5 to 6.31)	2.8 (2.22 to 3.5)	-47.6 (-58.0 to -34.1)
		Chlamydia spp.	1000 (859 to 1160)	792 (688 to 908)	-21.0 (-30.4 to -11.3)	3.43 (2.93 to 3.94)	1.81 (1.44 to 2.23)	-47.0 (-57.6 to -33.2)
		Enterobacter spp.	94.8 (76.5 to 116)	150 (126 to 177)	59.3 (39.7 to 80.9)	1.23 (1 to 1.5)	1.31 (1.02 to 1.63)	6.9 (-14.8 to 33.7)
		Escherichia coli	385 (329 to 447)	503 (434 to 575)	30.9 (14.8 to 46.8)	5.19 (4.41 to 6.03)	4.54 (3.63 to 5.64)	-12.2 (-30.4 to 10.1)
		Fungus	168 (140 to 199)	211 (178 to 249)	26.0 (10.2 to 43.0)	2.24 (1.85 to 2.67)	1.88 (1.5 to 2.35)	-15.5 (-31.9 to 4.8)
		Group B Streptococcus	218 (185 to 253)	304 (261 to 350)	39.4 (22.6 to 57.6)	2.09 (1.75 to 2.43)	1.95 (1.56 to 2.41)	-6.5 (-25.9 to 17.0)
		Haemophilus influenzae	692 (601 to 784)	736 (642 to 838)	6.5 (-5.9 to 19.1)	3.56 (3.07 to 4.04)	1.73 (1.41 to 2.13)	-51.2 (-61.5 to -39.3)
		Influenza	1470 (1280 to 1640)	539 (164 to 1150)	-63.3 (-88.6 to -20.1)	14.8 (12.8 to 16.6)	3.73 (1.08 to 8.54)	-74.7 (-92.7 to -42.4)
		Klebsiella pneumoniae	715 (624 to 802)	822 (719 to 933)	15.2 (2.2 to 29.0)	9.57 (8.33 to 10.8)	7.38 (6.01 to 9.02)	-22.7 (-38.2 to -4.3)
		Legionella spp.	322 (273 to 373)	454 (390 to 517)	41.5 (24.3 to 59.9)	2.97 (2.5 to 3.48)	2.8 (2.25 to 3.46)	-5.1 (-24.2 to 18.6)
		Mycoplasma spp.	678 (594 to 768)	672 (587 to 768)	-0.7 (-12.3 to 11.3)	2.41 (2.09 to 2.74)	1.6 (1.29 to 1.95)	-33.4 (-46.6 to -16.8)
		Polymicrobial	55.6 (36.5 to 83.9)	63.8 (40.8 to 99.5)	14.5 (-0.6 to 29.9)	0.76 (0.51 to 1.13)	0.58 (0.35 to 0.91)	-23.1 (-39.3 to -2.2)
		Pseudomonas aeruginosa	541 (471 to 611)	873 (760 to 991)	61.5 (43.9 to 81.8)	5.03 (4.33 to 5.71)	5.44 (4.41 to 6.66)	8.4 (-14.3 to 36.6)
		Respiratory syncytial virus	143 (123 to 164)	56 (17.1 to 118)	-60.8 (-88.1 to -14.6)	0.66 (0.57 to 0.75)	0.18 (0.05 to 0.4)	-73.0 (-92.3 to -37.5)

		Staphylococcus aureus	1030 (909 to 1150)	1730 (1520 to 1970)	68.4 (50.1 to 87.0)	19.6 (17.1 to 22)	22.1 (17.9 to 27.1)	13.0 (-9.5 to 41.2)
		Streptococcus pneumoniae	4150 (3670 to 4590)	3190 (2790 to 3610)	-23.2 (-31.6 to -14.2)	32.9 (28.8 to 36.6)	16.9 (13.7 to 20.8)	-48.4 (-58.7 to -35.4)
		Other viruses	1310 (1160 to 1460)	1720 (1510 to 1950)	31.3 (17.0 to 46.7)	4.09 (3.56 to 4.59)	3.59 (2.92 to 4.4)	-11.9 (-29.3 to 10.2)
		Other bacterial pathogens	1550 (1340 to 1750)	1460 (1240 to 1690)	-5.4 (-16.3 to 6.0)	11.8 (10 to 13.5)	7.49 (5.88 to 9.35)	-36.4 (-49.4 to -20.1)
Mongolia	All Ages	*Acinetobacter baumannii*	1430 (1220 to 1660)	898 (785 to 1020)	-37.1 (-43.6 to -30.2)	51.5 (38.1 to 72.8)	14.3 (11.3 to 18.5)	-71.7 (-78.9 to -62.9)
		Chlamydia spp.	4950 (4420 to 5590)	2080 (1910 to 2280)	-57.8 (-61.6 to -53.2)	72.8 (58.9 to 87.3)	10.6 (8.37 to 13.4)	-85.3 (-89.0 to -80.6)
		Enterobacter spp.	311 (253 to 378)	315 (272 to 367)	1.5 (-10.6 to 14.0)	18.2 (13.3 to 25.2)	5.69 (4.35 to 7.61)	-68.3 (-76.4 to -57.9)
		Escherichia coli	1230 (1070 to 1410)	905 (821 to 1000)	-26.0 (-33.6 to -18.2)	75.8 (60.7 to 94.1)	17.8 (14.1 to 22.2)	-76.3 (-82.5 to -69.2)
		Fungus	745 (623 to 877)	707 (619 to 808)	-4.7 (-15.3 to 6.4)	16.8 (12.7 to 21.8)	8.32 (6.67 to 10.1)	-49.8 (-61.1 to -36.5)
		Group B Streptococcus	1010 (875 to 1160)	841 (758 to 931)	-16.1 (-24.6 to -7.3)	50.9 (39.8 to 63.6)	12.8 (10 to 16.1)	-74.5 (-81.6 to -65.6)
		Haemophilus influenzae	5230 (4580 to 5980)	2100 (1920 to 2330)	-59.7 (-63.8 to -55.5)	147 (121 to 176)	10 (8.01 to 12.5)	-93.1 (-94.8 to -91.0)
		Influenza	5940 (5330 to 6550)	125 (7.98 to 510)	-97.9 (-99.9 to -91.4)	257 (217 to 299)	1.56 (0.1 to 6.45)	-99.4 (-100.0 to -97.4)
		Klebsiella pneumoniae	3710 (3330 to 4130)	2020 (1870 to 2190)	-45.3 (-50.1 to -39.8)	238 (199 to 281)	37.1 (29.8 to 45.5)	-84.3 (-87.9 to -79.8)
		Legionella spp.	855 (734 to 984)	991 (900 to 1100)	16.3 (4.3 to 29.8)	19.4 (14.1 to 26.3)	9.84 (8.01 to 12.1)	-48.2 (-62.0 to -31.6)
		Mycoplasma spp.	5580 (5010 to 6180)	3470 (3160 to 3800)	-37.7 (-43.1 to -31.9)	68.6 (56.4 to 82.2)	15.2 (12.3 to 18.6)	-77.7 (-82.9 to -71.3)
		Polymicrobial	214 (160 to 290)	149 (107 to 210)	-30.4 (-39.5 to -20.6)	20.5 (11.9 to 33.6)	5.3 (3.26 to 8.62)	-73.5 (-82.7 to -60.5)
		Pseudomonas aeruginosa	1680 (1510 to 1860)	1900 (1750 to 2070)	13.2 (2.6 to 23.9)	68.9 (56.6 to 82.3)	24.1 (19.5 to 29.2)	-64.8 (-73.5 to -55.0)
		Respiratory syncytial virus	4900 (4300 to 5560)	82.9 (5.27 to 332)	-98.3 (-99.9 to -93.4)	181 (148 to 214)	1.04 (0.07 to 4.41)	-99.4 (-100.0 to -97.5)
		Staphylococcus aureus	2940 (2680 to 3220)	3170 (2940 to 3410)	7.9 (0.2 to 16.5)	192 (161 to 222)	68.5 (56.5 to 81.6)	-64.2 (-71.5 to -55.4)
		Streptococcus pneumoniae	35400 (32100 to 38700)	12500 (11600 to 13500)	-64.6 (-67.3 to -61.6)	1250 (1070 to 1440)	124 (101 to 153)	-90.0 (-92.3 to -87.4)
		Other viruses	14100 (12600 to 15500)	7180 (6620 to 7800)	-48.9 (-53.1 to -44.3)	240 (202 to 278)	34.8 (27.5 to 44.4)	-85.4 (-89.1 to -80.8)
		Other bacterial pathogens	3550 (3180 to 3930)	2350 (2100 to 2590)	-33.9 (-39.6 to -27.6)	62.3 (51.2 to 74.5)	18.3 (15.1 to 22)	-70.5 (-76.6 to -63.3)
	Under 5	*Acinetobacter baumannii*	202 (149 to 274)	42.2 (30.1 to 59)	-79.1 (-82.2 to -75.7)	37.4 (24.1 to 57.5)	4.57 (2.49 to 7.92)	-87.7 (-92.6 to -80.5)
		Chlamydia spp.	2040 (1750 to 2410)	384 (330 to 447)	-81.1 (-83.6 to -78.3)	65 (51.6 to 79.3)	5.92 (4.01 to 8.29)	-90.8 (-94.0 to -86.7)

		Enterobacter spp.	125 (94.4 to 164)	49.4 (37.9 to 63.1)	-60.2 (-66.2 to -53.9)	16.2 (11.6 to 23.1)	2.83 (1.69 to 4.56)	-82.4 (-88.6 to -73.9)
		Escherichia coli	541 (447 to 651)	176 (149 to 206)	-67.4 (-71.8 to -62.5)	67.9 (53.5 to 85.5)	9.56 (6.45 to 13.4)	-85.8 (-90.6 to -79.4)
		Fungus	107 (73.5 to 149)	31.2 (22.2 to 42.6)	-70.8 (-75.4 to -66.1)	10.2 (6.74 to 14.5)	1.07 (0.68 to 1.6)	-89.3 (-92.8 to -85.0)
		Group B Streptococcus	427 (351 to 516)	152 (127 to 182)	-64.3 (-69.2 to -59.4)	46.6 (35.5 to 59)	7.47 (5.08 to 10.7)	-83.7 (-89.4 to -76.1)
		Haemophilus influenzae	3310 (2810 to 3900)	615 (529 to 716)	-81.4 (-84.0 to -78.5)	139 (114 to 168)	5.9 (4.17 to 8.09)	-95.7 (-97.1 to -93.9)
		Influenza	2930 (2540 to 3360)	25 (1.59 to 98.9)	-99.1 (-99.9 to -96.6)	234 (196 to 274)	0.75 (0.05 to 3.19)	-99.7 (-100.0 to -98.6)
		Klebsiella pneumoniae	2050 (1750 to 2370)	423 (368 to 489)	-79.3 (-81.8 to -76.5)	220 (181 to 263)	19.4 (13.4 to 27.3)	-91.1 (-93.9 to -87.5)
		Legionella spp.	113 (79.1 to 156)	43.1 (31.3 to 59.1)	-61.6 (-66.7 to -56.0)	14 (8.94 to 20.8)	2.74 (1.7 to 4.36)	-79.9 (-87.6 to -69.9)
		Mycoplasma spp.	2010 (1720 to 2330)	657 (566 to 763)	-67.3 (-71.5 to -62.7)	59.8 (48.3 to 73.4)	7.54 (5.33 to 10.4)	-87.3 (-91.3 to -81.9)
		Polymicrobial	76.3 (50.1 to 113)	25.5 (15.9 to 38.7)	-66.5 (-71.8 to -60.6)	18.9 (10.5 to 31.7)	3.91 (1.87 to 7.08)	-79.0 (-87.7 to -66.8)
		Pseudomonas aeruginosa	717 (613 to 830)	362 (315 to 420)	-49.4 (-55.8 to -41.9)	61.6 (49.6 to 74.8)	12.3 (8.53 to 17.2)	-79.8 (-86.5 to -71.1)
		Respiratory syncytial virus	4480 (3910 to 5120)	64.2 (4.01 to 257)	-98.6 (-99.9 to -94.4)	179 (147 to 212)	0.97 (0.06 to 4.14)	-99.5 (-100.0 to -97.6)
		Staphylococcus aureus	1090 (948 to 1260)	444 (385 to 518)	-59.3 (-63.7 to -54.2)	163 (134 to 191)	25.7 (18 to 34.9)	-84.2 (-89.2 to -77.9)
		Streptococcus pneumoniae	20200 (17700 to 23100)	2990 (2620 to 3450)	-85.2 (-86.8 to -83.5)	1160 (990 to 1360)	65.3 (46 to 88.3)	-94.3 (-96.1 to -92.1)
		Other viruses	9810 (8620 to 11200)	2500 (2190 to 2890)	-74.4 (-77.3 to -71.4)	230 (193 to 269)	23.5 (16.4 to 31.9)	-89.7 (-92.9 to -85.5)
		Other bacterial pathogens	646 (516 to 796)	143 (115 to 175)	-77.7 (-80.8 to -74.1)	43.8 (33.7 to 55.3)	4.37 (2.86 to 6.46)	-89.9 (-93.4 to -85.4)
	5-14 years	*Acinetobacter baumannii*	321 (246 to 408)	130 (101 to 165)	-59.4 (-65.6 to -53.0)	2.32 (1.74 to 3.01)	0.79 (0.6 to 1.02)	-65.2 (-75.9 to -50.9)
		Chlamydia spp.	930 (726 to 1170)	340 (267 to 419)	-63.3 (-68.6 to -57.7)	1.67 (1.26 to 2.17)	0.52 (0.39 to 0.67)	-68.5 (-77.9 to -55.3)
		Enterobacter spp.	49.7 (37.9 to 66)	41.6 (32.1 to 54.1)	-15.9 (-28.6 to -1.9)	0.34 (0.25 to 0.45)	0.24 (0.18 to 0.32)	-27.8 (-49.5 to 2.6)
		Escherichia coli	164 (128 to 207)	103 (81.4 to 127)	-37.0 (-46.7 to -25.9)	1.16 (0.87 to 1.49)	0.62 (0.46 to 0.8)	-45.9 (-62.2 to -23.3)
		Fungus	199 (152 to 256)	125 (96.4 to 159)	-36.9 (-47.0 to -26.4)	1.39 (1.03 to 1.78)	0.74 (0.56 to 0.97)	-45.9 (-62.3 to -23.8)
		Group B Streptococcus	183 (141 to 230)	134 (105 to 169)	-26.7 (-38.5 to -14.7)	0.92 (0.69 to 1.22)	0.57 (0.43 to 0.73)	-37.1 (-55.9 to -8.6)
		Haemophilus influenzae	648 (517 to 809)	303 (239 to 375)	-53.2 (-59.9 to -46.2)	1.75 (1.33 to 2.24)	0.47 (0.35 to 0.59)	-73.0 (-81.0 to -61.8)
		Influenza	974 (777 to 1200)	20.1 (1.27 to 81)	-97.9 (-99.9 to -91.7)	5.15 (3.92 to 6.47)	0.09 (0.01 to 0.37)	-98.2 (-99.9 to -92.2)

		Klebsiella pneumoniae	486 (388 to 603)	270 (216 to 335)	-44.2 (-52.0 to -36.1)	3.42 (2.62 to 4.35)	1.61 (1.24 to 2.05)	-52.1 (-66.5 to -32.2)
		Legionella spp.	227 (177 to 283)	173 (137 to 214)	-23.5 (-35.0 to -10.3)	1.1 (0.81 to 1.4)	0.71 (0.54 to 0.9)	-34.3 (-53.6 to -6.0)
		Mycoplasma spp.	1520 (1210 to 1870)	805 (645 to 999)	-46.8 (-54.2 to -38.4)	2.84 (2.14 to 3.58)	1.27 (0.98 to 1.62)	-54.3 (-67.9 to -35.7)
		Polymicrobial	38.2 (26.1 to 56)	20.4 (12.8 to 30.6)	-46.6 (-55.7 to -37.0)	0.27 (0.18 to 0.4)	0.12 (0.08 to 0.19)	-54.2 (-68.1 to -36.2)
		Pseudomonas aeruginosa	273 (217 to 338)	263 (209 to 321)	-3.5 (-17.6 to 11.9)	1.34 (1.01 to 1.69)	1.09 (0.83 to 1.38)	-17.2 (-42.0 to 16.2)
		Respiratory syncytial virus	166 (131 to 206)	4.72 (0.29 to 19.5)	-97.1 (-99.8 to -88.7)	0.4 (0.3 to 0.51)	0.01 (0 to 0.04)	-97.5 (-99.8 to -89.4)
		Staphylococcus aureus	529 (429 to 652)	471 (381 to 578)	-10.8 (-22.7 to 2.3)	5.3 (4.07 to 6.61)	3.99 (3.01 to 5.04)	-23.5 (-46.0 to 8.2)
		Streptococcus pneumoniae	5860 (4740 to 7170)	2350 (1900 to 2890)	-59.9 (-65.3 to -54.0)	24.4 (18.7 to 30.6)	8.28 (6.41 to 10.5)	-65.5 (-75.7 to -51.3)
		Other viruses	1710 (1380 to 2080)	1190 (969 to 1460)	-30.1 (-39.8 to -20.0)	2.8 (2.14 to 3.5)	1.66 (1.29 to 2.09)	-40.0 (-57.5 to -16.1)
		Other bacterial pathogens	751 (598 to 935)	339 (266 to 429)	-54.8 (-61.4 to -47.6)	2.97 (2.28 to 3.76)	1.14 (0.85 to 1.45)	-61.1 (-72.8 to -45.4)
	15-49 years	*Acinetobacter baumannii*	278 (232 to 328)	201 (169 to 238)	-27.6 (-36.3 to -17.6)	2.53 (1.96 to 3.2)	2.41 (1.85 to 3.04)	-4.2 (-24.5 to 20.0)
		Chlamydia spp.	805 (686 to 937)	525 (453 to 602)	-34.6 (-42.0 to -26.7)	1.82 (1.43 to 2.29)	1.57 (1.24 to 1.98)	-13.5 (-32.7 to 8.3)
		Enterobacter spp.	43.1 (34.2 to 52.8)	64.3 (53.4 to 76.4)	50.1 (30.7 to 71.4)	0.37 (0.28 to 0.48)	0.73 (0.56 to 0.94)	98.5 (54.1 to 150.5)
		Escherichia coli	142 (120 to 167)	159 (137 to 185)	12.4 (-1.4 to 26.7)	1.27 (0.99 to 1.6)	1.87 (1.46 to 2.34)	48.6 (17.4 to 86.8)
		Fungus	172 (142 to 208)	193 (163 to 227)	12.5 (-0.4 to 28.0)	1.52 (1.13 to 1.94)	2.24 (1.73 to 2.87)	48.9 (16.6 to 88.7)
		Group B Streptococcus	159 (133 to 190)	207 (179 to 240)	30.7 (14.2 to 47.0)	1.01 (0.77 to 1.28)	1.73 (1.35 to 2.18)	72.9 (34.3 to 117.4)
		Haemophilus influenzae	561 (484 to 643)	468 (414 to 530)	-16.5 (-25.6 to -6.6)	1.91 (1.51 to 2.36)	1.41 (1.11 to 1.74)	-25.7 (-41.0 to -8.0)
		Influenza	844 (738 to 949)	31.2 (2 to 125)	-96.3 (-99.8 to -84.9)	5.63 (4.46 to 6.88)	0.27 (0.02 to 1.14)	-95.1 (-99.7 to -79.3)
		Klebsiella pneumoniae	421 (365 to 473)	418 (372 to 474)	-0.5 (-10.6 to 10.6)	3.74 (2.98 to 4.65)	4.89 (3.91 to 6.06)	31.6 (3.2 to 64.9)
		Legionella spp.	196 (166 to 230)	267 (233 to 305)	36.5 (20.1 to 55.0)	1.2 (0.93 to 1.52)	2.15 (1.68 to 2.68)	80.5 (42.3 to 126.8)
		Mycoplasma spp.	1310 (1150 to 1490)	1240 (1110 to 1410)	-5.1 (-15.4 to 5.6)	3.1 (2.43 to 3.82)	3.87 (3.07 to 4.77)	25.6 (-1.1 to 57.0)
		Polymicrobial	33.1 (22.8 to 46.3)	31.6 (20.6 to 46.6)	-4.8 (-18.7 to 10.1)	0.3 (0.21 to 0.44)	0.38 (0.24 to 0.58)	25.9 (-0.6 to 58.8)
		Pseudomonas aeruginosa	237 (203 to 269)	407 (359 to 464)	72.1 (51.5 to 93.2)	1.46 (1.16 to 1.79)	3.3 (2.6 to 4.1)	127.6 (79.9 to 183.1)
		Respiratory syncytial virus	143 (123 to 163)	7.32 (0.47 to 29.5)	-94.9 (-99.7 to -79.3)	0.44 (0.35 to 0.54)	0.03 (0 to 0.12)	-93.3 (-99.6 to -72.0)

		Staphylococcus aureus	459 (401 to 516)	728 (649 to 819)	59.1 (43.5 to 76.5)	5.79 (4.63 to 7.03)	12.1 (9.66 to 15)	110.4 (66.1 to 161.9)
		Streptococcus pneumoniae	5080 (4500 to 5620)	3630 (3250 to 4050)	-28.4 (-35.1 to -20.8)	26.7 (21.5 to 32.4)	25.1 (20.2 to 30.9)	-5.3 (-24.6 to 18.1)
		Other viruses	1480 (1310 to 1650)	1850 (1650 to 2070)	24.7 (12.2 to 38.4)	3.07 (2.45 to 3.75)	5.03 (4.02 to 6.2)	64.9 (31.1 to 105.7)
		Other bacterial pathogens	650 (557 to 745)	524 (455 to 605)	-19.3 (-28.0 to -9.7)	3.25 (2.62 to 3.96)	3.45 (2.72 to 4.3)	6.9 (-15.3 to 33.0)
	50-69 years	*Acinetobacter baumannii*	343 (284 to 410)	334 (278 to 393)	-2.4 (-15.4 to 12.5)	4.73 (3.58 to 6.1)	4.01 (3.08 to 5.13)	-14.0 (-39.4 to 16.4)
		Chlamydia spp.	589 (502 to 683)	496 (430 to 569)	-15.5 (-26.0 to -3.2)	2.01 (1.54 to 2.57)	1.48 (1.14 to 1.88)	-25.5 (-46.6 to -0.7)
		Enterobacter spp.	51.7 (41 to 64.8)	102 (85.2 to 123)	98.8 (72.5 to 130.2)	0.68 (0.49 to 0.9)	1.16 (0.88 to 1.49)	75.2 (24.8 to 138.3)
		Escherichia coli	188 (159 to 221)	275 (236 to 319)	46.5 (26.7 to 67.3)	2.54 (1.92 to 3.25)	3.23 (2.51 to 4.11)	29.1 (-7.6 to 76.9)
		Fungus	182 (149 to 220)	271 (229 to 320)	49.8 (29.4 to 72.2)	2.42 (1.81 to 3.17)	3.14 (2.38 to 4.02)	32.0 (-6.1 to 78.2)
		Group B Streptococcus	135 (113 to 161)	229 (195 to 267)	70.7 (46.4 to 96.5)	1.29 (0.97 to 1.68)	1.91 (1.46 to 2.44)	50.4 (7.0 to 100.7)
		Haemophilus influenzae	407 (349 to 473)	475 (410 to 549)	17.1 (2.6 to 32.8)	2.09 (1.62 to 2.65)	1.43 (1.11 to 1.81)	-30.6 (-50.3 to -6.7)
		Influenza	527 (455 to 596)	26.1 (1.67 to 107)	-95.0 (-99.7 to -79.7)	5.31 (4.12 to 6.67)	0.23 (0.01 to 0.92)	-95.7 (-99.7 to -82.8)
		Klebsiella pneumoniae	391 (341 to 442)	560 (489 to 636)	43.7 (26.1 to 62.9)	5.24 (4.11 to 6.62)	6.55 (5.11 to 8.24)	26.6 (-9.8 to 68.1)
		Legionella spp.	200 (165 to 237)	360 (313 to 416)	81.0 (56.4 to 108.7)	1.85 (1.39 to 2.37)	2.9 (2.25 to 3.64)	59.5 (14.5 to 114.3)
		Mycoplasma spp.	436 (377 to 503)	517 (447 to 590)	18.8 (3.8 to 35.6)	1.56 (1.21 to 1.95)	1.61 (1.23 to 2.04)	4.6 (-25.3 to 38.7)
		Polymicrobial	34.1 (23.8 to 48.8)	43.4 (28.9 to 63.9)	27.3 (7.6 to 48.8)	0.47 (0.31 to 0.71)	0.52 (0.32 to 0.78)	12.1 (-22.0 to 52.9)
		Pseudomonas aeruginosa	243 (210 to 279)	544 (474 to 624)	124.3 (95.6 to 154.1)	2.27 (1.74 to 2.88)	4.42 (3.41 to 5.58)	97.6 (42.2 to 164.8)
		Respiratory syncytial virus	66.2 (56 to 76.2)	4.4 (0.27 to 18.1)	-93.3 (-99.6 to -73.2)	0.3 (0.23 to 0.39)	0.02 (0 to 0.07)	-94.2 (-99.7 to -76.6)
		Staphylococcus aureus	414 (359 to 466)	878 (770 to 992)	112.3 (87.7 to 139.4)	7.9 (6.14 to 9.9)	14.6 (11.4 to 18.2)	87.0 (33.8 to 150.6)
		Streptococcus pneumoniae	2260 (1990 to 2530)	2220 (1950 to 2510)	-1.7 (-12.8 to 11.1)	18 (14 to 22.4)	15.4 (12 to 19.3)	-13.4 (-37.5 to 15.6)
		Other viruses	579 (503 to 653)	1030 (909 to 1170)	78.9 (58.5 to 103.1)	1.81 (1.41 to 2.28)	2.81 (2.18 to 3.52)	57.6 (12.6 to 111.3)
		Other bacterial pathogens	711 (615 to 812)	759 (650 to 877)	6.9 (-6.7 to 21.8)	5.37 (4.21 to 6.8)	5 (3.85 to 6.31)	-5.6 (-32.5 to 27.5)
	70+ years	*Acinetobacter baumannii*	286 (234 to 346)	191 (161 to 225)	-32.9 (-41.5 to -22.9)	4.51 (3.32 to 5.93)	2.57 (1.91 to 3.3)	-42.1 (-58.6 to -19.9)
		Chlamydia spp.	584 (485 to 691)	338 (290 to 390)	-41.9 (-49.1 to -33.3)	2.29 (1.69 to 2.96)	1.13 (0.87 to 1.45)	-49.9 (-64.5 to -30.2)

		Enterobacter spp.	41.9 (32.2 to 53.3)	56.9 (46.3 to 68.5)	36.7 (18.8 to 60.2)	0.63 (0.44 to 0.87)	0.73 (0.53 to 0.93)	18.0 (-17.5 to 65.6)
		Escherichia coli	192 (158 to 229)	193 (164 to 222)	0.7 (-11.0 to 16.1)	2.98 (2.2 to 3.89)	2.54 (1.97 to 3.24)	-13.1 (-38.1 to 20.8)
		Fungus	84.3 (67.9 to 102)	86.4 (72.5 to 101)	3.0 (-10.3 to 19.4)	1.29 (0.93 to 1.67)	1.12 (0.85 to 1.45)	-11.2 (-36.9 to 22.4)
		Group B Streptococcus	103 (84.7 to 124)	120 (103 to 139)	17.4 (1.9 to 35.9)	1.13 (0.83 to 1.48)	1.13 (0.87 to 1.43)	1.2 (-27.5 to 40.8)
		Haemophilus influenzae	305 (255 to 361)	245 (212 to 280)	-19.5 (-28.8 to -8.0)	1.8 (1.33 to 2.31)	0.83 (0.64 to 1.06)	-53.3 (-66.5 to -34.5)
		Influenza	667 (565 to 776)	22.7 (1.44 to 92.8)	-96.6 (-99.8 to -86.0)	7.7 (5.83 to 9.8)	0.22 (0.01 to 0.93)	-97.1 (-99.8 to -88.1)
		Klebsiella pneumoniae	358 (302 to 418)	353 (306 to 399)	-1.2 (-11.9 to 12.6)	5.51 (4.17 to 7.06)	4.63 (3.59 to 5.87)	-14.8 (-38.9 to 16.6)
		Legionella spp.	120 (99 to 145)	148 (128 to 171)	24.4 (7.7 to 43.6)	1.27 (0.94 to 1.65)	1.34 (1.03 to 1.7)	7.3 (-24.6 to 49.0)
		Mycoplasma spp.	307 (257 to 361)	250 (217 to 283)	-18.4 (-27.9 to -6.9)	1.26 (0.93 to 1.62)	0.87 (0.67 to 1.1)	-29.5 (-49.8 to -2.4)
		Polymicrobial	32.5 (22.1 to 46.9)	28.4 (18.4 to 42.3)	-12.5 (-26.0 to 3.6)	0.51 (0.33 to 0.78)	0.38 (0.24 to 0.58)	-24.6 (-46.7 to 4.2)
		Pseudomonas aeruginosa	211 (179 to 248)	324 (281 to 368)	54.2 (36.3 to 76.4)	2.25 (1.67 to 2.86)	2.95 (2.27 to 3.7)	33.0 (-6.0 to 83.6)
		Respiratory syncytial virus	51.7 (43.2 to 61.8)	2.36 (0.15 to 9.37)	-95.4 (-99.7 to -81.5)	0.27 (0.2 to 0.35)	0.01 (0 to 0.04)	-96.1 (-99.8 to -84.0)
		Staphylococcus aureus	446 (378 to 516)	649 (565 to 730)	45.9 (30.9 to 65.9)	9.76 (7.34 to 12.3)	12.1 (9.46 to 15.2)	25.9 (-9.6 to 74.1)
		Streptococcus pneumoniae	1940 (1650 to 2230)	1310 (1140 to 1460)	-32.4 (-39.5 to -23.3)	17.7 (13.4 to 22.3)	10.1 (7.87 to 12.8)	-41.7 (-57.7 to -19.2)
		Other viruses	490 (419 to 569)	601 (524 to 674)	23.0 (9.8 to 39.4)	1.75 (1.32 to 2.23)	1.83 (1.42 to 2.3)	6.1 (-23.9 to 46.2)
		Other bacterial pathogens	792 (666 to 929)	580 (493 to 670)	-26.5 (-35.2 to -15.3)	6.94 (5.23 to 8.94)	4.34 (3.33 to 5.57)	-36.5 (-54.8 to -12.3)
Tajikistan	All Ages	*Acinetobacter baumannii*	3280 (2850 to 3770)	4620 (4040 to 5260)	40.8 (27.9 to 54.1)	165 (114 to 238)	114 (80.6 to 166)	-29.8 (-48.4 to -8.6)
		Chlamydia spp.	12800 (11500 to 14400)	11000 (10100 to 12000)	-14.1 (-21.2 to -6.0)	191 (167 to 218)	101 (74.7 to 135)	-46.9 (-62.0 to -29.7)
		Enterobacter spp.	954 (793 to 1120)	1400 (1200 to 1620)	47.1 (33.2 to 62.4)	59.5 (42.4 to 84.9)	44.8 (30.3 to 65)	-24.0 (-46.7 to 0.1)
		Escherichia coli	3460 (3040 to 3910)	4190 (3790 to 4620)	21.4 (10.8 to 32.4)	217 (183 to 260)	146 (107 to 192)	-32.7 (-51.6 to -11.0)
		Fungus	2080 (1770 to 2440)	3220 (2780 to 3690)	54.9 (41.2 to 71.6)	43.1 (34.6 to 53.1)	43 (32.9 to 54.1)	0.3 (-21.0 to 27.1)
		Group B Streptococcus	3040 (2680 to 3450)	3910 (3560 to 4340)	29.0 (17.4 to 40.4)	172 (147 to 204)	116 (82.1 to 154)	-32.4 (-51.6 to -9.1)
		Haemophilus influenzae	15700 (14100 to 17600)	10500 (9580 to 11500)	-33.0 (-38.9 to -27.0)	376 (318 to 442)	83 (59.3 to 108)	-77.8 (-84.3 to -70.1)
		Influenza	17400 (15900 to 19000)	2140 (1040 to 3660)	-87.7 (-94.1 to -78.9)	640 (559 to 724)	44.2 (19.6 to 80.5)	-93.1 (-96.9 to -87.1)

		Klebsiella pneumoniae	10300 (9310 to 11400)	9950 (9200 to 10700)	-3.0 (-11.0 to 5.2)	565 (493 to 646)	306 (226 to 393)	-45.6 (-61.1 to -28.3)
		Legionella spp.	2910 (2560 to 3300)	4370 (3970 to 4830)	50.7 (37.0 to 65.9)	80.4 (61.2 to 104)	65.8 (48.3 to 87.5)	-17.6 (-37.2 to 6.6)
		Mycoplasma spp.	16700 (15100 to 18500)	16900 (15500 to 18500)	1.5 (-6.9 to 10.7)	181 (157 to 208)	116 (86 to 149)	-36.0 (-53.4 to -16.8)
		Polymicrobial	640 (473 to 875)	734 (527 to 1020)	14.7 (-1.7 to 31.2)	90.2 (51.8 to 143)	59.1 (31.7 to 101)	-33.9 (-55.4 to -6.7)
		Pseudomonas aeruginosa	5490 (4980 to 6030)	8340 (7690 to 8990)	52.0 (40.2 to 63.8)	220 (193 to 252)	177 (131 to 228)	-19.2 (-41.0 to 6.2)
		Respiratory syncytial virus	16700 (15100 to 18600)	1530 (745 to 2630)	-90.8 (-95.6 to -84.4)	543 (479 to 617)	38 (16.8 to 70.9)	-93.0 (-97.0 to -87.0)
		Staphylococcus aureus	8870 (8210 to 9600)	13900 (12900 to 14900)	56.5 (46.6 to 67.8)	509 (451 to 573)	442 (336 to 551)	-13.0 (-34.1 to 10.8)
		Streptococcus pneumoniae	102000 (93500 to 111000)	70500 (65900 to 75500)	-30.5 (-34.9 to -25.5)	2680 (2330 to 3050)	1160 (843 to 1500)	-56.8 (-69.1 to -42.7)
		Other viruses	46200 (42100 to 50800)	36000 (33400 to 38700)	-21.9 (-27.3 to -16.1)	603 (526 to 692)	304 (216 to 397)	-49.4 (-64.2 to -32.2)
		Other bacterial pathogens	9660 (8740 to 10600)	11300 (10200 to 12500)	17.5 (7.7 to 27.7)	174 (146 to 211)	120 (93.2 to 150)	-30.9 (-45.7 to -13.9)
	Under 5	*Acinetobacter baumannii*	566 (404 to 780)	288 (209 to 392)	-49.1 (-54.0 to -43.4)	124 (71.8 to 197)	64.1 (33.9 to 113)	-48.0 (-66.1 to -25.3)
		Chlamydia spp.	5420 (4680 to 6230)	2520 (2220 to 2880)	-53.4 (-58.9 to -47.6)	165 (141 to 190)	76.8 (52.2 to 108)	-53.2 (-69.2 to -34.0)
		Enterobacter spp.	398 (307 to 511)	275 (212 to 355)	-30.7 (-38.8 to -21.9)	51.6 (34.2 to 77.2)	32.4 (19 to 53.3)	-36.7 (-58.2 to -11.9)
		Escherichia coli	1570 (1320 to 1860)	972 (834 to 1120)	-38.0 (-45.5 to -30.1)	188 (155 to 229)	108 (72.8 to 154)	-42.4 (-62.0 to -18.9)
		Fungus	289 (204 to 401)	177 (125 to 243)	-38.4 (-46.8 to -29.8)	18.9 (12.9 to 26.8)	10.3 (6.13 to 15.8)	-45.0 (-64.4 to -20.6)
		Group B Streptococcus	1360 (1150 to 1600)	880 (754 to 1030)	-35.0 (-42.0 to -27.2)	156 (131 to 187)	91.9 (60.8 to 129)	-40.7 (-60.4 to -15.9)
		Haemophilus influenzae	9920 (8610 to 11500)	3560 (3080 to 4090)	-64.0 (-68.2 to -59.0)	346 (290 to 410)	63 (41.9 to 86.7)	-81.7 (-88.0 to -74.1)
		Influenza	8550 (7570 to 9700)	552 (268 to 944)	-93.5 (-96.9 to -88.9)	546 (469 to 628)	30.7 (13.3 to 56.4)	-94.4 (-97.5 to -89.5)
		Klebsiella pneumoniae	5680 (4930 to 6520)	2570 (2240 to 2930)	-54.7 (-59.4 to -50.0)	500 (430 to 578)	223 (150 to 306)	-55.3 (-70.1 to -37.1)
		Legionella spp.	426 (309 to 576)	251 (185 to 338)	-41.0 (-46.8 to -34.1)	56.7 (37.9 to 80)	34.6 (19.9 to 53.2)	-38.6 (-59.2 to -12.1)
		Mycoplasma spp.	5920 (5150 to 6820)	3490 (3070 to 3990)	-40.9 (-47.1 to -34.0)	146 (124 to 172)	77.5 (52.1 to 108)	-46.8 (-64.6 to -24.7)
		Polymicrobial	292 (183 to 445)	165 (104 to 249)	-43.5 (-48.6 to -36.9)	85 (47.1 to 138)	52.5 (25.9 to 93.5)	-37.9 (-59.9 to -10.4)
		Pseudomonas aeruginosa	2380 (2080 to 2740)	1830 (1610 to 2080)	-23.0 (-31.8 to -13.8)	189 (163 to 220)	126 (84.9 to 173)	-32.9 (-55.8 to -5.5)
		Respiratory syncytial virus	15300 (13700 to 17100)	1240 (601 to 2120)	-91.9 (-96.1 to -86.2)	537 (473 to 611)	36.9 (16.3 to 68.9)	-93.1 (-97.0 to -87.1)

		Staphylococcus aureus	3290 (2900 to 3710)	2400 (2120 to 2710)	-26.9 (-33.9 to -19.8)	394 (338 to 451)	256 (170 to 349)	-34.9 (-56.2 to -8.3)
		Streptococcus pneumoniae	57000 (50900 to 64200)	22100 (19600 to 25000)	-61.2 (-64.7 to -57.6)	2340 (2000 to 2700)	843 (566 to 1150)	-63.9 (-76.3 to -48.9)
		Other viruses	31800 (28400 to 35800)	14900 (13200 to 16700)	-53.2 (-57.6 to -48.8)	561 (488 to 648)	251 (170 to 340)	-55.1 (-70.4 to -37.3)
		Other bacterial pathogens	1780 (1440 to 2180)	857 (687 to 1040)	-51.8 (-57.4 to -45.6)	107 (83.1 to 140)	51.3 (31.9 to 75)	-52.0 (-68.2 to -33.0)
	5-14 years	*Acinetobacter baumannii*	707 (563 to 877)	671 (533 to 829)	-4.7 (-18.6 to 11.9)	7.07 (5.78 to 8.58)	7.52 (5.46 to 9.97)	6.8 (-22.1 to 39.6)
		Chlamydia spp.	2350 (1920 to 2910)	1720 (1400 to 2060)	-26.6 (-36.8 to -14.6)	5.83 (4.82 to 6.9)	4.77 (3.47 to 6.19)	-17.7 (-39.8 to 6.9)
		Enterobacter spp.	148 (116 to 186)	180 (141 to 228)	22.5 (5.0 to 44.2)	1.4 (1.1 to 1.75)	1.91 (1.36 to 2.55)	37.4 (1.1 to 79.4)
		Escherichia coli	453 (367 to 556)	464 (375 to 558)	2.8 (-12.2 to 20.9)	4.44 (3.67 to 5.33)	5.1 (3.74 to 6.6)	15.2 (-16.0 to 48.8)
		Fungus	549 (434 to 693)	572 (464 to 708)	4.6 (-10.8 to 22.4)	5.3 (4.26 to 6.44)	6.19 (4.5 to 8.02)	17.2 (-14.4 to 51.1)
		Group B Streptococcus	524 (422 to 647)	595 (486 to 720)	14.0 (-2.0 to 33.8)	3.64 (3.01 to 4.4)	4.64 (3.39 to 6.1)	27.8 (-6.5 to 65.6)
		Haemophilus influenzae	1930 (1570 to 2350)	1430 (1170 to 1710)	-25.4 (-35.5 to -12.8)	7.21 (6.02 to 8.46)	4.09 (3.04 to 5.24)	-43.0 (-58.0 to -27.3)
		Influenza	2850 (2380 to 3470)	324 (153 to 562)	-88.6 (-94.6 to -80.6)	20.9 (17.6 to 24.2)	2.65 (1.22 to 4.75)	-87.2 (-94.2 to -75.9)
		Klebsiella pneumoniae	1340 (1110 to 1630)	1280 (1050 to 1520)	-4.3 (-17.0 to 11.3)	13 (11 to 15.3)	13.9 (10.5 to 17.9)	7.3 (-21.6 to 36.9)
		Legionella spp.	746 (611 to 920)	763 (621 to 914)	2.7 (-11.8 to 20.1)	5 (4.11 to 5.95)	5.73 (4.15 to 7.4)	15.1 (-16.5 to 48.7)
		Mycoplasma spp.	4440 (3680 to 5450)	3830 (3140 to 4550)	-13.5 (-25.2 to 1.0)	11.5 (9.76 to 13.5)	11.1 (8.21 to 14.2)	-3.1 (-29.1 to 23.9)
		Polymicrobial	96.2 (65.2 to 141)	95.6 (62.6 to 141)	-0.4 (-14.5 to 17.7)	0.96 (0.65 to 1.39)	1.06 (0.66 to 1.65)	11.7 (-18.3 to 45.0)
		Pseudomonas aeruginosa	876 (719 to 1060)	1130 (938 to 1340)	29.6 (11.6 to 51.2)	5.92 (4.98 to 6.9)	8.57 (6.34 to 11)	45.3 (7.0 to 88.0)
		Respiratory syncytial virus	554 (458 to 677)	74.7 (36.1 to 129)	-86.5 (-93.4 to -76.8)	1.85 (1.55 to 2.16)	0.28 (0.13 to 0.51)	-84.9 (-93.1 to -71.4)
		Staphylococcus aureus	1590 (1340 to 1940)	2010 (1670 to 2390)	26.9 (10.0 to 48.1)	22 (18.6 to 25.7)	31.2 (23.1 to 40.1)	42.3 (4.6 to 81.7)
		Streptococcus pneumoniae	16800 (14200 to 20400)	12000 (9940 to 14200)	-28.4 (-37.6 to -17.1)	97.1 (83.1 to 111)	77.6 (57.9 to 99.2)	-19.7 (-40.8 to 2.2)
		Other viruses	5620 (4720 to 6790)	5440 (4490 to 6410)	-3.0 (-15.6 to 12.5)	12.7 (10.8 to 14.6)	13.8 (10.4 to 17.7)	8.8 (-20.6 to 39.6)
		Other bacterial pathogens	2040 (1680 to 2460)	1650 (1340 to 1970)	-19.2 (-30.2 to -5.6)	11.2 (9.35 to 13.2)	10.1 (7.37 to 13.1)	-9.3 (-33.4 to 15.9)
	15-49 years	*Acinetobacter baumannii*	675 (571 to 792)	1070 (901 to 1270)	59.1 (40.1 to 81.4)	8.13 (6.71 to 10.1)	10.8 (7.99 to 14.6)	33.9 (-1.8 to 75.9)
		Chlamydia spp.	2240 (1940 to 2560)	2740 (2360 to 3110)	22.6 (9.3 to 38.4)	6.7 (5.61 to 8.03)	6.88 (5.12 to 9.16)	3.1 (-24.2 to 33.7)

		Enterobacter spp.	141 (115 to 171)	287 (238 to 343)	104.6 (79.1 to 134.1)	1.61 (1.27 to 2.04)	2.76 (1.99 to 3.81)	72.1 (26.9 to 126.7)
		Escherichia coli	432 (371 to 499)	741 (639 to 845)	71.7 (52.5 to 94.7)	5.11 (4.26 to 6.1)	7.35 (5.43 to 9.79)	44.4 (7.0 to 93.8)
		Fungus	524 (439 to 629)	913 (772 to 1070)	74.7 (55.3 to 99.8)	6.1 (4.95 to 7.43)	8.92 (6.5 to 11.9)	46.9 (8.8 to 93.3)
		Group B Streptococcus	500 (426 to 593)	950 (812 to 1090)	90.5 (68.4 to 118.4)	4.19 (3.38 to 5.11)	6.69 (4.97 to 8.85)	60.2 (17.2 to 111.4)
		Haemophilus influenzae	1840 (1620 to 2070)	2290 (2000 to 2590)	24.6 (11.4 to 41.3)	8.29 (7.07 to 9.86)	5.9 (4.39 to 7.76)	-28.6 (-46.9 to -5.7)
		Influenza	2720 (2460 to 3050)	518 (242 to 890)	-80.9 (-90.8 to -67.2)	24 (20.6 to 28.3)	3.83 (1.71 to 7.09)	-84.0 (-93.1 to -70.2)
		Klebsiella pneumoniae	1280 (1130 to 1430)	2040 (1800 to 2280)	59.8 (42.9 to 79.2)	15 (12.8 to 17.7)	20.1 (15.1 to 26.4)	34.4 (0.5 to 75.8)
		Legionella spp.	712 (610 to 823)	1220 (1060 to 1380)	71.5 (50.0 to 95.6)	5.75 (4.76 to 6.96)	8.26 (6.19 to 10.9)	44.3 (6.5 to 89.1)
		Mycoplasma spp.	4240 (3770 to 4750)	6110 (5380 to 6850)	44.4 (28.1 to 62.6)	13.2 (11.3 to 15.8)	16 (12.1 to 20.9)	21.5 (-10.6 to 59.7)
		Polymicrobial	91.8 (62.6 to 133)	153 (101 to 224)	66.3 (45.2 to 88.2)	1.1 (0.73 to 1.58)	1.54 (0.96 to 2.42)	39.9 (2.2 to 84.7)
		Pseudomonas aeruginosa	836 (737 to 939)	1810 (1600 to 2030)	116.4 (92.3 to 142.9)	6.81 (5.83 to 7.99)	12.4 (9.22 to 16.2)	82.1 (34.4 to 137.2)
		Respiratory syncytial virus	529 (464 to 599)	119 (56.8 to 206)	-77.4 (-89.1 to -61.4)	2.13 (1.81 to 2.49)	0.4 (0.17 to 0.76)	-81.0 (-92.0 to -64.6)
		Staphylococcus aureus	1520 (1360 to 1680)	3220 (2860 to 3570)	111.9 (91.1 to 136.2)	25.3 (21.9 to 29.6)	45.1 (34.1 to 58.9)	78.3 (33.2 to 134.9)
		Streptococcus pneumoniae	16100 (14600 to 17700)	19200 (17100 to 21200)	19.6 (8.0 to 33.9)	112 (96.6 to 130)	112 (84.6 to 147)	0.6 (-25.5 to 32.3)
		Other viruses	5360 (4860 to 5920)	8680 (7730 to 9610)	62.1 (46.1 to 81.3)	14.6 (12.6 to 17.2)	19.9 (15.1 to 25.9)	36.4 (1.2 to 78.9)
		Other bacterial pathogens	1950 (1700 to 2200)	2630 (2280 to 3000)	35.0 (20.4 to 53.1)	12.9 (10.9 to 15.2)	14.6 (10.8 to 19.4)	13.6 (-15.5 to 48.0)
	50-69 years	*Acinetobacter baumannii*	781 (663 to 912)	1640 (1390 to 1920)	110.7 (86.3 to 134.7)	12.5 (10.1 to 15.3)	16.8 (12.5 to 21.7)	34.8 (-1.9 to 78.0)
		Chlamydia spp.	1530 (1340 to 1750)	2390 (2100 to 2710)	56.5 (38.2 to 75.5)	6.11 (5 to 7.38)	6.08 (4.52 to 7.81)	0.1 (-26.9 to 31.6)
		Enterobacter spp.	158 (131 to 194)	421 (350 to 507)	166.8 (133.1 to 200.8)	2.41 (1.88 to 3.05)	4.08 (3 to 5.33)	70.7 (23.2 to 124.4)
		Escherichia coli	536 (465 to 617)	1180 (1030 to 1350)	120.8 (96.8 to 146.5)	8.44 (6.85 to 10.2)	11.8 (8.77 to 15.1)	41.3 (3.0 to 87.5)
		Fungus	517 (434 to 609)	1180 (1010 to 1390)	128.7 (101.9 to 155.3)	8.02 (6.47 to 9.86)	11.6 (8.57 to 15.1)	46.3 (6.6 to 93.5)
		Group B Streptococcus	397 (339 to 465)	973 (840 to 1120)	145.5 (114.0 to 174.9)	4.44 (3.58 to 5.43)	6.92 (5.19 to 8.81)	57.1 (12.8 to 105.4)
		Haemophilus influenzae	1250 (1100 to 1420)	2130 (1870 to 2430)	70.6 (51.8 to 89.2)	7.49 (6.25 to 8.96)	5.54 (4.12 to 7.1)	-25.6 (-46.0 to -2.2)
		Influenza	1590 (1420 to 1780)	399 (191 to 687)	-74.8 (-88.2 to -56.7)	18.7 (15.7 to 22)	2.98 (1.27 to 5.45)	-84.0 (-93.1 to -69.6)

		Klebsiella pneumoniae	1110 (989 to 1240)	2490 (2210 to 2790)	124.4 (101.3 to 147.7)	17.4 (14.6 to 20.4)	24.8 (18.8 to 31.3)	43.6 (4.2 to 88.5)
		Legionella spp.	679 (582 to 786)	1520 (1330 to 1720)	123.9 (97.7 to 152.3)	7.31 (5.97 to 8.86)	10.4 (7.8 to 13)	43.3 (3.2 to 90.8)
		Mycoplasma spp.	1320 (1160 to 1490)	2360 (2070 to 2680)	79.4 (58.5 to 99.8)	5.48 (4.54 to 6.57)	6.24 (4.68 to 7.9)	14.8 (-17.5 to 52.7)
		Polymicrobial	88.4 (61.3 to 128)	193 (130 to 285)	118.5 (91.7 to 145.8)	1.41 (0.94 to 2.07)	1.96 (1.23 to 3.05)	39.8 (1.0 to 80.8)
		Pseudomonas aeruginosa	804 (711 to 902)	2230 (1970 to 2510)	178.3 (145.0 to 208.9)	8.73 (7.27 to 10.3)	15.4 (11.8 to 19.4)	78.1 (28.8 to 135.6)
		Respiratory syncytial virus	229 (200 to 261)	66.5 (31.8 to 117)	-70.9 (-86.2 to -50.1)	1.23 (1.02 to 1.47)	0.23 (0.1 to 0.42)	-81.4 (-92.0 to -64.9)
		Staphylococcus aureus	1290 (1150 to 1430)	3570 (3170 to 4000)	178.1 (149.3 to 207.1)	28.6 (24.2 to 33.7)	50.5 (38.3 to 63.7)	78.0 (28.5 to 132.9)
		Streptococcus pneumoniae	6710 (6090 to 7450)	10800 (9660 to 12000)	60.9 (44.2 to 77.8)	62.1 (52.5 to 72.9)	63.5 (48.1 to 80.2)	2.9 (-25.5 to 34.7)
		Other viruses	1960 (1770 to 2180)	4430 (3970 to 4950)	126.3 (103.7 to 150.1)	7.13 (5.99 to 8.4)	10.3 (7.72 to 13)	44.8 (4.4 to 89.7)
		Other bacterial pathogens	2000 (1760 to 2260)	3510 (3050 to 4020)	76.0 (55.0 to 96.2)	17.6 (14.8 to 20.8)	19.7 (14.6 to 25.4)	12.7 (-18.5 to 46.2)
	70+ years	*Acinetobacter baumannii*	554 (459 to 668)	945 (790 to 1130)	71.0 (47.3 to 95.5)	13.3 (10.6 to 16.6)	15.3 (11.2 to 20.2)	15.9 (-16.8 to 56.8)
		Chlamydia spp.	1300 (1090 to 1520)	1640 (1410 to 1900)	27.1 (9.3 to 45.1)	7.71 (6.24 to 9.38)	6.59 (4.91 to 8.65)	-13.9 (-39.0 to 15.9)
		Enterobacter spp.	110 (87.7 to 137)	236 (192 to 289)	116.6 (86.4 to 147.8)	2.49 (1.88 to 3.18)	3.62 (2.55 to 4.87)	46.7 (6.0 to 99.6)
		Escherichia coli	468 (388 to 556)	836 (714 to 983)	79.3 (54.4 to 104.6)	11 (8.81 to 13.3)	13.2 (9.83 to 17.3)	21.4 (-13.1 to 63.2)
		Fungus	205 (164 to 247)	378 (314 to 457)	85.7 (60.3 to 113.9)	4.73 (3.71 to 5.88)	5.91 (4.29 to 7.78)	25.7 (-9.0 to 70.7)
		Group B Streptococcus	259 (216 to 304)	514 (442 to 594)	99.3 (70.4 to 128.2)	4.31 (3.45 to 5.26)	5.78 (4.37 to 7.58)	35.0 (-2.9 to 82.3)
		Haemophilus influenzae	798 (680 to 930)	1100 (943 to 1270)	38.5 (19.6 to 57.4)	7.15 (5.79 to 8.59)	4.54 (3.44 to 5.85)	-36.1 (-54.0 to -15.0)
		Influenza	1720 (1470 to 1950)	349 (169 to 605)	-79.6 (-90.0 to -65.2)	30.1 (24.6 to 35.4)	4.12 (1.71 to 7.6)	-86.2 (-94.1 to -74.1)
		Klebsiella pneumoniae	869 (752 to 994)	1580 (1370 to 1830)	82.2 (58.6 to 107.1)	20.3 (16.5 to 24.2)	24.9 (18.9 to 32.3)	23.4 (-11.1 to 67.9)
		Legionella spp.	346 (288 to 411)	628 (541 to 733)	81.8 (55.3 to 108.5)	5.57 (4.45 to 6.84)	6.8 (5.06 to 8.96)	23.1 (-11.6 to 68.2)
		Mycoplasma spp.	791 (676 to 912)	1150 (994 to 1320)	45.6 (26.5 to 65.2)	4.91 (3.97 to 5.88)	4.8 (3.63 to 6.25)	-1.3 (-28.9 to 32.0)
		Polymicrobial	71.9 (48.6 to 105)	128 (83.2 to 189)	77.4 (52.7 to 104.1)	1.71 (1.1 to 2.5)	2.05 (1.25 to 3.27)	20.2 (-13.6 to 63.2)
		Pseudomonas aeruginosa	594 (512 to 688)	1340 (1160 to 1530)	125.9 (95.3 to 157.2)	9.63 (7.85 to 11.6)	14.6 (11.2 to 19.1)	53.0 (10.1 to 105.6)
		Respiratory syncytial virus	152 (129 to 178)	35.9 (17.5 to 62.5)	-76.4 (-88.6 to -59.7)	1.22 (0.98 to 1.47)	0.19 (0.08 to 0.36)	-84.0 (-93.2 to -70.1)

		Staphylococcus aureus	1180 (1020 to 1350)	2660 (2320 to 3060)	125.8 (96.8 to 155.6)	39.2 (32.2 to 47.1)	59.5 (45.3 to 77)	52.9 (9.7 to 105.1)
		Streptococcus pneumoniae	4900 (4230 to 5540)	6390 (5590 to 7280)	30.6 (13.5 to 47.6)	67.7 (55.7 to 80.2)	59.5 (45.8 to 76.9)	-11.5 (-36.2 to 19.2)
		Other viruses	1420 (1230 to 1620)	2590 (2280 to 2970)	83.7 (59.9 to 108.6)	7.69 (6.31 to 9.13)	9.5 (7.3 to 12.3)	24.5 (-10.6 to 68.0)
		Other bacterial pathogens	1900 (1620 to 2210)	2700 (2310 to 3200)	42.9 (22.8 to 62.2)	25.2 (20.2 to 30.3)	24.3 (17.8 to 31.7)	-3.1 (-30.5 to 32.0)
Turkmenistan	All Ages	*Acinetobacter baumannii*	1620 (1400 to 1870)	1090 (961 to 1230)	-32.3 (-38.6 to -25.5)	85.5 (56.7 to 128)	26.9 (20.5 to 36)	-68.0 (-74.8 to -58.6)
		Chlamydia spp.	7300 (6610 to 8130)	3450 (3160 to 3760)	-52.7 (-56.8 to -48.4)	102 (89.9 to 117)	28.1 (23.8 to 33.4)	-72.4 (-77.5 to -66.6)
		Enterobacter spp.	578 (479 to 683)	531 (467 to 601)	-7.7 (-17.8 to 2.7)	34.3 (23.7 to 49.5)	15.6 (12.3 to 20.3)	-53.7 (-64.7 to -40.2)
		Escherichia coli	2060 (1820 to 2350)	1540 (1410 to 1670)	-25.2 (-32.4 to -17.9)	121 (102 to 143)	50.2 (42.3 to 58.9)	-58.3 (-66.3 to -48.8)
		Fungus	1120 (950 to 1290)	1090 (964 to 1230)	-2.2 (-11.3 to 7.2)	20.9 (16.9 to 25.8)	16.9 (13.5 to 21)	-18.7 (-34.5 to 0.6)
		Group B Streptococcus	1830 (1610 to 2060)	1420 (1290 to 1550)	-22.2 (-29.6 to -14.4)	98.6 (84 to 116)	35.5 (29.8 to 42.3)	-63.8 (-70.8 to -55.2)
		Haemophilus influenzae	10200 (9110 to 11500)	4470 (4070 to 4870)	-56.3 (-60.9 to -51.4)	215 (184 to 251)	36.2 (30.2 to 43.2)	-83.0 (-86.5 to -79.2)
		Influenza	10800 (9920 to 11900)	779 (377 to 1330)	-92.8 (-96.6 to -87.6)	354 (313 to 401)	15.3 (6.92 to 27.2)	-95.7 (-98.0 to -92.1)
		Klebsiella pneumoniae	6320 (5770 to 6970)	3380 (3140 to 3650)	-46.4 (-51.2 to -41.3)	310 (273 to 354)	99.2 (84.4 to 117)	-67.9 (-73.6 to -60.4)
		Legionella spp.	1680 (1480 to 1910)	1890 (1730 to 2060)	13.2 (3.4 to 24.3)	45.8 (34.5 to 59.5)	25.9 (21.5 to 31.3)	-42.6 (-55.5 to -27.3)
		Mycoplasma spp.	9970 (9010 to 11000)	7080 (6480 to 7710)	-28.9 (-34.9 to -22.8)	99.4 (87 to 115)	52.9 (44.4 to 62.2)	-46.6 (-56.7 to -35.0)
		Polymicrobial	372 (269 to 523)	226 (159 to 324)	-39.1 (-50.0 to -28.2)	54.2 (31.2 to 86.5)	15.4 (9.07 to 24)	-71.2 (-77.4 to -63.4)
		Pseudomonas aeruginosa	3460 (3140 to 3800)	3650 (3370 to 3930)	5.8 (-2.9 to 14.9)	128 (112 to 147)	77.1 (65.8 to 90.3)	-39.6 (-50.9 to -26.5)
		Respiratory syncytial virus	12400 (11300 to 13700)	799 (391 to 1360)	-93.5 (-96.9 to -89.1)	331 (292 to 377)	16.8 (7.45 to 30.9)	-94.9 (-97.7 to -90.5)
		Staphylococcus aureus	5340 (4930 to 5810)	5350 (4980 to 5710)	0.2 (-7.2 to 7.6)	281 (248 to 314)	177 (151 to 206)	-36.7 (-47.7 to -24.5)
		Streptococcus pneumoniae	64200 (59100 to 69600)	23500 (21800 to 25500)	-63.3 (-66.3 to -60.3)	1480 (1320 to 1670)	356 (304 to 414)	-75.9 (-80.4 to -70.7)
		Other viruses	32000 (29400 to 34900)	15500 (14200 to 16800)	-51.5 (-55.9 to -47.2)	357 (315 to 405)	124 (104 to 146)	-65.1 (-72.2 to -57.1)
		Other bacterial pathogens	5220 (4700 to 5820)	3900 (3490 to 4310)	-25.3 (-30.9 to -18.7)	88.2 (73.3 to 107)	42.7 (35.3 to 50.6)	-51.3 (-60.7 to -40.7)
	Under 5	*Acinetobacter baumannii*	365 (264 to 506)	77.9 (55.9 to 108)	-78.6 (-81.6 to -75.3)	69.9 (40.9 to 113)	13.4 (8.08 to 21.5)	-80.8 (-85.3 to -75.3)
		Chlamydia spp.	3650 (3190 to 4220)	840 (722 to 972)	-76.9 (-80.3 to -73.5)	91.6 (79.5 to 106)	19.6 (15.5 to 24.5)	-78.5 (-83.5 to -72.4)

		Enterobacter spp.	286 (222 to 370)	116 (91.1 to 146)	-59.2 (-65.6 to -51.8)	30.9 (20.5 to 46.2)	10.3 (7.22 to 14.6)	-66.0 (-74.6 to -54.3)
		Escherichia coli	1100 (928 to 1300)	429 (364 to 502)	-60.9 (-67.3 to -54.5)	109 (90.3 to 131)	35.6 (28.2 to 44.1)	-67.2 (-74.8 to -57.5)
		Fungus	204 (142 to 285)	70.8 (50.7 to 95.3)	-65.1 (-71.1 to -58.9)	10.5 (7.42 to 14.8)	4.07 (2.77 to 5.65)	-60.9 (-70.8 to -49.4)
		Group B Streptococcus	954 (806 to 1130)	349 (296 to 408)	-63.3 (-68.7 to -57.5)	91.4 (77 to 109)	25.8 (20.5 to 32.4)	-71.6 (-78.1 to -63.4)
		Haemophilus influenzae	7180 (6230 to 8280)	1720 (1490 to 1960)	-75.9 (-79.7 to -72.2)	201 (171 to 237)	27.3 (21.8 to 33.9)	-86.3 (-89.6 to -82.5)
		Influenza	6150 (5470 to 6960)	191 (94.8 to 325)	-96.9 (-98.5 to -94.7)	314 (274 to 361)	9.63 (4.24 to 17.8)	-96.9 (-98.6 to -94.3)
		Klebsiella pneumoniae	3980 (3510 to 4500)	916 (791 to 1050)	-76.9 (-80.2 to -73.7)	283 (247 to 325)	67.3 (53.5 to 83.3)	-76.1 (-81.6 to -69.1)
		Legionella spp.	313 (226 to 427)	117 (85.6 to 161)	-62.7 (-68.4 to -56.3)	34.9 (24 to 48.4)	10.3 (7.25 to 13.9)	-70.1 (-77.8 to -60.6)
		Mycoplasma spp.	4240 (3730 to 4840)	2090 (1790 to 2380)	-50.7 (-58.4 to -43.8)	83.7 (71.8 to 98.1)	36.4 (29.1 to 44.7)	-56.2 (-66.3 to -43.5)
		Polymicrobial	201 (125 to 307)	56.1 (34.3 to 85.7)	-72.1 (-76.0 to -68.0)	52.1 (29.1 to 84.5)	13.2 (6.88 to 21.8)	-74.6 (-80.7 to -66.9)
		Pseudomonas aeruginosa	1770 (1540 to 2020)	1040 (905 to 1200)	-40.9 (-50.1 to -32.2)	114 (98.8 to 132)	53.7 (43 to 65.8)	-52.7 (-63.7 to -38.9)
		Respiratory syncytial virus	11600 (10500 to 12900)	675 (331 to 1150)	-94.2 (-97.2 to -90.0)	328 (289 to 374)	16.3 (7.19 to 30)	-95.0 (-97.8 to -90.7)
		Staphylococcus aureus	2400 (2130 to 2680)	1030 (904 to 1170)	-56.9 (-62.8 to -51.9)	231 (200 to 263)	97.6 (78.2 to 122)	-57.6 (-67.1 to -46.0)
		Streptococcus pneumoniae	40900 (36900 to 45600)	6220 (5420 to 7070)	-84.8 (-86.8 to -82.9)	1330 (1170 to 1520)	227 (181 to 278)	-82.9 (-86.7 to -78.1)
		Other viruses	24100 (21600 to 26800)	6410 (5590 to 7280)	-73.3 (-76.9 to -70.2)	337 (295 to 385)	97.5 (78.4 to 119)	-71.0 (-77.4 to -62.7)
		Other bacterial pathogens	1240 (999 to 1510)	357 (288 to 439)	-71.1 (-75.6 to -65.9)	60.7 (46.6 to 79.7)	16.5 (12.3 to 21.8)	-72.7 (-79.0 to -64.9)
	5-14 years	*Acinetobacter baumannii*	353 (280 to 438)	162 (129 to 198)	-53.8 (-60.5 to -46.0)	2.96 (2.45 to 3.48)	1.59 (1.27 to 1.96)	-46.1 (-56.8 to -33.2)
		Chlamydia spp.	1240 (1010 to 1520)	537 (436 to 654)	-56.5 (-62.7 to -48.9)	2.59 (2.19 to 3.03)	1.31 (1.05 to 1.59)	-49.2 (-59.4 to -37.1)
		Enterobacter spp.	83.5 (64.6 to 106)	68.4 (54.3 to 85.1)	-17.7 (-30.0 to -2.7)	0.66 (0.54 to 0.81)	0.64 (0.5 to 0.78)	-3.9 (-24.2 to 18.7)
		Escherichia coli	249 (203 to 306)	163 (132 to 197)	-34.0 (-44.0 to -22.1)	2.05 (1.74 to 2.39)	1.57 (1.29 to 1.9)	-23.0 (-38.6 to -5.1)
		Fungus	301 (236 to 377)	198 (158 to 242)	-34.0 (-44.0 to -22.5)	2.44 (2.02 to 2.92)	1.87 (1.51 to 2.3)	-23.0 (-38.0 to -4.1)
		Group B Streptococcus	292 (233 to 361)	214 (174 to 259)	-26.2 (-37.6 to -13.1)	1.71 (1.44 to 2.01)	1.46 (1.18 to 1.78)	-13.9 (-31.4 to 6.8)
		Haemophilus influenzae	1090 (881 to 1310)	583 (478 to 700)	-46.4 (-53.9 to -36.7)	3.43 (2.97 to 3.96)	1.42 (1.16 to 1.68)	-58.6 (-66.7 to -48.8)
		Influenza	1600 (1320 to 1930)	122 (59.9 to 211)	-92.3 (-96.3 to -86.8)	9.86 (8.67 to 11.3)	0.88 (0.39 to 1.6)	-91.1 (-95.9 to -83.5)

		Klebsiella pneumoniae	734 (601 to 884)	443 (363 to 528)	-39.5 (-47.8 to -28.6)	6.01 (5.26 to 6.89)	4.23 (3.47 to 5.01)	-29.4 (-43.2 to -13.0)
		Legionella spp.	441 (355 to 543)	338 (278 to 407)	-22.9 (-34.0 to -8.9)	2.49 (2.1 to 2.92)	2.23 (1.84 to 2.68)	-10.0 (-28.1 to 10.8)
		Mycoplasma spp.	2500 (2050 to 3010)	1440 (1180 to 1710)	-42.2 (-50.3 to -32.3)	5.44 (4.69 to 6.23)	3.65 (2.98 to 4.39)	-32.6 (-46.0 to -17.4)
		Polymicrobial	50.8 (33.3 to 74)	29.4 (18.3 to 44.2)	-42.1 (-51.4 to -31.1)	0.42 (0.28 to 0.61)	0.29 (0.18 to 0.44)	-32.4 (-46.6 to -15.2)
		Pseudomonas aeruginosa	512 (419 to 617)	464 (381 to 555)	-8.9 (-21.9 to 7.8)	2.91 (2.54 to 3.34)	3.08 (2.53 to 3.69)	6.3 (-14.9 to 32.4)
		Respiratory syncytial virus	330 (268 to 400)	31.9 (15.1 to 55)	-90.3 (-95.3 to -83.5)	0.92 (0.8 to 1.08)	0.1 (0.05 to 0.19)	-88.7 (-94.8 to -79.2)
		Staphylococcus aureus	905 (751 to 1090)	777 (637 to 926)	-13.9 (-25.1 to 1.0)	10.5 (9.27 to 11.9)	10.6 (8.66 to 12.5)	0.5 (-19.3 to 23.7)
		Streptococcus pneumoniae	9400 (7820 to 11200)	4400 (3620 to 5230)	-53.0 (-59.3 to -44.9)	45.5 (40.2 to 51.4)	24.9 (20.5 to 29.5)	-45.2 (-55.9 to -32.7)
		Other viruses	3320 (2740 to 3960)	2400 (1970 to 2850)	-27.5 (-37.1 to -14.9)	6.32 (5.56 to 7.15)	5.33 (4.38 to 6.34)	-15.4 (-31.9 to 4.0)
		Other bacterial pathogens	1110 (903 to 1350)	570 (455 to 688)	-48.7 (-55.8 to -39.9)	5.14 (4.42 to 5.93)	3.07 (2.48 to 3.72)	-40.0 (-51.8 to -25.8)
	15-49 years	*Acinetobacter baumannii*	307 (261 to 364)	258 (220 to 302)	-15.8 (-24.9 to -7.1)	3.27 (2.77 to 3.82)	3.58 (2.67 to 4.71)	9.9 (-17.7 to 47.2)
		Chlamydia spp.	1080 (933 to 1240)	853 (747 to 967)	-20.8 (-28.9 to -13.7)	2.85 (2.48 to 3.27)	2.95 (2.19 to 3.92)	3.4 (-22.8 to 36.9)
		Enterobacter spp.	72.7 (59.8 to 87.5)	109 (92.1 to 126)	49.8 (32.4 to 67.5)	0.73 (0.61 to 0.88)	1.43 (1.05 to 1.91)	95.7 (45.8 to 162.9)
		Escherichia coli	216 (186 to 249)	259 (227 to 296)	20.2 (8.1 to 32.4)	2.26 (1.97 to 2.59)	3.54 (2.67 to 4.66)	56.9 (16.6 to 107.1)
		Fungus	262 (218 to 308)	314 (269 to 363)	20.1 (7.5 to 32.2)	2.7 (2.27 to 3.15)	4.22 (3.17 to 5.63)	56.8 (16.8 to 109.4)
		Group B Streptococcus	254 (219 to 300)	340 (293 to 392)	34.4 (20.6 to 49.5)	1.88 (1.62 to 2.19)	3.3 (2.49 to 4.36)	75.5 (29.6 to 132.2)
		Haemophilus influenzae	950 (841 to 1070)	926 (817 to 1050)	-2.4 (-11.8 to 7.1)	3.79 (3.38 to 4.25)	3.19 (2.42 to 4.26)	-15.6 (-37.1 to 10.0)
		Influenza	1390 (1260 to 1560)	194 (93.6 to 335)	-86.0 (-93.3 to -76.0)	10.9 (9.8 to 12)	1.96 (0.91 to 3.32)	-82.0 (-91.7 to -68.3)
		Klebsiella pneumoniae	639 (569 to 714)	703 (622 to 785)	10.2 (0.2 to 20.2)	6.64 (5.97 to 7.39)	9.53 (7.17 to 12.5)	43.9 (7.1 to 90.4)
		Legionella spp.	383 (330 to 439)	537 (473 to 609)	40.4 (25.4 to 55.9)	2.74 (2.38 to 3.18)	5.01 (3.77 to 6.51)	83.4 (36.9 to 144.9)
		Mycoplasma spp.	2170 (1940 to 2440)	2280 (2030 to 2550)	5.2 (-4.9 to 15.1)	6 (5.35 to 6.65)	8.22 (6.19 to 10.9)	37.4 (2.1 to 80.0)
		Polymicrobial	44.2 (29.4 to 64.4)	46.7 (29.8 to 70.5)	5.5 (-7.0 to 18.0)	0.47 (0.32 to 0.67)	0.64 (0.4 to 1.02)	37.7 (3.0 to 85.0)
		Pseudomonas aeruginosa	445 (392 to 505)	737 (653 to 824)	65.8 (49.1 to 81.8)	3.21 (2.85 to 3.58)	6.94 (5.26 to 9.06)	116.5 (60.9 to 187.8)
		Respiratory syncytial virus	287 (254 to 323)	50.7 (24.2 to 86.8)	-82.3 (-91.5 to -69.8)	1.02 (0.9 to 1.15)	0.23 (0.11 to 0.4)	-77.1 (-89.6 to -59.6)

		Staphylococcus aureus	788 (711 to 874)	1230 (1100 to 1380)	56.8 (42.3 to 71.1)	11.6 (10.5 to 12.8)	23.8 (18 to 31.2)	104.8 (52.3 to 171.6)
		Streptococcus pneumoniae	8170 (7450 to 9010)	6980 (6230 to 7760)	-14.5 (-22.4 to -6.9)	50.3 (45.8 to 54.6)	56 (42.3 to 73.4)	11.6 (-16.4 to 47.6)
		Other viruses	2890 (2620 to 3200)	3800 (3400 to 4230)	31.9 (19.4 to 43.5)	6.98 (6.3 to 7.66)	12 (9.02 to 15.9)	72.3 (29.3 to 128.8)
		Other bacterial pathogens	969 (848 to 1100)	905 (780 to 1040)	-6.5 (-16.0 to 2.7)	5.67 (5.01 to 6.41)	6.91 (5.16 to 9.16)	22.2 (-8.9 to 64.7)
	50-69 years	*Acinetobacter baumannii*	343 (290 to 402)	366 (312 to 429)	6.9 (-6.3 to 19.7)	5.21 (4.48 to 6.07)	4.84 (3.61 to 6.29)	-6.8 (-30.8 to 22.3)
		Chlamydia spp.	713 (615 to 813)	697 (622 to 784)	-2.0 (-13.5 to 9.7)	2.69 (2.38 to 3.01)	2.29 (1.74 to 3.01)	-14.6 (-36.6 to 11.2)
		Enterobacter spp.	78.9 (64.8 to 95.2)	148 (127 to 171)	88.3 (65.5 to 112.7)	1.13 (0.94 to 1.36)	1.86 (1.35 to 2.44)	64.2 (23.2 to 115.8)
		Escherichia coli	259 (223 to 300)	386 (343 to 437)	49.5 (30.4 to 68.4)	3.86 (3.38 to 4.33)	5.02 (3.76 to 6.55)	30.3 (-3.7 to 69.2)
		Fungus	250 (212 to 296)	377 (328 to 433)	51.4 (32.1 to 69.4)	3.66 (3.12 to 4.24)	4.82 (3.61 to 6.31)	32.0 (-1.4 to 71.8)
		Group B Streptococcus	195 (166 to 229)	325 (284 to 370)	67.4 (45.5 to 87.6)	2.06 (1.8 to 2.37)	3 (2.26 to 3.92)	46.0 (7.1 to 91.4)
		Haemophilus influenzae	622 (550 to 709)	790 (703 to 892)	27.1 (11.7 to 41.0)	3.53 (3.16 to 3.93)	2.59 (1.93 to 3.38)	-26.6 (-45.9 to -3.9)
		Influenza	787 (702 to 882)	139 (65.9 to 242)	-82.3 (-91.7 to -68.7)	8.74 (7.91 to 9.56)	1.33 (0.61 to 2.33)	-84.7 (-93.0 to -73.1)
		Klebsiella pneumoniae	536 (482 to 605)	782 (700 to 867)	46.2 (29.6 to 62.5)	7.93 (7.21 to 8.67)	10.1 (7.51 to 13)	27.5 (-4.8 to 65.8)
		Legionella spp.	353 (304 to 408)	622 (554 to 700)	76.6 (54.6 to 99.3)	3.6 (3.14 to 4.1)	5.52 (4.13 to 7.16)	53.9 (16.1 to 103.8)
		Mycoplasma spp.	653 (578 to 743)	831 (744 to 924)	27.6 (12.4 to 43.1)	2.57 (2.3 to 2.83)	2.85 (2.14 to 3.67)	11.2 (-16.3 to 45.0)
		Polymicrobial	41.1 (28.1 to 60.2)	54.8 (35.8 to 84.2)	33.3 (15.6 to 51.3)	0.62 (0.42 to 0.9)	0.72 (0.45 to 1.16)	16.2 (-13.6 to 52.8)
		Pseudomonas aeruginosa	413 (364 to 467)	851 (761 to 950)	106.2 (82.0 to 131.0)	4.25 (3.82 to 4.69)	7.62 (5.7 to 9.85)	79.8 (33.9 to 135.1)
		Respiratory syncytial virus	120 (104 to 136)	26.3 (12.4 to 45.4)	-78.0 (-89.7 to -61.4)	0.61 (0.53 to 0.68)	0.12 (0.05 to 0.21)	-81.0 (-91.4 to -66.6)
		Staphylococcus aureus	644 (571 to 720)	1270 (1150 to 1410)	98.0 (75.0 to 120.3)	13.5 (12.3 to 14.8)	23.3 (17.4 to 30.1)	72.6 (28.4 to 123.8)
		Streptococcus pneumoniae	3300 (2940 to 3680)	3620 (3270 to 4010)	9.9 (-2.9 to 22.3)	28.9 (26.6 to 31.3)	27.6 (20.7 to 35.6)	-4.2 (-28.7 to 24.1)
		Other viruses	1020 (911 to 1140)	1770 (1590 to 1960)	74.3 (54.0 to 93.8)	3.51 (3.21 to 3.81)	5.32 (4 to 6.88)	52.0 (14.1 to 97.2)
		Other bacterial pathogens	959 (834 to 1090)	1120 (985 to 1280)	17.5 (3.6 to 31.4)	7.99 (7.1 to 8.9)	8.18 (6.08 to 10.7)	2.6 (-23.2 to 33.1)
	70+ years	*Acinetobacter baumannii*	251 (212 to 301)	229 (197 to 269)	-8.6 (-18.9 to 2.9)	4.15 (3.47 to 4.86)	3.5 (2.68 to 4.53)	-15.1 (-36.5 to 11.1)
		Chlamydia spp.	623 (534 to 721)	521 (457 to 601)	-16.2 (-25.8 to -5.9)	2.55 (2.19 to 2.92)	1.98 (1.54 to 2.52)	-22.2 (-41.6 to 0.6)

		Enterobacter spp.	56.3 (45.9 to 68.9)	90.4 (75.7 to 108)	61.0 (39.7 to 81.1)	0.88 (0.71 to 1.08)	1.31 (0.98 to 1.69)	49.4 (12.8 to 96.5)
		Escherichia coli	233 (200 to 276)	297 (262 to 342)	27.8 (12.1 to 43.3)	3.78 (3.23 to 4.42)	4.46 (3.44 to 5.76)	18.6 (-11.0 to 53.6)
		Fungus	102 (84.5 to 123)	132 (112 to 153)	29.4 (14.2 to 45.3)	1.63 (1.35 to 1.95)	1.95 (1.47 to 2.47)	20.1 (-8.6 to 56.8)
		Group B Streptococcus	131 (112 to 154)	187 (162 to 214)	43.1 (26.4 to 62.4)	1.51 (1.27 to 1.75)	1.99 (1.56 to 2.54)	32.8 (-0.7 to 71.6)
		Haemophilus influenzae	411 (360 to 476)	446 (393 to 510)	8.7 (-3.2 to 21.6)	2.54 (2.19 to 2.9)	1.69 (1.32 to 2.14)	-33.2 (-49.5 to -13.9)
		Influenza	877 (773 to 997)	133 (62.7 to 234)	-84.9 (-92.6 to -74.0)	10.6 (9.29 to 11.9)	1.47 (0.67 to 2.65)	-86.0 (-93.7 to -74.9)
		Klebsiella pneumoniae	433 (382 to 496)	540 (483 to 617)	25.0 (10.8 to 39.3)	6.97 (6.06 to 7.84)	8.06 (6.27 to 10.2)	16.0 (-11.9 to 49.1)
		Legionella spp.	186 (158 to 219)	280 (246 to 320)	51.0 (32.4 to 69.7)	2.06 (1.73 to 2.41)	2.88 (2.23 to 3.67)	40.1 (4.7 to 82.5)
		Mycoplasma spp.	405 (355 to 466)	440 (392 to 504)	9.1 (-3.9 to 22.0)	1.73 (1.49 to 1.98)	1.74 (1.36 to 2.22)	1.2 (-24.2 to 31.2)
		Polymicrobial	34.5 (22.9 to 51.6)	39.4 (25.2 to 60.5)	14.0 (-1.5 to 29.1)	0.57 (0.38 to 0.85)	0.6 (0.37 to 0.98)	5.7 (-20.5 to 38.3)
		Pseudomonas aeruginosa	316 (277 to 361)	555 (492 to 629)	76.3 (56.1 to 97.1)	3.53 (3.04 to 4.02)	5.75 (4.48 to 7.34)	63.6 (22.0 to 111.3)
		Respiratory syncytial virus	82.4 (71.1 to 96.4)	15.5 (7.38 to 27.4)	-81.2 (-90.9 to -67.6)	0.45 (0.39 to 0.52)	0.08 (0.04 to 0.14)	-82.6 (-92.2 to -68.5)
		Staphylococcus aureus	611 (541 to 695)	1030 (921 to 1160)	69.3 (50.7 to 88.1)	14 (12.3 to 15.7)	21.9 (17 to 27.8)	57.1 (18.9 to 102.5)
		Streptococcus pneumoniae	2490 (2220 to 2810)	2330 (2100 to 2640)	-6.0 (-16.6 to 4.2)	23.7 (20.8 to 26.5)	20.6 (16.1 to 26.1)	-12.8 (-34.2 to 11.8)
		Other viruses	760 (675 to 862)	1130 (1010 to 1280)	49.1 (32.3 to 65.1)	2.85 (2.5 to 3.2)	3.92 (3.08 to 4.95)	38.3 (4.3 to 78.7)
		Other bacterial pathogens	940 (817 to 1080)	943 (819 to 1090)	0.4 (-11.2 to 12.4)	8.63 (7.38 to 9.9)	8.02 (6.13 to 10.2)	-6.6 (-29.5 to 22.3)
Uzbekistan	All Ages	*Acinetobacter baumannii*	7930 (6910 to 9120)	7910 (6960 to 8980)	-0.1 (-9.2 to 9.3)	377 (247 to 563)	202 (135 to 302)	-46.0 (-55.8 to -34.6)
		Chlamydia spp.	34700 (31500 to 38300)	27600 (25200 to 30200)	-20.2 (-27.4 to -13.1)	443 (396 to 495)	202 (169 to 242)	-54.2 (-62.3 to -44.6)
		Enterobacter spp.	2940 (2490 to 3450)	4830 (4240 to 5490)	64.7 (47.5 to 82.5)	160 (112 to 236)	118 (86.4 to 165)	-25.5 (-40.0 to -9.0)
		Escherichia coli	10100 (8960 to 11300)	14100 (12900 to 15500)	40.6 (27.6 to 53.7)	544 (469 to 641)	371 (308 to 446)	-31.6 (-43.4 to -17.8)
		Fungus	6130 (5250 to 7080)	10000 (8840 to 11400)	63.4 (48.2 to 78.2)	90.3 (73.1 to 109)	93.5 (78.4 to 110)	4.1 (-12.4 to 22.6)
		Group B Streptococcus	8990 (8020 to 10200)	12300 (11100 to 13500)	36.8 (24.2 to 50.2)	456 (400 to 524)	288 (239 to 346)	-36.8 (-48.5 to -23.0)
		Haemophilus influenzae	48800 (43900 to 54700)	37900 (34500 to 41000)	-22.2 (-29.3 to -15.4)	957 (834 to 1090)	213 (179 to 252)	-77.6 (-81.8 to -72.9)
		Influenza	53700 (49700 to 59000)	7240 (3550 to 12400)	-86.5 (-93.5 to -76.6)	1560 (1410 to 1720)	97.7 (43.6 to 172)	-93.7 (-97.2 to -88.7)

		Klebsiella pneumoniae	29900 (27400 to 33100)	26800 (24700 to 29000)	-10.4 (-17.6 to -2.7)	1340 (1200 to 1490)	614 (521 to 728)	-54.1 (-61.8 to -44.9)
		Legionella spp.	9860 (8760 to 11100)	17300 (15800 to 19000)	76.0 (61.0 to 92.0)	223 (171 to 288)	192 (158 to 236)	-13.0 (-29.3 to 4.6)
		Mycoplasma spp.	51800 (47600 to 57100)	55200 (50700 to 60000)	6.6 (-1.5 to 14.8)	441 (393 to 497)	298 (255 to 354)	-32.4 (-43.4 to -19.0)
		Polymicrobial	1720 (1260 to 2390)	1990 (1390 to 2910)	15.5 (-3.3 to 33.0)	265 (150 to 431)	169 (93.5 to 276)	-35.8 (-49.1 to -19.0)
		Pseudomonas aeruginosa	18100 (16600 to 19800)	29900 (27700 to 32400)	65.4 (51.7 to 79.3)	603 (543 to 672)	497 (419 to 585)	-17.3 (-31.3 to -0.8)
		Respiratory syncytial virus	56400 (50900 to 63000)	5170 (2530 to 8870)	-90.8 (-95.5 to -84.3)	1560 (1420 to 1720)	105 (47.2 to 188)	-93.2 (-97.0 to -87.9)
		Staphylococcus aureus	28000 (26100 to 30400)	49000 (45600 to 52800)	74.9 (63.3 to 86.9)	1260 (1150 to 1380)	1100 (961 to 1260)	-12.9 (-25.6 to 1.2)
		Streptococcus pneumoniae	310000 (288000 to 337000)	156000 (145000 to 167000)	-49.6 (-53.5 to -46.0)	6350 (5860 to 6920)	1470 (1280 to 1690)	-76.8 (-80.4 to -72.8)
		Other viruses	157000 (144000 to 172000)	113000 (105000 to 122000)	-27.7 (-33.5 to -21.8)	1630 (1490 to 1780)	687 (580 to 816)	-57.7 (-64.8 to -49.2)
		Other bacterial pathogens	28100 (25500 to 31300)	33700 (30200 to 37800)	20.0 (10.5 to 29.7)	386 (320 to 467)	260 (216 to 312)	-32.3 (-42.4 to -20.7)
	Under 5	*Acinetobacter baumannii*	1320 (951 to 1830)	451 (316 to 630)	-65.9 (-70.6 to -61.3)	315 (183 to 498)	141 (75.9 to 244)	-55.1 (-65.8 to -42.6)
		Chlamydia spp.	14200 (12300 to 16500)	5130 (4390 to 5950)	-63.9 (-69.2 to -58.0)	396 (352 to 446)	157 (124 to 195)	-60.3 (-68.7 to -50.7)
		Enterobacter spp.	1190 (923 to 1510)	786 (619 to 989)	-33.8 (-43.9 to -22.7)	145 (96.9 to 219)	87.1 (55.7 to 133)	-39.5 (-52.4 to -23.5)
		Escherichia coli	4470 (3780 to 5290)	3000 (2540 to 3470)	-32.5 (-42.4 to -21.2)	492 (420 to 587)	281 (218 to 358)	-42.6 (-54.6 to -28.1)
		Fungus	842 (588 to 1170)	459 (325 to 623)	-45.2 (-54.5 to -34.4)	43.3 (30.3 to 59.3)	20.1 (13.9 to 28.5)	-53.3 (-64.4 to -39.8)
		Group B Streptococcus	3850 (3270 to 4540)	2300 (1950 to 2710)	-40.1 (-48.1 to -31.0)	423 (365 to 490)	231 (185 to 289)	-45.2 (-57.4 to -31.2)
		Haemophilus influenzae	30300 (26400 to 35200)	11700 (10100 to 13300)	-61.2 (-66.5 to -55.6)	895 (775 to 1030)	160 (128 to 200)	-82.0 (-86.1 to -77.4)
		Influenza	25800 (23000 to 29200)	1340 (660 to 2290)	-94.8 (-97.5 to -91.1)	1370 (1230 to 1530)	62.2 (28 to 110)	-95.4 (-98.0 to -91.8)
		Klebsiella pneumoniae	16300 (14300 to 18700)	5610 (4870 to 6390)	-65.5 (-69.9 to -60.7)	1220 (1080 to 1360)	446 (356 to 555)	-63.3 (-71.0 to -54.3)
		Legionella spp.	1320 (945 to 1790)	769 (570 to 1060)	-41.4 (-49.3 to -32.6)	170 (120 to 236)	103 (73 to 143)	-38.9 (-53.4 to -22.2)
		Mycoplasma spp.	17800 (15500 to 20400)	12900 (11200 to 14700)	-27.1 (-37.4 to -16.5)	366 (318 to 421)	210 (169 to 261)	-42.3 (-54.2 to -27.4)
		Polymicrobial	769 (484 to 1170)	380 (231 to 576)	-50.6 (-56.7 to -44.0)	256 (142 to 422)	156 (82.1 to 262)	-38.9 (-52.5 to -22.1)
		Pseudomonas aeruginosa	7640 (6680 to 8770)	6630 (5760 to 7510)	-13.0 (-25.3 to -0.7)	536 (478 to 605)	369 (297 to 454)	-31.0 (-45.4 to -14.1)
		Respiratory syncytial virus	51200 (45900 to 57700)	4120 (2040 to 7050)	-91.9 (-96.0 to -86.2)	1540 (1400 to 1700)	103 (46 to 183)	-93.3 (-97.0 to -88.2)

		Staphylococcus aureus	10200 (9020 to 11600)	7040 (6130 to 8010)	-31.0 (-39.0 to -22.9)	1030 (925 to 1140)	619 (500 to 767)	-39.9 (-52.1 to -25.1)
		Streptococcus pneumoniae	172000 (153000 to 194000)	31100 (27300 to 35200)	-81.9 (-84.1 to -79.8)	5650 (5180 to 6220)	888 (715 to 1100)	-84.3 (-87.5 to -80.4)
		Other viruses	107000 (95400 to 120000)	38400 (33700 to 43300)	-64.0 (-68.4 to -60.0)	1530 (1400 to 1680)	550 (443 to 674)	-64.0 (-71.4 to -55.4)
		Other bacterial pathogens	5050 (4020 to 6180)	2240 (1810 to 2730)	-55.5 (-62.5 to -47.4)	265 (203 to 349)	117 (80.1 to 166)	-55.9 (-65.6 to -43.5)
	5-14 years	*Acinetobacter baumannii*	1830 (1510 to 2230)	1110 (895 to 1340)	-39.3 (-47.9 to -30.2)	12.9 (10.9 to 15.1)	8.2 (6.75 to 9.75)	-36.4 (-47.3 to -23.1)
		Chlamydia spp.	6840 (5710 to 8300)	4400 (3630 to 5360)	-35.6 (-44.3 to -26.1)	12 (10.5 to 13.7)	8.07 (6.84 to 9.58)	-32.5 (-44.0 to -19.9)
		Enterobacter spp.	495 (398 to 611)	621 (501 to 760)	25.9 (8.2 to 44.9)	3.31 (2.78 to 3.95)	4.35 (3.6 to 5.22)	31.9 (9.2 to 57.6)
		Escherichia coli	1430 (1180 to 1730)	1540 (1270 to 1840)	7.8 (-7.4 to 23.4)	9.87 (8.66 to 11.3)	11.1 (9.48 to 13.1)	13.0 (-5.6 to 36.2)
		Fungus	1730 (1400 to 2130)	1720 (1410 to 2090)	0.0 (-13.4 to 14.4)	11.8 (9.95 to 13.7)	12.3 (10.3 to 14.7)	4.8 (-13.1 to 25.7)
		Group B Streptococcus	1700 (1400 to 2100)	1890 (1560 to 2290)	11.4 (-3.7 to 27.7)	8.36 (7.24 to 9.68)	9.72 (8.23 to 11.4)	16.8 (-2.7 to 40.5)
		Haemophilus influenzae	6480 (5470 to 7670)	5240 (4310 to 6210)	-18.9 (-29.0 to -8.3)	17.1 (15.1 to 19.2)	9.8 (8.43 to 11.4)	-42.5 (-51.7 to -32.4)
		Influenza	9440 (8020 to 11100)	1170 (550 to 2010)	-87.6 (-94.0 to -77.9)	48.7 (43.8 to 53.8)	6.29 (2.85 to 10.9)	-87.1 (-94.0 to -76.9)
		Klebsiella pneumoniae	4210 (3560 to 5000)	3550 (2990 to 4200)	-15.6 (-25.9 to -4.1)	29 (26 to 31.9)	25.5 (22 to 29.8)	-11.5 (-25.3 to 5.0)
		Legionella spp.	2730 (2280 to 3260)	2940 (2460 to 3480)	8.1 (-6.7 to 24.5)	12.9 (11.1 to 15)	14.6 (12.5 to 17.2)	13.3 (-5.8 to 35.7)
		Mycoplasma spp.	14700 (12400 to 17400)	11900 (9870 to 14200)	-19.3 (-29.7 to -8.0)	26.9 (24.1 to 29.9)	22.7 (19.6 to 26.3)	-15.4 (-29.3 to 0.8)
		Polymicrobial	280 (187 to 416)	261 (162 to 394)	-6.9 (-20.2 to 6.9)	1.96 (1.31 to 2.91)	1.91 (1.24 to 2.87)	-2.4 (-19.1 to 18.3)
		Pseudomonas aeruginosa	3140 (2630 to 3710)	3910 (3270 to 4620)	24.9 (9.0 to 42.4)	15 (13.3 to 16.8)	19.5 (16.8 to 22.7)	30.9 (9.4 to 55.5)
		Respiratory syncytial virus	2060 (1740 to 2470)	259 (122 to 448)	-87.4 (-93.9 to -77.5)	4.85 (4.31 to 5.45)	0.64 (0.29 to 1.12)	-86.8 (-93.9 to -76.6)
		Staphylococcus aureus	5400 (4640 to 6310)	7120 (6000 to 8410)	32.2 (16.4 to 49.3)	52.7 (47.4 to 58)	72.9 (63.1 to 84)	38.6 (17.6 to 64.1)
		Streptococcus pneumoniae	54900 (47400 to 64100)	30300 (25400 to 35800)	-44.8 (-51.8 to -37.6)	223 (204 to 244)	129 (112 to 149)	-42.2 (-51.0 to -31.5)
		Other viruses	20600 (17600 to 24000)	18900 (15900 to 22400)	-7.7 (-19.2 to 4.2)	32.9 (29.8 to 36.1)	31.7 (27.4 to 36.7)	-3.3 (-18.1 to 14.2)
		Other bacterial pathogens	6350 (5260 to 7530)	4760 (3910 to 5690)	-24.9 (-34.9 to -14.5)	24.6 (21.6 to 27.8)	19.3 (16.2 to 22.7)	-21.2 (-34.1 to -5.9)
	15-49 years	*Acinetobacter baumannii*	1620 (1360 to 1910)	1750 (1510 to 2030)	8.3 (-3.1 to 21.1)	14.8 (12.8 to 17.1)	14.5 (11.9 to 17.4)	-1.9 (-18.3 to 16.6)
		Chlamydia spp.	6050 (5230 to 6910)	6940 (6130 to 7860)	15.0 (3.3 to 27.5)	13.7 (12.3 to 15.3)	14.3 (11.9 to 16.9)	4.2 (-12.6 to 23.9)

		Enterobacter spp.	438 (361 to 524)	979 (834 to 1130)	124.6 (99.1 to 149.2)	3.79 (3.19 to 4.42)	7.68 (6.29 to 9.26)	103.5 (69.0 to 142.5)
		Escherichia coli	1260 (1090 to 1460)	2420 (2150 to 2730)	92.4 (73.7 to 115.0)	11.3 (10.1 to 12.7)	19.7 (16.6 to 23.2)	74.4 (44.5 to 108.0)
		Fungus	1530 (1290 to 1820)	2720 (2350 to 3110)	78.4 (60.0 to 99.5)	13.5 (11.6 to 15.4)	21.7 (18 to 25.8)	61.7 (35.4 to 92.1)
		Group B Streptococcus	1500 (1300 to 1790)	2980 (2630 to 3370)	98.9 (75.6 to 122.4)	9.56 (8.39 to 10.9)	17.2 (14.3 to 20.4)	80.2 (50.3 to 114.6)
		Haemophilus influenzae	5720 (5050 to 6460)	8270 (7430 to 9160)	44.7 (30.3 to 58.7)	19.5 (17.8 to 21.4)	17.3 (14.7 to 20.1)	-11.3 (-25.9 to 4.5)
		Influenza	8340 (7510 to 9370)	1840 (889 to 3220)	-77.9 (-89.3 to -61.7)	55.7 (51.9 to 59.6)	11.1 (4.99 to 20.2)	-80.0 (-91.0 to -62.9)
		Klebsiella pneumoniae	3720 (3300 to 4170)	5600 (5060 to 6190)	50.6 (36.6 to 64.9)	33.1 (30.6 to 35.5)	45.1 (38.5 to 53)	36.5 (15.2 to 61.1)
		Legionella spp.	2420 (2080 to 2780)	4650 (4140 to 5220)	92.9 (71.8 to 115.7)	14.8 (13 to 16.7)	25.8 (21.9 to 30.1)	74.8 (46.0 to 108.4)
		Mycoplasma spp.	13000 (11500 to 14600)	18700 (16900 to 20600)	44.0 (30.6 to 59.0)	30.7 (28.1 to 33.3)	40.1 (34 to 47)	30.5 (10.0 to 53.6)
		Polymicrobial	247 (166 to 368)	411 (261 to 624)	66.2 (46.0 to 87.6)	2.24 (1.52 to 3.31)	3.38 (2.15 to 5.18)	50.6 (25.6 to 82.5)
		Pseudomonas aeruginosa	2770 (2430 to 3160)	6160 (5550 to 6810)	122.8 (100.9 to 146.3)	17.1 (15.6 to 18.7)	34.5 (29.2 to 40.2)	101.9 (70.8 to 141.6)
		Respiratory syncytial virus	1820 (1600 to 2060)	409 (195 to 709)	-77.5 (-89.0 to -61.0)	5.55 (5.06 to 6.08)	1.13 (0.5 to 2.06)	-79.6 (-91.0 to -62.8)
		Staphylococcus aureus	4770 (4260 to 5340)	11200 (10200 to 12400)	136.0 (115.0 to 158.7)	60.3 (56.2 to 64.2)	129 (110 to 151)	113.8 (80.6 to 151.9)
		Streptococcus pneumoniae	48500 (43700 to 54100)	47700 (43600 to 52200)	-1.5 (-10.6 to 7.9)	255 (241 to 270)	228 (196 to 265)	-10.8 (-24.4 to 5.2)
		Other viruses	18200 (16400 to 20200)	29900 (27300 to 32800)	64.7 (49.1 to 81.1)	37.6 (35.3 to 39.9)	56 (47.8 to 65.3)	49.2 (26.5 to 75.3)
		Other bacterial pathogens	5620 (4880 to 6410)	7510 (6620 to 8530)	33.9 (20.9 to 49.3)	28.1 (25.4 to 31.2)	34.2 (28.7 to 40.3)	21.5 (2.6 to 44.7)
	50-69 years	*Acinetobacter baumannii*	1760 (1500 to 2090)	3010 (2560 to 3540)	71.1 (51.9 to 90.5)	19.5 (17.1 to 22.2)	22.3 (18 to 27.2)	14.5 (-6.9 to 37.2)
		Chlamydia spp.	3890 (3400 to 4460)	6850 (6040 to 7840)	76.5 (58.5 to 96.5)	10.7 (9.63 to 11.7)	12.6 (10.2 to 15.2)	18.1 (-3.6 to 41.7)
		Enterobacter spp.	462 (387 to 553)	1610 (1380 to 1900)	250.1 (210.2 to 293.4)	4.84 (4.07 to 5.69)	11.3 (9.05 to 13.9)	134.3 (87.3 to 185.0)
		Escherichia coli	1470 (1270 to 1690)	4340 (3850 to 4980)	195.4 (164.1 to 229.4)	15.9 (14.2 to 17.8)	31.5 (25.7 to 37.9)	97.7 (60.6 to 138.5)
		Fungus	1420 (1200 to 1660)	3940 (3400 to 4590)	179.1 (148.2 to 211.3)	15.1 (13.2 to 17.3)	28.2 (22.6 to 34.3)	86.8 (51.2 to 123.6)
		Group B Streptococcus	1120 (964 to 1310)	3440 (2970 to 3940)	206.8 (172.0 to 243.3)	8.64 (7.65 to 9.77)	17.7 (14.3 to 21.4)	105.3 (64.9 to 150.6)
		Haemophilus influenzae	3650 (3260 to 4170)	8550 (7590 to 9710)	134.6 (111.6 to 159.3)	15.1 (13.9 to 16.4)	16 (13.1 to 19)	6.2 (-13.6 to 28.4)
		Influenza	4580 (4140 to 5160)	1600 (770 to 2780)	-65.0 (-83.0 to -39.2)	37.1 (34.6 to 39.8)	8.68 (3.75 to 16.1)	-76.6 (-89.7 to -57.5)

		Klebsiella pneumoniae	3040 (2730 to 3410)	7600 (6770 to 8610)	150.4 (126.0 to 175.7)	32.7 (30.6 to 34.9)	54.8 (44.9 to 65.1)	67.5 (37.0 to 99.9)
		Legionella spp.	2160 (1870 to 2500)	6490 (5730 to 7360)	200.6 (166.7 to 237.7)	16.1 (14.3 to 18)	32.2 (26.2 to 38.8)	101.1 (63.7 to 144.2)
		Mycoplasma spp.	3800 (3390 to 4340)	8090 (7210 to 9190)	113.3 (92.0 to 135.8)	10.9 (9.94 to 11.9)	15.5 (12.8 to 18.7)	42.8 (16.7 to 71.6)
		Polymicrobial	224 (152 to 327)	585 (382 to 899)	160.9 (129.5 to 194.9)	2.46 (1.68 to 3.58)	4.3 (2.68 to 6.78)	74.6 (41.3 to 113.5)
		Pseudomonas aeruginosa	2500 (2230 to 2830)	8530 (7580 to 9610)	240.9 (205.4 to 278.8)	18.7 (17.3 to 20.3)	42.7 (34.9 to 51)	128.2 (86.0 to 173.6)
		Respiratory syncytial virus	740 (649 to 848)	256 (119 to 448)	-65.4 (-83.2 to -39.6)	2.73 (2.45 to 3.03)	0.63 (0.27 to 1.15)	-76.9 (-89.9 to -57.6)
		Staphylococcus aureus	3790 (3430 to 4260)	14000 (12600 to 15800)	270.1 (234.6 to 309.0)	58 (54.6 to 61.7)	144 (118 to 171)	147.7 (101.0 to 195.2)
		Streptococcus pneumoniae	19000 (17300 to 21300)	30400 (27300 to 34200)	59.6 (44.5 to 75.5)	121 (115 to 128)	130 (106 to 154)	6.8 (-12.8 to 27.3)
		Other viruses	6240 (5630 to 6990)	17000 (15200 to 19100)	173.0 (146.3 to 202.0)	15.6 (14.8 to 16.5)	28.5 (23.4 to 34.1)	82.7 (48.8 to 117.8)
		Other bacterial pathogens	5400 (4780 to 6180)	11300 (9750 to 13100)	108.4 (88.4 to 132.0)	32.8 (29.8 to 36)	45.8 (37.4 to 55.8)	39.6 (13.6 to 67.6)
	70+ years	*Acinetobacter baumannii*	1390 (1160 to 1650)	1590 (1350 to 1870)	14.7 (-0.7 to 30.8)	15.3 (13.1 to 17.7)	15.8 (13.2 to 19.1)	3.9 (-13.4 to 22.3)
		Chlamydia spp.	3660 (3130 to 4220)	4320 (3780 to 4990)	18.3 (3.0 to 32.7)	9.98 (8.79 to 11.2)	10.7 (8.85 to 12.7)	7.2 (-10.0 to 26.9)
		Enterobacter spp.	355 (288 to 433)	831 (693 to 989)	134.8 (102.5 to 167.6)	3.7 (3.08 to 4.47)	7.84 (6.34 to 9.5)	112.6 (75.4 to 153.3)
		Escherichia coli	1430 (1220 to 1670)	2820 (2460 to 3230)	98.1 (71.9 to 121.2)	15.4 (13.4 to 17.4)	27.5 (22.8 to 32.6)	79.4 (49.7 to 112.1)
		Fungus	623 (515 to 745)	1160 (989 to 1380)	87.2 (62.8 to 111.0)	6.61 (5.63 to 7.73)	11.2 (9.21 to 13.6)	69.5 (41.0 to 100.3)
		Group B Streptococcus	813 (690 to 952)	1670 (1460 to 1910)	105.7 (77.9 to 133.3)	6.22 (5.33 to 7.15)	11.6 (9.62 to 13.6)	86.3 (55.6 to 122.6)
		Haemophilus influenzae	2590 (2260 to 2960)	4070 (3610 to 4650)	57.3 (37.6 to 74.8)	10.7 (9.54 to 11.8)	10.3 (8.65 to 12)	-3.6 (-19.3 to 14.0)
		Influenza	5500 (4810 to 6210)	1290 (613 to 2240)	-76.5 (-88.6 to -59.5)	44.2 (40.1 to 48.2)	9.39 (4.15 to 17.2)	-78.7 (-90.6 to -60.9)
		Klebsiella pneumoniae	2640 (2330 to 3010)	4430 (3940 to 5050)	67.9 (47.3 to 87.2)	28.3 (25.7 to 30.9)	43 (36.2 to 50.5)	52.0 (27.5 to 79.2)
		Legionella spp.	1230 (1040 to 1430)	2470 (2170 to 2820)	101.5 (74.2 to 126.5)	9.06 (7.88 to 10.3)	16.5 (13.7 to 19.3)	82.5 (51.7 to 119.0)
		Mycoplasma spp.	2540 (2200 to 2910)	3620 (3220 to 4140)	43.0 (25.8 to 60.1)	7.22 (6.44 to 8)	9.33 (7.77 to 10.9)	29.5 (8.9 to 52.7)
		Polymicrobial	202 (133 to 310)	354 (224 to 548)	75.0 (50.7 to 100.3)	2.21 (1.48 to 3.3)	3.5 (2.16 to 5.46)	58.4 (31.7 to 89.4)
		Pseudomonas aeruginosa	2060 (1800 to 2360)	4690 (4210 to 5380)	128.6 (99.5 to 156.7)	15.3 (13.7 to 16.8)	31.6 (26.6 to 37.1)	107.0 (72.9 to 144.6)
		Respiratory syncytial virus	548 (473 to 634)	127 (60.9 to 218)	-76.8 (-88.8 to -59.4)	2.01 (1.77 to 2.26)	0.42 (0.19 to 0.77)	-79.0 (-90.7 to -61.2)

		Staphylococcus aureus	3870 (3420 to 4380)	9590 (8550 to 10900)	148.2 (117.0 to 175.7)	58.9 (53.2 to 63.8)	132 (111 to 154)	124.7 (88.9 to 163.7)
		Streptococcus pneumoniae	15500 (13700 to 17300)	16500 (14800 to 18800)	7.0 (-5.8 to 18.5)	98 (89.1 to 106)	94.8 (79.8 to 111)	-3.1 (-18.3 to 13.7)
		Other viruses	5010 (4430 to 5650)	9150 (8150 to 10400)	83.0 (60.8 to 102.3)	12.5 (11.2 to 13.5)	20.6 (17.2 to 24)	65.7 (39.3 to 94.9)
		Other bacterial pathogens	5710 (4940 to 6580)	7960 (6950 to 9250)	39.7 (21.5 to 56.8)	34.8 (30.8 to 38.8)	44.1 (36.6 to 52.2)	26.7 (6.4 to 48.8)
Central Europe	All Ages	*Acinetobacter baumannii*	24400 (21500 to 27500)	12100 (10700 to 13700)	-50.3 (-53.9 to -46.6)	604 (535 to 679)	580 (506 to 659)	-3.8 (-13.1 to 5.9)
		Chlamydia spp.	91600 (84400 to 99500)	42200 (39400 to 45100)	-53.9 (-56.8 to -50.8)	550 (512 to 588)	464 (417 to 503)	-15.7 (-22.9 to -8.1)
		Enterobacter spp.	11300 (9950 to 12800)	12600 (11400 to 13900)	11.6 (2.2 to 21.5)	267 (234 to 302)	542 (474 to 603)	103.3 (81.4 to 126.5)
		Escherichia coli	34800 (31600 to 38100)	31000 (29100 to 33200)	-10.8 (-16.7 to -4.2)	892 (820 to 965)	1420 (1280 to 1530)	58.8 (44.0 to 73.9)
		Fungus	24300 (21500 to 27400)	19400 (17800 to 21200)	-20.0 (-25.8 to -13.5)	480 (432 to 534)	776 (700 to 850)	61.7 (46.2 to 77.6)
		Group B Streptococcus	28500 (25800 to 31600)	25400 (23800 to 27300)	-10.6 (-17.4 to -3.6)	482 (443 to 525)	760 (684 to 824)	58.1 (42.9 to 73.8)
		Haemophilus influenzae	124000 (116000 to 133000)	70800 (66900 to 75100)	-42.9 (-46.0 to -39.8)	1070 (1010 to 1140)	715 (653 to 767)	-33.3 (-39.0 to -27.7)
		Influenza	166000 (155000 to 175000)	2770 (927 to 6730)	-98.3 (-99.4 to -96.0)	2950 (2810 to 3070)	94.8 (28.3 to 241)	-96.8 (-99.0 to -91.7)
		Klebsiella pneumoniae	81600 (76700 to 86900)	56600 (53400 to 60100)	-30.6 (-34.2 to -27.3)	1930 (1840 to 2010)	2490 (2250 to 2680)	28.8 (18.5 to 38.5)
		Legionella spp.	53800 (48900 to 59100)	55400 (51800 to 58900)	3.1 (-4.6 to 11.4)	802 (732 to 880)	1620 (1470 to 1750)	102.3 (83.3 to 121.8)
		Mycoplasma spp.	143000 (132000 to 155000)	82300 (77900 to 86800)	-42.5 (-45.7 to -39.3)	708 (667 to 750)	741 (680 to 796)	4.7 (-4.6 to 13.5)
		Polymicrobial	4850 (3320 to 7110)	3420 (2140 to 5440)	-29.9 (-38.3 to -21.1)	147 (105 to 207)	158 (98.7 to 254)	6.8 (-18.4 to 32.0)
		Pseudomonas aeruginosa	75000 (70000 to 80800)	91100 (86000 to 96700)	21.5 (14.8 to 28.2)	1240 (1170 to 1310)	2770 (2520 to 2970)	123.2 (104.0 to 142.3)
		Respiratory syncytial virus	98700 (89500 to 109000)	1320 (407 to 3380)	-98.7 (-99.6 to -96.7)	841 (796 to 886)	9.45 (2.88 to 23.5)	-98.9 (-99.7 to -97.2)
		Staphylococcus aureus	112000 (105000 to 118000)	136000 (128000 to 145000)	22.0 (17.6 to 26.9)	3810 (3630 to 3960)	8810 (7940 to 9450)	131.1 (114.1 to 147.3)
		Streptococcus pneumoniae	739000 (697000 to 787000)	297000 (282000 to 313000)	-59.8 (-61.4 to -58.2)	9100 (8770 to 9380)	6820 (6220 to 7300)	-25.0 (-30.9 to -19.7)
		Other viruses	384000 (359000 to 411000)	254000 (241000 to 268000)	-33.8 (-36.8 to -30.8)	1880 (1810 to 1960)	2090 (1910 to 2240)	11.4 (2.4 to 19.3)
		Other bacterial pathogens	121000 (109000 to 134000)	80600 (71700 to 90200)	-33.5 (-37.2 to -29.8)	1700 (1540 to 1860)	2190 (1900 to 2460)	28.3 (17.4 to 39.1)
	Under 5	*Acinetobacter baumannii*	1110 (774 to 1540)	225 (151 to 333)	-79.7 (-82.5 to -76.7)	56.1 (35.4 to 86.6)	5.67 (3.14 to 9.89)	-90.0 (-92.3 to -87.5)
		Chlamydia spp.	13500 (11700 to 15600)	2760 (2390 to 3160)	-79.5 (-81.8 to -77.0)	108 (97.5 to 118)	8.13 (6.86 to 9.38)	-92.4 (-93.7 to -91.1)

		Enterobacter spp.	1420 (1110 to 1800)	640 (500 to 816)	-54.8 (-61.0 to -48.2)	44.8 (34.6 to 58.7)	6.71 (4.88 to 9.15)	-85.0 (-87.9 to -81.9)
		Escherichia coli	4810 (4100 to 5660)	1910 (1630 to 2220)	-60.1 (-65.5 to -54.7)	150 (133 to 169)	19.4 (16.3 to 22.6)	-87.1 (-89.3 to -84.6)
		Fungus	914 (642 to 1250)	309 (222 to 415)	-66.1 (-70.9 to -60.9)	21.8 (15.6 to 29.3)	2.03 (1.44 to 2.76)	-90.6 (-92.6 to -88.5)
		Group B Streptococcus	4270 (3590 to 5000)	1680 (1430 to 1970)	-60.7 (-65.4 to -55.9)	109 (96.5 to 122)	14.5 (12.2 to 16.8)	-86.7 (-89.0 to -84.3)
		Haemophilus influenzae	36300 (31600 to 41800)	8870 (7690 to 10200)	-75.5 (-78.3 to -72.2)	368 (336 to 408)	17.7 (14.9 to 20.5)	-95.2 (-96.1 to -94.2)
		Influenza	30300 (26800 to 34000)	183 (55.8 to 455)	-99.4 (-99.8 to -98.5)	586 (543 to 630)	0.8 (0.31 to 1.66)	-99.9 (-99.9 to -99.7)
		Klebsiella pneumoniae	17600 (15500 to 19900)	3960 (3460 to 4550)	-77.4 (-79.7 to -74.9)	469 (431 to 507)	33.6 (28.3 to 38.8)	-92.8 (-94.0 to -91.5)
		Legionella spp.	1740 (1270 to 2350)	754 (548 to 1020)	-56.5 (-61.3 to -50.9)	45.8 (34.7 to 58.7)	7.54 (5.67 to 9.6)	-83.5 (-86.5 to -80.0)
		Mycoplasma spp.	20700 (18100 to 23900)	9010 (7780 to 10300)	-56.4 (-61.3 to -51.3)	145 (131 to 159)	18.6 (15.6 to 21.6)	-87.1 (-89.4 to -84.7)
		Polymicrobial	827 (505 to 1290)	286 (163 to 457)	-65.5 (-69.9 to -60.2)	51.5 (29.5 to 82.2)	9.07 (4.56 to 15.2)	-82.5 (-86.4 to -78.1)
		Pseudomonas aeruginosa	10100 (8830 to 11600)	6290 (5460 to 7190)	-37.8 (-44.9 to -30.4)	202 (183 to 220)	36.5 (30.9 to 42.2)	-81.9 (-85.2 to -78.5)
		Respiratory syncytial virus	72500 (64500 to 81600)	785 (233 to 2060)	-98.9 (-99.7 to -97.2)	668 (626 to 708)	1.59 (0.61 to 3.31)	-99.8 (-99.9 to -99.5)
		Staphylococcus aureus	12500 (11100 to 14200)	5940 (5160 to 6810)	-52.5 (-56.6 to -48.4)	460 (427 to 492)	63.5 (53.6 to 73.4)	-86.2 (-88.5 to -83.7)
		Streptococcus pneumoniae	198000 (176000 to 223000)	28300 (24700 to 32400)	-85.7 (-86.8 to -84.5)	2850 (2680 to 3020)	114 (96 to 132)	-96.0 (-96.7 to -95.3)
		Other viruses	149000 (132000 to 167000)	42900 (37600 to 49000)	-71.2 (-73.5 to -68.7)	840 (794 to 893)	72.2 (61.1 to 83.7)	-91.4 (-92.9 to -89.9)
		Other bacterial pathogens	5330 (4250 to 6580)	1400 (1120 to 1730)	-73.6 (-76.9 to -70.1)	90.1 (73.4 to 110)	7.96 (5.89 to 10.5)	-91.2 (-92.9 to -89.4)
	5-14 years	*Acinetobacter baumannii*	2530 (2000 to 3170)	506 (401 to 632)	-80.0 (-81.9 to -78.0)	8.21 (7 to 9.42)	1.06 (0.92 to 1.21)	-87.1 (-88.4 to -85.7)
		Chlamydia spp.	11500 (9260 to 14400)	2460 (1970 to 3050)	-78.5 (-80.4 to -76.6)	9.17 (8.18 to 10.2)	1.31 (1.19 to 1.44)	-85.7 (-87.1 to -84.1)
		Enterobacter spp.	1060 (831 to 1350)	538 (421 to 685)	-49.4 (-54.9 to -44.1)	3.21 (2.78 to 3.71)	1.04 (0.93 to 1.18)	-67.4 (-71.2 to -63.2)
		Escherichia coli	2750 (2190 to 3410)	1070 (857 to 1330)	-61.0 (-64.7 to -57.4)	8.61 (7.75 to 9.45)	2.19 (2 to 2.38)	-74.5 (-77.0 to -71.8)
		Fungus	3310 (2630 to 4170)	1230 (984 to 1550)	-62.8 (-66.4 to -59.0)	10.2 (8.99 to 11.6)	2.47 (2.21 to 2.76)	-75.8 (-78.3 to -73.1)
		Group B Streptococcus	3450 (2750 to 4300)	1490 (1190 to 1860)	-56.8 (-61.0 to -52.7)	7.66 (6.8 to 8.61)	2.14 (1.93 to 2.37)	-72.0 (-74.9 to -68.9)
		Haemophilus influenzae	14000 (11400 to 17200)	4300 (3480 to 5340)	-69.2 (-71.6 to -66.8)	16.6 (15.2 to 17.8)	2.37 (2.18 to 2.55)	-85.7 (-86.9 to -84.3)
		Influenza	19800 (16400 to 24100)	155 (49 to 379)	-99.2 (-99.7 to -98.1)	46.1 (42.9 to 48.8)	0.21 (0.08 to 0.48)	-99.5 (-99.8 to -99.0)

		Klebsiella pneumoniae	8070 (6600 to 9880)	2620 (2130 to 3240)	-67.6 (-69.8 to -65.1)	25.1 (23.4 to 26.7)	5.33 (4.94 to 5.69)	-78.8 (-80.5 to -76.8)
		Legionella spp.	6890 (5630 to 8570)	3260 (2640 to 4000)	-52.6 (-57.1 to -47.9)	14.6 (13.1 to 16.2)	4.53 (4.17 to 4.88)	-69.0 (-72.1 to -65.7)
		Mycoplasma spp.	30900 (25200 to 37600)	9770 (7850 to 12200)	-68.4 (-70.9 to -66.0)	25.5 (23.4 to 27.2)	5.31 (4.95 to 5.68)	-79.1 (-81.1 to -76.9)
		Polymicrobial	467 (297 to 705)	147 (86.8 to 235)	-68.8 (-72.6 to -64.4)	1.5 (0.98 to 2.25)	0.31 (0.18 to 0.5)	-79.6 (-82.2 to -76.8)
		Pseudomonas aeruginosa	7580 (6140 to 9250)	4250 (3450 to 5290)	-43.9 (-48.4 to -39.2)	16.2 (15 to 17.5)	5.84 (5.42 to 6.24)	-64.0 (-67.3 to -60.6)
		Respiratory syncytial virus	5340 (4360 to 6540)	53.3 (16.7 to 134)	-99.0 (-99.7 to -97.6)	5.62 (5.15 to 6.1)	0.03 (0.01 to 0.07)	-99.4 (-99.8 to -98.7)
		Staphylococcus aureus	11800 (9740 to 14400)	6370 (5150 to 7870)	-46.1 (-49.6 to -42.3)	52 (48.5 to 54.9)	18 (16.8 to 19.2)	-65.3 (-68.1 to -62.2)
		Streptococcus pneumoniae	112000 (92400 to 136000)	25500 (20800 to 31700)	-77.3 (-78.8 to -75.7)	206 (193 to 216)	31.1 (29.1 to 33)	-84.9 (-86.0 to -83.6)
		Other viruses	51600 (42300 to 62700)	21700 (17600 to 27000)	-57.9 (-60.6 to -55.0)	37 (34.5 to 38.9)	10.2 (9.52 to 10.8)	-72.3 (-74.5 to -69.9)
		Other bacterial pathogens	11900 (9560 to 14800)	3240 (2550 to 4060)	-72.9 (-75.2 to -70.7)	21 (18.9 to 23.3)	3.78 (3.34 to 4.25)	-82.0 (-83.6 to -80.2)
	15-49 years	*Acinetobacter baumannii*	2720 (2300 to 3190)	886 (755 to 1030)	-67.4 (-69.9 to -64.8)	41.6 (35.9 to 47.8)	21.6 (18.6 to 24.9)	-47.9 (-53.0 to -42.5)
		Chlamydia spp.	12500 (11100 to 14100)	4320 (3870 to 4800)	-65.4 (-67.5 to -63.0)	47.4 (43.4 to 52.2)	26.1 (23.6 to 28.8)	-44.9 (-49.6 to -39.6)
		Enterobacter spp.	1170 (998 to 1370)	934 (808 to 1070)	-20.0 (-27.3 to -12.4)	17 (14.9 to 19.5)	21.1 (18.5 to 24)	24.4 (9.8 to 39.3)
		Escherichia coli	3000 (2660 to 3380)	1860 (1690 to 2060)	-37.9 (-42.4 to -33.4)	45.2 (41.2 to 49.3)	43.7 (39.9 to 47.6)	-3.2 (-11.9 to 7.0)
		Fungus	3620 (3130 to 4150)	2140 (1900 to 2410)	-40.6 (-45.3 to -35.8)	53.7 (47.5 to 59.9)	49.9 (44.6 to 55.6)	-6.9 (-16.0 to 2.5)
		Group B Streptococcus	3780 (3320 to 4380)	2590 (2320 to 2900)	-31.4 (-37.0 to -26.0)	40.4 (36.2 to 45.5)	43.2 (39.2 to 47.7)	7.1 (-3.7 to 18.7)
		Haemophilus influenzae	15300 (13800 to 16900)	7520 (6830 to 8250)	-51.0 (-53.6 to -48.5)	88.1 (82.3 to 93.9)	46.7 (43.2 to 50.6)	-46.9 (-51.3 to -42.3)
		Influenza	21700 (19700 to 23900)	269 (92.5 to 638)	-98.8 (-99.6 to -97.1)	244 (232 to 256)	4.63 (1.38 to 11.5)	-98.1 (-99.4 to -95.3)
		Klebsiella pneumoniae	8820 (7990 to 9680)	4570 (4150 to 5000)	-48.1 (-50.5 to -45.8)	132 (125 to 139)	108 (100 to 117)	-17.9 (-24.3 to -11.0)
		Legionella spp.	7630 (6720 to 8610)	5670 (5160 to 6210)	-25.6 (-31.6 to -19.8)	78.8 (71.5 to 87.2)	91.4 (84.2 to 99)	16.1 (4.7 to 27.9)
		Mycoplasma spp.	34000 (30800 to 37400)	17000 (15500 to 18600)	-49.8 (-52.6 to -47.0)	135 (127 to 144)	107 (98.2 to 115)	-20.9 (-28.0 to -13.3)
		Polymicrobial	508 (325 to 770)	257 (153 to 415)	-49.8 (-55.1 to -44.1)	7.74 (5.05 to 11.8)	6.14 (3.67 to 9.93)	-21.0 (-30.6 to -10.3)
		Pseudomonas aeruginosa	8380 (7500 to 9290)	7380 (6710 to 8100)	-11.9 (-16.8 to -6.6)	87.3 (81.4 to 93.3)	119 (110 to 128)	36.9 (24.3 to 49.8)
		Respiratory syncytial virus	5920 (5270 to 6600)	92 (30.1 to 224)	-98.4 (-99.5 to -96.2)	30.4 (28.1 to 32.8)	0.73 (0.22 to 1.87)	-97.6 (-99.3 to -93.9)

		Staphylococcus aureus	13000 (11800 to 14200)	11100 (10100 to 12100)	-14.9 (-18.3 to -11.7)	277 (264 to 288)	365 (338 to 393)	32.0 (21.4 to 42.2)
		Streptococcus pneumoniae	123000 (112000 to 134000)	44700 (40900 to 48500)	-63.6 (-64.9 to -62.4)	1090 (1050 to 1120)	633 (590 to 678)	-41.8 (-46.2 to -37.5)
		Other viruses	57200 (52200 to 62300)	37900 (34600 to 41200)	-33.7 (-36.1 to -31.5)	199 (192 to 208)	208 (193 to 223)	4.4 (-3.9 to 12.7)
		Other bacterial pathogens	13000 (11400 to 14700)	5660 (4930 to 6470)	-56.6 (-59.0 to -54.0)	110 (99.4 to 121)	75.6 (66.5 to 84.9)	-31.2 (-37.3 to -24.4)
	50-69 years	*Acinetobacter baumannii*	9450 (8150 to 10800)	4170 (3630 to 4720)	-55.9 (-59.0 to -52.6)	163 (143 to 185)	143 (125 to 163)	-12.2 (-21.1 to -3.2)
		Chlamydia spp.	25800 (23200 to 28900)	11700 (10600 to 12800)	-54.8 (-57.6 to -51.8)	111 (101 to 121)	98 (87.8 to 108)	-11.5 (-20.0 to -2.5)
		Enterobacter spp.	4020 (3480 to 4620)	4200 (3750 to 4690)	4.8 (-3.9 to 14.0)	66.1 (57.5 to 75.3)	133 (117 to 149)	101.4 (77.0 to 127.5)
		Escherichia coli	11300 (10100 to 12600)	9090 (8340 to 9870)	-19.7 (-25.2 to -13.7)	193 (175 to 209)	297 (270 to 323)	54.4 (39.8 to 70.8)
		Fungus	10900 (9540 to 12300)	8500 (7660 to 9450)	-21.7 (-27.3 to -15.5)	182 (163 to 203)	276 (247 to 306)	51.7 (36.2 to 68.2)
		Group B Streptococcus	9170 (8110 to 10400)	8160 (7420 to 8970)	-10.8 (-17.9 to -4.3)	111 (100 to 124)	190 (171 to 209)	72.3 (53.9 to 91.9)
		Haemophilus influenzae	31800 (29100 to 34500)	21100 (19500 to 22800)	-33.5 (-36.6 to -30.1)	206 (195 to 220)	181 (167 to 197)	-12.0 (-20.0 to -3.3)
		Influenza	38800 (35700 to 41900)	641 (215 to 1550)	-98.3 (-99.4 to -96.1)	492 (466 to 518)	15.6 (4.44 to 39.8)	-96.8 (-99.1 to -91.8)
		Klebsiella pneumoniae	23300 (21500 to 25200)	16900 (15700 to 18200)	-27.5 (-31.0 to -24.1)	394 (373 to 415)	556 (512 to 601)	41.4 (29.3 to 54.3)
		Legionella spp.	22400 (20000 to 25000)	21700 (20000 to 23400)	-3.1 (-10.5 to 4.7)	262 (237 to 287)	487 (446 to 530)	86.7 (66.9 to 107.3)
		Mycoplasma spp.	32200 (29400 to 35000)	20300 (18700 to 21900)	-37.0 (-40.5 to -33.4)	145 (135 to 156)	178 (163 to 194)	22.8 (10.8 to 34.6)
		Polymicrobial	1480 (964 to 2260)	995 (611 to 1620)	-33.2 (-40.2 to -25.5)	25.4 (16.7 to 38.7)	33.1 (20.5 to 54)	29.5 (13.7 to 48.2)
		Pseudomonas aeruginosa	24800 (22600 to 27100)	28000 (25900 to 30300)	13.1 (6.7 to 19.8)	291 (271 to 311)	637 (586 to 688)	118.8 (98.0 to 141.5)
		Respiratory syncytial virus	7890 (7080 to 8770)	158 (49.9 to 387)	-98.0 (-99.4 to -95.1)	45.8 (41.6 to 50.3)	1.79 (0.5 to 4.68)	-96.1 (-99.0 to -89.6)
		Staphylococcus aureus	33600 (30900 to 36100)	37600 (34900 to 40400)	11.9 (8.2 to 16.7)	809 (772 to 846)	1740 (1590 to 1870)	114.7 (97.4 to 134.6)
		Streptococcus pneumoniae	156000 (145000 to 167000)	77400 (71800 to 83200)	-50.6 (-52.0 to -48.6)	1560 (1500 to 1630)	1530 (1410 to 1640)	-2.3 (-10.4 to 6.3)
		Other viruses	64300 (59400 to 68900)	58800 (54700 to 63200)	-8.5 (-11.8 to -4.7)	254 (242 to 266)	451 (415 to 488)	77.8 (63.6 to 93.0)
		Other bacterial pathogens	40600 (35900 to 45200)	23200 (20400 to 26100)	-42.9 (-46.0 to -39.6)	387 (349 to 424)	431 (375 to 487)	11.5 (1.7 to 22.6)
	70+ years	*Acinetobacter baumannii*	8610 (7380 to 9980)	6330 (5470 to 7250)	-26.4 (-31.6 to -20.9)	335 (292 to 380)	409 (349 to 468)	22.3 (10.6 to 34.8)
		Chlamydia spp.	28300 (25200 to 31600)	21000 (19100 to 23200)	-25.9 (-30.8 to -20.5)	275 (248 to 299)	330 (290 to 362)	20.1 (9.1 to 31.8)

		Enterobacter spp.	3650 (3120 to 4270)	6300 (5500 to 7070)	73.1 (57.7 to 89.1)	136 (117 to 157)	380 (326 to 427)	180.3 (149.3 to 213.7)
		Escherichia coli	12900 (11400 to 14500)	17000 (15600 to 18800)	32.3 (23.3 to 42.3)	496 (449 to 544)	1050 (932 to 1140)	112.7 (92.0 to 132.9)
		Fungus	5610 (4840 to 6470)	7250 (6470 to 8180)	29.4 (19.6 to 39.8)	213 (187 to 241)	446 (391 to 499)	109.9 (89.9 to 131.2)
		Group B Streptococcus	7820 (6920 to 8870)	11500 (10400 to 12700)	47.3 (35.4 to 59.0)	214 (191 to 239)	510 (447 to 559)	139.1 (115.5 to 163.8)
		Haemophilus influenzae	26700 (24300 to 29500)	29000 (26600 to 31700)	8.5 (2.5 to 14.4)	393 (363 to 420)	467 (414 to 504)	18.7 (8.9 to 28.6)
		Influenza	54900 (50000 to 60100)	1530 (515 to 3730)	-97.2 (-99.1 to -93.4)	1580 (1470 to 1670)	73.5 (21.6 to 189)	-95.3 (-98.6 to -88.0)
		Klebsiella pneumoniae	23800 (21800 to 26100)	28500 (26300 to 31100)	19.8 (13.7 to 25.9)	910 (844 to 962)	1780 (1590 to 1930)	95.9 (80.4 to 111.4)
		Legionella spp.	15100 (13400 to 17000)	24000 (22000 to 26400)	58.9 (46.4 to 71.6)	401 (361 to 444)	1030 (909 to 1120)	157.0 (131.3 to 183.8)
		Mycoplasma spp.	25400 (23000 to 28300)	26200 (24100 to 28900)	3.2 (-2.8 to 9.4)	258 (236 to 279)	433 (385 to 470)	67.6 (52.5 to 82.4)
		Polymicrobial	1560 (990 to 2460)	1740 (1020 to 2830)	10.5 (-1.6 to 23.1)	60.6 (38.9 to 95.1)	109 (65.4 to 181)	78.9 (56.7 to 103.1)
		Pseudomonas aeruginosa	24200 (22100 to 26800)	45200 (41500 to 49200)	87.1 (75.7 to 98.7)	646 (596 to 690)	1970 (1750 to 2130)	205.7 (178.5 to 234.5)
		Respiratory syncytial virus	6970 (6200 to 7850)	235 (75.8 to 585)	-96.6 (-98.9 to -91.7)	91.9 (83.3 to 101)	5.31 (1.49 to 14.2)	-94.2 (-98.4 to -84.6)
		Staphylococcus aureus	40600 (37100 to 44400)	75100 (69300 to 81700)	85.0 (77.5 to 92.9)	2210 (2060 to 2330)	6630 (5870 to 7130)	199.3 (176.5 to 221.4)
		Streptococcus pneumoniae	150000 (137000 to 164000)	121000 (112000 to 132000)	-18.9 (-21.9 to -15.8)	3390 (3180 to 3550)	4520 (4000 to 4860)	33.2 (23.5 to 42.6)
		Other viruses	61500 (56000 to 67100)	92500 (85600 to 101000)	50.6 (44.0 to 56.9)	550 (515 to 579)	1350 (1190 to 1460)	145.8 (127.9 to 165.1)
		Other bacterial pathogens	50300 (44500 to 57000)	47100 (41400 to 53700)	-6.3 (-11.8 to -1.2)	1100 (975 to 1210)	1670 (1440 to 1900)	52.1 (38.9 to 65.9)
Albania	All Ages	*Acinetobacter baumannii*	847 (733 to 974)	193 (168 to 220)	-77.2 (-79.6 to -74.6)	28.9 (23 to 36.2)	6.17 (4.77 to 8.3)	-78.4 (-84.1 to -68.9)
		Chlamydia spp.	3770 (3410 to 4160)	926 (843 to 1020)	-75.4 (-77.8 to -72.5)	31.6 (27 to 35.8)	6.89 (5.41 to 8.98)	-78.1 (-83.7 to -70.1)
		Enterobacter spp.	373 (316 to 435)	271 (244 to 302)	-27.0 (-36.6 to -17.7)	12.6 (9.87 to 15.6)	7.75 (5.99 to 10.1)	-37.8 (-55.6 to -11.2)
		Escherichia coli	1240 (1110 to 1380)	738 (676 to 810)	-40.3 (-46.6 to -34.1)	44.4 (37.7 to 50.8)	22.9 (18 to 29.7)	-48.0 (-61.4 to -27.6)
		Fungus	713 (618 to 823)	412 (367 to 459)	-42.1 (-48.8 to -34.6)	15.5 (12.8 to 18.4)	10 (7.67 to 12.9)	-34.8 (-52.0 to -9.3)
		Group B Streptococcus	1060 (946 to 1190)	550 (500 to 605)	-48.1 (-53.8 to -41.7)	27.9 (23.5 to 32.3)	11 (8.68 to 14.2)	-60.3 (-70.7 to -44.7)
		Haemophilus influenzae	5780 (5190 to 6420)	1770 (1630 to 1910)	-69.4 (-72.7 to -66.0)	73 (62 to 84.2)	11.4 (9.09 to 14.5)	-84.3 (-88.2 to -78.7)
		Influenza	6490 (5910 to 7080)	56.1 (21.3 to 119)	-99.1 (-99.7 to -98.2)	151 (130 to 170)	1.22 (0.44 to 2.74)	-99.2 (-99.7 to -98.2)

		Klebsiella pneumoniae	3480 (3160 to 3820)	1110 (1020 to 1210)	-68.0 (-71.2 to -64.7)	113 (97.2 to 127)	32.2 (25.5 to 41.8)	-71.2 (-78.4 to -60.6)
		Legionella spp.	1330 (1180 to 1500)	1130 (1030 to 1230)	-14.8 (-23.8 to -5.0)	24.4 (20.3 to 28.3)	20.8 (16.4 to 27.3)	-13.9 (-35.5 to 18.7)
		Mycoplasma spp.	5720 (5190 to 6320)	1860 (1720 to 2000)	-67.5 (-70.3 to -64.5)	37.6 (32.6 to 42.6)	10.5 (8.47 to 13.4)	-71.9 (-78.5 to -62.4)
		Polymicrobial	198 (142 to 279)	77.8 (49.4 to 121)	-60.9 (-69.5 to -51.6)	11.7 (7.41 to 17.6)	2.81 (1.71 to 4.52)	-75.4 (-84.8 to -62.1)
		Pseudomonas aeruginosa	2400 (2180 to 2630)	1750 (1600 to 1910)	-27.0 (-33.9 to -19.5)	54.4 (46.8 to 61.3)	34.6 (27.2 to 44.8)	-36.0 (-52.1 to -12.8)
		Respiratory syncytial virus	7100 (6230 to 7960)	27.4 (10.3 to 58.9)	-99.6 (-99.9 to -99.2)	98.8 (83.9 to 115)	0.18 (0.07 to 0.41)	-99.8 (-99.9 to -99.6)
		Staphylococcus aureus	3600 (3330 to 3870)	2930 (2690 to 3200)	-18.5 (-25.7 to -11.0)	153 (132 to 171)	126 (99.3 to 166)	-17.3 (-38.1 to 14.8)
		Streptococcus pneumoniae	35100 (31900 to 38300)	5080 (4700 to 5470)	-85.5 (-86.8 to -84.2)	589 (509 to 666)	75 (59.8 to 96.9)	-87.2 (-90.3 to -82.6)
		Other viruses	19500 (17500 to 21500)	5070 (4720 to 5430)	-73.9 (-76.4 to -71.6)	141 (121 to 161)	27.7 (22.4 to 35.2)	-80.3 (-85.1 to -73.4)
		Other bacterial pathogens	3540 (3170 to 3940)	1680 (1470 to 1920)	-52.5 (-57.3 to -47.7)	59.8 (49.3 to 68.6)	30.5 (23.2 to 40.6)	-48.6 (-61.8 to -27.6)
	Under 5	*Acinetobacter baumannii*	128 (89 to 179)	6.58 (4.27 to 10.2)	-94.9 (-95.9 to -93.7)	10.4 (6.32 to 16.1)	0.41 (0.18 to 0.84)	-96.0 (-97.8 to -93.4)
		Chlamydia spp.	1460 (1240 to 1700)	92.9 (79.6 to 108)	-93.6 (-94.5 to -92.6)	18 (14.9 to 21.4)	0.6 (0.39 to 0.87)	-96.6 (-97.9 to -94.9)
		Enterobacter spp.	138 (108 to 177)	21.8 (17 to 28)	-84.1 (-86.8 to -81.2)	6.96 (4.96 to 9.7)	0.51 (0.3 to 0.82)	-92.6 (-95.4 to -88.5)
		Escherichia coli	490 (414 to 581)	74 (62.4 to 86.9)	-84.8 (-87.2 to -82.3)	23.9 (19.4 to 29.4)	1.57 (1.04 to 2.27)	-93.4 (-95.8 to -90.1)
		Fungus	92.5 (65.1 to 127)	10.6 (7.53 to 14.4)	-88.5 (-90.6 to -86.3)	3.23 (2.21 to 4.49)	0.16 (0.09 to 0.23)	-95.1 (-96.8 to -92.8)
		Group B Streptococcus	430 (359 to 511)	56.6 (47.7 to 67.4)	-86.8 (-88.7 to -84.6)	17.9 (14.5 to 21.8)	1.09 (0.7 to 1.6)	-93.8 (-96.2 to -90.2)
		Haemophilus influenzae	3510 (3000 to 4080)	335 (289 to 390)	-90.4 (-91.8 to -88.9)	54.7 (44.9 to 65.3)	1.35 (0.93 to 1.85)	-97.5 (-98.4 to -96.4)
		Influenza	2960 (2540 to 3370)	5.34 (1.98 to 11.5)	-99.8 (-99.9 to -99.6)	87 (72.7 to 102)	0.06 (0.02 to 0.15)	-99.9 (-100.0 to -99.8)
		Klebsiella pneumoniae	1790 (1540 to 2050)	118 (101 to 137)	-93.4 (-94.2 to -92.4)	72 (60 to 85.3)	2.32 (1.57 to 3.28)	-96.7 (-97.9 to -95.2)
		Legionella spp.	160 (116 to 219)	25 (17.9 to 33.8)	-84.4 (-86.7 to -81.5)	7.19 (5.06 to 9.82)	0.56 (0.33 to 0.91)	-92.1 (-95.2 to -87.5)
		Mycoplasma spp.	2030 (1740 to 2350)	329 (285 to 381)	-83.7 (-85.9 to -81.1)	21.8 (18 to 25.7)	1.45 (0.98 to 1.99)	-93.3 (-95.6 to -90.1)
		Polymicrobial	85.1 (53.3 to 132)	9.22 (5.3 to 14.8)	-89.2 (-91.0 to -86.9)	8.85 (4.88 to 14.5)	0.67 (0.29 to 1.32)	-92.4 (-95.9 to -87.0)
		Pseudomonas aeruginosa	931 (799 to 1090)	205 (177 to 239)	-78.0 (-81.2 to -74.3)	29.7 (24.8 to 35.3)	2.72 (1.82 to 3.79)	-90.8 (-94.1 to -86.2)
		Respiratory syncytial virus	6440 (5620 to 7270)	19.3 (7.18 to 41.9)	-99.7 (-99.9 to -99.4)	94.6 (79.9 to 111)	0.12 (0.04 to 0.27)	-99.9 (-100.0 to -99.7)

		Staphylococcus aureus	1190 (1030 to 1360)	203 (175 to 235)	-83.0 (-84.8 to -80.8)	67.1 (55.8 to 78.7)	4.92 (3.38 to 6.71)	-92.6 (-95.1 to -89.2)
		Streptococcus pneumoniae	19500 (17000 to 21900)	638 (555 to 734)	-96.7 (-97.1 to -96.3)	415 (348 to 487)	5.71 (3.92 to 7.78)	-98.6 (-99.1 to -98.0)
		Other viruses	13300 (11600 to 15100)	1190 (1040 to 1370)	-91.1 (-92.0 to -89.9)	115 (97.5 to 135)	4.69 (3.22 to 6.42)	-95.9 (-97.3 to -94.0)
		Other bacterial pathogens	548 (432 to 675)	48.8 (38.6 to 61.2)	-91.1 (-92.5 to -89.4)	14.2 (10.9 to 18)	0.6 (0.37 to 0.87)	-95.8 (-97.3 to -93.6)
	5-14 years	*Acinetobacter baumannii*	162 (126 to 207)	11 (8.47 to 13.9)	-93.2 (-94.2 to -92.2)	0.92 (0.74 to 1.14)	0.03 (0.02 to 0.04)	-97.3 (-98.2 to -96.1)
		Chlamydia spp.	668 (529 to 832)	70.6 (54.6 to 88.7)	-89.4 (-90.9 to -87.8)	0.95 (0.76 to 1.15)	0.04 (0.03 to 0.06)	-95.7 (-97.1 to -93.9)
		Enterobacter spp.	54.6 (42.6 to 71)	15.5 (11.8 to 19.9)	-71.5 (-75.9 to -66.6)	0.3 (0.23 to 0.38)	0.03 (0.02 to 0.05)	-88.5 (-92.3 to -83.4)
		Escherichia coli	149 (119 to 184)	34.1 (26.4 to 42.6)	-77.1 (-80.2 to -73.5)	0.83 (0.68 to 1.02)	0.08 (0.05 to 0.1)	-90.8 (-93.8 to -87.1)
		Fungus	180 (141 to 225)	35.3 (27.7 to 44.6)	-80.4 (-83.1 to -77.0)	0.99 (0.79 to 1.22)	0.08 (0.05 to 0.11)	-92.1 (-94.7 to -88.9)
		Group B Streptococcus	183 (144 to 226)	42.3 (33.3 to 53.8)	-76.8 (-80.1 to -72.7)	0.72 (0.58 to 0.9)	0.07 (0.05 to 0.09)	-90.6 (-93.7 to -86.7)
		Haemophilus influenzae	715 (581 to 877)	136 (108 to 171)	-80.9 (-83.3 to -78.0)	1.53 (1.25 to 1.84)	0.08 (0.05 to 0.11)	-94.9 (-96.6 to -92.7)
		Influenza	1030 (836 to 1250)	4.16 (1.51 to 9.43)	-99.6 (-99.8 to -99.1)	4.29 (3.52 to 5.13)	0.01 (0 to 0.02)	-99.8 (-99.9 to -99.6)
		Klebsiella pneumoniae	439 (355 to 538)	68.3 (53.6 to 86.1)	-84.4 (-86.4 to -82.2)	2.44 (2.01 to 2.94)	0.15 (0.11 to 0.21)	-93.7 (-95.8 to -91.2)
		Legionella spp.	325 (259 to 404)	90.8 (71.8 to 114)	-72.0 (-76.0 to -67.4)	1.24 (1 to 1.52)	0.14 (0.1 to 0.19)	-88.7 (-92.4 to -83.9)
		Mycoplasma spp.	1610 (1290 to 1960)	271 (212 to 340)	-83.1 (-85.2 to -80.6)	2.37 (1.92 to 2.85)	0.16 (0.11 to 0.22)	-93.2 (-95.4 to -90.2)
		Polymicrobial	27.3 (17.2 to 41.4)	4.42 (2.56 to 6.98)	-83.9 (-86.7 to -80.7)	0.15 (0.1 to 0.23)	0.01 (0.01 to 0.02)	-93.5 (-95.7 to -90.7)
		Pseudomonas aeruginosa	366 (294 to 447)	109 (85 to 137)	-70.1 (-74.0 to -65.5)	1.41 (1.15 to 1.7)	0.17 (0.12 to 0.23)	-88.0 (-91.9 to -82.9)
		Respiratory syncytial virus	248 (199 to 305)	1.09 (0.39 to 2.45)	-99.6 (-99.8 to -99.1)	0.47 (0.38 to 0.57)	0 (0 to 0)	-99.8 (-99.9 to -99.6)
		Staphylococcus aureus	600 (488 to 733)	184 (143 to 230)	-69.4 (-73.1 to -65.1)	4.74 (3.91 to 5.68)	0.58 (0.42 to 0.79)	-87.6 (-91.7 to -82.4)
		Streptococcus pneumoniae	5910 (4790 to 7130)	552 (434 to 688)	-90.6 (-91.8 to -89.4)	19.4 (16.1 to 23)	0.73 (0.52 to 0.98)	-96.2 (-97.5 to -94.7)
		Other viruses	2440 (1980 to 2930)	534 (423 to 660)	-78.1 (-80.8 to -75.0)	3.15 (2.59 to 3.78)	0.28 (0.2 to 0.38)	-91.2 (-94.0 to -87.6)
		Other bacterial pathogens	657 (520 to 808)	93 (72.1 to 118)	-85.8 (-87.7 to -83.7)	2.06 (1.67 to 2.51)	0.12 (0.08 to 0.16)	-94.3 (-96.1 to -91.8)
	15-49 years	*Acinetobacter baumannii*	98.5 (82.9 to 119)	15.4 (12.8 to 18.4)	-84.3 (-86.1 to -82.5)	1.45 (1.2 to 1.76)	0.21 (0.16 to 0.27)	-85.6 (-89.1 to -81.1)
		Chlamydia spp.	406 (347 to 470)	99 (84.6 to 114)	-75.6 (-77.9 to -72.9)	1.48 (1.24 to 1.76)	0.33 (0.26 to 0.42)	-77.6 (-83.2 to -71.2)

		Enterobacter spp.	33.1 (27.3 to 40)	21.8 (18.4 to 25.7)	-34.1 (-42.8 to -25.0)	0.46 (0.38 to 0.57)	0.28 (0.21 to 0.36)	-39.6 (-54.6 to -21.7)
		Escherichia coli	90.6 (77.3 to 105)	47.8 (41.1 to 55)	-47.1 (-53.0 to -41.2)	1.31 (1.09 to 1.54)	0.63 (0.49 to 0.8)	-51.5 (-63.6 to -36.5)
		Fungus	109 (90.8 to 129)	49.5 (42 to 57.5)	-54.6 (-59.8 to -49.2)	1.56 (1.3 to 1.86)	0.64 (0.5 to 0.82)	-58.4 (-69.1 to -46.2)
		Group B Streptococcus	111 (94.3 to 131)	59.4 (50.8 to 69.6)	-46.3 (-52.3 to -40.0)	1.14 (0.94 to 1.37)	0.56 (0.43 to 0.72)	-50.7 (-63.3 to -36.3)
		Haemophilus influenzae	434 (376 to 497)	191 (165 to 220)	-55.9 (-60.0 to -51.4)	2.39 (2.05 to 2.81)	0.64 (0.5 to 0.81)	-73.0 (-79.7 to -64.8)
		Influenza	624 (545 to 703)	5.83 (2.21 to 12.9)	-99.1 (-99.7 to -98.0)	6.72 (5.8 to 7.78)	0.06 (0.02 to 0.12)	-99.2 (-99.7 to -98.1)
		Klebsiella pneumoniae	267 (233 to 302)	95.9 (82.5 to 109)	-64.0 (-67.3 to -60.8)	3.83 (3.28 to 4.48)	1.26 (0.98 to 1.59)	-67.0 (-75.1 to -57.9)
		Legionella spp.	197 (169 to 229)	127 (110 to 145)	-35.3 (-42.1 to -27.6)	1.95 (1.63 to 2.31)	1.15 (0.9 to 1.45)	-40.7 (-54.9 to -23.1)
		Mycoplasma spp.	974 (848 to 1110)	381 (330 to 433)	-60.9 (-64.5 to -57.1)	3.71 (3.17 to 4.29)	1.33 (1.03 to 1.68)	-64.1 (-73.0 to -53.8)
		Polymicrobial	16.6 (10.7 to 24.7)	6.21 (3.62 to 9.93)	-62.7 (-68.2 to -56.7)	0.24 (0.16 to 0.36)	0.08 (0.05 to 0.14)	-65.8 (-75.0 to -54.7)
		Pseudomonas aeruginosa	222 (193 to 254)	153 (131 to 175)	-31.0 (-37.5 to -24.6)	2.21 (1.89 to 2.58)	1.39 (1.08 to 1.77)	-36.7 (-52.0 to -17.9)
		Respiratory syncytial virus	151 (131 to 173)	1.53 (0.58 to 3.35)	-99.0 (-99.6 to -97.8)	0.74 (0.63 to 0.88)	0.01 (0 to 0.01)	-99.1 (-99.7 to -98.0)
		Staphylococcus aureus	364 (320 to 412)	258 (224 to 293)	-29.2 (-35.2 to -22.9)	7.43 (6.4 to 8.61)	4.8 (3.74 to 6.08)	-35.1 (-50.7 to -16.5)
		Streptococcus pneumoniae	3580 (3160 to 4040)	774 (673 to 880)	-78.4 (-80.1 to -76.6)	30.4 (26.5 to 35.3)	6 (4.69 to 7.6)	-80.2 (-85.0 to -74.5)
		Other viruses	1480 (1300 to 1670)	749 (649 to 852)	-49.4 (-53.6 to -45.1)	4.94 (4.28 to 5.75)	2.28 (1.79 to 2.88)	-53.6 (-65.0 to -40.4)
		Other bacterial pathogens	399 (342 to 461)	131 (109 to 153)	-67.2 (-70.4 to -64.0)	3.22 (2.71 to 3.81)	0.97 (0.73 to 1.23)	-69.9 (-77.2 to -60.9)
	50-69 years	*Acinetobacter baumannii*	212 (179 to 247)	62.9 (53.3 to 72.9)	-70.3 (-73.9 to -66.3)	3.75 (3.03 to 4.63)	0.94 (0.67 to 1.27)	-74.8 (-82.6 to -65.1)
		Chlamydia spp.	518 (455 to 597)	234 (204 to 263)	-54.7 (-60.1 to -48.6)	2.27 (1.84 to 2.74)	0.87 (0.62 to 1.14)	-61.6 (-74.1 to -46.8)
		Enterobacter spp.	69.4 (58.2 to 82.7)	84.9 (73 to 97.1)	22.8 (6.5 to 40.8)	1.16 (0.92 to 1.45)	1.2 (0.86 to 1.62)	4.3 (-28.7 to 45.7)
		Escherichia coli	209 (183 to 242)	201 (176 to 226)	-3.8 (-15.7 to 9.4)	3.63 (2.95 to 4.41)	2.94 (2.14 to 3.92)	-18.3 (-43.0 to 12.6)
		Fungus	201 (171 to 236)	170 (146 to 195)	-15.1 (-25.9 to -3.1)	3.44 (2.78 to 4.25)	2.45 (1.79 to 3.25)	-28.0 (-50.2 to 0.2)
		Group B Streptococcus	164 (142 to 191)	162 (141 to 185)	-0.8 (-14.4 to 13.0)	2.02 (1.63 to 2.48)	1.68 (1.22 to 2.23)	-15.8 (-42.1 to 17.2)
		Haemophilus influenzae	549 (488 to 621)	462 (405 to 516)	-15.6 (-25.1 to -5.4)	3.63 (2.98 to 4.32)	1.72 (1.26 to 2.29)	-52.3 (-67.3 to -33.8)
		Influenza	680 (610 to 762)	12.2 (4.59 to 26.3)	-98.2 (-99.3 to -96.1)	8.8 (7.27 to 10.5)	0.13 (0.05 to 0.29)	-98.5 (-99.5 to -96.6)

		Klebsiella pneumoniae	432 (387 to 485)	306 (269 to 341)	-29.0 (-37.2 to -20.6)	7.44 (6.14 to 8.87)	4.45 (3.22 to 5.92)	-39.7 (-57.9 to -16.5)
		Legionella spp.	351 (302 to 407)	419 (367 to 469)	20.0 (4.8 to 37.0)	4.16 (3.36 to 5.01)	4.2 (3.03 to 5.56)	1.8 (-27.9 to 39.0)
		Mycoplasma spp.	565 (504 to 643)	382 (336 to 427)	-32.3 (-40.5 to -24.4)	2.58 (2.11 to 3.08)	1.47 (1.07 to 1.95)	-42.5 (-60.2 to -21.1)
		Polymicrobial	29.7 (19.9 to 44.8)	20.9 (12.6 to 33.7)	-30.0 (-41.3 to -18.0)	0.52 (0.34 to 0.77)	0.31 (0.17 to 0.53)	-40.5 (-59.9 to -17.1)
		Pseudomonas aeruginosa	398 (353 to 450)	492 (433 to 549)	23.9 (9.6 to 38.8)	4.76 (3.91 to 5.71)	4.96 (3.58 to 6.61)	5.2 (-27.1 to 46.7)
		Respiratory syncytial virus	121 (105 to 138)	2.24 (0.83 to 4.8)	-98.2 (-99.3 to -96.0)	0.72 (0.57 to 0.87)	0.01 (0 to 0.02)	-98.5 (-99.5 to -96.6)
		Staphylococcus aureus	575 (516 to 644)	763 (670 to 849)	33.0 (18.3 to 48.5)	14.1 (11.6 to 16.7)	15.8 (11.4 to 21)	12.9 (-21.6 to 55.5)
		Streptococcus pneumoniae	2790 (2510 to 3120)	1200 (1060 to 1340)	-56.8 (-61.6 to -51.8)	28.4 (23.4 to 33.8)	10.3 (7.5 to 13.7)	-63.3 (-74.4 to -49.2)
		Other viruses	1010 (908 to 1130)	1010 (889 to 1130)	0.3 (-10.8 to 12.1)	4.04 (3.33 to 4.81)	3.41 (2.46 to 4.55)	-14.8 (-41.0 to 18.4)
		Other bacterial pathogens	761 (659 to 873)	460 (391 to 532)	-39.4 (-46.5 to -31.9)	7.39 (6.07 to 8.94)	3.78 (2.69 to 5.09)	-48.5 (-64.8 to -30.0)
	70+ years	*Acinetobacter baumannii*	246 (205 to 292)	96.8 (82.2 to 115)	-60.6 (-65.5 to -55.1)	12.4 (9.4 to 14.9)	4.59 (3.48 to 6.36)	-62.3 (-73.3 to -43.2)
		Chlamydia spp.	715 (621 to 823)	429 (374 to 500)	-39.8 (-47.4 to -31.8)	8.92 (6.81 to 10.6)	5.06 (3.85 to 6.86)	-42.6 (-59.6 to -12.2)
		Enterobacter spp.	78.4 (64.8 to 94.9)	127 (109 to 148)	63.2 (41.0 to 85.5)	3.73 (2.75 to 4.59)	5.73 (4.31 to 7.72)	55.8 (12.0 to 136.7)
		Escherichia coli	298 (257 to 347)	381 (332 to 437)	27.9 (12.9 to 44.5)	14.7 (11.3 to 17.5)	17.7 (13.6 to 23.8)	22.1 (-13.5 to 84.1)
		Fungus	130 (109 to 155)	146 (125 to 170)	12.8 (-1.6 to 29.9)	6.32 (4.81 to 7.63)	6.7 (5.02 to 9.01)	7.7 (-22.7 to 62.0)
		Group B Streptococcus	175 (150 to 202)	229 (201 to 264)	31.8 (14.7 to 49.7)	6.11 (4.65 to 7.34)	7.58 (5.76 to 10.3)	25.8 (-10.4 to 93.1)
		Haemophilus influenzae	573 (505 to 650)	642 (566 to 733)	12.2 (-0.9 to 26.0)	10.8 (8.31 to 12.5)	7.59 (5.88 to 10.2)	-28.6 (-48.9 to 8.2)
		Influenza	1200 (1070 to 1340)	28.6 (10.7 to 60.9)	-97.6 (-99.1 to -94.9)	44.1 (34 to 50.7)	0.96 (0.34 to 2.12)	-97.8 (-99.2 to -95.2)
		Klebsiella pneumoniae	552 (491 to 622)	520 (460 to 594)	-5.6 (-16.2 to 5.3)	27 (20.9 to 31.3)	24.1 (18.6 to 32.6)	-9.8 (-35.5 to 36.3)
		Legionella spp.	292 (253 to 344)	465 (410 to 534)	59.5 (38.1 to 81.2)	9.86 (7.66 to 11.8)	14.8 (11.4 to 20)	52.2 (10.1 to 127.9)
		Mycoplasma spp.	554 (487 to 635)	497 (438 to 569)	-10.0 (-20.5 to 1.0)	7.21 (5.55 to 8.38)	6.11 (4.69 to 8.22)	-14.1 (-38.5 to 30.7)
		Polymicrobial	39.5 (26.1 to 60)	37 (21.9 to 60.4)	-6.9 (-22.0 to 9.1)	1.97 (1.24 to 3.03)	1.74 (0.97 to 3.11)	-11.1 (-37.9 to 34.3)
		Pseudomonas aeruginosa	480 (421 to 546)	790 (695 to 903)	64.7 (45.9 to 85.3)	16.3 (12.5 to 18.9)	25.4 (19.5 to 34.2)	57.3 (12.8 to 139.2)
		Respiratory syncytial virus	132 (115 to 152)	3.23 (1.25 to 6.84)	-97.5 (-99.1 to -94.8)	2.22 (1.7 to 2.61)	0.05 (0.02 to 0.11)	-97.7 (-99.2 to -95.0)

		Staphylococcus aureus	862 (766 to 970)	1520 (1350 to 1730)	76.7 (57.4 to 96.1)	60 (46.2 to 69.4)	100 (76.9 to 135)	68.8 (21.6 to 156.4)
		Streptococcus pneumoniae	3330 (2970 to 3720)	1910 (1690 to 2170)	-42.6 (-49.0 to -36.2)	96.5 (74.1 to 112)	52.2 (40.4 to 70.1)	-45.2 (-60.6 to -17.2)
		Other viruses	1190 (1060 to 1330)	1580 (1400 to 1800)	33.3 (18.7 to 47.7)	13.6 (10.5 to 15.7)	17 (13.3 to 22.9)	27.3 (-8.5 to 92.9)
		Other bacterial pathogens	1180 (1020 to 1350)	949 (811 to 1110)	-19.5 (-28.9 to -9.4)	33 (25.2 to 38.9)	25.1 (18.8 to 33.9)	-22.9 (-44.8 to 16.1)
Bosnia and Herzegovina	All Ages	*Acinetobacter baumannii*	899 (778 to 1020)	386 (331 to 442)	-57.0 (-60.7 to -52.5)	12.5 (10.4 to 15)	8.37 (6.14 to 10.8)	-32.8 (-51.1 to -14.4)
		Chlamydia spp.	3230 (2940 to 3560)	1150 (1050 to 1250)	-64.4 (-67.2 to -61.4)	9.52 (8.1 to 11)	5.77 (4.29 to 7.45)	-39.2 (-55.5 to -21.5)
		Enterobacter spp.	345 (295 to 397)	271 (238 to 303)	-21.3 (-29.3 to -13.4)	4.18 (3.42 to 5.03)	5.34 (3.96 to 6.81)	28.8 (-6.4 to 66.8)
		Escherichia coli	1100 (992 to 1230)	683 (622 to 747)	-38.0 (-43.7 to -31.8)	14.5 (12.4 to 17)	14.7 (10.8 to 18.6)	2.0 (-26.3 to 30.7)
		Fungus	796 (692 to 915)	464 (418 to 516)	-41.5 (-46.9 to -35.8)	8.33 (6.95 to 10)	8.21 (6.08 to 10.5)	-0.9 (-28.4 to 29.0)
		Group B Streptococcus	923 (823 to 1040)	580 (529 to 634)	-37.1 (-42.8 to -31.3)	7.86 (6.69 to 9.19)	7.89 (5.88 to 9.99)	0.9 (-26.3 to 30.0)
		Haemophilus influenzae	4090 (3720 to 4490)	1620 (1500 to 1760)	-60.2 (-63.4 to -56.9)	14.4 (12.5 to 16.4)	8.5 (6.3 to 10.8)	-40.7 (-56.6 to -25.6)
		Influenza	5280 (4880 to 5730)	50.4 (19 to 108)	-99.0 (-99.6 to -98.0)	43 (37.1 to 49.3)	0.73 (0.25 to 1.63)	-98.3 (-99.4 to -96.2)
		Klebsiella pneumoniae	2720 (2500 to 2960)	1430 (1320 to 1550)	-47.3 (-51.4 to -42.9)	29.1 (25.1 to 33.2)	28.3 (21 to 35.8)	-2.2 (-29.5 to 23.8)
		Legionella spp.	1510 (1350 to 1690)	1200 (1100 to 1310)	-20.4 (-27.3 to -13.0)	12.2 (10.4 to 14.4)	15.8 (11.6 to 20)	29.9 (-5.6 to 68.1)
		Mycoplasma spp.	4920 (4420 to 5390)	2050 (1880 to 2220)	-58.2 (-61.2 to -55.2)	10.7 (9.29 to 12.4)	8.07 (6.01 to 10.2)	-24.5 (-44.9 to -3.7)
		Polymicrobial	167 (117 to 237)	81.9 (52 to 130)	-51.2 (-58.6 to -43.3)	2.83 (1.96 to 4.01)	1.83 (1.09 to 3.08)	-35.5 (-56.8 to -10.2)
		Pseudomonas aeruginosa	2180 (1990 to 2380)	1970 (1810 to 2130)	-9.3 (-16.7 to -1.1)	17.9 (15.3 to 20.7)	27.4 (20.3 to 34.8)	53.8 (12.1 to 96.1)
		Respiratory syncytial virus	3350 (2940 to 3780)	22.1 (8.42 to 47.9)	-99.3 (-99.7 to -98.6)	7.92 (6.6 to 9.37)	0.07 (0.03 to 0.16)	-99.1 (-99.7 to -97.9)
		Staphylococcus aureus	3360 (3100 to 3640)	2870 (2650 to 3110)	-14.5 (-20.3 to -8.2)	57.3 (49.1 to 66.1)	85.3 (63 to 109)	49.4 (9.6 to 89.5)
		Streptococcus pneumoniae	25400 (23500 to 27600)	8880 (8270 to 9550)	-65.0 (-67.3 to -62.8)	122 (107 to 138)	85.9 (63.6 to 108)	-29.3 (-48.2 to -10.5)
		Other viruses	12400 (11300 to 13600)	6120 (5660 to 6610)	-50.5 (-54.1 to -47.1)	20.6 (17.9 to 23.2)	21.3 (15.9 to 26.9)	4.1 (-23.8 to 31.3)
		Other bacterial pathogens	3810 (3400 to 4250)	2040 (1790 to 2290)	-46.4 (-51.0 to -41.7)	29.1 (24 to 34.2)	25.6 (18.9 to 33)	-11.8 (-35.7 to 14.2)
	Under 5	*Acinetobacter baumannii*	48 (33.7 to 67.2)	5.15 (3.57 to 7.37)	-89.3 (-90.7 to -87.9)	1.06 (0.57 to 1.88)	0.1 (0.04 to 0.19)	-90.9 (-94.6 to -84.6)
		Chlamydia spp.	561 (473 to 655)	66.6 (54.8 to 80.6)	-88.1 (-89.6 to -86.4)	1.18 (0.9 to 1.49)	0.1 (0.07 to 0.15)	-91.2 (-94.4 to -86.4)

		Enterobacter spp.	54.2 (41.3 to 69.4)	12.3 (9.14 to 16.3)	-77.2 (-80.6 to -73.4)	0.52 (0.31 to 0.84)	0.07 (0.04 to 0.13)	-85.4 (-90.8 to -77.2)
		Escherichia coli	191 (159 to 230)	34.6 (28.2 to 42.1)	-81.9 (-84.3 to -79.1)	1.6 (1.2 to 2.11)	0.19 (0.13 to 0.28)	-87.8 (-92.2 to -81.4)
		Fungus	36.3 (25.1 to 50)	6.79 (4.73 to 9.48)	-81.2 (-83.8 to -78.1)	0.1 (0.06 to 0.15)	0.01 (0.01 to 0.02)	-86.5 (-91.7 to -78.9)
		Group B Streptococcus	167 (138 to 200)	33 (26.9 to 41.1)	-80.3 (-82.8 to -77.4)	1.48 (1.11 to 1.91)	0.18 (0.12 to 0.26)	-87.9 (-92.3 to -80.2)
		Haemophilus influenzae	1390 (1160 to 1650)	192 (156 to 232)	-86.2 (-88.1 to -84.0)	2.69 (1.97 to 3.45)	0.14 (0.1 to 0.19)	-94.7 (-96.6 to -92.1)
		Influenza	1170 (988 to 1360)	3.57 (1.34 to 7.87)	-99.7 (-99.9 to -99.4)	3.91 (2.89 to 5)	0.01 (0 to 0.02)	-99.8 (-99.9 to -99.6)
		Klebsiella pneumoniae	699 (590 to 813)	105 (86.5 to 128)	-84.9 (-86.7 to -82.8)	3.45 (2.6 to 4.36)	0.36 (0.26 to 0.5)	-89.4 (-93.0 to -84.0)
		Legionella spp.	63.4 (45 to 86.2)	13.7 (9.52 to 19.3)	-78.4 (-81.3 to -75.2)	0.66 (0.43 to 0.94)	0.1 (0.06 to 0.15)	-85.3 (-91.2 to -75.8)
		Mycoplasma spp.	799 (675 to 937)	148 (122 to 181)	-81.4 (-83.8 to -78.7)	1.05 (0.78 to 1.36)	0.12 (0.08 to 0.16)	-88.5 (-92.5 to -82.9)
		Polymicrobial	32.5 (20 to 51.1)	4.79 (2.82 to 7.59)	-85.2 (-87.3 to -83.2)	1.04 (0.54 to 1.77)	0.14 (0.06 to 0.28)	-86.0 (-92.0 to -76.0)
		Pseudomonas aeruginosa	371 (312 to 435)	105 (85.6 to 127)	-71.8 (-75.3 to -67.5)	1.86 (1.42 to 2.32)	0.3 (0.21 to 0.42)	-83.6 (-89.4 to -75.6)
		Respiratory syncytial virus	2590 (2220 to 2980)	11.7 (4.43 to 25.6)	-99.5 (-99.8 to -99.0)	5.29 (4.03 to 6.61)	0.01 (0 to 0.03)	-99.8 (-99.9 to -99.4)
		Staphylococcus aureus	474 (403 to 546)	119 (97.1 to 145)	-74.9 (-77.9 to -71.5)	3.11 (2.3 to 3.95)	0.5 (0.35 to 0.67)	-83.7 (-89.3 to -75.7)
		Streptococcus pneumoniae	7680 (6510 to 8850)	1040 (854 to 1270)	-86.4 (-88.0 to -84.6)	14.5 (9.98 to 18.7)	1.44 (1 to 1.94)	-89.8 (-93.5 to -84.6)
		Other viruses	5360 (4520 to 6160)	1110 (905 to 1340)	-79.4 (-81.8 to -76.6)	4.58 (3.33 to 5.88)	0.66 (0.46 to 0.88)	-85.3 (-90.4 to -78.1)
		Other bacterial pathogens	214 (168 to 267)	30.7 (23.7 to 39.1)	-85.6 (-87.5 to -83.6)	0.79 (0.52 to 1.13)	0.08 (0.05 to 0.13)	-89.7 (-93.4 to -84.6)
	5-14 years	*Acinetobacter baumannii*	126 (95.1 to 163)	22.9 (17 to 30.2)	-81.8 (-83.9 to -79.2)	0.2 (0.15 to 0.26)	0.02 (0.02 to 0.03)	-88.6 (-91.8 to -84.8)
		Chlamydia spp.	530 (404 to 685)	94.3 (70.2 to 124)	-82.2 (-84.2 to -79.8)	0.21 (0.16 to 0.27)	0.02 (0.02 to 0.03)	-88.8 (-92.0 to -85.1)
		Enterobacter spp.	44.3 (33.5 to 58.3)	16.3 (12.1 to 21.9)	-63.1 (-67.8 to -57.8)	0.07 (0.05 to 0.09)	0.02 (0.01 to 0.02)	-76.8 (-83.5 to -68.7)
		Escherichia coli	120 (91.3 to 153)	33.5 (25.1 to 43.5)	-72.0 (-75.5 to -68.1)	0.19 (0.14 to 0.23)	0.03 (0.03 to 0.04)	-82.4 (-87.4 to -76.3)
		Fungus	145 (108 to 188)	41 (30.3 to 53.8)	-71.6 (-75.1 to -67.6)	0.22 (0.17 to 0.28)	0.04 (0.03 to 0.05)	-82.2 (-87.2 to -76.3)
		Group B Streptococcus	147 (111 to 191)	47.9 (36 to 62.5)	-67.5 (-71.4 to -62.9)	0.16 (0.12 to 0.21)	0.03 (0.03 to 0.04)	-79.6 (-85.4 to -72.6)
		Haemophilus influenzae	581 (451 to 744)	138 (104 to 179)	-76.2 (-78.8 to -73.0)	0.34 (0.27 to 0.42)	0.04 (0.03 to 0.05)	-88.3 (-91.6 to -84.5)
		Influenza	834 (644 to 1060)	4.01 (1.37 to 9.09)	-99.5 (-99.8 to -99.0)	0.96 (0.76 to 1.19)	0 (0 to 0.01)	-99.7 (-99.9 to -99.4)

		Klebsiella pneumoniae	353 (272 to 452)	93 (69.9 to 122)	-73.6 (-76.5 to -70.1)	0.54 (0.43 to 0.68)	0.09 (0.07 to 0.11)	-83.5 (-87.9 to -78.1)
		Legionella spp.	268 (203 to 347)	99.3 (75.2 to 128)	-62.9 (-67.3 to -57.3)	0.28 (0.22 to 0.36)	0.07 (0.05 to 0.08)	-76.7 (-83.4 to -69.3)
		Mycoplasma spp.	1300 (994 to 1660)	340 (258 to 444)	-73.8 (-76.7 to -70.3)	0.53 (0.42 to 0.66)	0.09 (0.07 to 0.11)	-83.6 (-88.1 to -78.3)
		Polymicrobial	21.6 (13.6 to 33.2)	5.07 (2.97 to 8.1)	-76.6 (-80.0 to -72.9)	0.03 (0.02 to 0.05)	0 (0 to 0.01)	-85.3 (-89.6 to -80.0)
		Pseudomonas aeruginosa	300 (227 to 382)	131 (99 to 171)	-56.2 (-61.3 to -50.2)	0.32 (0.25 to 0.4)	0.09 (0.07 to 0.11)	-72.5 (-80.3 to -63.3)
		Respiratory syncytial virus	205 (157 to 264)	1.42 (0.48 to 3.22)	-99.3 (-99.7 to -98.5)	0.11 (0.09 to 0.14)	0 (0 to 0)	-99.6 (-99.9 to -99.1)
		Staphylococcus aureus	489 (375 to 620)	190 (144 to 244)	-61.2 (-65.0 to -56.1)	1.07 (0.84 to 1.32)	0.26 (0.2 to 0.32)	-75.6 (-82.3 to -67.8)
		Streptococcus pneumoniae	4770 (3730 to 6040)	1030 (781 to 1340)	-78.4 (-80.6 to -75.6)	4.34 (3.43 to 5.37)	0.58 (0.46 to 0.72)	-86.5 (-90.2 to -82.1)
		Other viruses	2010 (1560 to 2540)	711 (537 to 926)	-64.6 (-68.4 to -60.0)	0.72 (0.57 to 0.89)	0.16 (0.12 to 0.2)	-77.8 (-83.9 to -70.5)
		Other bacterial pathogens	526 (404 to 681)	116 (86.8 to 152)	-78.0 (-80.5 to -75.2)	0.46 (0.36 to 0.58)	0.06 (0.05 to 0.08)	-86.2 (-90.0 to -81.6)
	15-49 years	*Acinetobacter baumannii*	122 (101 to 147)	29.4 (24.2 to 35.2)	-75.9 (-78.2 to -73.5)	0.93 (0.75 to 1.17)	0.24 (0.16 to 0.34)	-73.6 (-83.0 to -63.4)
		Chlamydia spp.	514 (439 to 600)	121 (103 to 141)	-76.4 (-78.5 to -73.9)	0.97 (0.79 to 1.18)	0.25 (0.16 to 0.34)	-74.2 (-83.4 to -64.3)
		Enterobacter spp.	43 (35.3 to 51.7)	21 (17.7 to 25.1)	-51.1 (-56.1 to -45.1)	0.31 (0.24 to 0.39)	0.16 (0.11 to 0.22)	-46.4 (-65.5 to -24.6)
		Escherichia coli	116 (98.1 to 135)	43 (37 to 49.6)	-63.0 (-66.3 to -58.8)	0.86 (0.71 to 1.06)	0.35 (0.22 to 0.46)	-59.4 (-74.0 to -44.4)
		Fungus	140 (116 to 165)	52.6 (44.7 to 61.7)	-62.4 (-66.2 to -58.0)	1.03 (0.83 to 1.26)	0.42 (0.28 to 0.58)	-58.8 (-73.7 to -43.4)
		Group B Streptococcus	143 (122 to 170)	61.4 (52.8 to 72.4)	-57.0 (-61.0 to -52.3)	0.75 (0.62 to 0.93)	0.35 (0.23 to 0.48)	-52.8 (-69.6 to -33.8)
		Haemophilus influenzae	563 (484 to 646)	177 (153 to 203)	-68.5 (-71.0 to -65.3)	1.59 (1.3 to 1.93)	0.43 (0.28 to 0.58)	-72.9 (-82.7 to -62.8)
		Influenza	808 (705 to 921)	5.12 (1.94 to 11.2)	-99.4 (-99.8 to -98.6)	4.48 (3.71 to 5.38)	0.03 (0.01 to 0.07)	-99.3 (-99.8 to -98.4)
		Klebsiella pneumoniae	342 (296 to 391)	119 (104 to 136)	-65.1 (-67.8 to -61.9)	2.52 (2.1 to 3.06)	0.96 (0.63 to 1.28)	-61.8 (-75.4 to -47.4)
		Legionella spp.	260 (220 to 301)	127 (110 to 145)	-50.9 (-55.3 to -45.4)	1.32 (1.09 to 1.61)	0.7 (0.47 to 0.94)	-46.2 (-65.5 to -25.2)
		Mycoplasma spp.	1260 (1100 to 1450)	437 (379 to 500)	-65.4 (-68.1 to -62.0)	2.47 (2.04 to 2.97)	0.93 (0.61 to 1.26)	-62.0 (-75.6 to -47.3)
		Polymicrobial	21 (13.5 to 31.3)	6.52 (3.95 to 10.5)	-69.1 (-72.7 to -65.0)	0.16 (0.1 to 0.24)	0.05 (0.03 to 0.09)	-66.1 (-78.8 to -52.9)
		Pseudomonas aeruginosa	291 (250 to 336)	169 (145 to 193)	-42.0 (-47.0 to -36.7)	1.49 (1.23 to 1.79)	0.94 (0.62 to 1.27)	-36.5 (-59.4 to -11.6)
		Respiratory syncytial virus	199 (171 to 227)	1.82 (0.68 to 3.93)	-99.1 (-99.7 to -98.0)	0.5 (0.42 to 0.6)	0 (0 to 0.01)	-99.0 (-99.7 to -97.7)

		Staphylococcus aureus	474 (412 to 539)	243 (211 to 277)	-48.6 (-52.3 to -43.9)	4.97 (4.13 to 5.94)	2.78 (1.83 to 3.71)	-43.6 (-64.0 to -22.2)
		Streptococcus pneumoniae	4630 (4060 to 5230)	1320 (1140 to 1500)	-71.4 (-73.4 to -68.9)	20.2 (16.8 to 24.2)	6.28 (4.12 to 8.36)	-68.7 (-79.9 to -56.7)
		Other viruses	1950 (1700 to 2210)	912 (792 to 1040)	-53.2 (-56.3 to -49.0)	3.35 (2.79 to 4.02)	1.71 (1.11 to 2.27)	-48.7 (-66.9 to -29.3)
		Other bacterial pathogens	511 (432 to 594)	148 (124 to 175)	-70.9 (-73.3 to -68.0)	2.12 (1.73 to 2.62)	0.67 (0.45 to 0.91)	-68.1 (-79.4 to -55.7)
	50-69 years	*Acinetobacter baumannii*	336 (282 to 395)	130 (110 to 152)	-61.2 (-65.6 to -56.6)	3.66 (2.92 to 4.54)	1.87 (1.29 to 2.48)	-48.6 (-65.5 to -29.8)
		Chlamydia spp.	836 (729 to 963)	308 (273 to 347)	-63.1 (-67.1 to -58.8)	2.26 (1.86 to 2.73)	1.09 (0.78 to 1.45)	-51.2 (-66.6 to -33.4)
		Enterobacter spp.	115 (96.8 to 137)	89 (77.1 to 103)	-22.2 (-31.8 to -12.2)	1.19 (0.93 to 1.47)	1.21 (0.85 to 1.6)	3.1 (-31.0 to 41.1)
		Escherichia coli	343 (297 to 393)	199 (176 to 223)	-41.7 (-48.1 to -34.8)	3.66 (3 to 4.44)	2.8 (1.98 to 3.65)	-22.8 (-47.4 to 3.9)
		Fungus	329 (277 to 383)	197 (171 to 225)	-40.0 (-46.9 to -32.7)	3.47 (2.76 to 4.26)	2.73 (1.92 to 3.62)	-20.6 (-47.2 to 8.3)
		Group B Streptococcus	270 (232 to 314)	183 (161 to 208)	-32.1 (-39.9 to -24.2)	2.05 (1.64 to 2.49)	1.82 (1.29 to 2.37)	-10.1 (-38.5 to 22.8)
		Haemophilus influenzae	908 (800 to 1030)	472 (420 to 528)	-47.9 (-53.3 to -42.3)	3.7 (3.04 to 4.41)	1.99 (1.41 to 2.61)	-45.9 (-63.0 to -27.0)
		Influenza	1120 (994 to 1260)	11.4 (4.26 to 24.4)	-99.0 (-99.6 to -97.8)	8.95 (7.35 to 10.6)	0.12 (0.04 to 0.25)	-98.7 (-99.6 to -97.0)
		Klebsiella pneumoniae	706 (629 to 797)	416 (371 to 463)	-41.1 (-47.2 to -34.8)	7.5 (6.15 to 8.98)	5.8 (4.1 to 7.55)	-22.0 (-46.9 to 4.6)
		Legionella spp.	588 (512 to 679)	460 (408 to 513)	-21.7 (-30.2 to -12.4)	4.3 (3.49 to 5.21)	4.42 (3.15 to 5.83)	3.6 (-29.9 to 39.6)
		Mycoplasma spp.	932 (821 to 1060)	494 (441 to 550)	-46.9 (-52.2 to -41.2)	2.63 (2.16 to 3.15)	1.83 (1.3 to 2.4)	-29.8 (-52.3 to -4.9)
		Polymicrobial	48 (31.9 to 70.7)	23.9 (14.6 to 38.6)	-50.5 (-57.1 to -42.9)	0.52 (0.32 to 0.81)	0.34 (0.19 to 0.58)	-34.5 (-55.9 to -10.3)
		Pseudomonas aeruginosa	665 (585 to 754)	606 (542 to 673)	-8.7 (-18.2 to 1.5)	4.9 (4.05 to 5.92)	5.88 (4.13 to 7.71)	20.8 (-18.1 to 62.3)
		Respiratory syncytial virus	204 (178 to 233)	2.94 (1.11 to 6.28)	-98.6 (-99.5 to -96.9)	0.74 (0.6 to 0.9)	0.01 (0 to 0.03)	-98.2 (-99.4 to -95.8)
		Staphylococcus aureus	952 (848 to 1060)	782 (697 to 868)	-17.7 (-25.8 to -9.2)	14.4 (11.9 to 17)	15.5 (11 to 20.2)	8.9 (-26.8 to 47.2)
		Streptococcus pneumoniae	4590 (4090 to 5120)	2140 (1910 to 2360)	-53.4 (-57.9 to -48.5)	28.8 (23.8 to 34.2)	17.7 (12.6 to 23)	-38.3 (-58.0 to -17.3)
		Other viruses	1690 (1500 to 1890)	1330 (1190 to 1470)	-21.3 (-29.0 to -13.1)	4.18 (3.44 to 4.96)	4.32 (3.08 to 5.66)	4.2 (-28.8 to 39.9)
		Other bacterial pathogens	1240 (1070 to 1410)	575 (493 to 657)	-53.7 (-58.4 to -48.8)	7.44 (5.95 to 8.95)	4.53 (3.16 to 6.07)	-38.6 (-58.0 to -15.9)
	70+ years	*Acinetobacter baumannii*	266 (222 to 315)	198 (166 to 231)	-25.3 (-34.1 to -14.9)	6.66 (5.24 to 8.11)	6.15 (4.48 to 8.06)	-7.0 (-33.8 to 22.5)
		Chlamydia spp.	787 (678 to 904)	558 (495 to 631)	-29.0 (-36.9 to -20.0)	4.9 (3.97 to 5.86)	4.3 (3.17 to 5.66)	-11.6 (-36.5 to 19.9)

		Enterobacter spp.	88.4 (72.4 to 106)	132 (113 to 152)	49.8 (30.7 to 71.5)	2.1 (1.61 to 2.62)	3.88 (2.86 to 5.07)	86.6 (32.1 to 151.2)
		Escherichia coli	333 (283 to 389)	372 (328 to 420)	12.2 (-1.2 to 26.8)	8.18 (6.69 to 9.81)	11.3 (8.34 to 14.5)	39.8 (0.1 to 87.1)
		Fungus	145 (120 to 173)	167 (145 to 192)	15.5 (1.1 to 32.0)	3.51 (2.81 to 4.3)	5.01 (3.75 to 6.5)	43.8 (2.6 to 91.2)
		Group B Streptococcus	196 (168 to 229)	255 (225 to 288)	30.6 (14.4 to 47.4)	3.42 (2.75 to 4.15)	5.51 (4.12 to 7.09)	62.7 (17.1 to 117.2)
		Haemophilus influenzae	647 (563 to 742)	646 (580 to 726)	0.2 (-10.7 to 12.5)	6.06 (4.95 to 7.2)	5.9 (4.38 to 7.65)	-2.0 (-29.0 to 28.3)
		Influenza	1350 (1180 to 1540)	26.3 (9.9 to 56)	-98.0 (-99.3 to -95.8)	24.7 (20.3 to 29.4)	0.58 (0.2 to 1.29)	-97.6 (-99.2 to -94.7)
		Klebsiella pneumoniae	616 (538 to 701)	696 (621 to 779)	13.4 (0.4 to 27.7)	15 (12.3 to 17.8)	21.1 (15.7 to 27.1)	41.2 (1.1 to 86.6)
		Legionella spp.	335 (286 to 390)	503 (448 to 567)	50.7 (33.1 to 71.3)	5.63 (4.55 to 6.82)	10.5 (7.78 to 13.6)	87.6 (34.6 to 150.4)
		Mycoplasma spp.	623 (538 to 715)	635 (567 to 711)	2.1 (-9.2 to 14.7)	4.04 (3.27 to 4.85)	5.11 (3.8 to 6.6)	27.2 (-9.8 to 68.6)
		Polymicrobial	43.5 (28.6 to 68)	41.6 (25.4 to 66.7)	-4.7 (-17.7 to 11.5)	1.08 (0.67 to 1.65)	1.28 (0.71 to 2.25)	18.7 (-16.3 to 59.7)
		Pseudomonas aeruginosa	548 (476 to 631)	959 (859 to 1080)	75.6 (56.2 to 97.6)	9.29 (7.48 to 11.1)	20.2 (15.1 to 26.1)	118.7 (55.1 to 189.5)
		Respiratory syncytial virus	152 (129 to 176)	4.19 (1.62 to 9.03)	-97.2 (-98.9 to -94.1)	1.27 (1.02 to 1.53)	0.04 (0.01 to 0.09)	-96.7 (-98.9 to -92.5)
		Staphylococcus aureus	974 (850 to 1110)	1540 (1380 to 1720)	58.4 (41.6 to 76.0)	33.8 (27.6 to 40.6)	66.2 (49.5 to 85.8)	97.3 (41.8 to 160.6)
		Streptococcus pneumoniae	3740 (3270 to 4240)	3350 (3010 to 3740)	-10.3 (-20.0 to 0.2)	54 (44 to 64)	59.9 (45 to 77.4)	11.7 (-19.6 to 47.2)
		Other viruses	1360 (1190 to 1550)	2060 (1850 to 2300)	51.5 (35.3 to 68.9)	7.74 (6.26 to 9.12)	14.5 (10.9 to 18.7)	88.6 (35.5 to 148.6)
		Other bacterial pathogens	1310 (1120 to 1530)	1170 (1010 to 1350)	-10.8 (-21.2 to 0.2)	18.3 (14.7 to 22.1)	20.2 (15 to 26.3)	11.2 (-20.3 to 49.5)
Bulgaria	All Ages	*Acinetobacter baumannii*	2700 (2350 to 3080)	686 (603 to 779)	-74.5 (-77.0 to -71.9)	79.5 (69.9 to 89.9)	24.3 (20.9 to 28.2)	-69.4 (-73.6 to -64.5)
		Chlamydia spp.	10200 (9310 to 11100)	3100 (2820 to 3390)	-69.4 (-72.1 to -66.5)	70 (64.7 to 76)	26.2 (22.7 to 29.8)	-62.5 (-67.9 to -57.0)
		Enterobacter spp.	1370 (1200 to 1570)	807 (721 to 895)	-40.8 (-46.8 to -34.9)	36.9 (32.3 to 42)	26.2 (22.7 to 30.4)	-28.8 (-38.8 to -17.9)
		Escherichia coli	4100 (3720 to 4530)	2290 (2080 to 2490)	-44.1 (-49.2 to -38.6)	120 (110 to 132)	78.8 (68.1 to 88.9)	-34.4 (-43.0 to -25.6)
		Fungus	2910 (2580 to 3290)	1330 (1190 to 1480)	-54.4 (-58.9 to -49.8)	70.2 (62.6 to 78.8)	41 (34.7 to 47.6)	-41.6 (-50.1 to -32.6)
		Group B Streptococcus	3330 (3000 to 3700)	1650 (1500 to 1810)	-50.4 (-55.3 to -45.6)	63.5 (57.6 to 69.9)	38.2 (33.3 to 43)	-39.8 (-47.6 to -30.8)
		Haemophilus influenzae	14100 (13100 to 15200)	5280 (4900 to 5670)	-62.5 (-65.5 to -59.6)	131 (123 to 139)	42.5 (37.2 to 47.8)	-67.5 (-71.8 to -63.2)
		Influenza	19100 (17900 to 20600)	163 (62.5 to 350)	-99.1 (-99.7 to -98.2)	388 (365 to 412)	3.99 (1.51 to 8.46)	-99.0 (-99.6 to -97.8)

		Klebsiella pneumoniae	9280 (8680 to 9960)	3450 (3180 to 3730)	-62.8 (-65.9 to -59.9)	246 (231 to 262)	114 (100 to 128)	-53.5 (-59.3 to -47.5)
		Legionella spp.	6820 (6150 to 7550)	3410 (3130 to 3710)	-49.9 (-54.8 to -45.1)	121 (109 to 134)	75.9 (66.2 to 85.7)	-37.3 (-46.4 to -27.5)
		Mycoplasma spp.	16100 (14900 to 17400)	5350 (4960 to 5710)	-66.8 (-69.4 to -64.3)	91.7 (85.2 to 98.6)	39.8 (34.7 to 44.8)	-56.6 (-62.6 to -50.4)
		Polymicrobial	547 (371 to 812)	247 (158 to 384)	-55.0 (-60.2 to -49.3)	18 (12.5 to 25.9)	8.99 (5.88 to 14.1)	-50.1 (-57.9 to -41.7)
		Pseudomonas aeruginosa	9200 (8520 to 9990)	4920 (4530 to 5320)	-46.4 (-51.2 to -42.2)	174 (162 to 186)	112 (98.5 to 127)	-35.6 (-44.2 to -26.9)
		Respiratory syncytial virus	10400 (9420 to 11500)	52.6 (19.9 to 112)	-99.5 (-99.8 to -98.9)	77 (72.6 to 81.8)	0.38 (0.15 to 0.83)	-99.5 (-99.8 to -98.9)
		Staphylococcus aureus	13400 (12600 to 14400)	8590 (7880 to 9310)	-36.0 (-41.2 to -30.9)	534 (502 to 567)	415 (363 to 466)	-22.2 (-32.1 to -11.5)
		Streptococcus pneumoniae	81700 (77100 to 87300)	16000 (14900 to 17200)	-80.4 (-81.8 to -79.0)	1100 (1040 to 1160)	289 (253 to 324)	-73.7 (-77.1 to -70.2)
		Other viruses	43200 (40400 to 46400)	13600 (12700 to 14500)	-68.4 (-70.7 to -66.2)	217 (206 to 229)	91.2 (80.4 to 103)	-58.0 (-63.3 to -52.5)
		Other bacterial pathogens	14500 (12900 to 16200)	5630 (4970 to 6390)	-61.1 (-64.7 to -57.6)	242 (217 to 268)	112 (95.8 to 129)	-53.6 (-59.8 to -47.1)
	Under 5	*Acinetobacter baumannii*	91 (63.1 to 130)	12.8 (8.39 to 19.2)	-85.9 (-88.2 to -83.5)	3.99 (2.43 to 6.38)	0.59 (0.33 to 0.98)	-85.3 (-88.6 to -82.2)
		Chlamydia spp.	1150 (975 to 1340)	180 (154 to 209)	-84.3 (-86.6 to -81.5)	6.97 (6.24 to 7.71)	0.9 (0.73 to 1.08)	-87.0 (-89.8 to -84.3)
		Enterobacter spp.	133 (102 to 169)	36.8 (28.7 to 47.2)	-72.2 (-76.6 to -67.6)	3.32 (2.51 to 4.44)	0.69 (0.49 to 0.95)	-79.3 (-83.5 to -74.8)
		Escherichia coli	432 (363 to 510)	129 (109 to 151)	-70.1 (-75.1 to -64.6)	10.4 (9.17 to 11.8)	2.2 (1.78 to 2.66)	-78.9 (-83.1 to -74.5)
		Fungus	81.7 (57.8 to 113)	18.6 (13.3 to 25.2)	-77.1 (-81.3 to -72.4)	1.38 (1 to 1.88)	0.23 (0.16 to 0.31)	-83.5 (-87.3 to -79.3)
		Group B Streptococcus	389 (326 to 466)	98.1 (82.9 to 116)	-74.7 (-78.5 to -70.3)	7.98 (7.08 to 8.99)	1.51 (1.22 to 1.81)	-81.1 (-84.8 to -77.4)
		Haemophilus influenzae	3390 (2930 to 3960)	608 (521 to 714)	-82.0 (-84.7 to -78.8)	25.5 (22.9 to 28.4)	2.11 (1.68 to 2.52)	-91.7 (-93.5 to -89.9)
		Influenza	2790 (2450 to 3230)	9.21 (3.48 to 20.1)	-99.7 (-99.9 to -99.3)	39.7 (36.2 to 43)	0.09 (0.03 to 0.2)	-99.8 (-99.9 to -99.5)
		Klebsiella pneumoniae	1570 (1360 to 1820)	218 (188 to 253)	-86.1 (-88.0 to -83.8)	30.8 (28 to 33.6)	3.43 (2.78 to 4.08)	-88.8 (-91.0 to -86.6)
		Legionella spp.	169 (123 to 233)	41.9 (30.3 to 56.5)	-75.1 (-78.7 to -71.1)	3.67 (2.8 to 4.7)	0.74 (0.54 to 0.97)	-79.9 (-83.9 to -75.7)
		Mycoplasma spp.	1910 (1640 to 2230)	559 (482 to 653)	-70.6 (-75.1 to -65.5)	9.8 (8.81 to 10.9)	2.03 (1.63 to 2.44)	-79.3 (-83.5 to -74.5)
		Polymicrobial	76.1 (44.8 to 115)	16.5 (9.87 to 26.1)	-78.2 (-80.9 to -75.0)	4.28 (2.37 to 6.9)	0.87 (0.44 to 1.5)	-79.7 (-84.1 to -74.9)
		Pseudomonas aeruginosa	979 (845 to 1140)	324 (281 to 378)	-66.8 (-71.7 to -61.2)	15.1 (13.6 to 16.6)	3.53 (2.87 to 4.19)	-76.5 (-81.3 to -71.8)
		Respiratory syncytial virus	7170 (6280 to 8260)	29.9 (11.1 to 65.2)	-99.6 (-99.8 to -99.1)	50.6 (47 to 54.7)	0.15 (0.06 to 0.32)	-99.7 (-99.9 to -99.4)

		Staphylococcus aureus	1170 (1030 to 1360)	338 (291 to 396)	-71.2 (-74.9 to -66.6)	32.1 (29.5 to 34.8)	6.71 (5.39 to 7.97)	-79.1 (-83.1 to -74.9)
		Streptococcus pneumoniae	18100 (15900 to 20900)	1250 (1080 to 1470)	-93.1 (-94.0 to -92.0)	187 (173 to 202)	9.22 (7.4 to 11)	-95.1 (-96.0 to -94.0)
		Other viruses	14600 (12800 to 16800)	2060 (1790 to 2390)	-85.9 (-87.7 to -83.6)	61.2 (56.7 to 66.3)	6.61 (5.34 to 7.88)	-89.2 (-91.3 to -87.0)
		Other bacterial pathogens	473 (371 to 587)	89.4 (71.8 to 111)	-81.1 (-84.3 to -77.2)	5.98 (4.8 to 7.39)	0.88 (0.65 to 1.15)	-85.3 (-88.4 to -82.1)
	5-14 years	*Acinetobacter baumannii*	214 (169 to 263)	26.5 (20.6 to 33.2)	-87.6 (-89.6 to -85.4)	0.81 (0.68 to 0.95)	0.12 (0.1 to 0.14)	-85.8 (-88.1 to -83.2)
		Chlamydia spp.	1040 (856 to 1280)	167 (131 to 209)	-84.0 (-86.5 to -81.4)	0.98 (0.86 to 1.11)	0.18 (0.15 to 0.21)	-81.7 (-84.5 to -78.2)
		Enterobacter spp.	105 (84.7 to 132)	31.8 (24.8 to 40.6)	-69.5 (-74.4 to -64.1)	0.38 (0.32 to 0.44)	0.13 (0.11 to 0.15)	-65.2 (-70.9 to -58.5)
		Escherichia coli	261 (215 to 317)	73.4 (57.5 to 91.1)	-71.9 (-76.5 to -67.1)	0.97 (0.85 to 1.09)	0.31 (0.27 to 0.36)	-67.9 (-72.8 to -61.9)
		Fungus	314 (257 to 383)	76.3 (59.1 to 96.2)	-75.7 (-79.6 to -71.6)	1.15 (0.98 to 1.31)	0.32 (0.27 to 0.38)	-72.2 (-76.6 to -67.1)
		Group B Streptococcus	334 (271 to 404)	89 (69.6 to 111)	-73.3 (-77.5 to -68.6)	0.88 (0.76 to 1)	0.27 (0.23 to 0.31)	-69.5 (-74.6 to -64.0)
		Haemophilus influenzae	1370 (1150 to 1660)	302 (239 to 375)	-78.0 (-81.5 to -74.3)	1.95 (1.73 to 2.14)	0.34 (0.29 to 0.39)	-82.7 (-85.4 to -79.6)
		Influenza	1930 (1620 to 2320)	8.58 (3.04 to 19.4)	-99.6 (-99.8 to -99.0)	5.36 (4.78 to 5.85)	0.03 (0.01 to 0.06)	-99.5 (-99.8 to -98.9)
		Klebsiella pneumoniae	766 (643 to 922)	151 (121 to 191)	-80.2 (-83.3 to -77.0)	2.83 (2.5 to 3.1)	0.64 (0.55 to 0.72)	-77.4 (-80.7 to -73.3)
		Legionella spp.	708 (584 to 859)	185 (146 to 230)	-73.9 (-78.2 to -69.2)	1.8 (1.56 to 2.05)	0.54 (0.46 to 0.61)	-70.2 (-74.8 to -64.5)
		Mycoplasma spp.	3020 (2510 to 3650)	588 (463 to 735)	-80.5 (-83.5 to -77.3)	2.97 (2.61 to 3.28)	0.66 (0.57 to 0.75)	-77.8 (-81.2 to -73.6)
		Polymicrobial	42.4 (26.6 to 64.3)	9.95 (5.85 to 15.9)	-76.6 (-80.6 to -72.5)	0.16 (0.1 to 0.25)	0.04 (0.03 to 0.07)	-73.3 (-77.9 to -68.1)
		Pseudomonas aeruginosa	771 (639 to 931)	216 (171 to 270)	-72.0 (-76.4 to -67.4)	1.98 (1.76 to 2.18)	0.63 (0.55 to 0.73)	-68.0 (-73.0 to -61.9)
		Respiratory syncytial virus	553 (458 to 665)	2.1 (0.75 to 4.72)	-99.6 (-99.9 to -99.2)	0.7 (0.62 to 0.78)	0 (0 to 0.01)	-99.6 (-99.8 to -99.1)
		Staphylococcus aureus	1170 (981 to 1400)	373 (295 to 462)	-68.0 (-73.0 to -62.8)	6.13 (5.47 to 6.69)	2.24 (1.95 to 2.54)	-63.5 (-69.0 to -56.6)
		Streptococcus pneumoniae	10900 (9070 to 13000)	1270 (1000 to 1580)	-88.3 (-90.1 to -86.5)	23.7 (21.3 to 25.8)	3.16 (2.75 to 3.6)	-86.7 (-88.6 to -84.2)
		Other viruses	5300 (4450 to 6350)	1110 (876 to 1370)	-79.1 (-82.3 to -75.7)	4.56 (4.08 to 4.96)	1.09 (0.95 to 1.23)	-76.1 (-79.6 to -71.6)
		Other bacterial pathogens	1130 (925 to 1360)	210 (164 to 266)	-81.3 (-84.4 to -78.0)	2.35 (2.02 to 2.66)	0.5 (0.43 to 0.6)	-78.7 (-82.0 to -74.8)
	15-49 years	*Acinetobacter baumannii*	256 (218 to 300)	53.7 (45.6 to 62.8)	-79.0 (-81.2 to -76.6)	4.5 (3.81 to 5.33)	1.5 (1.22 to 1.79)	-66.6 (-72.8 to -60.5)
		Chlamydia spp.	1250 (1110 to 1400)	338 (296 to 381)	-72.9 (-75.7 to -69.9)	5.46 (4.84 to 6.23)	2.34 (1.94 to 2.76)	-57.1 (-65.0 to -48.9)

		Enterobacter spp.	125 (107 to 145)	64.5 (55.4 to 73.9)	-48.5 (-54.0 to -42.5)	2.09 (1.78 to 2.46)	1.7 (1.41 to 2.05)	-18.3 (-33.4 to -1.7)
		Escherichia coli	313 (278 to 351)	149 (131 to 167)	-52.4 (-57.1 to -47.2)	5.4 (4.75 to 6.08)	4.06 (3.39 to 4.75)	-24.5 (-38.3 to -10.0)
		Fungus	377 (329 to 427)	155 (135 to 177)	-58.9 (-63.2 to -54.0)	6.41 (5.5 to 7.35)	4.17 (3.42 to 4.91)	-34.8 (-47.0 to -21.8)
		Group B Streptococcus	400 (350 to 460)	180 (156 to 205)	-54.8 (-59.8 to -49.6)	4.9 (4.22 to 5.62)	3.5 (2.9 to 4.12)	-28.3 (-41.6 to -14.3)
		Haemophilus influenzae	1640 (1500 to 1800)	612 (547 to 681)	-62.8 (-66.0 to -59.2)	10.8 (9.72 to 12)	4.4 (3.7 to 5.11)	-59.3 (-66.6 to -51.8)
		Influenza	2320 (2120 to 2520)	17.3 (6.6 to 37.4)	-99.3 (-99.7 to -98.4)	29.8 (27.1 to 32.7)	0.34 (0.13 to 0.72)	-98.9 (-99.6 to -97.6)
		Klebsiella pneumoniae	917 (835 to 1000)	306 (273 to 342)	-66.6 (-69.4 to -63.5)	15.7 (14.2 to 17.4)	8.33 (7.01 to 9.65)	-47.0 (-56.5 to -37.2)
		Legionella spp.	849 (747 to 957)	374 (332 to 419)	-55.9 (-60.2 to -50.8)	10 (8.84 to 11.4)	6.99 (5.92 to 8.11)	-30.0 (-42.9 to -16.5)
		Mycoplasma spp.	3620 (3280 to 3970)	1190 (1070 to 1320)	-67.0 (-70.1 to -63.6)	16.5 (14.8 to 18.3)	8.6 (7.16 to 9.98)	-47.7 (-57.0 to -38.2)
		Polymicrobial	50.9 (32.3 to 77.8)	20.2 (12.3 to 32)	-60.4 (-64.9 to -55.6)	0.89 (0.57 to 1.39)	0.56 (0.33 to 0.89)	-37.3 (-49.1 to -25.1)
		Pseudomonas aeruginosa	923 (834 to 1020)	437 (391 to 490)	-52.6 (-56.9 to -47.9)	11 (9.81 to 12.2)	8.25 (7.01 to 9.56)	-24.8 (-38.5 to -10.5)
		Respiratory syncytial virus	662 (593 to 731)	4.24 (1.58 to 8.93)	-99.4 (-99.8 to -98.6)	3.89 (3.47 to 4.37)	0.04 (0.01 to 0.08)	-99.0 (-99.6 to -97.9)
		Staphylococcus aureus	1400 (1270 to 1520)	757 (677 to 840)	-45.9 (-50.5 to -40.7)	34.1 (31 to 37.3)	29.2 (24.9 to 33.9)	-14.2 (-29.5 to 1.1)
		Streptococcus pneumoniae	13000 (11900 to 14000)	2570 (2310 to 2850)	-80.2 (-81.9 to -78.3)	132 (120 to 144)	41.3 (35 to 47.6)	-68.6 (-74.3 to -62.9)
		Other viruses	6350 (5810 to 6860)	2250 (2020 to 2480)	-64.6 (-67.5 to -61.1)	25.3 (23 to 27.8)	14.2 (12 to 16.4)	-43.9 (-53.8 to -33.6)
		Other bacterial pathogens	1350 (1180 to 1520)	426 (365 to 492)	-68.4 (-71.4 to -64.9)	13.1 (11.5 to 14.9)	6.54 (5.43 to 7.76)	-49.8 (-58.8 to -40.1)
	50-69 years	*Acinetobacter baumannii*	1160 (1000 to 1340)	250 (214 to 286)	-78.4 (-80.8 to -76.0)	25.6 (21.8 to 30.1)	6.93 (5.76 to 8.23)	-72.8 (-77.7 to -67.6)
		Chlamydia spp.	3350 (2990 to 3750)	917 (811 to 1020)	-72.6 (-75.4 to -69.7)	18.4 (16.2 to 20.9)	6.32 (5.3 to 7.4)	-65.5 (-71.6 to -58.8)
		Enterobacter spp.	551 (475 to 638)	288 (253 to 325)	-47.7 (-54.1 to -41.8)	11.6 (9.83 to 13.4)	7.58 (6.4 to 9.02)	-34.1 (-46.5 to -20.3)
		Escherichia coli	1520 (1360 to 1690)	719 (642 to 802)	-52.6 (-57.5 to -47.5)	32.9 (29 to 37.2)	19.6 (16.5 to 22.8)	-40.3 (-50.6 to -29.0)
		Fungus	1460 (1280 to 1650)	609 (532 to 689)	-58.1 (-62.7 to -53.2)	31.1 (27.1 to 35.9)	16.3 (13.6 to 19.3)	-47.3 (-56.7 to -36.3)
		Group B Streptococcus	1240 (1090 to 1410)	566 (499 to 637)	-54.4 (-59.6 to -48.9)	19.1 (16.6 to 21.9)	10.9 (9.18 to 12.8)	-42.6 (-53.1 to -30.6)
		Haemophilus influenzae	4370 (3970 to 4770)	1680 (1510 to 1840)	-61.6 (-65.3 to -57.8)	36.1 (32.3 to 40.3)	12 (10.2 to 13.9)	-66.6 (-72.5 to -60.5)
		Influenza	5300 (4860 to 5770)	41.4 (16 to 88.2)	-99.2 (-99.7 to -98.3)	85.7 (77.1 to 94.8)	0.81 (0.3 to 1.75)	-99.0 (-99.6 to -98.0)

		Klebsiella pneumoniae	3120 (2860 to 3410)	1100 (991 to 1220)	-64.6 (-68.2 to -61.2)	67.3 (60.5 to 74.3)	29.9 (25.4 to 34.5)	-55.5 (-63.1 to -47.3)
		Legionella spp.	3170 (2820 to 3550)	1410 (1270 to 1570)	-55.4 (-60.5 to -50.1)	47 (41 to 53.5)	26.3 (22.3 to 30.4)	-43.8 (-53.9 to -33.1)
		Mycoplasma spp.	4410 (4000 to 4840)	1390 (1250 to 1530)	-68.4 (-71.8 to -65.2)	25.2 (22.4 to 28.2)	9.99 (8.44 to 11.5)	-60.3 (-67.3 to -52.4)
		Polymicrobial	192 (123 to 293)	77.8 (47.8 to 124)	-59.6 (-64.8 to -54.7)	4.21 (2.68 to 6.55)	2.14 (1.32 to 3.44)	-49.1 (-58.3 to -39.4)
		Pseudomonas aeruginosa	3480 (3150 to 3830)	1620 (1460 to 1790)	-53.3 (-58.1 to -48.9)	52 (46.3 to 58.3)	30.5 (25.8 to 35.2)	-41.2 (-51.4 to -30.1)
		Respiratory syncytial virus	1120 (991 to 1260)	7.14 (2.69 to 15.1)	-99.4 (-99.7 to -98.6)	8.27 (7.25 to 9.44)	0.06 (0.02 to 0.14)	-99.2 (-99.7 to -98.3)
		Staphylococcus aureus	4630 (4260 to 5030)	2560 (2310 to 2810)	-44.7 (-50.2 to -39.3)	142 (127 to 156)	98.5 (83.5 to 113)	-30.4 (-42.4 to -17.2)
		Streptococcus pneumoniae	21300 (19700 to 22900)	4520 (4090 to 4970)	-78.7 (-80.8 to -76.7)	271 (245 to 298)	72.4 (61.4 to 83.2)	-73.2 (-77.7 to -68.3)
		Other viruses	9090 (8380 to 9850)	3410 (3080 to 3760)	-62.4 (-66.2 to -58.8)	45.5 (41.1 to 50.4)	21.5 (18.3 to 24.7)	-52.7 (-60.6 to -44.0)
		Other bacterial pathogens	5410 (4770 to 6080)	1720 (1480 to 1970)	-68.1 (-71.8 to -64.9)	65.8 (57.2 to 75.1)	26.4 (21.8 to 31.1)	-59.8 (-66.9 to -52.1)
	70+ years	*Acinetobacter baumannii*	977 (835 to 1160)	344 (293 to 402)	-64.8 (-69.3 to -60.0)	44.6 (38.6 to 50.9)	15.1 (12.9 to 17.7)	-66.0 (-70.9 to -60.8)
		Chlamydia spp.	3370 (2960 to 3850)	1500 (1320 to 1740)	-55.2 (-61.1 to -49.3)	38.2 (34.2 to 42.3)	16.5 (14.3 to 18.8)	-56.8 (-62.7 to -50.1)
		Enterobacter spp.	453 (389 to 541)	386 (333 to 451)	-14.5 (-25.8 to -2.8)	19.6 (16.9 to 22.9)	16.1 (13.9 to 18.7)	-17.4 (-29.0 to -4.1)
		Escherichia coli	1570 (1400 to 1810)	1220 (1070 to 1390)	-22.5 (-31.7 to -12.6)	70.4 (63.2 to 78.4)	52.6 (45.8 to 59.6)	-25.2 (-35.1 to -14.3)
		Fungus	684 (587 to 798)	467 (402 to 546)	-31.6 (-40.6 to -22.8)	30.2 (26.2 to 34.1)	19.9 (17.1 to 23.1)	-34.0 (-42.9 to -23.4)
		Group B Streptococcus	963 (845 to 1110)	715 (627 to 823)	-25.5 (-35.5 to -15.3)	30.6 (27.3 to 34.4)	22 (19 to 24.9)	-28.1 (-37.9 to -16.7)
		Haemophilus influenzae	3320 (3000 to 3750)	2080 (1850 to 2370)	-37.2 (-44.9 to -29.8)	56.7 (51.6 to 61.6)	23.7 (20.7 to 26.8)	-58.2 (-63.7 to -52.1)
		Influenza	6800 (6150 to 7670)	86.8 (33 to 189)	-98.7 (-99.5 to -97.3)	227 (210 to 245)	2.71 (1.01 to 5.76)	-98.8 (-99.6 to -97.4)
		Klebsiella pneumoniae	2900 (2610 to 3290)	1670 (1490 to 1900)	-42.2 (-49.3 to -35.2)	129 (119 to 140)	71.9 (63.1 to 80.6)	-44.2 (-51.2 to -36.8)
		Legionella spp.	1920 (1680 to 2230)	1400 (1240 to 1590)	-27.1 (-36.6 to -17.2)	58.9 (52.4 to 65.8)	41.4 (36 to 46.6)	-29.6 (-39.1 to -18.4)
		Mycoplasma spp.	3140 (2810 to 3590)	1620 (1430 to 1860)	-48.4 (-54.7 to -42.1)	37.2 (33.7 to 40.9)	18.5 (16.1 to 20.9)	-50.2 (-56.8 to -43.0)
		Polymicrobial	186 (115 to 291)	123 (74.2 to 200)	-33.9 (-42.8 to -24.3)	8.41 (5.34 to 13.2)	5.37 (3.32 to 8.66)	-36.2 (-45.3 to -26.8)
		Pseudomonas aeruginosa	3050 (2740 to 3480)	2330 (2070 to 2650)	-23.7 (-33.3 to -14.8)	94.3 (86.3 to 102)	69.4 (60.8 to 78.4)	-26.3 (-35.6 to -15.8)
		Respiratory syncytial virus	887 (782 to 1010)	9.22 (3.57 to 20.1)	-99.0 (-99.6 to -97.8)	13.5 (12.2 to 15.1)	0.13 (0.05 to 0.28)	-99.0 (-99.6 to -97.9)

		Staphylococcus aureus	5060 (4600 to 5700)	4560 (4080 to 5190)	-9.7 (-20.3 to 1.0)	320 (296 to 345)	279 (245 to 312)	-12.8 (-23.9 to -0.4)
		Streptococcus pneumoniae	18500 (16700 to 20800)	6410 (5720 to 7280)	-65.2 (-69.4 to -61.1)	486 (452 to 523)	163 (143 to 183)	-66.4 (-70.7 to -61.6)
		Other viruses	7800 (7070 to 8790)	4780 (4260 to 5440)	-38.6 (-45.8 to -31.3)	80.7 (74.7 to 87.1)	47.8 (42.1 to 53.7)	-40.7 (-47.9 to -32.3)
		Other bacterial pathogens	6110 (5360 to 7090)	3180 (2750 to 3680)	-47.9 (-54.5 to -41.4)	155 (138 to 174)	78 (66.6 to 90.1)	-49.7 (-56.6 to -42.6)
Croatia	All Ages	*Acinetobacter baumannii*	497 (428 to 573)	284 (244 to 327)	-42.9 (-48.9 to -36.3)	11.3 (9.82 to 12.9)	12.6 (10.6 to 14.6)	11.3 (-3.4 to 26.7)
		Chlamydia spp.	2160 (1960 to 2390)	908 (829 to 1000)	-57.9 (-62.1 to -53.5)	11.5 (10.5 to 12.6)	9.54 (8.33 to 10.6)	-17.2 (-27.8 to -5.8)
		Enterobacter spp.	362 (319 to 412)	323 (286 to 363)	-10.6 (-20.8 to 0.4)	7.48 (6.56 to 8.46)	13.1 (11.2 to 15)	75.7 (51.8 to 100.7)
		Escherichia coli	991 (890 to 1100)	706 (645 to 784)	-28.6 (-36.1 to -20.5)	22.5 (20.4 to 24.7)	31.3 (27.4 to 34.7)	39.1 (21.0 to 57.2)
		Fungus	709 (626 to 797)	456 (413 to 511)	-35.5 (-42.6 to -28.2)	12.7 (11.3 to 14.1)	16.3 (14.3 to 18.5)	29.0 (12.2 to 46.8)
		Group B Streptococcus	829 (747 to 927)	626 (572 to 687)	-24.4 (-32.5 to -16.3)	12 (10.9 to 13.2)	17.7 (15.4 to 19.6)	47.2 (28.2 to 67.7)
		Haemophilus influenzae	3550 (3280 to 3850)	1600 (1480 to 1750)	-54.9 (-58.8 to -51.2)	24.7 (23.1 to 26.3)	15.6 (13.7 to 17.2)	-37.1 (-45.0 to -29.7)
		Influenza	4880 (4520 to 5300)	59.1 (22.6 to 126)	-98.8 (-99.5 to -97.4)	77.7 (72.1 to 82.8)	1.82 (0.66 to 3.89)	-97.7 (-99.2 to -94.9)
		Klebsiella pneumoniae	2180 (2030 to 2360)	1430 (1320 to 1570)	-34.1 (-39.9 to -28.2)	44.3 (41.3 to 47)	59.5 (52.4 to 65.7)	34.4 (18.5 to 49.7)
		Legionella spp.	2090 (1880 to 2320)	1530 (1410 to 1680)	-26.6 (-34.8 to -18.1)	28 (25.4 to 30.9)	40.9 (36.1 to 45.2)	46.2 (26.8 to 67.6)
		Mycoplasma spp.	4050 (3710 to 4440)	1940 (1800 to 2100)	-51.9 (-55.9 to -47.8)	17.6 (16.2 to 18.9)	16.4 (14.3 to 18)	-7.0 (-18.1 to 4.7)
		Polymicrobial	118 (75.4 to 184)	73.5 (42.8 to 119)	-38.1 (-46.4 to -29.2)	3.02 (2.06 to 4.47)	3.37 (2.03 to 5.5)	10.6 (-8.9 to 32.6)
		Pseudomonas aeruginosa	2670 (2450 to 2940)	2650 (2440 to 2910)	-0.9 (-10.3 to 8.5)	38.9 (35.9 to 41.8)	76.1 (66.6 to 84)	95.7 (72.4 to 119.2)
		Respiratory syncytial virus	2600 (2320 to 2900)	28.3 (10.9 to 60.7)	-98.9 (-99.6 to -97.7)	12.7 (11.9 to 13.7)	0.22 (0.08 to 0.47)	-98.3 (-99.4 to -96.3)
		Staphylococcus aureus	3620 (3360 to 3930)	3580 (3290 to 3950)	-1.1 (-9.8 to 7.1)	113 (104 to 120)	221 (194 to 242)	95.9 (73.2 to 116.9)
		Streptococcus pneumoniae	19600 (18300 to 21300)	8030 (7460 to 8710)	-59.0 (-61.9 to -56.2)	201 (188 to 211)	168 (149 to 185)	-16.3 (-25.9 to -7.4)
		Other viruses	11800 (11000 to 12900)	7220 (6700 to 7810)	-39.0 (-43.1 to -35.1)	44.2 (41.7 to 46.5)	56 (49.5 to 61.5)	26.7 (12.3 to 39.6)
		Other bacterial pathogens	3500 (3080 to 3970)	1930 (1690 to 2230)	-44.8 (-50.4 to -38.8)	46 (40.6 to 51.7)	49.8 (41.9 to 58.2)	8.3 (-5.2 to 21.8)
	Under 5	*Acinetobacter baumannii*	13.2 (8.92 to 19.5)	2.94 (2.04 to 4.29)	-77.6 (-80.6 to -74.3)	0.48 (0.26 to 0.81)	0.13 (0.06 to 0.23)	-73.5 (-81.2 to -64.3)
		Chlamydia spp.	178 (149 to 212)	41.8 (34.4 to 51)	-76.5 (-79.6 to -73.1)	0.69 (0.6 to 0.8)	0.14 (0.11 to 0.19)	-79.0 (-84.7 to -72.1)

		Enterobacter spp.	25.5 (19.3 to 33.1)	11.2 (8.17 to 14.8)	-56.2 (-62.7 to -49.3)	0.42 (0.3 to 0.61)	0.14 (0.09 to 0.22)	-65.9 (-75.5 to -54.7)
		Escherichia coli	75.7 (62.4 to 91.3)	27.2 (21.8 to 33.3)	-64.0 (-68.8 to -58.3)	1.16 (0.99 to 1.36)	0.33 (0.24 to 0.44)	-71.4 (-79.4 to -62.6)
		Fungus	14.3 (9.87 to 19.8)	5.3 (3.66 to 7.47)	-62.7 (-68.1 to -56.7)	0.12 (0.09 to 0.16)	0.03 (0.02 to 0.04)	-76.7 (-83.6 to -69.0)
		Group B Streptococcus	70.7 (58.2 to 87)	27.7 (22.3 to 34.8)	-60.8 (-66.2 to -54.7)	0.98 (0.84 to 1.13)	0.3 (0.22 to 0.39)	-69.1 (-77.6 to -59.2)
		Haemophilus influenzae	649 (537 to 773)	141 (113 to 174)	-78.2 (-81.2 to -74.9)	2.7 (2.34 to 3.1)	0.22 (0.16 to 0.28)	-91.8 (-94.2 to -89.3)
		Influenza	524 (440 to 622)	3.15 (1.24 to 6.98)	-99.4 (-99.8 to -98.7)	4 (3.51 to 4.54)	0.01 (0.01 to 0.03)	-99.6 (-99.9 to -99.2)
		Klebsiella pneumoniae	273 (230 to 327)	73.5 (59.9 to 90)	-73.1 (-76.3 to -69.7)	2.99 (2.61 to 3.43)	0.61 (0.45 to 0.77)	-79.6 (-85.1 to -73.2)
		Legionella spp.	36.1 (25.8 to 49.4)	13.2 (9.15 to 18.6)	-63.5 (-68.5 to -57.8)	0.53 (0.4 to 0.69)	0.18 (0.12 to 0.25)	-65.3 (-75.3 to -53.8)
		Mycoplasma spp.	357 (296 to 426)	125 (101 to 153)	-65.1 (-69.7 to -60.2)	0.99 (0.85 to 1.14)	0.23 (0.17 to 0.3)	-76.7 (-83.1 to -69.2)
		Polymicrobial	14.2 (8.11 to 22.9)	3.67 (2.1 to 5.99)	-74.1 (-77.4 to -70.4)	0.68 (0.35 to 1.13)	0.25 (0.11 to 0.46)	-62.6 (-74.4 to -48.6)
		Pseudomonas aeruginosa	206 (173 to 247)	109 (88.8 to 134)	-46.9 (-54.3 to -39.3)	1.87 (1.63 to 2.16)	0.67 (0.49 to 0.87)	-64.1 (-73.8 to -52.8)
		Respiratory syncytial virus	1590 (1350 to 1880)	13.5 (5.19 to 30.1)	-99.2 (-99.7 to -98.2)	6.33 (5.58 to 7.15)	0.03 (0.01 to 0.07)	-99.5 (-99.8 to -98.9)
		Staphylococcus aureus	229 (193 to 273)	111 (89.8 to 135)	-51.5 (-56.7 to -45.9)	3.46 (3.06 to 3.92)	1.11 (0.82 to 1.42)	-67.7 (-76.3 to -57.8)
		Streptococcus pneumoniae	3330 (2780 to 3950)	738 (603 to 897)	-77.8 (-80.3 to -75.3)	17.8 (15.7 to 20)	2.68 (1.98 to 3.43)	-84.9 (-88.9 to -80.3)
		Other viruses	3190 (2670 to 3780)	998 (818 to 1220)	-68.7 (-72.1 to -65.1)	6.97 (6.15 to 7.86)	1.5 (1.11 to 1.91)	-78.5 (-84.2 to -72.0)
		Other bacterial pathogens	81.1 (63.1 to 104)	21.6 (16.2 to 28.1)	-73.3 (-77.0 to -69.4)	0.6 (0.46 to 0.8)	0.13 (0.08 to 0.19)	-78.3 (-84.6 to -71.4)
	5-14 years	*Acinetobacter baumannii*	34.2 (25.1 to 45.5)	11.6 (8.73 to 15.2)	-65.9 (-69.8 to -61.6)	0.08 (0.07 to 0.1)	0.02 (0.01 to 0.02)	-81.7 (-85.8 to -77.0)
		Chlamydia spp.	199 (149 to 261)	53 (39.8 to 67.9)	-73.3 (-76.1 to -70.2)	0.12 (0.1 to 0.14)	0.02 (0.01 to 0.02)	-85.7 (-88.9 to -81.8)
		Enterobacter spp.	24.6 (18 to 32.7)	13.7 (10.2 to 18.1)	-44.2 (-50.8 to -36.9)	0.06 (0.05 to 0.07)	0.02 (0.01 to 0.02)	-70.1 (-76.7 to -62.3)
		Escherichia coli	55.9 (42.1 to 73.3)	23.9 (18.1 to 30.8)	-57.1 (-62.0 to -51.9)	0.14 (0.11 to 0.16)	0.03 (0.02 to 0.04)	-77.0 (-82.1 to -70.5)
		Fungus	67.2 (50 to 88.9)	29.4 (22.3 to 38.2)	-56.1 (-60.9 to -50.7)	0.16 (0.14 to 0.19)	0.04 (0.03 to 0.05)	-76.5 (-81.8 to -70.2)
		Group B Streptococcus	74.8 (55.4 to 97.5)	37.1 (28.1 to 47.9)	-50.2 (-56.2 to -43.7)	0.13 (0.11 to 0.15)	0.03 (0.03 to 0.04)	-73.3 (-79.4 to -66.2)
		Haemophilus influenzae	324 (247 to 420)	98.7 (75.2 to 128)	-69.5 (-72.5 to -66.3)	0.3 (0.26 to 0.34)	0.03 (0.03 to 0.04)	-88.7 (-91.2 to -85.8)
		Influenza	446 (335 to 573)	3.28 (1.15 to 7.26)	-99.3 (-99.7 to -98.4)	0.8 (0.7 to 0.92)	0 (0 to 0.01)	-99.6 (-99.9 to -99.1)

		Klebsiella pneumoniae	164 (124 to 214)	64.9 (49.7 to 83.2)	-60.2 (-64.0 to -56.2)	0.39 (0.34 to 0.45)	0.08 (0.07 to 0.1)	-78.7 (-83.4 to -73.2)
		Legionella spp.	190 (142 to 249)	90.6 (69.4 to 116)	-52.2 (-57.7 to -46.6)	0.31 (0.27 to 0.37)	0.08 (0.06 to 0.1)	-74.3 (-80.3 to -67.3)
		Mycoplasma spp.	698 (526 to 898)	248 (191 to 323)	-64.3 (-68.0 to -60.1)	0.44 (0.38 to 0.52)	0.08 (0.07 to 0.1)	-80.8 (-85.1 to -76.0)
		Polymicrobial	8.06 (4.7 to 12.8)	3.15 (1.8 to 5.16)	-61.0 (-65.5 to -55.6)	0.02 (0.01 to 0.03)	0 (0 to 0.01)	-79.1 (-83.7 to -73.3)
		Pseudomonas aeruginosa	200 (152 to 259)	125 (95.2 to 160)	-37.6 (-43.8 to -30.8)	0.33 (0.28 to 0.38)	0.11 (0.09 to 0.13)	-66.5 (-73.7 to -58.0)
		Respiratory syncytial virus	152 (114 to 197)	1.49 (0.51 to 3.34)	-99.0 (-99.6 to -97.9)	0.12 (0.11 to 0.14)	0 (0 to 0)	-99.5 (-99.8 to -98.8)
		Staphylococcus aureus	279 (212 to 360)	164 (125 to 211)	-41.3 (-46.4 to -35.7)	0.95 (0.82 to 1.09)	0.3 (0.24 to 0.36)	-68.5 (-75.4 to -60.7)
		Streptococcus pneumoniae	2440 (1860 to 3160)	691 (529 to 890)	-71.7 (-74.2 to -69.1)	3.46 (3.01 to 3.97)	0.52 (0.43 to 0.63)	-84.8 (-88.1 to -81.0)
		Other viruses	1420 (1080 to 1830)	637 (489 to 822)	-55.0 (-59.0 to -50.8)	0.79 (0.69 to 0.91)	0.19 (0.15 to 0.23)	-75.8 (-81.1 to -69.9)
		Other bacterial pathogens	237 (178 to 309)	74.7 (56.3 to 97.3)	-68.4 (-71.7 to -64.6)	0.32 (0.27 to 0.38)	0.05 (0.04 to 0.07)	-83.0 (-86.7 to -78.8)
	15-49 years	*Acinetobacter baumannii*	45.9 (37.6 to 55.2)	16.7 (13.6 to 20)	-63.5 (-67.5 to -59.5)	0.65 (0.54 to 0.75)	0.24 (0.2 to 0.29)	-62.2 (-68.2 to -55.5)
		Chlamydia spp.	267 (232 to 307)	76.1 (65.5 to 88.4)	-71.4 (-74.3 to -68.4)	0.94 (0.84 to 1.05)	0.28 (0.23 to 0.32)	-70.4 (-75.4 to -65.5)
		Enterobacter spp.	33 (27.7 to 38.8)	19.7 (16.5 to 23.5)	-40.4 (-46.8 to -33.7)	0.44 (0.38 to 0.51)	0.27 (0.23 to 0.32)	-38.3 (-48.5 to -27.2)
		Escherichia coli	75 (64.7 to 85.9)	34.3 (29.6 to 39.3)	-54.2 (-59.1 to -49.2)	1.04 (0.93 to 1.16)	0.49 (0.43 to 0.56)	-52.6 (-60.4 to -44.7)
		Fungus	90.1 (76.8 to 105)	42.2 (35.7 to 48.7)	-53.1 (-58.1 to -47.6)	1.23 (1.08 to 1.4)	0.6 (0.5 to 0.69)	-51.5 (-59.1 to -43.3)
		Group B Streptococcus	100 (86.4 to 118)	53.3 (45.9 to 62.2)	-46.8 (-52.9 to -40.7)	0.99 (0.86 to 1.13)	0.54 (0.46 to 0.62)	-44.9 (-53.8 to -34.8)
		Haemophilus influenzae	434 (380 to 494)	142 (123 to 162)	-67.4 (-70.4 to -64.2)	2.3 (2.1 to 2.5)	0.53 (0.46 to 0.6)	-76.8 (-80.2 to -73.0)
		Influenza	598 (528 to 678)	4.7 (1.79 to 10.1)	-99.2 (-99.7 to -98.3)	6.19 (5.7 to 6.71)	0.05 (0.02 to 0.1)	-99.2 (-99.7 to -98.3)
		Klebsiella pneumoniae	219 (193 to 247)	93.2 (80.9 to 106)	-57.5 (-61.0 to -53.9)	3.02 (2.78 to 3.32)	1.33 (1.16 to 1.5)	-56.0 (-62.3 to -48.9)
		Legionella spp.	255 (222 to 293)	130 (114 to 148)	-48.9 (-54.7 to -43.0)	2.42 (2.16 to 2.71)	1.28 (1.11 to 1.45)	-47.1 (-55.7 to -37.2)
		Mycoplasma spp.	936 (827 to 1060)	357 (311 to 408)	-61.9 (-65.5 to -58.0)	3.43 (3.13 to 3.74)	1.35 (1.17 to 1.52)	-60.5 (-66.5 to -53.6)
		Polymicrobial	10.8 (6.5 to 17.2)	4.54 (2.59 to 7.43)	-58.3 (-63.3 to -53.2)	0.15 (0.09 to 0.24)	0.07 (0.04 to 0.11)	-56.9 (-64.0 to -49.1)
		Pseudomonas aeruginosa	268 (235 to 307)	179 (156 to 205)	-33.3 (-39.7 to -26.4)	2.57 (2.32 to 2.83)	1.77 (1.54 to 1.99)	-30.9 (-41.8 to -19.5)
		Respiratory syncytial virus	203 (178 to 231)	2.13 (0.79 to 4.58)	-99.0 (-99.6 to -97.7)	0.96 (0.87 to 1.06)	0.01 (0 to 0.02)	-98.9 (-99.6 to -97.7)

		Staphylococcus aureus	374 (330 to 422)	235 (205 to 267)	-37.2 (-42.4 to -32.2)	7.33 (6.77 to 7.93)	4.75 (4.13 to 5.32)	-35.0 (-44.6 to -24.9)
		Streptococcus pneumoniae	3280 (2900 to 3690)	992 (872 to 1130)	-69.7 (-72.1 to -67.2)	26.7 (24.8 to 28.9)	8.36 (7.31 to 9.37)	-68.7 (-73.2 to -63.9)
		Other viruses	1900 (1690 to 2130)	914 (799 to 1040)	-51.9 (-55.8 to -48.0)	6.1 (5.64 to 6.58)	3.03 (2.65 to 3.38)	-50.2 (-57.5 to -42.7)
		Other bacterial pathogens	318 (267 to 372)	107 (89.2 to 128)	-66.2 (-69.5 to -62.7)	2.47 (2.16 to 2.83)	0.86 (0.72 to 1.01)	-65.0 (-70.3 to -59.5)
	50-69 years	*Acinetobacter baumannii*	216 (183 to 254)	84.9 (71.4 to 99.8)	-60.6 (-65.9 to -55.4)	2.87 (2.46 to 3.31)	1.88 (1.56 to 2.22)	-34.3 (-45.2 to -21.7)
		Chlamydia spp.	742 (654 to 847)	220 (195 to 250)	-70.3 (-74.1 to -66.8)	2.45 (2.18 to 2.74)	1.21 (1.03 to 1.39)	-50.5 (-58.6 to -40.8)
		Enterobacter spp.	151 (130 to 175)	95.3 (82.3 to 109)	-36.6 (-45.6 to -27.8)	1.9 (1.65 to 2.17)	2 (1.67 to 2.36)	5.7 (-12.8 to 26.0)
		Escherichia coli	378 (333 to 428)	181 (162 to 204)	-52.0 (-58.5 to -45.2)	4.93 (4.42 to 5.45)	3.94 (3.37 to 4.48)	-20.0 (-33.2 to -5.2)
		Fungus	361 (311 to 415)	181 (158 to 205)	-49.9 (-57.0 to -43.2)	4.65 (4.07 to 5.26)	3.87 (3.31 to 4.49)	-16.6 (-30.4 to -1.5)
		Group B Streptococcus	324 (282 to 376)	180 (160 to 205)	-44.1 (-51.7 to -36.9)	3 (2.65 to 3.39)	2.78 (2.37 to 3.23)	-6.9 (-22.4 to 10.3)
		Haemophilus influenzae	1200 (1070 to 1340)	437 (391 to 489)	-63.4 (-67.7 to -59.1)	5.95 (5.46 to 6.42)	2.49 (2.15 to 2.84)	-58.1 (-64.8 to -50.7)
		Influenza	1420 (1280 to 1580)	12 (4.54 to 25.8)	-99.2 (-99.7 to -98.2)	13.8 (12.7 to 15)	0.19 (0.07 to 0.4)	-98.6 (-99.5 to -97.0)
		Klebsiella pneumoniae	775 (697 to 868)	378 (340 to 422)	-51.1 (-56.9 to -45.6)	10 (9.22 to 10.9)	8.16 (7.06 to 9.33)	-18.5 (-31.2 to -5.0)
		Legionella spp.	988 (871 to 1130)	536 (480 to 597)	-45.6 (-52.9 to -38.2)	8.82 (7.87 to 9.8)	7.97 (6.83 to 9.09)	-9.4 (-25.5 to 8.3)
		Mycoplasma spp.	1180 (1050 to 1320)	454 (407 to 508)	-61.5 (-66.3 to -57.2)	4.07 (3.69 to 4.47)	2.61 (2.23 to 2.99)	-35.8 (-46.1 to -24.3)
		Polymicrobial	42.4 (25.7 to 67.4)	19 (11 to 32.5)	-55.3 (-62.2 to -48.3)	0.56 (0.34 to 0.89)	0.42 (0.24 to 0.69)	-25.5 (-38.7 to -10.6)
		Pseudomonas aeruginosa	1050 (930 to 1180)	731 (655 to 816)	-30.1 (-38.6 to -21.3)	9.43 (8.62 to 10.3)	11 (9.49 to 12.4)	16.5 (-2.8 to 36.4)
		Respiratory syncytial virus	358 (314 to 405)	3.92 (1.48 to 8.35)	-98.9 (-99.6 to -97.6)	1.59 (1.42 to 1.78)	0.03 (0.01 to 0.06)	-98.2 (-99.4 to -96.2)
		Staphylococcus aureus	1290 (1150 to 1430)	863 (778 to 965)	-32.8 (-40.8 to -25.7)	23.7 (21.9 to 25.5)	26.5 (23 to 30.1)	12.0 (-5.2 to 30.6)
		Streptococcus pneumoniae	5570 (5030 to 6200)	1850 (1670 to 2060)	-66.7 (-70.5 to -63.3)	42.7 (39.4 to 46)	23.6 (20.5 to 26.6)	-44.6 (-53.3 to -35.5)
		Other viruses	2830 (2550 to 3140)	1550 (1400 to 1730)	-45.0 (-51.4 to -39.3)	8.52 (7.86 to 9.15)	7.79 (6.75 to 8.81)	-8.3 (-22.6 to 6.8)
		Other bacterial pathogens	1320 (1130 to 1520)	474 (404 to 551)	-64.0 (-68.9 to -59.8)	9.68 (8.53 to 10.9)	5.79 (4.76 to 6.83)	-40.0 (-49.7 to -29.6)
	70+ years	*Acinetobacter baumannii*	188 (159 to 223)	168 (141 to 196)	-10.8 (-21.2 to 0.7)	7.21 (6.2 to 8.36)	10.3 (8.66 to 12)	42.9 (24.5 to 62.2)
		Chlamydia spp.	772 (675 to 881)	518 (458 to 591)	-32.8 (-40.9 to -24.9)	7.35 (6.51 to 8.13)	7.89 (6.85 to 8.85)	7.7 (-5.9 to 22.7)

		Enterobacter spp.	128 (109 to 152)	184 (157 to 212)	43.4 (25.9 to 62.3)	4.66 (3.99 to 5.35)	10.7 (9 to 12.2)	129.8 (97.4 to 162.5)
		Escherichia coli	406 (356 to 464)	440 (390 to 501)	8.6 (-5.1 to 22.3)	15.2 (13.6 to 16.9)	26.5 (23 to 29.5)	74.1 (50.4 to 98.4)
		Fungus	176 (152 to 205)	199 (174 to 230)	13.3 (-0.3 to 28.7)	6.52 (5.67 to 7.38)	11.8 (10.3 to 13.5)	81.5 (57.2 to 106.6)
		Group B Streptococcus	260 (227 to 301)	327 (290 to 371)	26.4 (10.5 to 42.7)	6.92 (6.14 to 7.75)	14 (12.1 to 15.6)	102.6 (75.4 to 131.7)
		Haemophilus influenzae	943 (843 to 1060)	778 (691 to 882)	-17.3 (-26.1 to -8.4)	13.5 (12.3 to 14.7)	12.3 (10.7 to 13.6)	-8.9 (-20.4 to 2.6)
		Influenza	1890 (1700 to 2100)	36 (14 to 77.5)	-98.1 (-99.3 to -95.9)	52.9 (48.2 to 57.2)	1.57 (0.56 to 3.38)	-97.0 (-98.9 to -93.4)
		Klebsiella pneumoniae	746 (672 to 837)	824 (733 to 929)	10.6 (-1.0 to 22.6)	27.8 (25.4 to 30)	49.3 (43.2 to 54.6)	77.2 (57.1 to 99.0)
		Legionella spp.	621 (543 to 712)	762 (680 to 864)	23.0 (7.1 to 39.8)	16 (14.2 to 17.8)	31.4 (27.5 to 34.8)	97.2 (71.1 to 126.3)
		Mycoplasma spp.	874 (777 to 992)	760 (683 to 866)	-12.9 (-22.2 to -2.6)	8.67 (7.78 to 9.53)	12.1 (10.5 to 13.4)	39.6 (22.6 to 57.6)
		Polymicrobial	42.5 (25.8 to 68.2)	43.1 (24.2 to 73.4)	1.1 (-12.6 to 16.0)	1.61 (0.98 to 2.6)	2.63 (1.5 to 4.45)	62.0 (38.4 to 86.4)
		Pseudomonas aeruginosa	953 (852 to 1080)	1500 (1340 to 1690)	58.2 (40.8 to 76.9)	24.7 (22.3 to 27)	62.6 (54.5 to 69.1)	153.5 (121.9 to 184.9)
		Respiratory syncytial virus	293 (258 to 336)	7.25 (2.8 to 15.6)	-97.5 (-99.0 to -94.6)	3.75 (3.35 to 4.15)	0.14 (0.05 to 0.32)	-96.1 (-98.6 to -91.4)
		Staphylococcus aureus	1460 (1310 to 1630)	2210 (1970 to 2490)	52.0 (36.5 to 67.9)	77.2 (70.4 to 83.4)	188 (165 to 206)	143.6 (115.7 to 170.8)
		Streptococcus pneumoniae	5010 (4510 to 5590)	3760 (3360 to 4270)	-24.8 (-32.5 to -17.0)	111 (101 to 119)	133 (117 to 147)	20.6 (6.8 to 33.7)
		Other viruses	2510 (2270 to 2810)	3120 (2800 to 3520)	24.4 (11.7 to 37.5)	21.8 (19.9 to 23.5)	43.4 (38.2 to 47.7)	99.4 (76.4 to 121.4)
		Other bacterial pathogens	1550 (1330 to 1790)	1250 (1070 to 1460)	-18.7 (-27.1 to -8.9)	32.9 (28.9 to 37.5)	43 (35.6 to 50.2)	30.5 (14.7 to 47.1)
Czechia	All Ages	*Acinetobacter baumannii*	1410 (1220 to 1620)	1030 (880 to 1200)	-26.8 (-34.4 to -18.8)	40 (34.8 to 45.5)	56.3 (47.1 to 65.7)	40.8 (20.2 to 61.4)
		Chlamydia spp.	5760 (5200 to 6340)	3170 (2870 to 3510)	-44.9 (-50.7 to -38.8)	38.5 (35.1 to 42)	41.2 (35.5 to 46.3)	7.1 (-8.6 to 22.9)
		Enterobacter spp.	883 (773 to 1020)	1130 (987 to 1290)	28.4 (12.6 to 44.1)	22.9 (20.2 to 26)	56.8 (47.6 to 65.1)	148.7 (111.6 to 188.2)
		Escherichia coli	2550 (2290 to 2830)	2480 (2260 to 2750)	-2.5 (-12.9 to 8.9)	72.2 (65.5 to 79.2)	135 (117 to 151)	87.7 (59.9 to 116.3)
		Fungus	1730 (1520 to 1950)	1600 (1430 to 1800)	-7.0 (-17.7 to 3.7)	38.8 (34.6 to 43.7)	72.1 (61.6 to 82.5)	86.1 (58.0 to 115.5)
		Group B Streptococcus	2050 (1830 to 2290)	2190 (1990 to 2420)	7.1 (-4.7 to 19.9)	37.5 (34 to 41.4)	76.7 (66.1 to 86.3)	105.1 (74.3 to 136.9)
		Haemophilus influenzae	8550 (7890 to 9260)	5410 (4970 to 5950)	-36.6 (-42.3 to -30.6)	75.5 (70.6 to 80.8)	65.1 (56.6 to 73.1)	-13.6 (-25.7 to -2.3)
		Influenza	12300 (11300 to 13300)	206 (80.9 to 442)	-98.3 (-99.3 to -96.4)	243 (225 to 260)	7.88 (2.91 to 17.1)	-96.8 (-98.8 to -92.9)

		Klebsiella pneumoniae	5530 (5120 to 6010)	5010 (4590 to 5510)	-9.3 (-17.4 to -0.6)	141 (132 to 152)	258 (224 to 286)	83.0 (57.8 to 107.5)
		Legionella spp.	4740 (4260 to 5270)	5230 (4780 to 5780)	10.6 (-3.2 to 22.9)	80 (72.6 to 88.7)	174 (151 to 195)	118.5 (85.6 to 152.6)
		Mycoplasma spp.	9780 (8980 to 10700)	6620 (6120 to 7230)	-32.3 (-38.1 to -26.3)	53.2 (49.3 to 57.4)	70.6 (61.4 to 78.6)	32.9 (13.4 to 50.8)
		Polymicrobial	315 (203 to 486)	262 (156 to 429)	-17.3 (-29.0 to -5.7)	10.2 (6.99 to 14.9)	14.4 (8.59 to 23.8)	41.0 (8.9 to 70.7)
		Pseudomonas aeruginosa	6300 (5770 to 6930)	9160 (8400 to 10000)	45.7 (30.8 to 60.7)	115 (106 to 125)	326 (282 to 364)	184.5 (142.1 to 224.6)
		Respiratory syncytial virus	5890 (5310 to 6560)	94 (36.6 to 202)	-98.4 (-99.4 to -96.6)	38.3 (35.8 to 40.7)	0.9 (0.34 to 1.96)	-97.7 (-99.1 to -94.9)
		Staphylococcus aureus	8930 (8250 to 9690)	12500 (11500 to 13700)	40.2 (27.3 to 52.8)	345 (322 to 371)	954 (825 to 1060)	176.4 (137.9 to 211.4)
		Streptococcus pneumoniae	48300 (44800 to 52000)	27200 (25300 to 29600)	-43.6 (-47.6 to -39.3)	622 (586 to 661)	718 (622 to 797)	15.4 (-0.5 to 29.8)
		Other viruses	27200 (25200 to 29300)	24200 (22600 to 26400)	-10.9 (-17.0 to -4.3)	129 (121 to 136)	237 (207 to 263)	84.7 (59.5 to 108.0)
		Other bacterial pathogens	9120 (8020 to 10300)	6730 (5820 to 7810)	-26.2 (-34.0 to -18.3)	149 (131 to 167)	214 (178 to 252)	44.1 (22.2 to 63.7)
	Under 5	*Acinetobacter baumannii*	37.1 (24.5 to 54.5)	10.5 (7.24 to 15.2)	-71.6 (-75.3 to -67.5)	1.81 (0.99 to 3.01)	0.37 (0.19 to 0.65)	-79.7 (-84.6 to -74.1)
		Chlamydia spp.	466 (396 to 550)	137 (113 to 165)	-70.7 (-74.1 to -66.5)	2.51 (2.21 to 2.82)	0.44 (0.34 to 0.54)	-82.5 (-86.4 to -78.4)
		Enterobacter spp.	61.7 (46.8 to 79.7)	36.3 (26.7 to 47.8)	-41.1 (-49.6 to -31.5)	1.42 (1 to 2.07)	0.43 (0.29 to 0.63)	-69.6 (-76.6 to -61.4)
		Escherichia coli	189 (159 to 226)	88.7 (71.5 to 108)	-53.0 (-59.3 to -46.0)	4.06 (3.51 to 4.61)	1.01 (0.8 to 1.27)	-75.0 (-80.5 to -69.2)
		Fungus	35.1 (24.3 to 48.8)	17 (11.8 to 23.8)	-51.5 (-58.1 to -43.5)	0.41 (0.29 to 0.54)	0.1 (0.07 to 0.13)	-76.4 (-82.3 to -70.1)
		Group B Streptococcus	176 (147 to 211)	90.9 (74.1 to 113)	-48.3 (-54.9 to -40.5)	3.43 (2.98 to 3.88)	0.9 (0.71 to 1.12)	-73.7 (-79.5 to -67.5)
		Haemophilus influenzae	1550 (1300 to 1840)	437 (357 to 531)	-71.8 (-75.6 to -67.4)	8.95 (7.97 to 10.1)	0.69 (0.54 to 0.86)	-92.2 (-94.1 to -90.3)
		Influenza	1260 (1070 to 1460)	10.1 (3.9 to 22)	-99.2 (-99.7 to -98.3)	13.3 (12 to 14.7)	0.05 (0.02 to 0.1)	-99.6 (-99.9 to -99.2)
		Klebsiella pneumoniae	677 (577 to 790)	234 (194 to 286)	-65.4 (-69.1 to -61.0)	10.3 (9.21 to 11.4)	1.91 (1.52 to 2.33)	-81.3 (-85.4 to -76.9)
		Legionella spp.	84.8 (61.7 to 115)	42.7 (30.3 to 59.3)	-49.6 (-56.2 to -42.2)	1.78 (1.36 to 2.28)	0.53 (0.38 to 0.69)	-70.0 (-77.1 to -62.2)
		Mycoplasma spp.	859 (725 to 1020)	395 (323 to 481)	-54.0 (-59.9 to -47.9)	3.32 (2.94 to 3.74)	0.74 (0.58 to 0.91)	-77.7 (-82.6 to -72.0)
		Polymicrobial	37.1 (21.8 to 59.9)	13.6 (7.55 to 22)	-63.4 (-68.4 to -57.2)	2.36 (1.24 to 3.95)	0.71 (0.34 to 1.25)	-69.9 (-78.1 to -60.5)
		Pseudomonas aeruginosa	477 (403 to 572)	348 (289 to 422)	-26.9 (-36.4 to -16.6)	6.12 (5.45 to 6.79)	2.1 (1.67 to 2.55)	-65.7 (-73.6 to -57.9)
		Respiratory syncytial virus	3600 (3110 to 4170)	43 (16.2 to 93.9)	-98.8 (-99.5 to -97.4)	20.1 (18.2 to 22)	0.11 (0.04 to 0.22)	-99.5 (-99.8 to -98.9)

		Staphylococcus aureus	542 (465 to 633)	354 (292 to 432)	-34.6 (-41.1 to -27.7)	11.3 (10.2 to 12.4)	3.6 (2.84 to 4.39)	-68.1 (-75.3 to -60.8)
		Streptococcus pneumoniae	8030 (6870 to 9330)	2300 (1920 to 2780)	-71.4 (-73.9 to -68.4)	58.5 (53.2 to 64.1)	8.68 (6.89 to 10.6)	-85.1 (-88.5 to -81.7)
		Other viruses	7210 (6180 to 8410)	3080 (2560 to 3740)	-57.3 (-61.0 to -52.5)	21.7 (19.7 to 23.8)	4.76 (3.78 to 5.76)	-78.0 (-82.8 to -72.9)
		Other bacterial pathogens	202 (157 to 255)	69.2 (52.3 to 89)	-65.8 (-70.3 to -60.8)	2.1 (1.61 to 2.74)	0.4 (0.28 to 0.57)	-80.8 (-85.3 to -76.0)
	5-14 years	*Acinetobacter baumannii*	90.4 (67.5 to 119)	36.3 (27 to 47.8)	-59.8 (-64.0 to -54.6)	0.29 (0.24 to 0.34)	0.08 (0.07 to 0.1)	-71.5 (-75.7 to -66.9)
		Chlamydia spp.	491 (377 to 641)	160 (120 to 206)	-67.4 (-71.1 to -63.2)	0.39 (0.35 to 0.44)	0.09 (0.08 to 0.1)	-76.9 (-80.3 to -73.1)
		Enterobacter spp.	56.1 (42.3 to 74.6)	41.3 (30.2 to 54.9)	-26.3 (-35.7 to -16.6)	0.17 (0.15 to 0.2)	0.09 (0.08 to 0.1)	-47.8 (-56.1 to -39.2)
		Escherichia coli	132 (102 to 172)	72.3 (54.8 to 93)	-45.2 (-51.5 to -38.6)	0.42 (0.37 to 0.46)	0.16 (0.14 to 0.18)	-61.2 (-67.2 to -54.7)
		Fungus	159 (120 to 206)	88.9 (67 to 117)	-44.0 (-50.3 to -36.9)	0.49 (0.44 to 0.56)	0.2 (0.17 to 0.22)	-60.3 (-66.7 to -53.3)
		Group B Streptococcus	174 (133 to 226)	112 (83.4 to 146)	-35.3 (-43.0 to -27.0)	0.39 (0.34 to 0.44)	0.18 (0.15 to 0.2)	-54.1 (-61.2 to -46.3)
		Haemophilus influenzae	737 (573 to 953)	290 (218 to 371)	-60.7 (-64.6 to -56.4)	0.89 (0.81 to 0.96)	0.17 (0.15 to 0.19)	-81.2 (-83.8 to -78.4)
		Influenza	1020 (803 to 1300)	9.92 (3.4 to 22.6)	-99.0 (-99.6 to -97.9)	2.41 (2.23 to 2.61)	0.02 (0.01 to 0.03)	-99.3 (-99.8 to -98.5)
		Klebsiella pneumoniae	387 (301 to 495)	195 (148 to 248)	-49.6 (-54.4 to -44.2)	1.21 (1.11 to 1.32)	0.43 (0.38 to 0.48)	-64.3 (-69.3 to -58.8)
		Legionella spp.	412 (318 to 535)	266 (202 to 338)	-35.2 (-42.5 to -27.2)	0.89 (0.79 to 1)	0.41 (0.36 to 0.46)	-54.1 (-61.1 to -46.1)
		Mycoplasma spp.	1600 (1240 to 2060)	739 (567 to 958)	-53.8 (-58.6 to -48.8)	1.33 (1.22 to 1.46)	0.44 (0.38 to 0.48)	-67.3 (-72.3 to -62.1)
		Polymicrobial	20 (12.1 to 31.4)	9.65 (5.36 to 16)	-51.9 (-58.4 to -45.0)	0.06 (0.04 to 0.1)	0.02 (0.01 to 0.04)	-65.9 (-71.7 to -59.8)
		Pseudomonas aeruginosa	439 (341 to 567)	372 (281 to 482)	-15.1 (-23.9 to -5.9)	0.95 (0.87 to 1.05)	0.57 (0.51 to 0.64)	-39.8 (-48.4 to -30.5)
		Respiratory syncytial virus	326 (255 to 420)	4.45 (1.54 to 10.1)	-98.6 (-99.5 to -97.1)	0.35 (0.32 to 0.39)	0 (0 to 0.01)	-99.1 (-99.7 to -98.0)
		Staphylococcus aureus	632 (492 to 804)	493 (372 to 630)	-22.1 (-28.8 to -14.7)	2.81 (2.6 to 3.04)	1.55 (1.38 to 1.72)	-44.8 (-52.3 to -36.6)
		Streptococcus pneumoniae	5670 (4420 to 7230)	2040 (1560 to 2600)	-64.0 (-67.1 to -60.9)	10.5 (9.76 to 11.3)	2.67 (2.38 to 2.95)	-74.5 (-77.9 to -70.9)
		Other viruses	3080 (2410 to 3910)	1860 (1420 to 2390)	-39.4 (-44.6 to -33.7)	2.24 (2.08 to 2.42)	0.96 (0.85 to 1.06)	-57.1 (-63.1 to -51.0)
		Other bacterial pathogens	564 (436 to 734)	224 (167 to 292)	-60.3 (-64.5 to -56.0)	1 (0.88 to 1.13)	0.28 (0.24 to 0.33)	-71.8 (-75.9 to -67.2)
	15-49 years	*Acinetobacter baumannii*	130 (108 to 155)	62.8 (52.2 to 75.3)	-51.7 (-56.9 to -46.3)	2.08 (1.76 to 2.41)	1.28 (1.04 to 1.55)	-38.3 (-49.0 to -26.6)
		Chlamydia spp.	708 (617 to 811)	277 (241 to 316)	-60.8 (-64.8 to -56.3)	2.81 (2.51 to 3.14)	1.4 (1.17 to 1.62)	-49.9 (-58.4 to -40.6)

		Enterobacter spp.	80.8 (68 to 95.7)	71.4 (60.7 to 83.9)	-11.4 (-22.1 to -0.3)	1.22 (1.06 to 1.4)	1.38 (1.14 to 1.65)	13.2 (-7.7 to 33.7)
		Escherichia coli	190 (165 to 219)	125 (110 to 142)	-34.1 (-40.9 to -26.5)	2.98 (2.67 to 3.31)	2.51 (2.14 to 2.9)	-15.8 (-30.2 to 0.4)
		Fungus	229 (196 to 265)	154 (134 to 176)	-32.6 (-40.0 to -24.6)	3.54 (3.08 to 3.98)	3.04 (2.55 to 3.56)	-13.9 (-28.9 to 2.1)
		Group B Streptococcus	250 (217 to 291)	194 (169 to 223)	-22.2 (-31.0 to -12.4)	2.78 (2.46 to 3.17)	2.76 (2.3 to 3.22)	-0.5 (-18.0 to 19.3)
		Haemophilus influenzae	1060 (942 to 1200)	501 (442 to 562)	-52.7 (-57.2 to -48.0)	6.35 (5.85 to 6.94)	2.59 (2.19 to 2.97)	-59.1 (-66.0 to -52.3)
		Influenza	1470 (1310 to 1650)	17.1 (6.5 to 36.6)	-98.8 (-99.6 to -97.5)	17.3 (16 to 18.7)	0.25 (0.09 to 0.53)	-98.6 (-99.5 to -96.9)
		Klebsiella pneumoniae	557 (495 to 624)	337 (298 to 378)	-39.4 (-44.4 to -33.8)	8.69 (8.04 to 9.46)	6.71 (5.74 to 7.65)	-22.6 (-35.5 to -9.9)
		Legionella spp.	593 (518 to 674)	461 (408 to 519)	-22.1 (-30.2 to -12.5)	6.37 (5.71 to 7.17)	6.32 (5.36 to 7.25)	-0.5 (-18.3 to 18.4)
		Mycoplasma spp.	2310 (2040 to 2590)	1280 (1140 to 1440)	-44.4 (-49.5 to -38.7)	9.55 (8.74 to 10.4)	6.77 (5.74 to 7.68)	-29.0 (-40.9 to -16.7)
		Polymicrobial	28.8 (17.7 to 45.2)	16.7 (9.38 to 27.5)	-42.1 (-49.8 to -34.2)	0.46 (0.28 to 0.71)	0.34 (0.19 to 0.55)	-26.0 (-40.7 to -11.4)
		Pseudomonas aeruginosa	632 (559 to 715)	644 (568 to 725)	2.1 (-7.4 to 12.0)	6.84 (6.23 to 7.54)	8.9 (7.58 to 10.1)	30.5 (8.3 to 53.2)
		Respiratory syncytial virus	469 (412 to 532)	7.67 (2.87 to 16.5)	-98.4 (-99.4 to -96.5)	2.5 (2.26 to 2.78)	0.05 (0.02 to 0.11)	-98.0 (-99.3 to -95.6)
		Staphylococcus aureus	911 (810 to 1020)	853 (758 to 951)	-6.3 (-13.4 to 1.7)	20.2 (18.8 to 21.8)	24.1 (20.6 to 27.4)	19.8 (0.4 to 38.8)
		Streptococcus pneumoniae	8160 (7290 to 9140)	3530 (3130 to 3930)	-56.8 (-60.0 to -53.0)	75.2 (70.3 to 81)	41.5 (35.5 to 47.4)	-44.7 (-53.6 to -35.5)
		Other viruses	4430 (3970 to 4960)	3230 (2860 to 3610)	-27.1 (-32.8 to -20.6)	16.1 (14.9 to 17.3)	14.9 (12.8 to 17)	-6.9 (-21.8 to 7.9)
		Other bacterial pathogens	812 (696 to 942)	387 (327 to 456)	-52.2 (-56.6 to -47.2)	7.14 (6.24 to 8.05)	4.35 (3.63 to 5.18)	-38.9 (-49.3 to -28.3)
	50-69 years	*Acinetobacter baumannii*	512 (431 to 597)	310 (259 to 367)	-39.4 (-46.6 to -31.5)	8.44 (7.35 to 9.68)	9.21 (7.64 to 10.9)	9.3 (-8.8 to 27.6)
		Chlamydia spp.	1650 (1450 to 1880)	776 (670 to 886)	-52.8 (-58.8 to -46.2)	6.74 (6.1 to 7.39)	5.72 (4.88 to 6.66)	-15.0 (-28.8 to -0.2)
		Enterobacter spp.	309 (266 to 360)	337 (290 to 392)	9.3 (-6.7 to 24.7)	4.82 (4.25 to 5.49)	9.47 (7.91 to 11.1)	96.9 (63.0 to 130.6)
		Escherichia coli	803 (711 to 906)	641 (559 to 731)	-20.0 (-30.1 to -9.3)	13 (11.9 to 14.2)	18.7 (15.9 to 21.3)	44.1 (20.8 to 68.5)
		Fungus	768 (665 to 885)	640 (552 to 737)	-16.5 (-28.0 to -5.1)	12.2 (10.9 to 13.7)	18.4 (15.7 to 21.3)	50.4 (26.8 to 76.9)
		Group B Streptococcus	676 (589 to 776)	638 (549 to 729)	-5.3 (-18.0 to 8.4)	7.75 (6.96 to 8.67)	13.2 (11.2 to 15.1)	70.6 (41.9 to 100.9)
		Haemophilus influenzae	2450 (2200 to 2730)	1510 (1320 to 1710)	-38.3 (-46.1 to -30.5)	15.1 (14.1 to 16.2)	11.3 (9.67 to 12.9)	-24.8 (-36.5 to -12.1)
		Influenza	2930 (2640 to 3250)	42.4 (16.5 to 91.4)	-98.6 (-99.4 to -96.9)	35.3 (33.3 to 37.5)	0.89 (0.33 to 1.92)	-97.5 (-99.0 to -94.6)

		Klebsiella pneumoniae	1650 (1490 to 1820)	1340 (1180 to 1510)	-18.6 (-28.3 to -8.7)	26.4 (24.8 to 28.1)	38.8 (33.4 to 44)	46.7 (25.1 to 69.3)
		Legionella spp.	1920 (1690 to 2180)	1850 (1630 to 2090)	-3.8 (-17.9 to 9.8)	21.3 (19.4 to 23.4)	36.8 (31.4 to 41.7)	73.4 (45.6 to 103.6)
		Mycoplasma spp.	2440 (2180 to 2730)	1580 (1380 to 1780)	-35.2 (-43.3 to -26.8)	10.4 (9.59 to 11.3)	12.1 (10.4 to 13.8)	16.7 (-2.2 to 35.5)
		Polymicrobial	94.3 (58.4 to 148)	68.2 (40 to 112)	-28.0 (-39.0 to -16.4)	1.54 (0.97 to 2.42)	2.01 (1.14 to 3.33)	29.8 (5.8 to 54.1)
		Pseudomonas aeruginosa	2070 (1840 to 2320)	2560 (2260 to 2880)	23.9 (8.0 to 40.5)	23.1 (21.4 to 24.8)	51.4 (44.3 to 58.3)	123.2 (87.8 to 159.4)
		Respiratory syncytial virus	690 (607 to 785)	13.7 (5.26 to 29.2)	-98.0 (-99.2 to -95.8)	3.8 (3.45 to 4.17)	0.13 (0.05 to 0.28)	-96.5 (-98.7 to -92.4)
		Staphylococcus aureus	2620 (2370 to 2910)	3050 (2690 to 3420)	16.5 (2.2 to 30.2)	59.8 (56.6 to 63.2)	125 (108 to 142)	109.9 (79.0 to 140.3)
		Streptococcus pneumoniae	11600 (10500 to 12800)	6400 (5670 to 7190)	-44.7 (-51.4 to -38.3)	110 (104 to 116)	110 (94.2 to 124)	-0.4 (-15.4 to 14.3)
		Other viruses	5510 (4980 to 6100)	5350 (4740 to 6010)	-2.7 (-14.1 to 8.2)	20.6 (19.4 to 21.8)	36 (30.9 to 40.9)	75.3 (49.6 to 101.5)
		Other bacterial pathogens	2830 (2440 to 3250)	1660 (1390 to 1950)	-41.1 (-48.4 to -33.5)	25.6 (22.9 to 28.6)	27.2 (22.7 to 32.2)	6.3 (-10.6 to 23.4)
	70+ years	*Acinetobacter baumannii*	639 (538 to 754)	610 (516 to 724)	-4.2 (-16.0 to 8.3)	27.4 (23.4 to 31.8)	45.3 (37.4 to 53.2)	65.8 (41.5 to 90.6)
		Chlamydia spp.	2450 (2110 to 2810)	1820 (1610 to 2070)	-25.5 (-35.0 to -15.4)	26.1 (23.1 to 28.8)	33.6 (28.7 to 37.9)	28.9 (9.1 to 48.6)
		Enterobacter spp.	375 (317 to 444)	645 (553 to 746)	72.6 (48.3 to 97.4)	15.3 (13.2 to 17.5)	45.4 (37.5 to 52.3)	198.7 (150.3 to 248.7)
		Escherichia coli	1230 (1070 to 1400)	1550 (1390 to 1760)	26.3 (10.1 to 44.0)	51.7 (46.3 to 57.3)	113 (96.9 to 127)	118.6 (85.2 to 152.5)
		Fungus	534 (455 to 622)	702 (618 to 816)	31.9 (14.4 to 50.0)	22.1 (19.3 to 25.4)	50.4 (42.2 to 58.1)	128.2 (93.5 to 163.8)
		Group B Streptococcus	774 (669 to 888)	1150 (1020 to 1310)	49.6 (30.2 to 71.6)	23.1 (20.5 to 25.9)	59.6 (50.6 to 67.6)	158.8 (118.3 to 200.3)
		Haemophilus influenzae	2750 (2450 to 3110)	2680 (2390 to 3030)	-2.6 (-14.6 to 9.1)	44.2 (40.4 to 48.1)	50.3 (43 to 56.4)	14.1 (-2.3 to 29.6)
		Influenza	5560 (4930 to 6260)	127 (48.4 to 272)	-97.7 (-99.1 to -95.2)	175 (159 to 189)	6.68 (2.45 to 14.3)	-96.2 (-98.6 to -91.7)
		Klebsiella pneumoniae	2260 (2020 to 2550)	2910 (2610 to 3270)	28.6 (13.9 to 44.2)	94.6 (86.4 to 103)	210 (180 to 234)	122.6 (91.3 to 153.1)
		Legionella spp.	1730 (1490 to 1990)	2620 (2340 to 2960)	52.0 (29.8 to 74.5)	49.7 (44.3 to 55.8)	130 (112 to 146)	163.0 (121.2 to 207.1)
		Mycoplasma spp.	2570 (2270 to 2910)	2630 (2350 to 2980)	2.3 (-10.8 to 16.0)	28.6 (25.7 to 31.7)	50.5 (43.6 to 56.5)	77.0 (50.9 to 102.7)
		Polymicrobial	135 (82.4 to 219)	154 (87.1 to 263)	13.8 (-2.6 to 31.0)	5.74 (3.53 to 9.12)	11.3 (6.39 to 18.8)	96.9 (61.6 to 130.5)
		Pseudomonas aeruginosa	2680 (2380 to 3040)	5240 (4700 to 5860)	95.7 (71.7 to 121.3)	77.9 (70.3 to 85.8)	264 (226 to 296)	238.7 (186.8 to 286.9)
		Respiratory syncytial virus	808 (700 to 921)	25.2 (9.87 to 53.6)	-96.9 (-98.7 to -93.4)	11.6 (10.4 to 13)	0.61 (0.23 to 1.32)	-94.7 (-98.0 to -88.5)

		Staphylococcus aureus	4230 (3770 to 4740)	7770 (6960 to 8810)	84.0 (63.4 to 106.3)	251 (230 to 273)	799 (684 to 892)	218.4 (173.3 to 259.6)
		Streptococcus pneumoniae	14900 (13200 to 16700)	13000 (11700 to 14600)	-12.7 (-22.5 to -2.3)	368 (337 to 399)	556 (475 to 620)	51.1 (29.5 to 70.6)
		Other viruses	6990 (6200 to 7880)	10700 (9670 to 12100)	53.7 (36.6 to 72.6)	68 (62.1 to 73.6)	181 (155 to 201)	165.9 (127.4 to 201.8)
		Other bacterial pathogens	4720 (4060 to 5470)	4380 (3740 to 5150)	-6.9 (-18.4 to 4.9)	113 (97.9 to 128)	182 (150 to 214)	61.3 (37.1 to 84.0)
Hungary	All Ages	*Acinetobacter baumannii*	1510 (1320 to 1730)	615 (538 to 706)	-59.2 (-63.1 to -55.0)	22.7 (20 to 25.8)	14.9 (12.6 to 17.1)	-34.3 (-43.1 to -25.1)
		Chlamydia spp.	6030 (5490 to 6600)	2760 (2520 to 3040)	-54.1 (-58.0 to -49.5)	20.6 (19 to 22.4)	15.3 (13.3 to 17.3)	-25.5 (-35.0 to -14.9)
		Enterobacter spp.	823 (721 to 936)	1020 (916 to 1130)	23.9 (11.2 to 38.3)	11.2 (9.79 to 12.7)	22.3 (19.3 to 25.2)	100.3 (73.1 to 129.5)
		Escherichia coli	2430 (2200 to 2680)	2560 (2350 to 2850)	5.4 (-4.3 to 15.5)	35.6 (32.4 to 38.7)	60.2 (53.1 to 66.8)	69.3 (47.6 to 91.1)
		Fungus	1700 (1500 to 1920)	1450 (1310 to 1620)	-14.1 (-22.5 to -4.5)	20.5 (18.3 to 22.9)	29.5 (25.3 to 33.6)	44.1 (25.6 to 64.0)
		Group B Streptococcus	2020 (1830 to 2240)	1960 (1790 to 2170)	-2.5 (-12.7 to 8.5)	19.4 (17.8 to 21.3)	29.6 (26 to 33.1)	52.6 (32.6 to 74.1)
		Haemophilus influenzae	8690 (8050 to 9340)	5430 (5030 to 5930)	-37.4 (-41.5 to -32.7)	38.9 (36.8 to 41.2)	26.9 (23.9 to 29.8)	-30.8 (-38.7 to -22.8)
		Influenza	11900 (11000 to 12700)	213 (81.1 to 458)	-98.2 (-99.3 to -96.1)	116 (109 to 124)	3.48 (1.29 to 7.47)	-97.0 (-98.9 to -93.5)
		Klebsiella pneumoniae	5560 (5190 to 6000)	3750 (3480 to 4120)	-32.5 (-36.7 to -27.3)	71.9 (67.6 to 76)	83.6 (73.7 to 92.7)	16.4 (3.1 to 29.6)
		Legionella spp.	4170 (3750 to 4600)	4100 (3770 to 4500)	-1.4 (-10.6 to 9.8)	37.5 (34.2 to 41.3)	60.8 (53.6 to 67.5)	62.5 (43.0 to 85.8)
		Mycoplasma spp.	10200 (9340 to 11200)	5850 (5400 to 6370)	-42.8 (-46.6 to -38.6)	28.4 (26.6 to 30.4)	24.6 (21.9 to 27.3)	-13.5 (-24.0 to -2.8)
		Polymicrobial	330 (224 to 484)	258 (157 to 418)	-22.3 (-33.7 to -10.1)	5.88 (4.13 to 8.32)	6.17 (3.72 to 9.9)	4.3 (-22.7 to 31.1)
		Pseudomonas aeruginosa	5670 (5230 to 6130)	6600 (6090 to 7210)	16.4 (8.0 to 26.1)	53.4 (49.7 to 57.1)	99.8 (88.1 to 110)	87.1 (63.5 to 109.7)
		Respiratory syncytial virus	6800 (6090 to 7550)	84.3 (32.2 to 181)	-98.8 (-99.5 to -97.3)	22.8 (21.3 to 24.4)	0.27 (0.1 to 0.58)	-98.8 (-99.6 to -97.4)
		Staphylococcus aureus	8220 (7640 to 8790)	11300 (10400 to 12400)	37.3 (29.1 to 47.4)	161 (151 to 170)	371 (327 to 412)	130.9 (105.6 to 157.6)
		Streptococcus pneumoniae	50900 (47300 to 54900)	15100 (14000 to 16400)	-70.3 (-71.8 to -68.4)	325 (309 to 339)	172 (151 to 190)	-47.1 (-52.9 to -41.4)
		Other viruses	27900 (25800 to 30200)	16800 (15700 to 18300)	-39.7 (-42.9 to -36.1)	65.1 (62.2 to 68.1)	67.9 (60 to 75.3)	4.3 (-7.5 to 15.6)
		Other bacterial pathogens	8580 (7590 to 9520)	5450 (4810 to 6220)	-36.4 (-41.6 to -30.2)	71.6 (64 to 79.8)	75.7 (64.3 to 87.2)	5.8 (-7.1 to 19.4)
	Under 5	*Acinetobacter baumannii*	61.6 (41.4 to 90.4)	12.1 (7.88 to 18.1)	-80.3 (-83.1 to -77.2)	1.61 (0.92 to 2.68)	0.18 (0.09 to 0.32)	-89.1 (-92.3 to -84.7)
		Chlamydia spp.	720 (607 to 846)	182 (149 to 220)	-74.6 (-78.3 to -70.4)	2.12 (1.87 to 2.39)	0.23 (0.17 to 0.31)	-89.1 (-92.1 to -85.3)

		Enterobacter spp.	87.4 (66.1 to 114)	53.2 (40.4 to 69.7)	-38.9 (-49.0 to -27.0)	1.11 (0.76 to 1.6)	0.23 (0.15 to 0.33)	-79.0 (-85.1 to -70.9)
		Escherichia coli	277 (233 to 331)	170 (139 to 205)	-38.7 (-48.4 to -28.1)	3.25 (2.83 to 3.73)	0.66 (0.49 to 0.89)	-79.7 (-85.2 to -72.7)
		Fungus	50.7 (34.9 to 71.1)	24.8 (17.3 to 33.8)	-51.0 (-59.5 to -41.4)	0.3 (0.22 to 0.41)	0.06 (0.04 to 0.09)	-79.3 (-85.5 to -71.1)
		Group B Streptococcus	257 (214 to 307)	131 (107 to 160)	-48.9 (-56.7 to -40.5)	2.79 (2.44 to 3.22)	0.48 (0.35 to 0.63)	-82.9 (-87.5 to -77.0)
		Haemophilus influenzae	2160 (1820 to 2570)	722 (593 to 877)	-66.6 (-71.3 to -61.1)	6.73 (5.91 to 7.56)	0.49 (0.36 to 0.66)	-92.7 (-94.7 to -90.0)
		Influenza	1770 (1500 to 2070)	13.5 (5.13 to 29)	-99.2 (-99.7 to -98.4)	10 (8.9 to 11.1)	0.03 (0.01 to 0.06)	-99.7 (-99.9 to -99.4)
		Klebsiella pneumoniae	987 (839 to 1150)	269 (223 to 325)	-72.7 (-76.0 to -68.6)	8 (7.17 to 8.94)	0.95 (0.71 to 1.26)	-88.1 (-91.2 to -84.1)
		Legionella spp.	116 (85.1 to 157)	56.8 (39.9 to 78.9)	-51.2 (-58.5 to -42.5)	1.38 (1.03 to 1.78)	0.25 (0.17 to 0.36)	-81.5 (-86.7 to -74.7)
		Mycoplasma spp.	1210 (1010 to 1420)	707 (585 to 858)	-41.6 (-49.3 to -33.4)	2.53 (2.22 to 2.87)	0.54 (0.4 to 0.73)	-78.5 (-84.5 to -70.8)
		Polymicrobial	56.8 (32.9 to 90.3)	18 (10.5 to 29)	-68.2 (-73.4 to -62.6)	1.92 (1 to 3.16)	0.32 (0.15 to 0.57)	-83.6 (-88.4 to -77.4)
		Pseudomonas aeruginosa	644 (549 to 756)	515 (425 to 624)	-19.8 (-30.2 to -8.1)	4.54 (4 to 5.09)	1.21 (0.9 to 1.62)	-73.2 (-80.6 to -63.9)
		Respiratory syncytial virus	4730 (4060 to 5460)	50.9 (19.4 to 111)	-98.9 (-99.6 to -97.7)	14.4 (13 to 15.9)	0.05 (0.02 to 0.11)	-99.6 (-99.9 to -99.2)
		Staphylococcus aureus	751 (638 to 865)	517 (428 to 619)	-31.1 (-38.4 to -22.6)	8.35 (7.5 to 9.3)	2.18 (1.61 to 2.89)	-73.8 (-80.7 to -65.1)
		Streptococcus pneumoniae	11400 (9690 to 13200)	1300 (1080 to 1570)	-88.5 (-89.7 to -87.2)	43 (38.6 to 47.5)	2 (1.47 to 2.7)	-95.3 (-96.6 to -93.7)
		Other viruses	9490 (8080 to 11000)	2830 (2350 to 3390)	-70.2 (-73.2 to -66.9)	15 (13.5 to 16.5)	1.96 (1.46 to 2.6)	-86.9 (-90.4 to -82.4)
		Other bacterial pathogens	298 (232 to 373)	103 (79.4 to 132)	-65.4 (-70.8 to -58.9)	1.67 (1.26 to 2.19)	0.23 (0.16 to 0.34)	-86.1 (-89.8 to -81.3)
	5-14 years	*Acinetobacter baumannii*	140 (105 to 185)	36 (26.7 to 47.8)	-74.3 (-77.6 to -70.7)	0.15 (0.13 to 0.18)	0.02 (0.02 to 0.02)	-87.3 (-89.7 to -84.4)
		Chlamydia spp.	707 (535 to 932)	225 (167 to 298)	-68.2 (-71.9 to -64.4)	0.19 (0.17 to 0.22)	0.03 (0.03 to 0.04)	-84.3 (-87.2 to -80.9)
		Enterobacter spp.	73.6 (54.4 to 98.1)	61.1 (45.4 to 80.7)	-16.9 (-27.2 to -6.2)	0.08 (0.07 to 0.09)	0.03 (0.03 to 0.04)	-58.9 (-66.9 to -50.3)
		Escherichia coli	181 (138 to 237)	126 (93.1 to 163)	-30.4 (-38.5 to -21.3)	0.19 (0.17 to 0.22)	0.07 (0.06 to 0.08)	-65.6 (-71.9 to -58.2)
		Fungus	217 (165 to 285)	129 (97.1 to 171)	-40.6 (-47.5 to -33.1)	0.23 (0.2 to 0.26)	0.07 (0.06 to 0.08)	-70.6 (-76.4 to -64.2)
		Group B Streptococcus	233 (175 to 303)	161 (120 to 213)	-30.5 (-38.9 to -20.9)	0.18 (0.15 to 0.2)	0.06 (0.05 to 0.07)	-65.6 (-72.0 to -58.3)
		Haemophilus influenzae	966 (735 to 1270)	446 (332 to 581)	-53.8 (-58.6 to -49.0)	0.4 (0.36 to 0.44)	0.06 (0.05 to 0.07)	-84.7 (-87.6 to -81.8)
		Influenza	1360 (1040 to 1750)	16.5 (5.77 to 36.9)	-98.8 (-99.5 to -97.4)	1.09 (0.98 to 1.2)	0.01 (0 to 0.01)	-99.4 (-99.8 to -98.7)

		Klebsiella pneumoniae	530 (404 to 687)	235 (177 to 308)	-55.6 (-59.9 to -50.8)	0.57 (0.5 to 0.63)	0.12 (0.1 to 0.14)	-78.1 (-81.7 to -73.9)
		Legionella spp.	510 (388 to 670)	341 (257 to 444)	-33.1 (-41.1 to -24.0)	0.38 (0.32 to 0.43)	0.12 (0.1 to 0.14)	-66.9 (-73.0 to -60.0)
		Mycoplasma spp.	2120 (1630 to 2710)	943 (707 to 1230)	-55.5 (-60.0 to -50.3)	0.6 (0.54 to 0.67)	0.13 (0.11 to 0.15)	-78.0 (-82.1 to -73.6)
		Polymicrobial	28.7 (17.4 to 45)	15.7 (8.91 to 25.5)	-45.7 (-52.9 to -37.5)	0.03 (0.02 to 0.05)	0.01 (0 to 0.01)	-73.2 (-78.7 to -67.2)
		Pseudomonas aeruginosa	552 (424 to 713)	439 (329 to 572)	-20.3 (-28.3 to -10.8)	0.41 (0.36 to 0.46)	0.16 (0.14 to 0.19)	-60.6 (-67.8 to -52.3)
		Respiratory syncytial virus	400 (309 to 522)	4.62 (1.61 to 10.3)	-98.8 (-99.6 to -97.5)	0.15 (0.13 to 0.16)	0 (0 to 0)	-99.4 (-99.8 to -98.8)
		Staphylococcus aureus	824 (630 to 1060)	734 (552 to 943)	-10.9 (-19.3 to -1.8)	1.25 (1.12 to 1.38)	0.55 (0.47 to 0.64)	-55.9 (-63.7 to -47.6)
		Streptococcus pneumoniae	7580 (5790 to 9720)	1730 (1290 to 2220)	-77.2 (-79.3 to -74.8)	4.79 (4.3 to 5.28)	0.54 (0.46 to 0.62)	-88.7 (-90.7 to -86.6)
		Other viruses	3820 (2920 to 4920)	1910 (1430 to 2460)	-50.0 (-54.6 to -45.0)	0.95 (0.85 to 1.05)	0.23 (0.2 to 0.27)	-75.3 (-79.6 to -70.6)
		Other bacterial pathogens	777 (597 to 1020)	309 (230 to 407)	-60.2 (-64.4 to -55.4)	0.47 (0.41 to 0.54)	0.09 (0.08 to 0.11)	-80.3 (-83.8 to -76.4)
	15-49 years	*Acinetobacter baumannii*	177 (147 to 213)	49.5 (41.5 to 59)	-72.0 (-75.2 to -68.6)	1.86 (1.57 to 2.17)	0.55 (0.45 to 0.66)	-70.2 (-75.6 to -64.9)
		Chlamydia spp.	893 (773 to 1020)	309 (267 to 356)	-65.4 (-68.9 to -61.2)	2.33 (2.08 to 2.62)	0.86 (0.72 to 0.99)	-63.1 (-69.6 to -56.4)
		Enterobacter spp.	93 (77.5 to 110)	83.9 (71.1 to 98.4)	-9.5 (-20.5 to 1.7)	0.93 (0.79 to 1.07)	0.89 (0.75 to 1.05)	-3.6 (-20.5 to 14.6)
		Escherichia coli	228 (196 to 262)	173 (150 to 197)	-24.3 (-32.4 to -14.8)	2.35 (2.08 to 2.62)	1.89 (1.62 to 2.16)	-19.3 (-33.5 to -5.2)
		Fungus	275 (233 to 319)	177 (153 to 206)	-35.4 (-43.1 to -27.3)	2.79 (2.42 to 3.2)	1.92 (1.61 to 2.23)	-31.1 (-43.8 to -18.3)
		Group B Streptococcus	294 (254 to 341)	222 (192 to 255)	-24.4 (-33.5 to -14.7)	2.15 (1.87 to 2.46)	1.73 (1.46 to 2)	-19.4 (-34.0 to -3.5)
		Haemophilus influenzae	1220 (1080 to 1370)	614 (538 to 700)	-49.7 (-54.1 to -45.2)	4.8 (4.37 to 5.25)	1.71 (1.46 to 1.94)	-64.2 (-70.3 to -58.3)
		Influenza	1710 (1520 to 1910)	22.6 (8.51 to 48.9)	-98.7 (-99.5 to -97.2)	13.2 (12 to 14.3)	0.18 (0.07 to 0.38)	-98.6 (-99.5 to -97.0)
		Klebsiella pneumoniae	669 (589 to 752)	323 (283 to 367)	-51.7 (-55.7 to -47.2)	6.86 (6.28 to 7.48)	3.52 (3.02 to 3.99)	-48.6 (-57.3 to -40.5)
		Legionella spp.	645 (563 to 742)	468 (411 to 532)	-27.2 (-35.4 to -18.4)	4.55 (4.04 to 5.13)	3.52 (2.98 to 4.03)	-22.4 (-36.5 to -7.1)
		Mycoplasma spp.	2680 (2370 to 3020)	1300 (1140 to 1480)	-51.6 (-55.9 to -46.6)	7.29 (6.64 to 7.97)	3.75 (3.2 to 4.24)	-48.4 (-57.5 to -39.2)
		Polymicrobial	36.4 (22.6 to 56.2)	21.6 (12.5 to 35)	-41.0 (-48.9 to -32.5)	0.38 (0.24 to 0.59)	0.24 (0.14 to 0.39)	-37.1 (-48.8 to -24.2)
		Pseudomonas aeruginosa	697 (612 to 787)	604 (527 to 687)	-13.3 (-21.4 to -3.5)	4.96 (4.5 to 5.46)	4.57 (3.93 to 5.2)	-7.6 (-24.6 to 7.7)
		Respiratory syncytial virus	505 (440 to 568)	6.32 (2.38 to 13.6)	-98.7 (-99.5 to -97.3)	1.77 (1.58 to 1.97)	0.02 (0.01 to 0.05)	-98.7 (-99.5 to -97.2)

		Staphylococcus aureus	1040 (924 to 1160)	1010 (887 to 1140)	-3.1 (-10.3 to 5.2)	15.2 (14 to 16.4)	15.6 (13.4 to 17.7)	3.3 (-14.1 to 20.4)
		Streptococcus pneumoniae	9580 (8540 to 10700)	2370 (2100 to 2690)	-75.2 (-77.0 to -73.0)	58 (53.5 to 62.6)	15.3 (13.1 to 17.3)	-73.6 (-78.0 to -69.4)
		Other viruses	4820 (4300 to 5380)	2620 (2310 to 2980)	-45.6 (-49.7 to -40.9)	11.5 (10.6 to 12.4)	6.65 (5.7 to 7.51)	-42.0 (-51.9 to -32.8)
		Other bacterial pathogens	982 (833 to 1140)	425 (359 to 496)	-56.7 (-60.8 to -52.0)	5.67 (4.98 to 6.43)	2.62 (2.16 to 3.05)	-53.8 (-61.8 to -45.6)
	50-69 years	*Acinetobacter baumannii*	546 (466 to 633)	194 (168 to 225)	-64.4 (-68.2 to -60.0)	6.29 (5.45 to 7.21)	3.51 (3 to 4.11)	-44.0 (-52.1 to -35.4)
		Chlamydia spp.	1630 (1450 to 1830)	685 (613 to 775)	-57.8 (-62.2 to -52.6)	4.65 (4.2 to 5.13)	3.08 (2.69 to 3.52)	-33.6 (-43.5 to -23.1)
		Enterobacter spp.	278 (240 to 320)	312 (278 to 355)	12.5 (-0.3 to 27.6)	3.04 (2.64 to 3.47)	5.36 (4.67 to 6.1)	77.0 (49.3 to 105.4)
		Escherichia coli	753 (668 to 841)	686 (623 to 766)	-8.7 (-18.9 to 1.9)	8.51 (7.69 to 9.38)	12.2 (10.8 to 13.6)	43.6 (25.0 to 64.4)
		Fungus	721 (631 to 817)	582 (517 to 663)	-19.2 (-28.2 to -9.0)	8.04 (7.1 to 9.05)	10.2 (8.8 to 11.7)	27.2 (9.3 to 46.8)
		Group B Streptococcus	621 (546 to 709)	573 (513 to 652)	-7.6 (-18.4 to 3.9)	4.98 (4.49 to 5.59)	7.23 (6.27 to 8.24)	45.4 (22.8 to 69.1)
		Haemophilus influenzae	2200 (2000 to 2410)	1460 (1330 to 1620)	-33.7 (-39.3 to -26.8)	9.5 (8.85 to 10.2)	6.6 (5.84 to 7.37)	-30.4 (-39.3 to -21.3)
		Influenza	2660 (2410 to 2920)	45.3 (17.4 to 97.8)	-98.3 (-99.3 to -96.3)	22.4 (21 to 24.1)	0.58 (0.21 to 1.25)	-97.4 (-99.0 to -94.4)
		Klebsiella pneumoniae	1550 (1400 to 1690)	1030 (939 to 1150)	-33.3 (-38.9 to -26.5)	17.4 (16.2 to 18.6)	18.2 (16.2 to 20.3)	4.9 (-7.8 to 18.6)
		Legionella spp.	1630 (1440 to 1840)	1470 (1350 to 1650)	-9.8 (-19.5 to 1.3)	12.6 (11.5 to 14)	17.9 (15.8 to 20)	41.9 (22.2 to 63.3)
		Mycoplasma spp.	2210 (1990 to 2440)	1200 (1090 to 1340)	-45.6 (-50.5 to -39.9)	6.61 (6.07 to 7.18)	5.65 (4.98 to 6.35)	-14.4 (-26.0 to -1.7)
		Polymicrobial	93 (59.9 to 143)	69.6 (41.5 to 115)	-25.6 (-35.4 to -14.8)	1.07 (0.68 to 1.68)	1.25 (0.75 to 2.02)	17.0 (-1.0 to 36.6)
		Pseudomonas aeruginosa	1780 (1610 to 1970)	1840 (1680 to 2060)	3.3 (-6.0 to 14.4)	13.9 (12.8 to 15.1)	22.6 (20 to 25.1)	62.5 (40.4 to 84.4)
		Respiratory syncytial virus	581 (516 to 647)	8.74 (3.3 to 18.9)	-98.5 (-99.4 to -96.7)	2.23 (2.02 to 2.48)	0.05 (0.02 to 0.11)	-97.7 (-99.2 to -95.1)
		Staphylococcus aureus	2340 (2130 to 2570)	2850 (2610 to 3180)	21.9 (12.6 to 33.4)	37.4 (35.1 to 39.9)	71.7 (64 to 79.6)	91.9 (68.5 to 116.8)
		Streptococcus pneumoniae	10600 (9670 to 11600)	3570 (3270 to 3980)	-66.4 (-69.0 to -63.3)	70.7 (66.6 to 75)	37.3 (33.2 to 41.4)	-47.1 (-53.3 to -40.7)
		Other viruses	4690 (4250 to 5090)	3520 (3210 to 3920)	-24.9 (-30.9 to -17.7)	12.2 (11.5 to 13)	14.5 (12.9 to 16.1)	18.1 (4.1 to 32.5)
		Other bacterial pathogens	2670 (2350 to 3010)	1430 (1240 to 1650)	-46.6 (-51.6 to -40.5)	17 (15.1 to 18.9)	14.3 (12.1 to 16.5)	-15.8 (-26.7 to -4.4)
	70+ years	*Acinetobacter baumannii*	586 (493 to 688)	323 (274 to 377)	-44.7 (-50.8 to -38.1)	12.8 (11 to 14.8)	10.7 (8.81 to 12.5)	-16.7 (-29.0 to -3.9)
		Chlamydia spp.	2080 (1810 to 2350)	1360 (1200 to 1580)	-34.4 (-41.4 to -26.2)	11.3 (10.1 to 12.7)	11.1 (9.48 to 12.7)	-1.3 (-15.6 to 15.4)

		Enterobacter spp.	291 (244 to 342)	507 (443 to 589)	74.8 (53.3 to 97.4)	6.03 (5.13 to 7.05)	15.8 (13.3 to 18.1)	163.2 (123.8 to 207.8)
		Escherichia coli	994 (859 to 1140)	1410 (1250 to 1600)	41.8 (26.5 to 58.2)	21.3 (18.9 to 23.7)	45.4 (39.4 to 51.2)	113.5 (83.8 to 146.1)
		Fungus	432 (363 to 503)	541 (470 to 634)	25.6 (10.9 to 42.1)	9.14 (7.86 to 10.5)	17.2 (14.4 to 20)	89.1 (61.5 to 121.4)
		Group B Streptococcus	613 (533 to 702)	878 (774 to 1010)	43.6 (25.2 to 61.5)	9.33 (8.21 to 10.5)	20.1 (17.2 to 22.7)	116.2 (83.4 to 152.8)
		Haemophilus influenzae	2130 (1890 to 2370)	2190 (1950 to 2480)	2.9 (-6.6 to 13.6)	17.5 (15.8 to 19.1)	18 (15.6 to 20.3)	3.4 (-10.1 to 18.1)
		Influenza	4350 (3870 to 4790)	115 (44.4 to 247)	-97.4 (-99.0 to -94.2)	69.6 (63.2 to 76)	2.68 (0.98 to 5.84)	-96.1 (-98.6 to -91.6)
		Klebsiella pneumoniae	1830 (1630 to 2030)	1900 (1690 to 2150)	3.6 (-5.9 to 13.6)	39.1 (35.5 to 42.5)	60.8 (52.5 to 68.3)	55.9 (35.4 to 76.7)
		Legionella spp.	1260 (1100 to 1440)	1770 (1570 to 2010)	40.1 (24.6 to 57.9)	18.6 (16.4 to 20.9)	39 (33.7 to 44.1)	111.0 (80.2 to 145.9)
		Mycoplasma spp.	2010 (1760 to 2260)	1700 (1520 to 1930)	-15.4 (-23.6 to -6.2)	11.4 (10.2 to 12.6)	14.5 (12.6 to 16.3)	27.3 (9.9 to 47.0)
		Polymicrobial	115 (70.7 to 183)	133 (77.4 to 225)	15.6 (0.2 to 32.5)	2.49 (1.55 to 3.9)	4.35 (2.53 to 7.22)	74.0 (46.1 to 104.7)
		Pseudomonas aeruginosa	2000 (1750 to 2230)	3200 (2850 to 3600)	60.5 (45.5 to 76.9)	29.5 (26.7 to 32.4)	71.3 (61.6 to 79.9)	141.6 (108.7 to 177.2)
		Respiratory syncytial virus	586 (504 to 664)	13.7 (5.32 to 29.8)	-97.7 (-99.1 to -94.8)	4.28 (3.82 to 4.78)	0.15 (0.05 to 0.32)	-96.6 (-98.7 to -92.6)
		Staphylococcus aureus	3260 (2900 to 3600)	6170 (5510 to 6930)	89.5 (73.5 to 107.0)	98.7 (89.7 to 107)	281 (244 to 316)	185.2 (149.2 to 226.4)
		Streptococcus pneumoniae	11800 (10400 to 13000)	6140 (5480 to 6950)	-47.8 (-52.3 to -43.0)	148 (135 to 161)	117 (101 to 131)	-21.4 (-31.1 to -10.5)
		Other viruses	5120 (4560 to 5660)	5970 (5320 to 6780)	16.7 (6.7 to 27.5)	25.4 (23.1 to 27.7)	44.6 (38.8 to 50)	75.6 (53.7 to 100.5)
		Other bacterial pathogens	3850 (3290 to 4410)	3190 (2750 to 3720)	-17.0 (-25.1 to -8.5)	46.8 (41 to 53.3)	58.5 (48.8 to 68.6)	25.2 (8.2 to 44.0)
Montenegro	All Ages	*Acinetobacter baumannii*	59.7 (51.8 to 68.6)	50.2 (43.3 to 57.6)	-15.8 (-23.9 to -7.0)	0.82 (0.67 to 1)	1.11 (0.89 to 1.37)	36.6 (6.7 to 72.3)
		Chlamydia spp.	287 (261 to 315)	162 (147 to 176)	-43.6 (-48.4 to -38.4)	0.85 (0.73 to 1)	0.8 (0.66 to 0.96)	-5.3 (-25.6 to 16.7)
		Enterobacter spp.	42.1 (36.6 to 47.7)	45.5 (40.4 to 51)	8.3 (-2.3 to 19.4)	0.51 (0.42 to 0.61)	0.9 (0.73 to 1.1)	78.0 (39.3 to 121.3)
		Escherichia coli	118 (107 to 130)	105 (95.7 to 114)	-11.1 (-19.1 to -2.6)	1.54 (1.32 to 1.81)	2.29 (1.89 to 2.74)	49.1 (18.9 to 83.9)
		Fungus	80 (70.4 to 91.2)	74.6 (66.9 to 82.9)	-6.6 (-15.3 to 2.4)	0.83 (0.69 to 0.99)	1.29 (1.04 to 1.57)	56.3 (23.0 to 95.6)
		Group B Streptococcus	105 (94.2 to 117)	96.4 (87.9 to 105)	-7.8 (-16.8 to 1.4)	0.88 (0.75 to 1.04)	1.28 (1.06 to 1.52)	45.5 (13.1 to 83.4)
		Haemophilus influenzae	516 (472 to 565)	268 (247 to 291)	-48.0 (-52.4 to -44.1)	1.98 (1.7 to 2.32)	1.19 (1 to 1.43)	-39.5 (-52.5 to -24.8)
		Influenza	640 (591 to 691)	8.46 (3.18 to 18.1)	-98.7 (-99.5 to -97.1)	5.38 (4.64 to 6.24)	0.12 (0.05 to 0.26)	-97.7 (-99.2 to -94.9)

		Klebsiella pneumoniae	291 (269 to 316)	223 (206 to 241)	-23.3 (-29.6 to -17.3)	3.26 (2.81 to 3.78)	4.42 (3.67 to 5.29)	36.2 (8.0 to 69.0)
		Legionella spp.	218 (196 to 242)	213 (196 to 231)	-2.3 (-11.0 to 7.6)	1.72 (1.45 to 2.01)	2.78 (2.32 to 3.34)	62.6 (27.3 to 102.5)
		Mycoplasma spp.	583 (529 to 645)	355 (326 to 386)	-39.1 (-43.6 to -34.7)	1.32 (1.14 to 1.53)	1.29 (1.08 to 1.54)	-2.0 (-21.4 to 21.0)
		Polymicrobial	16 (10.8 to 23.3)	12.2 (7.73 to 19.4)	-24.1 (-36.1 to -12.5)	0.27 (0.18 to 0.41)	0.26 (0.16 to 0.42)	-3.5 (-38.2 to 36.9)
		Pseudomonas aeruginosa	309 (284 to 337)	353 (323 to 381)	14.1 (4.6 to 24.2)	2.55 (2.2 to 2.97)	4.89 (4.08 to 5.84)	92.6 (52.3 to 138.3)
		Respiratory syncytial virus	577 (515 to 655)	5.34 (2.04 to 11.5)	-99.1 (-99.7 to -98.0)	1.79 (1.41 to 2.29)	0.01 (0.01 to 0.03)	-99.2 (-99.7 to -98.3)
		Staphylococcus aureus	423 (390 to 456)	483 (447 to 522)	14.5 (6.0 to 24.1)	7.24 (6.26 to 8.42)	14.6 (12.2 to 17.4)	103.1 (62.2 to 150.6)
		Streptococcus pneumoniae	2990 (2750 to 3260)	1450 (1340 to 1570)	-51.6 (-54.9 to -48.7)	16.1 (13.9 to 18.6)	13.2 (11 to 15.7)	-17.3 (-34.8 to 1.1)
		Other viruses	1950 (1780 to 2140)	1170 (1080 to 1280)	-39.8 (-44.1 to -36.1)	3.91 (3.32 to 4.63)	3.79 (3.15 to 4.48)	-2.4 (-24.4 to 20.2)
		Other bacterial pathogens	386 (343 to 432)	292 (254 to 332)	-24.4 (-30.9 to -17.0)	2.89 (2.4 to 3.45)	3.82 (3.07 to 4.67)	32.6 (5.4 to 66.8)
	Under 5	*Acinetobacter baumannii*	4.51 (3.03 to 6.64)	1.14 (0.78 to 1.67)	-74.7 (-77.4 to -71.7)	0.1 (0.05 to 0.18)	0.01 (0 to 0.02)	-91.0 (-95.5 to -85.8)
		Chlamydia spp.	58.1 (49.5 to 68.1)	14.5 (12.1 to 17.4)	-75.0 (-78.1 to -71.8)	0.16 (0.12 to 0.23)	0.01 (0.01 to 0.02)	-92.9 (-96.4 to -88.8)
		Enterobacter spp.	7.81 (6.02 to 10)	3.16 (2.38 to 4.13)	-59.4 (-64.7 to -53.3)	0.09 (0.06 to 0.13)	0.01 (0.01 to 0.01)	-89.4 (-94.6 to -83.3)
		Escherichia coli	23.8 (20.1 to 28.6)	8.33 (6.84 to 9.98)	-64.9 (-69.5 to -60.2)	0.26 (0.19 to 0.36)	0.02 (0.01 to 0.03)	-90.8 (-95.4 to -85.4)
		Fungus	4.44 (3.12 to 6.11)	1.58 (1.11 to 2.17)	-64.3 (-69.4 to -59.1)	0.03 (0.02 to 0.05)	0 (0 to 0)	-92.2 (-96.3 to -87.1)
		Group B Streptococcus	22.1 (18.5 to 26.6)	8.26 (6.75 to 10.1)	-62.6 (-67.2 to -57.5)	0.21 (0.15 to 0.29)	0.02 (0.01 to 0.03)	-90.1 (-95.0 to -84.3)
		Haemophilus influenzae	197 (169 to 231)	42.3 (35 to 51.1)	-78.5 (-81.3 to -75.4)	0.64 (0.47 to 0.87)	0.02 (0.01 to 0.03)	-97.0 (-98.5 to -95.0)
		Influenza	160 (138 to 187)	0.88 (0.34 to 1.95)	-99.4 (-99.8 to -98.8)	0.97 (0.7 to 1.33)	0 (0 to 0)	-99.9 (-100.0 to -99.7)
		Klebsiella pneumoniae	85.5 (73.2 to 100)	22.5 (18.7 to 27)	-73.6 (-77.0 to -70.6)	0.72 (0.53 to 1.01)	0.05 (0.03 to 0.07)	-93.4 (-96.6 to -89.3)
		Legionella spp.	10.8 (7.8 to 14.6)	3.69 (2.62 to 5.09)	-65.6 (-70.0 to -61.4)	0.1 (0.07 to 0.16)	0.01 (0.01 to 0.02)	-89.4 (-94.6 to -83.4)
		Mycoplasma spp.	109 (92.3 to 129)	37 (30.4 to 44.5)	-66.0 (-70.5 to -61.4)	0.24 (0.17 to 0.33)	0.02 (0.01 to 0.03)	-92.2 (-96.1 to -87.3)
		Polymicrobial	4.56 (2.65 to 7.3)	1.3 (0.75 to 2.11)	-71.4 (-74.7 to -67.3)	0.12 (0.06 to 0.22)	0.01 (0.01 to 0.03)	-88.3 (-94.2 to -81.2)
		Pseudomonas aeruginosa	60.9 (52.2 to 72.1)	28.5 (23.8 to 34.5)	-53.1 (-59.2 to -47.2)	0.41 (0.3 to 0.58)	0.04 (0.03 to 0.06)	-88.9 (-94.4 to -82.1)
		Respiratory syncytial virus	463 (402 to 537)	3.34 (1.28 to 7.23)	-99.3 (-99.7 to -98.4)	1.41 (1.03 to 1.91)	0 (0 to 0)	-99.8 (-100.0 to -99.7)

		Staphylococcus aureus	69 (59.6 to 81)	30.2 (24.9 to 36.4)	-56.3 (-61.2 to -51.8)	0.82 (0.6 to 1.12)	0.08 (0.05 to 0.11)	-89.9 (-94.9 to -83.9)
		Streptococcus pneumoniae	1020 (880 to 1180)	228 (189 to 272)	-77.6 (-80.0 to -75.3)	4.48 (3.26 to 6.16)	0.22 (0.14 to 0.32)	-94.9 (-97.4 to -91.6)
		Other viruses	928 (803 to 1080)	266 (220 to 318)	-71.3 (-74.5 to -68.3)	1.65 (1.2 to 2.29)	0.11 (0.07 to 0.16)	-93.3 (-96.6 to -89.1)
		Other bacterial pathogens	25.5 (19.9 to 32.1)	6.9 (5.25 to 8.74)	-72.9 (-76.6 to -69.2)	0.14 (0.1 to 0.21)	0.01 (0.01 to 0.02)	-92.9 (-96.4 to -88.6)
	5-14 years	*Acinetobacter baumannii*	8.59 (6.44 to 11.2)	3.94 (2.9 to 5.21)	-54.1 (-59.3 to -48.4)	0.01 (0.01 to 0.02)	0 (0 to 0)	-78.6 (-85.6 to -69.1)
		Chlamydia spp.	47.4 (35.9 to 61.1)	16.9 (12.8 to 22.3)	-64.2 (-68.3 to -59.8)	0.02 (0.01 to 0.02)	0 (0 to 0)	-83.3 (-88.8 to -76.0)
		Enterobacter spp.	5.5 (4.1 to 7.2)	3.56 (2.66 to 4.76)	-35.2 (-42.7 to -27.1)	0.01 (0.01 to 0.01)	0 (0 to 0)	-69.8 (-79.7 to -56.7)
		Escherichia coli	12.9 (9.77 to 16.5)	6.75 (5.11 to 8.82)	-47.4 (-53.5 to -41.0)	0.02 (0.02 to 0.02)	0 (0 to 0.01)	-75.5 (-83.7 to -65.5)
		Fungus	15.5 (11.7 to 19.8)	8.3 (6.26 to 10.9)	-46.2 (-52.7 to -39.4)	0.02 (0.02 to 0.03)	0.01 (0 to 0.01)	-75.0 (-83.3 to -63.8)
		Group B Streptococcus	16.9 (12.9 to 21.6)	10 (7.46 to 13.2)	-40.7 (-47.7 to -33.1)	0.02 (0.01 to 0.02)	0.01 (0 to 0.01)	-72.4 (-81.5 to -60.4)
		Haemophilus influenzae	72.2 (55.8 to 91.9)	28.1 (21.3 to 36.7)	-61.1 (-65.3 to -56.7)	0.04 (0.03 to 0.05)	0.01 (0 to 0.01)	-87.4 (-91.5 to -82.0)
		Influenza	100 (77.3 to 128)	0.86 (0.3 to 1.92)	-99.1 (-99.7 to -98.1)	0.11 (0.09 to 0.14)	0 (0 to 0)	-99.6 (-99.9 to -99.1)
		Klebsiella pneumoniae	37.6 (29.1 to 48.4)	18.8 (14.2 to 24.6)	-50.1 (-55.4 to -44.6)	0.06 (0.04 to 0.07)	0.01 (0.01 to 0.02)	-76.8 (-84.4 to -66.7)
		Legionella spp.	40.8 (31.4 to 52.1)	22.5 (17.2 to 29)	-44.6 (-51.2 to -37.6)	0.04 (0.03 to 0.05)	0.01 (0.01 to 0.01)	-74.2 (-82.9 to -63.6)
		Mycoplasma spp.	157 (120 to 201)	69 (53 to 90.7)	-55.9 (-60.6 to -51.0)	0.06 (0.05 to 0.08)	0.01 (0.01 to 0.02)	-79.5 (-86.2 to -71.1)
		Polymicrobial	1.92 (1.15 to 3.06)	0.96 (0.55 to 1.58)	-50.2 (-56.1 to -43.4)	0 (0 to 0)	0 (0 to 0)	-76.8 (-84.4 to -66.9)
		Pseudomonas aeruginosa	43.3 (32.9 to 55.1)	30.3 (23.1 to 39.8)	-30.0 (-37.7 to -22.1)	0.05 (0.04 to 0.06)	0.01 (0.01 to 0.02)	-67.4 (-78.2 to -53.8)
		Respiratory syncytial virus	32.3 (24.8 to 41.2)	0.34 (0.12 to 0.77)	-98.9 (-99.6 to -97.7)	0.02 (0.01 to 0.02)	0 (0 to 0)	-99.5 (-99.8 to -98.9)
		Staphylococcus aureus	62 (48.1 to 79.1)	41.9 (32.1 to 54.9)	-32.4 (-39.3 to -25.3)	0.13 (0.1 to 0.16)	0.04 (0.03 to 0.06)	-68.5 (-78.8 to -55.1)
		Streptococcus pneumoniae	553 (429 to 705)	203 (156 to 267)	-63.3 (-67.1 to -59.6)	0.5 (0.39 to 0.61)	0.08 (0.06 to 0.11)	-82.9 (-88.5 to -75.6)
		Other viruses	304 (235 to 387)	161 (124 to 213)	-47.0 (-52.3 to -41.5)	0.11 (0.08 to 0.13)	0.03 (0.02 to 0.04)	-75.3 (-83.3 to -64.8)
		Other bacterial pathogens	54.7 (42 to 71.3)	22.3 (16.7 to 29.3)	-59.3 (-63.7 to -54.4)	0.05 (0.04 to 0.06)	0.01 (0.01 to 0.01)	-81.0 (-87.2 to -72.9)
	15-49 years	*Acinetobacter baumannii*	7.8 (6.25 to 9.46)	4.75 (3.9 to 5.68)	-39.1 (-44.5 to -32.9)	0.06 (0.05 to 0.08)	0.04 (0.03 to 0.05)	-35.4 (-52.1 to -15.9)
		Chlamydia spp.	43 (36.2 to 50)	20.4 (17.7 to 23.4)	-52.5 (-56.6 to -48.2)	0.08 (0.07 to 0.1)	0.04 (0.03 to 0.05)	-49.6 (-62.8 to -33.9)

		Enterobacter spp.	4.99 (4.15 to 5.91)	4.28 (3.62 to 5.05)	-13.9 (-22.0 to -5.5)	0.04 (0.03 to 0.05)	0.03 (0.03 to 0.04)	-8.8 (-32.4 to 21.7)
		Escherichia coli	11.7 (9.86 to 13.5)	8.12 (7.08 to 9.23)	-30.2 (-36.4 to -24.1)	0.09 (0.07 to 0.11)	0.06 (0.05 to 0.08)	-26.0 (-44.6 to -3.2)
		Fungus	14 (11.8 to 16.4)	9.98 (8.58 to 11.5)	-28.6 (-35.0 to -21.2)	0.1 (0.09 to 0.13)	0.08 (0.06 to 0.1)	-24.4 (-44.2 to -1.5)
		Group B Streptococcus	15.4 (13 to 18.3)	12.1 (10.5 to 13.8)	-21.3 (-29.0 to -13.2)	0.08 (0.07 to 0.1)	0.07 (0.05 to 0.09)	-16.6 (-37.6 to 9.6)
		Haemophilus influenzae	65.5 (56.8 to 75.5)	33.8 (29.7 to 38.3)	-48.4 (-52.4 to -44.1)	0.19 (0.16 to 0.22)	0.07 (0.06 to 0.09)	-61.8 (-71.6 to -50.2)
		Influenza	90.9 (78.7 to 104)	1.04 (0.4 to 2.23)	-98.9 (-99.6 to -97.5)	0.51 (0.43 to 0.61)	0.01 (0 to 0.01)	-98.8 (-99.6 to -97.5)
		Klebsiella pneumoniae	34.1 (29.6 to 38.9)	22.6 (19.8 to 25.5)	-33.8 (-38.6 to -28.8)	0.26 (0.22 to 0.31)	0.18 (0.14 to 0.22)	-29.9 (-47.7 to -8.7)
		Legionella spp.	37 (31.6 to 42.7)	27.1 (23.7 to 30.6)	-26.5 (-33.1 to -19.1)	0.19 (0.16 to 0.23)	0.15 (0.12 to 0.18)	-22.1 (-42.2 to 3.2)
		Mycoplasma spp.	142 (123 to 163)	83.1 (72.8 to 93.9)	-41.5 (-46.3 to -36.4)	0.28 (0.24 to 0.34)	0.17 (0.14 to 0.22)	-38.0 (-53.3 to -18.8)
		Polymicrobial	1.75 (1.05 to 2.76)	1.16 (0.68 to 1.89)	-33.9 (-40.6 to -26.9)	0.01 (0.01 to 0.02)	0.01 (0.01 to 0.02)	-30.0 (-47.4 to -8.7)
		Pseudomonas aeruginosa	39.3 (34.1 to 45.2)	36.5 (32.1 to 41.4)	-7.1 (-14.4 to 1.1)	0.2 (0.17 to 0.24)	0.2 (0.16 to 0.25)	-1.5 (-27.2 to 28.0)
		Respiratory syncytial virus	29.3 (25.1 to 33.4)	0.41 (0.16 to 0.88)	-98.6 (-99.4 to -97.0)	0.08 (0.06 to 0.09)	0 (0 to 0)	-98.6 (-99.5 to -96.9)
		Staphylococcus aureus	56.2 (48.7 to 63.8)	50.4 (44.5 to 56.6)	-10.2 (-16.4 to -3.7)	0.6 (0.51 to 0.71)	0.57 (0.45 to 0.71)	-4.8 (-28.7 to 24.0)
		Streptococcus pneumoniae	502 (437 to 567)	244 (215 to 275)	-51.3 (-54.5 to -47.9)	2.24 (1.89 to 2.65)	1.15 (0.92 to 1.42)	-48.4 (-61.1 to -32.7)
		Other viruses	276 (241 to 312)	194 (172 to 218)	-29.6 (-34.4 to -24.3)	0.48 (0.41 to 0.58)	0.36 (0.28 to 0.44)	-25.4 (-44.0 to -2.9)
		Other bacterial pathogens	49.7 (41.2 to 57.8)	26.8 (22.5 to 31.5)	-45.9 (-50.2 to -40.8)	0.21 (0.17 to 0.26)	0.12 (0.09 to 0.15)	-42.7 (-57.4 to -25.3)
	50-69 years	*Acinetobacter baumannii*	19.1 (15.9 to 22.5)	18.7 (15.7 to 21.9)	-1.7 (-12.8 to 9.4)	0.21 (0.16 to 0.27)	0.25 (0.18 to 0.33)	19.7 (-18.1 to 67.9)
		Chlamydia spp.	62.1 (54 to 71)	46.2 (40.6 to 51.9)	-25.5 (-34.4 to -16.5)	0.17 (0.13 to 0.22)	0.15 (0.11 to 0.2)	-9.3 (-38.3 to 29.5)
		Enterobacter spp.	11.8 (9.99 to 13.8)	16.2 (13.8 to 18.5)	37.2 (21.0 to 54.2)	0.12 (0.09 to 0.16)	0.2 (0.15 to 0.27)	67.0 (13.0 to 133.3)
		Escherichia coli	30.5 (26.6 to 35.1)	33.5 (29.7 to 37.5)	10.1 (-2.1 to 22.7)	0.33 (0.26 to 0.42)	0.43 (0.32 to 0.57)	34.0 (-10.2 to 85.6)
		Fungus	29.2 (25.1 to 34)	33.2 (28.8 to 37.7)	14.1 (1.4 to 27.7)	0.31 (0.24 to 0.39)	0.42 (0.31 to 0.56)	39.0 (-5.0 to 95.1)
		Group B Streptococcus	25.8 (22.1 to 29.8)	32 (28.1 to 35.9)	24.3 (9.1 to 39.0)	0.2 (0.15 to 0.25)	0.29 (0.22 to 0.39)	51.4 (2.2 to 117.5)
		Haemophilus influenzae	93.8 (82.6 to 106)	80.1 (70.8 to 88.6)	-14.5 (-23.7 to -5.5)	0.38 (0.31 to 0.49)	0.28 (0.21 to 0.36)	-27.3 (-50.3 to 3.9)
		Influenza	112 (98.3 to 126)	2.05 (0.79 to 4.42)	-98.2 (-99.3 to -96.0)	0.9 (0.71 to 1.13)	0.02 (0.01 to 0.04)	-97.8 (-99.3 to -95.0)

		Klebsiella pneumoniae	62.6 (55.6 to 69.7)	69.9 (62.3 to 77.3)	11.9 (0.3 to 23.9)	0.67 (0.53 to 0.85)	0.9 (0.68 to 1.19)	36.2 (-7.1 to 90.8)
		Legionella spp.	74.4 (65.1 to 85.3)	87 (77.5 to 96.7)	17.2 (4.7 to 31.6)	0.55 (0.43 to 0.69)	0.77 (0.58 to 1.02)	42.8 (-4.1 to 101.0)
		Mycoplasma spp.	93.3 (81.7 to 106)	83.5 (74.1 to 92.7)	-10.3 (-19.8 to -0.1)	0.26 (0.21 to 0.33)	0.29 (0.22 to 0.38)	9.3 (-25.2 to 55.7)
		Polymicrobial	3.55 (2.2 to 5.68)	3.77 (2.25 to 6.19)	6.0 (-6.8 to 19.1)	0.04 (0.02 to 0.06)	0.05 (0.03 to 0.09)	29.0 (-12.7 to 81.6)
		Pseudomonas aeruginosa	79.8 (69.7 to 89.8)	117 (103 to 130)	46.5 (31.5 to 62.1)	0.59 (0.47 to 0.74)	1.04 (0.79 to 1.38)	78.3 (20.1 to 150.7)
		Respiratory syncytial virus	26.7 (23.3 to 30.6)	0.59 (0.22 to 1.29)	-97.8 (-99.1 to -95.2)	0.1 (0.08 to 0.12)	0 (0 to 0.01)	-97.4 (-99.1 to -94.1)
		Staphylococcus aureus	100 (88.7 to 112)	144 (128 to 159)	43.8 (29.0 to 58.5)	1.53 (1.2 to 1.92)	2.64 (2 to 3.49)	75.1 (18.9 to 149.2)
		Streptococcus pneumoniae	442 (392 to 493)	351 (313 to 388)	-20.5 (-28.6 to -12.6)	2.8 (2.22 to 3.54)	2.67 (2.04 to 3.49)	-3.2 (-34.4 to 37.5)
		Other viruses	213 (189 to 238)	252 (224 to 278)	18.5 (6.9 to 30.1)	0.53 (0.42 to 0.67)	0.75 (0.57 to 0.98)	44.3 (-2.0 to 104.4)
		Other bacterial pathogens	107 (91.3 to 123)	92.3 (78.3 to 106)	-13.8 (-23.3 to -3.9)	0.65 (0.51 to 0.82)	0.67 (0.5 to 0.9)	5.1 (-28.8 to 48.3)
	70+ years	*Acinetobacter baumannii*	19.7 (16.5 to 23.7)	21.7 (18 to 25.6)	10.2 (-5.4 to 26.6)	0.44 (0.35 to 0.55)	0.82 (0.64 to 1.01)	87.0 (46.3 to 135.7)
		Chlamydia spp.	76.7 (66.2 to 88.8)	63.9 (55.7 to 72.9)	-16.5 (-28.3 to -4.1)	0.42 (0.35 to 0.51)	0.6 (0.49 to 0.72)	41.7 (9.9 to 78.8)
		Enterobacter spp.	11.9 (9.98 to 14.3)	18.3 (15.5 to 21.2)	53.7 (32.0 to 77.5)	0.25 (0.2 to 0.31)	0.65 (0.52 to 0.81)	161.0 (101.4 to 230.7)
		Escherichia coli	38.9 (33.6 to 44.9)	47.8 (41.8 to 54.4)	23.3 (7.0 to 40.6)	0.85 (0.7 to 1.03)	1.76 (1.44 to 2.13)	109.3 (62.5 to 163.4)
		Fungus	16.9 (14.2 to 19.9)	21.5 (18.5 to 24.9)	27.9 (11.0 to 46.1)	0.36 (0.29 to 0.44)	0.78 (0.63 to 0.96)	117.1 (69.4 to 173.6)
		Group B Streptococcus	24.5 (21 to 28.6)	34 (29.7 to 38.7)	39.2 (19.9 to 59.4)	0.38 (0.31 to 0.47)	0.89 (0.72 to 1.08)	136.5 (83.1 to 201.1)
		Haemophilus influenzae	87.7 (76.1 to 99.8)	83.8 (73.4 to 94.9)	-4.2 (-17.3 to 9.3)	0.73 (0.61 to 0.88)	0.82 (0.68 to 1)	13.5 (-11.0 to 41.6)
		Influenza	177 (154 to 201)	3.62 (1.36 to 7.73)	-97.9 (-99.2 to -95.6)	2.88 (2.41 to 3.46)	0.1 (0.04 to 0.21)	-96.6 (-98.8 to -92.5)
		Klebsiella pneumoniae	71.5 (62.4 to 81.4)	89.4 (78.8 to 101)	25.3 (8.9 to 41.8)	1.55 (1.3 to 1.86)	3.28 (2.69 to 3.98)	112.8 (66.1 to 165.7)
		Legionella spp.	55.5 (47.8 to 64.9)	72.7 (63.7 to 82.2)	31.3 (13.3 to 50.6)	0.83 (0.68 to 1.01)	1.84 (1.51 to 2.22)	123.0 (72.6 to 182.7)
		Mycoplasma spp.	81.8 (71.1 to 93.9)	82 (72 to 93)	0.5 (-12.9 to 14.9)	0.47 (0.39 to 0.57)	0.8 (0.65 to 0.98)	70.7 (34.1 to 115.0)
		Polymicrobial	4.22 (2.6 to 6.76)	5.01 (2.96 to 8.32)	18.7 (1.5 to 35.7)	0.09 (0.06 to 0.15)	0.19 (0.11 to 0.31)	101.5 (56.0 to 152.0)
		Pseudomonas aeruginosa	86.1 (74.5 to 98.7)	141 (123 to 159)	64.1 (42.1 to 87.4)	1.3 (1.08 to 1.56)	3.59 (2.95 to 4.35)	178.7 (116.3 to 247.9)
		Respiratory syncytial virus	26 (22.3 to 30.4)	0.64 (0.25 to 1.38)	-97.5 (-99.1 to -94.6)	0.19 (0.16 to 0.23)	0.01 (0 to 0.02)	-95.9 (-98.6 to -91.0)

		Staphylococcus aureus	135 (118 to 154)	217 (191 to 244)	61.1 (40.9 to 83.4)	4.16 (3.47 to 5)	11.3 (9.29 to 13.7)	173.6 (114.4 to 240.9)
		Streptococcus pneumoniae	472 (413 to 538)	420 (369 to 475)	-10.9 (-22.2 to 1.2)	6.06 (5.06 to 7.19)	9.11 (7.49 to 11)	51.3 (18.7 to 88.9)
		Other viruses	225 (196 to 256)	298 (262 to 336)	32.7 (15.1 to 51.1)	1.13 (0.95 to 1.35)	2.54 (2.09 to 3.07)	125.4 (76.6 to 182.2)
		Other bacterial pathogens	149 (126 to 174)	143 (121 to 168)	-3.5 (-16.3 to 10.6)	1.85 (1.49 to 2.25)	3.01 (2.39 to 3.66)	64.1 (29.7 to 107.5)
North Macedonia	All Ages	*Acinetobacter baumannii*	329 (285 to 377)	170 (147 to 194)	-48.3 (-53.0 to -42.9)	4.69 (3.91 to 5.63)	3.03 (2.32 to 3.86)	-34.9 (-51.4 to -16.3)
		Chlamydia spp.	1320 (1190 to 1450)	536 (491 to 587)	-59.2 (-62.6 to -55.4)	5.08 (4.42 to 5.9)	2.15 (1.7 to 2.68)	-57.4 (-67.9 to -47.0)
		Enterobacter spp.	140 (122 to 161)	126 (112 to 141)	-10.2 (-19.5 to 0.1)	2.15 (1.76 to 2.6)	2.02 (1.6 to 2.51)	-5.0 (-29.6 to 22.5)
		Escherichia coli	445 (401 to 498)	307 (281 to 336)	-31.0 (-37.5 to -24.3)	7.48 (6.42 to 8.78)	5.47 (4.34 to 6.73)	-26.5 (-43.4 to -7.1)
		Fungus	306 (265 to 351)	226 (203 to 253)	-25.8 (-32.6 to -18.3)	3.22 (2.68 to 3.88)	3.16 (2.48 to 3.95)	-1.2 (-24.9 to 27.0)
		Group B Streptococcus	383 (340 to 433)	279 (254 to 306)	-27.2 (-33.8 to -19.5)	4.31 (3.73 to 5.08)	3.01 (2.39 to 3.71)	-29.9 (-46.7 to -11.1)
		Haemophilus influenzae	1850 (1680 to 2060)	806 (745 to 877)	-56.3 (-60.2 to -52.5)	12.2 (10.4 to 14.6)	2.94 (2.35 to 3.64)	-75.8 (-82.2 to -69.0)
		Influenza	2270 (2090 to 2470)	23.6 (9.15 to 50.9)	-99.0 (-99.6 to -97.7)	26.7 (22.8 to 31.2)	0.28 (0.1 to 0.58)	-99.0 (-99.6 to -97.8)
		Klebsiella pneumoniae	1160 (1070 to 1270)	663 (609 to 724)	-42.7 (-47.2 to -37.5)	19.2 (16.5 to 22.7)	10.6 (8.43 to 13.1)	-44.3 (-58.2 to -28.9)
		Legionella spp.	599 (534 to 671)	586 (536 to 645)	-2.0 (-10.8 to 7.8)	4.79 (4.04 to 5.63)	6.13 (4.83 to 7.58)	28.8 (-1.6 to 65.4)
		Mycoplasma spp.	2140 (1940 to 2390)	1100 (1010 to 1190)	-48.7 (-52.6 to -44.6)	6.58 (5.62 to 7.6)	3.2 (2.56 to 3.94)	-51.1 (-63.1 to -37.4)
		Polymicrobial	66.9 (47.1 to 95)	37.7 (24.3 to 58.7)	-44.0 (-52.2 to -35.5)	1.44 (0.98 to 2.12)	0.68 (0.42 to 1.11)	-52.2 (-69.1 to -33.0)
		Pseudomonas aeruginosa	911 (833 to 996)	931 (855 to 1020)	2.3 (-5.6 to 11.8)	9.73 (8.5 to 11.3)	10.5 (8.34 to 12.8)	8.0 (-18.1 to 35.1)
		Respiratory syncytial virus	1830 (1630 to 2080)	13 (5.08 to 28)	-99.3 (-99.7 to -98.5)	15.3 (12.5 to 19.1)	0.03 (0.01 to 0.07)	-99.8 (-99.9 to -99.5)
		Staphylococcus aureus	1380 (1270 to 1500)	1320 (1210 to 1440)	-4.6 (-11.2 to 3.4)	28.7 (24.9 to 33.1)	32 (25.5 to 39.4)	11.7 (-14.3 to 40.3)
		Streptococcus pneumoniae	11500 (10500 to 12600)	4530 (4200 to 4890)	-60.5 (-63.3 to -57.5)	103 (87.3 to 124)	33.5 (26.8 to 41.3)	-67.3 (-75.7 to -58.2)
		Other viruses	6040 (5500 to 6680)	3220 (2980 to 3500)	-46.6 (-50.8 to -42.3)	24.6 (20.4 to 30.2)	8.57 (6.91 to 10.5)	-64.8 (-74.3 to -54.1)
		Other bacterial pathogens	1460 (1300 to 1620)	903 (792 to 1020)	-38.2 (-43.3 to -32.5)	11.4 (9.77 to 13.4)	9.44 (7.34 to 12)	-17.1 (-36.8 to 3.8)
	Under 5	*Acinetobacter baumannii*	25.5 (17.8 to 35.5)	3.42 (2.35 to 4.94)	-86.6 (-88.3 to -84.9)	1.05 (0.66 to 1.67)	0.05 (0.02 to 0.08)	-95.6 (-97.3 to -93.2)
		Chlamydia spp.	311 (264 to 367)	43.5 (37 to 51)	-86.0 (-87.8 to -84.1)	2.34 (1.87 to 3)	0.06 (0.04 to 0.08)	-97.3 (-98.5 to -96.2)

		Enterobacter spp.	30.9 (24 to 39.7)	8 (6.18 to 10.4)	-74.0 (-77.6 to -70.0)	0.92 (0.66 to 1.26)	0.04 (0.03 to 0.07)	-95.1 (-97.2 to -93.0)
		Escherichia coli	108 (90.7 to 130)	22.6 (18.8 to 27)	-79.0 (-81.9 to -75.9)	3.2 (2.51 to 4.08)	0.12 (0.08 to 0.16)	-96.2 (-97.8 to -94.6)
		Fungus	20.7 (14.4 to 28.6)	4.24 (3.03 to 5.88)	-79.4 (-82.5 to -76.1)	0.52 (0.34 to 0.78)	0.01 (0.01 to 0.02)	-97.5 (-98.8 to -96.0)
		Group B Streptococcus	94 (77.3 to 112)	21.7 (18.2 to 26.3)	-76.8 (-79.7 to -73.6)	2.18 (1.75 to 2.77)	0.1 (0.07 to 0.13)	-95.3 (-97.2 to -93.2)
		Haemophilus influenzae	798 (680 to 940)	115 (96.3 to 137)	-85.5 (-87.5 to -83.2)	8.27 (6.46 to 10.5)	0.1 (0.06 to 0.14)	-98.7 (-99.4 to -98.1)
		Influenza	670 (578 to 777)	2.23 (0.86 to 4.92)	-99.7 (-99.9 to -99.3)	13.4 (10.5 to 17.2)	0.01 (0 to 0.01)	-100.0 (-100.0 to -99.9)
		Klebsiella pneumoniae	397 (341 to 464)	60.9 (51.5 to 72)	-84.6 (-86.6 to -82.7)	10.7 (8.41 to 13.8)	0.25 (0.16 to 0.33)	-97.6 (-98.7 to -96.5)
		Legionella spp.	36.2 (25.8 to 49.5)	9.2 (6.6 to 12.5)	-74.5 (-77.9 to -70.9)	0.86 (0.61 to 1.2)	0.05 (0.03 to 0.07)	-94.0 (-96.4 to -91.3)
		Mycoplasma spp.	458 (389 to 543)	101 (84.4 to 118)	-78.0 (-81.0 to -74.9)	3.27 (2.56 to 4.22)	0.1 (0.06 to 0.13)	-97.0 (-98.4 to -95.5)
		Polymicrobial	17.4 (10.8 to 26.7)	3.48 (2.03 to 5.48)	-80.0 (-82.8 to -76.9)	0.85 (0.49 to 1.39)	0.07 (0.03 to 0.12)	-92.2 (-95.2 to -88.4)
		Pseudomonas aeruginosa	215 (184 to 255)	68.9 (58.4 to 82.2)	-67.9 (-72.1 to -63.4)	4.27 (3.4 to 5.48)	0.21 (0.14 to 0.28)	-95.0 (-97.2 to -92.8)
		Respiratory syncytial virus	1520 (1320 to 1740)	7.71 (2.98 to 16.8)	-99.5 (-99.8 to -98.9)	14.4 (11.7 to 18.1)	0.01 (0 to 0.02)	-99.9 (-100.0 to -99.8)
		Staphylococcus aureus	273 (237 to 320)	75.1 (63.5 to 88.3)	-72.5 (-75.7 to -69.3)	10.4 (8.25 to 13.3)	0.39 (0.25 to 0.53)	-96.2 (-98.0 to -94.2)
		Streptococcus pneumoniae	4410 (3830 to 5100)	637 (539 to 752)	-85.5 (-87.1 to -83.9)	66.9 (52.5 to 86.6)	1.22 (0.76 to 1.69)	-98.1 (-99.1 to -97.1)
		Other viruses	3150 (2740 to 3630)	654 (554 to 767)	-79.2 (-81.4 to -76.9)	19 (14.9 to 24.4)	0.52 (0.32 to 0.71)	-97.2 (-98.6 to -95.7)
		Other bacterial pathogens	121 (95 to 150)	19.5 (15.2 to 24.6)	-83.8 (-86.2 to -81.4)	2.01 (1.51 to 2.67)	0.05 (0.03 to 0.08)	-97.3 (-98.5 to -95.9)
	5-14 years	*Acinetobacter baumannii*	58.5 (44.8 to 75.3)	13.6 (10.2 to 17.6)	-76.7 (-79.8 to -73.6)	0.09 (0.07 to 0.12)	0.01 (0.01 to 0.02)	-87.0 (-90.8 to -81.9)
		Chlamydia spp.	252 (198 to 318)	56.6 (43.2 to 73)	-77.5 (-80.5 to -74.4)	0.1 (0.08 to 0.12)	0.01 (0.01 to 0.02)	-87.4 (-91.1 to -82.4)
		Enterobacter spp.	21.7 (16.8 to 28.4)	10 (7.57 to 13.3)	-53.7 (-60.5 to -47.0)	0.03 (0.03 to 0.04)	0.01 (0.01 to 0.01)	-74.1 (-81.8 to -64.0)
		Escherichia coli	58 (45.2 to 73.1)	20.3 (15.4 to 25.9)	-64.9 (-69.6 to -60.0)	0.09 (0.07 to 0.11)	0.02 (0.01 to 0.02)	-80.3 (-86.2 to -72.6)
		Fungus	69.9 (53.5 to 90)	25 (19 to 32.4)	-64.2 (-68.8 to -59.6)	0.11 (0.08 to 0.14)	0.02 (0.02 to 0.03)	-79.9 (-85.8 to -72.0)
		Group B Streptococcus	71.7 (55 to 91.2)	29.1 (22.2 to 37.7)	-59.3 (-65.0 to -53.5)	0.08 (0.06 to 0.1)	0.02 (0.01 to 0.02)	-77.2 (-84.0 to -68.3)
		Haemophilus influenzae	284 (227 to 354)	86.3 (66.2 to 109)	-69.6 (-73.6 to -65.8)	0.17 (0.14 to 0.21)	0.02 (0.01 to 0.03)	-88.1 (-91.7 to -83.5)
		Influenza	407 (323 to 504)	2.46 (0.87 to 5.53)	-99.4 (-99.8 to -98.7)	0.48 (0.38 to 0.58)	0 (0 to 0)	-99.7 (-99.9 to -99.3)

		Klebsiella pneumoniae	170 (135 to 213)	57.7 (44.5 to 73.4)	-66.1 (-70.3 to -62.0)	0.26 (0.21 to 0.32)	0.05 (0.04 to 0.06)	-81.0 (-86.6 to -73.5)
		Legionella spp.	134 (106 to 168)	61.9 (47.7 to 78.1)	-53.6 (-59.7 to -47.2)	0.14 (0.11 to 0.18)	0.04 (0.03 to 0.05)	-74.0 (-81.5 to -63.5)
		Mycoplasma spp.	636 (498 to 792)	207 (160 to 266)	-67.4 (-71.6 to -63.0)	0.26 (0.21 to 0.32)	0.05 (0.04 to 0.06)	-81.8 (-87.1 to -74.5)
		Polymicrobial	10.3 (6.48 to 15.6)	3.06 (1.79 to 4.87)	-70.4 (-75.0 to -65.8)	0.02 (0.01 to 0.03)	0 (0 to 0)	-83.4 (-88.4 to -76.8)
		Pseudomonas aeruginosa	149 (116 to 186)	81.1 (62.2 to 104)	-45.5 (-52.8 to -38.4)	0.16 (0.13 to 0.2)	0.05 (0.04 to 0.06)	-69.5 (-78.6 to -58.0)
		Respiratory syncytial virus	103 (80.9 to 128)	0.88 (0.31 to 1.97)	-99.1 (-99.7 to -98.1)	0.05 (0.04 to 0.07)	0 (0 to 0)	-99.5 (-99.8 to -98.9)
		Staphylococcus aureus	240 (190 to 296)	117 (90.4 to 149)	-51.3 (-57.4 to -45.7)	0.53 (0.42 to 0.64)	0.14 (0.11 to 0.19)	-72.7 (-80.7 to -62.5)
		Streptococcus pneumoniae	2320 (1850 to 2860)	639 (495 to 814)	-72.5 (-76.0 to -69.2)	2.13 (1.7 to 2.59)	0.32 (0.25 to 0.42)	-84.6 (-89.1 to -78.7)
		Other viruses	1000 (798 to 1230)	452 (348 to 572)	-54.9 (-60.7 to -49.6)	0.36 (0.29 to 0.44)	0.09 (0.07 to 0.12)	-74.8 (-82.0 to -65.1)
		Other bacterial pathogens	254 (198 to 327)	70.5 (53.4 to 90.4)	-72.2 (-75.8 to -68.7)	0.22 (0.17 to 0.28)	0.03 (0.03 to 0.05)	-84.4 (-89.0 to -78.4)
	15-49 years	*Acinetobacter baumannii*	47.2 (39 to 57.1)	20.3 (16.8 to 24.3)	-57.1 (-61.1 to -53.0)	0.3 (0.24 to 0.38)	0.12 (0.08 to 0.16)	-61.0 (-72.6 to -47.3)
		Chlamydia spp.	203 (174 to 238)	84.3 (72.4 to 98.5)	-58.5 (-61.9 to -54.6)	0.32 (0.27 to 0.39)	0.12 (0.09 to 0.16)	-62.3 (-73.4 to -48.9)
		Enterobacter spp.	17.5 (14.4 to 21.1)	14.9 (12.5 to 17.8)	-14.6 (-24.1 to -5.0)	0.11 (0.08 to 0.13)	0.08 (0.06 to 0.11)	-22.4 (-46.0 to 6.2)
		Escherichia coli	46.8 (39.8 to 54.9)	30.3 (26.1 to 35)	-35.2 (-40.9 to -28.5)	0.3 (0.24 to 0.36)	0.17 (0.13 to 0.23)	-41.1 (-58.8 to -19.9)
		Fungus	56.4 (46.8 to 66.2)	37.2 (31.5 to 42.9)	-33.9 (-40.0 to -27.2)	0.35 (0.28 to 0.43)	0.21 (0.15 to 0.28)	-40.0 (-57.7 to -18.7)
		Group B Streptococcus	57.8 (49 to 68.4)	43.4 (37.2 to 50.9)	-24.8 (-31.8 to -17.5)	0.26 (0.21 to 0.32)	0.17 (0.13 to 0.23)	-31.7 (-52.0 to -6.0)
		Haemophilus influenzae	230 (199 to 265)	128 (112 to 147)	-44.0 (-48.4 to -39.3)	0.55 (0.46 to 0.66)	0.19 (0.14 to 0.26)	-64.5 (-75.0 to -52.2)
		Influenza	328 (287 to 376)	3.65 (1.39 to 7.91)	-98.9 (-99.6 to -97.6)	1.54 (1.29 to 1.84)	0.01 (0.01 to 0.03)	-99.0 (-99.6 to -97.9)
		Klebsiella pneumoniae	138 (120 to 158)	86 (74.9 to 98.5)	-37.4 (-42.3 to -32.2)	0.86 (0.72 to 1.04)	0.48 (0.36 to 0.64)	-43.2 (-60.0 to -23.1)
		Legionella spp.	108 (91.7 to 126)	92.2 (79.9 to 105)	-14.3 (-21.9 to -5.6)	0.47 (0.38 to 0.57)	0.36 (0.26 to 0.47)	-22.2 (-45.9 to 6.2)
		Mycoplasma spp.	513 (446 to 587)	308 (268 to 353)	-39.9 (-44.8 to -34.9)	0.85 (0.71 to 1.02)	0.46 (0.34 to 0.61)	-45.4 (-61.5 to -26.1)
		Polymicrobial	8.3 (5.32 to 12.4)	4.56 (2.72 to 7.32)	-45.3 (-51.8 to -38.1)	0.05 (0.03 to 0.08)	0.03 (0.02 to 0.04)	-50.4 (-65.6 to -31.9)
		Pseudomonas aeruginosa	120 (104 to 139)	121 (105 to 139)	0.5 (-7.8 to 9.3)	0.52 (0.44 to 0.63)	0.47 (0.35 to 0.63)	-8.8 (-36.1 to 23.1)
		Respiratory syncytial virus	82.8 (71.5 to 95.2)	1.31 (0.49 to 2.83)	-98.4 (-99.4 to -96.6)	0.18 (0.15 to 0.22)	0 (0 to 0.01)	-98.6 (-99.5 to -97.0)

		Staphylococcus aureus	193 (170 to 223)	174 (152 to 199)	-10.1 (-16.7 to -3.0)	1.72 (1.44 to 2.06)	1.39 (1.02 to 1.84)	-18.4 (-42.2 to 11.4)
		Streptococcus pneumoniae	1870 (1650 to 2140)	951 (834 to 1090)	-49.2 (-52.8 to -45.4)	6.94 (5.83 to 8.23)	3.18 (2.32 to 4.19)	-53.9 (-67.3 to -37.4)
		Other viruses	809 (712 to 920)	672 (587 to 769)	-16.8 (-23.1 to -10.6)	1.18 (0.99 to 1.4)	0.88 (0.64 to 1.16)	-24.5 (-46.3 to 2.8)
		Other bacterial pathogens	205 (174 to 240)	105 (88.4 to 124)	-48.7 (-52.8 to -44.3)	0.72 (0.59 to 0.88)	0.34 (0.24 to 0.44)	-53.3 (-67.0 to -36.1)
	50-69 years	*Acinetobacter baumannii*	103 (86 to 120)	64.7 (54.9 to 75.8)	-36.8 (-43.5 to -29.1)	1.05 (0.83 to 1.32)	0.77 (0.55 to 1.04)	-25.2 (-49.2 to 3.5)
		Chlamydia spp.	261 (226 to 300)	156 (137 to 179)	-40.0 (-46.0 to -33.2)	0.66 (0.53 to 0.81)	0.47 (0.33 to 0.62)	-29.1 (-51.3 to -0.8)
		Enterobacter spp.	36.9 (31 to 43.9)	45.9 (39.4 to 53.1)	24.8 (10.1 to 41.3)	0.36 (0.28 to 0.45)	0.52 (0.38 to 0.71)	47.7 (-0.1 to 107.2)
		Escherichia coli	109 (93.5 to 124)	102 (90.3 to 115)	-6.0 (-16.2 to 4.9)	1.09 (0.87 to 1.34)	1.2 (0.87 to 1.61)	11.2 (-24.4 to 55.1)
		Fungus	104 (88.8 to 122)	101 (87.2 to 116)	-3.3 (-14.2 to 8.3)	1.03 (0.8 to 1.31)	1.17 (0.83 to 1.56)	14.4 (-21.5 to 58.6)
		Group B Streptococcus	86.3 (73.9 to 101)	93.9 (81.8 to 107)	9.1 (-2.8 to 22.8)	0.61 (0.49 to 0.76)	0.78 (0.57 to 1.06)	29.1 (-12.9 to 78.4)
		Haemophilus influenzae	292 (256 to 331)	245 (217 to 276)	-16.0 (-24.5 to -6.3)	1.11 (0.9 to 1.36)	0.76 (0.56 to 1.02)	-30.7 (-52.3 to -5.1)
		Influenza	360 (314 to 403)	5.87 (2.19 to 12.7)	-98.4 (-99.4 to -96.4)	2.69 (2.17 to 3.25)	0.05 (0.02 to 0.1)	-98.1 (-99.4 to -95.9)
		Klebsiella pneumoniae	224 (197 to 250)	213 (190 to 238)	-5.0 (-14.3 to 5.8)	2.23 (1.81 to 2.71)	2.48 (1.8 to 3.31)	12.3 (-22.9 to 54.5)
		Legionella spp.	193 (166 to 220)	241 (213 to 272)	25.3 (11.5 to 39.6)	1.32 (1.04 to 1.63)	1.94 (1.4 to 2.62)	48.2 (1.5 to 107.7)
		Mycoplasma spp.	299 (261 to 338)	256 (226 to 288)	-14.4 (-23.0 to -5.1)	0.79 (0.64 to 0.95)	0.79 (0.58 to 1.07)	1.2 (-30.4 to 41.9)
		Polymicrobial	15 (9.96 to 22.9)	12.1 (7.33 to 19.2)	-19.8 (-30.7 to -8.1)	0.15 (0.1 to 0.24)	0.14 (0.08 to 0.25)	-5.2 (-36.1 to 32.2)
		Pseudomonas aeruginosa	217 (188 to 245)	316 (279 to 353)	45.8 (30.8 to 62.7)	1.5 (1.2 to 1.84)	2.56 (1.85 to 3.48)	72.4 (18.1 to 140.8)
		Respiratory syncytial virus	67.2 (58 to 76.7)	1.54 (0.57 to 3.33)	-97.7 (-99.1 to -95.0)	0.23 (0.18 to 0.28)	0.01 (0 to 0.01)	-97.4 (-99.1 to -94.3)
		Staphylococcus aureus	307 (270 to 342)	404 (359 to 452)	31.8 (18.9 to 45.7)	4.34 (3.51 to 5.21)	6.7 (4.89 to 8.93)	55.9 (6.6 to 115.6)
		Streptococcus pneumoniae	1470 (1290 to 1630)	1110 (986 to 1230)	-24.6 (-32.0 to -16.6)	8.64 (7.03 to 10.4)	7.63 (5.61 to 10.3)	-10.9 (-38.5 to 23.2)
		Other viruses	555 (488 to 616)	698 (621 to 779)	25.9 (13.7 to 40.1)	1.28 (1.04 to 1.55)	1.89 (1.38 to 2.54)	48.9 (2.7 to 105.6)
		Other bacterial pathogens	393 (338 to 452)	295 (254 to 341)	-25.0 (-33.0 to -16.6)	2.21 (1.77 to 2.71)	1.94 (1.4 to 2.62)	-11.2 (-39.8 to 21.9)
	70+ years	*Acinetobacter baumannii*	95 (79.8 to 113)	67.8 (57.1 to 80.6)	-28.5 (-36.8 to -19.0)	2.2 (1.75 to 2.73)	2.08 (1.6 to 2.68)	-4.5 (-27.8 to 22.9)
		Chlamydia spp.	289 (248 to 332)	195 (170 to 228)	-32.2 (-39.2 to -23.3)	1.66 (1.36 to 1.98)	1.49 (1.17 to 1.86)	-9.4 (-31.5 to 15.3)

		Enterobacter spp.	33.4 (27.3 to 39.8)	46.9 (39.7 to 54.9)	41.1 (23.8 to 61.4)	0.73 (0.57 to 0.91)	1.37 (1.07 to 1.7)	88.7 (39.6 to 144.6)
		Escherichia coli	124 (107 to 144)	131 (114 to 151)	6.3 (-5.5 to 20.6)	2.81 (2.31 to 3.35)	3.96 (3.15 to 4.91)	42.1 (5.9 to 79.9)
		Fungus	54 (45.3 to 64)	58.9 (50.3 to 68.7)	9.4 (-3.0 to 24.1)	1.21 (0.97 to 1.47)	1.75 (1.36 to 2.19)	46.2 (10.2 to 87.3)
		Group B Streptococcus	73.4 (63.1 to 86.2)	90.3 (78.3 to 105)	23.4 (9.1 to 39.4)	1.18 (0.96 to 1.43)	1.93 (1.54 to 2.42)	64.9 (23.6 to 110.7)
		Haemophilus influenzae	244 (212 to 276)	231 (203 to 264)	-5.1 (-14.4 to 6.8)	2.11 (1.74 to 2.5)	1.86 (1.47 to 2.32)	-11.5 (-32.2 to 11.9)
		Influenza	508 (445 to 572)	9.35 (3.59 to 20.5)	-98.2 (-99.3 to -96.0)	8.58 (7.14 to 10.1)	0.2 (0.07 to 0.44)	-97.6 (-99.2 to -94.9)
		Klebsiella pneumoniae	229 (201 to 260)	246 (216 to 281)	7.4 (-3.3 to 20.7)	5.16 (4.32 to 6.1)	7.36 (5.91 to 9.15)	43.5 (9.0 to 81.0)
		Legionella spp.	129 (111 to 149)	182 (159 to 210)	41.6 (25.7 to 60.2)	1.99 (1.64 to 2.4)	3.75 (2.97 to 4.64)	89.4 (42.6 to 142.8)
		Mycoplasma spp.	235 (203 to 268)	227 (199 to 262)	-3.2 (-13.3 to 9.0)	1.4 (1.16 to 1.66)	1.8 (1.43 to 2.26)	29.3 (-2.8 to 64.9)
		Polymicrobial	15.9 (10.3 to 24.3)	14.5 (8.69 to 23.4)	-9.4 (-21.8 to 4.4)	0.37 (0.23 to 0.57)	0.44 (0.25 to 0.75)	21.1 (-10.4 to 54.0)
		Pseudomonas aeruginosa	210 (182 to 239)	345 (303 to 395)	64.8 (48.2 to 86.5)	3.28 (2.72 to 3.87)	7.17 (5.74 to 8.96)	120.3 (66.1 to 182.0)
		Respiratory syncytial virus	58.5 (50.2 to 67.1)	1.51 (0.58 to 3.29)	-97.4 (-99.0 to -94.4)	0.45 (0.37 to 0.54)	0.01 (0.01 to 0.03)	-96.6 (-98.8 to -92.7)
		Staphylococcus aureus	368 (321 to 416)	548 (483 to 628)	49.0 (35.2 to 66.6)	11.8 (9.91 to 13.8)	23.3 (18.7 to 28.9)	99.2 (51.1 to 152.2)
		Streptococcus pneumoniae	1400 (1230 to 1580)	1190 (1050 to 1370)	-14.8 (-23.1 to -5.0)	18.7 (15.6 to 21.8)	21.1 (16.8 to 26.3)	13.9 (-13.2 to 44.6)
		Other viruses	523 (458 to 589)	744 (657 to 851)	42.4 (28.5 to 59.3)	2.74 (2.27 to 3.21)	5.18 (4.12 to 6.47)	90.3 (45.8 to 141.5)
		Other bacterial pathogens	488 (415 to 564)	413 (350 to 485)	-15.2 (-23.8 to -4.5)	6.27 (5.17 to 7.53)	7.07 (5.48 to 9.01)	13.5 (-14.6 to 46.3)
Poland	All Ages	*Acinetobacter baumannii*	6580 (5730 to 7480)	4590 (4060 to 5180)	-30.1 (-35.6 to -24.2)	151 (134 to 171)	273 (236 to 316)	80.7 (59.7 to 102.5)
		Chlamydia spp.	23100 (21100 to 25200)	14600 (13700 to 15700)	-36.4 (-41.1 to -31.4)	122 (111 to 132)	201 (175 to 225)	65.6 (47.1 to 86.3)
		Enterobacter spp.	2740 (2380 to 3130)	4510 (4090 to 4980)	65.2 (49.4 to 81.5)	57 (49.7 to 65.3)	243 (207 to 277)	326.9 (270.7 to 388.4)
		Escherichia coli	8650 (7830 to 9600)	10800 (10100 to 11500)	24.9 (15.3 to 35.0)	198 (180 to 217)	622 (549 to 687)	214.7 (178.4 to 253.4)
		Fungus	6040 (5320 to 6870)	6820 (6240 to 7500)	13.2 (4.1 to 23.3)	111 (98.9 to 124)	345 (303 to 388)	210.6 (173.4 to 249.2)
		Group B Streptococcus	6910 (6210 to 7740)	9110 (8530 to 9720)	32.2 (20.1 to 43.5)	99.1 (90.4 to 109)	338 (297 to 374)	241.7 (199.0 to 283.0)
		Haemophilus influenzae	28400 (26300 to 30800)	24100 (22700 to 25500)	-15.3 (-20.4 to -10.2)	195 (184 to 206)	296 (262 to 327)	51.9 (36.9 to 68.3)
		Influenza	39600 (37000 to 42400)	1290 (147 to 4580)	-96.8 (-99.6 to -88.6)	619 (583 to 653)	54.8 (5.83 to 189)	-91.1 (-99.0 to -69.5)

		Klebsiella pneumoniae	19700 (18400 to 21200)	20700 (19600 to 21900)	5.4 (-0.5 to 11.1)	397 (375 to 419)	1130 (999 to 1240)	184.0 (155.5 to 212.2)
		Legionella spp.	12700 (11400 to 14100)	19300 (18100 to 20600)	52.5 (39.1 to 66.0)	174 (158 to 191)	714 (633 to 792)	310.6 (261.7 to 364.1)
		Mycoplasma spp.	33700 (30900 to 36800)	28800 (27300 to 30400)	-14.4 (-20.3 to -8.1)	141 (131 to 150)	316 (281 to 348)	125.0 (100.8 to 149.4)
		Polymicrobial	1240 (854 to 1820)	1230 (772 to 1940)	-1.4 (-13.6 to 10.5)	29.4 (20.6 to 42.8)	68.1 (41.6 to 110)	129.2 (86.4 to 174.2)
		Pseudomonas aeruginosa	17600 (16300 to 19100)	33600 (31700 to 35600)	91.2 (79.4 to 103.4)	258 (240 to 275)	1260 (1120 to 1400)	390.5 (336.3 to 446.0)
		Respiratory syncytial virus	19500 (17700 to 21500)	712 (78.5 to 2540)	-96.4 (-99.6 to -87.4)	91.6 (86.7 to 96.7)	5.09 (0.54 to 17.7)	-94.4 (-99.4 to -80.7)
		Staphylococcus aureus	27000 (25200 to 28900)	48700 (46100 to 51600)	80.4 (72.4 to 88.5)	841 (791 to 881)	3980 (3500 to 4380)	373.4 (328.0 to 420.0)
		Streptococcus pneumoniae	171000 (160000 to 184000)	107000 (102000 to 113000)	-37.1 (-40.2 to -33.9)	1690 (1620 to 1760)	3010 (2680 to 3300)	78.0 (61.0 to 95.1)
		Other viruses	82600 (76800 to 89300)	93400 (88500 to 98300)	13.2 (7.4 to 19.8)	296 (284 to 308)	917 (814 to 1010)	210.3 (180.5 to 240.2)
		Other bacterial pathogens	31000 (27900 to 34500)	28100 (25000 to 31500)	-9.3 (-14.9 to -3.3)	413 (373 to 451)	971 (828 to 1120)	134.8 (108.5 to 161.2)
	Under 5	*Acinetobacter baumannii*	235 (160 to 334)	103 (68.4 to 154)	-56.2 (-61.5 to -49.7)	5.21 (3.22 to 8.1)	0.71 (0.37 to 1.25)	-86.5 (-90.1 to -82.1)
		Chlamydia spp.	2610 (2240 to 3030)	1150 (996 to 1310)	-56.0 (-60.9 to -50.6)	8.78 (7.92 to 9.74)	0.97 (0.75 to 1.21)	-89.0 (-91.7 to -86.0)
		Enterobacter spp.	273 (211 to 349)	274 (213 to 350)	0.8 (-12.6 to 15.7)	3.77 (2.79 to 5.15)	0.85 (0.58 to 1.23)	-77.3 (-83.1 to -71.1)
		Escherichia coli	925 (785 to 1100)	791 (675 to 919)	-14.2 (-25.2 to -2.5)	12.3 (10.9 to 13.9)	2.33 (1.8 to 2.92)	-81.1 (-85.4 to -76.0)
		Fungus	170 (119 to 236)	129 (93.8 to 175)	-23.4 (-35.0 to -11.1)	1.6 (1.13 to 2.16)	0.25 (0.17 to 0.35)	-84.2 (-88.3 to -79.9)
		Group B Streptococcus	839 (703 to 999)	723 (618 to 846)	-13.7 (-24.3 to -2.8)	9.44 (8.38 to 10.6)	1.82 (1.4 to 2.28)	-80.7 (-85.2 to -75.4)
		Haemophilus influenzae	6780 (5790 to 7920)	3500 (3030 to 4030)	-48.4 (-54.6 to -41.6)	28.7 (26 to 31.8)	1.86 (1.43 to 2.3)	-93.5 (-95.1 to -91.8)
		Influenza	5640 (4920 to 6450)	97.4 (10.5 to 350)	-98.3 (-99.8 to -94.0)	45 (40.9 to 49)	0.16 (0.02 to 0.6)	-99.6 (-100.0 to -98.7)
		Klebsiella pneumoniae	3310 (2860 to 3790)	1690 (1460 to 1940)	-48.8 (-54.2 to -43.2)	36.2 (33 to 39.8)	4.23 (3.29 to 5.22)	-88.3 (-91.0 to -85.3)
		Legionella spp.	341 (251 to 455)	326 (242 to 432)	-4.0 (-16.1 to 9.2)	4.07 (3.1 to 5.23)	0.96 (0.67 to 1.29)	-76.4 (-82.1 to -69.4)
		Mycoplasma spp.	3870 (3320 to 4490)	3790 (3290 to 4330)	-1.7 (-13.2 to 10.1)	11.2 (10 to 12.5)	2.21 (1.71 to 2.76)	-80.2 (-85.1 to -74.9)
		Polymicrobial	182 (109 to 289)	133 (74.3 to 216)	-27.4 (-37.0 to -16.4)	4.98 (2.78 to 7.87)	1.16 (0.56 to 2.05)	-76.8 (-83.2 to -69.2)
		Pseudomonas aeruginosa	1900 (1630 to 2200)	2770 (2400 to 3170)	46.4 (30.3 to 63.7)	16.4 (14.8 to 18.1)	4.8 (3.74 to 5.94)	-70.6 (-77.5 to -63.0)
		Respiratory syncytial virus	13400 (11800 to 15300)	451 (48.6 to 1620)	-96.6 (-99.6 to -88.4)	53.1 (49.4 to 57.3)	0.37 (0.04 to 1.34)	-99.3 (-99.9 to -97.5)

		Staphylococcus aureus	2320 (2030 to 2650)	2510 (2170 to 2870)	8.1 (-0.8 to 17.1)	35.5 (32.8 to 38.3)	8.06 (6.31 to 9.91)	-77.3 (-82.5 to -71.4)
		Streptococcus pneumoniae	36600 (31900 to 42100)	11800 (10200 to 13400)	-67.8 (-70.4 to -65.3)	212 (196 to 228)	14.6 (11.3 to 18)	-93.1 (-94.7 to -91.3)
		Other viruses	27200 (23700 to 31100)	18200 (16000 to 20700)	-32.9 (-38.1 to -27.8)	63.8 (59.1 to 69)	9.29 (7.2 to 11.4)	-85.4 (-88.7 to -81.8)
		Other bacterial pathogens	1010 (802 to 1230)	583 (462 to 722)	-42.1 (-49.4 to -34.2)	7.13 (5.67 to 8.85)	0.95 (0.68 to 1.29)	-86.7 (-90.0 to -83.2)
	5-14 years	*Acinetobacter baumannii*	617 (461 to 818)	177 (142 to 219)	-71.2 (-75.0 to -66.8)	1.09 (0.94 to 1.24)	0.29 (0.24 to 0.33)	-73.7 (-76.5 to -70.7)
		Chlamydia spp.	2760 (2080 to 3580)	778 (637 to 942)	-71.6 (-75.3 to -67.2)	1.2 (1.09 to 1.33)	0.31 (0.28 to 0.34)	-74.0 (-76.8 to -71.2)
		Enterobacter spp.	248 (182 to 329)	175 (140 to 217)	-28.7 (-38.5 to -17.2)	0.41 (0.36 to 0.48)	0.27 (0.24 to 0.3)	-34.9 (-42.7 to -26.5)
		Escherichia coli	649 (492 to 843)	335 (275 to 405)	-48.1 (-55.0 to -40.8)	1.12 (1.01 to 1.22)	0.53 (0.48 to 0.58)	-52.6 (-57.6 to -47.4)
		Fungus	783 (584 to 1040)	401 (326 to 495)	-48.4 (-55.7 to -40.7)	1.33 (1.18 to 1.49)	0.63 (0.56 to 0.7)	-52.9 (-58.3 to -47.5)
		Group B Streptococcus	811 (609 to 1050)	487 (393 to 596)	-39.5 (-47.9 to -30.1)	0.99 (0.89 to 1.11)	0.55 (0.49 to 0.6)	-44.7 (-51.1 to -38.6)
		Haemophilus influenzae	3250 (2470 to 4260)	1320 (1100 to 1610)	-59.1 (-64.1 to -53.9)	2.14 (1.99 to 2.29)	0.54 (0.49 to 0.58)	-74.8 (-77.3 to -72.5)
		Influenza	4630 (3560 to 5960)	67 (7.32 to 242)	-98.6 (-99.8 to -94.9)	5.96 (5.66 to 6.27)	0.08 (0.01 to 0.28)	-98.7 (-99.9 to -95.3)
		Klebsiella pneumoniae	1910 (1460 to 2480)	877 (731 to 1060)	-53.8 (-59.3 to -47.4)	3.27 (3.09 to 3.47)	1.38 (1.27 to 1.48)	-57.8 (-61.5 to -54.0)
		Legionella spp.	1570 (1180 to 2050)	1050 (869 to 1270)	-32.4 (-41.3 to -22.1)	1.85 (1.67 to 2.05)	1.14 (1.04 to 1.23)	-38.2 (-45.4 to -31.3)
		Mycoplasma spp.	7230 (5520 to 9270)	3120 (2580 to 3790)	-56.6 (-61.8 to -50.5)	3.3 (3.1 to 3.5)	1.31 (1.21 to 1.4)	-60.3 (-64.0 to -56.7)
		Polymicrobial	112 (69.9 to 173)	47.3 (27.8 to 75.8)	-57.9 (-64.9 to -50.4)	0.2 (0.13 to 0.3)	0.08 (0.05 to 0.12)	-61.6 (-66.4 to -56.0)
		Pseudomonas aeruginosa	1740 (1330 to 2230)	1430 (1180 to 1710)	-17.4 (-28.0 to -5.9)	2.07 (1.93 to 2.22)	1.56 (1.43 to 1.68)	-24.5 (-32.1 to -17.1)
		Respiratory syncytial virus	1210 (929 to 1580)	24.5 (2.62 to 88.5)	-98.0 (-99.8 to -92.8)	0.71 (0.66 to 0.77)	0.01 (0 to 0.05)	-98.1 (-99.8 to -93.4)
		Staphylococcus aureus	2750 (2120 to 3520)	2080 (1730 to 2500)	-23.8 (-32.6 to -14.2)	6.7 (6.38 to 6.99)	4.66 (4.29 to 4.98)	-30.4 (-36.5 to -24.8)
		Streptococcus pneumoniae	26300 (20200 to 33700)	8540 (7120 to 10300)	-67.3 (-71.0 to -63.2)	26.7 (25.7 to 27.7)	7.96 (7.34 to 8.47)	-70.2 (-72.8 to -67.8)
		Other viruses	11800 (9020 to 15000)	7290 (6080 to 8800)	-37.6 (-45.0 to -29.5)	4.69 (4.49 to 4.9)	2.67 (2.46 to 2.85)	-43.0 (-48.2 to -38.2)
		Other bacterial pathogens	2830 (2150 to 3680)	1030 (835 to 1280)	-63.4 (-68.2 to -58.2)	2.74 (2.48 to 3.02)	0.92 (0.8 to 1.03)	-66.5 (-69.9 to -63.3)
	15-49 years	*Acinetobacter baumannii*	686 (568 to 815)	317 (270 to 368)	-53.7 (-58.0 to -49.1)	9.07 (7.87 to 10.4)	8.87 (7.45 to 10.3)	-2.1 (-14.5 to 10.7)
		Chlamydia spp.	3060 (2660 to 3520)	1400 (1260 to 1550)	-54.3 (-58.2 to -50.2)	10.1 (9.14 to 11.1)	9.7 (8.47 to 11)	-3.4 (-15.0 to 8.9)

		Enterobacter spp.	275 (230 to 325)	314 (273 to 357)	14.6 (2.3 to 27.5)	3.45 (3.02 to 3.96)	8.34 (7.19 to 9.62)	142.5 (109.3 to 179.2)
		Escherichia coli	721 (626 to 829)	600 (544 to 661)	-16.6 (-24.0 to -8.8)	9.35 (8.49 to 10.2)	16.5 (14.6 to 18.3)	76.5 (54.5 to 100.3)
		Fungus	869 (739 to 1010)	718 (642 to 806)	-17.1 (-24.8 to -8.5)	11.1 (9.83 to 12.5)	19.4 (17.1 to 22.1)	75.3 (53.3 to 97.9)
		Group B Streptococcus	900 (779 to 1050)	873 (786 to 976)	-2.8 (-12.2 to 6.5)	8.29 (7.44 to 9.29)	17 (15.1 to 19.2)	105.7 (78.7 to 131.9)
		Haemophilus influenzae	3610 (3190 to 4070)	2370 (2170 to 2580)	-34.3 (-38.8 to -29.4)	17.9 (16.6 to 19.1)	16.7 (15 to 18.5)	-6.3 (-16.9 to 4.6)
		Influenza	5140 (4570 to 5780)	120 (13 to 422)	-97.7 (-99.7 to -91.8)	49.7 (47.4 to 52.5)	2.5 (0.26 to 8.64)	-95.0 (-99.5 to -82.7)
		Klebsiella pneumoniae	2120 (1890 to 2370)	1570 (1440 to 1710)	-25.7 (-30.8 to -20.5)	27.3 (25.9 to 28.8)	42.9 (38.5 to 47.2)	57.1 (39.8 to 75.3)
		Legionella spp.	1740 (1500 to 1990)	1890 (1730 to 2060)	8.7 (-1.5 to 19.6)	15.5 (14 to 17)	35.5 (31.6 to 39.7)	129.9 (101.8 to 160.9)
		Mycoplasma spp.	8030 (7120 to 9010)	5590 (5130 to 6090)	-30.2 (-35.4 to -24.7)	27.5 (25.9 to 29.2)	40.5 (36.3 to 44.9)	47.6 (30.1 to 64.5)
		Polymicrobial	125 (79.7 to 188)	84.9 (50.2 to 138)	-32.4 (-41.0 to -23.3)	1.64 (1.06 to 2.47)	2.36 (1.41 to 3.82)	43.1 (22.2 to 65.1)
		Pseudomonas aeruginosa	1930 (1700 to 2180)	2560 (2340 to 2790)	32.8 (22.7 to 43.7)	17.3 (16.1 to 18.5)	48.4 (43.5 to 53.9)	181.0 (147.1 to 215.4)
		Respiratory syncytial virus	1340 (1180 to 1520)	43.7 (4.64 to 153)	-96.7 (-99.6 to -88.5)	5.93 (5.47 to 6.41)	0.42 (0.04 to 1.45)	-93.0 (-99.3 to -75.6)
		Staphylococcus aureus	3050 (2720 to 3400)	3730 (3430 to 4050)	22.5 (15.1 to 30.2)	55.9 (53.4 to 58.3)	145 (130 to 159)	159.1 (130.7 to 186.7)
		Streptococcus pneumoniae	29200 (26100 to 32400)	15300 (14100 to 16600)	-47.5 (-50.3 to -44.6)	223 (215 to 231)	247 (223 to 272)	11.1 (-1.0 to 22.2)
		Other viruses	13100 (11700 to 14500)	13100 (12000 to 14100)	0.2 (-5.3 to 6.1)	39.1 (37.6 to 40.8)	83 (74.3 to 91.4)	112.1 (89.3 to 134.7)
		Other bacterial pathogens	3140 (2700 to 3600)	1850 (1610 to 2110)	-41.2 (-45.7 to -36.5)	22.8 (20.8 to 25.1)	28.5 (24.4 to 32.8)	24.7 (9.7 to 39.6)
	50-69 years	*Acinetobacter baumannii*	2460 (2110 to 2840)	1530 (1330 to 1740)	-37.6 (-42.5 to -32.5)	38.5 (34 to 43.5)	63.4 (53.9 to 73.8)	64.7 (42.7 to 87.1)
		Chlamydia spp.	6490 (5770 to 7290)	3880 (3530 to 4270)	-40.1 (-44.4 to -35.6)	25.3 (22.9 to 27.6)	39.9 (34.8 to 45.2)	58.0 (38.2 to 79.0)
		Enterobacter spp.	958 (819 to 1120)	1460 (1300 to 1620)	52.6 (38.0 to 68.8)	14.2 (12.5 to 16.2)	57.2 (49.2 to 66)	303.0 (244.0 to 366.7)
		Escherichia coli	2770 (2440 to 3120)	3040 (2790 to 3290)	10.0 (1.6 to 19.1)	42.6 (39.2 to 46.9)	123 (109 to 138)	190.3 (152.7 to 230.6)
		Fungus	2660 (2310 to 3040)	2940 (2650 to 3250)	10.7 (1.5 to 20.8)	40.3 (35.7 to 45.2)	118 (103 to 134)	192.2 (152.2 to 233.3)
		Group B Streptococcus	2220 (1930 to 2550)	2840 (2570 to 3110)	28.3 (17.1 to 39.6)	24.2 (21.8 to 27)	81.8 (71.8 to 92.3)	238.7 (191.0 to 285.5)
		Haemophilus influenzae	7580 (6870 to 8340)	6890 (6320 to 7430)	-9.1 (-14.6 to -3.9)	44.5 (41.6 to 47.5)	71.9 (64.1 to 80.5)	61.7 (43.0 to 81.8)
		Influenza	9300 (8460 to 10100)	291 (32.7 to 1030)	-96.9 (-99.7 to -89.0)	107 (101 to 113)	8.97 (0.97 to 31)	-91.6 (-99.1 to -71.2)

		Klebsiella pneumoniae	5700 (5210 to 6230)	5970 (5510 to 6420)	4.7 (-0.6 to 9.8)	87.2 (82.7 to 92.1)	241 (213 to 267)	176.3 (144.4 to 208.6)
		Legionella spp.	5140 (4540 to 5760)	7430 (6820 to 8030)	44.9 (32.1 to 57.5)	54.1 (48.9 to 59.4)	207 (183 to 231)	282.4 (232.6 to 336.7)
		Mycoplasma spp.	7730 (6980 to 8510)	6910 (6340 to 7470)	-10.5 (-15.8 to -4.9)	31.4 (29.1 to 33.7)	74.1 (65.4 to 82.8)	136.3 (107.3 to 166.7)
		Polymicrobial	372 (244 to 563)	339 (208 to 548)	-9.4 (-20.5 to 1.9)	5.8 (3.79 to 8.79)	14 (8.4 to 22.6)	139.2 (103.3 to 179.0)
		Pseudomonas aeruginosa	5740 (5190 to 6360)	10000 (9260 to 10800)	75.0 (63.8 to 86.0)	61 (57.1 to 65)	281 (250 to 314)	361.9 (303.9 to 421.1)
		Respiratory syncytial virus	1800 (1600 to 2010)	77.2 (8.43 to 271)	-95.7 (-99.5 to -85.0)	9.42 (8.57 to 10.4)	1.08 (0.11 to 3.72)	-88.5 (-98.8 to -60.5)
		Staphylococcus aureus	7990 (7290 to 8680)	13100 (12100 to 14000)	63.9 (57.6 to 70.4)	174 (166 to 181)	751 (665 to 833)	332.7 (281.7 to 382.5)
		Streptococcus pneumoniae	37800 (34700 to 41000)	27000 (25000 to 29000)	-28.4 (-31.2 to -25.9)	342 (328 to 356)	645 (576 to 714)	89.0 (67.5 to 110.3)
		Other viruses	14800 (13500 to 16000)	20800 (19300 to 22300)	40.7 (35.0 to 46.3)	52.5 (50.1 to 55.1)	195 (174 to 216)	271.4 (229.6 to 314.4)
		Other bacterial pathogens	9970 (8800 to 11200)	7810 (6810 to 8840)	-21.6 (-26.4 to -16.9)	86 (77.8 to 94.1)	178 (152 to 205)	107.3 (81.7 to 132.4)
	70+ years	*Acinetobacter baumannii*	2590 (2210 to 3020)	2470 (2130 to 2830)	-4.6 (-12.8 to 3.3)	97.6 (84.9 to 112)	200 (168 to 234)	105.4 (80.9 to 130.0)
		Chlamydia spp.	8150 (7190 to 9240)	7450 (6770 to 8190)	-8.4 (-15.4 to -1.4)	76.3 (68.1 to 83.7)	150 (129 to 169)	97.2 (74.8 to 122.1)
		Enterobacter spp.	984 (823 to 1160)	2290 (2000 to 2560)	133.4 (111.4 to 158.6)	35.1 (30.1 to 40.9)	176 (148 to 201)	402.6 (335.8 to 477.8)
		Escherichia coli	3590 (3140 to 4140)	6020 (5530 to 6590)	68.2 (54.0 to 82.5)	133 (119 to 147)	479 (415 to 533)	262.1 (219.7 to 306.4)
		Fungus	1560 (1330 to 1830)	2640 (2350 to 2950)	69.3 (54.1 to 85.0)	56.9 (49.7 to 64.9)	207 (178 to 235)	264.5 (220.5 to 311.7)
		Group B Streptococcus	2140 (1880 to 2450)	4200 (3830 to 4590)	96.2 (78.4 to 113.9)	56.2 (49.7 to 62.9)	237 (202 to 265)	322.5 (269.9 to 375.4)
		Haemophilus influenzae	7200 (6450 to 8120)	10000 (9180 to 10900)	39.0 (30.0 to 48.0)	102 (93.2 to 109)	205 (177 to 227)	101.7 (80.9 to 124.8)
		Influenza	14900 (13500 to 16500)	711 (81.8 to 2500)	-95.2 (-99.5 to -83.5)	411 (382 to 438)	43.1 (4.6 to 148)	-89.5 (-98.9 to -64.0)
		Klebsiella pneumoniae	6620 (6020 to 7380)	10600 (9780 to 11500)	60.1 (51.0 to 69.4)	243 (225 to 259)	839 (726 to 929)	244.7 (209.6 to 280.6)
		Legionella spp.	3900 (3440 to 4440)	8620 (7920 to 9440)	121.6 (101.1 to 142.7)	98.6 (88.1 to 109)	470 (408 to 521)	377.0 (320.4 to 437.9)
		Mycoplasma spp.	6890 (6190 to 7790)	9420 (8680 to 10300)	36.9 (27.4 to 46.2)	67.3 (61.2 to 72.9)	198 (171 to 221)	194.7 (162.7 to 227.1)
		Polymicrobial	450 (293 to 702)	627 (367 to 1010)	38.6 (20.8 to 56.3)	16.8 (10.8 to 26.1)	50.5 (30.5 to 82.4)	198.3 (155.7 to 246.6)
		Pseudomonas aeruginosa	6300 (5710 to 7070)	16800 (15500 to 18300)	167.6 (149.2 to 186.0)	161 (148 to 173)	926 (804 to 1030)	476.2 (413.4 to 539.8)
		Respiratory syncytial virus	1780 (1560 to 2030)	116 (13.2 to 407)	-93.4 (-99.3 to -77.6)	22.4 (20.2 to 24.6)	3.21 (0.34 to 11.2)	-85.6 (-98.5 to -50.3)

		Staphylococcus aureus	10900 (9890 to 12100)	27300 (25300 to 29600)	150.7 (138.5 to 162.9)	569 (527 to 603)	3070 (2680 to 3390)	439.7 (388.3 to 493.8)
		Streptococcus pneumoniae	41000 (37300 to 45300)	44800 (41700 to 48700)	9.5 (4.2 to 14.5)	890 (829 to 939)	2100 (1820 to 2320)	135.7 (113.2 to 158.1)
		Other viruses	15800 (14400 to 17600)	34100 (31600 to 37000)	115.2 (104.5 to 126.2)	135 (125 to 143)	627 (544 to 694)	363.2 (319.8 to 409.1)
		Other bacterial pathogens	14100 (12300 to 16200)	16900 (14800 to 19100)	19.9 (12.0 to 28.4)	295 (263 to 325)	762 (640 to 881)	158.6 (131.0 to 187.9)
Romania	All Ages	*Acinetobacter baumannii*	6590 (5730 to 7510)	2590 (2270 to 2940)	-60.7 (-63.9 to -57.0)	160 (142 to 181)	126 (108 to 145)	-21.3 (-30.9 to -10.3)
		Chlamydia spp.	24100 (21900 to 26400)	9290 (8550 to 10100)	-61.5 (-64.4 to -58.2)	158 (146 to 169)	107 (94.9 to 119)	-32.2 (-39.8 to -24.2)
		Enterobacter spp.	2700 (2330 to 3110)	2290 (2050 to 2550)	-15.1 (-23.5 to -6.5)	67.9 (58.7 to 77.6)	103 (89.1 to 117)	51.3 (30.7 to 73.2)
		Escherichia coli	8540 (7670 to 9520)	5880 (5420 to 6420)	-31.1 (-37.0 to -25.1)	233 (213 to 253)	279 (249 to 309)	19.9 (6.3 to 33.7)
		Fungus	6130 (5330 to 6950)	3880 (3510 to 4290)	-36.7 (-41.9 to -30.8)	121 (107 to 135)	167 (146 to 190)	38.3 (20.8 to 58.4)
		Group B Streptococcus	7090 (6290 to 7920)	4820 (4470 to 5240)	-31.9 (-37.6 to -25.6)	133 (121 to 146)	153 (136 to 171)	15.2 (1.2 to 30.8)
		Haemophilus influenzae	32100 (29200 to 35300)	14900 (13900 to 15900)	-53.6 (-57.5 to -49.8)	348 (325 to 373)	166 (149 to 185)	-52.3 (-57.6 to -46.6)
		Influenza	40800 (37800 to 44000)	238 (31.5 to 840)	-99.4 (-99.9 to -97.9)	809 (765 to 849)	8.23 (1.07 to 29.5)	-99.0 (-99.9 to -96.4)
		Klebsiella pneumoniae	21000 (19400 to 22700)	11200 (10500 to 12100)	-46.6 (-51.0 to -42.7)	572 (542 to 602)	507 (453 to 562)	-11.3 (-20.7 to -1.5)
		Legionella spp.	12000 (10700 to 13300)	10800 (9930 to 11700)	-10.3 (-17.8 to -2.3)	175 (158 to 194)	332 (294 to 372)	90.5 (66.8 to 115.6)
		Mycoplasma spp.	37000 (34000 to 40500)	17500 (16300 to 18800)	-52.6 (-55.7 to -49.1)	212 (199 to 226)	174 (157 to 193)	-17.6 (-26.9 to -8.0)
		Polymicrobial	1220 (852 to 1770)	674 (428 to 1060)	-45.3 (-52.0 to -37.7)	41.2 (29.5 to 57)	33.6 (21.8 to 52.5)	-18.6 (-38.5 to 2.9)
		Pseudomonas aeruginosa	17300 (15900 to 18800)	16500 (15300 to 17800)	-4.7 (-11.5 to 2.7)	307 (289 to 328)	516 (460 to 571)	67.9 (49.1 to 88.4)
		Respiratory syncytial virus	27400 (23900 to 31900)	112 (14.8 to 379)	-99.6 (-99.9 to -98.6)	374 (350 to 396)	1.11 (0.14 to 3.93)	-99.7 (-100.0 to -98.9)
		Staphylococcus aureus	26300 (24200 to 28200)	24000 (22300 to 25900)	-8.7 (-15.2 to -2.1)	929 (880 to 975)	1590 (1420 to 1780)	70.8 (53.3 to 90.8)
		Streptococcus pneumoniae	196000 (181000 to 212000)	66700 (62500 to 71400)	-65.8 (-68.4 to -63.5)	3010 (2870 to 3130)	1610 (1450 to 1790)	-46.3 (-52.0 to -40.4)
		Other viruses	98800 (90300 to 110000)	49800 (46700 to 53300)	-49.5 (-54.0 to -45.4)	671 (637 to 705)	445 (400 to 491)	-33.7 (-40.7 to -26.5)
		Other bacterial pathogens	29200 (26000 to 32500)	16900 (14900 to 18900)	-42.2 (-46.4 to -37.3)	394 (355 to 432)	463 (396 to 524)	17.6 (4.5 to 32.4)
	Under 5	*Acinetobacter baumannii*	334 (239 to 455)	48.9 (32.6 to 71.9)	-85.4 (-88.2 to -82.2)	23.7 (16 to 34.9)	2.52 (1.42 to 4.34)	-89.5 (-92.2 to -86.6)
		Chlamydia spp.	4360 (3650 to 5340)	591 (512 to 687)	-86.4 (-88.7 to -84.0)	54.2 (48.6 to 60)	3.75 (3.23 to 4.28)	-93.1 (-94.1 to -91.9)

		Enterobacter spp.	430 (327 to 555)	116 (89 to 145)	-72.9 (-78.0 to -67.7)	21.3 (16.8 to 27.6)	2.83 (2.06 to 3.88)	-86.7 (-89.3 to -83.8)
		Escherichia coli	1510 (1260 to 1840)	365 (315 to 426)	-75.7 (-80.1 to -71.1)	74.4 (65.3 to 84.7)	8.31 (7.02 to 9.57)	-88.8 (-90.8 to -86.8)
		Fungus	297 (207 to 415)	58 (41.1 to 78.5)	-80.3 (-84.3 to -76.2)	12.3 (8.78 to 16.6)	0.9 (0.64 to 1.23)	-92.6 (-94.2 to -90.8)
		Group B Streptococcus	1300 (1070 to 1580)	317 (271 to 370)	-75.4 (-79.4 to -71.1)	50.4 (44 to 57.2)	6.24 (5.3 to 7.19)	-87.6 (-89.5 to -85.4)
		Haemophilus influenzae	11400 (9510 to 13600)	1840 (1590 to 2120)	-83.7 (-86.6 to -80.7)	194 (175 to 214)	8.4 (7.08 to 9.79)	-95.7 (-96.5 to -94.7)
		Influenza	9560 (8060 to 11600)	14.9 (1.92 to 50.7)	-99.8 (-100.0 to -99.5)	314 (290 to 339)	0.2 (0.03 to 0.72)	-99.9 (-100.0 to -99.8)
		Klebsiella pneumoniae	5640 (4780 to 6820)	779 (676 to 899)	-86.1 (-88.5 to -83.8)	250 (229 to 273)	15.3 (13.1 to 17.7)	-93.9 (-94.9 to -92.7)
		Legionella spp.	493 (352 to 687)	146 (107 to 203)	-70.2 (-74.7 to -65.1)	19.9 (14.9 to 26.1)	3.22 (2.42 to 4.11)	-83.7 (-86.6 to -80.3)
		Mycoplasma spp.	6520 (5460 to 7920)	1910 (1650 to 2210)	-70.6 (-75.7 to -65.3)	76.8 (69.5 to 85)	8.76 (7.36 to 10.2)	-88.6 (-90.6 to -86.3)
		Polymicrobial	218 (134 to 323)	59.4 (33.8 to 95.2)	-72.8 (-78.5 to -65.8)	19.3 (11.2 to 30.4)	3.8 (1.98 to 6.44)	-80.4 (-84.8 to -75.1)
		Pseudomonas aeruginosa	3040 (2560 to 3660)	1150 (1010 to 1320)	-61.8 (-68.4 to -55.0)	100 (90.2 to 110)	15.9 (13.5 to 18.2)	-84.1 (-86.7 to -81.1)
		Respiratory syncytial virus	21600 (18200 to 25800)	65.3 (8.35 to 220)	-99.7 (-100.0 to -98.9)	337 (315 to 359)	0.42 (0.05 to 1.49)	-99.9 (-100.0 to -99.5)
		Staphylococcus aureus	3900 (3310 to 4750)	1030 (897 to 1190)	-73.4 (-77.9 to -69.1)	244 (224 to 262)	26.5 (22.5 to 30.9)	-89.1 (-91.0 to -87.1)
		Streptococcus pneumoniae	63200 (53300 to 76500)	5790 (5040 to 6680)	-90.8 (-92.4 to -89.3)	1580 (1470 to 1680)	56.5 (47.6 to 66.7)	-96.4 (-97.0 to -95.7)
		Other viruses	45200 (38400 to 54800)	8160 (7130 to 9360)	-81.9 (-84.9 to -79.2)	450 (419 to 481)	32.9 (27.9 to 38.3)	-92.7 (-93.9 to -91.3)
		Other bacterial pathogens	1720 (1340 to 2170)	293 (236 to 363)	-82.8 (-85.8 to -79.5)	46.9 (38.3 to 57)	3.66 (2.76 to 4.8)	-92.2 (-93.7 to -90.4)
	5-14 years	*Acinetobacter baumannii*	782 (625 to 967)	104 (81.5 to 129)	-86.7 (-88.8 to -84.6)	3.54 (2.99 to 4.09)	0.38 (0.32 to 0.45)	-89.2 (-90.7 to -87.5)
		Chlamydia spp.	3380 (2730 to 4180)	518 (409 to 635)	-84.6 (-87.1 to -82.3)	3.8 (3.34 to 4.3)	0.47 (0.41 to 0.53)	-87.6 (-89.3 to -85.6)
		Enterobacter spp.	293 (230 to 367)	93 (72.7 to 117)	-68.1 (-73.3 to -62.3)	1.25 (1.06 to 1.47)	0.32 (0.27 to 0.37)	-74.2 (-78.1 to -70.0)
		Escherichia coli	779 (631 to 961)	193 (153 to 235)	-75.1 (-79.2 to -71.3)	3.46 (3.04 to 3.85)	0.69 (0.61 to 0.78)	-79.9 (-82.6 to -76.8)
		Fungus	940 (745 to 1160)	228 (181 to 282)	-75.7 (-79.4 to -71.8)	4.11 (3.54 to 4.76)	0.81 (0.7 to 0.92)	-80.3 (-83.1 to -77.3)
		Group B Streptococcus	965 (773 to 1200)	267 (211 to 327)	-72.3 (-76.6 to -67.9)	3.04 (2.64 to 3.47)	0.68 (0.59 to 0.78)	-77.6 (-80.6 to -74.1)
		Haemophilus influenzae	3830 (3160 to 4600)	863 (697 to 1050)	-77.4 (-80.8 to -73.9)	6.49 (5.84 to 7.08)	0.83 (0.74 to 0.93)	-87.2 (-88.9 to -85.3)
		Influenza	5480 (4500 to 6590)	13.2 (1.74 to 44.7)	-99.8 (-100.0 to -99.2)	18.2 (16.5 to 19.7)	0.04 (0 to 0.12)	-99.8 (-100.0 to -99.3)

		Klebsiella pneumoniae	2290 (1890 to 2760)	510 (412 to 625)	-77.7 (-81.1 to -74.4)	10.1 (9.09 to 11)	1.82 (1.62 to 2.02)	-82.0 (-84.2 to -79.3)
		Legionella spp.	1810 (1470 to 2200)	596 (480 to 725)	-66.9 (-72.1 to -61.2)	5.48 (4.83 to 6.2)	1.46 (1.29 to 1.65)	-73.2 (-76.8 to -69.2)
		Mycoplasma spp.	8560 (7040 to 10400)	1920 (1540 to 2340)	-77.5 (-81.0 to -74.1)	10 (9.02 to 11)	1.82 (1.62 to 2.02)	-81.8 (-84.3 to -79.2)
		Polymicrobial	138 (88.6 to 209)	27.7 (16.4 to 44.5)	-80.0 (-83.8 to -76.2)	0.62 (0.41 to 0.93)	0.1 (0.06 to 0.16)	-83.9 (-86.3 to -80.8)
		Pseudomonas aeruginosa	2010 (1640 to 2420)	730 (582 to 893)	-63.7 (-69.2 to -58.1)	6.17 (5.5 to 6.79)	1.81 (1.6 to 2.02)	-70.6 (-74.6 to -66.0)
		Respiratory syncytial virus	1390 (1130 to 1690)	4.38 (0.57 to 15)	-99.7 (-100.0 to -98.9)	2.1 (1.88 to 2.33)	0.01 (0 to 0.02)	-99.7 (-100.0 to -99.1)
		Staphylococcus aureus	3230 (2660 to 3900)	1090 (872 to 1320)	-66.3 (-71.5 to -61.3)	20.3 (18.3 to 21.9)	5.5 (4.91 to 6.13)	-72.8 (-76.3 to -68.8)
		Streptococcus pneumoniae	31300 (25900 to 37600)	5580 (4530 to 6790)	-82.1 (-84.7 to -79.6)	81.6 (74.3 to 88.2)	11.8 (10.5 to 13.1)	-85.6 (-87.4 to -83.4)
		Other viruses	13500 (11200 to 16200)	4160 (3380 to 5060)	-69.2 (-73.8 to -64.8)	13.9 (12.6 to 15)	3.45 (3.08 to 3.83)	-75.1 (-78.3 to -71.6)
		Other bacterial pathogens	3410 (2740 to 4190)	654 (509 to 811)	-80.8 (-83.8 to -77.9)	8.48 (7.48 to 9.5)	1.32 (1.14 to 1.51)	-84.5 (-86.5 to -82.1)
	15-49 years	*Acinetobacter baumannii*	833 (711 to 982)	210 (178 to 246)	-74.8 (-77.2 to -72.3)	15.3 (13.1 to 17.7)	6.9 (5.71 to 8.12)	-55.0 (-61.1 to -48.1)
		Chlamydia spp.	3600 (3200 to 4080)	1050 (928 to 1180)	-70.9 (-73.4 to -68.2)	16.5 (14.7 to 18.4)	8.55 (7.33 to 9.78)	-48.0 (-55.0 to -40.5)
		Enterobacter spp.	311 (265 to 370)	188 (162 to 215)	-39.5 (-46.3 to -32.6)	5.44 (4.67 to 6.36)	5.86 (4.93 to 6.88)	8.0 (-8.5 to 25.4)
		Escherichia coli	830 (734 to 941)	390 (347 to 437)	-52.9 (-57.4 to -48.5)	15 (13.4 to 16.6)	12.6 (10.9 to 14.2)	-15.8 (-27.2 to -4.0)
		Fungus	1000 (860 to 1160)	460 (405 to 526)	-53.9 (-58.4 to -49.1)	17.8 (15.6 to 20.3)	14.6 (12.5 to 16.8)	-17.8 (-29.7 to -5.6)
		Group B Streptococcus	1030 (900 to 1190)	539 (473 to 606)	-47.5 (-52.5 to -42.2)	13.2 (11.6 to 15)	12.3 (10.6 to 14.1)	-6.2 (-19.6 to 7.5)
		Haemophilus influenzae	4080 (3680 to 4500)	1740 (1560 to 1920)	-57.2 (-61.1 to -53.6)	28.1 (25.6 to 30.7)	15.1 (13.3 to 17)	-46.4 (-53.8 to -38.9)
		Influenza	5840 (5300 to 6390)	26.7 (3.4 to 95.1)	-99.5 (-99.9 to -98.4)	78.7 (72.8 to 84.8)	0.65 (0.09 to 2.29)	-99.2 (-99.9 to -97.1)
		Klebsiella pneumoniae	2440 (2210 to 2690)	1030 (923 to 1130)	-57.7 (-61.3 to -54.1)	43.8 (40.4 to 47.2)	33 (28.9 to 37.1)	-24.6 (-34.2 to -14.5)
		Legionella spp.	1920 (1680 to 2200)	1200 (1080 to 1340)	-37.3 (-43.4 to -30.8)	23.8 (21.1 to 26.6)	26.6 (23.3 to 29.8)	12.0 (-4.1 to 27.8)
		Mycoplasma spp.	9110 (8230 to 10100)	3870 (3470 to 4270)	-57.4 (-60.8 to -53.7)	43.5 (39.8 to 47.3)	33 (29 to 36.9)	-24.0 (-34.2 to -13.1)
		Polymicrobial	147 (93.6 to 221)	55.9 (33.4 to 88.4)	-62.1 (-67.0 to -57.0)	2.69 (1.77 to 4.02)	1.83 (1.11 to 2.93)	-32.4 (-43.1 to -20.5)
		Pseudomonas aeruginosa	2140 (1920 to 2380)	1470 (1320 to 1640)	-31.1 (-36.9 to -24.7)	26.7 (24.4 to 29.1)	32.8 (28.7 to 36.9)	23.0 (6.4 to 40.6)
		Respiratory syncytial virus	1480 (1310 to 1650)	8.87 (1.14 to 30.8)	-99.4 (-99.9 to -97.9)	9.09 (8.25 to 10)	0.1 (0.01 to 0.34)	-98.9 (-99.9 to -96.1)

		Staphylococcus aureus	3440 (3150 to 3760)	2190 (1970 to 2410)	-36.2 (-41.5 to -31.3)	87.8 (81.4 to 94)	99.9 (87.8 to 112)	13.9 (-1.3 to 29.7)
		Streptococcus pneumoniae	33300 (30600 to 36200)	11300 (10100 to 12400)	-66.1 (-68.9 to -63.4)	354 (330 to 377)	214 (189 to 239)	-39.5 (-47.5 to -31.2)
		Other viruses	14400 (13200 to 15700)	8410 (7570 to 9220)	-41.6 (-46.4 to -37.0)	60.2 (55.9 to 64.5)	62.7 (55.5 to 70.3)	4.2 (-9.4 to 18.2)
		Other bacterial pathogens	3630 (3190 to 4120)	1320 (1140 to 1510)	-63.6 (-66.7 to -60.3)	36.8 (32.9 to 40.9)	23.9 (20.3 to 27.4)	-34.9 (-43.9 to -25.4)
	50-69 years	*Acinetobacter baumannii*	2740 (2320 to 3190)	1000 (860 to 1150)	-63.4 (-67.1 to -59.1)	48.7 (42.3 to 56.4)	42.2 (34.9 to 50.2)	-13.2 (-27.9 to 3.7)
		Chlamydia spp.	7000 (6090 to 7910)	2910 (2610 to 3240)	-58.4 (-62.5 to -53.7)	31 (27.7 to 34.6)	30.4 (25.8 to 35.5)	-1.5 (-17.7 to 16.6)
		Enterobacter spp.	994 (841 to 1170)	864 (766 to 984)	-12.8 (-23.0 to -2.0)	16.8 (14.3 to 19.6)	34.5 (28.8 to 41.1)	106.6 (68.9 to 148.6)
		Escherichia coli	2920 (2550 to 3300)	1970 (1790 to 2190)	-32.4 (-39.2 to -25.0)	51.1 (45.3 to 57.2)	81.5 (69.5 to 94.7)	60.1 (33.3 to 89.8)
		Fungus	2810 (2390 to 3220)	1860 (1660 to 2110)	-33.4 (-40.4 to -25.8)	48.3 (42 to 54.9)	76 (63.5 to 90.4)	57.7 (31.6 to 88.0)
		Group B Streptococcus	2320 (2000 to 2670)	1740 (1570 to 1960)	-24.7 (-32.6 to -16.0)	28.8 (25.3 to 32.6)	51.2 (43.2 to 60.3)	78.5 (48.1 to 113.4)
		Haemophilus influenzae	7870 (6940 to 8700)	4950 (4510 to 5440)	-36.9 (-42.7 to -30.0)	52.4 (47.5 to 57.8)	54.8 (46.9 to 63.7)	4.8 (-12.1 to 24.1)
		Influenza	9690 (8620 to 10700)	64.3 (8.41 to 220)	-99.3 (-99.9 to -97.6)	126 (115 to 138)	2 (0.26 to 6.96)	-98.4 (-99.8 to -94.5)
		Klebsiella pneumoniae	6020 (5370 to 6620)	3790 (3460 to 4150)	-37.0 (-42.8 to -30.4)	105 (95.4 to 114)	156 (134 to 181)	49.3 (25.5 to 75.9)
		Legionella spp.	5210 (4500 to 5910)	4700 (4260 to 5190)	-9.5 (-18.7 to 0.9)	62.2 (54.8 to 69.8)	133 (113 to 155)	114.5 (78.8 to 154.8)
		Mycoplasma spp.	8050 (7100 to 8950)	4820 (4390 to 5320)	-40.0 (-45.7 to -33.7)	37.1 (33.5 to 41.3)	52.7 (45.2 to 61.1)	42.2 (18.8 to 67.7)
		Polymicrobial	402 (264 to 600)	221 (137 to 354)	-45.2 (-52.5 to -37.3)	7.11 (4.73 to 10.7)	9.26 (5.69 to 15.1)	29.8 (5.7 to 56.0)
		Pseudomonas aeruginosa	5850 (5190 to 6490)	5770 (5230 to 6330)	-1.3 (-10.3 to 9.7)	70.6 (64 to 78.1)	165 (141 to 191)	133.8 (94.9 to 175.1)
		Respiratory syncytial virus	1820 (1570 to 2060)	15.6 (2.04 to 54.1)	-99.1 (-99.9 to -96.9)	10.8 (9.6 to 12.1)	0.22 (0.03 to 0.78)	-97.9 (-99.7 to -92.8)
		Staphylococcus aureus	8270 (7330 to 9040)	7610 (6930 to 8330)	-7.7 (-15.8 to 1.4)	204 (187 to 221)	445 (382 to 516)	118.6 (83.1 to 156.4)
		Streptococcus pneumoniae	39500 (35500 to 43000)	19500 (17900 to 21300)	-50.5 (-54.7 to -45.4)	406 (370 to 438)	475 (409 to 550)	17.3 (-1.5 to 38.1)
		Other viruses	15000 (13400 to 16400)	13000 (11800 to 14200)	-13.2 (-20.8 to -4.5)	60.4 (55.3 to 65.7)	124 (106 to 143)	105.7 (72.1 to 141.0)
		Other bacterial pathogens	10600 (9190 to 11900)	5540 (4850 to 6280)	-47.5 (-52.4 to -41.7)	103 (91.4 to 116)	129 (107 to 150)	24.5 (3.1 to 48.0)
	70+ years	*Acinetobacter baumannii*	1900 (1600 to 2240)	1220 (1040 to 1420)	-35.6 (-42.5 to -28.2)	68.9 (59.2 to 80.4)	74 (62.4 to 86.5)	7.5 (-7.0 to 23.9)
		Chlamydia spp.	5800 (5010 to 6630)	4230 (3720 to 4740)	-26.9 (-34.6 to -19.3)	52.2 (45.9 to 58.5)	63.6 (55.5 to 72)	22.1 (5.4 to 39.7)

		Enterobacter spp.	673 (562 to 810)	1030 (885 to 1180)	53.2 (34.7 to 72.3)	23.1 (19.4 to 26.9)	59 (50.2 to 68.3)	155.9 (120.4 to 193.7)
		Escherichia coli	2500 (2130 to 2880)	2960 (2630 to 3330)	18.7 (5.1 to 31.9)	88.7 (78.6 to 99.5)	176 (154 to 198)	98.3 (72.7 to 127.7)
		Fungus	1090 (907 to 1290)	1270 (1100 to 1460)	16.9 (3.9 to 31.0)	38.1 (32.9 to 43.9)	74.2 (64.4 to 85.7)	95.3 (69.9 to 126.5)
		Group B Streptococcus	1480 (1270 to 1730)	1950 (1730 to 2190)	32.4 (16.7 to 47.7)	37.3 (32.7 to 42.4)	82.3 (71.9 to 94)	121.1 (91.9 to 154.5)
		Haemophilus influenzae	4930 (4320 to 5590)	5450 (4880 to 6090)	10.8 (-1.3 to 22.2)	66.8 (60.1 to 73.4)	86.6 (76.4 to 97.6)	29.9 (13.6 to 48.0)
		Influenza	10200 (9030 to 11500)	119 (15.6 to 409)	-98.8 (-99.8 to -95.9)	272 (247 to 299)	5.34 (0.69 to 19.3)	-98.0 (-99.7 to -92.9)
		Klebsiella pneumoniae	4620 (4080 to 5210)	5100 (4580 to 5690)	10.8 (-1.2 to 21.9)	163 (148 to 179)	301 (266 to 340)	85.0 (61.9 to 110.1)
		Legionella spp.	2600 (2230 to 3000)	4130 (3670 to 4660)	59.1 (40.3 to 77.2)	63.3 (55.7 to 71.4)	168 (146 to 190)	165.7 (131.3 to 205.9)
		Mycoplasma spp.	4730 (4130 to 5380)	4980 (4470 to 5570)	5.5 (-6.1 to 16.0)	44.4 (39.5 to 49.2)	78.1 (68.8 to 87.6)	76.2 (53.6 to 102.3)
		Polymicrobial	320 (204 to 497)	310 (183 to 507)	-3.8 (-17.0 to 10.8)	11.5 (7.48 to 17.7)	18.6 (11.2 to 30.4)	60.8 (35.1 to 90.1)
		Pseudomonas aeruginosa	4240 (3720 to 4820)	7330 (6570 to 8160)	73.4 (54.8 to 91.6)	104 (93.3 to 115)	300 (266 to 338)	189.6 (153.9 to 232.7)
		Respiratory syncytial virus	1180 (1010 to 1360)	17.8 (2.33 to 60.7)	-98.5 (-99.8 to -94.6)	14.3 (12.7 to 16.2)	0.36 (0.05 to 1.32)	-97.4 (-99.7 to -90.7)
		Staphylococcus aureus	7440 (6520 to 8370)	12000 (10800 to 13400)	62.1 (45.9 to 79.5)	373 (339 to 407)	1010 (893 to 1140)	170.8 (137.2 to 208.2)
		Streptococcus pneumoniae	28300 (25000 to 31700)	24600 (22000 to 27400)	-13.0 (-21.9 to -4.3)	590 (540 to 638)	857 (759 to 966)	45.4 (27.7 to 64.9)
		Other viruses	10600 (9280 to 11900)	16100 (14400 to 18000)	52.6 (36.8 to 67.2)	87 (79.1 to 94.7)	221 (196 to 250)	154.8 (124.2 to 189.4)
		Other bacterial pathogens	9830 (8490 to 11400)	9050 (7820 to 10400)	-7.8 (-17.1 to 1.8)	198 (175 to 221)	305 (259 to 350)	54.3 (34.4 to 77.7)
Serbia	All Ages	*Acinetobacter baumannii*	1220 (1050 to 1390)	790 (686 to 903)	-35.0 (-41.5 to -27.6)	26.3 (21.8 to 32.3)	23.2 (18.3 to 29)	-11.0 (-32.6 to 14.3)
		Chlamydia spp.	4590 (4160 to 5040)	2430 (2230 to 2660)	-47.0 (-51.8 to -41.4)	23 (19.9 to 27.3)	16.9 (13.8 to 20.7)	-26.2 (-42.8 to -7.0)
		Enterobacter spp.	540 (468 to 614)	667 (594 to 750)	23.8 (10.9 to 39.1)	10.7 (8.83 to 13)	18 (14.4 to 22.6)	68.8 (28.7 to 114.0)
		Escherichia coli	1680 (1500 to 1860)	1630 (1490 to 1790)	-2.8 (-12.3 to 7.7)	36.4 (31 to 42.9)	47.8 (38.9 to 58.6)	32.2 (2.4 to 67.9)
		Fungus	1190 (1040 to 1350)	1080 (977 to 1210)	-8.5 (-17.4 to 1.4)	19.2 (16.1 to 23)	26.2 (21 to 31.9)	37.2 (5.0 to 74.7)
		Group B Streptococcus	1400 (1250 to 1560)	1370 (1260 to 1500)	-1.9 (-11.4 to 8.9)	20.1 (17.2 to 23.9)	25.7 (21 to 31.6)	28.8 (-2.1 to 61.8)
		Haemophilus influenzae	6320 (5820 to 6950)	3690 (3420 to 4010)	-41.5 (-46.7 to -36.1)	43.2 (37.2 to 51.4)	24.3 (19.8 to 29.6)	-43.4 (-56.3 to -28.1)
		Influenza	8060 (7440 to 8730)	123 (47.2 to 262)	-98.5 (-99.4 to -96.7)	116 (101 to 135)	2.51 (0.9 to 5.47)	-97.8 (-99.2 to -95.2)

		Klebsiella pneumoniae	4080 (3780 to 4420)	3230 (2980 to 3510)	-20.6 (-27.8 to -13.4)	78 (68 to 91)	89 (72.3 to 110)	14.7 (-10.5 to 43.6)
		Legionella spp.	2470 (2200 to 2760)	3000 (2740 to 3280)	22.1 (9.7 to 35.9)	30.6 (25.9 to 36.3)	53.6 (44 to 65.9)	76.2 (34.1 to 124.7)
		Mycoplasma spp.	7230 (6590 to 7970)	4560 (4240 to 4910)	-36.9 (-42.0 to -32.0)	28.3 (24.8 to 32.6)	25.1 (20.6 to 30.4)	-11.2 (-30.8 to 10.5)
		Polymicrobial	243 (166 to 349)	184 (113 to 294)	-24.9 (-37.7 to -11.5)	6.87 (4.66 to 10.1)	5.49 (3.28 to 8.91)	-19.5 (-46.8 to 12.6)
		Pseudomonas aeruginosa	3520 (3220 to 3840)	4940 (4550 to 5360)	40.5 (27.8 to 54.1)	48.3 (41.7 to 56.3)	93.9 (76.9 to 116)	95.4 (50.7 to 146.5)
		Respiratory syncytial virus	5550 (4980 to 6280)	49.9 (19.2 to 108)	-99.1 (-99.7 to -98.0)	36.2 (29.2 to 49.2)	0.25 (0.09 to 0.54)	-99.3 (-99.8 to -98.4)
		Staphylococcus aureus	5250 (4850 to 5670)	7160 (6580 to 7790)	36.4 (25.2 to 49.2)	148 (128 to 174)	292 (239 to 359)	98.0 (53.7 to 148.7)
		Streptococcus pneumoniae	38000 (35200 to 41100)	18200 (16900 to 19600)	-52.1 (-55.5 to -48.4)	361 (316 to 421)	255 (209 to 311)	-29.2 (-44.7 to -11.9)
		Other viruses	19800 (18200 to 21700)	13800 (12800 to 14800)	-30.5 (-36.1 to -24.8)	74.1 (63.8 to 89.3)	70.6 (58.4 to 86.4)	-4.1 (-26.5 to 21.0)
		Other bacterial pathogens	5700 (5080 to 6340)	4630 (4040 to 5280)	-18.7 (-26.5 to -10.1)	68.2 (57.7 to 82)	79 (62.1 to 100)	16.5 (-11.6 to 48.1)
	Under 5	*Acinetobacter baumannii*	68.2 (47.2 to 95.2)	7.22 (5.08 to 10.2)	-89.4 (-90.9 to -87.7)	3.05 (1.63 to 5.35)	0.17 (0.09 to 0.31)	-94.4 (-97.0 to -91.6)
		Chlamydia spp.	824 (700 to 969)	102 (83.8 to 124)	-87.6 (-89.5 to -85.5)	4.93 (3.75 to 7.14)	0.2 (0.14 to 0.26)	-95.9 (-97.7 to -94.2)
		Enterobacter spp.	86.3 (66.6 to 111)	21.8 (16.3 to 28.9)	-74.6 (-78.8 to -70.2)	2.15 (1.41 to 3.38)	0.16 (0.1 to 0.24)	-92.5 (-95.9 to -89.1)
		Escherichia coli	294 (246 to 353)	64.1 (52 to 78)	-78.1 (-81.4 to -74.3)	6.96 (5.21 to 10.2)	0.42 (0.31 to 0.55)	-93.8 (-96.6 to -91.3)
		Fungus	55.7 (38.6 to 78.3)	11.5 (7.99 to 15.9)	-79.2 (-82.8 to -75.3)	0.87 (0.56 to 1.29)	0.04 (0.02 to 0.05)	-95.7 (-97.7 to -93.3)
		Group B Streptococcus	261 (216 to 316)	57.3 (46.3 to 71.3)	-78.0 (-81.3 to -74.3)	5.42 (4.13 to 8.15)	0.36 (0.26 to 0.47)	-93.3 (-96.3 to -90.4)
		Haemophilus influenzae	2210 (1890 to 2600)	321 (263 to 392)	-85.4 (-87.6 to -83.2)	15.9 (12.1 to 22.5)	0.31 (0.22 to 0.41)	-98.0 (-98.9 to -97.0)
		Influenza	1840 (1590 to 2140)	5.59 (2.14 to 12.2)	-99.7 (-99.9 to -99.3)	24.7 (18.8 to 34.8)	0.02 (0.01 to 0.04)	-99.9 (-100.0 to -99.8)
		Klebsiella pneumoniae	1070 (919 to 1250)	155 (127 to 190)	-85.5 (-87.7 to -83.2)	20 (15.1 to 28.6)	0.75 (0.54 to 0.99)	-96.1 (-97.9 to -94.4)
		Legionella spp.	105 (74.8 to 143)	24.6 (16.8 to 34.5)	-76.5 (-80.3 to -72.4)	2.37 (1.59 to 3.7)	0.19 (0.13 to 0.27)	-91.6 (-95.5 to -87.7)
		Mycoplasma spp.	1260 (1070 to 1500)	320 (262 to 392)	-74.6 (-78.5 to -70.4)	6.19 (4.74 to 8.86)	0.34 (0.24 to 0.45)	-94.4 (-97.0 to -91.9)
		Polymicrobial	50.6 (30.6 to 79.9)	7.32 (4.38 to 11.6)	-85.5 (-87.6 to -83.1)	2.97 (1.54 to 5.46)	0.27 (0.13 to 0.48)	-90.8 (-95.2 to -86.2)
		Pseudomonas aeruginosa	611 (521 to 720)	219 (180 to 268)	-64.0 (-69.8 to -58.2)	9.23 (7.1 to 13.3)	0.76 (0.55 to 1.01)	-91.5 (-95.4 to -87.6)
		Respiratory syncytial virus	4350 (3790 to 5050)	23.8 (9.05 to 52)	-99.5 (-99.8 to -98.8)	29.8 (23 to 42.6)	0.03 (0.01 to 0.08)	-99.9 (-100.0 to -99.7)

		Staphylococcus aureus	758 (653 to 880)	210 (173 to 258)	-72.2 (-76.1 to -68.3)	19.6 (14.9 to 27.8)	1.24 (0.88 to 1.65)	-93.5 (-96.5 to -90.7)
		Streptococcus pneumoniae	12000 (10500 to 14000)	1260 (1030 to 1540)	-89.5 (-91.0 to -88.1)	115 (87.3 to 163)	2.67 (1.87 to 3.56)	-97.6 (-98.7 to -96.5)
		Other viruses	8960 (7780 to 10400)	1690 (1390 to 2070)	-81.2 (-83.7 to -78.6)	35.1 (26.7 to 49.2)	1.51 (1.07 to 2)	-95.6 (-97.6 to -93.5)
		Other bacterial pathogens	326 (253 to 406)	52 (39.8 to 67.4)	-84.0 (-86.5 to -81.3)	3.97 (2.81 to 5.95)	0.17 (0.11 to 0.26)	-95.5 (-97.6 to -93.4)
	5-14 years	*Acinetobacter baumannii*	142 (108 to 184)	35.7 (26.7 to 47)	-74.9 (-77.7 to -71.4)	0.44 (0.31 to 0.6)	0.03 (0.02 to 0.04)	-92.9 (-95.6 to -88.9)
		Chlamydia spp.	639 (485 to 820)	155 (117 to 201)	-75.7 (-78.5 to -72.8)	0.49 (0.35 to 0.65)	0.03 (0.02 to 0.05)	-93.1 (-95.8 to -89.3)
		Enterobacter spp.	57.9 (43.2 to 75.7)	30.9 (22.9 to 41)	-46.5 (-53.6 to -38.9)	0.17 (0.12 to 0.23)	0.03 (0.02 to 0.04)	-84.8 (-90.7 to -76.7)
		Escherichia coli	151 (115 to 194)	60.7 (46.4 to 78.9)	-59.8 (-64.7 to -54.5)	0.46 (0.34 to 0.61)	0.05 (0.04 to 0.07)	-88.6 (-92.9 to -82.4)
		Fungus	182 (138 to 234)	73.6 (55.6 to 95.8)	-59.6 (-64.5 to -54.0)	0.55 (0.4 to 0.73)	0.06 (0.04 to 0.09)	-88.5 (-92.9 to -82.4)
		Group B Streptococcus	189 (144 to 242)	87.8 (66.1 to 114)	-53.5 (-59.0 to -47.2)	0.41 (0.3 to 0.54)	0.05 (0.04 to 0.07)	-86.8 (-91.9 to -79.5)
		Haemophilus influenzae	759 (590 to 968)	250 (190 to 323)	-67.1 (-70.6 to -63.1)	0.88 (0.64 to 1.17)	0.06 (0.04 to 0.08)	-93.5 (-96.0 to -90.0)
		Influenza	1080 (840 to 1370)	7.63 (2.59 to 17.2)	-99.3 (-99.7 to -98.4)	2.45 (1.8 to 3.23)	0 (0 to 0.01)	-99.8 (-99.9 to -99.6)
		Klebsiella pneumoniae	444 (346 to 567)	165 (127 to 213)	-62.9 (-66.8 to -58.4)	1.34 (0.99 to 1.77)	0.14 (0.1 to 0.19)	-89.5 (-93.6 to -83.7)
		Legionella spp.	368 (283 to 476)	190 (146 to 244)	-48.4 (-54.6 to -40.9)	0.77 (0.56 to 1.03)	0.11 (0.08 to 0.15)	-85.3 (-91.0 to -77.4)
		Mycoplasma spp.	1690 (1310 to 2130)	597 (458 to 768)	-64.6 (-68.3 to -60.4)	1.36 (1 to 1.8)	0.13 (0.09 to 0.18)	-90.0 (-93.8 to -84.5)
		Polymicrobial	26 (16.3 to 39.7)	8.79 (5.11 to 14.3)	-66.4 (-71.3 to -61.1)	0.08 (0.05 to 0.13)	0.01 (0 to 0.01)	-90.5 (-94.1 to -85.1)
		Pseudomonas aeruginosa	407 (314 to 517)	252 (194 to 325)	-38.1 (-44.8 to -30.5)	0.86 (0.62 to 1.13)	0.15 (0.1 to 0.2)	-82.4 (-89.3 to -72.8)
		Respiratory syncytial virus	284 (221 to 362)	2.79 (0.94 to 6.29)	-99.0 (-99.6 to -97.9)	0.29 (0.21 to 0.39)	0 (0 to 0)	-99.7 (-99.9 to -99.4)
		Staphylococcus aureus	642 (501 to 811)	363 (281 to 467)	-43.4 (-49.1 to -37.2)	2.76 (2.03 to 3.59)	0.43 (0.3 to 0.59)	-83.9 (-90.1 to -75.2)
		Streptococcus pneumoniae	6130 (4800 to 7710)	1730 (1340 to 2220)	-71.8 (-74.7 to -68.8)	11 (8.07 to 14.4)	0.86 (0.6 to 1.17)	-92.0 (-95.1 to -87.7)
		Other viruses	2760 (2160 to 3470)	1340 (1040 to 1740)	-51.3 (-56.2 to -45.9)	1.94 (1.42 to 2.55)	0.26 (0.18 to 0.36)	-86.2 (-91.5 to -78.7)
		Other bacterial pathogens	658 (509 to 843)	199 (149 to 258)	-69.8 (-73.1 to -66.0)	1.12 (0.81 to 1.51)	0.09 (0.06 to 0.13)	-91.4 (-94.7 to -86.6)
	15-49 years	*Acinetobacter baumannii*	145 (120 to 175)	56.9 (47.2 to 67.2)	-60.7 (-64.4 to -57.0)	1.68 (1.32 to 2.16)	0.61 (0.45 to 0.8)	-63.5 (-74.6 to -50.7)
		Chlamydia spp.	651 (558 to 757)	247 (215 to 281)	-62.0 (-65.3 to -58.4)	1.88 (1.5 to 2.33)	0.65 (0.49 to 0.85)	-64.8 (-75.7 to -51.9)

		Enterobacter spp.	59 (49 to 70.5)	49.2 (42 to 57.7)	-16.3 (-25.5 to -6.9)	0.65 (0.5 to 0.83)	0.5 (0.37 to 0.66)	-22.4 (-46.7 to 6.2)
		Escherichia coli	154 (132 to 179)	96.8 (84.5 to 110)	-37.0 (-42.8 to -30.8)	1.75 (1.4 to 2.19)	1.01 (0.78 to 1.32)	-41.6 (-59.3 to -20.9)
		Fungus	186 (155 to 219)	117 (100 to 135)	-36.7 (-42.7 to -30.2)	2.08 (1.66 to 2.63)	1.21 (0.91 to 1.58)	-41.3 (-59.9 to -20.3)
		Group B Streptococcus	193 (164 to 228)	140 (122 to 162)	-27.2 (-34.0 to -19.7)	1.56 (1.23 to 1.97)	1.04 (0.78 to 1.35)	-32.5 (-53.7 to -6.8)
		Haemophilus influenzae	773 (668 to 898)	398 (351 to 449)	-48.5 (-52.3 to -44.3)	3.36 (2.67 to 4.18)	1.11 (0.84 to 1.44)	-66.5 (-76.6 to -54.2)
		Influenza	1100 (967 to 1260)	12.1 (4.59 to 26.1)	-98.9 (-99.6 to -97.6)	9.36 (7.52 to 11.5)	0.09 (0.03 to 0.19)	-99.0 (-99.7 to -97.8)
		Klebsiella pneumoniae	452 (397 to 516)	263 (231 to 296)	-41.9 (-46.0 to -37.1)	5.12 (4.12 to 6.35)	2.73 (2.09 to 3.53)	-46.1 (-62.4 to -26.1)
		Legionella spp.	375 (323 to 435)	302 (267 to 342)	-19.2 (-26.9 to -10.8)	2.92 (2.3 to 3.66)	2.16 (1.64 to 2.79)	-25.1 (-48.6 to 2.3)
		Mycoplasma spp.	1720 (1500 to 1970)	951 (842 to 1070)	-44.6 (-48.9 to -39.8)	5.17 (4.15 to 6.39)	2.62 (2 to 3.42)	-48.7 (-64.2 to -29.5)
		Polymicrobial	26.5 (16.8 to 40.3)	14 (8.34 to 22.7)	-47.3 (-53.9 to -40.2)	0.31 (0.19 to 0.47)	0.15 (0.09 to 0.24)	-51.2 (-66.2 to -33.3)
		Pseudomonas aeruginosa	415 (360 to 477)	401 (354 to 453)	-3.1 (-10.7 to 5.4)	3.26 (2.62 to 4.05)	2.89 (2.22 to 3.75)	-10.2 (-37.5 to 22.1)
		Respiratory syncytial virus	290 (252 to 333)	4.43 (1.65 to 9.54)	-98.5 (-99.4 to -96.7)	1.12 (0.9 to 1.39)	0.02 (0.01 to 0.03)	-98.6 (-99.5 to -97.0)
		Staphylococcus aureus	654 (574 to 745)	578 (514 to 649)	-11.4 (-17.5 to -5.0)	10.5 (8.45 to 13.1)	8.53 (6.51 to 11)	-17.9 (-42.7 to 12.4)
		Streptococcus pneumoniae	6250 (5490 to 7090)	2760 (2450 to 3090)	-55.8 (-58.8 to -52.6)	41.8 (33.7 to 51.4)	16.9 (13 to 22)	-59.1 (-71.3 to -44.3)
		Other viruses	2810 (2480 to 3190)	2140 (1910 to 2400)	-23.8 (-29.0 to -18.3)	7.4 (5.96 to 9.14)	5.17 (3.96 to 6.69)	-29.4 (-50.3 to -3.8)
		Other bacterial pathogens	671 (568 to 776)	317 (270 to 370)	-52.7 (-56.5 to -48.6)	4.28 (3.39 to 5.35)	1.86 (1.35 to 2.43)	-56.1 (-69.3 to -40.1)
	50-69 years	*Acinetobacter baumannii*	493 (416 to 581)	273 (232 to 316)	-44.6 (-51.3 to -37.2)	6.98 (5.42 to 8.97)	5.03 (3.69 to 6.6)	-26.9 (-50.0 to 2.8)
		Chlamydia spp.	1310 (1140 to 1490)	682 (603 to 766)	-47.9 (-53.8 to -41.0)	4.6 (3.61 to 5.78)	3.12 (2.35 to 4.02)	-31.2 (-52.9 to -2.4)
		Enterobacter spp.	195 (164 to 228)	227 (198 to 259)	16.6 (2.4 to 34.2)	2.62 (2.02 to 3.36)	3.96 (2.92 to 5.15)	53.9 (3.4 to 115.2)
		Escherichia coli	562 (491 to 639)	487 (431 to 546)	-13.1 (-23.1 to -1.4)	7.8 (6.17 to 9.75)	8.82 (6.64 to 11.3)	14.7 (-21.5 to 60.9)
		Fungus	539 (463 to 625)	476 (416 to 540)	-11.6 (-21.7 to 1.0)	7.37 (5.73 to 9.4)	8.49 (6.26 to 11.2)	16.8 (-20.0 to 63.1)
		Group B Streptococcus	450 (388 to 519)	452 (397 to 511)	0.6 (-11.6 to 14.6)	4.44 (3.45 to 5.67)	5.8 (4.31 to 7.39)	32.9 (-10.1 to 87.5)
		Haemophilus influenzae	1540 (1350 to 1740)	1140 (1010 to 1280)	-25.8 (-34.0 to -16.3)	8.18 (6.49 to 10.2)	5.54 (4.17 to 7.11)	-31.2 (-53.0 to -5.0)
		Influenza	1890 (1680 to 2120)	29.2 (11.4 to 62.6)	-98.5 (-99.4 to -96.7)	19.6 (15.5 to 24.2)	0.38 (0.13 to 0.83)	-98.0 (-99.4 to -95.5)

		Klebsiella pneumoniae	1160 (1030 to 1300)	986 (878 to 1090)	-14.7 (-23.9 to -3.9)	16 (12.8 to 19.9)	17.7 (13.4 to 22.8)	12.7 (-22.7 to 58.1)
		Legionella spp.	1050 (913 to 1200)	1180 (1050 to 1320)	12.7 (-0.9 to 28.8)	9.99 (7.75 to 12.6)	14.6 (11.1 to 18.8)	48.8 (1.5 to 110.1)
		Mycoplasma spp.	1570 (1400 to 1770)	1170 (1030 to 1300)	-25.4 (-33.8 to -16.0)	5.76 (4.53 to 7.11)	5.6 (4.21 to 7.2)	-1.5 (-33.4 to 37.2)
		Polymicrobial	75.2 (48.5 to 115)	55.7 (34 to 89.4)	-26.3 (-37.1 to -15.0)	1.06 (0.65 to 1.62)	1.02 (0.57 to 1.7)	-2.6 (-34.5 to 38.6)
		Pseudomonas aeruginosa	1170 (1040 to 1330)	1570 (1400 to 1750)	33.7 (18.9 to 50.7)	11.2 (8.95 to 14)	19.6 (14.8 to 25.2)	76.5 (20.3 to 142.9)
		Respiratory syncytial virus	369 (319 to 420)	7.76 (2.98 to 16.6)	-97.9 (-99.2 to -95.4)	1.74 (1.34 to 2.2)	0.05 (0.02 to 0.1)	-97.3 (-99.1 to -93.9)
		Staphylococcus aureus	1630 (1450 to 1810)	2010 (1800 to 2230)	23.9 (11.2 to 39.6)	31.9 (25.5 to 39.3)	51.5 (39 to 66)	63.6 (12.1 to 127.8)
		Streptococcus pneumoniae	7680 (6840 to 8540)	4820 (4300 to 5330)	-37.1 (-43.4 to -29.6)	62.7 (50 to 77.1)	51.3 (39 to 65.4)	-17.0 (-43.2 to 15.2)
		Other viruses	3020 (2690 to 3370)	3360 (3010 to 3730)	11.4 (0.2 to 25.5)	9.7 (7.75 to 11.9)	14.1 (10.7 to 18.1)	47.1 (0.6 to 105.7)
		Other bacterial pathogens	2020 (1760 to 2290)	1330 (1140 to 1540)	-34.1 (-41.6 to -25.3)	15.7 (12.4 to 19.9)	13.5 (9.92 to 17.7)	-12.8 (-40.1 to 23.0)
	70+ years	*Acinetobacter baumannii*	368 (308 to 432)	417 (355 to 488)	13.9 (-0.3 to 30.7)	14.1 (10.9 to 18)	17.4 (13.5 to 22.2)	24.6 (-8.9 to 67.4)
		Chlamydia spp.	1170 (1010 to 1340)	1240 (1100 to 1400)	7.1 (-5.1 to 22.0)	11.1 (8.86 to 13.9)	12.9 (10.2 to 16.1)	17.2 (-13.2 to 54.3)
		Enterobacter spp.	142 (119 to 169)	338 (291 to 389)	139.6 (107.4 to 178.1)	5.16 (4.03 to 6.57)	13.4 (10.4 to 17)	162.2 (92.8 to 244.6)
		Escherichia coli	515 (442 to 593)	917 (819 to 1040)	78.6 (56.9 to 105.7)	19.4 (15.6 to 24.5)	37.5 (29.6 to 46.9)	95.5 (43.8 to 158.3)
		Fungus	224 (190 to 262)	406 (356 to 470)	81.8 (59.5 to 108.1)	8.32 (6.56 to 10.5)	16.4 (12.8 to 20.5)	98.9 (47.3 to 162.5)
		Group B Streptococcus	308 (265 to 356)	635 (566 to 721)	106.7 (80.6 to 137.2)	8.24 (6.57 to 10.4)	18.4 (14.7 to 23.3)	126.3 (66.4 to 201.4)
		Haemophilus influenzae	1040 (902 to 1180)	1580 (1420 to 1780)	52.6 (34.7 to 73.6)	14.9 (12.1 to 18.6)	17.3 (13.7 to 21.5)	17.1 (-14.4 to 56.7)
		Influenza	2150 (1890 to 2420)	68.1 (26.1 to 146)	-96.8 (-98.8 to -93.0)	60.3 (49.1 to 74.9)	2.02 (0.72 to 4.52)	-96.6 (-98.8 to -92.4)
		Klebsiella pneumoniae	951 (839 to 1070)	1670 (1490 to 1870)	75.4 (56.6 to 98.0)	35.6 (29.1 to 44.2)	67.6 (53.9 to 84.3)	91.9 (43.2 to 150.3)
		Legionella spp.	565 (482 to 659)	1300 (1160 to 1470)	131.6 (101.6 to 165.1)	14.5 (11.5 to 18.2)	36.5 (29.2 to 45.5)	153.5 (86.0 to 236.6)
		Mycoplasma spp.	993 (869 to 1130)	1520 (1360 to 1710)	53.3 (35.1 to 75.2)	9.87 (7.93 to 12.3)	16.4 (12.9 to 20.7)	67.7 (23.7 to 124.5)
		Polymicrobial	64.3 (40.9 to 98.7)	97.9 (57.6 to 160)	51.6 (27.6 to 74.7)	2.45 (1.5 to 3.85)	4.04 (2.28 to 6.74)	65.9 (21.8 to 121.7)
		Pseudomonas aeruginosa	912 (800 to 1040)	2500 (2250 to 2810)	174.7 (142.2 to 214.3)	23.7 (19.2 to 29.3)	70.5 (56.1 to 88.2)	200.7 (120.3 to 300.3)
		Respiratory syncytial virus	258 (222 to 294)	11.1 (4.32 to 24)	-95.7 (-98.3 to -90.6)	3.3 (2.67 to 4.14)	0.15 (0.05 to 0.33)	-95.4 (-98.4 to -89.8)

		Staphylococcus aureus	1570 (1390 to 1770)	4000 (3600 to 4490)	154.7 (126.6 to 188.7)	83.6 (67.8 to 103)	231 (185 to 286)	178.8 (106.8 to 268.5)
		Streptococcus pneumoniae	5900 (5230 to 6590)	7610 (6880 to 8510)	29.3 (15.5 to 45.5)	130 (106 to 162)	183 (146 to 227)	41.5 (5.1 to 87.1)
		Other viruses	2300 (2030 to 2580)	5250 (4740 to 5870)	129.0 (105.5 to 158.9)	20 (16.2 to 24.7)	49.6 (39.6 to 61.9)	150.7 (84.1 to 233.5)
		Other bacterial pathogens	2020 (1730 to 2310)	2730 (2340 to 3170)	35.5 (19.7 to 53.5)	43.1 (34.2 to 53.6)	63.4 (48.8 to 81.1)	48.5 (9.8 to 97.3)
Slovakia	All Ages	*Acinetobacter baumannii*	1130 (978 to 1300)	411 (360 to 468)	-63.7 (-67.5 to -59.4)	47.7 (38.4 to 56.3)	17.1 (13.9 to 20.8)	-63.9 (-71.8 to -54.6)
		Chlamydia spp.	4500 (4110 to 4960)	1920 (1760 to 2110)	-57.3 (-61.3 to -52.9)	43.4 (36.7 to 49.7)	19.2 (15.7 to 22.8)	-55.6 (-64.9 to -44.6)
		Enterobacter spp.	632 (551 to 723)	742 (670 to 823)	17.7 (4.5 to 31.0)	24.1 (19.6 to 28.3)	28.4 (23.3 to 34.4)	18.7 (-6.6 to 49.4)
		Escherichia coli	1870 (1700 to 2080)	1880 (1730 to 2060)	0.7 (-8.4 to 11.4)	77.4 (64.4 to 89)	78 (64.6 to 92.6)	1.3 (-20.1 to 27.0)
		Fungus	1280 (1140 to 1460)	1060 (957 to 1180)	-17.0 (-26.3 to -8.1)	43.2 (35.7 to 50.3)	36.5 (29.6 to 43.6)	-14.8 (-33.4 to 7.7)
		Group B Streptococcus	1520 (1360 to 1700)	1400 (1270 to 1540)	-7.8 (-18.1 to 2.4)	40.6 (34.5 to 46.8)	37.4 (31 to 44.4)	-7.3 (-26.2 to 15.5)
		Haemophilus influenzae	6470 (5960 to 7010)	3870 (3590 to 4190)	-40.1 (-44.7 to -35.5)	81.5 (70.5 to 92)	35.7 (30.1 to 42.1)	-56.0 (-64.5 to -45.5)
		Influenza	9000 (8360 to 9740)	263 (74.5 to 620)	-97.1 (-99.1 to -93.1)	253 (213 to 287)	7.29 (2.02 to 17.8)	-97.1 (-99.2 to -92.9)
		Klebsiella pneumoniae	4190 (3890 to 4540)	2610 (2420 to 2830)	-37.7 (-42.6 to -33.0)	154 (131 to 174)	103 (85.6 to 122)	-32.6 (-45.7 to -16.5)
		Legionella spp.	3230 (2910 to 3610)	3010 (2780 to 3270)	-6.7 (-16.4 to 3.5)	81 (68.6 to 93.9)	76.2 (63.5 to 89.7)	-5.4 (-25.2 to 18.4)
		Mycoplasma spp.	7480 (6840 to 8150)	3830 (3560 to 4130)	-48.7 (-53.0 to -44.6)	57.5 (49.1 to 65)	31.2 (26.3 to 36.8)	-45.6 (-56.1 to -33.1)
		Polymicrobial	243 (164 to 366)	185 (113 to 295)	-24.2 (-34.9 to -13.2)	11.7 (7.91 to 17.3)	7.83 (4.75 to 12.8)	-32.9 (-49.8 to -12.4)
		Pseudomonas aeruginosa	4380 (4020 to 4810)	4620 (4280 to 5000)	5.5 (-2.8 to 14.6)	116 (98 to 133)	124 (103 to 148)	7.4 (-14.5 to 33.9)
		Respiratory syncytial virus	4830 (4340 to 5350)	83.7 (23.7 to 197)	-98.3 (-99.5 to -95.9)	43.8 (39.4 to 48.2)	0.67 (0.19 to 1.59)	-98.5 (-99.6 to -96.3)
		Staphylococcus aureus	6350 (5880 to 6900)	8160 (7570 to 8830)	28.7 (18.6 to 38.1)	354 (300 to 402)	475 (393 to 563)	34.7 (7.2 to 67.1)
		Streptococcus pneumoniae	37500 (34900 to 40300)	9790 (9120 to 10600)	-73.9 (-75.7 to -72.0)	676 (585 to 754)	205 (171 to 242)	-69.5 (-75.4 to -62.6)
		Other viruses	20500 (19100 to 22100)	11100 (10400 to 11900)	-46.0 (-49.6 to -42.3)	135 (118 to 149)	86.3 (72.6 to 102)	-35.7 (-47.7 to -21.8)
		Other bacterial pathogens	6640 (5870 to 7530)	3910 (3420 to 4430)	-41.1 (-46.6 to -35.7)	158 (129 to 185)	94.4 (76.1 to 116)	-39.9 (-52.7 to -24.6)
	Under 5	*Acinetobacter baumannii*	37.3 (25.3 to 53.1)	6.52 (4.27 to 9.87)	-82.5 (-85.1 to -79.8)	2.51 (1.38 to 4.3)	0.28 (0.14 to 0.52)	-88.6 (-92.3 to -83.8)
		Chlamydia spp.	474 (402 to 556)	96.5 (81.1 to 113)	-79.6 (-82.6 to -76.4)	3.57 (3.03 to 4.15)	0.52 (0.39 to 0.68)	-85.2 (-89.4 to -80.4)

		Enterobacter spp.	58 (43.9 to 73.5)	28.7 (22.2 to 36.5)	-50.3 (-58.0 to -40.7)	1.87 (1.3 to 2.7)	0.56 (0.39 to 0.76)	-69.4 (-79.2 to -57.6)
		Escherichia coli	184 (154 to 218)	92.1 (77.2 to 109)	-49.9 (-57.3 to -41.7)	5.53 (4.61 to 6.54)	1.73 (1.29 to 2.24)	-68.4 (-77.2 to -58.0)
		Fungus	34.7 (24.6 to 47.8)	13.1 (9.32 to 17.8)	-62.0 (-68.9 to -54.3)	0.57 (0.41 to 0.78)	0.2 (0.13 to 0.29)	-64.2 (-74.7 to -52.4)
		Group B Streptococcus	168 (140 to 202)	70.3 (58.4 to 83.8)	-58.0 (-64.5 to -50.9)	4.6 (3.86 to 5.5)	1.08 (0.79 to 1.4)	-76.3 (-83.2 to -67.8)
		Haemophilus influenzae	1480 (1260 to 1730)	394 (333 to 464)	-73.2 (-76.9 to -69.3)	12.1 (10.6 to 13.9)	1.62 (1.22 to 2.03)	-86.5 (-90.1 to -82.5)
		Influenza	1210 (1040 to 1400)	12.7 (3.6 to 29.8)	-98.9 (-99.7 to -97.5)	18.3 (16 to 20.8)	0.14 (0.04 to 0.33)	-99.2 (-99.8 to -98.2)
		Klebsiella pneumoniae	667 (572 to 775)	141 (121 to 166)	-78.8 (-81.5 to -75.9)	14.3 (12.4 to 16.3)	2.55 (1.94 to 3.24)	-82.1 (-86.8 to -76.6)
		Legionella spp.	76.2 (54.6 to 105)	30.8 (21.7 to 41.9)	-59.5 (-65.3 to -53.3)	2.27 (1.67 to 3.01)	0.52 (0.35 to 0.72)	-76.7 (-84.2 to -68.0)
		Mycoplasma spp.	826 (701 to 975)	364 (308 to 425)	-55.8 (-61.8 to -49.4)	4.57 (3.93 to 5.24)	1.6 (1.21 to 2)	-64.8 (-74.4 to -53.7)
		Polymicrobial	33.5 (19.8 to 53.2)	10.2 (5.97 to 16.3)	-69.4 (-73.5 to -64.8)	3.02 (1.6 to 5.03)	0.5 (0.24 to 0.9)	-83.3 (-89.2 to -75.9)
		Pseudomonas aeruginosa	438 (370 to 511)	268 (228 to 314)	-38.6 (-47.2 to -29.4)	7.96 (6.84 to 9.18)	3.3 (2.48 to 4.16)	-58.4 (-69.5 to -45.3)
		Respiratory syncytial virus	3250 (2830 to 3730)	44.8 (12.7 to 105)	-98.6 (-99.6 to -96.8)	25.8 (22.4 to 29.2)	0.24 (0.07 to 0.56)	-99.1 (-99.7 to -97.8)
		Staphylococcus aureus	513 (442 to 588)	279 (235 to 328)	-45.7 (-51.9 to -38.9)	15.2 (13.3 to 17.2)	6.6 (5.04 to 8.3)	-56.4 (-67.9 to -43.4)
		Streptococcus pneumoniae	7790 (6790 to 8950)	659 (560 to 774)	-91.5 (-92.5 to -90.6)	81 (71.6 to 90.9)	6.15 (4.68 to 7.69)	-92.4 (-94.4 to -90.2)
		Other viruses	6590 (5720 to 7540)	1490 (1270 to 1740)	-77.4 (-79.7 to -75.0)	28.3 (24.8 to 31.9)	5.78 (4.39 to 7.23)	-79.5 (-85.0 to -73.7)
		Other bacterial pathogens	200 (157 to 250)	54.1 (42.7 to 68)	-72.9 (-77.0 to -68.1)	2.92 (2.24 to 3.81)	0.6 (0.42 to 0.82)	-79.2 (-85.0 to -72.3)
	5-14 years	*Acinetobacter baumannii*	96.8 (74.1 to 123)	15 (11.5 to 19.4)	-84.4 (-87.0 to -81.8)	0.39 (0.31 to 0.48)	0.04 (0.03 to 0.05)	-89.6 (-92.4 to -86.4)
		Chlamydia spp.	495 (390 to 621)	101 (77.4 to 129)	-79.6 (-82.8 to -76.3)	0.5 (0.42 to 0.6)	0.07 (0.05 to 0.08)	-86.4 (-90.1 to -82.2)
		Enterobacter spp.	52.5 (41.1 to 67.4)	28.1 (21.5 to 36.4)	-46.3 (-54.8 to -37.1)	0.2 (0.16 to 0.25)	0.07 (0.06 to 0.09)	-64.0 (-73.5 to -52.9)
		Escherichia coli	128 (101 to 161)	58.7 (45 to 74.5)	-54.0 (-61.8 to -46.5)	0.51 (0.42 to 0.6)	0.16 (0.12 to 0.19)	-69.2 (-77.5 to -59.8)
		Fungus	154 (120 to 194)	58.7 (45.3 to 75)	-61.7 (-67.9 to -55.0)	0.6 (0.49 to 0.72)	0.15 (0.12 to 0.19)	-74.4 (-81.3 to -66.8)
		Group B Streptococcus	165 (129 to 207)	73.5 (56 to 93.8)	-55.4 (-63.0 to -47.4)	0.47 (0.38 to 0.55)	0.14 (0.11 to 0.17)	-70.1 (-78.0 to -61.2)
		Haemophilus influenzae	689 (555 to 849)	210 (162 to 267)	-69.5 (-74.0 to -65.1)	1.04 (0.87 to 1.23)	0.14 (0.11 to 0.18)	-86.2 (-89.8 to -82.2)
		Influenza	965 (772 to 1180)	13.2 (3.53 to 31.5)	-98.6 (-99.6 to -96.8)	2.86 (2.41 to 3.34)	0.02 (0.01 to 0.06)	-99.2 (-99.8 to -97.9)

		Klebsiella pneumoniae	375 (301 to 456)	105 (81.6 to 132)	-71.9 (-76.2 to -67.9)	1.48 (1.25 to 1.72)	0.28 (0.22 to 0.34)	-81.2 (-85.9 to -75.7)
		Legionella spp.	368 (293 to 457)	156 (121 to 198)	-57.4 (-64.8 to -50.1)	1 (0.83 to 1.18)	0.28 (0.22 to 0.35)	-71.5 (-79.2 to -62.9)
		Mycoplasma spp.	1510 (1200 to 1860)	425 (330 to 540)	-71.8 (-76.1 to -67.6)	1.58 (1.34 to 1.85)	0.3 (0.23 to 0.37)	-81.1 (-85.9 to -75.5)
		Polymicrobial	20.1 (12.2 to 30.8)	7.16 (4.1 to 11.8)	-64.5 (-70.7 to -57.7)	0.08 (0.05 to 0.13)	0.02 (0.01 to 0.03)	-76.3 (-82.6 to -68.9)
		Pseudomonas aeruginosa	397 (316 to 487)	198 (153 to 249)	-50.0 (-57.7 to -42.5)	1.09 (0.91 to 1.28)	0.36 (0.28 to 0.44)	-66.5 (-75.5 to -56.6)
		Respiratory syncytial virus	289 (230 to 357)	3.5 (0.94 to 8.54)	-98.8 (-99.7 to -97.2)	0.39 (0.33 to 0.46)	0 (0 to 0.01)	-99.3 (-99.8 to -98.2)
		Staphylococcus aureus	588 (469 to 719)	339 (263 to 428)	-42.3 (-50.8 to -34.2)	3.3 (2.8 to 3.85)	1.26 (0.99 to 1.56)	-61.4 (-71.5 to -50.3)
		Streptococcus pneumoniae	5380 (4330 to 6590)	734 (571 to 925)	-86.3 (-88.4 to -84.5)	12.6 (10.6 to 14.7)	1.14 (0.89 to 1.41)	-90.9 (-93.2 to -88.3)
		Other viruses	2750 (2200 to 3350)	854 (662 to 1070)	-68.9 (-73.6 to -64.7)	2.52 (2.14 to 2.96)	0.52 (0.41 to 0.64)	-79.2 (-84.4 to -73.2)
		Other bacterial pathogens	549 (431 to 681)	140 (107 to 178)	-74.5 (-78.4 to -70.4)	1.22 (1.01 to 1.47)	0.21 (0.16 to 0.26)	-82.9 (-87.3 to -77.6)
	15-49 years	*Acinetobacter baumannii*	111 (93 to 132)	30.3 (25.7 to 35.6)	-72.5 (-76.1 to -68.6)	2.63 (2.11 to 3.22)	0.63 (0.49 to 0.81)	-75.8 (-82.1 to -68.8)
		Chlamydia spp.	566 (495 to 649)	203 (178 to 231)	-64.1 (-68.2 to -59.3)	3.33 (2.79 to 3.97)	1.05 (0.82 to 1.32)	-68.4 (-76.7 to -59.2)
		Enterobacter spp.	60 (50.8 to 70.7)	56.7 (48.9 to 65.5)	-5.3 (-17.4 to 7.3)	1.35 (1.1 to 1.64)	1.12 (0.88 to 1.41)	-16.6 (-38.9 to 5.9)
		Escherichia coli	146 (126 to 168)	118 (105 to 132)	-18.9 (-28.1 to -8.4)	3.4 (2.83 to 4.04)	2.41 (1.91 to 3.02)	-28.6 (-47.4 to -7.3)
		Fungus	176 (150 to 205)	118 (103 to 135)	-32.5 (-41.4 to -23.4)	4.03 (3.3 to 4.8)	2.38 (1.87 to 3.02)	-40.6 (-55.5 to -22.9)
		Group B Streptococcus	189 (163 to 220)	148 (130 to 168)	-21.3 (-31.2 to -10.6)	3.12 (2.61 to 3.76)	2.14 (1.71 to 2.68)	-30.7 (-50.1 to -10.5)
		Haemophilus influenzae	788 (695 to 889)	423 (376 to 472)	-46.3 (-51.5 to -40.7)	6.99 (5.97 to 8.26)	2.22 (1.77 to 2.78)	-68.1 (-76.1 to -59.4)
		Influenza	1100 (985 to 1250)	26.4 (7.56 to 62.7)	-97.6 (-99.3 to -94.3)	19.1 (16.3 to 22.5)	0.36 (0.1 to 0.9)	-98.1 (-99.5 to -95.2)
		Klebsiella pneumoniae	428 (382 to 481)	212 (189 to 236)	-50.5 (-55.1 to -45.4)	9.9 (8.42 to 11.6)	4.29 (3.43 to 5.37)	-56.4 (-67.4 to -44.4)
		Legionella spp.	421 (367 to 482)	315 (283 to 351)	-24.9 (-33.9 to -14.9)	6.7 (5.62 to 7.98)	4.4 (3.53 to 5.45)	-33.9 (-51.6 to -15.5)
		Mycoplasma spp.	1720 (1540 to 1950)	855 (762 to 957)	-50.3 (-55.6 to -44.7)	10.6 (8.98 to 12.4)	4.6 (3.69 to 5.73)	-56.3 (-67.3 to -43.8)
		Polymicrobial	23 (14.2 to 36.1)	14.4 (8.39 to 23.5)	-37.5 (-46.6 to -27.7)	0.54 (0.33 to 0.84)	0.3 (0.17 to 0.5)	-45.0 (-59.4 to -28.7)
		Pseudomonas aeruginosa	454 (402 to 514)	399 (357 to 445)	-11.9 (-21.2 to -2.5)	7.28 (6.2 to 8.55)	5.61 (4.48 to 7.02)	-22.4 (-42.6 to -0.6)
		Respiratory syncytial virus	330 (291 to 374)	7.03 (1.97 to 16.5)	-97.9 (-99.4 to -95.0)	2.61 (2.22 to 3.1)	0.04 (0.01 to 0.11)	-98.3 (-99.5 to -95.8)

		Staphylococcus aureus	673 (603 to 758)	683 (613 to 757)	1.7 (-7.5 to 11.7)	22.1 (18.7 to 25.8)	19.7 (15.8 to 24.6)	-10.5 (-33.2 to 13.9)
		Streptococcus pneumoniae	6150 (5540 to 6900)	1480 (1330 to 1650)	-75.9 (-78.1 to -73.7)	84.2 (71.9 to 98.5)	17.7 (14.3 to 22.1)	-78.8 (-84.2 to -72.8)
		Other viruses	3150 (2820 to 3520)	1720 (1550 to 1910)	-45.2 (-50.3 to -39.9)	16.9 (14.3 to 19.8)	8.1 (6.5 to 10.1)	-51.8 (-63.9 to -38.5)
		Other bacterial pathogens	627 (542 to 726)	282 (241 to 327)	-55.0 (-60.0 to -49.5)	8.18 (6.73 to 9.84)	3.23 (2.51 to 4.12)	-60.3 (-70.7 to -48.7)
	50-69 years	*Acinetobacter baumannii*	415 (351 to 490)	150 (127 to 175)	-63.7 (-68.1 to -58.8)	12.3 (9.79 to 15.3)	3.76 (2.81 to 4.92)	-69.2 (-78.6 to -58.1)
		Chlamydia spp.	1250 (1100 to 1440)	572 (509 to 648)	-54.3 (-59.6 to -48.8)	9.27 (7.49 to 11.1)	3.56 (2.66 to 4.54)	-61.2 (-72.6 to -47.5)
		Enterobacter spp.	218 (184 to 256)	267 (235 to 303)	22.6 (6.6 to 38.7)	6.16 (4.89 to 7.61)	6.33 (4.75 to 8.21)	4.1 (-27.7 to 43.1)
		Escherichia coli	587 (514 to 664)	595 (531 to 668)	1.6 (-9.5 to 14.5)	17.1 (13.8 to 20.7)	14.6 (10.9 to 18.7)	-13.8 (-39.2 to 17.4)
		Fungus	562 (489 to 653)	492 (429 to 563)	-12.2 (-23.7 to -1.4)	16.2 (12.9 to 19.7)	11.9 (8.93 to 15.3)	-25.5 (-47.6 to 2.4)
		Group B Streptococcus	486 (421 to 561)	486 (426 to 553)	0.2 (-12.9 to 13.4)	10.1 (8.16 to 12.2)	8.46 (6.33 to 10.9)	-15.0 (-40.1 to 14.8)
		Haemophilus influenzae	1730 (1540 to 1940)	1260 (1120 to 1400)	-26.9 (-34.4 to -19.3)	19.3 (15.7 to 23.1)	7.99 (6.02 to 10.2)	-58.1 (-70.0 to -42.5)
		Influenza	2090 (1870 to 2320)	67.6 (19.8 to 160)	-96.8 (-99.0 to -92.2)	45.4 (37.5 to 54.1)	1.12 (0.31 to 2.74)	-97.5 (-99.3 to -93.7)
		Klebsiella pneumoniae	1210 (1080 to 1340)	851 (759 to 945)	-29.3 (-36.4 to -21.9)	34.9 (28.4 to 41.4)	20.7 (15.5 to 26.3)	-40.1 (-57.4 to -19.3)
		Legionella spp.	1300 (1140 to 1480)	1250 (1120 to 1400)	-3.3 (-14.7 to 9.3)	25.9 (21 to 31.2)	21 (16 to 26.7)	-17.9 (-41.9 to 11.8)
		Mycoplasma spp.	1740 (1540 to 1940)	1000 (888 to 1120)	-42.1 (-49.0 to -35.6)	13.4 (11 to 16)	6.51 (4.92 to 8.26)	-50.9 (-65.1 to -33.4)
		Polymicrobial	71.8 (45.8 to 112)	59.1 (35.1 to 95.9)	-18.1 (-29.5 to -5.7)	2.12 (1.31 to 3.37)	1.47 (0.84 to 2.51)	-30.5 (-51.2 to -4.9)
		Pseudomonas aeruginosa	1410 (1260 to 1590)	1530 (1370 to 1710)	8.7 (-2.7 to 20.7)	28.4 (23 to 34)	26 (19.5 to 33.1)	-7.7 (-33.9 to 26.6)
		Respiratory syncytial virus	463 (408 to 527)	12.3 (3.52 to 28.7)	-97.3 (-99.2 to -93.7)	4.59 (3.74 to 5.58)	0.09 (0.03 to 0.23)	-98.0 (-99.4 to -94.9)
		Staphylococcus aureus	1840 (1650 to 2040)	2450 (2190 to 2730)	33.1 (19.9 to 46.3)	75.9 (62.4 to 89.6)	84.9 (64.1 to 108)	12.9 (-19.0 to 53.7)
		Streptococcus pneumoniae	8330 (7480 to 9220)	2840 (2550 to 3160)	-65.8 (-69.2 to -62.5)	143 (117 to 169)	41 (30.9 to 52.3)	-71.0 (-79.2 to -60.7)
		Other viruses	3720 (3350 to 4140)	2910 (2600 to 3230)	-21.7 (-29.5 to -13.7)	25.1 (20.6 to 29.7)	16.5 (12.4 to 20.9)	-33.6 (-52.5 to -10.2)
		Other bacterial pathogens	2080 (1800 to 2400)	1200 (1020 to 1390)	-42.3 (-48.9 to -36.0)	34 (27.2 to 41.5)	16.5 (12.3 to 21.7)	-51.0 (-65.5 to -32.5)
	70+ years	*Acinetobacter baumannii*	474 (400 to 563)	209 (180 to 244)	-55.8 (-61.2 to -50.0)	29.9 (22.8 to 36.4)	12.4 (9.87 to 15.3)	-58.1 (-67.7 to -45.4)
		Chlamydia spp.	1710 (1510 to 1970)	949 (847 to 1080)	-44.4 (-51.4 to -37.3)	26.7 (21.1 to 31.6)	14 (11.3 to 16.9)	-47.2 (-59.4 to -31.9)

		Enterobacter spp.	243 (206 to 291)	362 (318 to 415)	49.2 (30.7 to 69.8)	14.5 (11 to 17.8)	20.3 (16.3 to 24.8)	41.5 (10.1 to 84.7)
		Escherichia coli	825 (725 to 948)	1020 (919 to 1150)	23.6 (9.5 to 39.7)	50.9 (40.2 to 60.1)	59.1 (48.3 to 70.6)	17.2 (-9.8 to 50.6)
		Fungus	358 (306 to 423)	382 (334 to 437)	6.8 (-6.6 to 21.4)	21.8 (17 to 26.4)	21.9 (17.5 to 26.5)	1.3 (-21.8 to 31.8)
		Group B Streptococcus	510 (441 to 588)	620 (552 to 704)	21.9 (6.1 to 37.8)	22.4 (17.3 to 26.8)	25.6 (20.9 to 30.9)	15.6 (-11.0 to 50.5)
		Haemophilus influenzae	1780 (1600 to 2020)	1580 (1430 to 1780)	-11.1 (-20.4 to -1.3)	42 (33.4 to 50)	23.7 (19.6 to 28.2)	-43.0 (-55.7 to -26.7)
		Influenza	3630 (3280 to 4090)	143 (41.1 to 338)	-96.1 (-98.8 to -90.5)	167 (133 to 198)	5.64 (1.54 to 13.8)	-96.6 (-99.1 to -91.7)
		Klebsiella pneumoniae	1520 (1370 to 1720)	1300 (1190 to 1460)	-14.0 (-22.8 to -5.2)	93.2 (74.4 to 110)	75.4 (61.5 to 90)	-18.5 (-36.2 to 4.8)
		Legionella spp.	1070 (933 to 1240)	1250 (1130 to 1410)	17.7 (2.7 to 33.5)	45.2 (35.9 to 53.8)	49.9 (40.9 to 59.4)	11.6 (-13.7 to 42.9)
		Mycoplasma spp.	1680 (1490 to 1920)	1180 (1070 to 1330)	-29.5 (-37.5 to -21.2)	27.4 (21.4 to 32.2)	18.2 (15 to 21.6)	-33.2 (-47.8 to -13.4)
		Polymicrobial	94.2 (58.9 to 150)	94.2 (54.4 to 154)	-0.3 (-14.0 to 14.7)	5.89 (3.58 to 9.43)	5.54 (3.23 to 9.38)	-5.4 (-27.9 to 23.7)
		Pseudomonas aeruginosa	1680 (1500 to 1910)	2220 (2010 to 2500)	32.3 (18.4 to 46.7)	71.7 (56.9 to 84.5)	89.2 (73.1 to 107)	25.5 (-2.6 to 62.7)
		Respiratory syncytial virus	496 (434 to 573)	16.1 (4.55 to 37.5)	-96.8 (-99.0 to -92.3)	10.4 (8.16 to 12.5)	0.29 (0.08 to 0.7)	-97.2 (-99.2 to -93.1)
		Staphylococcus aureus	2730 (2450 to 3070)	4410 (4020 to 4930)	61.9 (45.4 to 79.2)	238 (190 to 281)	363 (298 to 430)	53.6 (20.3 to 98.0)
		Streptococcus pneumoniae	9800 (8860 to 11100)	4070 (3690 to 4550)	-58.4 (-62.5 to -54.1)	356 (282 to 420)	139 (116 to 165)	-60.5 (-69.1 to -49.4)
		Other viruses	4330 (3910 to 4900)	4120 (3740 to 4610)	-4.8 (-14.2 to 5.1)	61.8 (49.2 to 72.9)	55.4 (45.5 to 65.6)	-9.7 (-29.1 to 16.5)
		Other bacterial pathogens	3190 (2760 to 3690)	2230 (1930 to 2580)	-29.8 (-37.5 to -21.6)	112 (85.7 to 133)	73.8 (58.5 to 91.6)	-33.3 (-48.6 to -14.0)
Slovenia	All Ages	*Acinetobacter baumannii*	259 (222 to 300)	146 (126 to 170)	-43.5 (-49.5 to -37.1)	7.66 (6.61 to 8.8)	5.38 (4.37 to 6.34)	-29.8 (-40.0 to -19.3)
		Chlamydia spp.	1120 (1010 to 1240)	562 (513 to 621)	-49.7 (-54.8 to -44.4)	7.85 (7.06 to 8.58)	4.9 (4.14 to 5.54)	-37.5 (-46.5 to -28.1)
		Enterobacter spp.	187 (163 to 212)	224 (197 to 252)	20.2 (6.2 to 34.6)	5.05 (4.41 to 5.77)	7.55 (6.29 to 8.75)	49.6 (27.0 to 73.0)
		Escherichia coli	525 (475 to 587)	500 (455 to 551)	-4.7 (-14.6 to 6.1)	15.4 (13.9 to 16.9)	17.9 (15.2 to 20.2)	16.4 (-0.8 to 33.2)
		Fungus	347 (307 to 393)	287 (260 to 319)	-17.0 (-25.4 to -7.6)	8.36 (7.37 to 9.42)	8.98 (7.53 to 10.3)	7.6 (-7.9 to 25.5)
		Group B Streptococcus	420 (378 to 470)	416 (380 to 457)	-0.8 (-11.3 to 10.5)	8.01 (7.18 to 8.94)	9.87 (8.43 to 11.2)	23.4 (5.5 to 43.3)
		Haemophilus influenzae	1760 (1630 to 1920)	1100 (1010 to 1200)	-37.7 (-42.8 to -32.4)	16.5 (15.3 to 17.7)	8.98 (7.65 to 10.1)	-45.5 (-53.0 to -38.2)
		Influenza	2570 (2370 to 2780)	42.4 (8.91 to 133)	-98.4 (-99.6 to -94.8)	53.6 (49 to 57.5)	1.08 (0.23 to 3.25)	-98.0 (-99.6 to -93.9)

		Klebsiella pneumoniae	1120 (1040 to 1220)	894 (821 to 975)	-20.2 (-26.6 to -12.9)	30.1 (27.6 to 32.4)	30.8 (26.2 to 34.6)	2.4 (-11.3 to 16.3)
		Legionella spp.	1050 (942 to 1160)	1050 (959 to 1140)	0.2 (-9.9 to 11.3)	18.7 (16.8 to 20.7)	23.9 (20.3 to 27)	27.8 (8.0 to 48.3)
		Mycoplasma spp.	1960 (1800 to 2140)	1240 (1150 to 1340)	-36.9 (-41.9 to -31.5)	11.6 (10.7 to 12.6)	8.99 (7.72 to 10.2)	-22.4 (-33.6 to -11.1)
		Polymicrobial	60.5 (38.2 to 95.1)	47.5 (26.8 to 79.9)	-22.0 (-32.6 to -10.0)	1.96 (1.3 to 2.95)	1.84 (1.11 to 3.07)	-6.8 (-22.6 to 9.3)
		Pseudomonas aeruginosa	1380 (1270 to 1520)	1800 (1650 to 1950)	30.0 (18.0 to 42.3)	26.4 (24.2 to 28.7)	42.4 (36.1 to 47.9)	60.6 (37.4 to 83.0)
		Respiratory syncytial virus	1210 (1080 to 1360)	18.8 (3.92 to 59.8)	-98.5 (-99.7 to -95.2)	7.94 (7.39 to 8.5)	0.12 (0.03 to 0.37)	-98.5 (-99.7 to -95.3)
		Staphylococcus aureus	1920 (1770 to 2080)	2550 (2340 to 2780)	33.1 (22.0 to 44.7)	77.6 (71.3 to 83.3)	128 (109 to 145)	65.1 (43.3 to 87.5)
		Streptococcus pneumoniae	9730 (9030 to 10500)	4420 (4090 to 4790)	-54.6 (-57.7 to -51.2)	135 (125 to 143)	80.9 (69.5 to 91)	-40.0 (-47.7 to -32.3)
		Other viruses	5770 (5330 to 6280)	4550 (4220 to 4930)	-21.0 (-26.2 to -15.5)	29.2 (27.3 to 31.1)	30.8 (26.5 to 34.6)	5.5 (-8.1 to 19.5)
		Other bacterial pathogens	1880 (1660 to 2140)	1270 (1100 to 1460)	-32.8 (-39.2 to -25.7)	31.9 (27.9 to 36.1)	26.4 (21.4 to 31.2)	-17.4 (-28.7 to -6.1)
	Under 5	*Acinetobacter baumannii*	5.39 (3.66 to 7.87)	1.34 (0.92 to 1.94)	-75.1 (-78.5 to -71.2)	0.24 (0.14 to 0.41)	0.08 (0.04 to 0.14)	-68.9 (-75.6 to -60.7)
		Chlamydia spp.	78.6 (65 to 95)	22.6 (18 to 28.3)	-71.3 (-75.4 to -67.2)	0.38 (0.33 to 0.43)	0.08 (0.07 to 0.1)	-78.6 (-82.7 to -73.9)
		Enterobacter spp.	11.2 (8.35 to 14.7)	7.11 (5.18 to 9.71)	-36.2 (-46.4 to -24.8)	0.23 (0.17 to 0.32)	0.09 (0.06 to 0.13)	-61.1 (-69.2 to -51.7)
		Escherichia coli	33.3 (27.2 to 40.6)	19.1 (15.1 to 23.7)	-42.5 (-51.0 to -33.2)	0.64 (0.55 to 0.73)	0.21 (0.17 to 0.25)	-67.2 (-73.4 to -60.0)
		Fungus	6.43 (4.43 to 9.06)	3.28 (2.26 to 4.65)	-48.8 (-57.0 to -39.2)	0.07 (0.05 to 0.1)	0.01 (0.01 to 0.02)	-79.5 (-84.5 to -73.9)
		Group B Streptococcus	30.7 (24.9 to 37.5)	17.1 (13.5 to 21.7)	-44.2 (-53.1 to -35.6)	0.52 (0.46 to 0.59)	0.18 (0.15 to 0.22)	-64.9 (-71.6 to -57.6)
		Haemophilus influenzae	290 (237 to 349)	97.2 (77.7 to 120)	-66.5 (-71.2 to -61.5)	1.56 (1.37 to 1.76)	0.13 (0.1 to 0.16)	-91.6 (-93.4 to -89.5)
		Influenza	235 (194 to 282)	1.73 (0.36 to 5.42)	-99.3 (-99.8 to -97.7)	2.34 (2.07 to 2.61)	0.01 (0 to 0.02)	-99.7 (-99.9 to -99.0)
		Klebsiella pneumoniae	122 (101 to 147)	40.5 (32.6 to 50.3)	-66.8 (-70.8 to -62.4)	1.73 (1.54 to 1.94)	0.32 (0.27 to 0.38)	-81.2 (-84.9 to -77.2)
		Legionella spp.	15.5 (11 to 21.3)	8.56 (5.73 to 12.3)	-44.8 (-53.1 to -35.5)	0.28 (0.22 to 0.36)	0.12 (0.09 to 0.15)	-57.8 (-66.9 to -47.2)
		Mycoplasma spp.	160 (131 to 194)	99.8 (79.9 to 124)	-37.5 (-45.7 to -28.4)	0.58 (0.5 to 0.65)	0.16 (0.12 to 0.19)	-73.1 (-79.0 to -66.7)
		Polymicrobial	5.53 (3.21 to 8.76)	1.85 (1.07 to 3.01)	-66.5 (-71.3 to -61.0)	0.34 (0.18 to 0.55)	0.17 (0.08 to 0.29)	-51.3 (-62.2 to -39.4)
		Pseudomonas aeruginosa	91.2 (75.1 to 112)	82.1 (65.8 to 101)	-9.8 (-22.0 to 2.8)	1.05 (0.92 to 1.18)	0.44 (0.37 to 0.52)	-57.9 (-66.1 to -48.6)
		Respiratory syncytial virus	710 (596 to 847)	9.2 (1.92 to 28.5)	-98.7 (-99.7 to -96.0)	3.62 (3.25 to 4.02)	0.02 (0 to 0.06)	-99.4 (-99.9 to -98.3)

		Staphylococcus aureus	102 (85.1 to 122)	71.1 (56.8 to 87.8)	-30.5 (-38.3 to -22.1)	2.01 (1.78 to 2.24)	0.65 (0.54 to 0.77)	-67.6 (-74.0 to -60.6)
		Streptococcus pneumoniae	1500 (1240 to 1810)	283 (227 to 351)	-81.1 (-83.1 to -79.0)	10.7 (9.46 to 11.9)	0.9 (0.73 to 1.07)	-91.6 (-93.3 to -89.6)
		Other viruses	1430 (1190 to 1720)	554 (444 to 684)	-61.3 (-65.5 to -57.1)	4.15 (3.69 to 4.64)	0.77 (0.63 to 0.91)	-81.5 (-85.0 to -77.3)
		Other bacterial pathogens	36.2 (27.7 to 46.5)	13.6 (10.2 to 17.9)	-62.3 (-68.2 to -56.1)	0.34 (0.26 to 0.44)	0.08 (0.05 to 0.11)	-77.5 (-83.1 to -71.1)
	5-14 years	*Acinetobacter baumannii*	15.1 (11 to 20.1)	5.72 (4.21 to 7.62)	-62.1 (-66.1 to -58.2)	0.05 (0.04 to 0.06)	0.01 (0.01 to 0.01)	-81.1 (-84.5 to -76.9)
		Chlamydia spp.	87.7 (65.4 to 114)	31.8 (23.6 to 41.6)	-63.6 (-67.3 to -59.8)	0.07 (0.06 to 0.08)	0.01 (0.01 to 0.01)	-81.9 (-85.2 to -77.9)
		Enterobacter spp.	10.9 (7.99 to 14.5)	9.15 (6.69 to 12.2)	-15.5 (-25.2 to -5.4)	0.03 (0.03 to 0.04)	0.01 (0.01 to 0.02)	-58.0 (-65.8 to -48.6)
		Escherichia coli	24.7 (18.4 to 32.4)	16.1 (11.9 to 21.1)	-34.6 (-41.6 to -27.3)	0.08 (0.07 to 0.09)	0.03 (0.02 to 0.03)	-67.4 (-73.0 to -60.4)
		Fungus	29.6 (22 to 38.5)	18.6 (13.8 to 24.8)	-37.1 (-43.5 to -30.1)	0.09 (0.08 to 0.11)	0.03 (0.02 to 0.03)	-68.7 (-74.4 to -62.1)
		Group B Streptococcus	33 (24.4 to 42.8)	24.2 (17.9 to 31.7)	-26.5 (-34.9 to -18.5)	0.07 (0.06 to 0.09)	0.03 (0.02 to 0.03)	-63.4 (-70.3 to -55.1)
		Haemophilus influenzae	143 (108 to 187)	64.5 (47.9 to 84.2)	-54.8 (-58.9 to -50.7)	0.17 (0.15 to 0.19)	0.03 (0.02 to 0.03)	-84.6 (-87.4 to -81.4)
		Influenza	196 (149 to 256)	2.23 (0.46 to 7.06)	-98.9 (-99.8 to -96.4)	0.47 (0.42 to 0.52)	0 (0 to 0.01)	-99.4 (-99.9 to -98.3)
		Klebsiella pneumoniae	72.1 (54.6 to 93.9)	37.4 (27.9 to 48.4)	-48.2 (-52.4 to -44.0)	0.23 (0.2 to 0.26)	0.06 (0.05 to 0.07)	-74.2 (-78.6 to -68.7)
		Legionella spp.	83.7 (63.2 to 110)	61.1 (45.9 to 78.9)	-26.9 (-35.0 to -18.8)	0.18 (0.16 to 0.21)	0.07 (0.06 to 0.08)	-63.6 (-70.6 to -55.4)
		Mycoplasma spp.	308 (229 to 399)	159 (120 to 209)	-48.2 (-52.9 to -43.8)	0.26 (0.23 to 0.29)	0.07 (0.06 to 0.08)	-74.2 (-78.7 to -69.0)
		Polymicrobial	3.56 (2.08 to 5.72)	1.96 (1.07 to 3.3)	-45.3 (-52.4 to -38.2)	0.01 (0.01 to 0.02)	0 (0 to 0.01)	-72.8 (-78.0 to -66.5)
		Pseudomonas aeruginosa	88.1 (66 to 115)	81.3 (61.3 to 105)	-7.6 (-16.0 to 1.4)	0.19 (0.17 to 0.22)	0.09 (0.08 to 0.1)	-54.0 (-62.3 to -44.4)
		Respiratory syncytial virus	66.8 (50.8 to 87.3)	0.95 (0.19 to 3.09)	-98.6 (-99.7 to -95.5)	0.07 (0.06 to 0.08)	0 (0 to 0)	-99.3 (-99.9 to -97.9)
		Staphylococcus aureus	123 (93.6 to 159)	110 (82.3 to 143)	-10.5 (-17.5 to -4.3)	0.55 (0.49 to 0.62)	0.24 (0.21 to 0.28)	-55.4 (-63.2 to -46.0)
		Streptococcus pneumoniae	1080 (820 to 1410)	376 (282 to 490)	-65.1 (-67.8 to -62.8)	2.01 (1.79 to 2.24)	0.35 (0.3 to 0.4)	-82.6 (-85.6 to -78.9)
		Other viruses	625 (475 to 813)	388 (290 to 504)	-37.9 (-42.6 to -33.5)	0.46 (0.41 to 0.51)	0.14 (0.12 to 0.16)	-69.1 (-74.4 to -62.6)
		Other bacterial pathogens	104 (78.7 to 137)	46 (33.7 to 60.4)	-55.9 (-60.1 to -51.6)	0.19 (0.16 to 0.22)	0.04 (0.03 to 0.05)	-78.0 (-81.8 to -73.2)
	15-49 years	*Acinetobacter baumannii*	19.8 (16.1 to 23.8)	6.89 (5.57 to 8.33)	-65.2 (-69.0 to -60.8)	0.39 (0.33 to 0.46)	0.14 (0.11 to 0.17)	-64.3 (-71.2 to -56.6)
		Chlamydia spp.	115 (99.1 to 133)	38.3 (32.7 to 44.7)	-66.6 (-70.1 to -62.8)	0.56 (0.49 to 0.64)	0.19 (0.16 to 0.22)	-65.8 (-72.3 to -58.2)

		Enterobacter spp.	14.2 (11.8 to 16.9)	11 (9.05 to 13.2)	-22.5 (-31.6 to -12.4)	0.26 (0.22 to 0.31)	0.21 (0.17 to 0.25)	-20.5 (-36.4 to -2.3)
		Escherichia coli	32.4 (27.8 to 37.3)	19.4 (16.7 to 22.4)	-40.0 (-46.6 to -32.7)	0.62 (0.55 to 0.7)	0.38 (0.31 to 0.44)	-38.4 (-50.3 to -26.3)
		Fungus	38.9 (32.9 to 45.6)	22.4 (19 to 26.2)	-42.3 (-48.6 to -35.3)	0.73 (0.64 to 0.84)	0.43 (0.36 to 0.52)	-40.8 (-52.3 to -27.7)
		Group B Streptococcus	43.3 (37.1 to 51.2)	29.1 (24.8 to 34.1)	-32.6 (-40.3 to -23.7)	0.59 (0.51 to 0.68)	0.4 (0.33 to 0.48)	-30.9 (-44.3 to -15.2)
		Haemophilus influenzae	187 (164 to 215)	77.6 (66.9 to 89.9)	-58.5 (-62.1 to -54.5)	1.36 (1.23 to 1.52)	0.4 (0.33 to 0.46)	-71.0 (-76.4 to -65.0)
		Influenza	258 (227 to 294)	2.68 (0.56 to 8.34)	-99.0 (-99.8 to -96.8)	3.67 (3.32 to 4.08)	0.04 (0.01 to 0.12)	-99.0 (-99.8 to -96.8)
		Klebsiella pneumoniae	94.6 (82.8 to 107)	45 (38.7 to 51.6)	-52.4 (-56.2 to -48.1)	1.8 (1.62 to 2)	0.87 (0.73 to 1.02)	-51.2 (-60.3 to -41.5)
		Legionella spp.	110 (94.5 to 127)	73.6 (63.2 to 84.1)	-32.9 (-40.3 to -24.9)	1.44 (1.27 to 1.62)	0.98 (0.81 to 1.15)	-31.2 (-44.9 to -16.6)
		Mycoplasma spp.	404 (352 to 460)	192 (166 to 222)	-52.5 (-56.8 to -47.8)	2.03 (1.83 to 2.29)	0.99 (0.82 to 1.15)	-51.3 (-60.0 to -41.2)
		Polymicrobial	4.68 (2.8 to 7.45)	2.36 (1.3 to 3.91)	-49.8 (-56.6 to -43.2)	0.09 (0.05 to 0.14)	0.05 (0.03 to 0.08)	-48.5 (-58.8 to -37.0)
		Pseudomonas aeruginosa	116 (100 to 133)	97.9 (84.4 to 113)	-15.2 (-23.2 to -5.9)	1.52 (1.36 to 1.71)	1.32 (1.11 to 1.54)	-13.0 (-29.4 to 5.2)
		Respiratory syncytial virus	87.7 (76.6 to 99.9)	1.14 (0.23 to 3.55)	-98.7 (-99.7 to -96.0)	0.57 (0.51 to 0.64)	0.01 (0 to 0.02)	-98.7 (-99.7 to -96.0)
		Staphylococcus aureus	161 (141 to 183)	132 (114 to 152)	-17.9 (-24.2 to -11.3)	4.35 (3.95 to 4.8)	3.66 (3.06 to 4.24)	-15.8 (-31.2 to 0.7)
		Streptococcus pneumoniae	1410 (1250 to 1600)	453 (392 to 519)	-68.0 (-70.3 to -65.5)	15.9 (14.5 to 17.5)	5.2 (4.37 to 6.01)	-67.2 (-73.3 to -60.9)
		Other viruses	820 (720 to 929)	467 (404 to 533)	-43.0 (-47.1 to -38.6)	3.62 (3.29 to 4.02)	2.11 (1.77 to 2.45)	-41.5 (-52.3 to -30.1)
		Other bacterial pathogens	137 (115 to 159)	55.5 (45.8 to 66.6)	-59.5 (-63.2 to -55.3)	1.47 (1.27 to 1.69)	0.61 (0.49 to 0.73)	-58.4 (-66.4 to -49.4)
	50-69 years	*Acinetobacter baumannii*	91.2 (76.7 to 108)	34.8 (29.5 to 40.8)	-61.8 (-66.2 to -57.0)	1.62 (1.37 to 1.9)	0.8 (0.64 to 0.98)	-50.0 (-59.6 to -39.4)
		Chlamydia spp.	313 (274 to 357)	108 (95.4 to 122)	-65.5 (-69.2 to -61.3)	1.38 (1.21 to 1.56)	0.62 (0.51 to 0.74)	-54.9 (-63.7 to -45.1)
		Enterobacter spp.	63.7 (54.5 to 74.1)	52.6 (45.2 to 60)	-17.1 (-27.7 to -6.3)	1.07 (0.92 to 1.23)	1.16 (0.93 to 1.41)	8.5 (-13.4 to 33.1)
		Escherichia coli	160 (139 to 183)	99.9 (89.2 to 111)	-37.3 (-44.5 to -29.6)	2.77 (2.46 to 3.14)	2.27 (1.85 to 2.68)	-17.9 (-34.1 to -1.4)
		Fungus	153 (132 to 178)	94.6 (82.8 to 108)	-37.9 (-44.8 to -30.5)	2.61 (2.27 to 3.01)	2.12 (1.71 to 2.57)	-18.7 (-34.4 to -0.7)
		Group B Streptococcus	137 (118 to 158)	96.4 (84.8 to 109)	-29.4 (-38.1 to -20.0)	1.69 (1.48 to 1.95)	1.55 (1.28 to 1.85)	-7.5 (-25.7 to 13.8)
		Haemophilus influenzae	505 (449 to 564)	243 (217 to 270)	-51.9 (-56.8 to -46.6)	3.35 (3.01 to 3.72)	1.44 (1.19 to 1.69)	-57.0 (-65.4 to -48.1)
		Influenza	600 (535 to 670)	6.78 (1.4 to 21.5)	-98.9 (-99.8 to -96.4)	7.77 (7.03 to 8.63)	0.11 (0.02 to 0.34)	-98.6 (-99.7 to -95.7)

		Klebsiella pneumoniae	327 (292 to 364)	188 (169 to 209)	-42.4 (-47.9 to -36.2)	5.65 (5.1 to 6.27)	4.25 (3.52 to 5)	-24.6 (-39.2 to -9.2)
		Legionella spp.	417 (367 to 475)	299 (269 to 331)	-28.2 (-36.4 to -19.4)	4.96 (4.39 to 5.66)	4.65 (3.81 to 5.48)	-5.9 (-24.9 to 15.0)
		Mycoplasma spp.	499 (444 to 562)	233 (209 to 260)	-53.3 (-58.0 to -48.1)	2.29 (2.05 to 2.59)	1.4 (1.15 to 1.66)	-38.8 (-50.4 to -25.5)
		Polymicrobial	17.9 (11 to 28.7)	9.79 (5.57 to 16.5)	-45.6 (-53.2 to -37.7)	0.32 (0.19 to 0.51)	0.22 (0.13 to 0.39)	-28.8 (-42.8 to -12.1)
		Pseudomonas aeruginosa	442 (392 to 496)	390 (349 to 433)	-11.6 (-20.9 to -2.3)	5.3 (4.76 to 5.94)	6.12 (5.03 to 7.27)	15.8 (-6.1 to 39.1)
		Respiratory syncytial virus	151 (131 to 171)	2.05 (0.43 to 6.48)	-98.6 (-99.7 to -95.8)	0.89 (0.77 to 1.02)	0.02 (0 to 0.05)	-98.3 (-99.6 to -94.7)
		Staphylococcus aureus	543 (488 to 606)	481 (432 to 531)	-11.3 (-19.0 to -2.6)	13.3 (12.1 to 14.8)	15.4 (12.8 to 18.2)	16.2 (-5.5 to 39.4)
		Streptococcus pneumoniae	2350 (2110 to 2620)	849 (763 to 937)	-63.8 (-67.2 to -60.2)	24 (21.8 to 26.5)	11.3 (9.36 to 13.3)	-52.6 (-61.2 to -43.3)
		Other viruses	1190 (1070 to 1330)	814 (732 to 907)	-31.6 (-37.7 to -24.7)	4.79 (4.35 to 5.31)	4.28 (3.56 to 5.04)	-10.5 (-26.9 to 7.3)
		Other bacterial pathogens	558 (481 to 648)	241 (206 to 281)	-56.7 (-61.1 to -51.5)	5.44 (4.69 to 6.31)	3.08 (2.46 to 3.78)	-43.2 (-54.5 to -31.8)
	70+ years	*Acinetobacter baumannii*	128 (108 to 151)	97.7 (82 to 116)	-23.4 (-33.4 to -12.8)	5.37 (4.57 to 6.26)	4.35 (3.45 to 5.14)	-18.9 (-30.8 to -5.8)
		Chlamydia spp.	524 (456 to 600)	361 (321 to 411)	-30.9 (-40.0 to -21.5)	5.46 (4.79 to 6.07)	3.99 (3.28 to 4.53)	-26.8 (-37.6 to -15.3)
		Enterobacter spp.	87 (74.2 to 102)	144 (124 to 166)	66.3 (43.2 to 90.3)	3.46 (2.94 to 4.03)	6.08 (4.95 to 7.06)	76.0 (48.8 to 105.2)
		Escherichia coli	275 (244 to 318)	345 (306 to 388)	25.8 (8.8 to 42.2)	11.3 (9.97 to 12.6)	15.1 (12.5 to 17)	33.1 (13.7 to 53.4)
		Fungus	119 (103 to 138)	148 (129 to 171)	24.7 (8.5 to 42.1)	4.85 (4.17 to 5.58)	6.38 (5.26 to 7.4)	31.9 (12.5 to 53.3)
		Group B Streptococcus	176 (153 to 201)	249 (221 to 282)	41.7 (23.4 to 61.9)	5.15 (4.49 to 5.85)	7.71 (6.43 to 8.8)	50.0 (27.9 to 73.9)
		Haemophilus influenzae	639 (576 to 718)	616 (550 to 691)	-3.5 (-15.1 to 8.6)	10 (9.01 to 10.9)	6.99 (5.82 to 7.92)	-30.3 (-40.1 to -20.4)
		Influenza	1280 (1160 to 1430)	28.9 (6.05 to 90.8)	-97.7 (-99.5 to -92.9)	39.3 (35.5 to 42.8)	0.92 (0.2 to 2.74)	-97.7 (-99.5 to -92.9)
		Klebsiella pneumoniae	506 (459 to 569)	583 (522 to 650)	15.5 (1.6 to 29.5)	20.7 (18.6 to 22.6)	25.3 (21.3 to 28.6)	22.3 (5.0 to 40.5)
		Legionella spp.	421 (369 to 484)	605 (538 to 680)	44.1 (24.9 to 64.3)	11.9 (10.5 to 13.3)	18.1 (15 to 20.5)	52.6 (28.8 to 76.8)
		Mycoplasma spp.	592 (529 to 676)	554 (493 to 620)	-6.3 (-18.3 to 5.2)	6.44 (5.7 to 7.13)	6.38 (5.32 to 7.24)	-0.8 (-14.9 to 14.1)
		Polymicrobial	28.8 (17.6 to 47.5)	31.6 (17.5 to 54.4)	9.1 (-7.4 to 26.6)	1.2 (0.72 to 1.97)	1.39 (0.79 to 2.44)	15.5 (-3.9 to 35.7)
		Pseudomonas aeruginosa	646 (579 to 735)	1140 (1020 to 1270)	77.4 (55.9 to 99.8)	18.4 (16.5 to 20.1)	34.4 (28.6 to 39.1)	87.8 (61.3 to 116.4)
		Respiratory syncytial virus	199 (175 to 228)	5.4 (1.12 to 17)	-97.3 (-99.4 to -91.4)	2.78 (2.45 to 3.12)	0.08 (0.02 to 0.24)	-97.2 (-99.4 to -91.3)

		Staphylococcus aureus	986 (890 to 1100)	1750 (1580 to 1950)	78.1 (57.8 to 98.0)	57.4 (51.6 to 62.3)	108 (90.7 to 122)	88.5 (62.5 to 115.3)
		Streptococcus pneumoniae	3390 (3070 to 3790)	2460 (2220 to 2740)	-27.4 (-35.7 to -18.9)	82.2 (74.2 to 88.9)	63.1 (52.6 to 71)	-23.2 (-33.4 to -12.4)
		Other viruses	1700 (1540 to 1910)	2330 (2100 to 2610)	37.2 (21.5 to 53.3)	16.2 (14.6 to 17.6)	23.5 (19.7 to 26.5)	45.2 (26.3 to 65.3)
		Other bacterial pathogens	1050 (912 to 1220)	909 (774 to 1060)	-13.1 (-23.3 to -2.0)	24.5 (21 to 27.9)	22.6 (18 to 26.8)	-7.9 (-20.5 to 5.5)
Eastern Europe	All Ages	*Acinetobacter baumannii*	64000 (55900 to 72800)	35600 (31200 to 40300)	-44.3 (-46.9 to -41.4)	490 (422 to 584)	471 (408 to 541)	-3.5 (-19.9 to 12.8)
		Chlamydia spp.	234000 (215000 to 255000)	146000 (136000 to 158000)	-37.4 (-40.3 to -34.4)	457 (430 to 483)	483 (435 to 531)	5.7 (-4.1 to 15.1)
		Enterobacter spp.	29300 (25700 to 33000)	31000 (27800 to 34300)	5.8 (-0.7 to 11.9)	222 (190 to 263)	388 (342 to 437)	75.7 (50.2 to 103.0)
		Escherichia coli	88800 (80500 to 97700)	81300 (75000 to 88400)	-8.4 (-12.8 to -3.9)	686 (637 to 735)	1050 (957 to 1160)	53.8 (40.0 to 67.3)
		Fungus	67200 (59500 to 76400)	60100 (54600 to 66400)	-10.6 (-15.3 to -5.6)	379 (340 to 418)	765 (672 to 858)	102.3 (82.3 to 123.5)
		Group B Streptococcus	73400 (66100 to 82000)	67700 (62300 to 73900)	-7.6 (-12.5 to -2.9)	465 (434 to 501)	628 (568 to 696)	35.1 (22.4 to 48.6)
		Haemophilus influenzae	315000 (291000 to 342000)	239000 (224000 to 255000)	-24.2 (-28.3 to -19.8)	965 (911 to 1020)	953 (869 to 1030)	-1.2 (-10.0 to 8.0)
		Influenza	418000 (389000 to 447000)	55100 (26100 to 102000)	-86.8 (-93.7 to -75.8)	2140 (2060 to 2220)	518 (224 to 999)	-75.8 (-89.7 to -52.9)
		Klebsiella pneumoniae	208000 (194000 to 223000)	156000 (147000 to 166000)	-25.0 (-28.4 to -21.6)	1520 (1460 to 1590)	1980 (1810 to 2170)	30.3 (18.1 to 42.5)
		Legionella spp.	145000 (131000 to 159000)	169000 (156000 to 183000)	16.7 (11.3 to 22.9)	634 (582 to 697)	1500 (1350 to 1670)	137.1 (114.4 to 160.8)
		Mycoplasma spp.	376000 (345000 to 409000)	291000 (272000 to 312000)	-22.7 (-26.1 to -18.9)	644 (613 to 679)	987 (895 to 1080)	53.3 (39.5 to 68.5)
		Polymicrobial	11600 (7740 to 17700)	9040 (5610 to 14500)	-22.8 (-29.4 to -16.0)	202 (131 to 295)	131 (86.9 to 205)	-33.6 (-58.1 to -3.4)
		Pseudomonas aeruginosa	193000 (178000 to 209000)	216000 (202000 to 231000)	11.9 (7.2 to 16.9)	970 (924 to 1020)	1920 (1730 to 2100)	97.5 (80.8 to 115.3)
		Respiratory syncytial virus	226000 (201000 to 255000)	17600 (8590 to 32400)	-92.2 (-96.2 to -86.2)	1070 (1020 to 1130)	68.5 (30.7 to 134)	-93.6 (-97.2 to -87.6)
		Staphylococcus aureus	287000 (269000 to 306000)	317000 (297000 to 337000)	10.4 (6.2 to 14.7)	2600 (2520 to 2680)	5740 (5250 to 6250)	120.4 (102.2 to 139.8)
		Streptococcus pneumoniae	1890000 (1760000 to 2020000)	1100000 (1030000 to 1170000)	-41.9 (-44.7 to -39.0)	7530 (7360 to 7720)	8210 (7470 to 8970)	9.1 (-0.7 to 19.5)
		Other viruses	958000 (886000 to 1040000)	729000 (684000 to 774000)	-23.9 (-28.3 to -19.6)	1720 (1670 to 1790)	2120 (1940 to 2310)	23.1 (12.0 to 34.2)
		Other bacterial pathogens	315000 (283000 to 347000)	255000 (228000 to 285000)	-19.1 (-22.4 to -15.7)	1080 (985 to 1180)	1860 (1620 to 2110)	72.0 (56.2 to 88.1)
	Under 5	*Acinetobacter baumannii*	1620 (1170 to 2230)	181 (130 to 244)	-88.8 (-90.3 to -87.2)	132 (75.6 to 213)	10.4 (5.7 to 17.4)	-92.1 (-93.2 to -91.0)
		Chlamydia spp.	26800 (22200 to 32200)	3530 (2900 to 4270)	-86.8 (-88.5 to -84.9)	168 (154 to 182)	13.9 (12.3 to 15.6)	-91.7 (-92.6 to -90.8)

		Enterobacter spp.	2820 (2100 to 3660)	612 (456 to 811)	-78.2 (-81.3 to -75.1)	78.9 (54.2 to 115)	9.57 (6.88 to 13.5)	-87.8 (-89.5 to -86.0)
		Escherichia coli	9700 (7890 to 11800)	2030 (1650 to 2440)	-79.0 (-82.0 to -76.0)	241 (214 to 272)	28 (24.3 to 31.8)	-88.4 (-89.7 to -87.0)
		Fungus	2020 (1400 to 2830)	357 (250 to 487)	-82.3 (-84.9 to -79.6)	21 (15 to 27.9)	2.57 (1.84 to 3.41)	-87.7 (-89.4 to -86.0)
		Group B Streptococcus	8060 (6460 to 9980)	1610 (1300 to 1970)	-79.9 (-82.4 to -77.0)	209 (189 to 232)	22.1 (19.4 to 24.9)	-89.4 (-90.7 to -88.2)
		Haemophilus influenzae	78500 (65400 to 94200)	13600 (11100 to 16300)	-82.6 (-84.9 to -80.0)	469 (427 to 517)	38.1 (33.8 to 42.2)	-91.9 (-92.9 to -90.9)
		Influenza	65900 (55800 to 77400)	1640 (846 to 2910)	-97.5 (-98.7 to -95.7)	700 (654 to 749)	10 (4.24 to 19.4)	-98.6 (-99.4 to -97.2)
		Klebsiella pneumoniae	37600 (31700 to 44300)	5120 (4200 to 6090)	-86.4 (-88.0 to -84.5)	582 (538 to 628)	53.2 (47.4 to 58.9)	-90.9 (-91.9 to -89.8)
		Legionella spp.	3170 (2170 to 4670)	747 (505 to 1070)	-76.4 (-79.5 to -73.0)	97.7 (73.1 to 127)	11.9 (9.28 to 15)	-87.8 (-89.4 to -85.7)
		Mycoplasma spp.	44700 (37500 to 53600)	11000 (8960 to 13100)	-75.4 (-78.7 to -72.1)	180 (164 to 198)	26.1 (23.2 to 29.2)	-85.5 (-87.2 to -83.9)
		Polymicrobial	949 (594 to 1450)	168 (101 to 263)	-82.3 (-84.5 to -80.0)	141 (76.8 to 226)	15.4 (7.93 to 25.6)	-89.1 (-90.8 to -87.4)
		Pseudomonas aeruginosa	21300 (17700 to 25600)	6010 (4940 to 7130)	-71.7 (-75.2 to -67.7)	310 (287 to 337)	47 (41.8 to 52.1)	-84.8 (-86.6 to -83.2)
		Respiratory syncytial virus	157000 (132000 to 183000)	6120 (3080 to 11100)	-96.1 (-98.0 to -93.1)	946 (893 to 1010)	19.1 (8.17 to 37.5)	-98.0 (-99.1 to -96.1)
		Staphylococcus aureus	27200 (23100 to 32000)	5960 (4920 to 7110)	-78.1 (-80.6 to -75.2)	569 (531 to 604)	78.3 (69.9 to 86.3)	-86.2 (-87.8 to -84.8)
		Streptococcus pneumoniae	437000 (374000 to 510000)	44500 (36600 to 52700)	-89.8 (-91.0 to -88.5)	2940 (2790 to 3080)	198 (176 to 218)	-93.3 (-94.0 to -92.5)
		Other viruses	333000 (283000 to 389000)	56800 (46800 to 67300)	-82.9 (-84.9 to -80.7)	952 (903 to 1010)	111 (99 to 121)	-88.4 (-89.6 to -87.1)
		Other bacterial pathogens	11300 (8710 to 14200)	1840 (1420 to 2350)	-83.7 (-85.9 to -81.2)	124 (95.1 to 165)	12.7 (9.77 to 16.7)	-89.8 (-91.1 to -88.5)
	5-14 years	*Acinetobacter baumannii*	5800 (4400 to 7470)	1470 (1120 to 1900)	-74.7 (-76.2 to -73.2)	7.63 (6.6 to 8.73)	1.74 (1.5 to 1.98)	-77.2 (-78.9 to -75.5)
		Chlamydia spp.	26900 (20800 to 34500)	8480 (6600 to 10700)	-68.4 (-70.3 to -66.6)	8.74 (8 to 9.6)	2.5 (2.27 to 2.73)	-71.4 (-73.3 to -69.5)
		Enterobacter spp.	2530 (1920 to 3300)	1310 (1000 to 1710)	-48.2 (-52.2 to -44.7)	3.12 (2.75 to 3.57)	1.46 (1.29 to 1.64)	-53.3 (-56.7 to -49.3)
		Escherichia coli	6490 (5000 to 8400)	2900 (2240 to 3690)	-55.4 (-58.2 to -52.8)	8.31 (7.58 to 9.09)	3.35 (3.07 to 3.64)	-59.6 (-62.4 to -56.9)
		Fungus	7820 (5950 to 10000)	3330 (2560 to 4240)	-57.3 (-60.0 to -54.4)	9.88 (8.78 to 11)	3.8 (3.41 to 4.22)	-61.5 (-64.4 to -58.8)
		Group B Streptococcus	8190 (6230 to 10300)	3820 (2950 to 4860)	-53.3 (-56.6 to -50.6)	7.43 (6.66 to 8.33)	3.13 (2.82 to 3.45)	-57.9 (-60.9 to -55.1)
		Haemophilus influenzae	33200 (26100 to 42400)	14600 (11500 to 18500)	-55.9 (-58.6 to -53.5)	16.2 (15.2 to 17.2)	5.29 (4.93 to 5.65)	-67.3 (-69.6 to -65.1)
		Influenza	47100 (37000 to 59700)	3290 (1500 to 6360)	-93.0 (-96.7 to -87.1)	44.9 (42.8 to 46.9)	2.61 (1.06 to 5.18)	-94.2 (-97.6 to -88.3)

		Klebsiella pneumoniae	19100 (15000 to 24500)	7540 (5920 to 9570)	-60.5 (-62.7 to -58.4)	24.3 (23.1 to 25.5)	8.67 (8.12 to 9.21)	-64.3 (-66.6 to -62.1)
		Legionella spp.	16500 (12700 to 21000)	9090 (7120 to 11500)	-44.8 (-48.2 to -41.5)	14.4 (13 to 15.9)	7.16 (6.53 to 7.78)	-50.1 (-53.5 to -46.7)
		Mycoplasma spp.	73500 (57000 to 93700)	30600 (23800 to 38900)	-58.4 (-60.8 to -56.3)	24.8 (23.3 to 26.3)	9.33 (8.7 to 9.93)	-62.4 (-64.9 to -60.1)
		Polymicrobial	1090 (682 to 1700)	406 (241 to 647)	-63.0 (-66.3 to -59.8)	1.42 (0.92 to 2.16)	0.48 (0.29 to 0.76)	-66.6 (-69.6 to -63.3)
		Pseudomonas aeruginosa	18100 (14200 to 22900)	10100 (7890 to 12800)	-44.2 (-47.5 to -41.3)	15.9 (14.9 to 17)	8 (7.45 to 8.55)	-49.8 (-53.1 to -46.7)
		Respiratory syncytial virus	12800 (10000 to 16400)	1040 (482 to 2000)	-91.9 (-96.1 to -85.3)	5.54 (5.15 to 5.97)	0.37 (0.15 to 0.72)	-93.3 (-97.2 to -86.8)
		Staphylococcus aureus	28100 (22100 to 35700)	15100 (11900 to 19300)	-46.3 (-49.3 to -43.7)	50.7 (48.8 to 52.6)	24.6 (23 to 25.9)	-51.6 (-54.7 to -48.7)
		Streptococcus pneumoniae	266000 (211000 to 340000)	91100 (71700 to 115000)	-65.7 (-67.7 to -64.1)	200 (194 to 205)	61.9 (58.1 to 65.2)	-69.0 (-71.1 to -67.3)
		Other viruses	124000 (97500 to 157000)	63100 (49400 to 79900)	-48.9 (-51.9 to -46.3)	36.4 (35.2 to 37.7)	16.8 (15.8 to 17.7)	-53.8 (-56.9 to -51.2)
		Other bacterial pathogens	28200 (22000 to 36200)	10500 (8110 to 13400)	-62.8 (-65.1 to -60.9)	20.2 (18.3 to 22.2)	6.82 (6.04 to 7.66)	-66.3 (-68.5 to -64.2)
	15-49 years	*Acinetobacter baumannii*	8000 (6720 to 9410)	4960 (4180 to 5750)	-38.0 (-41.7 to -33.7)	60.7 (52.6 to 69.6)	85.9 (70.9 to 104)	41.5 (22.9 to 61.1)
		Chlamydia spp.	37100 (32900 to 41900)	28300 (25200 to 31600)	-23.7 (-27.8 to -18.6)	69.8 (63.9 to 76.8)	120 (104 to 136)	71.9 (52.7 to 91.7)
		Enterobacter spp.	3500 (2970 to 4060)	4350 (3760 to 4970)	24.5 (16.1 to 33.9)	25.1 (21.9 to 28.5)	70.2 (59.3 to 81.4)	180.7 (145.9 to 218.1)
		Escherichia coli	8970 (7920 to 10200)	9630 (8630 to 10700)	7.4 (1.1 to 14.5)	66.5 (60.7 to 72.8)	161 (142 to 182)	141.8 (115.5 to 167.6)
		Fungus	10800 (9310 to 12400)	11100 (9870 to 12500)	2.9 (-3.4 to 10.1)	79.1 (70.1 to 88.1)	183 (158 to 210)	132.1 (105.7 to 160.8)
		Group B Streptococcus	11300 (9930 to 13100)	12700 (11300 to 14300)	12.4 (5.4 to 19.6)	59.5 (53.4 to 66.6)	151 (131 to 172)	153.6 (124.1 to 184.9)
		Haemophilus influenzae	45900 (41200 to 50900)	48400 (44000 to 53200)	5.6 (0.1 to 12.2)	130 (122 to 138)	250 (225 to 277)	92.7 (73.7 to 113.1)
		Influenza	65000 (59200 to 72200)	10200 (4790 to 19200)	-84.2 (-92.8 to -70.5)	360 (345 to 375)	124 (56.4 to 238)	-65.4 (-84.3 to -34.4)
		Klebsiella pneumoniae	26300 (23800 to 29000)	25100 (23000 to 27500)	-4.6 (-9.3 to 1.2)	194 (185 to 204)	419 (374 to 468)	115.7 (91.4 to 141.1)
		Legionella spp.	22800 (20000 to 25700)	30000 (27100 to 33200)	31.9 (24.3 to 41.3)	116 (105 to 127)	343 (304 to 387)	196.8 (163.5 to 230.9)
		Mycoplasma spp.	102000 (92000 to 112000)	101000 (92300 to 111000)	-0.1 (-5.3 to 5.9)	199 (187 to 211)	447 (398 to 499)	125.1 (100.2 to 151.1)
		Polymicrobial	1510 (957 to 2280)	1360 (825 to 2180)	-10.6 (-18.4 to -2.2)	11.4 (7.34 to 17.3)	23.1 (14.1 to 37.1)	101.8 (77.1 to 128.2)
		Pseudomonas aeruginosa	25100 (22500 to 27900)	33500 (30400 to 36800)	33.9 (26.6 to 42.3)	128 (120 to 136)	387 (342 to 435)	202.5 (168.5 to 238.6)
		Respiratory syncytial virus	17700 (15800 to 19700)	3210 (1510 to 5910)	-81.9 (-91.6 to -66.5)	44.6 (41.5 to 48)	17.8 (8.26 to 33.7)	-60.0 (-81.4 to -25.0)

		Staphylococcus aureus	38900 (35300 to 42700)	50200 (45900 to 54700)	29.1 (22.6 to 36.5)	407 (391 to 422)	1180 (1060 to 1320)	191.0 (159.5 to 223.7)
		Streptococcus pneumoniae	367000 (336000 to 402000)	303000 (279000 to 329000)	-17.5 (-21.5 to -12.7)	1600 (1560 to 1640)	2990 (2680 to 3320)	86.4 (66.0 to 107.7)
		Other viruses	171000 (156000 to 187000)	209000 (192000 to 228000)	22.4 (16.8 to 29.2)	292 (283 to 303)	807 (724 to 897)	176.0 (146.0 to 206.9)
		Other bacterial pathogens	38900 (33800 to 44200)	34800 (30200 to 39500)	-10.5 (-15.2 to -4.8)	162 (147 to 178)	327 (280 to 376)	101.8 (79.9 to 125.0)
	50-69 years	*Acinetobacter baumannii*	30000 (25800 to 34600)	16600 (14200 to 19000)	-44.8 (-47.7 to -41.7)	182 (161 to 205)	218 (185 to 257)	19.8 (5.6 to 35.1)
		Chlamydia spp.	82300 (73500 to 92200)	55800 (50400 to 62400)	-32.1 (-35.2 to -29.1)	123 (113 to 135)	184 (163 to 206)	49.2 (33.5 to 66.0)
		Enterobacter spp.	12700 (11000 to 14800)	14200 (12500 to 16100)	11.6 (4.8 to 18.6)	72.7 (63.7 to 82.3)	178 (154 to 204)	145.1 (116.9 to 175.2)
		Escherichia coli	36100 (31900 to 40500)	34500 (31100 to 38400)	-4.2 (-8.9 to 0.4)	213 (196 to 232)	449 (400 to 501)	110.8 (88.8 to 134.5)
		Fungus	34600 (30200 to 39400)	31800 (28200 to 35800)	-7.9 (-12.8 to -2.6)	202 (180 to 225)	407 (353 to 464)	102.2 (80.6 to 125.0)
		Group B Streptococcus	29100 (25500 to 33100)	29200 (26000 to 32700)	0.3 (-5.0 to 5.2)	122 (110 to 136)	269 (236 to 303)	120.3 (96.6 to 146.1)
		Haemophilus influenzae	101000 (91600 to 111000)	96300 (88000 to 105000)	-4.5 (-8.6 to -0.1)	227 (215 to 241)	392 (353 to 433)	72.8 (55.3 to 91.5)
		Influenza	123000 (113000 to 135000)	18200 (8660 to 33700)	-85.2 (-93.1 to -72.4)	543 (518 to 566)	175 (76.9 to 342)	-67.7 (-85.9 to -37.6)
		Klebsiella pneumoniae	74200 (68100 to 80800)	64400 (59100 to 70500)	-13.2 (-16.6 to -9.6)	436 (415 to 457)	829 (745 to 924)	90.3 (70.3 to 112.6)
		Legionella spp.	70400 (62300 to 78600)	83300 (75800 to 92100)	18.3 (12.5 to 24.6)	284 (260 to 311)	741 (660 to 834)	161.0 (134.1 to 189.4)
		Mycoplasma spp.	102000 (92600 to 112000)	90000 (82100 to 99200)	-12.0 (-15.6 to -8.3)	160 (149 to 170)	309 (275 to 344)	93.9 (74.5 to 115.2)
		Polymicrobial	4720 (3070 to 7190)	3800 (2340 to 6010)	-19.9 (-26.1 to -13.0)	28.3 (18.5 to 43.2)	49.9 (30.4 to 81.3)	75.6 (53.6 to 98.5)
		Pseudomonas aeruginosa	78000 (70600 to 86400)	93000 (84900 to 102000)	19.2 (14.2 to 24.6)	318 (299 to 337)	831 (739 to 928)	161.6 (134.6 to 190.6)
		Respiratory syncytial virus	24800 (22000 to 27600)	4180 (2000 to 7690)	-83.1 (-92.0 to -68.8)	49.7 (45.2 to 54.3)	18.2 (8.11 to 34.4)	-63.3 (-83.5 to -30.2)
		Staphylococcus aureus	107000 (97600 to 116000)	124000 (113000 to 135000)	16.0 (11.4 to 20.6)	889 (858 to 922)	2260 (2030 to 2500)	154.8 (128.9 to 182.6)
		Streptococcus pneumoniae	497000 (457000 to 540000)	370000 (340000 to 405000)	-25.5 (-28.4 to -22.7)	1730 (1680 to 1770)	2830 (2540 to 3140)	63.6 (46.5 to 81.7)
		Other viruses	202000 (186000 to 219000)	225000 (207000 to 247000)	11.5 (7.4 to 15.8)	276 (266 to 286)	676 (608 to 749)	145.5 (120.3 to 172.0)
		Other bacterial pathogens	129000 (114000 to 145000)	103000 (90500 to 117000)	-20.1 (-23.7 to -16.6)	429 (389 to 468)	755 (649 to 866)	76.1 (58.0 to 95.9)
	70+ years	*Acinetobacter baumannii*	18600 (15800 to 21700)	12500 (10700 to 14500)	-32.8 (-36.5 to -28.7)	108 (94.7 to 123)	155 (132 to 177)	43.4 (31.9 to 55.7)
		Chlamydia spp.	61000 (53800 to 69000)	50400 (45100 to 56800)	-17.4 (-21.8 to -12.9)	87.8 (79.9 to 95.6)	163 (145 to 180)	85.3 (71.2 to 99.6)

		Enterobacter spp.	7750 (6520 to 9100)	10500 (9160 to 12100)	36.1 (26.9 to 46.0)	42.3 (36.5 to 48.5)	129 (113 to 147)	205.7 (179.4 to 232.7)
		Escherichia coli	27600 (24200 to 31800)	32300 (28900 to 36600)	16.9 (10.5 to 23.8)	156 (143 to 171)	413 (371 to 454)	163.8 (143.2 to 184.0)
		Fungus	12000 (10300 to 14000)	13500 (11900 to 15400)	12.3 (5.5 to 19.7)	67.1 (59.4 to 75.6)	168 (148 to 190)	151.1 (131.3 to 172.0)
		Group B Streptococcus	16700 (14700 to 19100)	20400 (18200 to 23000)	22.3 (15.2 to 29.6)	67 (59.9 to 74.8)	183 (163 to 205)	173.8 (152.5 to 196.0)
		Haemophilus influenzae	56800 (51100 to 63800)	66000 (59900 to 73800)	16.2 (10.3 to 22.6)	122 (114 to 130)	267 (241 to 289)	118.2 (102.0 to 135.4)
		Influenza	117000 (106000 to 130000)	21600 (10200 to 39300)	-81.5 (-91.1 to -66.0)	493 (468 to 516)	205 (82.7 to 407)	-58.4 (-83.5 to -17.3)
		Klebsiella pneumoniae	51000 (46100 to 57000)	53800 (49000 to 59900)	5.7 (0.8 to 11.2)	287 (271 to 302)	674 (610 to 727)	134.6 (117.6 to 151.6)
		Legionella spp.	31800 (28000 to 36100)	45600 (41000 to 51200)	43.6 (35.7 to 52.3)	122 (111 to 135)	400 (359 to 442)	226.8 (201.8 to 252.8)
		Mycoplasma spp.	54100 (48400 to 61000)	57800 (52100 to 65300)	6.9 (1.7 to 12.6)	80.7 (74.1 to 86.6)	195 (174 to 214)	141.3 (123.2 to 159.5)
		Polymicrobial	3360 (2160 to 5320)	3310 (1990 to 5470)	-2.1 (-10.2 to 6.8)	19.4 (12.4 to 30.2)	42.4 (25.7 to 70.6)	117.7 (95.7 to 139.1)
		Pseudomonas aeruginosa	50900 (46000 to 57200)	73700 (67100 to 82500)	44.9 (37.9 to 52.9)	198 (185 to 211)	642 (578 to 700)	224.3 (200.4 to 248.1)
		Respiratory syncytial virus	14600 (12800 to 16600)	3040 (1480 to 5480)	-79.1 (-89.7 to -61.8)	27.9 (25.5 to 30.4)	12.9 (5.19 to 25.4)	-53.7 (-81.3 to -8.5)
		Staphylococcus aureus	86200 (78000 to 95200)	122000 (111000 to 136000)	41.5 (35.2 to 48.5)	688 (650 to 718)	2190 (1990 to 2370)	217.9 (196.0 to 240.4)
		Streptococcus pneumoniae	319000 (290000 to 355000)	288000 (263000 to 322000)	-9.8 (-13.7 to -5.4)	1060 (1010 to 1100)	2140 (1950 to 2310)	101.1 (86.8 to 115.5)
		Other viruses	129000 (116000 to 144000)	174000 (159000 to 195000)	35.2 (29.3 to 41.6)	168 (158 to 175)	512 (462 to 554)	204.8 (183.4 to 227.1)
		Other bacterial pathogens	108000 (94000 to 123000)	105000 (92000 to 120000)	-2.7 (-7.5 to 2.8)	347 (312 to 381)	760 (658 to 863)	119.1 (102.5 to 135.8)
Belarus	All Ages	*Acinetobacter baumannii*	2950 (2560 to 3370)	1280 (1070 to 1500)	-56.6 (-61.3 to -51.7)	18.1 (15.1 to 22.6)	11.8 (9.35 to 14.8)	-34.1 (-49.3 to -15.3)
		Chlamydia spp.	11300 (10300 to 12500)	5610 (4980 to 6280)	-50.3 (-55.2 to -45.2)	16.9 (15.3 to 18.5)	12.5 (9.94 to 15.4)	-26.0 (-40.2 to -10.1)
		Enterobacter spp.	1440 (1250 to 1650)	1180 (1010 to 1360)	-17.9 (-26.8 to -8.3)	8.53 (7.09 to 10.5)	10.2 (8 to 12.8)	20.1 (-9.4 to 56.6)
		Escherichia coli	4340 (3910 to 4800)	2940 (2610 to 3310)	-32.3 (-39.1 to -24.5)	26 (23.5 to 28.7)	25.2 (20.3 to 31)	-3.1 (-21.6 to 18.5)
		Fungus	3170 (2800 to 3600)	2240 (1960 to 2560)	-29.1 (-36.6 to -21.5)	13.4 (11.7 to 15.1)	21.2 (16.6 to 26.7)	59.2 (24.0 to 99.5)
		Group B Streptococcus	3580 (3210 to 4020)	2570 (2260 to 2910)	-28.2 (-35.7 to -20.3)	17.9 (16 to 20)	16.6 (13.3 to 20.6)	-7.2 (-25.2 to 13.3)
		Haemophilus influenzae	15800 (14400 to 17300)	9960 (8910 to 11100)	-37.0 (-43.4 to -29.9)	35.4 (32 to 39.4)	30.8 (24.6 to 38)	-12.9 (-29.5 to 5.8)
		Influenza	21000 (19500 to 22700)	5290 (1410 to 12000)	-74.9 (-93.5 to -42.7)	79.2 (73.2 to 85.6)	33.4 (8.52 to 82.1)	-57.8 (-89.2 to 2.8)

		Klebsiella pneumoniae	10200 (9490 to 11100)	6230 (5600 to 6920)	-39.1 (-45.3 to -32.7)	55.6 (50.9 to 60.5)	53.4 (42.6 to 65.9)	-4.0 (-21.9 to 17.3)
		Legionella spp.	7080 (6360 to 7860)	7330 (6510 to 8220)	3.6 (-6.6 to 14.6)	24 (21.4 to 26.8)	47.2 (37.4 to 58.9)	96.4 (55.5 to 144.5)
		Mycoplasma spp.	18700 (17000 to 20600)	12100 (10900 to 13500)	-35.1 (-41.5 to -29.1)	23.1 (20.9 to 25)	29.8 (23.8 to 36.9)	29.2 (2.9 to 59.2)
		Polymicrobial	554 (363 to 845)	310 (187 to 506)	-44.3 (-51.5 to -36.5)	8.43 (5.35 to 12.6)	3.09 (1.93 to 4.92)	-62.4 (-77.3 to -40.4)
		Pseudomonas aeruginosa	9660 (8850 to 10500)	9140 (8180 to 10200)	-5.3 (-14.4 to 4.7)	37.1 (33.9 to 40.4)	55.5 (44.2 to 68.4)	49.6 (20.7 to 82.6)
		Respiratory syncytial virus	12300 (10600 to 14100)	2240 (601 to 5120)	-81.7 (-95.2 to -59.3)	41.4 (35.5 to 48.2)	5.38 (1.37 to 13.4)	-86.9 (-96.7 to -68.6)
		Staphylococcus aureus	14300 (13300 to 15300)	11900 (10800 to 13300)	-16.4 (-24.4 to -7.8)	97.4 (90.2 to 104)	145 (115 to 177)	48.5 (19.2 to 81.7)
		Streptococcus pneumoniae	94800 (87500 to 103000)	52700 (47700 to 58700)	-44.4 (-49.6 to -38.8)	265 (244 to 287)	271 (216 to 335)	2.1 (-17.2 to 24.3)
		Other viruses	50000 (45600 to 55300)	35000 (31400 to 39000)	-30.0 (-37.4 to -22.4)	62.6 (56.4 to 68.8)	68.2 (54.9 to 84.1)	9.1 (-11.1 to 32.6)
		Other bacterial pathogens	15300 (13600 to 17100)	10100 (8640 to 11700)	-34.4 (-40.9 to -27.5)	40.3 (35.7 to 45.2)	48.7 (38.4 to 60.8)	21.0 (-2.9 to 49.6)
	Under 5	*Acinetobacter baumannii*	80.2 (57.4 to 111)	9.31 (6.29 to 13.3)	-88.4 (-90.7 to -85.7)	5.52 (3 to 9.57)	0.23 (0.12 to 0.41)	-95.8 (-97.1 to -94.1)
		Chlamydia spp.	1410 (1140 to 1720)	206 (158 to 264)	-85.3 (-88.3 to -81.7)	6.45 (5.37 to 7.78)	0.29 (0.22 to 0.39)	-95.4 (-96.7 to -93.6)
		Enterobacter spp.	153 (114 to 205)	35 (24.6 to 48.4)	-77.1 (-81.8 to -71.2)	3.22 (2.05 to 4.97)	0.22 (0.14 to 0.33)	-93.2 (-95.2 to -90.3)
		Escherichia coli	521 (412 to 649)	101 (77.2 to 130)	-80.6 (-84.6 to -75.8)	9.51 (7.59 to 11.6)	0.56 (0.42 to 0.75)	-94.1 (-95.7 to -91.8)
		Fungus	110 (75.5 to 155)	20.7 (14.2 to 29.4)	-81.0 (-84.8 to -76.1)	0.69 (0.47 to 0.96)	0.05 (0.03 to 0.08)	-92.7 (-94.9 to -89.5)
		Group B Streptococcus	432 (340 to 548)	89.6 (67.5 to 118)	-79.2 (-83.4 to -73.9)	8.59 (7.13 to 10.4)	0.48 (0.36 to 0.64)	-94.3 (-96.0 to -92.0)
		Haemophilus influenzae	4310 (3490 to 5300)	880 (685 to 1120)	-79.5 (-83.8 to -74.3)	17.3 (14.2 to 20.7)	1 (0.73 to 1.36)	-94.2 (-96.0 to -91.8)
		Influenza	3600 (2940 to 4330)	265 (71.3 to 595)	-92.6 (-98.0 to -84.1)	25.2 (20.9 to 30.1)	0.62 (0.16 to 1.46)	-97.5 (-99.4 to -94.0)
		Klebsiella pneumoniae	2030 (1660 to 2480)	357 (279 to 451)	-82.3 (-85.8 to -78.0)	21.1 (17.6 to 25.2)	1.24 (0.91 to 1.71)	-94.1 (-95.7 to -91.7)
		Legionella spp.	174 (116 to 265)	46.9 (29.9 to 70.8)	-72.9 (-78.6 to -66.3)	4.17 (3 to 5.67)	0.28 (0.2 to 0.39)	-93.0 (-95.1 to -90.3)
		Mycoplasma spp.	2450 (1980 to 2990)	538 (413 to 688)	-77.9 (-82.6 to -72.4)	6.54 (5.38 to 7.87)	0.44 (0.33 to 0.6)	-93.2 (-95.2 to -90.3)
		Polymicrobial	46.6 (28.8 to 71.4)	7.95 (4.58 to 13.2)	-83.0 (-86.6 to -78.7)	6.24 (3.31 to 10.4)	0.38 (0.18 to 0.67)	-93.9 (-95.7 to -91.4)
		Pseudomonas aeruginosa	1180 (955 to 1440)	328 (254 to 425)	-72.1 (-77.7 to -65.0)	12.3 (10.3 to 14.7)	0.97 (0.72 to 1.31)	-92.0 (-94.3 to -88.8)
		Respiratory syncytial virus	8780 (7160 to 10500)	923 (257 to 2060)	-89.4 (-97.2 to -77.4)	36.7 (30.7 to 43.6)	1.15 (0.3 to 2.72)	-96.8 (-99.2 to -92.4)

		Staphylococcus aureus	1500 (1230 to 1800)	352 (278 to 448)	-76.4 (-81.3 to -70.5)	20.9 (17.5 to 24.9)	1.65 (1.22 to 2.25)	-92.1 (-94.4 to -88.9)
		Streptococcus pneumoniae	23900 (19600 to 28400)	4270 (3330 to 5400)	-82.1 (-85.8 to -77.5)	99.5 (84 to 117)	6.67 (4.88 to 9.22)	-93.2 (-95.2 to -90.4)
		Other viruses	18700 (15300 to 22300)	4780 (3740 to 6020)	-74.3 (-79.8 to -67.8)	34.1 (28.9 to 39.9)	3.09 (2.28 to 4.24)	-90.9 (-93.5 to -87.1)
		Other bacterial pathogens	607 (461 to 783)	107 (77.6 to 144)	-82.3 (-85.9 to -77.9)	4.67 (3.34 to 6.75)	0.26 (0.17 to 0.38)	-94.3 (-96.0 to -91.9)
	5-14 years	*Acinetobacter baumannii*	298 (221 to 391)	74.2 (53.1 to 99.4)	-75.0 (-78.2 to -71.3)	0.2 (0.17 to 0.25)	0.03 (0.02 to 0.03)	-86.1 (-88.8 to -82.8)
		Chlamydia spp.	1420 (1070 to 1840)	453 (332 to 598)	-68.0 (-71.8 to -63.6)	0.24 (0.2 to 0.28)	0.04 (0.04 to 0.05)	-82.2 (-85.5 to -78.0)
		Enterobacter spp.	138 (102 to 184)	69.3 (50.4 to 93.6)	-49.5 (-56.5 to -41.9)	0.09 (0.07 to 0.11)	0.03 (0.02 to 0.03)	-72.0 (-77.3 to -64.9)
		Escherichia coli	348 (261 to 453)	145 (106 to 191)	-58.2 (-63.4 to -52.0)	0.23 (0.2 to 0.28)	0.05 (0.05 to 0.06)	-76.8 (-81.1 to -71.7)
		Fungus	419 (312 to 545)	174 (128 to 233)	-58.3 (-63.6 to -51.7)	0.28 (0.23 to 0.34)	0.06 (0.05 to 0.08)	-76.9 (-81.1 to -71.6)
		Group B Streptococcus	442 (327 to 572)	202 (146 to 266)	-54.2 (-59.9 to -47.9)	0.21 (0.17 to 0.25)	0.05 (0.04 to 0.06)	-74.6 (-79.4 to -68.8)
		Haemophilus influenzae	1800 (1370 to 2350)	833 (616 to 1090)	-53.8 (-59.4 to -47.0)	0.47 (0.4 to 0.54)	0.11 (0.09 to 0.13)	-76.1 (-80.6 to -70.4)
		Influenza	2550 (1950 to 3290)	432 (111 to 1010)	-83.0 (-95.7 to -60.2)	1.28 (1.1 to 1.48)	0.12 (0.03 to 0.29)	-90.5 (-97.6 to -77.1)
		Klebsiella pneumoniae	1020 (774 to 1320)	417 (308 to 546)	-59.2 (-64.0 to -53.0)	0.69 (0.59 to 0.79)	0.16 (0.13 to 0.18)	-77.3 (-81.5 to -72.1)
		Legionella spp.	912 (689 to 1190)	552 (412 to 719)	-39.5 (-46.7 to -30.7)	0.42 (0.36 to 0.5)	0.14 (0.12 to 0.17)	-66.4 (-72.6 to -58.7)
		Mycoplasma spp.	3980 (3010 to 5180)	1800 (1330 to 2350)	-54.7 (-60.2 to -48.1)	0.71 (0.61 to 0.82)	0.18 (0.15 to 0.21)	-74.8 (-79.5 to -69.0)
		Polymicrobial	57.6 (35.2 to 89.5)	19.6 (11.2 to 32.5)	-66.2 (-71.1 to -60.7)	0.04 (0.02 to 0.06)	0.01 (0 to 0.01)	-81.2 (-85.1 to -76.5)
		Pseudomonas aeruginosa	998 (757 to 1280)	594 (439 to 776)	-40.5 (-47.7 to -31.9)	0.47 (0.4 to 0.54)	0.15 (0.13 to 0.18)	-66.9 (-72.9 to -59.4)
		Respiratory syncytial virus	709 (542 to 924)	164 (41.6 to 384)	-76.9 (-94.0 to -45.8)	0.16 (0.14 to 0.19)	0.02 (0.01 to 0.05)	-87.1 (-96.7 to -68.8)
		Staphylococcus aureus	1530 (1170 to 1970)	797 (589 to 1030)	-47.9 (-54.1 to -40.7)	1.46 (1.26 to 1.67)	0.42 (0.35 to 0.49)	-71.1 (-76.3 to -64.3)
		Streptococcus pneumoniae	14400 (11000 to 18500)	5990 (4420 to 7790)	-58.3 (-63.2 to -52.4)	5.7 (4.91 to 6.56)	1.32 (1.11 to 1.54)	-76.8 (-81.1 to -71.5)
		Other viruses	6830 (5230 to 8800)	4060 (3000 to 5260)	-40.6 (-47.5 to -32.3)	1.07 (0.92 to 1.22)	0.35 (0.29 to 0.41)	-67.0 (-73.1 to -59.6)
		Other bacterial pathogens	1510 (1140 to 1950)	578 (427 to 772)	-61.6 (-66.1 to -55.7)	0.57 (0.48 to 0.68)	0.12 (0.1 to 0.14)	-78.6 (-82.6 to -73.8)
	15-49 years	*Acinetobacter baumannii*	368 (308 to 440)	141 (116 to 167)	-61.7 (-66.0 to -57.4)	1.97 (1.65 to 2.33)	2.25 (1.71 to 2.94)	14.7 (-12.4 to 50.6)
		Chlamydia spp.	1750 (1510 to 2000)	857 (747 to 981)	-50.9 (-56.2 to -45.7)	2.32 (1.99 to 2.67)	3.4 (2.62 to 4.34)	47.3 (12.1 to 90.9)

		Enterobacter spp.	170 (142 to 202)	131 (110 to 154)	-22.6 (-32.2 to -12.8)	0.86 (0.71 to 1.03)	1.99 (1.48 to 2.61)	132.1 (75.3 to 204.3)
		Escherichia coli	430 (373 to 498)	275 (242 to 314)	-35.8 (-43.2 to -28.3)	2.25 (1.94 to 2.6)	4.32 (3.33 to 5.5)	92.4 (47.2 to 150.6)
		Fungus	518 (439 to 606)	330 (288 to 386)	-36.1 (-43.2 to -28.9)	2.68 (2.27 to 3.13)	5.1 (3.85 to 6.59)	91.5 (46.5 to 151.5)
		Group B Streptococcus	545 (470 to 633)	382 (332 to 439)	-29.8 (-37.3 to -22.3)	2.03 (1.73 to 2.38)	4.26 (3.24 to 5.5)	110.5 (60.7 to 173.9)
		Haemophilus influenzae	2230 (1960 to 2510)	1580 (1390 to 1770)	-29.1 (-36.8 to -21.1)	4.45 (3.9 to 5.06)	8.78 (6.72 to 11.3)	97.9 (51.0 to 158.0)
		Influenza	3150 (2780 to 3530)	819 (212 to 1860)	-74.0 (-93.2 to -39.9)	12.3 (10.9 to 13.9)	9.66 (2.44 to 23.5)	-21.2 (-80.2 to 91.7)
		Klebsiella pneumoniae	1260 (1120 to 1410)	789 (700 to 882)	-37.4 (-44.2 to -30.5)	6.57 (5.81 to 7.45)	12.3 (9.49 to 15.7)	87.7 (44.4 to 143.4)
		Legionella spp.	1130 (978 to 1290)	1040 (915 to 1180)	-7.1 (-16.9 to 3.3)	4.04 (3.5 to 4.67)	11.2 (8.68 to 14.3)	178.3 (111.6 to 264.0)
		Mycoplasma spp.	4920 (4290 to 5570)	3410 (3030 to 3850)	-30.5 (-37.8 to -22.8)	6.81 (5.97 to 7.74)	14.1 (11.1 to 18.1)	108.5 (60.0 to 172.9)
		Polymicrobial	71.2 (44.4 to 108)	37.1 (21.7 to 60.8)	-48.1 (-55.0 to -41.0)	0.38 (0.24 to 0.57)	0.59 (0.34 to 1.01)	55.5 (16.3 to 104.7)
		Pseudomonas aeruginosa	1230 (1080 to 1390)	1120 (994 to 1280)	-8.7 (-18.8 to 1.0)	4.46 (3.89 to 5.09)	12.2 (9.48 to 15.6)	173.7 (109.3 to 259.0)
		Respiratory syncytial virus	876 (760 to 977)	310 (78.1 to 712)	-64.6 (-90.7 to -18.5)	1.56 (1.36 to 1.8)	1.67 (0.42 to 4.05)	7.2 (-73.0 to 160.5)
		Staphylococcus aureus	1890 (1670 to 2120)	1510 (1350 to 1680)	-20.1 (-28.6 to -11.7)	14 (12.4 to 15.7)	33.4 (25.9 to 42.5)	139.6 (82.8 to 213.7)
		Streptococcus pneumoniae	17700 (15700 to 19800)	11300 (10100 to 12600)	-36.0 (-43.0 to -29.4)	54.6 (48.4 to 61.6)	105 (81.4 to 133)	91.9 (47.4 to 149.7)
		Other viruses	8430 (7480 to 9430)	7670 (6860 to 8560)	-8.8 (-18.4 to 1.0)	10.2 (9.01 to 11.6)	27.8 (21.6 to 35.5)	173.2 (110.1 to 256.4)
		Other bacterial pathogens	1860 (1580 to 2140)	1090 (931 to 1280)	-41.1 (-47.5 to -34.6)	5.47 (4.7 to 6.31)	9.64 (7.23 to 12.6)	76.9 (35.7 to 130.6)
	50-69 years	*Acinetobacter baumannii*	1220 (1040 to 1430)	613 (508 to 731)	-49.7 (-55.6 to -42.7)	5.81 (4.98 to 6.83)	6.92 (5.31 to 8.93)	19.4 (-9.2 to 52.6)
		Chlamydia spp.	3430 (3010 to 3910)	2200 (1900 to 2560)	-35.7 (-43.2 to -27.4)	4.06 (3.58 to 4.59)	6.18 (4.75 to 7.71)	52.5 (15.4 to 95.7)
		Enterobacter spp.	547 (465 to 635)	554 (468 to 650)	1.6 (-11.4 to 16.0)	2.47 (2.1 to 2.91)	5.93 (4.53 to 7.63)	141.1 (80.5 to 211.4)
		Escherichia coli	1530 (1330 to 1730)	1280 (1120 to 1470)	-16.0 (-25.8 to -4.9)	7.15 (6.25 to 8.09)	14.2 (11 to 17.8)	99.4 (52.9 to 156.1)
		Fungus	1470 (1270 to 1680)	1230 (1040 to 1430)	-16.1 (-26.2 to -5.1)	6.75 (5.81 to 7.74)	13.4 (10.4 to 17.1)	99.0 (51.1 to 155.6)
		Group B Streptococcus	1240 (1080 to 1430)	1140 (978 to 1330)	-8.0 (-18.9 to 3.7)	4.12 (3.57 to 4.74)	8.97 (6.93 to 11.4)	118.3 (66.0 to 180.5)
		Haemophilus influenzae	4330 (3890 to 4810)	4050 (3560 to 4600)	-6.4 (-17.3 to 6.0)	7.72 (6.92 to 8.59)	15.9 (12.3 to 20)	106.9 (56.7 to 166.8)
		Influenza	5280 (4750 to 5870)	1810 (471 to 4130)	-65.7 (-91.0 to -22.0)	18.4 (16.4 to 20.4)	15 (3.81 to 36.9)	-18.1 (-79.3 to 102.8)

		Klebsiella pneumoniae	3140 (2840 to 3470)	2610 (2290 to 2970)	-17.0 (-26.5 to -6.2)	14.6 (13.1 to 16.2)	28.7 (22.1 to 35.9)	97.0 (50.8 to 153.1)
		Legionella spp.	3080 (2690 to 3490)	3750 (3250 to 4300)	21.9 (7.7 to 37.7)	9.85 (8.59 to 11.2)	28.4 (22.1 to 36.1)	189.2 (118.9 to 268.9)
		Mycoplasma spp.	4390 (3890 to 4890)	3970 (3470 to 4520)	-9.4 (-19.8 to 2.1)	5.41 (4.81 to 6.08)	11.6 (9.02 to 14.5)	115.0 (62.6 to 177.1)
		Polymicrobial	197 (126 to 301)	135 (78.5 to 220)	-31.9 (-41.2 to -21.4)	0.93 (0.6 to 1.43)	1.51 (0.85 to 2.54)	61.7 (21.2 to 107.8)
		Pseudomonas aeruginosa	3400 (3020 to 3790)	4060 (3560 to 4670)	19.5 (6.2 to 34.7)	11 (9.8 to 12.3)	31 (23.9 to 38.9)	183.7 (115.7 to 265.6)
		Respiratory syncytial virus	1090 (951 to 1230)	505 (129 to 1180)	-53.6 (-87.9 to 5.6)	1.73 (1.51 to 1.96)	1.92 (0.48 to 4.73)	11.0 (-72.1 to 178.7)
		Staphylococcus aureus	4590 (4130 to 5050)	4810 (4230 to 5450)	5.0 (-6.4 to 18.3)	30.3 (27.2 to 33.7)	75.2 (58.2 to 94.2)	149.1 (89.6 to 218.9)
		Streptococcus pneumoniae	21200 (19300 to 23200)	17900 (15700 to 20400)	-15.6 (-25.0 to -4.7)	58.3 (52.9 to 64.6)	117 (89.9 to 147)	100.2 (52.4 to 157.5)
		Other viruses	8840 (7960 to 9720)	10700 (9370 to 12100)	20.7 (7.1 to 36.3)	9.55 (8.62 to 10.6)	27.3 (21.1 to 34.4)	186.5 (118.6 to 267.7)
		Other bacterial pathogens	5460 (4770 to 6200)	4220 (3590 to 4970)	-22.7 (-31.2 to -12.8)	14.3 (12.5 to 16.4)	26.3 (20.1 to 33.6)	83.7 (39.6 to 135.4)
	70+ years	*Acinetobacter baumannii*	981 (827 to 1150)	440 (360 to 533)	-55.1 (-61.2 to -48.9)	4.57 (3.84 to 5.35)	2.41 (1.93 to 2.95)	-47.0 (-57.1 to -35.6)
		Chlamydia spp.	3290 (2870 to 3730)	1890 (1600 to 2190)	-42.6 (-50.1 to -34.4)	3.8 (3.29 to 4.33)	2.57 (2.09 to 3.08)	-32.3 (-44.8 to -18.0)
		Enterobacter spp.	429 (361 to 506)	388 (322 to 462)	-9.4 (-22.3 to 3.9)	1.89 (1.58 to 2.22)	2.01 (1.61 to 2.47)	6.9 (-13.9 to 30.7)
		Escherichia coli	1510 (1320 to 1730)	1130 (967 to 1320)	-25.0 (-34.8 to -14.2)	6.89 (5.94 to 7.85)	6.08 (4.95 to 7.28)	-11.5 (-27.9 to 7.0)
		Fungus	657 (559 to 767)	491 (412 to 582)	-25.2 (-34.7 to -14.3)	2.96 (2.49 to 3.44)	2.6 (2.11 to 3.18)	-11.7 (-28.2 to 7.4)
		Group B Streptococcus	918 (794 to 1060)	752 (635 to 884)	-17.9 (-28.7 to -6.5)	2.97 (2.55 to 3.46)	2.87 (2.33 to 3.46)	-3.2 (-21.7 to 17.3)
		Haemophilus influenzae	3140 (2800 to 3520)	2620 (2240 to 3000)	-16.5 (-27.4 to -4.5)	5.47 (4.82 to 6.1)	5.01 (4.12 to 5.99)	-8.2 (-26.0 to 10.8)
		Influenza	6460 (5800 to 7170)	1960 (512 to 4480)	-69.5 (-92.0 to -31.3)	22 (19.5 to 24.4)	7.97 (2.03 to 19.6)	-63.6 (-91.1 to -8.4)
		Klebsiella pneumoniae	2790 (2490 to 3110)	2060 (1760 to 2370)	-25.9 (-35.2 to -16.0)	12.6 (11.2 to 14.1)	11 (9.04 to 13.2)	-12.6 (-29.2 to 5.5)
		Legionella spp.	1780 (1550 to 2030)	1940 (1650 to 2260)	8.7 (-5.4 to 23.9)	5.57 (4.84 to 6.34)	7.12 (5.82 to 8.48)	28.3 (3.3 to 56.2)
		Mycoplasma spp.	2980 (2650 to 3370)	2410 (2070 to 2790)	-19.1 (-29.5 to -7.9)	3.6 (3.11 to 4.06)	3.42 (2.8 to 4.1)	-4.6 (-23.0 to 15.5)
		Polymicrobial	181 (115 to 284)	111 (63.5 to 186)	-39.2 (-48.2 to -29.1)	0.84 (0.53 to 1.31)	0.6 (0.35 to 1.02)	-28.3 (-42.1 to -11.6)
		Pseudomonas aeruginosa	2850 (2530 to 3200)	3030 (2590 to 3500)	6.7 (-6.9 to 21.3)	8.97 (7.89 to 10)	11.3 (9.29 to 13.4)	25.8 (2.1 to 51.4)
		Respiratory syncytial virus	820 (716 to 941)	338 (88.4 to 775)	-58.7 (-89.1 to -6.9)	1.27 (1.11 to 1.45)	0.63 (0.15 to 1.53)	-50.7 (-88.0 to 24.6)

		Staphylococcus aureus	4780 (4300 to 5320)	4470 (3830 to 5140)	-6.3 (-18.3 to 6.2)	30.8 (27.2 to 34.1)	33.9 (27.9 to 40.2)	10.5 (-10.3 to 33.3)
		Streptococcus pneumoniae	17600 (15900 to 19600)	13200 (11400 to 15100)	-24.7 (-34.0 to -14.3)	47.2 (41.7 to 52.1)	41.8 (34.5 to 49.6)	-11.2 (-28.1 to 7.0)
		Other viruses	7240 (6520 to 8040)	7790 (6690 to 8900)	7.7 (-6.0 to 22.2)	7.63 (6.74 to 8.48)	9.68 (7.98 to 11.5)	27.1 (3.0 to 52.9)
		Other bacterial pathogens	5890 (5140 to 6730)	4060 (3350 to 4820)	-31.0 (-40.3 to -21.3)	15.2 (13 to 17.5)	12.4 (9.96 to 15.3)	-18.5 (-33.8 to -1.1)
Estonia	All Ages	*Acinetobacter baumannii*	500 (427 to 573)	268 (230 to 308)	-46.3 (-52.0 to -40.0)	3.21 (2.8 to 3.71)	3.28 (2.77 to 3.82)	2.5 (-14.3 to 20.1)
		Chlamydia spp.	1840 (1660 to 2030)	765 (691 to 836)	-58.4 (-62.3 to -54.1)	2.96 (2.73 to 3.2)	2.25 (1.95 to 2.58)	-23.9 (-35.0 to -12.5)
		Enterobacter spp.	247 (211 to 284)	259 (229 to 290)	4.7 (-6.9 to 17.9)	1.53 (1.33 to 1.74)	2.93 (2.48 to 3.41)	92.2 (61.0 to 125.3)
		Escherichia coli	734 (652 to 821)	567 (519 to 625)	-22.5 (-30.6 to -13.6)	4.61 (4.22 to 5.03)	6.9 (5.9 to 7.79)	50.0 (26.9 to 71.8)
		Fungus	558 (490 to 631)	405 (362 to 450)	-27.3 (-34.8 to -19.3)	3.03 (2.7 to 3.4)	4.29 (3.68 to 4.89)	41.9 (20.4 to 63.8)
		Group B Streptococcus	602 (534 to 679)	515 (467 to 562)	-14.3 (-23.6 to -4.1)	2.98 (2.71 to 3.26)	4.14 (3.6 to 4.68)	39.3 (18.8 to 59.7)
		Haemophilus influenzae	2510 (2290 to 2730)	1240 (1140 to 1350)	-50.4 (-54.6 to -46.0)	6.15 (5.73 to 6.59)	3.38 (2.93 to 3.8)	-45.0 (-52.5 to -37.4)
		Influenza	3420 (3140 to 3700)	73.3 (15.9 to 198)	-97.9 (-99.5 to -94.2)	14.9 (14.1 to 15.9)	0.64 (0.14 to 1.78)	-95.7 (-99.1 to -88.2)
		Klebsiella pneumoniae	1660 (1530 to 1810)	1170 (1070 to 1270)	-29.6 (-35.4 to -23.3)	10.1 (9.45 to 10.7)	13.6 (11.8 to 15.2)	34.6 (16.5 to 52.7)
		Legionella spp.	1280 (1140 to 1420)	1190 (1090 to 1290)	-6.8 (-16.7 to 4.0)	5.09 (4.62 to 5.67)	9.11 (7.89 to 10.3)	79.3 (51.7 to 108.4)
		Mycoplasma spp.	3010 (2740 to 3290)	1610 (1490 to 1740)	-46.5 (-50.8 to -42.1)	4.67 (4.33 to 5.05)	4.14 (3.61 to 4.66)	-11.3 (-23.7 to 1.4)
		Polymicrobial	93 (61.1 to 143)	61.9 (36.8 to 101)	-33.9 (-43.2 to -25.0)	1.06 (0.72 to 1.53)	0.8 (0.5 to 1.28)	-24.1 (-50.7 to 6.1)
		Pseudomonas aeruginosa	1660 (1500 to 1820)	2020 (1850 to 2200)	21.8 (11.3 to 33.5)	7.01 (6.54 to 7.49)	16.2 (14 to 18.3)	131.7 (100.8 to 165.8)
		Respiratory syncytial virus	1610 (1420 to 1840)	30.6 (6.5 to 81.4)	-98.1 (-99.6 to -94.9)	5.25 (4.82 to 5.74)	0.08 (0.02 to 0.22)	-98.4 (-99.7 to -95.6)
		Staphylococcus aureus	2430 (2220 to 2630)	2790 (2570 to 3020)	15.0 (6.7 to 24.3)	19.5 (18.3 to 20.8)	47.2 (41 to 53.1)	141.8 (109.9 to 174.3)
		Streptococcus pneumoniae	14800 (13600 to 15900)	6380 (5910 to 6870)	-56.7 (-59.5 to -53.7)	50.8 (47.9 to 53.7)	39.1 (33.9 to 44)	-22.9 (-32.7 to -13.2)
		Other viruses	7540 (6930 to 8260)	5360 (4970 to 5780)	-28.8 (-33.8 to -23.2)	11 (10.3 to 11.7)	12.4 (10.8 to 14)	13.4 (-1.4 to 27.8)
		Other bacterial pathogens	2650 (2320 to 2980)	1550 (1340 to 1760)	-41.5 (-46.9 to -35.9)	8.27 (7.44 to 9.18)	10.8 (9.01 to 12.9)	30.6 (12.2 to 49.4)
	Under 5	*Acinetobacter baumannii*	8.61 (6.16 to 11.9)	1.53 (1.1 to 2.11)	-82.2 (-84.9 to -79.5)	0.51 (0.29 to 0.86)	0.03 (0.02 to 0.06)	-93.3 (-94.8 to -91.5)
		Chlamydia spp.	154 (126 to 187)	25.8 (21.1 to 31.7)	-83.2 (-85.8 to -80.3)	0.66 (0.57 to 0.75)	0.04 (0.03 to 0.05)	-93.9 (-95.1 to -92.5)

		Enterobacter spp.	17.2 (12.8 to 22.7)	6.43 (4.69 to 8.72)	-62.4 (-68.6 to -55.0)	0.33 (0.23 to 0.48)	0.04 (0.02 to 0.05)	-88.8 (-91.2 to -85.8)
		Escherichia coli	57.8 (46.7 to 71.1)	16.4 (13.2 to 20.1)	-71.5 (-76.3 to -66.5)	0.98 (0.85 to 1.13)	0.09 (0.07 to 0.11)	-91.0 (-92.8 to -88.8)
		Fungus	12.1 (8.31 to 17)	3.34 (2.3 to 4.6)	-72.4 (-77.2 to -67.1)	0.09 (0.06 to 0.12)	0.01 (0.01 to 0.01)	-90.7 (-92.7 to -88.1)
		Group B Streptococcus	48.2 (38 to 60.1)	16.1 (12.9 to 20.3)	-66.5 (-72.1 to -59.8)	0.85 (0.74 to 0.97)	0.08 (0.06 to 0.1)	-90.7 (-92.6 to -88.3)
		Haemophilus influenzae	483 (395 to 593)	81.2 (65.8 to 100)	-83.1 (-85.9 to -80.1)	1.97 (1.73 to 2.25)	0.05 (0.04 to 0.07)	-97.2 (-97.8 to -96.5)
		Influenza	403 (332 to 485)	3.02 (0.65 to 8)	-99.2 (-99.8 to -98.0)	2.93 (2.59 to 3.3)	0.01 (0 to 0.02)	-99.8 (-100.0 to -99.4)
		Klebsiella pneumoniae	225 (186 to 271)	44 (36.3 to 53.6)	-80.4 (-83.1 to -77.3)	2.38 (2.09 to 2.69)	0.16 (0.14 to 0.2)	-93.1 (-94.4 to -91.5)
		Legionella spp.	19.8 (13.1 to 29.8)	6.72 (4.52 to 10)	-65.9 (-71.5 to -59.6)	0.41 (0.31 to 0.55)	0.04 (0.03 to 0.06)	-89.0 (-91.4 to -85.9)
		Mycoplasma spp.	273 (225 to 333)	75.2 (61.5 to 92)	-72.4 (-76.6 to -67.8)	0.75 (0.66 to 0.86)	0.06 (0.05 to 0.08)	-91.5 (-93.3 to -89.5)
		Polymicrobial	5.14 (3.2 to 8.02)	1.32 (0.8 to 2.11)	-74.4 (-77.8 to -70.4)	0.58 (0.31 to 0.97)	0.06 (0.03 to 0.11)	-89.5 (-92.0 to -86.2)
		Pseudomonas aeruginosa	134 (110 to 164)	62.4 (51 to 76.7)	-53.2 (-60.6 to -44.9)	1.33 (1.16 to 1.5)	0.17 (0.14 to 0.21)	-86.9 (-89.5 to -83.8)
		Respiratory syncytial virus	1000 (835 to 1200)	12.1 (2.58 to 32.5)	-98.8 (-99.7 to -96.8)	4.12 (3.69 to 4.59)	0.01 (0 to 0.04)	-99.7 (-99.9 to -99.0)
		Staphylococcus aureus	168 (140 to 202)	66.2 (54.1 to 80.8)	-60.6 (-65.7 to -54.8)	2.42 (2.16 to 2.72)	0.3 (0.25 to 0.36)	-87.6 (-90.0 to -84.8)
		Streptococcus pneumoniae	2660 (2220 to 3170)	435 (359 to 530)	-83.7 (-85.8 to -81.3)	12.4 (11.1 to 13.8)	0.72 (0.6 to 0.87)	-94.2 (-95.3 to -92.9)
		Other viruses	2130 (1770 to 2560)	546 (450 to 660)	-74.3 (-77.7 to -70.4)	4.21 (3.77 to 4.68)	0.37 (0.31 to 0.45)	-91.1 (-92.8 to -89.1)
		Other bacterial pathogens	67.1 (50.5 to 85.6)	13.2 (9.89 to 16.9)	-80.3 (-83.5 to -76.9)	0.5 (0.38 to 0.67)	0.04 (0.02 to 0.05)	-93.0 (-94.4 to -91.2)
	5-14 years	*Acinetobacter baumannii*	35 (25.8 to 46.1)	10.6 (7.96 to 14)	-69.6 (-73.1 to -65.5)	0.04 (0.03 to 0.05)	0 (0 to 0)	-90.1 (-91.9 to -88.0)
		Chlamydia spp.	170 (128 to 224)	43.5 (32.7 to 56.7)	-74.3 (-76.9 to -71.1)	0.05 (0.04 to 0.05)	0 (0 to 0)	-91.6 (-93.1 to -89.9)
		Enterobacter spp.	16.9 (12.5 to 22.5)	10.6 (7.9 to 14.3)	-37.3 (-45.0 to -28.4)	0.02 (0.01 to 0.02)	0 (0 to 0)	-79.5 (-83.3 to -75.2)
		Escherichia coli	42.3 (32 to 54.9)	19 (14.5 to 25)	-54.9 (-60.1 to -49.2)	0.05 (0.04 to 0.05)	0.01 (0.01 to 0.01)	-85.2 (-87.9 to -82.2)
		Fungus	51 (38.1 to 66.9)	23.5 (17.8 to 31.2)	-53.8 (-59.3 to -47.4)	0.05 (0.05 to 0.06)	0.01 (0.01 to 0.01)	-84.9 (-87.5 to -81.7)
		Group B Streptococcus	54 (40.3 to 69.8)	29.2 (22 to 38.4)	-45.9 (-52.0 to -38.9)	0.04 (0.04 to 0.05)	0.01 (0.01 to 0.01)	-82.3 (-85.7 to -78.6)
		Haemophilus influenzae	222 (168 to 286)	73.8 (56 to 96.5)	-66.7 (-69.9 to -63.0)	0.09 (0.08 to 0.1)	0.01 (0.01 to 0.01)	-92.8 (-94.1 to -91.4)
		Influenza	313 (239 to 400)	4.04 (0.83 to 11.3)	-98.7 (-99.7 to -96.5)	0.25 (0.23 to 0.28)	0 (0 to 0)	-99.6 (-99.9 to -98.8)

		Klebsiella pneumoniae	124 (94.2 to 161)	51.8 (39.4 to 67.5)	-58.3 (-62.0 to -53.9)	0.13 (0.12 to 0.15)	0.02 (0.02 to 0.02)	-86.3 (-88.7 to -83.7)
		Legionella spp.	114 (85.2 to 148)	65 (49.2 to 84.3)	-42.9 (-49.5 to -35.7)	0.08 (0.07 to 0.1)	0.02 (0.01 to 0.02)	-81.3 (-84.6 to -77.5)
		Mycoplasma spp.	489 (368 to 626)	188 (145 to 249)	-61.4 (-65.2 to -57.3)	0.14 (0.12 to 0.16)	0.02 (0.01 to 0.02)	-87.4 (-89.6 to -84.8)
		Polymicrobial	6.9 (4.21 to 10.8)	2.66 (1.5 to 4.32)	-61.7 (-67.0 to -56.2)	0.01 (0 to 0.01)	0 (0 to 0)	-87.5 (-89.9 to -84.7)
		Pseudomonas aeruginosa	124 (94.6 to 160)	92.2 (70.5 to 121)	-25.7 (-33.0 to -17.4)	0.09 (0.08 to 0.1)	0.02 (0.02 to 0.03)	-75.7 (-80.1 to -70.7)
		Respiratory syncytial virus	88.9 (67.7 to 116)	1.71 (0.35 to 4.78)	-98.1 (-99.6 to -94.8)	0.03 (0.03 to 0.04)	0 (0 to 0)	-99.4 (-99.9 to -98.2)
		Staphylococcus aureus	189 (142 to 241)	126 (96.6 to 165)	-33.3 (-38.7 to -27.1)	0.29 (0.26 to 0.32)	0.06 (0.05 to 0.07)	-78.2 (-81.9 to -74.0)
		Streptococcus pneumoniae	1760 (1330 to 2250)	526 (406 to 686)	-70.1 (-72.5 to -67.3)	1.12 (1.01 to 1.23)	0.11 (0.09 to 0.13)	-90.2 (-91.8 to -88.3)
		Other viruses	853 (646 to 1090)	458 (354 to 601)	-46.4 (-50.7 to -41.4)	0.21 (0.19 to 0.23)	0.04 (0.03 to 0.04)	-82.4 (-85.3 to -79.0)
		Other bacterial pathogens	183 (138 to 238)	59.1 (44.5 to 78.1)	-67.6 (-70.8 to -64.0)	0.11 (0.1 to 0.13)	0.01 (0.01 to 0.01)	-89.4 (-91.2 to -87.2)
	15-49 years	*Acinetobacter baumannii*	61.3 (51.4 to 72.5)	22.1 (18.4 to 26.1)	-63.9 (-67.7 to -59.5)	0.5 (0.42 to 0.58)	0.2 (0.16 to 0.24)	-60.2 (-67.3 to -52.4)
		Chlamydia spp.	297 (262 to 342)	90.7 (79.3 to 102)	-69.4 (-72.5 to -65.9)	0.6 (0.53 to 0.67)	0.2 (0.17 to 0.23)	-66.3 (-72.2 to -59.8)
		Enterobacter spp.	29.6 (25 to 34.8)	22 (18.8 to 25.4)	-25.5 (-34.7 to -16.1)	0.23 (0.19 to 0.26)	0.19 (0.15 to 0.22)	-17.8 (-32.9 to -0.5)
		Escherichia coli	74.2 (64.1 to 85.4)	39.7 (35 to 45.1)	-46.4 (-52.2 to -39.9)	0.59 (0.52 to 0.66)	0.35 (0.3 to 0.4)	-40.8 (-51.5 to -29.9)
		Fungus	89.3 (76.5 to 104)	48.9 (42.8 to 56.3)	-45.1 (-51.2 to -38.3)	0.7 (0.6 to 0.81)	0.42 (0.35 to 0.5)	-39.4 (-50.8 to -28.5)
		Group B Streptococcus	94.6 (81.9 to 109)	60.8 (53.4 to 68.7)	-35.6 (-42.8 to -27.5)	0.53 (0.46 to 0.62)	0.38 (0.32 to 0.44)	-29.0 (-42.1 to -14.7)
		Haemophilus influenzae	389 (347 to 439)	154 (136 to 171)	-60.4 (-64.1 to -56.7)	1.18 (1.06 to 1.31)	0.34 (0.29 to 0.39)	-71.1 (-76.0 to -65.9)
		Influenza	548 (493 to 614)	8.42 (1.85 to 23.1)	-98.5 (-99.7 to -95.8)	3.25 (2.95 to 3.56)	0.06 (0.01 to 0.15)	-98.3 (-99.6 to -95.3)
		Klebsiella pneumoniae	218 (195 to 244)	108 (95.8 to 121)	-50.4 (-54.8 to -45.7)	1.72 (1.56 to 1.89)	0.94 (0.8 to 1.08)	-45.2 (-54.2 to -35.6)
		Legionella spp.	200 (175 to 229)	135 (120 to 151)	-32.1 (-40.1 to -23.3)	1.09 (0.95 to 1.23)	0.81 (0.69 to 0.94)	-25.1 (-39.2 to -10.0)
		Mycoplasma spp.	856 (767 to 962)	393 (348 to 437)	-54.1 (-58.0 to -49.4)	1.79 (1.61 to 1.99)	0.91 (0.78 to 1.05)	-49.4 (-58.0 to -40.2)
		Polymicrobial	12.1 (7.64 to 18.5)	5.54 (3.23 to 9.04)	-54.5 (-60.6 to -48.2)	0.1 (0.06 to 0.15)	0.05 (0.03 to 0.08)	-49.8 (-58.9 to -38.9)
		Pseudomonas aeruginosa	218 (194 to 247)	192 (171 to 214)	-11.6 (-20.1 to -2.7)	1.19 (1.07 to 1.33)	1.16 (1 to 1.33)	-2.5 (-19.3 to 15.5)
		Respiratory syncytial virus	156 (137 to 176)	3.56 (0.78 to 9.65)	-97.7 (-99.5 to -93.8)	0.42 (0.38 to 0.47)	0.01 (0 to 0.03)	-97.5 (-99.5 to -93.0)

		Staphylococcus aureus	331 (297 to 369)	262 (233 to 290)	-20.7 (-27.1 to -13.8)	3.71 (3.39 to 4.07)	3.24 (2.79 to 3.73)	-12.5 (-26.7 to 2.4)
		Streptococcus pneumoniae	3080 (2780 to 3430)	1100 (977 to 1220)	-64.4 (-67.1 to -61.3)	14.4 (13.1 to 15.7)	5.63 (4.83 to 6.49)	-60.7 (-66.9 to -54.0)
		Other viruses	1500 (1350 to 1670)	953 (849 to 1060)	-36.2 (-41.2 to -30.7)	2.74 (2.51 to 3.01)	1.93 (1.65 to 2.22)	-29.6 (-40.9 to -17.5)
		Other bacterial pathogens	320 (279 to 370)	123 (105 to 143)	-61.5 (-65.1 to -57.3)	1.43 (1.25 to 1.63)	0.6 (0.5 to 0.73)	-57.4 (-64.8 to -49.6)
	50-69 years	*Acinetobacter baumannii*	238 (199 to 277)	103 (87 to 121)	-56.5 (-61.9 to -51.0)	1.38 (1.19 to 1.59)	0.97 (0.81 to 1.17)	-29.3 (-41.8 to -15.4)
		Chlamydia spp.	684 (589 to 775)	242 (213 to 274)	-64.6 (-68.6 to -60.1)	0.99 (0.88 to 1.11)	0.56 (0.48 to 0.66)	-42.5 (-52.3 to -31.4)
		Enterobacter spp.	112 (94.2 to 131)	98.5 (85.7 to 113)	-11.6 (-22.8 to 1.1)	0.62 (0.53 to 0.71)	0.88 (0.74 to 1.02)	43.5 (16.9 to 73.2)
		Escherichia coli	309 (266 to 351)	193 (173 to 218)	-37.3 (-44.5 to -29.1)	1.76 (1.57 to 1.97)	1.78 (1.52 to 2.05)	1.8 (-16.7 to 21.5)
		Fungus	296 (252 to 341)	193 (170 to 221)	-34.6 (-42.1 to -25.9)	1.66 (1.46 to 1.89)	1.76 (1.47 to 2.05)	6.2 (-13.4 to 25.8)
		Group B Streptococcus	253 (215 to 290)	190 (168 to 214)	-24.7 (-34.1 to -14.6)	1.02 (0.89 to 1.16)	1.24 (1.06 to 1.43)	22.4 (0.8 to 45.8)
		Haemophilus influenzae	886 (781 to 990)	437 (392 to 489)	-50.6 (-55.4 to -45.6)	1.92 (1.73 to 2.12)	1.02 (0.87 to 1.16)	-46.9 (-55.6 to -37.7)
		Influenza	1080 (949 to 1190)	19.8 (4.45 to 53.1)	-98.2 (-99.6 to -95.0)	4.57 (4.15 to 5.01)	0.14 (0.03 to 0.38)	-97.0 (-99.4 to -91.7)
		Klebsiella pneumoniae	635 (562 to 703)	404 (363 to 456)	-36.3 (-42.6 to -29.8)	3.59 (3.28 to 3.93)	3.71 (3.19 to 4.23)	3.5 (-13.3 to 21.0)
		Legionella spp.	640 (554 to 726)	515 (464 to 573)	-19.4 (-29.1 to -8.8)	2.49 (2.2 to 2.82)	3.25 (2.77 to 3.73)	31.0 (8.2 to 56.2)
		Mycoplasma spp.	895 (782 to 1000)	461 (413 to 516)	-48.4 (-54.0 to -42.7)	1.34 (1.21 to 1.49)	1.12 (0.96 to 1.28)	-16.1 (-30.6 to -1.1)
		Polymicrobial	39.2 (25 to 61.3)	21.4 (12.9 to 34.6)	-45.6 (-53.6 to -37.9)	0.23 (0.14 to 0.35)	0.2 (0.12 to 0.33)	-11.7 (-28.7 to 6.3)
		Pseudomonas aeruginosa	703 (619 to 789)	724 (651 to 812)	3.2 (-7.9 to 15.2)	2.76 (2.5 to 3.03)	4.62 (3.96 to 5.28)	67.7 (40.3 to 98.0)
		Respiratory syncytial virus	226 (197 to 258)	6.03 (1.32 to 16.5)	-97.3 (-99.4 to -92.8)	0.44 (0.39 to 0.49)	0.02 (0 to 0.05)	-95.7 (-99.1 to -87.8)
		Staphylococcus aureus	940 (826 to 1040)	889 (803 to 991)	-5.3 (-14.9 to 3.7)	7.55 (6.91 to 8.25)	11.6 (9.94 to 13.2)	53.8 (29.9 to 80.2)
		Streptococcus pneumoniae	4320 (3810 to 4770)	1880 (1700 to 2100)	-56.3 (-60.5 to -52.3)	14.4 (13.2 to 15.7)	10.2 (8.81 to 11.6)	-29.1 (-40.5 to -17.2)
		Other viruses	1840 (1630 to 2030)	1490 (1350 to 1660)	-18.6 (-26.8 to -10.6)	2.42 (2.21 to 2.63)	3.19 (2.74 to 3.63)	32.3 (10.9 to 54.7)
		Other bacterial pathogens	1100 (948 to 1260)	503 (431 to 584)	-54.3 (-59.1 to -49.1)	3.52 (3.1 to 3.96)	2.61 (2.16 to 3.12)	-25.7 (-37.7 to -12.3)
	70+ years	*Acinetobacter baumannii*	157 (131 to 189)	130 (109 to 155)	-16.8 (-27.5 to -5.0)	0.79 (0.67 to 0.93)	2.08 (1.68 to 2.44)	164.9 (120.8 to 212.1)
		Chlamydia spp.	538 (458 to 628)	363 (316 to 417)	-32.3 (-40.5 to -23.3)	0.67 (0.58 to 0.76)	1.44 (1.18 to 1.67)	115.6 (80.1 to 151.7)

		Enterobacter spp.	71.9 (59.8 to 86.4)	121 (103 to 141)	69.0 (45.3 to 95.0)	0.34 (0.29 to 0.4)	1.83 (1.5 to 2.14)	438.0 (343.0 to 538.2)
		Escherichia coli	250 (214 to 297)	299 (264 to 345)	19.8 (4.8 to 37.2)	1.23 (1.07 to 1.4)	4.67 (3.92 to 5.34)	281.3 (215.9 to 345.8)
		Fungus	109 (90.7 to 130)	136 (117 to 159)	25.0 (8.8 to 42.8)	0.53 (0.45 to 0.61)	2.09 (1.72 to 2.44)	298.1 (229.0 to 367.6)
		Group B Streptococcus	153 (129 to 179)	220 (190 to 252)	44.0 (26.0 to 65.9)	0.53 (0.46 to 0.61)	2.44 (2.02 to 2.81)	358.6 (276.1 to 436.9)
		Haemophilus influenzae	527 (458 to 609)	497 (437 to 571)	-5.5 (-16.5 to 7.0)	0.99 (0.87 to 1.1)	1.96 (1.65 to 2.24)	99.0 (68.3 to 131.6)
		Influenza	1080 (941 to 1240)	38 (8.38 to 103)	-96.5 (-99.2 to -90.5)	3.95 (3.54 to 4.39)	0.44 (0.1 to 1.22)	-88.8 (-97.6 to -69.2)
		Klebsiella pneumoniae	462 (402 to 534)	562 (496 to 642)	21.8 (8.6 to 36.5)	2.25 (2.02 to 2.51)	8.73 (7.32 to 10)	288.0 (225.0 to 349.7)
		Legionella spp.	304 (259 to 358)	466 (411 to 533)	54.1 (33.3 to 78.9)	1.02 (0.89 to 1.15)	4.99 (4.19 to 5.71)	390.7 (306.2 to 478.2)
		Mycoplasma spp.	499 (435 to 577)	491 (432 to 562)	-1.3 (-12.2 to 12.1)	0.65 (0.57 to 0.73)	2.03 (1.7 to 2.34)	214.4 (163.5 to 270.7)
		Polymicrobial	29.6 (18.8 to 47.1)	31 (17.7 to 52.4)	4.0 (-12.9 to 21.1)	0.15 (0.09 to 0.23)	0.49 (0.28 to 0.8)	231.0 (172.7 to 300.8)
		Pseudomonas aeruginosa	483 (418 to 563)	950 (835 to 1090)	97.3 (73.9 to 123.5)	1.64 (1.46 to 1.83)	10.3 (8.61 to 11.7)	528.3 (427.8 to 633.4)
		Respiratory syncytial virus	140 (119 to 164)	7.14 (1.51 to 19.2)	-94.9 (-98.9 to -86.4)	0.23 (0.21 to 0.27)	0.04 (0.01 to 0.1)	-83.8 (-96.6 to -54.7)
		Staphylococcus aureus	802 (700 to 921)	1450 (1280 to 1660)	81.0 (62.3 to 102.4)	5.56 (4.98 to 6.17)	32 (27.1 to 36.5)	476.4 (387.7 to 568.0)
		Streptococcus pneumoniae	2930 (2570 to 3360)	2440 (2160 to 2790)	-16.5 (-25.0 to -6.7)	8.46 (7.62 to 9.37)	22.4 (19 to 25.7)	165.8 (125.1 to 206.2)
		Other viruses	1230 (1080 to 1410)	1910 (1690 to 2200)	55.7 (40.1 to 75.0)	1.4 (1.26 to 1.55)	6.92 (5.86 to 7.92)	395.7 (318.0 to 472.9)
		Other bacterial pathogens	973 (825 to 1150)	848 (716 to 999)	-12.7 (-22.6 to -1.7)	2.71 (2.35 to 3.12)	7.53 (6.09 to 9.09)	178.4 (133.5 to 224.0)
Latvia	All Ages	*Acinetobacter baumannii*	838 (725 to 955)	358 (312 to 408)	-57.2 (-61.6 to -52.1)	5.95 (5.22 to 6.81)	4.46 (3.87 to 5.11)	-24.9 (-35.6 to -13.4)
		Chlamydia spp.	3010 (2730 to 3310)	1330 (1220 to 1460)	-55.6 (-59.7 to -50.9)	5.27 (4.89 to 5.67)	4.01 (3.52 to 4.53)	-23.7 (-33.4 to -13.0)
		Enterobacter spp.	394 (344 to 450)	455 (407 to 504)	15.9 (3.0 to 28.9)	2.67 (2.32 to 3.06)	5.22 (4.52 to 5.97)	96.6 (67.2 to 127.6)
		Escherichia coli	1190 (1060 to 1320)	1160 (1060 to 1260)	-2.4 (-12.4 to 7.7)	8.17 (7.49 to 8.94)	13.9 (12.2 to 15.5)	70.3 (47.6 to 93.0)
		Fungus	891 (788 to 1010)	729 (652 to 817)	-18.1 (-27.2 to -8.4)	5.45 (4.87 to 6.11)	8.17 (7.09 to 9.29)	50.0 (27.2 to 72.8)
		Group B Streptococcus	960 (858 to 1080)	896 (815 to 984)	-6.5 (-17.1 to 3.7)	5.13 (4.72 to 5.66)	7.46 (6.55 to 8.37)	45.7 (25.7 to 67.9)
		Haemophilus influenzae	3930 (3610 to 4270)	2270 (2110 to 2470)	-42.1 (-46.9 to -37.5)	10.3 (9.65 to 11)	6.37 (5.6 to 7.13)	-38.0 (-45.4 to -30.5)
		Influenza	5450 (5010 to 5900)	45.9 (1.93 to 237)	-99.2 (-100.0 to -95.7)	26.2 (24.7 to 27.8)	0.4 (0.02 to 2.16)	-98.5 (-99.9 to -91.7)

		Klebsiella pneumoniae	2670 (2460 to 2880)	1750 (1610 to 1900)	-34.2 (-39.3 to -28.9)	17.6 (16.6 to 18.7)	20.5 (18.3 to 22.8)	17.0 (3.3 to 31.0)
		Legionella spp.	1990 (1790 to 2220)	1790 (1650 to 1940)	-10.2 (-19.3 to -0.6)	8.91 (8.06 to 9.84)	14.3 (12.5 to 16)	60.4 (37.5 to 85.3)
		Mycoplasma spp.	4730 (4330 to 5210)	2510 (2320 to 2720)	-46.8 (-51.1 to -42.5)	8.04 (7.49 to 8.64)	7.05 (6.16 to 7.88)	-12.2 (-23.4 to -0.2)
		Polymicrobial	153 (99.6 to 233)	123 (75.9 to 197)	-19.5 (-29.5 to -8.8)	1.77 (1.25 to 2.5)	1.65 (1.08 to 2.56)	-6.1 (-34.3 to 23.9)
		Pseudomonas aeruginosa	2610 (2380 to 2860)	2800 (2590 to 3030)	7.5 (-2.1 to 17.1)	12.1 (11.3 to 12.9)	22.6 (20 to 25.2)	86.8 (63.9 to 110.8)
		Respiratory syncytial virus	2400 (2100 to 2730)	12.9 (0.53 to 66.4)	-99.5 (-100.0 to -97.3)	7.67 (7.15 to 8.2)	0.04 (0 to 0.2)	-99.5 (-100.0 to -97.4)
		Staphylococcus aureus	3870 (3570 to 4200)	4900 (4540 to 5290)	26.7 (17.1 to 36.2)	34.7 (32.6 to 36.8)	82.6 (73 to 91.9)	138.2 (111.4 to 167.9)
		Streptococcus pneumoniae	23200 (21500 to 25000)	6860 (6350 to 7390)	-70.5 (-72.6 to -68.3)	86.2 (81.8 to 90.8)	45.2 (40.1 to 50.2)	-47.6 (-53.7 to -41.2)
		Other viruses	11500 (10600 to 12600)	6580 (6080 to 7110)	-42.9 (-47.6 to -38.6)	17.6 (16.7 to 18.5)	16.5 (14.6 to 18.4)	-6.3 (-17.0 to 4.6)
		Other bacterial pathogens	4310 (3810 to 4810)	2560 (2250 to 2910)	-40.5 (-45.9 to -34.6)	15.2 (13.6 to 16.9)	17.6 (15 to 20.4)	15.6 (0.6 to 31.6)
	Under 5	*Acinetobacter baumannii*	13.5 (9.62 to 18.6)	2.16 (1.55 to 2.97)	-83.9 (-86.6 to -80.9)	0.77 (0.43 to 1.28)	0.11 (0.06 to 0.19)	-86.3 (-89.4 to -83.0)
		Chlamydia spp.	234 (191 to 285)	43.5 (35.1 to 54.2)	-81.3 (-84.4 to -77.6)	0.96 (0.86 to 1.07)	0.11 (0.09 to 0.13)	-88.5 (-90.8 to -86.0)
		Enterobacter spp.	25.4 (18.6 to 33.4)	11.6 (8.64 to 15.7)	-54.1 (-63.4 to -44.1)	0.47 (0.32 to 0.71)	0.09 (0.06 to 0.14)	-80.0 (-84.2 to -75.2)
		Escherichia coli	86.3 (68.6 to 106)	39 (31.2 to 47.9)	-54.6 (-63.2 to -45.5)	1.42 (1.24 to 1.63)	0.26 (0.21 to 0.32)	-81.5 (-85.3 to -77.5)
		Fungus	18.1 (12.4 to 24.9)	6.05 (4.22 to 8.23)	-66.3 (-72.8 to -58.5)	0.12 (0.08 to 0.16)	0.02 (0.01 to 0.02)	-85.2 (-88.7 to -81.4)
		Group B Streptococcus	71.8 (56.5 to 89.2)	28.8 (22.7 to 36.2)	-59.7 (-67.1 to -51.1)	1.24 (1.09 to 1.41)	0.22 (0.17 to 0.26)	-82.6 (-86.0 to -78.7)
		Haemophilus influenzae	712 (578 to 871)	162 (131 to 199)	-77.1 (-81.1 to -72.6)	2.74 (2.43 to 3.06)	0.13 (0.11 to 0.16)	-95.1 (-96.1 to -93.9)
		Influenza	595 (491 to 719)	1.58 (0.06 to 8.22)	-99.7 (-100.0 to -98.6)	4.05 (3.68 to 4.45)	0 (0 to 0.02)	-99.9 (-100.0 to -99.4)
		Klebsiella pneumoniae	335 (273 to 407)	64.3 (51.5 to 78.2)	-80.8 (-84.0 to -77.2)	3.34 (3.01 to 3.69)	0.37 (0.3 to 0.44)	-89.0 (-91.1 to -86.8)
		Legionella spp.	28.9 (19.5 to 43)	10.8 (7.28 to 15.6)	-62.6 (-69.6 to -54.6)	0.6 (0.44 to 0.79)	0.12 (0.09 to 0.16)	-80.3 (-84.5 to -75.5)
		Mycoplasma spp.	404 (328 to 492)	164 (131 to 200)	-59.2 (-65.9 to -51.5)	1.04 (0.93 to 1.17)	0.17 (0.14 to 0.21)	-83.3 (-86.8 to -79.5)
		Polymicrobial	7.99 (4.96 to 12.3)	2.34 (1.41 to 3.75)	-70.8 (-75.2 to -65.8)	0.87 (0.46 to 1.43)	0.17 (0.09 to 0.3)	-80.1 (-84.6 to -74.4)
		Pseudomonas aeruginosa	195 (158 to 240)	112 (90.7 to 137)	-42.2 (-52.0 to -30.6)	1.86 (1.68 to 2.06)	0.42 (0.34 to 0.5)	-77.5 (-81.8 to -72.9)
		Respiratory syncytial virus	1450 (1200 to 1750)	5.6 (0.22 to 28.9)	-99.6 (-100.0 to -98.0)	5.68 (5.2 to 6.18)	0.01 (0 to 0.05)	-99.8 (-100.0 to -99.1)

		Staphylococcus aureus	247 (203 to 298)	117 (94.9 to 143)	-52.4 (-59.2 to -44.4)	3.33 (3.04 to 3.64)	0.69 (0.56 to 0.82)	-79.4 (-83.5 to -75.2)
		Streptococcus pneumoniae	3940 (3230 to 4790)	319 (258 to 392)	-91.9 (-93.1 to -90.6)	16.8 (15.4 to 18.2)	0.59 (0.47 to 0.71)	-96.5 (-97.2 to -95.7)
		Other viruses	3080 (2540 to 3730)	613 (497 to 749)	-80.1 (-82.9 to -76.9)	5.63 (5.2 to 6.1)	0.6 (0.49 to 0.71)	-89.4 (-91.4 to -87.2)
		Other bacterial pathogens	100 (75.3 to 128)	24.4 (18.4 to 31.5)	-75.6 (-80.1 to -69.9)	0.72 (0.54 to 0.97)	0.09 (0.06 to 0.14)	-87.0 (-89.7 to -84.1)
	5-14 years	*Acinetobacter baumannii*	52.5 (38.2 to 69.7)	13.3 (9.84 to 17.4)	-74.6 (-77.8 to -71.5)	0.06 (0.05 to 0.08)	0 (0 to 0)	-94.2 (-95.4 to -92.7)
		Chlamydia spp.	250 (185 to 329)	72.7 (53.8 to 93.9)	-70.8 (-73.9 to -67.2)	0.07 (0.07 to 0.09)	0.01 (0 to 0.01)	-93.3 (-94.6 to -91.7)
		Enterobacter spp.	24.2 (17.5 to 32.7)	17.7 (13.2 to 23.3)	-26.9 (-36.3 to -17.3)	0.03 (0.02 to 0.03)	0 (0 to 0.01)	-83.2 (-86.7 to -79.0)
		Escherichia coli	61.4 (45.5 to 80.5)	37.6 (28 to 48.3)	-38.5 (-45.6 to -31.0)	0.07 (0.06 to 0.08)	0.01 (0.01 to 0.01)	-85.9 (-88.6 to -82.2)
		Fungus	73.9 (54.1 to 97.8)	39.2 (29.2 to 51)	-46.8 (-53.3 to -40.0)	0.09 (0.07 to 0.1)	0.01 (0.01 to 0.01)	-87.8 (-90.4 to -84.6)
		Group B Streptococcus	77.9 (57.2 to 101)	48.1 (36.1 to 62.3)	-38.0 (-45.7 to -30.0)	0.07 (0.06 to 0.08)	0.01 (0.01 to 0.01)	-85.7 (-88.7 to -82.2)
		Haemophilus influenzae	318 (236 to 420)	125 (92.3 to 159)	-60.8 (-64.5 to -56.7)	0.14 (0.13 to 0.16)	0.01 (0.01 to 0.01)	-94.0 (-95.2 to -92.6)
		Influenza	449 (335 to 588)	2.4 (0.1 to 12.7)	-99.5 (-100.0 to -97.2)	0.4 (0.35 to 0.45)	0 (0 to 0)	-99.9 (-100.0 to -99.3)
		Klebsiella pneumoniae	180 (135 to 237)	71.2 (53.8 to 92)	-60.4 (-64.2 to -56.5)	0.21 (0.19 to 0.24)	0.02 (0.02 to 0.02)	-90.9 (-92.7 to -88.8)
		Legionella spp.	161 (120 to 213)	91.9 (69.1 to 119)	-42.7 (-50.2 to -35.5)	0.13 (0.11 to 0.15)	0.02 (0.01 to 0.02)	-86.8 (-89.6 to -83.7)
		Mycoplasma spp.	702 (520 to 920)	279 (211 to 360)	-60.1 (-64.2 to -55.9)	0.22 (0.19 to 0.25)	0.02 (0.02 to 0.02)	-90.8 (-92.6 to -88.7)
		Polymicrobial	10.2 (6.3 to 16)	5.03 (2.93 to 8.19)	-50.6 (-57.3 to -43.4)	0.01 (0.01 to 0.02)	0 (0 to 0)	-88.6 (-91.0 to -85.6)
		Pseudomonas aeruginosa	176 (131 to 231)	123 (93.1 to 159)	-29.7 (-36.7 to -21.7)	0.14 (0.13 to 0.16)	0.02 (0.02 to 0.03)	-83.8 (-87.0 to -79.9)
		Respiratory syncytial virus	125 (94.3 to 166)	0.64 (0.03 to 3.39)	-99.5 (-100.0 to -97.3)	0.05 (0.04 to 0.06)	0 (0 to 0)	-99.9 (-100.0 to -99.4)
		Staphylococcus aureus	270 (203 to 352)	210 (158 to 269)	-22.0 (-28.8 to -14.9)	0.45 (0.4 to 0.5)	0.08 (0.07 to 0.1)	-82.1 (-85.5 to -77.9)
		Streptococcus pneumoniae	2530 (1910 to 3310)	523 (397 to 674)	-79.3 (-81.1 to -77.5)	1.77 (1.58 to 1.97)	0.08 (0.07 to 0.1)	-95.2 (-96.1 to -94.1)
		Other viruses	1200 (906 to 1580)	516 (392 to 667)	-57.1 (-60.9 to -53.4)	0.33 (0.29 to 0.37)	0.03 (0.03 to 0.04)	-90.1 (-92.1 to -87.8)
		Other bacterial pathogens	266 (199 to 351)	94.4 (69.8 to 123)	-64.4 (-68.2 to -60.6)	0.18 (0.15 to 0.21)	0.01 (0.01 to 0.02)	-91.8 (-93.4 to -89.9)
	15-49 years	*Acinetobacter baumannii*	104 (86.6 to 122)	32 (27.1 to 37.7)	-69.0 (-72.7 to -64.5)	0.9 (0.77 to 1.05)	0.43 (0.36 to 0.52)	-52.0 (-60.5 to -42.6)
		Chlamydia spp.	492 (436 to 561)	175 (154 to 199)	-64.4 (-68.4 to -59.9)	1.06 (0.94 to 1.18)	0.58 (0.49 to 0.68)	-44.9 (-54.2 to -34.7)

		Enterobacter spp.	47.8 (40.3 to 56.2)	42.4 (36.7 to 48.7)	-10.9 (-21.5 to 2.1)	0.39 (0.33 to 0.46)	0.54 (0.46 to 0.64)	38.1 (14.6 to 66.1)
		Escherichia coli	121 (106 to 138)	90.5 (80.3 to 101)	-25.0 (-33.1 to -15.0)	1.03 (0.9 to 1.15)	1.19 (1.01 to 1.38)	16.2 (-2.7 to 37.8)
		Fungus	146 (126 to 171)	94.3 (82 to 109)	-35.1 (-43.1 to -25.9)	1.22 (1.06 to 1.39)	1.22 (1.02 to 1.43)	0.5 (-16.6 to 20.6)
		Group B Streptococcus	153 (133 to 178)	116 (102 to 131)	-24.4 (-33.6 to -14.5)	0.93 (0.81 to 1.06)	1.08 (0.89 to 1.27)	17.1 (-3.8 to 42.2)
		Haemophilus influenzae	627 (564 to 707)	299 (268 to 333)	-52.2 (-56.5 to -47.1)	2.03 (1.86 to 2.22)	0.99 (0.85 to 1.14)	-51.0 (-58.8 to -42.4)
		Influenza	886 (802 to 992)	5.77 (0.24 to 29)	-99.3 (-100.0 to -96.6)	5.61 (5.16 to 6.11)	0.06 (0 to 0.3)	-99.0 (-100.0 to -94.6)
		Klebsiella pneumoniae	355 (321 to 398)	171 (153 to 192)	-51.7 (-56.1 to -46.5)	3 (2.74 to 3.29)	2.24 (1.91 to 2.57)	-25.2 (-37.2 to -12.4)
		Legionella spp.	317 (280 to 365)	221 (196 to 246)	-30.1 (-38.0 to -20.5)	1.84 (1.63 to 2.09)	1.99 (1.7 to 2.3)	8.3 (-10.8 to 30.5)
		Mycoplasma spp.	1380 (1250 to 1560)	672 (602 to 746)	-51.4 (-56.3 to -46.0)	3.1 (2.83 to 3.43)	2.33 (1.98 to 2.67)	-24.7 (-37.4 to -11.5)
		Polymicrobial	20 (12.6 to 30.5)	12.1 (7.19 to 19.2)	-39.8 (-47.9 to -30.8)	0.17 (0.11 to 0.27)	0.16 (0.1 to 0.26)	-6.7 (-23.9 to 12.9)
		Pseudomonas aeruginosa	347 (310 to 392)	297 (265 to 329)	-14.2 (-23.1 to -4.4)	2.03 (1.84 to 2.24)	2.69 (2.32 to 3.1)	32.9 (11.1 to 57.4)
		Respiratory syncytial virus	246 (219 to 277)	1.54 (0.06 to 8)	-99.4 (-100.0 to -96.7)	0.71 (0.64 to 0.79)	0.01 (0 to 0.04)	-99.0 (-100.0 to -94.9)
		Staphylococcus aureus	532 (483 to 596)	505 (454 to 564)	-4.9 (-12.9 to 5.5)	6.39 (5.87 to 6.94)	9.39 (8.05 to 10.8)	47.3 (24.7 to 72.6)
		Streptococcus pneumoniae	4990 (4550 to 5600)	1260 (1130 to 1390)	-74.8 (-76.9 to -72.2)	24.9 (23 to 27)	9.72 (8.34 to 11.2)	-60.9 (-66.8 to -54.4)
		Other viruses	2370 (2160 to 2670)	1240 (1110 to 1370)	-47.7 (-52.1 to -42.2)	4.66 (4.28 to 5.07)	3.77 (3.23 to 4.32)	-18.9 (-31.6 to -5.5)
		Other bacterial pathogens	524 (453 to 602)	227 (195 to 264)	-56.6 (-61.1 to -51.4)	2.49 (2.18 to 2.83)	1.68 (1.38 to 1.97)	-32.6 (-43.9 to -20.3)
	50-69 years	*Acinetobacter baumannii*	388 (329 to 451)	150 (126 to 176)	-61.3 (-65.9 to -56.1)	2.53 (2.21 to 2.92)	1.63 (1.37 to 1.92)	-35.6 (-46.1 to -25.1)
		Chlamydia spp.	1090 (961 to 1240)	459 (405 to 524)	-57.9 (-62.2 to -52.7)	1.77 (1.57 to 1.99)	1.24 (1.05 to 1.43)	-30.0 (-41.6 to -17.1)
		Enterobacter spp.	174 (148 to 202)	188 (165 to 218)	8.4 (-5.3 to 22.9)	1.08 (0.93 to 1.24)	1.93 (1.64 to 2.23)	80.2 (49.5 to 114.3)
		Escherichia coli	486 (429 to 552)	428 (380 to 487)	-11.8 (-22.0 to -1.7)	3.11 (2.77 to 3.47)	4.55 (3.92 to 5.16)	46.6 (22.3 to 72.7)
		Fungus	465 (402 to 535)	369 (321 to 425)	-20.5 (-30.9 to -10.0)	2.94 (2.56 to 3.35)	3.87 (3.3 to 4.49)	32.0 (9.4 to 54.6)
		Group B Streptococcus	395 (341 to 455)	355 (310 to 407)	-9.9 (-21.6 to 1.3)	1.8 (1.57 to 2.03)	2.68 (2.29 to 3.1)	49.8 (24.2 to 76.9)
		Haemophilus influenzae	1380 (1230 to 1540)	859 (770 to 970)	-37.5 (-43.6 to -31.5)	3.36 (3.04 to 3.72)	2.31 (1.99 to 2.62)	-31.3 (-41.8 to -20.9)
		Influenza	1680 (1510 to 1860)	13.8 (0.56 to 71.2)	-99.2 (-100.0 to -95.7)	8.01 (7.33 to 8.75)	0.11 (0 to 0.6)	-98.6 (-99.9 to -92.6)

		Klebsiella pneumoniae	999 (900 to 1100)	663 (593 to 751)	-33.5 (-40.1 to -27.2)	6.36 (5.83 to 6.92)	7.01 (6.08 to 7.94)	10.5 (-6.6 to 26.8)
		Legionella spp.	978 (863 to 1110)	827 (737 to 935)	-15.2 (-24.7 to -4.9)	4.29 (3.79 to 4.82)	6.02 (5.23 to 6.84)	40.9 (16.6 to 65.1)
		Mycoplasma spp.	1390 (1240 to 1570)	735 (650 to 829)	-47.2 (-52.9 to -41.3)	2.36 (2.14 to 2.61)	2.06 (1.77 to 2.33)	-12.2 (-26.6 to 2.0)
		Polymicrobial	62.5 (40.3 to 96)	46.6 (28.5 to 75.6)	-25.6 (-35.5 to -14.8)	0.41 (0.26 to 0.62)	0.5 (0.31 to 0.8)	23.6 (2.8 to 46.5)
		Pseudomonas aeruginosa	1080 (971 to 1210)	1070 (959 to 1210)	-0.3 (-11.3 to 10.3)	4.77 (4.34 to 5.25)	7.88 (6.85 to 8.93)	65.6 (40.6 to 92.9)
		Respiratory syncytial virus	345 (304 to 389)	2.54 (0.1 to 13.4)	-99.3 (-100.0 to -96.2)	0.75 (0.67 to 0.85)	0.01 (0 to 0.05)	-98.8 (-100.0 to -93.4)
		Staphylococcus aureus	1460 (1320 to 1600)	1710 (1520 to 1920)	17.2 (5.7 to 27.4)	13.2 (12.1 to 14.3)	25.6 (22.3 to 29)	94.8 (65.3 to 124.7)
		Streptococcus pneumoniae	6740 (6130 to 7410)	2270 (2030 to 2540)	-66.4 (-69.6 to -63.4)	25.4 (23.4 to 27.5)	14.2 (12.3 to 16)	-44.1 (-52.5 to -36.0)
		Other viruses	2810 (2550 to 3090)	2020 (1790 to 2260)	-28.1 (-35.1 to -21.6)	4.16 (3.83 to 4.53)	4.96 (4.29 to 5.63)	19.5 (1.6 to 38.0)
		Other bacterial pathogens	1740 (1520 to 1980)	910 (778 to 1060)	-47.5 (-53.2 to -41.9)	6.24 (5.51 to 6.99)	5.44 (4.53 to 6.42)	-12.7 (-26.6 to 2.3)
	70+ years	*Acinetobacter baumannii*	280 (234 to 331)	160 (136 to 189)	-42.7 (-50.4 to -33.9)	1.68 (1.45 to 1.95)	2.29 (1.93 to 2.66)	36.4 (17.4 to 57.6)
		Chlamydia spp.	940 (807 to 1080)	584 (508 to 668)	-37.7 (-45.1 to -29.4)	1.4 (1.25 to 1.56)	2.08 (1.77 to 2.38)	48.5 (28.2 to 70.9)
		Enterobacter spp.	123 (101 to 147)	196 (168 to 226)	60.3 (38.5 to 83.9)	0.7 (0.6 to 0.81)	2.65 (2.23 to 3.04)	282.1 (226.4 to 346.2)
		Escherichia coli	432 (367 to 500)	561 (492 to 642)	30.4 (14.4 to 48.0)	2.54 (2.25 to 2.84)	7.88 (6.78 to 8.83)	210.7 (168.8 to 253.5)
		Fungus	188 (156 to 222)	220 (188 to 256)	17.5 (1.6 to 33.5)	1.09 (0.95 to 1.25)	3.04 (2.57 to 3.51)	179.9 (141.3 to 224.8)
		Group B Streptococcus	262 (222 to 303)	348 (303 to 397)	33.2 (16.1 to 53.2)	1.1 (0.96 to 1.24)	3.47 (2.96 to 3.96)	217.5 (170.6 to 268.4)
		Haemophilus influenzae	898 (786 to 1020)	828 (731 to 935)	-7.6 (-17.6 to 3.7)	2.02 (1.82 to 2.22)	2.93 (2.54 to 3.27)	45.6 (27.6 to 65.8)
		Influenza	1840 (1610 to 2080)	22.4 (0.95 to 114)	-98.8 (-99.9 to -93.7)	8.1 (7.39 to 8.79)	0.23 (0.01 to 1.24)	-97.1 (-99.9 to -84.6)
		Klebsiella pneumoniae	797 (691 to 903)	782 (689 to 877)	-1.7 (-12.2 to 9.7)	4.66 (4.24 to 5.08)	10.9 (9.47 to 12.2)	134.3 (106.1 to 165.6)
		Legionella spp.	509 (435 to 587)	636 (560 to 720)	25.3 (9.2 to 44.0)	2.05 (1.81 to 2.29)	6.11 (5.27 to 6.92)	198.7 (156.9 to 246.4)
		Mycoplasma spp.	852 (745 to 970)	664 (586 to 751)	-21.9 (-31.3 to -11.6)	1.33 (1.2 to 1.47)	2.46 (2.12 to 2.76)	86.1 (62.3 to 112.6)
		Polymicrobial	51.8 (32.3 to 81.6)	57.1 (33.7 to 92)	10.0 (-6.1 to 27.4)	0.31 (0.2 to 0.49)	0.81 (0.49 to 1.33)	162.0 (123.4 to 207.8)
		Pseudomonas aeruginosa	813 (701 to 928)	1200 (1060 to 1350)	47.4 (30.2 to 67.0)	3.3 (3.01 to 3.62)	11.6 (10 to 12.9)	251.1 (206.6 to 299.7)
		Respiratory syncytial virus	234 (200 to 269)	2.54 (0.11 to 12.9)	-98.9 (-100.0 to -94.5)	0.47 (0.42 to 0.52)	0.01 (0 to 0.06)	-97.5 (-99.9 to -86.4)

		Staphylococcus aureus	1360 (1190 to 1540)	2360 (2090 to 2660)	73.3 (55.5 to 93.1)	11.4 (10.4 to 12.3)	46.8 (40.5 to 52.3)	312.9 (265.8 to 366.4)
		Streptococcus pneumoniae	5020 (4380 to 5650)	2490 (2200 to 2820)	-50.3 (-55.3 to -44.6)	17.4 (15.9 to 18.8)	20.6 (17.8 to 23)	18.5 (5.1 to 33.4)
		Other viruses	2070 (1800 to 2340)	2190 (1940 to 2480)	6.3 (-4.9 to 18.0)	2.81 (2.57 to 3.04)	7.12 (6.2 to 7.95)	153.4 (124.4 to 186.4)
		Other bacterial pathogens	1680 (1450 to 1950)	1300 (1100 to 1520)	-22.4 (-31.2 to -11.8)	5.61 (4.96 to 6.31)	10.4 (8.7 to 12.1)	85.2 (61.5 to 111.3)
Lithuania	All Ages	*Acinetobacter baumannii*	902 (783 to 1040)	675 (578 to 784)	-25.0 (-34.4 to -14.7)	4.42 (3.79 to 5.19)	7.32 (6.29 to 8.46)	66.2 (40.4 to 94.9)
		Chlamydia spp.	3490 (3170 to 3870)	2120 (1880 to 2390)	-39.1 (-46.8 to -30.6)	4.23 (3.9 to 4.59)	5.56 (4.86 to 6.26)	31.5 (13.7 to 49.8)
		Enterobacter spp.	473 (412 to 544)	665 (575 to 757)	40.8 (21.9 to 61.5)	2.22 (1.94 to 2.59)	6.69 (5.78 to 7.67)	202.0 (154.8 to 252.1)
		Escherichia coli	1400 (1260 to 1570)	1670 (1480 to 1880)	19.2 (4.4 to 36.7)	6.57 (6.02 to 7.2)	17.4 (15.3 to 19.6)	166.0 (128.0 to 202.2)
		Fungus	1020 (897 to 1160)	1090 (947 to 1240)	6.7 (-6.9 to 21.8)	4.33 (3.84 to 4.86)	11 (9.53 to 12.6)	153.8 (116.2 to 194.0)
		Group B Streptococcus	1150 (1030 to 1280)	1330 (1180 to 1500)	16.1 (1.7 to 32.7)	4.37 (3.98 to 4.8)	9.74 (8.49 to 10.9)	123.3 (91.6 to 156.2)
		Haemophilus influenzae	4880 (4470 to 5340)	3280 (2930 to 3690)	-32.8 (-40.5 to -23.6)	9.11 (8.46 to 9.77)	8.12 (7.14 to 9.1)	-10.7 (-22.1 to 0.6)
		Influenza	6760 (6190 to 7390)	4610 (2240 to 7830)	-31.8 (-66.6 to 15.9)	21.9 (20.6 to 23.4)	37.7 (16.2 to 71.4)	72.1 (-27.3 to 225.2)
		Klebsiella pneumoniae	3190 (2940 to 3500)	2840 (2520 to 3170)	-11.1 (-21.2 to 1.3)	14.5 (13.6 to 15.5)	29 (25.6 to 32.6)	100.2 (75.0 to 125.4)
		Legionella spp.	2440 (2180 to 2720)	2620 (2340 to 2920)	7.6 (-4.8 to 22.5)	7.53 (6.82 to 8.34)	18.6 (16.3 to 21)	147.8 (111.1 to 187.4)
		Mycoplasma spp.	5890 (5310 to 6460)	3760 (3370 to 4200)	-36.0 (-43.0 to -27.7)	7.13 (6.64 to 7.66)	9.44 (8.36 to 10.5)	32.5 (15.4 to 49.6)
		Polymicrobial	174 (113 to 271)	187 (115 to 299)	6.9 (-7.6 to 23.6)	1.58 (1.07 to 2.26)	2.12 (1.37 to 3.27)	36.1 (-10.4 to 89.8)
		Pseudomonas aeruginosa	3250 (2960 to 3590)	4310 (3840 to 4820)	33.0 (17.3 to 50.5)	10.3 (9.53 to 11.1)	30.5 (26.8 to 34.2)	195.7 (156.8 to 235.6)
		Respiratory syncytial virus	3360 (2950 to 3860)	1390 (684 to 2320)	-58.7 (-79.6 to -32.4)	8.1 (7.42 to 8.79)	3.68 (1.58 to 6.92)	-54.5 (-80.6 to -15.3)
		Staphylococcus aureus	4750 (4370 to 5160)	7130 (6380 to 7990)	50.3 (33.8 to 69.5)	28.4 (26.5 to 30.5)	105 (92.5 to 117)	269.2 (223.3 to 316.2)
		Streptococcus pneumoniae	28900 (26500 to 31500)	12100 (10800 to 13500)	-58.0 (-62.4 to -52.8)	75.9 (71.5 to 80.3)	70.5 (62.5 to 78.7)	-7.1 (-18.2 to 4.3)
		Other viruses	15300 (13900 to 16800)	10300 (9240 to 11600)	-32.3 (-39.9 to -23.7)	16.9 (15.9 to 18)	22.8 (20.2 to 25.3)	35.1 (19.6 to 51.3)
		Other bacterial pathogens	5060 (4510 to 5680)	4040 (3470 to 4670)	-20.1 (-29.2 to -9.8)	11.7 (10.4 to 13.1)	24.1 (20.6 to 28.1)	107.0 (79.6 to 135.7)
	Under 5	*Acinetobacter baumannii*	17.1 (12.3 to 23.8)	3.62 (2.46 to 5.25)	-78.7 (-84.1 to -72.3)	0.77 (0.44 to 1.27)	0.1 (0.06 to 0.18)	-86.6 (-89.3 to -83.8)
		Chlamydia spp.	314 (257 to 383)	66.5 (49.1 to 88.5)	-78.7 (-84.0 to -72.1)	0.98 (0.86 to 1.11)	0.11 (0.09 to 0.13)	-88.5 (-90.5 to -86.3)

		Enterobacter spp.	36.1 (26.4 to 48.4)	16.2 (11.1 to 23)	-54.7 (-66.6 to -40.5)	0.51 (0.35 to 0.75)	0.09 (0.06 to 0.14)	-81.8 (-85.0 to -77.9)
		Escherichia coli	120 (94.9 to 148)	53.6 (39.6 to 71.9)	-55.0 (-66.5 to -40.7)	1.49 (1.28 to 1.73)	0.26 (0.22 to 0.31)	-82.6 (-85.8 to -79.2)
		Fungus	25.2 (17.3 to 35.2)	8.83 (5.88 to 12.9)	-64.7 (-74.0 to -52.9)	0.13 (0.09 to 0.18)	0.02 (0.01 to 0.03)	-84.9 (-88.3 to -81.0)
		Group B Streptococcus	100 (78.8 to 126)	41.3 (30.2 to 56.3)	-58.5 (-69.4 to -45.3)	1.3 (1.14 to 1.49)	0.21 (0.18 to 0.25)	-83.6 (-86.4 to -80.3)
		Haemophilus influenzae	1020 (823 to 1240)	227 (169 to 305)	-77.6 (-83.0 to -70.7)	2.98 (2.64 to 3.38)	0.14 (0.12 to 0.17)	-95.2 (-96.1 to -94.1)
		Influenza	845 (691 to 1020)	149 (77.2 to 248)	-82.3 (-90.9 to -71.4)	4.41 (3.95 to 4.89)	0.35 (0.15 to 0.67)	-92.0 (-96.5 to -84.8)
		Klebsiella pneumoniae	466 (382 to 564)	99.2 (74.6 to 133)	-78.6 (-83.7 to -72.5)	3.57 (3.17 to 3.98)	0.39 (0.33 to 0.46)	-89.0 (-90.9 to -86.9)
		Legionella spp.	42.1 (28.1 to 62.2)	15.2 (9.48 to 23.4)	-63.7 (-72.9 to -51.5)	0.64 (0.48 to 0.84)	0.11 (0.08 to 0.15)	-82.5 (-85.9 to -79.0)
		Mycoplasma spp.	573 (467 to 693)	248 (186 to 332)	-56.5 (-66.9 to -43.3)	1.12 (0.99 to 1.26)	0.19 (0.16 to 0.23)	-82.8 (-85.8 to -79.3)
		Polymicrobial	10.5 (6.5 to 16)	3.19 (1.9 to 5.36)	-69.4 (-76.5 to -60.7)	0.91 (0.48 to 1.5)	0.16 (0.08 to 0.28)	-82.4 (-85.9 to -78.4)
		Pseudomonas aeruginosa	285 (230 to 350)	166 (124 to 220)	-41.4 (-56.4 to -23.7)	2.05 (1.82 to 2.28)	0.44 (0.37 to 0.51)	-78.7 (-82.4 to -74.5)
		Respiratory syncytial virus	2150 (1780 to 2580)	592 (300 to 991)	-72.5 (-86.1 to -56.5)	6.37 (5.69 to 7.04)	0.74 (0.32 to 1.41)	-88.4 (-95.1 to -78.0)
		Staphylococcus aureus	354 (292 to 426)	163 (121 to 219)	-53.8 (-64.3 to -40.3)	3.68 (3.3 to 4.07)	0.7 (0.6 to 0.83)	-80.8 (-84.0 to -76.9)
		Streptococcus pneumoniae	5570 (4610 to 6650)	550 (413 to 732)	-90.1 (-92.4 to -87.2)	18.6 (16.6 to 20.5)	0.8 (0.66 to 0.95)	-95.7 (-96.5 to -94.7)
		Other viruses	4550 (3750 to 5450)	936 (704 to 1250)	-79.4 (-84.1 to -73.5)	6.46 (5.75 to 7.14)	0.66 (0.55 to 0.76)	-89.8 (-91.5 to -87.8)
		Other bacterial pathogens	139 (105 to 179)	36.6 (25.9 to 50.6)	-73.5 (-80.4 to -64.8)	0.76 (0.57 to 1.02)	0.1 (0.07 to 0.14)	-87.1 (-89.5 to -84.5)
	5-14 years	*Acinetobacter baumannii*	77.2 (55.8 to 102)	23.4 (17.3 to 30.6)	-69.6 (-73.9 to -64.6)	0.06 (0.05 to 0.07)	0.01 (0.01 to 0.01)	-82.5 (-85.7 to -78.8)
		Chlamydia spp.	384 (283 to 511)	107 (80.2 to 140)	-72.2 (-75.9 to -67.9)	0.08 (0.07 to 0.09)	0.01 (0.01 to 0.01)	-84.0 (-86.9 to -80.3)
		Enterobacter spp.	39.5 (28.4 to 53.1)	23.9 (17.9 to 32)	-39.3 (-47.7 to -29.7)	0.03 (0.03 to 0.04)	0.01 (0.01 to 0.01)	-65.1 (-71.7 to -57.7)
		Escherichia coli	97.4 (73.2 to 128)	49.2 (36.9 to 64.3)	-49.4 (-56.1 to -41.2)	0.08 (0.07 to 0.09)	0.02 (0.02 to 0.03)	-70.9 (-76.0 to -64.3)
		Fungus	117 (85.5 to 156)	55.2 (40.7 to 73)	-52.8 (-59.2 to -45.7)	0.09 (0.08 to 0.1)	0.02 (0.02 to 0.03)	-72.9 (-77.7 to -66.7)
		Group B Streptococcus	125 (91.7 to 164)	66.3 (49.1 to 87.1)	-46.8 (-53.6 to -38.4)	0.07 (0.06 to 0.08)	0.02 (0.02 to 0.03)	-69.4 (-75.1 to -62.4)
		Haemophilus influenzae	518 (392 to 685)	171 (127 to 225)	-66.8 (-70.9 to -62.0)	0.15 (0.14 to 0.17)	0.02 (0.02 to 0.02)	-87.4 (-89.5 to -84.8)
		Influenza	727 (549 to 952)	227 (103 to 410)	-68.9 (-85.0 to -46.5)	0.42 (0.38 to 0.47)	0.08 (0.03 to 0.15)	-81.0 (-92.0 to -64.5)

		Klebsiella pneumoniae	286 (216 to 377)	111 (83.5 to 143)	-61.1 (-65.7 to -55.6)	0.22 (0.2 to 0.25)	0.05 (0.04 to 0.06)	-77.6 (-81.5 to -73.1)
		Legionella spp.	271 (202 to 359)	126 (94.5 to 164)	-53.4 (-59.5 to -46.2)	0.14 (0.12 to 0.17)	0.04 (0.03 to 0.05)	-73.2 (-77.9 to -66.9)
		Mycoplasma spp.	1140 (859 to 1490)	388 (292 to 504)	-65.8 (-69.6 to -60.8)	0.23 (0.21 to 0.26)	0.05 (0.04 to 0.05)	-80.3 (-83.9 to -76.3)
		Polymicrobial	15.6 (9.65 to 24.6)	7.07 (4.07 to 11.2)	-54.7 (-61.8 to -47.3)	0.01 (0.01 to 0.02)	0 (0 to 0.01)	-74.0 (-78.7 to -68.2)
		Pseudomonas aeruginosa	294 (222 to 387)	172 (130 to 225)	-41.2 (-48.7 to -32.5)	0.16 (0.14 to 0.18)	0.05 (0.04 to 0.06)	-66.2 (-72.1 to -59.1)
		Respiratory syncytial virus	212 (159 to 280)	66 (29.8 to 119)	-68.9 (-85.2 to -46.0)	0.06 (0.05 to 0.06)	0.01 (0 to 0.02)	-81.0 (-92.1 to -64.4)
		Staphylococcus aureus	441 (333 to 574)	283 (214 to 369)	-35.7 (-43.0 to -26.8)	0.48 (0.44 to 0.54)	0.18 (0.15 to 0.21)	-63.0 (-69.3 to -55.2)
		Streptococcus pneumoniae	4070 (3100 to 5340)	901 (682 to 1170)	-77.8 (-80.3 to -74.8)	1.86 (1.68 to 2.06)	0.24 (0.2 to 0.28)	-87.3 (-89.4 to -84.6)
		Other viruses	2030 (1530 to 2670)	794 (601 to 1030)	-60.7 (-65.1 to -55.1)	0.36 (0.33 to 0.4)	0.08 (0.07 to 0.1)	-77.4 (-81.2 to -72.9)
		Other bacterial pathogens	419 (314 to 557)	136 (101 to 178)	-67.5 (-71.6 to -63.0)	0.18 (0.16 to 0.21)	0.03 (0.03 to 0.04)	-81.3 (-84.6 to -77.5)
	15-49 years	*Acinetobacter baumannii*	110 (90.8 to 132)	52.7 (43.5 to 63)	-52.1 (-58.7 to -44.5)	0.81 (0.69 to 0.94)	0.65 (0.55 to 0.77)	-19.0 (-31.1 to -6.1)
		Chlamydia spp.	549 (475 to 631)	240 (206 to 276)	-56.2 (-61.8 to -50.2)	1 (0.9 to 1.11)	0.74 (0.63 to 0.84)	-25.9 (-37.4 to -14.4)
		Enterobacter spp.	56.4 (47.1 to 66.4)	53.7 (45.5 to 62.4)	-4.4 (-17.8 to 10.7)	0.39 (0.34 to 0.45)	0.63 (0.53 to 0.73)	61.6 (34.7 to 89.9)
		Escherichia coli	139 (120 to 161)	111 (96.2 to 127)	-20.3 (-30.9 to -8.6)	1 (0.9 to 1.11)	1.35 (1.17 to 1.53)	34.8 (14.7 to 57.2)
		Fungus	168 (143 to 196)	124 (106 to 145)	-25.7 (-35.8 to -14.5)	1.19 (1.04 to 1.36)	1.49 (1.27 to 1.72)	25.6 (6.7 to 45.6)
		Group B Streptococcus	179 (154 to 211)	149 (129 to 172)	-16.3 (-27.5 to -3.7)	0.91 (0.8 to 1.04)	1.29 (1.09 to 1.47)	41.6 (18.7 to 65.4)
		Haemophilus influenzae	740 (645 to 837)	386 (339 to 441)	-47.8 (-53.7 to -40.8)	2.03 (1.86 to 2.21)	1.18 (1.03 to 1.34)	-41.6 (-50.3 to -33.6)
		Influenza	1040 (916 to 1170)	509 (242 to 870)	-51.0 (-76.2 to -16.2)	5.58 (5.16 to 6.02)	4.92 (2.13 to 9.41)	-11.6 (-62.2 to 68.9)
		Klebsiella pneumoniae	408 (359 to 460)	250 (221 to 284)	-38.8 (-45.7 to -31.0)	2.92 (2.7 to 3.17)	3.02 (2.63 to 3.37)	3.5 (-10.8 to 18.1)
		Legionella spp.	387 (335 to 444)	283 (246 to 325)	-26.6 (-36.1 to -15.4)	1.91 (1.7 to 2.14)	2.36 (2.06 to 2.66)	24.1 (4.9 to 44.9)
		Mycoplasma spp.	1620 (1430 to 1830)	874 (771 to 994)	-46.1 (-52.5 to -38.7)	3.09 (2.83 to 3.37)	2.81 (2.45 to 3.18)	-8.8 (-22.5 to 4.5)
		Polymicrobial	22.4 (13.9 to 34.8)	15.9 (9.28 to 25.4)	-28.8 (-39.3 to -17.0)	0.16 (0.1 to 0.25)	0.2 (0.12 to 0.31)	20.5 (1.9 to 41.0)
		Pseudomonas aeruginosa	420 (368 to 477)	388 (342 to 440)	-7.4 (-18.6 to 4.8)	2.08 (1.91 to 2.29)	3.26 (2.85 to 3.64)	56.6 (33.2 to 80.9)
		Respiratory syncytial virus	303 (261 to 342)	148 (71.2 to 255)	-51.0 (-76.6 to -15.0)	0.74 (0.67 to 0.82)	0.65 (0.28 to 1.22)	-11.8 (-62.8 to 65.6)

		Staphylococcus aureus	630 (555 to 708)	637 (560 to 718)	1.2 (-10.1 to 13.9)	6.41 (5.92 to 6.91)	11 (9.63 to 12.2)	71.2 (47.2 to 95.4)
		Streptococcus pneumoniae	5820 (5150 to 6510)	2030 (1790 to 2290)	-65.1 (-68.9 to -60.9)	24.6 (22.9 to 26.4)	14.5 (12.7 to 16.2)	-41.0 (-49.2 to -32.7)
		Other viruses	2900 (2560 to 3250)	1790 (1580 to 2020)	-38.2 (-44.9 to -30.8)	4.82 (4.47 to 5.18)	5.03 (4.4 to 5.65)	4.5 (-10.3 to 18.8)
		Other bacterial pathogens	600 (508 to 691)	306 (259 to 358)	-48.8 (-55.0 to -41.9)	2.42 (2.14 to 2.71)	2.09 (1.76 to 2.45)	-13.4 (-25.6 to -0.2)
	50-69 years	*Acinetobacter baumannii*	360 (303 to 420)	272 (226 to 314)	-24.3 (-34.3 to -12.8)	1.76 (1.5 to 2.08)	2.83 (2.36 to 3.35)	61.2 (31.0 to 93.5)
		Chlamydia spp.	1060 (931 to 1200)	706 (607 to 801)	-33.2 (-42.0 to -23.4)	1.29 (1.13 to 1.47)	1.82 (1.53 to 2.14)	42.2 (16.8 to 71.6)
		Enterobacter spp.	178 (152 to 209)	264 (228 to 303)	48.6 (29.2 to 71.9)	0.83 (0.71 to 0.96)	2.61 (2.19 to 3.06)	216.4 (157.3 to 282.7)
		Escherichia coli	487 (427 to 554)	591 (520 to 666)	21.7 (6.8 to 41.4)	2.33 (2.05 to 2.64)	6.03 (5.13 to 6.98)	159.3 (110.7 to 209.2)
		Fungus	466 (399 to 534)	539 (464 to 619)	16.0 (0.2 to 33.7)	2.2 (1.89 to 2.54)	5.43 (4.56 to 6.42)	147.1 (101.5 to 196.3)
		Group B Streptococcus	400 (347 to 460)	513 (445 to 579)	28.5 (11.5 to 48.6)	1.36 (1.17 to 1.58)	3.71 (3.13 to 4.32)	173.8 (120.7 to 229.7)
		Haemophilus influenzae	1410 (1260 to 1560)	1200 (1060 to 1340)	-14.8 (-24.5 to -3.2)	2.59 (2.31 to 2.91)	3.09 (2.63 to 3.6)	19.9 (-1.7 to 41.7)
		Influenza	1710 (1530 to 1890)	1330 (636 to 2280)	-22.3 (-62.9 to 35.6)	6.13 (5.49 to 6.85)	10.8 (4.51 to 20.6)	76.0 (-27.1 to 234.8)
		Klebsiella pneumoniae	999 (893 to 1110)	1030 (907 to 1150)	2.9 (-8.9 to 17.7)	4.77 (4.26 to 5.32)	10.4 (8.89 to 12.1)	119.2 (79.5 to 160.8)
		Legionella spp.	1040 (902 to 1180)	1180 (1040 to 1320)	14.0 (-0.3 to 31.7)	3.42 (2.97 to 3.91)	8.26 (6.99 to 9.65)	142.9 (96.2 to 195.8)
		Mycoplasma spp.	1420 (1260 to 1580)	1120 (985 to 1260)	-21.1 (-30.3 to -9.4)	1.8 (1.6 to 2.05)	3.02 (2.58 to 3.5)	68.0 (36.3 to 103.3)
		Polymicrobial	60.7 (38.3 to 94.2)	67.8 (40.9 to 109)	11.7 (-3.1 to 31.1)	0.3 (0.18 to 0.46)	0.7 (0.43 to 1.12)	137.9 (94.4 to 189.6)
		Pseudomonas aeruginosa	1140 (1010 to 1270)	1600 (1410 to 1790)	40.9 (23.8 to 61.3)	3.77 (3.35 to 4.24)	11.3 (9.62 to 13.1)	200.1 (145.8 to 260.9)
		Respiratory syncytial virus	369 (324 to 418)	275 (131 to 475)	-25.2 (-64.8 to 30.0)	0.6 (0.53 to 0.7)	1.02 (0.42 to 1.94)	69.4 (-30.7 to 218.9)
		Staphylococcus aureus	1500 (1350 to 1650)	2380 (2100 to 2650)	58.6 (40.2 to 79.5)	10.2 (9.11 to 11.4)	34.3 (29.3 to 39.7)	237.7 (176.3 to 302.0)
		Streptococcus pneumoniae	6850 (6150 to 7530)	3880 (3430 to 4310)	-43.2 (-49.4 to -35.1)	19.3 (17.4 to 21.6)	23.3 (19.9 to 26.9)	20.9 (-0.7 to 43.9)
		Other viruses	2980 (2680 to 3300)	3080 (2730 to 3430)	3.5 (-7.9 to 17.4)	3.31 (2.96 to 3.7)	7.27 (6.21 to 8.43)	120.5 (81.2 to 161.6)
		Other bacterial pathogens	1730 (1500 to 1970)	1370 (1170 to 1580)	-20.6 (-29.9 to -9.9)	4.66 (4.09 to 5.36)	7.86 (6.47 to 9.4)	69.2 (38.6 to 102.8)
	70+ years	*Acinetobacter baumannii*	337 (284 to 409)	324 (269 to 389)	-3.7 (-17.9 to 12.4)	1.02 (0.87 to 1.18)	3.73 (3.16 to 4.37)	266.6 (216.2 to 321.1)
		Chlamydia spp.	1180 (1030 to 1370)	1000 (854 to 1160)	-15.0 (-27.3 to -1.0)	0.89 (0.79 to 1)	2.87 (2.45 to 3.28)	223.5 (179.1 to 270.2)

		Enterobacter spp.	163 (136 to 193)	307 (253 to 359)	89.0 (59.9 to 123.6)	0.47 (0.4 to 0.54)	3.35 (2.83 to 3.88)	619.7 (522.8 to 735.7)
		Escherichia coli	561 (486 to 655)	866 (741 to 1000)	54.9 (31.0 to 82.0)	1.66 (1.49 to 1.87)	9.78 (8.44 to 11.1)	489.7 (408.8 to 573.5)
		Fungus	244 (206 to 291)	359 (299 to 422)	47.6 (24.7 to 72.5)	0.71 (0.62 to 0.81)	3.99 (3.41 to 4.63)	461.9 (383.7 to 541.5)
		Group B Streptococcus	345 (297 to 406)	561 (483 to 647)	63.5 (39.4 to 90.2)	0.72 (0.63 to 0.82)	4.5 (3.87 to 5.1)	522.6 (432.3 to 622.7)
		Haemophilus influenzae	1200 (1060 to 1370)	1290 (1110 to 1500)	8.3 (-5.6 to 25.5)	1.35 (1.23 to 1.49)	3.68 (3.17 to 4.15)	172.7 (139.1 to 210.8)
		Influenza	2440 (2170 to 2780)	2400 (1180 to 4090)	-1.4 (-51.7 to 71.0)	5.4 (4.92 to 5.89)	21.6 (9.26 to 40.8)	300.9 (69.5 to 663.2)
		Klebsiella pneumoniae	1030 (919 to 1190)	1350 (1160 to 1550)	30.9 (13.6 to 51.1)	3.05 (2.78 to 3.32)	15.2 (13.1 to 17)	398.5 (335.7 to 462.1)
		Legionella spp.	702 (614 to 819)	1010 (871 to 1170)	45.0 (23.9 to 68.1)	1.42 (1.26 to 1.6)	7.85 (6.76 to 8.87)	452.4 (374.9 to 537.2)
		Mycoplasma spp.	1130 (998 to 1310)	1130 (971 to 1300)	0.3 (-13.6 to 16.7)	0.88 (0.79 to 0.98)	3.37 (2.89 to 3.81)	281.9 (231.5 to 334.5)
		Polymicrobial	65.3 (40 to 106)	92.7 (55.9 to 149)	42.1 (18.4 to 68.0)	0.2 (0.12 to 0.31)	1.06 (0.64 to 1.7)	441.1 (367.6 to 527.0)
		Pseudomonas aeruginosa	1110 (980 to 1280)	1990 (1720 to 2300)	79.2 (54.1 to 108.2)	2.27 (2.06 to 2.49)	15.5 (13.3 to 17.2)	582.5 (494.8 to 678.1)
		Respiratory syncytial virus	325 (282 to 379)	307 (152 to 529)	-5.0 (-53.8 to 63.6)	0.33 (0.29 to 0.37)	1.26 (0.54 to 2.41)	285.8 (64.4 to 623.1)
		Staphylococcus aureus	1820 (1620 to 2080)	3670 (3170 to 4240)	101.7 (75.2 to 132.4)	7.64 (6.98 to 8.31)	58.6 (50.6 to 65.7)	668.1 (578.0 to 763.4)
		Streptococcus pneumoniae	6610 (5890 to 7500)	4760 (4140 to 5480)	-27.8 (-37.3 to -17.0)	11.5 (10.6 to 12.6)	31.7 (27.4 to 35.5)	174.9 (141.9 to 208.8)
		Other viruses	2850 (2530 to 3240)	3740 (3230 to 4310)	31.7 (14.4 to 52.6)	1.95 (1.78 to 2.13)	9.77 (8.46 to 11)	401.4 (339.7 to 463.0)
		Other bacterial pathogens	2170 (1870 to 2540)	2190 (1840 to 2580)	1.0 (-13.1 to 17.3)	3.65 (3.2 to 4.14)	14 (11.8 to 16.5)	284.9 (234.4 to 339.7)
Republic of Moldova	All Ages	*Acinetobacter baumannii*	1860 (1620 to 2140)	1030 (908 to 1160)	-44.6 (-49.8 to -39.3)	25.1 (19.8 to 31.6)	16.6 (14.1 to 19.3)	-33.0 (-47.5 to -16.0)
		Chlamydia spp.	6560 (5990 to 7240)	3300 (3030 to 3570)	-49.6 (-54.4 to -45.1)	23.4 (21.3 to 25.7)	12.9 (11.3 to 14.6)	-44.9 (-53.1 to -36.1)
		Enterobacter spp.	698 (603 to 810)	730 (645 to 810)	4.8 (-6.0 to 15.5)	9.88 (7.85 to 12.7)	10.9 (9.28 to 12.5)	11.4 (-15.1 to 39.9)
		Escherichia coli	2210 (1990 to 2480)	1950 (1780 to 2130)	-11.6 (-19.9 to -3.5)	31.6 (28.3 to 35.6)	29.5 (25.8 to 33.7)	-6.3 (-20.2 to 8.8)
		Fungus	1710 (1490 to 1960)	1470 (1310 to 1640)	-13.5 (-22.1 to -4.4)	13.9 (12.2 to 15.6)	22.1 (18.8 to 25.4)	59.4 (37.4 to 83.7)
		Group B Streptococcus	1870 (1660 to 2120)	1620 (1470 to 1760)	-13.5 (-21.9 to -5.3)	23.8 (21.3 to 26.8)	18.2 (15.9 to 20.9)	-23.3 (-36.2 to -10.4)
		Haemophilus influenzae	8460 (7700 to 9440)	4690 (4320 to 5050)	-44.5 (-49.5 to -39.5)	47 (41.8 to 52.7)	17.8 (15.7 to 20.2)	-62.0 (-68.1 to -55.3)
		Influenza	10500 (9690 to 11500)	1100 (373 to 2180)	-89.5 (-96.4 to -79.1)	91.9 (84.8 to 99.8)	11.7 (4.1 to 24.2)	-87.2 (-95.6 to -73.4)

		Klebsiella pneumoniae	5580 (5140 to 6150)	3630 (3350 to 3910)	-34.9 (-40.4 to -29.2)	71.7 (65.4 to 78.8)	54 (47.2 to 61)	-24.5 (-36.0 to -13.0)
		Legionella spp.	3100 (2770 to 3490)	3170 (2900 to 3450)	2.4 (-7.7 to 13.0)	22.6 (20 to 25.8)	33.4 (29.2 to 37.9)	48.2 (25.8 to 73.4)
		Mycoplasma spp.	10000 (9210 to 11100)	5860 (5410 to 6300)	-41.6 (-46.5 to -36.8)	28.4 (26.1 to 31.2)	22.7 (19.9 to 25.7)	-20.2 (-31.5 to -8.7)
		Polymicrobial	320 (221 to 463)	240 (152 to 374)	-25.4 (-35.2 to -15.6)	12.3 (7.44 to 18.9)	4.85 (3.33 to 7)	-59.3 (-73.1 to -40.5)
		Pseudomonas aeruginosa	4360 (3990 to 4810)	4610 (4250 to 4960)	5.7 (-3.6 to 15.0)	39.6 (36.2 to 43.4)	47.7 (42.1 to 54.1)	20.9 (2.6 to 39.1)
		Respiratory syncytial virus	6940 (6080 to 7940)	384 (129 to 755)	-94.4 (-98.1 to -89.0)	62.3 (54.6 to 71.2)	1.94 (0.66 to 3.97)	-96.9 (-99.0 to -93.6)
		Staphylococcus aureus	6680 (6210 to 7270)	7350 (6800 to 7920)	10.2 (1.1 to 19.0)	98.7 (92.1 to 106)	152 (135 to 172)	54.5 (33.3 to 76.6)
		Streptococcus pneumoniae	52500 (48600 to 57700)	20000 (18600 to 21400)	-61.9 (-65.1 to -58.6)	342 (315 to 371)	170 (151 to 191)	-50.0 (-57.2 to -42.8)
		Other viruses	25400 (23200 to 28300)	13600 (12600 to 14500)	-46.5 (-51.6 to -41.6)	79.4 (71.9 to 87.8)	45.6 (40.3 to 51.2)	-42.4 (-51.3 to -33.3)
		Other bacterial pathogens	7550 (6820 to 8520)	5380 (4790 to 5990)	-28.7 (-34.8 to -22.4)	40.3 (36.2 to 45.3)	44.8 (38.5 to 51.7)	11.1 (-4.7 to 28.8)
	Under 5	*Acinetobacter baumannii*	85.2 (61.9 to 118)	8.51 (6.19 to 11.6)	-90.0 (-91.4 to -88.4)	10.7 (5.84 to 17.1)	1.02 (0.53 to 1.84)	-90.5 (-93.2 to -86.9)
		Chlamydia spp.	1180 (989 to 1420)	146 (119 to 178)	-87.6 (-89.6 to -85.3)	12.9 (11 to 14.9)	1.08 (0.79 to 1.47)	-91.6 (-94.0 to -88.5)
		Enterobacter spp.	110 (82.4 to 143)	25.3 (18.9 to 33.1)	-76.9 (-81.0 to -72.3)	5.39 (3.52 to 8.24)	0.69 (0.41 to 1.07)	-87.1 (-90.9 to -82.0)
		Escherichia coli	398 (320 to 486)	91.1 (74.7 to 111)	-77.0 (-80.8 to -72.9)	17.2 (14.3 to 20.8)	2.03 (1.46 to 2.77)	-88.1 (-91.6 to -83.7)
		Fungus	80.9 (55.8 to 114)	15.2 (10.5 to 21.3)	-81.1 (-84.3 to -77.0)	1.34 (0.94 to 1.86)	0.12 (0.08 to 0.19)	-90.6 (-93.5 to -86.9)
		Group B Streptococcus	330 (266 to 404)	68.9 (55.7 to 84.6)	-79.0 (-82.4 to -75.3)	15.2 (12.9 to 18)	1.76 (1.31 to 2.4)	-88.4 (-91.7 to -84.2)
		Haemophilus influenzae	2990 (2480 to 3590)	429 (354 to 514)	-85.6 (-87.8 to -83.1)	30.9 (26 to 36.5)	1.19 (0.88 to 1.61)	-96.1 (-97.2 to -94.7)
		Influenza	2530 (2130 to 2970)	53 (18.2 to 103)	-97.9 (-99.3 to -95.8)	46.1 (39.7 to 53.2)	0.5 (0.16 to 1.03)	-98.9 (-99.7 to -97.7)
		Klebsiella pneumoniae	1520 (1260 to 1810)	184 (151 to 223)	-87.9 (-89.6 to -85.9)	40.1 (34.6 to 46.8)	3.15 (2.33 to 4.33)	-92.1 (-94.3 to -89.2)
		Legionella spp.	118 (82 to 172)	25.1 (16.7 to 37.1)	-78.8 (-82.1 to -74.8)	6.66 (4.78 to 9.18)	0.89 (0.61 to 1.29)	-86.6 (-90.4 to -81.4)
		Mycoplasma spp.	1720 (1440 to 2060)	452 (375 to 543)	-73.7 (-77.5 to -69.1)	12.2 (10.4 to 14.4)	1.45 (1.07 to 1.95)	-88.0 (-91.5 to -83.8)
		Polymicrobial	47.9 (30.5 to 71.9)	6.72 (4.14 to 10.2)	-86.0 (-88.0 to -83.8)	10.1 (5.41 to 16.5)	1.35 (0.66 to 2.33)	-86.6 (-90.4 to -81.4)
		Pseudomonas aeruginosa	770 (638 to 920)	243 (201 to 293)	-68.3 (-72.8 to -62.9)	20.1 (17.2 to 23.5)	2.93 (2.18 to 3.93)	-85.4 (-89.6 to -80.0)
		Respiratory syncytial virus	5430 (4580 to 6370)	188 (64.7 to 365)	-96.5 (-98.8 to -93.2)	58.5 (50.8 to 67.4)	0.96 (0.31 to 2.01)	-98.4 (-99.5 to -96.5)

		Staphylococcus aureus	1020 (858 to 1210)	245 (202 to 294)	-76.0 (-79.2 to -72.4)	36.2 (31 to 41.9)	4.51 (3.35 to 6.03)	-87.5 (-91.0 to -83.0)
		Streptococcus pneumoniae	17000 (14300 to 19900)	1220 (1010 to 1470)	-92.8 (-93.7 to -91.7)	184 (160 to 212)	6.9 (5.09 to 9.27)	-96.2 (-97.3 to -94.9)
		Other viruses	11600 (9790 to 13500)	1590 (1320 to 1920)	-86.2 (-88.0 to -84.3)	55.5 (48.2 to 64)	4.52 (3.36 to 6.07)	-91.8 (-94.1 to -88.9)
		Other bacterial pathogens	463 (361 to 587)	72.1 (56.1 to 93.7)	-84.4 (-86.7 to -81.2)	8.85 (6.43 to 12.2)	0.82 (0.53 to 1.25)	-90.7 (-93.3 to -87.2)
	5-14 years	*Acinetobacter baumannii*	224 (172 to 285)	38.4 (29.5 to 48.5)	-82.8 (-84.9 to -80.5)	0.36 (0.3 to 0.43)	0.07 (0.05 to 0.08)	-80.8 (-84.4 to -76.2)
		Chlamydia spp.	928 (721 to 1160)	173 (136 to 216)	-81.3 (-83.6 to -78.8)	0.37 (0.31 to 0.43)	0.08 (0.06 to 0.09)	-79.1 (-83.0 to -74.3)
		Enterobacter spp.	76.1 (57.3 to 99.4)	27.5 (21.3 to 35.4)	-63.7 (-68.5 to -58.7)	0.12 (0.09 to 0.14)	0.05 (0.04 to 0.06)	-59.4 (-67.0 to -49.6)
		Escherichia coli	208 (160 to 260)	62.7 (49 to 78.7)	-69.7 (-73.1 to -65.9)	0.32 (0.27 to 0.38)	0.11 (0.09 to 0.13)	-66.1 (-72.4 to -58.0)
		Fungus	251 (194 to 319)	71.8 (55.8 to 91.2)	-71.3 (-74.8 to -67.4)	0.38 (0.32 to 0.46)	0.12 (0.1 to 0.15)	-67.9 (-74.0 to -59.9)
		Group B Streptococcus	254 (193 to 321)	81.1 (63.7 to 102)	-68.0 (-71.8 to -64.1)	0.28 (0.23 to 0.34)	0.1 (0.08 to 0.12)	-64.2 (-71.2 to -55.2)
		Haemophilus influenzae	997 (797 to 1240)	247 (195 to 307)	-75.2 (-78.0 to -72.1)	0.59 (0.51 to 0.68)	0.11 (0.09 to 0.13)	-81.0 (-84.4 to -76.5)
		Influenza	1430 (1140 to 1770)	58.8 (19.2 to 120)	-95.9 (-98.5 to -91.9)	1.67 (1.45 to 1.91)	0.08 (0.02 to 0.16)	-95.5 (-98.5 to -90.5)
		Klebsiella pneumoniae	611 (486 to 755)	155 (123 to 193)	-74.6 (-77.5 to -71.6)	0.95 (0.82 to 1.09)	0.27 (0.22 to 0.31)	-71.6 (-77.0 to -64.9)
		Legionella spp.	454 (355 to 576)	151 (119 to 190)	-66.6 (-70.6 to -62.4)	0.48 (0.41 to 0.58)	0.18 (0.15 to 0.21)	-62.6 (-69.9 to -53.8)
		Mycoplasma spp.	2240 (1750 to 2790)	541 (427 to 674)	-75.8 (-78.5 to -72.8)	0.92 (0.79 to 1.06)	0.25 (0.21 to 0.29)	-72.9 (-78.1 to -66.2)
		Polymicrobial	37.8 (24.3 to 57.3)	9.77 (5.82 to 15.2)	-74.2 (-77.7 to -70.2)	0.06 (0.04 to 0.09)	0.02 (0.01 to 0.03)	-71.2 (-76.8 to -63.9)
		Pseudomonas aeruginosa	511 (401 to 627)	191 (150 to 238)	-62.5 (-66.9 to -57.8)	0.55 (0.47 to 0.64)	0.23 (0.19 to 0.27)	-58.0 (-65.8 to -48.3)
		Respiratory syncytial virus	347 (271 to 436)	15.6 (5.15 to 32.1)	-95.5 (-98.4 to -91.0)	0.18 (0.16 to 0.21)	0.01 (0 to 0.02)	-95.0 (-98.4 to -89.6)
		Staphylococcus aureus	837 (663 to 1030)	319 (253 to 395)	-61.9 (-66.2 to -57.4)	1.84 (1.6 to 2.11)	0.78 (0.66 to 0.91)	-57.3 (-65.1 to -47.3)
		Streptococcus pneumoniae	8230 (6590 to 10100)	1470 (1170 to 1830)	-82.1 (-84.1 to -79.9)	7.54 (6.57 to 8.59)	1.5 (1.27 to 1.75)	-80.0 (-83.7 to -75.2)
		Other viruses	3410 (2740 to 4200)	1010 (803 to 1260)	-70.2 (-73.6 to -66.7)	1.23 (1.06 to 1.42)	0.41 (0.35 to 0.48)	-66.6 (-72.9 to -58.8)
		Other bacterial pathogens	913 (718 to 1150)	202 (159 to 254)	-77.8 (-80.4 to -75.0)	0.8 (0.68 to 0.93)	0.2 (0.16 to 0.24)	-75.1 (-79.8 to -69.3)
	15-49 years	*Acinetobacter baumannii*	283 (239 to 335)	139 (119 to 161)	-50.7 (-56.0 to -45.1)	3.14 (2.68 to 3.69)	2.56 (2.16 to 3.02)	-18.2 (-30.5 to -5.0)
		Chlamydia spp.	1170 (1030 to 1340)	626 (552 to 703)	-46.5 (-51.6 to -40.5)	3.23 (2.85 to 3.63)	2.86 (2.46 to 3.29)	-11.2 (-24.1 to 2.1)

		Enterobacter spp.	96.1 (80.7 to 115)	99.7 (85 to 114)	4.0 (-7.2 to 16.2)	1.01 (0.86 to 1.19)	1.74 (1.47 to 2.04)	72.7 (47.0 to 102.1)
		Escherichia coli	262 (230 to 298)	227 (202 to 255)	-13.1 (-21.8 to -3.5)	2.85 (2.51 to 3.22)	4.1 (3.58 to 4.71)	44.2 (22.9 to 67.5)
		Fungus	316 (269 to 371)	260 (228 to 297)	-17.6 (-26.6 to -8.0)	3.39 (2.92 to 3.9)	4.63 (3.94 to 5.36)	36.7 (16.8 to 58.9)
		Group B Streptococcus	321 (277 to 373)	294 (258 to 330)	-8.2 (-17.8 to 2.4)	2.48 (2.16 to 2.83)	3.76 (3.26 to 4.31)	52.4 (29.2 to 78.1)
		Haemophilus influenzae	1260 (1120 to 1410)	894 (799 to 991)	-28.8 (-35.3 to -21.1)	5.22 (4.71 to 5.78)	4.21 (3.72 to 4.78)	-19.1 (-30.9 to -6.9)
		Influenza	1810 (1640 to 2010)	213 (73.3 to 427)	-88.2 (-95.9 to -75.8)	14.7 (13.4 to 16)	2.83 (0.96 to 5.95)	-80.7 (-93.5 to -59.3)
		Klebsiella pneumoniae	771 (697 to 860)	561 (502 to 619)	-27.2 (-33.8 to -19.8)	8.34 (7.64 to 9.16)	10.1 (8.84 to 11.3)	20.8 (3.6 to 39.9)
		Legionella spp.	573 (500 to 657)	548 (487 to 608)	-4.3 (-14.3 to 6.6)	4.27 (3.75 to 4.85)	6.76 (5.94 to 7.71)	58.9 (35.3 to 84.7)
		Mycoplasma spp.	2820 (2540 to 3160)	1960 (1760 to 2170)	-30.5 (-36.9 to -23.4)	8.11 (7.36 to 8.93)	9.34 (8.19 to 10.6)	15.4 (-1.3 to 33.1)
		Polymicrobial	47.8 (30.9 to 70.9)	35.4 (21.8 to 55.1)	-26.1 (-34.7 to -16.2)	0.53 (0.34 to 0.79)	0.65 (0.4 to 1)	22.6 (3.0 to 43.3)
		Pseudomonas aeruginosa	645 (574 to 720)	693 (618 to 767)	7.7 (-2.5 to 19.1)	4.84 (4.37 to 5.35)	8.63 (7.6 to 9.84)	78.7 (53.2 to 107.3)
		Respiratory syncytial virus	438 (387 to 491)	56.6 (19.5 to 113)	-87.1 (-95.6 to -73.9)	1.62 (1.46 to 1.81)	0.34 (0.12 to 0.72)	-78.8 (-92.8 to -55.2)
		Staphylococcus aureus	1060 (958 to 1170)	1150 (1030 to 1270)	9.4 (-0.3 to 20.5)	16.2 (15 to 17.8)	29.4 (26 to 33.2)	81.5 (55.9 to 109.4)
		Streptococcus pneumoniae	10400 (9490 to 11400)	5320 (4820 to 5870)	-48.7 (-53.2 to -43.4)	66.4 (61.3 to 72.2)	56.5 (50 to 63.6)	-14.8 (-27.0 to -2.0)
		Other viruses	4300 (3930 to 4720)	3680 (3320 to 4050)	-14.5 (-21.9 to -5.8)	10.8 (9.99 to 11.8)	15.3 (13.6 to 17.3)	41.9 (22.0 to 63.2)
		Other bacterial pathogens	1150 (1020 to 1310)	733 (643 to 828)	-36.4 (-42.5 to -29.6)	7.02 (6.23 to 7.94)	7.41 (6.34 to 8.61)	5.7 (-9.2 to 22.3)
	50-69 years	*Acinetobacter baumannii*	908 (778 to 1060)	542 (463 to 624)	-40.1 (-46.9 to -32.9)	7.78 (6.74 to 8.97)	9.08 (7.59 to 10.8)	17.0 (-1.0 to 38.9)
		Chlamydia spp.	2220 (1970 to 2510)	1420 (1250 to 1600)	-36.1 (-43.3 to -28.4)	4.72 (4.2 to 5.33)	5.89 (5.05 to 6.84)	24.9 (6.2 to 46.6)
		Enterobacter spp.	299 (254 to 354)	374 (325 to 427)	25.4 (10.0 to 41.2)	2.43 (2.04 to 2.83)	5.93 (4.96 to 6.95)	145.1 (107.4 to 189.5)
		Escherichia coli	900 (792 to 1020)	932 (823 to 1040)	3.8 (-7.2 to 15.7)	7.57 (6.69 to 8.48)	15.3 (13.1 to 17.5)	102.8 (71.8 to 140.0)
		Fungus	865 (741 to 1000)	859 (747 to 976)	-0.5 (-11.9 to 12.2)	7.16 (6.16 to 8.18)	13.9 (11.6 to 16.2)	94.5 (63.5 to 128.9)
		Group B Streptococcus	706 (613 to 814)	775 (680 to 877)	10.0 (-2.8 to 23.2)	4.21 (3.67 to 4.77)	9.03 (7.78 to 10.6)	115.0 (80.6 to 153.5)
		Haemophilus influenzae	2360 (2130 to 2650)	2070 (1850 to 2300)	-12.2 (-21.5 to -2.5)	7.58 (6.87 to 8.41)	8.88 (7.64 to 10.3)	17.5 (0.2 to 37.1)
		Influenza	2930 (2650 to 3260)	420 (141 to 843)	-85.6 (-95.1 to -71.1)	18.4 (16.7 to 20.1)	5.06 (1.78 to 10.5)	-72.4 (-90.4 to -42.9)

		Klebsiella pneumoniae	1860 (1680 to 2060)	1690 (1500 to 1860)	-8.8 (-17.8 to 1.6)	15.5 (14.1 to 17)	27.6 (23.7 to 31.7)	78.2 (52.1 to 107.6)
		Legionella spp.	1510 (1330 to 1720)	1740 (1540 to 1940)	15.4 (2.4 to 29.5)	8.71 (7.74 to 9.85)	19.6 (16.7 to 22.6)	125.5 (91.6 to 164.0)
		Mycoplasma spp.	2430 (2180 to 2730)	1970 (1750 to 2200)	-18.8 (-27.3 to -9.7)	5.39 (4.9 to 5.99)	8.55 (7.35 to 9.86)	58.7 (35.1 to 85.0)
		Polymicrobial	127 (85.8 to 188)	114 (71.8 to 180)	-10.5 (-22.2 to 1.4)	1.08 (0.71 to 1.61)	1.9 (1.19 to 2.97)	74.9 (45.4 to 105.9)
		Pseudomonas aeruginosa	1720 (1540 to 1930)	2200 (1950 to 2430)	28.3 (14.0 to 43.6)	9.96 (9.09 to 10.9)	24.9 (21.5 to 28.6)	150.7 (114.8 to 193.6)
		Respiratory syncytial virus	525 (466 to 596)	81 (26.7 to 163)	-84.5 (-94.7 to -68.9)	1.5 (1.33 to 1.69)	0.45 (0.16 to 0.92)	-70.3 (-89.8 to -39.3)
		Staphylococcus aureus	2480 (2250 to 2740)	3270 (2920 to 3600)	32.3 (18.8 to 47.2)	29.4 (27 to 32)	75.8 (65.7 to 87)	158.6 (120.2 to 201.9)
		Streptococcus pneumoniae	12000 (11000 to 13200)	7600 (6780 to 8350)	-36.6 (-42.9 to -29.6)	59.3 (54.6 to 64.4)	73.4 (63.5 to 84.1)	23.9 (6.0 to 44.2)
		Other viruses	4360 (3960 to 4830)	4650 (4150 to 5130)	6.9 (-4.4 to 18.6)	8.46 (7.74 to 9.25)	17.7 (15.3 to 20.2)	109.0 (78.5 to 142.8)
		Other bacterial pathogens	3270 (2890 to 3720)	2500 (2170 to 2850)	-23.4 (-31.6 to -14.9)	15.4 (13.6 to 17.3)	23 (19.4 to 27.1)	49.9 (27.6 to 76.3)
	70+ years	*Acinetobacter baumannii*	364 (300 to 440)	303 (257 to 353)	-16.6 (-26.5 to -5.6)	3.08 (2.66 to 3.6)	3.86 (3.2 to 4.6)	25.5 (6.3 to 46.9)
		Chlamydia spp.	1060 (903 to 1250)	943 (821 to 1080)	-10.9 (-21.5 to 1.6)	2.23 (1.98 to 2.51)	2.99 (2.53 to 3.43)	34.0 (14.4 to 56.3)
		Enterobacter spp.	117 (94.6 to 144)	203 (172 to 239)	74.7 (54.0 to 100.1)	0.94 (0.79 to 1.09)	2.46 (2.04 to 2.88)	162.9 (122.6 to 210.9)
		Escherichia coli	444 (377 to 529)	640 (558 to 732)	44.6 (27.1 to 64.7)	3.69 (3.23 to 4.18)	8 (6.89 to 9.23)	117.5 (85.2 to 153.8)
		Fungus	194 (160 to 236)	267 (229 to 313)	38.7 (22.2 to 58.0)	1.58 (1.35 to 1.81)	3.3 (2.77 to 3.85)	108.6 (76.5 to 145.2)
		Group B Streptococcus	260 (220 to 307)	397 (345 to 452)	53.2 (35.2 to 75.4)	1.53 (1.33 to 1.75)	3.53 (3.02 to 4.06)	130.6 (95.5 to 171.4)
		Haemophilus influenzae	855 (740 to 1000)	1040 (916 to 1180)	22.3 (8.2 to 38.6)	2.71 (2.43 to 3)	3.41 (2.95 to 3.89)	26.0 (7.7 to 47.1)
		Influenza	1790 (1560 to 2070)	356 (122 to 697)	-80.0 (-93.0 to -59.9)	11.1 (10.1 to 12.2)	3.28 (1.16 to 6.75)	-70.3 (-89.9 to -39.1)
		Klebsiella pneumoniae	821 (715 to 962)	1040 (914 to 1170)	27.0 (12.7 to 43.6)	6.78 (6.14 to 7.44)	12.9 (11.1 to 14.9)	91.1 (63.6 to 122.6)
		Legionella spp.	437 (373 to 513)	700 (610 to 797)	60.7 (41.7 to 81.3)	2.48 (2.2 to 2.83)	5.99 (5.13 to 6.84)	141.8 (104.8 to 180.6)
		Mycoplasma spp.	826 (713 to 968)	932 (822 to 1050)	13.2 (-0.4 to 27.8)	1.81 (1.62 to 2.01)	3.08 (2.62 to 3.52)	70.2 (44.5 to 98.6)
		Polymicrobial	58.7 (38.3 to 92.4)	73.3 (44.8 to 119)	24.7 (8.4 to 45.1)	0.49 (0.32 to 0.75)	0.93 (0.57 to 1.51)	87.6 (56.8 to 120.1)
		Pseudomonas aeruginosa	718 (621 to 836)	1280 (1120 to 1440)	78.7 (58.0 to 102.1)	4.11 (3.69 to 4.56)	11 (9.52 to 12.5)	168.9 (128.3 to 213.8)
		Respiratory syncytial virus	198 (168 to 233)	42.4 (14.2 to 83.8)	-78.5 (-92.5 to -57.1)	0.56 (0.49 to 0.63)	0.18 (0.06 to 0.36)	-68.1 (-89.2 to -34.4)

		Staphylococcus aureus	1290 (1120 to 1480)	2370 (2090 to 2670)	84.3 (63.4 to 107.2)	15.1 (13.7 to 16.6)	41.8 (36.2 to 47.6)	177.4 (136.0 to 223.3)
		Streptococcus pneumoniae	4960 (4340 to 5730)	4370 (3860 to 4880)	-11.7 (-21.7 to -0.8)	24.2 (22.1 to 26.4)	32.1 (27.9 to 36.7)	32.9 (13.3 to 54.7)
		Other viruses	1780 (1560 to 2060)	2650 (2330 to 2970)	49.0 (31.9 to 67.0)	3.42 (3.09 to 3.73)	7.65 (6.61 to 8.73)	124.2 (91.5 to 161.1)
		Other bacterial pathogens	1760 (1490 to 2070)	1870 (1590 to 2170)	6.7 (-5.4 to 20.8)	8.27 (7.24 to 9.3)	13.3 (11.1 to 15.5)	60.8 (35.9 to 87.4)
Russian Federation	All Ages	*Acinetobacter baumannii*	43500 (37800 to 49400)	22700 (19900 to 25800)	-47.8 (-50.6 to -45.1)	340 (294 to 404)	325 (279 to 374)	-4.2 (-19.2 to 9.8)
		Chlamydia spp.	158000 (144000 to 172000)	104000 (96000 to 113000)	-33.8 (-37.1 to -30.6)	319 (301 to 338)	365 (328 to 401)	14.5 (4.1 to 24.8)
		Enterobacter spp.	19500 (16900 to 22200)	21500 (19100 to 23800)	10.5 (3.5 to 17.7)	153 (131 to 181)	288 (251 to 324)	89.1 (61.5 to 116.8)
		Escherichia coli	59300 (53700 to 65300)	57600 (52800 to 63000)	-2.7 (-7.8 to 2.5)	477 (445 to 511)	802 (726 to 887)	68.4 (51.6 to 84.5)
		Fungus	45100 (39700 to 51300)	41700 (37700 to 46500)	-7.4 (-12.6 to -2.0)	264 (236 to 292)	558 (495 to 630)	111.9 (90.4 to 133.2)
		Group B Streptococcus	49000 (44100 to 54800)	46900 (42900 to 51600)	-4.2 (-9.3 to 0.7)	319 (297 to 344)	461 (415 to 511)	44.5 (30.6 to 58.2)
		Haemophilus influenzae	212000 (195000 to 231000)	177000 (165000 to 189000)	-16.6 (-21.6 to -11.6)	680 (645 to 717)	754 (688 to 821)	11.1 (0.6 to 21.2)
		Influenza	278000 (259000 to 298000)	37900 (10000 to 84500)	-86.4 (-96.4 to -69.8)	1500 (1450 to 1550)	386 (99.6 to 863)	-74.4 (-93.3 to -42.0)
		Klebsiella pneumoniae	140000 (130000 to 150000)	107000 (100000 to 115000)	-23.3 (-26.9 to -19.5)	1070 (1030 to 1120)	1460 (1330 to 1590)	36.1 (24.1 to 48.8)
		Legionella spp.	95100 (85900 to 106000)	121000 (111000 to 132000)	27.8 (21.4 to 34.6)	432 (397 to 473)	1140 (1020 to 1270)	163.8 (137.8 to 189.6)
		Mycoplasma spp.	251000 (231000 to 274000)	208000 (193000 to 225000)	-17.2 (-21.1 to -12.9)	447 (425 to 470)	726 (661 to 790)	62.5 (47.2 to 76.9)
		Polymicrobial	7830 (5190 to 11700)	6240 (3870 to 10100)	-20.8 (-28.3 to -12.9)	134 (87.3 to 193)	95.5 (61.3 to 151)	-27.7 (-53.9 to 4.3)
		Pseudomonas aeruginosa	128000 (118000 to 138000)	149000 (138000 to 161000)	16.8 (11.5 to 22.5)	670 (639 to 703)	1410 (1280 to 1550)	111.0 (91.5 to 129.7)
		Respiratory syncytial virus	155000 (138000 to 175000)	11500 (3050 to 25500)	-92.6 (-98.0 to -83.7)	760 (729 to 792)	49.7 (12.6 to 113)	-93.5 (-98.4 to -85.3)
		Staphylococcus aureus	190000 (177000 to 202000)	219000 (205000 to 234000)	15.7 (10.8 to 20.6)	1820 (1760 to 1870)	4270 (3890 to 4650)	135.3 (114.4 to 155.2)
		Streptococcus pneumoniae	1270000 (1180000 to 1360000)	768000 (720000 to 818000)	-39.5 (-42.6 to -36.3)	5360 (5250 to 5460)	6040 (5530 to 6540)	12.7 (3.0 to 22.4)
		Other viruses	643000 (592000 to 696000)	514000 (481000 to 548000)	-19.9 (-24.9 to -15.1)	1230 (1190 to 1260)	1570 (1440 to 1700)	27.5 (16.4 to 38.5)
		Other bacterial pathogens	210000 (188000 to 232000)	183000 (162000 to 204000)	-12.9 (-16.9 to -8.9)	754 (689 to 817)	1430 (1250 to 1640)	89.8 (72.6 to 107.6)
	Under 5	*Acinetobacter baumannii*	1160 (836 to 1600)	117 (84.1 to 160)	-89.9 (-91.6 to -88.2)	87.4 (51.2 to 141)	6.45 (3.57 to 10.9)	-92.7 (-93.8 to -91.6)
		Chlamydia spp.	19000 (15800 to 22900)	2390 (1930 to 2900)	-87.4 (-89.3 to -85.4)	117 (108 to 126)	9.54 (8.32 to 10.8)	-91.8 (-92.9 to -90.9)

		Enterobacter spp.	1990 (1480 to 2580)	410 (305 to 546)	-79.3 (-82.6 to -75.9)	54.2 (38 to 78)	6.6 (4.78 to 9.1)	-87.7 (-89.8 to -85.9)
		Escherichia coli	6870 (5600 to 8320)	1410 (1130 to 1740)	-79.4 (-82.5 to -76.1)	168 (149 to 187)	19.9 (17 to 22.6)	-88.1 (-89.7 to -86.6)
		Fungus	1430 (994 to 2000)	239 (168 to 327)	-83.2 (-86.0 to -80.3)	16 (11.4 to 21.2)	2 (1.43 to 2.66)	-87.5 (-89.5 to -85.6)
		Group B Streptococcus	5700 (4590 to 7070)	1080 (859 to 1340)	-81.0 (-83.7 to -78.0)	142 (129 to 155)	14.8 (12.9 to 16.9)	-89.5 (-91.0 to -88.2)
		Haemophilus influenzae	55400 (46400 to 66000)	9950 (8080 to 12000)	-82.0 (-84.7 to -79.2)	339 (311 to 370)	31.6 (27.5 to 35.4)	-90.7 (-91.9 to -89.6)
		Influenza	46500 (39500 to 54300)	994 (275 to 2180)	-97.9 (-99.4 to -95.4)	512 (481 to 543)	7.49 (1.85 to 16.8)	-98.5 (-99.6 to -96.7)
		Klebsiella pneumoniae	26600 (22500 to 31400)	3420 (2790 to 4100)	-87.1 (-88.9 to -85.2)	422 (395 to 450)	38.6 (33.7 to 43.1)	-90.9 (-92.1 to -89.7)
		Legionella spp.	2220 (1530 to 3290)	525 (357 to 758)	-76.3 (-79.8 to -72.6)	65.5 (49.6 to 84.8)	8.05 (6.19 to 10.1)	-87.6 (-89.5 to -85.6)
		Mycoplasma spp.	31600 (26500 to 37800)	8010 (6490 to 9600)	-74.6 (-78.3 to -70.8)	131 (120 to 143)	20.5 (17.7 to 23.1)	-84.3 (-86.4 to -82.4)
		Polymicrobial	675 (425 to 1040)	117 (70.1 to 185)	-82.7 (-85.2 to -80.0)	92.1 (50.1 to 148)	9.72 (4.99 to 16.5)	-89.5 (-91.1 to -87.7)
		Pseudomonas aeruginosa	14900 (12500 to 17900)	4160 (3390 to 5000)	-72.1 (-76.0 to -67.6)	218 (203 to 233)	34.5 (30.1 to 38.7)	-84.1 (-86.3 to -82.3)
		Respiratory syncytial virus	110000 (93100 to 128000)	3830 (1060 to 8190)	-96.5 (-99.0 to -92.4)	673 (644 to 704)	14.4 (3.62 to 32.5)	-97.9 (-99.5 to -95.2)
		Staphylococcus aureus	19200 (16300 to 22500)	3950 (3230 to 4750)	-79.4 (-82.1 to -76.3)	414 (391 to 435)	57.9 (50.6 to 64.3)	-86.0 (-87.8 to -84.5)
		Streptococcus pneumoniae	309000 (263000 to 360000)	27300 (22200 to 32600)	-91.2 (-92.3 to -89.9)	2210 (2120 to 2300)	142 (125 to 158)	-93.6 (-94.4 to -92.9)
		Other viruses	233000 (199000 to 272000)	38200 (31300 to 45300)	-83.6 (-85.7 to -81.4)	707 (678 to 738)	84.6 (74 to 93.7)	-88.0 (-89.6 to -86.8)
		Other bacterial pathogens	8000 (6180 to 10000)	1290 (974 to 1660)	-83.9 (-86.4 to -81.3)	88.5 (68.7 to 115)	9.22 (7.13 to 12.1)	-89.6 (-91.0 to -88.2)
	5-14 years	*Acinetobacter baumannii*	3960 (2990 to 5110)	940 (718 to 1210)	-76.3 (-77.8 to -74.8)	5.69 (4.92 to 6.49)	1.18 (1.01 to 1.35)	-79.3 (-80.8 to -77.8)
		Chlamydia spp.	18200 (14000 to 23500)	6040 (4720 to 7630)	-66.7 (-68.7 to -64.8)	6.48 (5.92 to 7.12)	1.88 (1.7 to 2.06)	-70.9 (-72.9 to -69.0)
		Enterobacter spp.	1690 (1280 to 2210)	911 (698 to 1180)	-46.0 (-50.2 to -42.1)	2.3 (2.01 to 2.62)	1.08 (0.95 to 1.22)	-52.8 (-56.3 to -49.0)
		Escherichia coli	4360 (3360 to 5630)	2050 (1590 to 2630)	-52.9 (-56.0 to -50.0)	6.13 (5.57 to 6.68)	2.52 (2.3 to 2.74)	-58.8 (-61.7 to -56.0)
		Fungus	5260 (3970 to 6770)	2320 (1770 to 2970)	-55.7 (-58.6 to -52.5)	7.29 (6.46 to 8.16)	2.82 (2.54 to 3.14)	-61.3 (-64.4 to -58.6)
		Group B Streptococcus	5480 (4170 to 6990)	2650 (2050 to 3380)	-51.6 (-55.0 to -48.5)	5.47 (4.9 to 6.13)	2.31 (2.08 to 2.56)	-57.7 (-60.6 to -54.9)
		Haemophilus influenzae	22200 (17300 to 28300)	10800 (8470 to 13700)	-51.1 (-54.1 to -48.2)	11.9 (11.2 to 12.6)	4.23 (3.93 to 4.52)	-64.4 (-66.9 to -62.0)
		Influenza	31500 (24600 to 39900)	2230 (609 to 5120)	-92.9 (-98.1 to -84.2)	33 (31.6 to 34.5)	2.04 (0.51 to 4.58)	-93.8 (-98.5 to -86.1)

		Klebsiella pneumoniae	12800 (10000 to 16400)	5230 (4120 to 6610)	-59.2 (-61.6 to -56.8)	17.9 (17.1 to 18.8)	6.39 (5.92 to 6.81)	-64.3 (-66.6 to -62.1)
		Legionella spp.	10900 (8420 to 14000)	6530 (5100 to 8220)	-40.1 (-43.9 to -36.4)	10.5 (9.53 to 11.5)	5.49 (5 to 6)	-47.6 (-51.2 to -44.1)
		Mycoplasma spp.	49100 (37900 to 62500)	21800 (17100 to 27500)	-55.6 (-58.2 to -53.1)	18.2 (17.2 to 19.3)	7.08 (6.55 to 7.55)	-61.2 (-63.9 to -58.8)
		Polymicrobial	740 (461 to 1140)	281 (168 to 450)	-62.2 (-65.5 to -58.6)	1.06 (0.68 to 1.61)	0.35 (0.21 to 0.56)	-66.9 (-70.3 to -63.5)
		Pseudomonas aeruginosa	12000 (9330 to 15200)	6950 (5440 to 8800)	-42.1 (-45.6 to -38.9)	11.7 (11 to 12.4)	5.9 (5.45 to 6.32)	-49.4 (-52.9 to -46.4)
		Respiratory syncytial virus	8450 (6570 to 10800)	676 (181 to 1560)	-92.0 (-97.9 to -82.2)	4.04 (3.76 to 4.35)	0.28 (0.07 to 0.63)	-93.1 (-98.3 to -84.4)
		Staphylococcus aureus	18800 (14700 to 23600)	10500 (8260 to 13300)	-44.0 (-47.3 to -41.2)	37.3 (36 to 38.5)	18.2 (17 to 19.2)	-51.1 (-54.1 to -48.3)
		Streptococcus pneumoniae	178000 (141000 to 226000)	64100 (50100 to 80900)	-64.0 (-66.0 to -62.1)	147 (144 to 151)	46.3 (43.4 to 48.8)	-68.5 (-70.5 to -66.8)
		Other viruses	81700 (64500 to 104000)	44800 (35200 to 56300)	-45.1 (-48.3 to -42.2)	26.6 (25.8 to 27.4)	12.7 (11.9 to 13.5)	-52.1 (-55.1 to -49.2)
		Other bacterial pathogens	19000 (14700 to 24400)	7480 (5770 to 9580)	-60.5 (-62.9 to -58.4)	14.9 (13.6 to 16.3)	5.16 (4.55 to 5.79)	-65.5 (-67.8 to -63.2)
	15-49 years	*Acinetobacter baumannii*	5420 (4540 to 6380)	3100 (2600 to 3640)	-42.7 (-46.3 to -38.9)	40.4 (35 to 46.4)	50.5 (42.9 to 58.3)	25.0 (13.2 to 36.9)
		Chlamydia spp.	24800 (21900 to 28200)	19900 (17700 to 22200)	-19.7 (-24.3 to -14.8)	46.1 (42.3 to 50.6)	80.6 (71.6 to 90.1)	75.1 (59.2 to 90.3)
		Enterobacter spp.	2310 (1960 to 2680)	3000 (2590 to 3450)	30.2 (21.1 to 40.5)	16.3 (14.2 to 18.6)	46.4 (40 to 52.8)	184.2 (155.2 to 213.6)
		Escherichia coli	5960 (5220 to 6770)	6770 (6010 to 7550)	13.7 (6.9 to 21.0)	43.6 (39.6 to 47.5)	108 (96.8 to 120)	148.1 (124.9 to 170.8)
		Fungus	7180 (6180 to 8240)	7660 (6770 to 8640)	6.8 (0.2 to 14.0)	51.9 (45.9 to 57.9)	121 (107 to 137)	133.0 (110.5 to 155.4)
		Group B Streptococcus	7490 (6540 to 8680)	8740 (7760 to 9920)	16.8 (9.3 to 24.3)	38.9 (34.9 to 43.7)	99.1 (87.8 to 111)	154.8 (131.0 to 177.9)
		Haemophilus influenzae	30300 (27000 to 33700)	35700 (32200 to 39200)	18.0 (11.9 to 25.8)	84.6 (79.5 to 89.6)	181 (165 to 198)	114.7 (95.0 to 134.6)
		Influenza	43000 (39000 to 47800)	7340 (1920 to 16000)	-82.9 (-95.4 to -62.0)	235 (225 to 244)	87.3 (22.3 to 197)	-62.8 (-90.3 to -15.5)
		Klebsiella pneumoniae	17500 (15800 to 19300)	17200 (15700 to 18800)	-1.5 (-6.5 to 4.2)	127 (122 to 133)	274 (249 to 298)	114.9 (95.4 to 135.0)
		Legionella spp.	14900 (13000 to 16800)	21500 (19300 to 23900)	44.6 (36.1 to 54.4)	74.7 (67.8 to 82.2)	235 (212 to 262)	215.5 (186.5 to 243.5)
		Mycoplasma spp.	67100 (60600 to 74400)	71900 (65300 to 79100)	7.2 (1.6 to 13.5)	130 (123 to 138)	304 (276 to 331)	133.9 (112.5 to 154.6)
		Polymicrobial	1010 (638 to 1540)	928 (567 to 1500)	-8.7 (-17.3 to 0.8)	7.51 (4.87 to 11.4)	15 (9.08 to 23.9)	99.2 (76.0 to 122.6)
		Pseudomonas aeruginosa	16400 (14700 to 18400)	22900 (20700 to 25300)	39.7 (31.8 to 48.1)	83 (78.1 to 88.2)	253 (228 to 276)	204.8 (176.8 to 231.7)
		Respiratory syncytial virus	11500 (10200 to 12900)	2220 (579 to 4850)	-80.7 (-94.8 to -56.9)	28.8 (26.8 to 30.9)	12.1 (3.02 to 27.3)	-58.1 (-89.2 to -5.1)

		Staphylococcus aureus	25600 (23200 to 28300)	34600 (31600 to 37900)	35.1 (27.9 to 42.8)	265 (256 to 274)	781 (713 to 848)	194.7 (168.5 to 219.6)
		Streptococcus pneumoniae	243000 (222000 to 266000)	211000 (195000 to 230000)	-13.1 (-17.4 to -8.4)	1050 (1030 to 1070)	1990 (1820 to 2150)	89.6 (73.3 to 106.0)
		Other viruses	112000 (102000 to 122000)	148000 (135000 to 161000)	32.3 (25.8 to 39.6)	189 (183 to 195)	546 (498 to 590)	188.8 (163.5 to 213.6)
		Other bacterial pathogens	25900 (22600 to 29400)	24700 (21400 to 28200)	-4.8 (-10.2 to 1.0)	106 (96.7 to 116)	221 (192 to 252)	108.1 (89.0 to 127.1)
	50-69 years	*Acinetobacter baumannii*	20900 (18000 to 24300)	10500 (8970 to 12100)	-49.9 (-52.6 to -47.2)	129 (115 to 146)	145 (123 to 169)	12.1 (-0.8 to 24.8)
		Chlamydia spp.	56800 (50400 to 63800)	39700 (35500 to 44500)	-30.0 (-33.3 to -26.6)	87.1 (79.8 to 94.8)	136 (119 to 154)	56.6 (39.4 to 74.5)
		Enterobacter spp.	8670 (7450 to 10100)	9840 (8580 to 11200)	13.7 (6.5 to 21.3)	50.7 (44.3 to 57.6)	129 (110 to 147)	154.4 (124.0 to 185.8)
		Escherichia coli	24700 (21800 to 27800)	24400 (21900 to 27300)	-1.1 (-6.0 to 3.8)	149 (137 to 162)	331 (292 to 371)	121.3 (96.4 to 144.8)
		Fungus	23700 (20600 to 27100)	22100 (19500 to 24900)	-6.7 (-11.7 to -1.4)	141 (126 to 158)	295 (256 to 337)	108.7 (85.3 to 131.9)
		Group B Streptococcus	19900 (17300 to 22700)	20200 (17900 to 22800)	1.8 (-3.3 to 7.0)	85.5 (77 to 94.9)	194 (170 to 222)	127.7 (101.7 to 152.3)
		Haemophilus influenzae	68600 (62100 to 75900)	71000 (64500 to 77900)	3.5 (-0.9 to 8.4)	158 (150 to 168)	306 (270 to 340)	93.1 (72.7 to 115.0)
		Influenza	83900 (76400 to 91900)	12600 (3310 to 27800)	-85.0 (-96.0 to -66.7)	379 (362 to 396)	127 (32.8 to 285)	-66.5 (-91.3 to -25.2)
		Klebsiella pneumoniae	50800 (46600 to 55700)	44100 (40300 to 48400)	-13.2 (-16.6 to -9.3)	306 (292 to 320)	594 (530 to 658)	94.3 (72.9 to 114.8)
		Legionella spp.	47500 (41900 to 53200)	59800 (53900 to 66600)	26.1 (19.9 to 32.6)	197 (180 to 215)	555 (489 to 627)	182.2 (150.6 to 213.2)
		Mycoplasma spp.	69700 (63100 to 77000)	64500 (58500 to 71100)	-7.5 (-11.4 to -3.5)	111 (104 to 119)	231 (203 to 257)	107.0 (84.3 to 129.5)
		Polymicrobial	3260 (2110 to 4940)	2610 (1600 to 4160)	-20.2 (-27.2 to -13.1)	20 (13 to 30.3)	35.8 (21.6 to 58.3)	78.5 (55.7 to 101.3)
		Pseudomonas aeruginosa	52700 (47500 to 58400)	63900 (58100 to 70700)	21.2 (16.3 to 26.5)	220 (208 to 234)	598 (527 to 669)	171.3 (142.1 to 199.9)
		Respiratory syncytial virus	16700 (14800 to 18700)	2800 (723 to 6150)	-83.2 (-95.6 to -62.8)	34.3 (31.3 to 37.6)	12.8 (3.25 to 29)	-62.6 (-90.1 to -16.5)
		Staphylococcus aureus	72400 (66100 to 79300)	85500 (78500 to 93500)	18.0 (13.4 to 23.0)	619 (598 to 640)	1640 (1460 to 1800)	164.1 (136.0 to 191.6)
		Streptococcus pneumoniae	339000 (312000 to 370000)	260000 (238000 to 284000)	-23.4 (-26.1 to -20.3)	1210 (1180 to 1240)	2070 (1840 to 2290)	71.5 (53.3 to 90.0)
		Other viruses	136000 (125000 to 149000)	159000 (145000 to 174000)	16.7 (12.5 to 21.4)	191 (184 to 197)	498 (443 to 551)	161.1 (132.9 to 189.4)
		Other bacterial pathogens	88600 (78000 to 100000)	73600 (64300 to 83700)	-17.0 (-20.8 to -13.3)	301 (274 to 328)	560 (480 to 644)	86.0 (66.9 to 106.9)
	70+ years	*Acinetobacter baumannii*	12000 (10200 to 14100)	8040 (6930 to 9370)	-33.0 (-37.2 to -28.5)	77.3 (67.7 to 87.6)	122 (103 to 140)	57.4 (43.9 to 71.9)
		Chlamydia spp.	38800 (34100 to 44400)	36300 (32300 to 41000)	-6.4 (-11.7 to -0.9)	62.1 (56.3 to 67.6)	136 (121 to 152)	119.7 (100.9 to 136.9)

		Enterobacter spp.	4840 (4040 to 5740)	7360 (6400 to 8510)	52.2 (41.2 to 63.8)	29.5 (25.5 to 34)	105 (91.3 to 120)	257.1 (222.3 to 291.6)
		Escherichia coli	17400 (15200 to 20200)	23000 (20400 to 26300)	32.3 (24.4 to 40.9)	110 (99.9 to 120)	341 (305 to 380)	210.6 (184.1 to 236.4)
		Fungus	7570 (6470 to 8950)	9440 (8260 to 10800)	24.8 (17.0 to 33.3)	47.1 (41.7 to 53.1)	138 (121 to 157)	192.9 (166.9 to 219.2)
		Group B Streptococcus	10500 (9100 to 12100)	14200 (12600 to 16200)	36.2 (27.5 to 44.5)	46.9 (42 to 52.2)	150 (133 to 167)	219.7 (193.3 to 246.2)
		Haemophilus influenzae	35500 (31600 to 40500)	49100 (44300 to 55100)	38.5 (30.8 to 46.9)	85.4 (79.9 to 90.8)	231 (207 to 251)	171.0 (149.5 to 193.5)
		Influenza	73200 (65600 to 82300)	14700 (3880 to 32400)	-79.9 (-94.7 to -55.6)	345 (326 to 361)	162 (41.9 to 362)	-53.0 (-87.7 to 6.3)
		Klebsiella pneumoniae	32100 (28900 to 36300)	37300 (33900 to 41600)	16.2 (10.3 to 22.3)	202 (191 to 212)	550 (495 to 599)	172.7 (150.4 to 194.2)
		Legionella spp.	19600 (17200 to 22700)	33000 (29500 to 37300)	68.8 (58.9 to 79.2)	84.6 (76.3 to 93.7)	335 (299 to 373)	296.2 (263.3 to 328.7)
		Mycoplasma spp.	33800 (30000 to 38700)	41900 (37500 to 47400)	23.8 (17.3 to 30.7)	56.4 (51.7 to 60.8)	164 (146 to 180)	190.6 (167.7 to 213.9)
		Polymicrobial	2140 (1390 to 3370)	2300 (1390 to 3820)	6.8 (-3.3 to 17.0)	13.7 (8.8 to 21.3)	34.5 (20.8 to 57.1)	150.6 (123.7 to 177.5)
		Pseudomonas aeruginosa	31500 (28100 to 35900)	51100 (46100 to 57400)	62.3 (53.8 to 71.4)	137 (128 to 146)	523 (466 to 574)	280.8 (249.3 to 310.7)
		Respiratory syncytial virus	8970 (7830 to 10400)	2010 (529 to 4420)	-77.6 (-94.0 to -50.3)	19.3 (17.6 to 21.1)	10.1 (2.56 to 22.7)	-47.6 (-86.7 to 17.8)
		Staphylococcus aureus	53800 (48200 to 60300)	84900 (77000 to 95000)	58.0 (49.8 to 66.3)	480 (451 to 501)	1780 (1600 to 1940)	270.7 (241.1 to 298.1)
		Streptococcus pneumoniae	200000 (181000 to 225000)	205000 (187000 to 230000)	2.6 (-2.5 to 7.9)	744 (706 to 770)	1790 (1620 to 1940)	140.8 (122.0 to 159.3)
		Other viruses	79600 (71600 to 89600)	124000 (112000 to 139000)	56.2 (48.6 to 64.3)	116 (109 to 121)	426 (384 to 463)	266.5 (238.7 to 296.0)
		Other bacterial pathogens	68100 (59200 to 78500)	75600 (66100 to 86600)	11.1 (4.9 to 17.7)	244 (220 to 269)	636 (549 to 729)	161.1 (140.0 to 183.1)
Ukraine	All Ages	*Acinetobacter baumannii*	13500 (11700 to 15400)	9350 (8220 to 10700)	-30.5 (-36.3 to -24.8)	93.2 (78.2 to 112)	103 (69.6 to 141)	10.9 (-27.0 to 55.2)
		Chlamydia spp.	50100 (45600 to 55000)	29000 (26800 to 31400)	-42.1 (-46.7 to -37.3)	86 (79 to 93.5)	81.1 (56.8 to 110)	-5.6 (-35.5 to 27.2)
		Enterobacter spp.	6590 (5780 to 7540)	6190 (5520 to 6950)	-5.9 (-13.9 to 1.6)	44.2 (37.3 to 53.6)	64.3 (43.9 to 88)	46.3 (-0.7 to 103.8)
		Escherichia coli	19700 (17700 to 21900)	15400 (14200 to 17000)	-21.6 (-28.1 to -15.1)	132 (120 to 145)	159 (110 to 215)	20.4 (-16.6 to 63.0)
		Fungus	14800 (13100 to 16600)	12400 (11300 to 13800)	-15.8 (-23.5 to -8.1)	75 (66 to 84)	140 (93.6 to 196)	87.5 (24.8 to 159.9)
		Group B Streptococcus	16200 (14500 to 18100)	13800 (12700 to 15200)	-14.3 (-21.1 to -7.2)	92.3 (83.6 to 102)	112 (77 to 149)	21.1 (-17.7 to 64.2)
		Haemophilus influenzae	67800 (62400 to 73600)	40900 (38100 to 44300)	-39.6 (-43.9 to -35.4)	177 (162 to 193)	132 (91.1 to 178)	-25.5 (-49.4 to 1.3)
		Influenza	92800 (85900 to 99900)	6070 (3210 to 9780)	-93.5 (-96.5 to -89.5)	403 (378 to 432)	48.2 (22.4 to 87.3)	-88.0 (-94.5 to -78.2)

		Klebsiella pneumoniae	44900 (41600 to 48400)	33100 (30900 to 36000)	-26.2 (-31.7 to -20.3)	279 (261 to 300)	351 (243 to 476)	25.7 (-14.3 to 71.0)
		Legionella spp.	33600 (30100 to 37400)	31200 (28700 to 34100)	-7.2 (-15.6 to 1.1)	134 (120 to 148)	241 (162 to 329)	80.3 (21.2 to 148.8)
		Mycoplasma spp.	82400 (75000 to 90000)	56800 (52900 to 60800)	-31.0 (-36.0 to -25.8)	126 (116 to 136)	188 (128 to 255)	49.6 (0.4 to 104.2)
		Polymicrobial	2520 (1640 to 3850)	1880 (1170 to 2970)	-25.6 (-32.7 to -18.3)	42.2 (26.9 to 63.1)	23.3 (14.1 to 37.9)	-43.4 (-67.9 to -8.3)
		Pseudomonas aeruginosa	44200 (40600 to 48100)	44400 (41200 to 48500)	0.5 (-7.6 to 8.0)	194 (180 to 210)	329 (227 to 446)	69.8 (15.8 to 130.1)
		Respiratory syncytial virus	44600 (39300 to 50600)	1990 (1060 to 3210)	-95.5 (-97.6 to -92.8)	189 (165 to 216)	7.66 (3.58 to 13.7)	-95.9 (-98.1 to -92.9)
		Staphylococcus aureus	65200 (60500 to 70300)	63200 (59000 to 68800)	-3.0 (-10.1 to 4.3)	509 (477 to 544)	935 (652 to 1260)	83.7 (25.9 to 150.5)
		Streptococcus pneumoniae	403000 (373000 to 436000)	231000 (215000 to 247000)	-42.7 (-46.7 to -38.8)	1350 (1270 to 1450)	1580 (1080 to 2140)	16.7 (-20.5 to 58.8)
		Other viruses	206000 (190000 to 225000)	144000 (135000 to 154000)	-30.1 (-34.9 to -25.0)	308 (285 to 333)	390 (271 to 523)	26.8 (-13.6 to 74.0)
		Other bacterial pathogens	71000 (63300 to 79600)	49100 (43600 to 55500)	-30.8 (-36.4 to -25.2)	212 (189 to 235)	283 (194 to 393)	33.9 (-7.4 to 82.6)
	Under 5	*Acinetobacter baumannii*	254 (185 to 349)	38.9 (28.1 to 53.2)	-84.6 (-86.7 to -82.6)	26.1 (13.9 to 44.1)	2.46 (1.28 to 4.39)	-90.5 (-93.1 to -87.3)
		Chlamydia spp.	4410 (3580 to 5340)	650 (534 to 782)	-85.2 (-87.3 to -82.8)	28.8 (24.4 to 34.1)	2.73 (2.07 to 3.43)	-90.4 (-92.9 to -87.4)
		Enterobacter spp.	489 (358 to 639)	107 (80.5 to 143)	-78.0 (-81.2 to -74.4)	14.8 (9.22 to 23.4)	1.84 (1.16 to 2.81)	-87.4 (-90.7 to -83.2)
		Escherichia coli	1650 (1300 to 2020)	317 (258 to 384)	-80.7 (-83.6 to -77.3)	42.9 (35 to 52.1)	4.91 (3.68 to 6.24)	-88.5 (-91.5 to -84.7)
		Fungus	344 (236 to 479)	64.1 (44.4 to 88.2)	-81.3 (-84.3 to -77.8)	2.64 (1.82 to 3.66)	0.36 (0.24 to 0.5)	-86.4 (-89.9 to -82.5)
		Group B Streptococcus	1380 (1080 to 1720)	288 (228 to 359)	-79.1 (-82.1 to -75.3)	39.8 (33 to 47.4)	4.48 (3.36 to 5.67)	-88.7 (-91.6 to -84.9)
		Haemophilus influenzae	13700 (11100 to 16700)	1890 (1520 to 2270)	-86.1 (-88.2 to -83.7)	73.9 (61.5 to 87.5)	3.95 (3.08 to 4.9)	-94.6 (-96.0 to -92.9)
		Influenza	11400 (9360 to 13700)	178 (94.6 to 294)	-98.4 (-99.2 to -97.5)	105 (89.6 to 123)	1.03 (0.5 to 1.72)	-99.0 (-99.5 to -98.3)
		Klebsiella pneumoniae	6380 (5240 to 7640)	953 (778 to 1150)	-85.0 (-87.1 to -82.5)	89.5 (75.9 to 105)	9.27 (7.09 to 11.4)	-89.6 (-92.2 to -86.5)
		Legionella spp.	563 (378 to 823)	117 (79.1 to 174)	-79.2 (-82.2 to -75.9)	19.7 (14.2 to 27.1)	2.39 (1.68 to 3.25)	-87.8 (-91.1 to -83.5)
		Mycoplasma spp.	7740 (6370 to 9450)	1480 (1200 to 1790)	-80.8 (-83.6 to -77.4)	27.6 (23.2 to 32.8)	3.27 (2.54 to 4.01)	-88.1 (-91.1 to -84.4)
		Polymicrobial	155 (96 to 244)	29.2 (17.9 to 44.9)	-81.2 (-83.5 to -78.8)	30.4 (16.2 to 51.3)	3.55 (1.72 to 6.09)	-88.3 (-91.5 to -84.3)
		Pseudomonas aeruginosa	3780 (3090 to 4610)	931 (758 to 1120)	-75.3 (-78.8 to -71.1)	55 (46.2 to 64.4)	7.57 (5.82 to 9.43)	-86.1 (-89.6 to -82.0)
		Respiratory syncytial virus	28200 (23400 to 34000)	570 (300 to 929)	-98.0 (-98.9 to -96.7)	161 (137 to 187)	1.85 (0.89 to 3.12)	-98.8 (-99.4 to -98.0)

		Staphylococcus aureus	4750 (3930 to 5730)	1060 (863 to 1290)	-77.6 (-80.6 to -73.9)	88.6 (75.6 to 103)	12.6 (9.79 to 15.4)	-85.7 (-89.3 to -81.5)
		Streptococcus pneumoniae	75400 (62700 to 90200)	10400 (8540 to 12500)	-86.1 (-88.1 to -83.8)	394 (338 to 458)	40 (31 to 49)	-89.8 (-92.3 to -86.9)
		Other viruses	59800 (49400 to 71300)	10200 (8270 to 12200)	-83.0 (-85.4 to -80.1)	140 (120 to 162)	17 (13.1 to 20.7)	-87.8 (-90.8 to -84.2)
		Other bacterial pathogens	1910 (1450 to 2440)	299 (227 to 382)	-84.3 (-86.7 to -81.5)	20.3 (14.3 to 29.5)	2.13 (1.42 to 3.19)	-89.5 (-92.1 to -86.3)
	5-14 years	*Acinetobacter baumannii*	1150 (872 to 1490)	367 (278 to 476)	-68.0 (-70.9 to -64.6)	1.22 (1.01 to 1.43)	0.44 (0.36 to 0.53)	-63.3 (-70.3 to -56.0)
		Chlamydia spp.	5540 (4270 to 7150)	1590 (1210 to 2020)	-71.2 (-74.0 to -67.9)	1.46 (1.25 to 1.67)	0.48 (0.4 to 0.56)	-67.0 (-73.1 to -60.6)
		Enterobacter spp.	547 (414 to 716)	249 (187 to 329)	-54.4 (-59.4 to -49.1)	0.55 (0.46 to 0.65)	0.28 (0.23 to 0.34)	-47.7 (-58.0 to -37.2)
		Escherichia coli	1370 (1070 to 1760)	528 (405 to 671)	-61.5 (-65.4 to -57.3)	1.43 (1.23 to 1.64)	0.63 (0.53 to 0.73)	-55.9 (-64.0 to -47.4)
		Fungus	1650 (1270 to 2130)	645 (493 to 824)	-60.9 (-64.9 to -56.6)	1.69 (1.42 to 1.98)	0.76 (0.63 to 0.89)	-55.2 (-63.8 to -46.6)
		Group B Streptococcus	1750 (1350 to 2230)	739 (566 to 943)	-57.7 (-62.1 to -52.9)	1.29 (1.1 to 1.5)	0.62 (0.52 to 0.73)	-51.5 (-60.8 to -42.3)
		Haemophilus influenzae	7170 (5660 to 9170)	2340 (1820 to 3010)	-67.3 (-70.3 to -63.7)	2.84 (2.49 to 3.2)	0.8 (0.68 to 0.93)	-71.6 (-77.1 to -66.2)
		Influenza	10100 (7940 to 12900)	337 (174 to 579)	-96.7 (-98.3 to -94.6)	7.84 (6.94 to 8.78)	0.3 (0.15 to 0.49)	-96.2 (-98.1 to -93.6)
		Klebsiella pneumoniae	4030 (3180 to 5140)	1500 (1170 to 1920)	-62.7 (-66.0 to -58.9)	4.16 (3.69 to 4.64)	1.77 (1.51 to 2.05)	-57.2 (-65.2 to -49.2)
		Legionella spp.	3670 (2870 to 4680)	1570 (1220 to 2000)	-57.1 (-61.3 to -52.4)	2.6 (2.24 to 3.01)	1.28 (1.07 to 1.5)	-50.8 (-59.8 to -41.2)
		Mycoplasma spp.	15800 (12300 to 20100)	5550 (4320 to 7050)	-64.9 (-68.2 to -61.4)	4.33 (3.81 to 4.87)	1.74 (1.48 to 2)	-59.8 (-67.2 to -51.9)
		Polymicrobial	225 (139 to 348)	80.5 (48.7 to 126)	-64.3 (-68.3 to -60.0)	0.24 (0.15 to 0.36)	0.1 (0.06 to 0.15)	-59.1 (-66.9 to -51.3)
		Pseudomonas aeruginosa	4000 (3110 to 5080)	1980 (1540 to 2510)	-50.5 (-55.1 to -45.2)	2.87 (2.52 to 3.26)	1.62 (1.38 to 1.88)	-43.3 (-54.1 to -32.4)
		Respiratory syncytial virus	2860 (2230 to 3680)	116 (59.9 to 197)	-96.0 (-97.9 to -93.4)	1.01 (0.88 to 1.15)	0.05 (0.02 to 0.08)	-95.4 (-97.7 to -92.3)
		Staphylococcus aureus	6100 (4830 to 7730)	2870 (2230 to 3670)	-52.9 (-57.4 to -47.9)	8.94 (7.94 to 9.95)	4.81 (4.11 to 5.58)	-46.0 (-55.9 to -35.7)
		Streptococcus pneumoniae	56900 (45000 to 71900)	17600 (13700 to 22300)	-69.0 (-71.9 to -65.8)	34.7 (31 to 38.7)	12.3 (10.5 to 14.2)	-64.5 (-71.0 to -57.8)
		Other viruses	27400 (21700 to 34600)	11500 (8930 to 14600)	-58.1 (-62.0 to -53.8)	6.59 (5.87 to 7.34)	3.15 (2.69 to 3.63)	-52.0 (-60.9 to -43.0)
		Other bacterial pathogens	5940 (4640 to 7530)	1920 (1480 to 2470)	-67.6 (-70.7 to -64.3)	3.46 (2.96 to 3.98)	1.28 (1.07 to 1.52)	-62.9 (-69.8 to -55.8)
	15-49 years	*Acinetobacter baumannii*	1660 (1390 to 1960)	1470 (1240 to 1710)	-11.2 (-19.5 to -1.4)	12.9 (10.9 to 15.3)	29.3 (19 to 41.2)	126.4 (47.1 to 211.0)
		Chlamydia spp.	8000 (7070 to 9060)	6370 (5620 to 7240)	-20.2 (-28.0 to -11.7)	15.5 (13.8 to 17.6)	31.5 (20.8 to 43.5)	103.4 (32.0 to 179.6)

		Enterobacter spp.	790 (670 to 929)	997 (858 to 1150)	26.5 (14.4 to 40.2)	5.84 (4.97 to 6.82)	18.8 (12.4 to 25.9)	222.5 (109.7 to 346.7)
		Escherichia coli	1990 (1750 to 2260)	2120 (1880 to 2390)	6.8 (-3.9 to 18.7)	15.2 (13.4 to 17)	41.2 (27.3 to 56.7)	172.2 (77.6 to 275.9)
		Fungus	2390 (2030 to 2770)	2590 (2280 to 2950)	8.4 (-3.0 to 20.5)	18 (15.6 to 20.5)	49.7 (32.3 to 68.9)	176.3 (80.2 to 281.5)
		Group B Streptococcus	2530 (2200 to 2920)	2960 (2610 to 3360)	17.3 (5.2 to 29.4)	13.7 (11.9 to 15.7)	41 (27.1 to 56.5)	199.0 (92.8 to 313.2)
		Haemophilus influenzae	10400 (9280 to 11500)	9390 (8440 to 10400)	-9.3 (-18.0 to -0.2)	30.2 (27.2 to 33.2)	52.9 (35.1 to 72.8)	75.1 (13.9 to 140.6)
		Influenza	14600 (13200 to 16200)	1350 (714 to 2190)	-90.7 (-95.0 to -85.1)	83.4 (75.8 to 91.3)	19.6 (8.86 to 35.5)	-76.4 (-89.3 to -57.4)
		Klebsiella pneumoniae	5830 (5250 to 6470)	6020 (5400 to 6670)	3.5 (-5.7 to 13.0)	44.3 (40.1 to 48.7)	117 (77.4 to 161)	163.7 (73.0 to 260.3)
		Legionella spp.	5300 (4660 to 6000)	6300 (5630 to 7030)	19.1 (7.0 to 31.5)	27.7 (24.4 to 31.1)	84 (55.3 to 115)	203.6 (98.8 to 316.7)
		Mycoplasma spp.	22800 (20500 to 25500)	22200 (20100 to 24600)	-2.6 (-11.9 to 6.8)	46.1 (41.7 to 51.2)	114 (75.8 to 156)	148.2 (63.2 to 241.4)
		Polymicrobial	326 (208 to 492)	323 (197 to 504)	-1.0 (-12.0 to 9.9)	2.52 (1.6 to 3.9)	6.37 (3.48 to 11)	152.3 (63.1 to 250.5)
		Pseudomonas aeruginosa	5780 (5160 to 6490)	7920 (7110 to 8820)	37.3 (23.7 to 51.0)	30.5 (27.3 to 33.7)	107 (70.8 to 146)	249.9 (128.2 to 380.1)
		Respiratory syncytial virus	4130 (3660 to 4620)	463 (244 to 762)	-88.8 (-94.0 to -82.1)	10.7 (9.57 to 11.9)	3.07 (1.37 to 5.57)	-71.4 (-87.0 to -47.9)
		Staphylococcus aureus	8810 (7950 to 9730)	11500 (10400 to 12700)	30.7 (19.0 to 43.2)	95.2 (86.9 to 104)	317 (212 to 432)	233.2 (119.2 to 356.4)
		Streptococcus pneumoniae	82200 (74800 to 90500)	70600 (63800 to 77900)	-14.1 (-21.6 to -6.2)	370 (340 to 401)	809 (540 to 1100)	119.0 (43.7 to 198.8)
		Other viruses	39700 (35900 to 43600)	46000 (41700 to 50900)	16.1 (5.5 to 26.9)	70.1 (64.2 to 76.2)	207 (139 to 282)	195.9 (94.5 to 303.5)
		Other bacterial pathogens	8580 (7440 to 9790)	7690 (6660 to 8830)	-10.2 (-18.9 to -0.9)	36.8 (32.6 to 41.8)	84.1 (54.6 to 117)	129.0 (49.9 to 214.6)
	50-69 years	*Acinetobacter baumannii*	5960 (5100 to 6930)	4390 (3730 to 5180)	-26.2 (-34.6 to -18.0)	33.2 (28.5 to 38.6)	51.4 (33.2 to 73.4)	55.0 (1.0 to 123.2)
		Chlamydia spp.	17000 (15000 to 19200)	11100 (9770 to 12700)	-34.8 (-42.3 to -27.3)	23.5 (20.7 to 26.8)	32.2 (21.1 to 45.3)	37.0 (-11.8 to 97.6)
		Enterobacter spp.	2760 (2380 to 3200)	2870 (2480 to 3340)	4.4 (-8.0 to 15.7)	14.6 (12.4 to 17.1)	31.8 (20.7 to 45.7)	119.3 (40.8 to 216.1)
		Escherichia coli	7650 (6760 to 8650)	6680 (5990 to 7620)	-12.4 (-22.7 to -2.7)	41.8 (36.7 to 47.4)	76.6 (49.7 to 110)	83.9 (17.8 to 165.8)
		Fungus	7330 (6360 to 8450)	6550 (5760 to 7480)	-10.4 (-21.2 to -0.2)	39.5 (34 to 45.4)	74.1 (47.8 to 107)	88.2 (22.5 to 174.5)
		Group B Streptococcus	6240 (5430 to 7100)	5990 (5250 to 6860)	-3.8 (-15.0 to 7.0)	24.2 (21 to 27.8)	48.8 (32.2 to 68.6)	102.1 (29.7 to 192.3)
		Haemophilus influenzae	21800 (19600 to 24100)	16700 (15000 to 18900)	-23.4 (-32.0 to -15.2)	45.5 (40.5 to 50.5)	55.3 (36.1 to 78.2)	21.8 (-21.2 to 75.3)
		Influenza	26500 (24000 to 29300)	2030 (1080 to 3290)	-92.3 (-95.9 to -87.6)	108 (97.6 to 120)	17.4 (7.74 to 32.1)	-83.9 (-93.0 to -69.8)

		Klebsiella pneumoniae	15700 (14300 to 17300)	13900 (12500 to 15600)	-11.6 (-21.5 to -2.1)	85.4 (76.7 to 94.5)	158 (103 to 224)	85.6 (20.1 to 167.0)
		Legionella spp.	15700 (13700 to 17800)	15400 (13700 to 17400)	-1.7 (-13.4 to 9.8)	58.7 (51.4 to 67.1)	121 (78.1 to 171)	106.6 (34.1 to 198.0)
		Mycoplasma spp.	22100 (19600 to 24600)	17300 (15500 to 19600)	-21.5 (-30.5 to -12.3)	31.8 (28.3 to 35.7)	52.4 (34.3 to 75)	64.9 (7.5 to 138.7)
		Polymicrobial	974 (628 to 1480)	800 (500 to 1240)	-18.0 (-28.2 to -7.9)	5.39 (3.46 to 8.29)	9.28 (5 to 16.2)	72.1 (10.5 to 150.2)
		Pseudomonas aeruginosa	17300 (15400 to 19200)	19400 (17400 to 21800)	12.5 (-0.7 to 24.8)	65.1 (57.9 to 73)	154 (101 to 217)	136.3 (52.2 to 242.6)
		Respiratory syncytial virus	5540 (4900 to 6230)	510 (270 to 827)	-90.8 (-95.0 to -85.2)	10.3 (8.96 to 11.7)	1.99 (0.88 to 3.68)	-80.7 (-91.6 to -63.7)
		Staphylococcus aureus	23100 (20800 to 25400)	25000 (22600 to 28200)	8.2 (-4.0 to 20.0)	179 (161 to 198)	405 (265 to 575)	127.2 (47.3 to 227.6)
		Streptococcus pneumoniae	107000 (96600 to 117000)	76600 (69600 to 86200)	-28.1 (-36.1 to -20.3)	343 (309 to 378)	517 (339 to 731)	51.1 (-1.9 to 117.1)
		Other viruses	45000 (40700 to 49200)	44500 (40200 to 50000)	-1.1 (-11.9 to 9.8)	56.9 (51.3 to 62.9)	118 (77.5 to 167)	107.8 (34.6 to 198.9)
		Other bacterial pathogens	27300 (23800 to 31000)	20200 (17500 to 23500)	-25.9 (-34.7 to -17.7)	83.7 (72.8 to 95.4)	130 (83.5 to 186)	55.7 (2.2 to 124.8)
	70+ years	*Acinetobacter baumannii*	4450 (3780 to 5220)	3080 (2620 to 3610)	-30.6 (-38.2 to -22.3)	19.8 (17.2 to 22.6)	19.1 (14.1 to 25.7)	-3.1 (-27.1 to 25.5)
		Chlamydia spp.	15100 (13300 to 17200)	9260 (8260 to 10700)	-38.7 (-45.3 to -31.3)	16.7 (15.1 to 18.5)	14.3 (10.8 to 18.4)	-14.4 (-35.5 to 11.1)
		Enterobacter spp.	2010 (1700 to 2370)	1960 (1690 to 2300)	-1.9 (-12.8 to 10.2)	8.45 (7.28 to 9.74)	11.6 (8.62 to 15.3)	37.0 (3.0 to 75.7)
		Escherichia coli	7020 (6130 to 8080)	5760 (5140 to 6530)	-17.7 (-26.7 to -7.7)	30.6 (27.5 to 33.8)	35.1 (26.4 to 45.6)	14.9 (-13.9 to 47.5)
		Fungus	3050 (2600 to 3540)	2560 (2230 to 3020)	-15.8 (-25.0 to -5.1)	13.1 (11.5 to 15)	15.4 (11.6 to 20.1)	17.6 (-11.5 to 51.6)
		Group B Streptococcus	4280 (3730 to 4910)	3860 (3430 to 4370)	-9.5 (-19.2 to 1.9)	13.2 (11.7 to 14.9)	16.7 (12.5 to 21.6)	26.3 (-5.6 to 62.3)
		Haemophilus influenzae	14700 (13200 to 16600)	10600 (9470 to 11900)	-28.0 (-35.2 to -19.3)	24.5 (22.5 to 26.4)	18.6 (14.1 to 24.2)	-23.9 (-42.4 to -2.2)
		Influenza	30100 (26900 to 33700)	2170 (1150 to 3540)	-92.8 (-96.2 to -88.2)	98.1 (90.7 to 106)	9.87 (4.81 to 17.1)	-89.9 (-95.1 to -82.0)
		Klebsiella pneumoniae	12900 (11600 to 14500)	10700 (9670 to 12100)	-16.9 (-25.6 to -6.9)	56.1 (51.8 to 60.4)	65 (49.5 to 83.9)	16.0 (-12.5 to 50.1)
		Legionella spp.	8430 (7390 to 9620)	7780 (6930 to 8830)	-7.6 (-17.5 to 4.2)	25.2 (22.5 to 28.1)	32.4 (24.5 to 42)	29.1 (-2.4 to 66.3)
		Mycoplasma spp.	13900 (12400 to 15700)	10300 (9210 to 11700)	-26.2 (-34.2 to -16.9)	16 (14.6 to 17.5)	16.5 (12.4 to 21.5)	3.0 (-22.5 to 32.3)
		Polymicrobial	835 (523 to 1330)	644 (388 to 1070)	-23.0 (-31.7 to -12.9)	3.68 (2.34 to 5.75)	3.97 (2.25 to 6.67)	7.5 (-19.1 to 38.7)
		Pseudomonas aeruginosa	13400 (11900 to 15200)	14200 (12800 to 15900)	5.8 (-5.5 to 18.4)	40.4 (36.9 to 43.9)	59.6 (45 to 77.7)	47.6 (11.4 to 89.2)
		Respiratory syncytial virus	3880 (3400 to 4420)	335 (176 to 546)	-91.3 (-95.4 to -85.9)	5.76 (5.2 to 6.38)	0.7 (0.33 to 1.22)	-87.9 (-94.2 to -78.4)

		Staphylococcus aureus	22400 (20000 to 24900)	22700 (20500 to 25400)	1.7 (-8.9 to 14.1)	138 (127 to 148)	196 (149 to 254)	42.0 (7.5 to 81.9)
		Streptococcus pneumoniae	82000 (73400 to 91500)	55400 (50000 to 62200)	-32.4 (-39.3 to -24.0)	210 (194 to 225)	198 (151 to 257)	-5.6 (-28.7 to 20.6)
		Other viruses	34200 (30600 to 38200)	31800 (28700 to 35700)	-7.0 (-16.6 to 4.2)	34.5 (31.8 to 36.9)	44.7 (33.8 to 57.8)	29.8 (-1.4 to 66.3)
		Other bacterial pathogens	27300 (23700 to 31500)	19000 (16500 to 22100)	-30.4 (-37.7 to -21.5)	67.5 (59.4 to 75.2)	65.6 (48.1 to 85.9)	-2.7 (-27.0 to 24.4)

**High-income**	All Ages	*Acinetobacter baumannii*	105000 (90700 to 120000)	76100 (67100 to 85700)	-27.3 (-33.1 to -21.3)	3980 (3410 to 4570)	3200 (2600 to 3660)	-19.8 (-27.4 to -12.8)
		Chlamydia spp.	464000 (425000 to 504000)	387000 (358000 to 421000)	-16.4 (-22.7 to -9.6)	4230 (3750 to 4610)	3850 (3220 to 4270)	-8.9 (-17.2 to -0.9)
		Enterobacter spp.	84100 (74100 to 94400)	161000 (147000 to 177000)	92.3 (75.9 to 109.6)	2970 (2560 to 3380)	5920 (4840 to 6650)	99.2 (79.0 to 117.2)
		Escherichia coli	229000 (209000 to 252000)	402000 (375000 to 431000)	75.6 (63.0 to 88.1)	9000 (7960 to 9840)	15900 (13300 to 17500)	76.9 (60.7 to 90.1)
		Fungus	148000 (133000 to 165000)	209000 (189000 to 230000)	41.7 (29.8 to 54.3)	4420 (3910 to 4960)	6690 (5610 to 7530)	51.4 (37.2 to 65.2)
		Group B Streptococcus	183000 (167000 to 202000)	289000 (266000 to 313000)	58.4 (43.7 to 72.2)	4450 (3890 to 4920)	7440 (6220 to 8260)	67.5 (51.1 to 83.1)
		Haemophilus influenzae	750000 (705000 to 802000)	835000 (783000 to 891000)	11.3 (6.2 to 16.8)	8780 (7900 to 9260)	7660 (6430 to 8370)	-12.8 (-19.6 to -7.7)
		Influenza	1140000 (1070000 to 1210000)	218000 (135000 to 342000)	-80.9 (-88.3 to -70.1)	32100 (28700 to 34100)	3780 (2550 to 5360)	-88.2 (-92.0 to -83.4)
		Klebsiella pneumoniae	478000 (451000 to 508000)	541000 (507000 to 577000)	13.1 (8.2 to 17.9)	16900 (15200 to 17900)	21400 (17900 to 23200)	26.0 (16.3 to 32.5)
		Legionella spp.	495000 (453000 to 541000)	710000 (666000 to 757000)	43.5 (33.1 to 54.7)	11300 (10100 to 12400)	17700 (14900 to 19400)	56.4 (42.2 to 69.9)
		Mycoplasma spp.	836000 (777000 to 899000)	776000 (729000 to 826000)	-7.1 (-12.0 to -1.8)	6010 (5420 to 6450)	6180 (5220 to 6740)	2.7 (-6.0 to 9.3)
		Polymicrobial	25500 (16000 to 40600)	36300 (21500 to 60500)	42.0 (26.6 to 58.0)	970 (599 to 1570)	1420 (824 to 2410)	45.3 (27.0 to 61.8)
		Pseudomonas aeruginosa	647000 (602000 to 703000)	1060000 (994000 to 1130000)	63.6 (54.6 to 72.6)	16400 (14600 to 17600)	28600 (23600 to 31300)	74.2 (58.3 to 85.8)
		Respiratory syncytial virus	465000 (429000 to 504000)	48900 (31100 to 74500)	-89.5 (-93.4 to -84.0)	3340 (3030 to 3590)	246 (166 to 345)	-92.6 (-95.0 to -89.6)
		Staphylococcus aureus	875000 (823000 to 933000)	1830000 (1720000 to 1960000)	108.8 (101.9 to 116.1)	47800 (42800 to 50400)	103000 (85700 to 112000)	115.2 (99.8 to 125.4)
		Streptococcus pneumoniae	4070000 (3840000 to 4310000)	2100000 (1990000 to 2230000)	-48.3 (-49.9 to -46.7)	72000 (65200 to 75400)	47300 (39600 to 51500)	-34.4 (-39.8 to -31.4)
		Other viruses	2470000 (2340000 to 2630000)	2440000 (2300000 to 2580000)	-1.5 (-5.1 to 2.0)	15900 (14500 to 16700)	19700 (16500 to 21400)	23.5 (13.6 to 29.8)
		Other bacterial pathogens	822000 (729000 to 926000)	860000 (757000 to 977000)	4.7 (-1.8 to 12.0)	18900 (16200 to 21400)	20900 (16700 to 24300)	10.3 (0.8 to 18.6)
	Under 5	*Acinetobacter baumannii*	2140 (1450 to 3070)	730 (476 to 1110)	-66.1 (-70.3 to -61.8)	48.5 (29.7 to 76.9)	9.3 (5.13 to 16.1)	-81.0 (-84.3 to -77.6)
		Chlamydia spp.	27100 (23100 to 31400)	10800 (9110 to 12700)	-60.1 (-64.7 to -55.2)	82.2 (75.2 to 89.8)	14.1 (12.1 to 16.2)	-82.8 (-85.2 to -80.6)
		Enterobacter spp.	3660 (2780 to 4720)	3280 (2510 to 4220)	-10.2 (-22.1 to 3.4)	42.5 (31.8 to 57.4)	14.4 (11 to 19.1)	-66.0 (-70.7 to -60.7)
		Escherichia coli	11100 (9340 to 13000)	10200 (8520 to 12000)	-7.8 (-19.9 to 3.7)	127 (114 to 142)	42.5 (37.2 to 48.5)	-66.6 (-70.8 to -62.2)
		Fungus	2050 (1440 to 2860)	1450 (1030 to 1970)	-29.1 (-39.2 to -17.8)	16.3 (11.9 to 21.6)	4.49 (3.22 to 5.93)	-72.3 (-77.0 to -67.0)
		Group B Streptococcus	10300 (8610 to 12200)	7910 (6610 to 9440)	-23.0 (-32.6 to -13.5)	99.3 (89.4 to 110)	28.6 (24.9 to 32.6)	-71.2 (-74.7 to -67.4)

		Haemophilus influenzae	90300 (76300 to 105000)	47600 (40300 to 56500)	-47.2 (-53.2 to -41.3)	307 (284 to 333)	38.6 (33.9 to 43.1)	-87.4 (-89.2 to -85.7)
		Influenza	74100 (63600 to 84900)	5080 (3120 to 7790)	-93.1 (-95.8 to -89.7)	483 (455 to 512)	13 (7.75 to 20.2)	-97.3 (-98.4 to -95.8)
		Klebsiella pneumoniae	39000 (33400 to 45100)	16000 (13500 to 18800)	-59.0 (-62.9 to -55.0)	363 (339 to 388)	62.7 (55.4 to 70)	-82.7 (-84.8 to -80.6)
		Legionella spp.	5160 (3750 to 6960)	3860 (2740 to 5350)	-25.1 (-33.5 to -16.2)	49.4 (38.2 to 62.5)	15.1 (11.5 to 19.1)	-69.4 (-73.4 to -65.2)
		Mycoplasma spp.	50900 (43400 to 59400)	43000 (36300 to 50900)	-15.5 (-23.7 to -6.4)	120 (110 to 131)	37.1 (32.4 to 41.5)	-69.0 (-72.9 to -65.1)
		Polymicrobial	2210 (1270 to 3560)	1230 (690 to 2010)	-44.3 (-50.2 to -38.5)	57.4 (30.7 to 93.7)	16.6 (8.54 to 28.2)	-71.2 (-75.6 to -66.8)
		Pseudomonas aeruginosa	29000 (24800 to 33800)	31800 (26900 to 37500)	9.9 (-1.7 to 21.6)	198 (183 to 215)	79.5 (70.6 to 88.2)	-59.8 (-64.3 to -55.2)
		Respiratory syncytial virus	224000 (194000 to 254000)	17600 (11000 to 26700)	-92.1 (-95.0 to -88.3)	683 (654 to 714)	21.4 (13.2 to 33)	-96.9 (-98.1 to -95.2)
		Staphylococcus aureus	32200 (27800 to 36900)	31600 (26800 to 37000)	-1.8 (-8.8 to 5.1)	401 (380 to 423)	152 (135 to 167)	-62.0 (-66.1 to -58.2)
		Streptococcus pneumoniae	467000 (405000 to 538000)	82900 (70500 to 97400)	-82.3 (-83.3 to -81.2)	2240 (2150 to 2330)	150 (132 to 167)	-93.3 (-94.1 to -92.6)
		Other viruses	435000 (374000 to 499000)	189000 (161000 to 220000)	-56.6 (-59.2 to -54.1)	796 (763 to 829)	141 (125 to 155)	-82.3 (-84.1 to -80.5)
		Other bacterial pathogens	11800 (9280 to 14600)	6170 (4860 to 7790)	-47.5 (-53.9 to -40.1)	71.3 (57.2 to 88.5)	15.1 (11.8 to 19.7)	-78.9 (-81.7 to -75.8)
	5-14 years	*Acinetobacter baumannii*	4090 (3100 to 5340)	1460 (1130 to 1900)	-64.1 (-67.4 to -60.7)	11.7 (9.95 to 13.7)	2.65 (2.3 to 3.04)	-77.2 (-80.1 to -73.9)
		Chlamydia spp.	21600 (16600 to 28000)	10300 (7910 to 13100)	-52.4 (-56.1 to -48.2)	15.1 (13.4 to 16.9)	4.26 (3.86 to 4.7)	-71.7 (-74.9 to -68.2)
		Enterobacter spp.	2550 (1870 to 3380)	2870 (2200 to 3720)	13.2 (2.1 to 25.0)	6.63 (5.73 to 7.57)	4.34 (3.88 to 4.85)	-34.3 (-41.5 to -26.6)
		Escherichia coli	5870 (4480 to 7590)	5880 (4590 to 7460)	0.4 (-8.2 to 9.2)	16 (14.4 to 17.7)	9.46 (8.76 to 10.2)	-40.8 (-46.9 to -34.5)
		Fungus	7060 (5340 to 9280)	5850 (4510 to 7560)	-17.0 (-24.9 to -8.8)	19 (16.7 to 21.4)	9.22 (8.26 to 10.3)	-51.3 (-57.0 to -45.6)
		Group B Streptococcus	7780 (5820 to 10100)	7470 (5770 to 9530)	-3.8 (-13.2 to 6.0)	15 (13.3 to 16.8)	8.4 (7.65 to 9.26)	-43.8 (-50.2 to -36.9)
		Haemophilus influenzae	33300 (25800 to 43000)	22800 (17800 to 29100)	-31.4 (-35.4 to -27.2)	33.6 (31 to 36.1)	8.98 (8.38 to 9.57)	-73.2 (-75.4 to -70.7)
		Influenza	46200 (36000 to 58800)	4210 (2470 to 6790)	-90.9 (-94.2 to -86.1)	93.2 (86.9 to 99.7)	5.25 (3.16 to 8.19)	-94.4 (-96.7 to -91.2)
		Klebsiella pneumoniae	17200 (13300 to 22000)	10400 (8220 to 13100)	-39.4 (-42.4 to -35.9)	46.5 (43.1 to 50.2)	16.2 (15.1 to 17.2)	-65.1 (-68.0 to -62.2)
		Legionella spp.	19500 (14900 to 25600)	18100 (14200 to 22800)	-7.0 (-14.7 to 0.9)	35.4 (32.2 to 39)	18.3 (17.2 to 19.4)	-48.2 (-53.0 to -42.7)
		Mycoplasma spp.	72000 (55000 to 91600)	47900 (37600 to 60400)	-33.4 (-37.6 to -29.1)	51.4 (47.4 to 55.2)	19.6 (18.4 to 20.9)	-61.7 (-65.3 to -58.1)
		Polymicrobial	880 (530 to 1380)	676 (387 to 1080)	-23.6 (-31.9 to -14.3)	2.46 (1.57 to 3.9)	1.14 (0.68 to 1.83)	-53.9 (-59.9 to -47.3)

		Pseudomonas aeruginosa	20500 (15700 to 26500)	21900 (17200 to 27500)	6.8 (-0.3 to 13.7)	37.8 (35 to 40.9)	23 (21.6 to 24.3)	-39.2 (-44.0 to -34.2)
		Respiratory syncytial virus	15600 (12000 to 20300)	1180 (712 to 1880)	-92.4 (-95.1 to -88.5)	14.1 (13 to 15.2)	0.66 (0.4 to 1.01)	-95.3 (-97.1 to -92.8)
		Staphylococcus aureus	28800 (22300 to 36900)	34500 (27400 to 43100)	20.0 (14.2 to 25.7)	110 (103 to 117)	76.1 (71.9 to 79.8)	-30.5 (-35.5 to -25.2)
		Streptococcus pneumoniae	253000 (197000 to 323000)	83900 (66100 to 106000)	-66.8 (-68.2 to -65.3)	403 (378 to 427)	73.9 (70 to 77.9)	-81.6 (-83.0 to -80.2)
		Other viruses	145000 (112000 to 186000)	97800 (77400 to 123000)	-32.6 (-35.6 to -29.3)	89.3 (84.5 to 94)	32.7 (31 to 34.2)	-63.4 (-65.7 to -60.7)
		Other bacterial pathogens	24900 (18900 to 32300)	14600 (11200 to 18700)	-41.4 (-45.7 to -36.8)	38.2 (33.5 to 43)	13 (11.4 to 14.6)	-66.0 (-69.5 to -62.2)
	15-49 years	*Acinetobacter baumannii*	10200 (8340 to 12200)	5280 (4490 to 6160)	-48.4 (-53.3 to -43.4)	91.9 (79.5 to 105)	47.4 (41.5 to 54.1)	-48.3 (-53.1 to -43.3)
		Chlamydia spp.	59300 (51600 to 67800)	37700 (33100 to 42500)	-36.4 (-41.5 to -31.0)	131 (120 to 143)	77.7 (71.5 to 85.2)	-40.5 (-45.3 to -34.9)
		Enterobacter spp.	7740 (6500 to 9080)	10900 (9300 to 12500)	40.7 (28.2 to 54.7)	64.2 (56.3 to 72.7)	80.9 (72.5 to 89.9)	26.3 (14.7 to 39.2)
		Escherichia coli	17100 (14800 to 19500)	22500 (20000 to 25200)	31.5 (20.6 to 42.1)	148 (136 to 160)	176 (166 to 186)	19.2 (9.7 to 28.7)
		Fungus	20600 (17600 to 23700)	21900 (19100 to 25000)	6.8 (-2.7 to 17.2)	175 (157 to 193)	170 (155 to 188)	-2.5 (-11.5 to 6.5)
		Group B Streptococcus	23200 (19900 to 27000)	28100 (24700 to 32000)	21.8 (10.5 to 33.5)	141 (128 to 157)	156 (142 to 170)	10.3 (0.1 to 21.1)
		Haemophilus influenzae	101000 (88700 to 114000)	83100 (73900 to 92500)	-17.5 (-22.0 to -12.8)	323 (308 to 337)	166 (157 to 175)	-48.6 (-51.5 to -45.7)
		Influenza	139000 (123000 to 157000)	19700 (11600 to 31400)	-85.9 (-91.5 to -77.9)	893 (864 to 927)	101 (59.5 to 155)	-88.7 (-93.3 to -82.5)
		Klebsiella pneumoniae	50000 (44300 to 56000)	37700 (33500 to 41800)	-24.6 (-27.9 to -20.9)	429 (411 to 448)	297 (283 to 311)	-30.7 (-34.0 to -27.5)
		Legionella spp.	62200 (53900 to 71100)	66400 (59200 to 73500)	6.9 (-1.6 to 16.2)	363 (335 to 394)	341 (323 to 359)	-5.9 (-13.9 to 1.9)
		Mycoplasma spp.	216000 (191000 to 243000)	177000 (158000 to 197000)	-18.0 (-22.7 to -13.2)	492 (467 to 517)	363 (345 to 381)	-26.1 (-30.9 to -21.6)
		Polymicrobial	2420 (1470 to 3900)	2550 (1490 to 4160)	5.0 (-5.8 to 16.6)	21.4 (13 to 34.3)	21 (12.3 to 33.6)	-2.1 (-12.4 to 8.9)
		Pseudomonas aeruginosa	64900 (56900 to 73900)	81800 (72800 to 91200)	26.3 (19.0 to 34.3)	382 (360 to 406)	429 (411 to 447)	12.2 (5.7 to 19.3)
		Respiratory syncytial virus	50400 (44100 to 57100)	5510 (3350 to 8630)	-89.1 (-93.2 to -83.0)	146 (136 to 156)	12.7 (7.58 to 19.4)	-91.3 (-94.8 to -86.7)
		Staphylococcus aureus	88100 (77900 to 99200)	131000 (117000 to 144000)	48.3 (43.1 to 53.9)	1070 (1040 to 1100)	1420 (1370 to 1470)	32.9 (28.4 to 37.7)
		Streptococcus pneumoniae	752000 (669000 to 845000)	288000 (258000 to 318000)	-61.6 (-62.8 to -60.4)	3810 (3740 to 3880)	1340 (1290 to 1380)	-64.9 (-66.1 to -63.7)
		Other viruses	463000 (413000 to 518000)	346000 (308000 to 382000)	-25.3 (-27.7 to -22.9)	914 (892 to 935)	603 (582 to 621)	-34.0 (-36.6 to -31.9)
		Other bacterial pathogens	71800 (60600 to 83100)	53600 (45800 to 62000)	-25.3 (-30.6 to -19.6)	349 (311 to 388)	238 (211 to 268)	-31.6 (-36.3 to -26.3)
	50-69 years	*Acinetobacter baumannii*	33800 (28900 to 39000)	22300 (19300 to 25400)	-34.1 (-39.7 to -28.7)	530 (464 to 601)	369 (326 to 414)	-30.3 (-36.2 to -24.7)

		Chlamydia spp.	120000 (107000 to 135000)	92500 (83100 to 102000)	-22.8 (-28.6 to -16.7)	458 (420 to 499)	351 (325 to 381)	-23.1 (-28.8 to -16.3)
		Enterobacter spp.	26600 (23300 to 30300)	46300 (41200 to 51500)	74.5 (59.7 to 90.2)	377 (336 to 423)	627 (575 to 683)	66.5 (52.9 to 80.9)
		Escherichia coli	64000 (57300 to 71300)	100000 (91400 to 109000)	56.5 (45.3 to 67.7)	950 (878 to 1020)	1410 (1340 to 1490)	49.0 (39.0 to 59.5)
		Fungus	61200 (53800 to 69000)	81700 (72400 to 91100)	33.7 (22.9 to 46.0)	895 (811 to 988)	1160 (1060 to 1260)	29.4 (18.7 to 39.9)
		Group B Streptococcus	55900 (49500 to 63300)	82300 (73700 to 91100)	47.5 (34.0 to 60.9)	586 (535 to 647)	831 (763 to 896)	42.1 (30.1 to 54.6)
		Haemophilus influenzae	209000 (191000 to 227000)	229000 (210000 to 249000)	9.5 (4.1 to 14.8)	1150 (1100 to 1190)	847 (807 to 885)	-26.1 (-29.7 to -22.6)
		Influenza	248000 (229000 to 271000)	49400 (28900 to 79500)	-80.1 (-88.3 to -67.8)	2730 (2620 to 2820)	389 (236 to 600)	-85.7 (-91.4 to -77.8)
		Klebsiella pneumoniae	131000 (121000 to 142000)	140000 (129000 to 152000)	7.3 (2.7 to 12.2)	1930 (1860 to 2010)	2040 (1960 to 2130)	5.8 (1.5 to 10.1)
		Legionella spp.	184000 (165000 to 204000)	238000 (219000 to 259000)	29.6 (20.0 to 40.6)	1830 (1700 to 1980)	2310 (2200 to 2410)	25.9 (17.2 to 35.7)
		Mycoplasma spp.	205000 (186000 to 225000)	177000 (161000 to 193000)	-13.7 (-19.0 to -7.9)	799 (751 to 849)	683 (648 to 718)	-14.4 (-19.4 to -9.3)
		Polymicrobial	6910 (4240 to 11200)	9310 (5530 to 15300)	34.5 (21.2 to 48.0)	105 (64.3 to 168)	137 (81.8 to 224)	30.3 (17.5 to 43.2)
		Pseudomonas aeruginosa	193000 (175000 to 212000)	278000 (255000 to 300000)	44.3 (36.1 to 52.7)	1940 (1840 to 2050)	2720 (2600 to 2820)	40.3 (32.8 to 48.3)
		Respiratory syncytial virus	67800 (60400 to 75100)	8870 (5320 to 14100)	-86.9 (-92.0 to -79.1)	334 (304 to 365)	32 (19.9 to 48.6)	-90.4 (-94.1 to -85.4)
		Staphylococcus aureus	229000 (211000 to 248000)	428000 (393000 to 462000)	87.1 (80.9 to 94.0)	4730 (4590 to 4870)	8430 (8110 to 8710)	78.0 (72.4 to 84.2)
		Streptococcus pneumoniae	954000 (881000 to 1030000)	499000 (457000 to 537000)	-47.7 (-49.3 to -45.9)	8300 (8060 to 8490)	4480 (4310 to 4640)	-46.0 (-47.8 to -44.1)
		Other viruses	526000 (486000 to 570000)	548000 (504000 to 591000)	4.3 (0.7 to 8.4)	1770 (1710 to 1820)	1820 (1750 to 1890)	3.2 (-0.5 to 6.9)
		Other bacterial pathogens	221000 (191000 to 251000)	201000 (173000 to 228000)	-8.9 (-15.0 to -2.4)	1850 (1660 to 2050)	1660 (1470 to 1860)	-10.1 (-15.9 to -4.1)
	70+ years	*Acinetobacter baumannii*	54500 (46300 to 63400)	46400 (40300 to 53100)	-14.7 (-21.4 to -7.8)	3300 (2790 to 3830)	2770 (2190 to 3200)	-16.2 (-24.3 to -8.6)
		Chlamydia spp.	236000 (210000 to 263000)	236000 (214000 to 263000)	0.3 (-7.6 to 8.9)	3550 (3080 to 3890)	3410 (2800 to 3810)	-4.0 (-12.8 to 4.7)
		Enterobacter spp.	43500 (37400 to 50200)	98000 (86900 to 109000)	125.7 (106.4 to 146.9)	2480 (2100 to 2830)	5200 (4110 to 5890)	109.3 (87.5 to 129.0)
		Escherichia coli	131000 (118000 to 148000)	263000 (241000 to 289000)	101.2 (86.5 to 115.9)	7760 (6770 to 8540)	14300 (11700 to 15800)	84.0 (66.8 to 97.9)
		Fungus	56800 (50100 to 64700)	98100 (87000 to 110000)	72.9 (58.4 to 88.6)	3320 (2870 to 3770)	5350 (4320 to 6120)	61.3 (45.2 to 76.3)
		Group B Streptococcus	85900 (76600 to 96000)	164000 (148000 to 181000)	90.9 (73.9 to 107.7)	3600 (3070 to 4030)	6420 (5210 to 7200)	78.2 (59.9 to 94.8)
		Haemophilus influenzae	317000 (289000 to 347000)	453000 (415000 to 496000)	43.0 (35.8 to 50.3)	6970 (6130 to 7430)	6600 (5400 to 7290)	-5.4 (-13.0 to 0.2)

		Influenza	634000 (582000 to 693000)	139000 (86900 to 218000)	-78.0 (-86.3 to -65.7)	27900 (24600 to 29800)	3270 (2230 to 4610)	-88.3 (-91.9 to -83.6)
		Klebsiella pneumoniae	241000 (221000 to 262000)	336000 (310000 to 367000)	39.7 (33.6 to 46.0)	14200 (12500 to 15100)	18900 (15500 to 20800)	33.5 (23.1 to 40.6)
		Legionella spp.	224000 (201000 to 250000)	383000 (352000 to 419000)	71.0 (58.0 to 85.0)	9050 (7860 to 9990)	15000 (12300 to 16700)	66.2 (50.4 to 80.8)
		Mycoplasma spp.	291000 (264000 to 324000)	331000 (304000 to 364000)	13.7 (6.8 to 20.9)	4550 (3970 to 4950)	5070 (4160 to 5620)	11.4 (1.4 to 18.9)
		Polymicrobial	13000 (7810 to 21800)	22600 (12800 to 38000)	72.5 (56.1 to 90.9)	783 (466 to 1290)	1240 (693 to 2100)	57.8 (40.1 to 74.3)
		Pseudomonas aeruginosa	340000 (310000 to 376000)	644000 (593000 to 700000)	89.7 (78.6 to 101.0)	13800 (12200 to 14900)	25300 (20500 to 27900)	82.9 (66.0 to 95.3)
		Respiratory syncytial virus	108000 (96400 to 121000)	15800 (9900 to 24300)	-85.4 (-90.7 to -77.6)	2160 (1880 to 2390)	180 (123 to 248)	-91.7 (-94.2 to -88.5)
		Staphylococcus aureus	498000 (458000 to 544000)	1200000 (1110000 to 1310000)	141.9 (133.2 to 150.9)	41500 (36600 to 44000)	92900 (75900 to 102000)	123.6 (107.1 to 134.5)
		Streptococcus pneumoniae	1640000 (1520000 to 1790000)	1150000 (1060000 to 1250000)	-30.1 (-32.7 to -27.7)	57300 (50600 to 60600)	41200 (33700 to 45300)	-28.1 (-34.0 to -24.7)
		Other viruses	906000 (833000 to 987000)	1260000 (1160000 to 1370000)	38.8 (33.2 to 44.2)	12300 (11000 to 13100)	17100 (14000 to 18700)	38.1 (27.0 to 45.1)
		Other bacterial pathogens	492000 (432000 to 565000)	585000 (510000 to 675000)	18.8 (11.0 to 27.3)	16600 (14100 to 18800)	18900 (14900 to 22200)	14.0 (3.7 to 22.8)
Australasia	All Ages	*Acinetobacter baumannii*	1260 (1070 to 1460)	943 (821 to 1080)	-24.9 (-33.1 to -16.4)	33.6 (28.3 to 38.9)	29.1 (22.9 to 34.4)	-13.2 (-26.4 to -1.6)
		Chlamydia spp.	6130 (5480 to 6780)	6110 (5560 to 6720)	-0.2 (-10.4 to 11.4)	39.1 (34.3 to 43.2)	45.7 (36.9 to 52.4)	17.1 (1.9 to 34.5)
		Enterobacter spp.	1230 (1080 to 1400)	3090 (2750 to 3410)	150.9 (123.6 to 180.4)	29.8 (25.4 to 34.1)	88.1 (69.6 to 101)	196.5 (153.1 to 237.5)
		Escherichia coli	3220 (2890 to 3600)	7440 (6780 to 8050)	131.3 (109.7 to 154.3)	86.9 (76.9 to 96.1)	234 (190 to 265)	169.7 (132.2 to 202.7)
		Fungus	2090 (1850 to 2360)	3700 (3300 to 4110)	77.6 (58.9 to 99.8)	42 (36.5 to 47.2)	88.3 (70.8 to 102)	110.6 (80.6 to 140.3)
		Group B Streptococcus	2630 (2360 to 2910)	5320 (4840 to 5840)	102.5 (79.8 to 126.6)	44.1 (38.5 to 49.2)	105 (85.4 to 119)	137.7 (105.8 to 170.6)
		Haemophilus influenzae	11100 (10200 to 12100)	15700 (14500 to 17000)	41.6 (31.7 to 51.6)	91.9 (82.5 to 98.5)	107 (87 to 121)	16.8 (2.4 to 29.7)
		Influenza	16400 (15000 to 17700)	384 (106 to 881)	-97.7 (-99.4 to -94.7)	317 (284 to 342)	8.44 (2.23 to 19.1)	-97.3 (-99.3 to -94.0)
		Klebsiella pneumoniae	6680 (6130 to 7210)	9060 (8360 to 9730)	35.6 (26.4 to 45.3)	163 (147 to 176)	271 (220 to 305)	65.6 (45.4 to 82.8)
		Legionella spp.	7730 (6940 to 8540)	13900 (12800 to 15000)	80.7 (62.9 to 100.0)	118 (104 to 131)	253 (208 to 285)	115.4 (84.1 to 143.5)
		Mycoplasma spp.	12200 (11200 to 13300)	13900 (12800 to 15000)	14.5 (6.0 to 23.7)	60.1 (53.8 to 65.5)	77.7 (63.8 to 87.7)	29.2 (13.7 to 44.3)
		Polymicrobial	350 (217 to 555)	615 (354 to 1050)	75.0 (49.9 to 102.2)	9.91 (6.29 to 15.4)	19.3 (10.7 to 33.1)	93.0 (56.7 to 130.2)
		Pseudomonas aeruginosa	9860 (9000 to 10900)	20100 (18500 to 21500)	103.7 (87.7 to 120.8)	169 (151 to 184)	404 (329 to 454)	139.6 (108.5 to 165.6)

		Respiratory syncytial virus	7550 (6930 to 8280)	95.4 (25.9 to 219)	-98.7 (-99.7 to -97.1)	42.8 (39.4 to 46)	0.54 (0.14 to 1.21)	-98.7 (-99.7 to -97.2)
		Staphylococcus aureus	12800 (11800 to 13900)	35600 (32700 to 38200)	177.8 (159.5 to 197.7)	475 (424 to 512)	1570 (1280 to 1760)	231.0 (193.3 to 263.6)
		Streptococcus pneumoniae	58600 (54300 to 63200)	32100 (29900 to 34200)	-45.3 (-48.6 to -42.1)	718 (648 to 769)	499 (406 to 558)	-30.5 (-39.0 to -23.9)
		Other viruses	38900 (36200 to 41900)	45500 (42500 to 48400)	17.0 (10.5 to 23.7)	173 (157 to 185)	262 (216 to 294)	51.9 (33.5 to 67.1)
		Other bacterial pathogens	11500 (9950 to 13000)	14500 (12700 to 16600)	26.3 (14.2 to 39.1)	180 (153 to 206)	266 (205 to 318)	48.0 (27.7 to 67.0)
	Under 5	*Acinetobacter baumannii*	28.8 (17.9 to 46.2)	11.7 (7.09 to 19.6)	-59.6 (-64.1 to -55.2)	0.98 (0.53 to 1.7)	0.3 (0.15 to 0.54)	-69.3 (-75.7 to -61.2)
		Chlamydia spp.	330 (282 to 385)	169 (140 to 206)	-48.6 (-55.3 to -42.0)	1.32 (1.17 to 1.48)	0.34 (0.28 to 0.41)	-74.0 (-78.7 to -68.2)
		Enterobacter spp.	57.3 (43 to 74.5)	65.2 (49.2 to 84.5)	14.2 (-3.2 to 34.5)	0.96 (0.68 to 1.42)	0.43 (0.29 to 0.63)	-55.3 (-64.0 to -45.8)
		Escherichia coli	155 (131 to 184)	193 (158 to 233)	25.0 (7.3 to 43.1)	2.47 (2.17 to 2.8)	1.1 (0.92 to 1.31)	-55.3 (-63.9 to -45.6)
		Fungus	26.8 (18.7 to 37.3)	25.7 (18.2 to 35.9)	-3.7 (-20.6 to 13.6)	0.23 (0.17 to 0.31)	0.08 (0.06 to 0.12)	-64.0 (-72.9 to -52.8)
		Group B Streptococcus	155 (131 to 183)	149 (121 to 182)	-3.5 (-16.8 to 10.1)	2.13 (1.89 to 2.4)	0.83 (0.68 to 1)	-60.8 (-68.2 to -52.2)
		Haemophilus influenzae	1350 (1150 to 1580)	900 (745 to 1090)	-33.5 (-41.4 to -25.1)	5.77 (5.19 to 6.4)	0.81 (0.66 to 0.99)	-85.9 (-89.0 to -82.4)
		Influenza	1070 (908 to 1240)	9.68 (2.67 to 22.1)	-99.1 (-99.8 to -97.9)	8.34 (7.58 to 9.16)	0.03 (0.01 to 0.06)	-99.7 (-99.9 to -99.3)
		Klebsiella pneumoniae	527 (448 to 613)	267 (221 to 322)	-49.4 (-54.8 to -43.7)	5.98 (5.4 to 6.57)	1.4 (1.17 to 1.67)	-76.5 (-80.9 to -71.3)
		Legionella spp.	91.7 (68.1 to 121)	79.3 (56.1 to 109)	-13.6 (-24.5 to -1.4)	1.3 (1.02 to 1.63)	0.53 (0.41 to 0.7)	-58.6 (-66.3 to -48.5)
		Mycoplasma spp.	726 (612 to 860)	757 (625 to 918)	4.3 (-7.9 to 17.2)	2.04 (1.81 to 2.3)	0.78 (0.64 to 0.95)	-61.4 (-69.8 to -52.1)
		Polymicrobial	41.8 (22.1 to 69.7)	25.2 (13.7 to 42.4)	-39.5 (-46.7 to -31.4)	1.66 (0.85 to 2.85)	0.7 (0.33 to 1.24)	-58.1 (-66.5 to -47.8)
		Pseudomonas aeruginosa	475 (405 to 564)	631 (521 to 765)	33.0 (17.3 to 49.4)	4.39 (3.93 to 4.84)	2.14 (1.77 to 2.54)	-51.3 (-60.5 to -40.7)
		Respiratory syncytial virus	3790 (3300 to 4330)	36.7 (10.1 to 84)	-99.0 (-99.7 to -97.8)	15.2 (13.9 to 16.6)	0.05 (0.01 to 0.13)	-99.6 (-99.9 to -99.2)
		Staphylococcus aureus	483 (414 to 559)	634 (528 to 754)	31.2 (19.9 to 42.4)	7.58 (6.93 to 8.26)	3.69 (3.05 to 4.37)	-51.3 (-60.7 to -40.5)
		Streptococcus pneumoniae	6580 (5670 to 7600)	1210 (1000 to 1460)	-81.7 (-83.0 to -80.2)	36.4 (33.4 to 39.5)	2.42 (1.97 to 2.93)	-93.3 (-94.7 to -91.8)
		Other viruses	7290 (6260 to 8410)	3540 (2950 to 4270)	-51.4 (-55.0 to -47.8)	16.3 (15 to 17.7)	3.39 (2.81 to 4.02)	-79.2 (-83.2 to -74.8)
		Other bacterial pathogens	156 (121 to 196)	104 (79.3 to 134)	-33.0 (-42.2 to -22.4)	1.21 (0.92 to 1.62)	0.36 (0.25 to 0.52)	-70.7 (-76.4 to -64.2)
	5-14 years	*Acinetobacter baumannii*	34.1 (24.5 to 46.3)	20.2 (15 to 27)	-40.7 (-47.4 to -33.6)	0.07 (0.06 to 0.09)	0.02 (0.01 to 0.02)	-74.8 (-78.8 to -70.7)

		Chlamydia spp.	229 (168 to 303)	190 (141 to 253)	-16.9 (-25.1 to -7.3)	0.12 (0.11 to 0.13)	0.04 (0.04 to 0.05)	-63.8 (-69.6 to -57.2)
		Enterobacter spp.	34 (24.2 to 46.4)	68.8 (50.1 to 90.8)	103.3 (80.1 to 129.1)	0.07 (0.06 to 0.08)	0.06 (0.05 to 0.07)	-10.0 (-23.8 to 5.8)
		Escherichia coli	71.2 (52.3 to 94)	136 (102 to 178)	91.2 (73.2 to 111.0)	0.15 (0.13 to 0.16)	0.12 (0.11 to 0.14)	-15.4 (-27.6 to -1.9)
		Fungus	85.4 (62.5 to 115)	127 (95.3 to 169)	48.7 (31.2 to 66.0)	0.17 (0.15 to 0.2)	0.11 (0.1 to 0.13)	-34.6 (-45.1 to -24.1)
		Group B Streptococcus	98.9 (72 to 130)	169 (127 to 222)	71.6 (51.8 to 93.0)	0.14 (0.13 to 0.16)	0.11 (0.09 to 0.12)	-24.4 (-36.0 to -9.9)
		Haemophilus influenzae	447 (335 to 591)	515 (381 to 666)	15.3 (6.8 to 24.8)	0.35 (0.32 to 0.38)	0.12 (0.11 to 0.14)	-65.5 (-69.9 to -60.3)
		Influenza	604 (454 to 786)	10.7 (2.72 to 24.3)	-98.2 (-99.5 to -96.0)	0.93 (0.86 to 1.01)	0.01 (0 to 0.02)	-99.3 (-99.8 to -98.3)
		Klebsiella pneumoniae	208 (156 to 269)	206 (156 to 268)	-0.9 (-7.0 to 6.2)	0.42 (0.39 to 0.46)	0.18 (0.16 to 0.21)	-56.6 (-62.1 to -50.1)
		Legionella spp.	293 (218 to 386)	437 (332 to 570)	49.4 (35.0 to 64.4)	0.41 (0.37 to 0.46)	0.27 (0.24 to 0.31)	-33.7 (-43.3 to -22.6)
		Mycoplasma spp.	946 (695 to 1230)	1030 (785 to 1340)	8.8 (0.4 to 18.0)	0.51 (0.47 to 0.56)	0.24 (0.22 to 0.28)	-52.3 (-58.9 to -44.4)
		Polymicrobial	9.28 (5.28 to 15.2)	13.7 (7.45 to 22.9)	47.4 (29.6 to 67.0)	0.02 (0.01 to 0.03)	0.01 (0.01 to 0.02)	-35.4 (-45.9 to -22.6)
		Pseudomonas aeruginosa	299 (222 to 390)	523 (396 to 675)	74.9 (61.6 to 88.6)	0.42 (0.38 to 0.47)	0.33 (0.29 to 0.37)	-22.7 (-33.5 to -11.3)
		Respiratory syncytial virus	238 (178 to 312)	3.18 (0.8 to 7.22)	-98.7 (-99.6 to -97.0)	0.17 (0.15 to 0.18)	0 (0 to 0)	-99.4 (-99.9 to -98.7)
		Staphylococcus aureus	389 (295 to 503)	841 (642 to 1080)	116.2 (104.1 to 129.8)	1.13 (1.05 to 1.23)	1.08 (0.96 to 1.21)	-4.1 (-16.4 to 9.6)
		Streptococcus pneumoniae	3250 (2470 to 4200)	1420 (1080 to 1820)	-56.3 (-58.6 to -53.7)	3.93 (3.63 to 4.23)	0.74 (0.66 to 0.83)	-81.1 (-83.5 to -78.5)
		Other viruses	2180 (1650 to 2830)	2120 (1620 to 2740)	-2.5 (-7.9 to 3.8)	1.03 (0.96 to 1.12)	0.44 (0.4 to 0.49)	-57.0 (-62.3 to -51.0)
		Other bacterial pathogens	296 (215 to 394)	295 (218 to 389)	-0.4 (-8.9 to 9.4)	0.34 (0.3 to 0.39)	0.15 (0.13 to 0.17)	-56.3 (-62.3 to -49.4)
	15-49 years	*Acinetobacter baumannii*	115 (90.4 to 140)	67.9 (55.3 to 82.1)	-40.6 (-47.2 to -33.3)	0.72 (0.6 to 0.84)	0.31 (0.26 to 0.36)	-56.9 (-63.2 to -50.1)
		Chlamydia spp.	773 (653 to 911)	647 (544 to 756)	-16.2 (-24.3 to -6.8)	1.2 (1.07 to 1.34)	0.74 (0.66 to 0.85)	-38.0 (-46.6 to -28.5)
		Enterobacter spp.	115 (93.8 to 139)	236 (194 to 283)	105.6 (83.2 to 129.4)	0.68 (0.58 to 0.78)	1.05 (0.9 to 1.2)	54.2 (32.5 to 78.4)
		Escherichia coli	241 (203 to 283)	465 (395 to 543)	93.7 (76.1 to 113.3)	1.48 (1.32 to 1.64)	2.14 (1.92 to 2.38)	45.4 (26.6 to 67.1)
		Fungus	288 (240 to 341)	432 (364 to 513)	50.2 (33.5 to 69.4)	1.74 (1.55 to 1.96)	1.95 (1.7 to 2.24)	12.1 (-3.9 to 29.6)
		Group B Streptococcus	334 (282 to 401)	578 (485 to 675)	73.4 (52.5 to 94.8)	1.46 (1.3 to 1.64)	1.88 (1.65 to 2.14)	29.6 (11.8 to 50.1)
		Haemophilus influenzae	1510 (1290 to 1750)	1760 (1500 to 2040)	16.7 (8.2 to 25.3)	3.54 (3.27 to 3.82)	2.09 (1.87 to 2.32)	-41.0 (-48.2 to -33.6)

		Influenza	2040 (1750 to 2370)	36.7 (10.1 to 82.8)	-98.2 (-99.5 to -95.9)	9.36 (8.73 to 10)	0.12 (0.03 to 0.27)	-98.7 (-99.7 to -97.1)
		Klebsiella pneumoniae	701 (601 to 814)	701 (595 to 807)	0.0 (-6.0 to 7.1)	4.28 (3.96 to 4.59)	3.17 (2.86 to 3.52)	-25.7 (-34.1 to -16.7)
		Legionella spp.	995 (842 to 1160)	1500 (1280 to 1720)	50.8 (36.2 to 66.6)	4.18 (3.79 to 4.64)	4.72 (4.25 to 5.24)	13.1 (-2.4 to 30.0)
		Mycoplasma spp.	3200 (2720 to 3690)	3500 (3010 to 4050)	9.6 (1.7 to 18.5)	5.19 (4.77 to 5.63)	4.22 (3.79 to 4.67)	-18.5 (-28.6 to -8.1)
		Polymicrobial	31.4 (17.8 to 51.6)	46.9 (25.9 to 80.3)	49.1 (31.3 to 69.5)	0.2 (0.11 to 0.32)	0.22 (0.12 to 0.36)	11.2 (-6.2 to 31.8)
		Pseudomonas aeruginosa	1010 (866 to 1180)	1790 (1530 to 2070)	76.3 (63.7 to 91.4)	4.3 (3.92 to 4.7)	5.66 (5.14 to 6.23)	31.7 (15.3 to 48.6)
		Respiratory syncytial virus	809 (688 to 949)	10.9 (2.92 to 25)	-98.7 (-99.6 to -96.9)	1.69 (1.55 to 1.86)	0.02 (0 to 0.04)	-99.1 (-99.8 to -97.9)
		Staphylococcus aureus	1320 (1130 to 1520)	2880 (2500 to 3320)	118.7 (106.7 to 133.2)	11.4 (10.7 to 12.2)	18.8 (17.1 to 20.6)	64.2 (45.9 to 82.9)
		Streptococcus pneumoniae	11000 (9440 to 12600)	4810 (4150 to 5530)	-56.2 (-58.5 to -53.6)	39.6 (37.2 to 42.2)	12.7 (11.5 to 13.9)	-67.9 (-71.6 to -64.3)
		Other viruses	7400 (6380 to 8510)	7250 (6200 to 8370)	-1.9 (-7.3 to 3.9)	10.5 (9.84 to 11.2)	7.68 (7 to 8.47)	-26.8 (-35.1 to -18.4)
		Other bacterial pathogens	1000 (816 to 1190)	1010 (826 to 1200)	0.6 (-8.3 to 10.7)	3.45 (3.01 to 3.92)	2.58 (2.18 to 2.99)	-25.1 (-35.2 to -14.3)
	50-69 years	*Acinetobacter baumannii*	424 (356 to 501)	260 (221 to 304)	-38.5 (-46.3 to -30.6)	3.95 (3.4 to 4.6)	1.84 (1.59 to 2.16)	-53.2 (-59.9 to -46.0)
		Chlamydia spp.	1690 (1460 to 1930)	1390 (1230 to 1570)	-17.6 (-27.3 to -7.3)	3.92 (3.49 to 4.41)	2.47 (2.17 to 2.8)	-36.7 (-45.1 to -26.5)
		Enterobacter spp.	411 (351 to 474)	853 (742 to 974)	108.0 (83.7 to 136.5)	3.64 (3.17 to 4.18)	5.83 (5.13 to 6.63)	60.7 (38.1 to 85.6)
		Escherichia coli	952 (831 to 1090)	1770 (1590 to 1960)	86.1 (65.7 to 106.3)	8.72 (7.82 to 9.69)	12.5 (11.2 to 13.8)	43.8 (25.7 to 63.7)
		Fungus	908 (783 to 1040)	1380 (1200 to 1580)	52.8 (34.9 to 72.1)	8.2 (7.3 to 9.15)	9.64 (8.41 to 10.9)	17.8 (1.6 to 37.1)
		Group B Streptococcus	847 (737 to 968)	1450 (1270 to 1630)	71.1 (49.5 to 92.9)	5.51 (4.88 to 6.25)	7.25 (6.35 to 8.15)	32.0 (13.3 to 53.4)
		Haemophilus influenzae	3270 (2910 to 3640)	4150 (3720 to 4600)	27.2 (15.3 to 39.2)	11.4 (10.5 to 12.4)	7.56 (6.83 to 8.39)	-33.8 (-41.6 to -25.6)
		Influenza	3810 (3360 to 4260)	74.1 (20.1 to 167)	-98.1 (-99.5 to -95.5)	26 (24 to 28.1)	0.37 (0.1 to 0.82)	-98.6 (-99.6 to -96.8)
		Klebsiella pneumoniae	1940 (1730 to 2170)	2270 (2040 to 2510)	16.9 (6.8 to 27.2)	17.7 (16.3 to 19.1)	15.9 (14.3 to 17.5)	-10.2 (-20.4 to 1.1)
		Legionella spp.	3020 (2640 to 3420)	4560 (4090 to 5060)	51.4 (34.6 to 69.6)	18.9 (17.1 to 21)	22.1 (19.8 to 24.5)	17.0 (0.9 to 32.9)
		Mycoplasma spp.	3170 (2800 to 3580)	2980 (2660 to 3300)	-6.0 (-15.5 to 3.8)	7.68 (6.92 to 8.43)	5.54 (5 to 6.13)	-27.6 (-36.2 to -18.3)
		Polymicrobial	96.4 (56.9 to 158)	148 (83.4 to 255)	53.3 (33.8 to 76.1)	0.89 (0.52 to 1.47)	1.06 (0.59 to 1.77)	18.1 (0.7 to 38.3)
		Pseudomonas aeruginosa	3110 (2730 to 3490)	5130 (4620 to 5650)	65.5 (49.1 to 82.6)	19.7 (17.9 to 21.5)	25 (22.7 to 27.5)	27.6 (11.3 to 43.9)
		Respiratory syncytial virus	1110 (968 to 1260)	14.4 (3.83 to 32.4)	-98.7 (-99.6 to -97.1)	3.47 (3.08 to 3.87)	0.03 (0.01 to 0.07)	-99.1 (-99.8 to -97.9)

		Staphylococcus aureus	3550 (3160 to 3950)	7990 (7180 to 8770)	125.2 (106.6 to 145.2)	46 (42.7 to 49.7)	80 (72.7 to 87.4)	74.1 (54.3 to 94.9)
		Streptococcus pneumoniae	14600 (13000 to 16200)	7440 (6690 to 8210)	-49.0 (-53.3 to -44.8)	78.8 (73.2 to 84.8)	30.7 (27.9 to 33.6)	-61.0 (-65.4 to -56.6)
		Other viruses	8600 (7660 to 9530)	9930 (8940 to 10900)	15.5 (5.7 to 25.4)	18.2 (16.9 to 19.6)	16.2 (14.8 to 17.8)	-11.1 (-20.9 to -0.3)
		Other bacterial pathogens	3270 (2790 to 3790)	3260 (2800 to 3780)	-0.1 (-10.6 to 11.0)	16.8 (14.6 to 19.4)	12.9 (11 to 15.2)	-23.0 (-33.0 to -12.2)
	70+ years	*Acinetobacter baumannii*	657 (548 to 783)	583 (498 to 674)	-11.0 (-21.1 to 0.3)	27.8 (23.2 to 32.4)	26.6 (20.6 to 31.6)	-4.3 (-18.7 to 9.2)
		Chlamydia spp.	3110 (2680 to 3520)	3720 (3290 to 4220)	19.7 (6.2 to 37.4)	32.5 (28 to 36.3)	42.1 (33.5 to 48.7)	29.7 (12.4 to 50.1)
		Enterobacter spp.	617 (524 to 724)	1870 (1630 to 2100)	203.5 (167.2 to 245.3)	24.4 (20.6 to 28.2)	80.8 (62.9 to 92.9)	231.4 (184.0 to 279.6)
		Escherichia coli	1800 (1570 to 2050)	4870 (4360 to 5400)	171.0 (143.2 to 203.3)	74.1 (64.2 to 82.3)	218 (175 to 249)	194.9 (154.5 to 230.8)
		Fungus	780 (672 to 902)	1730 (1500 to 1970)	122.6 (95.3 to 153.5)	31.6 (27 to 36.2)	76.5 (60.4 to 89)	142.2 (106.8 to 176.3)
		Group B Streptococcus	1200 (1040 to 1360)	2980 (2630 to 3350)	149.4 (119.0 to 184.3)	34.9 (29.8 to 39.3)	94.7 (75.3 to 109)	171.7 (134.4 to 210.4)
		Haemophilus influenzae	4530 (4040 to 5030)	8400 (7530 to 9290)	85.6 (68.6 to 104.6)	70.8 (61.9 to 77)	96.8 (77.1 to 110)	36.6 (19.9 to 52.1)
		Influenza	8910 (7920 to 9850)	253 (69.1 to 580)	-97.2 (-99.2 to -93.5)	272 (238 to 296)	7.92 (2.09 to 18)	-97.1 (-99.2 to -93.4)
		Klebsiella pneumoniae	3300 (2950 to 3650)	5610 (5030 to 6180)	70.2 (56.4 to 86.4)	135 (119 to 148)	250 (201 to 283)	85.1 (63.0 to 104.9)
		Legionella spp.	3330 (2900 to 3770)	7370 (6590 to 8140)	121.6 (98.2 to 151.2)	92.9 (80.5 to 105)	226 (181 to 256)	143.1 (108.5 to 175.5)
		Mycoplasma spp.	4130 (3640 to 4660)	5660 (5070 to 6250)	37.2 (23.5 to 53.8)	44.7 (38.7 to 49.4)	66.9 (53.1 to 76)	49.6 (32.0 to 67.8)
		Polymicrobial	171 (96.5 to 286)	381 (211 to 661)	122.7 (93.7 to 158.3)	7.14 (4.15 to 12.2)	17.3 (9.37 to 30.1)	141.1 (104.6 to 180.2)
		Pseudomonas aeruginosa	4970 (4420 to 5590)	12000 (10800 to 13200)	141.9 (119.0 to 166.9)	140 (123 to 153)	371 (297 to 420)	165.1 (132.0 to 194.8)
		Respiratory syncytial virus	1600 (1400 to 1820)	30.3 (8.04 to 69.9)	-98.1 (-99.5 to -95.7)	22.2 (19.2 to 24.7)	0.43 (0.11 to 0.98)	-98.1 (-99.5 to -95.6)
		Staphylococcus aureus	7080 (6310 to 7800)	23200 (20900 to 25600)	228.8 (203.5 to 258.8)	409 (358 to 444)	1470 (1180 to 1660)	259.2 (219.2 to 296.7)
		Streptococcus pneumoniae	23200 (20700 to 25500)	17200 (15500 to 18900)	-25.7 (-31.6 to -18.8)	559 (486 to 607)	453 (362 to 511)	-19.1 (-28.8 to -10.7)
		Other viruses	13400 (12000 to 14900)	22700 (20400 to 24900)	68.9 (55.4 to 84.9)	127 (112 to 137)	235 (188 to 265)	85.3 (64.9 to 104.8)
		Other bacterial pathogens	6750 (5780 to 7840)	9800 (8420 to 11400)	45.5 (30.5 to 63.1)	158 (134 to 182)	250 (190 to 301)	58.3 (36.5 to 78.8)
Australia	All Ages	*Acinetobacter baumannii*	901 (765 to 1050)	717 (621 to 822)	-20.2 (-29.7 to -10.0)	20.8 (17.7 to 24.2)	22.4 (17.5 to 26.6)	8.2 (-9.1 to 23.9)
		Chlamydia spp.	4480 (3960 to 4980)	4820 (4340 to 5330)	7.8 (-4.2 to 21.3)	24.8 (21.9 to 27.4)	36.7 (29.7 to 42.3)	47.9 (26.8 to 69.7)
		Enterobacter spp.	922 (799 to 1050)	2500 (2220 to 2780)	172.2 (140.4 to 205.8)	19.5 (16.7 to 22.5)	72.4 (57.6 to 83.3)	271.1 (214.3 to 328.2)

		Escherichia coli	2370 (2110 to 2660)	6020 (5440 to 6540)	154.5 (127.8 to 181.5)	55.9 (49.4 to 62.2)	192 (155 to 218)	243.8 (193.2 to 288.5)
		Fungus	1560 (1380 to 1770)	2960 (2630 to 3290)	90.1 (67.9 to 114.8)	27.8 (24.3 to 31.3)	72.2 (57.9 to 83.7)	159.5 (119.3 to 198.3)
		Group B Streptococcus	1960 (1750 to 2180)	4270 (3850 to 4690)	118.4 (92.7 to 146.8)	28.8 (25.3 to 32.1)	85.5 (69.6 to 97.5)	197.0 (155.1 to 241.4)
		Haemophilus influenzae	8310 (7590 to 9090)	12700 (11600 to 13800)	52.9 (40.2 to 65.4)	60.7 (55 to 65.2)	88.2 (71.4 to 99.8)	45.2 (25.4 to 62.1)
		Influenza	12200 (11100 to 13300)	313 (85.5 to 718)	-97.4 (-99.3 to -94.1)	206 (184 to 223)	6.97 (1.85 to 15.8)	-96.6 (-99.1 to -92.4)
		Klebsiella pneumoniae	4930 (4470 to 5360)	7180 (6570 to 7750)	45.8 (34.5 to 58.5)	106 (95.5 to 114)	218 (177 to 245)	106.1 (79.5 to 129.8)
		Legionella spp.	5900 (5250 to 6550)	11300 (10300 to 12200)	92.1 (71.5 to 114.3)	79.7 (70.9 to 88.7)	209 (171 to 236)	162.4 (122.3 to 199.2)
		Mycoplasma spp.	9190 (8340 to 10100)	11000 (10000 to 11800)	19.4 (9.6 to 30.2)	40.2 (36 to 43.8)	63.2 (51.8 to 71.4)	57.2 (37.0 to 76.9)
		Polymicrobial	254 (157 to 406)	491 (280 to 834)	92.3 (63.6 to 124.6)	6.19 (3.9 to 9.85)	15.5 (8.53 to 26.8)	147.7 (101.3 to 196.2)
		Pseudomonas aeruginosa	7440 (6730 to 8270)	16100 (14700 to 17400)	117.2 (97.1 to 138.1)	112 (100 to 123)	330 (269 to 371)	194.0 (153.3 to 228.3)
		Respiratory syncytial virus	5620 (5150 to 6200)	74.5 (20.3 to 171)	-98.7 (-99.6 to -97.0)	28.5 (26.3 to 30.7)	0.43 (0.11 to 0.97)	-98.5 (-99.6 to -96.6)
		Staphylococcus aureus	9580 (8680 to 10500)	28900 (26300 to 31100)	202.2 (178.0 to 228.0)	311 (279 to 335)	1300 (1050 to 1460)	315.7 (262.2 to 359.5)
		Streptococcus pneumoniae	43700 (40200 to 47400)	24800 (22900 to 26500)	-43.2 (-47.2 to -39.2)	473 (430 to 507)	396 (320 to 444)	-16.3 (-26.9 to -7.6)
		Other viruses	29500 (27200 to 31900)	36200 (33500 to 38600)	22.9 (14.9 to 30.8)	117 (106 to 125)	214 (175 to 240)	83.1 (59.8 to 102.3)
		Other bacterial pathogens	8440 (7260 to 9660)	11500 (10100 to 13300)	36.9 (21.9 to 51.5)	115 (98.3 to 132)	215 (164 to 258)	86.4 (59.4 to 111.2)
	Under 5	*Acinetobacter baumannii*	19.3 (11.9 to 30.8)	8.2 (4.89 to 13.7)	-57.7 (-63.2 to -52.2)	0.55 (0.3 to 0.95)	0.17 (0.08 to 0.31)	-69.1 (-76.2 to -59.9)
		Chlamydia spp.	227 (192 to 268)	124 (101 to 152)	-45.5 (-52.8 to -37.7)	0.78 (0.68 to 0.88)	0.21 (0.17 to 0.26)	-73.4 (-79.0 to -66.3)
		Enterobacter spp.	40.5 (29.9 to 52.5)	49.3 (36.7 to 64.4)	22.0 (3.2 to 43.9)	0.58 (0.42 to 0.85)	0.27 (0.18 to 0.39)	-53.0 (-63.8 to -40.8)
		Escherichia coli	108 (90.4 to 130)	146 (118 to 178)	35.0 (15.4 to 55.9)	1.49 (1.3 to 1.7)	0.72 (0.57 to 0.88)	-51.8 (-62.7 to -39.0)
		Fungus	18.9 (13.1 to 26.4)	19.3 (13.6 to 27.1)	2.4 (-16.4 to 22.0)	0.15 (0.11 to 0.2)	0.06 (0.04 to 0.09)	-60.7 (-72.1 to -46.0)
		Group B Streptococcus	108 (91.1 to 129)	111 (89.8 to 137)	3.1 (-12.1 to 18.0)	1.26 (1.1 to 1.44)	0.52 (0.41 to 0.64)	-59.0 (-68.4 to -47.9)
		Haemophilus influenzae	963 (808 to 1140)	683 (561 to 836)	-29.0 (-38.0 to -19.5)	3.68 (3.26 to 4.1)	0.58 (0.45 to 0.73)	-84.2 (-88.2 to -79.4)
		Influenza	757 (638 to 894)	7.4 (2.03 to 16.9)	-99.0 (-99.7 to -97.8)	5.35 (4.82 to 5.91)	0.02 (0 to 0.04)	-99.7 (-99.9 to -99.2)
		Klebsiella pneumoniae	371 (312 to 436)	197 (162 to 241)	-46.9 (-52.8 to -40.5)	3.75 (3.35 to 4.18)	0.9 (0.72 to 1.1)	-75.9 (-81.3 to -69.4)

		Legionella spp.	65.3 (48.2 to 86.4)	59.6 (41.7 to 82.8)	-8.6 (-21.0 to 5.1)	0.78 (0.61 to 0.99)	0.33 (0.24 to 0.43)	-57.8 (-67.1 to -46.5)
		Mycoplasma spp.	516 (432 to 617)	565 (461 to 692)	9.6 (-4.2 to 23.9)	1.3 (1.14 to 1.49)	0.54 (0.42 to 0.67)	-58.7 (-69.2 to -46.6)
		Polymicrobial	28.6 (15 to 48.1)	18.2 (9.81 to 30.7)	-36.2 (-44.7 to -26.5)	0.95 (0.49 to 1.64)	0.4 (0.18 to 0.74)	-57.9 (-67.9 to -45.7)
		Pseudomonas aeruginosa	341 (287 to 409)	475 (388 to 576)	39.6 (21.8 to 57.7)	2.77 (2.45 to 3.07)	1.42 (1.13 to 1.77)	-48.5 (-60.3 to -34.3)
		Respiratory syncytial virus	2740 (2350 to 3190)	27.6 (7.5 to 63.9)	-99.0 (-99.7 to -97.7)	9.83 (8.89 to 10.8)	0.04 (0.01 to 0.08)	-99.6 (-99.9 to -99.1)
		Staphylococcus aureus	345 (293 to 404)	483 (397 to 580)	40.0 (27.0 to 53.6)	4.86 (4.37 to 5.37)	2.55 (2.03 to 3.15)	-47.3 (-59.7 to -32.9)
		Streptococcus pneumoniae	4670 (3970 to 5470)	872 (714 to 1060)	-81.3 (-82.9 to -79.6)	23.8 (21.5 to 26.2)	1.64 (1.26 to 2.08)	-93.1 (-94.9 to -91.0)
		Other viruses	5280 (4470 to 6200)	2650 (2190 to 3210)	-49.8 (-53.6 to -45.3)	10.8 (9.84 to 11.9)	2.31 (1.83 to 2.86)	-78.6 (-83.6 to -72.6)
		Other bacterial pathogens	109 (84.1 to 138)	77.2 (58.1 to 100)	-29.1 (-39.2 to -17.2)	0.74 (0.57 to 0.99)	0.23 (0.16 to 0.33)	-69.5 (-76.5 to -61.2)
	5-14 years	*Acinetobacter baumannii*	24.3 (17.4 to 33.1)	13.6 (9.99 to 18.4)	-43.6 (-50.4 to -35.8)	0.05 (0.04 to 0.06)	0.01 (0.01 to 0.02)	-70.2 (-75.4 to -64.6)
		Chlamydia spp.	167 (122 to 221)	136 (99.3 to 180)	-18.5 (-27.6 to -8.2)	0.08 (0.07 to 0.1)	0.04 (0.03 to 0.04)	-56.9 (-64.4 to -48.4)
		Enterobacter spp.	25.2 (17.8 to 34.4)	50.8 (37 to 67.6)	102.0 (77.9 to 129.2)	0.05 (0.04 to 0.06)	0.05 (0.04 to 0.06)	6.9 (-11.2 to 26.9)
		Escherichia coli	52.4 (38.3 to 69.6)	100 (74.7 to 132)	91.9 (71.6 to 114.1)	0.1 (0.09 to 0.12)	0.11 (0.09 to 0.12)	1.5 (-14.4 to 19.5)
		Fungus	62.8 (45.9 to 84)	92.2 (68.3 to 124)	47.2 (29.1 to 66.6)	0.12 (0.11 to 0.14)	0.1 (0.08 to 0.11)	-22.1 (-35.6 to -7.8)
		Group B Streptococcus	73.1 (52.8 to 96.9)	124 (92 to 164)	69.8 (48.6 to 92.4)	0.1 (0.09 to 0.12)	0.09 (0.08 to 0.11)	-10.2 (-25.5 to 8.6)
		Haemophilus influenzae	332 (247 to 440)	381 (283 to 499)	14.9 (5.4 to 25.6)	0.25 (0.23 to 0.28)	0.1 (0.09 to 0.12)	-59.1 (-64.8 to -52.1)
		Influenza	448 (334 to 579)	7.95 (2.02 to 18.1)	-98.2 (-99.5 to -96.0)	0.67 (0.61 to 0.74)	0.01 (0 to 0.01)	-99.1 (-99.8 to -98.0)
		Klebsiella pneumoniae	153 (114 to 198)	148 (112 to 194)	-2.9 (-10.2 to 5.8)	0.3 (0.28 to 0.34)	0.16 (0.14 to 0.18)	-48.6 (-56.0 to -39.8)
		Legionella spp.	221 (163 to 290)	324 (244 to 429)	46.8 (30.9 to 63.1)	0.3 (0.27 to 0.34)	0.23 (0.2 to 0.27)	-22.3 (-34.8 to -8.6)
		Mycoplasma spp.	702 (517 to 913)	743 (559 to 975)	6.0 (-3.2 to 16.7)	0.37 (0.33 to 0.41)	0.21 (0.18 to 0.24)	-43.9 (-52.4 to -33.4)
		Polymicrobial	6.74 (3.76 to 11)	9.94 (5.34 to 16.7)	47.1 (28.3 to 67.6)	0.01 (0.01 to 0.02)	0.01 (0.01 to 0.02)	-22.2 (-35.9 to -5.6)
		Pseudomonas aeruginosa	225 (166 to 293)	383 (288 to 499)	70.8 (55.9 to 85.8)	0.31 (0.28 to 0.35)	0.28 (0.24 to 0.32)	-9.6 (-23.3 to 5.7)
		Respiratory syncytial virus	180 (135 to 237)	2.32 (0.58 to 5.31)	-98.7 (-99.6 to -97.1)	0.12 (0.11 to 0.14)	0 (0 to 0)	-99.4 (-99.8 to -98.5)
		Staphylococcus aureus	290 (217 to 375)	623 (472 to 812)	115.1 (101.1 to 132.0)	0.82 (0.74 to 0.9)	0.93 (0.81 to 1.04)	13.8 (-2.3 to 32.5)

		Streptococcus pneumoniae	2400 (1790 to 3100)	991 (746 to 1290)	-58.7 (-61.3 to -55.5)	2.82 (2.56 to 3.11)	0.62 (0.54 to 0.7)	-78.2 (-81.2 to -74.7)
		Other viruses	1640 (1220 to 2130)	1550 (1170 to 2020)	-5.5 (-11.6 to 2.5)	0.76 (0.69 to 0.84)	0.38 (0.33 to 0.42)	-50.0 (-57.1 to -41.9)
		Other bacterial pathogens	217 (158 to 290)	213 (156 to 282)	-2.0 (-11.4 to 8.4)	0.24 (0.21 to 0.28)	0.13 (0.11 to 0.15)	-48.1 (-55.9 to -39.2)
	15-49 years	*Acinetobacter baumannii*	87.5 (69 to 107)	49.9 (40.6 to 60.7)	-42.8 (-49.5 to -35.4)	0.56 (0.47 to 0.66)	0.27 (0.22 to 0.31)	-52.3 (-59.9 to -43.9)
		Chlamydia spp.	600 (506 to 710)	496 (415 to 582)	-17.3 (-26.1 to -7.1)	0.96 (0.85 to 1.07)	0.66 (0.58 to 0.76)	-31.0 (-41.2 to -19.7)
		Enterobacter spp.	90.9 (74 to 110)	186 (153 to 224)	104.9 (81.7 to 130.1)	0.55 (0.47 to 0.64)	0.94 (0.8 to 1.09)	71.0 (45.7 to 98.2)
		Escherichia coli	189 (159 to 223)	367 (309 to 429)	94.6 (76.2 to 115.7)	1.19 (1.06 to 1.33)	1.93 (1.71 to 2.15)	62.4 (39.7 to 88.2)
		Fungus	226 (189 to 269)	337 (283 to 402)	49.3 (31.8 to 68.8)	1.41 (1.25 to 1.59)	1.75 (1.51 to 2.01)	24.6 (6.2 to 44.7)
		Group B Streptococcus	264 (222 to 315)	453 (377 to 530)	72.2 (50.3 to 95.3)	1.18 (1.05 to 1.33)	1.69 (1.47 to 1.93)	43.7 (22.8 to 67.5)
		Haemophilus influenzae	1200 (1020 to 1390)	1390 (1180 to 1620)	16.6 (7.4 to 26.3)	2.88 (2.64 to 3.13)	1.88 (1.68 to 2.1)	-34.5 (-43.3 to -25.9)
		Influenza	1610 (1380 to 1860)	29.1 (7.97 to 65.7)	-98.2 (-99.5 to -95.9)	7.59 (7.03 to 8.19)	0.11 (0.03 to 0.24)	-98.6 (-99.6 to -96.8)
		Klebsiella pneumoniae	550 (470 to 639)	542 (459 to 627)	-1.5 (-8.2 to 6.5)	3.45 (3.17 to 3.73)	2.83 (2.54 to 3.15)	-17.8 (-27.6 to -7.0)
		Legionella spp.	796 (671 to 933)	1180 (1010 to 1370)	48.9 (34.2 to 66.3)	3.43 (3.09 to 3.81)	4.25 (3.82 to 4.76)	24.3 (6.3 to 43.8)
		Mycoplasma spp.	2530 (2150 to 2930)	2720 (2330 to 3150)	7.5 (-0.9 to 16.9)	4.21 (3.85 to 4.6)	3.77 (3.37 to 4.19)	-10.3 (-22.1 to 2.0)
		Polymicrobial	24.4 (13.6 to 40.7)	36.4 (20 to 62.8)	49.2 (30.6 to 70.0)	0.16 (0.09 to 0.26)	0.19 (0.11 to 0.33)	24.6 (4.4 to 49.0)
		Pseudomonas aeruginosa	810 (691 to 946)	1400 (1190 to 1630)	73.2 (59.8 to 90.3)	3.52 (3.2 to 3.86)	5.08 (4.59 to 5.63)	44.6 (25.3 to 63.8)
		Respiratory syncytial virus	648 (550 to 762)	8.51 (2.28 to 19.5)	-98.7 (-99.6 to -97.0)	1.39 (1.26 to 1.53)	0.01 (0 to 0.03)	-99.0 (-99.7 to -97.7)
		Staphylococcus aureus	1050 (898 to 1210)	2280 (1950 to 2640)	118.2 (104.8 to 135.3)	9.3 (8.62 to 10)	16.9 (15.3 to 18.7)	82.1 (60.6 to 104.3)
		Streptococcus pneumoniae	8660 (7470 to 9950)	3620 (3110 to 4190)	-58.1 (-60.7 to -55.0)	32.1 (29.9 to 34.3)	11.2 (10.1 to 12.4)	-65.0 (-69.4 to -60.8)
		Other viruses	5920 (5080 to 6820)	5670 (4810 to 6590)	-4.1 (-10.2 to 2.6)	8.62 (8.03 to 9.26)	6.89 (6.23 to 7.63)	-20.0 (-29.4 to -10.1)
		Other bacterial pathogens	785 (638 to 935)	779 (635 to 933)	-0.6 (-10.4 to 10.5)	2.77 (2.41 to 3.18)	2.3 (1.94 to 2.68)	-17.0 (-28.8 to -4.3)
	50-69 years	*Acinetobacter baumannii*	315 (262 to 372)	199 (167 to 234)	-36.7 (-45.3 to -27.5)	2.99 (2.54 to 3.5)	1.53 (1.31 to 1.8)	-48.6 (-56.4 to -40.0)
		Chlamydia spp.	1280 (1100 to 1480)	1100 (966 to 1250)	-13.4 (-24.4 to -1.3)	3.01 (2.64 to 3.43)	2.11 (1.84 to 2.4)	-29.7 (-40.2 to -17.5)
		Enterobacter spp.	317 (269 to 369)	694 (598 to 794)	119.4 (91.5 to 152.8)	2.85 (2.46 to 3.31)	5.06 (4.42 to 5.78)	78.0 (51.9 to 108.2)
		Escherichia coli	728 (630 to 838)	1440 (1280 to 1600)	98.0 (74.2 to 123.1)	6.78 (6.07 to 7.61)	10.9 (9.68 to 12)	60.6 (39.0 to 85.5)

		Fungus	694 (593 to 802)	1120 (962 to 1280)	61.1 (40.2 to 84.1)	6.37 (5.58 to 7.22)	8.3 (7.22 to 9.45)	30.7 (11.3 to 53.7)
		Group B Streptococcus	651 (563 to 747)	1170 (1010 to 1330)	80.2 (55.6 to 106.8)	4.3 (3.77 to 4.94)	6.26 (5.47 to 7.09)	46.1 (23.4 to 71.3)
		Haemophilus influenzae	2520 (2220 to 2840)	3380 (2990 to 3760)	34.2 (19.8 to 48.4)	8.96 (8.15 to 9.8)	6.55 (5.89 to 7.3)	-26.7 (-36.3 to -15.6)
		Influenza	2930 (2580 to 3310)	60.7 (16.4 to 136)	-97.9 (-99.4 to -95.3)	20.4 (18.5 to 22.2)	0.32 (0.08 to 0.72)	-98.4 (-99.6 to -96.4)
		Klebsiella pneumoniae	1490 (1310 to 1680)	1810 (1620 to 2010)	21.9 (9.4 to 34.8)	13.7 (12.5 to 15)	13.6 (12.2 to 15)	-1.1 (-13.7 to 12.8)
		Legionella spp.	2360 (2050 to 2700)	3720 (3310 to 4160)	58.0 (38.7 to 80.1)	15 (13.4 to 16.8)	19.2 (17.1 to 21.4)	28.2 (9.3 to 47.5)
		Mycoplasma spp.	2440 (2130 to 2780)	2390 (2120 to 2660)	-2.1 (-13.2 to 8.9)	6.01 (5.37 to 6.67)	4.76 (4.27 to 5.28)	-20.6 (-31.0 to -8.7)
		Polymicrobial	72.9 (42.9 to 120)	119 (67.4 to 207)	62.9 (40.5 to 89.0)	0.69 (0.4 to 1.14)	0.91 (0.51 to 1.53)	32.2 (10.6 to 55.9)
		Pseudomonas aeruginosa	2420 (2120 to 2740)	4150 (3710 to 4590)	71.9 (52.5 to 91.4)	15.5 (13.9 to 17.2)	21.6 (19.5 to 23.8)	39.5 (19.9 to 59.2)
		Respiratory syncytial virus	871 (754 to 994)	11.6 (3.03 to 26.1)	-98.7 (-99.6 to -97.0)	2.76 (2.43 to 3.1)	0.03 (0.01 to 0.06)	-99.0 (-99.7 to -97.7)
		Staphylococcus aureus	2750 (2430 to 3090)	6520 (5790 to 7180)	137.7 (114.7 to 163.9)	36.1 (33 to 39.3)	69.5 (62.8 to 76.3)	92.9 (68.5 to 118.7)
		Streptococcus pneumoniae	11200 (9980 to 12600)	5830 (5200 to 6440)	-48.0 (-53.2 to -42.7)	61.5 (56.3 to 67)	25.9 (23.4 to 28.4)	-57.9 (-63.1 to -52.3)
		Other viruses	6720 (5950 to 7540)	8000 (7140 to 8820)	19.2 (7.2 to 31.6)	14.5 (13.2 to 15.7)	14 (12.6 to 15.4)	-3.3 (-15.3 to 9.6)
		Other bacterial pathogens	2500 (2120 to 2910)	2610 (2220 to 3060)	4.8 (-7.5 to 18.4)	13.1 (11.2 to 15.2)	11.1 (9.43 to 13.1)	-14.9 (-26.5 to -1.6)
	70+ years	*Acinetobacter baumannii*	455 (376 to 544)	446 (379 to 519)	-1.6 (-13.9 to 11.7)	16.6 (13.9 to 19.5)	20.5 (15.7 to 24.3)	23.2 (3.9 to 42.3)
		Chlamydia spp.	2200 (1890 to 2520)	2960 (2590 to 3370)	34.6 (18.2 to 54.9)	20 (17.3 to 22.4)	33.7 (26.9 to 39)	68.6 (44.8 to 95.0)
		Enterobacter spp.	448 (379 to 526)	1520 (1310 to 1720)	241.1 (195.4 to 294.1)	15.5 (13.1 to 17.9)	66.1 (51.4 to 76.3)	327.1 (264.8 to 391.3)
		Escherichia coli	1300 (1130 to 1490)	3970 (3520 to 4420)	207.8 (171.8 to 246.7)	46.4 (40.3 to 51.8)	179 (143 to 204)	285.3 (228.0 to 335.3)
		Fungus	560 (477 to 650)	1400 (1200 to 1600)	150.5 (117.5 to 187.3)	19.8 (16.9 to 22.6)	62 (49 to 72.6)	213.6 (165.1 to 260.5)
		Group B Streptococcus	865 (746 to 992)	2410 (2110 to 2730)	180.1 (142.0 to 222.5)	22 (18.9 to 24.7)	77 (60.9 to 88.6)	250.6 (199.4 to 305.0)
		Haemophilus influenzae	3290 (2900 to 3680)	6860 (6070 to 7610)	108.6 (86.5 to 132.9)	45 (39.6 to 49)	79.1 (62.5 to 90.4)	75.8 (52.5 to 97.1)
		Influenza	6460 (5660 to 7190)	208 (57 to 477)	-96.8 (-99.1 to -92.7)	172 (151 to 188)	6.52 (1.73 to 14.8)	-96.2 (-99.0 to -91.5)
		Klebsiella pneumoniae	2370 (2090 to 2650)	4490 (3980 to 4960)	89.6 (72.0 to 109.9)	84.4 (74.4 to 92.3)	200 (160 to 228)	137.3 (106.0 to 164.7)
		Legionella spp.	2460 (2110 to 2810)	6020 (5320 to 6680)	145.7 (116.6 to 180.4)	60.2 (52.3 to 67.3)	185 (148 to 210)	207.6 (162.5 to 252.0)
		Mycoplasma spp.	2990 (2610 to 3390)	4550 (4030 to 5030)	52.2 (34.9 to 71.8)	28.3 (24.6 to 31.3)	53.9 (42.7 to 61.6)	90.5 (65.5 to 116.3)

		Polymicrobial	121 (68.3 to 205)	307 (169 to 529)	153.3 (118.2 to 196.3)	4.38 (2.52 to 7.46)	14 (7.46 to 24.3)	217.1 (166.6 to 270.2)
		Pseudomonas aeruginosa	3650 (3210 to 4160)	9730 (8610 to 10700)	167.3 (138.8 to 198.4)	90.1 (78.8 to 99.3)	302 (242 to 342)	234.6 (188.6 to 275.3)
		Respiratory syncytial virus	1180 (1030 to 1360)	24.5 (6.56 to 56.2)	-97.9 (-99.5 to -95.3)	14.4 (12.5 to 16)	0.35 (0.09 to 0.8)	-97.6 (-99.4 to -94.5)
		Staphylococcus aureus	5150 (4530 to 5700)	19000 (16900 to 21000)	269.6 (235.0 to 310.4)	260 (229 to 283)	1210 (965 to 1360)	362.7 (303.1 to 413.9)
		Streptococcus pneumoniae	16700 (14800 to 18600)	13500 (12000 to 14800)	-19.2 (-26.8 to -10.9)	353 (311 to 384)	357 (284 to 404)	1.1 (-11.8 to 12.3)
		Other viruses	9900 (8760 to 11000)	18300 (16200 to 20200)	85.3 (68.0 to 105.1)	81.9 (72 to 89)	190 (153 to 215)	131.9 (103.5 to 157.6)
		Other bacterial pathogens	4840 (4080 to 5640)	7870 (6710 to 9120)	63.0 (43.9 to 83.9)	98.4 (83.1 to 114)	201 (152 to 242)	104.2 (74.6 to 131.7)
New Zealand	All Ages	*Acinetobacter baumannii*	357 (303 to 415)	226 (197 to 258)	-36.6 (-43.4 to -29.3)	12.8 (10.7 to 14.8)	6.65 (5.26 to 7.85)	-48.0 (-55.5 to -41.3)
		Chlamydia spp.	1660 (1480 to 1830)	1290 (1180 to 1430)	-21.7 (-29.5 to -12.9)	14.3 (12.4 to 15.8)	9.08 (7.29 to 10.4)	-36.3 (-44.7 to -27.3)
		Enterobacter spp.	312 (273 to 356)	586 (525 to 645)	88.2 (67.9 to 110.8)	10.2 (8.63 to 11.8)	15.7 (12.4 to 18.3)	54.2 (32.5 to 76.3)
		Escherichia coli	849 (758 to 945)	1410 (1300 to 1540)	66.6 (49.5 to 83.9)	31 (26.8 to 34.4)	42.2 (34.3 to 47.5)	36.2 (18.2 to 53.4)
		Fungus	526 (465 to 595)	738 (663 to 819)	40.7 (25.2 to 58.2)	14.1 (12.2 to 16.1)	16.1 (13 to 18.7)	14.2 (-1.0 to 30.7)
		Group B Streptococcus	672 (602 to 750)	1050 (964 to 1150)	56.4 (39.4 to 74.4)	15.3 (13.2 to 17.2)	19.3 (15.7 to 22.1)	25.9 (9.0 to 42.4)
		Haemophilus influenzae	2810 (2600 to 3050)	3040 (2820 to 3280)	8.3 (0.0 to 16.1)	31.2 (27.7 to 33.8)	19.1 (15.5 to 21.6)	-38.6 (-45.6 to -31.5)
		Influenza	4220 (3890 to 4600)	71.3 (19.3 to 164)	-98.3 (-99.5 to -96.1)	111 (96.9 to 121)	1.47 (0.38 to 3.3)	-98.7 (-99.7 to -97.0)
		Klebsiella pneumoniae	1750 (1620 to 1910)	1870 (1740 to 2020)	7.1 (-0.9 to 15.4)	57.7 (50.5 to 62.9)	52.8 (42.7 to 59.6)	-8.5 (-18.8 to 1.3)
		Legionella spp.	1830 (1650 to 2030)	2640 (2430 to 2850)	44.2 (29.5 to 59.8)	38.1 (33.1 to 42.8)	44.5 (36 to 50.2)	17.0 (1.4 to 31.7)
		Mycoplasma spp.	2990 (2760 to 3270)	2970 (2750 to 3200)	-0.7 (-8.6 to 7.3)	19.9 (17.5 to 22)	14.5 (11.8 to 16.4)	-27.1 (-36.1 to -18.2)
		Polymicrobial	96.1 (60.9 to 152)	125 (72.8 to 203)	29.3 (10.8 to 46.7)	3.71 (2.42 to 5.73)	3.81 (2.22 to 6.39)	1.9 (-16.1 to 19.8)
		Pseudomonas aeruginosa	2420 (2210 to 2670)	3930 (3650 to 4250)	62.6 (49.4 to 76.8)	56.4 (49.3 to 62.1)	74.2 (59.7 to 83.7)	31.5 (14.0 to 47.0)
		Respiratory syncytial virus	1930 (1760 to 2110)	20.9 (5.67 to 47.6)	-98.9 (-99.7 to -97.5)	14.3 (13 to 15.5)	0.1 (0.03 to 0.24)	-99.3 (-99.8 to -98.3)
		Staphylococcus aureus	3250 (2990 to 3530)	6690 (6200 to 7260)	106.1 (90.9 to 121.8)	164 (143 to 178)	279 (225 to 314)	70.2 (52.0 to 89.4)
		Streptococcus pneumoniae	14900 (13900 to 16100)	7250 (6750 to 7780)	-51.3 (-54.4 to -48.1)	245 (216 to 266)	103 (83.9 to 116)	-57.8 (-62.6 to -53.3)
		Other viruses	9450 (8830 to 10200)	9340 (8710 to 10000)	-1.2 (-7.3 to 5.2)	56.1 (49.7 to 60.7)	48.8 (39.9 to 54.9)	-13.0 (-22.6 to -3.8)
		Other bacterial pathogens	3030 (2630 to 3480)	2930 (2540 to 3330)	-3.3 (-11.9 to 6.7)	64.3 (54.2 to 74.4)	51.1 (39.6 to 60.5)	-20.6 (-30.6 to -10.4)

	Under 5	*Acinetobacter baumannii*	9.52 (5.95 to 15)	3.49 (2.12 to 5.66)	-63.5 (-67.6 to -59.4)	0.43 (0.23 to 0.75)	0.13 (0.07 to 0.24)	-69.5 (-76.2 to -62.0)
		Chlamydia spp.	102 (88 to 119)	45.5 (38.2 to 54.3)	-55.5 (-61.3 to -49.2)	0.54 (0.48 to 0.61)	0.14 (0.11 to 0.16)	-74.8 (-79.4 to -69.8)
		Enterobacter spp.	16.8 (12.9 to 21.8)	16 (12.1 to 20.7)	-4.6 (-19.3 to 11.6)	0.38 (0.26 to 0.57)	0.16 (0.1 to 0.23)	-58.9 (-66.3 to -50.3)
		Escherichia coli	46.4 (39.7 to 54.9)	47.1 (38.7 to 56.6)	1.6 (-13.1 to 16.6)	0.98 (0.85 to 1.12)	0.38 (0.32 to 0.45)	-60.5 (-67.6 to -52.5)
		Fungus	7.89 (5.6 to 10.9)	6.43 (4.58 to 8.86)	-18.2 (-32.0 to -3.1)	0.08 (0.06 to 0.11)	0.02 (0.02 to 0.03)	-70.1 (-76.9 to -62.4)
		Group B Streptococcus	46.5 (39.4 to 54.7)	37.7 (31.3 to 45.5)	-18.9 (-29.7 to -6.9)	0.87 (0.77 to 0.99)	0.32 (0.26 to 0.38)	-63.4 (-70.1 to -56.0)
		Haemophilus influenzae	391 (332 to 453)	216 (181 to 259)	-44.5 (-51.7 to -36.9)	2.09 (1.86 to 2.35)	0.23 (0.19 to 0.28)	-88.7 (-91.1 to -86.2)
		Influenza	309 (265 to 355)	2.27 (0.64 to 5.22)	-99.3 (-99.8 to -98.3)	2.99 (2.66 to 3.31)	0.01 (0 to 0.02)	-99.7 (-99.9 to -99.4)
		Klebsiella pneumoniae	157 (135 to 180)	70.2 (59.1 to 83.3)	-55.1 (-60.5 to -49.4)	2.23 (1.99 to 2.48)	0.5 (0.42 to 0.58)	-77.6 (-81.5 to -73.1)
		Legionella spp.	26.5 (19.5 to 34.5)	19.7 (14.2 to 26.5)	-25.6 (-35.6 to -14.8)	0.52 (0.41 to 0.66)	0.21 (0.16 to 0.27)	-59.8 (-67.0 to -50.8)
		Mycoplasma spp.	211 (180 to 247)	193 (162 to 231)	-8.4 (-20.5 to 4.1)	0.74 (0.65 to 0.83)	0.25 (0.21 to 0.29)	-66.1 (-72.5 to -59.1)
		Polymicrobial	13.1 (7.08 to 22.2)	7 (3.75 to 11.8)	-46.6 (-53.0 to -39.7)	0.71 (0.36 to 1.2)	0.3 (0.14 to 0.52)	-58.3 (-67.0 to -49.2)
		Pseudomonas aeruginosa	134 (115 to 157)	156 (132 to 185)	16.4 (1.8 to 31.3)	1.63 (1.45 to 1.82)	0.71 (0.6 to 0.83)	-56.0 (-63.8 to -46.8)
		Respiratory syncytial virus	1050 (920 to 1180)	9.11 (2.54 to 20.8)	-99.1 (-99.8 to -98.0)	5.41 (4.9 to 5.95)	0.02 (0 to 0.04)	-99.7 (-99.9 to -99.3)
		Staphylococcus aureus	139 (120 to 158)	152 (128 to 180)	9.5 (-1.1 to 21.0)	2.72 (2.46 to 3)	1.13 (0.95 to 1.32)	-58.3 (-65.4 to -50.5)
		Streptococcus pneumoniae	1910 (1650 to 2190)	336 (283 to 399)	-82.4 (-84.1 to -80.6)	12.6 (11.4 to 13.8)	0.78 (0.65 to 0.92)	-93.8 (-94.9 to -92.5)
		Other viruses	2010 (1750 to 2290)	891 (756 to 1050)	-55.7 (-59.8 to -51.4)	5.48 (4.96 to 6.01)	1.08 (0.91 to 1.26)	-80.2 (-83.7 to -76.4)
		Other bacterial pathogens	46.5 (36.3 to 57.9)	26.9 (20.6 to 34.4)	-42.1 (-50.0 to -32.7)	0.47 (0.35 to 0.65)	0.13 (0.09 to 0.2)	-72.5 (-77.7 to -66.3)
	5-14 years	*Acinetobacter baumannii*	9.84 (7.05 to 13.3)	6.51 (4.89 to 8.66)	-33.6 (-40.8 to -25.9)	0.02 (0.02 to 0.03)	0 (0 to 0)	-85.1 (-87.5 to -82.2)
		Chlamydia spp.	62.8 (46.1 to 83.5)	54.7 (41.6 to 71.6)	-12.6 (-21.1 to -2.8)	0.04 (0.03 to 0.04)	0.01 (0.01 to 0.01)	-80.4 (-83.4 to -76.7)
		Enterobacter spp.	8.72 (6.28 to 11.8)	18 (13.4 to 23.7)	107.1 (83.6 to 134.3)	0.02 (0.02 to 0.02)	0.01 (0.01 to 0.01)	-53.6 (-61.0 to -44.8)
		Escherichia coli	18.8 (13.7 to 24.9)	35.6 (26.9 to 46.4)	89.6 (70.4 to 110.2)	0.04 (0.04 to 0.05)	0.02 (0.02 to 0.02)	-57.5 (-63.5 to -49.5)
		Fungus	22.6 (16.6 to 30.1)	34.4 (26 to 45.5)	52.9 (34.7 to 72.5)	0.05 (0.04 to 0.06)	0.02 (0.01 to 0.02)	-65.7 (-71.1 to -59.1)
		Group B Streptococcus	25.8 (18.8 to 33.7)	45.4 (34.5 to 59)	76.9 (57.3 to 99.7)	0.04 (0.04 to 0.05)	0.02 (0.01 to 0.02)	-60.3 (-66.4 to -52.8)

		Haemophilus influenzae	115 (84.4 to 151)	133 (101 to 175)	16.4 (6.2 to 27.5)	0.1 (0.09 to 0.11)	0.02 (0.01 to 0.02)	-82.3 (-84.9 to -79.4)
		Influenza	156 (116 to 203)	2.73 (0.7 to 6.13)	-98.2 (-99.5 to -96.0)	0.26 (0.24 to 0.28)	0 (0 to 0)	-99.6 (-99.9 to -99.2)
		Klebsiella pneumoniae	54.9 (40.8 to 71.9)	57.5 (44 to 74.1)	4.8 (-3.2 to 13.6)	0.12 (0.11 to 0.13)	0.03 (0.03 to 0.03)	-76.5 (-79.8 to -72.4)
		Legionella spp.	72.2 (53.3 to 95.3)	113 (87 to 147)	57.5 (41.4 to 76.2)	0.11 (0.1 to 0.12)	0.04 (0.03 to 0.04)	-64.7 (-70.0 to -58.1)
		Mycoplasma spp.	244 (179 to 317)	285 (219 to 367)	16.8 (6.9 to 28.2)	0.14 (0.13 to 0.16)	0.04 (0.03 to 0.04)	-73.8 (-77.7 to -69.3)
		Polymicrobial	2.54 (1.45 to 4.12)	3.78 (2.08 to 6.19)	48.1 (29.8 to 68.3)	0.01 (0 to 0.01)	0 (0 to 0)	-66.8 (-72.3 to -59.7)
		Pseudomonas aeruginosa	74.5 (54.8 to 97.2)	140 (108 to 180)	87.7 (71.3 to 106.9)	0.11 (0.1 to 0.13)	0.05 (0.04 to 0.05)	-57.9 (-64.1 to -51.1)
		Respiratory syncytial virus	58.3 (43.3 to 77)	0.86 (0.22 to 1.92)	-98.5 (-99.6 to -96.7)	0.04 (0.04 to 0.05)	0 (0 to 0)	-99.7 (-99.9 to -99.3)
		Staphylococcus aureus	99.5 (73.5 to 130)	218 (168 to 279)	119.3 (103.9 to 137.5)	0.31 (0.29 to 0.34)	0.15 (0.14 to 0.17)	-50.8 (-57.3 to -42.9)
		Streptococcus pneumoniae	845 (621 to 1100)	427 (330 to 550)	-49.3 (-52.8 to -45.2)	1.1 (1.02 to 1.2)	0.12 (0.11 to 0.14)	-88.6 (-90.2 to -86.8)
		Other viruses	538 (398 to 705)	572 (441 to 738)	6.6 (-0.5 to 16.0)	0.28 (0.25 to 0.3)	0.07 (0.06 to 0.07)	-76.1 (-79.2 to -72.1)
		Other bacterial pathogens	78.8 (57.8 to 105)	82 (61.2 to 108)	4.3 (-5.7 to 15.4)	0.1 (0.09 to 0.11)	0.02 (0.02 to 0.03)	-76.6 (-80.1 to -72.5)
	15-49 years	*Acinetobacter baumannii*	27.1 (21.4 to 33.1)	18 (14.7 to 21.8)	-33.2 (-41.0 to -25.3)	0.15 (0.13 to 0.18)	0.04 (0.03 to 0.05)	-73.8 (-77.3 to -69.7)
		Chlamydia spp.	173 (146 to 203)	152 (128 to 176)	-12.1 (-20.8 to -2.6)	0.24 (0.22 to 0.27)	0.08 (0.07 to 0.09)	-65.6 (-70.0 to -60.7)
		Enterobacter spp.	24 (19.5 to 29)	49.8 (41.2 to 59.2)	108.3 (85.1 to 133.0)	0.13 (0.11 to 0.15)	0.1 (0.09 to 0.12)	-18.4 (-29.1 to -5.6)
		Escherichia coli	51.8 (43.5 to 60.6)	98.6 (84.1 to 114)	90.8 (72.2 to 110.0)	0.29 (0.26 to 0.32)	0.21 (0.19 to 0.23)	-25.2 (-34.7 to -14.5)
		Fungus	62.1 (51.6 to 73.8)	95.3 (79.9 to 112)	53.8 (37.3 to 71.9)	0.34 (0.3 to 0.38)	0.2 (0.18 to 0.23)	-39.7 (-47.5 to -30.4)
		Group B Streptococcus	70.9 (60 to 85.4)	126 (107 to 147)	78.0 (58.8 to 100.4)	0.28 (0.25 to 0.31)	0.19 (0.17 to 0.22)	-30.3 (-39.5 to -20.1)
		Haemophilus influenzae	315 (269 to 366)	369 (315 to 424)	17.1 (8.0 to 26.7)	0.66 (0.61 to 0.72)	0.21 (0.19 to 0.22)	-69.0 (-72.3 to -65.1)
		Influenza	429 (370 to 500)	7.58 (2.1 to 17.5)	-98.2 (-99.5 to -95.9)	1.77 (1.64 to 1.91)	0.01 (0 to 0.03)	-99.3 (-99.8 to -98.6)
		Klebsiella pneumoniae	151 (130 to 175)	159 (137 to 184)	5.5 (-1.9 to 13.3)	0.83 (0.77 to 0.9)	0.34 (0.31 to 0.37)	-58.7 (-63.3 to -53.5)
		Legionella spp.	199 (169 to 232)	314 (269 to 359)	58.5 (42.8 to 75.5)	0.75 (0.68 to 0.84)	0.47 (0.42 to 0.51)	-37.9 (-46.2 to -29.2)
		Mycoplasma spp.	672 (578 to 776)	789 (678 to 902)	17.5 (7.7 to 27.1)	0.98 (0.9 to 1.07)	0.45 (0.41 to 0.49)	-54.0 (-59.3 to -47.7)
		Polymicrobial	7.01 (4.02 to 11.3)	10.5 (5.74 to 17.7)	49.0 (31.4 to 69.1)	0.04 (0.02 to 0.06)	0.02 (0.01 to 0.04)	-41.6 (-50.1 to -31.3)

		Pseudomonas aeruginosa	205 (175 to 239)	386 (334 to 443)	88.8 (73.5 to 105.1)	0.78 (0.71 to 0.86)	0.58 (0.53 to 0.63)	-26.0 (-34.5 to -16.9)
		Respiratory syncytial virus	160 (137 to 187)	2.38 (0.64 to 5.5)	-98.5 (-99.6 to -96.6)	0.3 (0.27 to 0.33)	0 (0 to 0)	-99.5 (-99.9 to -98.8)
		Staphylococcus aureus	274 (236 to 317)	604 (522 to 692)	120.7 (107.1 to 135.8)	2.14 (1.99 to 2.3)	1.84 (1.7 to 2)	-13.5 (-22.5 to -3.2)
		Streptococcus pneumoniae	2320 (2010 to 2660)	1180 (1020 to 1350)	-49.0 (-52.0 to -45.7)	7.55 (7.06 to 8.09)	1.51 (1.38 to 1.63)	-80.0 (-82.2 to -77.7)
		Other viruses	1480 (1280 to 1700)	1580 (1370 to 1800)	7.3 (1.0 to 14.4)	1.89 (1.76 to 2.03)	0.79 (0.73 to 0.86)	-58.0 (-62.2 to -53.1)
		Other bacterial pathogens	217 (178 to 258)	227 (188 to 271)	4.9 (-4.5 to 15.5)	0.67 (0.59 to 0.77)	0.28 (0.24 to 0.32)	-58.8 (-63.8 to -53.1)
	50-69 years	*Acinetobacter baumannii*	109 (90.8 to 129)	61.4 (51.9 to 71.8)	-43.6 (-50.6 to -36.2)	0.97 (0.82 to 1.12)	0.32 (0.27 to 0.37)	-67.2 (-71.9 to -62.1)
		Chlamydia spp.	412 (360 to 467)	286 (251 to 328)	-30.5 (-39.2 to -21.5)	0.91 (0.81 to 1.02)	0.36 (0.32 to 0.41)	-59.6 (-65.2 to -53.4)
		Enterobacter spp.	93.8 (80.3 to 108)	159 (137 to 182)	69.6 (48.1 to 93.0)	0.79 (0.69 to 0.9)	0.77 (0.68 to 0.87)	-1.5 (-15.4 to 13.9)
		Escherichia coli	224 (196 to 254)	330 (295 to 371)	47.7 (30.1 to 66.0)	1.94 (1.73 to 2.16)	1.66 (1.51 to 1.83)	-14.2 (-25.5 to -1.8)
		Fungus	214 (184 to 245)	269 (234 to 310)	26.3 (10.1 to 42.7)	1.83 (1.61 to 2.07)	1.34 (1.17 to 1.52)	-26.7 (-37.0 to -14.8)
		Group B Streptococcus	196 (169 to 224)	276 (243 to 315)	41.2 (23.9 to 61.0)	1.21 (1.07 to 1.38)	0.99 (0.87 to 1.11)	-18.0 (-29.1 to -5.2)
		Haemophilus influenzae	746 (662 to 830)	772 (692 to 862)	3.7 (-6.7 to 14.5)	2.47 (2.25 to 2.7)	1 (0.9 to 1.11)	-59.2 (-64.2 to -53.3)
		Influenza	874 (777 to 971)	13.4 (3.68 to 30.6)	-98.5 (-99.6 to -96.4)	5.66 (5.16 to 6.19)	0.05 (0.01 to 0.11)	-99.2 (-99.8 to -98.1)
		Klebsiella pneumoniae	458 (408 to 504)	460 (413 to 512)	0.7 (-9.3 to 10.9)	3.95 (3.6 to 4.33)	2.3 (2.07 to 2.54)	-41.5 (-48.5 to -33.8)
		Legionella spp.	660 (576 to 744)	844 (754 to 945)	28.1 (12.9 to 44.7)	3.92 (3.52 to 4.39)	2.91 (2.63 to 3.2)	-25.6 (-35.9 to -15.0)
		Mycoplasma spp.	728 (642 to 814)	592 (529 to 663)	-18.7 (-27.2 to -9.4)	1.67 (1.51 to 1.85)	0.79 (0.71 to 0.87)	-52.8 (-58.6 to -46.0)
		Polymicrobial	23.5 (14.2 to 38.2)	29.2 (16.5 to 49.1)	23.8 (7.3 to 41.5)	0.21 (0.12 to 0.34)	0.15 (0.08 to 0.25)	-28.1 (-39.0 to -15.8)
		Pseudomonas aeruginosa	687 (603 to 769)	981 (878 to 1100)	43.1 (28.3 to 58.8)	4.12 (3.72 to 4.54)	3.41 (3.1 to 3.75)	-16.9 (-27.5 to -5.4)
		Respiratory syncytial virus	242 (210 to 274)	2.77 (0.75 to 6.3)	-98.9 (-99.7 to -97.4)	0.71 (0.63 to 0.8)	0 (0 to 0.01)	-99.4 (-99.8 to -98.6)
		Staphylococcus aureus	807 (721 to 889)	1470 (1320 to 1640)	82.9 (65.6 to 101.3)	9.89 (9.05 to 10.8)	10.5 (9.51 to 11.5)	6.2 (-6.3 to 20.0)
		Streptococcus pneumoniae	3380 (3030 to 3720)	1610 (1450 to 1800)	-52.2 (-57.0 to -47.7)	17.3 (15.8 to 18.8)	4.78 (4.33 to 5.23)	-72.3 (-75.5 to -68.6)
		Other viruses	1880 (1690 to 2080)	1930 (1720 to 2150)	2.5 (-7.8 to 12.6)	3.78 (3.44 to 4.13)	2.25 (2.04 to 2.46)	-40.5 (-47.5 to -32.7)
		Other bacterial pathogens	774 (652 to 889)	651 (552 to 758)	-15.7 (-25.0 to -5.8)	3.77 (3.28 to 4.32)	1.85 (1.58 to 2.16)	-51.0 (-57.3 to -43.1)

	70+ years	*Acinetobacter baumannii*	201 (166 to 240)	136 (117 to 159)	-32.1 (-40.2 to -23.0)	11.2 (9.21 to 13.1)	6.16 (4.79 to 7.32)	-45.0 (-53.0 to -37.8)
		Chlamydia spp.	907 (781 to 1040)	757 (671 to 857)	-16.3 (-26.1 to -3.8)	12.5 (10.7 to 14)	8.49 (6.75 to 9.77)	-32.2 (-41.3 to -22.2)
		Enterobacter spp.	169 (142 to 196)	343 (300 to 388)	104.2 (79.8 to 133.1)	8.91 (7.42 to 10.4)	14.7 (11.4 to 17.1)	65.3 (42.1 to 89.7)
		Escherichia coli	508 (442 to 587)	900 (806 to 1000)	77.8 (57.2 to 100.6)	27.7 (23.7 to 31.1)	39.9 (32.2 to 45.2)	43.9 (24.6 to 62.7)
		Fungus	220 (188 to 258)	333 (291 to 380)	52.1 (33.0 to 74.4)	11.8 (9.97 to 13.6)	14.6 (11.5 to 17)	23.1 (6.5 to 40.7)
		Group B Streptococcus	333 (286 to 385)	564 (502 to 635)	70.1 (48.3 to 93.8)	12.9 (10.9 to 14.7)	17.7 (14.2 to 20.5)	37.6 (19.0 to 56.4)
		Haemophilus influenzae	1240 (1100 to 1400)	1550 (1400 to 1720)	24.9 (12.7 to 38.3)	25.8 (22.3 to 28.4)	17.7 (14.1 to 20.1)	-31.6 (-39.3 to -23.3)
		Influenza	2460 (2170 to 2760)	45.3 (12.3 to 106)	-98.2 (-99.5 to -95.8)	100 (86.2 to 110)	1.4 (0.36 to 3.16)	-98.6 (-99.6 to -96.8)
		Klebsiella pneumoniae	931 (825 to 1050)	1130 (1020 to 1250)	21.3 (9.5 to 33.0)	50.5 (43.5 to 55.5)	49.6 (39.7 to 56.3)	-1.9 (-13.1 to 9.3)
		Legionella spp.	877 (756 to 1000)	1350 (1220 to 1500)	54.3 (37.0 to 73.8)	32.8 (28 to 37.2)	40.9 (32.7 to 46.3)	24.9 (7.6 to 40.7)
		Mycoplasma spp.	1140 (998 to 1290)	1110 (1000 to 1240)	-2.0 (-12.7 to 9.6)	16.4 (14 to 18.3)	13 (10.3 to 14.8)	-20.7 (-30.4 to -10.6)
		Polymicrobial	49.8 (28.9 to 82.7)	74.4 (41.5 to 127)	49.1 (28.4 to 71.8)	2.75 (1.61 to 4.61)	3.34 (1.84 to 5.74)	20.7 (3.1 to 40.0)
		Pseudomonas aeruginosa	1320 (1160 to 1500)	2270 (2050 to 2520)	72.3 (54.4 to 92.3)	49.8 (42.9 to 55.3)	69.4 (55.2 to 78.6)	39.4 (20.6 to 56.2)
		Respiratory syncytial virus	419 (364 to 481)	5.77 (1.56 to 13.3)	-98.6 (-99.6 to -96.9)	7.78 (6.62 to 8.81)	0.08 (0.02 to 0.18)	-99.0 (-99.7 to -97.6)
		Staphylococcus aureus	1930 (1710 to 2170)	4240 (3830 to 4690)	120.3 (100.1 to 141.7)	149 (128 to 163)	265 (212 to 300)	78.3 (58.8 to 98.8)
		Streptococcus pneumoniae	6430 (5720 to 7220)	3690 (3320 to 4090)	-42.5 (-48.1 to -36.8)	206 (178 to 227)	96.1 (77 to 109)	-53.5 (-58.7 to -48.3)
		Other viruses	3540 (3140 to 3970)	4360 (3930 to 4850)	23.4 (11.6 to 36.3)	44.7 (38.4 to 48.9)	44.6 (35.8 to 50.5)	-0.1 (-10.6 to 11.3)
		Other bacterial pathogens	1910 (1640 to 2250)	1940 (1650 to 2250)	1.5 (-9.4 to 14.1)	59.3 (49.4 to 68.9)	48.8 (37.4 to 58)	-17.7 (-28.1 to -7.1)
High-income Asia Pacific	All Ages	*Acinetobacter baumannii*	14300 (12500 to 16400)	7950 (6910 to 9080)	-44.5 (-48.3 to -40.2)	971 (830 to 1120)	783 (605 to 919)	-19.5 (-30.1 to -11.2)
		Chlamydia spp.	68900 (63100 to 75300)	49900 (46000 to 54400)	-27.5 (-32.8 to -22.4)	1040 (918 to 1130)	1170 (947 to 1320)	13.2 (-0.3 to 24.6)
		Enterobacter spp.	11200 (9830 to 12600)	18200 (16300 to 20200)	63.0 (50.3 to 75.7)	725 (626 to 822)	1680 (1310 to 1940)	131.4 (101.2 to 155.3)
		Escherichia coli	30500 (27900 to 33600)	45500 (42300 to 49200)	49.4 (40.1 to 58.3)	2200 (1950 to 2410)	4530 (3590 to 5100)	105.5 (80.0 to 124.1)
		Fungus	20500 (18300 to 23000)	22300 (20300 to 24500)	8.9 (0.2 to 17.8)	1090 (957 to 1210)	1820 (1450 to 2100)	67.7 (46.8 to 84.2)
		Group B Streptococcus	26200 (23800 to 29000)	33100 (30600 to 35900)	26.8 (17.3 to 36.5)	1080 (944 to 1200)	2110 (1690 to 2380)	94.3 (70.2 to 114.0)
		Haemophilus influenzae	119000 (110000 to 128000)	117000 (109000 to 125000)	-1.6 (-7.6 to 4.3)	2190 (1970 to 2310)	2470 (2000 to 2770)	12.8 (-0.2 to 22.4)

		Influenza	163000 (153000 to 174000)	462 (47.6 to 2320)	-99.7 (-100.0 to -98.6)	7790 (6980 to 8260)	33.1 (2.98 to 175)	-99.6 (-100.0 to -97.8)
		Klebsiella pneumoniae	69800 (65500 to 74700)	67300 (63600 to 71900)	-3.5 (-8.8 to 1.8)	4120 (3720 to 4360)	6300 (5030 to 7010)	52.5 (34.9 to 64.6)
		Legionella spp.	64500 (59000 to 70400)	94500 (88400 to 101000)	46.7 (37.0 to 56.3)	2770 (2480 to 3030)	5760 (4650 to 6440)	107.9 (83.3 to 127.9)
		Mycoplasma spp.	140000 (128000 to 153000)	109000 (102000 to 117000)	-21.8 (-25.9 to -17.3)	1460 (1310 to 1560)	1970 (1610 to 2200)	34.9 (18.6 to 45.6)
		Polymicrobial	3690 (2410 to 5670)	3850 (2230 to 6540)	3.3 (-12.7 to 19.2)	236 (146 to 383)	372 (203 to 651)	55.9 (31.7 to 78.2)
		Pseudomonas aeruginosa	85900 (79800 to 92700)	136000 (127000 to 146000)	58.1 (50.2 to 66.1)	3980 (3560 to 4270)	8750 (7010 to 9770)	119.8 (93.6 to 138.4)
		Respiratory syncytial virus	101000 (91300 to 113000)	160 (18.9 to 761)	-99.8 (-100.0 to -99.2)	757 (683 to 818)	2.36 (0.22 to 12.4)	-99.7 (-100.0 to -98.4)
		Staphylococcus aureus	116000 (109000 to 124000)	210000 (198000 to 224000)	80.7 (72.5 to 88.6)	11600 (10400 to 12300)	29300 (23600 to 32700)	151.7 (123.5 to 170.9)
		Streptococcus pneumoniae	680000 (635000 to 730000)	347000 (328000 to 369000)	-48.9 (-51.6 to -46.4)	17500 (15800 to 18300)	16600 (13400 to 18500)	-5.0 (-15.7 to 2.5)
		Other viruses	429000 (400000 to 465000)	379000 (358000 to 403000)	-11.7 (-16.5 to -7.0)	3790 (3420 to 3990)	6580 (5330 to 7310)	73.2 (53.0 to 86.4)
		Other bacterial pathogens	106000 (94200 to 119000)	114000 (100000 to 130000)	7.4 (1.3 to 13.9)	4640 (4010 to 5270)	6610 (5100 to 7860)	42.2 (25.0 to 54.6)
	Under 5	*Acinetobacter baumannii*	682 (471 to 978)	102 (65.8 to 155)	-85.1 (-87.2 to -82.8)	11 (6.4 to 18.6)	0.51 (0.27 to 0.88)	-95.3 (-96.4 to -93.9)
		Chlamydia spp.	8670 (7360 to 10100)	1940 (1610 to 2320)	-77.6 (-80.5 to -74.3)	15.5 (12.9 to 18.5)	0.92 (0.81 to 1.04)	-94.0 (-95.0 to -92.8)
		Enterobacter spp.	1130 (863 to 1450)	567 (426 to 742)	-49.7 (-57.3 to -41.2)	8.09 (5.66 to 11.6)	1.01 (0.77 to 1.32)	-87.3 (-90.1 to -84.1)
		Escherichia coli	3470 (2930 to 4130)	1720 (1410 to 2070)	-50.4 (-57.8 to -42.9)	23.9 (19.8 to 28.9)	2.85 (2.49 to 3.25)	-88.0 (-90.0 to -85.6)
		Fungus	652 (456 to 903)	252 (179 to 350)	-61.1 (-67.7 to -54.1)	2.49 (1.76 to 3.37)	0.32 (0.23 to 0.43)	-87.0 (-89.6 to -84.1)
		Group B Streptococcus	3210 (2660 to 3840)	1360 (1110 to 1670)	-57.4 (-63.3 to -51.3)	19.9 (16.5 to 23.9)	1.92 (1.66 to 2.18)	-90.3 (-92.0 to -88.4)
		Haemophilus influenzae	28600 (24200 to 33800)	9920 (8240 to 12000)	-65.3 (-70.2 to -60.4)	53.4 (45.9 to 62.4)	3.38 (2.99 to 3.82)	-93.6 (-94.7 to -92.4)
		Influenza	23300 (20000 to 26900)	16.9 (2.05 to 76.8)	-99.9 (-100.0 to -99.7)	80.2 (69.3 to 92.7)	0.02 (0 to 0.08)	-100.0 (-100.0 to -99.9)
		Klebsiella pneumoniae	12500 (10700 to 14600)	2840 (2360 to 3430)	-77.3 (-80.1 to -74.2)	62.1 (53.2 to 71.8)	4.36 (3.89 to 4.86)	-92.9 (-94.0 to -91.6)
		Legionella spp.	1560 (1120 to 2130)	786 (546 to 1090)	-49.5 (-56.4 to -41.9)	9.92 (7.3 to 13.2)	1.17 (0.89 to 1.5)	-88.1 (-90.6 to -85.4)
		Mycoplasma spp.	15900 (13600 to 18700)	9390 (7800 to 11400)	-40.8 (-48.5 to -32.7)	20.1 (17 to 23.7)	3.32 (2.95 to 3.72)	-83.4 (-86.1 to -80.3)
		Polymicrobial	652 (380 to 1040)	186 (104 to 303)	-71.6 (-75.0 to -67.6)	12.8 (7.01 to 21.2)	1.09 (0.54 to 1.9)	-91.5 (-93.5 to -89.2)
		Pseudomonas aeruginosa	8850 (7450 to 10400)	6340 (5270 to 7650)	-28.3 (-37.8 to -17.9)	35.3 (30.5 to 40.7)	6.54 (5.85 to 7.26)	-81.4 (-84.3 to -78.2)

		Respiratory syncytial virus	67600 (58900 to 77000)	79.2 (9.67 to 368)	-99.9 (-100.0 to -99.5)	117 (104 to 132)	0.04 (0.01 to 0.19)	-100.0 (-100.0 to -99.8)
		Staphylococcus aureus	10000 (8590 to 11600)	5470 (4530 to 6560)	-45.4 (-50.7 to -39.9)	66.9 (58.5 to 76.4)	10.9 (9.76 to 12.1)	-83.6 (-85.9 to -81.0)
		Streptococcus pneumoniae	149000 (129000 to 171000)	19200 (16000 to 23000)	-87.1 (-88.4 to -85.9)	354 (308 to 409)	14.7 (13.2 to 16.4)	-95.8 (-96.4 to -95.2)
		Other viruses	136000 (117000 to 156000)	41700 (34700 to 49700)	-69.2 (-72.1 to -66.5)	129 (115 to 146)	13.2 (11.9 to 14.6)	-89.7 (-91.0 to -88.2)
		Other bacterial pathogens	3750 (2930 to 4670)	1190 (908 to 1530)	-68.3 (-73.2 to -63.4)	12.7 (9.52 to 16.7)	1.11 (0.87 to 1.4)	-91.2 (-92.8 to -89.2)
	5-14 years	*Acinetobacter baumannii*	1760 (1340 to 2260)	279 (213 to 367)	-84.2 (-85.8 to -82.3)	5.33 (4.11 to 6.61)	0.22 (0.19 to 0.26)	-95.8 (-96.6 to -94.7)
		Chlamydia spp.	9150 (7020 to 11800)	2350 (1800 to 3010)	-74.3 (-76.5 to -71.8)	6.54 (5.27 to 7.81)	0.47 (0.43 to 0.52)	-92.8 (-94.1 to -91.1)
		Enterobacter spp.	1030 (771 to 1350)	632 (473 to 822)	-38.7 (-45.4 to -31.7)	2.62 (2.12 to 3.17)	0.48 (0.42 to 0.55)	-81.5 (-84.7 to -77.6)
		Escherichia coli	2430 (1860 to 3130)	1220 (936 to 1540)	-49.8 (-54.4 to -45.0)	6.6 (5.43 to 7.88)	0.96 (0.88 to 1.05)	-85.3 (-87.8 to -82.2)
		Fungus	2920 (2260 to 3770)	1290 (979 to 1660)	-55.9 (-60.2 to -51.3)	7.83 (6.37 to 9.46)	1 (0.9 to 1.11)	-87.1 (-89.3 to -84.1)
		Group B Streptococcus	3190 (2410 to 4060)	1630 (1240 to 2080)	-48.9 (-53.8 to -43.5)	6.05 (4.96 to 7.2)	0.91 (0.82 to 1.01)	-84.8 (-87.3 to -81.9)
		Haemophilus influenzae	13500 (10500 to 17300)	6240 (4800 to 7970)	-53.9 (-57.5 to -50.3)	13.6 (11.5 to 15.8)	1.25 (1.16 to 1.35)	-90.7 (-92.1 to -88.9)
		Influenza	18800 (14700 to 23800)	18.2 (2.33 to 83.2)	-99.9 (-100.0 to -99.5)	37.2 (31.4 to 43)	0.01 (0 to 0.05)	-100.0 (-100.0 to -99.9)
		Klebsiella pneumoniae	7110 (5610 to 9030)	2630 (2050 to 3310)	-63.0 (-66.0 to -60.1)	19.2 (16.1 to 22.4)	2.05 (1.9 to 2.22)	-89.3 (-91.0 to -87.0)
		Legionella spp.	7680 (5980 to 9880)	4810 (3710 to 6110)	-37.3 (-42.2 to -32.0)	13.2 (11.3 to 15.5)	2.59 (2.41 to 2.79)	-80.3 (-83.3 to -77.1)
		Mycoplasma spp.	29400 (22800 to 37400)	11700 (9000 to 14900)	-60.3 (-63.3 to -57.2)	20.6 (17.3 to 23.8)	2.42 (2.24 to 2.62)	-88.2 (-90.0 to -85.9)
		Polymicrobial	372 (225 to 575)	134 (73.7 to 222)	-64.2 (-69.2 to -58.7)	1.06 (0.67 to 1.71)	0.11 (0.06 to 0.18)	-89.9 (-92.0 to -87.3)
		Pseudomonas aeruginosa	8140 (6250 to 10500)	5190 (4040 to 6600)	-36.1 (-40.6 to -31.2)	14.3 (12.2 to 16.6)	2.82 (2.63 to 3.04)	-80.2 (-83.0 to -76.6)
		Respiratory syncytial virus	6100 (4730 to 7840)	6.21 (0.81 to 28.8)	-99.9 (-100.0 to -99.5)	5.19 (4.45 to 5.95)	0 (0 to 0.01)	-100.0 (-100.0 to -99.9)
		Staphylococcus aureus	11600 (9050 to 14700)	7590 (5900 to 9500)	-34.6 (-39.2 to -30.2)	43 (36.8 to 49.6)	8.48 (7.99 to 9.05)	-80.2 (-83.1 to -76.5)
		Streptococcus pneumoniae	104000 (81100 to 133000)	26200 (20200 to 33000)	-74.8 (-76.6 to -73.1)	163 (139 to 188)	11.9 (11.2 to 12.8)	-92.6 (-93.8 to -91.1)
		Other viruses	57300 (44600 to 73000)	29700 (23100 to 37400)	-48.1 (-51.4 to -44.8)	33.4 (29 to 37.9)	5.38 (5.05 to 5.76)	-83.8 (-86.0 to -80.9)
		Other bacterial pathogens	10400 (8030 to 13300)	3520 (2660 to 4550)	-66.0 (-68.8 to -62.9)	15.9 (13 to 19)	1.56 (1.34 to 1.77)	-90.1 (-91.8 to -88.0)
	15-49 years	*Acinetobacter baumannii*	2160 (1750 to 2590)	464 (376 to 554)	-78.5 (-80.1 to -76.8)	19 (16.2 to 22)	3.14 (2.68 to 3.63)	-83.5 (-85.1 to -81.7)

		Chlamydia spp.	11800 (10100 to 13700)	3900 (3360 to 4450)	-67.0 (-69.2 to -64.8)	25.8 (23.4 to 28.6)	6.53 (5.94 to 7.16)	-74.7 (-76.8 to -72.2)
		Enterobacter spp.	1420 (1180 to 1690)	1040 (871 to 1240)	-26.6 (-32.5 to -20.5)	11.8 (10.3 to 13.3)	6.68 (5.87 to 7.59)	-43.1 (-48.6 to -37.6)
		Escherichia coli	3250 (2770 to 3740)	2010 (1740 to 2310)	-38.3 (-42.2 to -34.3)	27.9 (25.4 to 30.6)	13.3 (12.3 to 14.5)	-52.2 (-56.0 to -48.0)
		Fungus	3910 (3300 to 4530)	2120 (1820 to 2460)	-45.6 (-49.5 to -41.3)	33.1 (29.5 to 37)	13.9 (12.6 to 15.4)	-57.9 (-61.8 to -53.7)
		Group B Streptococcus	4330 (3700 to 5120)	2680 (2310 to 3120)	-37.9 (-42.5 to -33.2)	26.4 (23.6 to 29.8)	12.6 (11.5 to 14)	-51.9 (-56.1 to -47.4)
		Haemophilus influenzae	18700 (16100 to 21300)	10300 (8950 to 11800)	-44.7 (-47.4 to -41.9)	61 (57.2 to 64.7)	17.4 (16.4 to 18.6)	-71.4 (-73.4 to -69.1)
		Influenza	25700 (22400 to 29300)	30.5 (4.29 to 135)	-99.9 (-100.0 to -99.5)	165 (156 to 173)	0.15 (0.02 to 0.67)	-99.9 (-100.0 to -99.6)
		Klebsiella pneumoniae	9510 (8250 to 10900)	4340 (3780 to 4940)	-54.3 (-56.5 to -52.3)	81.3 (76.2 to 86)	28.6 (26.9 to 30.5)	-64.8 (-67.2 to -62.2)
		Legionella spp.	11000 (9360 to 12600)	7930 (6890 to 9080)	-27.6 (-32.2 to -22.7)	64.1 (58.8 to 70)	36 (33.5 to 38.6)	-43.8 (-47.9 to -39.1)
		Mycoplasma spp.	40300 (34900 to 46000)	19300 (16700 to 22000)	-52.1 (-54.5 to -49.5)	91.2 (85.2 to 96.9)	33.7 (31.6 to 36.1)	-63.0 (-65.7 to -60.1)
		Polymicrobial	481 (291 to 759)	222 (121 to 372)	-54.2 (-59.3 to -48.3)	4.2 (2.59 to 6.67)	1.5 (0.86 to 2.53)	-64.6 (-69.2 to -59.9)
		Pseudomonas aeruginosa	11500 (9870 to 13200)	8560 (7430 to 9800)	-25.6 (-28.9 to -22.1)	68 (63.4 to 72.7)	39.2 (36.7 to 41.9)	-42.2 (-45.8 to -37.9)
		Respiratory syncytial virus	8760 (7560 to 10000)	10.4 (1.5 to 46.3)	-99.9 (-100.0 to -99.5)	25.5 (23.6 to 27.3)	0.02 (0 to 0.1)	-99.9 (-100.0 to -99.6)
		Staphylococcus aureus	16100 (14000 to 18300)	12500 (10900 to 14200)	-22.1 (-25.3 to -18.5)	195 (185 to 203)	118 (112 to 124)	-39.5 (-43.1 to -35.3)
		Streptococcus pneumoniae	141000 (123000 to 160000)	43400 (37800 to 49600)	-69.3 (-70.4 to -68.0)	713 (679 to 743)	168 (159 to 178)	-76.5 (-78.0 to -74.7)
		Other viruses	81800 (71000 to 92400)	49100 (42900 to 55900)	-39.9 (-42.3 to -37.5)	162 (155 to 168)	75 (71.3 to 79.4)	-53.5 (-56.2 to -50.3)
		Other bacterial pathogens	13800 (11500 to 16200)	5830 (4830 to 6880)	-57.8 (-60.1 to -55.2)	66.6 (59.3 to 74.4)	21.7 (19 to 24.9)	-67.4 (-69.9 to -64.5)
	50-69 years	*Acinetobacter baumannii*	3850 (3280 to 4440)	1390 (1190 to 1600)	-63.9 (-66.2 to -61.5)	136 (118 to 156)	46.3 (40.4 to 52.7)	-65.9 (-69.0 to -62.7)
		Chlamydia spp.	13600 (12100 to 15300)	6920 (6190 to 7690)	-49.2 (-52.2 to -46.0)	120 (109 to 131)	57.7 (52.4 to 63.2)	-51.7 (-55.9 to -47.4)
		Enterobacter spp.	2950 (2550 to 3360)	3050 (2680 to 3430)	3.7 (-4.0 to 11.4)	98.3 (87.5 to 111)	98 (87.3 to 109)	-0.1 (-9.2 to 9.1)
		Escherichia coli	7180 (6420 to 8030)	6410 (5840 to 7010)	-10.6 (-15.7 to -5.5)	248 (227 to 267)	213 (197 to 230)	-14.0 (-20.6 to -7.0)
		Fungus	6850 (6020 to 7820)	5470 (4850 to 6140)	-20.0 (-25.5 to -14.1)	233 (211 to 258)	179 (161 to 197)	-23.3 (-29.9 to -16.7)
		Group B Streptococcus	6230 (5460 to 7080)	5530 (4980 to 6130)	-11.1 (-17.4 to -4.6)	153 (139 to 170)	130 (119 to 144)	-14.6 (-22.2 to -6.8)
		Haemophilus influenzae	23400 (21200 to 25600)	18500 (16700 to 20100)	-21.1 (-25.1 to -17.2)	308 (292 to 324)	156 (145 to 167)	-49.4 (-53.0 to -45.6)

		Influenza	27600 (25200 to 30100)	49.1 (6.71 to 216)	-99.8 (-100.0 to -99.2)	710 (673 to 742)	1.24 (0.15 to 5.57)	-99.8 (-100.0 to -99.2)
		Klebsiella pneumoniae	14700 (13400 to 16000)	10100 (9230 to 11000)	-31.2 (-34.4 to -28.0)	504 (479 to 528)	331 (310 to 354)	-34.2 (-38.6 to -29.6)
		Legionella spp.	20300 (18200 to 22600)	19700 (17900 to 21500)	-2.7 (-8.7 to 3.3)	478 (441 to 520)	447 (415 to 480)	-6.5 (-13.6 to 1.3)
		Mycoplasma spp.	23000 (20600 to 25300)	15000 (13600 to 16500)	-34.5 (-38.0 to -31.0)	209 (195 to 224)	131 (121 to 141)	-37.5 (-42.1 to -32.6)
		Polymicrobial	780 (477 to 1260)	559 (319 to 944)	-28.7 (-35.4 to -21.4)	27.3 (16.9 to 44.1)	18.8 (10.7 to 31.7)	-31.7 (-39.9 to -23.5)
		Pseudomonas aeruginosa	21200 (19100 to 23500)	21000 (19000 to 23000)	-0.7 (-5.5 to 4.6)	505 (474 to 536)	482 (450 to 513)	-4.6 (-10.9 to 2.3)
		Respiratory syncytial virus	7400 (6560 to 8220)	11.9 (1.7 to 52)	-99.8 (-100.0 to -99.3)	86.8 (79.2 to 95.4)	0.14 (0.02 to 0.61)	-99.8 (-100.0 to -99.3)
		Staphylococcus aureus	25300 (23000 to 27700)	27800 (25300 to 30000)	9.8 (5.1 to 14.6)	1230 (1180 to 1280)	1300 (1230 to 1380)	5.9 (-0.6 to 12.7)
		Streptococcus pneumoniae	107000 (98200 to 117000)	48900 (44600 to 52800)	-54.4 (-56.1 to -52.5)	2180 (2090 to 2260)	940 (876 to 1000)	-56.8 (-59.6 to -53.8)
		Other viruses	57900 (53100 to 63000)	48300 (44200 to 52200)	-16.6 (-20.0 to -13.1)	461 (441 to 479)	369 (345 to 393)	-20.1 (-24.9 to -14.7)
		Other bacterial pathogens	24900 (21500 to 28500)	15500 (13300 to 17700)	-37.9 (-41.2 to -34.2)	484 (432 to 542)	287 (249 to 330)	-40.7 (-45.1 to -36.0)
	70+ years	*Acinetobacter baumannii*	5870 (4980 to 6870)	5720 (4940 to 6620)	-2.5 (-9.3 to 4.6)	800 (675 to 929)	733 (558 to 865)	-8.5 (-20.1 to 0.8)
		Chlamydia spp.	25600 (22900 to 28800)	34800 (31500 to 38700)	35.9 (26.7 to 45.9)	869 (760 to 955)	1110 (881 to 1260)	27.4 (12.9 to 40.1)
		Enterobacter spp.	4660 (4010 to 5380)	12900 (11400 to 14500)	177.8 (155.0 to 200.9)	604 (513 to 690)	1570 (1220 to 1830)	160.1 (127.0 to 187.0)
		Escherichia coli	14100 (12700 to 15900)	34100 (31300 to 37300)	141.7 (126.4 to 157.0)	1890 (1650 to 2080)	4300 (3380 to 4850)	126.5 (98.3 to 146.7)
		Fungus	6130 (5370 to 7010)	13100 (11700 to 14700)	114.6 (97.9 to 131.2)	809 (699 to 916)	1630 (1260 to 1900)	101.0 (75.8 to 121.4)
		Group B Streptococcus	9230 (8180 to 10300)	21900 (19900 to 24100)	138.1 (119.8 to 156.0)	878 (751 to 978)	1960 (1550 to 2220)	123.0 (96.0 to 145.4)
		Haemophilus influenzae	34300 (31400 to 37600)	71600 (65900 to 78000)	109.2 (97.9 to 120.2)	1750 (1540 to 1870)	2290 (1830 to 2580)	30.8 (15.8 to 41.9)
		Influenza	67900 (62500 to 74000)	347 (31.8 to 1810)	-99.5 (-100.0 to -97.3)	6790 (6010 to 7250)	31.6 (2.81 to 168)	-99.5 (-100.0 to -97.6)
		Klebsiella pneumoniae	25900 (23900 to 28400)	47400 (43900 to 51400)	82.9 (73.8 to 92.4)	3460 (3060 to 3670)	5930 (4680 to 6640)	71.3 (51.9 to 84.6)
		Legionella spp.	24000 (21400 to 26900)	61300 (56300 to 66800)	155.7 (138.0 to 174.1)	2200 (1940 to 2430)	5280 (4190 to 5940)	139.2 (111.3 to 162.7)
		Mycoplasma spp.	31500 (28500 to 35000)	54100 (49600 to 59000)	71.9 (62.2 to 81.7)	1110 (981 to 1210)	1800 (1430 to 2030)	60.9 (42.7 to 73.8)
		Polymicrobial	1410 (835 to 2350)	2750 (1510 to 4740)	94.3 (76.7 to 113.8)	191 (114 to 317)	351 (191 to 615)	82.3 (58.1 to 104.5)
		Pseudomonas aeruginosa	36200 (33100 to 40200)	94700 (87600 to 103000)	161.6 (147.2 to 176.3)	3360 (2950 to 3620)	8220 (6490 to 9220)	144.7 (115.9 to 165.0)

		Respiratory syncytial virus	11400 (10200 to 12900)	51.8 (4.76 to 274)	-99.5 (-100.0 to -97.6)	523 (458 to 578)	2.16 (0.19 to 11.3)	-99.6 (-100.0 to -97.9)
		Staphylococcus aureus	53200 (48900 to 58200)	157000 (145000 to 169000)	194.5 (180.5 to 208.1)	10100 (8940 to 10700)	27800 (22100 to 31200)	175.8 (145.7 to 196.2)
		Streptococcus pneumoniae	178000 (165000 to 195000)	209000 (194000 to 226000)	17.5 (12.1 to 22.8)	14000 (12500 to 14900)	15500 (12300 to 17300)	9.9 (-2.0 to 18.4)
		Other viruses	96900 (89500 to 106000)	210000 (195000 to 227000)	117.1 (106.2 to 127.4)	3010 (2660 to 3190)	6110 (4890 to 6840)	103.1 (81.5 to 118.5)
		Other bacterial pathogens	53400 (46800 to 61300)	88100 (76900 to 101000)	65.0 (55.6 to 75.0)	4060 (3480 to 4650)	6300 (4830 to 7510)	54.8 (35.9 to 68.3)
Brunei Darussalam	All Ages	*Acinetobacter baumannii*	27.2 (23.5 to 31.3)	41.5 (36.3 to 47.3)	53.2 (37.5 to 70.3)	1.25 (1.05 to 1.46)	1.79 (1.45 to 2.16)	44.4 (18.1 to 73.9)
		Chlamydia spp.	131 (117 to 146)	120 (110 to 131)	-7.9 (-16.5 to 1.1)	0.98 (0.85 to 1.14)	0.96 (0.82 to 1.12)	-1.1 (-18.0 to 17.8)
		Enterobacter spp.	12.5 (10.5 to 14.6)	25.1 (22.2 to 28.4)	101.8 (81.3 to 123.5)	0.42 (0.34 to 0.51)	0.9 (0.74 to 1.06)	114.4 (76.9 to 154.1)
		Escherichia coli	39.1 (34.6 to 43.7)	60.5 (55.8 to 66.2)	55.3 (40.1 to 70.9)	1.49 (1.28 to 1.75)	2.46 (2.08 to 2.88)	65.3 (36.0 to 98.3)
		Fungus	26.8 (22.8 to 31.1)	48.1 (42.9 to 54)	79.8 (61.2 to 99.3)	0.84 (0.69 to 0.99)	1.47 (1.23 to 1.74)	76.4 (44.5 to 113.5)
		Group B Streptococcus	37.3 (32.9 to 42.4)	63 (57.2 to 69.3)	69.4 (52.6 to 86.8)	0.76 (0.66 to 0.88)	1.38 (1.16 to 1.6)	80.6 (49.6 to 112.8)
		Haemophilus influenzae	201 (179 to 225)	161 (148 to 178)	-19.5 (-26.5 to -11.9)	1.49 (1.3 to 1.69)	1.05 (0.9 to 1.23)	-29.1 (-40.1 to -17.6)
		Influenza	229 (208 to 252)	0.49 (0.09 to 2.18)	-99.8 (-100.0 to -99.0)	4.7 (4.12 to 5.36)	0.01 (0 to 0.05)	-99.8 (-100.0 to -98.8)
		Klebsiella pneumoniae	116 (105 to 130)	142 (131 to 153)	22.3 (12.4 to 32.2)	3.05 (2.68 to 3.49)	4.84 (4.1 to 5.63)	59.3 (33.5 to 85.4)
		Legionella spp.	50.5 (44.6 to 57.2)	95.9 (88 to 105)	90.4 (70.3 to 110.4)	1.23 (1.05 to 1.45)	2.24 (1.9 to 2.64)	82.6 (50.3 to 118.2)
		Mycoplasma spp.	243 (215 to 272)	275 (250 to 300)	13.3 (3.9 to 23.2)	1.15 (1.01 to 1.31)	1.33 (1.14 to 1.54)	15.7 (-3.6 to 35.2)
		Polymicrobial	6.25 (4.44 to 8.84)	8.96 (6.04 to 13.2)	43.0 (24.4 to 61.8)	0.24 (0.17 to 0.36)	0.35 (0.22 to 0.53)	42.5 (14.0 to 70.8)
		Pseudomonas aeruginosa	83 (74.7 to 92.4)	179 (167 to 194)	116.1 (96.3 to 135.7)	1.85 (1.62 to 2.13)	4.29 (3.66 to 4.97)	132.8 (92.9 to 175.2)
		Respiratory syncytial virus	232 (205 to 265)	0.41 (0.07 to 1.89)	-99.8 (-100.0 to -99.2)	0.73 (0.63 to 0.85)	0 (0 to 0.01)	-99.8 (-100.0 to -99.1)
		Staphylococcus aureus	125 (114 to 137)	265 (247 to 285)	112.3 (97.6 to 126.2)	6.18 (5.39 to 7.1)	14.2 (12.1 to 16.5)	130.1 (92.3 to 171.9)
		Streptococcus pneumoniae	1300 (1180 to 1440)	1010 (939 to 1100)	-22.0 (-26.8 to -16.9)	13.4 (11.9 to 15.1)	13.1 (11.2 to 15)	-2.3 (-17.1 to 12.5)
		Other viruses	711 (638 to 792)	740 (683 to 805)	4.1 (-2.6 to 11.3)	2.26 (2.01 to 2.55)	3.25 (2.79 to 3.71)	43.8 (22.3 to 66.7)
		Other bacterial pathogens	118 (104 to 132)	155 (138 to 173)	31.7 (20.4 to 44.4)	3.12 (2.66 to 3.69)	3.93 (3.28 to 4.65)	26.5 (2.9 to 50.4)
	Under 5	*Acinetobacter baumannii*	3.51 (2.48 to 4.91)	2.15 (1.51 to 3.02)	-38.6 (-45.6 to -31.2)	0.06 (0.03 to 0.09)	0.03 (0.01 to 0.05)	-49.8 (-68.3 to -25.4)
		Chlamydia spp.	42.9 (36.3 to 50.7)	21.9 (19 to 25.4)	-48.9 (-55.0 to -42.0)	0.08 (0.07 to 0.11)	0.04 (0.03 to 0.05)	-58.2 (-70.8 to -42.3)

		Enterobacter spp.	4.16 (3.17 to 5.37)	3.92 (3.06 to 4.97)	-5.4 (-17.7 to 7.5)	0.03 (0.02 to 0.05)	0.02 (0.01 to 0.04)	-31.8 (-52.5 to -3.5)
		Escherichia coli	14.7 (12.2 to 17.8)	11.3 (9.65 to 13.4)	-22.5 (-32.5 to -12.4)	0.12 (0.09 to 0.15)	0.07 (0.05 to 0.09)	-42.3 (-59.5 to -21.0)
		Fungus	2.83 (1.97 to 3.95)	2.12 (1.49 to 2.86)	-24.8 (-35.3 to -13.3)	0.01 (0.01 to 0.02)	0.01 (0 to 0.01)	-46.0 (-64.3 to -19.8)
		Group B Streptococcus	12.7 (10.5 to 15.3)	11 (9.26 to 13.1)	-13.3 (-23.8 to -2.2)	0.09 (0.07 to 0.12)	0.06 (0.04 to 0.08)	-38.5 (-58.8 to -13.9)
		Haemophilus influenzae	108 (91.1 to 127)	45.2 (38.5 to 53)	-58.0 (-63.2 to -52.3)	0.25 (0.2 to 0.31)	0.04 (0.03 to 0.06)	-83.0 (-88.3 to -75.6)
		Influenza	90.8 (77.6 to 106)	0.1 (0.02 to 0.45)	-99.9 (-100.0 to -99.5)	0.38 (0.3 to 0.48)	0 (0 to 0)	-99.9 (-100.0 to -99.7)
		Klebsiella pneumoniae	54.2 (46.2 to 64.1)	29.5 (25.4 to 34.3)	-45.5 (-51.2 to -39.1)	0.32 (0.25 to 0.4)	0.13 (0.1 to 0.18)	-57.6 (-69.5 to -40.6)
		Legionella spp.	4.8 (3.4 to 6.59)	3.91 (2.88 to 5.3)	-18.3 (-27.9 to -7.5)	0.04 (0.03 to 0.05)	0.03 (0.02 to 0.04)	-33.9 (-56.3 to -4.5)
		Mycoplasma spp.	62.1 (52.2 to 73.1)	43.8 (37.6 to 51.3)	-29.3 (-37.5 to -20.2)	0.1 (0.08 to 0.12)	0.05 (0.04 to 0.07)	-49.3 (-64.3 to -27.1)
		Polymicrobial	2.32 (1.44 to 3.62)	1.86 (1.11 to 2.92)	-19.8 (-29.2 to -9.2)	0.05 (0.03 to 0.09)	0.03 (0.02 to 0.06)	-31.7 (-59.7 to 4.9)
		Pseudomonas aeruginosa	28.8 (24.3 to 34.3)	31.3 (27 to 36.5)	9.0 (-4.6 to 22.6)	0.14 (0.11 to 0.18)	0.11 (0.08 to 0.14)	-25.2 (-47.0 to 2.9)
		Respiratory syncytial virus	202 (175 to 234)	0.31 (0.05 to 1.41)	-99.8 (-100.0 to -99.3)	0.45 (0.36 to 0.55)	0 (0 to 0)	-99.9 (-100.0 to -99.5)
		Staphylococcus aureus	36.9 (31.7 to 43.2)	36.4 (31.5 to 42.2)	-1.3 (-10.1 to 8.0)	0.3 (0.24 to 0.38)	0.21 (0.15 to 0.28)	-29.8 (-50.3 to -0.3)
		Streptococcus pneumoniae	599 (516 to 699)	259 (225 to 300)	-56.7 (-60.4 to -52.9)	1.75 (1.37 to 2.18)	0.56 (0.4 to 0.78)	-67.6 (-77.3 to -53.4)
		Other viruses	421 (360 to 492)	253 (220 to 293)	-39.7 (-44.7 to -34.4)	0.51 (0.4 to 0.63)	0.23 (0.16 to 0.32)	-54.4 (-68.0 to -34.6)
		Other bacterial pathogens	16.5 (13 to 20.5)	9.09 (7.16 to 11.2)	-44.9 (-51.8 to -37.7)	0.06 (0.05 to 0.09)	0.03 (0.02 to 0.04)	-55.7 (-69.1 to -39.0)
	5-14 years	*Acinetobacter baumannii*	7.67 (5.84 to 9.79)	6.41 (5 to 8.04)	-16.3 (-26.0 to -4.7)	0.03 (0.02 to 0.03)	0.01 (0.01 to 0.01)	-71.7 (-80.1 to -62.6)
		Chlamydia spp.	32.5 (25 to 41.2)	20.3 (15.8 to 25.3)	-37.1 (-44.7 to -28.5)	0.03 (0.02 to 0.03)	0.01 (0 to 0.01)	-78.8 (-84.9 to -72.2)
		Enterobacter spp.	2.74 (2.07 to 3.57)	3.56 (2.75 to 4.58)	30.5 (14.4 to 48.1)	0.01 (0.01 to 0.01)	0 (0 to 0)	-55.9 (-68.5 to -41.2)
		Escherichia coli	7.38 (5.67 to 9.25)	7.42 (5.84 to 9.14)	0.9 (-11.7 to 13.6)	0.02 (0.02 to 0.03)	0.01 (0.01 to 0.01)	-65.9 (-75.5 to -55.2)
		Fungus	8.9 (6.7 to 11.4)	9.16 (7.18 to 11.5)	3.2 (-9.8 to 17.3)	0.03 (0.02 to 0.04)	0.01 (0.01 to 0.01)	-65.1 (-75.4 to -54.4)
		Group B Streptococcus	9.08 (6.89 to 11.4)	10.6 (8.27 to 13.1)	17.1 (1.8 to 34.1)	0.02 (0.02 to 0.03)	0.01 (0.01 to 0.01)	-60.5 (-71.5 to -47.9)
		Haemophilus influenzae	35.8 (28 to 44.8)	24.7 (19.4 to 30.5)	-31.0 (-38.1 to -23.2)	0.04 (0.04 to 0.06)	0.01 (0.01 to 0.01)	-84.5 (-88.8 to -79.7)
		Influenza	51.4 (39.9 to 63.7)	0.08 (0.01 to 0.34)	-99.8 (-100.0 to -99.3)	0.12 (0.1 to 0.15)	0 (0 to 0)	-99.9 (-100.0 to -99.8)

		Klebsiella pneumoniae	21.7 (16.9 to 26.9)	20 (16 to 24.5)	-7.5 (-16.8 to 2.5)	0.07 (0.06 to 0.09)	0.02 (0.02 to 0.03)	-68.8 (-77.4 to -59.5)
		Legionella spp.	16.6 (12.9 to 20.9)	17.9 (14.3 to 22)	8.2 (-5.0 to 22.2)	0.04 (0.03 to 0.05)	0.01 (0.01 to 0.02)	-63.5 (-74.1 to -52.1)
		Mycoplasma spp.	80.3 (61.4 to 99.7)	65.9 (52.2 to 80.5)	-17.7 (-27.2 to -7.6)	0.07 (0.05 to 0.09)	0.02 (0.01 to 0.02)	-72.2 (-79.9 to -63.6)
		Polymicrobial	1.32 (0.84 to 1.98)	1.24 (0.74 to 1.9)	-6.6 (-19.2 to 6.9)	0 (0 to 0.01)	0 (0 to 0)	-68.5 (-77.6 to -58.3)
		Pseudomonas aeruginosa	18.6 (14.3 to 23.2)	26.8 (21.2 to 32.9)	44.4 (27.8 to 61.8)	0.04 (0.03 to 0.05)	0.02 (0.02 to 0.02)	-51.2 (-64.8 to -36.3)
		Respiratory syncytial virus	12.7 (9.84 to 16.1)	0.03 (0 to 0.11)	-99.8 (-100.0 to -99.1)	0.01 (0.01 to 0.02)	0 (0 to 0)	-99.9 (-100.0 to -99.7)
		Staphylococcus aureus	30.2 (23.2 to 37.3)	41.3 (32.8 to 50.2)	37.3 (24.0 to 51.6)	0.14 (0.11 to 0.17)	0.06 (0.05 to 0.08)	-53.6 (-66.6 to -39.9)
		Streptococcus pneumoniae	294 (228 to 363)	189 (150 to 229)	-35.6 (-42.0 to -29.0)	0.56 (0.44 to 0.69)	0.12 (0.1 to 0.15)	-78.3 (-84.4 to -71.8)
		Other viruses	125 (96.6 to 154)	126 (99.1 to 153)	0.9 (-9.0 to 11.6)	0.09 (0.07 to 0.12)	0.03 (0.03 to 0.04)	-65.9 (-75.6 to -56.1)
		Other bacterial pathogens	32.3 (25.2 to 40.7)	23.7 (18.2 to 29.8)	-26.5 (-34.7 to -17.4)	0.06 (0.05 to 0.07)	0.01 (0.01 to 0.02)	-75.2 (-82.2 to -67.3)
	15-49 years	*Acinetobacter baumannii*	7.54 (6.31 to 9.03)	11 (9.22 to 13)	45.7 (29.7 to 63.7)	0.12 (0.09 to 0.14)	0.11 (0.09 to 0.14)	-4.0 (-24.8 to 22.9)
		Chlamydia spp.	31.9 (27.3 to 36.8)	34.8 (30.1 to 39.8)	9.4 (-1.4 to 22.4)	0.12 (0.1 to 0.15)	0.09 (0.07 to 0.11)	-28.0 (-43.4 to -8.5)
		Enterobacter spp.	2.69 (2.2 to 3.25)	6.08 (5.11 to 7.15)	127.0 (101.0 to 155.7)	0.04 (0.03 to 0.05)	0.06 (0.05 to 0.07)	49.5 (16.8 to 88.6)
		Escherichia coli	7.25 (6.13 to 8.39)	12.7 (11 to 14.4)	75.5 (56.2 to 97.5)	0.11 (0.09 to 0.13)	0.12 (0.1 to 0.15)	15.6 (-9.1 to 46.1)
		Fungus	8.74 (7.26 to 10.4)	15.7 (13.3 to 18.2)	79.6 (59.3 to 101.6)	0.13 (0.1 to 0.16)	0.15 (0.12 to 0.19)	18.2 (-6.8 to 49.3)
		Group B Streptococcus	8.92 (7.51 to 10.6)	18.1 (15.7 to 21)	103.7 (81.6 to 129.6)	0.1 (0.08 to 0.12)	0.13 (0.1 to 0.16)	34.1 (5.1 to 68.0)
		Haemophilus influenzae	35.2 (30.3 to 40.3)	42.2 (37 to 48.2)	20.0 (8.5 to 31.7)	0.2 (0.17 to 0.24)	0.11 (0.09 to 0.13)	-47.3 (-58.0 to -33.8)
		Influenza	50.5 (44 to 57.5)	0.14 (0.02 to 0.6)	-99.7 (-100.0 to -98.8)	0.57 (0.47 to 0.67)	0 (0 to 0)	-99.8 (-100.0 to -99.2)
		Klebsiella pneumoniae	21.3 (18.5 to 24.3)	34.3 (30.1 to 38.9)	60.9 (46.4 to 76.2)	0.32 (0.26 to 0.38)	0.34 (0.28 to 0.4)	5.9 (-15.5 to 31.5)
		Legionella spp.	16.3 (13.8 to 19)	30.6 (26.8 to 34.9)	88.2 (67.0 to 111.8)	0.17 (0.14 to 0.2)	0.21 (0.17 to 0.25)	23.9 (-2.2 to 58.0)
		Mycoplasma spp.	78.8 (68.5 to 89.9)	113 (99 to 128)	43.2 (29.4 to 58.3)	0.31 (0.26 to 0.37)	0.29 (0.25 to 0.35)	-5.7 (-24.9 to 18.4)
		Polymicrobial	1.3 (0.84 to 1.95)	2.12 (1.3 to 3.28)	62.5 (41.2 to 85.2)	0.02 (0.01 to 0.03)	0.02 (0.01 to 0.03)	6.9 (-18.8 to 33.9)
		Pseudomonas aeruginosa	18.3 (15.8 to 21)	45.8 (40.2 to 52)	151.3 (125.5 to 178.2)	0.19 (0.16 to 0.23)	0.31 (0.26 to 0.38)	65.4 (30.4 to 109.3)
		Respiratory syncytial virus	12.5 (10.8 to 14.4)	0.04 (0.01 to 0.2)	-99.6 (-99.9 to -98.4)	0.06 (0.05 to 0.08)	0 (0 to 0)	-99.8 (-100.0 to -98.9)
		Staphylococcus aureus	29.6 (25.8 to 33.7)	70.7 (62.5 to 79.3)	138.8 (118.8 to 159.5)	0.63 (0.52 to 0.75)	0.99 (0.83 to 1.18)	57.2 (25.7 to 97.4)

		Streptococcus pneumoniae	289 (255 to 327)	323 (285 to 363)	12.0 (2.8 to 21.3)	2.56 (2.12 to 3.02)	1.88 (1.57 to 2.24)	-26.3 (-41.0 to -8.0)
		Other viruses	122 (107 to 139)	215 (190 to 242)	75.6 (61.4 to 91.5)	0.43 (0.35 to 0.5)	0.49 (0.41 to 0.59)	15.6 (-7.1 to 45.6)
		Other bacterial pathogens	31.8 (26.8 to 36.9)	40.6 (34.2 to 47)	27.9 (14.5 to 41.5)	0.27 (0.22 to 0.32)	0.22 (0.18 to 0.27)	-15.7 (-33.5 to 6.0)
	50-69 years	*Acinetobacter baumannii*	3.86 (3.26 to 4.58)	12 (10.2 to 14.1)	212.1 (167.8 to 257.0)	0.25 (0.2 to 0.32)	0.45 (0.35 to 0.56)	81.9 (36.9 to 133.9)
		Chlamydia spp.	9.66 (8.32 to 11.2)	21.6 (19 to 24.8)	124.2 (93.1 to 160.1)	0.16 (0.12 to 0.2)	0.2 (0.16 to 0.25)	30.7 (-0.3 to 67.1)
		Enterobacter spp.	1.34 (1.1 to 1.61)	6.36 (5.47 to 7.43)	377.4 (312.8 to 454.5)	0.08 (0.06 to 0.11)	0.23 (0.18 to 0.28)	178.3 (108.9 to 261.6)
		Escherichia coli	3.98 (3.42 to 4.6)	14.4 (12.7 to 16.5)	263.0 (213.8 to 315.8)	0.26 (0.2 to 0.32)	0.53 (0.42 to 0.66)	111.6 (59.1 to 175.4)
		Fungus	3.82 (3.22 to 4.51)	14.4 (12.5 to 16.7)	279.5 (230.2 to 337.2)	0.24 (0.19 to 0.3)	0.53 (0.41 to 0.66)	121.1 (68.2 to 185.7)
		Group B Streptococcus	3.14 (2.66 to 3.68)	13.2 (11.5 to 15.2)	322.1 (262.8 to 381.4)	0.14 (0.11 to 0.18)	0.35 (0.28 to 0.42)	146.0 (86.3 to 217.6)
		Haemophilus influenzae	10.6 (9.17 to 12.1)	28.2 (25 to 32.1)	167.7 (134.6 to 205.6)	0.26 (0.2 to 0.32)	0.27 (0.21 to 0.33)	4.1 (-19.8 to 35.0)
		Influenza	13.1 (11.5 to 14.9)	0.08 (0.01 to 0.34)	-99.4 (-99.9 to -97.4)	0.62 (0.5 to 0.77)	0 (0 to 0.01)	-99.7 (-100.0 to -98.5)
		Klebsiella pneumoniae	8.2 (7.23 to 9.29)	30.3 (26.8 to 34.2)	270.2 (226.3 to 320.7)	0.52 (0.42 to 0.64)	1.12 (0.88 to 1.36)	115.8 (63.8 to 175.8)
		Legionella spp.	6.89 (5.93 to 8.05)	27.2 (24 to 30.9)	296.2 (241.2 to 357.8)	0.3 (0.24 to 0.38)	0.69 (0.55 to 0.84)	130.9 (74.9 to 204.4)
		Mycoplasma spp.	10.8 (9.57 to 12.4)	30.7 (27 to 34.4)	183.8 (146.9 to 222.8)	0.18 (0.14 to 0.23)	0.3 (0.24 to 0.37)	65.5 (25.6 to 113.6)
		Polymicrobial	0.55 (0.36 to 0.84)	1.91 (1.2 to 3.03)	244.6 (195.3 to 298.9)	0.04 (0.02 to 0.06)	0.07 (0.04 to 0.11)	100.9 (51.9 to 162.5)
		Pseudomonas aeruginosa	7.78 (6.75 to 8.87)	40.3 (35.5 to 45.4)	419.4 (358.2 to 499.0)	0.34 (0.28 to 0.43)	1.03 (0.82 to 1.26)	202.8 (128.8 to 289.4)
		Respiratory syncytial virus	2.39 (2.07 to 2.77)	0.02 (0 to 0.08)	-99.3 (-99.9 to -96.6)	0.05 (0.04 to 0.07)	0 (0 to 0)	-99.6 (-99.9 to -98.1)
		Staphylococcus aureus	11.1 (9.74 to 12.5)	56 (50 to 63.2)	406.0 (347.2 to 473.3)	1 (0.8 to 1.23)	2.93 (2.33 to 3.56)	194.9 (125.3 to 276.5)
		Streptococcus pneumoniae	53.4 (47 to 60.3)	130 (116 to 146)	144.5 (115.2 to 176.1)	2.01 (1.61 to 2.47)	2.84 (2.28 to 3.45)	42.5 (7.8 to 82.4)
		Other viruses	19.8 (17.4 to 22.4)	79.3 (70.2 to 88.8)	301.3 (253.8 to 354.4)	0.29 (0.23 to 0.36)	0.68 (0.54 to 0.83)	133.8 (77.7 to 198.9)
		Other bacterial pathogens	14.4 (12.4 to 16.6)	38.7 (33 to 44.8)	168.9 (134.5 to 208.4)	0.52 (0.4 to 0.65)	0.8 (0.63 to 0.98)	56.9 (20.0 to 102.5)
	70+ years	*Acinetobacter baumannii*	4.57 (3.84 to 5.37)	9.98 (8.43 to 11.7)	119.1 (90.1 to 151.3)	0.8 (0.65 to 0.98)	1.19 (0.95 to 1.45)	50.8 (19.2 to 82.2)
		Chlamydia spp.	13.6 (11.8 to 15.7)	21.4 (18.8 to 24.2)	57.4 (37.4 to 80.2)	0.59 (0.49 to 0.71)	0.64 (0.52 to 0.75)	8.3 (-14.2 to 32.5)
		Enterobacter spp.	1.54 (1.27 to 1.85)	5.15 (4.39 to 5.98)	235.2 (189.7 to 284.8)	0.26 (0.2 to 0.32)	0.58 (0.47 to 0.71)	130.6 (82.3 to 181.3)

		Escherichia coli	5.78 (4.97 to 6.67)	14.7 (13 to 16.8)	154.8 (122.4 to 190.8)	0.99 (0.82 to 1.21)	1.72 (1.43 to 2.05)	75.3 (37.5 to 113.0)
		Fungus	2.52 (2.13 to 2.94)	6.69 (5.8 to 7.78)	166.4 (132.5 to 206.6)	0.42 (0.34 to 0.52)	0.77 (0.63 to 0.94)	83.3 (45.6 to 124.2)
		Group B Streptococcus	3.41 (2.92 to 3.95)	10.1 (8.88 to 11.4)	196.3 (158.4 to 236.9)	0.41 (0.34 to 0.5)	0.84 (0.69 to 1)	103.9 (63.9 to 150.6)
		Haemophilus influenzae	11.3 (9.84 to 12.7)	21.1 (18.9 to 23.7)	88.0 (64.5 to 112.5)	0.74 (0.62 to 0.88)	0.63 (0.53 to 0.76)	-13.7 (-30.3 to 4.6)
		Influenza	23.5 (20.9 to 26.3)	0.1 (0.02 to 0.43)	-99.6 (-99.9 to -98.1)	3 (2.5 to 3.53)	0.01 (0 to 0.04)	-99.7 (-100.0 to -98.8)
		Klebsiella pneumoniae	10.7 (9.51 to 11.9)	27.7 (24.8 to 31)	159.9 (131.2 to 192.8)	1.82 (1.52 to 2.16)	3.23 (2.68 to 3.85)	78.8 (43.6 to 115.7)
		Legionella spp.	5.86 (5.02 to 6.77)	16.3 (14.4 to 18.3)	178.2 (140.7 to 219.7)	0.69 (0.56 to 0.83)	1.3 (1.08 to 1.54)	91.4 (53.4 to 134.9)
		Mycoplasma spp.	10.9 (9.48 to 12.3)	21.6 (19.2 to 24.4)	99.3 (75.7 to 126.8)	0.49 (0.41 to 0.58)	0.67 (0.55 to 0.8)	37.1 (9.6 to 67.8)
		Polymicrobial	0.75 (0.49 to 1.15)	1.82 (1.12 to 2.88)	142.0 (106.1 to 180.3)	0.13 (0.08 to 0.2)	0.22 (0.13 to 0.35)	66.5 (32.5 to 104.5)
		Pseudomonas aeruginosa	9.58 (8.41 to 10.8)	34.9 (31.3 to 39.1)	264.7 (219.3 to 317.6)	1.13 (0.95 to 1.34)	2.82 (2.36 to 3.35)	150.9 (99.9 to 202.8)
		Respiratory syncytial virus	2.66 (2.31 to 3.04)	0.01 (0 to 0.06)	-99.5 (-99.9 to -97.6)	0.16 (0.13 to 0.18)	0 (0 to 0)	-99.7 (-99.9 to -98.4)
		Staphylococcus aureus	17 (15 to 19)	60.3 (54.3 to 67.3)	255.3 (214.4 to 301.7)	4.1 (3.45 to 4.85)	9.98 (8.33 to 11.7)	144.4 (97.4 to 195.5)
		Streptococcus pneumoniae	65 (57.6 to 72.4)	111 (101 to 125)	71.7 (53.3 to 93.6)	6.54 (5.51 to 7.7)	7.68 (6.43 to 9.04)	18.1 (-4.7 to 42.1)
		Other viruses	23.9 (21.2 to 26.6)	67.1 (60.3 to 75.2)	181.7 (149.0 to 218.2)	0.94 (0.79 to 1.11)	1.82 (1.52 to 2.13)	93.8 (56.6 to 132.7)
		Other bacterial pathogens	22.8 (19.6 to 25.9)	43 (37.3 to 49.1)	88.8 (65.9 to 115.2)	2.21 (1.83 to 2.69)	2.86 (2.33 to 3.43)	30.0 (3.4 to 58.6)
Japan	All Ages	*Acinetobacter baumannii*	9910 (8540 to 11500)	5880 (5100 to 6720)	-40.6 (-44.7 to -36.0)	807 (687 to 931)	621 (475 to 729)	-23.1 (-33.1 to -15.5)
		Chlamydia spp.	48600 (44300 to 53000)	37800 (34700 to 41300)	-22.2 (-27.4 to -16.8)	898 (791 to 981)	952 (758 to 1080)	5.9 (-7.5 to 16.1)
		Enterobacter spp.	9000 (7870 to 10100)	14300 (12800 to 15800)	59.0 (46.8 to 70.8)	658 (564 to 748)	1400 (1080 to 1610)	112.2 (83.4 to 134.1)
		Escherichia coli	23900 (21800 to 26300)	36000 (33300 to 38900)	50.3 (40.8 to 59.6)	1970 (1740 to 2160)	3760 (2970 to 4230)	90.6 (65.4 to 107.4)
		Fungus	15600 (14000 to 17500)	16700 (15100 to 18400)	7.1 (-1.4 to 15.7)	961 (845 to 1080)	1490 (1180 to 1730)	55.0 (34.0 to 71.2)
		Group B Streptococcus	19900 (18100 to 22000)	25500 (23500 to 27600)	28.3 (18.3 to 38.6)	965 (838 to 1070)	1740 (1380 to 1970)	80.0 (56.6 to 98.7)
		Haemophilus influenzae	87300 (81700 to 93800)	88700 (83000 to 95300)	1.7 (-3.8 to 7.1)	1960 (1760 to 2080)	2030 (1630 to 2270)	3.5 (-9.2 to 12.0)
		Influenza	126000 (118000 to 134000)	401 (17.5 to 2300)	-99.7 (-100.0 to -98.2)	7060 (6330 to 7500)	29.8 (1.25 to 172)	-99.6 (-100.0 to -97.6)
		Klebsiella pneumoniae	51700 (48600 to 55300)	51600 (48400 to 55100)	-0.2 (-5.4 to 5.0)	3670 (3290 to 3890)	5150 (4090 to 5750)	40.1 (22.7 to 50.9)

		Legionella spp.	54300 (49500 to 59200)	72400 (67600 to 77900)	33.5 (24.3 to 42.5)	2560 (2290 to 2800)	4750 (3800 to 5320)	85.7 (62.4 to 103.8)
		Mycoplasma spp.	99600 (91600 to 108000)	79600 (74500 to 85300)	-20.0 (-24.3 to -15.4)	1290 (1150 to 1390)	1590 (1280 to 1780)	23.9 (8.3 to 33.9)
		Polymicrobial	2670 (1670 to 4220)	2990 (1720 to 5110)	11.2 (-2.9 to 24.6)	200 (120 to 330)	307 (168 to 537)	51.8 (29.4 to 70.5)
		Pseudomonas aeruginosa	70900 (65900 to 76700)	106000 (98800 to 113000)	48.9 (41.5 to 56.8)	3670 (3280 to 3950)	7240 (5730 to 8080)	97.2 (72.6 to 113.0)
		Respiratory syncytial virus	68600 (62000 to 75900)	131 (5.82 to 733)	-99.8 (-100.0 to -98.9)	646 (580 to 704)	2.11 (0.09 to 12)	-99.7 (-100.0 to -98.2)
		Staphylococcus aureus	94200 (88400 to 100000)	166000 (156000 to 178000)	76.7 (68.7 to 84.8)	10600 (9510 to 11200)	24500 (19400 to 27300)	129.9 (102.5 to 147.2)
		Streptococcus pneumoniae	479000 (449000 to 512000)	251000 (237000 to 267000)	-47.6 (-50.3 to -45.2)	15500 (14000 to 16300)	13200 (10600 to 14700)	-14.7 (-25.2 to -8.3)
		Other viruses	318000 (297000 to 342000)	283000 (267000 to 300000)	-11.0 (-15.6 to -6.6)	3440 (3100 to 3620)	5390 (4320 to 5990)	56.5 (37.5 to 68.1)
		Other bacterial pathogens	85000 (75200 to 96100)	88400 (77600 to 101000)	4.1 (-2.1 to 10.4)	4170 (3600 to 4750)	5410 (4110 to 6440)	29.5 (13.5 to 40.8)
	Under 5	*Acinetobacter baumannii*	268 (176 to 401)	71 (45.6 to 108)	-73.6 (-76.4 to -70.7)	2.58 (1.48 to 4.27)	0.32 (0.17 to 0.56)	-87.7 (-89.9 to -85.1)
		Chlamydia spp.	3720 (3120 to 4390)	1360 (1120 to 1620)	-63.5 (-68.0 to -58.6)	4.42 (4.01 to 4.84)	0.63 (0.54 to 0.73)	-85.7 (-87.9 to -83.4)
		Enterobacter spp.	604 (457 to 785)	414 (312 to 539)	-31.3 (-40.5 to -21.0)	2.97 (2.19 to 4.05)	0.72 (0.56 to 0.95)	-75.4 (-79.8 to -70.5)
		Escherichia coli	1690 (1410 to 2010)	1250 (1030 to 1500)	-26.0 (-36.1 to -15.9)	8.02 (7.22 to 9.01)	2.07 (1.78 to 2.42)	-74.1 (-78.1 to -70.1)
		Fungus	317 (225 to 439)	181 (129 to 250)	-42.6 (-51.6 to -32.8)	1.01 (0.74 to 1.34)	0.24 (0.17 to 0.33)	-76.1 (-80.6 to -71.1)
		Group B Streptococcus	1620 (1340 to 1940)	987 (802 to 1200)	-39.0 (-47.0 to -31.4)	6.4 (5.77 to 7.07)	1.35 (1.14 to 1.57)	-78.9 (-82.0 to -75.5)
		Haemophilus influenzae	15300 (12700 to 18000)	7140 (5960 to 8610)	-53.2 (-59.2 to -47.3)	21.6 (19.7 to 23.6)	2.53 (2.21 to 2.91)	-88.2 (-90.1 to -86.1)
		Influenza	12200 (10300 to 14200)	13.3 (0.59 to 72.8)	-99.9 (-100.0 to -99.4)	32.4 (30.1 to 34.7)	0.01 (0 to 0.08)	-100.0 (-100.0 to -99.8)
		Klebsiella pneumoniae	6070 (5140 to 7130)	2010 (1670 to 2410)	-67.0 (-70.6 to -63.0)	22.6 (20.9 to 24.4)	3.12 (2.72 to 3.57)	-86.2 (-88.0 to -84.0)
		Legionella spp.	910 (656 to 1250)	572 (400 to 797)	-37.2 (-44.8 to -28.6)	3.7 (2.86 to 4.61)	0.83 (0.62 to 1.07)	-77.6 (-81.2 to -73.7)
		Mycoplasma spp.	8280 (6960 to 9810)	6680 (5560 to 8060)	-19.2 (-28.7 to -9.9)	7.88 (7.18 to 8.65)	2.48 (2.17 to 2.82)	-68.5 (-73.3 to -63.1)
		Polymicrobial	336 (188 to 555)	136 (75.9 to 223)	-59.6 (-63.5 to -55.2)	4.03 (2.09 to 6.72)	0.7 (0.34 to 1.23)	-82.6 (-85.7 to -79.3)
		Pseudomonas aeruginosa	5120 (4300 to 6090)	4600 (3820 to 5540)	-9.9 (-20.1 to 1.2)	14.8 (13.5 to 16.3)	4.87 (4.28 to 5.51)	-67.1 (-72.1 to -62.0)
		Respiratory syncytial virus	40900 (35300 to 47100)	62.5 (2.71 to 342)	-99.8 (-100.0 to -99.1)	53.8 (50.7 to 56.9)	0.03 (0 to 0.17)	-99.9 (-100.0 to -99.7)
		Staphylococcus aureus	5430 (4630 to 6360)	4010 (3320 to 4780)	-26.2 (-32.4 to -20.3)	28.3 (26.5 to 30.1)	8.23 (7.26 to 9.29)	-70.9 (-74.8 to -66.9)

		Streptococcus pneumoniae	76400 (64800 to 88400)	12900 (10800 to 15400)	-83.0 (-84.4 to -81.8)	150 (143 to 158)	10.4 (9.13 to 11.7)	-93.1 (-94.0 to -92.1)
		Other viruses	80900 (68500 to 93800)	29700 (24900 to 35300)	-63.3 (-66.2 to -60.6)	63.7 (60.3 to 67.1)	9.83 (8.67 to 11.1)	-84.6 (-86.6 to -82.3)
		Other bacterial pathogens	1790 (1390 to 2260)	839 (646 to 1090)	-52.9 (-59.7 to -46.0)	4.33 (3.42 to 5.46)	0.79 (0.62 to 1.01)	-81.6 (-84.7 to -78.2)
	5-14 years	*Acinetobacter baumannii*	616 (450 to 833)	166 (127 to 217)	-72.9 (-75.5 to -70.3)	1.12 (0.96 to 1.3)	0.14 (0.12 to 0.16)	-87.3 (-88.3 to -86.3)
		Chlamydia spp.	3970 (2940 to 5260)	1480 (1140 to 1880)	-62.6 (-66.1 to -59.1)	1.79 (1.64 to 1.95)	0.31 (0.28 to 0.34)	-82.5 (-83.8 to -81.2)
		Enterobacter spp.	557 (398 to 756)	420 (314 to 548)	-24.4 (-31.7 to -16.2)	0.96 (0.84 to 1.09)	0.34 (0.3 to 0.38)	-64.6 (-67.9 to -61.5)
		Escherichia coli	1200 (882 to 1590)	807 (622 to 1020)	-32.3 (-38.2 to -26.2)	2.14 (1.95 to 2.33)	0.68 (0.62 to 0.72)	-68.3 (-70.6 to -65.9)
		Fungus	1440 (1050 to 1930)	838 (639 to 1080)	-41.4 (-47.0 to -35.4)	2.52 (2.27 to 2.8)	0.69 (0.62 to 0.77)	-72.5 (-74.8 to -70.3)
		Group B Streptococcus	1640 (1190 to 2180)	1070 (818 to 1360)	-34.7 (-41.2 to -28.0)	2.08 (1.88 to 2.33)	0.64 (0.58 to 0.7)	-69.4 (-72.0 to -66.9)
		Haemophilus influenzae	7330 (5510 to 9690)	4070 (3150 to 5170)	-44.3 (-48.7 to -39.8)	4.99 (4.74 to 5.26)	0.87 (0.81 to 0.92)	-82.6 (-83.7 to -81.5)
		Influenza	9960 (7470 to 13000)	14.4 (0.61 to 80)	-99.9 (-100.0 to -99.2)	13.3 (12.7 to 13.9)	0.01 (0 to 0.05)	-99.9 (-100.0 to -99.6)
		Klebsiella pneumoniae	3490 (2620 to 4550)	1670 (1300 to 2100)	-52.0 (-55.9 to -47.8)	6.19 (5.9 to 6.49)	1.39 (1.32 to 1.47)	-77.5 (-78.8 to -76.2)
		Legionella spp.	4650 (3470 to 6180)	3170 (2450 to 4010)	-31.5 (-37.7 to -24.9)	5.68 (5.22 to 6.16)	1.82 (1.7 to 1.93)	-67.9 (-70.3 to -65.5)
		Mycoplasma spp.	15600 (11600 to 20400)	7510 (5830 to 9540)	-51.7 (-55.4 to -47.3)	7.35 (6.93 to 7.78)	1.66 (1.57 to 1.76)	-77.4 (-78.8 to -75.9)
		Polymicrobial	161 (91.9 to 260)	87 (47.6 to 146)	-46.0 (-52.0 to -39.3)	0.29 (0.17 to 0.48)	0.07 (0.04 to 0.12)	-74.7 (-77.3 to -72.2)
		Pseudomonas aeruginosa	4790 (3580 to 6310)	3430 (2670 to 4330)	-28.2 (-34.1 to -22.2)	5.9 (5.52 to 6.27)	1.98 (1.86 to 2.1)	-66.4 (-68.4 to -64.3)
		Respiratory syncytial virus	3760 (2840 to 4980)	4.92 (0.21 to 27.7)	-99.9 (-100.0 to -99.2)	2.28 (2.12 to 2.46)	0 (0 to 0.01)	-99.9 (-100.0 to -99.7)
		Staphylococcus aureus	6370 (4820 to 8320)	5070 (3970 to 6340)	-20.2 (-26.3 to -14.0)	16 (15.4 to 16.7)	6 (5.72 to 6.27)	-62.6 (-64.6 to -60.5)
		Streptococcus pneumoniae	53900 (40800 to 70300)	15800 (12400 to 19700)	-70.6 (-72.8 to -68.3)	56.5 (54.8 to 58.2)	7.78 (7.46 to 8.14)	-86.2 (-87.0 to -85.5)
		Other viruses	34600 (26200 to 45200)	19100 (14900 to 23700)	-44.7 (-48.8 to -40.2)	14.3 (13.8 to 14.8)	3.69 (3.52 to 3.87)	-74.1 (-75.5 to -72.7)
		Other bacterial pathogens	5010 (3670 to 6730)	2250 (1700 to 2890)	-54.9 (-58.7 to -51.0)	5.01 (4.43 to 5.64)	1.06 (0.92 to 1.2)	-78.9 (-80.3 to -77.3)
	15-49 years	*Acinetobacter baumannii*	1060 (838 to 1270)	262 (214 to 313)	-75.2 (-76.9 to -73.4)	8.87 (7.6 to 10.3)	1.88 (1.61 to 2.15)	-78.8 (-80.2 to -77.3)
		Chlamydia spp.	6810 (5800 to 7910)	2330 (2010 to 2640)	-65.8 (-67.9 to -63.6)	14.2 (13 to 15.4)	4.15 (3.82 to 4.53)	-70.7 (-72.7 to -68.8)
		Enterobacter spp.	955 (788 to 1150)	660 (552 to 784)	-30.8 (-35.7 to -25.8)	7.6 (6.66 to 8.65)	4.49 (3.98 to 5.04)	-40.8 (-45.2 to -36.3)

		Escherichia coli	2050 (1730 to 2380)	1270 (1100 to 1460)	-38.0 (-41.7 to -34.4)	16.9 (15.5 to 18.4)	8.94 (8.34 to 9.51)	-47.0 (-50.3 to -43.7)
		Fungus	2460 (2070 to 2870)	1320 (1130 to 1520)	-46.3 (-50.2 to -42.5)	20 (18.1 to 22.1)	9.16 (8.32 to 10.1)	-54.1 (-57.4 to -50.7)
		Group B Streptococcus	2820 (2390 to 3350)	1680 (1450 to 1960)	-40.2 (-44.6 to -35.9)	16.5 (14.9 to 18.3)	8.4 (7.69 to 9.19)	-48.9 (-52.9 to -45.1)
		Haemophilus influenzae	12600 (10800 to 14400)	6410 (5580 to 7300)	-49.0 (-51.3 to -46.6)	39.5 (37.5 to 41.4)	11.5 (10.9 to 12.1)	-70.9 (-72.3 to -69.6)
		Influenza	17100 (14700 to 19500)	23 (1.01 to 131)	-99.9 (-100.0 to -99.2)	105 (101 to 109)	0.12 (0.01 to 0.64)	-99.9 (-100.0 to -99.4)
		Klebsiella pneumoniae	5990 (5170 to 6870)	2630 (2300 to 2970)	-56.1 (-58.0 to -54.3)	49 (46.9 to 51.3)	18.4 (17.5 to 19.3)	-62.4 (-64.2 to -60.8)
		Legionella spp.	7970 (6780 to 9220)	4990 (4360 to 5680)	-37.3 (-41.3 to -33.2)	44.9 (41.3 to 48.8)	24 (22.7 to 25.3)	-46.4 (-49.8 to -42.7)
		Mycoplasma spp.	26800 (23100 to 30700)	11800 (10300 to 13400)	-55.8 (-57.8 to -53.7)	58.1 (55.1 to 61.4)	22 (20.9 to 23.1)	-62.2 (-64.3 to -60.2)
		Polymicrobial	276 (161 to 448)	137 (74.5 to 228)	-50.6 (-54.8 to -45.7)	2.3 (1.36 to 3.77)	0.98 (0.56 to 1.65)	-57.7 (-61.6 to -53.4)
		Pseudomonas aeruginosa	8210 (7040 to 9460)	5390 (4710 to 6180)	-34.3 (-37.3 to -31.2)	46.6 (43.6 to 49.6)	26.2 (24.9 to 27.6)	-43.8 (-46.5 to -41.0)
		Respiratory syncytial virus	6440 (5550 to 7410)	7.84 (0.34 to 44.5)	-99.9 (-100.0 to -99.3)	18 (16.8 to 19.3)	0.02 (0 to 0.1)	-99.9 (-100.0 to -99.4)
		Staphylococcus aureus	10900 (9420 to 12400)	7970 (7000 to 9020)	-26.9 (-29.8 to -24.1)	127 (123 to 131)	79.3 (76.4 to 82.1)	-37.5 (-40.1 to -35.0)
		Streptococcus pneumoniae	92400 (80200 to 105000)	24900 (21800 to 28200)	-73.1 (-74.1 to -72.1)	447 (435 to 458)	103 (99.3 to 106)	-77.0 (-77.9 to -76.1)
		Other viruses	59300 (51400 to 67400)	30000 (26300 to 34000)	-49.4 (-51.4 to -47.4)	113 (110 to 116)	48.9 (47 to 50.5)	-56.7 (-58.4 to -54.9)
		Other bacterial pathogens	8590 (7090 to 10200)	3540 (2950 to 4170)	-58.7 (-60.9 to -56.4)	39.7 (35.2 to 44.6)	14 (12.3 to 15.9)	-64.6 (-66.7 to -62.5)
	50-69 years	*Acinetobacter baumannii*	2910 (2470 to 3390)	816 (695 to 941)	-72.0 (-74.0 to -69.9)	101 (88.7 to 116)	30.3 (26.3 to 34.7)	-70.1 (-72.3 to -67.9)
		Chlamydia spp.	11100 (9860 to 12500)	4250 (3810 to 4740)	-61.7 (-64.2 to -58.9)	96.1 (87.9 to 105)	39.2 (35.9 to 42.8)	-59.1 (-62.1 to -55.9)
		Enterobacter spp.	2560 (2220 to 2930)	1980 (1730 to 2230)	-22.4 (-28.3 to -16.6)	84.4 (75.1 to 95.4)	69.8 (62.9 to 77.1)	-17.2 (-24.0 to -10.4)
		Escherichia coli	6070 (5420 to 6810)	4150 (3760 to 4540)	-31.7 (-35.9 to -27.5)	207 (190 to 224)	151 (141 to 160)	-27.1 (-31.7 to -22.3)
		Fungus	5790 (5070 to 6570)	3490 (3090 to 3890)	-39.7 (-43.9 to -34.9)	195 (176 to 216)	125 (113 to 137)	-35.7 (-40.6 to -30.7)
		Group B Streptococcus	5340 (4680 to 6070)	3550 (3190 to 3940)	-33.4 (-38.4 to -28.2)	129 (117 to 144)	91.9 (83.8 to 99.7)	-28.9 (-34.6 to -23.0)
		Haemophilus influenzae	20300 (18400 to 22300)	11800 (10700 to 12800)	-42.1 (-45.0 to -39.2)	265 (251 to 278)	109 (102 to 115)	-58.8 (-61.1 to -56.5)
		Influenza	23800 (21700 to 26000)	36.9 (1.64 to 207)	-99.8 (-100.0 to -99.1)	607 (576 to 633)	0.98 (0.04 to 5.42)	-99.8 (-100.0 to -99.1)
		Klebsiella pneumoniae	12400 (11300 to 13600)	6320 (5770 to 6850)	-49.1 (-51.6 to -46.6)	421 (401 to 441)	229 (215 to 241)	-45.7 (-48.5 to -42.6)

		Legionella spp.	18100 (16200 to 20200)	12700 (11500 to 13800)	-30.0 (-34.6 to -25.4)	424 (391 to 460)	316 (296 to 336)	-25.4 (-30.7 to -19.4)
		Mycoplasma spp.	19800 (17800 to 21900)	9360 (8500 to 10200)	-52.8 (-55.4 to -50.1)	179 (167 to 192)	90 (84.1 to 96.2)	-49.7 (-52.9 to -46.7)
		Polymicrobial	634 (381 to 1040)	355 (203 to 609)	-44.3 (-49.2 to -38.8)	21.9 (13.1 to 36.2)	13.1 (7.48 to 22.2)	-40.6 (-46.1 to -34.5)
		Pseudomonas aeruginosa	18800 (17000 to 20800)	13500 (12300 to 14700)	-28.2 (-32.0 to -24.3)	444 (417 to 473)	340 (319 to 358)	-23.4 (-27.6 to -19.1)
		Respiratory syncytial virus	6640 (5880 to 7410)	8.88 (0.4 to 49.3)	-99.9 (-100.0 to -99.2)	77.2 (70.1 to 84.9)	0.11 (0 to 0.6)	-99.9 (-100.0 to -99.2)
		Staphylococcus aureus	22000 (20100 to 24100)	18100 (16500 to 19600)	-17.7 (-21.2 to -14.0)	1060 (1020 to 1100)	932 (883 to 973)	-12.2 (-16.3 to -7.7)
		Streptococcus pneumoniae	92000 (84100 to 100000)	29200 (26600 to 31600)	-68.3 (-69.6 to -67.0)	1850 (1780 to 1910)	625 (592 to 656)	-66.2 (-67.7 to -64.5)
		Other viruses	51700 (47400 to 56300)	30500 (27800 to 33000)	-41.1 (-43.6 to -38.5)	408 (391 to 425)	256 (243 to 269)	-37.2 (-40.1 to -33.9)
		Other bacterial pathogens	21000 (18000 to 24000)	9640 (8280 to 11000)	-54.0 (-56.5 to -51.1)	402 (356 to 450)	198 (173 to 224)	-50.9 (-53.8 to -47.5)
	70+ years	*Acinetobacter baumannii*	5050 (4280 to 5950)	4560 (3950 to 5260)	-9.6 (-16.3 to -2.7)	693 (586 to 803)	588 (444 to 694)	-15.2 (-26.1 to -6.9)
		Chlamydia spp.	23000 (20500 to 25800)	28300 (25700 to 31500)	23.6 (14.9 to 32.9)	782 (683 to 859)	908 (714 to 1030)	16.0 (1.6 to 27.4)
		Enterobacter spp.	4320 (3720 to 4990)	10800 (9490 to 12100)	150.5 (130.2 to 171.3)	562 (477 to 641)	1320 (1010 to 1530)	135.0 (103.8 to 159.3)
		Escherichia coli	12900 (11600 to 14600)	28500 (26100 to 31100)	120.5 (106.1 to 134.8)	1740 (1520 to 1910)	3600 (2810 to 4060)	106.9 (79.9 to 125.0)
		Fungus	5600 (4910 to 6410)	10900 (9660 to 12200)	94.4 (78.8 to 109.8)	742 (642 to 843)	1360 (1050 to 1590)	82.4 (58.7 to 101.2)
		Group B Streptococcus	8500 (7550 to 9510)	18200 (16600 to 20000)	114.9 (97.6 to 131.4)	811 (694 to 903)	1640 (1280 to 1870)	101.7 (76.5 to 122.3)
		Haemophilus influenzae	31800 (29100 to 34800)	59300 (54700 to 64700)	86.9 (76.5 to 97.1)	1630 (1440 to 1740)	1910 (1510 to 2140)	16.9 (2.8 to 26.3)
		Influenza	62800 (57700 to 68500)	314 (13.7 to 1790)	-99.5 (-100.0 to -97.1)	6300 (5590 to 6720)	28.7 (1.2 to 166)	-99.5 (-100.0 to -97.4)
		Klebsiella pneumoniae	23700 (21800 to 25900)	38900 (36100 to 42200)	64.3 (55.6 to 73.3)	3170 (2810 to 3380)	4900 (3840 to 5490)	54.2 (35.7 to 66.1)
		Legionella spp.	22600 (20100 to 25300)	51000 (46800 to 55500)	125.8 (109.5 to 142.6)	2080 (1830 to 2300)	4410 (3470 to 4970)	111.8 (85.8 to 132.8)
		Mycoplasma spp.	29100 (26300 to 32400)	44300 (40600 to 48300)	52.3 (43.2 to 61.2)	1030 (911 to 1120)	1480 (1170 to 1670)	42.9 (25.4 to 54.4)
		Polymicrobial	1260 (743 to 2120)	2280 (1250 to 3910)	79.7 (63.7 to 97.4)	172 (101 to 284)	292 (159 to 510)	68.6 (45.8 to 88.4)
		Pseudomonas aeruginosa	34000 (31000 to 37800)	78700 (72700 to 85300)	131.6 (118.2 to 145.0)	3160 (2770 to 3410)	6870 (5380 to 7690)	117.3 (90.5 to 134.2)
		Respiratory syncytial virus	10800 (9630 to 12200)	46.8 (2.03 to 272)	-99.6 (-100.0 to -97.5)	494 (433 to 547)	1.95 (0.08 to 11.1)	-99.6 (-100.0 to -97.8)
		Staphylococcus aureus	49400 (45400 to 54100)	131000 (121000 to 142000)	165.6 (152.4 to 177.9)	9390 (8310 to 9990)	23400 (18400 to 26200)	149.2 (119.9 to 167.8)

		Streptococcus pneumoniae	164000 (152000 to 180000)	168000 (156000 to 182000)	2.3 (-2.6 to 7.1)	13000 (11500 to 13800)	12500 (9860 to 13900)	-4.0 (-15.5 to 2.9)
		Other viruses	91200 (84300 to 99800)	173000 (160000 to 188000)	90.1 (80.0 to 99.1)	2840 (2510 to 3010)	5070 (4010 to 5660)	78.3 (57.5 to 91.2)
		Other bacterial pathogens	48700 (42600 to 56000)	72200 (62900 to 83200)	48.4 (39.3 to 57.6)	3720 (3190 to 4250)	5190 (3920 to 6200)	39.4 (22.5 to 51.6)
Republic of Korea	All Ages	*Acinetobacter baumannii*	4060 (3500 to 4680)	1710 (1490 to 1960)	-57.7 (-62.5 to -52.6)	140 (117 to 170)	132 (99.5 to 162)	-5.1 (-29.3 to 15.8)
		Chlamydia spp.	18700 (16800 to 20900)	10100 (9260 to 10900)	-45.8 (-51.5 to -40.1)	115 (99.5 to 137)	181 (142 to 217)	59.1 (20.2 to 92.7)
		Enterobacter spp.	1950 (1680 to 2270)	3250 (2870 to 3630)	67.1 (44.2 to 91.1)	53.3 (44.4 to 64.4)	229 (176 to 277)	332.3 (222.4 to 430.6)
		Escherichia coli	5860 (5260 to 6520)	7880 (7230 to 8570)	34.9 (20.2 to 50.6)	184 (158 to 225)	619 (482 to 747)	238.5 (154.4 to 313.2)
		Fungus	4380 (3740 to 5070)	4600 (4170 to 5080)	5.3 (-7.4 to 19.6)	101 (85.9 to 119)	268 (212 to 321)	166.4 (104.7 to 224.4)
		Group B Streptococcus	5650 (5000 to 6420)	6300 (5770 to 6860)	11.8 (-0.5 to 25.6)	95.2 (82.2 to 113)	297 (232 to 355)	213.7 (136.5 to 279.7)
		Haemophilus influenzae	28400 (25500 to 31700)	23000 (21300 to 24800)	-18.7 (-27.7 to -9.7)	183 (161 to 215)	357 (286 to 423)	95.9 (48.6 to 136.0)
		Influenza	33800 (30700 to 37100)	11 (9.94 to 12.1)	-100.0 (-100.0 to -100.0)	580 (506 to 693)	0.58 (0.45 to 0.75)	-99.9 (-99.9 to -99.9)
		Klebsiella pneumoniae	16500 (15000 to 18200)	13100 (12200 to 14100)	-20.3 (-28.4 to -12.0)	368 (323 to 438)	939 (736 to 1110)	156.3 (93.9 to 207.4)
		Legionella spp.	8960 (7940 to 10200)	18500 (17000 to 19900)	106.9 (85.7 to 130.6)	162 (140 to 193)	824 (658 to 977)	410.9 (291.1 to 517.9)
		Mycoplasma spp.	36900 (32800 to 41400)	24700 (22800 to 26800)	-32.7 (-38.6 to -26.7)	137 (123 to 159)	303 (238 to 358)	121.2 (70.8 to 161.7)
		Polymicrobial	928 (656 to 1310)	706 (412 to 1180)	-24.8 (-42.7 to -3.6)	30.5 (21.1 to 44.5)	53.1 (28.8 to 91.9)	73.2 (13.8 to 139.4)
		Pseudomonas aeruginosa	13300 (12000 to 14700)	25200 (23300 to 27200)	90.2 (72.1 to 108.7)	241 (211 to 282)	1240 (972 to 1460)	416.5 (292.5 to 523.9)
		Respiratory syncytial virus	29800 (26400 to 33800)	4.71 (4.08 to 5.42)	-100.0 (-100.0 to -100.0)	92.8 (78.4 to 110)	0.05 (0.04 to 0.06)	-100.0 (-100.0 to -99.9)
		Staphylococcus aureus	19600 (17900 to 21500)	36100 (33500 to 38800)	84.3 (67.3 to 102.4)	779 (676 to 941)	3920 (3050 to 4660)	404.9 (280.1 to 507.9)
		Streptococcus pneumoniae	184000 (166000 to 202000)	80800 (75100 to 86600)	-55.9 (-59.8 to -52.1)	1590 (1420 to 1860)	2790 (2220 to 3310)	75.6 (35.2 to 107.7)
		Other viruses	101000 (91200 to 112000)	80300 (74600 to 86100)	-20.3 (-27.2 to -13.2)	281 (251 to 327)	978 (779 to 1160)	249.1 (170.4 to 315.8)
		Other bacterial pathogens	19000 (16700 to 21500)	21400 (18700 to 24300)	12.7 (1.8 to 25.5)	380 (321 to 462)	987 (746 to 1230)	160.7 (95.3 to 214.3)
	Under 5	*Acinetobacter baumannii*	386 (268 to 542)	20.8 (13.9 to 31)	-94.6 (-95.5 to -93.6)	7.87 (4.34 to 13.9)	0.09 (0.04 to 0.16)	-98.9 (-99.4 to -98.2)
		Chlamydia spp.	4650 (3970 to 5430)	435 (349 to 539)	-90.6 (-92.2 to -88.8)	10.2 (7.63 to 13.1)	0.15 (0.1 to 0.19)	-98.5 (-99.1 to -97.9)
		Enterobacter spp.	486 (372 to 623)	115 (84.5 to 155)	-76.3 (-81.0 to -70.7)	4.65 (2.92 to 7.11)	0.15 (0.1 to 0.22)	-96.7 (-98.0 to -94.9)

		Escherichia coli	1650 (1370 to 1970)	352 (280 to 434)	-78.7 (-82.6 to -74.2)	14.5 (10.9 to 18.9)	0.41 (0.3 to 0.55)	-97.1 (-98.1 to -95.7)
		Fungus	313 (217 to 440)	54 (37.3 to 75.1)	-82.7 (-86.2 to -78.6)	1.32 (0.85 to 1.93)	0.04 (0.03 to 0.07)	-96.5 (-97.9 to -94.6)
		Group B Streptococcus	1470 (1200 to 1760)	282 (227 to 353)	-80.8 (-84.1 to -77.1)	12.4 (9.18 to 16.1)	0.29 (0.21 to 0.39)	-97.6 (-98.5 to -96.5)
		Haemophilus influenzae	12400 (10400 to 14700)	2130 (1720 to 2620)	-82.8 (-85.8 to -79.3)	28.5 (21.5 to 36.9)	0.49 (0.35 to 0.67)	-98.2 (-98.9 to -97.4)
		Influenza	10300 (8840 to 12000)	0.47 (0.37 to 0.58)	-100.0 (-100.0 to -100.0)	42.9 (32.6 to 54.7)	0 (0 to 0)	-100.0 (-100.0 to -100.0)
		Klebsiella pneumoniae	6030 (5150 to 7050)	627 (507 to 778)	-89.6 (-91.3 to -87.7)	35.7 (27.3 to 44.9)	0.66 (0.48 to 0.86)	-98.1 (-98.8 to -97.3)
		Legionella spp.	591 (425 to 807)	162 (107 to 229)	-72.6 (-77.8 to -66.8)	5.66 (3.77 to 8.32)	0.18 (0.12 to 0.26)	-96.7 (-98.1 to -95.0)
		Mycoplasma spp.	7070 (5960 to 8270)	2120 (1700 to 2630)	-70.0 (-74.9 to -64.1)	11 (8.22 to 14.3)	0.5 (0.36 to 0.68)	-95.3 (-97.0 to -93.2)
		Polymicrobial	288 (175 to 446)	33.9 (19.4 to 54.7)	-88.3 (-90.4 to -85.8)	8.11 (4.15 to 13.8)	0.18 (0.08 to 0.34)	-97.8 (-98.8 to -96.4)
		Pseudomonas aeruginosa	3430 (2910 to 4020)	1340 (1080 to 1650)	-60.9 (-67.6 to -53.0)	18.4 (14 to 23.4)	0.95 (0.68 to 1.25)	-94.7 (-96.6 to -92.3)
		Respiratory syncytial virus	24500 (21300 to 28100)	2.37 (1.89 to 2.94)	-100.0 (-100.0 to -100.0)	56 (42.8 to 71.8)	0 (0 to 0)	-100.0 (-100.0 to -100.0)
		Staphylococcus aureus	4260 (3660 to 4920)	1110 (893 to 1360)	-74.0 (-77.9 to -69.4)	34.4 (26.3 to 43.8)	1.5 (1.08 to 1.99)	-95.6 (-97.1 to -93.5)
		Streptococcus pneumoniae	67500 (58400 to 78000)	4690 (3790 to 5740)	-93.0 (-94.1 to -91.9)	182 (137 to 233)	2.4 (1.7 to 3.22)	-98.7 (-99.1 to -98.0)
		Other viruses	50300 (43200 to 58300)	9200 (7480 to 11200)	-81.7 (-84.4 to -78.8)	57.4 (43.3 to 73.2)	1.96 (1.41 to 2.62)	-96.5 (-97.8 to -94.8)
		Other bacterial pathogens	1830 (1430 to 2260)	264 (195 to 348)	-85.6 (-88.3 to -82.5)	7.58 (5.18 to 10.7)	0.17 (0.11 to 0.24)	-97.7 (-98.5 to -96.6)
	5-14 years	*Acinetobacter baumannii*	1110 (853 to 1420)	92.3 (68.7 to 124)	-91.7 (-93.0 to -90.2)	3.99 (2.87 to 5.24)	0.05 (0.04 to 0.07)	-98.7 (-99.1 to -98.1)
		Chlamydia spp.	4970 (3870 to 6270)	736 (554 to 948)	-85.2 (-87.4 to -82.7)	4.45 (3.24 to 5.64)	0.1 (0.08 to 0.13)	-97.7 (-98.4 to -96.5)
		Enterobacter spp.	450 (344 to 582)	179 (131 to 236)	-60.2 (-67.7 to -52.2)	1.54 (1.1 to 2)	0.09 (0.07 to 0.12)	-93.8 (-95.8 to -90.8)
		Escherichia coli	1180 (918 to 1480)	344 (261 to 445)	-70.7 (-75.2 to -65.6)	4.16 (3.11 to 5.32)	0.19 (0.15 to 0.24)	-95.4 (-96.8 to -93.3)
		Fungus	1420 (1100 to 1790)	378 (281 to 492)	-73.3 (-77.6 to -68.2)	4.94 (3.6 to 6.32)	0.2 (0.16 to 0.26)	-95.8 (-97.1 to -93.8)
		Group B Streptococcus	1470 (1140 to 1840)	470 (355 to 606)	-68.0 (-72.9 to -62.4)	3.69 (2.7 to 4.66)	0.18 (0.14 to 0.23)	-95.0 (-96.6 to -92.7)
		Haemophilus influenzae	5900 (4710 to 7430)	1850 (1400 to 2400)	-68.7 (-73.4 to -63.7)	7.96 (5.95 to 10)	0.26 (0.2 to 0.32)	-96.7 (-97.7 to -95.1)
		Influenza	8400 (6680 to 10500)	0.78 (0.58 to 1.01)	-100.0 (-100.0 to -100.0)	22.2 (16.6 to 28.1)	0 (0 to 0)	-100.0 (-100.0 to -100.0)
		Klebsiella pneumoniae	3460 (2740 to 4280)	808 (616 to 1020)	-76.6 (-80.2 to -72.9)	12.1 (9.03 to 15.2)	0.44 (0.34 to 0.55)	-96.3 (-97.5 to -94.6)

		Legionella spp.	2860 (2260 to 3600)	1400 (1060 to 1810)	-51.0 (-58.6 to -42.6)	6.92 (5.14 to 8.84)	0.52 (0.41 to 0.66)	-92.3 (-94.7 to -88.8)
		Mycoplasma spp.	13100 (10300 to 16400)	3530 (2660 to 4530)	-73.1 (-77.1 to -68.6)	12.2 (9.06 to 15.3)	0.5 (0.4 to 0.64)	-95.8 (-97.1 to -93.7)
		Polymicrobial	202 (126 to 307)	39.4 (21.7 to 65)	-80.7 (-84.7 to -76.2)	0.72 (0.44 to 1.17)	0.02 (0.01 to 0.04)	-97.0 (-98.0 to -95.5)
		Pseudomonas aeruginosa	3160 (2470 to 3940)	1500 (1140 to 1930)	-52.6 (-60.1 to -44.5)	7.72 (5.71 to 9.83)	0.56 (0.44 to 0.71)	-92.6 (-94.8 to -88.9)
		Respiratory syncytial virus	2210 (1740 to 2760)	0.28 (0.21 to 0.37)	-100.0 (-100.0 to -100.0)	2.66 (1.99 to 3.33)	0 (0 to 0)	-100.0 (-100.0 to -100.0)
		Staphylococcus aureus	4990 (3980 to 6150)	2130 (1630 to 2710)	-57.2 (-63.6 to -50.5)	24.9 (18.7 to 31.3)	1.63 (1.29 to 2.07)	-93.3 (-95.4 to -90.0)
		Streptococcus pneumoniae	47700 (38000 to 59100)	8860 (6790 to 11300)	-81.4 (-84.2 to -78.5)	99.1 (74.4 to 125)	2.83 (2.25 to 3.61)	-97.1 (-98.0 to -95.7)
		Other viruses	21400 (17100 to 26700)	9110 (6990 to 11600)	-57.5 (-63.8 to -50.6)	17.5 (13 to 21.9)	1.14 (0.91 to 1.45)	-93.4 (-95.4 to -90.0)
		Other bacterial pathogens	5120 (4000 to 6510)	1080 (807 to 1400)	-79.0 (-82.2 to -75.4)	10.1 (7.46 to 12.9)	0.33 (0.25 to 0.42)	-96.7 (-97.7 to -95.1)
	15-49 years	*Acinetobacter baumannii*	1020 (834 to 1230)	162 (129 to 196)	-84.2 (-86.0 to -82.2)	8.85 (7.24 to 10.5)	0.91 (0.73 to 1.15)	-89.7 (-91.7 to -86.9)
		Chlamydia spp.	4590 (3880 to 5360)	1290 (1090 to 1510)	-71.8 (-74.7 to -68.7)	9.87 (8.27 to 11.5)	1.81 (1.5 to 2.19)	-81.6 (-85.2 to -76.6)
		Enterobacter spp.	415 (336 to 500)	313 (256 to 381)	-24.5 (-35.3 to -12.6)	3.41 (2.77 to 4.11)	1.67 (1.33 to 2.06)	-50.7 (-61.2 to -36.9)
		Escherichia coli	1080 (910 to 1270)	603 (512 to 703)	-44.3 (-50.6 to -37.6)	9.21 (7.81 to 10.7)	3.34 (2.77 to 4.03)	-63.6 (-70.8 to -54.5)
		Fungus	1310 (1090 to 1550)	662 (552 to 784)	-49.3 (-55.2 to -42.3)	11 (9.07 to 12.9)	3.61 (2.94 to 4.43)	-66.9 (-73.8 to -57.7)
		Group B Streptococcus	1360 (1140 to 1630)	823 (698 to 982)	-39.2 (-46.0 to -31.6)	8.18 (6.82 to 9.7)	3.23 (2.68 to 3.97)	-60.2 (-68.3 to -50.1)
		Haemophilus influenzae	5450 (4660 to 6270)	3230 (2740 to 3760)	-40.6 (-46.3 to -34.0)	17.6 (14.9 to 20)	4.57 (3.83 to 5.49)	-74.0 (-79.1 to -67.2)
		Influenza	7750 (6650 to 8900)	1.37 (1.15 to 1.6)	-100.0 (-100.0 to -100.0)	49.1 (42.1 to 55.8)	0.01 (0 to 0.01)	-100.0 (-100.0 to -100.0)
		Klebsiella pneumoniae	3190 (2740 to 3660)	1410 (1200 to 1650)	-55.6 (-59.6 to -50.8)	26.9 (23.2 to 30.5)	7.78 (6.46 to 9.37)	-71.0 (-76.7 to -64.0)
		Legionella spp.	2640 (2210 to 3100)	2450 (2090 to 2860)	-7.0 (-17.1 to 4.8)	15.3 (12.9 to 17.8)	9.27 (7.73 to 11.2)	-39.2 (-51.3 to -23.9)
		Mycoplasma spp.	12100 (10400 to 13900)	6180 (5260 to 7180)	-48.9 (-53.7 to -43.4)	27.2 (23 to 30.6)	9.02 (7.54 to 10.9)	-66.6 (-73.1 to -57.9)
		Polymicrobial	187 (119 to 285)	69.2 (37.3 to 115)	-63.4 (-69.7 to -56.0)	1.61 (1.03 to 2.47)	0.39 (0.22 to 0.66)	-76.1 (-81.6 to -68.3)
		Pseudomonas aeruginosa	2920 (2490 to 3370)	2630 (2240 to 3070)	-9.9 (-18.1 to -0.8)	17.1 (14.6 to 19.4)	10 (8.38 to 12.2)	-41.2 (-52.6 to -26.3)
		Respiratory syncytial virus	2040 (1730 to 2350)	0.49 (0.41 to 0.59)	-100.0 (-100.0 to -100.0)	5.89 (4.97 to 6.74)	0 (0 to 0)	-100.0 (-100.0 to -100.0)
		Staphylococcus aureus	4600 (3970 to 5280)	3740 (3210 to 4330)	-18.8 (-26.3 to -10.4)	55.2 (47.1 to 62.1)	29.2 (24.5 to 35.1)	-46.9 (-57.2 to -33.6)

		Streptococcus pneumoniae	44000 (38000 to 50300)	15500 (13300 to 18000)	-64.7 (-67.8 to -61.1)	220 (187 to 247)	50.5 (42.4 to 60.9)	-76.9 (-81.3 to -71.2)
		Other viruses	19800 (17000 to 22600)	16000 (13700 to 18500)	-19.2 (-26.3 to -11.1)	38.8 (33.4 to 43.6)	20.4 (17.1 to 24.6)	-47.2 (-57.3 to -33.8)
		Other bacterial pathogens	4730 (3950 to 5560)	1880 (1530 to 2260)	-60.1 (-64.0 to -55.8)	22.5 (19 to 26.1)	5.86 (4.73 to 7.4)	-73.9 (-79.0 to -67.5)
	50-69 years	*Acinetobacter baumannii*	839 (708 to 977)	462 (394 to 539)	-44.8 (-51.0 to -37.8)	29.3 (23.9 to 36.6)	12 (9.41 to 14.9)	-58.7 (-69.6 to -45.8)
		Chlamydia spp.	2230 (1940 to 2550)	2170 (1920 to 2450)	-2.4 (-12.2 to 9.1)	19.3 (16.1 to 23.6)	14 (11.2 to 17.3)	-27.0 (-46.3 to -6.7)
		Enterobacter spp.	331 (280 to 390)	865 (746 to 987)	162.4 (125.8 to 203.0)	11 (9 to 13.6)	21.3 (16.8 to 26.2)	96.1 (42.4 to 159.6)
		Escherichia coli	954 (830 to 1100)	1830 (1630 to 2040)	92.2 (72.1 to 114.8)	32.7 (27.3 to 40.1)	46.6 (37.6 to 57)	43.7 (5.9 to 85.9)
		Fungus	915 (779 to 1070)	1610 (1410 to 1840)	76.4 (55.7 to 99.4)	31 (25.5 to 37.4)	40.4 (32.3 to 50.2)	31.8 (-3.9 to 73.2)
		Group B Streptococcus	764 (650 to 888)	1610 (1420 to 1810)	110.8 (87.5 to 138.4)	18.6 (15.3 to 22.8)	29 (23.1 to 35.8)	57.6 (15.0 to 103.0)
		Haemophilus influenzae	2620 (2320 to 2940)	5440 (4890 to 6020)	107.9 (87.0 to 131.0)	34.3 (29 to 41)	35.4 (28.4 to 43.3)	4.1 (-22.5 to 34.5)
		Influenza	3210 (2850 to 3570)	1.98 (1.76 to 2.23)	-99.9 (-99.9 to -99.9)	82.2 (69.8 to 98.3)	0.04 (0.03 to 0.05)	-100.0 (-100.0 to -99.9)
		Klebsiella pneumoniae	1960 (1740 to 2180)	3080 (2760 to 3400)	56.9 (43.3 to 73.9)	67 (56.9 to 80.2)	77.9 (62.9 to 95)	17.3 (-13.8 to 51.3)
		Legionella spp.	1780 (1550 to 2040)	5740 (5140 to 6370)	222.6 (189.1 to 261.4)	41.9 (34.8 to 51)	100 (80.5 to 122)	141.1 (79.6 to 211.5)
		Mycoplasma spp.	2670 (2360 to 2990)	4630 (4140 to 5150)	73.9 (58.9 to 93.2)	24.2 (20.1 to 29.3)	31.2 (25 to 38.5)	30.0 (-4.8 to 68.0)
		Polymicrobial	128 (83.3 to 194)	164 (93.3 to 280)	27.6 (6.3 to 52.7)	4.44 (2.82 to 6.83)	4.25 (2.3 to 7.24)	-4.6 (-34.6 to 28.7)
		Pseudomonas aeruginosa	1990 (1740 to 2230)	6150 (5490 to 6810)	209.7 (182.4 to 242.7)	47.1 (39.6 to 56.2)	108 (87.3 to 132)	131.5 (70.9 to 197.7)
		Respiratory syncytial virus	625 (544 to 709)	0.52 (0.46 to 0.6)	-99.9 (-99.9 to -99.9)	7.29 (6.12 to 8.77)	0 (0 to 0.01)	-99.9 (-100.0 to -99.9)
		Staphylococcus aureus	2760 (2450 to 3070)	7790 (7030 to 8630)	182.7 (158.8 to 211.9)	134 (115 to 160)	280 (228 to 342)	111.4 (55.7 to 171.7)
		Streptococcus pneumoniae	13000 (11600 to 14400)	16300 (14700 to 18000)	25.0 (15.4 to 37.9)	263 (225 to 314)	244 (198 to 296)	-6.5 (-31.2 to 20.0)
		Other viruses	5130 (4560 to 5690)	14600 (13200 to 16100)	185.5 (162.0 to 215.6)	40.7 (34.7 to 48.7)	86.1 (69.7 to 105)	113.4 (56.4 to 173.6)
		Other bacterial pathogens	3430 (2970 to 3970)	4740 (4080 to 5470)	38.3 (25.0 to 54.6)	66 (55.2 to 80.7)	68 (53 to 85.5)	3.7 (-22.7 to 33.5)
	70+ years	*Acinetobacter baumannii*	708 (601 to 833)	977 (827 to 1130)	38.4 (23.2 to 55.7)	89.9 (72.1 to 114)	119 (87.1 to 148)	33.6 (-4.1 to 65.4)
		Chlamydia spp.	2240 (1960 to 2570)	5470 (4850 to 6140)	144.6 (120.3 to 172.3)	70.7 (58.4 to 89)	165 (127 to 199)	136.1 (70.4 to 195.4)
		Enterobacter spp.	272 (228 to 323)	1780 (1550 to 2020)	557.4 (471.5 to 652.6)	32.8 (26.2 to 41.8)	206 (155 to 253)	534.5 (349.6 to 688.9)
		Escherichia coli	989 (862 to 1140)	4750 (4270 to 5300)	381.6 (333.1 to 435.4)	123 (102 to 157)	568 (434 to 693)	364.9 (237.5 to 471.7)

		Fungus	431 (363 to 504)	1900 (1660 to 2150)	341.9 (290.9 to 397.7)	52.9 (42.7 to 66.3)	224 (170 to 272)	326.5 (209.7 to 432.9)
		Group B Streptococcus	592 (510 to 680)	3120 (2780 to 3470)	428.1 (370.0 to 491.7)	52.4 (42.7 to 65.8)	265 (203 to 320)	409.8 (266.4 to 531.0)
		Haemophilus influenzae	1990 (1770 to 2250)	10400 (9410 to 11500)	421.0 (372.3 to 476.2)	94.7 (79.1 to 119)	317 (246 to 379)	236.9 (145.7 to 315.2)
		Influenza	4130 (3670 to 4640)	6.38 (5.71 to 7.21)	-99.8 (-99.9 to -99.8)	383 (320 to 474)	0.54 (0.42 to 0.69)	-99.9 (-99.9 to -99.8)
		Klebsiella pneumoniae	1830 (1630 to 2050)	7170 (6510 to 7870)	293.2 (257.4 to 333.6)	226 (189 to 282)	852 (657 to 1020)	279.5 (174.8 to 364.7)
		Legionella spp.	1080 (942 to 1260)	8730 (7880 to 9700)	708.3 (623.2 to 798.2)	92.3 (75.6 to 117)	714 (553 to 856)	680.4 (467.3 to 865.4)
		Mycoplasma spp.	1910 (1690 to 2160)	8290 (7480 to 9220)	335.8 (295.5 to 381.0)	62.7 (51.8 to 79.5)	262 (200 to 315)	320.7 (209.8 to 414.8)
		Polymicrobial	124 (79.9 to 190)	399 (225 to 693)	219.6 (164.8 to 279.9)	15.6 (9.55 to 24.4)	48.3 (25.9 to 83.5)	208.6 (117.6 to 298.6)
		Pseudomonas aeruginosa	1750 (1550 to 1990)	13600 (12200 to 15000)	676.0 (607.6 to 754.7)	150 (126 to 186)	1120 (855 to 1340)	649.1 (446.5 to 830.2)
		Respiratory syncytial virus	495 (430 to 566)	1.04 (0.92 to 1.17)	-99.8 (-99.8 to -99.8)	21 (17.3 to 26.1)	0.04 (0.03 to 0.05)	-99.8 (-99.9 to -99.8)
		Staphylococcus aureus	3020 (2680 to 3380)	21300 (19400 to 23500)	608.4 (547.6 to 671.5)	531 (443 to 672)	3600 (2790 to 4330)	583.9 (402.8 to 737.3)
		Streptococcus pneumoniae	11300 (10200 to 12700)	35400 (32200 to 38900)	213.3 (186.9 to 242.8)	829 (691 to 1040)	2490 (1930 to 3010)	202.4 (122.2 to 269.6)
		Other viruses	4400 (3930 to 4950)	31400 (28700 to 34500)	615.3 (553.6 to 682.6)	127 (106 to 159)	869 (674 to 1040)	590.5 (407.5 to 739.7)
		Other bacterial pathogens	3880 (3380 to 4450)	13400 (11700 to 15600)	246.5 (213.1 to 284.7)	274 (223 to 347)	913 (684 to 1150)	235.4 (142.7 to 310.8)
Singapore	All Ages	*Acinetobacter baumannii*	333 (293 to 379)	320 (278 to 366)	-3.9 (-11.8 to 4.3)	23.4 (20.2 to 26.6)	28.1 (23 to 32.6)	20.0 (5.6 to 33.1)
		Chlamydia spp.	1570 (1450 to 1700)	1960 (1800 to 2150)	24.6 (14.9 to 34.1)	22.6 (20.4 to 24.6)	39.9 (33.6 to 44.3)	76.8 (56.0 to 94.8)
		Enterobacter spp.	226 (201 to 254)	654 (584 to 728)	190.2 (163.1 to 216.3)	13.5 (11.8 to 15.3)	52.2 (43 to 59.3)	285.9 (236.2 to 331.1)
		Escherichia coli	628 (573 to 684)	1600 (1480 to 1750)	155.6 (135.4 to 175.6)	42.8 (38.8 to 46.8)	142 (119 to 158)	232.0 (193.5 to 264.2)
		Fungus	443 (396 to 493)	919 (832 to 1010)	107.9 (89.6 to 126.9)	23.3 (20.8 to 26.1)	62 (51.8 to 70.6)	165.9 (133.0 to 194.4)
		Group B Streptococcus	571 (521 to 631)	1260 (1160 to 1370)	121.2 (102.8 to 140.5)	21.7 (19.5 to 23.9)	67.5 (56.3 to 75.1)	210.8 (175.6 to 243.4)
		Haemophilus influenzae	2670 (2490 to 2870)	4650 (4330 to 4990)	74.1 (61.9 to 87.1)	43.5 (40.5 to 46.2)	81.9 (69.4 to 89.8)	88.2 (66.3 to 107.0)
		Influenza	3430 (3240 to 3650)	49 (5.49 to 170)	-98.6 (-99.8 to -95.0)	145 (135 to 154)	2.66 (0.29 to 9.31)	-98.2 (-99.8 to -93.5)
		Klebsiella pneumoniae	1530 (1450 to 1630)	2540 (2370 to 2740)	65.8 (54.9 to 77.6)	83.1 (77 to 88.3)	205 (173 to 225)	147.1 (119.3 to 169.0)
		Legionella spp.	1210 (1110 to 1310)	3570 (3310 to 3860)	195.8 (172.0 to 220.3)	48.4 (43.9 to 53.1)	184 (155 to 203)	280.1 (235.1 to 319.8)

		Mycoplasma spp.	3290 (3050 to 3560)	4750 (4420 to 5100)	44.6 (35.6 to 53.6)	31.4 (29.1 to 33.7)	66.5 (56 to 73)	112.0 (88.0 to 133.1)
		Polymicrobial	90.2 (62.1 to 130)	147 (87.6 to 242)	62.0 (31.4 to 94.8)	5.36 (3.54 to 8.29)	12.3 (7.04 to 20.8)	125.9 (84.1 to 166.1)
		Pseudomonas aeruginosa	1650 (1540 to 1780)	4870 (4530 to 5260)	195.0 (174.7 to 216.2)	68.7 (63.6 to 74)	271 (228 to 297)	293.9 (248.7 to 330.2)
		Respiratory syncytial virus	2680 (2430 to 2950)	23.6 (2.5 to 82.4)	-99.1 (-99.9 to -96.9)	17.8 (16.7 to 19)	0.2 (0.02 to 0.71)	-98.9 (-99.9 to -95.9)
		Staphylococcus aureus	2280 (2160 to 2420)	7180 (6720 to 7750)	214.6 (194.5 to 234.7)	208 (194 to 220)	887 (743 to 971)	325.4 (277.9 to 362.3)
		Streptococcus pneumoniae	15900 (15000 to 16900)	14600 (13700 to 15600)	-8.1 (-13.4 to -2.6)	364 (341 to 382)	573 (487 to 626)	57.5 (39.6 to 71.6)
		Other viruses	9920 (9310 to 10600)	15500 (14500 to 16600)	56.2 (46.7 to 65.8)	73.1 (68.7 to 76.9)	210 (178 to 230)	187.2 (153.8 to 212.0)
		Other bacterial pathogens	2130 (1900 to 2340)	4080 (3600 to 4650)	91.8 (78.1 to 105.9)	89.9 (79.4 to 101)	216 (174 to 251)	139.6 (111.5 to 164.0)
	Under 5	*Acinetobacter baumannii*	23.9 (15.7 to 36.9)	7.89 (4.97 to 12.5)	-67.0 (-71.8 to -61.7)	0.5 (0.29 to 0.82)	0.08 (0.04 to 0.14)	-84.0 (-88.3 to -79.2)
		Chlamydia spp.	264 (228 to 303)	128 (107 to 152)	-51.5 (-58.7 to -43.2)	0.78 (0.68 to 0.88)	0.11 (0.09 to 0.13)	-85.9 (-89.0 to -82.4)
		Enterobacter spp.	35.7 (26.9 to 45.4)	34.4 (26.1 to 44.9)	-3.4 (-20.5 to 17.2)	0.44 (0.31 to 0.62)	0.11 (0.08 to 0.16)	-74.2 (-80.3 to -67.1)
		Escherichia coli	108 (91.9 to 126)	104 (85.6 to 124)	-3.4 (-19.4 to 14.4)	1.26 (1.09 to 1.45)	0.3 (0.24 to 0.36)	-76.3 (-81.0 to -71.1)
		Fungus	18.9 (13.4 to 25.7)	15.2 (10.8 to 21.2)	-19.3 (-33.7 to -2.8)	0.15 (0.1 to 0.2)	0.03 (0.02 to 0.04)	-81.6 (-85.8 to -76.8)
		Group B Streptococcus	104 (87.7 to 122)	84.8 (70.3 to 103)	-18.0 (-31.0 to -5.4)	1.02 (0.9 to 1.17)	0.22 (0.18 to 0.28)	-77.9 (-82.7 to -72.6)
		Haemophilus influenzae	856 (747 to 983)	602 (506 to 720)	-29.5 (-41.1 to -16.4)	3.02 (2.67 to 3.39)	0.31 (0.25 to 0.37)	-89.7 (-92.0 to -87.1)
		Influenza	692 (614 to 782)	3.09 (0.32 to 10.6)	-99.6 (-100.0 to -98.5)	4.57 (4.08 to 5.11)	0 (0 to 0.01)	-99.9 (-100.0 to -99.7)
		Klebsiella pneumoniae	373 (327 to 424)	180 (151 to 215)	-51.7 (-58.4 to -43.8)	3.45 (3.06 to 3.85)	0.45 (0.37 to 0.54)	-86.9 (-89.7 to -84.0)
		Legionella spp.	51 (37.7 to 67.3)	48.5 (34.1 to 66.8)	-5.0 (-20.4 to 12.1)	0.52 (0.4 to 0.68)	0.14 (0.1 to 0.19)	-72.6 (-79.1 to -65.4)
		Mycoplasma spp.	473 (411 to 542)	551 (464 to 660)	16.7 (-0.6 to 35.5)	1.13 (0.99 to 1.28)	0.29 (0.24 to 0.35)	-74.1 (-79.5 to -68.1)
		Polymicrobial	25.8 (14.7 to 41.4)	14.3 (7.8 to 23.5)	-44.5 (-52.9 to -34.8)	0.64 (0.35 to 1.04)	0.17 (0.08 to 0.31)	-73.5 (-80.6 to -65.4)
		Pseudomonas aeruginosa	269 (234 to 309)	364 (307 to 437)	35.5 (15.1 to 58.0)	1.97 (1.74 to 2.23)	0.62 (0.5 to 0.74)	-68.8 (-75.3 to -61.5)
		Respiratory syncytial virus	2020 (1790 to 2260)	14.1 (1.49 to 48.2)	-99.3 (-99.9 to -97.6)	6.68 (6.02 to 7.36)	0.01 (0 to 0.03)	-99.9 (-100.0 to -99.5)
		Staphylococcus aureus	299 (265 to 337)	322 (271 to 385)	7.6 (-6.5 to 22.7)	3.85 (3.45 to 4.28)	0.98 (0.81 to 1.17)	-74.4 (-79.6 to -68.8)
		Streptococcus pneumoniae	4370 (3870 to 4880)	1270 (1070 to 1530)	-70.9 (-74.8 to -67.0)	20.9 (18.9 to 22.9)	1.38 (1.13 to 1.65)	-93.4 (-94.7 to -91.9)

		Other viruses	3960 (3520 to 4440)	2540 (2160 to 3030)	-35.8 (-44.3 to -27.5)	7.68 (6.91 to 8.44)	1.21 (1 to 1.44)	-84.2 (-87.3 to -80.8)
		Other bacterial pathogens	112 (88 to 138)	73.9 (57.1 to 94.5)	-33.9 (-45.2 to -21.5)	0.68 (0.53 to 0.88)	0.12 (0.08 to 0.16)	-83.0 (-86.8 to -78.9)
	5-14 years	*Acinetobacter baumannii*	32.9 (25.5 to 41.2)	14.3 (11 to 18.5)	-56.5 (-61.8 to -50.3)	0.2 (0.16 to 0.23)	0.02 (0.02 to 0.03)	-88.2 (-89.8 to -86.3)
		Chlamydia spp.	180 (145 to 222)	118 (91.6 to 150)	-34.2 (-42.0 to -25.2)	0.26 (0.23 to 0.3)	0.05 (0.04 to 0.05)	-82.1 (-84.6 to -79.3)
		Enterobacter spp.	20.8 (16.6 to 26.2)	29.6 (22.3 to 38.3)	42.9 (24.0 to 63.3)	0.12 (0.1 to 0.14)	0.05 (0.04 to 0.05)	-61.2 (-66.9 to -54.5)
		Escherichia coli	48.7 (39.6 to 60)	58.2 (45.1 to 73.3)	19.6 (4.9 to 36.0)	0.28 (0.25 to 0.32)	0.09 (0.08 to 0.1)	-67.5 (-72.1 to -62.5)
		Fungus	58.5 (46.9 to 72)	62.1 (48.1 to 79.9)	6.2 (-6.8 to 20.5)	0.34 (0.29 to 0.38)	0.1 (0.08 to 0.11)	-71.1 (-75.2 to -66.3)
		Group B Streptococcus	64.1 (51.3 to 78.7)	77.4 (59.8 to 98.3)	20.9 (5.7 to 37.3)	0.26 (0.23 to 0.3)	0.09 (0.08 to 0.1)	-67.2 (-71.9 to -61.9)
		Haemophilus influenzae	273 (224 to 332)	302 (235 to 376)	10.8 (-2.7 to 26.7)	0.6 (0.55 to 0.66)	0.12 (0.11 to 0.14)	-79.5 (-82.4 to -76.3)
		Influenza	378 (312 to 461)	2.9 (0.3 to 10.1)	-99.2 (-99.9 to -97.4)	1.64 (1.5 to 1.78)	0 (0 to 0.01)	-99.8 (-100.0 to -99.4)
		Klebsiella pneumoniae	143 (117 to 175)	129 (101 to 161)	-9.5 (-20.2 to 2.7)	0.82 (0.75 to 0.9)	0.2 (0.18 to 0.23)	-75.4 (-78.5 to -71.7)
		Legionella spp.	153 (125 to 187)	224 (176 to 280)	46.5 (28.1 to 67.9)	0.61 (0.54 to 0.68)	0.24 (0.21 to 0.28)	-60.2 (-65.6 to -54.1)
		Mycoplasma spp.	592 (481 to 721)	562 (437 to 699)	-5.0 (-15.7 to 7.6)	0.91 (0.82 to 0.99)	0.23 (0.2 to 0.26)	-74.2 (-77.7 to -70.0)
		Polymicrobial	7.31 (4.43 to 11.4)	6.61 (3.66 to 10.9)	-10.4 (-23.6 to 5.3)	0.04 (0.03 to 0.07)	0.01 (0.01 to 0.02)	-75.7 (-79.6 to -71.1)
		Pseudomonas aeruginosa	163 (132 to 201)	240 (188 to 298)	47.5 (29.7 to 67.3)	0.65 (0.59 to 0.72)	0.26 (0.23 to 0.3)	-59.9 (-65.2 to -53.8)
		Respiratory syncytial virus	121 (98.5 to 147)	0.98 (0.1 to 3.42)	-99.2 (-99.9 to -97.2)	0.24 (0.21 to 0.27)	0 (0 to 0)	-99.8 (-100.0 to -99.3)
		Staphylococcus aureus	234 (192 to 286)	355 (277 to 440)	51.6 (34.1 to 71.1)	1.92 (1.76 to 2.07)	0.79 (0.7 to 0.89)	-58.8 (-64.1 to -52.7)
		Streptococcus pneumoniae	2090 (1720 to 2530)	1310 (1020 to 1630)	-37.3 (-44.5 to -29.1)	7.15 (6.54 to 7.72)	1.22 (1.08 to 1.37)	-83.0 (-85.1 to -80.4)
		Other viruses	1140 (945 to 1390)	1410 (1110 to 1750)	23.5 (9.1 to 39.5)	1.54 (1.4 to 1.66)	0.52 (0.46 to 0.58)	-66.5 (-70.9 to -61.6)
		Other bacterial pathogens	207 (166 to 257)	174 (133 to 220)	-16.4 (-26.6 to -4.7)	0.68 (0.59 to 0.77)	0.15 (0.13 to 0.18)	-77.2 (-80.3 to -73.7)
	15-49 years	*Acinetobacter baumannii*	73.2 (61.3 to 86.6)	29.5 (24.3 to 35)	-59.7 (-64.1 to -54.9)	1.19 (1.02 to 1.38)	0.23 (0.2 to 0.27)	-80.3 (-82.4 to -78.0)
		Chlamydia spp.	401 (351 to 456)	244 (214 to 277)	-39.0 (-45.5 to -33.0)	1.62 (1.46 to 1.8)	0.48 (0.44 to 0.54)	-70.2 (-73.3 to -66.5)
		Enterobacter spp.	46.2 (39.4 to 54.1)	61.1 (51.8 to 72.3)	32.5 (15.6 to 48.7)	0.71 (0.62 to 0.82)	0.46 (0.4 to 0.53)	-35.2 (-42.9 to -26.7)
		Escherichia coli	108 (95.7 to 122)	120 (104 to 136)	10.9 (-1.3 to 22.6)	1.73 (1.56 to 1.91)	0.94 (0.85 to 1.04)	-45.8 (-51.5 to -39.9)

		Fungus	130 (114 to 150)	128 (110 to 147)	-1.5 (-12.3 to 10.8)	2.05 (1.82 to 2.3)	0.99 (0.87 to 1.11)	-51.8 (-57.5 to -46.1)
		Group B Streptococcus	143 (125 to 165)	160 (139 to 184)	12.0 (-1.0 to 25.0)	1.62 (1.44 to 1.82)	0.88 (0.79 to 0.99)	-45.2 (-51.7 to -38.3)
		Haemophilus influenzae	607 (545 to 673)	623 (548 to 699)	2.7 (-8.2 to 14.0)	3.7 (3.42 to 3.99)	1.26 (1.16 to 1.38)	-65.8 (-69.2 to -62.0)
		Influenza	843 (762 to 931)	5.97 (0.65 to 20.8)	-99.3 (-99.9 to -97.5)	10 (9.36 to 10.8)	0.03 (0 to 0.1)	-99.7 (-100.0 to -99.0)
		Klebsiella pneumoniae	317 (285 to 351)	266 (235 to 297)	-16.1 (-25.2 to -7.4)	5.04 (4.68 to 5.41)	2.07 (1.89 to 2.24)	-59.0 (-62.9 to -54.6)
		Legionella spp.	341 (301 to 386)	463 (410 to 520)	35.8 (20.7 to 51.5)	3.73 (3.38 to 4.11)	2.47 (2.25 to 2.71)	-33.6 (-40.4 to -26.1)
		Mycoplasma spp.	1320 (1190 to 1480)	1160 (1020 to 1300)	-12.0 (-21.5 to -2.8)	5.56 (5.13 to 6.04)	2.39 (2.19 to 2.6)	-56.9 (-61.0 to -52.1)
		Polymicrobial	16.3 (9.84 to 25.7)	13.6 (7.58 to 23)	-16.9 (-29.0 to -5.0)	0.26 (0.16 to 0.42)	0.11 (0.06 to 0.18)	-59.4 (-65.1 to -53.4)
		Pseudomonas aeruginosa	363 (325 to 404)	496 (434 to 558)	36.7 (22.7 to 51.3)	4 (3.68 to 4.35)	2.67 (2.45 to 2.9)	-33.1 (-39.4 to -26.0)
		Respiratory syncytial virus	270 (240 to 302)	2.02 (0.22 to 7.02)	-99.3 (-99.9 to -97.4)	1.47 (1.34 to 1.6)	0 (0 to 0.02)	-99.7 (-100.0 to -98.9)
		Staphylococcus aureus	521 (472 to 573)	732 (643 to 818)	40.6 (25.8 to 54.8)	11.8 (11 to 12.6)	8.08 (7.44 to 8.72)	-31.2 (-37.7 to -23.8)
		Streptococcus pneumoniae	4660 (4230 to 5100)	2710 (2380 to 3010)	-41.9 (-47.9 to -36.2)	43.8 (41.1 to 46.5)	12.4 (11.4 to 13.5)	-71.6 (-74.2 to -68.7)
		Other viruses	2550 (2310 to 2800)	2910 (2560 to 3250)	14.4 (2.8 to 25.8)	9.41 (8.84 to 10)	5.26 (4.85 to 5.69)	-44.0 (-49.3 to -38.2)
		Other bacterial pathogens	462 (399 to 527)	358 (301 to 420)	-22.5 (-30.8 to -14.2)	4.14 (3.68 to 4.66)	1.57 (1.35 to 1.83)	-62.0 (-66.0 to -57.8)
	50-69 years	*Acinetobacter baumannii*	92.9 (80.2 to 108)	98.3 (83.8 to 115)	5.9 (-5.2 to 18.0)	4.87 (4.22 to 5.59)	3.53 (2.99 to 4.12)	-27.3 (-36.7 to -16.7)
		Chlamydia spp.	301 (267 to 335)	479 (424 to 537)	59.3 (43.2 to 74.7)	3.92 (3.5 to 4.37)	4.27 (3.78 to 4.83)	9.3 (-5.0 to 24.3)
		Enterobacter spp.	56.9 (49.5 to 65.1)	197 (170 to 223)	246.4 (205.6 to 286.5)	2.83 (2.44 to 3.25)	6.7 (5.82 to 7.62)	137.6 (105.1 to 172.0)
		Escherichia coli	147 (131 to 164)	423 (378 to 470)	187.1 (158.3 to 216.3)	7.58 (6.77 to 8.42)	14.9 (13.3 to 16.5)	96.9 (72.8 to 123.4)
		Fungus	141 (125 to 159)	363 (320 to 407)	157.7 (128.6 to 186.3)	7.15 (6.29 to 8.12)	12.6 (11.1 to 14.4)	76.8 (51.9 to 102.7)
		Group B Streptococcus	124 (110 to 141)	363 (322 to 407)	192.0 (160.1 to 223.6)	4.54 (3.97 to 5.13)	9.07 (7.99 to 10.2)	100.3 (74.5 to 130.3)
		Haemophilus influenzae	452 (410 to 491)	1220 (1090 to 1340)	170.2 (142.0 to 195.3)	8.86 (8.1 to 9.72)	11.2 (10.1 to 12.3)	26.2 (10.4 to 44.6)
		Influenza	540 (492 to 588)	10.2 (1.1 to 36.3)	-98.1 (-99.8 to -93.5)	20.7 (18.9 to 22.7)	0.23 (0.03 to 0.79)	-98.9 (-99.9 to -96.1)
		Klebsiella pneumoniae	302 (276 to 327)	675 (606 to 740)	123.5 (102.6 to 144.3)	15.5 (14.1 to 17)	23.6 (21.3 to 26.1)	53.3 (35.2 to 73.2)
		Legionella spp.	357 (320 to 396)	1260 (1130 to 1390)	254.2 (217.8 to 291.4)	12.6 (11.3 to 14)	30.4 (27.2 to 33.9)	143.0 (112.7 to 175.1)

		Mycoplasma spp.	450 (405 to 492)	1000 (894 to 1110)	123.6 (101.4 to 145.1)	6.1 (5.52 to 6.74)	9.34 (8.35 to 10.4)	53.4 (34.4 to 74.5)
		Polymicrobial	17.2 (10.8 to 27.2)	37.8 (21.6 to 62.8)	117.8 (89.5 to 148.8)	0.9 (0.56 to 1.46)	1.35 (0.77 to 2.25)	49.4 (26.1 to 75.6)
		Pseudomonas aeruginosa	383 (347 to 421)	1350 (1200 to 1490)	252.0 (218.5 to 286.4)	13.6 (12.4 to 15)	32.7 (29.5 to 36.1)	141.5 (112.0 to 175.2)
		Respiratory syncytial virus	128 (113 to 142)	2.48 (0.26 to 8.94)	-98.1 (-99.8 to -93.3)	2.24 (1.99 to 2.52)	0.03 (0 to 0.09)	-98.9 (-99.9 to -96.0)
		Staphylococcus aureus	483 (440 to 523)	1780 (1600 to 1960)	268.5 (232.4 to 302.5)	35.1 (32.1 to 38.2)	88.6 (79.6 to 97.5)	152.8 (123.2 to 186.4)
		Streptococcus pneumoniae	2130 (1950 to 2310)	3340 (2990 to 3650)	56.5 (41.8 to 70.9)	64.5 (59.1 to 69.9)	69.1 (62.2 to 76.2)	7.4 (-5.1 to 22.0)
		Other viruses	1020 (935 to 1110)	3120 (2790 to 3430)	205.5 (176.0 to 234.3)	12.1 (11.1 to 13.2)	25.4 (22.9 to 28.1)	109.6 (83.8 to 137.4)
		Other bacterial pathogens	519 (455 to 587)	1050 (891 to 1200)	101.9 (82.2 to 121.5)	15 (13.2 to 17)	20.7 (17.7 to 24.1)	38.8 (21.5 to 58.9)
	70+ years	*Acinetobacter baumannii*	111 (95.1 to 127)	170 (145 to 198)	54.0 (40.2 to 68.3)	16.7 (14.1 to 19.1)	24.2 (19.5 to 28.3)	45.4 (27.4 to 62.2)
		Chlamydia spp.	427 (385 to 471)	989 (892 to 1100)	131.6 (111.2 to 153.3)	16 (14.2 to 17.7)	35 (29 to 39.2)	118.8 (94.4 to 141.5)
		Enterobacter spp.	66.1 (57.7 to 75.3)	332 (292 to 376)	403.5 (350.1 to 449.9)	9.44 (8.13 to 10.9)	44.9 (36.3 to 51.3)	375.5 (315.5 to 432.5)
		Escherichia coli	216 (192 to 240)	899 (817 to 999)	317.3 (280.0 to 353.8)	31.9 (28.7 to 35.5)	126 (103 to 141)	294.1 (248.5 to 333.3)
		Fungus	93.6 (82.2 to 106)	350 (311 to 398)	274.6 (236.4 to 309.2)	13.7 (12 to 15.5)	48.3 (39 to 55.6)	253.8 (210.6 to 292.1)
		Group B Streptococcus	136 (122 to 152)	576 (520 to 641)	324.5 (283.9 to 364.7)	14.3 (12.5 to 16)	57.2 (46.9 to 64.1)	300.9 (253.3 to 343.6)
		Haemophilus influenzae	485 (447 to 523)	1900 (1740 to 2080)	292.8 (260.5 to 323.6)	27.4 (25 to 29.4)	69.1 (57 to 76.5)	152.5 (124.5 to 177.7)
		Influenza	978 (910 to 1050)	26.8 (2.97 to 94.4)	-97.3 (-99.7 to -90.5)	108 (98.9 to 116)	2.39 (0.26 to 8.26)	-97.8 (-99.8 to -92.1)
		Klebsiella pneumoniae	397 (367 to 426)	1290 (1190 to 1410)	224.8 (198.9 to 251.3)	58.4 (53 to 62.6)	179 (147 to 197)	206.8 (173.2 to 235.6)
		Legionella spp.	306 (274 to 339)	1570 (1430 to 1740)	414.9 (365.6 to 462.0)	30.9 (27.6 to 34.2)	150 (124 to 168)	386.3 (328.5 to 437.3)
		Mycoplasma spp.	453 (413 to 494)	1470 (1340 to 1620)	225.0 (198.2 to 251.2)	17.7 (15.9 to 19.2)	54.3 (44.7 to 60.3)	206.9 (172.0 to 237.7)
		Polymicrobial	23.5 (14.4 to 38)	74.9 (41.6 to 129)	216.6 (177.9 to 257.7)	3.52 (2.15 to 5.73)	10.6 (5.96 to 18.2)	199.1 (155.6 to 241.5)
		Pseudomonas aeruginosa	475 (435 to 517)	2430 (2230 to 2680)	411.6 (366.8 to 453.1)	48.5 (44.2 to 52.8)	234 (193 to 259)	383.2 (327.4 to 430.5)
		Respiratory syncytial virus	143 (129 to 159)	4.03 (0.44 to 14.1)	-97.2 (-99.7 to -90.2)	7.21 (6.4 to 7.97)	0.16 (0.02 to 0.57)	-97.7 (-99.8 to -92.0)
		Staphylococcus aureus	745 (694 to 800)	3990 (3680 to 4360)	435.7 (394.0 to 473.3)	156 (142 to 166)	788 (649 to 868)	405.9 (349.9 to 450.6)
		Streptococcus pneumoniae	2620 (2440 to 2790)	5950 (5490 to 6510)	127.5 (109.7 to 143.3)	227 (208 to 242)	489 (405 to 540)	114.9 (91.9 to 134.7)

		Other viruses	1240 (1160 to 1320)	5490 (5070 to 6010)	344.0 (309.5 to 375.9)	42.3 (38.6 to 45.2)	178 (147 to 196)	319.4 (275.3 to 356.3)
		Other bacterial pathogens	827 (729 to 928)	2430 (2120 to 2790)	193.5 (169.1 to 216.3)	69.4 (60.6 to 78.4)	193 (153 to 226)	177.7 (145.0 to 205.8)
High-income North America	All Ages	*Acinetobacter baumannii*	42900 (36800 to 49700)	29500 (26000 to 33300)	-31.0 (-37.7 to -24.1)	983 (829 to 1140)	507 (418 to 584)	-48.4 (-53.5 to -43.1)
		Chlamydia spp.	198000 (180000 to 215000)	170000 (155000 to 187000)	-13.9 (-21.7 to -5.2)	1110 (960 to 1210)	712 (602 to 797)	-35.6 (-41.2 to -29.2)
		Enterobacter spp.	39100 (34400 to 44100)	74400 (67500 to 81900)	90.5 (72.9 to 110.8)	833 (709 to 946)	1190 (1000 to 1330)	43.3 (29.7 to 56.7)
		Escherichia coli	104000 (94700 to 116000)	190000 (176000 to 204000)	82.5 (67.8 to 97.0)	2440 (2130 to 2680)	3270 (2770 to 3580)	33.9 (23.3 to 44.3)
		Fungus	66900 (59600 to 74600)	97900 (87600 to 109000)	46.7 (33.0 to 61.6)	1230 (1070 to 1390)	1430 (1230 to 1600)	16.1 (4.8 to 27.5)
		Group B Streptococcus	83000 (75200 to 91900)	132000 (120000 to 145000)	59.8 (42.9 to 76.8)	1230 (1060 to 1380)	1500 (1280 to 1670)	21.4 (9.9 to 33.7)
		Haemophilus influenzae	332000 (311000 to 355000)	382000 (354000 to 412000)	14.8 (8.3 to 21.2)	2460 (2180 to 2610)	1540 (1320 to 1680)	-37.1 (-40.9 to -33.5)
		Influenza	519000 (486000 to 557000)	163000 (79700 to 282000)	-68.7 (-84.7 to -45.1)	8900 (7780 to 9520)	1810 (860 to 3220)	-79.7 (-90.2 to -63.8)
		Klebsiella pneumoniae	211000 (198000 to 226000)	233000 (216000 to 251000)	10.2 (4.1 to 16.2)	4600 (4060 to 4910)	3880 (3270 to 4230)	-15.6 (-20.2 to -11.3)
		Legionella spp.	239000 (217000 to 261000)	317000 (295000 to 341000)	33.2 (22.5 to 44.8)	3290 (2880 to 3610)	3380 (2900 to 3690)	2.9 (-5.6 to 12.1)
		Mycoplasma spp.	368000 (342000 to 395000)	338000 (315000 to 363000)	-7.9 (-13.9 to -1.4)	1710 (1520 to 1840)	1200 (1040 to 1300)	-29.7 (-34.7 to -24.7)
		Polymicrobial	11100 (6860 to 18200)	17000 (10100 to 28100)	51.8 (34.8 to 69.4)	255 (157 to 416)	288 (167 to 483)	12.5 (-0.5 to 24.9)
		Pseudomonas aeruginosa	307000 (284000 to 335000)	455000 (424000 to 489000)	48.1 (38.3 to 57.4)	4680 (4130 to 5060)	5160 (4370 to 5600)	10.3 (2.9 to 17.3)
		Respiratory syncytial virus	187000 (175000 to 203000)	34200 (16900 to 59000)	-81.8 (-90.8 to -68.1)	968 (870 to 1050)	114 (55.6 to 201)	-88.2 (-94.3 to -79.5)
		Staphylococcus aureus	409000 (383000 to 439000)	835000 (777000 to 900000)	104.3 (95.1 to 114.2)	13400 (11800 to 14200)	20300 (17200 to 22000)	51.9 (45.2 to 58.8)
		Streptococcus pneumoniae	1760000 (1660000 to 1870000)	802000 (751000 to 860000)	-54.3 (-56.3 to -52.2)	19900 (17800 to 21100)	7360 (6270 to 7950)	-63.1 (-65.0 to -61.5)
		Other viruses	1100000 (1040000 to 1170000)	995000 (931000 to 1060000)	-9.5 (-13.9 to -5.2)	4620 (4140 to 4890)	3430 (2940 to 3710)	-25.8 (-29.6 to -22.5)
		Other bacterial pathogens	374000 (329000 to 427000)	378000 (332000 to 431000)	1.2 (-6.5 to 9.6)	5080 (4270 to 5810)	3780 (3080 to 4390)	-25.7 (-31.7 to -19.6)
	Under 5	*Acinetobacter baumannii*	590 (368 to 936)	336 (197 to 548)	-43.4 (-50.6 to -35.8)	9.25 (5.29 to 15.3)	2.47 (1.35 to 4.21)	-73.4 (-76.9 to -69.9)
		Chlamydia spp.	6500 (5590 to 7520)	4090 (3430 to 4790)	-37.1 (-45.4 to -28.0)	15.1 (13.9 to 16.5)	4.57 (3.9 to 5.29)	-69.8 (-74.2 to -65.4)
		Enterobacter spp.	1110 (837 to 1430)	1340 (1030 to 1720)	21.5 (4.4 to 41.5)	10.5 (7.69 to 14.4)	5.42 (4.13 to 6.93)	-47.8 (-57.0 to -36.9)
		Escherichia coli	3020 (2560 to 3570)	4160 (3460 to 4910)	38.2 (19.2 to 56.9)	27.8 (25.2 to 30.9)	16.5 (14 to 19.1)	-40.6 (-49.6 to -30.6)

		Fungus	515 (363 to 711)	544 (388 to 748)	6.1 (-11.9 to 24.5)	3.34 (2.46 to 4.44)	1.85 (1.3 to 2.53)	-44.4 (-55.0 to -31.9)
		Group B Streptococcus	3030 (2550 to 3580)	3210 (2680 to 3830)	6.3 (-7.6 to 21.6)	22.6 (20.4 to 25.1)	10.1 (8.57 to 11.6)	-55.3 (-61.9 to -48.5)
		Haemophilus influenzae	25100 (21400 to 29300)	18300 (15500 to 21400)	-27.1 (-36.1 to -17.5)	70.6 (65.5 to 76.8)	16.7 (14.3 to 19.3)	-76.3 (-80.0 to -72.1)
		Influenza	20300 (17400 to 23600)	3580 (1740 to 6210)	-82.4 (-91.3 to -70.2)	109 (103 to 116)	8.43 (3.85 to 15.4)	-92.3 (-96.4 to -85.7)
		Klebsiella pneumoniae	9970 (8570 to 11600)	5850 (4960 to 6860)	-41.2 (-47.9 to -33.7)	75.2 (70.4 to 80.8)	22.5 (19.5 to 25.5)	-70.1 (-74.2 to -65.5)
		Legionella spp.	1740 (1290 to 2310)	1580 (1140 to 2130)	-9.2 (-20.6 to 2.6)	13.2 (10.5 to 16.4)	5.25 (4.04 to 6.66)	-60.2 (-65.7 to -53.4)
		Mycoplasma spp.	14000 (11900 to 16500)	15200 (13000 to 17800)	8.9 (-4.2 to 22.5)	26.7 (24.6 to 29.4)	14.4 (12.3 to 16.5)	-46.0 (-54.5 to -37.1)
		Polymicrobial	837 (448 to 1400)	641 (350 to 1070)	-23.1 (-32.5 to -12.9)	14.9 (7.63 to 25)	4.89 (2.5 to 8.3)	-67.1 (-71.4 to -62.6)
		Pseudomonas aeruginosa	9020 (7740 to 10600)	11800 (10000 to 13800)	31.2 (15.0 to 48.1)	51.7 (47.4 to 56.1)	31 (27 to 35)	-39.9 (-48.6 to -31.2)
		Respiratory syncytial virus	71400 (62300 to 81800)	11800 (5750 to 20600)	-83.4 (-91.6 to -71.7)	187 (178 to 196)	13.3 (6.19 to 24.1)	-92.9 (-96.6 to -87.1)
		Staphylococcus aureus	9190 (7910 to 10600)	12500 (10700 to 14500)	36.4 (22.6 to 50.5)	96.5 (90.9 to 102)	64.2 (55.6 to 73.1)	-33.4 (-42.7 to -24.1)
		Streptococcus pneumoniae	123000 (106000 to 141000)	25500 (21700 to 29600)	-79.3 (-81.2 to -77.3)	493 (472 to 515)	51.4 (44.7 to 58.5)	-89.6 (-91.0 to -88.0)
		Other viruses	132000 (114000 to 151000)	66800 (57100 to 77500)	-49.5 (-54.0 to -44.8)	212 (202 to 223)	55.7 (48.6 to 62.7)	-73.7 (-77.4 to -70.0)
		Other bacterial pathogens	2990 (2340 to 3760)	2290 (1800 to 2900)	-23.4 (-34.7 to -10.2)	14.7 (11.5 to 18.6)	5.43 (4.28 to 6.93)	-62.9 (-68.4 to -56.4)
	5-14 years	*Acinetobacter baumannii*	689 (494 to 939)	374 (290 to 481)	-45.4 (-53.3 to -37.0)	1.66 (1.42 to 1.93)	0.7 (0.6 to 0.8)	-58.0 (-62.9 to -52.4)
		Chlamydia spp.	4450 (3290 to 5920)	3270 (2560 to 4160)	-26.2 (-35.8 to -14.8)	2.67 (2.45 to 2.92)	1.52 (1.35 to 1.69)	-43.0 (-49.3 to -35.7)
		Enterobacter spp.	646 (464 to 884)	1010 (786 to 1290)	58.2 (37.3 to 84.6)	1.48 (1.29 to 1.68)	1.8 (1.59 to 2.02)	21.9 (8.2 to 36.5)
		Escherichia coli	1370 (994 to 1830)	2170 (1710 to 2750)	59.5 (38.3 to 81.6)	3.24 (2.97 to 3.51)	4 (3.67 to 4.36)	23.6 (10.5 to 36.2)
		Fungus	1640 (1210 to 2220)	1990 (1570 to 2550)	21.9 (4.8 to 41.8)	3.83 (3.47 to 4.23)	3.6 (3.18 to 4.07)	-6.0 (-17.4 to 5.8)
		Group B Streptococcus	1890 (1380 to 2520)	2590 (2050 to 3320)	37.9 (17.7 to 60.5)	3.18 (2.86 to 3.52)	3.37 (3.02 to 3.77)	6.3 (-6.4 to 19.4)
		Haemophilus influenzae	8360 (6260 to 11000)	7640 (6010 to 9620)	-8.1 (-18.7 to 3.5)	7.34 (6.96 to 7.72)	3.66 (3.35 to 3.97)	-50.1 (-54.5 to -45.8)
		Influenza	11500 (8650 to 15000)	2810 (1320 to 5060)	-75.5 (-87.9 to -56.4)	20.4 (19.5 to 21.2)	3.42 (1.58 to 6.11)	-83.2 (-92.2 to -70.1)
		Klebsiella pneumoniae	3990 (2970 to 5200)	3150 (2510 to 3920)	-20.7 (-29.1 to -11.3)	9.39 (8.94 to 9.83)	5.72 (5.28 to 6.12)	-39.1 (-43.9 to -34.5)
		Legionella spp.	5400 (4020 to 7170)	6020 (4780 to 7540)	12.1 (-2.5 to 27.7)	8.75 (8.1 to 9.48)	7.49 (6.89 to 8.11)	-14.2 (-23.2 to -5.1)
		Mycoplasma spp.	17800 (13200 to 23300)	16300 (12900 to 20500)	-8.3 (-18.8 to 3.7)	11.1 (10.5 to 11.7)	7.84 (7.22 to 8.44)	-29.5 (-35.8 to -23.0)

		Polymicrobial	182 (104 to 295)	231 (131 to 373)	27.4 (8.5 to 48.4)	0.44 (0.26 to 0.72)	0.43 (0.25 to 0.71)	-1.2 (-13.6 to 12.7)
		Pseudomonas aeruginosa	5600 (4170 to 7360)	7420 (5950 to 9240)	33.3 (17.8 to 50.1)	9.14 (8.59 to 9.73)	9.32 (8.65 to 9.97)	2.0 (-6.5 to 11.3)
		Respiratory syncytial virus	4430 (3330 to 5840)	763 (362 to 1400)	-82.7 (-91.4 to -69.4)	3.57 (3.32 to 3.83)	0.42 (0.2 to 0.76)	-88.2 (-94.4 to -79.0)
		Staphylococcus aureus	7400 (5550 to 9660)	12200 (9880 to 15200)	66.0 (48.8 to 85.3)	24.7 (23.8 to 25.6)	31.6 (29.4 to 33.6)	27.8 (18.9 to 37.0)
		Streptococcus pneumoniae	61200 (45900 to 79600)	21100 (16900 to 26200)	-65.3 (-68.9 to -61.5)	85.1 (82.6 to 87.7)	22.3 (20.8 to 23.8)	-73.7 (-75.7 to -71.9)
		Other viruses	40200 (30100 to 52300)	28400 (22900 to 35400)	-29.1 (-36.6 to -21.0)	22 (21.3 to 22.7)	11.8 (11 to 12.6)	-46.1 (-49.9 to -42.2)
		Other bacterial pathogens	5670 (4130 to 7600)	4770 (3690 to 6120)	-15.5 (-26.0 to -4.1)	7.53 (6.7 to 8.45)	4.91 (4.25 to 5.61)	-34.8 (-41.3 to -27.8)
	15-49 years	*Acinetobacter baumannii*	3800 (3080 to 4530)	1960 (1650 to 2310)	-48.2 (-54.4 to -41.9)	28.8 (24.6 to 33.4)	14 (12.1 to 15.9)	-51.3 (-56.6 to -45.7)
		Chlamydia spp.	24500 (21400 to 28100)	17100 (15000 to 19400)	-30.0 (-37.1 to -22.9)	46.3 (42.3 to 50.5)	30.4 (27.2 to 33.8)	-34.3 (-40.9 to -26.8)
		Enterobacter spp.	3550 (2990 to 4180)	5320 (4570 to 6120)	50.1 (34.4 to 68.1)	25.5 (22.2 to 29)	36 (31.9 to 40.2)	41.5 (25.9 to 57.9)
		Escherichia coli	7540 (6540 to 8610)	11400 (10100 to 12700)	51.3 (37.5 to 65.4)	56 (51.2 to 60.8)	80 (74.6 to 85.7)	42.9 (30.1 to 56.8)
		Fungus	9050 (7780 to 10400)	10400 (9010 to 12000)	15.6 (3.1 to 29.2)	66.3 (59.8 to 73.3)	72.1 (64.3 to 80.9)	8.9 (-2.7 to 21.2)
		Group B Streptococcus	10400 (8990 to 12200)	13600 (11800 to 15500)	30.8 (15.7 to 46.4)	54.9 (49.6 to 61)	67.5 (60.9 to 74.6)	23.2 (10.1 to 37.9)
		Haemophilus influenzae	46100 (40700 to 51900)	40100 (35800 to 44700)	-12.9 (-18.8 to -6.4)	129 (122 to 135)	73.4 (68.2 to 78.4)	-43.0 (-47.3 to -38.7)
		Influenza	63500 (56200 to 71500)	14700 (7080 to 26200)	-76.8 (-88.7 to -59.3)	351 (338 to 366)	68 (31.8 to 121)	-80.7 (-90.9 to -65.7)
		Klebsiella pneumoniae	22000 (19500 to 24700)	16500 (14700 to 18400)	-24.8 (-29.8 to -19.8)	162 (155 to 170)	115 (108 to 122)	-29.4 (-34.0 to -24.6)
		Legionella spp.	29700 (25900 to 34000)	31500 (28100 to 35100)	6.2 (-4.0 to 16.5)	151 (140 to 164)	150 (140 to 161)	-0.5 (-10.2 to 9.3)
		Mycoplasma spp.	98100 (86700 to 111000)	85200 (75500 to 95100)	-13.1 (-19.4 to -6.8)	193 (182 to 204)	157 (148 to 167)	-18.5 (-24.7 to -12.2)
		Polymicrobial	1010 (584 to 1640)	1220 (704 to 1990)	20.8 (6.8 to 35.8)	7.57 (4.45 to 12.3)	8.65 (5.04 to 14.1)	14.2 (1.2 to 29.3)
		Pseudomonas aeruginosa	30800 (27000 to 35200)	38900 (34700 to 43100)	26.4 (17.4 to 36.1)	158 (148 to 168)	187 (177 to 198)	18.7 (10.4 to 27.9)
		Respiratory syncytial virus	24400 (21500 to 27700)	4010 (1980 to 7060)	-83.5 (-91.8 to -71.1)	61.4 (57 to 65.9)	8.4 (3.96 to 15)	-86.3 (-93.5 to -75.6)
		Staphylococcus aureus	40700 (36200 to 45900)	64000 (57500 to 70500)	57.4 (48.8 to 65.8)	427 (411 to 442)	633 (604 to 666)	48.5 (40.9 to 56.6)
		Streptococcus pneumoniae	337000 (300000 to 380000)	111000 (99300 to 122000)	-67.1 (-68.8 to -65.6)	1480 (1440 to 1520)	450 (429 to 470)	-69.5 (-71.1 to -68.0)
		Other viruses	221000 (197000 to 249000)	149000 (134000 to 164000)	-32.8 (-36.4 to -29.5)	380 (368 to 392)	238 (226 to 250)	-37.3 (-41.0 to -33.9)
		Other bacterial pathogens	31200 (26300 to 36500)	25000 (21300 to 29000)	-19.9 (-26.9 to -12.2)	131 (116 to 147)	98.3 (86 to 111)	-24.7 (-31.6 to -17.5)

	50-69 years	*Acinetobacter baumannii*	14200 (12000 to 16400)	9840 (8540 to 11300)	-30.3 (-37.1 to -23.4)	142 (123 to 161)	101 (89.2 to 113)	-28.4 (-35.2 to -20.9)
		Chlamydia spp.	54100 (48100 to 61100)	47500 (42600 to 53100)	-12.0 (-20.1 to -3.4)	135 (123 to 147)	122 (111 to 133)	-9.5 (-18.0 to 0.1)
		Enterobacter spp.	12900 (11200 to 14700)	24900 (22100 to 27600)	93.6 (75.3 to 114.0)	122 (108 to 136)	242 (221 to 264)	99.4 (80.4 to 119.6)
		Escherichia coli	30200 (26900 to 33700)	55100 (50100 to 60100)	82.9 (68.6 to 97.1)	296 (274 to 317)	557 (523 to 589)	88.8 (74.4 to 103.0)
		Fungus	28800 (25300 to 32500)	43300 (38000 to 48500)	50.5 (36.6 to 65.9)	278 (252 to 308)	431 (388 to 475)	55.0 (39.8 to 70.8)
		Group B Streptococcus	26700 (23500 to 30400)	43700 (38800 to 48600)	64.0 (46.5 to 81.2)	186 (169 to 205)	313 (281 to 341)	68.8 (52.2 to 86.3)
		Haemophilus influenzae	101000 (91300 to 110000)	123000 (112000 to 134000)	22.4 (15.1 to 29.5)	370 (352 to 386)	325 (306 to 344)	-12.1 (-17.5 to -6.7)
		Influenza	120000 (109000 to 130000)	39200 (19000 to 68400)	-67.2 (-84.3 to -42.0)	874 (833 to 909)	261 (120 to 466)	-70.1 (-86.0 to -47.0)
		Klebsiella pneumoniae	61600 (56400 to 67100)	71300 (65400 to 77500)	15.7 (9.3 to 22.0)	600 (574 to 626)	713 (673 to 751)	18.8 (12.3 to 25.2)
		Legionella spp.	91500 (81900 to 101000)	124000 (113000 to 135000)	35.5 (24.8 to 47.1)	614 (567 to 661)	850 (802 to 901)	38.6 (27.4 to 50.8)
		Mycoplasma spp.	98400 (88400 to 108000)	90000 (81700 to 98700)	-8.4 (-14.8 to -1.3)	255 (239 to 272)	239 (223 to 255)	-6.3 (-13.2 to 1.0)
		Polymicrobial	3120 (1870 to 5090)	4990 (2930 to 8210)	59.6 (42.6 to 77.5)	31 (18.4 to 50)	51.2 (30.5 to 83.7)	65.0 (47.8 to 82.4)
		Pseudomonas aeruginosa	95500 (86300 to 105000)	141000 (129000 to 153000)	48.2 (38.6 to 57.3)	645 (607 to 688)	978 (925 to 1030)	51.7 (41.4 to 62.1)
		Respiratory syncytial virus	34000 (30200 to 37700)	6720 (3290 to 11800)	-80.2 (-90.2 to -64.9)	113 (103 to 124)	20.3 (9.54 to 35.9)	-82.0 (-91.4 to -68.3)
		Staphylococcus aureus	111000 (102000 to 121000)	230000 (211000 to 250000)	107.5 (98.6 to 116.9)	1540 (1480 to 1590)	3280 (3120 to 3430)	113.8 (103.8 to 124.2)
		Streptococcus pneumoniae	454000 (416000 to 491000)	232000 (211000 to 251000)	-49.0 (-51.2 to -46.5)	2620 (2530 to 2700)	1360 (1290 to 1420)	-48.1 (-50.6 to -45.7)
		Other viruses	261000 (239000 to 284000)	272000 (249000 to 295000)	4.1 (-0.7 to 9.0)	592 (568 to 612)	627 (594 to 658)	6.1 (0.7 to 11.2)
		Other bacterial pathogens	103000 (88500 to 117000)	104000 (90000 to 119000)	1.3 (-6.4 to 9.5)	569 (508 to 636)	592 (520 to 674)	4.1 (-4.2 to 12.7)
	70+ years	*Acinetobacter baumannii*	23700 (19900 to 27800)	17000 (14700 to 19600)	-28.0 (-35.2 to -20.3)	802 (664 to 939)	388 (307 to 452)	-51.5 (-56.7 to -46.3)
		Chlamydia spp.	108000 (96000 to 122000)	97900 (87600 to 111000)	-9.1 (-17.9 to 0.5)	908 (772 to 1010)	554 (450 to 631)	-38.9 (-44.5 to -32.7)
		Enterobacter spp.	21000 (17900 to 24300)	41900 (36800 to 47100)	100.2 (80.7 to 122.1)	673 (559 to 775)	906 (729 to 1030)	34.7 (21.6 to 47.7)
		Escherichia coli	62000 (55200 to 70600)	117000 (106000 to 129000)	88.9 (72.7 to 105.2)	2060 (1760 to 2280)	2610 (2140 to 2900)	26.8 (16.0 to 37.1)
		Fungus	26900 (23600 to 30800)	41700 (36500 to 47300)	55.5 (40.0 to 72.1)	879 (739 to 1010)	919 (739 to 1060)	4.6 (-6.1 to 15.4)
		Group B Streptococcus	41000 (36400 to 46200)	69400 (61800 to 77600)	69.5 (51.9 to 87.8)	966 (802 to 1090)	1100 (897 to 1250)	14.0 (2.3 to 25.9)
		Haemophilus influenzae	152000 (139000 to 169000)	192000 (174000 to 212000)	26.5 (18.5 to 34.9)	1880 (1620 to 2030)	1130 (918 to 1250)	-40.1 (-44.2 to -36.3)

		Influenza	304000 (278000 to 335000)	102000 (49900 to 177000)	-66.4 (-83.6 to -41.4)	7550 (6460 to 8150)	1470 (699 to 2620)	-80.6 (-90.6 to -65.6)
		Klebsiella pneumoniae	114000 (104000 to 125000)	136000 (124000 to 150000)	19.7 (12.3 to 27.5)	3750 (3230 to 4050)	3030 (2440 to 3350)	-19.4 (-24.6 to -14.7)
		Legionella spp.	110000 (98600 to 124000)	154000 (141000 to 170000)	40.3 (28.0 to 54.0)	2500 (2120 to 2780)	2370 (1930 to 2640)	-5.3 (-13.8 to 3.4)
		Mycoplasma spp.	139000 (126000 to 156000)	132000 (120000 to 146000)	-5.2 (-12.3 to 2.5)	1220 (1040 to 1340)	781 (633 to 871)	-36.1 (-41.1 to -31.1)
		Polymicrobial	5990 (3520 to 10100)	9880 (5630 to 16500)	64.8 (46.3 to 84.7)	201 (116 to 338)	223 (126 to 381)	10.5 (-1.8 to 22.5)
		Pseudomonas aeruginosa	166000 (151000 to 186000)	255000 (232000 to 280000)	53.6 (42.8 to 65.0)	3820 (3280 to 4160)	3960 (3200 to 4350)	3.7 (-4.6 to 10.6)
		Respiratory syncytial virus	53300 (47600 to 60500)	10800 (5380 to 18700)	-79.6 (-89.9 to -64.5)	603 (514 to 672)	71.3 (34.4 to 125)	-88.2 (-94.2 to -79.6)
		Staphylococcus aureus	241000 (220000 to 266000)	516000 (472000 to 568000)	114.6 (103.4 to 126.8)	11300 (9720 to 12100)	16300 (13300 to 17900)	44.3 (36.8 to 51.5)
		Streptococcus pneumoniae	782000 (716000 to 864000)	413000 (379000 to 455000)	-47.1 (-50.0 to -43.9)	15300 (13200 to 16400)	5470 (4430 to 6020)	-64.2 (-66.3 to -62.5)
		Other viruses	444000 (408000 to 492000)	479000 (438000 to 528000)	7.9 (2.2 to 14.2)	3410 (2950 to 3670)	2490 (2040 to 2750)	-27.0 (-31.3 to -23.2)
		Other bacterial pathogens	231000 (201000 to 267000)	242000 (211000 to 279000)	4.7 (-3.6 to 14.2)	4360 (3600 to 5020)	3080 (2410 to 3600)	-29.5 (-35.4 to -23.3)
Canada	All Ages	*Acinetobacter baumannii*	3870 (3320 to 4480)	2960 (2530 to 3440)	-23.6 (-30.6 to -14.9)	86.6 (73.9 to 99.4)	56.1 (45.3 to 65.4)	-35.2 (-41.9 to -28.5)
		Chlamydia spp.	16100 (14700 to 17800)	15900 (14300 to 17600)	-1.6 (-10.8 to 8.6)	88 (77.6 to 96.3)	74.4 (61.8 to 83.4)	-15.5 (-24.2 to -7.1)
		Enterobacter spp.	3780 (3320 to 4250)	7300 (6450 to 8280)	93.2 (76.2 to 114.9)	78.3 (66.8 to 89.2)	129 (106 to 146)	65.0 (48.0 to 81.3)
		Escherichia coli	9460 (8590 to 10500)	16800 (15300 to 18800)	78.0 (63.8 to 97.2)	218 (192 to 238)	327 (272 to 364)	49.8 (35.0 to 64.3)
		Fungus	6210 (5590 to 6960)	9210 (8250 to 10500)	48.6 (34.4 to 65.2)	108 (95.2 to 121)	140 (117 to 159)	29.8 (16.2 to 44.2)
		Group B Streptococcus	7830 (7080 to 8630)	12800 (11600 to 14400)	64.1 (48.6 to 82.0)	112 (98.4 to 124)	159 (133 to 179)	41.9 (28.3 to 56.4)
		Haemophilus influenzae	27500 (25300 to 30000)	36900 (33700 to 40900)	34.5 (24.7 to 47.9)	143 (127 to 153)	168 (142 to 186)	17.7 (7.2 to 28.8)
		Influenza	50300 (46400 to 54900)	6850 (1560 to 16800)	-86.4 (-96.9 to -66.5)	847 (750 to 912)	93.6 (21.3 to 235)	-88.9 (-97.5 to -72.2)
		Klebsiella pneumoniae	19100 (17600 to 20900)	24100 (21900 to 26700)	25.8 (15.9 to 38.5)	409 (364 to 440)	448 (376 to 496)	9.3 (-0.5 to 18.8)
		Legionella spp.	21800 (19800 to 24000)	35600 (32500 to 39400)	63.8 (50.2 to 81.8)	288 (258 to 314)	407 (343 to 451)	41.4 (27.6 to 55.9)
		Mycoplasma spp.	31700 (29300 to 34300)	35000 (32300 to 38400)	10.4 (2.7 to 19.7)	138 (123 to 149)	134 (113 to 148)	-2.7 (-11.5 to 6.0)
		Polymicrobial	1010 (605 to 1640)	1450 (819 to 2520)	43.4 (27.1 to 61.4)	22.5 (13.5 to 37.2)	27.7 (15.3 to 47.6)	22.0 (7.1 to 36.6)
		Pseudomonas aeruginosa	30100 (27600 to 33100)	52100 (47400 to 57700)	73.3 (59.4 to 90.3)	446 (397 to 481)	657 (552 to 729)	47.5 (33.9 to 60.6)
		Respiratory syncytial virus	19400 (17900 to 21000)	1710 (389 to 4210)	-91.2 (-98.0 to -78.4)	91.6 (82.6 to 99)	7.16 (1.63 to 17.9)	-92.2 (-98.3 to -80.6)
		Staphylococcus aureus	40000 (36700 to 43600)	82400 (75000 to 91700)	106.4 (91.3 to 127.4)	1280 (1140 to 1370)	2250 (1900 to 2500)	75.6 (60.2 to 91.1)

		Streptococcus pneumoniae	144000 (133000 to 156000)	100000 (92600 to 110000)	-30.0 (-34.6 to -23.8)	1570 (1400 to 1670)	1000 (851 to 1110)	-35.9 (-41.3 to -30.6)
		Other viruses	98300 (91500 to 106000)	120000 (111000 to 132000)	22.2 (14.6 to 32.6)	399 (358 to 426)	456 (386 to 505)	14.1 (4.2 to 23.7)
		Other bacterial pathogens	31600 (27500 to 36100)	37700 (32400 to 43800)	19.5 (9.2 to 31.7)	423 (362 to 483)	424 (341 to 497)	0.3 (-9.1 to 10.0)
	Under 5	*Acinetobacter baumannii*	46.5 (29.4 to 72.7)	19.1 (11.6 to 31.6)	-59.1 (-64.5 to -52.9)	0.63 (0.35 to 1.09)	0.16 (0.08 to 0.3)	-74.9 (-80.5 to -67.9)
		Chlamydia spp.	560 (483 to 650)	241 (199 to 291)	-57.0 (-61.7 to -51.2)	1.04 (0.93 to 1.16)	0.22 (0.18 to 0.27)	-78.8 (-83.0 to -74.2)
		Enterobacter spp.	103 (78 to 132)	84.3 (62.5 to 112)	-18.2 (-28.6 to -5.9)	0.78 (0.58 to 1.06)	0.28 (0.2 to 0.38)	-64.6 (-71.7 to -56.1)
		Escherichia coli	280 (238 to 333)	233 (191 to 282)	-16.7 (-27.3 to -5.9)	2.05 (1.82 to 2.29)	0.71 (0.57 to 0.86)	-65.4 (-71.9 to -57.6)
		Fungus	47.6 (33.8 to 66.2)	33.4 (23.6 to 47.1)	-29.8 (-40.0 to -19.7)	0.24 (0.18 to 0.32)	0.07 (0.05 to 0.1)	-70.9 (-77.5 to -63.1)
		Group B Streptococcus	280 (236 to 335)	198 (162 to 243)	-29.2 (-37.5 to -19.9)	1.66 (1.48 to 1.86)	0.52 (0.42 to 0.64)	-68.7 (-74.8 to -61.6)
		Haemophilus influenzae	1760 (1500 to 2070)	1160 (958 to 1410)	-33.8 (-41.2 to -25.0)	2.73 (2.47 to 3.01)	0.69 (0.56 to 0.84)	-74.6 (-79.9 to -68.2)
		Influenza	1920 (1640 to 2240)	96.1 (22.4 to 235)	-95.0 (-98.8 to -87.8)	8.12 (7.44 to 8.89)	0.16 (0.03 to 0.41)	-98.0 (-99.6 to -94.8)
		Klebsiella pneumoniae	724 (619 to 851)	393 (325 to 480)	-45.7 (-51.7 to -38.9)	4.44 (4.05 to 4.85)	1.07 (0.88 to 1.29)	-75.8 (-80.4 to -70.3)
		Legionella spp.	153 (112 to 203)	111 (79 to 151)	-27.4 (-35.9 to -17.5)	0.95 (0.74 to 1.2)	0.32 (0.24 to 0.42)	-65.9 (-72.8 to -57.6)
		Mycoplasma spp.	1400 (1200 to 1650)	1010 (839 to 1230)	-27.9 (-35.9 to -19.0)	2.1 (1.9 to 2.33)	0.63 (0.51 to 0.76)	-70.1 (-76.2 to -63.2)
		Polymicrobial	67.3 (35.5 to 114)	39.5 (20.9 to 67.8)	-41.4 (-49.0 to -33.2)	1.06 (0.54 to 1.81)	0.37 (0.17 to 0.67)	-65.9 (-73.9 to -55.9)
		Pseudomonas aeruginosa	908 (783 to 1060)	850 (697 to 1030)	-6.4 (-17.1 to 4.7)	4.07 (3.68 to 4.48)	1.57 (1.29 to 1.9)	-61.3 (-68.8 to -52.9)
		Respiratory syncytial virus	7550 (6490 to 8750)	423 (101 to 1040)	-94.4 (-98.7 to -86.2)	15.5 (14.2 to 16.8)	0.35 (0.08 to 0.88)	-97.8 (-99.5 to -94.2)
		Staphylococcus aureus	886 (753 to 1040)	793 (649 to 950)	-10.5 (-19.6 to -0.3)	7.33 (6.74 to 7.99)	2.73 (2.24 to 3.26)	-62.7 (-69.8 to -54.8)
		Streptococcus pneumoniae	9240 (7910 to 10800)	2190 (1800 to 2660)	-76.3 (-78.7 to -73.7)	29.2 (26.8 to 32.1)	2.82 (2.29 to 3.38)	-90.3 (-92.3 to -88.1)
		Other viruses	9750 (8370 to 11400)	5460 (4500 to 6590)	-44.0 (-49.5 to -38.2)	12.5 (11.4 to 13.6)	3.01 (2.49 to 3.59)	-75.7 (-80.5 to -70.5)
		Other bacterial pathogens	255 (199 to 320)	143 (109 to 184)	-43.9 (-50.7 to -36.3)	1 (0.78 to 1.28)	0.25 (0.18 to 0.35)	-74.9 (-80.0 to -69.2)
	5-14 years	*Acinetobacter baumannii*	67.1 (48.8 to 90.8)	35.4 (25.9 to 47.2)	-47.2 (-52.6 to -41.5)	0.17 (0.14 to 0.2)	0.03 (0.03 to 0.04)	-79.2 (-82.3 to -75.6)
		Chlamydia spp.	392 (290 to 514)	290 (217 to 381)	-26.0 (-33.0 to -18.2)	0.24 (0.22 to 0.27)	0.07 (0.06 to 0.08)	-70.9 (-75.3 to -65.7)
		Enterobacter spp.	67.3 (48.7 to 90.1)	93.7 (67.8 to 124)	39.5 (25.2 to 55.5)	0.16 (0.14 to 0.18)	0.09 (0.07 to 0.1)	-45.1 (-53.9 to -35.5)

		Escherichia coli	135 (98.7 to 176)	177 (133 to 231)	31.5 (19.0 to 44.3)	0.33 (0.29 to 0.36)	0.17 (0.15 to 0.19)	-48.3 (-55.8 to -39.8)
		Fungus	163 (121 to 218)	179 (134 to 238)	9.6 (-1.9 to 22.1)	0.39 (0.35 to 0.44)	0.17 (0.14 to 0.2)	-56.9 (-63.4 to -49.4)
		Group B Streptococcus	192 (143 to 251)	238 (178 to 313)	24.0 (10.7 to 37.9)	0.33 (0.29 to 0.38)	0.16 (0.14 to 0.18)	-51.2 (-58.7 to -42.7)
		Haemophilus influenzae	751 (569 to 972)	704 (533 to 920)	-6.2 (-14.4 to 2.4)	0.5 (0.45 to 0.54)	0.18 (0.16 to 0.2)	-63.6 (-68.8 to -57.6)
		Influenza	1210 (905 to 1550)	114 (25.7 to 287)	-90.5 (-97.9 to -76.7)	2.19 (1.99 to 2.4)	0.08 (0.02 to 0.19)	-96.5 (-99.2 to -91.3)
		Klebsiella pneumoniae	393 (294 to 502)	311 (235 to 401)	-20.7 (-27.4 to -14.1)	0.95 (0.86 to 1.04)	0.3 (0.26 to 0.33)	-68.8 (-73.1 to -64.0)
		Legionella spp.	530 (398 to 685)	636 (484 to 820)	20.3 (8.8 to 31.6)	0.88 (0.8 to 0.98)	0.42 (0.37 to 0.47)	-52.7 (-59.6 to -44.8)
		Mycoplasma spp.	1640 (1220 to 2120)	1570 (1200 to 2050)	-4.0 (-12.4 to 4.9)	1.05 (0.96 to 1.16)	0.4 (0.35 to 0.45)	-62.2 (-67.7 to -56.1)
		Polymicrobial	17.9 (9.96 to 29.5)	18.7 (10.1 to 31.2)	4.1 (-7.7 to 16.5)	0.04 (0.03 to 0.07)	0.02 (0.01 to 0.03)	-59.0 (-65.5 to -51.5)
		Pseudomonas aeruginosa	590 (445 to 762)	776 (591 to 1000)	31.7 (20.5 to 43.3)	0.99 (0.9 to 1.09)	0.51 (0.45 to 0.58)	-48.2 (-55.4 to -39.2)
		Respiratory syncytial virus	484 (366 to 625)	40.9 (9.53 to 101)	-91.5 (-98.1 to -79.1)	0.4 (0.36 to 0.44)	0.01 (0 to 0.03)	-96.9 (-99.3 to -92.1)
		Staphylococcus aureus	781 (590 to 1000)	1130 (864 to 1460)	45.3 (33.7 to 56.5)	2.69 (2.47 to 2.93)	1.53 (1.35 to 1.72)	-42.8 (-50.8 to -33.9)
		Streptococcus pneumoniae	5400 (4070 to 6930)	2670 (2050 to 3460)	-50.5 (-54.3 to -46.6)	7.73 (7.07 to 8.48)	1.5 (1.32 to 1.69)	-80.5 (-83.2 to -77.5)
		Other viruses	3940 (2960 to 5080)	3380 (2580 to 4400)	-14.1 (-20.6 to -7.6)	2.22 (2.03 to 2.42)	0.75 (0.66 to 0.84)	-66.2 (-70.7 to -60.8)
		Other bacterial pathogens	518 (384 to 684)	437 (320 to 578)	-15.4 (-23.4 to -7.4)	0.71 (0.61 to 0.81)	0.23 (0.2 to 0.28)	-66.7 (-71.5 to -61.5)
	15-49 years	*Acinetobacter baumannii*	350 (281 to 424)	181 (146 to 218)	-48.3 (-53.1 to -42.7)	1.85 (1.56 to 2.17)	0.89 (0.74 to 1.04)	-52.0 (-58.0 to -45.7)
		Chlamydia spp.	2040 (1740 to 2400)	1480 (1270 to 1730)	-27.4 (-34.2 to -20.8)	2.69 (2.43 to 2.97)	1.81 (1.61 to 2.04)	-32.7 (-40.7 to -23.9)
		Enterobacter spp.	350 (288 to 420)	478 (394 to 569)	36.8 (23.3 to 51.9)	1.76 (1.53 to 2.01)	2.23 (1.93 to 2.55)	26.8 (10.5 to 44.0)
		Escherichia coli	701 (594 to 823)	902 (777 to 1040)	28.9 (17.9 to 40.4)	3.64 (3.3 to 3.98)	4.35 (3.92 to 4.86)	19.5 (5.8 to 35.0)
		Fungus	850 (717 to 996)	912 (773 to 1080)	7.5 (-2.5 to 18.5)	4.35 (3.88 to 4.84)	4.33 (3.81 to 4.89)	-0.3 (-13.6 to 12.7)
		Group B Streptococcus	1000 (851 to 1190)	1210 (1040 to 1420)	21.6 (9.5 to 34.2)	3.69 (3.31 to 4.11)	4.15 (3.7 to 4.66)	12.7 (-1.1 to 28.6)
		Haemophilus influenzae	3910 (3350 to 4530)	3590 (3090 to 4150)	-8.0 (-15.4 to 0.2)	5.5 (5.13 to 5.88)	4.62 (4.15 to 5.1)	-15.9 (-25.9 to -5.8)
		Influenza	6280 (5410 to 7250)	585 (131 to 1450)	-90.7 (-97.9 to -77.2)	24.4 (22.7 to 26)	1.97 (0.44 to 4.93)	-91.9 (-98.2 to -80.1)
		Klebsiella pneumoniae	2040 (1780 to 2360)	1590 (1360 to 1840)	-22.3 (-28.2 to -16.1)	10.6 (9.79 to 11.3)	7.6 (6.9 to 8.41)	-27.9 (-36.5 to -19.3)
		Legionella spp.	2760 (2390 to 3200)	3250 (2800 to 3720)	17.9 (7.6 to 28.7)	9.81 (9.02 to 10.7)	10.7 (9.69 to 11.9)	9.3 (-4.0 to 22.6)

		Mycoplasma spp.	8520 (7390 to 9840)	8010 (6920 to 9270)	-5.9 (-13.1 to 2.2)	11.7 (10.8 to 12.6)	10.2 (9.21 to 11.3)	-12.7 (-22.3 to -2.4)
		Polymicrobial	93.4 (54.2 to 152)	95.7 (51.9 to 161)	2.1 (-9.2 to 14.4)	0.49 (0.28 to 0.81)	0.47 (0.26 to 0.78)	-5.4 (-17.7 to 9.2)
		Pseudomonas aeruginosa	3070 (2660 to 3560)	3960 (3420 to 4570)	29.1 (18.7 to 40.2)	11 (10.2 to 11.9)	13.2 (12 to 14.6)	19.7 (6.1 to 34.5)
		Respiratory syncytial virus	2520 (2160 to 2940)	209 (46.3 to 518)	-91.7 (-98.2 to -79.7)	4.45 (4.1 to 4.83)	0.32 (0.07 to 0.81)	-92.8 (-98.4 to -82.2)
		Staphylococcus aureus	4060 (3530 to 4690)	5790 (4970 to 6660)	42.4 (33.3 to 52.5)	29.8 (28 to 31.8)	39.4 (36.1 to 43.3)	32.1 (17.5 to 46.5)
		Streptococcus pneumoniae	28100 (24400 to 32200)	13600 (11800 to 15700)	-51.4 (-54.5 to -47.9)	85.9 (80.8 to 91.5)	38.6 (35.3 to 42.4)	-55.0 (-59.7 to -50.0)
		Other viruses	20500 (17800 to 23600)	17300 (14800 to 19800)	-15.7 (-21.2 to -9.9)	24.6 (23.1 to 26.3)	19.2 (17.6 to 21.1)	-21.9 (-30.2 to -13.4)
		Other bacterial pathogens	2700 (2230 to 3210)	2230 (1850 to 2670)	-17.1 (-24.2 to -9.2)	7.87 (6.88 to 8.92)	6.05 (5.14 to 7.02)	-23.1 (-32.0 to -13.2)
	50-69 years	*Acinetobacter baumannii*	1320 (1110 to 1540)	928 (783 to 1090)	-29.3 (-37.3 to -20.9)	10.5 (8.94 to 12.3)	6.25 (5.34 to 7.25)	-40.5 (-48.9 to -31.0)
		Chlamydia spp.	4530 (3960 to 5210)	4190 (3680 to 4800)	-7.4 (-18.4 to 3.5)	9.02 (7.94 to 10.2)	7.02 (6.18 to 7.91)	-22.0 (-33.0 to -10.0)
		Enterobacter spp.	1280 (1100 to 1470)	2300 (1990 to 2630)	80.7 (59.1 to 104.2)	9.7 (8.4 to 11.2)	14.7 (12.9 to 16.6)	52.1 (29.9 to 75.0)
		Escherichia coli	2820 (2480 to 3210)	4590 (4060 to 5190)	63.2 (46.0 to 81.9)	22.2 (19.6 to 24.8)	30.4 (27.2 to 33.7)	37.4 (19.0 to 57.9)
		Fungus	2730 (2370 to 3090)	3890 (3400 to 4470)	43.1 (26.3 to 60.9)	21.1 (18.3 to 24.1)	25.4 (22.3 to 28.8)	20.5 (3.3 to 39.1)
		Group B Streptococcus	2580 (2240 to 2940)	4030 (3550 to 4620)	56.5 (38.1 to 75.8)	14.4 (12.6 to 16.2)	18.9 (16.7 to 21.4)	31.7 (14.5 to 52.6)
		Haemophilus influenzae	8640 (7700 to 9730)	11500 (10200 to 13000)	33.0 (18.6 to 47.2)	18.4 (16.6 to 20.5)	20.2 (18.4 to 22.5)	10.3 (-4.7 to 26.4)
		Influenza	11900 (10700 to 13400)	1540 (346 to 3800)	-87.1 (-97.1 to -68.2)	70 (63 to 77.6)	7.11 (1.62 to 17.7)	-89.8 (-97.6 to -74.7)
		Klebsiella pneumoniae	5800 (5190 to 6510)	6950 (6190 to 7820)	20.1 (7.4 to 32.9)	45.3 (40.8 to 50.1)	45.7 (41.2 to 50.5)	1.1 (-12.5 to 15.6)
		Legionella spp.	8540 (7590 to 9680)	13200 (11800 to 15000)	55.1 (37.5 to 72.5)	45.9 (40.9 to 51.2)	59.8 (53.8 to 66.4)	30.6 (12.9 to 50.9)
		Mycoplasma spp.	8580 (7610 to 9670)	9250 (8230 to 10400)	8.1 (-3.2 to 19.4)	17.8 (15.9 to 19.9)	16.1 (14.4 to 18)	-9.0 (-21.4 to 4.3)
		Polymicrobial	293 (173 to 484)	403 (228 to 685)	37.3 (19.7 to 55.9)	2.33 (1.36 to 3.86)	2.7 (1.5 to 4.52)	15.6 (-1.3 to 34.4)
		Pseudomonas aeruginosa	9590 (8560 to 10800)	15400 (13700 to 17300)	61.1 (44.1 to 79.2)	52 (46.9 to 58)	70.3 (63.3 to 77.6)	35.6 (17.6 to 55.6)
		Respiratory syncytial virus	3530 (3090 to 4000)	370 (81.7 to 908)	-89.5 (-97.6 to -74.3)	9.45 (8.31 to 10.6)	0.78 (0.18 to 1.96)	-91.7 (-98.1 to -79.4)
		Staphylococcus aureus	11200 (9920 to 12500)	21300 (18900 to 23900)	91.2 (71.1 to 111.5)	124 (112 to 136)	199 (180 to 220)	61.0 (39.1 to 84.5)
		Streptococcus pneumoniae	38200 (34200 to 43100)	27400 (24400 to 30800)	-28.2 (-35.5 to -20.7)	177 (159 to 194)	107 (96.4 to 117)	-39.5 (-47.3 to -30.8)
		Other viruses	24500 (21900 to 27500)	31700 (28200 to 35500)	29.7 (16.6 to 43.9)	44.5 (40 to 48.8)	48.4 (43.8 to 53.7)	9.2 (-4.9 to 25.3)
		Other bacterial pathogens	8970 (7630 to 10300)	9720 (8180 to 11400)	8.6 (-3.7 to 21.2)	39.6 (34.3 to 46)	36.2 (30.6 to 42.3)	-8.5 (-21.2 to 5.4)

	70+ years	*Acinetobacter baumannii*	2090 (1760 to 2460)	1790 (1510 to 2140)	-14.3 (-22.6 to -2.1)	73.4 (61.7 to 84.7)	48.8 (38.7 to 57.4)	-33.6 (-40.8 to -26.8)
		Chlamydia spp.	8610 (7630 to 9690)	9660 (8500 to 11100)	12.3 (0.5 to 26.6)	75 (65.1 to 82.6)	65.2 (53 to 73.8)	-13.0 (-22.3 to -3.9)
		Enterobacter spp.	1980 (1710 to 2270)	4340 (3730 to 5070)	119.0 (97.8 to 148.3)	65.9 (56 to 75.4)	112 (89.5 to 128)	69.7 (51.5 to 86.7)
		Escherichia coli	5520 (4930 to 6180)	10900 (9660 to 12600)	97.8 (79.4 to 123.5)	190 (165 to 208)	291 (239 to 326)	53.3 (37.7 to 67.8)
		Fungus	2420 (2130 to 2750)	4200 (3620 to 4930)	73.5 (55.6 to 97.9)	82 (70.3 to 92.9)	110 (88.9 to 127)	34.5 (20.3 to 49.3)
		Group B Streptococcus	3770 (3330 to 4250)	7150 (6280 to 8320)	89.7 (70.5 to 114.0)	92.1 (79.3 to 102)	135 (110 to 154)	47.0 (32.8 to 62.6)
		Haemophilus influenzae	12400 (11200 to 13800)	20000 (17700 to 22900)	61.2 (47.0 to 81.0)	116 (101 to 125)	142 (117 to 159)	23.1 (11.9 to 34.6)
		Influenza	28900 (26100 to 31800)	4520 (1030 to 11100)	-84.4 (-96.4 to -61.3)	742 (650 to 803)	84.3 (19.3 to 213)	-88.6 (-97.5 to -71.3)
		Klebsiella pneumoniae	10200 (9210 to 11200)	14800 (13200 to 17000)	45.6 (32.7 to 63.6)	348 (306 to 377)	393 (323 to 438)	12.8 (2.4 to 22.6)
		Legionella spp.	9790 (8720 to 10900)	18400 (16300 to 21100)	88.1 (70.3 to 114.4)	230 (201 to 253)	336 (277 to 375)	45.7 (30.7 to 60.4)
		Mycoplasma spp.	11600 (10400 to 12800)	15100 (13500 to 17500)	31.0 (18.8 to 46.8)	105 (91.4 to 115)	107 (87.8 to 119)	1.5 (-8.1 to 11.1)
		Polymicrobial	534 (302 to 904)	893 (478 to 1570)	66.4 (47.7 to 90.3)	18.6 (10.7 to 31.5)	24.1 (13.1 to 42.2)	29.0 (13.9 to 44.0)
		Pseudomonas aeruginosa	15900 (14400 to 17700)	31000 (27500 to 35700)	95.3 (77.2 to 119.1)	377 (330 to 410)	572 (468 to 637)	51.3 (36.7 to 65.1)
		Respiratory syncytial virus	5280 (4690 to 5970)	669 (155 to 1650)	-87.3 (-97.1 to -68.4)	61.8 (53.5 to 68.5)	5.7 (1.29 to 14.1)	-90.8 (-97.9 to -76.9)
		Staphylococcus aureus	23000 (20800 to 25300)	53400 (47600 to 61300)	131.8 (112.4 to 159.5)	1120 (985 to 1200)	2010 (1660 to 2240)	79.6 (63.4 to 94.9)
		Streptococcus pneumoniae	62700 (56800 to 69200)	54600 (48500 to 62000)	-12.9 (-20.2 to -1.4)	1270 (1110 to 1360)	855 (708 to 955)	-32.5 (-38.8 to -26.7)
		Other viruses	39700 (35800 to 43600)	62300 (55500 to 71000)	57.2 (43.9 to 76.4)	315 (276 to 341)	384 (319 to 429)	21.8 (11.0 to 32.5)
		Other bacterial pathogens	19100 (16500 to 22100)	25200 (21200 to 30000)	31.6 (19.5 to 48.2)	374 (316 to 427)	382 (300 to 449)	2.1 (-7.7 to 11.8)
Greenland	All Ages	*Acinetobacter baumannii*	14.1 (12.3 to 16.1)	9.39 (8.14 to 10.9)	-33.2 (-41.4 to -24.5)	0.34 (0.27 to 0.4)	0.15 (0.12 to 0.19)	-54.5 (-64.8 to -40.7)
		Chlamydia spp.	43.8 (39.7 to 48.7)	33.3 (29.7 to 37.3)	-23.7 (-32.1 to -14.7)	0.26 (0.22 to 0.3)	0.13 (0.11 to 0.16)	-48.6 (-60.0 to -34.6)
		Enterobacter spp.	4.53 (3.89 to 5.25)	8.89 (7.75 to 10.2)	96.6 (74.0 to 121.0)	0.1 (0.08 to 0.12)	0.13 (0.11 to 0.17)	30.1 (-0.3 to 69.1)
		Escherichia coli	14.8 (13.3 to 16.6)	26.3 (23.6 to 29.4)	77.7 (59.3 to 97.2)	0.37 (0.31 to 0.43)	0.43 (0.35 to 0.54)	16.9 (-8.1 to 51.7)
		Fungus	11.2 (9.75 to 13)	16.5 (14.4 to 19)	47.4 (29.5 to 66.4)	0.22 (0.18 to 0.26)	0.22 (0.18 to 0.28)	2.1 (-21.6 to 31.8)
		Group B Streptococcus	12 (10.7 to 13.6)	18.4 (16.3 to 20.7)	53.4 (34.7 to 72.7)	0.2 (0.16 to 0.23)	0.2 (0.16 to 0.24)	0.3 (-21.4 to 28.0)
		Haemophilus influenzae	47.3 (43.2 to 51.7)	46.8 (42.3 to 51.8)	-1.0 (-9.9 to 9.2)	0.41 (0.35 to 0.48)	0.18 (0.14 to 0.22)	-56.9 (-65.7 to -45.6)
		Influenza	64.9 (60.1 to 70.5)	14.2 (7.32 to 24)	-78.2 (-88.9 to -63.3)	1.16 (0.96 to 1.32)	0.15 (0.08 to 0.28)	-86.7 (-93.5 to -76.2)

		Klebsiella pneumoniae	34.3 (31.7 to 37.4)	35.4 (32.2 to 39.1)	3.4 (-6.1 to 13.3)	0.81 (0.68 to 0.93)	0.56 (0.46 to 0.7)	-30.6 (-45.1 to -11.6)
		Legionella spp.	20 (17.6 to 22.7)	30 (27 to 33.6)	50.7 (33.5 to 69.1)	0.29 (0.24 to 0.34)	0.3 (0.24 to 0.37)	3.7 (-19.2 to 33.2)
		Mycoplasma spp.	59.7 (54.5 to 65.5)	48.9 (44.4 to 53.7)	-17.9 (-25.6 to -10.6)	0.3 (0.25 to 0.34)	0.16 (0.14 to 0.2)	-45.1 (-56.3 to -31.4)
		Polymicrobial	2.35 (1.66 to 3.36)	3.18 (2.08 to 4.86)	34.7 (13.8 to 56.5)	0.06 (0.04 to 0.09)	0.05 (0.03 to 0.08)	-15.7 (-36.7 to 11.6)
		Pseudomonas aeruginosa	27.2 (24.8 to 30)	44.7 (40.5 to 49.4)	64.4 (48.4 to 81.2)	0.44 (0.37 to 0.51)	0.48 (0.39 to 0.6)	8.8 (-14.2 to 39.7)
		Respiratory syncytial virus	28.4 (25.7 to 31.2)	2.78 (1.46 to 4.74)	-90.2 (-94.9 to -83.4)	0.26 (0.22 to 0.32)	0.01 (0.01 to 0.02)	-95.7 (-98.0 to -92.2)
		Staphylococcus aureus	43.3 (39.8 to 47.2)	91.2 (82.9 to 100)	110.5 (91.4 to 129.5)	1.46 (1.22 to 1.67)	2.09 (1.72 to 2.63)	44.2 (14.0 to 84.8)
		Streptococcus pneumoniae	294 (274 to 317)	119 (109 to 131)	-59.5 (-63.0 to -56.1)	3.71 (3.15 to 4.25)	1.02 (0.84 to 1.25)	-72.4 (-78.3 to -65.1)
		Other viruses	126 (117 to 136)	97 (89.5 to 105)	-23.0 (-29.2 to -16.5)	0.65 (0.55 to 0.75)	0.32 (0.27 to 0.39)	-50.4 (-61.1 to -37.2)
		Other bacterial pathogens	53.8 (48 to 60.6)	55.9 (49 to 63.5)	3.9 (-7.1 to 16.5)	0.74 (0.61 to 0.86)	0.53 (0.42 to 0.67)	-28.5 (-44.3 to -7.5)
	Under 5	*Acinetobacter baumannii*	0.5 (0.34 to 0.72)	0.1 (0.07 to 0.15)	-79.8 (-82.6 to -76.7)	0.02 (0.01 to 0.03)	0 (0 to 0)	-89.9 (-93.6 to -85.9)
		Chlamydia spp.	4.73 (4.04 to 5.49)	1.18 (0.98 to 1.39)	-75.1 (-78.7 to -71.2)	0.04 (0.03 to 0.05)	0 (0 to 0.01)	-89.2 (-93.2 to -84.7)
		Enterobacter spp.	0.44 (0.34 to 0.57)	0.23 (0.18 to 0.3)	-46.5 (-55.9 to -35.9)	0.01 (0.01 to 0.02)	0 (0 to 0)	-78.2 (-86.1 to -69.4)
		Escherichia coli	1.55 (1.31 to 1.84)	0.89 (0.74 to 1.06)	-42.5 (-51.7 to -32.6)	0.05 (0.04 to 0.07)	0.01 (0.01 to 0.01)	-76.9 (-84.9 to -67.2)
		Fungus	0.27 (0.19 to 0.38)	0.12 (0.08 to 0.17)	-54.7 (-62.9 to -45.5)	0.01 (0.01 to 0.01)	0 (0 to 0)	-81.2 (-88.4 to -72.3)
		Group B Streptococcus	1.43 (1.2 to 1.69)	0.64 (0.53 to 0.77)	-55.2 (-61.9 to -47.7)	0.04 (0.03 to 0.05)	0.01 (0 to 0.01)	-81.7 (-88.3 to -74.3)
		Haemophilus influenzae	10.4 (8.92 to 12.1)	3.17 (2.68 to 3.76)	-69.5 (-73.9 to -64.7)	0.12 (0.09 to 0.16)	0.01 (0.01 to 0.01)	-91.8 (-94.8 to -88.2)
		Influenza	8.73 (7.59 to 10.1)	0.48 (0.25 to 0.82)	-94.5 (-97.2 to -90.8)	0.2 (0.15 to 0.26)	0 (0 to 0.01)	-98.0 (-99.1 to -96.1)
		Klebsiella pneumoniae	5.4 (4.66 to 6.22)	1.33 (1.12 to 1.57)	-75.3 (-78.7 to -71.8)	0.16 (0.13 to 0.21)	0.02 (0.01 to 0.02)	-89.4 (-93.3 to -85.2)
		Legionella spp.	0.54 (0.39 to 0.72)	0.22 (0.16 to 0.29)	-59.6 (-65.7 to -52.0)	0.01 (0.01 to 0.02)	0 (0 to 0)	-82.5 (-89.0 to -75.6)
		Mycoplasma spp.	6.04 (5.17 to 6.98)	2.99 (2.53 to 3.53)	-50.4 (-56.9 to -42.3)	0.05 (0.04 to 0.06)	0.01 (0.01 to 0.01)	-79.7 (-87.3 to -71.1)
		Polymicrobial	0.35 (0.21 to 0.56)	0.11 (0.07 to 0.18)	-68.0 (-72.9 to -62.2)	0.02 (0.01 to 0.02)	0 (0 to 0)	-84.6 (-90.6 to -78.1)
		Pseudomonas aeruginosa	2.8 (2.41 to 3.26)	1.76 (1.48 to 2.07)	-37.1 (-45.6 to -27.1)	0.06 (0.05 to 0.08)	0.02 (0.01 to 0.02)	-74.6 (-83.7 to -64.3)
		Respiratory syncytial virus	18.8 (16.5 to 21.4)	1.19 (0.62 to 2.03)	-93.7 (-96.7 to -89.4)	0.2 (0.16 to 0.26)	0 (0 to 0.01)	-97.7 (-99.0 to -95.6)

		Staphylococcus aureus	3.51 (3.05 to 4.01)	2.13 (1.79 to 2.52)	-39.2 (-46.6 to -31.3)	0.15 (0.12 to 0.2)	0.04 (0.03 to 0.05)	-75.0 (-84.2 to -64.5)
		Streptococcus pneumoniae	56.5 (49.8 to 64.8)	5.4 (4.56 to 6.34)	-90.4 (-91.5 to -89.2)	0.98 (0.74 to 1.28)	0.04 (0.03 to 0.05)	-96.0 (-97.5 to -94.3)
		Other viruses	37.7 (33.1 to 42.9)	9.15 (7.77 to 10.7)	-75.7 (-78.4 to -72.5)	0.26 (0.2 to 0.34)	0.03 (0.02 to 0.03)	-89.7 (-93.4 to -85.2)
		Other bacterial pathogens	1.67 (1.32 to 2.02)	0.54 (0.42 to 0.67)	-67.9 (-73.0 to -62.2)	0.03 (0.02 to 0.04)	0 (0 to 0.01)	-86.5 (-91.4 to -81.2)
	5-14 years	*Acinetobacter baumannii*	0.5 (0.38 to 0.67)	0.18 (0.14 to 0.24)	-64.3 (-69.1 to -59.4)	0 (0 to 0)	0 (0 to 0)	-85.1 (-89.9 to -78.7)
		Chlamydia spp.	2 (1.49 to 2.63)	0.98 (0.74 to 1.31)	-50.8 (-56.8 to -44.7)	0 (0 to 0)	0 (0 to 0)	-79.4 (-86.5 to -70.6)
		Enterobacter spp.	0.16 (0.12 to 0.21)	0.18 (0.13 to 0.24)	15.7 (0.2 to 30.7)	0 (0 to 0)	0 (0 to 0)	-51.7 (-67.8 to -30.2)
		Escherichia coli	0.44 (0.33 to 0.57)	0.47 (0.35 to 0.62)	7.7 (-6.1 to 21.7)	0 (0 to 0)	0 (0 to 0)	-55.0 (-69.4 to -37.1)
		Fungus	0.53 (0.39 to 0.7)	0.45 (0.34 to 0.6)	-15.0 (-25.8 to -3.4)	0 (0 to 0)	0 (0 to 0)	-64.5 (-76.3 to -49.5)
		Group B Streptococcus	0.53 (0.39 to 0.7)	0.52 (0.39 to 0.7)	-0.7 (-14.1 to 12.4)	0 (0 to 0)	0 (0 to 0)	-58.5 (-72.2 to -41.2)
		Haemophilus influenzae	2.05 (1.55 to 2.67)	1.31 (0.99 to 1.74)	-36.1 (-43.0 to -28.8)	0 (0 to 0.01)	0 (0 to 0)	-82.0 (-87.6 to -74.7)
		Influenza	2.96 (2.24 to 3.79)	0.39 (0.2 to 0.71)	-86.7 (-93.2 to -77.0)	0.01 (0.01 to 0.02)	0 (0 to 0)	-95.1 (-97.8 to -90.6)
		Klebsiella pneumoniae	1.28 (0.98 to 1.66)	0.72 (0.54 to 0.94)	-44.3 (-50.3 to -38.7)	0.01 (0.01 to 0.01)	0 (0 to 0)	-76.7 (-83.9 to -67.0)
		Legionella spp.	0.91 (0.68 to 1.2)	0.8 (0.61 to 1.06)	-12.1 (-23.8 to -1.1)	0 (0 to 0)	0 (0 to 0)	-63.3 (-75.6 to -47.3)
		Mycoplasma spp.	4.62 (3.44 to 5.88)	3.09 (2.37 to 4.04)	-33.0 (-40.4 to -26.1)	0.01 (0 to 0.01)	0 (0 to 0)	-72.0 (-81.2 to -60.2)
		Polymicrobial	0.08 (0.05 to 0.12)	0.07 (0.04 to 0.1)	-17.5 (-29.4 to -5.5)	0 (0 to 0)	0 (0 to 0)	-65.5 (-77.4 to -50.2)
		Pseudomonas aeruginosa	1.03 (0.78 to 1.34)	1.08 (0.81 to 1.4)	4.9 (-7.0 to 16.6)	0 (0 to 0)	0 (0 to 0)	-56.2 (-70.0 to -38.0)
		Respiratory syncytial virus	0.69 (0.52 to 0.9)	0.08 (0.04 to 0.14)	-88.9 (-94.2 to -80.6)	0 (0 to 0)	0 (0 to 0)	-95.9 (-98.1 to -92.1)
		Staphylococcus aureus	1.71 (1.3 to 2.2)	2.09 (1.6 to 2.73)	22.1 (10.3 to 33.4)	0.01 (0.01 to 0.02)	0.01 (0 to 0.01)	-49.0 (-65.2 to -28.3)
		Streptococcus pneumoniae	17.1 (12.9 to 21.8)	4.62 (3.52 to 6.04)	-73.0 (-75.6 to -70.6)	0.05 (0.04 to 0.07)	0.01 (0 to 0.01)	-88.7 (-92.3 to -83.8)
		Other viruses	6.81 (5.14 to 8.76)	3.85 (2.97 to 5.04)	-43.4 (-48.9 to -38.1)	0.01 (0.01 to 0.01)	0 (0 to 0)	-76.3 (-83.8 to -66.5)
		Other bacterial pathogens	1.93 (1.47 to 2.51)	1.12 (0.83 to 1.48)	-41.8 (-49.1 to -34.4)	0.01 (0 to 0.01)	0 (0 to 0)	-75.6 (-83.7 to -65.6)
	15-49 years	*Acinetobacter baumannii*	2.74 (2.25 to 3.29)	0.86 (0.72 to 1.03)	-68.6 (-72.6 to -64.0)	0.04 (0.03 to 0.04)	0.01 (0 to 0.01)	-79.9 (-86.6 to -72.0)
		Chlamydia spp.	10.9 (9.3 to 12.7)	4.69 (4.03 to 5.44)	-56.8 (-62.0 to -51.2)	0.03 (0.03 to 0.04)	0.01 (0.01 to 0.01)	-72.4 (-81.8 to -61.2)

		Enterobacter spp.	0.85 (0.7 to 1.02)	0.86 (0.72 to 1.02)	1.5 (-11.8 to 15.7)	0.01 (0.01 to 0.01)	0.01 (0 to 0.01)	-35.0 (-56.7 to -8.8)
		Escherichia coli	2.37 (2 to 2.77)	2.24 (1.94 to 2.57)	-5.5 (-16.2 to 6.4)	0.03 (0.02 to 0.04)	0.02 (0.01 to 0.02)	-39.5 (-60.3 to -16.5)
		Fungus	2.86 (2.39 to 3.4)	2.13 (1.78 to 2.5)	-25.4 (-34.6 to -15.6)	0.04 (0.03 to 0.04)	0.02 (0.01 to 0.02)	-52.2 (-69.0 to -33.2)
		Group B Streptococcus	2.87 (2.43 to 3.42)	2.49 (2.14 to 2.91)	-12.9 (-23.6 to -0.9)	0.03 (0.02 to 0.03)	0.01 (0.01 to 0.02)	-44.3 (-62.9 to -23.1)
		Haemophilus influenzae	11.1 (9.7 to 12.7)	6.24 (5.47 to 7.14)	-43.9 (-49.6 to -37.6)	0.05 (0.04 to 0.07)	0.01 (0.01 to 0.02)	-75.8 (-83.9 to -66.6)
		Influenza	16.1 (14.1 to 18.3)	1.88 (0.94 to 3.22)	-88.3 (-94.0 to -80.1)	0.15 (0.12 to 0.19)	0.01 (0 to 0.02)	-93.4 (-96.9 to -87.7)
		Klebsiella pneumoniae	6.98 (6.11 to 7.97)	3.41 (2.96 to 3.9)	-51.1 (-55.4 to -46.0)	0.09 (0.07 to 0.11)	0.03 (0.02 to 0.04)	-68.7 (-79.1 to -56.6)
		Legionella spp.	4.93 (4.23 to 5.71)	3.79 (3.3 to 4.34)	-22.9 (-31.6 to -12.8)	0.04 (0.03 to 0.05)	0.02 (0.01 to 0.03)	-50.7 (-67.2 to -30.5)
		Mycoplasma spp.	25.1 (21.9 to 28.8)	14.7 (12.8 to 16.9)	-41.2 (-47.0 to -34.9)	0.08 (0.07 to 0.1)	0.03 (0.02 to 0.04)	-62.4 (-74.9 to -48.0)
		Polymicrobial	0.44 (0.29 to 0.67)	0.32 (0.2 to 0.49)	-27.6 (-37.8 to -16.3)	0.01 (0 to 0.01)	0 (0 to 0)	-53.7 (-69.2 to -34.7)
		Pseudomonas aeruginosa	5.58 (4.83 to 6.45)	5.13 (4.44 to 5.81)	-7.9 (-17.1 to 2.4)	0.05 (0.04 to 0.06)	0.03 (0.02 to 0.04)	-41.1 (-60.7 to -18.7)
		Respiratory syncytial virus	3.74 (3.24 to 4.3)	0.36 (0.18 to 0.64)	-90.2 (-95.0 to -83.2)	0.02 (0.01 to 0.02)	0 (0 to 0)	-94.5 (-97.5 to -89.9)
		Staphylococcus aureus	9.32 (8.14 to 10.5)	9.98 (8.75 to 11.3)	7.2 (-1.4 to 17.3)	0.17 (0.13 to 0.2)	0.11 (0.08 to 0.16)	-31.4 (-54.7 to -4.1)
		Streptococcus pneumoniae	92.8 (81.4 to 105)	22 (19.4 to 24.9)	-76.3 (-78.2 to -74.0)	0.69 (0.55 to 0.84)	0.1 (0.07 to 0.14)	-84.8 (-89.9 to -78.9)
		Other viruses	37 (32.6 to 41.7)	18.4 (16.1 to 20.8)	-50.3 (-54.4 to -45.6)	0.11 (0.09 to 0.13)	0.03 (0.02 to 0.05)	-68.2 (-78.6 to -56.0)
		Other bacterial pathogens	10.5 (8.96 to 12.2)	5.34 (4.56 to 6.24)	-48.9 (-54.6 to -42.5)	0.07 (0.06 to 0.09)	0.02 (0.02 to 0.03)	-67.3 (-78.2 to -54.2)
	50-69 years	*Acinetobacter baumannii*	6.19 (5.21 to 7.34)	4.86 (4.1 to 5.74)	-21.3 (-32.2 to -10.3)	0.11 (0.08 to 0.13)	0.05 (0.04 to 0.07)	-49.0 (-63.0 to -31.7)
		Chlamydia spp.	14.6 (12.5 to 16.9)	14.5 (12.6 to 16.5)	-0.3 (-14.0 to 14.2)	0.06 (0.05 to 0.08)	0.04 (0.03 to 0.05)	-35.4 (-52.6 to -15.1)
		Enterobacter spp.	1.87 (1.55 to 2.25)	4.53 (3.87 to 5.29)	143.6 (108.3 to 180.1)	0.03 (0.02 to 0.04)	0.05 (0.04 to 0.06)	57.9 (13.9 to 111.1)
		Escherichia coli	5.75 (4.93 to 6.63)	12.2 (10.7 to 13.9)	113.7 (84.0 to 141.7)	0.1 (0.08 to 0.12)	0.13 (0.1 to 0.17)	38.4 (1.1 to 82.1)
		Fungus	5.52 (4.7 to 6.51)	9.97 (8.45 to 11.6)	81.0 (55.4 to 107.1)	0.09 (0.07 to 0.11)	0.11 (0.08 to 0.14)	17.3 (-15.0 to 55.4)
		Group B Streptococcus	4.46 (3.81 to 5.25)	9 (7.67 to 10.4)	102.4 (71.9 to 131.6)	0.05 (0.04 to 0.06)	0.07 (0.05 to 0.09)	31.1 (-4.7 to 72.8)
		Haemophilus influenzae	14.8 (12.9 to 16.9)	22.1 (19.5 to 25.2)	50.2 (30.9 to 69.3)	0.09 (0.08 to 0.11)	0.06 (0.05 to 0.08)	-34.3 (-51.8 to -14.0)
		Influenza	18.4 (16.2 to 20.8)	5.54 (2.77 to 9.35)	-69.8 (-84.8 to -48.7)	0.23 (0.19 to 0.27)	0.04 (0.02 to 0.07)	-82.7 (-91.8 to -68.8)

		Klebsiella pneumoniae	11.9 (10.5 to 13.5)	16.9 (14.9 to 19.1)	43.2 (24.5 to 60.2)	0.2 (0.16 to 0.24)	0.18 (0.14 to 0.23)	-7.3 (-31.5 to 21.4)
		Legionella spp.	9.18 (7.85 to 10.7)	16.8 (14.7 to 19)	83.7 (57.6 to 109.8)	0.1 (0.08 to 0.13)	0.12 (0.1 to 0.16)	19.0 (-13.7 to 57.6)
		Mycoplasma spp.	15.3 (13.3 to 17.4)	17.7 (15.5 to 20)	16.2 (0.2 to 31.1)	0.07 (0.05 to 0.08)	0.05 (0.04 to 0.06)	-24.7 (-44.7 to -0.5)
		Polymicrobial	0.84 (0.55 to 1.26)	1.49 (0.95 to 2.3)	78.1 (53.3 to 106.6)	0.01 (0.01 to 0.02)	0.02 (0.01 to 0.03)	15.4 (-16.7 to 54.6)
		Pseudomonas aeruginosa	10.5 (9.17 to 11.9)	21.3 (18.7 to 23.8)	103.3 (77.6 to 128.2)	0.12 (0.1 to 0.15)	0.16 (0.12 to 0.2)	31.7 (-3.4 to 72.5)
		Respiratory syncytial virus	3.17 (2.73 to 3.64)	0.7 (0.36 to 1.18)	-78.0 (-88.9 to -62.0)	0.02 (0.01 to 0.02)	0 (0 to 0)	-87.4 (-94.2 to -77.0)
		Staphylococcus aureus	15.4 (13.6 to 17.3)	40.4 (35.8 to 45.2)	162.9 (130.6 to 195.4)	0.36 (0.3 to 0.43)	0.62 (0.48 to 0.79)	70.3 (25.6 to 122.9)
		Streptococcus pneumoniae	75.8 (67.2 to 84.9)	50.8 (45.1 to 56.8)	-32.8 (-41.3 to -24.8)	0.75 (0.61 to 0.89)	0.32 (0.26 to 0.41)	-56.5 (-68.0 to -43.3)
		Other viruses	26.4 (23.5 to 29.7)	38.4 (34 to 42.9)	45.4 (26.8 to 62.9)	0.1 (0.08 to 0.12)	0.1 (0.08 to 0.12)	-5.8 (-30.8 to 24.1)
		Other bacterial pathogens	21 (18.2 to 24.2)	25.3 (21.6 to 29.1)	20.9 (4.1 to 36.8)	0.2 (0.16 to 0.24)	0.15 (0.12 to 0.2)	-21.6 (-42.9 to 2.3)
	70+ years	*Acinetobacter baumannii*	4.15 (3.43 to 4.92)	3.39 (2.85 to 4.11)	-18.1 (-29.5 to -5.5)	0.17 (0.13 to 0.21)	0.09 (0.07 to 0.12)	-47.8 (-61.1 to -28.1)
		Chlamydia spp.	11.6 (9.99 to 13.5)	12 (10.2 to 14.2)	3.7 (-10.3 to 18.7)	0.12 (0.09 to 0.14)	0.08 (0.06 to 0.1)	-33.9 (-51.0 to -10.1)
		Enterobacter spp.	1.22 (1 to 1.47)	3.08 (2.56 to 3.69)	153.5 (117.3 to 190.3)	0.05 (0.04 to 0.06)	0.08 (0.06 to 0.1)	61.6 (19.6 to 121.4)
		Escherichia coli	4.73 (4.04 to 5.52)	10.5 (9 to 12.3)	122.3 (94.8 to 153.4)	0.19 (0.15 to 0.23)	0.27 (0.21 to 0.36)	41.7 (7.5 to 92.7)
		Fungus	2.06 (1.7 to 2.46)	3.87 (3.25 to 4.58)	88.4 (61.4 to 118.1)	0.08 (0.06 to 0.1)	0.1 (0.07 to 0.13)	20.0 (-10.9 to 66.4)
		Group B Streptococcus	2.74 (2.33 to 3.23)	5.76 (4.91 to 6.75)	110.7 (80.0 to 145.2)	0.08 (0.06 to 0.1)	0.1 (0.08 to 0.14)	34.2 (-0.2 to 85.4)
		Haemophilus influenzae	8.91 (7.73 to 10.3)	13.9 (12 to 16.3)	56.3 (35.9 to 76.4)	0.14 (0.11 to 0.16)	0.09 (0.07 to 0.12)	-32.7 (-49.6 to -9.2)
		Influenza	18.7 (16.3 to 21.3)	5.87 (2.99 to 10)	-68.6 (-84.3 to -47.0)	0.56 (0.44 to 0.67)	0.1 (0.05 to 0.19)	-82.3 (-91.5 to -67.1)
		Klebsiella pneumoniae	8.76 (7.66 to 10.1)	13 (11.2 to 15.4)	49.0 (30.9 to 68.4)	0.35 (0.28 to 0.42)	0.33 (0.26 to 0.44)	-5.1 (-28.8 to 28.7)
		Legionella spp.	4.42 (3.77 to 5.17)	8.43 (7.3 to 9.91)	91.1 (65.3 to 118.8)	0.12 (0.1 to 0.15)	0.15 (0.11 to 0.2)	21.8 (-10.0 to 68.8)
		Mycoplasma spp.	8.65 (7.5 to 9.98)	10.4 (8.93 to 12.3)	20.9 (4.7 to 37.1)	0.09 (0.07 to 0.11)	0.07 (0.05 to 0.1)	-23.0 (-42.2 to 7.3)
		Polymicrobial	0.64 (0.42 to 0.97)	1.19 (0.75 to 1.86)	85.3 (57.2 to 114.8)	0.03 (0.02 to 0.04)	0.03 (0.02 to 0.05)	18.1 (-12.3 to 64.0)
		Pseudomonas aeruginosa	7.32 (6.38 to 8.5)	15.4 (13.4 to 18.2)	111.5 (85.4 to 139.9)	0.2 (0.16 to 0.24)	0.27 (0.21 to 0.36)	34.8 (0.8 to 82.7)
		Respiratory syncytial virus	1.99 (1.71 to 2.32)	0.45 (0.23 to 0.77)	-77.1 (-88.6 to -61.2)	0.03 (0.02 to 0.03)	0 (0 to 0.01)	-87.1 (-94.0 to -76.0)

		Staphylococcus aureus	13.4 (11.7 to 15.3)	36.5 (31.6 to 42.7)	173.5 (140.9 to 206.3)	0.76 (0.6 to 0.91)	1.32 (1.02 to 1.75)	74.3 (31.0 to 136.4)
		Streptococcus pneumoniae	52.3 (46 to 59.7)	36.5 (31.8 to 42.8)	-30.1 (-38.3 to -21.9)	1.24 (0.98 to 1.47)	0.55 (0.43 to 0.73)	-55.5 (-66.6 to -40.1)
		Other viruses	18 (15.8 to 20.6)	27.2 (23.6 to 32)	51.3 (32.8 to 69.6)	0.17 (0.13 to 0.2)	0.16 (0.12 to 0.22)	-3.6 (-27.7 to 29.6)
		Other bacterial pathogens	18.8 (16.1 to 22)	23.6 (19.9 to 27.8)	25.8 (9.6 to 42.9)	0.43 (0.34 to 0.52)	0.34 (0.26 to 0.47)	-19.8 (-40.8 to 9.0)
United States of America	All Ages	*Acinetobacter baumannii*	39000 (33200 to 45200)	26600 (23400 to 30100)	-31.8 (-38.8 to -24.5)	896 (756 to 1040)	450 (372 to 518)	-49.7 (-54.9 to -44.2)
		Chlamydia spp.	181000 (164000 to 198000)	154000 (140000 to 170000)	-14.9 (-23.1 to -6.0)	1020 (882 to 1120)	638 (539 to 716)	-37.3 (-43.0 to -30.9)
		Enterobacter spp.	35400 (31000 to 39900)	67100 (60600 to 74100)	90.2 (71.5 to 111.9)	754 (641 to 859)	1060 (896 to 1180)	41.1 (27.5 to 55.3)
		Escherichia coli	94600 (85700 to 106000)	173000 (159000 to 186000)	83.0 (67.2 to 98.5)	2220 (1930 to 2450)	2940 (2500 to 3220)	32.3 (21.9 to 42.8)
		Fungus	60600 (54000 to 67900)	88700 (79100 to 98700)	46.5 (32.1 to 62.0)	1120 (976 to 1270)	1290 (1110 to 1440)	14.8 (3.4 to 26.4)
		Group B Streptococcus	75200 (67900 to 83800)	120000 (109000 to 131000)	59.4 (41.8 to 77.2)	1120 (965 to 1250)	1340 (1140 to 1490)	19.4 (7.3 to 31.9)
		Haemophilus influenzae	305000 (285000 to 326000)	345000 (319000 to 373000)	13.1 (6.3 to 20.0)	2310 (2050 to 2460)	1380 (1180 to 1500)	-40.5 (-44.0 to -36.9)
		Influenza	469000 (438000 to 505000)	156000 (72500 to 276000)	-66.8 (-84.9 to -40.9)	8050 (7020 to 8620)	1710 (783 to 3130)	-78.7 (-90.4 to -61.6)
		Klebsiella pneumoniae	192000 (180000 to 206000)	209000 (193000 to 226000)	8.7 (2.4 to 15.1)	4190 (3690 to 4480)	3440 (2910 to 3750)	-18.0 (-22.7 to -13.7)
		Legionella spp.	217000 (197000 to 238000)	282000 (261000 to 303000)	30.1 (18.9 to 42.2)	3000 (2620 to 3300)	2980 (2550 to 3250)	-0.8 (-9.2 to 8.3)
		Mycoplasma spp.	336000 (311000 to 362000)	303000 (282000 to 327000)	-9.6 (-15.9 to -2.9)	1570 (1390 to 1700)	1060 (933 to 1160)	-32.1 (-36.9 to -27.0)
		Polymicrobial	10100 (6250 to 16500)	15500 (9230 to 25600)	52.7 (35.1 to 71.2)	233 (143 to 380)	261 (152 to 435)	11.6 (-2.0 to 24.1)
		Pseudomonas aeruginosa	277000 (256000 to 302000)	403000 (374000 to 433000)	45.4 (35.2 to 55.6)	4230 (3720 to 4590)	4510 (3830 to 4890)	6.4 (-0.8 to 13.5)
		Respiratory syncytial virus	168000 (156000 to 182000)	32500 (15300 to 57100)	-80.7 (-91.2 to -65.8)	876 (787 to 948)	107 (48.3 to 194)	-87.8 (-94.4 to -78.1)
		Staphylococcus aureus	369000 (345000 to 396000)	753000 (698000 to 811000)	104.1 (93.8 to 115.1)	12100 (10600 to 12900)	18100 (15300 to 19600)	49.4 (43.0 to 56.3)
		Streptococcus pneumoniae	1610000 (1520000 to 1720000)	702000 (655000 to 754000)	-56.5 (-58.6 to -54.3)	18400 (16400 to 19500)	6350 (5440 to 6870)	-65.5 (-67.2 to -63.9)
		Other viruses	1000000 (946000 to 1070000)	874000 (815000 to 939000)	-12.6 (-17.1 to -8.0)	4220 (3770 to 4480)	2970 (2570 to 3210)	-29.6 (-33.1 to -26.3)
		Other bacterial pathogens	343000 (301000 to 391000)	341000 (298000 to 388000)	-0.5 (-8.6 to 8.5)	4660 (3920 to 5340)	3350 (2730 to 3900)	-28.0 (-33.8 to -21.9)
	Under 5	*Acinetobacter baumannii*	543 (338 to 863)	316 (185 to 517)	-42.0 (-49.4 to -33.9)	8.6 (4.89 to 14.1)	2.31 (1.27 to 3.88)	-73.3 (-76.9 to -69.5)
		Chlamydia spp.	5940 (5080 to 6870)	3840 (3220 to 4490)	-35.1 (-44.2 to -25.4)	14.1 (12.9 to 15.4)	4.34 (3.69 to 5.03)	-69.1 (-73.7 to -64.5)

		Enterobacter spp.	1000 (752 to 1300)	1250 (964 to 1610)	25.6 (7.1 to 47.8)	9.67 (7.08 to 13.3)	5.14 (3.91 to 6.55)	-46.3 (-56.2 to -35.0)
		Escherichia coli	2730 (2310 to 3240)	3920 (3260 to 4640)	43.9 (23.4 to 64.4)	25.7 (23.2 to 28.7)	15.8 (13.4 to 18.3)	-38.6 (-48.3 to -27.9)
		Fungus	467 (329 to 645)	510 (365 to 704)	9.8 (-9.6 to 29.8)	3.09 (2.25 to 4.11)	1.78 (1.24 to 2.44)	-42.2 (-53.5 to -29.0)
		Group B Streptococcus	2740 (2310 to 3270)	3010 (2510 to 3580)	10.0 (-4.9 to 26.8)	20.9 (18.8 to 23.2)	9.56 (8.1 to 11)	-54.2 (-61.0 to -47.1)
		Haemophilus influenzae	23300 (19800 to 27300)	17100 (14500 to 20100)	-26.6 (-36.2 to -16.1)	67.8 (62.6 to 73.8)	16 (13.6 to 18.6)	-76.4 (-80.2 to -72.1)
		Influenza	18400 (15700 to 21400)	3480 (1650 to 6070)	-81.1 (-91.0 to -67.5)	101 (94.8 to 108)	8.26 (3.74 to 15.2)	-91.8 (-96.3 to -84.8)
		Klebsiella pneumoniae	9240 (7930 to 10700)	5460 (4620 to 6410)	-40.8 (-48.0 to -32.9)	70.6 (66 to 76)	21.4 (18.4 to 24.4)	-69.7 (-74.1 to -64.9)
		Legionella spp.	1590 (1170 to 2100)	1470 (1060 to 1980)	-7.4 (-19.3 to 5.0)	12.2 (9.71 to 15.2)	4.93 (3.79 to 6.26)	-59.7 (-65.4 to -52.7)
		Mycoplasma spp.	12600 (10700 to 14900)	14200 (12000 to 16600)	13.1 (-1.0 to 28.3)	24.6 (22.5 to 27.2)	13.8 (11.7 to 15.8)	-43.9 (-53.0 to -34.1)
		Polymicrobial	769 (412 to 1290)	601 (330 to 1010)	-21.5 (-31.4 to -10.5)	13.8 (7.09 to 23.2)	4.52 (2.32 to 7.65)	-67.2 (-71.5 to -62.5)
		Pseudomonas aeruginosa	8110 (6940 to 9510)	11000 (9330 to 12800)	35.5 (17.5 to 54.0)	47.5 (43.6 to 51.7)	29.4 (25.5 to 33.3)	-38.0 (-47.4 to -28.9)
		Respiratory syncytial virus	63800 (55700 to 73000)	11400 (5350 to 20100)	-82.1 (-91.5 to -69.0)	171 (162 to 180)	12.9 (5.88 to 23.8)	-92.5 (-96.5 to -86.1)
		Staphylococcus aureus	8300 (7140 to 9580)	11700 (9970 to 13600)	41.5 (26.3 to 57.0)	89 (83.6 to 94.4)	61.4 (53.1 to 70.2)	-30.9 (-40.9 to -21.0)
		Streptococcus pneumoniae	114000 (97800 to 131000)	23300 (19800 to 27200)	-79.5 (-81.5 to -77.4)	462 (442 to 484)	48.6 (41.9 to 55.2)	-89.5 (-91.0 to -87.9)
		Other viruses	122000 (105000 to 140000)	61300 (52400 to 71100)	-49.9 (-54.7 to -44.8)	199 (190 to 209)	52.7 (45.7 to 59.4)	-73.6 (-77.4 to -69.8)
		Other bacterial pathogens	2740 (2130 to 3440)	2140 (1690 to 2740)	-21.4 (-33.3 to -7.1)	13.6 (10.7 to 17.4)	5.17 (4.06 to 6.62)	-61.9 (-67.8 to -54.9)
	5-14 years	*Acinetobacter baumannii*	622 (443 to 851)	338 (264 to 432)	-45.1 (-53.4 to -36.2)	1.49 (1.27 to 1.74)	0.66 (0.57 to 0.76)	-55.5 (-60.9 to -49.6)
		Chlamydia spp.	4060 (3000 to 5400)	2970 (2340 to 3780)	-26.2 (-36.5 to -14.5)	2.42 (2.21 to 2.65)	1.45 (1.29 to 1.62)	-40.2 (-46.9 to -32.3)
		Enterobacter spp.	579 (413 to 793)	920 (719 to 1170)	60.5 (38.0 to 88.6)	1.32 (1.15 to 1.5)	1.71 (1.51 to 1.93)	30.0 (14.9 to 45.9)
		Escherichia coli	1230 (898 to 1650)	1990 (1570 to 2510)	62.6 (39.8 to 86.2)	2.91 (2.67 to 3.16)	3.83 (3.5 to 4.17)	31.8 (17.4 to 44.9)
		Fungus	1480 (1080 to 2000)	1810 (1430 to 2320)	23.3 (4.7 to 44.8)	3.44 (3.11 to 3.81)	3.43 (3.02 to 3.88)	-0.1 (-12.3 to 12.7)
		Group B Streptococcus	1700 (1230 to 2260)	2350 (1860 to 3000)	39.5 (17.8 to 64.5)	2.84 (2.56 to 3.16)	3.21 (2.86 to 3.59)	13.1 (-1.1 to 27.5)
		Haemophilus influenzae	7610 (5690 to 10000)	6940 (5480 to 8700)	-8.3 (-19.5 to 4.1)	6.84 (6.48 to 7.2)	3.48 (3.18 to 3.77)	-49.1 (-53.6 to -44.6)
		Influenza	10300 (7720 to 13500)	2690 (1190 to 4940)	-73.8 (-87.7 to -52.2)	18.2 (17.4 to 19)	3.34 (1.51 to 6.05)	-81.6 (-91.8 to -66.9)

		Klebsiella pneumoniae	3600 (2680 to 4700)	2840 (2270 to 3520)	-20.7 (-30.0 to -10.4)	8.43 (8.02 to 8.85)	5.42 (5.01 to 5.8)	-35.7 (-40.9 to -30.7)
		Legionella spp.	4870 (3630 to 6480)	5380 (4280 to 6720)	11.2 (-4.4 to 28.0)	7.86 (7.26 to 8.55)	7.07 (6.49 to 7.67)	-9.9 (-19.4 to 0.0)
		Mycoplasma spp.	16200 (11900 to 21200)	14700 (11700 to 18500)	-8.7 (-20.1 to 4.6)	10.1 (9.49 to 10.7)	7.44 (6.84 to 8.03)	-26.0 (-32.8 to -19.0)
		Polymicrobial	164 (94.1 to 265)	213 (121 to 344)	30.0 (9.3 to 52.3)	0.39 (0.23 to 0.64)	0.41 (0.24 to 0.68)	5.3 (-8.2 to 20.7)
		Pseudomonas aeruginosa	5010 (3720 to 6590)	6640 (5340 to 8260)	33.5 (16.4 to 51.7)	8.15 (7.66 to 8.67)	8.8 (8.16 to 9.43)	8.2 (-1.0 to 18.1)
		Respiratory syncytial virus	3940 (2960 to 5220)	722 (322 to 1330)	-81.6 (-91.3 to -66.8)	3.16 (2.93 to 3.39)	0.41 (0.19 to 0.74)	-87.1 (-94.0 to -76.7)
		Staphylococcus aureus	6620 (4940 to 8680)	11100 (8940 to 13800)	68.4 (49.7 to 90.3)	22 (21.2 to 22.9)	30.1 (28 to 31.9)	36.5 (26.6 to 46.6)
		Streptococcus pneumoniae	55800 (41700 to 72500)	18400 (14700 to 22800)	-66.7 (-70.6 to -62.6)	77.3 (75 to 79.7)	20.8 (19.4 to 22.2)	-73.0 (-75.1 to -71.1)
		Other viruses	36300 (27100 to 47200)	25000 (20300 to 31000)	-30.7 (-38.8 to -22.0)	19.8 (19.1 to 20.5)	11.1 (10.3 to 11.8)	-43.9 (-47.9 to -39.6)
		Other bacterial pathogens	5150 (3750 to 6900)	4330 (3360 to 5530)	-15.5 (-26.7 to -3.2)	6.82 (6.06 to 7.64)	4.67 (4.04 to 5.34)	-31.4 (-38.4 to -23.9)
	15-49 years	*Acinetobacter baumannii*	3440 (2800 to 4120)	1780 (1500 to 2090)	-48.2 (-54.6 to -41.3)	26.9 (23 to 31.2)	13.1 (11.3 to 14.9)	-51.2 (-56.7 to -45.4)
		Chlamydia spp.	22500 (19700 to 25800)	15600 (13700 to 17700)	-30.3 (-37.7 to -22.9)	43.6 (39.8 to 47.7)	28.6 (25.5 to 31.9)	-34.3 (-41.1 to -26.7)
		Enterobacter spp.	3200 (2690 to 3770)	4840 (4160 to 5570)	51.5 (35.2 to 70.4)	23.7 (20.7 to 27)	33.7 (29.9 to 37.8)	42.7 (26.9 to 60.0)
		Escherichia coli	6830 (5920 to 7830)	10500 (9340 to 11700)	53.6 (38.8 to 68.1)	52.4 (47.8 to 56.9)	75.6 (70.4 to 81.2)	44.6 (31.5 to 58.9)
		Fungus	8190 (7030 to 9450)	9520 (8230 to 11000)	16.4 (3.3 to 30.6)	61.9 (55.8 to 68.6)	67.7 (60.3 to 76.1)	9.6 (-2.4 to 22.4)
		Group B Streptococcus	9410 (8140 to 11000)	12400 (10800 to 14100)	31.8 (16.0 to 47.9)	51.2 (46.1 to 56.9)	63.4 (57 to 70.1)	24.1 (10.5 to 39.2)
		Haemophilus influenzae	42100 (37200 to 47400)	36500 (32600 to 40700)	-13.3 (-19.4 to -6.6)	123 (117 to 129)	68.7 (63.8 to 73.5)	-44.2 (-48.5 to -39.8)
		Influenza	57200 (50600 to 64400)	14200 (6480 to 25600)	-75.2 (-89.0 to -55.7)	327 (314 to 341)	66 (29.7 to 119)	-79.8 (-90.9 to -64.0)
		Klebsiella pneumoniae	19900 (17700 to 22400)	14900 (13300 to 16600)	-25.1 (-30.4 to -19.8)	152 (145 to 159)	107 (100 to 114)	-29.5 (-34.2 to -24.5)
		Legionella spp.	27000 (23500 to 30800)	28300 (25300 to 31500)	5.0 (-5.9 to 16.0)	142 (131 to 154)	140 (130 to 150)	-1.1 (-10.7 to 8.9)
		Mycoplasma spp.	89600 (79500 to 101000)	77100 (68700 to 86200)	-13.8 (-20.5 to -7.2)	181 (171 to 192)	147 (138 to 157)	-18.8 (-25.3 to -12.4)
		Polymicrobial	912 (530 to 1480)	1120 (652 to 1840)	22.8 (7.9 to 38.1)	7.08 (4.16 to 11.5)	8.18 (4.77 to 13.4)	15.6 (2.1 to 31.4)
		Pseudomonas aeruginosa	27700 (24300 to 31600)	34900 (31300 to 38600)	26.1 (16.9 to 36.4)	147 (137 to 156)	174 (164 to 184)	18.7 (10.2 to 28.2)
		Respiratory syncytial virus	21800 (19300 to 24800)	3800 (1740 to 6850)	-82.6 (-92.1 to -68.6)	57 (52.9 to 61.1)	8.08 (3.71 to 14.7)	-85.8 (-93.6 to -74.5)
		Staphylococcus aureus	36600 (32600 to 41200)	58200 (52400 to 64000)	59.1 (50.1 to 68.1)	397 (382 to 411)	594 (566 to 625)	49.8 (41.9 to 58.1)
		Streptococcus pneumoniae	309000 (276000 to 346000)	97000 (87300 to 107000)	-68.6 (-70.4 to -66.9)	1390 (1350 to 1430)	411 (391 to 431)	-70.4 (-72.1 to -68.8)

		Other viruses	201000 (179000 to 225000)	131000 (118000 to 145000)	-34.6 (-38.5 to -31.0)	356 (345 to 368)	219 (208 to 230)	-38.4 (-41.9 to -35.0)
		Other bacterial pathogens	28500 (24000 to 33300)	22800 (19300 to 26400)	-20.1 (-27.5 to -12.3)	123 (109 to 138)	92.3 (80.7 to 104)	-24.8 (-31.7 to -17.4)
	50-69 years	*Acinetobacter baumannii*	12800 (10800 to 14900)	8910 (7730 to 10200)	-30.4 (-37.7 to -23.3)	131 (113 to 150)	94.9 (83.7 to 106)	-27.4 (-34.7 to -19.3)
		Chlamydia spp.	49600 (44000 to 56100)	43300 (38800 to 48400)	-12.4 (-21.0 to -3.3)	126 (115 to 137)	115 (105 to 126)	-8.6 (-17.1 to 1.4)
		Enterobacter spp.	11600 (10000 to 13200)	22600 (20100 to 25100)	95.1 (75.8 to 117.0)	112 (99.2 to 126)	228 (207 to 249)	103.6 (83.2 to 125.1)
		Escherichia coli	27300 (24300 to 30600)	50500 (45900 to 55000)	84.9 (69.8 to 100.4)	273 (253 to 294)	527 (493 to 557)	93.0 (78.0 to 108.2)
		Fungus	26100 (22700 to 29500)	39400 (34500 to 44100)	51.3 (36.7 to 67.5)	257 (233 to 284)	405 (365 to 447)	57.9 (41.9 to 74.2)
		Group B Streptococcus	24100 (21200 to 27500)	39600 (35200 to 44100)	64.8 (46.3 to 83.3)	171 (156 to 189)	294 (264 to 322)	72.0 (54.6 to 90.1)
		Haemophilus influenzae	92100 (83300 to 100000)	112000 (102000 to 122000)	21.4 (13.9 to 28.9)	351 (334 to 367)	305 (287 to 323)	-13.2 (-18.6 to -8.0)
		Influenza	108000 (98100 to 118000)	37600 (17200 to 66600)	-65.0 (-84.2 to -37.1)	804 (766 to 837)	254 (114 to 458)	-68.4 (-85.7 to -43.5)
		Klebsiella pneumoniae	55800 (51000 to 60900)	64300 (59000 to 69900)	15.3 (8.7 to 21.9)	555 (531 to 579)	667 (628 to 704)	20.3 (13.4 to 27.1)
		Legionella spp.	82900 (74000 to 92000)	111000 (101000 to 121000)	33.5 (21.9 to 45.6)	568 (525 to 611)	790 (743 to 838)	39.3 (27.7 to 51.8)
		Mycoplasma spp.	89800 (80800 to 98800)	80700 (73200 to 88500)	-10.0 (-16.4 to -2.7)	237 (222 to 253)	223 (208 to 238)	-6.1 (-13.4 to 1.7)
		Polymicrobial	2830 (1700 to 4600)	4590 (2700 to 7490)	62.0 (43.8 to 80.9)	28.7 (17 to 46.4)	48.5 (29 to 79)	69.0 (50.9 to 87.4)
		Pseudomonas aeruginosa	85900 (77500 to 94800)	126000 (115000 to 136000)	46.7 (36.7 to 56.7)	593 (557 to 632)	908 (856 to 955)	53.1 (42.4 to 64.2)
		Respiratory syncytial virus	30400 (27100 to 33800)	6350 (2930 to 11300)	-79.1 (-90.5 to -62.3)	104 (94.2 to 114)	19.5 (8.92 to 35.4)	-81.1 (-91.4 to -66.0)
		Staphylococcus aureus	99900 (91600 to 108000)	209000 (191000 to 227000)	109.4 (99.1 to 119.6)	1410 (1360 to 1460)	3080 (2920 to 3230)	118.5 (108.1 to 129.3)
		Streptococcus pneumoniae	416000 (380000 to 451000)	204000 (186000 to 222000)	-50.9 (-53.3 to -48.3)	2440 (2360 to 2520)	1250 (1190 to 1310)	-48.8 (-51.4 to -46.2)
		Other viruses	237000 (216000 to 257000)	240000 (219000 to 261000)	1.4 (-3.5 to 6.6)	547 (526 to 567)	579 (546 to 609)	5.8 (0.5 to 11.3)
		Other bacterial pathogens	94200 (80600 to 107000)	94700 (81600 to 108000)	0.7 (-7.2 to 9.4)	529 (472 to 591)	556 (489 to 630)	5.1 (-3.6 to 13.7)
	70+ years	*Acinetobacter baumannii*	21600 (18100 to 25500)	15200 (13200 to 17500)	-29.3 (-36.7 to -21.5)	728 (601 to 853)	340 (267 to 396)	-53.3 (-58.5 to -48.1)
		Chlamydia spp.	99300 (88100 to 112000)	88300 (78700 to 100000)	-11.0 (-20.1 to -0.8)	832 (703 to 924)	489 (398 to 557)	-41.3 (-46.8 to -35.0)
		Enterobacter spp.	19000 (16200 to 22100)	37600 (33100 to 42100)	98.3 (77.6 to 121.8)	607 (503 to 701)	794 (638 to 903)	30.9 (17.6 to 44.0)
		Escherichia coli	56400 (50200 to 64600)	106000 (96000 to 117000)	88.0 (70.9 to 105.4)	1870 (1590 to 2070)	2320 (1900 to 2570)	24.1 (13.4 to 34.4)
		Fungus	24400 (21400 to 28100)	37500 (32700 to 42600)	53.8 (37.6 to 70.7)	797 (670 to 920)	809 (649 to 931)	1.5 (-9.1 to 12.2)

		Group B Streptococcus	37200 (32900 to 42000)	62200 (55300 to 69700)	67.5 (48.9 to 87.0)	874 (724 to 985)	966 (787 to 1100)	10.6 (-1.4 to 22.6)
		Haemophilus influenzae	140000 (127000 to 155000)	172000 (156000 to 190000)	23.5 (14.7 to 32.5)	1760 (1510 to 1910)	984 (801 to 1090)	-44.2 (-48.1 to -40.6)
		Influenza	275000 (251000 to 305000)	97700 (46100 to 172000)	-64.5 (-84.0 to -36.9)	6800 (5800 to 7360)	1380 (624 to 2540)	-79.7 (-90.8 to -63.3)
		Klebsiella pneumoniae	103000 (94200 to 115000)	121000 (110000 to 133000)	17.2 (9.4 to 25.2)	3400 (2920 to 3670)	2630 (2130 to 2920)	-22.7 (-27.8 to -18.2)
		Legionella spp.	100000 (89700 to 113000)	136000 (124000 to 150000)	35.7 (23.1 to 50.0)	2270 (1920 to 2520)	2030 (1650 to 2260)	-10.5 (-18.6 to -2.0)
		Mycoplasma spp.	128000 (115000 to 143000)	117000 (105000 to 129000)	-8.5 (-15.6 to -0.4)	1120 (948 to 1230)	674 (548 to 754)	-39.6 (-44.5 to -34.6)
		Polymicrobial	5450 (3220 to 9170)	8980 (5160 to 14900)	64.6 (45.6 to 85.4)	183 (106 to 307)	199 (113 to 339)	8.7 (-3.8 to 20.9)
		Pseudomonas aeruginosa	150000 (136000 to 169000)	224000 (203000 to 247000)	49.2 (38.1 to 61.3)	3440 (2950 to 3760)	3390 (2740 to 3730)	-1.6 (-9.3 to 5.4)
		Respiratory syncytial virus	48000 (42700 to 54700)	10200 (4760 to 18100)	-78.8 (-90.3 to -61.9)	541 (460 to 605)	65.6 (29.8 to 120)	-87.9 (-94.4 to -78.2)
		Staphylococcus aureus	217000 (198000 to 241000)	463000 (422000 to 508000)	112.8 (100.6 to 126.4)	10200 (8710 to 10900)	14300 (11600 to 15700)	40.5 (33.2 to 47.4)
		Streptococcus pneumoniae	719000 (657000 to 799000)	359000 (328000 to 396000)	-50.1 (-53.1 to -46.7)	14000 (12100 to 15000)	4620 (3740 to 5090)	-67.1 (-69.1 to -65.4)
		Other viruses	405000 (370000 to 451000)	417000 (379000 to 460000)	3.1 (-2.8 to 10.0)	3100 (2670 to 3340)	2110 (1720 to 2330)	-32.0 (-35.8 to -28.4)
		Other bacterial pathogens	212000 (183000 to 246000)	217000 (189000 to 250000)	2.3 (-6.3 to 12.4)	3990 (3290 to 4600)	2690 (2100 to 3170)	-32.4 (-38.2 to -26.4)
Southern Latin America	All Ages	*Acinetobacter baumannii*	14900 (13000 to 17000)	15900 (14000 to 17900)	6.7 (-4.2 to 16.6)	450 (400 to 510)	829 (714 to 946)	84.3 (67.3 to 103.0)
		Chlamydia spp.	49600 (45200 to 54500)	51200 (47000 to 55300)	3.3 (-5.4 to 12.4)	342 (311 to 371)	616 (538 to 675)	80.1 (64.5 to 97.9)
		Enterobacter spp.	5270 (4560 to 6100)	13300 (11900 to 14900)	152.9 (129.4 to 176.5)	145 (125 to 167)	618 (532 to 696)	327.6 (283.4 to 371.0)
		Escherichia coli	17600 (15800 to 19500)	37000 (33900 to 40300)	110.9 (91.9 to 128.5)	533 (478 to 586)	1890 (1670 to 2080)	255.7 (221.1 to 288.8)
		Fungus	11900 (10400 to 13500)	22400 (20100 to 24900)	89.1 (72.1 to 107.2)	276 (243 to 313)	909 (795 to 1020)	229.4 (196.0 to 262.9)
		Group B Streptococcus	13600 (12200 to 15200)	27800 (25400 to 30200)	104.7 (86.3 to 124.9)	257 (231 to 286)	883 (778 to 968)	243.5 (209.4 to 278.2)
		Haemophilus influenzae	57900 (53100 to 63100)	71200 (66100 to 76600)	23.1 (13.2 to 32.5)	513 (479 to 547)	784 (701 to 850)	52.8 (40.9 to 64.0)
		Influenza	79500 (73900 to 85200)	14200 (6340 to 26500)	-82.2 (-91.9 to -66.7)	1630 (1510 to 1720)	509 (226 to 983)	-68.6 (-85.9 to -39.7)
		Klebsiella pneumoniae	41100 (38200 to 44400)	59600 (55400 to 64000)	45.1 (34.7 to 55.7)	1080 (1010 to 1150)	2870 (2560 to 3110)	165.8 (144.9 to 185.0)
		Legionella spp.	22300 (20000 to 24900)	47900 (44400 to 51700)	115.4 (96.3 to 135.8)	398 (358 to 441)	1440 (1280 to 1570)	262.6 (224.9 to 297.8)
		Mycoplasma spp.	66200 (60700 to 71900)	83800 (78500 to 89500)	26.8 (17.2 to 36.1)	348 (323 to 374)	731 (658 to 792)	110.4 (93.2 to 127.7)

		Polymicrobial	2520 (1730 to 3680)	4360 (2770 to 6820)	72.3 (50.5 to 94.2)	82.9 (59.2 to 120)	226 (143 to 354)	171.2 (125.7 to 215.6)
		Pseudomonas aeruginosa	32600 (30100 to 35400)	80000 (74400 to 86000)	145.4 (128.1 to 164.0)	628 (583 to 672)	2580 (2310 to 2790)	310.9 (275.0 to 345.1)
		Respiratory syncytial virus	42300 (37100 to 48100)	4230 (1940 to 7860)	-90.0 (-95.4 to -81.4)	277 (263 to 292)	32 (14.1 to 60.9)	-88.4 (-94.9 to -77.8)
		Staphylococcus aureus	52100 (48800 to 55700)	144000 (134000 to 154000)	176.1 (158.5 to 192.9)	2130 (1980 to 2250)	10200 (9050 to 11000)	378.0 (345.3 to 409.7)
		Streptococcus pneumoniae	355000 (332000 to 382000)	251000 (236000 to 267000)	-29.2 (-33.8 to -24.9)	4500 (4260 to 4670)	6070 (5450 to 6520)	34.9 (25.1 to 43.4)
		Other viruses	165000 (152000 to 179000)	200000 (188000 to 213000)	21.4 (12.6 to 30.1)	761 (725 to 793)	1720 (1540 to 1840)	125.3 (108.0 to 140.2)
		Other bacterial pathogens	62500 (56200 to 68800)	89000 (78800 to 99200)	42.6 (31.5 to 53.7)	1100 (996 to 1210)	2680 (2280 to 3030)	143.1 (122.1 to 164.6)
	Under 5	*Acinetobacter baumannii*	539 (381 to 727)	149 (107 to 203)	-72.3 (-75.9 to -68.1)	19.4 (12.5 to 29)	3.83 (2.21 to 6.46)	-80.4 (-85.4 to -74.7)
		Chlamydia spp.	7090 (5910 to 8450)	2440 (2000 to 2960)	-65.5 (-71.0 to -60.1)	36 (32.7 to 39.5)	5.53 (4.41 to 6.96)	-84.6 (-87.8 to -80.6)
		Enterobacter spp.	655 (492 to 841)	512 (384 to 672)	-21.5 (-34.0 to -7.1)	13.7 (10.5 to 18.1)	4.04 (2.92 to 5.6)	-70.4 (-76.7 to -62.9)
		Escherichia coli	2370 (1930 to 2850)	1800 (1480 to 2190)	-23.7 (-35.4 to -11.5)	47.7 (41.9 to 53.6)	13 (10.5 to 16.1)	-72.6 (-78.3 to -65.6)
		Fungus	474 (324 to 662)	291 (205 to 398)	-38.2 (-48.8 to -26.3)	6.87 (4.87 to 9.16)	1.42 (0.97 to 1.97)	-79.3 (-84.3 to -73.6)
		Group B Streptococcus	1990 (1630 to 2400)	1340 (1080 to 1630)	-32.3 (-42.6 to -21.5)	34.6 (30.7 to 38.8)	9.11 (7.37 to 11.3)	-73.6 (-79.2 to -66.6)
		Haemophilus influenzae	17500 (14500 to 20800)	7570 (6240 to 9090)	-56.7 (-62.6 to -50.4)	113 (103 to 125)	10.3 (8.27 to 12.8)	-90.8 (-92.7 to -88.4)
		Influenza	14800 (12500 to 17300)	707 (321 to 1310)	-95.2 (-97.8 to -91.1)	180 (167 to 194)	2.91 (1.22 to 5.76)	-98.4 (-99.3 to -96.8)
		Klebsiella pneumoniae	8950 (7530 to 10500)	3370 (2780 to 4060)	-62.3 (-66.9 to -56.8)	149 (138 to 162)	21.9 (17.6 to 27)	-85.3 (-88.2 to -81.7)
		Legionella spp.	715 (501 to 1010)	460 (310 to 666)	-35.6 (-45.2 to -25.0)	13.7 (10.1 to 17.6)	4 (2.86 to 5.49)	-70.6 (-76.5 to -63.0)
		Mycoplasma spp.	10100 (8510 to 12000)	7460 (6180 to 9020)	-26.1 (-36.1 to -15.1)	44.9 (40.6 to 49.7)	11.1 (8.97 to 13.8)	-75.1 (-80.3 to -69.1)
		Polymicrobial	315 (200 to 473)	132 (82.5 to 202)	-58.0 (-65.0 to -50.3)	16 (9.32 to 25.4)	4.98 (2.58 to 8.47)	-69.0 (-76.6 to -60.2)
		Pseudomonas aeruginosa	4530 (3760 to 5380)	4630 (3820 to 5610)	2.4 (-11.9 to 16.8)	59.6 (54.4 to 65.6)	20.6 (16.6 to 25.4)	-65.4 (-72.4 to -57.5)
		Respiratory syncytial virus	31700 (26900 to 36900)	2230 (1030 to 4150)	-93.0 (-96.7 to -86.9)	192 (180 to 205)	4.56 (1.94 to 8.88)	-97.6 (-99.0 to -95.3)
		Staphylococcus aureus	5990 (5060 to 6960)	5060 (4170 to 6060)	-15.4 (-24.5 to -4.9)	139 (129 to 149)	40.3 (32.5 to 49.2)	-70.9 (-76.7 to -64.3)
		Streptococcus pneumoniae	99100 (84100 to 115000)	17800 (14800 to 21600)	-82.0 (-84.0 to -79.8)	877 (827 to 935)	53.6 (43.3 to 65.6)	-93.9 (-95.1 to -92.4)
		Other viruses	67300 (57100 to 78400)	27700 (23100 to 33400)	-58.8 (-63.1 to -54.0)	241 (227 to 258)	36 (29.1 to 44)	-85.1 (-88.0 to -81.7)

		Other bacterial pathogens	2730 (2140 to 3430)	1240 (959 to 1570)	-54.5 (-61.3 to -46.7)	28.9 (23.3 to 35.3)	5.13 (3.82 to 6.9)	-82.2 (-86.1 to -77.5)
	5-14 years	*Acinetobacter baumannii*	1040 (775 to 1350)	519 (398 to 670)	-49.9 (-55.4 to -44.2)	2.56 (2.2 to 2.95)	1.34 (1.13 to 1.56)	-47.5 (-55.2 to -39.3)
		Chlamydia spp.	4240 (3240 to 5490)	2400 (1830 to 3090)	-43.3 (-48.9 to -37.2)	2.59 (2.32 to 2.89)	1.52 (1.33 to 1.73)	-41.3 (-49.5 to -32.4)
		Enterobacter spp.	344 (255 to 453)	458 (345 to 600)	33.6 (18.7 to 49.3)	0.8 (0.69 to 0.93)	1.09 (0.94 to 1.27)	36.4 (16.5 to 57.0)
		Escherichia coli	943 (718 to 1220)	1050 (802 to 1330)	11.2 (-0.4 to 22.6)	2.27 (2.03 to 2.53)	2.58 (2.29 to 2.91)	13.8 (-1.0 to 29.4)
		Fungus	1140 (845 to 1480)	1120 (851 to 1450)	-1.5 (-12.1 to 9.7)	2.71 (2.35 to 3.08)	2.73 (2.34 to 3.14)	1.2 (-12.8 to 16.1)
		Group B Streptococcus	1150 (866 to 1480)	1320 (1010 to 1670)	14.7 (2.4 to 27.5)	1.97 (1.73 to 2.24)	2.31 (2.03 to 2.62)	17.6 (0.7 to 36.5)
		Haemophilus influenzae	4500 (3480 to 5810)	3350 (2560 to 4250)	-25.5 (-31.9 to -19.5)	4.14 (3.81 to 4.5)	2.19 (1.94 to 2.44)	-47.0 (-53.1 to -40.0)
		Influenza	6480 (5070 to 8230)	618 (264 to 1190)	-90.5 (-95.6 to -81.9)	11.7 (10.8 to 12.5)	1.11 (0.5 to 2.17)	-90.4 (-95.7 to -81.4)
		Klebsiella pneumoniae	2780 (2140 to 3590)	2080 (1600 to 2680)	-25.0 (-30.7 to -19.0)	6.65 (6.15 to 7.19)	5.14 (4.61 to 5.72)	-22.7 (-31.8 to -12.4)
		Legionella spp.	2040 (1560 to 2630)	2260 (1740 to 2900)	11.3 (0.0 to 23.3)	3.36 (2.99 to 3.77)	3.79 (3.37 to 4.23)	13.2 (-2.1 to 29.6)
		Mycoplasma spp.	10100 (7800 to 12900)	7990 (6190 to 10200)	-20.9 (-27.9 to -14.0)	6.44 (5.93 to 6.96)	5.22 (4.65 to 5.81)	-18.9 (-29.0 to -7.7)
		Polymicrobial	173 (109 to 260)	155 (94.6 to 239)	-10.8 (-21.1 to 1.1)	0.42 (0.28 to 0.64)	0.39 (0.24 to 0.6)	-7.9 (-22.3 to 8.3)
		Pseudomonas aeruginosa	2290 (1760 to 2910)	3090 (2390 to 3950)	34.8 (23.0 to 47.2)	3.81 (3.48 to 4.16)	5.23 (4.7 to 5.81)	37.4 (20.9 to 55.7)
		Respiratory syncytial virus	1550 (1200 to 2000)	154 (65.4 to 296)	-90.1 (-95.4 to -81.4)	1.27 (1.16 to 1.39)	0.13 (0.06 to 0.24)	-90.0 (-95.6 to -80.8)
		Staphylococcus aureus	3780 (2960 to 4820)	5350 (4120 to 6740)	41.7 (31.5 to 51.9)	12.9 (12.1 to 13.8)	18.6 (16.7 to 20.6)	44.6 (28.2 to 62.4)
		Streptococcus pneumoniae	37200 (29200 to 47200)	16400 (12700 to 20900)	-55.9 (-59.1 to -52.7)	52.8 (49.6 to 55.9)	24.1 (21.7 to 26.6)	-54.4 (-59.7 to -48.7)
		Other viruses	15300 (11900 to 19300)	13100 (10200 to 16800)	-14.5 (-20.4 to -8.5)	8.51 (7.94 to 9.07)	7.43 (6.7 to 8.23)	-12.7 (-22.5 to -2.2)
		Other bacterial pathogens	4150 (3170 to 5370)	2850 (2180 to 3650)	-31.3 (-37.5 to -24.7)	5.61 (5.04 to 6.2)	3.97 (3.41 to 4.56)	-29.2 (-38.2 to -19.7)
	15-49 years	*Acinetobacter baumannii*	1870 (1570 to 2220)	1660 (1430 to 1940)	-10.9 (-20.9 to 0.5)	18.8 (16.4 to 21.6)	22.1 (19.1 to 25.4)	17.5 (4.4 to 31.5)
		Chlamydia spp.	7660 (6670 to 8730)	7600 (6740 to 8600)	-0.6 (-10.4 to 9.4)	19.2 (17.3 to 21.3)	25 (22.4 to 27.9)	30.4 (17.5 to 45.7)
		Enterobacter spp.	623 (517 to 733)	1430 (1240 to 1640)	130.2 (105.2 to 157.4)	5.97 (5.14 to 6.9)	17.9 (15.6 to 20.3)	200.5 (166.7 to 238.0)
		Escherichia coli	1710 (1470 to 1960)	3280 (2920 to 3650)	92.5 (73.9 to 114.5)	16.9 (15.2 to 18.7)	42.5 (38.8 to 46.4)	151.7 (124.5 to 180.3)
		Fungus	2060 (1750 to 2400)	3510 (3070 to 4020)	71.0 (52.5 to 90.4)	20.1 (17.5 to 22.8)	45 (39.4 to 50.8)	123.8 (98.9 to 151.0)
		Group B Streptococcus	2080 (1820 to 2420)	4130 (3650 to 4700)	98.6 (76.9 to 121.0)	14.7 (13 to 16.6)	38 (34.2 to 42.2)	159.5 (130.1 to 189.4)
		Haemophilus influenzae	8150 (7220 to 9170)	10500 (9430 to 11600)	28.5 (17.3 to 39.4)	30.9 (28.5 to 33.4)	36 (32.9 to 39.3)	16.8 (6.7 to 27.6)

		Influenza	11700 (10500 to 13100)	1990 (892 to 3790)	-83.1 (-92.4 to -68.2)	86.9 (81.6 to 92.5)	18.4 (8.22 to 35.6)	-78.8 (-90.8 to -59.0)
		Klebsiella pneumoniae	5020 (4480 to 5600)	6550 (5920 to 7290)	30.6 (20.5 to 41.8)	49.5 (46.1 to 52.9)	84.6 (78 to 91.7)	71.0 (55.6 to 87.0)
		Legionella spp.	3700 (3180 to 4240)	7020 (6300 to 7820)	90.5 (70.7 to 112.1)	25.2 (22.4 to 28.2)	62.2 (56.6 to 67.8)	147.9 (119.7 to 176.5)
		Mycoplasma spp.	18300 (16300 to 20500)	25000 (22600 to 27700)	36.9 (24.7 to 48.6)	48 (44.5 to 51.8)	85.8 (79.1 to 93.1)	78.9 (61.4 to 96.4)
		Polymicrobial	313 (202 to 464)	489 (303 to 760)	56.0 (36.2 to 76.4)	3.14 (2.09 to 4.79)	6.44 (3.97 to 9.9)	104.7 (77.9 to 132.8)
		Pseudomonas aeruginosa	4160 (3680 to 4680)	9610 (8700 to 10700)	131.3 (111.0 to 152.0)	28.6 (26.5 to 30.8)	86 (79.2 to 93)	201.4 (173.9 to 230.7)
		Respiratory syncytial virus	2820 (2480 to 3180)	496 (221 to 927)	-82.4 (-92.2 to -66.9)	9.55 (8.77 to 10.5)	2.09 (0.92 to 4.02)	-78.1 (-90.3 to -58.3)
		Staphylococcus aureus	6840 (6100 to 7600)	16700 (15200 to 18400)	143.9 (125.3 to 161.8)	96 (90.5 to 101)	305 (283 to 328)	218.2 (191.1 to 245.6)
		Streptococcus pneumoniae	67400 (60500 to 74500)	51800 (46900 to 57000)	-23.1 (-28.8 to -17.6)	393 (374 to 412)	396 (368 to 424)	0.6 (-7.7 to 9.0)
		Other viruses	27700 (24900 to 30600)	40700 (36800 to 44800)	46.8 (36.5 to 57.5)	63.8 (60.3 to 67.4)	122 (113 to 131)	91.2 (75.4 to 106.9)
		Other bacterial pathogens	7510 (6480 to 8570)	8970 (7870 to 10200)	19.5 (8.2 to 31.1)	41.7 (37.8 to 45.9)	65.3 (58.1 to 73.5)	56.7 (42.2 to 72.7)
	50-69 years	*Acinetobacter baumannii*	5310 (4570 to 6170)	5200 (4510 to 5920)	-1.9 (-11.8 to 7.5)	92.8 (81.1 to 105)	141 (124 to 164)	52.2 (35.3 to 70.3)
		Chlamydia spp.	12900 (11400 to 14500)	13300 (11900 to 14700)	3.3 (-6.4 to 13.6)	56.1 (50.5 to 62)	88.2 (78.9 to 98.3)	57.5 (41.0 to 77.0)
		Enterobacter spp.	1720 (1470 to 2020)	4240 (3740 to 4780)	147.8 (121.8 to 173.8)	28.7 (24.6 to 33.3)	106 (93.2 to 119)	270.0 (229.7 to 315.4)
		Escherichia coli	5190 (4560 to 5840)	10400 (9330 to 11400)	100.1 (80.7 to 119.5)	89.6 (80.5 to 98.9)	269 (244 to 295)	200.9 (168.5 to 237.4)
		Fungus	4990 (4330 to 5710)	9210 (8180 to 10300)	85.1 (67.0 to 105.1)	84.8 (74.4 to 96)	236 (210 to 267)	179.1 (146.8 to 212.9)
		Group B Streptococcus	4060 (3530 to 4660)	8470 (7580 to 9420)	109.0 (86.6 to 129.0)	49.8 (43.8 to 56.7)	156 (140 to 174)	213.7 (176.9 to 252.3)
		Haemophilus influenzae	13600 (12300 to 15000)	20300 (18400 to 22200)	49.8 (37.8 to 61.0)	89.5 (82.8 to 96.7)	139 (127 to 152)	56.0 (41.5 to 71.7)
		Influenza	16800 (15300 to 18500)	3160 (1410 to 6000)	-81.2 (-91.6 to -64.3)	217 (203 to 233)	60 (25.8 to 118)	-72.3 (-88.0 to -45.9)
		Klebsiella pneumoniae	10700 (9800 to 11700)	17200 (15600 to 18700)	60.4 (47.9 to 72.9)	184 (170 to 196)	445 (409 to 482)	142.5 (120.9 to 166.3)
		Legionella spp.	8640 (7570 to 9790)	17700 (16100 to 19400)	104.9 (84.2 to 126.6)	103 (91.7 to 114)	310 (283 to 337)	202.7 (170.6 to 242.1)
		Mycoplasma spp.	14000 (12500 to 15400)	18300 (16600 to 20200)	31.5 (19.9 to 42.8)	63.7 (58.4 to 69.7)	126 (115 to 137)	97.4 (77.4 to 120.3)
		Polymicrobial	739 (497 to 1110)	1260 (804 to 1980)	69.9 (49.9 to 90.2)	12.9 (8.58 to 19.4)	33.4 (21.1 to 51.4)	158.6 (125.4 to 195.3)
		Pseudomonas aeruginosa	9810 (8830 to 10800)	23400 (21300 to 25600)	138.7 (118.8 to 158.7)	117 (108 to 127)	415 (383 to 451)	254.3 (218.3 to 293.7)

		Respiratory syncytial virus	2990 (2630 to 3360)	543 (242 to 1030)	-81.8 (-91.8 to -65.6)	17.7 (15.9 to 19.6)	4.69 (2 to 9.1)	-73.4 (-88.4 to -48.4)
		Staphylococcus aureus	14200 (13000 to 15500)	37800 (34500 to 41100)	166.1 (147.4 to 185.1)	347 (325 to 369)	1370 (1270 to 1490)	296.7 (261.2 to 335.4)
		Streptococcus pneumoniae	69100 (63400 to 74700)	62200 (57100 to 67700)	-9.9 (-16.2 to -3.6)	702 (662 to 740)	955 (883 to 1040)	36.2 (24.3 to 49.5)
		Other viruses	24900 (22700 to 27000)	44700 (40900 to 48600)	80.0 (67.0 to 92.8)	99.7 (93.6 to 106)	266 (245 to 288)	166.8 (142.5 to 192.4)
		Other bacterial pathogens	18900 (16700 to 21100)	24100 (20800 to 27300)	27.7 (16.8 to 39.2)	182 (165 to 201)	352 (311 to 397)	93.3 (74.6 to 114.8)
	70+ years	*Acinetobacter baumannii*	6120 (5270 to 7070)	8320 (7150 to 9610)	36.1 (21.7 to 50.1)	316 (276 to 366)	661 (558 to 757)	108.9 (88.7 to 131.1)
		Chlamydia spp.	17700 (15600 to 19900)	25400 (22600 to 28700)	43.7 (29.5 to 57.7)	228 (202 to 253)	496 (423 to 550)	117.2 (97.7 to 139.3)
		Enterobacter spp.	1940 (1630 to 2290)	6670 (5720 to 7660)	245.4 (209.4 to 280.7)	95.7 (81.2 to 112)	489 (411 to 557)	412.5 (357.1 to 468.2)
		Escherichia coli	7370 (6470 to 8320)	20500 (18100 to 23000)	179.0 (152.0 to 206.9)	376 (333 to 421)	1570 (1360 to 1740)	316.7 (276.5 to 357.4)
		Fungus	3210 (2730 to 3730)	8270 (7160 to 9420)	157.9 (131.7 to 184.9)	162 (140 to 185)	623 (529 to 710)	285.9 (245.0 to 326.7)
		Group B Streptococcus	4310 (3800 to 4930)	12500 (11000 to 13900)	191.1 (162.8 to 220.7)	156 (136 to 176)	678 (582 to 750)	333.9 (290.0 to 380.7)
		Haemophilus influenzae	14200 (12800 to 15700)	29500 (26300 to 32600)	108.8 (91.7 to 125.1)	276 (249 to 300)	596 (519 to 653)	115.9 (98.4 to 132.4)
		Influenza	29600 (27000 to 32400)	7680 (3460 to 14300)	-74.0 (-88.4 to -51.2)	1130 (1020 to 1210)	427 (191 to 825)	-62.2 (-83.1 to -26.9)
		Klebsiella pneumoniae	13600 (12500 to 15000)	30400 (27200 to 33700)	123.2 (105.7 to 140.5)	692 (630 to 741)	2320 (2010 to 2530)	234.7 (207.0 to 259.7)
		Legionella spp.	7210 (6340 to 8210)	20500 (18300 to 22800)	185.5 (156.6 to 215.8)	253 (222 to 285)	1060 (928 to 1180)	319.7 (275.5 to 362.1)
		Mycoplasma spp.	13700 (12300 to 15200)	25000 (22400 to 27800)	83.0 (67.0 to 98.4)	185 (166 to 201)	503 (434 to 554)	172.8 (148.7 to 196.5)
		Polymicrobial	979 (637 to 1500)	2330 (1430 to 3730)	136.7 (108.6 to 167.0)	50.5 (33.1 to 77.7)	181 (111 to 288)	257.3 (214.3 to 305.2)
		Pseudomonas aeruginosa	11800 (10700 to 13200)	39300 (35200 to 43600)	232.3 (204.4 to 260.4)	419 (380 to 452)	2050 (1780 to 2240)	390.4 (347.4 to 432.5)
		Respiratory syncytial virus	3250 (2890 to 3670)	813 (366 to 1500)	-75.0 (-88.6 to -52.7)	56.9 (50.7 to 62.7)	20.6 (9.11 to 39.2)	-63.8 (-84.1 to -30.2)
		Staphylococcus aureus	21300 (19500 to 23400)	78900 (70700 to 86800)	270.9 (243.4 to 297.6)	1540 (1400 to 1640)	8460 (7360 to 9190)	449.6 (409.5 to 487.7)
		Streptococcus pneumoniae	82200 (75500 to 89300)	103000 (92300 to 113000)	25.2 (16.3 to 34.0)	2470 (2260 to 2610)	4640 (4030 to 5040)	87.6 (74.7 to 100.0)
		Other viruses	29300 (26900 to 32000)	73400 (65900 to 80500)	150.5 (132.2 to 167.8)	348 (317 to 371)	1280 (1130 to 1390)	269.1 (243.4 to 294.4)
		Other bacterial pathogens	29200 (25800 to 32900)	51900 (45100 to 59000)	77.8 (62.6 to 93.8)	844 (747 to 932)	2250 (1890 to 2560)	167.0 (142.5 to 191.6)
Argentina	All Ages	*Acinetobacter baumannii*	9660 (8410 to 11000)	13500 (11900 to 15300)	40.3 (24.5 to 54.5)	245 (216 to 279)	741 (638 to 847)	202.2 (171.4 to 234.2)

		Chlamydia spp.	31600 (28600 to 34700)	41900 (38400 to 45500)	33.1 (20.3 to 46.2)	184 (168 to 200)	535 (469 to 587)	190.6 (164.2 to 219.5)
		Enterobacter spp.	3270 (2800 to 3810)	10400 (9220 to 11700)	220.2 (187.0 to 254.1)	75.3 (64.8 to 87.3)	520 (447 to 587)	591.9 (516.0 to 664.3)
		Escherichia coli	11000 (9840 to 12300)	29400 (26500 to 32200)	167.3 (139.9 to 193.1)	280 (251 to 309)	1610 (1420 to 1770)	473.2 (415.2 to 529.1)
		Fungus	7470 (6510 to 8540)	18000 (16100 to 20000)	141.5 (116.7 to 166.7)	146 (128 to 167)	778 (677 to 877)	433.6 (376.9 to 490.0)
		Group B Streptococcus	8480 (7560 to 9570)	22100 (20000 to 24100)	160.5 (133.3 to 188.4)	135 (122 to 151)	749 (662 to 822)	454.5 (398.0 to 513.3)
		Haemophilus influenzae	36000 (32600 to 39600)	55800 (51400 to 60400)	55.3 (40.5 to 69.2)	275 (256 to 294)	662 (593 to 719)	141.2 (120.1 to 160.5)
		Influenza	49400 (45700 to 53500)	12200 (4790 to 23700)	-75.3 (-90.2 to -51.6)	854 (793 to 907)	458 (175 to 916)	-46.3 (-78.5 to 7.1)
		Klebsiella pneumoniae	25800 (23800 to 28100)	48100 (44300 to 52000)	86.3 (69.6 to 101.6)	577 (538 to 615)	2470 (2200 to 2670)	327.1 (289.2 to 361.4)
		Legionella spp.	13600 (12100 to 15200)	36900 (33800 to 40100)	172.1 (143.5 to 201.2)	202 (181 to 225)	1200 (1070 to 1310)	493.5 (429.4 to 554.4)
		Mycoplasma spp.	41400 (37600 to 45300)	66800 (62300 to 71500)	61.7 (48.4 to 75.4)	185 (171 to 199)	621 (563 to 673)	235.6 (206.9 to 264.7)
		Polymicrobial	1590 (1090 to 2320)	3550 (2270 to 5500)	121.8 (91.5 to 151.6)	43.7 (31.1 to 63.1)	195 (123 to 306)	343.7 (270.5 to 415.3)
		Pseudomonas aeruginosa	20000 (18200 to 21800)	62100 (57200 to 67000)	211.3 (182.9 to 238.6)	323 (297 to 346)	2160 (1930 to 2330)	568.8 (507.0 to 625.3)
		Respiratory syncytial virus	25900 (22400 to 29700)	3600 (1390 to 7120)	-86.1 (-94.4 to -72.8)	155 (146 to 165)	28.7 (11.2 to 57)	-81.5 (-92.8 to -62.6)
		Staphylococcus aureus	32200 (29900 to 34700)	112000 (103000 to 121000)	248.6 (219.5 to 273.6)	1110 (1030 to 1170)	8540 (7580 to 9230)	672.6 (611.5 to 729.6)
		Streptococcus pneumoniae	222000 (205000 to 242000)	204000 (191000 to 218000)	-8.1 (-15.7 to -1.0)	2430 (2300 to 2540)	5230 (4710 to 5630)	115.2 (97.8 to 130.6)
		Other viruses	101000 (92200 to 110000)	155000 (144000 to 166000)	53.8 (40.3 to 67.0)	410 (389 to 430)	1430 (1290 to 1540)	249.3 (219.3 to 273.9)
		Other bacterial pathogens	39200 (35100 to 43500)	71700 (63300 to 80300)	83.2 (65.6 to 100.3)	578 (520 to 634)	2300 (1960 to 2600)	297.9 (262.0 to 336.0)
	Under 5	*Acinetobacter baumannii*	342 (242 to 465)	122 (87.9 to 168)	-64.2 (-69.5 to -57.9)	10.6 (7.03 to 15.5)	3.05 (1.74 to 5.16)	-71.4 (-79.2 to -62.4)
		Chlamydia spp.	4520 (3690 to 5440)	1950 (1600 to 2390)	-56.7 (-63.9 to -48.7)	21.4 (19.2 to 23.8)	4.59 (3.58 to 5.87)	-78.5 (-83.3 to -72.7)
		Enterobacter spp.	406 (300 to 534)	391 (291 to 515)	-3.5 (-20.5 to 15.9)	7.8 (6.05 to 10.2)	3.25 (2.35 to 4.53)	-58.2 (-67.7 to -46.8)
		Escherichia coli	1490 (1190 to 1820)	1390 (1130 to 1720)	-6.1 (-21.7 to 10.2)	27.9 (24.3 to 31.6)	10.7 (8.48 to 13.4)	-61.5 (-70.3 to -51.1)
		Fungus	300 (204 to 419)	228 (160 to 317)	-23.6 (-37.5 to -7.1)	4.33 (3.05 to 5.81)	1.22 (0.82 to 1.7)	-71.7 (-78.8 to -63.6)
		Group B Streptococcus	1240 (998 to 1510)	1040 (826 to 1290)	-16.0 (-29.5 to -0.3)	19.4 (17.1 to 22)	7.34 (5.83 to 9.3)	-62.1 (-70.9 to -51.1)
		Haemophilus influenzae	10900 (8900 to 13200)	5800 (4740 to 7130)	-46.8 (-54.7 to -38.1)	67.9 (61.3 to 75.9)	8.76 (6.92 to 10.9)	-87.0 (-89.9 to -83.4)

		Influenza	9300 (7720 to 10900)	602 (226 to 1200)	-93.5 (-97.5 to -87.3)	110 (100 to 120)	2.62 (0.98 to 5.31)	-97.6 (-99.1 to -95.1)
		Klebsiella pneumoniae	5660 (4690 to 6740)	2670 (2180 to 3270)	-52.8 (-59.3 to -45.1)	90.9 (82.8 to 100)	18.4 (14.6 to 23)	-79.7 (-84.0 to -74.4)
		Legionella spp.	435 (300 to 626)	343 (231 to 502)	-21.0 (-33.5 to -6.6)	7.37 (5.52 to 9.49)	3.1 (2.2 to 4.32)	-57.7 (-67.4 to -45.0)
		Mycoplasma spp.	6340 (5270 to 7530)	5750 (4710 to 7030)	-9.0 (-23.1 to 5.8)	27.2 (24.3 to 30.4)	9.37 (7.48 to 11.7)	-65.4 (-73.0 to -56.1)
		Polymicrobial	191 (120 to 286)	101 (62.8 to 155)	-46.8 (-56.0 to -36.8)	8.11 (4.76 to 12.8)	3.75 (1.93 to 6.5)	-53.9 (-66.5 to -38.6)
		Pseudomonas aeruginosa	2790 (2300 to 3320)	3480 (2850 to 4270)	25.3 (5.9 to 45.9)	34.5 (31.1 to 38.5)	16.8 (13.3 to 21)	-51.3 (-62.0 to -38.7)
		Respiratory syncytial virus	19400 (16200 to 22900)	1880 (706 to 3750)	-90.3 (-96.2 to -81.1)	112 (104 to 121)	4.05 (1.52 to 8.28)	-96.4 (-98.6 to -92.6)
		Staphylococcus aureus	3730 (3120 to 4390)	3860 (3150 to 4690)	3.4 (-9.9 to 18.6)	83.9 (77.4 to 91.5)	33.5 (26.7 to 41.4)	-60.0 (-68.3 to -49.9)
		Streptococcus pneumoniae	62400 (52100 to 73200)	14200 (11700 to 17500)	-77.1 (-80.1 to -73.9)	546 (504 to 595)	46.7 (37.1 to 57.8)	-91.4 (-93.2 to -89.2)
		Other viruses	41400 (34700 to 48500)	21100 (17400 to 25600)	-49.1 (-55.8 to -42.0)	146 (135 to 159)	29.9 (23.8 to 37)	-79.5 (-83.7 to -74.2)
		Other bacterial pathogens	1730 (1340 to 2190)	975 (752 to 1250)	-43.4 (-52.7 to -33.1)	17.4 (14.2 to 21.1)	4.27 (3.2 to 5.79)	-75.3 (-80.9 to -68.1)
	5-14 years	*Acinetobacter baumannii*	711 (525 to 937)	427 (328 to 553)	-39.7 (-47.2 to -31.8)	1.73 (1.46 to 2.02)	1.17 (0.98 to 1.37)	-31.9 (-43.0 to -19.9)
		Chlamydia spp.	2860 (2140 to 3770)	1890 (1430 to 2420)	-33.6 (-41.3 to -25.0)	1.72 (1.52 to 1.94)	1.29 (1.11 to 1.48)	-25.0 (-36.7 to -12.2)
		Enterobacter spp.	226 (165 to 302)	341 (258 to 448)	51.6 (34.1 to 71.2)	0.52 (0.44 to 0.61)	0.88 (0.76 to 1.04)	71.2 (43.0 to 100.6)
		Escherichia coli	626 (470 to 822)	791 (604 to 1010)	26.7 (12.0 to 42.0)	1.49 (1.3 to 1.68)	2.12 (1.85 to 2.42)	43.1 (22.2 to 67.0)
		Fungus	756 (557 to 1000)	855 (648 to 1110)	13.4 (-0.7 to 28.1)	1.77 (1.52 to 2.04)	2.26 (1.93 to 2.63)	28.1 (8.1 to 50.2)
		Group B Streptococcus	761 (562 to 989)	995 (766 to 1280)	31.3 (15.4 to 47.7)	1.28 (1.11 to 1.48)	1.9 (1.65 to 2.18)	48.3 (24.1 to 75.8)
		Haemophilus influenzae	2960 (2230 to 3850)	2500 (1910 to 3180)	-15.3 (-24.2 to -6.4)	2.68 (2.42 to 2.98)	1.79 (1.58 to 2.03)	-33.1 (-42.4 to -22.3)
		Influenza	4270 (3270 to 5510)	517 (203 to 1060)	-87.9 (-95.3 to -75.8)	7.58 (6.91 to 8.29)	0.99 (0.38 to 2)	-86.9 (-95.0 to -73.5)
		Klebsiella pneumoniae	1840 (1410 to 2400)	1610 (1240 to 2080)	-12.8 (-20.8 to -4.0)	4.36 (3.94 to 4.78)	4.29 (3.78 to 4.82)	-1.5 (-15.2 to 13.6)
		Legionella spp.	1320 (989 to 1750)	1640 (1270 to 2130)	25.2 (10.5 to 40.8)	2.15 (1.88 to 2.43)	3.02 (2.65 to 3.42)	41.4 (20.0 to 65.9)
		Mycoplasma spp.	6660 (5040 to 8570)	6040 (4640 to 7760)	-9.1 (-18.6 to 1.5)	4.18 (3.77 to 4.61)	4.29 (3.78 to 4.85)	2.7 (-11.9 to 19.6)
		Polymicrobial	116 (73.1 to 175)	121 (73.7 to 183)	3.5 (-9.1 to 18.3)	0.28 (0.19 to 0.42)	0.33 (0.21 to 0.5)	16.9 (-2.6 to 38.4)
		Pseudomonas aeruginosa	1490 (1130 to 1920)	2270 (1760 to 2910)	52.8 (36.6 to 70.0)	2.45 (2.2 to 2.69)	4.21 (3.7 to 4.74)	72.5 (47.8 to 100.2)
		Respiratory syncytial virus	1000 (764 to 1300)	128 (49.5 to 263)	-87.2 (-94.9 to -74.3)	0.81 (0.72 to 0.9)	0.11 (0.04 to 0.22)	-86.2 (-94.7 to -72.0)

		Staphylococcus aureus	2480 (1890 to 3200)	3970 (3060 to 5020)	60.4 (45.7 to 76.5)	8.32 (7.58 to 9.09)	15.1 (13.3 to 16.9)	81.2 (57.0 to 108.5)
		Streptococcus pneumoniae	24600 (18800 to 31600)	12800 (9860 to 16400)	-48.0 (-52.7 to -42.8)	34.4 (31.6 to 37.2)	20.2 (18 to 22.6)	-41.3 (-49.1 to -32.7)
		Other viruses	9900 (7640 to 12600)	9630 (7440 to 12400)	-2.5 (-11.2 to 7.0)	5.44 (4.97 to 5.91)	5.98 (5.33 to 6.69)	10.1 (-5.2 to 26.5)
		Other bacterial pathogens	2760 (2100 to 3620)	2190 (1680 to 2840)	-20.5 (-29.1 to -11.4)	3.68 (3.26 to 4.13)	3.3 (2.81 to 3.83)	-10.0 (-23.0 to 3.9)
	15-49 years	*Acinetobacter baumannii*	1200 (996 to 1430)	1450 (1240 to 1690)	21.1 (5.9 to 38.1)	10.7 (9.25 to 12.4)	19.4 (16.7 to 22.4)	82.1 (59.0 to 105.5)
		Chlamydia spp.	4820 (4170 to 5550)	6400 (5640 to 7260)	33.3 (17.8 to 49.5)	10.7 (9.58 to 11.9)	21.3 (19.1 to 23.9)	100.5 (77.7 to 125.8)
		Enterobacter spp.	381 (312 to 457)	1160 (989 to 1330)	204.4 (168.9 to 246.6)	3.22 (2.76 to 3.76)	14.7 (12.8 to 16.8)	358.1 (301.2 to 423.0)
		Escherichia coli	1060 (904 to 1230)	2680 (2390 to 3010)	154.4 (125.2 to 188.0)	9.23 (8.22 to 10.3)	35.3 (32 to 38.9)	282.8 (236.9 to 330.1)
		Fungus	1280 (1060 to 1500)	2900 (2510 to 3330)	127.7 (99.6 to 156.3)	11 (9.47 to 12.6)	37.6 (32.8 to 42.8)	242.6 (199.5 to 286.1)
		Group B Streptococcus	1280 (1100 to 1490)	3370 (2960 to 3860)	163.8 (129.8 to 198.8)	7.96 (6.99 to 9.04)	31.5 (28.2 to 35.2)	296.8 (246.3 to 347.0)
		Haemophilus influenzae	4990 (4350 to 5670)	8460 (7590 to 9450)	70.0 (52.3 to 88.9)	16.6 (15.2 to 18.2)	29.7 (27 to 32.6)	79.0 (60.2 to 97.2)
		Influenza	7200 (6340 to 8140)	1750 (670 to 3440)	-75.6 (-90.7 to -52.3)	47 (43.5 to 50.7)	16.4 (6.47 to 33.3)	-65.0 (-86.0 to -28.8)
		Klebsiella pneumoniae	3110 (2740 to 3500)	5440 (4910 to 6080)	75.2 (58.6 to 93.9)	27 (25.1 to 29.3)	71.1 (65.3 to 77.7)	163.5 (136.4 to 190.3)
		Legionella spp.	2220 (1910 to 2570)	5570 (4980 to 6220)	151.5 (121.8 to 185.0)	13.3 (11.7 to 15.1)	50.2 (45.3 to 55)	278.4 (231.8 to 327.8)
		Mycoplasma spp.	11200 (9860 to 12700)	20500 (18400 to 22700)	82.6 (63.0 to 102.6)	25.9 (23.8 to 28.1)	71.1 (65 to 77.7)	174.7 (144.7 to 205.6)
		Polymicrobial	197 (126 to 291)	410 (254 to 634)	107.8 (79.2 to 137.6)	1.74 (1.17 to 2.61)	5.46 (3.38 to 8.39)	212.7 (169.2 to 257.8)
		Pseudomonas aeruginosa	2510 (2190 to 2840)	7700 (6900 to 8550)	206.8 (174.1 to 240.1)	15.2 (13.9 to 16.5)	69.9 (63.8 to 76)	361.6 (313.4 to 411.4)
		Respiratory syncytial virus	1690 (1480 to 1920)	434 (169 to 841)	-74.3 (-90.0 to -49.7)	5.03 (4.56 to 5.5)	1.85 (0.73 to 3.73)	-63.2 (-85.5 to -25.8)
		Staphylococcus aureus	4180 (3670 to 4710)	13400 (12100 to 14900)	222.2 (190.1 to 254.4)	51.6 (48.4 to 55.2)	250 (231 to 271)	384.6 (335.3 to 430.2)
		Streptococcus pneumoniae	41500 (36700 to 46300)	43200 (38900 to 47700)	4.4 (-5.4 to 14.0)	213 (200 to 226)	334 (308 to 361)	57.0 (41.6 to 71.4)
		Other viruses	16700 (14800 to 18700)	32600 (29500 to 36100)	95.7 (77.4 to 114.1)	33.7 (31.5 to 36)	99.2 (91.2 to 107)	194.5 (166.1 to 222.8)
		Other bacterial pathogens	4660 (3980 to 5380)	7430 (6510 to 8500)	59.7 (42.4 to 77.9)	22.8 (20.5 to 25.3)	54.8 (48.6 to 62.2)	140.7 (113.9 to 169.1)
	50-69 years	*Acinetobacter baumannii*	3490 (2970 to 4090)	4480 (3870 to 5130)	28.8 (14.3 to 42.5)	52.3 (45.4 to 60)	129 (113 to 150)	147.0 (115.3 to 179.5)
		Chlamydia spp.	8270 (7220 to 9440)	11000 (9810 to 12300)	33.5 (19.6 to 47.5)	30.8 (27.5 to 34.5)	78.8 (70.3 to 88.2)	156.1 (126.1 to 191.1)

		Enterobacter spp.	1070 (904 to 1270)	3370 (2940 to 3830)	214.7 (178.6 to 250.4)	15.3 (12.9 to 18)	92 (80.6 to 104)	503.9 (428.7 to 584.1)
		Escherichia coli	3290 (2850 to 3760)	8350 (7450 to 9280)	154.5 (126.6 to 182.2)	48.4 (42.7 to 54.3)	236 (212 to 259)	388.3 (331.0 to 452.3)
		Fungus	3160 (2720 to 3670)	7480 (6590 to 8430)	137.1 (111.0 to 165.7)	45.9 (39.8 to 52.8)	208 (184 to 237)	354.9 (295.8 to 414.6)
		Group B Streptococcus	2560 (2210 to 2970)	6810 (6040 to 7630)	166.8 (136.7 to 197.3)	26.7 (23.5 to 30.4)	137 (122 to 153)	412.0 (346.1 to 486.0)
		Haemophilus influenzae	8500 (7560 to 9480)	16100 (14500 to 17800)	90.4 (71.7 to 107.4)	47.7 (43.2 to 52.4)	122 (111 to 133)	155.5 (126.9 to 186.0)
		Influenza	10600 (9470 to 11700)	2760 (1080 to 5470)	-73.9 (-89.5 to -47.7)	116 (106 to 126)	55.3 (22 to 112)	-52.2 (-81.1 to -4.1)
		Klebsiella pneumoniae	6790 (6100 to 7510)	14000 (12600 to 15400)	106.9 (87.2 to 124.9)	99.3 (91 to 109)	393 (360 to 428)	296.9 (253.7 to 343.2)
		Legionella spp.	5320 (4580 to 6070)	13800 (12400 to 15200)	159.5 (130.6 to 191.1)	53.6 (47.3 to 60.4)	266 (242 to 291)	397.9 (336.9 to 468.4)
		Mycoplasma spp.	8770 (7790 to 9810)	14800 (13300 to 16300)	69.0 (51.3 to 85.5)	34.1 (30.9 to 37.8)	110 (100 to 121)	224.3 (186.7 to 265.6)
		Polymicrobial	476 (318 to 704)	1040 (669 to 1630)	118.1 (90.7 to 146.3)	7.09 (4.76 to 10.6)	29.7 (18.9 to 45.6)	318.5 (262.2 to 381.4)
		Pseudomonas aeruginosa	6070 (5390 to 6770)	18400 (16600 to 20300)	204.2 (174.1 to 233.0)	61.7 (56 to 67.6)	359 (331 to 392)	483.6 (417.0 to 556.2)
		Respiratory syncytial virus	1840 (1600 to 2080)	471 (184 to 933)	-74.4 (-89.8 to -48.3)	9.21 (8.21 to 10.3)	4.31 (1.72 to 8.66)	-53.1 (-81.5 to -6.5)
		Staphylococcus aureus	8880 (8010 to 9780)	29900 (27100 to 32700)	237.4 (205.7 to 264.7)	185 (171 to 200)	1190 (1100 to 1290)	547.4 (479.8 to 617.0)
		Streptococcus pneumoniae	43500 (39400 to 47600)	51100 (46500 to 55900)	17.5 (6.7 to 27.3)	376 (349 to 405)	847 (780 to 920)	125.4 (101.6 to 150.5)
		Other viruses	15300 (13800 to 16900)	35200 (31900 to 38500)	130.0 (107.4 to 149.1)	52.1 (48.2 to 56.4)	230 (210 to 250)	341.3 (295.2 to 393.0)
		Other bacterial pathogens	12000 (10500 to 13600)	19700 (17000 to 22400)	64.3 (48.0 to 80.2)	98.9 (89 to 110)	312 (275 to 352)	215.7 (180.3 to 254.8)
	70+ years	*Acinetobacter baumannii*	3920 (3340 to 4550)	7040 (6010 to 8160)	80.2 (57.1 to 101.5)	170 (147 to 196)	588 (496 to 674)	246.2 (209.4 to 284.6)
		Chlamydia spp.	11100 (9760 to 12500)	20700 (18100 to 23400)	86.8 (65.1 to 108.4)	120 (105 to 133)	429 (368 to 476)	258.9 (223.9 to 298.6)
		Enterobacter spp.	1180 (985 to 1410)	5170 (4360 to 5970)	340.4 (285.5 to 393.8)	48.4 (41 to 56.9)	409 (343 to 468)	746.3 (651.2 to 844.2)
		Escherichia coli	4540 (3950 to 5180)	16100 (14000 to 18100)	256.1 (211.7 to 298.2)	193 (170 to 217)	1320 (1150 to 1470)	584.4 (512.6 to 656.5)
		Fungus	1980 (1670 to 2320)	6560 (5610 to 7510)	231.8 (189.9 to 272.8)	83.1 (71.5 to 96)	529 (448 to 604)	537.6 (465.7 to 609.1)
		Group B Streptococcus	2640 (2320 to 3020)	9840 (8570 to 11000)	273.3 (227.3 to 316.0)	79.8 (69.1 to 90.6)	572 (493 to 635)	617.5 (539.7 to 698.9)
		Haemophilus influenzae	8610 (7720 to 9560)	22900 (20000 to 25500)	166.4 (136.4 to 192.6)	140 (126 to 154)	500 (435 to 548)	258.1 (227.0 to 290.0)
		Influenza	18100 (16400 to 19800)	6580 (2510 to 12900)	-63.5 (-85.5 to -27.9)	574 (517 to 618)	383 (146 to 764)	-33.1 (-73.3 to 34.0)

		Klebsiella pneumoniae	8410 (7640 to 9300)	24300 (21400 to 27100)	189.4 (158.4 to 217.4)	356 (322 to 384)	1980 (1730 to 2160)	456.2 (406.5 to 502.9)
		Legionella spp.	4310 (3760 to 4890)	15600 (13600 to 17400)	263.1 (218.0 to 307.6)	125 (109 to 141)	873 (764 to 970)	597.8 (518.5 to 675.4)
		Mycoplasma spp.	8340 (7490 to 9310)	19700 (17400 to 22000)	136.5 (108.4 to 161.5)	93.8 (83.5 to 103)	426 (369 to 470)	354.5 (310.6 to 398.1)
		Polymicrobial	613 (403 to 934)	1880 (1140 to 2990)	205.2 (164.0 to 248.2)	26.4 (17.4 to 40.9)	156 (95.7 to 247)	486.6 (415.1 to 570.0)
		Pseudomonas aeruginosa	7110 (6370 to 7940)	30200 (26600 to 33600)	325.6 (278.1 to 369.9)	209 (188 to 227)	1710 (1490 to 1860)	717.9 (639.7 to 795.0)
		Respiratory syncytial virus	1940 (1710 to 2190)	692 (264 to 1350)	-64.2 (-85.9 to -28.5)	28.1 (25 to 31.2)	18.4 (7.01 to 36.6)	-34.3 (-74.0 to 32.0)
		Staphylococcus aureus	12900 (11700 to 14200)	61000 (53700 to 67300)	372.1 (322.5 to 415.2)	777 (701 to 832)	7050 (6150 to 7680)	807.2 (728.3 to 878.7)
		Streptococcus pneumoniae	50300 (45800 to 54800)	82700 (73000 to 91400)	64.4 (47.0 to 79.0)	1260 (1140 to 1340)	3980 (3470 to 4330)	215.9 (189.5 to 240.8)
		Other viruses	17500 (16000 to 19100)	56400 (49700 to 62400)	221.8 (187.2 to 250.6)	172 (157 to 185)	1070 (934 to 1160)	518.4 (467.5 to 567.0)
		Other bacterial pathogens	18000 (16000 to 20400)	41400 (35500 to 47200)	129.9 (103.0 to 154.6)	435 (384 to 483)	1920 (1610 to 2190)	342.4 (299.4 to 388.4)
Chile	All Ages	*Acinetobacter baumannii*	4250 (3730 to 4840)	1790 (1580 to 2010)	-58.0 (-62.0 to -53.1)	181 (160 to 205)	63.4 (52.3 to 72.5)	-65.0 (-68.7 to -61.0)
		Chlamydia spp.	14700 (13400 to 16100)	7010 (6440 to 7610)	-52.2 (-56.6 to -47.4)	139 (126 to 152)	58.1 (49.5 to 64.4)	-58.3 (-63.1 to -53.7)
		Enterobacter spp.	1610 (1400 to 1850)	2240 (2020 to 2470)	39.3 (24.3 to 55.4)	60.9 (52.4 to 69.9)	71.9 (60.7 to 80.8)	18.4 (4.3 to 33.2)
		Escherichia coli	5290 (4770 to 5840)	5820 (5390 to 6360)	10.2 (-0.5 to 22.1)	221 (198 to 243)	205 (176 to 226)	-7.4 (-18.7 to 3.1)
		Fungus	3560 (3100 to 4030)	3410 (3090 to 3780)	-4.1 (-13.9 to 6.4)	115 (100 to 130)	94.9 (81.6 to 106)	-17.1 (-26.7 to -6.9)
		Group B Streptococcus	4140 (3720 to 4610)	4450 (4110 to 4840)	7.7 (-4.0 to 18.6)	107 (96 to 119)	98 (84.1 to 108)	-8.6 (-18.9 to 2.6)
		Haemophilus influenzae	17800 (16400 to 19300)	12000 (11200 to 12900)	-32.8 (-37.9 to -27.1)	210 (194 to 226)	88.7 (77.2 to 96.7)	-57.7 (-61.9 to -53.7)
		Influenza	24300 (22700 to 26000)	1340 (366 to 2990)	-94.5 (-98.5 to -87.8)	676 (625 to 720)	32.3 (8.96 to 71.5)	-95.2 (-98.7 to -89.4)
		Klebsiella pneumoniae	12400 (11600 to 13300)	9000 (8430 to 9740)	-27.4 (-32.6 to -21.8)	443 (409 to 471)	298 (258 to 327)	-32.7 (-39.4 to -26.6)
		Legionella spp.	6930 (6220 to 7720)	8800 (8210 to 9530)	27.3 (14.6 to 41.8)	171 (153 to 190)	185 (161 to 203)	8.1 (-4.6 to 20.4)
		Mycoplasma spp.	20300 (18700 to 22000)	13400 (12500 to 14500)	-33.7 (-38.7 to -28.6)	143 (132 to 155)	82.5 (72.8 to 89.8)	-42.5 (-48.3 to -37.2)
		Polymicrobial	752 (521 to 1110)	614 (376 to 979)	-19.0 (-31.4 to -5.6)	34.6 (24.6 to 49.5)	22.2 (13.8 to 35.5)	-36.2 (-48.9 to -23.4)
		Pseudomonas aeruginosa	10100 (9360 to 11000)	14300 (13400 to 15500)	41.7 (30.3 to 53.7)	267 (245 to 287)	321 (278 to 351)	20.2 (6.7 to 32.1)
		Respiratory syncytial virus	13600 (12100 to 15500)	470 (132 to 1040)	-96.5 (-99.0 to -92.3)	108 (102 to 114)	2.3 (0.65 to 5.02)	-97.9 (-99.4 to -95.3)
		Staphylococcus aureus	15900 (14900 to 17100)	24600 (23000 to 26500)	54.3 (43.9 to 65.5)	898 (826 to 952)	1210 (1040 to 1320)	34.8 (22.2 to 46.7)

		Streptococcus pneumoniae	109000 (102000 to 116000)	37900 (35600 to 40700)	-65.1 (-67.4 to -62.8)	1820 (1710 to 1910)	636 (554 to 692)	-65.1 (-68.3 to -62.3)
		Other viruses	52000 (48200 to 56300)	36200 (33900 to 39100)	-30.3 (-35.3 to -25.3)	309 (291 to 325)	218 (191 to 236)	-29.6 (-36.6 to -23.7)
		Other bacterial pathogens	18600 (16700 to 20700)	13300 (11800 to 15000)	-28.6 (-34.7 to -21.8)	460 (413 to 507)	276 (229 to 317)	-40.0 (-46.7 to -33.6)
	Under 5	*Acinetobacter baumannii*	168 (120 to 229)	21 (14.9 to 28.8)	-87.5 (-89.4 to -85.4)	7.84 (4.89 to 12.2)	0.63 (0.33 to 1.09)	-92.1 (-94.1 to -89.6)
		Chlamydia spp.	2180 (1820 to 2600)	370 (298 to 450)	-82.9 (-86.2 to -79.2)	13.1 (11.8 to 14.3)	0.71 (0.6 to 0.83)	-94.5 (-95.5 to -93.5)
		Enterobacter spp.	209 (160 to 273)	94.8 (71.1 to 125)	-54.4 (-64.1 to -43.8)	5.24 (3.92 to 7.15)	0.61 (0.42 to 0.86)	-88.4 (-90.6 to -85.6)
		Escherichia coli	742 (615 to 896)	314 (253 to 382)	-57.6 (-66.0 to -48.0)	17.7 (15.5 to 20)	1.72 (1.47 to 2.03)	-90.2 (-91.8 to -88.4)
		Fungus	146 (101 to 204)	48.9 (34.2 to 67.2)	-66.4 (-73.4 to -57.8)	2.29 (1.61 to 3.05)	0.14 (0.1 to 0.18)	-94.1 (-95.3 to -92.7)
		Group B Streptococcus	632 (518 to 765)	238 (190 to 291)	-62.2 (-69.5 to -53.5)	13.5 (11.9 to 15.1)	1.37 (1.15 to 1.61)	-89.8 (-91.6 to -87.7)
		Haemophilus influenzae	5530 (4690 to 6600)	1370 (1120 to 1660)	-75.1 (-79.9 to -69.6)	40 (36.2 to 44.2)	1.09 (0.93 to 1.27)	-97.3 (-97.8 to -96.7)
		Influenza	4660 (3970 to 5480)	72.1 (20.4 to 164)	-98.4 (-99.6 to -96.6)	63.2 (58.1 to 68.5)	0.18 (0.05 to 0.4)	-99.7 (-99.9 to -99.4)
		Klebsiella pneumoniae	2780 (2350 to 3280)	546 (446 to 664)	-80.3 (-83.7 to -76.2)	52 (47.7 to 56.8)	2.55 (2.18 to 2.96)	-95.1 (-95.9 to -94.2)
		Legionella spp.	235 (165 to 333)	94 (63.8 to 133)	-60.0 (-67.5 to -51.4)	5.57 (4.13 to 7.23)	0.73 (0.55 to 0.94)	-86.8 (-89.4 to -83.7)
		Mycoplasma spp.	3180 (2690 to 3790)	1350 (1100 to 1640)	-57.3 (-65.3 to -47.9)	15.8 (14.2 to 17.6)	1.27 (1.08 to 1.48)	-92.0 (-93.3 to -90.5)
		Polymicrobial	106 (65.8 to 159)	24.3 (14.6 to 37.5)	-77.0 (-81.0 to -72.7)	6.97 (3.95 to 11.1)	1.02 (0.51 to 1.79)	-85.4 (-88.8 to -81.2)
		Pseudomonas aeruginosa	1460 (1220 to 1740)	918 (749 to 1120)	-36.8 (-48.0 to -22.9)	22.3 (20.2 to 24.5)	2.88 (2.49 to 3.34)	-87.0 (-89.0 to -84.7)
		Respiratory syncytial virus	10300 (8820 to 12100)	261 (73.8 to 591)	-97.4 (-99.3 to -94.3)	71 (66.1 to 75.9)	0.35 (0.1 to 0.78)	-99.5 (-99.9 to -98.9)
		Staphylococcus aureus	1890 (1620 to 2210)	942 (761 to 1140)	-50.1 (-58.6 to -40.4)	49.1 (45.5 to 52.9)	4.9 (4.22 to 5.64)	-90.0 (-91.5 to -88.3)
		Streptococcus pneumoniae	30900 (26600 to 36200)	2860 (2340 to 3480)	-90.7 (-92.3 to -88.9)	298 (277 to 319)	5 (4.25 to 5.8)	-98.3 (-98.6 to -98.0)
		Other viruses	21600 (18500 to 25300)	5340 (4350 to 6490)	-75.2 (-79.3 to -70.2)	85.2 (79.4 to 91.4)	4.53 (3.89 to 5.22)	-94.7 (-95.5 to -93.8)
		Other bacterial pathogens	847 (669 to 1060)	204 (156 to 264)	-75.8 (-80.6 to -70.2)	10.3 (8.23 to 12.8)	0.64 (0.46 to 0.88)	-93.8 (-95.1 to -92.1)
	5-14 years	*Acinetobacter baumannii*	273 (207 to 353)	70.3 (52.7 to 91)	-74.1 (-77.7 to -69.9)	0.77 (0.66 to 0.9)	0.14 (0.12 to 0.16)	-82.1 (-84.7 to -79.3)
		Chlamydia spp.	1150 (891 to 1460)	391 (295 to 502)	-65.9 (-70.4 to -61.0)	0.8 (0.71 to 0.91)	0.19 (0.17 to 0.21)	-76.4 (-79.6 to -72.8)
		Enterobacter spp.	96.7 (73 to 126)	91.1 (68 to 119)	-5.6 (-18.7 to 10.6)	0.26 (0.22 to 0.3)	0.17 (0.14 to 0.19)	-34.6 (-45.0 to -23.4)

		Escherichia coli	261 (203 to 334)	194 (147 to 247)	-25.5 (-35.7 to -13.5)	0.72 (0.64 to 0.81)	0.37 (0.33 to 0.42)	-48.4 (-55.6 to -40.3)
		Fungus	315 (241 to 401)	204 (154 to 265)	-35.1 (-44.4 to -24.9)	0.86 (0.74 to 0.98)	0.38 (0.34 to 0.44)	-55.1 (-61.6 to -47.2)
		Group B Streptococcus	321 (251 to 411)	250 (191 to 322)	-21.9 (-32.4 to -9.0)	0.63 (0.55 to 0.72)	0.34 (0.3 to 0.38)	-46.0 (-54.0 to -37.0)
		Haemophilus influenzae	1270 (1000 to 1580)	665 (502 to 849)	-47.4 (-54.1 to -40.1)	1.33 (1.2 to 1.47)	0.33 (0.29 to 0.36)	-75.2 (-78.7 to -72.1)
		Influenza	1820 (1460 to 2280)	69.9 (19.3 to 162)	-96.1 (-98.9 to -91.3)	3.74 (3.44 to 4.08)	0.09 (0.03 to 0.21)	-97.5 (-99.3 to -94.5)
		Klebsiella pneumoniae	768 (614 to 977)	374 (286 to 478)	-51.3 (-57.2 to -44.3)	2.11 (1.92 to 2.32)	0.71 (0.64 to 0.79)	-66.3 (-70.8 to -61.7)
		Legionella spp.	586 (460 to 742)	493 (377 to 631)	-15.7 (-27.3 to -2.0)	1.11 (0.97 to 1.26)	0.64 (0.58 to 0.72)	-41.6 (-50.6 to -31.4)
		Mycoplasma spp.	2840 (2240 to 3580)	1520 (1170 to 1970)	-46.3 (-52.6 to -38.3)	2.07 (1.87 to 2.28)	0.77 (0.69 to 0.85)	-62.8 (-67.8 to -57.8)
		Polymicrobial	46.9 (29.8 to 71)	25.8 (15 to 41)	-45.3 (-54.3 to -35.0)	0.13 (0.09 to 0.2)	0.05 (0.03 to 0.08)	-62.2 (-68.6 to -54.5)
		Pseudomonas aeruginosa	657 (514 to 826)	652 (498 to 837)	-0.6 (-13.3 to 14.0)	1.25 (1.13 to 1.38)	0.86 (0.77 to 0.95)	-31.2 (-40.7 to -21.2)
		Respiratory syncytial virus	449 (354 to 569)	19.5 (5.38 to 44.8)	-95.6 (-98.8 to -90.1)	0.42 (0.38 to 0.47)	0.01 (0 to 0.03)	-97.2 (-99.2 to -93.6)
		Staphylococcus aureus	1070 (851 to 1340)	1080 (825 to 1370)	1.3 (-10.1 to 15.2)	4.15 (3.84 to 4.53)	2.91 (2.61 to 3.22)	-29.9 (-38.7 to -20.6)
		Streptococcus pneumoniae	10400 (8320 to 13000)	2950 (2260 to 3760)	-71.6 (-74.8 to -67.9)	16.9 (15.6 to 18.2)	3.31 (2.98 to 3.65)	-80.3 (-82.7 to -77.9)
		Other viruses	4400 (3500 to 5460)	2800 (2140 to 3570)	-36.4 (-43.5 to -28.2)	2.81 (2.58 to 3.05)	1.23 (1.11 to 1.36)	-56.0 (-61.6 to -50.3)
		Other bacterial pathogens	1150 (896 to 1460)	506 (381 to 656)	-55.8 (-61.2 to -49.6)	1.77 (1.57 to 1.99)	0.54 (0.46 to 0.62)	-69.3 (-73.4 to -64.8)
	15-49 years	*Acinetobacter baumannii*	575 (482 to 679)	167 (140 to 199)	-70.9 (-74.4 to -67.1)	7.54 (6.5 to 8.71)	2.08 (1.81 to 2.39)	-72.3 (-75.8 to -68.6)
		Chlamydia spp.	2430 (2120 to 2770)	929 (807 to 1070)	-61.7 (-65.9 to -56.8)	7.91 (7.07 to 8.8)	2.88 (2.58 to 3.21)	-63.5 (-67.4 to -58.8)
		Enterobacter spp.	204 (171 to 239)	216 (184 to 252)	6.2 (-8.5 to 21.8)	2.54 (2.16 to 2.94)	2.55 (2.26 to 2.87)	1.1 (-12.1 to 15.0)
		Escherichia coli	551 (481 to 628)	460 (400 to 524)	-16.2 (-26.0 to -5.4)	7.08 (6.31 to 7.91)	5.63 (5.14 to 6.14)	-20.2 (-29.5 to -10.3)
		Fungus	665 (567 to 772)	484 (418 to 560)	-27.0 (-36.1 to -16.6)	8.43 (7.3 to 9.64)	5.84 (5.16 to 6.52)	-30.5 (-39.8 to -21.0)
		Group B Streptococcus	678 (591 to 784)	594 (516 to 685)	-12.2 (-23.4 to -0.8)	6.19 (5.42 to 7.05)	5.16 (4.63 to 5.74)	-16.4 (-27.3 to -5.0)
		Haemophilus influenzae	2670 (2390 to 2990)	1580 (1390 to 1800)	-40.9 (-46.8 to -33.9)	13.1 (11.9 to 14.4)	5.02 (4.59 to 5.45)	-61.7 (-65.6 to -56.9)
		Influenza	3830 (3450 to 4270)	166 (47.2 to 373)	-95.7 (-98.8 to -90.3)	36.8 (34.1 to 39.8)	1.43 (0.4 to 3.13)	-96.1 (-98.9 to -91.4)
		Klebsiella pneumoniae	1620 (1440 to 1800)	887 (781 to 1000)	-45.2 (-50.8 to -39.0)	20.7 (19.1 to 22.6)	10.8 (9.91 to 11.7)	-47.9 (-53.3 to -42.0)

		Legionella spp.	1240 (1070 to 1420)	1170 (1030 to 1320)	-5.1 (-16.9 to 8.4)	10.9 (9.63 to 12.4)	9.8 (8.93 to 10.7)	-9.7 (-21.7 to 2.2)
		Mycoplasma spp.	5990 (5330 to 6650)	3610 (3210 to 4090)	-39.6 (-45.7 to -32.8)	20.3 (18.7 to 22.2)	11.7 (10.8 to 12.7)	-42.5 (-48.7 to -36.0)
		Polymicrobial	99 (64.4 to 149)	61.3 (36.7 to 97.1)	-38.5 (-47.7 to -28.0)	1.29 (0.85 to 1.95)	0.76 (0.44 to 1.19)	-41.5 (-50.6 to -31.4)
		Pseudomonas aeruginosa	1390 (1230 to 1550)	1550 (1360 to 1750)	11.8 (-0.3 to 24.1)	12.3 (11.2 to 13.5)	13.1 (12.1 to 14.2)	6.4 (-5.1 to 18.1)
		Respiratory syncytial virus	948 (835 to 1060)	46.5 (12.9 to 105)	-95.1 (-98.6 to -89.1)	4.15 (3.77 to 4.59)	0.18 (0.05 to 0.41)	-95.6 (-98.8 to -90.2)
		Staphylococcus aureus	2250 (2020 to 2490)	2560 (2270 to 2900)	14.0 (3.2 to 25.5)	40.9 (37.7 to 43.9)	44.3 (41 to 47.8)	8.5 (-1.6 to 19.4)
		Streptococcus pneumoniae	21900 (19800 to 24100)	7010 (6220 to 7950)	-68.1 (-71.0 to -64.9)	166 (155 to 178)	50.4 (46.8 to 54.3)	-69.6 (-72.4 to -66.5)
		Other viruses	9280 (8390 to 10200)	6630 (5880 to 7510)	-28.5 (-35.2 to -21.6)	27.6 (25.6 to 29.8)	18.8 (17.3 to 20.2)	-31.9 (-38.5 to -25.1)
		Other bacterial pathogens	2420 (2090 to 2750)	1200 (1020 to 1410)	-50.3 (-55.7 to -44.3)	17.4 (15.6 to 19.4)	8.25 (7.21 to 9.35)	-52.6 (-57.9 to -46.9)
	50-69 years	*Acinetobacter baumannii*	1500 (1290 to 1730)	568 (490 to 661)	-62.0 (-66.4 to -56.7)	36.4 (31.5 to 42.2)	9.12 (7.81 to 10.6)	-74.9 (-78.4 to -70.7)
		Chlamydia spp.	3740 (3340 to 4200)	1760 (1580 to 1970)	-52.8 (-58.1 to -46.9)	22.6 (19.9 to 25.4)	7.03 (6.23 to 8.02)	-68.8 (-73.2 to -63.7)
		Enterobacter spp.	516 (442 to 603)	693 (610 to 780)	34.8 (17.9 to 54.4)	11.9 (9.99 to 13.9)	10.6 (9.26 to 12.1)	-10.8 (-24.4 to 5.4)
		Escherichia coli	1540 (1360 to 1730)	1580 (1420 to 1750)	2.7 (-7.8 to 16.7)	36.7 (32.3 to 41.6)	24.8 (22.2 to 27.8)	-32.0 (-41.8 to -20.2)
		Fungus	1480 (1290 to 1690)	1370 (1210 to 1540)	-6.7 (-17.5 to 6.6)	34.7 (30.2 to 40.1)	21.4 (18.8 to 24.4)	-38.2 (-47.6 to -27.5)
		Group B Streptococcus	1210 (1060 to 1390)	1320 (1180 to 1480)	9.1 (-4.4 to 24.0)	20.5 (17.8 to 23.5)	14.8 (13 to 16.7)	-27.8 (-38.6 to -14.8)
		Haemophilus influenzae	4090 (3730 to 4510)	3310 (3000 to 3640)	-19.0 (-27.0 to -9.5)	37.1 (33.6 to 41.3)	13.5 (12.1 to 15.1)	-63.5 (-68.6 to -57.9)
		Influenza	5050 (4610 to 5540)	288 (78.4 to 637)	-94.3 (-98.4 to -87.2)	89.8 (81.1 to 98.5)	3.19 (0.9 to 7.11)	-96.4 (-99.0 to -92.0)
		Klebsiella pneumoniae	3170 (2910 to 3490)	2510 (2290 to 2770)	-20.7 (-28.5 to -11.6)	75.1 (67.8 to 82.2)	39.3 (35.5 to 43.6)	-47.5 (-54.2 to -39.7)
		Legionella spp.	2660 (2360 to 2990)	3180 (2870 to 3530)	20.0 (5.6 to 36.8)	43.3 (38 to 49)	34.3 (30.8 to 38.1)	-20.6 (-31.9 to -7.3)
		Mycoplasma spp.	4190 (3790 to 4620)	2860 (2590 to 3150)	-31.6 (-38.7 to -23.4)	26.4 (23.6 to 29.4)	11.9 (10.7 to 13.3)	-54.7 (-61.0 to -47.8)
		Polymicrobial	215 (143 to 325)	171 (104 to 274)	-20.8 (-31.8 to -7.7)	5.19 (3.43 to 7.89)	2.73 (1.65 to 4.45)	-47.5 (-56.9 to -37.3)
		Pseudomonas aeruginosa	3000 (2720 to 3290)	4060 (3680 to 4490)	35.6 (21.4 to 51.8)	49.3 (44 to 55)	44.2 (39.7 to 49.3)	-10.2 (-22.2 to 3.3)
		Respiratory syncytial virus	923 (820 to 1030)	55.3 (15.3 to 121)	-94.0 (-98.3 to -86.8)	7.49 (6.56 to 8.44)	0.28 (0.08 to 0.62)	-96.3 (-99.0 to -91.6)
		Staphylococcus aureus	4280 (3950 to 4670)	6270 (5720 to 6900)	46.5 (32.3 to 62.4)	144 (131 to 158)	140 (126 to 155)	-3.0 (-15.2 to 11.6)

		Streptococcus pneumoniae	20600 (19100 to 22300)	9150 (8340 to 10100)	-55.6 (-59.8 to -51.0)	289 (263 to 314)	84.8 (76.8 to 94.2)	-70.6 (-74.4 to -66.2)
		Other viruses	7640 (7030 to 8270)	7870 (7180 to 8650)	3.1 (-6.6 to 14.4)	42.1 (38.3 to 46)	28.7 (25.8 to 31.9)	-31.7 (-40.6 to -21.6)
		Other bacterial pathogens	5570 (4940 to 6270)	3490 (3010 to 4010)	-37.3 (-44.2 to -29.9)	74.5 (65.7 to 83.7)	30.9 (26.7 to 35.5)	-58.4 (-64.1 to -51.9)
	70+ years	*Acinetobacter baumannii*	1740 (1480 to 2040)	959 (821 to 1110)	-44.7 (-50.9 to -37.3)	129 (112 to 148)	51.4 (41.6 to 59.4)	-60.0 (-64.5 to -55.3)
		Chlamydia spp.	5180 (4580 to 5890)	3550 (3140 to 4000)	-31.3 (-39.2 to -23.0)	95.1 (83.6 to 105)	47.3 (39.3 to 53)	-50.3 (-56.4 to -44.3)
		Enterobacter spp.	584 (491 to 697)	1140 (992 to 1310)	96.2 (68.9 to 122.6)	41 (34.3 to 47.8)	58 (47.2 to 66)	42.0 (24.0 to 60.9)
		Escherichia coli	2190 (1920 to 2510)	3270 (2930 to 3650)	49.5 (32.2 to 68.0)	159 (140 to 178)	172 (146 to 191)	8.2 (-5.3 to 20.9)
		Fungus	956 (814 to 1120)	1300 (1130 to 1480)	35.8 (18.9 to 53.3)	68.4 (58.9 to 78.7)	67.1 (55.5 to 76.7)	-1.7 (-13.8 to 11.0)
		Group B Streptococcus	1290 (1130 to 1480)	2050 (1820 to 2300)	58.8 (39.0 to 79.9)	66.6 (57.9 to 75)	76.4 (63.4 to 85.4)	14.9 (0.9 to 29.5)
		Haemophilus influenzae	4270 (3830 to 4800)	5040 (4520 to 5600)	18.0 (5.3 to 30.3)	118 (106 to 129)	68.8 (57.5 to 75.9)	-41.9 (-48.1 to -36.2)
		Influenza	8910 (8070 to 9960)	741 (203 to 1650)	-91.7 (-97.7 to -81.5)	483 (434 to 520)	27.4 (7.69 to 60.9)	-94.3 (-98.4 to -87.4)
		Klebsiella pneumoniae	4060 (3690 to 4530)	4680 (4210 to 5190)	15.4 (3.3 to 28.5)	293 (265 to 315)	244 (206 to 270)	-16.5 (-25.8 to -8.4)
		Legionella spp.	2220 (1940 to 2530)	3870 (3470 to 4290)	74.7 (53.3 to 97.9)	110 (96.6 to 124)	139 (117 to 155)	26.5 (10.7 to 41.5)
		Mycoplasma spp.	4120 (3690 to 4640)	4090 (3680 to 4540)	-0.4 (-10.9 to 12.0)	78.9 (70 to 86.3)	56.8 (48.1 to 62.9)	-27.9 (-35.7 to -20.7)
		Polymicrobial	286 (185 to 438)	332 (194 to 545)	15.4 (-1.4 to 35.3)	21 (13.7 to 32.5)	17.7 (10.5 to 29.1)	-16.5 (-29.6 to -3.1)
		Pseudomonas aeruginosa	3630 (3260 to 4080)	7150 (6450 to 7910)	97.5 (75.9 to 119.1)	182 (163 to 198)	260 (218 to 288)	42.9 (26.5 to 57.6)
		Respiratory syncytial virus	1010 (886 to 1150)	87.6 (24.1 to 193)	-91.3 (-97.6 to -80.4)	24.8 (22 to 27.6)	1.48 (0.41 to 3.25)	-94.0 (-98.4 to -86.6)
		Staphylococcus aureus	6440 (5850 to 7190)	13700 (12400 to 15200)	113.3 (91.1 to 135.2)	660 (594 to 705)	1020 (858 to 1120)	54.4 (39.1 to 68.1)
		Streptococcus pneumoniae	24700 (22500 to 27400)	15900 (14300 to 17500)	-35.4 (-41.8 to -28.8)	1050 (953 to 1120)	492 (414 to 542)	-53.2 (-58.0 to -49.1)
		Other viruses	9030 (8220 to 10100)	13500 (12200 to 15000)	50.1 (34.6 to 66.0)	151 (137 to 162)	165 (139 to 181)	8.6 (-1.9 to 18.5)
		Other bacterial pathogens	8660 (7620 to 9880)	7900 (6810 to 9130)	-8.7 (-18.7 to 2.3)	356 (315 to 395)	236 (191 to 273)	-33.8 (-41.3 to -26.8)
Uruguay	All Ages	*Acinetobacter baumannii*	970 (834 to 1120)	547 (483 to 620)	-43.4 (-49.2 to -37.1)	23.5 (20.4 to 27)	25 (21 to 28.7)	6.4 (-6.0 to 18.8)
		Chlamydia spp.	3350 (3020 to 3750)	2200 (2000 to 2400)	-34.2 (-40.7 to -27.1)	18.7 (16.8 to 20.5)	23.4 (20.1 to 26.3)	25.5 (11.3 to 40.3)
		Enterobacter spp.	395 (340 to 459)	637 (568 to 706)	61.5 (45.0 to 79.2)	8.64 (7.36 to 9.97)	26.3 (22.3 to 29.7)	205.5 (171.9 to 243.6)
		Escherichia coli	1290 (1150 to 1460)	1840 (1690 to 2000)	43.3 (29.7 to 57.5)	31 (27.5 to 34.3)	83.1 (72.1 to 92)	168.7 (138.6 to 196.8)

		Fungus	837 (728 to 956)	988 (883 to 1100)	18.3 (6.2 to 31.0)	15.5 (13.5 to 17.6)	35.4 (30.4 to 40.1)	129.4 (102.2 to 157.1)
		Group B Streptococcus	975 (862 to 1100)	1280 (1170 to 1410)	31.4 (17.2 to 44.5)	14.8 (13.2 to 16.6)	36.1 (31.2 to 40.2)	144.0 (115.8 to 174.9)
		Haemophilus influenzae	4070 (3700 to 4500)	3420 (3160 to 3690)	-15.7 (-22.6 to -9.4)	28.7 (26.2 to 30.9)	33.1 (28.8 to 36.4)	15.3 (4.6 to 25.7)
		Influenza	5810 (5310 to 6390)	602 (282 to 1050)	-89.6 (-95.0 to -81.7)	95.4 (87.6 to 102)	18.8 (9.05 to 32.4)	-80.3 (-90.5 to -65.8)
		Klebsiella pneumoniae	2880 (2640 to 3180)	2540 (2350 to 2730)	-11.8 (-18.5 to -5.0)	60.7 (55.6 to 65)	109 (94 to 119)	79.0 (63.3 to 95.5)
		Legionella spp.	1760 (1560 to 1980)	2200 (2040 to 2390)	25.8 (13.3 to 39.4)	24.8 (22.1 to 27.7)	59.4 (51.5 to 65.4)	139.7 (113.6 to 166.2)
		Mycoplasma spp.	4530 (4140 to 4970)	3630 (3360 to 3920)	-19.8 (-26.2 to -13.9)	19.1 (17.4 to 20.7)	27.8 (24.7 to 30.4)	46.0 (31.7 to 60.0)
		Polymicrobial	175 (115 to 266)	201 (127 to 316)	14.6 (-0.6 to 30.5)	4.69 (3.31 to 6.77)	9.19 (5.71 to 14.7)	94.8 (56.3 to 130.0)
		Pseudomonas aeruginosa	2530 (2310 to 2810)	3600 (3330 to 3880)	42.1 (29.8 to 54.3)	38.8 (35.2 to 42)	103 (89.6 to 113)	166.6 (138.8 to 191.9)
		Respiratory syncytial virus	2850 (2490 to 3250)	162 (76.5 to 282)	-94.3 (-97.3 to -90.1)	14.1 (13.1 to 15)	1.02 (0.49 to 1.77)	-92.8 (-96.5 to -87.5)
		Staphylococcus aureus	3960 (3630 to 4370)	7030 (6460 to 7580)	77.7 (64.6 to 90.9)	129 (118 to 139)	445 (385 to 485)	244.9 (214.5 to 274.8)
		Streptococcus pneumoniae	24200 (22300 to 26400)	9270 (8630 to 9930)	-61.7 (-64.2 to -59.2)	244 (227 to 259)	201 (174 to 218)	-17.9 (-24.9 to -11.1)
		Other viruses	11700 (10700 to 12800)	8530 (7920 to 9140)	-26.9 (-32.0 to -22.0)	42.3 (39.5 to 44.9)	66 (57.7 to 71.9)	56.1 (42.0 to 69.9)
		Other bacterial pathogens	4620 (4090 to 5230)	3980 (3530 to 4440)	-13.8 (-21.9 to -4.9)	64.7 (57.1 to 71.6)	106 (89.1 to 121)	63.9 (45.6 to 82.4)
	Under 5	*Acinetobacter baumannii*	28.3 (20.2 to 39)	5.89 (4.24 to 8.02)	-79.2 (-81.8 to -76.0)	0.96 (0.58 to 1.5)	0.15 (0.08 to 0.26)	-84.7 (-88.7 to -79.9)
		Chlamydia spp.	388 (319 to 467)	113 (92.2 to 139)	-70.7 (-75.1 to -66.0)	1.51 (1.35 to 1.68)	0.23 (0.17 to 0.29)	-85.0 (-88.7 to -80.7)
		Enterobacter spp.	39.2 (29.7 to 50.5)	26.4 (19.7 to 35.1)	-32.5 (-43.4 to -20.3)	0.65 (0.47 to 0.9)	0.18 (0.13 to 0.26)	-71.7 (-78.9 to -62.9)
		Escherichia coli	137 (111 to 164)	95.9 (78.2 to 117)	-29.5 (-40.4 to -17.9)	2.11 (1.83 to 2.41)	0.6 (0.47 to 0.77)	-71.3 (-78.5 to -62.6)
		Fungus	27.2 (18.7 to 37.6)	14.4 (10.1 to 19.7)	-46.6 (-55.6 to -36.9)	0.26 (0.18 to 0.35)	0.06 (0.04 to 0.09)	-74.4 (-81.5 to -65.8)
		Group B Streptococcus	117 (94.4 to 141)	67.1 (53.7 to 82.5)	-42.3 (-51.8 to -32.7)	1.66 (1.46 to 1.87)	0.39 (0.3 to 0.51)	-76.2 (-82.1 to -69.3)
		Haemophilus influenzae	1050 (856 to 1260)	395 (327 to 480)	-62.2 (-67.3 to -56.9)	4.72 (4.18 to 5.28)	0.49 (0.37 to 0.63)	-89.6 (-92.3 to -86.3)
		Influenza	879 (734 to 1050)	32.1 (14.9 to 54.6)	-96.3 (-98.3 to -93.7)	7.35 (6.66 to 8.11)	0.12 (0.05 to 0.2)	-98.4 (-99.3 to -97.2)
		Klebsiella pneumoniae	514 (429 to 609)	154 (127 to 187)	-70.0 (-73.8 to -66.2)	5.98 (5.36 to 6.66)	0.91 (0.7 to 1.15)	-84.8 (-88.5 to -80.3)
		Legionella spp.	45 (31.7 to 63.8)	23.2 (15.5 to 33.6)	-48.5 (-56.4 to -40.2)	0.72 (0.54 to 0.94)	0.17 (0.12 to 0.24)	-75.9 (-81.9 to -68.5)

		Mycoplasma spp.	599 (498 to 714)	358 (293 to 433)	-40.0 (-47.8 to -31.6)	1.85 (1.64 to 2.07)	0.49 (0.38 to 0.63)	-73.4 (-80.0 to -65.0)
		Polymicrobial	18.5 (11.5 to 27.8)	6.36 (3.94 to 9.7)	-65.5 (-71.7 to -57.7)	0.93 (0.52 to 1.52)	0.2 (0.11 to 0.36)	-77.9 (-83.7 to -70.8)
		Pseudomonas aeruginosa	282 (233 to 339)	226 (186 to 274)	-19.8 (-30.7 to -8.3)	2.75 (2.48 to 3.06)	0.92 (0.71 to 1.16)	-66.7 (-74.7 to -56.9)
		Respiratory syncytial virus	2020 (1700 to 2390)	89.6 (41.7 to 154)	-95.6 (-97.9 to -92.3)	8.77 (8.02 to 9.56)	0.16 (0.08 to 0.29)	-98.1 (-99.1 to -96.7)
		Staphylococcus aureus	360 (298 to 426)	264 (218 to 319)	-26.6 (-34.7 to -18.2)	5.8 (5.22 to 6.35)	1.92 (1.49 to 2.44)	-66.8 (-74.9 to -57.1)
		Streptococcus pneumoniae	5810 (4860 to 6920)	715 (589 to 862)	-87.7 (-89.0 to -86.4)	33.9 (30.6 to 37.2)	1.94 (1.49 to 2.49)	-94.3 (-95.7 to -92.5)
		Other viruses	4250 (3530 to 5060)	1280 (1060 to 1540)	-69.8 (-73.0 to -66.6)	10.2 (9.25 to 11.2)	1.55 (1.19 to 1.98)	-84.8 (-88.4 to -80.3)
		Other bacterial pathogens	156 (119 to 199)	59.4 (45.9 to 75.8)	-61.7 (-67.3 to -55.2)	1.2 (0.95 to 1.49)	0.22 (0.16 to 0.3)	-81.6 (-86.1 to -76.0)
	5-14 years	*Acinetobacter baumannii*	53.7 (39.3 to 71.6)	21.1 (15.7 to 28)	-60.6 (-65.3 to -55.2)	0.06 (0.05 to 0.07)	0.03 (0.02 to 0.04)	-50.9 (-61.0 to -39.5)
		Chlamydia spp.	237 (177 to 309)	121 (90.5 to 160)	-48.7 (-54.7 to -42.1)	0.07 (0.06 to 0.08)	0.04 (0.04 to 0.05)	-36.1 (-48.8 to -21.5)
		Enterobacter spp.	21.1 (15.3 to 28.5)	25.6 (19 to 33.8)	21.9 (6.3 to 38.1)	0.02 (0.02 to 0.03)	0.04 (0.03 to 0.04)	51.7 (20.0 to 86.7)
		Escherichia coli	55.5 (40.8 to 72.7)	60.8 (46 to 78.4)	9.9 (-2.7 to 23.5)	0.06 (0.06 to 0.07)	0.09 (0.07 to 0.1)	36.9 (10.3 to 66.5)
		Fungus	66.9 (48.8 to 88.5)	59.8 (44.7 to 78.4)	-10.3 (-21.6 to 1.9)	0.08 (0.06 to 0.09)	0.08 (0.07 to 0.1)	11.6 (-11.7 to 38.1)
		Group B Streptococcus	69.1 (50.4 to 89.9)	71.8 (53.3 to 94.7)	4.2 (-9.1 to 17.7)	0.06 (0.05 to 0.07)	0.07 (0.06 to 0.09)	29.8 (2.9 to 60.5)
		Haemophilus influenzae	276 (205 to 362)	187 (140 to 245)	-32.1 (-39.2 to -24.9)	0.12 (0.11 to 0.14)	0.07 (0.06 to 0.08)	-41.9 (-53.3 to -30.2)
		Influenza	394 (296 to 509)	30.2 (13.6 to 54)	-92.3 (-96.4 to -86.6)	0.34 (0.3 to 0.38)	0.03 (0.01 to 0.05)	-90.9 (-95.8 to -84.0)
		Klebsiella pneumoniae	163 (122 to 213)	101 (76.5 to 131)	-38.1 (-44.0 to -31.6)	0.19 (0.17 to 0.21)	0.14 (0.12 to 0.17)	-23.0 (-37.9 to -6.5)
		Legionella spp.	132 (97.9 to 174)	124 (93.7 to 163)	-5.9 (-16.5 to 5.5)	0.1 (0.09 to 0.12)	0.12 (0.1 to 0.14)	17.2 (-6.4 to 45.4)
		Mycoplasma spp.	615 (458 to 800)	427 (323 to 558)	-30.5 (-37.7 to -22.8)	0.19 (0.16 to 0.21)	0.16 (0.14 to 0.19)	-13.5 (-30.3 to 4.4)
		Polymicrobial	9.65 (6.02 to 14.7)	8.29 (4.92 to 12.9)	-14.2 (-25.7 to -2.5)	0.01 (0.01 to 0.02)	0.01 (0.01 to 0.02)	6.8 (-15.4 to 31.3)
		Pseudomonas aeruginosa	147 (110 to 190)	165 (124 to 214)	12.5 (1.3 to 25.5)	0.12 (0.1 to 0.13)	0.16 (0.14 to 0.19)	40.0 (13.7 to 67.2)
		Respiratory syncytial virus	102 (76.9 to 133)	6.81 (3.03 to 12.2)	-93.3 (-96.8 to -88.2)	0.04 (0.04 to 0.05)	0 (0 to 0.01)	-92.1 (-96.3 to -86.0)
		Staphylococcus aureus	233 (176 to 301)	301 (229 to 390)	29.1 (17.2 to 41.6)	0.38 (0.34 to 0.43)	0.61 (0.52 to 0.7)	60.7 (30.5 to 94.0)
		Streptococcus pneumoniae	2240 (1680 to 2880)	693 (525 to 901)	-69.0 (-71.7 to -66.2)	1.52 (1.36 to 1.7)	0.58 (0.5 to 0.68)	-61.4 (-68.9 to -53.6)
		Other viruses	992 (750 to 1280)	637 (485 to 830)	-35.7 (-41.5 to -30.0)	0.26 (0.24 to 0.3)	0.21 (0.18 to 0.24)	-19.9 (-35.1 to -3.6)

		Other bacterial pathogens	242 (180 to 316)	148 (111 to 194)	-38.7 (-45.2 to -31.7)	0.16 (0.14 to 0.18)	0.12 (0.1 to 0.14)	-23.6 (-38.0 to -7.9)
	15-49 years	*Acinetobacter baumannii*	95.6 (79.1 to 115)	47.3 (39.9 to 56)	-50.4 (-56.5 to -44.5)	0.59 (0.5 to 0.68)	0.57 (0.49 to 0.65)	-3.3 (-15.6 to 10.2)
		Chlamydia spp.	422 (362 to 490)	272 (235 to 311)	-35.5 (-42.7 to -28.0)	0.64 (0.58 to 0.72)	0.81 (0.72 to 0.91)	25.7 (10.4 to 42.7)
		Enterobacter spp.	37.5 (31.2 to 44.4)	57.3 (49.1 to 66.2)	53.2 (34.8 to 71.4)	0.22 (0.19 to 0.25)	0.65 (0.57 to 0.74)	198.6 (160.4 to 240.3)
		Escherichia coli	98.8 (85 to 115)	136 (120 to 154)	38.2 (24.1 to 53.7)	0.6 (0.53 to 0.67)	1.6 (1.46 to 1.75)	169.4 (135.3 to 205.5)
		Fungus	119 (99.4 to 140)	134 (115 to 155)	12.8 (-0.2 to 28.4)	0.71 (0.61 to 0.81)	1.55 (1.35 to 1.77)	119.7 (89.6 to 151.4)
		Group B Streptococcus	123 (105 to 144)	161 (139 to 186)	31.1 (15.3 to 47.9)	0.53 (0.46 to 0.6)	1.34 (1.19 to 1.5)	155.4 (119.8 to 192.3)
		Haemophilus influenzae	492 (429 to 562)	419 (372 to 475)	-14.6 (-22.6 to -6.2)	1.13 (1.04 to 1.24)	1.29 (1.17 to 1.4)	14.4 (2.3 to 27.4)
		Influenza	702 (618 to 794)	67.7 (32 to 119)	-90.4 (-95.5 to -83.5)	3.15 (2.92 to 3.42)	0.56 (0.27 to 0.98)	-82.1 (-91.6 to -68.2)
		Klebsiella pneumoniae	290 (254 to 330)	226 (200 to 254)	-22.2 (-29.1 to -14.9)	1.74 (1.61 to 1.89)	2.63 (2.42 to 2.86)	51.6 (36.0 to 67.9)
		Legionella spp.	236 (202 to 272)	278 (246 to 315)	18.3 (5.6 to 32.5)	0.97 (0.86 to 1.1)	2.23 (2.04 to 2.45)	130.6 (99.9 to 162.9)
		Mycoplasma spp.	1100 (961 to 1250)	955 (848 to 1080)	-12.7 (-20.4 to -4.4)	1.74 (1.6 to 1.9)	2.96 (2.71 to 3.25)	70.2 (51.4 to 90.0)
		Polymicrobial	17.2 (10.9 to 26.5)	18.6 (11.4 to 29.2)	7.9 (-5.1 to 23.6)	0.1 (0.07 to 0.16)	0.22 (0.13 to 0.34)	110.2 (79.6 to 144.5)
		Pseudomonas aeruginosa	261 (227 to 298)	369 (329 to 413)	41.5 (28.0 to 55.8)	1.09 (0.99 to 1.19)	2.99 (2.75 to 3.25)	175.7 (144.9 to 208.1)
		Respiratory syncytial virus	181 (157 to 207)	15.3 (7.23 to 27)	-91.6 (-96.1 to -85.5)	0.37 (0.34 to 0.41)	0.06 (0.03 to 0.1)	-84.4 (-92.6 to -72.2)
		Staphylococcus aureus	415 (364 to 471)	673 (601 to 751)	62.3 (49.6 to 76.7)	3.53 (3.29 to 3.79)	11.2 (10.4 to 12)	216.3 (186.5 to 249.0)
		Streptococcus pneumoniae	3990 (3520 to 4510)	1550 (1390 to 1730)	-61.1 (-64.1 to -57.8)	14.1 (13.2 to 15.1)	10.7 (9.93 to 11.5)	-24.1 (-31.2 to -16.6)
		Other viruses	1770 (1550 to 1990)	1430 (1280 to 1600)	-19.1 (-25.4 to -12.1)	2.46 (2.28 to 2.65)	3.87 (3.59 to 4.19)	57.7 (42.4 to 73.8)
		Other bacterial pathogens	431 (365 to 501)	332 (285 to 383)	-22.9 (-31.0 to -14.7)	1.45 (1.3 to 1.62)	2.19 (1.92 to 2.46)	50.4 (32.9 to 68.9)
	50-69 years	*Acinetobacter baumannii*	326 (277 to 388)	153 (134 to 177)	-52.8 (-58.5 to -46.8)	4.11 (3.57 to 4.77)	2.98 (2.58 to 3.41)	-27.3 (-36.9 to -16.4)
		Chlamydia spp.	853 (747 to 995)	487 (434 to 543)	-42.8 (-50.1 to -35.6)	2.67 (2.38 to 2.98)	2.35 (2.09 to 2.64)	-11.8 (-23.2 to 1.3)
		Enterobacter spp.	124 (105 to 148)	174 (153 to 196)	40.5 (23.3 to 59.2)	1.49 (1.27 to 1.71)	3.2 (2.84 to 3.65)	116.6 (87.7 to 149.6)
		Escherichia coli	362 (317 to 423)	435 (392 to 482)	20.6 (6.3 to 34.4)	4.47 (3.98 to 5)	8.29 (7.48 to 9.22)	86.0 (62.5 to 112.7)
		Fungus	347 (296 to 404)	361 (316 to 406)	4.3 (-8.3 to 17.4)	4.23 (3.66 to 4.82)	6.78 (5.93 to 7.77)	60.7 (38.1 to 83.5)

		Group B Streptococcus	288 (248 to 336)	336 (297 to 376)	16.9 (2.1 to 30.4)	2.53 (2.21 to 2.9)	4.55 (4 to 5.11)	80.3 (54.9 to 108.1)
		Haemophilus influenzae	984 (879 to 1130)	842 (768 to 923)	-14.3 (-23.3 to -5.6)	4.64 (4.21 to 5.08)	4.21 (3.8 to 4.65)	-9.2 (-19.6 to 2.2)
		Influenza	1210 (1080 to 1370)	113 (53.9 to 200)	-90.6 (-95.6 to -83.2)	11.2 (10.2 to 12.1)	1.53 (0.73 to 2.65)	-86.3 (-93.4 to -75.8)
		Klebsiella pneumoniae	744 (669 to 847)	625 (570 to 681)	-15.9 (-24.7 to -8.2)	9.15 (8.36 to 9.96)	11.8 (10.8 to 13)	29.7 (15.1 to 46.4)
		Legionella spp.	663 (580 to 767)	712 (646 to 783)	7.8 (-5.2 to 21.5)	5.6 (4.99 to 6.28)	9.29 (8.37 to 10.2)	66.2 (44.2 to 89.3)
		Mycoplasma spp.	1000 (890 to 1160)	684 (619 to 754)	-31.8 (-39.5 to -24.2)	3.28 (2.98 to 3.61)	3.44 (3.1 to 3.81)	5.2 (-8.3 to 19.1)
		Polymicrobial	48.9 (31.8 to 74.6)	49.4 (31.2 to 76.8)	0.9 (-12.7 to 14.3)	0.61 (0.4 to 0.93)	0.96 (0.59 to 1.51)	55.6 (31.5 to 83.3)
		Pseudomonas aeruginosa	742 (658 to 844)	895 (817 to 978)	21.1 (8.1 to 33.4)	6.33 (5.74 to 6.98)	11.8 (10.7 to 12.9)	86.7 (64.0 to 111.9)
		Respiratory syncytial virus	232 (201 to 267)	16.9 (7.83 to 29.7)	-92.7 (-96.5 to -87.3)	0.97 (0.86 to 1.09)	0.1 (0.05 to 0.18)	-89.3 (-94.9 to -81.1)
		Staphylococcus aureus	1040 (927 to 1170)	1570 (1430 to 1710)	51.5 (36.2 to 65.2)	18.1 (16.7 to 19.6)	42.2 (38.4 to 46.4)	133.5 (107.7 to 162.0)
		Streptococcus pneumoniae	4920 (4440 to 5590)	2010 (1840 to 2180)	-59.1 (-63.3 to -55.4)	35.7 (32.9 to 38.6)	22.5 (20.5 to 24.5)	-37.0 (-44.1 to -29.1)
		Other viruses	1900 (1710 to 2160)	1660 (1520 to 1810)	-12.6 (-21.2 to -4.8)	5.44 (5.01 to 5.91)	7.32 (6.68 to 8.02)	34.7 (19.3 to 51.6)
		Other bacterial pathogens	1300 (1140 to 1510)	908 (792 to 1030)	-30.1 (-37.7 to -22.0)	9.03 (8.06 to 10.1)	9.72 (8.51 to 11.1)	7.8 (-5.7 to 22.5)
	70+ years	*Acinetobacter baumannii*	466 (392 to 552)	319 (276 to 370)	-31.2 (-39.7 to -21.7)	17.8 (15 to 20.6)	21.2 (17.5 to 24.7)	19.7 (5.2 to 34.7)
		Chlamydia spp.	1450 (1240 to 1700)	1210 (1060 to 1380)	-16.5 (-26.8 to -4.7)	13.8 (12 to 15.4)	20 (16.8 to 22.5)	45.2 (28.3 to 63.8)
		Enterobacter spp.	173 (143 to 211)	353 (306 to 405)	105.0 (78.7 to 134.1)	6.26 (5.23 to 7.4)	22.3 (18.4 to 25.4)	256.5 (215.7 to 303.0)
		Escherichia coli	635 (541 to 752)	1110 (986 to 1250)	76.0 (54.9 to 97.5)	23.7 (20.7 to 26.6)	72.6 (61.8 to 80.9)	206.1 (169.4 to 240.1)
		Fungus	276 (231 to 328)	419 (357 to 483)	52.1 (33.3 to 73.6)	10.2 (8.77 to 11.9)	26.9 (22.6 to 31)	164.6 (132.0 to 199.2)
		Group B Streptococcus	378 (320 to 443)	643 (566 to 727)	70.6 (49.4 to 92.5)	10 (8.63 to 11.5)	29.8 (25 to 33.5)	196.8 (159.9 to 236.7)
		Haemophilus influenzae	1270 (1100 to 1460)	1580 (1400 to 1760)	25.0 (11.2 to 39.7)	18.1 (16 to 19.8)	27 (22.9 to 30.1)	49.5 (34.7 to 64.9)
		Influenza	2630 (2280 to 3040)	359 (168 to 625)	-86.3 (-93.3 to -75.8)	73.4 (65.8 to 79.6)	16.5 (7.99 to 28.3)	-77.4 (-89.2 to -60.7)
		Klebsiella pneumoniae	1170 (1030 to 1360)	1430 (1280 to 1600)	22.7 (9.3 to 36.8)	43.6 (38.9 to 47.5)	93 (78.7 to 103)	113.4 (93.7 to 134.7)
		Legionella spp.	681 (576 to 801)	1070 (948 to 1190)	57.3 (37.8 to 78.9)	17.4 (15.2 to 19.7)	47.6 (39.9 to 53)	173.6 (141.8 to 205.6)
		Mycoplasma spp.	1210 (1060 to 1400)	1200 (1070 to 1340)	-0.5 (-12.2 to 11.6)	12 (10.7 to 13.2)	20.8 (17.6 to 23.1)	73.1 (54.3 to 91.4)
		Polymicrobial	80.2 (50.3 to 128)	118 (72.9 to 190)	47.2 (25.6 to 69.1)	3.04 (1.98 to 4.63)	7.8 (4.77 to 12.6)	156.0 (121.5 to 194.1)

		Pseudomonas aeruginosa	1100 (964 to 1280)	1940 (1720 to 2160)	76.6 (56.5 to 97.0)	28.5 (25.3 to 31.2)	87.5 (74.5 to 96.7)	207.2 (173.9 to 238.6)
		Respiratory syncytial virus	310 (264 to 364)	33 (15.6 to 57.1)	-89.3 (-94.9 to -81.0)	3.95 (3.45 to 4.42)	0.69 (0.33 to 1.2)	-82.4 (-91.5 to -69.6)
		Staphylococcus aureus	1920 (1680 to 2200)	4230 (3790 to 4680)	121.0 (97.7 to 145.9)	101 (90.8 to 110)	389 (328 to 426)	284.4 (248.8 to 321.8)
		Streptococcus pneumoniae	7230 (6350 to 8310)	4300 (3850 to 4750)	-40.4 (-46.6 to -34.1)	159 (143 to 172)	165 (140 to 181)	3.7 (-5.8 to 13.5)
		Other viruses	2770 (2420 to 3200)	3520 (3140 to 3900)	27.5 (14.3 to 42.1)	23.9 (21.5 to 25.9)	53.1 (44.9 to 58.4)	121.8 (101.4 to 143.3)
		Other bacterial pathogens	2490 (2130 to 2900)	2530 (2190 to 2890)	1.9 (-9.7 to 14.3)	52.9 (45.8 to 58.8)	93.8 (77.5 to 107)	77.5 (57.5 to 98.5)
Western Europe	All Ages	*Acinetobacter baumannii*	31400 (27100 to 36200)	21800 (19000 to 25000)	-30.5 (-36.2 to -24.3)	1550 (1320 to 1790)	1050 (833 to 1210)	-32.2 (-39.4 to -25.6)
		Chlamydia spp.	142000 (129000 to 155000)	110000 (102000 to 121000)	-22.0 (-28.4 to -15.3)	1710 (1510 to 1870)	1310 (1090 to 1460)	-23.5 (-31.5 to -15.7)
		Enterobacter spp.	27200 (24000 to 30600)	52300 (47100 to 57700)	92.5 (74.8 to 110.1)	1240 (1060 to 1410)	2350 (1900 to 2640)	89.0 (67.2 to 108.9)
		Escherichia coli	73900 (67200 to 81300)	123000 (114000 to 133000)	66.0 (52.8 to 78.8)	3740 (3310 to 4090)	6010 (5000 to 6600)	60.6 (43.3 to 74.7)
		Fungus	46400 (41700 to 52000)	62700 (57100 to 69100)	35.3 (23.4 to 47.8)	1790 (1580 to 2000)	2450 (2040 to 2770)	37.0 (21.8 to 50.4)
		Group B Streptococcus	57600 (52300 to 63500)	90800 (83800 to 98800)	57.9 (43.6 to 73.0)	1830 (1590 to 2020)	2860 (2360 to 3180)	56.3 (39.5 to 72.9)
		Haemophilus influenzae	231000 (216000 to 247000)	250000 (233000 to 268000)	8.6 (3.1 to 14.4)	3530 (3180 to 3740)	2750 (2310 to 3010)	-22.1 (-28.7 to -17.1)
		Influenza	363000 (341000 to 387000)	40100 (26100 to 59400)	-89.0 (-92.8 to -83.8)	13500 (12100 to 14300)	1420 (915 to 2110)	-89.5 (-93.2 to -84.4)
		Klebsiella pneumoniae	149000 (140000 to 159000)	172000 (160000 to 184000)	15.3 (9.6 to 21.0)	6970 (6260 to 7380)	8030 (6720 to 8770)	15.1 (5.1 to 22.3)
		Legionella spp.	162000 (148000 to 178000)	236000 (220000 to 254000)	45.8 (33.3 to 58.7)	4760 (4220 to 5210)	6890 (5760 to 7560)	44.8 (29.9 to 59.4)
		Mycoplasma spp.	250000 (232000 to 269000)	230000 (216000 to 246000)	-7.7 (-13.1 to -1.9)	2440 (2190 to 2630)	2200 (1840 to 2410)	-9.8 (-18.1 to -2.9)
		Polymicrobial	7760 (4730 to 12700)	10500 (5980 to 17900)	35.1 (20.1 to 51.0)	385 (232 to 634)	512 (289 to 879)	32.0 (14.3 to 48.6)
		Pseudomonas aeruginosa	211000 (196000 to 230000)	367000 (343000 to 392000)	73.8 (63.5 to 84.9)	6920 (6210 to 7430)	11700 (9600 to 12700)	68.2 (51.6 to 80.9)
		Respiratory syncytial virus	127000 (117000 to 138000)	10200 (6680 to 15100)	-91.9 (-94.7 to -88.1)	1290 (1160 to 1400)	97.8 (62.5 to 146)	-92.4 (-95.2 to -88.7)
		Staphylococcus aureus	285000 (268000 to 304000)	603000 (563000 to 648000)	111.5 (102.8 to 120.8)	20200 (18100 to 21300)	41600 (34300 to 45400)	105.7 (88.6 to 116.8)
		Streptococcus pneumoniae	1220000 (1150000 to 1290000)	670000 (630000 to 715000)	-45.0 (-47.0 to -43.0)	29400 (26700 to 30900)	16700 (14000 to 18200)	-43.1 (-47.9 to -40.2)
		Other viruses	742000 (702000 to 789000)	818000 (770000 to 870000)	10.3 (5.9 to 14.7)	6570 (5970 to 6900)	7690 (6470 to 8360)	16.9 (7.5 to 23.3)
		Other bacterial pathogens	268000 (236000 to 303000)	264000 (231000 to 303000)	-1.2 (-7.8 to 6.4)	7890 (6750 to 8920)	7510 (5960 to 8780)	-4.8 (-14.0 to 3.3)

	Under 5	*Acinetobacter baumannii*	305 (203 to 450)	132 (85.9 to 201)	-56.9 (-61.6 to -51.7)	7.91 (4.66 to 12.9)	2.19 (1.1 to 3.93)	-72.7 (-77.8 to -67.6)
		Chlamydia spp.	4520 (3790 to 5360)	2170 (1780 to 2640)	-51.9 (-57.8 to -46.0)	14.3 (13.1 to 15.5)	2.78 (2.38 to 3.18)	-80.5 (-83.1 to -77.8)
		Enterobacter spp.	714 (540 to 931)	797 (589 to 1060)	11.9 (-3.6 to 29.0)	9.29 (6.96 to 12.5)	3.47 (2.53 to 4.74)	-62.6 (-67.8 to -56.8)
		Escherichia coli	2040 (1680 to 2460)	2300 (1850 to 2780)	13.1 (-1.8 to 27.7)	25.5 (23.2 to 28.3)	9.03 (7.84 to 10.3)	-64.6 (-69.3 to -60.0)
		Fungus	384 (266 to 537)	335 (235 to 469)	-12.4 (-25.5 to 1.9)	3.31 (2.42 to 4.33)	0.82 (0.58 to 1.09)	-75.2 (-79.5 to -70.5)
		Group B Streptococcus	1920 (1580 to 2330)	1840 (1490 to 2260)	-3.8 (-16.3 to 9.0)	20.1 (18.2 to 22.1)	6.66 (5.73 to 7.6)	-66.8 (-71.3 to -62.6)
		Haemophilus influenzae	17700 (14600 to 21100)	11000 (9040 to 13400)	-38.0 (-44.6 to -30.9)	64.6 (60.3 to 69.4)	7.33 (6.47 to 8.17)	-88.6 (-90.2 to -87.0)
		Influenza	14600 (12100 to 17200)	776 (504 to 1190)	-94.7 (-96.4 to -92.2)	104 (98.9 to 110)	1.61 (1.03 to 2.44)	-98.5 (-99.0 to -97.7)
		Klebsiella pneumoniae	7020 (5880 to 8340)	3640 (3000 to 4440)	-48.1 (-53.1 to -42.8)	70.8 (66.8 to 74.9)	12.5 (11.1 to 14)	-82.3 (-84.3 to -80.1)
		Legionella spp.	1050 (752 to 1450)	955 (655 to 1330)	-9.2 (-20.2 to 2.4)	11.4 (8.89 to 14.1)	4.12 (3.19 to 5.17)	-63.6 (-68.7 to -57.9)
		Mycoplasma spp.	10200 (8490 to 12400)	10200 (8380 to 12400)	-0.5 (-10.7 to 10.6)	26.1 (24.1 to 28.4)	7.48 (6.57 to 8.36)	-71.2 (-74.9 to -67.2)
		Polymicrobial	365 (206 to 592)	249 (137 to 407)	-31.9 (-38.9 to -24.7)	12.1 (6.31 to 20.4)	4.95 (2.39 to 8.68)	-59.2 (-65.9 to -52.6)
		Pseudomonas aeruginosa	6120 (5140 to 7390)	8430 (6940 to 10200)	37.8 (23.2 to 54.8)	47.1 (43.5 to 51)	19.2 (17 to 21.5)	-59.2 (-64.5 to -54.1)
		Respiratory syncytial virus	49200 (41700 to 58100)	3370 (2200 to 5200)	-93.1 (-95.4 to -90.0)	172 (165 to 179)	3.49 (2.21 to 5.28)	-98.0 (-98.7 to -97.0)
		Staphylococcus aureus	6500 (5460 to 7660)	7890 (6510 to 9540)	21.4 (12.9 to 30.8)	90.7 (86.2 to 95.3)	32.9 (29.3 to 36.4)	-63.8 (-67.9 to -59.9)
		Streptococcus pneumoniae	89900 (75100 to 106000)	19200 (15800 to 23200)	-78.7 (-80.2 to -77.2)	477 (461 to 495)	27.9 (24.7 to 30.8)	-94.2 (-94.8 to -93.5)
		Other viruses	92400 (77600 to 109000)	49100 (40600 to 59400)	-46.9 (-50.5 to -43.5)	197 (189 to 204)	32.5 (28.9 to 35.7)	-83.5 (-85.3 to -81.7)
		Other bacterial pathogens	2150 (1650 to 2720)	1360 (1030 to 1770)	-36.8 (-45.1 to -28.1)	13.9 (11.1 to 17.4)	3.04 (2.3 to 4.17)	-78.1 (-81.7 to -73.9)
	5-14 years	*Acinetobacter baumannii*	560 (410 to 761)	271 (201 to 361)	-51.5 (-55.9 to -46.8)	2.06 (1.77 to 2.38)	0.37 (0.32 to 0.43)	-81.9 (-83.8 to -79.9)
		Chlamydia spp.	3530 (2610 to 4640)	2070 (1560 to 2710)	-41.4 (-45.8 to -36.3)	3.18 (2.9 to 3.48)	0.71 (0.64 to 0.78)	-77.7 (-79.8 to -75.3)
		Enterobacter spp.	491 (357 to 662)	700 (512 to 923)	43.0 (29.5 to 58.3)	1.67 (1.46 to 1.89)	0.92 (0.81 to 1.03)	-44.8 (-50.7 to -38.5)
		Escherichia coli	1060 (782 to 1380)	1320 (996 to 1710)	24.4 (13.9 to 34.7)	3.74 (3.41 to 4.08)	1.79 (1.67 to 1.92)	-52.1 (-56.6 to -47.6)
		Fungus	1270 (942 to 1690)	1330 (996 to 1750)	4.5 (-4.8 to 14.6)	4.43 (4 to 4.89)	1.77 (1.6 to 1.96)	-59.9 (-63.9 to -55.6)
		Group B Streptococcus	1450 (1060 to 1900)	1760 (1330 to 2310)	21.6 (9.5 to 34.1)	3.63 (3.27 to 4.06)	1.69 (1.54 to 1.86)	-53.2 (-58.4 to -47.2)

		Haemophilus influenzae	6410 (4890 to 8390)	5050 (3810 to 6580)	-21.2 (-25.8 to -16.5)	8.16 (7.75 to 8.59)	1.76 (1.64 to 1.88)	-78.5 (-80.0 to -76.8)
		Influenza	8800 (6640 to 11400)	759 (453 to 1230)	-91.4 (-94.3 to -87.4)	23.1 (22.1 to 24.1)	0.7 (0.45 to 1.07)	-96.9 (-98.0 to -95.4)
		Klebsiella pneumoniae	3090 (2330 to 3990)	2340 (1780 to 3010)	-24.3 (-27.6 to -20.5)	10.9 (10.3 to 11.4)	3.14 (2.95 to 3.32)	-71.1 (-73.1 to -69.2)
		Legionella spp.	4050 (3040 to 5360)	4530 (3460 to 5860)	12.0 (2.5 to 22.1)	9.66 (8.94 to 10.6)	4.18 (3.91 to 4.43)	-56.6 (-61.1 to -52.2)
		Mycoplasma spp.	13700 (10200 to 17800)	10900 (8390 to 14400)	-20.2 (-25.0 to -15.5)	12.7 (12 to 13.5)	3.9 (3.66 to 4.15)	-69.3 (-71.9 to -66.6)
		Polymicrobial	144 (83.2 to 233)	142 (77.3 to 236)	-1.9 (-12.9 to 8.9)	0.52 (0.31 to 0.85)	0.2 (0.11 to 0.33)	-62.6 (-66.9 to -57.7)
		Pseudomonas aeruginosa	4200 (3170 to 5520)	5660 (4330 to 7320)	35.0 (26.2 to 43.5)	10.1 (9.48 to 10.8)	5.27 (4.95 to 5.59)	-47.9 (-51.8 to -43.8)
		Respiratory syncytial virus	3290 (2480 to 4300)	258 (153 to 420)	-92.2 (-94.9 to -88.4)	3.9 (3.63 to 4.18)	0.11 (0.07 to 0.17)	-97.2 (-98.2 to -95.8)
		Staphylococcus aureus	5610 (4230 to 7290)	8520 (6520 to 10900)	52.0 (47.0 to 57.5)	27.8 (26.7 to 28.9)	16.3 (15.4 to 17.2)	-41.3 (-44.8 to -37.7)
		Streptococcus pneumoniae	47300 (35900 to 61600)	18800 (14400 to 24300)	-60.2 (-61.3 to -58.9)	97.8 (94.5 to 101)	14.8 (14.1 to 15.6)	-84.9 (-85.8 to -83.9)
		Other viruses	30200 (22800 to 39300)	24500 (18900 to 31600)	-18.8 (-21.3 to -16.1)	24.3 (23.4 to 25.2)	7.6 (7.2 to 8)	-68.7 (-70.7 to -66.8)
		Other bacterial pathogens	4420 (3300 to 5850)	3130 (2330 to 4120)	-29.0 (-34.2 to -23.7)	8.77 (7.78 to 9.83)	2.39 (2.07 to 2.72)	-72.8 (-75.2 to -70.4)
	15-49 years	*Acinetobacter baumannii*	2310 (1860 to 2770)	1120 (920 to 1350)	-51.4 (-56.0 to -46.6)	24.6 (21.1 to 28.5)	7.92 (6.88 to 9.04)	-67.8 (-70.9 to -64.4)
		Chlamydia spp.	14500 (12500 to 16800)	8430 (7240 to 9680)	-42.0 (-46.5 to -36.8)	38.3 (35.1 to 41.6)	15 (13.8 to 16.4)	-60.7 (-64.1 to -57.0)
		Enterobacter spp.	2030 (1680 to 2400)	2840 (2360 to 3370)	40.2 (26.6 to 54.7)	20.3 (17.8 to 23)	19.3 (17.3 to 21.5)	-4.5 (-13.6 to 5.3)
		Escherichia coli	4370 (3760 to 5020)	5330 (4630 to 6110)	22.0 (11.7 to 32.2)	45.4 (41.6 to 49.1)	37.9 (35.7 to 40.2)	-16.4 (-23.3 to -8.8)
		Fungus	5250 (4480 to 6100)	5400 (4650 to 6290)	2.9 (-6.7 to 13.0)	53.7 (48.2 to 59.2)	37.5 (34 to 41.2)	-30.1 (-36.6 to -23.3)
		Group B Streptococcus	6000 (5140 to 7040)	7160 (6150 to 8270)	19.5 (7.3 to 31.7)	44.1 (39.8 to 49.1)	35.8 (32.8 to 39.1)	-18.8 (-27.2 to -10.4)
		Haemophilus influenzae	26500 (23000 to 30000)	20500 (17900 to 23400)	-22.5 (-26.9 to -17.9)	98.9 (94.1 to 104)	37.1 (35.2 to 39.2)	-62.5 (-64.7 to -60.1)
		Influenza	36400 (31900 to 40900)	2880 (1850 to 4230)	-92.1 (-94.8 to -88.4)	281 (270 to 292)	13.9 (8.91 to 21.2)	-95.0 (-96.8 to -92.5)
		Klebsiella pneumoniae	12800 (11200 to 14400)	9560 (8350 to 10900)	-25.1 (-28.7 to -20.9)	132 (126 to 138)	66.3 (63.1 to 69.5)	-49.6 (-52.3 to -46.7)
		Legionella spp.	16800 (14400 to 19200)	18400 (16100 to 20900)	9.9 (0.2 to 20.3)	118 (109 to 128)	88 (83.6 to 92.7)	-25.5 (-32.5 to -18.3)
		Mycoplasma spp.	56500 (49400 to 64100)	44400 (38800 to 50500)	-21.4 (-26.5 to -16.6)	154 (146 to 163)	82.2 (78.3 to 86.1)	-46.7 (-50.3 to -43.2)
		Polymicrobial	594 (351 to 965)	577 (315 to 974)	-3.3 (-14.0 to 8.1)	6.26 (3.69 to 10.2)	4.14 (2.33 to 6.9)	-34.1 (-41.4 to -26.3)

		Pseudomonas aeruginosa	17400 (15000 to 19900)	23000 (20200 to 26300)	32.6 (24.4 to 41.5)	124 (116 to 132)	111 (106 to 116)	-10.4 (-16.1 to -4.8)
		Respiratory syncytial virus	13600 (11800 to 15500)	983 (636 to 1470)	-92.8 (-95.3 to -89.3)	47.8 (44.6 to 51.1)	2.12 (1.36 to 3.27)	-95.6 (-97.2 to -93.4)
		Staphylococcus aureus	23200 (20400 to 26200)	34600 (30600 to 39300)	49.1 (43.4 to 55.0)	339 (328 to 351)	344 (332 to 357)	1.5 (-2.5 to 5.5)
		Streptococcus pneumoniae	195000 (173000 to 219000)	77600 (68400 to 87700)	-60.2 (-61.6 to -58.8)	1190 (1160 to 1220)	313 (303 to 323)	-73.7 (-74.7 to -72.5)
		Other viruses	125000 (110000 to 140000)	100000 (88100 to 113000)	-19.9 (-22.5 to -17.0)	297 (289 to 306)	160 (154 to 165)	-46.3 (-48.6 to -44.1)
		Other bacterial pathogens	18200 (15200 to 21300)	12800 (10700 to 15200)	-30.0 (-34.9 to -24.3)	106 (94.2 to 119)	50.5 (44.4 to 57.1)	-52.4 (-56.1 to -48.8)
	50-69 years	*Acinetobacter baumannii*	10100 (8630 to 11700)	5560 (4760 to 6420)	-44.8 (-49.6 to -39.6)	155 (135 to 177)	78.2 (68.1 to 87.7)	-49.6 (-54.2 to -44.9)
		Chlamydia spp.	37700 (33600 to 42300)	23400 (20900 to 26000)	-38.0 (-42.7 to -32.2)	143 (131 to 156)	81.3 (74.8 to 88.3)	-43.2 (-47.9 to -37.8)
		Enterobacter spp.	8650 (7550 to 9860)	13300 (11700 to 14900)	54.0 (40.4 to 68.3)	125 (111 to 141)	175 (159 to 193)	40.6 (27.1 to 54.3)
		Escherichia coli	20600 (18400 to 22900)	26500 (24000 to 28900)	28.9 (18.9 to 39.4)	308 (284 to 332)	363 (343 to 382)	17.8 (8.5 to 27.1)
		Fungus	19600 (17400 to 22100)	22400 (19800 to 25200)	14.0 (4.0 to 24.7)	290 (263 to 322)	302 (275 to 331)	4.1 (-5.0 to 13.5)
		Group B Streptococcus	18100 (16000 to 20500)	23200 (20700 to 25800)	28.4 (16.3 to 40.8)	192 (175 to 212)	225 (206 to 244)	17.3 (6.0 to 29.2)
		Haemophilus influenzae	68100 (62300 to 74100)	62600 (56700 to 68700)	-8.1 (-13.2 to -2.7)	367 (351 to 384)	219 (208 to 231)	-40.3 (-43.7 to -36.7)
		Influenza	80600 (74100 to 87900)	6930 (4440 to 10400)	-91.4 (-94.4 to -87.3)	900 (864 to 938)	66.1 (42.9 to 98.1)	-92.7 (-95.3 to -89.1)
		Klebsiella pneumoniae	42100 (38700 to 45600)	39700 (36100 to 43300)	-5.6 (-11.0 to -0.3)	627 (599 to 653)	540 (512 to 567)	-13.9 (-18.8 to -9.3)
		Legionella spp.	60700 (54300 to 67400)	72600 (66100 to 79400)	19.9 (9.8 to 30.7)	619 (572 to 670)	677 (641 to 714)	9.5 (0.2 to 19.1)
		Mycoplasma spp.	66600 (60600 to 73200)	50600 (45600 to 55600)	-24.0 (-29.3 to -18.8)	263 (246 to 281)	183 (172 to 193)	-30.6 (-35.3 to -25.7)
		Polymicrobial	2170 (1310 to 3520)	2360 (1340 to 3960)	8.2 (-3.5 to 20.9)	33 (19.8 to 53.4)	32.8 (18.7 to 55.9)	-1.1 (-12.0 to 10.0)
		Pseudomonas aeruginosa	63400 (57600 to 69900)	87400 (79300 to 95500)	38.0 (28.9 to 47.8)	653 (617 to 692)	821 (781 to 863)	25.8 (17.5 to 34.4)
		Respiratory syncytial virus	22300 (20000 to 24700)	1580 (1010 to 2380)	-92.9 (-95.4 to -89.3)	113 (103 to 123)	6.82 (4.37 to 10.2)	-94.0 (-96.2 to -91.0)
		Staphylococcus aureus	74400 (68700 to 80900)	124000 (113000 to 135000)	66.1 (58.7 to 74.5)	1570 (1520 to 1630)	2380 (2280 to 2490)	51.6 (44.7 to 58.3)
		Streptococcus pneumoniae	309000 (286000 to 334000)	149000 (135000 to 162000)	-51.9 (-53.9 to -49.5)	2720 (2630 to 2800)	1190 (1140 to 1250)	-56.1 (-58.1 to -54.0)
		Other viruses	173000 (160000 to 188000)	174000 (159000 to 189000)	0.3 (-4.1 to 5.4)	597 (574 to 617)	546 (522 to 570)	-8.5 (-12.8 to -4.0)
		Other bacterial pathogens	70800 (61200 to 80600)	54000 (46200 to 61900)	-23.6 (-29.2 to -17.5)	596 (530 to 664)	417 (365 to 471)	-30.1 (-35.2 to -25.0)
	70+ years	*Acinetobacter baumannii*	18200 (15400 to 21300)	14700 (12700 to 17000)	-18.7 (-25.3 to -11.2)	1360 (1150 to 1580)	959 (749 to 1110)	-29.3 (-36.8 to -22.4)

		Chlamydia spp.	81300 (72400 to 90300)	74300 (67200 to 82900)	-8.6 (-16.3 to 0.2)	1510 (1310 to 1660)	1210 (989 to 1360)	-20.1 (-28.4 to -11.7)
		Enterobacter spp.	15300 (13300 to 17700)	34700 (30500 to 38800)	126.5 (105.0 to 148.6)	1090 (920 to 1240)	2150 (1700 to 2430)	97.8 (74.5 to 118.8)
		Escherichia coli	45900 (41200 to 51300)	87100 (79800 to 95800)	90.1 (74.1 to 106.0)	3360 (2940 to 3680)	5600 (4590 to 6190)	66.6 (48.4 to 81.6)
		Fungus	19900 (17500 to 22600)	33300 (29400 to 37700)	67.9 (52.4 to 84.9)	1440 (1250 to 1630)	2110 (1700 to 2410)	46.7 (30.3 to 61.6)
		Group B Streptococcus	30100 (26900 to 33700)	56900 (51300 to 63100)	88.9 (70.9 to 107.1)	1570 (1340 to 1750)	2590 (2100 to 2900)	65.0 (46.7 to 83.0)
		Haemophilus influenzae	112000 (103000 to 122000)	151000 (138000 to 166000)	35.2 (27.1 to 43.5)	2990 (2650 to 3190)	2490 (2050 to 2740)	-17.0 (-24.0 to -11.4)
		Influenza	223000 (205000 to 241000)	28700 (18700 to 42800)	-87.1 (-91.6 to -81.0)	12200 (10800 to 13000)	1340 (855 to 1990)	-89.0 (-93.0 to -83.7)
		Klebsiella pneumoniae	84100 (77700 to 91300)	117000 (107000 to 127000)	38.6 (31.2 to 46.4)	6130 (5420 to 6530)	7410 (6100 to 8140)	20.8 (10.2 to 28.5)
		Legionella spp.	79700 (71300 to 88600)	140000 (128000 to 153000)	75.6 (59.7 to 91.9)	4000 (3500 to 4410)	6110 (5010 to 6770)	52.9 (36.9 to 68.8)
		Mycoplasma spp.	103000 (93300 to 113000)	114000 (104000 to 126000)	11.4 (3.8 to 19.0)	1990 (1750 to 2150)	1930 (1580 to 2130)	-3.0 (-12.1 to 4.5)
		Polymicrobial	4490 (2670 to 7560)	7220 (3980 to 12400)	60.0 (42.2 to 78.8)	333 (196 to 553)	470 (259 to 812)	40.1 (22.3 to 56.7)
		Pseudomonas aeruginosa	120000 (110000 to 133000)	243000 (222000 to 264000)	101.9 (88.9 to 116.1)	6090 (5380 to 6560)	10700 (8670 to 11800)	75.5 (57.6 to 88.8)
		Respiratory syncytial virus	38300 (34200 to 42800)	4030 (2560 to 6010)	-89.5 (-93.2 to -84.1)	955 (835 to 1060)	85.3 (54.2 to 126)	-91.1 (-94.3 to -86.7)
		Staphylococcus aureus	175000 (162000 to 190000)	428000 (394000 to 465000)	144.3 (133.1 to 156.2)	18200 (16100 to 19200)	38800 (31500 to 42500)	113.4 (95.3 to 124.9)
		Streptococcus pneumoniae	578000 (535000 to 627000)	406000 (373000 to 442000)	-29.8 (-33.1 to -26.6)	24900 (22200 to 26400)	15200 (12500 to 16700)	-39.1 (-44.3 to -35.9)
		Other viruses	322000 (297000 to 347000)	471000 (432000 to 514000)	46.5 (39.0 to 53.8)	5450 (4860 to 5780)	6940 (5730 to 7610)	27.2 (16.6 to 34.1)
		Other bacterial pathogens	172000 (151000 to 197000)	193000 (167000 to 223000)	12.3 (4.3 to 21.5)	7160 (6080 to 8140)	7040 (5510 to 8260)	-1.8 (-11.3 to 6.7)
Andorra	All Ages	*Acinetobacter baumannii*	2.03 (1.71 to 2.38)	2.73 (2.34 to 3.14)	34.6 (18.9 to 52.7)	0.06 (0.04 to 0.08)	0.08 (0.05 to 0.1)	33.9 (-13.4 to 94.3)
		Chlamydia spp.	10.3 (9.26 to 11.5)	14.7 (13.3 to 16.4)	43.2 (27.6 to 61.2)	0.07 (0.05 to 0.09)	0.1 (0.07 to 0.14)	47.0 (-4.8 to 111.2)
		Enterobacter spp.	2.12 (1.83 to 2.43)	7.46 (6.65 to 8.44)	253.3 (211.1 to 297.0)	0.06 (0.04 to 0.08)	0.19 (0.13 to 0.26)	255.2 (129.3 to 411.0)
		Escherichia coli	5.39 (4.82 to 6.02)	16.8 (15.3 to 18.4)	212.5 (179.1 to 249.6)	0.16 (0.11 to 0.2)	0.48 (0.32 to 0.64)	212.7 (103.1 to 351.7)
		Fungus	3.66 (3.21 to 4.15)	9.22 (8.22 to 10.4)	152.4 (123.3 to 182.9)	0.08 (0.06 to 0.11)	0.2 (0.13 to 0.27)	150.0 (59.5 to 263.0)
		Group B Streptococcus	4.53 (4.07 to 5.09)	12.9 (11.8 to 14.3)	185.8 (150.3 to 221.1)	0.08 (0.06 to 0.11)	0.23 (0.16 to 0.31)	190.3 (85.3 to 313.0)
		Haemophilus influenzae	19.4 (17.8 to 21.2)	35.9 (32.9 to 39.3)	84.8 (69.4 to 102.5)	0.17 (0.13 to 0.23)	0.22 (0.15 to 0.29)	29.4 (-16.3 to 83.7)

		Influenza	28.3 (25.9 to 31)	5.25 (2.73 to 9.04)	-81.4 (-90.6 to -67.4)	0.58 (0.43 to 0.78)	0.11 (0.05 to 0.19)	-81.1 (-91.4 to -66.7)
		Klebsiella pneumoniae	11.4 (10.4 to 12.4)	23.5 (21.5 to 25.6)	107.2 (89.0 to 127.3)	0.3 (0.22 to 0.39)	0.63 (0.43 to 0.85)	116.3 (40.3 to 207.2)
		Legionella spp.	13.8 (12.4 to 15.3)	35.3 (32.3 to 38.8)	155.9 (128.7 to 186.1)	0.23 (0.17 to 0.31)	0.58 (0.39 to 0.77)	155.8 (63.3 to 271.2)
		Mycoplasma spp.	22.3 (20.3 to 24.4)	33.5 (30.9 to 36.5)	50.8 (38.4 to 64.8)	0.12 (0.09 to 0.16)	0.18 (0.12 to 0.24)	55.5 (0.0 to 124.0)
		Polymicrobial	0.55 (0.34 to 0.89)	1.42 (0.78 to 2.42)	154.6 (118.2 to 194.6)	0.02 (0.01 to 0.03)	0.04 (0.02 to 0.07)	150.3 (57.2 to 265.1)
		Pseudomonas aeruginosa	17.2 (15.6 to 19.1)	52.4 (48 to 57.7)	205.0 (176.1 to 237.1)	0.32 (0.23 to 0.42)	0.96 (0.65 to 1.3)	207.1 (98.6 to 339.2)
		Respiratory syncytial virus	13.1 (11.9 to 14.6)	1.31 (0.67 to 2.21)	-90.0 (-94.8 to -82.8)	0.08 (0.06 to 0.1)	0.01 (0 to 0.01)	-90.1 (-95.6 to -82.2)
		Staphylococcus aureus	22.1 (20.2 to 24.1)	85.1 (78.1 to 93.3)	286.3 (252.5 to 323.3)	0.88 (0.64 to 1.17)	3.41 (2.3 to 4.56)	293.8 (154.6 to 463.1)
		Streptococcus pneumoniae	104 (96.1 to 113)	91.5 (84.4 to 100)	-11.8 (-18.5 to -4.3)	1.36 (1.01 to 1.8)	1.3 (0.88 to 1.73)	-2.9 (-37.2 to 38.6)
		Other viruses	70.5 (65.3 to 77)	120 (111 to 130)	69.8 (56.8 to 83.8)	0.34 (0.25 to 0.44)	0.64 (0.43 to 0.85)	91.7 (23.9 to 172.5)
		Other bacterial pathogens	19.3 (16.5 to 22.3)	35.5 (30.8 to 41.1)	84.6 (64.9 to 106.1)	0.32 (0.23 to 0.43)	0.59 (0.39 to 0.81)	85.1 (20.9 to 163.6)
	Under 5	*Acinetobacter baumannii*	0.03 (0.02 to 0.05)	0.01 (0.01 to 0.02)	-62.7 (-67.4 to -57.5)	0 (0 to 0)	0 (0 to 0)	-94.2 (-98.2 to -88.1)
		Chlamydia spp.	0.48 (0.4 to 0.58)	0.21 (0.17 to 0.26)	-56.3 (-62.8 to -49.2)	0 (0 to 0)	0 (0 to 0)	-94.2 (-97.3 to -89.9)
		Enterobacter spp.	0.08 (0.06 to 0.11)	0.08 (0.06 to 0.11)	-2.5 (-17.9 to 16.2)	0 (0 to 0)	0 (0 to 0)	-88.3 (-94.6 to -79.6)
		Escherichia coli	0.23 (0.19 to 0.28)	0.23 (0.18 to 0.29)	2.5 (-13.4 to 19.3)	0 (0 to 0)	0 (0 to 0)	-87.8 (-94.2 to -78.9)
		Fungus	0.04 (0.03 to 0.06)	0.03 (0.02 to 0.05)	-22.2 (-36.4 to -7.0)	0 (0 to 0)	0 (0 to 0)	-89.7 (-95.0 to -81.9)
		Group B Streptococcus	0.22 (0.18 to 0.27)	0.19 (0.15 to 0.23)	-14.5 (-28.0 to 0.2)	0 (0 to 0)	0 (0 to 0)	-89.9 (-95.3 to -82.4)
		Haemophilus influenzae	2.18 (1.8 to 2.64)	1.12 (0.9 to 1.39)	-48.4 (-55.4 to -40.5)	0.01 (0.01 to 0.01)	0 (0 to 0)	-95.8 (-97.9 to -92.7)
		Influenza	1.73 (1.43 to 2.08)	0.07 (0.04 to 0.12)	-95.8 (-97.8 to -93.0)	0.01 (0.01 to 0.02)	0 (0 to 0)	-99.5 (-99.8 to -98.9)
		Klebsiella pneumoniae	0.84 (0.69 to 1.01)	0.36 (0.29 to 0.44)	-56.9 (-62.4 to -50.5)	0.01 (0.01 to 0.01)	0 (0 to 0)	-94.2 (-97.3 to -89.9)
		Legionella spp.	0.13 (0.09 to 0.18)	0.1 (0.07 to 0.14)	-24.4 (-35.2 to -12.2)	0 (0 to 0)	0 (0 to 0)	-90.5 (-96.0 to -82.6)
		Mycoplasma spp.	1.18 (0.96 to 1.43)	1.06 (0.85 to 1.31)	-9.7 (-22.0 to 3.6)	0 (0 to 0.01)	0 (0 to 0)	-88.4 (-94.2 to -80.0)
		Polymicrobial	0.04 (0.02 to 0.07)	0.02 (0.01 to 0.04)	-44.3 (-51.9 to -35.6)	0 (0 to 0)	0 (0 to 0)	-91.9 (-97.7 to -83.0)
		Pseudomonas aeruginosa	0.75 (0.62 to 0.92)	0.9 (0.72 to 1.1)	19.2 (3.1 to 37.0)	0.01 (0 to 0.01)	0 (0 to 0)	-85.4 (-93.1 to -74.6)

		Respiratory syncytial virus	6.19 (5.23 to 7.35)	0.33 (0.17 to 0.57)	-94.7 (-97.2 to -91.0)	0.03 (0.02 to 0.04)	0 (0 to 0)	-99.3 (-99.8 to -98.6)
		Staphylococcus aureus	0.78 (0.65 to 0.94)	0.82 (0.66 to 1.01)	5.3 (-5.6 to 18.5)	0.01 (0.01 to 0.02)	0 (0 to 0)	-86.5 (-93.4 to -76.5)
		Streptococcus pneumoniae	10.8 (9.05 to 12.9)	1.84 (1.47 to 2.26)	-83.0 (-84.7 to -81.0)	0.07 (0.05 to 0.11)	0 (0 to 0)	-97.8 (-98.9 to -96.2)
		Other viruses	12.2 (10.2 to 14.5)	5.05 (4.06 to 6.2)	-58.7 (-62.5 to -54.3)	0.03 (0.02 to 0.05)	0 (0 to 0)	-94.5 (-97.3 to -90.5)
		Other bacterial pathogens	0.24 (0.19 to 0.31)	0.14 (0.1 to 0.18)	-44.1 (-52.8 to -34.5)	0 (0 to 0)	0 (0 to 0)	-92.7 (-96.5 to -87.2)
	5-14 years	*Acinetobacter baumannii*	0.05 (0.04 to 0.07)	0.03 (0.02 to 0.04)	-37.3 (-44.3 to -29.0)	0 (0 to 0)	0 (0 to 0)	-79.6 (-87.6 to -69.1)
		Chlamydia spp.	0.36 (0.26 to 0.48)	0.26 (0.19 to 0.34)	-28.3 (-36.2 to -19.4)	0 (0 to 0)	0 (0 to 0)	-76.7 (-86.2 to -65.1)
		Enterobacter spp.	0.05 (0.04 to 0.08)	0.09 (0.07 to 0.13)	71.5 (51.7 to 92.8)	0 (0 to 0)	0 (0 to 0)	-44.4 (-65.9 to -16.5)
		Escherichia coli	0.11 (0.08 to 0.15)	0.17 (0.12 to 0.22)	50.7 (35.1 to 68.2)	0 (0 to 0)	0 (0 to 0)	-51.1 (-70.4 to -26.2)
		Fungus	0.14 (0.1 to 0.18)	0.17 (0.13 to 0.24)	28.3 (13.0 to 44.4)	0 (0 to 0)	0 (0 to 0)	-58.4 (-75.1 to -37.5)
		Group B Streptococcus	0.16 (0.12 to 0.21)	0.23 (0.17 to 0.31)	45.8 (28.6 to 64.9)	0 (0 to 0)	0 (0 to 0)	-52.7 (-70.9 to -28.2)
		Haemophilus influenzae	0.72 (0.54 to 0.95)	0.69 (0.51 to 0.91)	-3.8 (-11.9 to 5.7)	0 (0 to 0)	0 (0 to 0)	-79.3 (-87.4 to -69.0)
		Influenza	0.97 (0.72 to 1.27)	0.09 (0.04 to 0.16)	-91.1 (-95.3 to -84.5)	0 (0 to 0)	0 (0 to 0)	-97.2 (-98.8 to -94.6)
		Klebsiella pneumoniae	0.33 (0.24 to 0.43)	0.32 (0.23 to 0.42)	-3.2 (-11.4 to 6.0)	0 (0 to 0)	0 (0 to 0)	-68.6 (-80.7 to -52.9)
		Legionella spp.	0.48 (0.35 to 0.64)	0.61 (0.46 to 0.8)	27.5 (11.8 to 42.3)	0 (0 to 0)	0 (0 to 0)	-58.6 (-74.7 to -38.3)
		Mycoplasma spp.	1.52 (1.11 to 1.99)	1.35 (1.01 to 1.79)	-10.6 (-19.6 to -1.7)	0 (0 to 0)	0 (0 to 0)	-71.0 (-82.3 to -56.4)
		Polymicrobial	0.01 (0.01 to 0.02)	0.02 (0.01 to 0.03)	25.5 (10.8 to 43.8)	0 (0 to 0)	0 (0 to 0)	-59.3 (-75.2 to -38.1)
		Pseudomonas aeruginosa	0.49 (0.36 to 0.64)	0.74 (0.55 to 0.98)	53.2 (39.0 to 70.4)	0 (0 to 0)	0 (0 to 0)	-50.3 (-69.6 to -25.3)
		Respiratory syncytial virus	0.39 (0.29 to 0.52)	0.03 (0.01 to 0.05)	-92.6 (-96.1 to -87.0)	0 (0 to 0)	0 (0 to 0)	-97.7 (-99.0 to -95.5)
		Staphylococcus aureus	0.63 (0.47 to 0.82)	1.15 (0.85 to 1.5)	83.1 (68.0 to 98.7)	0 (0 to 0)	0 (0 to 0)	-40.6 (-63.6 to -11.4)
		Streptococcus pneumoniae	5.18 (3.86 to 6.78)	2.42 (1.8 to 3.17)	-53.3 (-57.1 to -49.4)	0.01 (0.01 to 0.01)	0 (0 to 0)	-84.9 (-90.7 to -77.4)
		Other viruses	3.56 (2.67 to 4.67)	3.43 (2.56 to 4.52)	-3.7 (-11.3 to 4.7)	0 (0 to 0)	0 (0 to 0)	-68.8 (-81.0 to -53.2)
		Other bacterial pathogens	0.47 (0.34 to 0.63)	0.4 (0.3 to 0.54)	-14.2 (-22.4 to -4.5)	0 (0 to 0)	0 (0 to 0)	-72.2 (-82.9 to -57.9)
	15-49 years	*Acinetobacter baumannii*	0.25 (0.2 to 0.32)	0.2 (0.16 to 0.25)	-18.8 (-28.7 to -8.5)	0 (0 to 0)	0 (0 to 0)	-51.5 (-74.0 to -23.1)

		Chlamydia spp.	1.74 (1.47 to 2.09)	1.61 (1.35 to 1.9)	-7.2 (-17.0 to 3.8)	0 (0 to 0)	0 (0 to 0)	-44.6 (-70.0 to -13.3)
		Enterobacter spp.	0.27 (0.21 to 0.32)	0.59 (0.48 to 0.72)	121.9 (95.6 to 150.1)	0 (0 to 0)	0 (0 to 0)	32.5 (-27.1 to 108.6)
		Escherichia coli	0.55 (0.46 to 0.66)	1.07 (0.91 to 1.26)	94.9 (74.6 to 115.8)	0 (0 to 0.01)	0 (0 to 0.01)	16.5 (-35.1 to 86.1)
		Fungus	0.66 (0.55 to 0.79)	1.09 (0.92 to 1.29)	66.0 (47.5 to 86.6)	0.01 (0 to 0.01)	0 (0 to 0.01)	-0.9 (-45.6 to 54.5)
		Group B Streptococcus	0.77 (0.64 to 0.93)	1.45 (1.22 to 1.71)	88.6 (65.9 to 110.9)	0 (0 to 0.01)	0 (0 to 0.01)	12.7 (-38.1 to 74.8)
		Haemophilus influenzae	3.5 (2.98 to 4.12)	4.35 (3.69 to 5.07)	24.4 (13.7 to 36.0)	0.01 (0.01 to 0.01)	0.01 (0 to 0.01)	-50.7 (-73.1 to -22.8)
		Influenza	4.72 (4.05 to 5.58)	0.54 (0.28 to 0.92)	-88.4 (-94.0 to -79.7)	0.03 (0.02 to 0.04)	0 (0 to 0)	-93.3 (-97.3 to -87.2)
		Klebsiella pneumoniae	1.6 (1.38 to 1.9)	2.01 (1.7 to 2.34)	25.3 (14.7 to 35.9)	0.01 (0.01 to 0.02)	0.01 (0.01 to 0.01)	-25.1 (-58.4 to 17.4)
		Legionella spp.	2.34 (1.98 to 2.77)	3.85 (3.27 to 4.47)	65.0 (47.2 to 84.0)	0.01 (0.01 to 0.02)	0.01 (0.01 to 0.02)	-1.4 (-45.4 to 54.1)
		Mycoplasma spp.	7.4 (6.29 to 8.68)	8.54 (7.25 to 9.94)	15.6 (4.8 to 26.6)	0.02 (0.01 to 0.02)	0.01 (0.01 to 0.01)	-30.9 (-62.5 to 7.6)
		Polymicrobial	0.07 (0.04 to 0.12)	0.12 (0.06 to 0.2)	62.4 (41.0 to 86.1)	0 (0 to 0)	0 (0 to 0)	-2.9 (-47.1 to 56.2)
		Pseudomonas aeruginosa	2.38 (2.03 to 2.83)	4.71 (4.01 to 5.52)	98.2 (80.1 to 117.2)	0.01 (0.01 to 0.02)	0.01 (0.01 to 0.02)	18.4 (-35.4 to 83.7)
		Respiratory syncytial virus	1.91 (1.61 to 2.27)	0.18 (0.09 to 0.31)	-90.4 (-95.0 to -83.2)	0.01 (0 to 0.01)	0 (0 to 0)	-94.4 (-97.7 to -89.3)
		Staphylococcus aureus	3.06 (2.62 to 3.61)	7.24 (6.2 to 8.42)	136.9 (117.8 to 157.0)	0.03 (0.02 to 0.05)	0.05 (0.03 to 0.07)	41.5 (-21.6 to 120.3)
		Streptococcus pneumoniae	25.3 (21.8 to 29.9)	15.3 (13 to 17.8)	-39.6 (-44.3 to -34.7)	0.12 (0.08 to 0.16)	0.04 (0.03 to 0.06)	-63.9 (-80.0 to -43.6)
		Other viruses	17.4 (15 to 20.5)	21.7 (18.5 to 25.1)	24.6 (14.3 to 34.7)	0.03 (0.02 to 0.04)	0.02 (0.01 to 0.03)	-25.5 (-58.6 to 15.6)
		Other bacterial pathogens	2.29 (1.86 to 2.76)	2.53 (2.09 to 3.04)	11.0 (0.1 to 22.3)	0.01 (0.01 to 0.01)	0.01 (0 to 0.01)	-33.6 (-63.9 to 3.6)
	50-69 years	*Acinetobacter baumannii*	0.72 (0.6 to 0.86)	0.86 (0.72 to 1.01)	18.9 (4.5 to 36.1)	0.01 (0.01 to 0.01)	0.01 (0 to 0.01)	-32.1 (-64.7 to 11.5)
		Chlamydia spp.	2.95 (2.53 to 3.39)	3.87 (3.37 to 4.45)	31.5 (16.8 to 49.7)	0.01 (0.01 to 0.01)	0.01 (0 to 0.01)	-25.0 (-61.2 to 22.5)
		Enterobacter spp.	0.74 (0.62 to 0.88)	2.35 (2 to 2.73)	219.2 (179.3 to 268.0)	0.01 (0.01 to 0.01)	0.02 (0.01 to 0.02)	82.2 (-5.8 to 195.5)
		Escherichia coli	1.69 (1.46 to 1.93)	4.6 (4.06 to 5.17)	173.1 (142.3 to 211.2)	0.02 (0.01 to 0.03)	0.03 (0.02 to 0.05)	56.0 (-20.1 to 157.5)
		Fungus	1.61 (1.38 to 1.87)	3.85 (3.31 to 4.45)	139.8 (110.9 to 173.6)	0.02 (0.01 to 0.03)	0.03 (0.02 to 0.04)	36.9 (-28.4 to 121.5)
		Group B Streptococcus	1.51 (1.3 to 1.75)	4.03 (3.51 to 4.62)	167.7 (133.2 to 206.8)	0.01 (0.01 to 0.02)	0.02 (0.01 to 0.03)	52.8 (-22.4 to 147.8)
		Haemophilus influenzae	5.87 (5.11 to 6.57)	11 (9.59 to 12.3)	86.9 (69.1 to 110.0)	0.03 (0.02 to 0.04)	0.02 (0.01 to 0.03)	-29.3 (-62.8 to 15.2)
		Influenza	6.82 (5.96 to 7.66)	1.17 (0.6 to 2.01)	-82.8 (-91.1 to -70.3)	0.07 (0.04 to 0.1)	0.01 (0 to 0.01)	-90.5 (-96.3 to -81.3)

		Klebsiella pneumoniae	3.45 (3.01 to 3.86)	6.73 (5.94 to 7.55)	95.6 (77.4 to 118.5)	0.04 (0.03 to 0.07)	0.05 (0.03 to 0.07)	11.7 (-41.3 to 84.3)
		Legionella spp.	5.52 (4.81 to 6.3)	13 (11.5 to 14.7)	136.4 (110.8 to 169.4)	0.05 (0.03 to 0.07)	0.06 (0.04 to 0.1)	35.0 (-30.3 to 121.7)
		Mycoplasma spp.	5.68 (4.94 to 6.44)	8.67 (7.59 to 9.78)	52.9 (36.4 to 72.3)	0.02 (0.01 to 0.03)	0.02 (0.01 to 0.02)	-12.7 (-54.9 to 42.1)
		Polymicrobial	0.17 (0.1 to 0.28)	0.4 (0.22 to 0.69)	136.1 (104.4 to 172.6)	0 (0 to 0)	0 (0 to 0.01)	34.8 (-31.2 to 124.6)
		Pseudomonas aeruginosa	5.65 (4.91 to 6.41)	15.5 (13.6 to 17.4)	174.7 (146.6 to 208.6)	0.05 (0.03 to 0.07)	0.08 (0.05 to 0.11)	56.7 (-18.7 to 155.7)
		Respiratory syncytial virus	2.04 (1.75 to 2.35)	0.27 (0.14 to 0.47)	-86.5 (-93.1 to -77.2)	0.01 (0.01 to 0.01)	0 (0 to 0)	-92.6 (-97.1 to -85.4)
		Staphylococcus aureus	6.4 (5.6 to 7.16)	22 (19.4 to 24.6)	243.9 (212.5 to 283.5)	0.12 (0.08 to 0.17)	0.22 (0.14 to 0.33)	96.3 (2.3 to 221.8)
		Streptococcus pneumoniae	26.1 (23.1 to 29.1)	24.4 (21.4 to 27.3)	-6.5 (-15.0 to 4.2)	0.2 (0.14 to 0.3)	0.1 (0.06 to 0.15)	-46.7 (-72.2 to -13.2)
		Other viruses	15.7 (13.8 to 17.5)	30.6 (26.9 to 34.3)	94.7 (76.9 to 118.4)	0.05 (0.03 to 0.07)	0.05 (0.03 to 0.08)	11.1 (-41.4 to 81.4)
		Other bacterial pathogens	5.78 (4.88 to 6.79)	9.16 (7.64 to 10.8)	58.6 (41.2 to 78.9)	0.04 (0.03 to 0.06)	0.04 (0.02 to 0.06)	-9.4 (-52.8 to 47.7)
	70+ years	*Acinetobacter baumannii*	0.98 (0.8 to 1.18)	1.62 (1.35 to 1.93)	67.0 (43.0 to 93.0)	0.04 (0.03 to 0.06)	0.07 (0.05 to 0.09)	54.6 (0.6 to 121.1)
		Chlamydia spp.	4.75 (4.11 to 5.51)	8.75 (7.69 to 10.1)	84.6 (59.3 to 113.8)	0.05 (0.04 to 0.07)	0.09 (0.06 to 0.12)	70.9 (11.9 to 145.7)
		Enterobacter spp.	0.97 (0.82 to 1.16)	4.34 (3.75 to 5.04)	348.2 (283.7 to 417.9)	0.04 (0.03 to 0.06)	0.17 (0.11 to 0.23)	314.8 (169.6 to 491.8)
		Escherichia coli	2.81 (2.43 to 3.24)	10.7 (9.54 to 12.1)	283.5 (232.1 to 340.8)	0.13 (0.09 to 0.17)	0.44 (0.3 to 0.59)	255.2 (134.0 to 408.3)
		Fungus	1.21 (1.03 to 1.42)	4.07 (3.52 to 4.71)	236.7 (190.8 to 289.7)	0.05 (0.04 to 0.07)	0.16 (0.11 to 0.22)	211.7 (105.4 to 348.4)
		Group B Streptococcus	1.88 (1.61 to 2.18)	7.03 (6.18 to 8.05)	275.9 (222.1 to 340.0)	0.06 (0.04 to 0.08)	0.2 (0.14 to 0.28)	247.9 (126.1 to 393.3)
		Haemophilus influenzae	7.16 (6.27 to 8.15)	18.7 (16.6 to 21.2)	162.5 (130.3 to 199.3)	0.12 (0.09 to 0.16)	0.2 (0.13 to 0.26)	61.0 (6.4 to 125.2)
		Influenza	14 (12.2 to 15.9)	3.38 (1.7 to 5.82)	-75.9 (-87.8 to -58.3)	0.47 (0.34 to 0.63)	0.1 (0.05 to 0.18)	-78.4 (-90.1 to -61.9)
		Klebsiella pneumoniae	5.14 (4.48 to 5.79)	14.1 (12.6 to 15.8)	174.7 (142.7 to 210.6)	0.23 (0.17 to 0.31)	0.58 (0.38 to 0.77)	154.3 (66.2 to 261.9)
		Legionella spp.	5.37 (4.66 to 6.18)	17.8 (15.7 to 20)	231.9 (185.8 to 282.4)	0.16 (0.12 to 0.22)	0.5 (0.33 to 0.66)	207.3 (104.0 to 341.3)
		Mycoplasma spp.	6.5 (5.6 to 7.41)	13.9 (12.4 to 15.7)	114.7 (88.8 to 146.4)	0.08 (0.06 to 0.1)	0.15 (0.1 to 0.2)	98.7 (31.3 to 185.3)
		Polymicrobial	0.26 (0.15 to 0.42)	0.87 (0.48 to 1.49)	231.5 (181.3 to 289.0)	0.01 (0.01 to 0.02)	0.04 (0.02 to 0.07)	206.8 (100.4 to 342.7)
		Pseudomonas aeruginosa	7.96 (6.93 to 9.13)	30.6 (27.2 to 34.8)	285.7 (238.1 to 341.0)	0.25 (0.18 to 0.33)	0.87 (0.58 to 1.17)	257.0 (137.0 to 406.4)
		Respiratory syncytial virus	2.59 (2.23 to 3)	0.49 (0.25 to 0.85)	-81.1 (-90.5 to -67.0)	0.04 (0.03 to 0.05)	0.01 (0 to 0.01)	-83.1 (-92.4 to -69.5)

		Staphylococcus aureus	11.2 (9.8 to 12.7)	54 (48.1 to 60.6)	382.8 (325.8 to 444.1)	0.71 (0.52 to 0.94)	3.13 (2.11 to 4.17)	346.9 (193.8 to 535.6)
		Streptococcus pneumoniae	36.4 (31.9 to 41)	47.6 (42.5 to 53.5)	31.2 (15.8 to 48.6)	0.96 (0.69 to 1.27)	1.15 (0.78 to 1.53)	21.5 (-19.6 to 72.2)
		Other viruses	21.6 (19 to 24.4)	59 (52.8 to 66.4)	173.4 (140.3 to 210.1)	0.22 (0.16 to 0.3)	0.56 (0.37 to 0.75)	153.1 (66.0 to 261.4)
		Other bacterial pathogens	10.5 (8.7 to 12.4)	23.3 (19.7 to 27.5)	122.7 (93.8 to 155.8)	0.27 (0.19 to 0.36)	0.54 (0.36 to 0.75)	106.3 (35.8 to 191.4)
Austria	All Ages	*Acinetobacter baumannii*	536 (463 to 618)	359 (309 to 414)	-32.9 (-39.9 to -25.8)	16.6 (14 to 19.1)	12.2 (9.69 to 14.3)	-26.4 (-35.1 to -18.2)
		Chlamydia spp.	2520 (2290 to 2770)	1520 (1380 to 1660)	-39.4 (-45.2 to -33.8)	18.8 (16.5 to 20.7)	12.5 (10.3 to 13.9)	-33.6 (-41.2 to -26.2)
		Enterobacter spp.	486 (425 to 553)	633 (559 to 710)	30.6 (16.8 to 44.1)	13.8 (11.9 to 15.8)	19.9 (16.1 to 22.7)	43.6 (25.7 to 61.6)
		Escherichia coli	1300 (1170 to 1430)	1400 (1270 to 1520)	7.9 (-2.9 to 19.0)	41.3 (36.5 to 45.4)	48.2 (40 to 53.7)	16.9 (2.8 to 30.5)
		Fungus	844 (753 to 947)	821 (736 to 912)	-2.5 (-12.4 to 7.3)	20.1 (17.6 to 22.5)	21.9 (18.3 to 24.8)	8.9 (-4.1 to 20.9)
		Group B Streptococcus	1040 (939 to 1160)	1160 (1060 to 1270)	11.8 (-0.2 to 23.4)	20.4 (18 to 22.6)	25.1 (20.9 to 28)	23.4 (9.6 to 37.7)
		Haemophilus influenzae	4300 (4020 to 4620)	3180 (2920 to 3440)	-26.0 (-31.7 to -20.3)	41.7 (37.7 to 44.6)	24.4 (20.6 to 27)	-41.4 (-47.4 to -35.7)
		Influenza	6500 (6070 to 6970)	419 (215 to 719)	-93.6 (-96.6 to -88.9)	149 (134 to 160)	10.2 (5.28 to 17.3)	-93.1 (-96.5 to -88.2)
		Klebsiella pneumoniae	2670 (2500 to 2870)	2410 (2210 to 2610)	-9.6 (-16.9 to -2.7)	77.1 (69.1 to 82.9)	78.7 (65.4 to 87.2)	2.0 (-8.5 to 11.2)
		Legionella spp.	2980 (2700 to 3280)	3140 (2860 to 3410)	5.4 (-5.3 to 16.7)	54.2 (48.1 to 59.9)	63.3 (53.1 to 69.9)	16.8 (3.3 to 30.3)
		Mycoplasma spp.	4780 (4420 to 5180)	3490 (3220 to 3750)	-27.0 (-33.2 to -20.9)	27.7 (24.9 to 30.1)	21.8 (18.1 to 24.1)	-21.4 (-29.7 to -13.3)
		Polymicrobial	136 (83.9 to 223)	129 (73.3 to 217)	-6.3 (-17.0 to 4.7)	4.2 (2.54 to 7.07)	4.35 (2.43 to 7.49)	3.0 (-11.0 to 16.2)
		Pseudomonas aeruginosa	3810 (3520 to 4170)	5030 (4600 to 5450)	32.1 (20.3 to 43.4)	77.7 (69.5 to 84.4)	112 (93.2 to 123)	43.9 (27.0 to 58.9)
		Respiratory syncytial virus	2580 (2380 to 2800)	159 (82.8 to 269)	-93.8 (-96.8 to -89.6)	14.7 (13.2 to 16)	0.93 (0.48 to 1.56)	-93.6 (-96.7 to -89.3)
		Staphylococcus aureus	5070 (4730 to 5450)	7160 (6570 to 7760)	41.3 (30.0 to 51.6)	224 (201 to 240)	346 (287 to 382)	54.4 (39.0 to 68.7)
		Streptococcus pneumoniae	23000 (21600 to 24500)	12000 (11100 to 12800)	-47.9 (-51.7 to -44.4)	331 (299 to 353)	200 (169 to 221)	-39.5 (-45.5 to -33.9)
		Other viruses	14500 (13600 to 15400)	12800 (11800 to 13700)	-11.9 (-18.3 to -6.4)	74.8 (67.8 to 79.9)	78.8 (66.2 to 86.8)	5.3 (-5.3 to 14.9)
		Other bacterial pathogens	4680 (4130 to 5280)	3500 (3010 to 4030)	-25.1 (-32.2 to -18.4)	87 (75.1 to 98.5)	70.6 (56.8 to 83.1)	-18.9 (-27.4 to -10.3)
	Under 5	*Acinetobacter baumannii*	6.63 (4.39 to 9.76)	2.6 (1.74 to 3.86)	-60.7 (-65.6 to -55.2)	0.08 (0.05 to 0.14)	0.02 (0.01 to 0.04)	-75.3 (-80.4 to -69.6)
		Chlamydia spp.	99.6 (83.4 to 118)	42.7 (34.5 to 52.5)	-57.1 (-63.4 to -50.5)	0.15 (0.13 to 0.17)	0.03 (0.02 to 0.03)	-82.4 (-85.7 to -79.0)
		Enterobacter spp.	16.2 (12.3 to 21)	13.9 (10.3 to 18.8)	-14.0 (-27.8 to 2.0)	0.1 (0.08 to 0.14)	0.03 (0.02 to 0.04)	-69.4 (-75.4 to -62.6)

		Escherichia coli	45.6 (38.1 to 55.1)	37.4 (30.1 to 46.1)	-17.9 (-30.6 to -3.2)	0.28 (0.24 to 0.32)	0.08 (0.06 to 0.09)	-72.2 (-77.2 to -66.6)
		Fungus	8.72 (6.1 to 12.2)	6.2 (4.35 to 8.78)	-28.7 (-41.1 to -15.3)	0.04 (0.03 to 0.05)	0.01 (0.01 to 0.01)	-79.3 (-83.8 to -74.0)
		Group B Streptococcus	43.1 (35.7 to 52.3)	33.1 (26.5 to 41.4)	-23.0 (-35.1 to -10.4)	0.22 (0.19 to 0.25)	0.06 (0.05 to 0.07)	-71.8 (-76.9 to -66.2)
		Haemophilus influenzae	419 (354 to 494)	197 (158 to 243)	-53.0 (-59.4 to -45.3)	0.78 (0.7 to 0.88)	0.07 (0.06 to 0.08)	-91.1 (-92.8 to -89.3)
		Influenza	334 (284 to 392)	11.9 (5.95 to 20.4)	-96.4 (-98.2 to -94.0)	1.19 (1.06 to 1.31)	0.01 (0.01 to 0.02)	-99.0 (-99.5 to -98.2)
		Klebsiella pneumoniae	166 (139 to 197)	76.6 (61.4 to 94.1)	-53.8 (-59.9 to -46.7)	0.82 (0.73 to 0.91)	0.13 (0.11 to 0.15)	-84.1 (-86.9 to -81.0)
		Legionella spp.	24.3 (17.2 to 33.4)	17.6 (12 to 25.1)	-27.5 (-38.0 to -16.3)	0.12 (0.1 to 0.16)	0.04 (0.03 to 0.05)	-69.4 (-75.4 to -63.1)
		Mycoplasma spp.	227 (191 to 272)	193 (155 to 240)	-15.1 (-27.6 to -1.6)	0.29 (0.25 to 0.33)	0.07 (0.06 to 0.09)	-74.7 (-79.4 to -69.0)
		Polymicrobial	8.11 (4.56 to 12.9)	4.15 (2.31 to 6.85)	-48.8 (-55.8 to -41.7)	0.13 (0.07 to 0.21)	0.04 (0.02 to 0.08)	-64.8 (-72.0 to -56.7)
		Pseudomonas aeruginosa	140 (117 to 169)	160 (129 to 197)	14.1 (-2.3 to 31.7)	0.53 (0.47 to 0.59)	0.18 (0.16 to 0.22)	-65.0 (-71.4 to -58.5)
		Respiratory syncytial virus	1130 (974 to 1330)	62.6 (31.8 to 108)	-94.5 (-97.1 to -90.6)	1.95 (1.77 to 2.15)	0.03 (0.02 to 0.05)	-98.4 (-99.2 to -97.1)
		Staphylococcus aureus	149 (127 to 175)	138 (112 to 170)	-7.6 (-18.5 to 4.2)	1.03 (0.93 to 1.14)	0.29 (0.24 to 0.34)	-71.5 (-76.5 to -66.3)
		Streptococcus pneumoniae	2100 (1810 to 2480)	524 (422 to 644)	-75.1 (-78.1 to -71.9)	5.56 (4.99 to 6.11)	0.42 (0.34 to 0.49)	-92.5 (-93.8 to -91.1)
		Other viruses	2250 (1920 to 2640)	1090 (886 to 1350)	-51.3 (-56.9 to -44.9)	2.37 (2.14 to 2.62)	0.36 (0.3 to 0.43)	-84.6 (-87.3 to -81.5)
		Other bacterial pathogens	48.6 (37.6 to 61.6)	26.1 (19.7 to 34.1)	-46.2 (-54.8 to -37.3)	0.15 (0.12 to 0.19)	0.03 (0.02 to 0.04)	-81.0 (-84.6 to -76.4)
	5-14 years	*Acinetobacter baumannii*	12.1 (9.03 to 16.2)	6.41 (4.71 to 8.53)	-47.2 (-54.9 to -39.6)	0.02 (0.01 to 0.02)	0 (0 to 0)	-76.1 (-80.1 to -72.2)
		Chlamydia spp.	79 (61.1 to 102)	39.7 (30 to 51.9)	-49.7 (-56.8 to -42.9)	0.03 (0.02 to 0.03)	0.01 (0 to 0.01)	-77.3 (-80.8 to -73.6)
		Enterobacter spp.	11.2 (8.46 to 14.7)	11.9 (8.6 to 15.9)	5.8 (-10.2 to 21.4)	0.01 (0.01 to 0.02)	0.01 (0.01 to 0.01)	-52.1 (-59.9 to -43.3)
		Escherichia coli	24 (18.6 to 31.3)	20.9 (15.6 to 27.3)	-13.0 (-26.6 to -0.9)	0.03 (0.03 to 0.03)	0.01 (0.01 to 0.01)	-60.6 (-66.8 to -54.3)
		Fungus	28.8 (22.3 to 37.2)	23.6 (17.7 to 31.2)	-17.8 (-29.0 to -6.5)	0.04 (0.03 to 0.04)	0.01 (0.01 to 0.02)	-62.8 (-68.6 to -56.5)
		Group B Streptococcus	33 (25.6 to 42.4)	30.8 (23 to 40.3)	-6.5 (-20.2 to 6.9)	0.03 (0.03 to 0.03)	0.01 (0.01 to 0.01)	-57.7 (-65.0 to -50.6)
		Haemophilus influenzae	148 (116 to 191)	87.8 (66 to 115)	-40.5 (-49.2 to -32.8)	0.07 (0.06 to 0.08)	0.01 (0.01 to 0.02)	-81.3 (-83.9 to -78.5)
		Influenza	200 (158 to 258)	10.2 (4.97 to 18.4)	-94.9 (-97.4 to -91.1)	0.19 (0.17 to 0.21)	0 (0 to 0.01)	-97.8 (-98.9 to -96.0)
		Klebsiella pneumoniae	69.9 (54.7 to 89.5)	47.3 (35.6 to 61.4)	-32.4 (-41.8 to -23.9)	0.09 (0.08 to 0.1)	0.03 (0.02 to 0.03)	-69.4 (-74.0 to -64.8)

		Legionella spp.	94.2 (74.4 to 122)	82.2 (62.2 to 107)	-12.7 (-26.5 to -0.1)	0.08 (0.07 to 0.09)	0.03 (0.03 to 0.04)	-60.5 (-67.0 to -53.8)
		Mycoplasma spp.	314 (243 to 404)	205 (155 to 269)	-34.7 (-43.7 to -26.4)	0.11 (0.09 to 0.12)	0.03 (0.03 to 0.04)	-70.5 (-74.9 to -66.1)
		Polymicrobial	3.2 (1.85 to 5.17)	2.44 (1.3 to 4.12)	-24.2 (-36.1 to -12.6)	0 (0 to 0.01)	0 (0 to 0)	-65.7 (-71.8 to -59.6)
		Pseudomonas aeruginosa	96.9 (75.1 to 125)	105 (78.9 to 137)	8.5 (-6.6 to 23.2)	0.09 (0.08 to 0.1)	0.04 (0.04 to 0.05)	-50.9 (-58.4 to -43.3)
		Respiratory syncytial virus	76.2 (60.5 to 97.5)	4.3 (2.11 to 7.82)	-94.4 (-97.1 to -90.2)	0.03 (0.03 to 0.04)	0 (0 to 0)	-97.5 (-98.8 to -95.6)
		Staphylococcus aureus	128 (100 to 164)	143 (108 to 185)	11.5 (-4.1 to 24.5)	0.23 (0.21 to 0.26)	0.12 (0.1 to 0.13)	-49.5 (-57.0 to -42.2)
		Streptococcus pneumoniae	1080 (853 to 1390)	471 (357 to 615)	-56.5 (-62.6 to -51.7)	0.81 (0.74 to 0.9)	0.16 (0.14 to 0.18)	-80.3 (-83.1 to -77.4)
		Other viruses	701 (548 to 900)	516 (392 to 670)	-26.4 (-36.4 to -18.0)	0.21 (0.19 to 0.23)	0.07 (0.06 to 0.08)	-66.7 (-71.6 to -61.8)
		Other bacterial pathogens	100 (77.4 to 128)	58.8 (43.5 to 78)	-41.3 (-49.8 to -33.7)	0.07 (0.06 to 0.08)	0.02 (0.02 to 0.02)	-73.4 (-77.5 to -69.3)
	15-49 years	*Acinetobacter baumannii*	50.6 (41.4 to 61.1)	26.3 (21.2 to 32.3)	-48.0 (-53.9 to -41.1)	0.32 (0.27 to 0.38)	0.09 (0.08 to 0.11)	-71.9 (-75.7 to -67.4)
		Chlamydia spp.	329 (285 to 379)	163 (137 to 193)	-50.5 (-56.1 to -44.0)	0.52 (0.47 to 0.58)	0.14 (0.12 to 0.16)	-73.2 (-76.7 to -69.2)
		Enterobacter spp.	46.7 (38.8 to 55.1)	48.7 (39.5 to 58.9)	4.3 (-9.1 to 18.4)	0.28 (0.24 to 0.33)	0.16 (0.14 to 0.18)	-43.6 (-51.0 to -35.3)
		Escherichia coli	99.9 (86 to 115)	85.6 (72 to 100)	-14.2 (-24.3 to -2.7)	0.63 (0.56 to 0.7)	0.29 (0.26 to 0.33)	-53.6 (-60.1 to -46.7)
		Fungus	120 (103 to 139)	96.9 (80 to 115)	-19.0 (-29.0 to -7.6)	0.74 (0.65 to 0.84)	0.32 (0.28 to 0.37)	-56.2 (-62.2 to -49.9)
		Group B Streptococcus	137 (118 to 161)	127 (107 to 150)	-7.8 (-18.6 to 4.5)	0.61 (0.54 to 0.69)	0.3 (0.27 to 0.34)	-50.2 (-57.2 to -42.8)
		Haemophilus influenzae	615 (541 to 693)	360 (307 to 422)	-41.4 (-47.2 to -34.3)	1.47 (1.34 to 1.61)	0.32 (0.29 to 0.36)	-78.0 (-80.7 to -74.8)
		Influenza	835 (737 to 944)	42 (21.8 to 71.4)	-95.0 (-97.4 to -91.5)	3.91 (3.59 to 4.24)	0.1 (0.05 to 0.17)	-97.4 (-98.7 to -95.6)
		Klebsiella pneumoniae	291 (256 to 330)	194 (165 to 227)	-33.4 (-40.2 to -25.6)	1.82 (1.66 to 1.99)	0.65 (0.59 to 0.73)	-64.0 (-68.4 to -59.3)
		Legionella spp.	393 (343 to 449)	337 (288 to 394)	-14.0 (-24.2 to -2.3)	1.69 (1.5 to 1.88)	0.78 (0.7 to 0.87)	-53.5 (-59.6 to -46.5)
		Mycoplasma spp.	1310 (1150 to 1470)	840 (719 to 983)	-35.7 (-42.7 to -27.8)	2.17 (1.96 to 2.38)	0.75 (0.68 to 0.84)	-65.2 (-69.5 to -60.0)
		Polymicrobial	13.4 (7.74 to 21.7)	10 (5.46 to 17.2)	-25.3 (-35.2 to -14.2)	0.09 (0.05 to 0.14)	0.03 (0.02 to 0.06)	-59.6 (-65.5 to -53.2)
		Pseudomonas aeruginosa	404 (352 to 461)	432 (366 to 505)	6.9 (-4.6 to 21.2)	1.75 (1.58 to 1.93)	1.01 (0.92 to 1.12)	-42.2 (-49.8 to -34.4)
		Respiratory syncytial virus	318 (279 to 362)	17.6 (9.05 to 30.4)	-94.4 (-97.1 to -90.5)	0.68 (0.61 to 0.75)	0.02 (0.01 to 0.03)	-97.1 (-98.5 to -95.1)
		Staphylococcus aureus	534 (472 to 601)	587 (499 to 683)	9.9 (-0.8 to 22.9)	4.74 (4.36 to 5.13)	2.81 (2.55 to 3.12)	-40.6 (-47.7 to -32.8)

		Streptococcus pneumoniae	4510 (3980 to 5070)	1930 (1650 to 2240)	-57.1 (-61.3 to -52.0)	16.7 (15.4 to 18)	3.85 (3.49 to 4.27)	-76.8 (-79.5 to -73.9)
		Other viruses	2920 (2590 to 3270)	2120 (1810 to 2460)	-27.5 (-34.3 to -19.2)	4.24 (3.9 to 4.61)	1.66 (1.5 to 1.84)	-60.8 (-65.6 to -55.8)
		Other bacterial pathogens	417 (352 to 491)	242 (198 to 291)	-42.1 (-48.2 to -34.5)	1.47 (1.27 to 1.68)	0.46 (0.39 to 0.54)	-68.7 (-72.7 to -64.1)
	50-69 years	*Acinetobacter baumannii*	181 (154 to 211)	99.5 (83.1 to 117)	-44.8 (-51.7 to -37.1)	1.94 (1.66 to 2.24)	1.04 (0.89 to 1.21)	-46.4 (-53.7 to -38.6)
		Chlamydia spp.	695 (616 to 786)	346 (302 to 394)	-50.1 (-56.0 to -43.6)	1.85 (1.66 to 2.06)	0.9 (0.8 to 1.01)	-51.6 (-58.1 to -44.0)
		Enterobacter spp.	162 (140 to 187)	175 (149 to 201)	8.2 (-5.1 to 22.4)	1.65 (1.44 to 1.88)	1.72 (1.52 to 1.97)	5.0 (-9.0 to 19.9)
		Escherichia coli	382 (339 to 429)	332 (292 to 372)	-12.9 (-23.5 to -1.6)	4.03 (3.61 to 4.5)	3.39 (3.04 to 3.76)	-15.6 (-26.4 to -2.9)
		Fungus	364 (320 to 415)	308 (266 to 352)	-15.4 (-25.8 to -4.6)	3.79 (3.34 to 4.33)	3.1 (2.73 to 3.5)	-18.0 (-29.1 to -5.8)
		Group B Streptococcus	337 (296 to 383)	316 (276 to 358)	-6.0 (-18.0 to 6.6)	2.52 (2.24 to 2.85)	2.29 (2.04 to 2.55)	-8.8 (-21.3 to 4.9)
		Haemophilus influenzae	1290 (1170 to 1420)	837 (738 to 938)	-34.9 (-41.8 to -27.6)	5.17 (4.73 to 5.66)	2.26 (2.06 to 2.49)	-56.1 (-61.4 to -50.1)
		Influenza	1500 (1380 to 1660)	80 (41.1 to 139)	-94.7 (-97.3 to -90.9)	11.8 (10.9 to 12.9)	0.59 (0.3 to 1.01)	-95.0 (-97.5 to -91.4)
		Klebsiella pneumoniae	780 (716 to 858)	601 (533 to 670)	-23.0 (-30.8 to -14.7)	8.18 (7.49 to 8.96)	6.1 (5.51 to 6.72)	-25.3 (-34.0 to -15.1)
		Legionella spp.	1150 (1040 to 1290)	1030 (911 to 1160)	-10.7 (-21.7 to 1.8)	8.33 (7.46 to 9.29)	7.19 (6.48 to 7.99)	-13.4 (-25.7 to -0.9)
		Mycoplasma spp.	1250 (1130 to 1390)	773 (682 to 868)	-38.2 (-45.2 to -30.7)	3.49 (3.14 to 3.84)	2.09 (1.89 to 2.3)	-40.1 (-47.6 to -31.7)
		Polymicrobial	39.6 (23.8 to 64.9)	31.2 (17.5 to 52.9)	-21.6 (-31.7 to -10.2)	0.42 (0.25 to 0.7)	0.32 (0.18 to 0.54)	-24.0 (-34.9 to -12.1)
		Pseudomonas aeruginosa	1200 (1080 to 1330)	1290 (1140 to 1450)	8.3 (-4.5 to 21.1)	8.71 (7.86 to 9.57)	9.12 (8.25 to 10.1)	5.0 (-7.2 to 19.6)
		Respiratory syncytial virus	423 (376 to 473)	23.8 (12.3 to 42)	-94.4 (-97.1 to -90.4)	1.52 (1.35 to 1.71)	0.08 (0.04 to 0.14)	-94.7 (-97.3 to -90.8)
		Staphylococcus aureus	1390 (1280 to 1530)	1600 (1420 to 1780)	15.1 (3.3 to 27.1)	20.7 (19 to 22.6)	23.1 (21 to 25.5)	11.7 (-1.1 to 26.4)
		Streptococcus pneumoniae	5810 (5340 to 6350)	2720 (2400 to 3040)	-53.1 (-57.7 to -48.2)	36 (33.2 to 39.2)	16.3 (14.8 to 17.9)	-54.5 (-59.6 to -48.5)
		Other viruses	3290 (3030 to 3600)	2740 (2410 to 3060)	-16.8 (-25.3 to -7.7)	8.02 (7.38 to 8.79)	6.46 (5.86 to 7.11)	-19.3 (-28.5 to -8.9)
		Other bacterial pathogens	1320 (1150 to 1510)	790 (659 to 925)	-40.0 (-46.7 to -32.3)	7.8 (6.76 to 8.97)	4.54 (3.89 to 5.26)	-41.7 (-48.9 to -33.6)
	70+ years	*Acinetobacter baumannii*	286 (243 to 337)	225 (191 to 261)	-21.4 (-30.9 to -12.6)	14.2 (11.9 to 16.5)	11.1 (8.68 to 13)	-22.3 (-31.9 to -13.1)
		Chlamydia spp.	1310 (1160 to 1490)	932 (817 to 1050)	-28.9 (-37.2 to -20.8)	16.2 (14.1 to 18)	11.4 (9.2 to 12.8)	-29.7 (-38.2 to -21.7)
		Enterobacter spp.	250 (216 to 291)	384 (331 to 438)	54.0 (34.7 to 72.0)	11.8 (10 to 13.6)	17.9 (14.3 to 20.6)	52.3 (32.4 to 72.3)
		Escherichia coli	744 (661 to 837)	920 (817 to 1030)	23.9 (9.4 to 38.7)	36.3 (31.7 to 40.2)	44.5 (36.2 to 49.9)	22.5 (7.0 to 37.5)
		Fungus	322 (280 to 370)	387 (338 to 441)	20.3 (5.7 to 34.7)	15.5 (13.4 to 17.6)	18.4 (14.9 to 21.2)	19.0 (3.8 to 33.2)

		Group B Streptococcus	490 (435 to 559)	655 (584 to 736)	33.8 (16.5 to 49.5)	17 (14.8 to 19)	22.4 (18.2 to 25.3)	32.3 (16.7 to 47.9)
		Haemophilus influenzae	1840 (1670 to 2040)	1700 (1520 to 1890)	-7.3 (-17.2 to 1.5)	34.2 (30.4 to 37)	21.8 (18 to 24.3)	-36.4 (-43.0 to -29.7)
		Influenza	3630 (3320 to 4000)	275 (141 to 473)	-92.4 (-96.0 to -86.9)	132 (117 to 143)	9.54 (4.93 to 16.2)	-92.8 (-96.3 to -87.7)
		Klebsiella pneumoniae	1360 (1250 to 1500)	1490 (1340 to 1670)	9.6 (-1.7 to 19.5)	66.2 (58.5 to 71.9)	71.8 (58.8 to 80.2)	8.4 (-3.0 to 18.9)
		Legionella spp.	1320 (1170 to 1480)	1670 (1490 to 1860)	27.1 (12.0 to 41.8)	44 (38.2 to 49)	55.2 (45.3 to 61.6)	25.7 (10.6 to 40.9)
		Mycoplasma spp.	1680 (1500 to 1870)	1480 (1330 to 1650)	-12.1 (-22.0 to -2.6)	21.7 (19.1 to 23.9)	18.8 (15.3 to 21.1)	-13.1 (-22.5 to -3.5)
		Polymicrobial	72.1 (42.6 to 121)	80.8 (44.1 to 139)	11.5 (-3.3 to 26.0)	3.56 (2.09 to 6.12)	3.95 (2.18 to 6.82)	10.3 (-4.7 to 24.9)
		Pseudomonas aeruginosa	1980 (1790 to 2210)	3040 (2720 to 3380)	54.1 (36.3 to 69.8)	66.6 (58.8 to 72.8)	101 (83 to 113)	52.3 (33.8 to 68.7)
		Respiratory syncytial virus	630 (559 to 711)	50.5 (26 to 88.5)	-92.0 (-95.8 to -86.0)	10.5 (9.15 to 11.7)	0.8 (0.41 to 1.35)	-92.3 (-96.1 to -87.0)
		Staphylococcus aureus	2860 (2610 to 3150)	4690 (4210 to 5210)	63.9 (46.8 to 78.1)	197 (176 to 213)	320 (261 to 355)	62.0 (45.0 to 77.7)
		Streptococcus pneumoniae	9480 (8670 to 10400)	6320 (5670 to 7010)	-33.2 (-40.1 to -27.4)	272 (242 to 293)	180 (148 to 200)	-34.0 (-41.0 to -27.4)
		Other viruses	5310 (4860 to 5850)	6290 (5630 to 6980)	18.4 (5.7 to 28.9)	59.9 (53.2 to 64.5)	70.2 (57.7 to 77.9)	17.1 (5.0 to 28.4)
		Other bacterial pathogens	2800 (2450 to 3230)	2390 (2010 to 2780)	-14.6 (-24.5 to -5.8)	77.5 (66.2 to 88.2)	65.6 (52.1 to 77.3)	-15.5 (-25.0 to -6.2)
Belgium	All Ages	*Acinetobacter baumannii*	956 (819 to 1110)	604 (522 to 697)	-36.7 (-44.1 to -29.6)	45.9 (38.6 to 53.7)	34.7 (27 to 40.8)	-24.3 (-34.5 to -14.4)
		Chlamydia spp.	4290 (3860 to 4760)	3450 (3100 to 3870)	-19.4 (-28.3 to -9.7)	50.8 (44.8 to 56.2)	49 (39.5 to 56.1)	-3.6 (-16.3 to 9.9)
		Enterobacter spp.	824 (720 to 939)	1730 (1540 to 1930)	110.6 (86.9 to 138.3)	36.7 (31.2 to 42.1)	92.6 (73.3 to 106)	152.8 (118.5 to 186.3)
		Escherichia coli	2250 (2010 to 2500)	4160 (3750 to 4560)	85.3 (65.3 to 105.7)	111 (96.9 to 122)	242 (195 to 273)	119.2 (90.3 to 146.3)
		Fungus	1410 (1250 to 1610)	2040 (1820 to 2280)	44.9 (28.4 to 63.2)	53.8 (47.2 to 60.9)	95.2 (76.9 to 110)	77.1 (53.4 to 100.5)
		Group B Streptococcus	1730 (1550 to 1930)	2940 (2660 to 3260)	70.2 (49.1 to 91.5)	54.2 (47.1 to 60.7)	111 (89.7 to 126)	105.1 (76.1 to 133.6)
		Haemophilus influenzae	6940 (6420 to 7580)	8240 (7510 to 8980)	18.8 (9.7 to 29.3)	110 (98 to 119)	108 (87.8 to 121)	-2.3 (-13.3 to 7.4)
		Influenza	10900 (10100 to 11800)	2920 (1070 to 5820)	-73.1 (-90.1 to -47.4)	395 (349 to 424)	126 (43.4 to 262)	-68.1 (-88.8 to -33.4)
		Klebsiella pneumoniae	4510 (4160 to 4930)	5290 (4830 to 5790)	17.5 (7.6 to 28.7)	206 (183 to 222)	296 (239 to 332)	43.5 (26.1 to 57.9)
		Legionella spp.	4920 (4410 to 5480)	7560 (6880 to 8270)	54.0 (36.6 to 71.1)	141 (124 to 157)	264 (214 to 296)	86.7 (62.7 to 112.7)
		Mycoplasma spp.	7350 (6740 to 8010)	6960 (6370 to 7570)	-5.1 (-13.2 to 3.5)	72.7 (64.4 to 79)	81.4 (66.8 to 91.9)	12.0 (-1.7 to 24.4)
		Polymicrobial	234 (141 to 381)	347 (199 to 595)	47.8 (29.1 to 67.7)	11.4 (6.85 to 18.9)	20.2 (11.1 to 34.5)	75.7 (47.2 to 102.7)

		Pseudomonas aeruginosa	6370 (5800 to 7060)	11400 (10300 to 12400)	79.1 (62.3 to 98.5)	204 (182 to 222)	434 (346 to 486)	113.0 (84.8 to 136.1)
		Respiratory syncytial virus	3570 (3260 to 3920)	629 (231 to 1250)	-82.4 (-93.5 to -65.0)	37.8 (33.9 to 41.3)	7.88 (2.72 to 16.6)	-79.1 (-92.7 to -56.5)
		Staphylococcus aureus	8590 (7930 to 9350)	20000 (18200 to 21900)	133.2 (114.0 to 154.5)	592 (525 to 639)	1650 (1330 to 1840)	177.8 (145.2 to 205.9)
		Streptococcus pneumoniae	36400 (33900 to 39400)	18300 (16800 to 19800)	-49.7 (-53.4 to -45.6)	875 (780 to 939)	557 (452 to 622)	-36.4 (-43.7 to -30.4)
		Other viruses	21800 (20400 to 23500)	24300 (22300 to 26300)	11.3 (2.8 to 20.4)	194 (174 to 207)	278 (227 to 310)	43.5 (27.3 to 57.0)
		Other bacterial pathogens	8180 (7130 to 9380)	8370 (7250 to 9660)	2.5 (-8.1 to 14.1)	234 (200 to 266)	283 (219 to 339)	21.2 (5.1 to 36.4)
	Under 5	*Acinetobacter baumannii*	7.43 (4.99 to 10.9)	3.05 (1.91 to 4.7)	-59.1 (-65.3 to -51.6)	0.22 (0.13 to 0.37)	0.09 (0.04 to 0.16)	-62.4 (-72.4 to -49.9)
		Chlamydia spp.	116 (94.3 to 142)	57.6 (45.7 to 73)	-50.3 (-58.0 to -40.8)	0.41 (0.36 to 0.45)	0.11 (0.09 to 0.14)	-72.7 (-79.3 to -65.4)
		Enterobacter spp.	18.4 (13.6 to 24.4)	22.2 (16 to 30.1)	20.7 (-0.6 to 46.5)	0.27 (0.2 to 0.37)	0.14 (0.1 to 0.2)	-46.9 (-59.9 to -32.5)
		Escherichia coli	52.5 (42.4 to 65)	66.1 (51.4 to 83.2)	26.4 (4.4 to 50.3)	0.73 (0.64 to 0.84)	0.38 (0.29 to 0.46)	-48.4 (-60.5 to -34.6)
		Fungus	10.2 (7 to 14.5)	9.32 (6.45 to 13.3)	-7.9 (-25.1 to 12.0)	0.1 (0.07 to 0.13)	0.03 (0.02 to 0.05)	-64.3 (-74.4 to -53.2)
		Group B Streptococcus	49 (39.2 to 61.5)	50.5 (39.5 to 64)	3.3 (-14.2 to 23.3)	0.57 (0.51 to 0.65)	0.27 (0.21 to 0.34)	-53.0 (-64.0 to -41.5)
		Haemophilus influenzae	482 (390 to 592)	309 (245 to 390)	-35.7 (-45.2 to -23.9)	2.01 (1.8 to 2.25)	0.3 (0.23 to 0.38)	-84.9 (-88.5 to -80.7)
		Influenza	386 (314 to 474)	46 (17.1 to 88.1)	-88.1 (-95.5 to -77.2)	3.03 (2.73 to 3.35)	0.14 (0.05 to 0.3)	-95.3 (-98.3 to -89.7)
		Klebsiella pneumoniae	193 (157 to 235)	95.2 (76.1 to 120)	-50.5 (-57.6 to -42.4)	2.11 (1.9 to 2.33)	0.5 (0.39 to 0.61)	-76.4 (-81.8 to -70.4)
		Legionella spp.	27 (19 to 37.8)	25.5 (17.1 to 36.3)	-5.6 (-21.5 to 11.7)	0.33 (0.25 to 0.41)	0.16 (0.12 to 0.22)	-49.5 (-61.8 to -36.3)
		Mycoplasma spp.	262 (214 to 324)	275 (219 to 344)	4.9 (-11.4 to 23.3)	0.74 (0.66 to 0.83)	0.3 (0.23 to 0.38)	-58.7 (-68.9 to -47.4)
		Polymicrobial	8.58 (4.93 to 13.9)	5.99 (3.33 to 9.76)	-30.1 (-40.9 to -17.2)	0.34 (0.18 to 0.59)	0.2 (0.09 to 0.36)	-43.5 (-58.3 to -26.6)
		Pseudomonas aeruginosa	159 (130 to 197)	227 (181 to 283)	43.0 (21.6 to 69.0)	1.35 (1.2 to 1.52)	0.77 (0.61 to 0.96)	-42.6 (-56.3 to -28.0)
		Respiratory syncytial virus	1280 (1060 to 1550)	183 (67.4 to 355)	-85.6 (-94.6 to -72.3)	4.94 (4.53 to 5.42)	0.29 (0.1 to 0.62)	-94.1 (-97.9 to -87.2)
		Staphylococcus aureus	171 (140 to 209)	224 (177 to 278)	30.6 (14.0 to 50.1)	2.63 (2.38 to 2.9)	1.37 (1.09 to 1.69)	-47.7 (-59.9 to -33.9)
		Streptococcus pneumoniae	2440 (2000 to 2960)	449 (358 to 561)	-81.6 (-83.8 to -78.8)	14.2 (12.8 to 15.6)	1 (0.78 to 1.24)	-92.9 (-94.6 to -91.0)
		Other viruses	2550 (2090 to 3100)	1260 (1000 to 1570)	-50.7 (-56.7 to -43.5)	5.94 (5.38 to 6.51)	1.27 (1 to 1.56)	-78.6 (-83.7 to -73.0)
		Other bacterial pathogens	56.5 (42.7 to 72.9)	36.7 (27.2 to 48.2)	-35.0 (-46.4 to -22.5)	0.4 (0.31 to 0.51)	0.12 (0.09 to 0.17)	-69.4 (-77.4 to -60.4)

	5-14 years	*Acinetobacter baumannii*	13.9 (10.1 to 18.7)	6.84 (5 to 9.24)	-50.6 (-56.6 to -44.1)	0.06 (0.05 to 0.07)	0.01 (0.01 to 0.02)	-73.5 (-78.7 to -67.9)
		Chlamydia spp.	88.5 (64.5 to 118)	58.3 (43.1 to 77.8)	-34.0 (-41.4 to -25.2)	0.09 (0.08 to 0.1)	0.03 (0.03 to 0.04)	-64.7 (-71.1 to -57.1)
		Enterobacter spp.	12.3 (8.88 to 16.6)	20.9 (15 to 28.3)	70.6 (50.2 to 94.2)	0.05 (0.04 to 0.05)	0.04 (0.04 to 0.05)	-8.6 (-25.9 to 10.4)
		Escherichia coli	26.5 (19.3 to 34.9)	40.4 (30.1 to 53.9)	52.5 (36.1 to 72.3)	0.1 (0.09 to 0.12)	0.08 (0.07 to 0.1)	-18.3 (-33.0 to -0.6)
		Fungus	31.8 (23.3 to 42.7)	39 (28.4 to 52.6)	22.7 (7.1 to 39.4)	0.12 (0.1 to 0.14)	0.08 (0.07 to 0.1)	-34.3 (-47.1 to -19.7)
		Group B Streptococcus	36.3 (26 to 48.2)	51.6 (38 to 69.3)	42.5 (25.5 to 62.0)	0.1 (0.09 to 0.12)	0.08 (0.06 to 0.09)	-23.6 (-38.6 to -6.5)
		Haemophilus influenzae	161 (121 to 211)	154 (115 to 204)	-4.9 (-13.4 to 5.9)	0.24 (0.21 to 0.27)	0.08 (0.07 to 0.1)	-66.0 (-71.9 to -59.3)
		Influenza	220 (163 to 288)	44.9 (15.1 to 93.5)	-79.6 (-92.5 to -59.0)	0.64 (0.57 to 0.72)	0.07 (0.02 to 0.14)	-89.2 (-96.3 to -77.1)
		Klebsiella pneumoniae	77.4 (57.4 to 100)	66.4 (49.4 to 88)	-14.2 (-21.7 to -5.1)	0.3 (0.27 to 0.34)	0.14 (0.12 to 0.16)	-54.0 (-62.0 to -45.0)
		Legionella spp.	102 (75.4 to 135)	131 (97.6 to 171)	28.8 (14.6 to 46.0)	0.27 (0.24 to 0.31)	0.19 (0.16 to 0.22)	-31.0 (-44.7 to -16.5)
		Mycoplasma spp.	344 (254 to 448)	306 (228 to 407)	-11.1 (-19.7 to -1.2)	0.36 (0.31 to 0.4)	0.17 (0.14 to 0.2)	-52.3 (-60.7 to -42.8)
		Polymicrobial	3.59 (2.02 to 5.83)	4.24 (2.27 to 7.18)	17.8 (1.9 to 35.2)	0.01 (0.01 to 0.02)	0.01 (0.01 to 0.02)	-36.9 (-49.4 to -21.4)
		Pseudomonas aeruginosa	105 (77.5 to 138)	160 (119 to 211)	52.5 (39.0 to 69.7)	0.28 (0.25 to 0.33)	0.23 (0.2 to 0.27)	-18.3 (-32.4 to -1.2)
		Respiratory syncytial virus	82.1 (61.6 to 108)	13.8 (4.74 to 28.7)	-83.3 (-93.8 to -66.1)	0.11 (0.1 to 0.12)	0.01 (0 to 0.02)	-91.2 (-96.9 to -81.0)
		Staphylococcus aureus	140 (104 to 183)	256 (191 to 334)	82.5 (67.9 to 99.4)	0.78 (0.69 to 0.87)	0.76 (0.64 to 0.87)	-2.2 (-19.3 to 17.2)
		Streptococcus pneumoniae	1190 (890 to 1550)	463 (346 to 607)	-61.1 (-64.1 to -57.6)	2.75 (2.45 to 3.07)	0.57 (0.49 to 0.66)	-79.2 (-82.8 to -75.2)
		Other viruses	757 (566 to 991)	673 (504 to 884)	-11.1 (-18.0 to -2.8)	0.69 (0.61 to 0.77)	0.33 (0.28 to 0.38)	-52.4 (-60.5 to -43.1)
		Other bacterial pathogens	111 (82 to 147)	90 (65.5 to 120)	-18.8 (-27.0 to -8.4)	0.24 (0.21 to 0.29)	0.11 (0.09 to 0.13)	-56.4 (-64.3 to -47.3)
	15-49 years	*Acinetobacter baumannii*	59.1 (47.1 to 72.1)	27.8 (22.5 to 33.7)	-52.9 (-58.6 to -46.6)	0.68 (0.57 to 0.8)	0.24 (0.21 to 0.29)	-64.0 (-68.7 to -58.8)
		Chlamydia spp.	377 (319 to 444)	237 (199 to 279)	-37.1 (-43.8 to -29.4)	1.08 (0.97 to 1.2)	0.52 (0.46 to 0.58)	-52.0 (-57.9 to -45.1)
		Enterobacter spp.	52.3 (42.3 to 63.6)	84.8 (70.5 to 101)	62.6 (42.9 to 84.2)	0.57 (0.49 to 0.66)	0.71 (0.62 to 0.81)	24.2 (7.1 to 42.2)
		Escherichia coli	113 (94.6 to 133)	164 (140 to 192)	45.4 (29.5 to 61.8)	1.28 (1.15 to 1.43)	1.42 (1.28 to 1.56)	11.0 (-3.5 to 26.1)
		Fungus	135 (112 to 160)	158 (132 to 187)	17.0 (3.6 to 31.1)	1.51 (1.34 to 1.7)	1.35 (1.17 to 1.53)	-10.6 (-22.9 to 2.9)
		Group B Streptococcus	155 (130 to 185)	210 (177 to 247)	35.8 (18.6 to 52.9)	1.24 (1.1 to 1.4)	1.29 (1.14 to 1.45)	3.8 (-10.7 to 20.5)
		Haemophilus influenzae	688 (586 to 796)	623 (531 to 724)	-9.3 (-17.2 to -1.1)	2.97 (2.74 to 3.22)	1.37 (1.25 to 1.52)	-53.8 (-59.0 to -47.9)

		Influenza	936 (801 to 1080)	182 (65.3 to 367)	-80.6 (-92.8 to -60.8)	7.9 (7.34 to 8.52)	1.16 (0.41 to 2.43)	-85.3 (-95.0 to -68.9)
		Klebsiella pneumoniae	330 (281 to 381)	269 (233 to 313)	-18.2 (-25.3 to -11.2)	3.71 (3.42 to 4)	2.31 (2.1 to 2.55)	-37.5 (-44.1 to -30.3)
		Legionella spp.	433 (365 to 505)	531 (457 to 614)	22.8 (9.6 to 36.7)	3.35 (3.03 to 3.69)	3.14 (2.85 to 3.47)	-6.2 (-18.4 to 7.3)
		Mycoplasma spp.	1470 (1240 to 1690)	1240 (1070 to 1440)	-15.2 (-22.8 to -6.8)	4.38 (4.02 to 4.75)	2.83 (2.58 to 3.1)	-35.2 (-42.7 to -27.2)
		Polymicrobial	15.3 (8.96 to 25)	17.3 (9.51 to 29.6)	12.3 (-2.6 to 28.7)	0.17 (0.1 to 0.29)	0.15 (0.08 to 0.25)	-14.2 (-27.0 to 0.4)
		Pseudomonas aeruginosa	447 (379 to 519)	649 (558 to 753)	45.4 (32.2 to 59.4)	3.49 (3.18 to 3.8)	3.87 (3.55 to 4.26)	11.1 (-2.1 to 24.3)
		Respiratory syncytial virus	350 (298 to 409)	55.7 (20.5 to 113)	-84.1 (-94.2 to -67.5)	1.35 (1.23 to 1.47)	0.16 (0.06 to 0.35)	-88.0 (-95.9 to -74.3)
		Staphylococcus aureus	597 (514 to 687)	1040 (897 to 1200)	74.0 (60.4 to 87.7)	9.54 (8.89 to 10.3)	12.7 (11.6 to 13.9)	32.9 (18.9 to 48.1)
		Streptococcus pneumoniae	5070 (4360 to 5810)	1880 (1630 to 2180)	-62.9 (-65.9 to -60.1)	33.7 (31.5 to 36.1)	9.54 (8.76 to 10.4)	-71.7 (-74.7 to -68.6)
		Other viruses	3230 (2760 to 3700)	2730 (2360 to 3150)	-15.3 (-22.0 to -8.7)	8.44 (7.87 to 9.05)	5.45 (4.99 to 5.99)	-35.3 (-42.1 to -28.0)
		Other bacterial pathogens	473 (389 to 561)	366 (299 to 437)	-22.6 (-30.5 to -14.1)	3 (2.61 to 3.4)	1.77 (1.52 to 2.05)	-40.8 (-48.4 to -32.9)
	50-69 years	*Acinetobacter baumannii*	321 (272 to 378)	158 (134 to 185)	-50.6 (-56.7 to -43.9)	5.54 (4.75 to 6.44)	2.78 (2.35 to 3.21)	-49.7 (-56.8 to -41.3)
		Chlamydia spp.	1210 (1060 to 1390)	761 (666 to 871)	-37.1 (-44.5 to -28.0)	5.19 (4.56 to 5.85)	3.32 (2.92 to 3.75)	-35.9 (-45.3 to -25.6)
		Enterobacter spp.	276 (239 to 320)	456 (398 to 522)	65.8 (44.9 to 88.7)	4.51 (3.88 to 5.16)	7.59 (6.64 to 8.58)	69.0 (43.9 to 97.4)
		Escherichia coli	659 (577 to 755)	934 (825 to 1050)	42.1 (25.3 to 61.6)	11.1 (9.91 to 12.5)	16.1 (14.5 to 17.9)	44.7 (25.3 to 66.9)
		Fungus	629 (539 to 724)	753 (653 to 858)	20.1 (4.8 to 37.8)	10.5 (9.19 to 12)	12.8 (11.2 to 14.5)	22.3 (4.1 to 42.4)
		Group B Streptococcus	578 (502 to 665)	782 (688 to 894)	35.8 (17.7 to 56.4)	6.93 (6.07 to 7.9)	9.55 (8.36 to 10.8)	38.4 (18.2 to 61.6)
		Haemophilus influenzae	2190 (1970 to 2450)	2150 (1910 to 2400)	-1.7 (-11.3 to 10.0)	14.1 (12.9 to 15.5)	9.41 (8.47 to 10.4)	-33.3 (-42.3 to -24.4)
		Influenza	2570 (2300 to 2870)	539 (191 to 1070)	-79.0 (-92.4 to -57.2)	32.4 (29.5 to 35.7)	6.82 (2.47 to 14)	-78.9 (-92.5 to -56.4)
		Klebsiella pneumoniae	1350 (1210 to 1500)	1270 (1130 to 1420)	-5.8 (-15.1 to 5.6)	22.6 (20.5 to 24.7)	21.7 (19.5 to 23.9)	-4.0 (-16.3 to 9.3)
		Legionella spp.	1940 (1710 to 2210)	2410 (2140 to 2690)	24.3 (9.5 to 40.9)	22.5 (20 to 25)	28.3 (25.5 to 31.4)	26.6 (9.2 to 46.8)
		Mycoplasma spp.	2140 (1910 to 2420)	1620 (1430 to 1820)	-24.4 (-33.1 to -14.6)	9.56 (8.62 to 10.6)	7.34 (6.62 to 8.12)	-23.0 (-33.2 to -11.8)
		Polymicrobial	69.3 (41.2 to 114)	80.9 (46.1 to 137)	16.4 (0.4 to 34.1)	1.19 (0.7 to 1.95)	1.41 (0.79 to 2.44)	18.6 (-1.4 to 38.9)
		Pseudomonas aeruginosa	2020 (1790 to 2290)	2810 (2500 to 3120)	39.3 (24.5 to 57.1)	23.6 (21.2 to 26.1)	33.4 (30.1 to 36.6)	41.9 (22.6 to 62.3)
		Respiratory syncytial virus	711 (619 to 811)	110 (39.9 to 222)	-84.4 (-94.4 to -68.2)	4.09 (3.6 to 4.58)	0.64 (0.23 to 1.31)	-84.4 (-94.4 to -67.2)
		Staphylococcus aureus	2370 (2120 to 2650)	4270 (3800 to 4740)	80.0 (62.8 to 100.8)	56.7 (51.7 to 62)	104 (94 to 114)	83.3 (60.1 to 107.5)

		Streptococcus pneumoniae	9960 (8990 to 11000)	4240 (3770 to 4710)	-57.3 (-61.3 to -52.6)	98.9 (90.3 to 108)	42.9 (38.8 to 47.3)	-56.5 (-61.8 to -50.7)
		Other viruses	5540 (4970 to 6130)	5420 (4820 to 6030)	-2.1 (-11.5 to 8.9)	21.6 (19.7 to 23.6)	21.5 (19.5 to 23.7)	-0.3 (-12.3 to 13.4)
		Other bacterial pathogens	2280 (1920 to 2640)	1770 (1490 to 2070)	-22.1 (-31.1 to -11.2)	21.6 (18.6 to 24.8)	17.1 (14.6 to 20.2)	-20.5 (-31.7 to -8.5)
	70+ years	*Acinetobacter baumannii*	555 (469 to 651)	408 (347 to 477)	-26.3 (-35.6 to -17.0)	39.4 (32.8 to 46.2)	31.6 (24.2 to 37.4)	-19.8 (-31.1 to -9.2)
		Chlamydia spp.	2500 (2190 to 2820)	2340 (2050 to 2660)	-6.2 (-17.6 to 6.6)	44.1 (38.3 to 49.4)	45 (35.7 to 52)	2.1 (-11.5 to 17.0)
		Enterobacter spp.	465 (399 to 538)	1150 (998 to 1310)	147.4 (117.1 to 183.5)	31.3 (26.4 to 36.3)	84.2 (65.2 to 97.5)	169.2 (131.6 to 206.5)
		Escherichia coli	1400 (1240 to 1570)	2950 (2630 to 3310)	111.9 (85.4 to 137.8)	97.4 (84.4 to 108)	225 (178 to 254)	130.6 (98.3 to 160.7)
		Fungus	605 (526 to 703)	1080 (941 to 1240)	79.1 (56.6 to 103.0)	41.6 (35.7 to 47.5)	81 (63.7 to 94.4)	94.8 (66.9 to 121.3)
		Group B Streptococcus	916 (805 to 1040)	1850 (1620 to 2100)	102.5 (76.9 to 130.1)	45.3 (38.8 to 51.3)	99.8 (79 to 115)	120.4 (88.4 to 152.1)
		Haemophilus influenzae	3410 (3100 to 3820)	5000 (4470 to 5600)	46.6 (31.9 to 62.1)	90.8 (79.1 to 98.6)	96.6 (76.6 to 109)	6.3 (-6.7 to 17.1)
		Influenza	6760 (6150 to 7490)	2110 (779 to 4250)	-68.8 (-88.6 to -38.2)	351 (306 to 381)	118 (40.7 to 246)	-66.5 (-88.2 to -30.2)
		Klebsiella pneumoniae	2560 (2340 to 2850)	3600 (3220 to 4020)	40.5 (26.3 to 55.7)	178 (155 to 193)	272 (214 to 306)	52.9 (33.6 to 68.7)
		Legionella spp.	2410 (2140 to 2740)	4460 (3970 to 5010)	85.4 (62.4 to 109.3)	115 (99.2 to 129)	232 (184 to 263)	101.8 (75.0 to 131.5)
		Mycoplasma spp.	3130 (2820 to 3510)	3520 (3160 to 3950)	12.7 (0.4 to 25.6)	57.6 (50.1 to 63.3)	70.7 (56.3 to 80.9)	22.7 (6.9 to 37.4)
		Polymicrobial	137 (80.4 to 228)	239 (134 to 411)	73.6 (50.0 to 98.1)	9.68 (5.63 to 16.1)	18.4 (9.97 to 31.6)	88.9 (58.4 to 118.8)
		Pseudomonas aeruginosa	3640 (3270 to 4080)	7540 (6730 to 8430)	107.8 (86.1 to 132.2)	175 (153 to 192)	396 (310 to 446)	126.1 (95.1 to 151.8)
		Respiratory syncytial virus	1150 (1020 to 1310)	266 (97.4 to 529)	-76.8 (-91.5 to -53.2)	27.3 (23.7 to 30.7)	6.78 (2.34 to 14.2)	-75.1 (-91.4 to -48.7)
		Staphylococcus aureus	5310 (4850 to 5870)	14200 (12800 to 15800)	168.5 (142.7 to 195.6)	523 (457 to 567)	1530 (1210 to 1720)	192.1 (156.5 to 222.5)
		Streptococcus pneumoniae	17700 (16200 to 19600)	11200 (10100 to 12500)	-36.4 (-42.3 to -30.0)	725 (634 to 785)	503 (399 to 566)	-30.8 (-39.1 to -23.8)
		Other viruses	9740 (8870 to 10800)	14200 (12700 to 15900)	45.9 (31.7 to 60.9)	157 (137 to 170)	249 (198 to 280)	58.8 (39.5 to 74.6)
		Other bacterial pathogens	5260 (4580 to 6100)	6110 (5230 to 7110)	16.2 (3.3 to 30.5)	208 (176 to 239)	264 (202 to 318)	26.7 (9.3 to 43.8)
Cyprus	All Ages	*Acinetobacter baumannii*	51.7 (44.1 to 59.7)	31.7 (27.4 to 36.5)	-38.5 (-45.5 to -30.1)	2.64 (2.1 to 3.28)	1.22 (0.96 to 1.52)	-53.4 (-64.2 to -40.7)
		Chlamydia spp.	209 (188 to 232)	163 (147 to 179)	-21.6 (-29.8 to -11.4)	2.53 (2.08 to 3.02)	1.5 (1.21 to 1.84)	-40.3 (-54.1 to -24.4)
		Enterobacter spp.	32.5 (28.4 to 37.1)	74.1 (65.4 to 82.7)	128.3 (98.7 to 158.4)	1.51 (1.22 to 1.84)	2.61 (2.08 to 3.18)	74.8 (34.8 to 122.5)
		Escherichia coli	93.7 (83.6 to 104)	169 (153 to 184)	80.4 (60.5 to 102.2)	4.96 (4.02 to 5.91)	6.66 (5.38 to 8.13)	35.3 (4.5 to 71.3)

		Fungus	63.7 (56.2 to 72.1)	96.9 (86.5 to 108)	52.5 (35.1 to 72.2)	2.35 (1.89 to 2.84)	2.79 (2.23 to 3.39)	19.5 (-7.1 to 49.9)
		Group B Streptococcus	74.7 (67.2 to 84)	134 (122 to 147)	80.5 (59.4 to 103.9)	2.34 (1.91 to 2.79)	3.22 (2.6 to 3.93)	38.7 (7.3 to 75.6)
		Haemophilus influenzae	305 (280 to 330)	384 (355 to 415)	26.2 (15.6 to 38.4)	4.6 (3.83 to 5.4)	3.07 (2.5 to 3.72)	-32.9 (-48.1 to -16.4)
		Influenza	445 (408 to 482)	52.1 (27.3 to 90)	-88.3 (-93.9 to -80.1)	16.7 (13.7 to 19.8)	1.45 (0.73 to 2.45)	-91.2 (-95.7 to -84.9)
		Klebsiella pneumoniae	200 (184 to 217)	251 (230 to 271)	25.4 (14.4 to 39.1)	9.31 (7.74 to 11)	9.18 (7.43 to 11)	-0.8 (-22.8 to 24.0)
		Legionella spp.	177 (157 to 197)	351 (322 to 380)	98.8 (76.2 to 122.1)	5.1 (4.21 to 6.1)	7.78 (6.36 to 9.39)	53.7 (17.0 to 96.4)
		Mycoplasma spp.	349 (319 to 382)	394 (361 to 427)	13.0 (3.6 to 23.6)	3.05 (2.51 to 3.56)	2.56 (2.08 to 3.07)	-15.6 (-33.4 to 4.7)
		Polymicrobial	11.4 (7.46 to 17.6)	15 (8.78 to 25.5)	31.3 (10.4 to 55.0)	0.58 (0.36 to 0.94)	0.58 (0.32 to 1)	-1.5 (-26.6 to 27.8)
		Pseudomonas aeruginosa	232 (210 to 255)	530 (487 to 574)	128.3 (105.9 to 154.0)	7.69 (6.35 to 9.05)	13.2 (10.7 to 16.1)	72.4 (32.9 to 118.7)
		Respiratory syncytial virus	190 (172 to 212)	18.4 (9.5 to 31)	-90.3 (-95.0 to -83.6)	1.66 (1.38 to 1.97)	0.11 (0.05 to 0.18)	-93.5 (-96.9 to -88.9)
		Staphylococcus aureus	330 (301 to 359)	836 (766 to 906)	154.1 (133.1 to 181.9)	24 (19.9 to 28.1)	46.1 (37.4 to 56.2)	92.8 (49.5 to 141.1)
		Streptococcus pneumoniae	1710 (1590 to 1840)	1080 (992 to 1160)	-37.1 (-41.3 to -31.9)	38 (31.8 to 44.2)	19.6 (16.1 to 23.8)	-48.0 (-58.9 to -35.5)
		Other viruses	946 (877 to 1020)	1340 (1240 to 1440)	42.0 (32.1 to 52.7)	7.41 (6.21 to 8.58)	8.84 (7.2 to 10.8)	20.1 (-5.4 to 48.9)
		Other bacterial pathogens	339 (298 to 383)	368 (317 to 424)	8.8 (-2.3 to 21.1)	10.6 (8.54 to 12.8)	8.55 (6.76 to 10.6)	-18.7 (-37.3 to 2.6)
	Under 5	*Acinetobacter baumannii*	1.06 (0.7 to 1.58)	0.34 (0.22 to 0.52)	-67.8 (-72.2 to -62.8)	0.03 (0.02 to 0.06)	0 (0 to 0.01)	-91.0 (-95.7 to -84.2)
		Chlamydia spp.	13.4 (11.1 to 16.2)	5.89 (4.73 to 7.27)	-56.1 (-62.5 to -49.1)	0.06 (0.04 to 0.09)	0 (0 to 0.01)	-92.5 (-96.2 to -88.0)
		Enterobacter spp.	1.79 (1.34 to 2.39)	2.09 (1.53 to 2.8)	17.1 (-1.8 to 39.5)	0.03 (0.02 to 0.05)	0 (0 to 0.01)	-83.0 (-91.3 to -72.4)
		Escherichia coli	5.47 (4.47 to 6.64)	6.05 (4.83 to 7.45)	10.7 (-6.1 to 28.0)	0.09 (0.06 to 0.14)	0.01 (0.01 to 0.02)	-84.7 (-92.2 to -74.9)
		Fungus	1.02 (0.7 to 1.43)	0.9 (0.63 to 1.28)	-11.4 (-27.1 to 6.0)	0.01 (0.01 to 0.02)	0 (0 to 0)	-87.8 (-94.1 to -79.6)
		Group B Streptococcus	5.09 (4.15 to 6.22)	4.89 (3.85 to 6.1)	-3.9 (-18.5 to 12.7)	0.07 (0.05 to 0.11)	0.01 (0.01 to 0.01)	-86.0 (-93.0 to -77.4)
		Haemophilus influenzae	45 (37.5 to 54)	29 (23.3 to 35.9)	-35.5 (-44.3 to -26.6)	0.23 (0.16 to 0.34)	0.01 (0.01 to 0.02)	-94.7 (-97.4 to -91.2)
		Influenza	36.6 (30.5 to 43.6)	1.82 (0.92 to 3.13)	-95.0 (-97.4 to -91.6)	0.35 (0.24 to 0.52)	0 (0 to 0)	-99.3 (-99.8 to -98.7)
		Klebsiella pneumoniae	19.6 (16.5 to 23.6)	9.78 (7.91 to 12.1)	-50.1 (-56.2 to -43.5)	0.26 (0.18 to 0.39)	0.02 (0.01 to 0.03)	-92.3 (-96.1 to -87.5)
		Legionella spp.	2.47 (1.76 to 3.38)	2.51 (1.68 to 3.58)	1.7 (-14.2 to 19.4)	0.04 (0.02 to 0.06)	0.01 (0 to 0.01)	-83.4 (-91.5 to -72.0)
		Mycoplasma spp.	25 (20.4 to 30.2)	27.9 (22.3 to 34.3)	11.7 (-3.1 to 28.2)	0.09 (0.06 to 0.13)	0.01 (0.01 to 0.02)	-85.1 (-92.6 to -75.5)

		Polymicrobial	1.07 (0.61 to 1.75)	0.62 (0.35 to 1.02)	-41.8 (-51.2 to -32.2)	0.04 (0.02 to 0.07)	0.01 (0 to 0.01)	-84.0 (-92.4 to -71.5)
		Pseudomonas aeruginosa	13.9 (11.5 to 16.8)	22.6 (18.2 to 27.9)	62.8 (40.8 to 87.6)	0.14 (0.1 to 0.22)	0.03 (0.02 to 0.04)	-78.5 (-89.1 to -64.9)
		Respiratory syncytial virus	105 (89 to 125)	8.31 (4.15 to 14.2)	-92.1 (-95.9 to -86.6)	0.5 (0.35 to 0.74)	0 (0 to 0.01)	-99.0 (-99.6 to -98.0)
		Staphylococcus aureus	15.8 (13.2 to 18.7)	20.8 (16.7 to 25.6)	31.7 (17.8 to 46.8)	0.29 (0.2 to 0.44)	0.05 (0.03 to 0.07)	-81.8 (-90.9 to -69.9)
		Streptococcus pneumoniae	233 (195 to 279)	52.9 (42.6 to 65.1)	-77.4 (-79.6 to -75.0)	1.63 (1.14 to 2.43)	0.05 (0.03 to 0.07)	-96.9 (-98.5 to -94.9)
		Other viruses	211 (177 to 252)	132 (106 to 162)	-37.7 (-43.5 to -31.0)	0.59 (0.42 to 0.88)	0.05 (0.04 to 0.07)	-90.9 (-95.5 to -85.1)
		Other bacterial pathogens	5.86 (4.47 to 7.48)	3.67 (2.73 to 4.88)	-37.4 (-46.4 to -26.7)	0.05 (0.03 to 0.08)	0 (0 to 0.01)	-90.5 (-95.1 to -84.5)
	5-14 years	*Acinetobacter baumannii*	1.64 (1.2 to 2.22)	0.63 (0.46 to 0.84)	-61.7 (-65.8 to -56.9)	0 (0 to 0.01)	0 (0 to 0)	-87.4 (-91.7 to -82.2)
		Chlamydia spp.	8.99 (6.64 to 11.9)	4.56 (3.33 to 6.13)	-49.2 (-54.5 to -43.2)	0.01 (0 to 0.01)	0 (0 to 0)	-83.3 (-88.9 to -76.1)
		Enterobacter spp.	1.04 (0.75 to 1.41)	1.52 (1.09 to 2.05)	47.0 (29.9 to 66.3)	0 (0 to 0)	0 (0 to 0)	-51.7 (-67.9 to -31.2)
		Escherichia coli	2.43 (1.78 to 3.22)	2.8 (2.04 to 3.72)	15.4 (4.3 to 28.4)	0.01 (0 to 0.01)	0 (0 to 0)	-62.1 (-74.9 to -45.7)
		Fungus	2.92 (2.14 to 3.89)	2.93 (2.15 to 3.89)	0.5 (-10.5 to 12.9)	0.01 (0.01 to 0.01)	0 (0 to 0)	-67.0 (-77.9 to -53.0)
		Group B Streptococcus	3.2 (2.3 to 4.24)	3.81 (2.81 to 5.07)	19.4 (6.0 to 34.7)	0.01 (0 to 0.01)	0 (0 to 0)	-60.8 (-74.0 to -44.5)
		Haemophilus influenzae	13.6 (10.2 to 18.1)	11.5 (8.46 to 15.2)	-15.1 (-22.3 to -6.8)	0.01 (0.01 to 0.02)	0 (0 to 0)	-81.5 (-87.7 to -73.9)
		Influenza	18.9 (14.2 to 25)	1.39 (0.68 to 2.52)	-92.6 (-96.1 to -87.6)	0.04 (0.03 to 0.05)	0 (0 to 0)	-97.7 (-98.9 to -95.7)
		Klebsiella pneumoniae	7.11 (5.39 to 9.47)	5.59 (4.13 to 7.3)	-21.4 (-27.6 to -13.6)	0.02 (0.01 to 0.02)	0 (0 to 0.01)	-74.2 (-82.8 to -63.7)
		Legionella spp.	7.64 (5.71 to 10.2)	9.86 (7.27 to 12.9)	29.3 (15.8 to 45.3)	0.01 (0.01 to 0.02)	0.01 (0 to 0.01)	-57.5 (-71.8 to -40.5)
		Mycoplasma spp.	29.5 (22 to 38.9)	22.9 (16.9 to 30.3)	-22.4 (-29.2 to -14.2)	0.02 (0.02 to 0.03)	0.01 (0 to 0.01)	-74.5 (-83.1 to -64.3)
		Polymicrobial	0.36 (0.22 to 0.57)	0.31 (0.17 to 0.53)	-14.5 (-27.2 to -0.4)	0 (0 to 0)	0 (0 to 0)	-71.9 (-81.4 to -59.5)
		Pseudomonas aeruginosa	8.13 (6.09 to 10.8)	12.1 (8.96 to 16)	49.2 (36.2 to 64.0)	0.01 (0.01 to 0.02)	0.01 (0.01 to 0.01)	-51.0 (-67.4 to -30.9)
		Respiratory syncytial virus	6.05 (4.53 to 8.04)	0.47 (0.23 to 0.86)	-92.2 (-96.0 to -86.9)	0.01 (0 to 0.01)	0 (0 to 0)	-97.5 (-98.8 to -95.5)
		Staphylococcus aureus	11.7 (8.82 to 15.4)	18.6 (13.7 to 24.3)	59.0 (47.0 to 74.4)	0.04 (0.03 to 0.05)	0.02 (0.02 to 0.03)	-47.7 (-65.1 to -26.8)
		Streptococcus pneumoniae	104 (79.4 to 138)	44.3 (32.7 to 57.9)	-57.6 (-60.8 to -53.5)	0.16 (0.12 to 0.2)	0.02 (0.02 to 0.03)	-86.1 (-90.7 to -80.5)
		Other viruses	57 (43.3 to 75.5)	57.8 (42.7 to 75.8)	1.3 (-6.1 to 11.4)	0.03 (0.03 to 0.04)	0.01 (0.01 to 0.01)	-66.7 (-77.7 to -53.4)

		Other bacterial pathogens	10.3 (7.67 to 13.8)	6.96 (5.06 to 9.33)	-32.7 (-39.0 to -25.7)	0.01 (0.01 to 0.02)	0 (0 to 0)	-77.8 (-85.3 to -68.7)
	15-49 years	*Acinetobacter baumannii*	5.72 (4.63 to 6.91)	3.29 (2.64 to 4.04)	-42.4 (-49.2 to -35.8)	0.04 (0.03 to 0.05)	0.01 (0.01 to 0.02)	-63.8 (-72.0 to -54.0)
		Chlamydia spp.	31.3 (26.5 to 36.9)	23.9 (19.9 to 28.4)	-23.7 (-31.6 to -15.2)	0.05 (0.04 to 0.06)	0.03 (0.02 to 0.03)	-52.0 (-62.9 to -39.0)
		Enterobacter spp.	3.61 (2.97 to 4.36)	7.96 (6.47 to 9.71)	121.1 (93.4 to 150.9)	0.02 (0.02 to 0.03)	0.03 (0.02 to 0.04)	39.0 (5.4 to 76.2)
		Escherichia coli	8.46 (7.18 to 9.96)	14.7 (12.3 to 17.4)	73.6 (54.5 to 93.8)	0.06 (0.05 to 0.07)	0.06 (0.05 to 0.07)	9.1 (-15.9 to 39.5)
		Fungus	10.2 (8.54 to 11.9)	15.3 (12.8 to 18.2)	51.1 (32.5 to 69.1)	0.07 (0.05 to 0.08)	0.06 (0.05 to 0.08)	-5.0 (-26.4 to 21.1)
		Group B Streptococcus	11.1 (9.39 to 13.3)	20 (16.6 to 23.8)	79.6 (57.9 to 102.6)	0.05 (0.04 to 0.06)	0.06 (0.05 to 0.07)	12.9 (-13.2 to 45.2)
		Haemophilus influenzae	47.4 (40.6 to 54.6)	60.4 (51.1 to 71.6)	27.6 (16.0 to 39.4)	0.12 (0.1 to 0.14)	0.06 (0.05 to 0.08)	-46.6 (-58.0 to -33.0)
		Influenza	65.8 (57 to 75.9)	7.3 (3.75 to 12.4)	-88.9 (-94.2 to -81.0)	0.32 (0.27 to 0.38)	0.02 (0.01 to 0.04)	-93.2 (-96.7 to -88.4)
		Klebsiella pneumoniae	24.8 (21.4 to 28.5)	29.3 (24.8 to 34.4)	18.3 (7.7 to 28.8)	0.16 (0.14 to 0.19)	0.12 (0.1 to 0.15)	-25.7 (-41.4 to -5.8)
		Legionella spp.	26.6 (22.6 to 31.1)	51.7 (43.5 to 60.9)	94.4 (71.7 to 117.3)	0.12 (0.1 to 0.14)	0.15 (0.12 to 0.18)	22.2 (-4.9 to 56.3)
		Mycoplasma spp.	103 (88.4 to 119)	120 (101 to 142)	16.7 (5.6 to 27.5)	0.18 (0.15 to 0.21)	0.13 (0.11 to 0.16)	-26.6 (-42.8 to -7.0)
		Polymicrobial	1.27 (0.76 to 2.01)	1.65 (0.9 to 2.82)	28.7 (10.0 to 50.6)	0.01 (0.01 to 0.01)	0.01 (0 to 0.01)	-19.2 (-38.9 to 3.7)
		Pseudomonas aeruginosa	28.3 (24.3 to 32.8)	63.5 (53.5 to 74.9)	124.4 (101.4 to 147.0)	0.13 (0.11 to 0.15)	0.18 (0.15 to 0.22)	41.1 (9.6 to 80.6)
		Respiratory syncytial virus	21.1 (18 to 24.5)	2.46 (1.27 to 4.3)	-88.3 (-94.0 to -79.6)	0.05 (0.04 to 0.05)	0 (0 to 0.01)	-92.9 (-96.5 to -87.8)
		Staphylococcus aureus	40.7 (35.3 to 46.8)	97.2 (82.7 to 114)	139.2 (118.6 to 159.4)	0.38 (0.32 to 0.44)	0.57 (0.47 to 0.7)	50.4 (18.7 to 89.7)
		Streptococcus pneumoniae	364 (316 to 417)	232 (197 to 272)	-36.2 (-41.5 to -31.3)	1.41 (1.19 to 1.63)	0.56 (0.46 to 0.7)	-59.9 (-68.2 to -49.6)
		Other viruses	199 (172 to 228)	303 (258 to 355)	52.3 (39.4 to 64.0)	0.3 (0.26 to 0.35)	0.29 (0.24 to 0.36)	-4.3 (-24.0 to 20.8)
		Other bacterial pathogens	36.1 (30 to 42.7)	36.5 (29.6 to 44.2)	1.2 (-8.7 to 12.3)	0.13 (0.11 to 0.16)	0.08 (0.07 to 0.11)	-36.3 (-50.4 to -18.8)
	50-69 years	*Acinetobacter baumannii*	19.4 (16.3 to 22.9)	10 (8.39 to 11.8)	-48.2 (-54.9 to -40.5)	0.26 (0.2 to 0.32)	0.1 (0.07 to 0.13)	-60.9 (-71.6 to -47.6)
		Chlamydia spp.	62.8 (54.2 to 73)	41.9 (36.5 to 47.8)	-33.1 (-41.3 to -23.0)	0.21 (0.16 to 0.25)	0.1 (0.08 to 0.13)	-49.6 (-63.0 to -32.3)
		Enterobacter spp.	11.9 (10 to 14)	23.2 (19.8 to 26.7)	96.1 (69.6 to 123.4)	0.15 (0.12 to 0.19)	0.22 (0.16 to 0.28)	47.8 (8.2 to 99.3)
		Escherichia coli	30.8 (26.6 to 35.1)	46.2 (40.9 to 52.1)	50.6 (31.8 to 70.8)	0.4 (0.32 to 0.49)	0.45 (0.35 to 0.57)	13.6 (-17.6 to 53.2)
		Fungus	29.4 (25 to 34.2)	39.4 (34.1 to 45)	34.5 (16.8 to 55.0)	0.38 (0.3 to 0.47)	0.38 (0.3 to 0.48)	1.4 (-26.6 to 35.9)
		Group B Streptococcus	25.9 (22.2 to 29.8)	40.7 (35.3 to 46.1)	57.5 (36.2 to 80.4)	0.24 (0.19 to 0.3)	0.28 (0.22 to 0.35)	18.8 (-13.1 to 64.1)
		Haemophilus influenzae	94.2 (82.4 to 107)	110 (97.2 to 123)	17.2 (4.0 to 31.7)	0.47 (0.38 to 0.57)	0.27 (0.21 to 0.34)	-41.3 (-56.7 to -21.8)

		Influenza	113 (98.6 to 126)	11.4 (5.89 to 20.2)	-89.9 (-94.7 to -82.3)	1.09 (0.88 to 1.33)	0.08 (0.04 to 0.14)	-92.6 (-96.3 to -86.6)
		Klebsiella pneumoniae	63.1 (55.3 to 70.9)	70.6 (62.8 to 78.6)	12.1 (0.4 to 26.6)	0.82 (0.65 to 0.99)	0.68 (0.53 to 0.85)	-15.5 (-38.2 to 12.7)
		Legionella spp.	74.4 (64.4 to 84.9)	127 (113 to 143)	71.8 (50.4 to 95.1)	0.66 (0.53 to 0.82)	0.85 (0.66 to 1.07)	29.6 (-6.0 to 73.2)
		Mycoplasma spp.	93.7 (82.3 to 106)	90.2 (79.7 to 102)	-3.6 (-14.3 to 8.7)	0.32 (0.26 to 0.4)	0.23 (0.18 to 0.29)	-27.3 (-46.9 to -2.1)
		Polymicrobial	3.59 (2.21 to 5.67)	4.16 (2.37 to 7.02)	15.1 (-3.1 to 36.1)	0.05 (0.03 to 0.08)	0.04 (0.02 to 0.07)	-13.2 (-38.3 to 18.9)
		Pseudomonas aeruginosa	79.8 (70 to 90)	153 (136 to 171)	92.8 (69.9 to 116.9)	0.72 (0.58 to 0.88)	1.03 (0.8 to 1.29)	45.3 (5.6 to 94.7)
		Respiratory syncytial virus	26.7 (23.1 to 30.5)	2.7 (1.39 to 4.74)	-89.9 (-94.9 to -82.1)	0.12 (0.1 to 0.15)	0.01 (0 to 0.02)	-92.6 (-96.3 to -86.6)
		Staphylococcus aureus	101 (88.3 to 113)	215 (190 to 240)	113.8 (91.4 to 139.1)	1.85 (1.49 to 2.26)	2.95 (2.3 to 3.68)	61.1 (17.7 to 115.5)
		Streptococcus pneumoniae	445 (390 to 498)	267 (237 to 298)	-39.8 (-45.7 to -33.0)	3.4 (2.74 to 4.16)	1.53 (1.18 to 1.91)	-54.6 (-66.6 to -39.6)
		Other viruses	213 (187 to 237)	307 (271 to 342)	44.5 (30.0 to 61.2)	0.64 (0.51 to 0.78)	0.69 (0.53 to 0.86)	8.9 (-19.8 to 46.4)
		Other bacterial pathogens	108 (92.8 to 125)	96.4 (81.3 to 111)	-10.8 (-21.7 to 0.5)	0.79 (0.62 to 0.99)	0.53 (0.4 to 0.68)	-32.6 (-50.7 to -10.0)
	70+ years	*Acinetobacter baumannii*	23.9 (19.7 to 28.6)	17.4 (14.8 to 20.3)	-26.7 (-36.3 to -14.4)	2.31 (1.8 to 2.9)	1.1 (0.86 to 1.38)	-51.7 (-63.8 to -38.0)
		Chlamydia spp.	92.2 (79.1 to 107)	87 (75.9 to 98.8)	-5.3 (-17.8 to 12.5)	2.21 (1.77 to 2.68)	1.37 (1.09 to 1.69)	-37.6 (-52.9 to -20.0)
		Enterobacter spp.	14.2 (11.9 to 16.9)	39.3 (33.8 to 44.9)	177.4 (135.2 to 224.6)	1.3 (1.03 to 1.62)	2.36 (1.86 to 2.89)	82.8 (37.4 to 134.6)
		Escherichia coli	46.5 (39.5 to 53.6)	98.8 (86.7 to 110)	113.1 (85.6 to 148.8)	4.41 (3.54 to 5.36)	6.13 (4.91 to 7.5)	40.4 (6.8 to 82.7)
		Fungus	20.2 (16.9 to 23.9)	38.3 (33 to 44.1)	90.3 (64.3 to 123.3)	1.89 (1.47 to 2.31)	2.34 (1.86 to 2.87)	25.4 (-5.2 to 61.4)
		Group B Streptococcus	29.3 (25.1 to 34.1)	65.1 (57.5 to 73.5)	122.9 (90.8 to 161.9)	1.97 (1.58 to 2.4)	2.87 (2.29 to 3.53)	46.8 (10.1 to 88.5)
		Haemophilus influenzae	105 (91 to 119)	173 (153 to 193)	65.8 (46.7 to 91.2)	3.77 (3.06 to 4.52)	2.72 (2.18 to 3.32)	-27.4 (-45.1 to -8.3)
		Influenza	211 (184 to 239)	30.2 (15.6 to 51.9)	-85.7 (-92.7 to -75.5)	14.9 (12.1 to 17.8)	1.35 (0.69 to 2.27)	-90.9 (-95.6 to -84.2)
		Klebsiella pneumoniae	85.6 (75 to 96.9)	135 (120 to 151)	58.6 (40.0 to 82.9)	8.06 (6.54 to 9.72)	8.35 (6.68 to 10.1)	4.5 (-20.7 to 32.4)
		Legionella spp.	65.9 (56.6 to 76.6)	160 (141 to 178)	143.1 (110.6 to 183.5)	4.27 (3.45 to 5.21)	6.77 (5.47 to 8.19)	60.2 (18.6 to 109.7)
		Mycoplasma spp.	97.6 (83.9 to 112)	133 (117 to 149)	36.5 (19.5 to 56.8)	2.44 (1.97 to 2.9)	2.18 (1.74 to 2.65)	-10.1 (-31.1 to 14.1)
		Polymicrobial	5.07 (3.14 to 8.2)	8.3 (4.66 to 14.4)	62.8 (34.7 to 95.7)	0.49 (0.29 to 0.8)	0.52 (0.28 to 0.92)	7.3 (-21.4 to 41.3)
		Pseudomonas aeruginosa	102 (88.9 to 117)	278 (245 to 310)	172.7 (140.9 to 212.8)	6.69 (5.39 to 7.96)	11.9 (9.58 to 14.5)	79.7 (37.8 to 130.1)
		Respiratory syncytial virus	30.9 (26.4 to 35.8)	4.41 (2.28 to 7.78)	-85.7 (-92.6 to -75.7)	0.99 (0.79 to 1.21)	0.09 (0.05 to 0.16)	-90.9 (-95.6 to -84.1)

		Staphylococcus aureus	161 (140 to 182)	485 (426 to 540)	202.5 (169.0 to 248.0)	21.5 (17.6 to 25.5)	42.5 (34.2 to 51.9)	99.2 (52.3 to 151.9)
		Streptococcus pneumoniae	564 (494 to 635)	479 (425 to 535)	-14.8 (-23.9 to -2.7)	31.4 (25.7 to 37.4)	17.5 (14.1 to 21.3)	-43.9 (-57.5 to -29.0)
		Other viruses	267 (234 to 301)	544 (479 to 608)	104.5 (80.8 to 134.3)	5.84 (4.78 to 6.97)	7.8 (6.3 to 9.55)	34.7 (2.5 to 71.2)
		Other bacterial pathogens	178 (151 to 208)	225 (189 to 263)	26.2 (10.7 to 45.1)	9.58 (7.65 to 11.7)	7.93 (6.19 to 9.95)	-16.7 (-37.0 to 6.4)
Denmark	All Ages	*Acinetobacter baumannii*	424 (365 to 490)	268 (231 to 306)	-36.8 (-43.6 to -28.9)	24.6 (20.9 to 28.4)	16 (13 to 18.6)	-34.8 (-42.3 to -27.3)
		Chlamydia spp.	1910 (1700 to 2100)	1550 (1410 to 1710)	-18.5 (-27.3 to -9.7)	27 (23.7 to 29.8)	22.8 (18.9 to 25.8)	-15.8 (-25.1 to -5.3)
		Enterobacter spp.	355 (309 to 403)	737 (657 to 817)	108.1 (84.2 to 132.6)	19.1 (16.2 to 22)	41 (33.5 to 46.3)	115.2 (88.2 to 142.6)
		Escherichia coli	986 (883 to 1090)	1800 (1650 to 1970)	83.2 (65.3 to 102.3)	58.4 (51.3 to 64.3)	109 (90.8 to 121)	87.2 (66.4 to 107.6)
		Fungus	595 (528 to 669)	870 (776 to 969)	46.7 (29.4 to 64.2)	27.7 (24.2 to 31.3)	42.4 (35.3 to 48.3)	53.4 (35.3 to 73.0)
		Group B Streptococcus	750 (672 to 836)	1270 (1150 to 1390)	69.2 (49.5 to 90.1)	28.2 (24.5 to 31.5)	49.5 (41.1 to 55.6)	75.6 (54.2 to 97.8)
		Haemophilus influenzae	3000 (2760 to 3230)	3620 (3330 to 3920)	20.9 (11.3 to 30.8)	57.1 (50.8 to 61.2)	49.2 (41.3 to 54.4)	-13.8 (-22.2 to -6.2)
		Influenza	4810 (4400 to 5200)	244 (41.6 to 683)	-94.9 (-99.1 to -85.7)	208 (185 to 224)	10.3 (1.75 to 29.2)	-95.1 (-99.2 to -86.2)
		Klebsiella pneumoniae	1970 (1810 to 2130)	2300 (2110 to 2510)	16.8 (7.2 to 26.0)	109 (96.7 to 117)	134 (111 to 149)	23.5 (12.5 to 34.4)
		Legionella spp.	2070 (1850 to 2280)	3230 (2970 to 3490)	56.4 (40.2 to 73.9)	72.3 (64 to 80.1)	117 (98.1 to 130)	62.7 (45.1 to 83.0)
		Mycoplasma spp.	3210 (2930 to 3480)	3110 (2870 to 3360)	-3.1 (-10.9 to 4.8)	37.7 (33.7 to 40.9)	36.9 (31.1 to 40.9)	-2.2 (-12.0 to 7.8)
		Polymicrobial	104 (62.7 to 169)	152 (85.9 to 258)	45.6 (27.1 to 66.0)	6.04 (3.63 to 10)	9.13 (5.12 to 15.6)	50.4 (29.0 to 71.6)
		Pseudomonas aeruginosa	2740 (2480 to 3000)	4860 (4470 to 5270)	77.8 (62.0 to 94.1)	105 (94 to 115)	192 (160 to 212)	82.1 (63.4 to 99.9)
		Respiratory syncytial virus	1550 (1410 to 1700)	54.5 (9.11 to 153)	-96.5 (-99.4 to -90.0)	19.1 (17.1 to 20.9)	0.63 (0.1 to 1.8)	-96.7 (-99.4 to -90.8)
		Staphylococcus aureus	3760 (3450 to 4050)	8540 (7850 to 9300)	127.5 (110.2 to 145.7)	311 (278 to 333)	729 (603 to 803)	134.7 (114.5 to 154.7)
		Streptococcus pneumoniae	15900 (14800 to 17100)	8210 (7620 to 8850)	-48.5 (-52.2 to -45.0)	457 (410 to 487)	258 (217 to 284)	-43.6 (-48.5 to -38.9)
		Other viruses	9470 (8790 to 10200)	10600 (9810 to 11300)	11.6 (4.1 to 18.8)	99.6 (89.4 to 106)	124 (105 to 137)	24.7 (14.3 to 35.2)
		Other bacterial pathogens	3610 (3170 to 4110)	3710 (3220 to 4270)	2.8 (-6.8 to 13.3)	124 (106 to 141)	130 (105 to 154)	5.3 (-5.5 to 16.5)
	Under 5	*Acinetobacter baumannii*	3.55 (2.36 to 5.14)	1.61 (1.04 to 2.43)	-54.8 (-60.7 to -48.5)	0.1 (0.06 to 0.17)	0.04 (0.02 to 0.07)	-63.1 (-72.4 to -52.1)
		Chlamydia spp.	52.8 (43.2 to 64)	30.1 (24.4 to 37.3)	-42.9 (-51.2 to -33.3)	0.19 (0.16 to 0.21)	0.05 (0.04 to 0.06)	-74.8 (-80.3 to -68.5)
		Enterobacter spp.	8.29 (6.15 to 10.9)	11.1 (8.19 to 14.8)	34.3 (13.1 to 59.0)	0.12 (0.09 to 0.16)	0.06 (0.04 to 0.08)	-52.6 (-64.1 to -40.0)

		Escherichia coli	23.7 (19.2 to 29)	33.4 (26.8 to 41.2)	41.7 (20.2 to 63.8)	0.33 (0.29 to 0.39)	0.15 (0.12 to 0.18)	-54.6 (-64.6 to -43.3)
		Fungus	4.53 (3.12 to 6.38)	4.72 (3.3 to 6.63)	4.6 (-14.2 to 24.4)	0.04 (0.03 to 0.06)	0.01 (0.01 to 0.02)	-70.9 (-78.7 to -61.3)
		Group B Streptococcus	22.2 (17.9 to 27.4)	25.5 (20.2 to 31.8)	15.0 (-3.5 to 33.2)	0.26 (0.23 to 0.3)	0.11 (0.09 to 0.14)	-57.2 (-66.4 to -47.0)
		Haemophilus influenzae	214 (175 to 259)	159 (129 to 198)	-25.5 (-35.0 to -14.8)	0.91 (0.8 to 1.04)	0.12 (0.09 to 0.14)	-87.1 (-90.3 to -83.4)
		Influenza	171 (140 to 207)	4.5 (0.77 to 12.2)	-97.4 (-99.5 to -92.7)	1.38 (1.21 to 1.55)	0.01 (0 to 0.03)	-99.3 (-99.9 to -98.0)
		Klebsiella pneumoniae	86.2 (70.9 to 104)	48.7 (39.3 to 60.1)	-43.5 (-50.2 to -35.6)	0.96 (0.85 to 1.08)	0.2 (0.16 to 0.24)	-79.3 (-83.7 to -74.5)
		Legionella spp.	12.1 (8.61 to 16.9)	12.9 (8.73 to 18.2)	6.3 (-8.6 to 24.1)	0.15 (0.11 to 0.18)	0.07 (0.05 to 0.09)	-52.8 (-62.9 to -40.7)
		Mycoplasma spp.	117 (94.4 to 142)	141 (114 to 175)	20.9 (4.5 to 38.0)	0.34 (0.29 to 0.38)	0.12 (0.09 to 0.14)	-64.9 (-73.3 to -55.3)
		Polymicrobial	4.13 (2.31 to 6.68)	3.11 (1.77 to 5.08)	-24.7 (-34.7 to -14.1)	0.16 (0.08 to 0.26)	0.09 (0.04 to 0.15)	-44.5 (-57.8 to -29.1)
		Pseudomonas aeruginosa	70.3 (57.5 to 85.4)	113 (91.6 to 139)	60.5 (39.7 to 84.4)	0.61 (0.53 to 0.69)	0.3 (0.24 to 0.36)	-50.6 (-61.6 to -38.2)
		Respiratory syncytial virus	562 (468 to 672)	17.8 (2.95 to 48.9)	-96.8 (-99.5 to -91.0)	2.22 (1.97 to 2.46)	0.02 (0 to 0.06)	-99.1 (-99.8 to -97.4)
		Staphylococcus aureus	76 (62.5 to 91.1)	111 (90.3 to 137)	46.3 (31.6 to 63.5)	1.19 (1.05 to 1.33)	0.52 (0.42 to 0.63)	-56.2 (-66.2 to -45.3)
		Streptococcus pneumoniae	1080 (896 to 1290)	236 (191 to 292)	-78.2 (-80.4 to -75.8)	6.43 (5.66 to 7.21)	0.38 (0.3 to 0.48)	-94.0 (-95.4 to -92.4)
		Other viruses	1120 (927 to 1340)	639 (523 to 786)	-43.0 (-48.1 to -37.0)	2.67 (2.36 to 2.99)	0.49 (0.39 to 0.59)	-81.7 (-85.7 to -77.0)
		Other bacterial pathogens	25.3 (19.2 to 32.9)	18.9 (14 to 24.9)	-25.2 (-36.1 to -13.0)	0.18 (0.14 to 0.23)	0.05 (0.03 to 0.07)	-72.8 (-79.5 to -65.5)
	5-14 years	*Acinetobacter baumannii*	6.15 (4.47 to 8.32)	3.19 (2.3 to 4.28)	-48.0 (-53.8 to -41.6)	0.03 (0.02 to 0.03)	0 (0 to 0.01)	-81.7 (-84.9 to -77.9)
		Chlamydia spp.	38.7 (28.3 to 51)	27.8 (20.4 to 36.7)	-28.2 (-35.8 to -19.7)	0.04 (0.04 to 0.05)	0.01 (0.01 to 0.01)	-74.7 (-79.1 to -69.4)
		Enterobacter spp.	5.29 (3.79 to 7.13)	9.42 (6.8 to 12.5)	78.4 (56.8 to 102.1)	0.02 (0.02 to 0.02)	0.01 (0.01 to 0.02)	-37.2 (-48.9 to -24.4)
		Escherichia coli	11.5 (8.41 to 15.1)	18.5 (13.6 to 24.2)	61.3 (44.8 to 78.7)	0.05 (0.04 to 0.05)	0.03 (0.02 to 0.03)	-43.3 (-53.1 to -31.9)
		Fungus	13.8 (10.1 to 18.3)	17.8 (13.2 to 23.6)	29.1 (14.0 to 45.0)	0.06 (0.05 to 0.07)	0.03 (0.02 to 0.03)	-54.6 (-62.6 to -44.2)
		Group B Streptococcus	15.7 (11.4 to 20.7)	23.6 (17.5 to 31.3)	50.5 (31.3 to 69.9)	0.05 (0.04 to 0.05)	0.02 (0.02 to 0.03)	-47.0 (-57.1 to -34.8)
		Haemophilus influenzae	69.6 (52.3 to 91.4)	69.7 (51.4 to 90.9)	0.2 (-8.2 to 10.1)	0.11 (0.1 to 0.12)	0.03 (0.02 to 0.03)	-76.4 (-80.2 to -71.6)
		Influenza	94.9 (71.4 to 123)	3.94 (0.59 to 11.2)	-95.9 (-99.3 to -88.3)	0.29 (0.26 to 0.33)	0 (0 to 0.01)	-98.7 (-99.8 to -96.1)
		Klebsiella pneumoniae	33.6 (25.1 to 43.7)	29.5 (22 to 38.5)	-12.0 (-19.1 to -4.3)	0.14 (0.12 to 0.15)	0.04 (0.04 to 0.05)	-69.0 (-74.2 to -63.1)

		Legionella spp.	43.4 (32.2 to 57.4)	59.4 (44.4 to 77.4)	37.2 (21.9 to 53.4)	0.12 (0.11 to 0.14)	0.06 (0.05 to 0.07)	-51.7 (-60.6 to -41.5)
		Mycoplasma spp.	149 (110 to 193)	145 (108 to 189)	-2.3 (-10.6 to 6.2)	0.16 (0.14 to 0.18)	0.06 (0.05 to 0.06)	-65.6 (-71.5 to -58.6)
		Polymicrobial	1.57 (0.89 to 2.57)	1.94 (1.04 to 3.22)	23.4 (8.1 to 40.3)	0.01 (0 to 0.01)	0 (0 to 0)	-56.6 (-64.8 to -46.2)
		Pseudomonas aeruginosa	44.9 (33.4 to 58.5)	72.3 (53.5 to 94.1)	61.1 (46.8 to 76.1)	0.13 (0.11 to 0.14)	0.07 (0.06 to 0.08)	-43.3 (-53.0 to -32.1)
		Respiratory syncytial virus	35 (26.2 to 45.8)	1.21 (0.19 to 3.49)	-96.6 (-99.4 to -90.3)	0.05 (0.04 to 0.06)	0 (0 to 0)	-98.9 (-99.8 to -96.9)
		Staphylococcus aureus	60.4 (45.2 to 78.7)	115 (85.4 to 148)	89.9 (76.6 to 104.3)	0.35 (0.32 to 0.39)	0.23 (0.2 to 0.27)	-33.2 (-43.8 to -20.4)
		Streptococcus pneumoniae	515 (386 to 672)	216 (162 to 281)	-57.9 (-60.7 to -54.9)	1.25 (1.12 to 1.39)	0.18 (0.16 to 0.21)	-85.2 (-87.6 to -82.3)
		Other viruses	323 (243 to 422)	298 (224 to 389)	-7.6 (-13.9 to -0.7)	0.31 (0.28 to 0.34)	0.1 (0.09 to 0.11)	-67.5 (-72.8 to -61.1)
		Other bacterial pathogens	48.2 (35.6 to 64.2)	42 (30.6 to 55.6)	-12.9 (-21.0 to -3.8)	0.11 (0.1 to 0.13)	0.03 (0.03 to 0.04)	-69.3 (-74.8 to -63.2)
	15-49 years	*Acinetobacter baumannii*	29.6 (23.7 to 36)	12.9 (10.5 to 15.5)	-56.5 (-61.5 to -51.0)	0.36 (0.31 to 0.42)	0.08 (0.07 to 0.1)	-77.2 (-80.6 to -73.5)
		Chlamydia spp.	186 (159 to 219)	112 (94.4 to 131)	-39.9 (-46.2 to -32.8)	0.57 (0.51 to 0.64)	0.18 (0.16 to 0.2)	-68.5 (-73.0 to -63.5)
		Enterobacter spp.	25.5 (21 to 30.4)	37.9 (31.2 to 45.4)	49.3 (31.9 to 68.7)	0.3 (0.26 to 0.34)	0.23 (0.2 to 0.27)	-21.9 (-33.4 to -9.3)
		Escherichia coli	55.4 (46.8 to 64.9)	74.6 (63.1 to 86.7)	35.0 (20.9 to 50.4)	0.67 (0.6 to 0.74)	0.47 (0.42 to 0.53)	-29.3 (-38.8 to -18.8)
		Fungus	66.4 (56 to 78.4)	71.6 (60.5 to 84.1)	8.1 (-5.3 to 20.9)	0.79 (0.69 to 0.9)	0.45 (0.38 to 0.51)	-43.4 (-52.1 to -33.2)
		Group B Streptococcus	75.6 (63.7 to 90)	95 (79.9 to 111)	26.0 (11.3 to 42.4)	0.65 (0.57 to 0.73)	0.42 (0.37 to 0.48)	-34.1 (-44.0 to -23.0)
		Haemophilus influenzae	335 (289 to 387)	281 (240 to 324)	-16.1 (-23.0 to -8.5)	1.54 (1.4 to 1.69)	0.45 (0.41 to 0.5)	-70.6 (-74.2 to -66.4)
		Influenza	457 (395 to 529)	15.7 (2.63 to 44.1)	-96.5 (-99.4 to -90.2)	4.11 (3.8 to 4.47)	0.07 (0.01 to 0.19)	-98.3 (-99.7 to -95.2)
		Klebsiella pneumoniae	162 (140 to 186)	119 (103 to 137)	-26.3 (-32.0 to -20.5)	1.94 (1.78 to 2.11)	0.75 (0.67 to 0.83)	-61.4 (-66.1 to -56.2)
		Legionella spp.	209 (178 to 243)	239 (206 to 276)	14.8 (3.1 to 28.0)	1.73 (1.54 to 1.93)	1.03 (0.92 to 1.15)	-39.9 (-48.5 to -30.8)
		Mycoplasma spp.	715 (619 to 822)	584 (502 to 673)	-18.2 (-25.3 to -10.8)	2.28 (2.08 to 2.51)	0.97 (0.88 to 1.08)	-57.2 (-62.6 to -51.0)
		Polymicrobial	7.57 (4.38 to 12.5)	7.85 (4.32 to 13.2)	3.3 (-9.7 to 16.7)	0.09 (0.06 to 0.15)	0.05 (0.03 to 0.08)	-45.9 (-54.6 to -36.3)
		Pseudomonas aeruginosa	216 (185 to 253)	291 (251 to 335)	34.9 (23.1 to 47.6)	1.8 (1.63 to 1.98)	1.27 (1.14 to 1.41)	-29.4 (-38.8 to -19.1)
		Respiratory syncytial virus	168 (144 to 195)	4.82 (0.84 to 13.6)	-97.1 (-99.5 to -91.8)	0.69 (0.62 to 0.76)	0.01 (0 to 0.03)	-98.6 (-99.8 to -96.1)
		Staphylococcus aureus	290 (252 to 336)	461 (398 to 533)	58.9 (48.1 to 71.0)	4.95 (4.56 to 5.35)	4.11 (3.71 to 4.55)	-16.8 (-26.5 to -5.9)

		Streptococcus pneumoniae	2480 (2160 to 2860)	872 (753 to 999)	-64.8 (-67.2 to -62.2)	17.6 (16.3 to 19)	3.23 (2.94 to 3.57)	-81.6 (-83.6 to -79.1)
		Other viruses	1560 (1360 to 1790)	1200 (1040 to 1370)	-22.7 (-27.9 to -17.5)	4.34 (4.03 to 4.7)	1.75 (1.59 to 1.94)	-59.5 (-64.2 to -54.0)
		Other bacterial pathogens	232 (192 to 277)	169 (140 to 203)	-27.1 (-34.3 to -19.2)	1.57 (1.36 to 1.79)	0.6 (0.51 to 0.7)	-61.8 (-67.0 to -55.9)
	50-69 years	*Acinetobacter baumannii*	116 (97.8 to 137)	64 (54.1 to 74.4)	-44.8 (-52.3 to -37.1)	2.25 (1.9 to 2.62)	1.1 (0.92 to 1.3)	-51.0 (-59.6 to -42.0)
		Chlamydia spp.	434 (378 to 497)	309 (273 to 347)	-28.5 (-37.9 to -19.6)	2.08 (1.84 to 2.36)	1.32 (1.13 to 1.52)	-36.5 (-46.8 to -24.2)
		Enterobacter spp.	97.2 (83.9 to 113)	177 (153 to 200)	82.8 (58.5 to 107.1)	1.78 (1.52 to 2.08)	2.88 (2.47 to 3.35)	62.4 (34.9 to 95.4)
		Escherichia coli	234 (204 to 265)	367 (328 to 405)	57.6 (39.0 to 76.6)	4.42 (3.94 to 5)	6.17 (5.42 to 7.05)	40.0 (17.6 to 65.9)
		Fungus	223 (193 to 256)	296 (258 to 337)	33.3 (15.8 to 51.2)	4.16 (3.6 to 4.77)	4.91 (4.22 to 5.73)	18.4 (-1.1 to 39.6)
		Group B Streptococcus	204 (177 to 235)	306 (267 to 345)	50.4 (30.0 to 71.3)	2.74 (2.41 to 3.14)	3.65 (3.1 to 4.27)	33.6 (10.4 to 59.0)
		Haemophilus influenzae	773 (686 to 858)	860 (767 to 953)	11.5 (-0.2 to 24.5)	5.58 (5.03 to 6.18)	3.69 (3.23 to 4.2)	-33.7 (-43.8 to -22.8)
		Influenza	907 (812 to 1010)	40.6 (6.82 to 115)	-95.5 (-99.2 to -87.6)	12.8 (11.6 to 14.1)	0.47 (0.08 to 1.35)	-96.3 (-99.3 to -89.2)
		Klebsiella pneumoniae	478 (429 to 532)	501 (448 to 553)	5.1 (-5.8 to 16.5)	8.98 (8.1 to 9.89)	8.37 (7.32 to 9.53)	-6.6 (-20.6 to 10.0)
		Legionella spp.	677 (593 to 767)	943 (843 to 1050)	39.7 (22.4 to 57.1)	8.77 (7.77 to 9.87)	10.8 (9.5 to 12.4)	24.1 (4.8 to 48.1)
		Mycoplasma spp.	756 (668 to 844)	648 (574 to 719)	-14.1 (-24.1 to -4.2)	3.78 (3.39 to 4.23)	2.87 (2.5 to 3.3)	-23.7 (-36.1 to -10.0)
		Polymicrobial	24.8 (15.1 to 40.6)	32 (17.8 to 54.1)	28.8 (9.7 to 49.1)	0.48 (0.28 to 0.76)	0.54 (0.31 to 0.92)	14.5 (-7.3 to 38.8)
		Pseudomonas aeruginosa	707 (622 to 796)	1090 (971 to 1210)	54.4 (36.8 to 72.3)	9.23 (8.27 to 10.3)	12.6 (11.1 to 14.4)	37.1 (15.1 to 60.4)
		Respiratory syncytial virus	248 (215 to 281)	8.26 (1.36 to 23)	-96.7 (-99.4 to -90.6)	1.59 (1.4 to 1.81)	0.04 (0.01 to 0.13)	-97.2 (-99.5 to -91.9)
		Staphylococcus aureus	835 (748 to 929)	1650 (1470 to 1820)	97.8 (76.9 to 119.6)	22.3 (20.3 to 24.8)	39.1 (34.3 to 44.8)	75.7 (49.5 to 105.9)
		Streptococcus pneumoniae	3520 (3150 to 3910)	1730 (1540 to 1910)	-50.9 (-55.9 to -45.7)	39.1 (35.5 to 43.2)	17 (15 to 19.4)	-56.4 (-62.8 to -48.8)
		Other viruses	1930 (1740 to 2150)	2130 (1890 to 2350)	10.3 (-1.0 to 21.7)	8.46 (7.66 to 9.32)	8.27 (7.25 to 9.46)	-2.0 (-16.7 to 15.1)
		Other bacterial pathogens	809 (693 to 933)	712 (605 to 820)	-11.8 (-22.3 to -0.6)	8.6 (7.38 to 9.95)	6.72 (5.67 to 8.04)	-21.6 (-34.5 to -7.8)
	70+ years	*Acinetobacter baumannii*	269 (226 to 315)	186 (159 to 217)	-30.6 (-38.5 to -21.3)	21.9 (18.3 to 25.4)	14.8 (11.8 to 17.3)	-32.2 (-40.2 to -24.2)
		Chlamydia spp.	1190 (1050 to 1340)	1070 (953 to 1210)	-10.1 (-20.6 to 1.4)	24.2 (20.9 to 26.8)	21.2 (17.3 to 24.1)	-12.2 (-22.2 to -0.7)
		Enterobacter spp.	219 (187 to 251)	501 (435 to 563)	129.9 (102.6 to 160.0)	16.9 (14.3 to 19.6)	37.9 (30.5 to 43)	124.6 (95.9 to 154.3)
		Escherichia coli	662 (583 to 739)	1310 (1180 to 1450)	98.2 (76.5 to 121.1)	52.9 (46.2 to 58.5)	102 (84.5 to 114)	93.6 (71.8 to 114.8)
		Fungus	287 (248 to 328)	480 (419 to 549)	67.7 (46.4 to 89.9)	22.6 (19.5 to 25.8)	37 (30.2 to 42.6)	63.9 (44.5 to 85.6)

		Group B Streptococcus	432 (379 to 485)	816 (727 to 918)	89.2 (63.9 to 115.4)	24.5 (21 to 27.5)	45.3 (37.4 to 51.3)	84.8 (61.6 to 109.2)
		Haemophilus influenzae	1610 (1440 to 1760)	2250 (2030 to 2520)	40.3 (26.3 to 54.3)	49 (43 to 52.9)	45 (37 to 49.9)	-8.2 (-17.3 to 0.5)
		Influenza	3180 (2860 to 3490)	179 (30.2 to 506)	-94.4 (-99.1 to -84.1)	190 (167 to 206)	9.75 (1.66 to 27.6)	-94.9 (-99.1 to -85.7)
		Klebsiella pneumoniae	1210 (1090 to 1330)	1600 (1450 to 1790)	32.3 (19.8 to 44.6)	96.5 (84.9 to 104)	125 (102 to 139)	29.2 (17.0 to 40.7)
		Legionella spp.	1120 (991 to 1260)	1970 (1780 to 2200)	75.7 (56.1 to 96.4)	61.5 (53.7 to 68.6)	105 (86.8 to 117)	71.6 (51.9 to 93.3)
		Mycoplasma spp.	1480 (1320 to 1640)	1590 (1430 to 1790)	8.0 (-3.0 to 19.1)	31.1 (27.2 to 34.1)	32.9 (27.2 to 36.7)	5.5 (-5.3 to 16.3)
		Polymicrobial	65.6 (38.5 to 108)	107 (59.5 to 185)	62.1 (39.9 to 85.7)	5.31 (3.12 to 8.88)	8.44 (4.71 to 14.4)	58.4 (35.8 to 81.2)
		Pseudomonas aeruginosa	1700 (1510 to 1870)	3290 (2980 to 3670)	94.2 (74.6 to 114.4)	93.7 (82.4 to 102)	178 (146 to 197)	89.7 (69.3 to 108.4)
		Respiratory syncytial virus	536 (475 to 603)	22.5 (3.72 to 63.9)	-95.8 (-99.3 to -88.2)	14.6 (12.7 to 16.3)	0.56 (0.09 to 1.6)	-96.2 (-99.4 to -89.4)
		Staphylococcus aureus	2500 (2250 to 2720)	6200 (5600 to 6930)	148.8 (126.6 to 171.6)	282 (249 to 304)	685 (560 to 758)	143.1 (121.5 to 164.2)
		Streptococcus pneumoniae	8350 (7550 to 9140)	5160 (4680 to 5750)	-38.2 (-43.7 to -32.7)	393 (347 to 422)	237 (196 to 261)	-39.6 (-45.3 to -34.5)
		Other viruses	4540 (4110 to 4950)	6290 (5670 to 7000)	38.8 (26.1 to 51.3)	83.9 (74.2 to 90.4)	114 (94.8 to 126)	35.6 (23.5 to 46.8)
		Other bacterial pathogens	2500 (2170 to 2860)	2770 (2370 to 3230)	10.9 (-0.7 to 23.1)	113 (96.2 to 130)	123 (97.6 to 145)	8.5 (-2.7 to 19.8)
Finland	All Ages	*Acinetobacter baumannii*	630 (548 to 719)	194 (169 to 222)	-69.2 (-71.8 to -66.2)	36.1 (30.6 to 41.5)	5.66 (4.44 to 6.65)	-84.3 (-86.1 to -82.6)
		Chlamydia spp.	2390 (2190 to 2590)	969 (891 to 1060)	-59.4 (-62.8 to -55.8)	33.5 (29.3 to 36.6)	6.8 (5.46 to 7.68)	-79.7 (-82.0 to -77.4)
		Enterobacter spp.	508 (450 to 568)	441 (395 to 493)	-13.0 (-22.0 to -3.5)	26.9 (23.2 to 30.6)	11.9 (9.44 to 13.7)	-55.8 (-61.2 to -50.5)
		Escherichia coli	1340 (1230 to 1470)	1010 (925 to 1100)	-25.0 (-31.2 to -18.0)	78.9 (69.3 to 86.6)	29.5 (23.7 to 33.2)	-62.6 (-66.8 to -58.7)
		Fungus	855 (762 to 943)	543 (490 to 598)	-36.4 (-42.2 to -29.9)	38.6 (33.9 to 43.3)	12.9 (10.6 to 14.8)	-66.6 (-70.3 to -62.7)
		Group B Streptococcus	1070 (974 to 1170)	786 (720 to 854)	-26.2 (-32.9 to -19.1)	39.3 (34.2 to 43.4)	14.7 (12 to 16.7)	-62.5 (-66.8 to -57.9)
		Haemophilus influenzae	3620 (3370 to 3850)	2180 (2020 to 2370)	-39.8 (-44.0 to -35.1)	47.6 (42.8 to 51.3)	14.7 (12.1 to 16.4)	-69.2 (-72.3 to -66.1)
		Influenza	6850 (6350 to 7290)	347 (101 to 781)	-94.9 (-98.5 to -88.5)	296 (262 to 319)	7.09 (2.07 to 16.6)	-97.6 (-99.3 to -94.5)
		Klebsiella pneumoniae	2700 (2500 to 2880)	1500 (1390 to 1630)	-44.2 (-48.1 to -40.0)	148 (132 to 159)	42.2 (34.2 to 47.5)	-71.4 (-74.5 to -68.6)
		Legionella spp.	2700 (2460 to 2920)	2080 (1920 to 2260)	-22.8 (-29.4 to -15.1)	92.3 (82.5 to 101)	36.7 (30 to 40.9)	-60.2 (-64.6 to -55.7)
		Mycoplasma spp.	4100 (3820 to 4390)	2230 (2060 to 2410)	-45.7 (-49.4 to -41.4)	46.7 (41.7 to 50.7)	12.3 (10.2 to 13.7)	-73.7 (-76.3 to -71.2)
		Polymicrobial	147 (89.3 to 239)	87.8 (50 to 150)	-40.6 (-46.9 to -33.6)	8.41 (5.05 to 13.7)	2.57 (1.46 to 4.41)	-69.7 (-73.6 to -65.6)

		Pseudomonas aeruginosa	3850 (3560 to 4150)	3260 (3020 to 3530)	-15.3 (-21.6 to -8.6)	146 (131 to 158)	62 (50.1 to 69.6)	-57.7 (-62.3 to -53.6)
		Respiratory syncytial virus	2340 (2150 to 2550)	112 (33 to 253)	-95.2 (-98.6 to -89.4)	26.3 (23.3 to 28.7)	0.58 (0.17 to 1.34)	-97.8 (-99.3 to -94.9)
		Staphylococcus aureus	5370 (4970 to 5720)	5060 (4680 to 5460)	-5.7 (-12.1 to 1.7)	442 (393 to 475)	208 (167 to 233)	-52.9 (-57.6 to -48.4)
		Streptococcus pneumoniae	19200 (17900 to 20300)	6440 (6000 to 6930)	-66.4 (-68.5 to -64.2)	542 (485 to 582)	95.3 (77.9 to 106)	-82.4 (-84.1 to -80.8)
		Other viruses	12000 (11300 to 12700)	7720 (7210 to 8310)	-35.7 (-39.6 to -31.6)	126 (113 to 135)	42.5 (35 to 47.4)	-66.3 (-69.5 to -63.1)
		Other bacterial pathogens	4530 (4020 to 5100)	2270 (1970 to 2630)	-49.8 (-53.7 to -45.5)	155 (133 to 176)	38.8 (30 to 46.1)	-75.0 (-77.9 to -72.3)
	Under 5	*Acinetobacter baumannii*	5.28 (3.49 to 7.84)	1.45 (0.98 to 2.17)	-72.6 (-76.2 to -69.1)	0.1 (0.06 to 0.15)	0.02 (0.01 to 0.04)	-79.4 (-84.0 to -74.3)
		Chlamydia spp.	76.8 (64 to 91.1)	26.4 (20.9 to 33.2)	-65.7 (-70.4 to -60.7)	0.16 (0.14 to 0.18)	0.03 (0.02 to 0.03)	-83.7 (-86.6 to -80.2)
		Enterobacter spp.	12.5 (9.33 to 16.2)	9.49 (6.86 to 12.8)	-24.2 (-35.5 to -12.3)	0.11 (0.08 to 0.15)	0.03 (0.02 to 0.05)	-69.9 (-75.6 to -63.1)
		Escherichia coli	36.3 (29.8 to 43.5)	26.5 (20.8 to 33.3)	-26.9 (-37.1 to -16.0)	0.3 (0.27 to 0.34)	0.08 (0.07 to 0.1)	-72.4 (-77.2 to -66.9)
		Fungus	6.6 (4.61 to 9.2)	4.11 (2.87 to 5.87)	-37.6 (-47.1 to -27.2)	0.04 (0.03 to 0.05)	0.01 (0.01 to 0.01)	-77.9 (-82.9 to -72.1)
		Group B Streptococcus	34.5 (28.1 to 42.5)	21.9 (17.2 to 27.7)	-36.3 (-45.4 to -27.5)	0.24 (0.21 to 0.27)	0.06 (0.05 to 0.08)	-74.0 (-78.5 to -68.6)
		Haemophilus influenzae	226 (187 to 271)	135 (107 to 169)	-40.1 (-48.3 to -31.8)	0.38 (0.34 to 0.42)	0.07 (0.06 to 0.09)	-80.4 (-84.6 to -75.7)
		Influenza	253 (209 to 305)	9.44 (2.82 to 21.2)	-96.3 (-98.9 to -91.9)	1.17 (1.04 to 1.31)	0.01 (0 to 0.03)	-98.7 (-99.6 to -97.1)
		Klebsiella pneumoniae	96.3 (80.1 to 117)	47.1 (37.5 to 58.9)	-51.1 (-57.3 to -44.7)	0.65 (0.58 to 0.73)	0.13 (0.1 to 0.15)	-80.7 (-84.2 to -76.7)
		Legionella spp.	16.8 (11.9 to 22.8)	11.5 (7.49 to 16.5)	-31.6 (-41.5 to -21.5)	0.13 (0.1 to 0.16)	0.04 (0.03 to 0.05)	-69.4 (-74.9 to -62.9)
		Mycoplasma spp.	186 (153 to 223)	124 (98.5 to 155)	-33.4 (-42.2 to -24.5)	0.3 (0.27 to 0.34)	0.07 (0.06 to 0.09)	-76.2 (-81.1 to -70.9)
		Polymicrobial	6.1 (3.4 to 10)	2.55 (1.41 to 4.25)	-58.2 (-64.7 to -52.0)	0.15 (0.08 to 0.24)	0.05 (0.02 to 0.08)	-68.7 (-75.5 to -60.8)
		Pseudomonas aeruginosa	110 (92.4 to 134)	104 (82.8 to 129)	-5.9 (-18.3 to 6.6)	0.55 (0.49 to 0.62)	0.19 (0.16 to 0.22)	-66.2 (-72.4 to -59.2)
		Respiratory syncytial virus	908 (760 to 1080)	44 (13.1 to 100)	-95.2 (-98.6 to -89.4)	2.06 (1.84 to 2.27)	0.03 (0.01 to 0.08)	-98.3 (-99.5 to -96.2)
		Staphylococcus aureus	114 (94.1 to 136)	95.4 (75.4 to 119)	-16.2 (-26.3 to -6.0)	1.03 (0.92 to 1.14)	0.31 (0.26 to 0.37)	-69.6 (-75.4 to -63.1)
		Streptococcus pneumoniae	1230 (1020 to 1470)	271 (214 to 340)	-78.0 (-80.7 to -75.4)	4.21 (3.71 to 4.72)	0.32 (0.27 to 0.39)	-92.3 (-93.9 to -90.5)
		Other viruses	1180 (981 to 1420)	661 (525 to 822)	-44.2 (-50.9 to -37.7)	1.65 (1.46 to 1.84)	0.34 (0.29 to 0.41)	-79.0 (-83.1 to -74.3)
		Other bacterial pathogens	34.4 (26.4 to 43.5)	16.7 (12.4 to 22.2)	-51.5 (-58.5 to -45.0)	0.15 (0.12 to 0.19)	0.03 (0.02 to 0.04)	-80.3 (-84.1 to -75.7)

	5-14 years	*Acinetobacter baumannii*	9.48 (6.9 to 12.8)	3.76 (2.74 to 5.02)	-60.3 (-64.5 to -55.8)	0.03 (0.03 to 0.04)	0.01 (0.01 to 0.01)	-77.9 (-81.3 to -73.9)
		Chlamydia spp.	50.7 (38 to 67.2)	28.4 (20.9 to 37.5)	-44.0 (-49.6 to -37.8)	0.04 (0.04 to 0.05)	0.01 (0.01 to 0.01)	-68.8 (-73.7 to -63.4)
		Enterobacter spp.	7.86 (5.69 to 10.7)	9.16 (6.55 to 12.1)	16.7 (4.2 to 30.1)	0.02 (0.02 to 0.03)	0.02 (0.01 to 0.02)	-35.1 (-45.7 to -23.6)
		Escherichia coli	16.5 (12.2 to 21.5)	17 (12.6 to 22.2)	3.2 (-7.0 to 14.4)	0.05 (0.05 to 0.06)	0.03 (0.03 to 0.04)	-42.6 (-51.9 to -32.4)
		Fungus	20 (14.9 to 26.7)	17.5 (13 to 23.4)	-12.4 (-21.5 to -2.8)	0.06 (0.06 to 0.07)	0.03 (0.03 to 0.04)	-51.2 (-59.0 to -42.1)
		Group B Streptococcus	23 (17 to 30.2)	23.5 (17.3 to 30.9)	2.2 (-8.7 to 13.7)	0.05 (0.05 to 0.06)	0.03 (0.03 to 0.03)	-43.1 (-52.5 to -32.5)
		Haemophilus influenzae	87.3 (66.7 to 114)	64 (47.3 to 83.7)	-26.6 (-33.8 to -19.5)	0.08 (0.07 to 0.08)	0.03 (0.03 to 0.04)	-59.4 (-65.7 to -52.1)
		Influenza	142 (108 to 185)	9.08 (2.72 to 20.8)	-93.6 (-98.1 to -85.6)	0.35 (0.31 to 0.38)	0.01 (0 to 0.03)	-96.6 (-99.1 to -92.2)
		Klebsiella pneumoniae	48.1 (36.5 to 62.3)	30.1 (22.4 to 38.8)	-37.3 (-42.3 to -32.0)	0.16 (0.14 to 0.17)	0.05 (0.05 to 0.06)	-65.1 (-70.4 to -59.1)
		Legionella spp.	57.9 (43.7 to 75.4)	61.1 (45.8 to 78.4)	5.6 (-4.9 to 16.3)	0.13 (0.12 to 0.14)	0.08 (0.07 to 0.09)	-41.2 (-51.0 to -30.6)
		Mycoplasma spp.	193 (145 to 250)	154 (116 to 201)	-19.7 (-26.3 to -12.5)	0.16 (0.15 to 0.18)	0.07 (0.06 to 0.08)	-55.3 (-62.2 to -47.2)
		Polymicrobial	2.32 (1.34 to 3.73)	1.83 (0.99 to 3.04)	-21.8 (-31.3 to -10.6)	0.01 (0 to 0.01)	0 (0 to 0.01)	-56.5 (-63.7 to -47.6)
		Pseudomonas aeruginosa	65.7 (49.8 to 85.2)	77.7 (58.3 to 100)	18.3 (8.0 to 29.5)	0.15 (0.13 to 0.16)	0.1 (0.08 to 0.11)	-34.2 (-44.2 to -23.1)
		Respiratory syncytial virus	52.4 (39.4 to 68.1)	3.44 (1.02 to 7.96)	-93.4 (-98.0 to -85.1)	0.06 (0.05 to 0.07)	0 (0 to 0)	-96.5 (-99.0 to -92.0)
		Staphylococcus aureus	90.6 (69 to 118)	111 (82.6 to 141)	22.1 (12.7 to 31.7)	0.42 (0.38 to 0.46)	0.28 (0.25 to 0.32)	-32.1 (-42.3 to -20.4)
		Streptococcus pneumoniae	642 (491 to 831)	267 (202 to 344)	-58.3 (-61.3 to -55.1)	1.23 (1.12 to 1.35)	0.28 (0.25 to 0.32)	-76.8 (-80.3 to -72.9)
		Other viruses	431 (329 to 559)	325 (246 to 418)	-24.7 (-30.4 to -19.2)	0.32 (0.29 to 0.36)	0.14 (0.12 to 0.16)	-58.1 (-64.4 to -50.9)
		Other bacterial pathogens	63.8 (47.3 to 84.7)	42.2 (30.9 to 55.6)	-33.8 (-39.7 to -27.7)	0.12 (0.1 to 0.13)	0.04 (0.04 to 0.05)	-63.1 (-69.0 to -56.8)
	15-49 years	*Acinetobacter baumannii*	46.4 (38.2 to 55.6)	13 (10.4 to 16.1)	-71.9 (-75.4 to -67.8)	0.64 (0.55 to 0.75)	0.09 (0.08 to 0.1)	-86.0 (-87.7 to -84.1)
		Chlamydia spp.	248 (217 to 282)	98.2 (81.6 to 116)	-60.3 (-65.2 to -55.1)	0.85 (0.77 to 0.94)	0.17 (0.15 to 0.19)	-80.2 (-82.5 to -77.7)
		Enterobacter spp.	38.4 (32.3 to 45.2)	31.7 (25.6 to 38.6)	-17.4 (-28.4 to -6.2)	0.5 (0.44 to 0.58)	0.21 (0.18 to 0.24)	-58.8 (-64.1 to -53.0)
		Escherichia coli	80.4 (70.1 to 91.4)	58.7 (49.1 to 69.5)	-27.0 (-36.2 to -17.4)	1.09 (0.99 to 1.2)	0.4 (0.36 to 0.44)	-63.6 (-67.8 to -58.8)
		Fungus	97.7 (83.8 to 113)	60.5 (50.5 to 71.9)	-38.0 (-45.8 to -29.2)	1.31 (1.17 to 1.46)	0.4 (0.36 to 0.45)	-69.1 (-72.7 to -65.1)
		Group B Streptococcus	112 (98 to 129)	81.3 (68 to 97)	-27.7 (-37.0 to -18.0)	1.09 (0.98 to 1.21)	0.39 (0.35 to 0.43)	-63.9 (-68.5 to -58.9)

		Haemophilus influenzae	427 (375 to 480)	222 (186 to 259)	-48.1 (-54.4 to -41.8)	1.55 (1.44 to 1.67)	0.4 (0.36 to 0.44)	-74.3 (-77.0 to -71.2)
		Influenza	696 (617 to 776)	31.5 (9.49 to 69.9)	-95.5 (-98.7 to -89.8)	7.06 (6.59 to 7.59)	0.15 (0.04 to 0.34)	-97.9 (-99.4 to -95.2)
		Klebsiella pneumoniae	235 (208 to 264)	104 (87.9 to 122)	-55.6 (-60.7 to -50.0)	3.18 (2.95 to 3.45)	0.7 (0.64 to 0.76)	-77.9 (-80.2 to -75.5)
		Legionella spp.	283 (249 to 320)	211 (178 to 247)	-25.2 (-34.8 to -14.9)	2.63 (2.41 to 2.87)	0.98 (0.9 to 1.07)	-62.7 (-67.1 to -58.0)
		Mycoplasma spp.	941 (834 to 1060)	535 (452 to 626)	-43.2 (-49.8 to -36.0)	3.38 (3.13 to 3.67)	0.96 (0.87 to 1.05)	-71.7 (-74.5 to -68.3)
		Polymicrobial	11.4 (6.71 to 18)	6.34 (3.4 to 10.8)	-44.6 (-52.3 to -35.9)	0.16 (0.09 to 0.25)	0.04 (0.02 to 0.07)	-72.4 (-76.2 to -68.0)
		Pseudomonas aeruginosa	321 (282 to 364)	269 (227 to 314)	-16.3 (-26.0 to -5.6)	3.02 (2.78 to 3.27)	1.26 (1.15 to 1.36)	-58.3 (-62.8 to -53.4)
		Respiratory syncytial virus	256 (224 to 289)	11.9 (3.64 to 26.5)	-95.3 (-98.6 to -89.6)	1.19 (1.09 to 1.3)	0.03 (0.01 to 0.06)	-97.8 (-99.4 to -95.1)
		Staphylococcus aureus	443 (394 to 494)	383 (326 to 446)	-13.6 (-22.6 to -4.1)	8.51 (7.95 to 9.12)	3.66 (3.4 to 3.95)	-56.9 (-61.0 to -52.4)
		Streptococcus pneumoniae	3140 (2800 to 3490)	926 (787 to 1080)	-70.5 (-73.5 to -67.3)	25.1 (23.5 to 26.9)	3.69 (3.43 to 3.97)	-85.3 (-86.8 to -83.8)
		Other viruses	2110 (1880 to 2350)	1120 (954 to 1310)	-46.7 (-52.2 to -40.8)	6.63 (6.2 to 7.1)	1.76 (1.63 to 1.9)	-73.4 (-76.0 to -70.7)
		Other bacterial pathogens	312 (263 to 364)	146 (120 to 177)	-53.1 (-58.7 to -46.9)	2.38 (2.09 to 2.69)	0.56 (0.48 to 0.64)	-76.6 (-79.2 to -73.8)
	50-69 years	*Acinetobacter baumannii*	203 (174 to 236)	49.7 (41.9 to 58.5)	-75.4 (-78.3 to -71.9)	3.82 (3.3 to 4.44)	0.57 (0.49 to 0.66)	-85.1 (-87.0 to -83.0)
		Chlamydia spp.	639 (572 to 713)	203 (177 to 229)	-68.2 (-72.2 to -63.6)	3 (2.69 to 3.32)	0.58 (0.52 to 0.65)	-80.7 (-83.3 to -77.9)
		Enterobacter spp.	163 (142 to 186)	113 (96.9 to 129)	-30.6 (-40.0 to -20.7)	2.91 (2.55 to 3.3)	1.22 (1.08 to 1.39)	-57.9 (-63.7 to -52.0)
		Escherichia coli	376 (337 to 419)	220 (195 to 246)	-41.4 (-48.8 to -33.6)	6.96 (6.3 to 7.67)	2.47 (2.24 to 2.73)	-64.4 (-69.0 to -59.8)
		Fungus	364 (317 to 411)	191 (167 to 217)	-47.4 (-54.3 to -40.0)	6.64 (5.94 to 7.48)	2.12 (1.88 to 2.38)	-68.1 (-72.3 to -63.8)
		Group B Streptococcus	337 (298 to 381)	198 (174 to 224)	-41.1 (-48.7 to -32.3)	4.43 (3.94 to 4.95)	1.58 (1.41 to 1.77)	-64.2 (-68.9 to -59.0)
		Haemophilus influenzae	1100 (997 to 1210)	535 (476 to 596)	-51.1 (-56.8 to -45.0)	5.41 (4.95 to 5.91)	1.59 (1.44 to 1.76)	-70.5 (-74.1 to -66.5)
		Influenza	1540 (1400 to 1690)	61.2 (17.7 to 139)	-96.0 (-98.9 to -91.0)	21.2 (19.5 to 23)	0.48 (0.14 to 1.12)	-97.7 (-99.3 to -94.8)
		Klebsiella pneumoniae	774 (706 to 856)	348 (311 to 390)	-55.0 (-60.2 to -49.1)	14.3 (13.1 to 15.6)	3.89 (3.53 to 4.3)	-72.7 (-76.1 to -69.2)
		Legionella spp.	1020 (912 to 1130)	636 (567 to 712)	-37.4 (-45.2 to -28.7)	12.9 (11.7 to 14.2)	4.89 (4.44 to 5.4)	-62.0 (-66.9 to -56.6)
		Mycoplasma spp.	1100 (995 to 1230)	449 (398 to 503)	-59.1 (-64.0 to -53.8)	5.38 (4.9 to 5.89)	1.33 (1.2 to 1.47)	-75.2 (-78.3 to -72.0)
		Polymicrobial	41.4 (25 to 66.9)	19.7 (11.1 to 33.4)	-52.5 (-59.2 to -45.5)	0.78 (0.46 to 1.24)	0.22 (0.13 to 0.38)	-71.2 (-75.5 to -66.6)

		Pseudomonas aeruginosa	1160 (1060 to 1290)	772 (689 to 859)	-33.6 (-41.4 to -25.5)	14.9 (13.6 to 16.2)	5.98 (5.44 to 6.6)	-59.7 (-64.6 to -54.6)
		Respiratory syncytial virus	418 (374 to 466)	15.5 (4.54 to 34.9)	-96.3 (-98.9 to -91.6)	2.63 (2.35 to 2.92)	0.06 (0.02 to 0.13)	-97.9 (-99.4 to -95.1)
		Staphylococcus aureus	1410 (1290 to 1560)	1040 (934 to 1160)	-26.1 (-34.7 to -17.2)	37 (34 to 40.3)	16.6 (15.1 to 18.3)	-55.1 (-60.4 to -49.6)
		Streptococcus pneumoniae	4960 (4520 to 5470)	1380 (1230 to 1530)	-72.2 (-75.4 to -68.8)	54 (49.9 to 58.5)	9.11 (8.31 to 10)	-83.1 (-85.1 to -81.0)
		Other viruses	2920 (2670 to 3210)	1570 (1390 to 1740)	-46.3 (-52.8 to -39.9)	12.5 (11.5 to 13.6)	4.07 (3.72 to 4.49)	-67.4 (-71.3 to -63.4)
		Other bacterial pathogens	1200 (1050 to 1380)	471 (398 to 555)	-60.9 (-65.5 to -55.7)	12.5 (10.9 to 14.2)	2.97 (2.56 to 3.44)	-76.2 (-79.2 to -73.2)
	70+ years	*Acinetobacter baumannii*	366 (314 to 427)	126 (108 to 146)	-65.5 (-68.7 to -61.8)	31.5 (26.3 to 36.3)	4.97 (3.84 to 5.9)	-84.2 (-86.1 to -82.4)
		Chlamydia spp.	1380 (1230 to 1520)	613 (551 to 689)	-55.4 (-59.6 to -50.6)	29.4 (25.4 to 32.4)	6.01 (4.72 to 6.85)	-79.6 (-82.0 to -77.1)
		Enterobacter spp.	287 (249 to 327)	278 (243 to 318)	-2.7 (-13.0 to 9.3)	23.4 (19.8 to 26.8)	10.4 (8.02 to 12.2)	-55.5 (-61.2 to -50.1)
		Escherichia coli	835 (749 to 924)	685 (621 to 762)	-17.9 (-25.5 to -8.7)	70.5 (61.2 to 77.7)	26.5 (20.9 to 30.1)	-62.4 (-67.0 to -58.2)
		Fungus	367 (322 to 411)	270 (238 to 306)	-26.2 (-33.9 to -17.6)	30.5 (26.1 to 34.7)	10.3 (8.07 to 12)	-66.2 (-70.2 to -62.1)
		Group B Streptococcus	559 (498 to 623)	461 (415 to 513)	-17.4 (-25.5 to -8.4)	33.5 (28.6 to 37.3)	12.7 (9.95 to 14.5)	-62.2 (-66.8 to -57.3)
		Haemophilus influenzae	1790 (1630 to 1950)	1220 (1110 to 1360)	-31.4 (-37.4 to -24.5)	40.2 (35.5 to 43.7)	12.6 (10.1 to 14.3)	-68.8 (-72.1 to -65.5)
		Influenza	4220 (3850 to 4590)	236 (69.8 to 530)	-94.4 (-98.3 to -87.2)	266 (232 to 289)	6.43 (1.83 to 15)	-97.6 (-99.3 to -94.5)
		Klebsiella pneumoniae	1540 (1410 to 1680)	974 (883 to 1080)	-36.8 (-42.1 to -30.7)	130 (113 to 140)	37.5 (29.9 to 42.4)	-71.1 (-74.4 to -68.1)
		Legionella spp.	1320 (1180 to 1460)	1160 (1060 to 1290)	-12.2 (-20.5 to -2.2)	76.5 (66.9 to 84.8)	30.8 (24.3 to 34.7)	-59.8 (-64.6 to -55.0)
		Mycoplasma spp.	1680 (1530 to 1840)	964 (880 to 1070)	-42.7 (-47.5 to -36.4)	37.5 (32.7 to 41.1)	9.85 (7.87 to 11.2)	-73.8 (-76.7 to -71.0)
		Polymicrobial	85.7 (50.3 to 141)	57.4 (31.5 to 99.5)	-33.4 (-41.3 to -24.9)	7.33 (4.39 to 12)	2.25 (1.23 to 3.93)	-69.5 (-73.7 to -65.3)
		Pseudomonas aeruginosa	2190 (1990 to 2400)	2040 (1850 to 2250)	-6.9 (-14.5 to 2.5)	128 (113 to 139)	54.5 (42.8 to 61.8)	-57.4 (-62.5 to -52.9)
		Respiratory syncytial virus	708 (635 to 792)	36.9 (11.1 to 83.5)	-94.8 (-98.5 to -88.1)	20.4 (17.6 to 22.6)	0.46 (0.13 to 1.08)	-97.7 (-99.3 to -94.8)
		Staphylococcus aureus	3310 (3010 to 3590)	3430 (3120 to 3770)	3.7 (-4.5 to 13.8)	395 (345 to 426)	187 (147 to 211)	-52.6 (-57.6 to -47.9)
		Streptococcus pneumoniae	9220 (8430 to 9970)	3600 (3280 to 3970)	-61.0 (-64.0 to -57.1)	458 (402 to 495)	81.9 (65.5 to 92.4)	-82.1 (-84.0 to -80.3)
		Other viruses	5370 (4890 to 5820)	4040 (3680 to 4470)	-24.7 (-30.5 to -17.4)	105 (91.9 to 114)	36.2 (29 to 40.9)	-65.5 (-69.1 to -62.1)
		Other bacterial pathogens	2920 (2560 to 3310)	1600 (1370 to 1870)	-45.2 (-50.2 to -40.0)	140 (118 to 159)	35.2 (26.8 to 42.1)	-74.9 (-77.8 to -72.1)

France	All Ages	*Acinetobacter baumannii*	4140 (3570 to 4800)	2600 (2240 to 3000)	-37.0 (-44.4 to -29.8)	256 (217 to 297)	131 (102 to 153)	-48.8 (-55.5 to -42.1)
		Chlamydia spp.	18400 (16700 to 20400)	15000 (13500 to 16600)	-18.7 (-27.8 to -8.7)	278 (243 to 308)	184 (149 to 210)	-33.8 (-42.6 to -24.8)
		Enterobacter spp.	3400 (2980 to 3860)	7430 (6580 to 8250)	119.2 (92.6 to 144.6)	194 (165 to 222)	346 (275 to 395)	79.0 (54.4 to 102.9)
		Escherichia coli	9400 (8510 to 10400)	17800 (16100 to 19500)	89.3 (68.5 to 110.5)	597 (522 to 661)	902 (730 to 1010)	51.3 (31.2 to 68.6)
		Fungus	5860 (5190 to 6630)	8640 (7720 to 9620)	47.7 (30.3 to 66.6)	281 (246 to 317)	350 (282 to 404)	24.6 (8.7 to 40.5)
		Group B Streptococcus	7250 (6550 to 8100)	12700 (11400 to 13900)	75.4 (54.3 to 96.8)	287 (250 to 322)	415 (333 to 471)	44.6 (25.0 to 65.8)
		Haemophilus influenzae	29200 (27000 to 31500)	35700 (32800 to 38800)	22.6 (11.4 to 34.4)	578 (515 to 621)	407 (335 to 455)	-29.6 (-37.3 to -22.3)
		Influenza	45800 (42700 to 49400)	7910 (3030 to 16400)	-82.7 (-93.5 to -64.1)	2120 (1890 to 2280)	295 (105 to 614)	-86.1 (-95.0 to -71.1)
		Klebsiella pneumoniae	19000 (17700 to 20700)	22900 (21000 to 25100)	20.7 (9.7 to 31.9)	1110 (981 to 1190)	1120 (906 to 1250)	1.0 (-10.2 to 10.8)
		Legionella spp.	19900 (17900 to 22100)	32800 (30100 to 35900)	65.5 (47.2 to 85.2)	726 (642 to 800)	996 (815 to 1120)	37.4 (20.1 to 53.8)
		Mycoplasma spp.	31700 (29100 to 34300)	31300 (28700 to 33900)	-1.0 (-9.8 to 7.6)	381 (338 to 414)	307 (250 to 344)	-19.3 (-28.4 to -10.4)
		Polymicrobial	997 (600 to 1620)	1480 (832 to 2490)	47.5 (27.1 to 70.0)	61.9 (37 to 103)	74.7 (41.1 to 127)	19.9 (1.1 to 38.9)
		Pseudomonas aeruginosa	26000 (23800 to 28600)	50000 (45900 to 54400)	92.4 (73.3 to 112.1)	1060 (951 to 1160)	1650 (1330 to 1850)	55.4 (37.4 to 72.6)
		Respiratory syncytial virus	15600 (14200 to 17200)	1860 (705 to 3860)	-88.0 (-95.5 to -75.5)	189 (170 to 207)	19 (6.7 to 39.6)	-89.9 (-96.4 to -79.0)
		Staphylococcus aureus	35600 (33200 to 38500)	86400 (79200 to 94200)	142.8 (120.8 to 165.3)	3150 (2800 to 3380)	6190 (4990 to 6900)	96.1 (74.9 to 114.1)
		Streptococcus pneumoniae	156000 (145000 to 167000)	82000 (75700 to 88900)	-47.4 (-51.5 to -43.0)	4630 (4160 to 4950)	2150 (1750 to 2400)	-53.6 (-58.7 to -49.4)
		Other viruses	92500 (86400 to 99300)	109000 (101000 to 118000)	17.5 (8.4 to 26.5)	998 (897 to 1060)	1070 (877 to 1190)	7.0 (-4.7 to 16.8)
		Other bacterial pathogens	34300 (30100 to 38900)	36300 (31300 to 41700)	5.8 (-5.4 to 18.3)	1270 (1080 to 1440)	1070 (841 to 1280)	-15.6 (-26.0 to -5.8)
	Under 5	*Acinetobacter baumannii*	36.7 (24.8 to 52.8)	15.6 (9.96 to 23.7)	-57.6 (-64.5 to -50.0)	0.94 (0.53 to 1.58)	0.34 (0.17 to 0.64)	-63.6 (-73.6 to -52.2)
		Chlamydia spp.	580 (465 to 708)	298 (237 to 378)	-48.5 (-56.3 to -40.4)	1.67 (1.49 to 1.86)	0.45 (0.36 to 0.55)	-72.9 (-78.6 to -66.8)
		Enterobacter spp.	89.5 (65.2 to 120)	115 (83.1 to 155)	29.0 (7.2 to 53.6)	1.07 (0.79 to 1.44)	0.58 (0.41 to 0.82)	-45.4 (-56.9 to -32.7)
		Escherichia coli	258 (205 to 323)	339 (263 to 427)	31.6 (10.2 to 53.2)	2.95 (2.6 to 3.34)	1.53 (1.26 to 1.85)	-48.0 (-58.0 to -37.0)
		Fungus	50.3 (34.7 to 70.8)	48.3 (33.2 to 67.9)	-3.5 (-20.2 to 16.4)	0.38 (0.28 to 0.51)	0.14 (0.1 to 0.19)	-63.0 (-71.5 to -53.7)
		Group B Streptococcus	239 (189 to 301)	261 (203 to 332)	9.6 (-7.9 to 27.6)	2.32 (2.06 to 2.64)	1.1 (0.87 to 1.35)	-52.5 (-62.6 to -41.8)

		Haemophilus influenzae	2350 (1920 to 2930)	1620 (1290 to 2030)	-31.1 (-40.6 to -21.0)	8 (7.17 to 8.88)	1.28 (1.06 to 1.51)	-83.9 (-87.0 to -80.6)
		Influenza	1890 (1540 to 2310)	150 (56.7 to 305)	-92.0 (-97.0 to -83.9)	12.1 (11.1 to 13.2)	0.37 (0.14 to 0.78)	-97.0 (-98.9 to -93.7)
		Klebsiella pneumoniae	951 (771 to 1170)	501 (395 to 627)	-47.3 (-53.9 to -40.4)	8.52 (7.73 to 9.33)	2.07 (1.72 to 2.45)	-75.7 (-80.3 to -70.7)
		Legionella spp.	129 (89.3 to 183)	134 (90.3 to 191)	4.2 (-11.7 to 21.5)	1.3 (1.01 to 1.63)	0.68 (0.5 to 0.87)	-47.6 (-58.8 to -34.7)
		Mycoplasma spp.	1280 (1040 to 1590)	1430 (1140 to 1800)	11.8 (-4.1 to 27.1)	2.94 (2.64 to 3.29)	1.26 (1.05 to 1.49)	-57.0 (-65.5 to -47.4)
		Polymicrobial	40.5 (23.4 to 65.5)	30.7 (17 to 49.7)	-24.3 (-35.3 to -11.5)	1.39 (0.73 to 2.32)	0.8 (0.36 to 1.4)	-43.2 (-58.2 to -25.2)
		Pseudomonas aeruginosa	763 (621 to 951)	1190 (941 to 1480)	56.1 (35.4 to 78.6)	5.35 (4.81 to 5.94)	3.23 (2.66 to 3.82)	-39.4 (-50.8 to -26.9)
		Respiratory syncytial virus	6100 (5020 to 7420)	615 (229 to 1250)	-89.9 (-96.1 to -79.3)	19.3 (17.7 to 21)	0.77 (0.28 to 1.61)	-96.0 (-98.6 to -91.8)
		Staphylococcus aureus	834 (680 to 1010)	1160 (921 to 1440)	39.3 (25.1 to 55.0)	10.4 (9.56 to 11.4)	5.7 (4.73 to 6.67)	-45.3 (-55.5 to -34.6)
		Streptococcus pneumoniae	12000 (9790 to 14700)	2440 (1940 to 3060)	-79.6 (-81.7 to -77.6)	56.4 (51.9 to 61.3)	4.34 (3.59 to 5.09)	-92.3 (-93.8 to -90.8)
		Other viruses	12200 (10000 to 15000)	6770 (5420 to 8480)	-44.7 (-50.0 to -39.4)	23.2 (21.3 to 25.3)	5.43 (4.56 to 6.38)	-76.5 (-80.7 to -72.1)
		Other bacterial pathogens	280 (211 to 367)	191 (139 to 253)	-31.6 (-42.1 to -20.4)	1.62 (1.28 to 2.05)	0.5 (0.37 to 0.7)	-68.9 (-75.2 to -61.2)
	5-14 years	*Acinetobacter baumannii*	81.7 (59.2 to 111)	36.7 (26.7 to 49.3)	-54.9 (-60.3 to -49.5)	0.24 (0.2 to 0.28)	0.05 (0.04 to 0.06)	-80.0 (-83.5 to -76.5)
		Chlamydia spp.	510 (374 to 670)	318 (232 to 421)	-37.5 (-44.0 to -29.9)	0.37 (0.33 to 0.41)	0.1 (0.09 to 0.12)	-72.3 (-76.5 to -67.7)
		Enterobacter spp.	68.7 (49.2 to 93.1)	113 (80.7 to 151)	64.2 (45.5 to 85.7)	0.19 (0.16 to 0.22)	0.14 (0.12 to 0.16)	-27.2 (-38.7 to -14.5)
		Escherichia coli	150 (110 to 198)	217 (159 to 285)	44.7 (29.6 to 60.7)	0.43 (0.38 to 0.49)	0.28 (0.24 to 0.31)	-35.9 (-46.0 to -25.4)
		Fungus	180 (132 to 242)	209 (153 to 278)	16.1 (2.6 to 30.7)	0.51 (0.45 to 0.58)	0.26 (0.22 to 0.3)	-48.5 (-56.6 to -39.1)
		Group B Streptococcus	205 (147 to 271)	280 (207 to 372)	37.3 (19.8 to 54.9)	0.42 (0.36 to 0.48)	0.25 (0.22 to 0.29)	-39.1 (-49.4 to -28.2)
		Haemophilus influenzae	904 (680 to 1180)	807 (598 to 1060)	-10.6 (-18.1 to -2.2)	0.99 (0.9 to 1.09)	0.26 (0.23 to 0.3)	-73.4 (-77.1 to -69.1)
		Influenza	1230 (926 to 1600)	150 (52.4 to 325)	-87.9 (-95.5 to -75.1)	2.65 (2.43 to 2.91)	0.14 (0.05 to 0.29)	-94.7 (-98.0 to -88.7)
		Klebsiella pneumoniae	439 (327 to 571)	345 (259 to 447)	-21.4 (-26.8 to -15.0)	1.26 (1.15 to 1.38)	0.44 (0.39 to 0.49)	-65.1 (-70.0 to -60.1)
		Legionella spp.	559 (418 to 734)	716 (539 to 926)	28.4 (14.4 to 43.0)	1.1 (0.99 to 1.24)	0.62 (0.55 to 0.7)	-43.1 (-52.1 to -34.0)
		Mycoplasma spp.	1930 (1410 to 2510)	1730 (1300 to 2260)	-10.6 (-18.1 to -2.3)	1.47 (1.33 to 1.62)	0.58 (0.52 to 0.65)	-60.4 (-66.3 to -54.2)
		Polymicrobial	20.6 (11.9 to 33.6)	22.4 (12.1 to 37.5)	8.2 (-6.0 to 25.0)	0.06 (0.04 to 0.1)	0.03 (0.02 to 0.05)	-52.0 (-60.5 to -41.9)

		Pseudomonas aeruginosa	580 (431 to 757)	885 (660 to 1150)	52.8 (39.9 to 66.9)	1.15 (1.04 to 1.28)	0.78 (0.69 to 0.87)	-32.3 (-41.9 to -22.1)
		Respiratory syncytial virus	450 (336 to 591)	48.2 (17 to 104)	-89.3 (-96.1 to -77.9)	0.44 (0.39 to 0.49)	0.02 (0.01 to 0.04)	-95.3 (-98.3 to -90.2)
		Staphylococcus aureus	783 (584 to 1010)	1370 (1030 to 1770)	75.2 (64.5 to 88.5)	3.19 (2.93 to 3.49)	2.47 (2.2 to 2.75)	-22.3 (-32.8 to -11.5)
		Streptococcus pneumoniae	6700 (5040 to 8710)	2530 (1910 to 3280)	-62.3 (-64.5 to -59.5)	11.4 (10.4 to 12.4)	1.89 (1.69 to 2.11)	-83.3 (-85.5 to -81.0)
		Other viruses	4170 (3120 to 5410)	3580 (2680 to 4650)	-14.1 (-19.0 to -7.4)	2.77 (2.55 to 3.04)	1.05 (0.94 to 1.18)	-61.9 (-67.1 to -56.7)
		Other bacterial pathogens	631 (464 to 838)	487 (354 to 650)	-22.7 (-29.8 to -15.0)	1.02 (0.88 to 1.16)	0.35 (0.29 to 0.41)	-65.7 (-71.0 to -60.3)
	15-49 years	*Acinetobacter baumannii*	306 (246 to 371)	129 (104 to 157)	-57.7 (-62.9 to -52.1)	3.44 (2.9 to 4.02)	0.77 (0.65 to 0.89)	-77.6 (-80.6 to -74.2)
		Chlamydia spp.	1910 (1610 to 2210)	1120 (936 to 1320)	-41.4 (-47.9 to -33.6)	5.32 (4.77 to 5.93)	1.65 (1.47 to 1.87)	-69.0 (-73.0 to -64.2)
		Enterobacter spp.	257 (211 to 306)	395 (323 to 473)	54.0 (35.4 to 74.2)	2.74 (2.36 to 3.15)	2.22 (1.94 to 2.54)	-18.5 (-29.9 to -6.0)
		Escherichia coli	562 (467 to 648)	762 (646 to 897)	35.7 (20.4 to 52.3)	6.2 (5.53 to 6.9)	4.44 (4.04 to 4.93)	-28.2 (-37.3 to -17.8)
		Fungus	675 (565 to 787)	734 (611 to 867)	8.9 (-4.3 to 23.6)	7.33 (6.45 to 8.23)	4.21 (3.69 to 4.75)	-42.4 (-50.5 to -33.9)
		Group B Streptococcus	765 (641 to 894)	983 (831 to 1170)	28.8 (12.0 to 46.4)	5.99 (5.3 to 6.75)	4.07 (3.61 to 4.58)	-31.9 (-41.3 to -21.3)
		Haemophilus influenzae	3380 (2900 to 3840)	2830 (2390 to 3330)	-16.1 (-24.1 to -8.1)	14.2 (13.1 to 15.5)	4.23 (3.86 to 4.68)	-70.2 (-73.7 to -66.3)
		Influenza	4620 (3950 to 5240)	523 (199 to 1100)	-88.6 (-95.7 to -76.6)	38 (35 to 41)	2.25 (0.81 to 4.67)	-94.1 (-97.9 to -87.6)
		Klebsiella pneumoniae	1640 (1410 to 1860)	1210 (1020 to 1410)	-26.2 (-32.9 to -18.8)	18 (16.6 to 19.5)	7.01 (6.38 to 7.69)	-61.0 (-65.3 to -56.1)
		Legionella spp.	2090 (1780 to 2410)	2510 (2130 to 2910)	20.4 (6.6 to 36.1)	15.8 (14.1 to 17.5)	10 (9.14 to 11)	-36.3 (-45.2 to -26.9)
		Mycoplasma spp.	7230 (6190 to 8210)	6060 (5150 to 7050)	-16.1 (-23.9 to -7.6)	21 (19.3 to 22.8)	9.31 (8.49 to 10.2)	-55.6 (-61.1 to -50.2)
		Polymicrobial	77.4 (45.1 to 126)	78.9 (43.4 to 134)	1.5 (-12.5 to 17.9)	0.86 (0.51 to 1.4)	0.47 (0.26 to 0.8)	-46.3 (-54.6 to -36.9)
		Pseudomonas aeruginosa	2170 (1850 to 2490)	3110 (2630 to 3600)	43.4 (29.5 to 58.5)	16.5 (15 to 18)	12.5 (11.4 to 13.7)	-24.1 (-33.2 to -14.8)
		Respiratory syncytial virus	1680 (1430 to 1930)	169 (63.6 to 355)	-90.0 (-96.2 to -79.4)	6.31 (5.71 to 7)	0.33 (0.12 to 0.69)	-94.8 (-98.2 to -89.0)
		Staphylococcus aureus	2930 (2540 to 3300)	4810 (4100 to 5590)	64.4 (51.4 to 79.3)	45.6 (42.2 to 48.9)	39.6 (36.5 to 43.3)	-13.0 (-22.1 to -3.4)
		Streptococcus pneumoniae	25100 (21700 to 28300)	8870 (7550 to 10300)	-64.6 (-67.5 to -61.5)	162 (151 to 174)	30.4 (28 to 33.1)	-81.3 (-83.2 to -79.2)
		Other viruses	15600 (13500 to 17600)	12600 (10700 to 14500)	-19.4 (-26.1 to -12.8)	39.7 (36.9 to 42.8)	16.9 (15.5 to 18.4)	-57.4 (-61.8 to -52.8)
		Other bacterial pathogens	2360 (1950 to 2770)	1710 (1390 to 2070)	-27.5 (-35.3 to -19.5)	14.6 (12.6 to 16.6)	5.6 (4.8 to 6.49)	-61.6 (-66.2 to -56.4)

	50-69 years	*Acinetobacter baumannii*	1270 (1070 to 1490)	614 (515 to 717)	-51.5 (-57.8 to -44.8)	21.8 (18.8 to 25.4)	7.61 (6.49 to 8.9)	-65.0 (-70.1 to -59.2)
		Chlamydia spp.	4680 (4120 to 5380)	2930 (2570 to 3340)	-37.1 (-45.5 to -27.8)	20 (17.8 to 22.5)	9.04 (7.89 to 10.3)	-54.7 (-61.1 to -47.1)
		Enterobacter spp.	1030 (893 to 1200)	1760 (1520 to 2000)	70.4 (45.1 to 93.9)	16.9 (14.6 to 19.5)	20.7 (18.1 to 23.5)	22.8 (4.2 to 42.8)
		Escherichia coli	2500 (2200 to 2850)	3560 (3150 to 3940)	42.7 (24.4 to 61.7)	42.2 (37.3 to 47.3)	43.3 (38.8 to 48.2)	2.8 (-11.4 to 19.7)
		Fungus	2390 (2070 to 2730)	2890 (2530 to 3290)	21.6 (5.6 to 40.0)	39.8 (34.9 to 45.4)	34.7 (30.2 to 39.6)	-12.4 (-25.7 to 1.5)
		Group B Streptococcus	2180 (1910 to 2520)	3010 (2630 to 3430)	38.7 (19.5 to 59.3)	26.1 (22.9 to 29.8)	26 (22.8 to 29.5)	0.0 (-14.6 to 17.0)
		Haemophilus influenzae	8220 (7390 to 9160)	8330 (7350 to 9290)	1.5 (-10.1 to 13.5)	53 (48.2 to 58.4)	26 (23.2 to 29.1)	-50.9 (-57.2 to -43.7)
		Influenza	9670 (8720 to 10800)	1290 (484 to 2680)	-86.6 (-95.0 to -72.2)	122 (111 to 134)	11.6 (4.07 to 23.9)	-90.5 (-96.7 to -79.5)
		Klebsiella pneumoniae	5110 (4620 to 5700)	4920 (4370 to 5440)	-3.7 (-15.4 to 8.4)	85.9 (78 to 94.5)	59.5 (53.1 to 66.1)	-30.6 (-39.7 to -20.6)
		Legionella spp.	7150 (6250 to 8170)	9440 (8380 to 10500)	32.3 (14.1 to 50.8)	82.7 (73.7 to 93.1)	78.6 (70.2 to 88.1)	-4.6 (-18.7 to 11.5)
		Mycoplasma spp.	8050 (7230 to 9090)	6330 (5600 to 7070)	-21.3 (-31.1 to -11.0)	35.9 (32.1 to 40.3)	20.3 (18.1 to 22.6)	-43.3 (-51.1 to -34.6)
		Polymicrobial	267 (161 to 430)	307 (172 to 518)	14.6 (-3.0 to 33.1)	4.58 (2.72 to 7.47)	3.79 (2.13 to 6.41)	-17.4 (-31.3 to -1.1)
		Pseudomonas aeruginosa	7480 (6650 to 8450)	11000 (9830 to 12200)	47.7 (29.3 to 66.0)	87.2 (79 to 96.6)	92.5 (83 to 103)	6.4 (-7.8 to 22.1)
		Respiratory syncytial virus	2610 (2300 to 2980)	274 (102 to 562)	-89.5 (-96.1 to -78.2)	15 (13.3 to 17)	1.12 (0.39 to 2.34)	-92.5 (-97.4 to -84.2)
		Staphylococcus aureus	8880 (8020 to 9870)	16400 (14600 to 18200)	85.2 (63.9 to 107.3)	212 (193 to 233)	282 (254 to 313)	33.4 (17.3 to 52.7)
		Streptococcus pneumoniae	37500 (33900 to 41700)	16900 (15000 to 18800)	-54.9 (-60.0 to -49.5)	373 (341 to 410)	121 (108 to 134)	-67.5 (-71.6 to -63.0)
		Other viruses	20400 (18300 to 22800)	21400 (19000 to 23700)	5.2 (-7.0 to 17.4)	79.7 (72.6 to 87.7)	60.3 (54.1 to 67)	-24.2 (-33.4 to -13.5)
		Other bacterial pathogens	8660 (7420 to 10000)	6860 (5750 to 8000)	-20.7 (-30.9 to -9.9)	82.2 (71.1 to 93.8)	46.9 (39.8 to 55)	-42.8 (-50.8 to -34.2)
	70+ years	*Acinetobacter baumannii*	2450 (2060 to 2870)	1810 (1540 to 2110)	-26.0 (-35.5 to -16.4)	229 (191 to 266)	122 (94.3 to 144)	-46.8 (-53.8 to -40.1)
		Chlamydia spp.	10800 (9540 to 12100)	10300 (9050 to 11600)	-4.1 (-16.4 to 9.2)	251 (216 to 280)	173 (139 to 198)	-31.0 (-40.5 to -21.3)
		Enterobacter spp.	1950 (1680 to 2250)	5050 (4360 to 5710)	159.9 (125.9 to 195.0)	173 (146 to 199)	323 (253 to 370)	86.9 (60.7 to 112.4)
		Escherichia coli	5930 (5280 to 6650)	12900 (11500 to 14300)	117.7 (92.8 to 143.9)	545 (473 to 607)	853 (682 to 961)	56.5 (35.6 to 74.6)
		Fungus	2570 (2230 to 2950)	4750 (4110 to 5450)	85.5 (60.8 to 113.6)	233 (201 to 266)	310 (245 to 362)	33.4 (15.6 to 52.0)
		Group B Streptococcus	3860 (3420 to 4390)	8160 (7190 to 9130)	111.6 (83.3 to 140.2)	252 (215 to 284)	383 (303 to 438)	52.1 (30.6 to 74.8)
		Haemophilus influenzae	14300 (13100 to 15800)	22100 (19800 to 24600)	54.8 (37.6 to 72.1)	501 (441 to 543)	375 (303 to 422)	-25.2 (-33.6 to -17.1)

		Influenza	28400 (26000 to 31000)	5790 (2170 to 12100)	-79.6 (-92.4 to -57.5)	1950 (1720 to 2100)	281 (99.9 to 581)	-85.5 (-94.8 to -70.1)
		Klebsiella pneumoniae	10900 (9970 to 12000)	16000 (14300 to 17700)	46.9 (30.6 to 63.2)	994 (872 to 1080)	1050 (842 to 1180)	5.6 (-6.5 to 16.3)
		Legionella spp.	9940 (8820 to 11200)	20000 (17800 to 22300)	101.9 (76.2 to 128.2)	625 (546 to 692)	906 (728 to 1020)	45.1 (26.2 to 63.6)
		Mycoplasma spp.	13200 (11900 to 14600)	15800 (14100 to 17500)	20.1 (6.7 to 34.1)	319 (279 to 350)	276 (220 to 310)	-13.7 (-23.8 to -3.8)
		Polymicrobial	592 (349 to 975)	1040 (567 to 1750)	74.9 (48.7 to 102.0)	55 (32.4 to 91.9)	69.7 (38 to 120)	25.7 (6.1 to 45.9)
		Pseudomonas aeruginosa	15000 (13500 to 16800)	33800 (30000 to 37400)	125.3 (100.7 to 151.9)	954 (842 to 1040)	1550 (1230 to 1740)	62.0 (42.6 to 80.8)
		Respiratory syncytial virus	4730 (4220 to 5330)	757 (280 to 1570)	-84.0 (-94.1 to -66.8)	148 (129 to 165)	16.8 (5.92 to 34.6)	-88.6 (-96.0 to -76.3)
		Staphylococcus aureus	22200 (20400 to 24300)	62700 (56200 to 69300)	182.5 (153.6 to 212.4)	2880 (2540 to 3110)	5860 (4680 to 6560)	103.1 (80.8 to 122.5)
		Streptococcus pneumoniae	74600 (68700 to 81400)	51300 (46000 to 56500)	-31.2 (-38.3 to -24.3)	4030 (3560 to 4350)	1990 (1590 to 2240)	-50.6 (-56.1 to -45.8)
		Other viruses	40100 (36900 to 43900)	64300 (57400 to 71000)	60.5 (44.0 to 77.4)	852 (752 to 917)	984 (796 to 1100)	15.4 (2.7 to 26.2)
		Other bacterial pathogens	22400 (19500 to 25500)	27100 (22900 to 31400)	20.9 (7.1 to 36.0)	1170 (991 to 1340)	1020 (792 to 1220)	-12.9 (-24.1 to -2.7)
Germany	All Ages	*Acinetobacter baumannii*	6160 (5230 to 7150)	5050 (4330 to 5780)	-17.8 (-26.6 to -7.2)	252 (214 to 291)	193 (155 to 225)	-23.5 (-32.7 to -14.4)
		Chlamydia spp.	26700 (23900 to 29600)	21700 (19500 to 24100)	-18.4 (-26.1 to -8.5)	266 (236 to 295)	203 (168 to 227)	-23.6 (-32.5 to -14.2)
		Enterobacter spp.	5030 (4400 to 5690)	9550 (8460 to 10800)	90.2 (69.8 to 113.1)	190 (164 to 219)	339 (275 to 386)	78.4 (54.9 to 99.4)
		Escherichia coli	13700 (12200 to 15300)	21700 (19600 to 23900)	58.3 (41.9 to 77.4)	576 (507 to 635)	837 (691 to 929)	45.4 (28.4 to 61.5)
		Fungus	8870 (7890 to 10000)	12000 (10800 to 13500)	35.8 (21.9 to 51.6)	285 (250 to 320)	377 (316 to 425)	32.2 (16.5 to 47.4)
		Group B Streptococcus	10800 (9600 to 12000)	17000 (15400 to 18800)	58.3 (41.6 to 76.8)	283 (248 to 319)	425 (353 to 475)	50.3 (31.4 to 68.2)
		Haemophilus influenzae	42000 (38600 to 45500)	45000 (40800 to 49200)	7.2 (-0.8 to 17.9)	438 (395 to 472)	393 (328 to 435)	-10.3 (-19.4 to -1.9)
		Influenza	66800 (61100 to 72600)	1690 (561 to 3990)	-97.5 (-99.2 to -94.0)	2060 (1820 to 2220)	47.8 (16.1 to 114)	-97.7 (-99.2 to -94.5)
		Klebsiella pneumoniae	27700 (25400 to 30200)	34500 (31200 to 37900)	24.5 (14.5 to 36.6)	1080 (965 to 1160)	1280 (1060 to 1420)	18.4 (6.1 to 30.2)
		Legionella spp.	29300 (26300 to 32400)	44300 (40200 to 48900)	51.2 (35.3 to 70.0)	714 (632 to 791)	1030 (865 to 1140)	44.7 (28.1 to 62.1)
		Mycoplasma spp.	46600 (42800 to 50900)	44600 (41100 to 48600)	-4.2 (-12.0 to 4.9)	378 (340 to 410)	348 (292 to 385)	-8.1 (-18.0 to 1.0)
		Polymicrobial	1460 (884 to 2360)	1960 (1110 to 3320)	33.7 (17.7 to 53.5)	60.2 (36.4 to 99.5)	75.1 (42.6 to 129)	23.9 (6.7 to 40.7)
		Pseudomonas aeruginosa	38400 (35000 to 42300)	71200 (64400 to 77900)	85.7 (68.1 to 105.6)	1040 (935 to 1130)	1800 (1480 to 2000)	73.1 (54.5 to 90.3)
		Respiratory syncytial virus	23800 (21700 to 26200)	484 (163 to 1120)	-98.0 (-99.3 to -95.2)	199 (181 to 217)	3.9 (1.3 to 9.19)	-98.0 (-99.3 to -95.4)
		Staphylococcus aureus	52300 (47700 to 56900)	109000 (98400 to 119000)	107.9 (91.9 to 128.6)	3070 (2740 to 3300)	5900 (4860 to 6500)	92.5 (73.1 to 110.4)

		Streptococcus pneumoniae	224000 (207000 to 243000)	147000 (135000 to 161000)	-34.2 (-38.7 to -28.3)	4490 (4050 to 4800)	2950 (2460 to 3250)	-34.4 (-40.6 to -28.6)
		Other viruses	133000 (123000 to 144000)	162000 (148000 to 176000)	22.1 (13.9 to 32.3)	983 (894 to 1050)	1220 (1020 to 1350)	24.2 (12.2 to 35.4)
		Other bacterial pathogens	49300 (42600 to 56000)	51200 (44300 to 59700)	4.1 (-5.8 to 16.9)	1200 (1030 to 1360)	1150 (914 to 1350)	-4.8 (-15.1 to 5.3)
	Under 5	*Acinetobacter baumannii*	52.1 (35.4 to 74.8)	22.7 (15.2 to 33.7)	-56.5 (-61.6 to -50.9)	0.98 (0.61 to 1.57)	0.21 (0.11 to 0.36)	-79.0 (-84.0 to -73.6)
		Chlamydia spp.	915 (746 to 1110)	385 (308 to 479)	-57.9 (-63.0 to -52.1)	2.36 (2.13 to 2.6)	0.29 (0.24 to 0.33)	-87.8 (-89.8 to -85.8)
		Enterobacter spp.	136 (102 to 178)	131 (95.7 to 175)	-3.7 (-17.1 to 11.8)	1.42 (1.09 to 1.84)	0.34 (0.25 to 0.47)	-75.9 (-80.2 to -71.0)
		Escherichia coli	404 (326 to 493)	366 (290 to 457)	-9.3 (-21.2 to 3.3)	4.14 (3.67 to 4.66)	0.88 (0.75 to 1.03)	-78.7 (-82.2 to -75.2)
		Fungus	76.6 (53.1 to 107)	57.7 (39.9 to 83)	-24.4 (-34.7 to -11.7)	0.62 (0.45 to 0.83)	0.09 (0.07 to 0.12)	-85.5 (-88.3 to -82.2)
		Group B Streptococcus	370 (297 to 462)	309 (246 to 385)	-16.5 (-27.5 to -4.1)	3.02 (2.67 to 3.43)	0.66 (0.55 to 0.78)	-78.1 (-81.7 to -74.5)
		Haemophilus influenzae	3100 (2520 to 3770)	1790 (1430 to 2240)	-42.3 (-48.5 to -34.5)	8.26 (7.63 to 8.98)	0.76 (0.65 to 0.87)	-90.8 (-92.3 to -89.3)
		Influenza	2840 (2310 to 3460)	29.2 (9.59 to 69)	-99.0 (-99.7 to -97.6)	18.5 (17.1 to 19.9)	0.04 (0.01 to 0.09)	-99.8 (-99.9 to -99.5)
		Klebsiella pneumoniae	1200 (979 to 1460)	664 (535 to 828)	-44.7 (-50.9 to -37.8)	10.9 (9.98 to 11.7)	1.39 (1.19 to 1.59)	-87.2 (-89.2 to -85.3)
		Legionella spp.	191 (132 to 265)	157 (106 to 222)	-17.4 (-27.7 to -5.8)	1.6 (1.22 to 2.02)	0.4 (0.3 to 0.51)	-74.9 (-79.4 to -69.8)
		Mycoplasma spp.	2200 (1780 to 2710)	1810 (1440 to 2260)	-17.8 (-27.5 to -6.4)	5.07 (4.58 to 5.6)	0.84 (0.72 to 0.96)	-83.4 (-86.2 to -80.9)
		Polymicrobial	56 (32.6 to 90)	36.9 (20.9 to 59.8)	-34.2 (-42.0 to -26.2)	1.37 (0.74 to 2.31)	0.45 (0.22 to 0.79)	-67.3 (-74.4 to -59.4)
		Pseudomonas aeruginosa	1210 (987 to 1480)	1480 (1190 to 1830)	22.3 (8.4 to 39.4)	7.95 (7.25 to 8.72)	2.07 (1.77 to 2.38)	-74.0 (-78.1 to -69.5)
		Respiratory syncytial virus	9750 (8020 to 11800)	146 (48.2 to 345)	-98.5 (-99.5 to -96.5)	30.1 (28 to 32.4)	0.09 (0.03 to 0.22)	-99.7 (-99.9 to -99.3)
		Staphylococcus aureus	1270 (1030 to 1540)	1300 (1040 to 1610)	2.6 (-7.3 to 14.3)	15.9 (14.6 to 17.1)	3.37 (2.91 to 3.86)	-78.7 (-81.9 to -75.6)
		Streptococcus pneumoniae	16600 (13600 to 20400)	3960 (3160 to 4930)	-76.1 (-78.3 to -73.4)	82.1 (76.3 to 88.2)	3.86 (3.37 to 4.4)	-95.3 (-96.0 to -94.6)
		Other viruses	15500 (12700 to 19000)	8980 (7220 to 11100)	-42.2 (-47.3 to -35.7)	30.7 (28.4 to 32.9)	3.73 (3.26 to 4.24)	-87.8 (-89.7 to -86.0)
		Other bacterial pathogens	422 (323 to 546)	238 (175 to 314)	-43.6 (-50.8 to -35.7)	2.34 (1.89 to 2.86)	0.32 (0.24 to 0.43)	-86.3 (-88.8 to -83.3)
	5-14 years	*Acinetobacter baumannii*	101 (73.5 to 138)	54.5 (39.7 to 72.8)	-46.1 (-52.2 to -39.8)	0.42 (0.35 to 0.49)	0.06 (0.05 to 0.07)	-85.4 (-87.7 to -83.0)
		Chlamydia spp.	616 (451 to 814)	353 (261 to 467)	-42.6 (-48.2 to -36.5)	0.63 (0.56 to 0.71)	0.1 (0.09 to 0.11)	-84.4 (-86.7 to -81.8)
		Enterobacter spp.	84.7 (61.2 to 115)	110 (78.6 to 147)	29.6 (14.0 to 45.1)	0.33 (0.28 to 0.38)	0.12 (0.1 to 0.14)	-64.8 (-70.4 to -58.9)

		Escherichia coli	184 (134 to 243)	198 (146 to 260)	7.7 (-4.5 to 19.6)	0.74 (0.66 to 0.82)	0.22 (0.19 to 0.24)	-70.8 (-75.1 to -66.2)
		Fungus	221 (162 to 297)	216 (159 to 289)	-2.5 (-13.6 to 8.3)	0.88 (0.77 to 1)	0.23 (0.2 to 0.26)	-73.5 (-77.6 to -68.9)
		Group B Streptococcus	251 (183 to 332)	283 (209 to 377)	12.6 (-1.1 to 25.0)	0.72 (0.63 to 0.81)	0.22 (0.19 to 0.25)	-69.4 (-73.9 to -64.3)
		Haemophilus influenzae	1090 (804 to 1430)	780 (582 to 1030)	-28.1 (-35.1 to -21.5)	1.3 (1.19 to 1.43)	0.22 (0.2 to 0.24)	-83.3 (-85.5 to -80.6)
		Influenza	1520 (1130 to 1970)	25.4 (8.17 to 61.4)	-98.3 (-99.4 to -96.1)	4.58 (4.18 to 5)	0.02 (0.01 to 0.05)	-99.6 (-99.8 to -98.9)
		Klebsiella pneumoniae	538 (395 to 703)	409 (307 to 535)	-24.0 (-31.0 to -16.8)	2.15 (1.96 to 2.35)	0.44 (0.4 to 0.5)	-79.4 (-82.1 to -76.2)
		Legionella spp.	678 (498 to 893)	719 (533 to 936)	6.2 (-5.9 to 18.3)	1.87 (1.65 to 2.09)	0.54 (0.48 to 0.6)	-71.2 (-75.7 to -66.4)
		Mycoplasma spp.	2330 (1700 to 3040)	1790 (1350 to 2380)	-23.0 (-30.6 to -15.6)	2.48 (2.24 to 2.74)	0.52 (0.46 to 0.58)	-79.1 (-82.0 to -75.7)
		Polymicrobial	25.4 (14.6 to 41.2)	22.9 (12.4 to 38.4)	-10.5 (-21.2 to 1.5)	0.1 (0.06 to 0.17)	0.03 (0.01 to 0.04)	-75.7 (-79.7 to -71.1)
		Pseudomonas aeruginosa	713 (528 to 938)	926 (698 to 1210)	30.0 (17.5 to 42.9)	1.98 (1.79 to 2.19)	0.7 (0.62 to 0.78)	-64.7 (-69.6 to -59.1)
		Respiratory syncytial virus	555 (413 to 729)	9.72 (3 to 23.4)	-98.3 (-99.4 to -95.9)	0.76 (0.68 to 0.84)	0 (0 to 0.01)	-99.5 (-99.8 to -98.9)
		Staphylococcus aureus	967 (706 to 1250)	1330 (994 to 1740)	37.1 (26.0 to 47.3)	5.5 (5.04 to 6)	2.04 (1.83 to 2.27)	-62.8 (-67.7 to -57.5)
		Streptococcus pneumoniae	8050 (5920 to 10400)	3690 (2800 to 4890)	-54.1 (-57.9 to -50.6)	19.1 (17.5 to 20.7)	2.37 (2.13 to 2.63)	-87.6 (-89.1 to -85.7)
		Other viruses	5050 (3730 to 6530)	4250 (3220 to 5630)	-15.8 (-22.9 to -9.2)	4.7 (4.3 to 5.11)	1.07 (0.96 to 1.2)	-77.2 (-80.1 to -73.8)
		Other bacterial pathogens	764 (559 to 1020)	521 (377 to 690)	-31.7 (-39.1 to -24.6)	1.73 (1.51 to 1.97)	0.32 (0.27 to 0.38)	-81.4 (-84.2 to -78.6)
	15-49 years	*Acinetobacter baumannii*	488 (393 to 595)	247 (201 to 301)	-49.4 (-55.0 to -43.0)	5.07 (4.31 to 5.95)	1.54 (1.32 to 1.8)	-69.5 (-73.0 to -65.6)
		Chlamydia spp.	2960 (2510 to 3460)	1600 (1340 to 1870)	-46.0 (-51.3 to -40.1)	7.65 (6.9 to 8.48)	2.48 (2.23 to 2.76)	-67.5 (-71.3 to -63.4)
		Enterobacter spp.	407 (334 to 486)	496 (408 to 597)	21.9 (8.5 to 36.8)	4.01 (3.48 to 4.62)	2.94 (2.55 to 3.36)	-26.6 (-36.1 to -16.3)
		Escherichia coli	886 (751 to 1030)	896 (761 to 1050)	1.2 (-8.8 to 13.3)	9.03 (8.12 to 9.96)	5.49 (4.98 to 6.02)	-39.0 (-46.2 to -31.0)
		Fungus	1070 (893 to 1250)	976 (822 to 1150)	-8.3 (-18.5 to 1.7)	10.7 (9.52 to 12)	5.9 (5.26 to 6.6)	-44.8 (-52.0 to -37.7)
		Group B Streptococcus	1210 (1020 to 1440)	1280 (1080 to 1510)	5.9 (-6.1 to 19.0)	8.76 (7.78 to 9.86)	5.57 (4.99 to 6.18)	-36.3 (-44.4 to -27.7)
		Haemophilus influenzae	5230 (4510 to 5980)	3530 (3020 to 4120)	-32.4 (-38.4 to -25.3)	15.9 (14.7 to 17.1)	5.55 (5.07 to 6.08)	-65.1 (-68.8 to -61.1)
		Influenza	7330 (6320 to 8310)	115 (38.4 to 272)	-98.4 (-99.5 to -96.3)	55.7 (52 to 59.8)	0.52 (0.17 to 1.19)	-99.1 (-99.7 to -97.8)
		Klebsiella pneumoniae	2590 (2240 to 2960)	1850 (1580 to 2160)	-28.5 (-34.2 to -21.6)	26.2 (24.3 to 28.2)	11.3 (10.3 to 12.2)	-57.0 (-61.7 to -52.1)

		Legionella spp.	3260 (2780 to 3800)	3250 (2800 to 3800)	-0.1 (-10.3 to 12.3)	22.8 (20.6 to 25)	13.7 (12.4 to 14.9)	-39.9 (-47.8 to -31.8)
		Mycoplasma spp.	11200 (9620 to 12800)	8110 (6970 to 9460)	-27.6 (-34.3 to -20.0)	30.2 (27.8 to 32.6)	13.1 (12 to 14.3)	-56.4 (-61.3 to -51.4)
		Polymicrobial	123 (72.9 to 198)	104 (57 to 177)	-15.9 (-26.6 to -4.3)	1.27 (0.75 to 2.09)	0.64 (0.36 to 1.07)	-49.3 (-56.4 to -41.8)
		Pseudomonas aeruginosa	3430 (2950 to 3950)	4190 (3590 to 4900)	22.3 (11.0 to 34.4)	24.1 (22.1 to 26.2)	17.7 (16.2 to 19.2)	-26.4 (-34.5 to -18.0)
		Respiratory syncytial virus	2670 (2290 to 3080)	43.8 (14.7 to 103)	-98.4 (-99.5 to -96.1)	9.27 (8.49 to 10.1)	0.09 (0.03 to 0.21)	-99.0 (-99.7 to -97.7)
		Staphylococcus aureus	4650 (4050 to 5320)	6000 (5160 to 7010)	29.0 (19.6 to 40.3)	67 (62.8 to 71.7)	51.9 (47.7 to 56.2)	-22.3 (-30.1 to -14.4)
		Streptococcus pneumoniae	38700 (33700 to 44100)	16700 (14500 to 19300)	-56.9 (-59.9 to -53.0)	232 (218 to 247)	60.2 (55.6 to 65)	-74.0 (-76.7 to -71.4)
		Other viruses	24300 (21100 to 27600)	19200 (16600 to 22300)	-20.8 (-26.4 to -13.6)	57.3 (53.6 to 61)	27.3 (25.2 to 29.6)	-52.3 (-57.2 to -47.4)
		Other bacterial pathogens	3680 (3050 to 4340)	2360 (1950 to 2860)	-35.8 (-41.5 to -28.5)	21 (18.5 to 23.9)	8.13 (6.99 to 9.3)	-61.3 (-66.0 to -56.7)
	50-69 years	*Acinetobacter baumannii*	2160 (1810 to 2550)	1410 (1170 to 1640)	-34.7 (-42.6 to -25.6)	32.9 (28.4 to 38)	21.2 (17.9 to 24.4)	-35.5 (-44.0 to -25.8)
		Chlamydia spp.	7750 (6780 to 8830)	5080 (4420 to 5810)	-34.4 (-41.7 to -24.9)	29.4 (26.2 to 32.8)	19 (17 to 21.4)	-35.2 (-43.8 to -25.5)
		Enterobacter spp.	1750 (1510 to 2020)	2670 (2310 to 3100)	53.0 (33.4 to 74.7)	25.3 (22 to 28.8)	38.1 (33.3 to 43.3)	51.1 (28.2 to 73.2)
		Escherichia coli	4200 (3660 to 4770)	5180 (4530 to 5890)	23.6 (9.6 to 40.3)	62.9 (56.5 to 69.7)	76.5 (69.4 to 84.5)	22.0 (5.5 to 39.3)
		Fungus	4020 (3520 to 4570)	4650 (4070 to 5340)	16.0 (2.3 to 31.7)	59.3 (52.4 to 67.3)	67.7 (59.7 to 76.1)	14.5 (-1.6 to 32.2)
		Group B Streptococcus	3670 (3210 to 4200)	4770 (4180 to 5450)	30.2 (14.2 to 48.1)	39 (34.6 to 44)	50 (44.4 to 56.2)	28.6 (10.5 to 47.7)
		Haemophilus influenzae	13500 (12200 to 15100)	12400 (10900 to 14000)	-8.2 (-17.9 to 2.8)	60.5 (55.4 to 65.8)	47 (42.7 to 51.8)	-22.2 (-31.6 to -12.1)
		Influenza	16400 (14700 to 18200)	330 (112 to 766)	-98.0 (-99.3 to -95.2)	183 (167 to 198)	3.58 (1.24 to 8.48)	-98.0 (-99.3 to -95.2)
		Klebsiella pneumoniae	8600 (7720 to 9540)	8750 (7690 to 9910)	1.9 (-9.1 to 14.2)	128 (118 to 139)	128 (116 to 141)	0.5 (-11.6 to 13.3)
		Legionella spp.	11900 (10400 to 13400)	14900 (13100 to 16800)	25.4 (10.4 to 42.9)	122 (110 to 134)	150 (136 to 166)	23.8 (7.6 to 42.3)
		Mycoplasma spp.	13300 (11800 to 15000)	11000 (9640 to 12300)	-17.5 (-26.7 to -6.5)	52.6 (47.8 to 57.9)	42.8 (38.6 to 47.3)	-18.6 (-28.7 to -7.4)
		Polymicrobial	451 (272 to 722)	485 (278 to 823)	7.1 (-6.9 to 24.0)	6.84 (4.12 to 11.1)	7.26 (4.15 to 12.3)	5.7 (-10.2 to 24.8)
		Pseudomonas aeruginosa	12600 (11300 to 14100)	18700 (16300 to 21000)	48.4 (32.1 to 67.6)	130 (119 to 143)	190 (173 to 210)	46.5 (27.9 to 66.8)
		Respiratory syncytial virus	4420 (3880 to 5010)	88.1 (30 to 207)	-98.0 (-99.3 to -95.3)	22.5 (19.9 to 25)	0.44 (0.15 to 1.03)	-98.1 (-99.3 to -95.3)
		Staphylococcus aureus	15000 (13500 to 16700)	24600 (21500 to 27800)	63.9 (47.6 to 83.3)	318 (293 to 344)	513 (466 to 563)	61.8 (43.2 to 82.1)
		Streptococcus pneumoniae	61800 (55500 to 68400)	35900 (31400 to 40500)	-41.9 (-47.7 to -35.0)	544 (501 to 587)	311 (283 to 342)	-42.6 (-49.2 to -35.3)
		Other viruses	34000 (30500 to 37700)	38000 (33300 to 42900)	12.1 (0.4 to 25.9)	118 (108 to 127)	130 (118 to 142)	10.6 (-1.6 to 24.5)

		Other bacterial pathogens	14400 (12200 to 16700)	11600 (9700 to 13500)	-19.6 (-28.5 to -8.9)	121 (105 to 137)	95.9 (81.6 to 112)	-20.6 (-30.3 to -9.6)
	70+ years	*Acinetobacter baumannii*	3360 (2790 to 3950)	3320 (2800 to 3870)	-0.9 (-12.1 to 13.6)	213 (179 to 248)	170 (132 to 200)	-20.1 (-30.7 to -10.2)
		Chlamydia spp.	14400 (12500 to 16200)	14300 (12600 to 16300)	-0.5 (-11.7 to 13.8)	226 (197 to 252)	182 (146 to 204)	-19.8 (-29.6 to -9.3)
		Enterobacter spp.	2650 (2260 to 3090)	6140 (5330 to 7020)	132.1 (102.5 to 163.7)	159 (135 to 185)	298 (236 to 341)	87.1 (62.1 to 110.9)
		Escherichia coli	8040 (7020 to 9140)	15000 (13300 to 16900)	87.5 (65.2 to 112.3)	499 (433 to 557)	754 (613 to 843)	51.1 (32.5 to 68.8)
		Fungus	3490 (3020 to 4050)	6130 (5350 to 7070)	75.9 (55.1 to 100.1)	214 (183 to 242)	303 (245 to 347)	41.8 (24.1 to 58.4)
		Group B Streptococcus	5260 (4550 to 5980)	10400 (9150 to 11700)	97.6 (74.4 to 123.4)	232 (199 to 263)	369 (300 to 418)	59.3 (38.2 to 79.6)
		Haemophilus influenzae	19000 (16900 to 21200)	26500 (23500 to 29700)	39.3 (25.1 to 55.9)	352 (309 to 385)	340 (277 to 379)	-3.6 (-14.2 to 6.4)
		Influenza	38800 (34400 to 42800)	1190 (395 to 2790)	-96.9 (-99.0 to -92.7)	1800 (1570 to 1950)	43.6 (14.7 to 104)	-97.6 (-99.2 to -94.3)
		Klebsiella pneumoniae	14800 (13100 to 16400)	22800 (20300 to 25400)	54.5 (39.5 to 72.7)	911 (802 to 990)	1140 (923 to 1270)	24.6 (10.9 to 37.5)
		Legionella spp.	13300 (11600 to 15100)	25300 (22500 to 28400)	90.2 (66.9 to 117.4)	566 (491 to 631)	867 (704 to 974)	53.3 (34.4 to 73.1)
		Mycoplasma spp.	17600 (15500 to 19900)	21900 (19600 to 24700)	25.2 (11.2 to 41.5)	288 (252 to 315)	290 (235 to 326)	0.9 (-11.1 to 11.6)
		Polymicrobial	806 (477 to 1340)	1310 (727 to 2240)	62.5 (41.5 to 87.9)	50.7 (30.2 to 84.8)	66.8 (37 to 115)	31.0 (12.9 to 49.4)
		Pseudomonas aeruginosa	20500 (18100 to 23000)	46000 (40800 to 51300)	125.1 (100.4 to 153.9)	876 (775 to 959)	1590 (1270 to 1780)	81.5 (60.7 to 100.7)
		Respiratory syncytial virus	6460 (5610 to 7390)	196 (65.5 to 458)	-97.0 (-99.0 to -92.7)	136 (118 to 153)	3.28 (1.09 to 7.71)	-97.6 (-99.2 to -94.3)
		Staphylococcus aureus	30300 (26900 to 33600)	75300 (66900 to 84300)	148.6 (124.7 to 177.1)	2660 (2350 to 2900)	5330 (4310 to 5930)	100.4 (79.0 to 120.6)
		Streptococcus pneumoniae	99100 (88200 to 110000)	87300 (77600 to 97500)	-11.8 (-20.2 to -1.5)	3620 (3190 to 3920)	2570 (2110 to 2860)	-28.9 (-36.3 to -22.1)
		Other viruses	53900 (48000 to 59600)	91400 (81400 to 103000)	70.0 (53.1 to 91.0)	773 (686 to 838)	1060 (859 to 1180)	37.0 (22.5 to 51.1)
		Other bacterial pathogens	30000 (25700 to 34500)	36600 (31100 to 43100)	21.9 (9.0 to 38.8)	1060 (890 to 1200)	1040 (816 to 1230)	-1.6 (-12.8 to 9.5)
Greece	All Ages	*Acinetobacter baumannii*	613 (524 to 709)	628 (545 to 719)	2.7 (-7.6 to 13.3)	23.2 (20 to 27)	57.2 (46.1 to 66.6)	146.4 (116.0 to 177.3)
		Chlamydia spp.	2930 (2620 to 3270)	2920 (2680 to 3200)	-0.2 (-9.3 to 9.9)	27 (23.9 to 30)	65.3 (54.1 to 73.1)	142.1 (111.1 to 171.6)
		Enterobacter spp.	593 (518 to 679)	1250 (1110 to 1380)	110.8 (90.3 to 131.4)	20.7 (17.8 to 23.8)	106 (85.2 to 120)	410.4 (339.8 to 471.9)
		Escherichia coli	1560 (1410 to 1750)	2950 (2710 to 3220)	89.3 (70.8 to 107.4)	60.5 (53.9 to 66.8)	272 (227 to 302)	350.4 (295.2 to 403.2)
		Fungus	1010 (895 to 1140)	1530 (1380 to 1690)	52.0 (37.3 to 69.3)	30.1 (26.4 to 34.1)	113 (93.7 to 129)	275.3 (226.8 to 323.7)

		Group B Streptococcus	1250 (1120 to 1400)	2210 (2020 to 2410)	77.9 (59.9 to 96.9)	30.3 (26.9 to 33.8)	132 (109 to 147)	334.4 (280.5 to 390.0)
		Haemophilus influenzae	5120 (4750 to 5570)	6030 (5590 to 6520)	17.8 (8.9 to 26.4)	62.5 (57 to 67)	124 (105 to 137)	98.6 (76.3 to 119.0)
		Influenza	7820 (7240 to 8550)	56.8 (10.6 to 179)	-99.3 (-99.9 to -97.7)	220 (199 to 237)	3.76 (0.7 to 11.8)	-98.3 (-99.7 to -94.8)
		Klebsiella pneumoniae	3170 (2940 to 3480)	4440 (4120 to 4810)	40.2 (29.1 to 50.3)	113 (103 to 121)	394 (328 to 435)	248.3 (209.7 to 281.4)
		Legionella spp.	3720 (3360 to 4130)	5640 (5220 to 6110)	52.1 (37.5 to 66.5)	83.8 (75.3 to 92.6)	312 (262 to 347)	272.6 (224.8 to 317.7)
		Mycoplasma spp.	5570 (5110 to 6070)	5770 (5390 to 6210)	3.8 (-4.4 to 11.9)	41.5 (37.5 to 45.5)	106 (88.2 to 117)	154.6 (123.7 to 184.1)
		Polymicrobial	159 (94 to 259)	263 (151 to 440)	65.1 (45.9 to 84.9)	6.18 (3.75 to 10.1)	24.1 (13.7 to 41.1)	288.0 (228.7 to 342.0)
		Pseudomonas aeruginosa	4720 (4320 to 5200)	9000 (8360 to 9740)	91.0 (75.5 to 106.5)	118 (106 to 129)	544 (455 to 601)	360.4 (303.9 to 410.8)
		Respiratory syncytial virus	2910 (2650 to 3200)	14.4 (2.68 to 45.2)	-99.5 (-99.9 to -98.5)	22.5 (20.5 to 24.5)	0.28 (0.05 to 0.86)	-98.8 (-99.8 to -96.2)
		Staphylococcus aureus	6190 (5750 to 6780)	14400 (13300 to 15600)	132.3 (115.4 to 148.5)	334 (301 to 359)	1870 (1560 to 2060)	459.0 (396.0 to 511.8)
		Streptococcus pneumoniae	26800 (25100 to 29100)	18500 (17200 to 19900)	-31.2 (-35.8 to -26.7)	490 (447 to 524)	879 (734 to 968)	79.4 (59.7 to 96.6)
		Other viruses	17200 (16000 to 18500)	20400 (19000 to 21900)	18.8 (10.7 to 26.7)	114 (104 to 122)	366 (309 to 403)	220.9 (185.0 to 250.8)
		Other bacterial pathogens	5640 (4920 to 6480)	6880 (5990 to 7860)	22.2 (11.5 to 32.8)	127 (110 to 144)	368 (294 to 431)	190.2 (155.2 to 223.3)
	Under 5	*Acinetobacter baumannii*	5.8 (3.83 to 8.59)	2.4 (1.63 to 3.49)	-58.5 (-63.1 to -53.3)	0.19 (0.1 to 0.33)	0.06 (0.03 to 0.11)	-69.0 (-76.1 to -60.4)
		Chlamydia spp.	92.4 (75.1 to 113)	44.9 (36.7 to 54.5)	-51.2 (-57.8 to -44.3)	0.26 (0.23 to 0.29)	0.08 (0.07 to 0.1)	-67.1 (-74.0 to -58.9)
		Enterobacter spp.	15.6 (11.6 to 20.6)	15 (11.1 to 20)	-3.2 (-17.9 to 14.4)	0.19 (0.13 to 0.28)	0.1 (0.07 to 0.14)	-47.1 (-59.4 to -32.2)
		Escherichia coli	43.2 (34.8 to 53)	44 (35.5 to 53.8)	2.3 (-12.9 to 17.0)	0.48 (0.42 to 0.55)	0.26 (0.21 to 0.32)	-45.0 (-57.1 to -31.1)
		Fungus	8.35 (5.8 to 11.8)	6.75 (4.73 to 9.56)	-18.8 (-32.5 to -3.5)	0.04 (0.03 to 0.06)	0.03 (0.02 to 0.04)	-39.7 (-55.9 to -20.6)
		Group B Streptococcus	40.8 (32.8 to 50.5)	35.6 (28.3 to 43.8)	-12.5 (-25.0 to 0.7)	0.42 (0.36 to 0.48)	0.19 (0.15 to 0.23)	-53.9 (-63.9 to -42.5)
		Haemophilus influenzae	407 (331 to 497)	215 (173 to 262)	-47.1 (-54.0 to -39.5)	1.11 (1 to 1.24)	0.23 (0.18 to 0.29)	-79.4 (-84.4 to -73.6)
		Influenza	324 (263 to 391)	0.84 (0.15 to 2.64)	-99.7 (-100.0 to -99.2)	1.6 (1.45 to 1.77)	0 (0 to 0.01)	-99.8 (-100.0 to -99.5)
		Klebsiella pneumoniae	158 (129 to 193)	74.5 (60.4 to 90.5)	-52.8 (-58.3 to -46.6)	1.15 (1.03 to 1.27)	0.4 (0.32 to 0.48)	-65.6 (-72.9 to -57.2)
		Legionella spp.	23.6 (16.5 to 32.5)	17.9 (12.2 to 25.1)	-24.2 (-34.4 to -12.7)	0.25 (0.2 to 0.32)	0.11 (0.08 to 0.15)	-55.3 (-65.4 to -43.8)
		Mycoplasma spp.	220 (178 to 271)	217 (176 to 264)	-1.2 (-13.5 to 11.9)	0.39 (0.35 to 0.44)	0.26 (0.2 to 0.32)	-34.9 (-50.5 to -16.0)

		Polymicrobial	7.22 (4.05 to 11.7)	3.88 (2.25 to 6.23)	-45.9 (-53.6 to -37.1)	0.33 (0.17 to 0.56)	0.13 (0.06 to 0.22)	-61.5 (-71.0 to -50.2)
		Pseudomonas aeruginosa	138 (112 to 170)	169 (137 to 205)	23.0 (7.7 to 40.8)	0.85 (0.76 to 0.96)	0.6 (0.47 to 0.73)	-29.8 (-46.0 to -11.9)
		Respiratory syncytial virus	1130 (933 to 1360)	4.03 (0.72 to 12.8)	-99.6 (-99.9 to -98.9)	2.95 (2.69 to 3.22)	0.01 (0 to 0.02)	-99.8 (-100.0 to -99.3)
		Staphylococcus aureus	145 (119 to 175)	150 (122 to 182)	3.4 (-6.5 to 14.5)	1.46 (1.33 to 1.6)	0.99 (0.78 to 1.22)	-32.4 (-47.2 to -15.4)
		Streptococcus pneumoniae	2030 (1670 to 2450)	424 (346 to 515)	-79.1 (-80.9 to -77.2)	6.98 (6.37 to 7.64)	1.04 (0.81 to 1.3)	-85.1 (-88.4 to -81.1)
		Other viruses	2240 (1840 to 2690)	987 (810 to 1200)	-55.8 (-59.4 to -51.6)	3.14 (2.87 to 3.44)	1.05 (0.82 to 1.3)	-66.7 (-74.0 to -58.0)
		Other bacterial pathogens	46.2 (34.9 to 60.2)	28 (21.3 to 36.5)	-39.1 (-47.9 to -29.3)	0.23 (0.18 to 0.32)	0.09 (0.07 to 0.13)	-59.4 (-68.4 to -48.9)
	5-14 years	*Acinetobacter baumannii*	13.8 (9.88 to 18.7)	6.69 (5.02 to 8.93)	-51.2 (-56.8 to -44.6)	0.04 (0.04 to 0.05)	0.01 (0.01 to 0.01)	-81.5 (-84.7 to -78.1)
		Chlamydia spp.	92.5 (67.7 to 122)	46.2 (35.2 to 60.2)	-49.8 (-55.4 to -43.4)	0.07 (0.06 to 0.08)	0.01 (0.01 to 0.02)	-81.0 (-84.3 to -77.3)
		Enterobacter spp.	13.6 (9.6 to 18.6)	14.1 (10.6 to 18.8)	4.1 (-8.2 to 17.7)	0.04 (0.03 to 0.05)	0.02 (0.01 to 0.02)	-60.6 (-67.5 to -52.8)
		Escherichia coli	28.7 (20.9 to 37.9)	26.3 (20.2 to 34.2)	-7.7 (-18.4 to 3.1)	0.09 (0.08 to 0.1)	0.03 (0.03 to 0.04)	-65.1 (-70.8 to -58.6)
		Fungus	34.3 (25.4 to 46.3)	27.9 (21.1 to 36.9)	-18.3 (-28.1 to -8.0)	0.11 (0.09 to 0.12)	0.03 (0.03 to 0.04)	-69.1 (-74.3 to -63.5)
		Group B Streptococcus	39.7 (29.1 to 52.8)	36.4 (27.7 to 47.3)	-8.1 (-18.8 to 4.1)	0.09 (0.08 to 0.1)	0.03 (0.03 to 0.04)	-65.2 (-71.4 to -58.9)
		Haemophilus influenzae	179 (136 to 237)	105 (80 to 135)	-41.4 (-47.6 to -35.5)	0.21 (0.19 to 0.24)	0.03 (0.03 to 0.04)	-85.3 (-87.6 to -82.7)
		Influenza	243 (182 to 317)	0.82 (0.15 to 2.69)	-99.7 (-99.9 to -98.9)	0.56 (0.51 to 0.62)	0 (0 to 0)	-99.9 (-100.0 to -99.6)
		Klebsiella pneumoniae	83.5 (63 to 108)	52.1 (40.1 to 66.1)	-37.4 (-43.7 to -31.1)	0.26 (0.23 to 0.29)	0.06 (0.05 to 0.07)	-76.3 (-79.9 to -72.0)
		Legionella spp.	117 (87.6 to 155)	92.1 (71.1 to 118)	-21.1 (-30.1 to -12.2)	0.25 (0.22 to 0.28)	0.07 (0.06 to 0.09)	-70.2 (-75.2 to -64.7)
		Mycoplasma spp.	380 (284 to 502)	231 (178 to 299)	-39.0 (-45.8 to -32.7)	0.31 (0.28 to 0.35)	0.07 (0.06 to 0.08)	-76.9 (-80.6 to -72.7)
		Polymicrobial	3.74 (2.11 to 6.08)	3.01 (1.65 to 4.99)	-19.6 (-30.3 to -9.6)	0.01 (0.01 to 0.02)	0 (0 to 0.01)	-69.6 (-75.0 to -63.9)
		Pseudomonas aeruginosa	120 (89.2 to 158)	116 (89.6 to 149)	-3.0 (-13.8 to 7.2)	0.26 (0.23 to 0.29)	0.09 (0.08 to 0.11)	-63.3 (-69.3 to -57.0)
		Respiratory syncytial virus	95.2 (71.5 to 126)	0.29 (0.05 to 0.95)	-99.7 (-99.9 to -99.0)	0.1 (0.09 to 0.11)	0 (0 to 0)	-99.9 (-100.0 to -99.7)
		Staphylococcus aureus	156 (118 to 204)	171 (131 to 219)	9.6 (-1.5 to 20.5)	0.69 (0.62 to 0.76)	0.28 (0.25 to 0.32)	-58.5 (-64.9 to -51.8)
		Streptococcus pneumoniae	1310 (990 to 1700)	452 (352 to 580)	-65.3 (-68.5 to -62.0)	2.39 (2.16 to 2.63)	0.31 (0.27 to 0.36)	-86.9 (-88.9 to -84.7)
		Other viruses	872 (659 to 1140)	531 (412 to 679)	-38.9 (-44.6 to -32.9)	0.63 (0.57 to 0.69)	0.14 (0.12 to 0.17)	-76.9 (-80.4 to -72.9)

		Other bacterial pathogens	119 (86.8 to 158)	68.1 (51.8 to 88.4)	-42.7 (-48.8 to -36.1)	0.21 (0.18 to 0.24)	0.05 (0.04 to 0.05)	-78.3 (-81.8 to -74.5)
	15-49 years	*Acinetobacter baumannii*	47.3 (37.5 to 57.7)	31.4 (26 to 37.5)	-33.3 (-41.5 to -25.4)	0.45 (0.38 to 0.52)	0.28 (0.24 to 0.33)	-36.6 (-44.5 to -28.3)
		Chlamydia spp.	317 (270 to 373)	217 (189 to 248)	-31.4 (-38.9 to -23.5)	0.74 (0.67 to 0.82)	0.48 (0.43 to 0.54)	-34.8 (-42.8 to -26.0)
		Enterobacter spp.	46.7 (38.2 to 56.6)	66.3 (55.7 to 77.1)	42.4 (25.4 to 59.1)	0.42 (0.36 to 0.48)	0.56 (0.49 to 0.65)	35.2 (18.1 to 53.5)
		Escherichia coli	98.3 (82.7 to 116)	124 (108 to 140)	26.2 (13.4 to 39.4)	0.91 (0.82 to 1.01)	1.09 (0.99 to 1.2)	19.9 (6.4 to 34.8)
		Fungus	118 (98.6 to 140)	131 (114 to 151)	11.7 (-1.7 to 24.3)	1.08 (0.95 to 1.2)	1.14 (1 to 1.28)	6.1 (-7.1 to 19.9)
		Group B Streptococcus	136 (115 to 164)	171 (148 to 196)	25.8 (10.0 to 41.4)	0.9 (0.8 to 1.01)	1.07 (0.95 to 1.2)	19.4 (3.9 to 36.3)
		Haemophilus influenzae	615 (528 to 717)	492 (433 to 558)	-19.9 (-27.5 to -12.4)	2.17 (2.02 to 2.33)	1.1 (1 to 1.2)	-49.5 (-54.9 to -43.6)
		Influenza	832 (719 to 960)	3.86 (0.72 to 12.3)	-99.5 (-99.9 to -98.5)	5.75 (5.36 to 6.13)	0.02 (0 to 0.08)	-99.6 (-99.9 to -98.7)
		Klebsiella pneumoniae	286 (247 to 332)	245 (215 to 277)	-14.3 (-22.6 to -6.9)	2.64 (2.46 to 2.82)	2.14 (1.95 to 2.35)	-18.6 (-27.1 to -9.0)
		Legionella spp.	402 (342 to 471)	433 (379 to 488)	7.9 (-3.9 to 19.4)	2.55 (2.32 to 2.8)	2.61 (2.37 to 2.86)	2.5 (-10.6 to 15.2)
		Mycoplasma spp.	1300 (1120 to 1510)	1090 (954 to 1230)	-16.5 (-24.5 to -8.6)	3.19 (2.95 to 3.44)	2.52 (2.3 to 2.77)	-20.7 (-29.5 to -11.3)
		Polymicrobial	12.9 (7.3 to 21.1)	14.2 (7.72 to 24.2)	10.0 (-3.4 to 23.6)	0.12 (0.07 to 0.2)	0.13 (0.07 to 0.21)	4.5 (-9.3 to 19.8)
		Pseudomonas aeruginosa	411 (352 to 480)	544 (477 to 617)	32.7 (19.1 to 45.8)	2.63 (2.42 to 2.86)	3.31 (3.04 to 3.62)	26.0 (11.5 to 41.5)
		Respiratory syncytial virus	327 (281 to 379)	1.38 (0.26 to 4.42)	-99.6 (-99.9 to -98.6)	1.03 (0.94 to 1.12)	0 (0 to 0.01)	-99.6 (-99.9 to -98.8)
		Staphylococcus aureus	536 (463 to 619)	802 (706 to 908)	49.9 (35.7 to 61.7)	7.01 (6.56 to 7.45)	9.98 (9.14 to 10.9)	42.4 (27.7 to 58.3)
		Streptococcus pneumoniae	4480 (3860 to 5170)	2120 (1880 to 2380)	-52.5 (-56.9 to -48.8)	24.4 (23 to 25.8)	11 (10.1 to 12)	-54.9 (-59.5 to -50.0)
		Other viruses	2990 (2580 to 3460)	2500 (2210 to 2800)	-16.4 (-24.1 to -9.5)	6.41 (6.02 to 6.77)	5.08 (4.66 to 5.53)	-20.6 (-28.8 to -11.8)
		Other bacterial pathogens	409 (334 to 488)	320 (270 to 374)	-21.6 (-30.2 to -14.1)	2.13 (1.86 to 2.41)	1.58 (1.38 to 1.81)	-25.5 (-33.6 to -16.2)
	50-69 years	*Acinetobacter baumannii*	211 (177 to 247)	140 (118 to 162)	-33.3 (-40.4 to -26.1)	3.25 (2.74 to 3.84)	2.93 (2.47 to 3.38)	-9.7 (-22.7 to 4.6)
		Chlamydia spp.	836 (732 to 961)	542 (480 to 609)	-35.0 (-41.6 to -27.8)	3.21 (2.82 to 3.65)	2.81 (2.51 to 3.18)	-11.9 (-24.2 to 3.2)
		Enterobacter spp.	202 (172 to 235)	280 (244 to 318)	38.6 (24.7 to 55.0)	2.96 (2.53 to 3.42)	5.53 (4.85 to 6.23)	87.8 (61.5 to 116.5)
		Escherichia coli	469 (413 to 534)	559 (501 to 619)	19.2 (7.5 to 32.1)	7.11 (6.35 to 7.92)	11.4 (10.4 to 12.6)	61.5 (39.1 to 87.7)
		Fungus	448 (388 to 515)	489 (433 to 554)	9.5 (-1.6 to 23.3)	6.68 (5.81 to 7.61)	9.88 (8.77 to 11.1)	48.3 (27.6 to 73.0)
		Group B Streptococcus	417 (361 to 478)	500 (443 to 558)	20.1 (6.2 to 34.5)	4.48 (3.95 to 5.09)	7.27 (6.42 to 8.14)	62.7 (40.0 to 89.0)
		Haemophilus influenzae	1610 (1430 to 1790)	1340 (1210 to 1490)	-16.3 (-23.3 to -8.3)	9.28 (8.44 to 10.2)	6.96 (6.25 to 7.67)	-24.8 (-34.6 to -14.3)

		Influenza	1870 (1670 to 2090)	8.73 (1.65 to 26.9)	-99.5 (-99.9 to -98.5)	21.2 (19.3 to 23.3)	0.13 (0.02 to 0.39)	-99.4 (-99.9 to -98.1)
		Klebsiella pneumoniae	958 (862 to 1070)	900 (808 to 993)	-6.0 (-13.7 to 2.7)	14.4 (13.1 to 16)	18.3 (16.5 to 20.2)	27.3 (11.5 to 44.8)
		Legionella spp.	1480 (1310 to 1670)	1540 (1390 to 1710)	4.8 (-5.5 to 16.2)	15.3 (13.6 to 17.2)	21.7 (19.6 to 23.8)	42.0 (22.3 to 63.7)
		Mycoplasma spp.	1560 (1380 to 1750)	1140 (1030 to 1270)	-26.6 (-33.3 to -19.1)	6.24 (5.59 to 6.99)	6.19 (5.58 to 6.84)	-0.5 (-14.4 to 14.2)
		Polymicrobial	47.7 (28.1 to 78.1)	51.9 (30 to 88.2)	8.6 (-4.2 to 23.0)	0.73 (0.42 to 1.19)	1.08 (0.61 to 1.83)	47.0 (24.7 to 72.1)
		Pseudomonas aeruginosa	1520 (1360 to 1710)	1890 (1690 to 2090)	24.2 (12.5 to 35.8)	15.9 (14.4 to 17.7)	26.7 (24 to 29.2)	68.2 (45.7 to 92.9)
		Respiratory syncytial virus	544 (472 to 616)	2.16 (0.4 to 6.89)	-99.6 (-99.9 to -98.7)	2.8 (2.47 to 3.17)	0.01 (0 to 0.04)	-99.5 (-99.9 to -98.4)
		Staphylococcus aureus	1750 (1570 to 1930)	2580 (2320 to 2850)	47.7 (35.6 to 60.7)	37.3 (34.1 to 41)	74.5 (67.5 to 81.6)	100.0 (74.3 to 127.5)
		Streptococcus pneumoniae	7200 (6490 to 8020)	3600 (3240 to 3960)	-49.9 (-53.9 to -45.5)	64.1 (58.6 to 70.1)	43.4 (39.3 to 47.4)	-32.2 (-40.9 to -22.9)
		Other viruses	4210 (3800 to 4660)	3840 (3450 to 4240)	-8.7 (-16.1 to -0.4)	14.7 (13.4 to 16.2)	18.2 (16.5 to 19.9)	23.7 (8.1 to 41.0)
		Other bacterial pathogens	1610 (1380 to 1880)	1220 (1030 to 1410)	-24.1 (-31.6 to -16.6)	13.7 (11.8 to 15.9)	14.1 (12.1 to 16.4)	2.9 (-11.2 to 17.9)
	70+ years	*Acinetobacter baumannii*	336 (282 to 400)	448 (383 to 518)	33.6 (18.9 to 48.0)	19.3 (16.4 to 22.6)	54 (43 to 62.9)	179.6 (146.5 to 215.3)
		Chlamydia spp.	1590 (1390 to 1820)	2070 (1850 to 2310)	30.3 (16.3 to 45.0)	22.7 (19.9 to 25.4)	61.9 (50.8 to 69.6)	172.7 (138.2 to 206.8)
		Enterobacter spp.	314 (268 to 371)	872 (757 to 987)	177.9 (147.8 to 209.1)	17.1 (14.7 to 19.8)	99.5 (79.5 to 113)	481.6 (404.6 to 549.3)
		Escherichia coli	921 (812 to 1060)	2200 (1980 to 2430)	139.1 (113.9 to 163.1)	51.9 (45.8 to 57.7)	259 (214 to 289)	400.2 (337.9 to 456.3)
		Fungus	398 (347 to 462)	872 (766 to 987)	119.5 (95.2 to 147.6)	22.1 (19.3 to 25.5)	102 (83.2 to 117)	359.3 (300.5 to 416.7)
		Group B Streptococcus	611 (536 to 704)	1470 (1310 to 1640)	140.8 (113.8 to 167.6)	24.5 (21.3 to 27.3)	123 (101 to 139)	403.8 (340.9 to 467.0)
		Haemophilus influenzae	2310 (2090 to 2610)	3870 (3500 to 4300)	67.8 (51.7 to 82.6)	49.7 (44.4 to 53.7)	116 (96.5 to 129)	132.9 (107.0 to 156.6)
		Influenza	4550 (4120 to 5110)	42.6 (7.97 to 134)	-99.1 (-99.8 to -97.1)	191 (171 to 206)	3.61 (0.68 to 11.3)	-98.1 (-99.6 to -94.3)
		Klebsiella pneumoniae	1690 (1530 to 1900)	3170 (2870 to 3490)	88.4 (70.5 to 104.1)	94.5 (84.5 to 102)	373 (307 to 413)	294.2 (251.8 to 331.9)
		Legionella spp.	1700 (1500 to 1940)	3560 (3210 to 3930)	110.2 (87.2 to 133.9)	65.4 (57.7 to 72.6)	288 (238 to 320)	339.7 (284.8 to 392.3)
		Mycoplasma spp.	2110 (1880 to 2410)	3100 (2800 to 3440)	47.2 (32.1 to 61.9)	31.4 (27.8 to 34.6)	96.6 (79.8 to 108)	208.0 (171.3 to 242.5)
		Polymicrobial	87.3 (50.4 to 146)	190 (108 to 327)	117.6 (90.7 to 145.1)	4.98 (2.91 to 8.36)	22.8 (12.7 to 39.1)	355.4 (291.7 to 415.7)
		Pseudomonas aeruginosa	2530 (2270 to 2890)	6290 (5700 to 6920)	149.0 (124.2 to 172.1)	98.5 (87.8 to 108)	513 (424 to 570)	421.0 (358.7 to 477.3)

		Respiratory syncytial virus	815 (723 to 933)	6.51 (1.21 to 20.2)	-99.2 (-99.9 to -97.4)	15.6 (13.8 to 17.4)	0.25 (0.05 to 0.79)	-98.4 (-99.7 to -95.0)
		Staphylococcus aureus	3610 (3270 to 4050)	10700 (9650 to 11700)	196.1 (169.1 to 220.8)	287 (257 to 310)	1780 (1480 to 1970)	519.5 (453.1 to 577.9)
		Streptococcus pneumoniae	11800 (10800 to 13300)	11900 (10700 to 13100)	0.3 (-9.1 to 8.3)	392 (353 to 422)	824 (680 to 911)	109.9 (86.7 to 129.9)
		Other viruses	6840 (6200 to 7700)	12500 (11300 to 13800)	83.1 (65.5 to 98.0)	89.2 (79.8 to 96.1)	342 (285 to 379)	283.1 (242.0 to 319.6)
		Other bacterial pathogens	3450 (2980 to 4020)	5240 (4510 to 6060)	52.2 (36.7 to 67.3)	110 (94.8 to 126)	352 (280 to 413)	218.7 (180.5 to 254.0)
Iceland	All Ages	*Acinetobacter baumannii*	16.2 (13.8 to 18.8)	11.9 (9.83 to 14.1)	-26.7 (-36.6 to -16.0)	1.06 (0.88 to 1.23)	0.64 (0.49 to 0.76)	-39.7 (-47.1 to -31.9)
		Chlamydia spp.	78.3 (70.1 to 87.1)	62.9 (54.5 to 72.2)	-19.6 (-30.2 to -7.4)	1.22 (1.05 to 1.35)	0.82 (0.66 to 0.94)	-33.1 (-41.4 to -24.2)
		Enterobacter spp.	18.1 (15.7 to 20.5)	30.5 (26.1 to 35.5)	69.5 (46.8 to 95.3)	1.08 (0.9 to 1.24)	1.51 (1.18 to 1.77)	40.4 (22.1 to 59.0)
		Escherichia coli	45.6 (41.2 to 50.9)	68.1 (58.9 to 78.9)	49.6 (29.5 to 72.0)	3.01 (2.57 to 3.35)	3.72 (2.98 to 4.27)	23.4 (8.0 to 38.3)
		Fungus	28 (25 to 31.8)	37.3 (32.2 to 43.2)	33.4 (15.4 to 53.3)	1.42 (1.21 to 1.61)	1.55 (1.26 to 1.8)	9.4 (-4.5 to 24.4)
		Group B Streptococcus	37.1 (33.5 to 41.2)	53.7 (46.6 to 61.9)	45.2 (24.5 to 68.0)	1.52 (1.29 to 1.7)	1.83 (1.48 to 2.11)	20.5 (3.6 to 36.0)
		Haemophilus influenzae	144 (133 to 156)	152 (131 to 174)	5.4 (-7.3 to 21.0)	1.97 (1.7 to 2.16)	1.83 (1.49 to 2.08)	-7.2 (-18.3 to 3.9)
		Influenza	243 (224 to 264)	324 (171 to 461)	33.3 (-29.3 to 86.9)	11.7 (10 to 12.8)	12.8 (6.42 to 19.9)	9.9 (-45.3 to 65.8)
		Klebsiella pneumoniae	91.3 (84.5 to 98.7)	100 (87.1 to 114)	9.5 (-3.4 to 25.3)	5.59 (4.8 to 6.12)	5.2 (4.18 to 5.94)	-7.0 (-17.2 to 3.4)
		Legionella spp.	110 (99.2 to 122)	149 (130 to 169)	35.4 (18.5 to 55.4)	4.24 (3.63 to 4.74)	4.71 (3.84 to 5.38)	11.0 (-2.4 to 25.3)
		Mycoplasma spp.	160 (147 to 174)	152 (133 to 174)	-4.5 (-16.4 to 8.4)	1.96 (1.69 to 2.16)	1.5 (1.22 to 1.71)	-23.5 (-31.8 to -14.3)
		Polymicrobial	4.61 (2.82 to 7.65)	5.84 (3.25 to 10)	25.9 (7.5 to 47.4)	0.29 (0.17 to 0.49)	0.31 (0.17 to 0.55)	5.0 (-10.2 to 19.6)
		Pseudomonas aeruginosa	149 (136 to 164)	228 (198 to 261)	52.6 (33.2 to 74.1)	6.35 (5.49 to 6.96)	7.96 (6.33 to 9.09)	25.3 (11.0 to 39.9)
		Respiratory syncytial virus	116 (105 to 129)	110 (59.6 to 157)	-5.0 (-47.2 to 32.6)	1.26 (1.1 to 1.39)	1.04 (0.52 to 1.6)	-17.5 (-58.1 to 24.4)
		Staphylococcus aureus	193 (176 to 209)	348 (303 to 401)	80.8 (59.9 to 106.4)	17.8 (15.3 to 19.5)	26.8 (21.6 to 30.4)	50.3 (33.7 to 67.0)
		Streptococcus pneumoniae	748 (696 to 805)	425 (373 to 486)	-43.1 (-49.8 to -35.6)	22.9 (19.8 to 24.9)	11.5 (9.39 to 13.1)	-49.6 (-55.2 to -44.1)
		Other viruses	525 (491 to 566)	541 (472 to 622)	3.1 (-9.3 to 17.2)	5.87 (5.07 to 6.38)	5.43 (4.42 to 6.14)	-7.5 (-17.9 to 2.8)
		Other bacterial pathogens	156 (136 to 178)	150 (126 to 178)	-3.5 (-16.2 to 9.2)	6.06 (5.04 to 7)	4.81 (3.69 to 5.77)	-20.7 (-30.2 to -10.8)
	Under 5	*Acinetobacter baumannii*	0.27 (0.17 to 0.42)	0.13 (0.07 to 0.21)	-53.5 (-64.6 to -39.3)	0.01 (0 to 0.01)	0 (0 to 0)	-76.0 (-81.9 to -68.9)
		Chlamydia spp.	4.12 (3.46 to 4.89)	1.91 (1.36 to 2.6)	-53.7 (-65.2 to -39.5)	0.01 (0.01 to 0.01)	0 (0 to 0)	-77.6 (-83.1 to -70.9)
		Enterobacter spp.	0.74 (0.55 to 0.98)	0.72 (0.49 to 1.01)	-2.9 (-27.8 to 26.7)	0.01 (0.01 to 0.01)	0 (0 to 0)	-59.8 (-70.1 to -46.9)

		Escherichia coli	2.04 (1.71 to 2.45)	1.98 (1.39 to 2.68)	-3.2 (-27.3 to 25.9)	0.02 (0.02 to 0.03)	0.01 (0.01 to 0.01)	-60.1 (-70.2 to -48.2)
		Fungus	0.36 (0.25 to 0.5)	0.29 (0.19 to 0.44)	-17.9 (-37.9 to 7.7)	0 (0 to 0)	0 (0 to 0)	-61.5 (-72.2 to -48.4)
		Group B Streptococcus	1.99 (1.64 to 2.46)	1.66 (1.17 to 2.26)	-16.5 (-37.7 to 9.0)	0.02 (0.02 to 0.02)	0.01 (0.01 to 0.01)	-65.5 (-73.6 to -55.4)
		Haemophilus influenzae	14 (11.5 to 16.7)	9.89 (7.09 to 13.4)	-29.1 (-46.2 to -8.6)	0.03 (0.03 to 0.04)	0.01 (0.01 to 0.01)	-69.5 (-77.7 to -59.1)
		Influenza	14.3 (11.9 to 16.9)	9.29 (4.94 to 14)	-34.9 (-63.9 to -7.8)	0.09 (0.08 to 0.11)	0.03 (0.01 to 0.04)	-71.2 (-86.2 to -51.6)
		Klebsiella pneumoniae	5.09 (4.24 to 6.08)	3.34 (2.38 to 4.48)	-34.3 (-50.2 to -15.3)	0.05 (0.04 to 0.06)	0.01 (0.01 to 0.02)	-70.0 (-77.8 to -61.2)
		Legionella spp.	1.14 (0.81 to 1.56)	0.92 (0.58 to 1.4)	-19.3 (-38.5 to 5.6)	0.01 (0.01 to 0.01)	0 (0 to 0.01)	-64.9 (-74.0 to -55.2)
		Mycoplasma spp.	11.6 (9.62 to 13.9)	9.16 (6.58 to 12.3)	-21.1 (-40.1 to 2.4)	0.03 (0.02 to 0.03)	0.01 (0.01 to 0.01)	-64.1 (-73.3 to -52.7)
		Polymicrobial	0.4 (0.22 to 0.67)	0.26 (0.13 to 0.46)	-35.9 (-51.4 to -16.0)	0.01 (0.01 to 0.02)	0 (0 to 0.01)	-68.8 (-76.2 to -59.5)
		Pseudomonas aeruginosa	7.02 (5.89 to 8.43)	7.9 (5.66 to 10.6)	12.6 (-15.1 to 45.3)	0.05 (0.04 to 0.06)	0.02 (0.02 to 0.03)	-52.1 (-63.5 to -37.5)
		Respiratory syncytial virus	59.2 (49.8 to 69.7)	45.1 (24.1 to 67.3)	-23.9 (-56.9 to 7.1)	0.19 (0.16 to 0.21)	0.06 (0.03 to 0.1)	-66.8 (-83.8 to -45.2)
		Staphylococcus aureus	6.62 (5.52 to 7.85)	7.06 (5.08 to 9.52)	6.5 (-18.3 to 37.2)	0.08 (0.07 to 0.1)	0.04 (0.03 to 0.05)	-52.5 (-64.2 to -37.8)
		Streptococcus pneumoniae	73.7 (61.3 to 87.3)	18.5 (13.4 to 25)	-74.9 (-80.8 to -67.7)	0.35 (0.3 to 0.41)	0.04 (0.03 to 0.05)	-88.5 (-91.5 to -85.0)
		Other viruses	76.4 (63.9 to 90.4)	48.4 (34.5 to 65.5)	-36.6 (-51.2 to -18.9)	0.15 (0.13 to 0.17)	0.04 (0.03 to 0.06)	-70.2 (-77.7 to -61.0)
		Other bacterial pathogens	1.94 (1.49 to 2.48)	1.2 (0.81 to 1.7)	-37.9 (-52.7 to -20.0)	0.01 (0.01 to 0.02)	0 (0 to 0)	-71.5 (-78.4 to -62.7)
	5-14 years	*Acinetobacter baumannii*	0.34 (0.25 to 0.47)	0.2 (0.14 to 0.27)	-42.0 (-50.4 to -33.0)	0 (0 to 0)	0 (0 to 0)	-74.7 (-80.1 to -68.0)
		Chlamydia spp.	2.28 (1.69 to 3.01)	1.6 (1.15 to 2.16)	-29.8 (-39.7 to -19.5)	0 (0 to 0)	0 (0 to 0)	-69.4 (-76.0 to -61.3)
		Enterobacter spp.	0.39 (0.28 to 0.53)	0.55 (0.39 to 0.75)	41.1 (20.8 to 62.7)	0 (0 to 0)	0 (0 to 0)	-38.6 (-51.8 to -21.5)
		Escherichia coli	0.78 (0.57 to 1.03)	1 (0.72 to 1.35)	29.4 (12.2 to 48.0)	0 (0 to 0.01)	0 (0 to 0)	-43.6 (-55.7 to -28.8)
		Fungus	0.94 (0.69 to 1.25)	1.02 (0.74 to 1.41)	9.6 (-6.0 to 25.6)	0.01 (0 to 0.01)	0 (0 to 0)	-52.3 (-62.6 to -39.5)
		Group B Streptococcus	1.11 (0.81 to 1.46)	1.39 (1.01 to 1.87)	25.5 (7.4 to 44.7)	0 (0 to 0.01)	0 (0 to 0)	-45.4 (-57.5 to -30.1)
		Haemophilus influenzae	4.73 (3.56 to 6.19)	3.86 (2.82 to 5.15)	-18.5 (-28.7 to -7.7)	0.01 (0.01 to 0.01)	0 (0 to 0)	-63.4 (-71.2 to -54.0)
		Influenza	6.93 (5.21 to 8.98)	7.48 (3.61 to 11.9)	7.9 (-43.0 to 54.7)	0.03 (0.03 to 0.04)	0.01 (0.01 to 0.02)	-54.6 (-77.6 to -28.6)
		Klebsiella pneumoniae	2.26 (1.7 to 2.94)	1.75 (1.29 to 2.32)	-22.8 (-32.2 to -13.3)	0.01 (0.01 to 0.02)	0 (0 to 0.01)	-66.4 (-73.2 to -57.6)

		Legionella spp.	3.35 (2.52 to 4.39)	3.76 (2.75 to 5.03)	12.1 (-3.1 to 28.2)	0.01 (0.01 to 0.02)	0.01 (0.01 to 0.01)	-51.2 (-62.0 to -37.8)
		Mycoplasma spp.	10.1 (7.54 to 13.1)	9.13 (6.72 to 12.2)	-9.4 (-21.2 to 2.1)	0.02 (0.01 to 0.02)	0.01 (0.01 to 0.01)	-60.5 (-68.8 to -50.6)
		Polymicrobial	0.1 (0.06 to 0.16)	0.1 (0.06 to 0.18)	5.8 (-10.5 to 22.6)	0 (0 to 0)	0 (0 to 0)	-53.9 (-64.1 to -40.5)
		Pseudomonas aeruginosa	3.54 (2.64 to 4.63)	4.72 (3.44 to 6.24)	33.5 (15.4 to 51.7)	0.02 (0.01 to 0.02)	0.01 (0.01 to 0.01)	-41.9 (-54.0 to -26.7)
		Respiratory syncytial virus	2.91 (2.2 to 3.79)	2.88 (1.39 to 4.62)	-0.9 (-48.0 to 41.8)	0.01 (0.01 to 0.01)	0 (0 to 0)	-58.3 (-79.5 to -33.5)
		Staphylococcus aureus	4.53 (3.4 to 5.88)	6.67 (4.89 to 8.8)	47.3 (29.1 to 64.2)	0.04 (0.04 to 0.05)	0.03 (0.02 to 0.03)	-35.8 (-48.8 to -19.4)
		Streptococcus pneumoniae	33.7 (25.4 to 43.9)	15.3 (11.2 to 20.3)	-54.6 (-60.2 to -49.2)	0.12 (0.11 to 0.14)	0.02 (0.02 to 0.03)	-80.2 (-84.2 to -75.2)
		Other viruses	25 (18.8 to 32.3)	19.8 (14.5 to 26.5)	-20.9 (-30.6 to -11.7)	0.04 (0.03 to 0.04)	0.01 (0.01 to 0.02)	-65.6 (-72.5 to -56.8)
		Other bacterial pathogens	3.08 (2.26 to 4.08)	2.45 (1.75 to 3.33)	-20.5 (-31.4 to -9.1)	0.01 (0.01 to 0.01)	0 (0 to 0)	-65.3 (-72.6 to -56.4)
	15-49 years	*Acinetobacter baumannii*	1.27 (1.02 to 1.54)	0.83 (0.65 to 1.02)	-34.7 (-44.0 to -24.4)	0.01 (0.01 to 0.02)	0.01 (0.01 to 0.01)	-50.5 (-57.7 to -42.4)
		Chlamydia spp.	8.39 (7.24 to 9.72)	6.63 (5.44 to 7.85)	-20.9 (-31.4 to -8.5)	0.02 (0.02 to 0.03)	0.01 (0.01 to 0.02)	-40.0 (-48.9 to -29.9)
		Enterobacter spp.	1.43 (1.18 to 1.73)	2.27 (1.83 to 2.77)	58.9 (36.4 to 83.9)	0.02 (0.01 to 0.02)	0.02 (0.02 to 0.02)	20.5 (1.5 to 41.8)
		Escherichia coli	2.85 (2.42 to 3.29)	4.15 (3.46 to 4.91)	45.8 (25.2 to 68.5)	0.03 (0.03 to 0.04)	0.04 (0.03 to 0.04)	10.6 (-5.7 to 28.6)
		Fungus	3.43 (2.92 to 4.01)	4.23 (3.46 to 5.11)	23.4 (6.1 to 42.0)	0.04 (0.03 to 0.04)	0.04 (0.03 to 0.04)	-6.4 (-21.5 to 10.5)
		Group B Streptococcus	4.07 (3.48 to 4.77)	5.74 (4.76 to 6.85)	41.3 (21.4 to 63.6)	0.03 (0.03 to 0.04)	0.04 (0.03 to 0.04)	7.2 (-9.8 to 26.4)
		Haemophilus influenzae	17.4 (15.1 to 19.8)	16 (13.3 to 18.9)	-8.2 (-19.8 to 5.2)	0.05 (0.05 to 0.05)	0.04 (0.03 to 0.04)	-28.2 (-38.2 to -16.6)
		Influenza	25.5 (22.3 to 28.9)	30.9 (16 to 45.4)	21.5 (-35.8 to 74.0)	0.22 (0.2 to 0.23)	0.19 (0.09 to 0.29)	-10.7 (-55.9 to 36.1)
		Klebsiella pneumoniae	8.32 (7.3 to 9.44)	7.23 (6.07 to 8.5)	-13.1 (-24.0 to -1.3)	0.09 (0.09 to 0.1)	0.06 (0.05 to 0.07)	-34.1 (-43.2 to -23.5)
		Legionella spp.	12.3 (10.7 to 14.2)	15.5 (12.9 to 18.2)	26.3 (9.8 to 46.5)	0.1 (0.09 to 0.11)	0.09 (0.08 to 0.1)	-4.2 (-17.8 to 13.4)
		Mycoplasma spp.	37 (32.5 to 42.4)	37.8 (31.8 to 44.6)	2.1 (-10.9 to 16.9)	0.11 (0.1 to 0.12)	0.09 (0.08 to 0.1)	-22.6 (-34.1 to -10.5)
		Polymicrobial	0.36 (0.21 to 0.6)	0.43 (0.23 to 0.75)	19.2 (2.3 to 39.0)	0 (0 to 0.01)	0 (0 to 0.01)	-9.6 (-23.8 to 6.8)
		Pseudomonas aeruginosa	13 (11.3 to 14.9)	19.5 (16.3 to 23)	50.4 (32.1 to 72.5)	0.1 (0.09 to 0.11)	0.12 (0.1 to 0.13)	14.0 (-1.8 to 31.3)
		Respiratory syncytial virus	10.7 (9.29 to 12.2)	11.9 (6.17 to 17.7)	11.6 (-40.8 to 60.7)	0.04 (0.04 to 0.05)	0.03 (0.02 to 0.05)	-18.0 (-59.9 to 25.9)
		Staphylococcus aureus	16.6 (14.6 to 18.8)	27.6 (23.3 to 32.1)	65.9 (47.4 to 86.8)	0.27 (0.25 to 0.29)	0.34 (0.3 to 0.38)	25.8 (8.5 to 45.2)
		Streptococcus pneumoniae	124 (109 to 140)	63.3 (53.5 to 74.1)	-48.9 (-54.6 to -42.3)	0.83 (0.77 to 0.89)	0.32 (0.29 to 0.36)	-61.2 (-66.4 to -55.2)

		Other viruses	91.8 (80.9 to 104)	81.7 (69.2 to 95.4)	-10.9 (-21.0 to 0.2)	0.24 (0.22 to 0.26)	0.16 (0.14 to 0.18)	-32.5 (-41.5 to -22.0)
		Other bacterial pathogens	11.3 (9.46 to 13.4)	10.1 (8.24 to 12.4)	-10.5 (-21.7 to 2.7)	0.07 (0.06 to 0.08)	0.05 (0.04 to 0.06)	-32.0 (-41.9 to -20.6)
	50-69 years	*Acinetobacter baumannii*	4.67 (3.89 to 5.53)	3.43 (2.78 to 4.14)	-26.5 (-36.7 to -15.0)	0.08 (0.07 to 0.1)	0.04 (0.04 to 0.05)	-48.4 (-56.2 to -39.3)
		Chlamydia spp.	18.3 (15.8 to 21)	14.9 (12.7 to 17.2)	-18.4 (-29.1 to -5.7)	0.08 (0.07 to 0.09)	0.05 (0.04 to 0.05)	-42.7 (-51.4 to -31.8)
		Enterobacter spp.	5.13 (4.37 to 6.01)	8.78 (7.32 to 10.2)	71.6 (47.0 to 98.1)	0.09 (0.08 to 0.1)	0.1 (0.09 to 0.12)	20.5 (1.6 to 43.1)
		Escherichia coli	11.3 (9.85 to 12.8)	16.9 (14.5 to 19.3)	50.1 (30.2 to 72.1)	0.2 (0.18 to 0.22)	0.21 (0.18 to 0.24)	5.4 (-10.6 to 23.8)
		Fungus	10.8 (9.31 to 12.5)	14.5 (12.4 to 16.9)	34.7 (15.7 to 55.9)	0.19 (0.16 to 0.21)	0.18 (0.15 to 0.2)	-5.4 (-20.4 to 11.8)
		Group B Streptococcus	10.3 (8.88 to 11.8)	15.2 (13 to 17.7)	48.0 (26.7 to 70.3)	0.13 (0.11 to 0.15)	0.13 (0.12 to 0.15)	3.9 (-12.8 to 24.0)
		Haemophilus influenzae	37.6 (33.3 to 42.3)	41.7 (36.1 to 47.8)	11.2 (-3.1 to 26.5)	0.17 (0.15 to 0.19)	0.14 (0.12 to 0.15)	-19.5 (-31.5 to -6.1)
		Influenza	47.4 (41.9 to 53.5)	65.4 (34.1 to 94.3)	38.1 (-28.4 to 95.9)	0.62 (0.56 to 0.68)	0.58 (0.28 to 0.89)	-5.9 (-53.9 to 46.2)
		Klebsiella pneumoniae	23 (20.5 to 26)	26.2 (22.6 to 29.9)	13.8 (-0.8 to 30.4)	0.4 (0.36 to 0.44)	0.32 (0.28 to 0.37)	-20.1 (-31.9 to -6.0)
		Legionella spp.	37.4 (32.8 to 42.4)	50.8 (43.9 to 58.4)	36.0 (17.6 to 56.4)	0.45 (0.4 to 0.5)	0.43 (0.38 to 0.49)	-4.5 (-18.9 to 12.3)
		Mycoplasma spp.	36.7 (32.4 to 41.6)	34.4 (29.5 to 39.7)	-6.1 (-18.5 to 6.6)	0.17 (0.15 to 0.19)	0.11 (0.1 to 0.13)	-34.1 (-44.4 to -22.3)
		Polymicrobial	1.11 (0.65 to 1.85)	1.46 (0.82 to 2.51)	31.5 (11.6 to 53.6)	0.02 (0.01 to 0.03)	0.02 (0.01 to 0.03)	-7.7 (-22.7 to 9.4)
		Pseudomonas aeruginosa	39.8 (35.3 to 45.2)	60.7 (52.6 to 69.7)	52.8 (33.0 to 74.6)	0.48 (0.44 to 0.54)	0.52 (0.45 to 0.59)	7.3 (-8.5 to 25.6)
		Respiratory syncytial virus	14.7 (12.8 to 16.7)	16.9 (8.86 to 24.3)	14.6 (-41.1 to 63.9)	0.09 (0.08 to 0.1)	0.07 (0.03 to 0.11)	-21.9 (-61.7 to 22.3)
		Staphylococcus aureus	44.8 (39.9 to 50.5)	81.7 (70.8 to 93.2)	82.6 (59.7 to 108.5)	1.12 (1.02 to 1.23)	1.43 (1.25 to 1.61)	28.2 (10.2 to 50.1)
		Streptococcus pneumoniae	165 (147 to 185)	103 (88.9 to 118)	-37.5 (-45.4 to -28.8)	1.71 (1.55 to 1.87)	0.75 (0.66 to 0.85)	-56.1 (-62.4 to -48.7)
		Other viruses	107 (95.2 to 120)	124 (107 to 141)	15.8 (1.2 to 31.9)	0.44 (0.4 to 0.48)	0.35 (0.31 to 0.4)	-18.7 (-30.3 to -5.1)
		Other bacterial pathogens	36.9 (31 to 43.2)	35.4 (29 to 42.3)	-4.0 (-16.2 to 9.9)	0.37 (0.31 to 0.42)	0.25 (0.21 to 0.3)	-32.5 (-43.1 to -20.4)
	70+ years	*Acinetobacter baumannii*	9.68 (8.11 to 11.4)	7.3 (5.89 to 8.84)	-24.5 (-35.2 to -11.6)	0.95 (0.78 to 1.11)	0.58 (0.45 to 0.7)	-38.5 (-46.2 to -30.5)
		Chlamydia spp.	45.2 (39.5 to 51.8)	37.9 (31.9 to 45)	-16.1 (-28.4 to -1.1)	1.1 (0.93 to 1.23)	0.75 (0.6 to 0.87)	-31.6 (-40.3 to -22.5)
		Enterobacter spp.	10.4 (8.9 to 12)	18.2 (15.1 to 21.8)	76.3 (49.9 to 105.6)	0.96 (0.8 to 1.11)	1.38 (1.04 to 1.63)	43.7 (24.9 to 63.1)
		Escherichia coli	28.7 (25.3 to 32.8)	44.1 (37.6 to 52)	54.2 (31.1 to 80.3)	2.75 (2.33 to 3.09)	3.46 (2.74 to 3.98)	25.7 (9.4 to 41.4)
		Fungus	12.5 (10.9 to 14.5)	17.2 (14.3 to 20.6)	38.3 (18.2 to 63.0)	1.18 (0.98 to 1.37)	1.33 (1.05 to 1.57)	12.8 (-2.0 to 28.7)
		Group B Streptococcus	19.6 (17.2 to 22.4)	29.7 (25.1 to 35.7)	52.0 (28.8 to 78.3)	1.34 (1.12 to 1.5)	1.66 (1.31 to 1.92)	23.9 (6.6 to 40.1)

		Haemophilus influenzae	70.2 (62.6 to 78.8)	80.2 (68.2 to 94.4)	14.3 (-1.3 to 33.6)	1.71 (1.45 to 1.89)	1.65 (1.32 to 1.9)	-4.0 (-15.5 to 7.9)
		Influenza	149 (134 to 168)	211 (111 to 305)	41.5 (-24.1 to 99.5)	10.7 (9.08 to 11.8)	12 (6 to 18.7)	12.2 (-44.2 to 69.2)
		Klebsiella pneumoniae	52.6 (47.2 to 58.7)	61.5 (52.4 to 72.4)	16.9 (1.1 to 35.3)	5.03 (4.24 to 5.54)	4.79 (3.8 to 5.52)	-4.7 (-15.3 to 6.2)
		Legionella spp.	55.8 (49.1 to 63.5)	77.8 (66.4 to 91.4)	39.7 (19.5 to 63.9)	3.67 (3.07 to 4.13)	4.18 (3.33 to 4.81)	13.9 (-0.4 to 28.6)
		Mycoplasma spp.	64.3 (57 to 72.3)	61.9 (52.4 to 73.1)	-3.5 (-17.1 to 12.4)	1.63 (1.38 to 1.81)	1.28 (1.02 to 1.47)	-21.4 (-30.4 to -11.8)
		Polymicrobial	2.64 (1.51 to 4.58)	3.58 (1.9 to 6.33)	35.1 (14.8 to 60.0)	0.26 (0.15 to 0.43)	0.28 (0.16 to 0.51)	10.1 (-5.4 to 25.5)
		Pseudomonas aeruginosa	86 (76.5 to 97.2)	135 (115 to 158)	57.0 (35.2 to 81.4)	5.7 (4.84 to 6.29)	7.3 (5.7 to 8.39)	27.9 (13.1 to 43.1)
		Respiratory syncytial virus	28.6 (25.4 to 33.1)	33.6 (18.1 to 48.8)	17.4 (-37.0 to 65.4)	0.94 (0.79 to 1.05)	0.87 (0.43 to 1.35)	-6.9 (-53.2 to 40.9)
		Staphylococcus aureus	120 (108 to 134)	225 (192 to 266)	87.6 (63.2 to 117.7)	16.3 (13.8 to 17.9)	25 (19.9 to 28.5)	52.9 (35.6 to 69.9)
		Streptococcus pneumoniae	352 (315 to 393)	226 (192 to 266)	-35.8 (-44.2 to -25.4)	19.9 (16.8 to 21.8)	10.4 (8.31 to 11.9)	-47.7 (-53.5 to -41.8)
		Other viruses	225 (202 to 251)	268 (228 to 316)	18.9 (3.3 to 37.9)	5.01 (4.23 to 5.49)	4.86 (3.89 to 5.53)	-3.1 (-14.0 to 7.6)
		Other bacterial pathogens	102 (88.1 to 119)	101 (83.7 to 122)	-1.4 (-15.1 to 14.5)	5.6 (4.61 to 6.51)	4.51 (3.42 to 5.44)	-19.6 (-29.5 to -9.4)
Ireland	All Ages	*Acinetobacter baumannii*	343 (293 to 399)	160 (138 to 185)	-53.3 (-58.5 to -47.3)	26.1 (22.3 to 30)	8.39 (6.51 to 9.79)	-67.8 (-72.7 to -63.5)
		Chlamydia spp.	1530 (1380 to 1690)	906 (823 to 1000)	-40.8 (-47.5 to -32.9)	28.4 (25.1 to 31.1)	11.5 (9.34 to 13.1)	-59.4 (-65.3 to -54.0)
		Enterobacter spp.	284 (249 to 326)	451 (401 to 508)	59.3 (39.0 to 81.3)	19.9 (17.3 to 22.6)	21.8 (17.4 to 25.2)	9.9 (-7.1 to 25.9)
		Escherichia coli	787 (709 to 876)	1030 (945 to 1130)	31.0 (16.0 to 47.1)	60.9 (54.8 to 66.9)	54.6 (43.9 to 61.4)	-10.4 (-23.2 to 0.7)
		Fungus	485 (430 to 549)	536 (478 to 600)	10.7 (-1.8 to 25.2)	29 (25.6 to 32.6)	22.1 (17.9 to 25.4)	-23.7 (-34.6 to -12.3)
		Group B Streptococcus	604 (542 to 672)	782 (705 to 861)	29.7 (14.2 to 46.0)	29.5 (26.1 to 32.5)	26.3 (21 to 29.9)	-10.8 (-23.7 to 2.0)
		Haemophilus influenzae	2450 (2250 to 2650)	2240 (2060 to 2460)	-8.5 (-17.2 to 0.1)	59.6 (54.5 to 63.4)	26.1 (21.3 to 29.3)	-56.2 (-61.8 to -51.3)
		Influenza	3830 (3520 to 4140)	492 (105 to 1430)	-87.1 (-97.3 to -63.5)	216 (198 to 230)	18.5 (3.84 to 54.4)	-91.4 (-98.2 to -75.0)
		Klebsiella pneumoniae	1590 (1470 to 1730)	1430 (1310 to 1560)	-10.3 (-18.8 to -1.4)	113 (103 to 121)	72.2 (58.6 to 81.1)	-36.3 (-45.1 to -29.4)
		Legionella spp.	1650 (1470 to 1830)	2140 (1970 to 2340)	30.1 (15.2 to 46.3)	74.8 (66.8 to 82.1)	66.8 (54.5 to 74.8)	-10.6 (-22.8 to 1.9)
		Mycoplasma spp.	2600 (2370 to 2820)	2130 (1960 to 2310)	-18.0 (-25.5 to -9.9)	39.2 (35.5 to 42.4)	20.9 (17.1 to 23.5)	-46.8 (-54.1 to -40.6)
		Polymicrobial	83.4 (50.2 to 137)	85.8 (48.8 to 145)	2.3 (-11.6 to 17.7)	6.4 (3.93 to 10.4)	4.55 (2.58 to 7.88)	-29.5 (-41.4 to -17.7)
		Pseudomonas aeruginosa	2180 (1980 to 2400)	3250 (2990 to 3540)	49.3 (33.7 to 65.7)	109 (99.1 to 118)	111 (88.5 to 124)	1.8 (-12.3 to 14.3)
		Respiratory syncytial virus	1380 (1250 to 1520)	146 (32.2 to 419)	-89.4 (-97.7 to -70.4)	20.4 (18.6 to 22)	1.41 (0.3 to 4.2)	-93.1 (-98.5 to -79.4)

		Staphylococcus aureus	2980 (2740 to 3230)	5210 (4800 to 5700)	75.1 (59.4 to 92.3)	322 (293 to 342)	388 (312 to 433)	20.6 (4.7 to 33.2)
		Streptococcus pneumoniae	13000 (12100 to 13900)	5710 (5280 to 6190)	-56.1 (-59.8 to -52.4)	476 (438 to 503)	152 (124 to 169)	-68.1 (-72.3 to -65.0)
		Other viruses	7800 (7270 to 8370)	7560 (6990 to 8220)	-3.0 (-11.1 to 4.6)	103 (95 to 109)	74.8 (61.6 to 83.3)	-27.7 (-37.0 to -20.7)
		Other bacterial pathogens	2860 (2500 to 3250)	2190 (1910 to 2530)	-23.3 (-31.3 to -14.3)	129 (113 to 145)	67.8 (52.2 to 79.9)	-47.5 (-55.2 to -41.1)
	Under 5	*Acinetobacter baumannii*	3.64 (2.42 to 5.39)	1.36 (0.85 to 2.13)	-63.0 (-69.5 to -55.3)	0.16 (0.09 to 0.27)	0.05 (0.02 to 0.09)	-69.0 (-77.1 to -60.8)
		Chlamydia spp.	57.3 (46.9 to 69.8)	23.8 (18.9 to 30.4)	-58.3 (-65.6 to -50.2)	0.26 (0.23 to 0.3)	0.06 (0.05 to 0.07)	-77.4 (-82.1 to -72.3)
		Enterobacter spp.	8.87 (6.69 to 11.8)	9.27 (6.71 to 12.8)	4.8 (-14.8 to 28.3)	0.17 (0.12 to 0.23)	0.08 (0.05 to 0.11)	-54.8 (-64.9 to -43.7)
		Escherichia coli	25.5 (20.6 to 31.6)	26.1 (20.1 to 32.9)	2.5 (-17.0 to 23.2)	0.47 (0.41 to 0.53)	0.19 (0.16 to 0.23)	-59.0 (-67.2 to -49.6)
		Fungus	4.97 (3.49 to 7.08)	3.81 (2.58 to 5.51)	-23.0 (-38.4 to -5.1)	0.06 (0.04 to 0.08)	0.02 (0.01 to 0.02)	-71.7 (-78.4 to -63.5)
		Group B Streptococcus	23.7 (18.9 to 29.5)	21.1 (16.3 to 27)	-10.8 (-28.2 to 6.9)	0.38 (0.33 to 0.43)	0.15 (0.12 to 0.18)	-60.8 (-68.7 to -52.2)
		Haemophilus influenzae	233 (189 to 286)	130 (102 to 164)	-44.2 (-54.0 to -33.1)	1.21 (1.07 to 1.36)	0.15 (0.12 to 0.18)	-87.6 (-90.5 to -84.6)
		Influenza	187 (152 to 227)	12.2 (2.72 to 34)	-93.4 (-98.6 to -82.5)	1.81 (1.63 to 2.02)	0.04 (0.01 to 0.12)	-97.6 (-99.5 to -93.1)
		Klebsiella pneumoniae	94.1 (77.1 to 115)	41.2 (32.5 to 52.3)	-56.1 (-63.6 to -47.8)	1.29 (1.15 to 1.43)	0.26 (0.22 to 0.31)	-79.6 (-83.7 to -75.1)
		Legionella spp.	12.8 (8.91 to 17.9)	11.5 (7.61 to 16.2)	-10.2 (-26.0 to 8.0)	0.21 (0.16 to 0.27)	0.1 (0.07 to 0.13)	-54.4 (-63.9 to -43.6)
		Mycoplasma spp.	127 (103 to 156)	118 (93.2 to 149)	-6.8 (-22.6 to 9.9)	0.44 (0.39 to 0.5)	0.15 (0.12 to 0.18)	-65.4 (-72.8 to -56.9)
		Polymicrobial	4.05 (2.35 to 6.65)	2.75 (1.49 to 4.55)	-32.3 (-42.8 to -20.1)	0.24 (0.12 to 0.41)	0.12 (0.06 to 0.22)	-50.1 (-62.9 to -36.0)
		Pseudomonas aeruginosa	75.7 (61.2 to 94)	100 (79.9 to 126)	33.0 (9.1 to 56.8)	0.83 (0.73 to 0.93)	0.42 (0.34 to 0.5)	-49.6 (-60.0 to -38.2)
		Respiratory syncytial virus	605 (502 to 728)	55.9 (12.5 to 156)	-90.7 (-97.9 to -74.5)	2.95 (2.68 to 3.26)	0.1 (0.02 to 0.3)	-96.5 (-99.3 to -89.9)
		Staphylococcus aureus	82.6 (67.9 to 99.7)	92.5 (73.7 to 116)	12.1 (-5.5 to 29.9)	1.58 (1.43 to 1.74)	0.68 (0.56 to 0.82)	-56.7 (-65.1 to -47.1)
		Streptococcus pneumoniae	1180 (972 to 1440)	218 (174 to 277)	-81.6 (-84.4 to -78.5)	8.33 (7.55 to 9.25)	0.56 (0.46 to 0.67)	-93.3 (-94.6 to -91.7)
		Other viruses	1210 (997 to 1470)	594 (476 to 751)	-51.0 (-58.3 to -43.0)	3.45 (3.13 to 3.83)	0.7 (0.58 to 0.83)	-79.6 (-83.5 to -75.1)
		Other bacterial pathogens	27.6 (21 to 35.8)	15.4 (11.2 to 20.6)	-44.3 (-55.1 to -33.0)	0.25 (0.19 to 0.32)	0.06 (0.04 to 0.09)	-74.4 (-80.1 to -67.4)
	5-14 years	*Acinetobacter baumannii*	7.63 (5.54 to 10.4)	3.02 (2.2 to 4.09)	-60.4 (-65.5 to -55.1)	0.05 (0.04 to 0.06)	0.01 (0 to 0.01)	-88.9 (-90.7 to -86.7)
		Chlamydia spp.	47.7 (35.3 to 62.7)	25.3 (18.6 to 33.5)	-46.9 (-52.9 to -40.4)	0.08 (0.07 to 0.09)	0.01 (0.01 to 0.01)	-85.1 (-87.6 to -82.0)

		Enterobacter spp.	6.45 (4.64 to 8.77)	9.04 (6.55 to 12.1)	40.6 (22.1 to 60.4)	0.04 (0.03 to 0.05)	0.02 (0.01 to 0.02)	-60.6 (-67.3 to -52.8)
		Escherichia coli	14.1 (10.4 to 18.4)	16.7 (12.5 to 22)	18.9 (4.7 to 33.6)	0.09 (0.08 to 0.1)	0.03 (0.03 to 0.03)	-66.6 (-72.0 to -60.5)
		Fungus	16.9 (12.5 to 22.5)	16.7 (12.4 to 22.4)	-1.1 (-13.0 to 12.3)	0.11 (0.09 to 0.12)	0.03 (0.03 to 0.03)	-72.3 (-76.8 to -67.0)
		Group B Streptococcus	19.2 (14.1 to 25.2)	22.5 (16.5 to 29.8)	17.4 (1.9 to 34.4)	0.09 (0.08 to 0.1)	0.03 (0.02 to 0.03)	-67.1 (-72.9 to -60.1)
		Haemophilus influenzae	84.8 (63.9 to 111)	65.8 (49.2 to 87.1)	-22.4 (-30.0 to -13.6)	0.21 (0.19 to 0.23)	0.03 (0.03 to 0.03)	-85.3 (-87.5 to -82.7)
		Influenza	116 (87.1 to 150)	12.4 (2.52 to 35.8)	-89.2 (-97.7 to -69.3)	0.56 (0.5 to 0.61)	0.02 (0 to 0.05)	-97.1 (-99.4 to -91.6)
		Klebsiella pneumoniae	41.1 (31 to 53)	28.8 (21.7 to 37.6)	-29.9 (-36.3 to -22.6)	0.26 (0.24 to 0.29)	0.05 (0.05 to 0.06)	-80.3 (-83.2 to -76.7)
		Legionella spp.	52.6 (39.5 to 69.1)	60.9 (45.8 to 79.4)	16.0 (1.8 to 31.7)	0.23 (0.2 to 0.26)	0.08 (0.07 to 0.09)	-67.5 (-72.7 to -61.4)
		Mycoplasma spp.	181 (134 to 236)	143 (107 to 188)	-21.2 (-29.5 to -12.8)	0.31 (0.28 to 0.34)	0.07 (0.06 to 0.08)	-77.9 (-81.3 to -74.0)
		Polymicrobial	1.93 (1.11 to 3.14)	1.72 (0.93 to 2.92)	-11.3 (-23.6 to 2.8)	0.01 (0.01 to 0.02)	0 (0 to 0.01)	-75.1 (-79.6 to -69.8)
		Pseudomonas aeruginosa	54.5 (40.8 to 70.9)	75.1 (56.1 to 99.5)	37.9 (23.3 to 52.6)	0.24 (0.22 to 0.27)	0.09 (0.08 to 0.11)	-61.3 (-67.0 to -54.2)
		Respiratory syncytial virus	42.3 (31.7 to 55.3)	4.44 (0.92 to 13)	-89.4 (-97.7 to -69.9)	0.09 (0.08 to 0.1)	0 (0 to 0.01)	-97.2 (-99.4 to -91.7)
		Staphylococcus aureus	73.5 (55.7 to 95.3)	110 (83.4 to 144)	50.2 (36.6 to 65.6)	0.67 (0.61 to 0.73)	0.28 (0.25 to 0.32)	-57.9 (-63.9 to -50.6)
		Streptococcus pneumoniae	629 (475 to 811)	230 (174 to 300)	-63.4 (-66.7 to -59.8)	2.38 (2.17 to 2.59)	0.24 (0.22 to 0.28)	-89.7 (-91.1 to -88.0)
		Other viruses	392 (296 to 508)	320 (242 to 420)	-18.2 (-25.6 to -10.1)	0.58 (0.53 to 0.64)	0.13 (0.12 to 0.15)	-77.0 (-80.4 to -72.9)
		Other bacterial pathogens	59.1 (43.8 to 78.6)	39.4 (28.7 to 52.9)	-33.2 (-40.3 to -25.6)	0.21 (0.18 to 0.24)	0.04 (0.03 to 0.05)	-81.2 (-84.2 to -77.7)
	15-49 years	*Acinetobacter baumannii*	21.4 (17.4 to 25.7)	10.4 (8.33 to 12.6)	-51.3 (-57.2 to -44.4)	0.35 (0.3 to 0.41)	0.11 (0.09 to 0.12)	-69.8 (-73.9 to -65.5)
		Chlamydia spp.	133 (115 to 156)	87.1 (73.4 to 103)	-34.6 (-41.8 to -26.1)	0.54 (0.49 to 0.6)	0.22 (0.2 to 0.25)	-59.5 (-64.7 to -54.0)
		Enterobacter spp.	18 (15.1 to 21.2)	31.2 (25.4 to 37.6)	73.0 (51.2 to 96.2)	0.28 (0.24 to 0.32)	0.3 (0.26 to 0.34)	7.1 (-7.4 to 22.4)
		Escherichia coli	39.4 (33.6 to 45.5)	57.6 (48.8 to 67.2)	46.3 (30.0 to 63.5)	0.64 (0.57 to 0.7)	0.57 (0.52 to 0.63)	-9.5 (-20.2 to 2.9)
		Fungus	47.3 (40 to 55)	57.5 (48.2 to 68.1)	21.7 (6.7 to 38.1)	0.75 (0.67 to 0.84)	0.56 (0.5 to 0.64)	-24.7 (-34.5 to -14.1)
		Group B Streptococcus	53.7 (45.9 to 63.5)	77.4 (65.1 to 92.4)	44.5 (26.9 to 63.6)	0.61 (0.55 to 0.69)	0.55 (0.48 to 0.62)	-10.6 (-22.1 to 3.1)
		Haemophilus influenzae	237 (206 to 270)	227 (193 to 264)	-4.5 (-14.2 to 5.1)	1.46 (1.35 to 1.58)	0.58 (0.52 to 0.64)	-60.1 (-64.6 to -55.7)
		Influenza	324 (282 to 366)	42.8 (9.44 to 122)	-86.8 (-97.2 to -62.2)	3.89 (3.64 to 4.17)	0.3 (0.06 to 0.89)	-92.2 (-98.4 to -77.7)

		Klebsiella pneumoniae	115 (101 to 130)	99.3 (84.4 to 115)	-13.7 (-21.8 to -4.4)	1.84 (1.72 to 1.98)	0.98 (0.89 to 1.07)	-46.6 (-52.1 to -40.5)
		Legionella spp.	147 (126 to 169)	210 (178 to 242)	42.8 (26.4 to 62.2)	1.62 (1.47 to 1.79)	1.43 (1.29 to 1.57)	-11.7 (-22.7 to 0.2)
		Mycoplasma spp.	507 (443 to 577)	492 (419 to 570)	-3.0 (-13.0 to 7.1)	2.16 (2 to 2.33)	1.29 (1.17 to 1.42)	-40.0 (-46.6 to -32.9)
		Polymicrobial	5.42 (3.23 to 8.92)	5.95 (3.19 to 10.2)	9.2 (-6.5 to 26.8)	0.09 (0.05 to 0.14)	0.06 (0.03 to 0.1)	-32.4 (-42.5 to -21.5)
		Pseudomonas aeruginosa	153 (132 to 175)	259 (221 to 301)	69.8 (53.3 to 89.3)	1.7 (1.56 to 1.84)	1.78 (1.61 to 1.95)	5.1 (-7.0 to 17.2)
		Respiratory syncytial virus	118 (103 to 135)	15.4 (3.32 to 43.9)	-87.0 (-97.2 to -62.4)	0.65 (0.59 to 0.71)	0.05 (0.01 to 0.14)	-92.3 (-98.4 to -77.6)
		Staphylococcus aureus	206 (180 to 233)	380 (324 to 440)	84.8 (68.8 to 102.4)	4.68 (4.4 to 4.99)	5.35 (4.88 to 5.83)	14.4 (2.9 to 26.1)
		Streptococcus pneumoniae	1760 (1550 to 1990)	792 (677 to 916)	-55.0 (-58.7 to -50.9)	16.7 (15.7 to 17.7)	4.64 (4.23 to 5.05)	-72.1 (-74.9 to -69.4)
		Other viruses	1100 (967 to 1240)	1100 (942 to 1270)	0.7 (-8.1 to 10.0)	4.08 (3.84 to 4.35)	2.54 (2.32 to 2.76)	-37.7 (-44.0 to -31.3)
		Other bacterial pathogens	165 (137 to 195)	136 (112 to 165)	-17.8 (-25.8 to -7.4)	1.5 (1.31 to 1.71)	0.76 (0.65 to 0.89)	-49.0 (-55.2 to -42.9)
	50-69 years	*Acinetobacter baumannii*	105 (88.9 to 124)	41.6 (35.2 to 48.3)	-60.4 (-65.5 to -54.3)	2.57 (2.2 to 2.99)	0.56 (0.47 to 0.65)	-78.3 (-81.4 to -75.0)
		Chlamydia spp.	389 (342 to 445)	194 (171 to 219)	-50.1 (-56.7 to -42.5)	2.36 (2.1 to 2.64)	0.64 (0.57 to 0.73)	-72.7 (-76.3 to -68.6)
		Enterobacter spp.	86.4 (74.6 to 101)	117 (101 to 134)	36.0 (16.3 to 57.0)	2 (1.73 to 2.28)	1.48 (1.28 to 1.68)	-25.6 (-36.1 to -14.4)
		Escherichia coli	209 (183 to 237)	228 (203 to 255)	9.9 (-4.3 to 26.1)	4.98 (4.48 to 5.52)	2.99 (2.7 to 3.31)	-39.9 (-48.0 to -31.4)
		Fungus	199 (173 to 230)	191 (166 to 220)	-3.6 (-16.5 to 11.6)	4.69 (4.12 to 5.35)	2.47 (2.16 to 2.82)	-47.2 (-54.7 to -38.8)
		Group B Streptococcus	182 (158 to 210)	201 (177 to 226)	10.8 (-4.2 to 28.3)	3.09 (2.74 to 3.49)	1.87 (1.65 to 2.09)	-39.4 (-47.9 to -29.7)
		Haemophilus influenzae	686 (614 to 771)	559 (498 to 620)	-18.4 (-28.6 to -7.4)	6.26 (5.75 to 6.79)	1.88 (1.7 to 2.08)	-69.9 (-73.5 to -66.0)
		Influenza	807 (724 to 903)	88.5 (18.5 to 254)	-89.0 (-97.7 to -69.2)	14.4 (13.3 to 15.7)	0.82 (0.17 to 2.42)	-94.3 (-98.8 to -83.3)
		Klebsiella pneumoniae	426 (385 to 475)	336 (300 to 372)	-21.1 (-30.3 to -10.3)	10.1 (9.31 to 11.1)	4.37 (3.94 to 4.83)	-56.8 (-62.0 to -51.1)
		Legionella spp.	598 (526 to 677)	665 (597 to 737)	11.6 (-3.6 to 28.4)	9.79 (8.8 to 10.9)	5.96 (5.38 to 6.6)	-39.0 (-47.5 to -29.8)
		Mycoplasma spp.	672 (597 to 759)	442 (394 to 490)	-34.1 (-42.6 to -24.3)	4.24 (3.84 to 4.71)	1.53 (1.37 to 1.69)	-64.0 (-68.4 to -58.8)
		Polymicrobial	22.2 (13.3 to 35.7)	19.5 (11 to 33.4)	-12.6 (-26.0 to 3.2)	0.54 (0.32 to 0.88)	0.26 (0.14 to 0.44)	-52.2 (-60.1 to -43.4)
		Pseudomonas aeruginosa	625 (556 to 709)	782 (697 to 864)	25.3 (10.1 to 43.0)	10.3 (9.41 to 11.3)	7.06 (6.37 to 7.75)	-31.4 (-40.1 to -21.4)
		Respiratory syncytial virus	218 (191 to 249)	21.2 (4.48 to 60.6)	-90.3 (-98.0 to -72.5)	1.78 (1.58 to 2)	0.09 (0.02 to 0.26)	-94.9 (-98.9 to -85.1)

		Staphylococcus aureus	742 (668 to 828)	1090 (980 to 1210)	47.6 (30.7 to 68.8)	25.1 (23.1 to 27.3)	20.2 (18.3 to 22.2)	-19.2 (-28.3 to -8.8)
		Streptococcus pneumoniae	3130 (2820 to 3490)	1250 (1120 to 1380)	-60.0 (-64.6 to -54.6)	44 (40.7 to 47.9)	9.63 (8.72 to 10.6)	-78.1 (-80.6 to -75.3)
		Other viruses	1710 (1540 to 1910)	1570 (1410 to 1730)	-8.1 (-18.7 to 4.2)	9.44 (8.68 to 10.3)	4.74 (4.3 to 5.2)	-49.7 (-55.5 to -43.3)
		Other bacterial pathogens	722 (618 to 836)	461 (391 to 536)	-36.1 (-44.0 to -26.8)	9.7 (8.38 to 11.1)	3.39 (2.89 to 3.98)	-65.0 (-69.2 to -60.2)
	70+ years	*Acinetobacter baumannii*	206 (173 to 241)	104 (88.5 to 122)	-49.4 (-55.6 to -42.4)	22.9 (19.4 to 26.6)	7.67 (5.86 to 8.99)	-66.5 (-71.7 to -61.9)
		Chlamydia spp.	906 (798 to 1010)	576 (512 to 659)	-36.3 (-44.3 to -26.7)	25.1 (22.1 to 27.8)	10.6 (8.43 to 12.1)	-57.9 (-64.2 to -51.9)
		Enterobacter spp.	164 (141 to 191)	285 (248 to 331)	73.8 (49.1 to 100.0)	17.4 (15 to 19.9)	19.9 (15.6 to 23.2)	14.9 (-3.9 to 32.6)
		Escherichia coli	500 (441 to 563)	700 (634 to 790)	40.5 (23.2 to 59.9)	54.7 (49 to 60.3)	50.8 (40.4 to 57.5)	-7.2 (-21.2 to 4.8)
		Fungus	217 (188 to 247)	266 (233 to 306)	23.2 (7.2 to 41.6)	23.4 (20.4 to 26.5)	19 (14.9 to 22.1)	-18.5 (-31.0 to -6.1)
		Group B Streptococcus	326 (288 to 369)	460 (408 to 523)	41.6 (23.3 to 61.2)	25.3 (22.2 to 28.1)	23.7 (18.6 to 27.2)	-6.4 (-20.5 to 7.6)
		Haemophilus influenzae	1210 (1090 to 1330)	1260 (1140 to 1420)	4.3 (-7.3 to 17.3)	50.4 (45.6 to 54)	23.5 (18.8 to 26.5)	-53.5 (-59.8 to -48.1)
		Influenza	2400 (2180 to 2620)	336 (69.9 to 974)	-86.0 (-97.0 to -60.0)	196 (177 to 209)	17.3 (3.6 to 50.8)	-91.2 (-98.2 to -74.0)
		Klebsiella pneumoniae	917 (838 to 1010)	924 (832 to 1040)	0.8 (-10.1 to 13.4)	99.8 (90.2 to 107)	66.6 (53.1 to 75.2)	-33.4 (-42.9 to -25.6)
		Legionella spp.	840 (736 to 943)	1190 (1080 to 1350)	42.6 (24.3 to 63.1)	62.9 (55.7 to 69.4)	59.3 (47.2 to 67.1)	-5.8 (-19.7 to 8.1)
		Mycoplasma spp.	1110 (997 to 1230)	933 (837 to 1060)	-15.8 (-25.5 to -4.4)	32.1 (28.6 to 35)	17.9 (14.2 to 20.3)	-44.4 (-53.0 to -37.3)
		Polymicrobial	49.8 (29.2 to 83.3)	55.8 (30.4 to 96)	11.7 (-4.6 to 29.7)	5.52 (3.28 to 9.16)	4.1 (2.24 to 7.24)	-26.2 (-39.3 to -13.3)
		Pseudomonas aeruginosa	1270 (1140 to 1410)	2030 (1830 to 2280)	60.2 (41.7 to 81.1)	96 (86.8 to 104)	102 (80.1 to 115)	5.9 (-9.4 to 19.3)
		Respiratory syncytial virus	400 (355 to 448)	49.6 (10.5 to 146)	-87.6 (-97.4 to -64.5)	14.9 (13.2 to 16.4)	1.17 (0.25 to 3.45)	-92.2 (-98.4 to -76.7)
		Staphylococcus aureus	1870 (1710 to 2050)	3530 (3200 to 3970)	88.7 (69.4 to 111.7)	290 (262 to 309)	362 (287 to 406)	24.7 (8.1 to 38.4)
		Streptococcus pneumoniae	6290 (5760 to 6880)	3210 (2910 to 3620)	-48.9 (-54.2 to -42.9)	405 (367 to 431)	137 (109 to 154)	-66.2 (-70.9 to -62.7)
		Other viruses	3390 (3100 to 3710)	3980 (3590 to 4490)	17.5 (5.1 to 31.6)	85.8 (77.6 to 91.4)	66.7 (53.6 to 75)	-22.4 (-33.0 to -14.1)
		Other bacterial pathogens	1890 (1640 to 2160)	1540 (1320 to 1790)	-18.3 (-27.7 to -7.5)	117 (102 to 132)	63.5 (48.1 to 75.3)	-45.9 (-54.1 to -39.1)
Israel	All Ages	*Acinetobacter baumannii*	294 (254 to 338)	284 (249 to 322)	-3.1 (-12.8 to 8.2)	11.4 (9.77 to 13.1)	12.6 (9.94 to 14.7)	10.8 (-4.5 to 25.1)
		Chlamydia spp.	1270 (1150 to 1400)	1520 (1390 to 1670)	20.1 (9.0 to 32.3)	11.4 (10 to 12.5)	15.7 (12.8 to 17.8)	38.3 (19.9 to 56.9)
		Enterobacter spp.	202 (176 to 230)	643 (581 to 712)	218.4 (186.1 to 254.4)	7.03 (6.01 to 8.04)	25.9 (20.6 to 29.4)	268.3 (217.1 to 316.3)

		Escherichia coli	581 (524 to 647)	1580 (1460 to 1730)	172.6 (148.0 to 200.3)	22.5 (19.8 to 24.8)	70.1 (56.7 to 78.4)	211.3 (170.8 to 250.0)
		Fungus	371 (328 to 419)	837 (757 to 926)	125.8 (102.0 to 150.4)	10.9 (9.57 to 12.2)	28.2 (22.8 to 32.2)	159.2 (123.5 to 193.2)
		Group B Streptococcus	461 (415 to 518)	1180 (1080 to 1300)	157.1 (129.3 to 186.0)	10.9 (9.52 to 12.1)	32.1 (26.2 to 36.4)	194.6 (153.5 to 234.9)
		Haemophilus influenzae	2000 (1850 to 2170)	3390 (3140 to 3680)	69.7 (57.9 to 82.2)	22 (19.8 to 23.6)	30.7 (25.2 to 34.2)	39.3 (22.7 to 53.6)
		Influenza	2860 (2650 to 3100)	474 (251 to 808)	-83.4 (-91.3 to -71.4)	77.2 (69.5 to 82.7)	14.9 (7.54 to 25)	-80.7 (-90.1 to -67.9)
		Klebsiella pneumoniae	1260 (1170 to 1370)	2150 (1990 to 2340)	69.9 (58.5 to 82.1)	42.8 (38.6 to 45.8)	89.8 (73.2 to 100)	109.8 (84.2 to 129.6)
		Legionella spp.	1080 (978 to 1200)	2750 (2550 to 2990)	153.6 (129.4 to 179.5)	24.4 (21.7 to 27)	70.7 (58.1 to 78.7)	189.9 (151.3 to 228.3)
		Mycoplasma spp.	2170 (2000 to 2370)	3480 (3210 to 3780)	60.3 (48.6 to 72.4)	14.6 (13.2 to 15.9)	24.3 (20 to 27.1)	66.5 (46.1 to 83.9)
		Polymicrobial	68.7 (44.1 to 107)	144 (84.8 to 235)	108.2 (80.6 to 139.4)	2.68 (1.75 to 4.16)	6.28 (3.58 to 10.8)	131.8 (90.5 to 170.7)
		Pseudomonas aeruginosa	1480 (1350 to 1630)	4280 (3970 to 4660)	189.2 (166.8 to 213.0)	36.4 (32.7 to 39.5)	119 (97.3 to 133)	227.3 (183.6 to 264.2)
		Respiratory syncytial virus	1570 (1390 to 1770)	175 (91.5 to 298)	-88.8 (-94.1 to -81.0)	9.38 (8.68 to 10.1)	0.95 (0.48 to 1.6)	-89.9 (-94.9 to -83.1)
		Staphylococcus aureus	2080 (1920 to 2260)	7280 (6760 to 7950)	250.5 (229.0 to 275.1)	111 (99.3 to 119)	455 (369 to 506)	308.3 (260.5 to 347.1)
		Streptococcus pneumoniae	11100 (10400 to 12100)	8340 (7760 to 9010)	-25.0 (-29.1 to -20.5)	180 (164 to 192)	174 (143 to 194)	-3.3 (-14.6 to 5.5)
		Other viruses	6660 (6150 to 7220)	10500 (9730 to 11300)	57.1 (48.8 to 66.8)	37.2 (34 to 39.4)	77.2 (63.8 to 85.6)	107.4 (82.6 to 127.6)
		Other bacterial pathogens	2060 (1820 to 2320)	3250 (2830 to 3720)	58.3 (44.2 to 73.7)	47.5 (40.9 to 53.6)	86 (66.8 to 102)	81.0 (56.9 to 103.4)
	Under 5	*Acinetobacter baumannii*	8.52 (5.77 to 12.2)	4.84 (3.19 to 7.3)	-43.4 (-51.5 to -34.7)	0.22 (0.13 to 0.36)	0.05 (0.02 to 0.09)	-78.0 (-83.9 to -70.7)
		Chlamydia spp.	123 (101 to 148)	84.3 (68 to 104)	-31.2 (-41.8 to -20.0)	0.39 (0.35 to 0.44)	0.07 (0.05 to 0.08)	-82.8 (-87.1 to -77.8)
		Enterobacter spp.	16.9 (12.6 to 21.9)	28.4 (21.1 to 37.9)	68.9 (40.6 to 102.2)	0.22 (0.16 to 0.3)	0.08 (0.05 to 0.11)	-65.3 (-73.8 to -55.2)
		Escherichia coli	51.1 (41.7 to 62.1)	87.3 (69.8 to 107)	71.4 (43.8 to 102.0)	0.65 (0.57 to 0.75)	0.22 (0.16 to 0.27)	-66.8 (-75.3 to -57.3)
		Fungus	9.86 (6.88 to 13.7)	12.6 (8.76 to 17.7)	28.1 (2.5 to 54.9)	0.09 (0.06 to 0.12)	0.02 (0.01 to 0.03)	-75.5 (-82.6 to -66.6)
		Group B Streptococcus	46.8 (37.8 to 57.1)	66.9 (52.7 to 83.4)	43.5 (19.5 to 68.3)	0.5 (0.44 to 0.57)	0.15 (0.12 to 0.19)	-69.9 (-77.4 to -61.2)
		Haemophilus influenzae	438 (360 to 526)	398 (321 to 488)	-8.9 (-22.5 to 5.5)	1.7 (1.48 to 1.91)	0.18 (0.14 to 0.22)	-89.4 (-92.4 to -86.0)
		Influenza	356 (293 to 426)	25.7 (12.9 to 45)	-92.8 (-96.3 to -87.8)	2.6 (2.31 to 2.92)	0.03 (0.02 to 0.06)	-98.7 (-99.4 to -97.6)
		Klebsiella pneumoniae	188 (154 to 223)	133 (108 to 163)	-29.1 (-39.6 to -18.8)	1.9 (1.68 to 2.12)	0.3 (0.23 to 0.37)	-84.1 (-88.1 to -79.6)

		Legionella spp.	23 (16.4 to 31.7)	31.7 (21.2 to 44.9)	37.6 (15.8 to 61.1)	0.26 (0.2 to 0.33)	0.09 (0.06 to 0.12)	-66.9 (-75.3 to -57.5)
		Mycoplasma spp.	243 (198 to 294)	365 (296 to 453)	51.0 (28.2 to 76.0)	0.64 (0.56 to 0.73)	0.18 (0.14 to 0.23)	-71.3 (-78.6 to -62.1)
		Polymicrobial	8.4 (4.93 to 13.5)	8.35 (4.76 to 13.5)	-0.6 (-14.3 to 14.2)	0.29 (0.16 to 0.47)	0.1 (0.05 to 0.18)	-65.3 (-74.8 to -53.2)
		Pseudomonas aeruginosa	136 (112 to 164)	283 (231 to 344)	108.8 (79.4 to 143.8)	1.08 (0.96 to 1.23)	0.43 (0.32 to 0.53)	-60.2 (-70.0 to -48.3)
		Respiratory syncytial virus	1050 (879 to 1240)	99.4 (50.2 to 172)	-90.5 (-95.1 to -83.8)	3.78 (3.4 to 4.19)	0.07 (0.03 to 0.12)	-98.2 (-99.1 to -96.8)
		Staphylococcus aureus	154 (128 to 183)	282 (228 to 346)	83.1 (59.6 to 107.2)	2.18 (1.96 to 2.43)	0.77 (0.59 to 0.96)	-64.4 (-73.5 to -54.7)
		Streptococcus pneumoniae	2280 (1880 to 2710)	644 (523 to 790)	-71.7 (-75.1 to -68.4)	12.2 (10.8 to 13.6)	0.65 (0.49 to 0.82)	-94.6 (-96.1 to -93.0)
		Other viruses	2120 (1760 to 2510)	1590 (1300 to 1950)	-24.8 (-33.8 to -16.4)	4.57 (4.08 to 5.07)	0.72 (0.54 to 0.89)	-84.3 (-88.3 to -79.7)
		Other bacterial pathogens	55.7 (42.4 to 71.1)	50.8 (37.9 to 66.6)	-8.5 (-24.1 to 7.7)	0.36 (0.28 to 0.46)	0.07 (0.05 to 0.1)	-79.8 (-85.3 to -73.7)
	5-14 years	*Acinetobacter baumannii*	12.6 (9.19 to 16.9)	9.42 (6.94 to 12.6)	-25.1 (-34.4 to -14.9)	0.04 (0.04 to 0.05)	0.01 (0.01 to 0.01)	-72.8 (-78.5 to -66.3)
		Chlamydia spp.	71.3 (52.9 to 94.4)	72.6 (54.3 to 97.1)	2.1 (-9.1 to 13.8)	0.06 (0.05 to 0.07)	0.02 (0.02 to 0.03)	-63.0 (-70.6 to -53.7)
		Enterobacter spp.	8.53 (6.22 to 11.5)	22.4 (16.3 to 29.9)	163.1 (128.7 to 199.3)	0.03 (0.02 to 0.03)	0.03 (0.02 to 0.03)	-4.6 (-24.6 to 19.5)
		Escherichia coli	19.7 (14.4 to 25.8)	45.4 (33.9 to 59.6)	131.2 (106.2 to 158.7)	0.07 (0.06 to 0.08)	0.06 (0.05 to 0.07)	-16.2 (-32.7 to 4.1)
		Fungus	23.6 (17.3 to 31.6)	44.1 (32.5 to 59.2)	86.9 (62.8 to 112.3)	0.08 (0.07 to 0.09)	0.05 (0.04 to 0.06)	-32.2 (-46.7 to -16.3)
		Group B Streptococcus	26.1 (18.9 to 34.6)	57.7 (43 to 77.9)	121.5 (91.6 to 150.3)	0.06 (0.05 to 0.07)	0.05 (0.04 to 0.06)	-19.6 (-36.1 to 1.3)
		Haemophilus influenzae	112 (85.5 to 147)	160 (119 to 213)	42.9 (28.5 to 57.7)	0.14 (0.13 to 0.16)	0.05 (0.04 to 0.06)	-65.2 (-71.8 to -57.5)
		Influenza	155 (116 to 200)	20.5 (10.1 to 37.3)	-86.8 (-92.9 to -77.4)	0.39 (0.35 to 0.43)	0.02 (0.01 to 0.03)	-95.3 (-97.7 to -91.6)
		Klebsiella pneumoniae	57.6 (43.4 to 75.1)	72.8 (55 to 95.8)	26.5 (15.3 to 38.4)	0.19 (0.17 to 0.22)	0.09 (0.07 to 0.11)	-54.1 (-62.8 to -43.4)
		Legionella spp.	64.5 (48.4 to 85.2)	134 (101 to 176)	109.0 (83.8 to 135.6)	0.15 (0.13 to 0.17)	0.11 (0.09 to 0.14)	-24.2 (-39.2 to -6.0)
		Mycoplasma spp.	243 (179 to 318)	361 (272 to 479)	49.0 (34.4 to 64.7)	0.22 (0.19 to 0.24)	0.12 (0.1 to 0.14)	-45.9 (-56.2 to -33.3)
		Polymicrobial	2.89 (1.72 to 4.67)	5.01 (2.75 to 8.44)	72.6 (47.5 to 99.4)	0.01 (0.01 to 0.02)	0.01 (0 to 0.01)	-37.4 (-50.8 to -21.2)
		Pseudomonas aeruginosa	68.2 (50.9 to 88.8)	171 (128 to 224)	151.4 (125.9 to 177.0)	0.16 (0.14 to 0.18)	0.14 (0.12 to 0.17)	-8.8 (-26.3 to 11.7)
		Respiratory syncytial virus	51.2 (38.1 to 66.9)	6.29 (3.06 to 11.5)	-87.7 (-93.6 to -78.5)	0.06 (0.05 to 0.07)	0 (0 to 0)	-95.7 (-97.9 to -92.2)
		Staphylococcus aureus	96.4 (72.3 to 126)	270 (203 to 352)	179.8 (155.3 to 203.2)	0.46 (0.41 to 0.51)	0.47 (0.39 to 0.56)	1.5 (-16.6 to 24.6)

		Streptococcus pneumoniae	853 (645 to 1110)	536 (404 to 709)	-37.1 (-42.6 to -31.9)	1.7 (1.52 to 1.88)	0.39 (0.32 to 0.46)	-77.2 (-81.2 to -71.9)
		Other viruses	481 (362 to 625)	672 (511 to 889)	39.8 (27.8 to 51.1)	0.38 (0.34 to 0.42)	0.19 (0.16 to 0.23)	-49.3 (-58.4 to -37.7)
		Other bacterial pathogens	83.5 (62 to 111)	105 (76.9 to 139)	26.0 (12.1 to 39.9)	0.16 (0.14 to 0.18)	0.07 (0.06 to 0.09)	-54.2 (-63.1 to -43.5)
	15-49 years	*Acinetobacter baumannii*	28.5 (22.7 to 34.8)	24.6 (20 to 29.8)	-13.4 (-23.1 to -2.8)	0.23 (0.2 to 0.27)	0.12 (0.1 to 0.14)	-47.3 (-54.3 to -39.6)
		Chlamydia spp.	161 (135 to 191)	190 (161 to 223)	18.1 (5.9 to 31.0)	0.32 (0.29 to 0.36)	0.23 (0.2 to 0.26)	-28.1 (-37.5 to -18.3)
		Enterobacter spp.	19.2 (15.7 to 23.4)	58.4 (48.7 to 69.7)	204.3 (171.8 to 242.9)	0.15 (0.13 to 0.17)	0.27 (0.24 to 0.31)	85.3 (60.4 to 112.2)
		Escherichia coli	44.4 (37.3 to 52.2)	118 (101 to 139)	167.5 (141.2 to 195.4)	0.35 (0.32 to 0.38)	0.57 (0.52 to 0.63)	62.9 (42.8 to 84.9)
		Fungus	53.3 (44.2 to 63.4)	115 (97.1 to 136)	116.2 (92.0 to 141.2)	0.42 (0.37 to 0.46)	0.55 (0.48 to 0.62)	31.7 (13.4 to 50.4)
		Group B Streptococcus	58.9 (49.3 to 71.3)	151 (128 to 178)	156.3 (127.4 to 187.0)	0.33 (0.29 to 0.37)	0.51 (0.45 to 0.58)	56.1 (35.7 to 80.9)
		Haemophilus influenzae	253 (216 to 294)	418 (356 to 487)	65.3 (51.6 to 78.9)	0.76 (0.71 to 0.82)	0.51 (0.46 to 0.57)	-32.5 (-40.0 to -24.6)
		Influenza	350 (298 to 408)	53.4 (27.7 to 89.2)	-84.7 (-92.2 to -73.9)	2.06 (1.93 to 2.2)	0.19 (0.09 to 0.32)	-91.0 (-95.4 to -84.7)
		Klebsiella pneumoniae	130 (111 to 150)	190 (163 to 220)	46.4 (35.6 to 58.4)	1.02 (0.95 to 1.1)	0.91 (0.83 to 1)	-10.8 (-20.0 to -0.4)
		Legionella spp.	145 (122 to 171)	351 (302 to 405)	141.8 (118.0 to 168.7)	0.79 (0.71 to 0.87)	1.16 (1.05 to 1.28)	47.2 (28.6 to 69.3)
		Mycoplasma spp.	547 (465 to 632)	942 (805 to 1100)	72.4 (58.6 to 87.5)	1.14 (1.06 to 1.23)	1.2 (1.08 to 1.32)	5.0 (-7.0 to 18.0)
		Polymicrobial	6.54 (3.92 to 10.5)	13.1 (7.44 to 21.6)	99.6 (74.6 to 126.5)	0.05 (0.03 to 0.08)	0.06 (0.04 to 0.11)	21.6 (2.7 to 41.1)
		Pseudomonas aeruginosa	154 (131 to 179)	447 (384 to 517)	190.8 (166.3 to 216.4)	0.84 (0.78 to 0.91)	1.48 (1.36 to 1.63)	77.1 (57.3 to 100.1)
		Respiratory syncytial virus	116 (98 to 136)	16.4 (8.44 to 27.7)	-85.8 (-92.7 to -75.7)	0.31 (0.28 to 0.34)	0.03 (0.01 to 0.04)	-91.6 (-95.7 to -85.6)
		Staphylococcus aureus	218 (185 to 252)	703 (605 to 815)	223.6 (202.5 to 245.5)	2.43 (2.28 to 2.58)	4.78 (4.4 to 5.22)	97.1 (77.1 to 117.8)
		Streptococcus pneumoniae	1920 (1640 to 2230)	1400 (1200 to 1620)	-27.2 (-31.4 to -22.2)	8.94 (8.43 to 9.47)	3.96 (3.64 to 4.32)	-55.7 (-59.8 to -51.1)
		Other viruses	1090 (926 to 1250)	1750 (1510 to 2020)	61.7 (52.0 to 73.3)	1.98 (1.87 to 2.1)	1.95 (1.79 to 2.13)	-1.5 (-10.8 to 9.7)
		Other bacterial pathogens	188 (155 to 224)	274 (226 to 328)	45.8 (32.2 to 60.9)	0.84 (0.74 to 0.94)	0.74 (0.64 to 0.86)	-11.1 (-21.8 to 1.3)
	50-69 years	*Acinetobacter baumannii*	94.6 (78.9 to 110)	79.3 (67.9 to 92.2)	-15.9 (-25.9 to -4.6)	1.32 (1.13 to 1.54)	0.98 (0.84 to 1.14)	-25.9 (-37.3 to -13.6)
		Chlamydia spp.	316 (276 to 358)	336 (295 to 380)	6.4 (-5.1 to 20.1)	1.1 (0.97 to 1.24)	1.03 (0.9 to 1.18)	-6.2 (-19.5 to 11.1)
		Enterobacter spp.	62.1 (53.1 to 72)	176 (153 to 200)	183.7 (149.3 to 222.6)	0.82 (0.71 to 0.95)	2.05 (1.79 to 2.35)	150.0 (112.9 to 191.1)

		Escherichia coli	158 (138 to 179)	372 (333 to 416)	135.9 (109.0 to 164.7)	2.17 (1.94 to 2.43)	4.5 (4.02 to 5.04)	107.9 (79.9 to 142.1)
		Fungus	151 (129 to 173)	307 (269 to 350)	103.6 (77.5 to 131.5)	2.05 (1.79 to 2.34)	3.66 (3.16 to 4.23)	79.4 (51.3 to 107.9)
		Group B Streptococcus	135 (117 to 155)	311 (273 to 352)	132.1 (102.4 to 163.7)	1.31 (1.15 to 1.49)	2.67 (2.33 to 3.04)	104.5 (73.0 to 143.0)
		Haemophilus influenzae	493 (437 to 547)	837 (751 to 938)	70.0 (53.5 to 88.9)	2.58 (2.34 to 2.82)	2.59 (2.3 to 2.89)	0.5 (-12.4 to 15.3)
		Influenza	587 (519 to 654)	89.8 (47.4 to 152)	-84.7 (-92.1 to -74.1)	6.02 (5.48 to 6.56)	0.78 (0.4 to 1.33)	-87.0 (-93.3 to -77.8)
		Klebsiella pneumoniae	324 (287 to 360)	530 (473 to 589)	63.6 (48.2 to 81.1)	4.43 (4.03 to 4.83)	6.37 (5.68 to 7.12)	44.1 (25.9 to 65.4)
		Legionella spp.	399 (346 to 451)	890 (797 to 995)	123.8 (98.7 to 153.0)	3.75 (3.33 to 4.17)	7.37 (6.53 to 8.26)	97.2 (66.8 to 128.8)
		Mycoplasma spp.	489 (430 to 545)	655 (585 to 732)	34.3 (19.8 to 50.4)	1.77 (1.58 to 1.97)	2.09 (1.86 to 2.36)	18.4 (3.2 to 37.1)
		Polymicrobial	18.1 (11.2 to 28.8)	34.5 (20.1 to 57.4)	90.3 (63.8 to 122.3)	0.25 (0.15 to 0.41)	0.42 (0.24 to 0.69)	67.8 (40.2 to 97.9)
		Pseudomonas aeruginosa	425 (373 to 477)	1060 (956 to 1190)	150.7 (126.7 to 179.3)	4.03 (3.63 to 4.45)	8.88 (7.91 to 9.92)	121.0 (91.2 to 154.4)
		Respiratory syncytial virus	144 (125 to 162)	17.9 (9.26 to 30.5)	-87.5 (-93.5 to -78.6)	0.67 (0.59 to 0.75)	0.07 (0.04 to 0.12)	-89.4 (-94.6 to -81.6)
		Staphylococcus aureus	529 (468 to 586)	1630 (1450 to 1800)	207.8 (180.5 to 239.2)	10.3 (9.38 to 11.2)	27.8 (24.7 to 31)	171.3 (138.4 to 210.9)
		Streptococcus pneumoniae	2310 (2050 to 2560)	1830 (1630 to 2030)	-20.9 (-27.8 to -12.8)	18.7 (17.1 to 20.3)	13 (11.6 to 14.4)	-30.3 (-38.8 to -20.5)
		Other viruses	1140 (1010 to 1260)	2060 (1840 to 2290)	80.7 (64.7 to 98.7)	3.62 (3.31 to 3.95)	5.76 (5.12 to 6.46)	59.3 (39.8 to 82.6)
		Other bacterial pathogens	555 (476 to 640)	743 (633 to 861)	34.1 (19.9 to 49.8)	4.28 (3.72 to 4.87)	5.05 (4.29 to 5.87)	18.3 (1.8 to 37.2)
	70+ years	*Acinetobacter baumannii*	149 (127 to 176)	166 (143 to 192)	11.2 (-1.0 to 25.5)	9.57 (8.07 to 11.1)	11.5 (8.84 to 13.4)	19.7 (2.1 to 36.0)
		Chlamydia spp.	596 (520 to 681)	837 (745 to 953)	40.8 (24.8 to 57.9)	9.47 (8.25 to 10.5)	14.3 (11.5 to 16.4)	51.5 (30.6 to 72.8)
		Enterobacter spp.	95.6 (81.8 to 113)	358 (312 to 406)	275.5 (231.1 to 323.5)	5.81 (4.9 to 6.7)	23.4 (18.3 to 26.8)	304.0 (245.3 to 358.8)
		Escherichia coli	307 (270 to 353)	956 (860 to 1080)	212.2 (178.8 to 248.4)	19.3 (16.9 to 21.5)	64.8 (51.4 to 73)	235.9 (189.1 to 278.5)
		Fungus	133 (115 to 155)	358 (316 to 411)	169.4 (136.7 to 203.5)	8.25 (7.14 to 9.4)	23.9 (18.6 to 27.7)	189.8 (149.1 to 230.0)
		Group B Streptococcus	195 (170 to 225)	597 (532 to 677)	207.1 (170.5 to 247.9)	8.7 (7.45 to 9.79)	28.7 (23.1 to 32.8)	230.4 (182.9 to 277.9)
		Haemophilus influenzae	702 (633 to 784)	1580 (1420 to 1770)	124.9 (104.7 to 146.2)	16.8 (14.8 to 18.3)	27.3 (22 to 30.8)	62.4 (42.7 to 80.0)
		Influenza	1410 (1270 to 1570)	285 (149 to 487)	-79.8 (-89.4 to -65.9)	66.1 (58.7 to 71.3)	13.9 (7.06 to 23.2)	-79.0 (-89.3 to -65.2)
		Klebsiella pneumoniae	565 (509 to 632)	1220 (1100 to 1380)	116.4 (97.7 to 136.6)	35.3 (31.2 to 38.2)	82.2 (65.8 to 92.1)	132.9 (104.0 to 156.0)

		Legionella spp.	453 (398 to 516)	1340 (1210 to 1500)	196.1 (165.4 to 231.7)	19.5 (17 to 21.8)	62 (49.8 to 69.7)	218.6 (174.6 to 262.4)
		Mycoplasma spp.	653 (582 to 740)	1160 (1050 to 1310)	77.7 (60.8 to 96.3)	10.8 (9.53 to 12)	20.7 (16.7 to 23.4)	91.2 (67.2 to 112.5)
		Polymicrobial	32.7 (19.9 to 53.3)	82.8 (46.5 to 141)	151.9 (117.9 to 191.8)	2.08 (1.28 to 3.4)	5.68 (3.18 to 9.89)	171.0 (128.0 to 211.1)
		Pseudomonas aeruginosa	699 (626 to 793)	2320 (2100 to 2590)	231.8 (200.9 to 267.1)	30.3 (26.8 to 33.2)	108 (86.3 to 122)	257.0 (207.6 to 300.7)
		Respiratory syncytial virus	213 (188 to 244)	35.1 (18.4 to 61.7)	-83.5 (-91.4 to -71.8)	4.55 (3.98 to 5.1)	0.78 (0.39 to 1.32)	-82.8 (-91.3 to -71.0)
		Staphylococcus aureus	1080 (977 to 1200)	4400 (3970 to 4930)	307.4 (273.5 to 344.5)	96 (84.6 to 103)	421 (337 to 472)	338.3 (286.1 to 382.8)
		Streptococcus pneumoniae	3760 (3400 to 4190)	3930 (3560 to 4400)	4.7 (-3.8 to 14.2)	139 (123 to 149)	156 (125 to 176)	12.7 (-0.7 to 23.6)
		Other viruses	1830 (1660 to 2050)	4380 (3960 to 4910)	139.1 (120.4 to 160.7)	26.6 (23.7 to 28.6)	68.6 (55.2 to 76.7)	157.3 (126.2 to 183.3)
		Other bacterial pathogens	1170 (1020 to 1350)	2080 (1790 to 2420)	77.4 (58.2 to 97.1)	41.8 (35.5 to 47.2)	80 (61.3 to 95)	91.2 (65.4 to 115.5)
Italy	All Ages	*Acinetobacter baumannii*	3000 (2580 to 3460)	2130 (1850 to 2430)	-29.1 (-35.4 to -21.7)	92.7 (77.8 to 107)	79.5 (61.4 to 93.5)	-14.3 (-25.9 to -4.0)
		Chlamydia spp.	14300 (13000 to 15700)	12200 (11200 to 13500)	-14.5 (-22.3 to -5.8)	108 (93.1 to 119)	113 (90.5 to 128)	4.4 (-7.7 to 16.5)
		Enterobacter spp.	2860 (2510 to 3230)	6390 (5730 to 7080)	123.7 (101.9 to 146.4)	81.6 (69 to 93.6)	223 (174 to 256)	172.7 (137.1 to 206.4)
		Escherichia coli	7540 (6840 to 8400)	14900 (13800 to 16300)	98.5 (80.7 to 114.8)	240 (209 to 264)	566 (450 to 636)	135.8 (108.9 to 160.6)
		Fungus	4910 (4340 to 5510)	7270 (6540 to 8070)	48.5 (34.0 to 63.9)	117 (101 to 132)	221 (177 to 253)	89.3 (64.8 to 111.8)
		Group B Streptococcus	6070 (5480 to 6710)	10800 (9820 to 11800)	77.8 (59.2 to 96.9)	120 (103 to 133)	264 (212 to 301)	120.0 (92.4 to 148.3)
		Haemophilus influenzae	25100 (23400 to 27000)	30100 (27900 to 32500)	20.0 (13.1 to 27.1)	250 (222 to 267)	255 (208 to 286)	2.0 (-8.2 to 11.0)
		Influenza	37800 (35100 to 40600)	4690 (2430 to 8150)	-87.6 (-93.5 to -78.9)	879 (769 to 942)	128 (65.3 to 218)	-85.4 (-92.5 to -75.2)
		Klebsiella pneumoniae	15400 (14400 to 16600)	19500 (18100 to 21100)	26.3 (19.6 to 33.3)	450 (397 to 483)	709 (571 to 791)	57.1 (41.5 to 70.0)
		Legionella spp.	17900 (16200 to 19600)	28700 (26700 to 31100)	60.7 (46.4 to 75.4)	325 (283 to 358)	653 (530 to 733)	101.2 (77.9 to 124.3)
		Mycoplasma spp.	27400 (25300 to 29700)	25600 (23800 to 27500)	-6.7 (-12.6 to 0.3)	166 (146 to 179)	196 (160 to 220)	18.3 (5.2 to 30.0)
		Polymicrobial	790 (481 to 1290)	1230 (690 to 2120)	54.4 (34.5 to 74.8)	24.9 (15.1 to 40.2)	46 (24.8 to 79.4)	83.3 (53.8 to 111.6)
		Pseudomonas aeruginosa	22700 (20900 to 24900)	43800 (40800 to 47400)	93.2 (80.7 to 106.7)	464 (409 to 504)	1090 (868 to 1210)	134.0 (109.0 to 155.4)
		Respiratory syncytial virus	15000 (13800 to 16300)	1090 (571 to 1860)	-92.7 (-96.2 to -87.5)	101 (91.6 to 109)	8.57 (4.33 to 14.5)	-91.5 (-95.7 to -85.7)
		Staphylococcus aureus	29800 (27800 to 32000)	74600 (69300 to 80900)	150.6 (139.1 to 162.1)	1320 (1150 to 1410)	3990 (3190 to 4440)	202.0 (173.1 to 224.5)

		Streptococcus pneumoniae	132000 (124000 to 141000)	69300 (64800 to 74400)	-47.5 (-49.9 to -45.0)	1970 (1750 to 2100)	1370 (1110 to 1520)	-30.5 (-37.4 to -25.4)
		Other viruses	84600 (79300 to 90100)	94500 (88800 to 101000)	11.7 (6.4 to 17.4)	464 (415 to 495)	704 (571 to 781)	51.3 (35.7 to 62.8)
		Other bacterial pathogens	27100 (23900 to 30900)	30400 (26300 to 35100)	12.3 (4.2 to 21.9)	502 (424 to 576)	669 (516 to 795)	33.2 (17.7 to 46.5)
	Under 5	*Acinetobacter baumannii*	41.4 (26.8 to 63.2)	13.3 (8.39 to 21.2)	-68.1 (-71.7 to -64.0)	1.08 (0.62 to 1.78)	0.18 (0.09 to 0.32)	-83.8 (-88.0 to -79.6)
		Chlamydia spp.	548 (460 to 643)	217 (179 to 260)	-60.4 (-65.7 to -54.5)	1.91 (1.73 to 2.11)	0.22 (0.17 to 0.28)	-88.3 (-91.1 to -85.7)
		Enterobacter spp.	93.2 (70 to 122)	86.8 (65 to 114)	-6.5 (-20.5 to 10.1)	1.33 (0.98 to 1.78)	0.3 (0.2 to 0.42)	-77.5 (-83.1 to -72.2)
		Escherichia coli	256 (213 to 308)	249 (203 to 297)	-2.4 (-16.5 to 11.9)	3.54 (3.18 to 3.98)	0.77 (0.59 to 0.94)	-78.3 (-83.5 to -73.4)
		Fungus	46.8 (32.8 to 65.7)	34.9 (24.6 to 48.2)	-25.1 (-38.2 to -11.1)	0.46 (0.33 to 0.6)	0.07 (0.04 to 0.1)	-85.0 (-89.0 to -80.7)
		Group B Streptococcus	248 (207 to 297)	197 (161 to 240)	-20.5 (-31.7 to -8.7)	2.81 (2.53 to 3.15)	0.56 (0.43 to 0.69)	-80.0 (-84.7 to -75.6)
		Haemophilus influenzae	2310 (1940 to 2730)	1170 (980 to 1410)	-49.3 (-55.8 to -41.8)	9.82 (9 to 10.7)	0.61 (0.46 to 0.75)	-93.8 (-95.3 to -92.3)
		Influenza	1830 (1550 to 2140)	76.4 (39.9 to 132)	-95.8 (-97.8 to -92.9)	14.7 (13.7 to 15.8)	0.12 (0.06 to 0.22)	-99.2 (-99.6 to -98.5)
		Klebsiella pneumoniae	903 (767 to 1060)	364 (305 to 436)	-59.6 (-64.3 to -54.6)	10.1 (9.36 to 10.9)	1.02 (0.79 to 1.24)	-89.9 (-92.2 to -87.7)
		Legionella spp.	145 (104 to 194)	105 (72.7 to 146)	-27.2 (-37.1 to -15.9)	1.66 (1.31 to 2.08)	0.36 (0.26 to 0.46)	-78.5 (-83.6 to -73.2)
		Mycoplasma spp.	1250 (1040 to 1490)	1060 (893 to 1280)	-14.6 (-25.3 to -3.4)	3.57 (3.26 to 3.93)	0.62 (0.47 to 0.77)	-82.5 (-86.8 to -78.2)
		Polymicrobial	56.1 (30.6 to 92.4)	28 (15.3 to 47.1)	-50.0 (-55.7 to -43.6)	1.75 (0.91 to 3)	0.43 (0.19 to 0.76)	-75.8 (-81.8 to -69.8)
		Pseudomonas aeruginosa	793 (669 to 949)	911 (759 to 1080)	15.1 (0.3 to 31.9)	6.8 (6.18 to 7.42)	1.66 (1.26 to 2.02)	-75.5 (-81.2 to -70.0)
		Respiratory syncytial virus	6390 (5490 to 7390)	331 (170 to 566)	-94.8 (-97.2 to -91.2)	25.1 (23.6 to 26.5)	0.27 (0.13 to 0.48)	-98.9 (-99.5 to -98.1)
		Staphylococcus aureus	825 (700 to 957)	861 (722 to 1030)	4.5 (-5.5 to 15.2)	12.9 (12.1 to 13.7)	2.84 (2.17 to 3.47)	-78.0 (-83.0 to -73.5)
		Streptococcus pneumoniae	11400 (9610 to 13300)	1750 (1470 to 2090)	-84.6 (-86.1 to -83.2)	68.3 (64.7 to 72.1)	2.09 (1.56 to 2.57)	-96.9 (-97.7 to -96.3)
		Other viruses	12500 (10600 to 14600)	5070 (4280 to 6030)	-59.4 (-63.2 to -55.7)	29.9 (28.2 to 31.6)	2.73 (2.07 to 3.32)	-90.9 (-93.0 to -88.9)
		Other bacterial pathogens	265 (207 to 335)	139 (105 to 178)	-47.6 (-55.3 to -38.8)	1.92 (1.5 to 2.41)	0.25 (0.17 to 0.35)	-87.0 (-90.4 to -83.4)
	5-14 years	*Acinetobacter baumannii*	66.6 (47.9 to 91.6)	25.6 (19.4 to 32.9)	-61.4 (-66.4 to -55.9)	0.2 (0.17 to 0.24)	0.03 (0.03 to 0.04)	-84.4 (-86.4 to -82.5)
		Chlamydia spp.	444 (325 to 596)	217 (167 to 279)	-50.8 (-56.9 to -44.2)	0.34 (0.31 to 0.37)	0.07 (0.06 to 0.07)	-80.1 (-82.6 to -77.7)
		Enterobacter spp.	64.9 (46.8 to 89)	81.7 (62 to 107)	26.8 (10.2 to 46.0)	0.19 (0.16 to 0.22)	0.1 (0.08 to 0.11)	-48.9 (-55.0 to -42.1)

		Escherichia coli	137 (100 to 182)	152 (117 to 194)	11.5 (-2.1 to 25.1)	0.41 (0.37 to 0.45)	0.18 (0.17 to 0.2)	-55.0 (-59.9 to -50.2)
		Fungus	164 (121 to 220)	149 (116 to 192)	-8.4 (-20.6 to 4.9)	0.49 (0.44 to 0.54)	0.18 (0.16 to 0.2)	-63.1 (-67.6 to -58.4)
		Group B Streptococcus	190 (138 to 253)	200 (152 to 255)	6.0 (-8.5 to 21.1)	0.4 (0.36 to 0.46)	0.17 (0.15 to 0.19)	-57.2 (-62.6 to -51.3)
		Haemophilus influenzae	854 (644 to 1130)	598 (464 to 764)	-29.6 (-37.3 to -21.3)	0.98 (0.92 to 1.04)	0.18 (0.17 to 0.2)	-81.1 (-82.8 to -79.3)
		Influenza	1160 (867 to 1520)	76 (37.8 to 136)	-93.4 (-96.6 to -88.8)	2.59 (2.45 to 2.72)	0.07 (0.03 to 0.12)	-97.4 (-98.7 to -95.5)
		Klebsiella pneumoniae	399 (297 to 522)	261 (205 to 330)	-34.2 (-41.0 to -26.7)	1.19 (1.12 to 1.27)	0.32 (0.29 to 0.34)	-73.4 (-75.9 to -71.0)
		Legionella spp.	555 (414 to 741)	529 (416 to 671)	-4.1 (-15.8 to 8.7)	1.14 (1.05 to 1.25)	0.44 (0.4 to 0.48)	-61.3 (-65.9 to -56.5)
		Mycoplasma spp.	1810 (1330 to 2390)	1180 (925 to 1520)	-34.5 (-41.8 to -26.8)	1.44 (1.34 to 1.54)	0.38 (0.35 to 0.42)	-73.6 (-76.2 to -70.8)
		Polymicrobial	18 (10.2 to 29.7)	15.8 (8.64 to 26.2)	-12.4 (-25.2 to 1.0)	0.05 (0.03 to 0.09)	0.02 (0.01 to 0.03)	-64.6 (-69.3 to -59.9)
		Pseudomonas aeruginosa	568 (421 to 751)	645 (507 to 814)	14.1 (1.5 to 27.7)	1.18 (1.1 to 1.26)	0.54 (0.5 to 0.59)	-53.9 (-58.4 to -49.3)
		Respiratory syncytial virus	451 (338 to 597)	24.8 (12.3 to 44)	-94.5 (-97.2 to -90.6)	0.46 (0.43 to 0.5)	0.01 (0.01 to 0.02)	-97.8 (-98.9 to -96.3)
		Staphylococcus aureus	744 (555 to 979)	1000 (788 to 1260)	35.8 (22.0 to 50.9)	3.15 (2.99 to 3.31)	1.73 (1.58 to 1.87)	-45.2 (-49.9 to -40.5)
		Streptococcus pneumoniae	6230 (4690 to 8230)	1890 (1480 to 2380)	-69.5 (-72.6 to -66.3)	11 (10.5 to 11.5)	1.35 (1.25 to 1.46)	-87.7 (-88.8 to -86.6)
		Other viruses	4130 (3120 to 5430)	2810 (2210 to 3550)	-31.6 (-38.5 to -24.2)	2.87 (2.73 to 3.01)	0.79 (0.73 to 0.86)	-72.4 (-74.8 to -69.9)
		Other bacterial pathogens	570 (418 to 767)	343 (260 to 441)	-39.5 (-46.3 to -32.4)	0.96 (0.85 to 1.08)	0.23 (0.2 to 0.27)	-75.6 (-78.1 to -72.8)
	15-49 years	*Acinetobacter baumannii*	252 (200 to 307)	92 (75.9 to 110)	-63.4 (-67.2 to -59.1)	2.05 (1.74 to 2.39)	0.58 (0.5 to 0.67)	-71.6 (-74.9 to -68.1)
		Chlamydia spp.	1680 (1410 to 1980)	781 (678 to 897)	-53.4 (-57.7 to -48.5)	3.39 (3.09 to 3.71)	1.23 (1.11 to 1.35)	-63.8 (-67.7 to -59.6)
		Enterobacter spp.	246 (200 to 296)	294 (246 to 346)	20.1 (6.8 to 33.7)	1.89 (1.65 to 2.16)	1.76 (1.56 to 1.99)	-6.7 (-16.9 to 4.2)
		Escherichia coli	518 (434 to 609)	546 (476 to 622)	5.6 (-4.6 to 16.1)	4.13 (3.77 to 4.5)	3.38 (3.12 to 3.64)	-18.0 (-25.3 to -9.9)
		Fungus	621 (517 to 729)	538 (463 to 618)	-13.2 (-22.4 to -3.0)	4.88 (4.39 to 5.41)	3.29 (2.95 to 3.66)	-32.6 (-40.1 to -25.2)
		Group B Streptococcus	718 (599 to 859)	718 (620 to 828)	0.5 (-10.6 to 12.5)	4.06 (3.66 to 4.54)	3.16 (2.86 to 3.5)	-22.0 (-30.5 to -12.5)
		Haemophilus influenzae	3230 (2750 to 3750)	2150 (1880 to 2450)	-33.3 (-38.3 to -28.6)	9.83 (9.28 to 10.4)	3.38 (3.17 to 3.62)	-65.6 (-68.2 to -62.8)
		Influenza	4370 (3750 to 5040)	273 (142 to 462)	-93.7 (-96.8 to -89.2)	26 (24.7 to 27.4)	1.22 (0.62 to 2.09)	-95.3 (-97.6 to -91.9)
		Klebsiella pneumoniae	1510 (1290 to 1740)	941 (827 to 1070)	-37.6 (-41.7 to -33.0)	12 (11.4 to 12.6)	5.8 (5.44 to 6.16)	-51.6 (-55.2 to -48.0)

		Legionella spp.	2100 (1780 to 2450)	1910 (1680 to 2160)	-9.1 (-17.9 to 0.7)	11.5 (10.5 to 12.5)	8.08 (7.56 to 8.63)	-29.4 (-36.4 to -22.1)
		Mycoplasma spp.	6850 (5840 to 7930)	4250 (3750 to 4860)	-37.9 (-42.9 to -32.8)	14.4 (13.5 to 15.4)	6.95 (6.52 to 7.39)	-51.8 (-55.7 to -47.7)
		Polymicrobial	68.2 (39.1 to 112)	56.8 (31 to 95.6)	-16.9 (-26.8 to -5.5)	0.55 (0.32 to 0.9)	0.36 (0.2 to 0.61)	-35.5 (-43.7 to -26.8)
		Pseudomonas aeruginosa	2150 (1830 to 2500)	2320 (2050 to 2630)	8.2 (0.0 to 17.4)	11.8 (11.1 to 12.7)	9.93 (9.36 to 10.6)	-16.0 (-22.9 to -8.9)
		Respiratory syncytial virus	1710 (1450 to 1990)	89.1 (46.1 to 150)	-94.8 (-97.3 to -90.9)	4.63 (4.29 to 5)	0.18 (0.09 to 0.32)	-96.1 (-98.0 to -93.2)
		Staphylococcus aureus	2810 (2420 to 3250)	3620 (3210 to 4070)	28.7 (20.9 to 37.1)	31.7 (30.4 to 33.1)	31.7 (30 to 33.3)	-0.1 (-6.7 to 6.7)
		Streptococcus pneumoniae	23600 (20400 to 27000)	6790 (6010 to 7660)	-71.1 (-72.8 to -69.3)	110 (106 to 115)	24.8 (23.5 to 26)	-77.6 (-79.0 to -76.1)
		Other viruses	15600 (13500 to 17900)	10100 (8950 to 11400)	-35.2 (-39.0 to -31.0)	28.8 (27.6 to 30.1)	14.5 (13.7 to 15.2)	-49.7 (-52.8 to -46.6)
		Other bacterial pathogens	2160 (1760 to 2570)	1240 (1030 to 1460)	-42.7 (-47.6 to -37.4)	9.67 (8.49 to 10.9)	4.31 (3.73 to 4.94)	-55.4 (-59.3 to -51.2)
	50-69 years	*Acinetobacter baumannii*	1050 (883 to 1220)	510 (435 to 595)	-51.3 (-56.0 to -46.5)	10.8 (9.33 to 12.5)	4.74 (4.1 to 5.39)	-56.0 (-61.1 to -50.6)
		Chlamydia spp.	4140 (3630 to 4710)	2460 (2170 to 2750)	-40.5 (-45.8 to -34.0)	10.6 (9.57 to 11.6)	5.68 (5.12 to 6.24)	-46.2 (-51.9 to -40.0)
		Enterobacter spp.	992 (854 to 1140)	1540 (1350 to 1750)	56.1 (41.1 to 71.9)	9.67 (8.55 to 10.9)	13.6 (12.2 to 15.1)	41.1 (25.4 to 57.6)
		Escherichia coli	2310 (2030 to 2600)	3050 (2750 to 3350)	32.3 (20.9 to 44.2)	23.3 (21.3 to 25.3)	27.9 (25.8 to 30.1)	19.6 (8.2 to 31.4)
		Fungus	2200 (1900 to 2510)	2500 (2190 to 2830)	13.4 (2.7 to 24.6)	21.9 (19.6 to 24.5)	22.4 (20.1 to 24.9)	2.5 (-8.4 to 15.0)
		Group B Streptococcus	2050 (1790 to 2330)	2620 (2340 to 2940)	28.2 (15.1 to 42.2)	14.7 (13.3 to 16.3)	17 (15.5 to 18.7)	16.0 (2.7 to 30.5)
		Haemophilus influenzae	7880 (7060 to 8670)	7210 (6500 to 7890)	-8.5 (-14.0 to -3.2)	30.3 (28.4 to 32.3)	16.7 (15.5 to 18)	-45.0 (-49.5 to -40.4)
		Influenza	9190 (8220 to 10100)	784 (411 to 1370)	-91.5 (-95.5 to -85.3)	69.2 (65.2 to 73.1)	5.16 (2.6 to 8.82)	-92.5 (-96.2 to -87.3)
		Klebsiella pneumoniae	4720 (4250 to 5160)	4250 (3850 to 4660)	-9.9 (-14.8 to -4.7)	47.3 (44.6 to 50.2)	38.6 (36 to 41.3)	-18.5 (-24.7 to -11.9)
		Legionella spp.	7210 (6390 to 8120)	8440 (7620 to 9300)	17.4 (7.3 to 28.2)	49.8 (45.7 to 54.4)	52.7 (49.1 to 56.6)	6.1 (-4.9 to 17.7)
		Mycoplasma spp.	7660 (6800 to 8500)	5500 (4960 to 6070)	-28.1 (-33.4 to -22.6)	20.4 (18.9 to 22.1)	13.3 (12.2 to 14.2)	-35.0 (-41.3 to -29.3)
		Polymicrobial	236 (140 to 385)	259 (145 to 443)	9.4 (-3.2 to 22.7)	2.41 (1.44 to 3.98)	2.4 (1.34 to 4.11)	-1.1 (-14.2 to 11.4)
		Pseudomonas aeruginosa	7440 (6630 to 8260)	9900 (8970 to 10800)	33.3 (24.4 to 43.0)	51.8 (48.2 to 55.5)	62.3 (58.1 to 66.4)	20.5 (10.4 to 31.4)
		Respiratory syncytial virus	2650 (2330 to 2970)	173 (90.3 to 299)	-93.5 (-96.6 to -88.9)	9.11 (8.22 to 10)	0.52 (0.26 to 0.88)	-94.3 (-97.1 to -90.3)
		Staphylococcus aureus	8560 (7740 to 9400)	14500 (13100 to 15800)	69.2 (61.7 to 77.3)	122 (116 to 129)	187 (175 to 198)	53.0 (41.9 to 64.5)
		Streptococcus pneumoniae	35400 (31800 to 38800)	14700 (13400 to 16100)	-58.4 (-60.3 to -56.5)	210 (200 to 220)	78.9 (73.8 to 83.8)	-62.4 (-65.2 to -59.6)

		Other viruses	20500 (18500 to 22500)	19300 (17500 to 21000)	-6.1 (-10.8 to -1.5)	47.9 (45.5 to 50.5)	40.7 (38.1 to 43.4)	-15.1 (-21.2 to -8.6)
		Other bacterial pathogens	7950 (6820 to 9170)	5860 (4970 to 6750)	-26.3 (-31.6 to -19.9)	45.1 (39.7 to 50.8)	30.1 (26.2 to 34.3)	-33.3 (-39.7 to -26.9)
	70+ years	*Acinetobacter baumannii*	1590 (1340 to 1880)	1490 (1280 to 1730)	-6.6 (-15.2 to 3.2)	78.5 (64.5 to 91.9)	73.9 (56.4 to 87.4)	-5.9 (-18.2 to 5.3)
		Chlamydia spp.	7490 (6650 to 8510)	8540 (7660 to 9620)	14.2 (3.5 to 25.6)	91.7 (77.7 to 102)	105 (83.6 to 121)	15.1 (2.1 to 28.4)
		Enterobacter spp.	1470 (1260 to 1710)	4390 (3860 to 4930)	199.5 (169.0 to 231.2)	68.6 (56.8 to 79.1)	207 (159 to 240)	201.8 (163.4 to 238.4)
		Escherichia coli	4320 (3820 to 4920)	10900 (9950 to 12100)	153.9 (129.9 to 176.2)	208 (178 to 232)	534 (419 to 602)	155.8 (127.9 to 182.9)
		Fungus	1870 (1640 to 2150)	4060 (3580 to 4600)	117.6 (95.1 to 141.1)	89 (74.3 to 102)	195 (152 to 227)	119.2 (91.6 to 145.1)
		Group B Streptococcus	2860 (2530 to 3230)	7030 (6290 to 7820)	146.1 (120.7 to 172.1)	98.1 (81.7 to 110)	243 (192 to 278)	148.0 (117.3 to 179.8)
		Haemophilus influenzae	10800 (9780 to 12000)	19000 (17200 to 21000)	75.6 (64.7 to 86.9)	199 (172 to 215)	234 (187 to 264)	17.7 (6.1 to 27.6)
		Influenza	21300 (19300 to 23600)	3480 (1780 to 6010)	-83.6 (-91.6 to -72.1)	766 (658 to 828)	122 (62.1 to 208)	-84.1 (-91.9 to -72.9)
		Klebsiella pneumoniae	7910 (7220 to 8770)	13700 (12500 to 15100)	73.0 (63.7 to 83.4)	380 (327 to 411)	663 (527 to 743)	74.3 (57.9 to 88.3)
		Legionella spp.	7880 (7020 to 8910)	17700 (16200 to 19500)	125.3 (104.3 to 147.2)	260 (222 to 290)	591 (469 to 668)	126.9 (101.6 to 153.4)
		Mycoplasma spp.	9850 (8850 to 11000)	13600 (12300 to 15100)	37.9 (27.9 to 48.8)	126 (107 to 138)	175 (139 to 198)	38.9 (24.5 to 52.4)
		Polymicrobial	411 (240 to 695)	868 (474 to 1520)	110.0 (84.4 to 136.4)	20.1 (11.6 to 33.7)	42.8 (23 to 74.4)	111.5 (82.8 to 139.2)
		Pseudomonas aeruginosa	11800 (10600 to 13300)	30100 (27500 to 33100)	155.7 (138.1 to 175.2)	392 (338 to 429)	1010 (796 to 1140)	157.7 (131.0 to 180.7)
		Respiratory syncytial virus	3780 (3360 to 4310)	475 (240 to 827)	-87.4 (-93.6 to -78.6)	62.2 (52.7 to 69.5)	7.58 (3.78 to 12.8)	-87.8 (-93.8 to -79.2)
		Staphylococcus aureus	16800 (15400 to 18700)	54700 (50000 to 60300)	224.7 (208.7 to 240.2)	1150 (981 to 1240)	3770 (2970 to 4210)	227.2 (197.5 to 251.4)
		Streptococcus pneumoniae	55400 (50400 to 61400)	44100 (40300 to 48400)	-20.2 (-24.1 to -16.2)	1570 (1360 to 1690)	1270 (1000 to 1410)	-19.6 (-27.1 to -13.7)
		Other viruses	31800 (29000 to 35200)	57200 (52400 to 62800)	80.1 (71.1 to 89.3)	355 (308 to 383)	645 (513 to 721)	81.5 (64.7 to 94.8)
		Other bacterial pathogens	16200 (14100 to 18800)	22900 (19600 to 26600)	41.4 (31.6 to 53.6)	444 (368 to 513)	635 (483 to 756)	42.7 (26.1 to 56.9)
Luxembourg	All Ages	*Acinetobacter baumannii*	26 (22.1 to 30.2)	15.1 (13.1 to 17.3)	-41.9 (-48.8 to -34.1)	1.17 (1.01 to 1.35)	0.61 (0.5 to 0.72)	-47.5 (-54.9 to -39.2)
		Chlamydia spp.	116 (105 to 129)	97.6 (87.7 to 108)	-16.0 (-26.0 to -5.8)	1.27 (1.14 to 1.39)	0.96 (0.81 to 1.09)	-24.6 (-35.3 to -13.1)
		Enterobacter spp.	21.6 (18.9 to 24.7)	51.7 (46.1 to 57.4)	140.5 (110.4 to 171.4)	0.89 (0.77 to 1.02)	1.94 (1.61 to 2.23)	117.6 (85.7 to 152.5)
		Escherichia coli	59 (52.8 to 65.9)	122 (112 to 133)	108.1 (86.3 to 132.0)	2.71 (2.43 to 2.96)	5.07 (4.34 to 5.72)	87.3 (62.0 to 113.2)
		Fungus	38 (33.7 to 42.8)	61.7 (54.5 to 69.1)	62.8 (42.1 to 84.0)	1.34 (1.19 to 1.49)	1.93 (1.62 to 2.23)	44.7 (24.8 to 67.4)

		Group B Streptococcus	46.3 (41.7 to 52.1)	88.6 (79.8 to 97.6)	91.7 (67.4 to 118.3)	1.33 (1.19 to 1.48)	2.28 (1.95 to 2.59)	71.6 (46.7 to 98.2)
		Haemophilus influenzae	188 (174 to 204)	258 (237 to 279)	37.0 (25.5 to 48.6)	2.69 (2.5 to 2.88)	2.23 (1.91 to 2.51)	-17.0 (-27.8 to -6.8)
		Influenza	288 (266 to 314)	39 (20.4 to 66.2)	-86.4 (-93.0 to -76.9)	9.61 (8.86 to 10.3)	1.15 (0.59 to 1.93)	-88.0 (-93.9 to -79.8)
		Klebsiella pneumoniae	121 (112 to 131)	149 (136 to 161)	23.6 (13.5 to 34.4)	5.07 (4.67 to 5.44)	5.82 (4.98 to 6.53)	14.9 (0.4 to 29.4)
		Legionella spp.	128 (114 to 142)	231 (212 to 251)	81.5 (62.1 to 102.8)	3.4 (3.07 to 3.74)	5.51 (4.73 to 6.23)	62.2 (40.8 to 86.1)
		Mycoplasma spp.	206 (189 to 223)	228 (209 to 247)	10.6 (1.4 to 20.3)	1.81 (1.65 to 1.96)	1.63 (1.4 to 1.83)	-9.8 (-21.6 to 2.5)
		Polymicrobial	6.33 (3.94 to 10.2)	10 (5.66 to 17.1)	56.9 (35.0 to 81.6)	0.29 (0.17 to 0.46)	0.41 (0.23 to 0.71)	41.8 (19.6 to 67.5)
		Pseudomonas aeruginosa	165 (150 to 182)	336 (310 to 363)	103.9 (84.9 to 123.1)	4.89 (4.46 to 5.3)	8.86 (7.6 to 9.93)	81.5 (58.7 to 104.9)
		Respiratory syncytial virus	106 (97.2 to 117)	10.2 (5.27 to 17.1)	-90.4 (-95.1 to -83.7)	0.92 (0.85 to 1)	0.07 (0.04 to 0.12)	-92.4 (-96.2 to -87.0)
		Staphylococcus aureus	224 (206 to 245)	597 (549 to 646)	167.0 (146.2 to 188.2)	14.3 (13.2 to 15.3)	34.6 (29.7 to 38.9)	141.8 (114.8 to 170.3)
		Streptococcus pneumoniae	1010 (937 to 1080)	516 (477 to 555)	-48.7 (-52.1 to -45.4)	21.6 (19.9 to 23)	10.4 (8.95 to 11.7)	-51.8 (-57.4 to -46.2)
		Other viruses	606 (566 to 649)	763 (706 to 819)	26.1 (17.4 to 34.5)	4.69 (4.34 to 5.02)	5.62 (4.9 to 6.3)	19.8 (5.8 to 34.3)
		Other bacterial pathogens	214 (187 to 245)	232 (200 to 267)	8.8 (-2.3 to 20.6)	5.73 (5.01 to 6.54)	5.63 (4.59 to 6.64)	-1.7 (-14.8 to 12.7)
	Under 5	*Acinetobacter baumannii*	0.3 (0.2 to 0.45)	0.15 (0.1 to 0.24)	-50.6 (-58.7 to -41.8)	0.01 (0 to 0.01)	0 (0 to 0.01)	-61.1 (-71.7 to -48.9)
		Chlamydia spp.	4.38 (3.61 to 5.32)	2.84 (2.27 to 3.54)	-34.9 (-45.7 to -23.1)	0.01 (0.01 to 0.01)	0 (0 to 0)	-69.8 (-76.8 to -61.7)
		Enterobacter spp.	0.68 (0.51 to 0.9)	1.17 (0.86 to 1.56)	72.2 (42.3 to 107.4)	0.01 (0.01 to 0.01)	0 (0 to 0.01)	-37.9 (-53.2 to -20.6)
		Escherichia coli	1.95 (1.58 to 2.39)	3.49 (2.75 to 4.34)	79.4 (50.8 to 109.4)	0.02 (0.02 to 0.02)	0.01 (0.01 to 0.02)	-40.1 (-54.7 to -23.9)
		Fungus	0.37 (0.26 to 0.52)	0.48 (0.33 to 0.68)	28.7 (3.5 to 56.0)	0 (0 to 0)	0 (0 to 0)	-56.8 (-68.9 to -41.3)
		Group B Streptococcus	1.84 (1.49 to 2.25)	2.62 (2.05 to 3.28)	42.9 (18.3 to 69.2)	0.02 (0.01 to 0.02)	0.01 (0.01 to 0.01)	-47.1 (-59.7 to -32.3)
		Haemophilus influenzae	17.5 (14.4 to 21.2)	16.2 (13 to 20.2)	-7.3 (-20.6 to 8.1)	0.05 (0.05 to 0.06)	0.01 (0.01 to 0.01)	-81.8 (-86.6 to -76.4)
		Influenza	14 (11.6 to 16.9)	1.09 (0.55 to 1.85)	-92.3 (-96.0 to -86.8)	0.08 (0.07 to 0.09)	0 (0 to 0)	-97.5 (-98.8 to -95.5)
		Klebsiella pneumoniae	7.08 (5.86 to 8.56)	4.69 (3.79 to 5.81)	-33.7 (-42.4 to -24.3)	0.06 (0.05 to 0.07)	0.02 (0.01 to 0.02)	-72.7 (-79.2 to -65.1)
		Legionella spp.	1 (0.7 to 1.37)	1.35 (0.9 to 1.93)	35.3 (15.3 to 59.6)	0.01 (0.01 to 0.01)	0.01 (0 to 0.01)	-41.3 (-55.0 to -25.3)
		Mycoplasma spp.	9.55 (7.8 to 11.7)	13.9 (11.1 to 17.4)	45.9 (24.8 to 68.9)	0.02 (0.02 to 0.02)	0.01 (0.01 to 0.01)	-51.3 (-63.6 to -35.8)

		Polymicrobial	0.35 (0.2 to 0.57)	0.32 (0.18 to 0.53)	-8.9 (-23.8 to 6.7)	0.01 (0.01 to 0.02)	0.01 (0 to 0.01)	-38.7 (-55.2 to -19.0)
		Pseudomonas aeruginosa	5.74 (4.71 to 6.98)	11.7 (9.41 to 14.5)	104.6 (76.3 to 136.8)	0.04 (0.03 to 0.04)	0.03 (0.02 to 0.03)	-31.5 (-47.5 to -12.9)
		Respiratory syncytial virus	45.7 (38.3 to 54.4)	4.15 (2.07 to 7)	-90.9 (-95.3 to -84.9)	0.13 (0.12 to 0.15)	0 (0 to 0.01)	-96.9 (-98.6 to -94.5)
		Staphylococcus aureus	6.21 (5.15 to 7.44)	11.8 (9.48 to 14.6)	90.6 (67.7 to 116.0)	0.07 (0.06 to 0.08)	0.05 (0.04 to 0.06)	-35.3 (-50.5 to -18.4)
		Streptococcus pneumoniae	88.6 (73.4 to 107)	20.9 (16.8 to 25.8)	-76.4 (-79.1 to -73.5)	0.38 (0.32 to 0.43)	0.03 (0.02 to 0.04)	-92.3 (-94.1 to -90.0)
		Other viruses	91 (75.3 to 109)	63.5 (51.3 to 78.2)	-30.2 (-37.8 to -21.4)	0.16 (0.14 to 0.18)	0.04 (0.03 to 0.05)	-73.7 (-79.7 to -66.5)
		Other bacterial pathogens	2.08 (1.57 to 2.69)	1.84 (1.36 to 2.42)	-11.5 (-25.8 to 5.3)	0.01 (0.01 to 0.01)	0 (0 to 0.01)	-64.7 (-73.6 to -53.9)
	5-14 years	*Acinetobacter baumannii*	0.44 (0.32 to 0.6)	0.26 (0.19 to 0.35)	-41.6 (-48.6 to -33.7)	0 (0 to 0)	0 (0 to 0)	-84.6 (-88.1 to -80.4)
		Chlamydia spp.	2.77 (2.05 to 3.64)	2.43 (1.78 to 3.23)	-12.0 (-21.7 to 0.2)	0 (0 to 0)	0 (0 to 0)	-76.8 (-82.1 to -70.3)
		Enterobacter spp.	0.38 (0.27 to 0.5)	0.94 (0.67 to 1.25)	150.3 (120.6 to 185.6)	0 (0 to 0)	0 (0 to 0)	-34.0 (-48.7 to -16.0)
		Escherichia coli	0.82 (0.6 to 1.08)	1.81 (1.35 to 2.37)	120.9 (94.8 to 146.3)	0 (0 to 0)	0 (0 to 0)	-41.8 (-54.8 to -25.7)
		Fungus	0.98 (0.72 to 1.32)	1.69 (1.23 to 2.24)	72.7 (50.2 to 97.0)	0 (0 to 0)	0 (0 to 0)	-54.5 (-64.4 to -41.7)
		Group B Streptococcus	1.12 (0.81 to 1.48)	2.27 (1.69 to 3.04)	103.8 (76.7 to 133.0)	0 (0 to 0)	0 (0 to 0)	-46.3 (-58.6 to -31.4)
		Haemophilus influenzae	4.93 (3.72 to 6.46)	6.87 (5.12 to 8.97)	39.4 (27.3 to 53.3)	0.01 (0.01 to 0.01)	0 (0 to 0)	-75.7 (-80.9 to -69.5)
		Influenza	6.73 (5.05 to 8.67)	0.9 (0.45 to 1.62)	-86.7 (-93.0 to -77.3)	0.02 (0.02 to 0.02)	0 (0 to 0)	-96.6 (-98.3 to -94.0)
		Klebsiella pneumoniae	2.39 (1.81 to 3.09)	2.8 (2.1 to 3.65)	17.2 (7.3 to 29.4)	0.01 (0.01 to 0.01)	0 (0 to 0)	-69.1 (-75.6 to -61.3)
		Legionella spp.	3.06 (2.26 to 4.02)	5.85 (4.36 to 7.66)	91.9 (70.3 to 115.9)	0.01 (0.01 to 0.01)	0 (0 to 0)	-49.4 (-60.5 to -35.4)
		Mycoplasma spp.	10.5 (7.79 to 13.7)	13 (9.68 to 17)	23.5 (11.8 to 35.6)	0.01 (0.01 to 0.01)	0 (0 to 0)	-67.4 (-74.4 to -58.6)
		Polymicrobial	0.11 (0.07 to 0.18)	0.18 (0.1 to 0.31)	62.1 (39.4 to 87.1)	0 (0 to 0)	0 (0 to 0)	-57.2 (-67.5 to -44.6)
		Pseudomonas aeruginosa	3.17 (2.37 to 4.16)	6.98 (5.21 to 9.05)	120.8 (100.4 to 142.6)	0.01 (0.01 to 0.01)	0 (0 to 0.01)	-41.8 (-53.8 to -27.8)
		Respiratory syncytial virus	2.46 (1.87 to 3.22)	0.26 (0.13 to 0.48)	-89.4 (-94.5 to -82.0)	0 (0 to 0)	0 (0 to 0)	-97.3 (-98.7 to -95.2)
		Staphylococcus aureus	4.27 (3.23 to 5.51)	11.5 (8.68 to 14.9)	170.0 (148.5 to 195.6)	0.02 (0.02 to 0.02)	0.02 (0.01 to 0.02)	-28.8 (-43.4 to -11.8)
		Streptococcus pneumoniae	36.5 (27.7 to 47.2)	18.6 (13.9 to 24)	-49.2 (-53.2 to -44.6)	0.08 (0.07 to 0.09)	0.01 (0.01 to 0.01)	-86.6 (-89.3 to -83.2)
		Other viruses	22.8 (17.2 to 29.6)	29.1 (21.9 to 37.6)	27.9 (18.5 to 39.2)	0.02 (0.02 to 0.02)	0.01 (0.01 to 0.01)	-66.3 (-73.1 to -58.1)

		Other bacterial pathogens	3.44 (2.53 to 4.57)	3.83 (2.79 to 5.06)	11.6 (0.1 to 24.5)	0.01 (0.01 to 0.01)	0 (0 to 0)	-70.5 (-76.9 to -63.0)
	15-49 years	*Acinetobacter baumannii*	2.22 (1.78 to 2.7)	1.23 (0.99 to 1.49)	-44.4 (-51.7 to -36.3)	0.02 (0.02 to 0.03)	0 (0 to 0.01)	-79.6 (-82.9 to -75.8)
		Chlamydia spp.	13.8 (11.7 to 16.2)	11.6 (9.58 to 13.7)	-16.2 (-25.8 to -5.8)	0.04 (0.03 to 0.04)	0.01 (0.01 to 0.01)	-69.2 (-74.2 to -63.7)
		Enterobacter spp.	1.87 (1.54 to 2.24)	4.45 (3.59 to 5.4)	138.4 (107.6 to 173.6)	0.02 (0.02 to 0.02)	0.02 (0.01 to 0.02)	-12.4 (-26.3 to 4.2)
		Escherichia coli	4.09 (3.44 to 4.79)	8.59 (7.23 to 10.1)	110.4 (88.2 to 136.7)	0.04 (0.04 to 0.05)	0.03 (0.03 to 0.04)	-22.7 (-34.2 to -9.3)
		Fungus	4.91 (4.13 to 5.77)	8.06 (6.68 to 9.64)	64.5 (41.7 to 88.0)	0.05 (0.05 to 0.06)	0.03 (0.03 to 0.04)	-39.5 (-49.2 to -29.2)
		Group B Streptococcus	5.57 (4.68 to 6.62)	10.8 (9 to 12.9)	94.1 (68.7 to 122.1)	0.04 (0.04 to 0.05)	0.03 (0.03 to 0.04)	-28.7 (-40.3 to -14.8)
		Haemophilus influenzae	24.6 (21.1 to 28.3)	32.7 (27.3 to 38.2)	32.7 (19.4 to 46.9)	0.1 (0.09 to 0.11)	0.03 (0.03 to 0.04)	-67.7 (-72.2 to -62.7)
		Influenza	33.6 (28.9 to 38.5)	4.26 (2.2 to 7.14)	-87.3 (-93.4 to -78.4)	0.27 (0.25 to 0.29)	0.01 (0.01 to 0.02)	-95.5 (-97.7 to -92.2)
		Klebsiella pneumoniae	11.9 (10.3 to 13.7)	13.3 (11.2 to 15.5)	11.6 (1.5 to 22.6)	0.13 (0.12 to 0.14)	0.05 (0.05 to 0.06)	-59.0 (-64.4 to -52.8)
		Legionella spp.	15.3 (13.1 to 17.8)	27.9 (23.5 to 32.5)	82.8 (63.0 to 105.6)	0.11 (0.1 to 0.13)	0.08 (0.07 to 0.09)	-32.8 (-43.0 to -21.0)
		Mycoplasma spp.	52.6 (45.3 to 60.2)	61.8 (52.2 to 72.4)	17.6 (5.9 to 30.0)	0.15 (0.14 to 0.16)	0.06 (0.06 to 0.07)	-56.8 (-63.1 to -50.0)
		Polymicrobial	0.56 (0.33 to 0.91)	0.87 (0.47 to 1.5)	54.4 (33.2 to 79.4)	0.01 (0 to 0.01)	0 (0 to 0.01)	-43.2 (-52.9 to -31.4)
		Pseudomonas aeruginosa	15.8 (13.5 to 18.3)	33.2 (28 to 38.7)	110.3 (88.2 to 133.0)	0.12 (0.11 to 0.13)	0.09 (0.08 to 0.1)	-22.7 (-33.0 to -9.9)
		Respiratory syncytial virus	12.3 (10.5 to 14.2)	1.24 (0.64 to 2.07)	-89.9 (-94.7 to -82.3)	0.05 (0.04 to 0.05)	0 (0 to 0)	-96.4 (-98.2 to -93.7)
		Staphylococcus aureus	21.3 (18.5 to 24.3)	54.8 (46.8 to 63.7)	157.1 (135.2 to 180.6)	0.33 (0.3 to 0.35)	0.31 (0.27 to 0.35)	-5.5 (-18.3 to 8.2)
		Streptococcus pneumoniae	182 (159 to 207)	88.3 (74.9 to 102)	-51.6 (-55.5 to -47.3)	1.16 (1.07 to 1.25)	0.21 (0.18 to 0.23)	-82.2 (-84.5 to -79.6)
		Other viruses	114 (98.6 to 130)	138 (118 to 160)	21.8 (11.8 to 32.7)	0.28 (0.26 to 0.31)	0.13 (0.11 to 0.14)	-55.2 (-61.0 to -48.3)
		Other bacterial pathogens	17.2 (14.2 to 20.3)	18.2 (14.8 to 22.2)	6.3 (-5.6 to 19.3)	0.1 (0.09 to 0.12)	0.04 (0.03 to 0.05)	-60.9 (-66.7 to -54.1)
	50-69 years	*Acinetobacter baumannii*	8.87 (7.38 to 10.4)	4.32 (3.65 to 5.06)	-51.1 (-57.5 to -43.9)	0.15 (0.13 to 0.17)	0.05 (0.04 to 0.06)	-68.0 (-73.8 to -61.7)
		Chlamydia spp.	32.8 (28.5 to 37.2)	23 (20 to 26.1)	-29.8 (-38.4 to -20.0)	0.14 (0.12 to 0.16)	0.06 (0.05 to 0.07)	-54.0 (-62.1 to -44.6)
		Enterobacter spp.	7.28 (6.19 to 8.41)	14.8 (12.8 to 16.8)	103.8 (76.3 to 135.2)	0.12 (0.1 to 0.14)	0.16 (0.13 to 0.18)	33.6 (9.6 to 61.1)
		Escherichia coli	17.6 (15.4 to 19.9)	30.2 (26.8 to 33.6)	72.5 (53.5 to 95.0)	0.29 (0.26 to 0.32)	0.33 (0.28 to 0.37)	13.1 (-6.0 to 33.8)
		Fungus	16.8 (14.4 to 19.3)	23.7 (20.4 to 27.1)	41.5 (21.7 to 63.6)	0.27 (0.24 to 0.31)	0.25 (0.21 to 0.3)	-7.2 (-23.0 to 12.0)

		Group B Streptococcus	15.3 (13.3 to 17.5)	24.9 (21.6 to 28.1)	62.7 (40.6 to 87.0)	0.18 (0.16 to 0.21)	0.19 (0.16 to 0.22)	6.7 (-13.2 to 28.9)
		Haemophilus influenzae	57.8 (50.7 to 64.6)	69.7 (61.8 to 77.2)	20.6 (8.3 to 33.8)	0.36 (0.33 to 0.4)	0.19 (0.16 to 0.22)	-47.7 (-56.0 to -38.2)
		Influenza	68 (60.3 to 75.1)	7.79 (4.04 to 13.3)	-88.5 (-94.1 to -80.4)	0.84 (0.77 to 0.93)	0.06 (0.03 to 0.1)	-92.7 (-96.3 to -87.3)
		Klebsiella pneumoniae	35.9 (31.9 to 39.7)	38.6 (34.3 to 42.7)	7.5 (-2.5 to 18.9)	0.59 (0.54 to 0.65)	0.42 (0.36 to 0.47)	-29.5 (-40.6 to -16.9)
		Legionella spp.	50.4 (43.9 to 56.9)	78 (69 to 87)	55.2 (36.4 to 74.9)	0.57 (0.5 to 0.65)	0.58 (0.5 to 0.66)	1.7 (-15.1 to 21.1)
		Mycoplasma spp.	56.6 (49.4 to 63.2)	49.9 (43.9 to 55.8)	-11.8 (-21.5 to -1.3)	0.25 (0.22 to 0.28)	0.14 (0.12 to 0.16)	-42.1 (-51.7 to -31.1)
		Polymicrobial	1.87 (1.12 to 3.01)	2.52 (1.43 to 4.29)	34.1 (13.8 to 56.5)	0.03 (0.02 to 0.05)	0.03 (0.02 to 0.05)	-12.1 (-29.0 to 7.9)
		Pseudomonas aeruginosa	52.7 (46.3 to 59)	89 (79 to 98.2)	69.2 (50.9 to 88.6)	0.6 (0.54 to 0.67)	0.67 (0.58 to 0.76)	11.0 (-6.5 to 31.9)
		Respiratory syncytial virus	18.4 (16 to 20.7)	1.52 (0.78 to 2.59)	-91.8 (-95.7 to -85.7)	0.1 (0.09 to 0.12)	0.01 (0 to 0.01)	-94.8 (-97.4 to -90.8)
		Staphylococcus aureus	62.5 (55.3 to 69)	139 (123 to 153)	122.9 (102.5 to 146.2)	1.46 (1.33 to 1.61)	2.13 (1.85 to 2.43)	46.1 (23.9 to 72.2)
		Streptococcus pneumoniae	264 (233 to 291)	122 (109 to 135)	-53.5 (-57.9 to -48.6)	2.57 (2.34 to 2.81)	0.78 (0.68 to 0.89)	-69.5 (-74.2 to -64.4)
		Other viruses	144 (127 to 159)	169 (150 to 187)	17.7 (6.9 to 29.4)	0.55 (0.5 to 0.61)	0.42 (0.37 to 0.48)	-22.8 (-34.6 to -9.8)
		Other bacterial pathogens	60.9 (51.4 to 70.2)	54.6 (45.8 to 63.8)	-10.2 (-20.6 to 0.7)	0.57 (0.49 to 0.65)	0.33 (0.28 to 0.4)	-41.0 (-51.3 to -29.6)
	70+ years	*Acinetobacter baumannii*	14.2 (11.8 to 17)	9.11 (7.69 to 10.7)	-35.6 (-44.4 to -25.1)	0.98 (0.84 to 1.15)	0.56 (0.45 to 0.65)	-43.4 (-51.7 to -34.4)
		Chlamydia spp.	62.6 (54.8 to 72.7)	57.7 (49.9 to 66.2)	-7.5 (-20.7 to 6.1)	1.08 (0.96 to 1.19)	0.88 (0.73 to 1.01)	-18.7 (-30.9 to -5.9)
		Enterobacter spp.	11.3 (9.73 to 13.3)	30.4 (25.9 to 34.6)	168.5 (131.5 to 211.7)	0.75 (0.64 to 0.86)	1.76 (1.45 to 2.03)	136.1 (101.5 to 175.0)
		Escherichia coli	34.5 (30.3 to 40)	78.3 (69 to 88)	127.4 (99.7 to 159.1)	2.35 (2.09 to 2.59)	4.69 (3.98 to 5.31)	99.9 (72.4 to 128.1)
		Fungus	15 (12.9 to 17.5)	27.8 (23.8 to 32.2)	86.5 (60.6 to 115.4)	1 (0.88 to 1.15)	1.64 (1.36 to 1.91)	63.9 (40.4 to 91.3)
		Group B Streptococcus	22.5 (19.6 to 25.9)	48.1 (42 to 55.1)	114.4 (82.9 to 146.9)	1.09 (0.96 to 1.22)	2.04 (1.73 to 2.33)	88.5 (60.5 to 118.6)
		Haemophilus influenzae	83.4 (74.4 to 95)	132 (116 to 149)	58.9 (41.3 to 80.7)	2.16 (1.97 to 2.33)	2 (1.69 to 2.26)	-7.6 (-20.2 to 4.5)
		Influenza	165 (148 to 188)	25 (12.7 to 42.9)	-84.9 (-92.3 to -74.1)	8.4 (7.67 to 9.06)	1.08 (0.55 to 1.8)	-87.2 (-93.4 to -78.5)
		Klebsiella pneumoniae	63.4 (56.7 to 71.9)	89.6 (79 to 101)	41.7 (26.2 to 59.3)	4.28 (3.91 to 4.63)	5.34 (4.54 to 6)	24.6 (8.3 to 40.7)
		Legionella spp.	58 (50.4 to 67.1)	118 (105 to 132)	104.5 (77.8 to 134.0)	2.7 (2.4 to 2.99)	4.85 (4.09 to 5.51)	79.8 (55.3 to 107.7)
		Mycoplasma spp.	76.7 (68.3 to 87.9)	88.9 (77.9 to 100)	16.2 (2.9 to 31.5)	1.38 (1.24 to 1.51)	1.41 (1.19 to 1.59)	2.2 (-12.2 to 17.4)
		Polymicrobial	3.44 (2.06 to 5.71)	6.1 (3.38 to 10.6)	76.7 (49.0 to 106.7)	0.24 (0.14 to 0.39)	0.37 (0.2 to 0.65)	55.4 (30.6 to 83.4)

		Pseudomonas aeruginosa	87.8 (78 to 101)	195 (172 to 218)	123.0 (97.5 to 152.1)	4.12 (3.74 to 4.49)	8.08 (6.84 to 9.07)	96.1 (70.6 to 122.2)
		Respiratory syncytial virus	27.6 (24.2 to 31.8)	3 (1.51 to 5.18)	-89.1 (-94.4 to -81.2)	0.64 (0.57 to 0.71)	0.06 (0.03 to 0.1)	-90.8 (-95.5 to -84.3)
		Staphylococcus aureus	129 (116 to 147)	380 (335 to 425)	193.7 (163.0 to 229.3)	12.4 (11.3 to 13.4)	32.1 (27.3 to 36.1)	158.2 (128.0 to 190.5)
		Streptococcus pneumoniae	435 (391 to 495)	266 (235 to 298)	-38.8 (-45.4 to -31.6)	17.4 (15.8 to 18.7)	9.36 (7.95 to 10.5)	-46.2 (-52.5 to -39.5)
		Other viruses	234 (210 to 266)	363 (319 to 408)	55.1 (38.6 to 73.1)	3.68 (3.35 to 3.99)	5.03 (4.31 to 5.64)	36.4 (19.3 to 53.7)
		Other bacterial pathogens	130 (112 to 152)	154 (130 to 181)	18.3 (4.1 to 33.2)	5.04 (4.37 to 5.77)	5.25 (4.25 to 6.22)	4.2 (-9.9 to 18.7)
Malta	All Ages	*Acinetobacter baumannii*	27.3 (23.5 to 31.8)	40.1 (34.4 to 46.8)	47.1 (33.3 to 60.7)	1.26 (1.08 to 1.45)	2.1 (1.66 to 2.5)	66.6 (41.8 to 89.9)
		Chlamydia spp.	121 (110 to 134)	136 (124 to 151)	12.2 (2.8 to 22.0)	1.33 (1.19 to 1.46)	1.76 (1.42 to 2.02)	31.8 (11.5 to 50.7)
		Enterobacter spp.	21.7 (19 to 24.7)	51 (44.9 to 57.4)	135.9 (111.9 to 160.4)	0.91 (0.79 to 1.04)	2.49 (1.99 to 2.96)	172.8 (130.4 to 213.8)
		Escherichia coli	59.4 (53.4 to 66.1)	113 (103 to 124)	89.8 (73.8 to 107.8)	2.79 (2.5 to 3.09)	6 (4.81 to 6.85)	114.8 (82.9 to 145.2)
		Fungus	39.4 (34.9 to 44.5)	67.1 (60.4 to 75.2)	70.6 (54.1 to 87.0)	1.4 (1.23 to 1.57)	2.84 (2.34 to 3.29)	103.3 (72.4 to 131.4)
		Group B Streptococcus	47.6 (42.7 to 53.2)	94.7 (86.7 to 105)	99.2 (80.5 to 120.6)	1.38 (1.22 to 1.54)	3.25 (2.63 to 3.73)	135.6 (100.6 to 172.6)
		Haemophilus influenzae	198 (183 to 215)	243 (225 to 265)	22.9 (14.1 to 32.1)	2.78 (2.54 to 2.99)	2.92 (2.36 to 3.32)	4.9 (-9.9 to 19.1)
		Influenza	292 (270 to 317)	32.3 (16.9 to 55.7)	-88.9 (-94.3 to -81.2)	9.77 (8.87 to 10.5)	1.24 (0.63 to 2.07)	-87.3 (-93.5 to -78.7)
		Klebsiella pneumoniae	125 (115 to 135)	218 (200 to 238)	74.6 (62.2 to 87.3)	5.25 (4.8 to 5.63)	11.1 (8.94 to 12.6)	110.9 (80.8 to 139.1)
		Legionella spp.	127 (113 to 141)	248 (228 to 271)	96.0 (77.4 to 116.0)	3.43 (3.08 to 3.79)	7.88 (6.4 to 8.98)	130.1 (97.0 to 162.6)
		Mycoplasma spp.	219 (201 to 240)	272 (252 to 295)	24.1 (15.0 to 34.1)	1.86 (1.69 to 2.02)	2.96 (2.43 to 3.38)	59.0 (36.0 to 80.4)
		Polymicrobial	6.6 (4.16 to 10.6)	11 (6.42 to 18.4)	65.9 (45.4 to 86.3)	0.32 (0.2 to 0.49)	0.58 (0.33 to 0.98)	83.1 (51.2 to 114.4)
		Pseudomonas aeruginosa	164 (149 to 180)	424 (390 to 463)	158.5 (138.9 to 179.3)	4.92 (4.46 to 5.35)	14.8 (11.9 to 16.9)	200.7 (157.3 to 240.7)
		Respiratory syncytial virus	127 (114 to 141)	11.2 (5.86 to 19.2)	-91.2 (-95.4 to -84.8)	1.03 (0.94 to 1.12)	0.13 (0.06 to 0.21)	-87.6 (-93.7 to -79.0)
		Staphylococcus aureus	223 (205 to 243)	575 (530 to 627)	158.3 (142.1 to 176.9)	14.5 (13.1 to 15.5)	43.1 (34.7 to 49)	197.8 (156.6 to 235.7)
		Streptococcus pneumoniae	1070 (997 to 1160)	1140 (1060 to 1240)	6.5 (0.4 to 13.1)	22.3 (20.3 to 23.8)	31.2 (25.2 to 35.4)	40.1 (20.2 to 57.6)
		Other viruses	650 (604 to 701)	1030 (960 to 1120)	58.8 (48.9 to 68.6)	4.78 (4.37 to 5.1)	10.7 (8.65 to 12.1)	123.3 (92.1 to 150.8)
		Other bacterial pathogens	214 (187 to 243)	313 (271 to 361)	46.4 (34.7 to 59.3)	5.88 (5.07 to 6.69)	9.66 (7.51 to 11.5)	64.2 (39.7 to 87.5)

	Under 5	*Acinetobacter baumannii*	0.46 (0.31 to 0.68)	0.19 (0.12 to 0.28)	-59.1 (-63.9 to -52.9)	0.02 (0.01 to 0.03)	0 (0 to 0.01)	-76.7 (-83.2 to -69.1)
		Chlamydia spp.	6.68 (5.52 to 7.99)	2.56 (2.13 to 3.06)	-61.6 (-66.3 to -56.2)	0.03 (0.02 to 0.03)	0.01 (0 to 0.01)	-77.8 (-83.6 to -71.1)
		Enterobacter spp.	1.01 (0.76 to 1.32)	0.72 (0.54 to 0.95)	-28.0 (-37.8 to -16.7)	0.02 (0.01 to 0.02)	0.01 (0 to 0.01)	-64.2 (-74.0 to -52.2)
		Escherichia coli	2.93 (2.38 to 3.56)	1.83 (1.49 to 2.21)	-37.4 (-46.0 to -27.1)	0.04 (0.04 to 0.05)	0.01 (0.01 to 0.02)	-68.2 (-76.4 to -58.8)
		Fungus	0.56 (0.38 to 0.79)	0.33 (0.23 to 0.46)	-41.2 (-49.9 to -31.1)	0 (0 to 0.01)	0 (0 to 0)	-66.1 (-75.5 to -54.1)
		Group B Streptococcus	2.74 (2.23 to 3.37)	1.8 (1.46 to 2.22)	-34.3 (-42.7 to -24.0)	0.04 (0.03 to 0.04)	0.01 (0.01 to 0.02)	-68.8 (-77.1 to -59.2)
		Haemophilus influenzae	26 (21.1 to 31.2)	9.36 (7.62 to 11.3)	-63.9 (-68.8 to -58.7)	0.1 (0.08 to 0.11)	0.01 (0.01 to 0.01)	-88.8 (-91.8 to -85.1)
		Influenza	20.9 (17.1 to 24.8)	0.63 (0.33 to 1.07)	-97.0 (-98.4 to -95.0)	0.14 (0.12 to 0.16)	0 (0 to 0)	-98.4 (-99.2 to -97.1)
		Klebsiella pneumoniae	10.6 (8.73 to 12.7)	4.36 (3.6 to 5.31)	-59.0 (-63.9 to -53.4)	0.11 (0.09 to 0.12)	0.03 (0.02 to 0.03)	-75.9 (-82.1 to -68.5)
		Legionella spp.	1.45 (1.03 to 2)	0.92 (0.65 to 1.28)	-36.2 (-44.2 to -26.5)	0.02 (0.02 to 0.03)	0.01 (0.01 to 0.01)	-66.2 (-75.3 to -55.5)
		Mycoplasma spp.	14.2 (11.5 to 17.2)	9.36 (7.7 to 11.4)	-34.0 (-42.5 to -24.2)	0.04 (0.03 to 0.04)	0.01 (0.01 to 0.02)	-65.1 (-74.2 to -53.4)
		Polymicrobial	0.52 (0.29 to 0.86)	0.28 (0.15 to 0.46)	-46.7 (-53.2 to -38.4)	0.03 (0.01 to 0.05)	0.01 (0 to 0.02)	-68.3 (-77.7 to -57.5)
		Pseudomonas aeruginosa	8.4 (6.88 to 10.2)	7.83 (6.44 to 9.49)	-6.5 (-18.4 to 7.4)	0.07 (0.06 to 0.08)	0.03 (0.02 to 0.04)	-55.3 (-67.1 to -41.7)
		Respiratory syncytial virus	66.3 (55.2 to 78.3)	3.25 (1.64 to 5.58)	-95.1 (-97.4 to -91.7)	0.24 (0.21 to 0.28)	0.01 (0 to 0.01)	-97.6 (-98.9 to -95.7)
		Staphylococcus aureus	9.19 (7.61 to 10.9)	6.97 (5.77 to 8.41)	-24.1 (-31.3 to -15.6)	0.13 (0.11 to 0.14)	0.05 (0.04 to 0.07)	-59.2 (-69.7 to -47.1)
		Streptococcus pneumoniae	132 (109 to 158)	38.7 (31.8 to 46.6)	-70.7 (-73.6 to -67.4)	0.62 (0.53 to 0.71)	0.11 (0.08 to 0.14)	-82.4 (-86.9 to -76.7)
		Other viruses	133 (110 to 157)	61.2 (50.7 to 74)	-53.8 (-58.5 to -48.8)	0.25 (0.22 to 0.29)	0.07 (0.05 to 0.09)	-72.7 (-79.9 to -64.2)
		Other bacterial pathogens	3.14 (2.4 to 4.04)	1.4 (1.06 to 1.8)	-55.2 (-60.9 to -48.1)	0.02 (0.02 to 0.03)	0.01 (0 to 0.01)	-74.4 (-81.2 to -66.7)
	5-14 years	*Acinetobacter baumannii*	0.7 (0.51 to 0.95)	0.34 (0.25 to 0.46)	-51.0 (-56.3 to -45.5)	0 (0 to 0)	0 (0 to 0)	-41.4 (-53.2 to -25.4)
		Chlamydia spp.	4.28 (3.18 to 5.63)	1.72 (1.25 to 2.3)	-59.7 (-63.6 to -55.4)	0 (0 to 0.01)	0 (0 to 0)	-51.7 (-61.6 to -38.2)
		Enterobacter spp.	0.56 (0.41 to 0.76)	0.46 (0.33 to 0.63)	-17.8 (-26.5 to -9.0)	0 (0 to 0)	0 (0 to 0)	-1.6 (-22.1 to 26.4)
		Escherichia coli	1.24 (0.92 to 1.63)	0.8 (0.58 to 1.06)	-35.9 (-41.9 to -29.2)	0.01 (0 to 0.01)	0 (0 to 0)	-23.2 (-38.6 to -2.3)
		Fungus	1.49 (1.1 to 1.98)	0.97 (0.7 to 1.3)	-35.2 (-41.6 to -28.1)	0.01 (0 to 0.01)	0 (0 to 0.01)	-22.4 (-37.9 to -0.5)
		Group B Streptococcus	1.68 (1.21 to 2.22)	1.24 (0.89 to 1.63)	-26.4 (-34.0 to -18.2)	0.01 (0 to 0.01)	0 (0 to 0.01)	-11.8 (-30.5 to 13.2)

		Haemophilus influenzae	7.38 (5.61 to 9.65)	3.39 (2.48 to 4.47)	-54.0 (-57.9 to -50.0)	0.01 (0.01 to 0.01)	0 (0 to 0.01)	-63.1 (-70.5 to -53.3)
		Influenza	10.1 (7.64 to 13.2)	0.38 (0.19 to 0.69)	-96.2 (-98.0 to -93.6)	0.03 (0.03 to 0.04)	0 (0 to 0)	-95.6 (-97.8 to -92.1)
		Klebsiella pneumoniae	3.63 (2.75 to 4.72)	2.09 (1.53 to 2.75)	-42.4 (-47.0 to -37.2)	0.02 (0.01 to 0.02)	0.01 (0.01 to 0.01)	-31.0 (-44.5 to -12.0)
		Legionella spp.	4.49 (3.4 to 5.93)	3.2 (2.35 to 4.18)	-28.7 (-36.2 to -21.1)	0.01 (0.01 to 0.02)	0.01 (0.01 to 0.01)	-14.7 (-32.3 to 8.8)
		Mycoplasma spp.	15.8 (11.8 to 20.6)	8.52 (6.29 to 11.3)	-46.1 (-50.3 to -41.2)	0.02 (0.01 to 0.02)	0.01 (0.01 to 0.01)	-35.5 (-48.7 to -17.5)
		Polymicrobial	0.17 (0.1 to 0.28)	0.1 (0.06 to 0.17)	-42.3 (-49.0 to -35.2)	0 (0 to 0)	0 (0 to 0)	-31.0 (-45.5 to -11.3)
		Pseudomonas aeruginosa	4.68 (3.53 to 6.18)	4.28 (3.13 to 5.62)	-8.5 (-16.4 to 1.1)	0.01 (0.01 to 0.02)	0.02 (0.01 to 0.02)	9.5 (-11.6 to 40.8)
		Respiratory syncytial virus	3.6 (2.72 to 4.74)	0.18 (0.09 to 0.33)	-95.0 (-97.4 to -91.5)	0.01 (0 to 0.01)	0 (0 to 0)	-94.2 (-97.1 to -89.7)
		Staphylococcus aureus	6.38 (4.87 to 8.31)	5.51 (4.05 to 7.2)	-13.7 (-19.8 to -6.5)	0.04 (0.03 to 0.04)	0.04 (0.03 to 0.05)	3.3 (-16.5 to 31.7)
		Streptococcus pneumoniae	55 (42.1 to 71.7)	23 (17 to 30.1)	-58.2 (-61.1 to -54.8)	0.14 (0.11 to 0.16)	0.07 (0.06 to 0.08)	-49.9 (-59.4 to -36.5)
		Other viruses	33.5 (25.3 to 43.5)	22 (16.3 to 28.9)	-34.3 (-38.9 to -29.0)	0.03 (0.03 to 0.04)	0.03 (0.02 to 0.03)	-21.4 (-36.7 to 0.1)
		Other bacterial pathogens	5.22 (3.85 to 6.96)	2.48 (1.81 to 3.29)	-52.6 (-56.7 to -47.7)	0.01 (0.01 to 0.01)	0.01 (0.01 to 0.01)	-43.2 (-54.7 to -27.4)
	15-49 years	*Acinetobacter baumannii*	2.65 (2.12 to 3.21)	1.95 (1.58 to 2.36)	-26.4 (-34.2 to -17.8)	0.03 (0.02 to 0.03)	0.02 (0.01 to 0.02)	-41.0 (-51.1 to -30.6)
		Chlamydia spp.	16.2 (13.8 to 19)	9.77 (8.38 to 11.4)	-39.4 (-45.2 to -32.7)	0.04 (0.04 to 0.04)	0.02 (0.02 to 0.02)	-51.4 (-59.6 to -42.6)
		Enterobacter spp.	2.12 (1.74 to 2.53)	2.61 (2.16 to 3.16)	23.5 (9.8 to 39.2)	0.02 (0.02 to 0.02)	0.02 (0.02 to 0.02)	-1.0 (-17.7 to 17.4)
		Escherichia coli	4.7 (3.97 to 5.48)	4.52 (3.87 to 5.23)	-3.7 (-13.5 to 7.2)	0.05 (0.04 to 0.05)	0.04 (0.03 to 0.04)	-22.7 (-36.1 to -9.0)
		Fungus	5.64 (4.75 to 6.64)	5.48 (4.64 to 6.37)	-2.7 (-12.5 to 8.2)	0.05 (0.05 to 0.06)	0.04 (0.04 to 0.05)	-21.9 (-34.4 to -7.7)
		Group B Streptococcus	6.35 (5.37 to 7.53)	7.01 (5.99 to 8.18)	10.6 (-1.1 to 24.3)	0.04 (0.04 to 0.05)	0.04 (0.03 to 0.04)	-11.3 (-25.8 to 5.2)
		Haemophilus influenzae	27.9 (23.9 to 32)	19.2 (16.7 to 22.3)	-30.9 (-37.3 to -24.4)	0.1 (0.09 to 0.11)	0.04 (0.03 to 0.04)	-62.8 (-68.8 to -56.4)
		Influenza	38.2 (33.1 to 43.5)	2.17 (1.12 to 3.69)	-94.3 (-97.1 to -90.3)	0.28 (0.25 to 0.3)	0.01 (0.01 to 0.02)	-95.6 (-97.8 to -92.4)
		Klebsiella pneumoniae	13.7 (11.8 to 15.6)	11.9 (10.3 to 13.6)	-13.4 (-20.6 to -5.5)	0.13 (0.12 to 0.14)	0.09 (0.08 to 0.1)	-30.6 (-41.2 to -19.0)
		Legionella spp.	17 (14.5 to 19.8)	18.1 (15.7 to 20.7)	7.0 (-4.0 to 19.1)	0.11 (0.1 to 0.13)	0.1 (0.08 to 0.11)	-14.2 (-28.7 to 0.6)
		Mycoplasma spp.	59.8 (51.4 to 68.3)	48.3 (41.8 to 55.8)	-19.0 (-26.5 to -11.5)	0.15 (0.14 to 0.17)	0.1 (0.09 to 0.11)	-35.1 (-45.5 to -23.8)
		Polymicrobial	0.66 (0.39 to 1.06)	0.57 (0.32 to 0.96)	-13.4 (-24.2 to -1.9)	0.01 (0 to 0.01)	0 (0 to 0.01)	-30.5 (-42.8 to -16.5)
		Pseudomonas aeruginosa	17.7 (15.1 to 20.5)	24.3 (21 to 27.9)	37.4 (24.6 to 51.5)	0.12 (0.11 to 0.13)	0.13 (0.11 to 0.15)	10.2 (-7.9 to 29.3)

		Respiratory syncytial virus	13.6 (11.7 to 15.8)	1.02 (0.53 to 1.74)	-92.5 (-96.1 to -87.1)	0.05 (0.04 to 0.05)	0 (0 to 0)	-94.2 (-97.1 to -89.7)
		Staphylococcus aureus	24.1 (20.9 to 27.4)	31.3 (27.2 to 35.9)	29.6 (19.5 to 40.4)	0.33 (0.3 to 0.36)	0.34 (0.3 to 0.39)	4.0 (-12.5 to 21.1)
		Streptococcus pneumoniae	208 (181 to 235)	131 (114 to 150)	-37.2 (-42.1 to -32.1)	1.19 (1.09 to 1.29)	0.6 (0.52 to 0.68)	-49.6 (-57.2 to -41.5)
		Other viruses	127 (110 to 143)	125 (108 to 143)	-1.4 (-9.1 to 7.0)	0.28 (0.26 to 0.31)	0.22 (0.2 to 0.26)	-20.9 (-32.8 to -8.0)
		Other bacterial pathogens	19.8 (16.4 to 23.3)	14.1 (11.7 to 16.8)	-28.8 (-35.4 to -21.7)	0.11 (0.09 to 0.12)	0.06 (0.05 to 0.07)	-42.8 (-52.5 to -33.1)
	50-69 years	*Acinetobacter baumannii*	9.88 (8.28 to 11.7)	10.1 (8.53 to 11.8)	2.7 (-8.3 to 14.4)	0.19 (0.16 to 0.22)	0.13 (0.11 to 0.16)	-28.7 (-39.1 to -17.2)
		Chlamydia spp.	35.6 (31.1 to 40.9)	28.7 (25.3 to 32.7)	-19.2 (-27.7 to -10.7)	0.17 (0.15 to 0.19)	0.09 (0.08 to 0.11)	-43.9 (-52.2 to -34.5)
		Enterobacter spp.	7.67 (6.52 to 8.97)	12.9 (11.2 to 14.8)	69.2 (48.5 to 89.5)	0.14 (0.12 to 0.16)	0.16 (0.14 to 0.19)	17.5 (-1.5 to 37.4)
		Escherichia coli	18.8 (16.4 to 21.3)	24.3 (21.6 to 27.2)	29.8 (16.5 to 44.1)	0.35 (0.31 to 0.39)	0.31 (0.28 to 0.35)	-9.9 (-23.0 to 4.5)
		Fungus	17.9 (15.4 to 20.7)	24 (21 to 27.2)	34.1 (18.7 to 49.4)	0.33 (0.29 to 0.37)	0.3 (0.27 to 0.35)	-6.9 (-20.7 to 8.8)
		Group B Streptococcus	16.3 (14 to 18.8)	24.2 (21.2 to 27.4)	49.3 (33.2 to 67.3)	0.22 (0.19 to 0.24)	0.22 (0.19 to 0.25)	3.7 (-13.2 to 21.9)
		Haemophilus influenzae	60.9 (53.7 to 68.3)	61.2 (54.7 to 68.4)	0.6 (-9.6 to 10.5)	0.43 (0.39 to 0.48)	0.2 (0.18 to 0.23)	-53.1 (-59.9 to -45.5)
		Influenza	71.8 (63.6 to 80.3)	5.67 (2.93 to 9.7)	-92.1 (-96.0 to -86.1)	1 (0.9 to 1.1)	0.05 (0.03 to 0.09)	-94.7 (-97.3 to -90.9)
		Klebsiella pneumoniae	38.4 (34.2 to 42.9)	49.9 (44.5 to 55.6)	30.1 (17.8 to 42.4)	0.71 (0.65 to 0.78)	0.64 (0.57 to 0.72)	-9.6 (-22.3 to 4.3)
		Legionella spp.	52.1 (45.2 to 59.2)	76.4 (68.2 to 85.8)	46.9 (30.2 to 63.7)	0.66 (0.59 to 0.75)	0.68 (0.59 to 0.76)	2.0 (-14.6 to 19.8)
		Mycoplasma spp.	59.8 (52.3 to 67.3)	62.4 (55.8 to 69.8)	4.4 (-5.8 to 14.0)	0.29 (0.26 to 0.33)	0.21 (0.19 to 0.24)	-27.5 (-38.2 to -16.6)
		Polymicrobial	2.04 (1.23 to 3.29)	2.45 (1.41 to 4.1)	19.8 (4.7 to 35.0)	0.04 (0.02 to 0.06)	0.03 (0.02 to 0.05)	-16.8 (-29.9 to -1.3)
		Pseudomonas aeruginosa	54.7 (48 to 61.6)	101 (90.6 to 113)	85.0 (66.9 to 103.6)	0.7 (0.63 to 0.78)	0.9 (0.8 to 1.02)	28.5 (10.6 to 48.7)
		Respiratory syncytial virus	19 (16.5 to 21.6)	1.9 (0.98 to 3.28)	-90.0 (-94.9 to -82.4)	0.12 (0.11 to 0.14)	0.01 (0 to 0.01)	-93.3 (-96.7 to -88.4)
		Staphylococcus aureus	65.7 (58.4 to 73.4)	118 (106 to 131)	79.0 (62.7 to 94.4)	1.73 (1.58 to 1.9)	2.14 (1.9 to 2.4)	24.3 (6.7 to 43.9)
		Streptococcus pneumoniae	280 (248 to 312)	250 (225 to 278)	-10.6 (-18.7 to -2.9)	3.06 (2.8 to 3.35)	1.9 (1.69 to 2.13)	-37.9 (-46.5 to -28.1)
		Other viruses	149 (132 to 167)	219 (197 to 243)	47.2 (33.7 to 60.9)	0.64 (0.58 to 0.7)	0.65 (0.58 to 0.73)	2.2 (-12.0 to 18.3)
		Other bacterial pathogens	65.2 (55.4 to 75.6)	63.5 (54.1 to 74.2)	-2.5 (-12.5 to 7.8)	0.68 (0.59 to 0.78)	0.46 (0.39 to 0.54)	-32.2 (-42.2 to -21.9)
	70+ years	*Acinetobacter baumannii*	13.6 (11.4 to 16.3)	27.5 (23.4 to 32.5)	102.5 (81.3 to 125.2)	1.03 (0.86 to 1.2)	1.95 (1.5 to 2.33)	89.7 (61.4 to 117.4)
		Chlamydia spp.	58.6 (51.4 to 67.4)	93.2 (83.3 to 106)	59.4 (44.7 to 76.2)	1.1 (0.96 to 1.21)	1.64 (1.3 to 1.89)	49.3 (26.7 to 70.4)
		Enterobacter spp.	10.3 (8.77 to 12.2)	34.3 (29.8 to 39.2)	233.5 (198.8 to 271.9)	0.74 (0.63 to 0.85)	2.3 (1.8 to 2.74)	212.5 (164.1 to 259.0)

		Escherichia coli	31.8 (27.9 to 36.7)	81.2 (73.4 to 91.3)	155.9 (131.2 to 183.4)	2.35 (2.07 to 2.61)	5.63 (4.46 to 6.45)	139.8 (103.9 to 175.1)
		Fungus	13.8 (11.8 to 16)	36.3 (31.9 to 41.5)	164.3 (137.4 to 193.9)	1 (0.86 to 1.15)	2.48 (2 to 2.91)	147.6 (110.0 to 182.5)
		Group B Streptococcus	20.6 (17.9 to 23.7)	60.4 (54.3 to 68.4)	194.3 (163.6 to 229.3)	1.08 (0.94 to 1.22)	2.98 (2.35 to 3.45)	175.8 (136.0 to 220.0)
		Haemophilus influenzae	75.8 (67.6 to 86.1)	150 (135 to 167)	98.3 (81.0 to 119.0)	2.14 (1.91 to 2.32)	2.66 (2.12 to 3.05)	24.7 (7.5 to 41.3)
		Influenza	151 (135 to 171)	23.4 (12.2 to 40.7)	-84.4 (-92.0 to -73.7)	8.32 (7.43 to 9.02)	1.17 (0.6 to 1.95)	-85.9 (-92.8 to -76.5)
		Klebsiella pneumoniae	58.4 (52.3 to 66.1)	150 (136 to 167)	156.6 (135.5 to 182.4)	4.29 (3.84 to 4.65)	10.3 (8.21 to 11.8)	140.4 (107.5 to 173.2)
		Legionella spp.	51.7 (45.3 to 59.5)	149 (135 to 167)	189.6 (159.8 to 224.3)	2.62 (2.3 to 2.92)	7.09 (5.65 to 8.14)	171.3 (132.5 to 210.1)
		Mycoplasma spp.	69.8 (61.9 to 80.1)	143 (130 to 162)	105.8 (87.2 to 126.8)	1.36 (1.21 to 1.5)	2.63 (2.1 to 3.02)	92.8 (65.2 to 119.0)
		Polymicrobial	3.22 (1.91 to 5.33)	7.63 (4.32 to 13)	136.2 (109.1 to 166.1)	0.24 (0.14 to 0.39)	0.54 (0.3 to 0.9)	121.3 (88.5 to 156.6)
		Pseudomonas aeruginosa	78.7 (70.1 to 90.6)	286 (260 to 319)	264.8 (230.3 to 303.4)	4.01 (3.57 to 4.41)	13.7 (10.8 to 15.7)	241.8 (193.6 to 289.9)
		Respiratory syncytial virus	24.6 (21.6 to 28.4)	4.85 (2.51 to 8.52)	-80.2 (-89.9 to -66.1)	0.62 (0.55 to 0.69)	0.11 (0.06 to 0.19)	-82.1 (-90.8 to -69.7)
		Staphylococcus aureus	117 (105 to 133)	414 (376 to 462)	253.0 (225.9 to 287.2)	12.3 (10.9 to 13.3)	40.6 (32.2 to 46.2)	230.8 (184.6 to 272.5)
		Streptococcus pneumoniae	397 (359 to 452)	700 (636 to 783)	76.3 (62.6 to 93.4)	17.3 (15.4 to 18.7)	28.6 (22.7 to 32.6)	65.2 (41.7 to 85.8)
		Other viruses	209 (188 to 236)	606 (550 to 678)	190.2 (167.4 to 218.1)	3.57 (3.19 to 3.86)	9.72 (7.7 to 11.1)	171.9 (133.7 to 205.8)
		Other bacterial pathogens	120 (104 to 141)	231 (199 to 270)	92.3 (75.1 to 112.5)	5.05 (4.31 to 5.77)	9.12 (7.04 to 10.9)	80.4 (53.4 to 105.3)
Monaco	All Ages	*Acinetobacter baumannii*	2.55 (2.13 to 3)	1.86 (1.61 to 2.14)	-26.7 (-35.7 to -17.1)	0.16 (0.12 to 0.21)	0.13 (0.1 to 0.17)	-19.2 (-38.1 to 6.6)
		Chlamydia spp.	12.8 (11.4 to 14.5)	11 (9.93 to 12.4)	-13.7 (-24.1 to -2.3)	0.2 (0.15 to 0.26)	0.19 (0.15 to 0.24)	-5.1 (-27.6 to 29.6)
		Enterobacter spp.	2.86 (2.45 to 3.3)	5.88 (5.23 to 6.64)	106.2 (80.7 to 131.6)	0.17 (0.13 to 0.22)	0.38 (0.3 to 0.48)	127.3 (71.4 to 208.8)
		Escherichia coli	7.45 (6.62 to 8.42)	14 (12.7 to 15.5)	87.9 (66.3 to 109.5)	0.49 (0.36 to 0.61)	0.99 (0.76 to 1.22)	105.8 (57.8 to 173.7)
		Fungus	4.36 (3.82 to 4.99)	6.61 (5.84 to 7.44)	51.9 (32.6 to 71.2)	0.23 (0.17 to 0.29)	0.38 (0.29 to 0.47)	68.0 (25.7 to 124.8)
		Group B Streptococcus	5.71 (5.1 to 6.43)	9.79 (8.78 to 10.9)	71.8 (51.6 to 94.1)	0.24 (0.18 to 0.31)	0.46 (0.35 to 0.56)	89.4 (44.3 to 154.4)
		Haemophilus influenzae	23.1 (21 to 25.3)	27.3 (24.8 to 30.1)	18.3 (7.2 to 29.4)	0.51 (0.38 to 0.63)	0.44 (0.34 to 0.54)	-12.6 (-32.9 to 16.7)
		Influenza	37.6 (34.1 to 41.3)	4.37 (2.3 to 7.61)	-88.4 (-93.9 to -80.0)	1.85 (1.37 to 2.28)	0.23 (0.11 to 0.38)	-87.6 (-94.0 to -78.6)
		Klebsiella pneumoniae	14.5 (13.2 to 16)	17.4 (15.8 to 19.2)	20.4 (9.4 to 31.2)	0.9 (0.67 to 1.12)	1.2 (0.92 to 1.47)	34.2 (3.0 to 78.9)

		Legionella spp.	18.3 (16.4 to 20.5)	25.8 (23.5 to 28.4)	41.4 (24.9 to 57.4)	0.72 (0.53 to 0.89)	1.11 (0.85 to 1.36)	56.1 (19.6 to 109.2)
		Mycoplasma spp.	23.4 (21.3 to 25.8)	22.2 (20.3 to 24.3)	-5.3 (-14.0 to 3.6)	0.33 (0.24 to 0.41)	0.33 (0.26 to 0.41)	2.0 (-20.9 to 33.9)
		Polymicrobial	0.7 (0.42 to 1.16)	1.13 (0.63 to 1.93)	60.1 (40.7 to 81.6)	0.05 (0.03 to 0.08)	0.08 (0.04 to 0.14)	77.3 (35.2 to 140.2)
		Pseudomonas aeruginosa	23.7 (21.2 to 26.4)	39.1 (35.7 to 42.8)	65.3 (48.3 to 82.0)	1.02 (0.76 to 1.27)	1.83 (1.41 to 2.26)	80.9 (40.1 to 143.4)
		Respiratory syncytial virus	11.3 (10.4 to 12.4)	0.88 (0.46 to 1.49)	-92.2 (-96.0 to -86.7)	0.18 (0.14 to 0.22)	0.01 (0.01 to 0.02)	-91.9 (-96.1 to -85.9)
		Staphylococcus aureus	30.7 (27.8 to 33.8)	68.8 (62.6 to 75.7)	124.6 (105.0 to 144.5)	2.83 (2.12 to 3.49)	6.93 (5.34 to 8.56)	147.0 (91.0 to 227.7)
		Streptococcus pneumoniae	116 (107 to 126)	58.7 (53.7 to 64.3)	-49.4 (-53.7 to -45.4)	3.92 (2.94 to 4.83)	2.23 (1.74 to 2.74)	-42.5 (-55.2 to -22.9)
		Other viruses	76.5 (70.6 to 82.8)	81.1 (74.4 to 88.5)	6.1 (-2.5 to 14.4)	0.97 (0.72 to 1.19)	1.17 (0.91 to 1.43)	22.0 (-5.4 to 62.4)
		Other bacterial pathogens	27 (23.1 to 31.4)	27.7 (23.7 to 32.2)	2.9 (-8.3 to 14.7)	1.02 (0.74 to 1.31)	1.14 (0.86 to 1.45)	12.9 (-12.5 to 51.0)
	Under 5	*Acinetobacter baumannii*	0.01 (0.01 to 0.02)	0.01 (0 to 0.01)	-41.4 (-48.6 to -33.6)	0 (0 to 0)	0 (0 to 0)	-43.7 (-73.0 to 3.7)
		Chlamydia spp.	0.19 (0.16 to 0.23)	0.14 (0.11 to 0.18)	-25.6 (-36.9 to -12.9)	0 (0 to 0)	0 (0 to 0)	-29.1 (-57.1 to 8.4)
		Enterobacter spp.	0.04 (0.03 to 0.05)	0.06 (0.04 to 0.08)	62.7 (36.6 to 94.2)	0 (0 to 0)	0 (0 to 0)	41.1 (-13.5 to 118.0)
		Escherichia coli	0.09 (0.08 to 0.12)	0.17 (0.14 to 0.21)	80.5 (52.5 to 111.1)	0 (0 to 0)	0 (0 to 0)	58.0 (-4.7 to 140.7)
		Fungus	0.02 (0.01 to 0.02)	0.02 (0.02 to 0.03)	34.9 (10.1 to 62.0)	0 (0 to 0)	0 (0 to 0)	41.4 (-17.3 to 118.3)
		Group B Streptococcus	0.09 (0.08 to 0.11)	0.13 (0.1 to 0.16)	41.8 (19.7 to 67.2)	0 (0 to 0)	0 (0 to 0)	19.5 (-26.8 to 86.0)
		Haemophilus influenzae	0.9 (0.75 to 1.09)	0.8 (0.64 to 0.99)	-11.8 (-23.9 to 1.4)	0 (0 to 0)	0 (0 to 0)	-43.7 (-66.1 to -14.3)
		Influenza	0.71 (0.58 to 0.85)	0.05 (0.03 to 0.09)	-92.6 (-96.1 to -87.5)	0.01 (0 to 0.01)	0 (0 to 0)	-92.8 (-96.9 to -86.5)
		Klebsiella pneumoniae	0.34 (0.28 to 0.41)	0.24 (0.19 to 0.3)	-29.0 (-37.8 to -18.9)	0 (0 to 0)	0 (0 to 0)	-27.9 (-55.6 to 9.0)
		Legionella spp.	0.06 (0.04 to 0.08)	0.07 (0.05 to 0.1)	18.2 (0.7 to 38.7)	0 (0 to 0)	0 (0 to 0)	3.0 (-38.3 to 65.4)
		Mycoplasma spp.	0.48 (0.39 to 0.59)	0.71 (0.57 to 0.89)	47.3 (27.1 to 68.7)	0 (0 to 0)	0 (0 to 0)	47.5 (-9.5 to 124.8)
		Polymicrobial	0.02 (0.01 to 0.03)	0.02 (0.01 to 0.03)	-16.4 (-29.0 to -0.6)	0 (0 to 0)	0 (0 to 0)	-26.7 (-66.7 to 41.3)
		Pseudomonas aeruginosa	0.32 (0.26 to 0.39)	0.6 (0.48 to 0.75)	87.7 (63.0 to 118.2)	0 (0 to 0)	0 (0 to 0.01)	75.0 (7.8 to 170.2)
		Respiratory syncytial virus	2.67 (2.25 to 3.19)	0.22 (0.11 to 0.37)	-91.9 (-95.7 to -86.4)	0.01 (0.01 to 0.01)	0 (0 to 0)	-92.3 (-96.7 to -85.4)
		Staphylococcus aureus	0.32 (0.27 to 0.39)	0.58 (0.47 to 0.72)	80.0 (61.6 to 101.9)	0 (0 to 0.01)	0.01 (0.01 to 0.01)	77.6 (9.7 to 168.1)
		Streptococcus pneumoniae	4.4 (3.69 to 5.29)	1.11 (0.89 to 1.37)	-74.8 (-77.2 to -72.0)	0.02 (0.02 to 0.04)	0.01 (0 to 0.01)	-74.1 (-84.2 to -60.3)

		Other viruses	5.21 (4.35 to 6.21)	3.29 (2.63 to 4.08)	-36.9 (-42.7 to -30.1)	0.01 (0.01 to 0.02)	0.01 (0.01 to 0.01)	-34.1 (-60.0 to -0.1)
		Other bacterial pathogens	0.1 (0.07 to 0.13)	0.09 (0.07 to 0.12)	-5.6 (-20.1 to 11.3)	0 (0 to 0)	0 (0 to 0)	-8.4 (-44.9 to 40.4)
	5-14 years	*Acinetobacter baumannii*	0.02 (0.01 to 0.02)	0.01 (0.01 to 0.02)	-18.8 (-28.0 to -8.4)	0 (0 to 0)	0 (0 to 0)	-33.8 (-55.4 to -2.4)
		Chlamydia spp.	0.12 (0.09 to 0.16)	0.12 (0.09 to 0.16)	0.7 (-11.1 to 13.3)	0 (0 to 0)	0 (0 to 0)	-17.9 (-44.2 to 21.3)
		Enterobacter spp.	0.02 (0.01 to 0.03)	0.05 (0.03 to 0.06)	136.0 (106.7 to 166.8)	0 (0 to 0)	0 (0 to 0)	92.5 (30.9 to 183.2)
		Escherichia coli	0.04 (0.03 to 0.05)	0.09 (0.07 to 0.12)	121.4 (96.6 to 147.4)	0 (0 to 0)	0 (0 to 0)	80.6 (20.3 to 163.6)
		Fungus	0.05 (0.04 to 0.06)	0.09 (0.06 to 0.11)	77.1 (54.6 to 99.1)	0 (0 to 0)	0 (0 to 0)	44.4 (-1.7 to 112.9)
		Group B Streptococcus	0.06 (0.04 to 0.08)	0.11 (0.08 to 0.15)	101.9 (76.9 to 128.6)	0 (0 to 0)	0 (0 to 0)	64.6 (12.1 to 143.4)
		Haemophilus influenzae	0.26 (0.2 to 0.35)	0.34 (0.25 to 0.44)	28.3 (16.7 to 40.2)	0 (0 to 0)	0 (0 to 0)	-30.9 (-52.9 to 0.9)
		Influenza	0.35 (0.26 to 0.46)	0.04 (0.02 to 0.08)	-87.5 (-93.5 to -78.8)	0 (0 to 0)	0 (0 to 0)	-90.1 (-95.5 to -81.4)
		Klebsiella pneumoniae	0.12 (0.09 to 0.15)	0.14 (0.11 to 0.18)	21.1 (11.5 to 30.6)	0 (0 to 0)	0 (0 to 0)	-1.3 (-32.5 to 43.2)
		Legionella spp.	0.18 (0.14 to 0.24)	0.3 (0.22 to 0.39)	64.9 (46.6 to 85.5)	0 (0 to 0)	0 (0 to 0)	34.4 (-8.6 to 97.1)
		Mycoplasma spp.	0.55 (0.4 to 0.72)	0.68 (0.5 to 0.9)	22.8 (11.1 to 35.2)	0 (0 to 0)	0 (0 to 0)	0.2 (-32.0 to 42.9)
		Polymicrobial	0 (0 to 0.01)	0.01 (0 to 0.02)	81.3 (58.4 to 107.1)	0 (0 to 0)	0 (0 to 0)	47.8 (-0.9 to 121.4)
		Pseudomonas aeruginosa	0.18 (0.13 to 0.24)	0.36 (0.27 to 0.48)	99.1 (79.8 to 118.6)	0 (0 to 0)	0 (0 to 0)	62.4 (10.8 to 134.6)
		Respiratory syncytial virus	0.15 (0.11 to 0.2)	0.01 (0.01 to 0.03)	-90.7 (-95.2 to -84.2)	0 (0 to 0)	0 (0 to 0)	-92.7 (-96.6 to -86.1)
		Staphylococcus aureus	0.23 (0.17 to 0.3)	0.58 (0.43 to 0.75)	150.6 (131.8 to 170.5)	0 (0 to 0)	0 (0 to 0)	104.3 (40.2 to 196.4)
		Streptococcus pneumoniae	1.87 (1.4 to 2.43)	1 (0.74 to 1.3)	-46.6 (-50.6 to -42.8)	0 (0 to 0.01)	0 (0 to 0)	-56.5 (-70.3 to -37.9)
		Other viruses	1.34 (1.01 to 1.76)	1.5 (1.12 to 1.98)	11.6 (2.8 to 20.5)	0 (0 to 0)	0 (0 to 0)	-9.0 (-37.3 to 31.2)
		Other bacterial pathogens	0.17 (0.12 to 0.22)	0.19 (0.14 to 0.26)	17.2 (5.1 to 30.4)	0 (0 to 0)	0 (0 to 0)	-4.4 (-35.3 to 38.7)
	15-49 years	*Acinetobacter baumannii*	0.12 (0.1 to 0.15)	0.07 (0.06 to 0.08)	-41.1 (-48.6 to -33.4)	0 (0 to 0)	0 (0 to 0)	-46.2 (-65.3 to -20.4)
		Chlamydia spp.	0.87 (0.73 to 1.01)	0.63 (0.54 to 0.73)	-27.0 (-35.3 to -18.1)	0 (0 to 0)	0 (0 to 0)	-33.2 (-57.1 to -1.3)
		Enterobacter spp.	0.14 (0.11 to 0.17)	0.24 (0.2 to 0.28)	71.1 (50.0 to 94.4)	0 (0 to 0)	0 (0 to 0)	56.4 (0.0 to 133.1)
		Escherichia coli	0.28 (0.24 to 0.33)	0.45 (0.39 to 0.52)	60.5 (41.8 to 78.8)	0 (0 to 0.01)	0.01 (0 to 0.01)	46.7 (-6.3 to 111.5)
		Fungus	0.34 (0.28 to 0.4)	0.43 (0.37 to 0.5)	28.5 (12.5 to 44.9)	0 (0 to 0.01)	0.01 (0 to 0.01)	17.4 (-24.7 to 71.7)
		Group B Streptococcus	0.4 (0.34 to 0.47)	0.58 (0.5 to 0.68)	46.4 (28.7 to 67.0)	0 (0 to 0.01)	0.01 (0 to 0.01)	33.8 (-13.8 to 97.0)

		Haemophilus influenzae	1.84 (1.59 to 2.11)	1.71 (1.49 to 1.94)	-7.0 (-15.6 to 1.7)	0.01 (0.01 to 0.01)	0.01 (0 to 0.01)	-43.8 (-63.9 to -19.9)
		Influenza	2.47 (2.12 to 2.83)	0.22 (0.12 to 0.38)	-90.9 (-95.4 to -84.3)	0.03 (0.02 to 0.03)	0 (0 to 0)	-92.0 (-96.3 to -85.4)
		Klebsiella pneumoniae	0.82 (0.71 to 0.94)	0.72 (0.62 to 0.82)	-12.2 (-19.6 to -4.4)	0.01 (0.01 to 0.01)	0.01 (0.01 to 0.01)	-19.8 (-48.0 to 15.0)
		Legionella spp.	1.27 (1.09 to 1.48)	1.52 (1.32 to 1.73)	19.6 (6.1 to 34.3)	0.01 (0.01 to 0.02)	0.01 (0.01 to 0.02)	9.3 (-30.5 to 61.7)
		Mycoplasma spp.	3.87 (3.35 to 4.45)	3.44 (3 to 3.93)	-10.9 (-19.4 to -2.3)	0.01 (0.01 to 0.02)	0.01 (0.01 to 0.02)	-18.6 (-48.3 to 18.4)
		Polymicrobial	0.04 (0.02 to 0.06)	0.05 (0.03 to 0.08)	31.4 (14.3 to 49.9)	0 (0 to 0)	0 (0 to 0)	20.2 (-22.2 to 75.8)
		Pseudomonas aeruginosa	1.28 (1.1 to 1.49)	1.85 (1.61 to 2.11)	44.4 (31.2 to 58.8)	0.01 (0.01 to 0.02)	0.02 (0.01 to 0.02)	32.0 (-15.3 to 90.3)
		Respiratory syncytial virus	1.04 (0.9 to 1.21)	0.07 (0.04 to 0.12)	-93.3 (-96.5 to -88.1)	0.01 (0 to 0.01)	0 (0 to 0)	-94.1 (-97.2 to -89.4)
		Staphylococcus aureus	1.61 (1.39 to 1.84)	2.93 (2.57 to 3.32)	81.7 (67.2 to 96.7)	0.03 (0.03 to 0.04)	0.05 (0.04 to 0.07)	66.1 (6.9 to 138.6)
		Streptococcus pneumoniae	13.1 (11.4 to 15)	5.08 (4.44 to 5.76)	-61.3 (-64.4 to -58.0)	0.11 (0.09 to 0.14)	0.04 (0.03 to 0.05)	-64.6 (-77.3 to -49.3)
		Other viruses	9.46 (8.19 to 10.8)	7.65 (6.67 to 8.63)	-19.1 (-25.6 to -12.6)	0.03 (0.03 to 0.04)	0.02 (0.02 to 0.03)	-26.0 (-52.0 to 6.0)
		Other bacterial pathogens	1.17 (0.97 to 1.39)	0.99 (0.82 to 1.18)	-15.0 (-23.3 to -5.3)	0.01 (0.01 to 0.01)	0.01 (0 to 0.01)	-22.3 (-49.3 to 11.8)
	50-69 years	*Acinetobacter baumannii*	0.7 (0.58 to 0.84)	0.44 (0.38 to 0.52)	-36.3 (-45.0 to -27.5)	0.01 (0.01 to 0.02)	0.01 (0.01 to 0.01)	-45.6 (-65.2 to -22.2)
		Chlamydia spp.	2.99 (2.58 to 3.48)	2.23 (1.96 to 2.55)	-25.4 (-34.9 to -14.5)	0.01 (0.01 to 0.02)	0.01 (0.01 to 0.01)	-36.4 (-59.3 to -7.4)
		Enterobacter spp.	0.79 (0.66 to 0.93)	1.42 (1.22 to 1.62)	79.6 (54.2 to 104.2)	0.01 (0.01 to 0.02)	0.02 (0.02 to 0.03)	53.1 (-2.1 to 120.7)
		Escherichia coli	1.77 (1.53 to 2.01)	2.84 (2.51 to 3.21)	61.3 (41.1 to 81.5)	0.03 (0.02 to 0.05)	0.05 (0.03 to 0.06)	37.5 (-11.7 to 100.6)
		Fungus	1.68 (1.44 to 1.94)	2.28 (1.99 to 2.62)	35.8 (17.8 to 53.7)	0.03 (0.02 to 0.04)	0.04 (0.03 to 0.05)	15.8 (-25.8 to 68.7)
		Group B Streptococcus	1.6 (1.38 to 1.84)	2.4 (2.08 to 2.74)	50.2 (29.5 to 70.8)	0.02 (0.02 to 0.03)	0.03 (0.02 to 0.04)	28.0 (-18.4 to 89.6)
		Haemophilus influenzae	6.3 (5.57 to 7.06)	6.63 (5.84 to 7.46)	5.3 (-6.2 to 17.1)	0.05 (0.03 to 0.06)	0.03 (0.02 to 0.03)	-40.7 (-62.0 to -14.2)
		Influenza	7.27 (6.44 to 8.14)	0.73 (0.39 to 1.26)	-89.9 (-94.7 to -82.2)	0.1 (0.08 to 0.14)	0.01 (0 to 0.02)	-91.7 (-96.2 to -84.4)
		Klebsiella pneumoniae	3.6 (3.2 to 4.07)	3.81 (3.38 to 4.26)	6.0 (-5.0 to 16.9)	0.07 (0.05 to 0.09)	0.06 (0.05 to 0.08)	-9.7 (-41.5 to 30.7)
		Legionella spp.	6.13 (5.33 to 6.95)	7.62 (6.72 to 8.56)	24.7 (9.1 to 40.9)	0.08 (0.06 to 0.11)	0.08 (0.06 to 0.11)	6.3 (-32.0 to 56.1)
		Mycoplasma spp.	6.07 (5.32 to 6.81)	4.92 (4.33 to 5.56)	-18.8 (-28.3 to -9.1)	0.03 (0.02 to 0.04)	0.02 (0.02 to 0.03)	-30.7 (-56.0 to -0.6)
		Polymicrobial	0.17 (0.1 to 0.28)	0.24 (0.14 to 0.41)	40.3 (20.6 to 60.2)	0 (0 to 0.01)	0 (0 to 0.01)	19.7 (-24.8 to 75.8)
		Pseudomonas aeruginosa	6.22 (5.45 to 7.02)	8.85 (7.81 to 9.88)	42.5 (26.5 to 59.1)	0.08 (0.06 to 0.11)	0.1 (0.07 to 0.13)	21.5 (-22.0 to 75.5)

		Respiratory syncytial virus	2.28 (1.98 to 2.61)	0.15 (0.08 to 0.26)	-93.3 (-96.5 to -88.1)	0.01 (0.01 to 0.02)	0 (0 to 0)	-94.4 (-97.6 to -89.5)
		Staphylococcus aureus	6.89 (6.11 to 7.7)	13.3 (11.8 to 14.9)	93.7 (74.0 to 113.8)	0.19 (0.14 to 0.25)	0.3 (0.22 to 0.39)	65.1 (6.8 to 140.0)
		Streptococcus pneumoniae	27.7 (24.7 to 31)	12.7 (11.3 to 14.2)	-54.2 (-58.9 to -49.7)	0.31 (0.23 to 0.42)	0.12 (0.09 to 0.16)	-60.9 (-74.9 to -43.8)
		Other viruses	17.4 (15.5 to 19.5)	17 (15.1 to 19)	-2.3 (-11.9 to 7.1)	0.08 (0.06 to 0.1)	0.06 (0.05 to 0.08)	-16.7 (-46.3 to 20.4)
		Other bacterial pathogens	6.02 (5.08 to 7.09)	5.3 (4.44 to 6.24)	-11.8 (-22.4 to -0.1)	0.06 (0.05 to 0.09)	0.05 (0.03 to 0.06)	-24.7 (-51.8 to 10.6)
	70+ years	*Acinetobacter baumannii*	1.7 (1.39 to 2.02)	1.32 (1.12 to 1.56)	-21.6 (-31.9 to -9.7)	0.15 (0.1 to 0.19)	0.12 (0.09 to 0.15)	-16.3 (-36.3 to 11.8)
		Chlamydia spp.	8.65 (7.48 to 9.9)	7.91 (6.96 to 9.08)	-8.2 (-20.4 to 5.4)	0.19 (0.14 to 0.23)	0.18 (0.14 to 0.23)	-2.0 (-26.0 to 34.3)
		Enterobacter spp.	1.87 (1.56 to 2.22)	4.12 (3.58 to 4.74)	120.8 (91.9 to 151.7)	0.16 (0.11 to 0.2)	0.36 (0.27 to 0.45)	135.9 (77.2 to 221.5)
		Escherichia coli	5.26 (4.57 to 6.03)	10.4 (9.29 to 11.7)	98.3 (73.0 to 124.5)	0.45 (0.33 to 0.56)	0.94 (0.72 to 1.17)	111.8 (61.7 to 182.4)
		Fungus	2.28 (1.95 to 2.64)	3.79 (3.23 to 4.35)	67.0 (43.7 to 91.7)	0.19 (0.14 to 0.24)	0.34 (0.26 to 0.42)	78.4 (33.4 to 139.4)
		Group B Streptococcus	3.56 (3.1 to 4.09)	6.56 (5.73 to 7.49)	84.7 (59.7 to 111.2)	0.22 (0.16 to 0.28)	0.42 (0.32 to 0.52)	97.2 (49.9 to 167.7)
		Haemophilus influenzae	13.8 (12.2 to 15.5)	17.8 (15.8 to 20)	29.5 (15.4 to 44.2)	0.45 (0.33 to 0.56)	0.41 (0.31 to 0.5)	-8.6 (-30.2 to 22.3)
		Influenza	26.8 (23.7 to 30)	3.32 (1.72 to 5.7)	-87.6 (-93.6 to -78.6)	1.71 (1.26 to 2.13)	0.22 (0.11 to 0.36)	-87.2 (-93.8 to -78.1)
		Klebsiella pneumoniae	9.63 (8.53 to 10.8)	12.5 (11.1 to 14.1)	30.3 (16.6 to 44.9)	0.82 (0.6 to 1.02)	1.13 (0.85 to 1.39)	39.2 (6.8 to 85.7)
		Legionella spp.	10.7 (9.34 to 12.2)	16.3 (14.6 to 18.4)	53.4 (32.4 to 73.6)	0.62 (0.45 to 0.78)	1.01 (0.77 to 1.24)	63.8 (24.6 to 120.8)
		Mycoplasma spp.	12.4 (10.9 to 14)	12.4 (11 to 14)	-0.1 (-11.9 to 11.8)	0.28 (0.21 to 0.35)	0.3 (0.22 to 0.37)	6.7 (-18.0 to 40.9)
		Polymicrobial	0.47 (0.27 to 0.8)	0.82 (0.45 to 1.4)	72.6 (50.1 to 99.2)	0.04 (0.02 to 0.07)	0.08 (0.04 to 0.14)	84.3 (39.1 to 151.5)
		Pseudomonas aeruginosa	15.7 (13.8 to 17.8)	27.5 (24.5 to 30.9)	75.2 (54.4 to 95.8)	0.93 (0.68 to 1.16)	1.71 (1.31 to 2.12)	87.1 (44.5 to 153.3)
		Respiratory syncytial virus	5.18 (4.5 to 5.93)	0.43 (0.22 to 0.74)	-91.7 (-95.7 to -85.8)	0.15 (0.11 to 0.19)	0.01 (0.01 to 0.02)	-91.5 (-95.9 to -85.3)
		Staphylococcus aureus	21.6 (19.2 to 24.3)	51.4 (46.1 to 57.4)	138.2 (113.5 to 162.7)	2.61 (1.92 to 3.24)	6.56 (5.01 to 8.16)	154.3 (95.5 to 238.0)
		Streptococcus pneumoniae	69.1 (61.4 to 77.4)	38.9 (34.8 to 43.5)	-43.7 (-49.6 to -37.8)	3.47 (2.56 to 4.32)	2.06 (1.58 to 2.56)	-39.8 (-53.5 to -19.3)
		Other viruses	43 (38.2 to 48.1)	51.6 (46.1 to 57.9)	20.2 (7.6 to 33.2)	0.85 (0.63 to 1.06)	1.08 (0.81 to 1.33)	28.3 (-1.3 to 70.8)
		Other bacterial pathogens	19.5 (16.4 to 23.1)	21.2 (17.9 to 24.6)	8.5 (-4.6 to 22.0)	0.95 (0.68 to 1.22)	1.09 (0.82 to 1.38)	15.9 (-10.3 to 56.2)
Netherlands	All Ages	*Acinetobacter baumannii*	727 (618 to 853)	500 (431 to 581)	-31.1 (-39.0 to -22.7)	43 (35.8 to 49.8)	33 (26.5 to 38.4)	-23.2 (-32.3 to -14.0)
		Chlamydia spp.	3620 (3230 to 3990)	3230 (2890 to 3610)	-10.6 (-20.2 to 0.1)	52.2 (45.1 to 58.1)	52.4 (43.2 to 59.8)	0.5 (-11.1 to 12.2)

		Enterobacter spp.	756 (657 to 861)	1750 (1550 to 1960)	131.4 (105.6 to 161.6)	41.4 (34.3 to 47.7)	107 (87.1 to 122)	159.0 (131.1 to 190.0)
		Escherichia coli	1990 (1780 to 2200)	4130 (3750 to 4560)	108.3 (87.0 to 131.7)	120 (103 to 134)	277 (228 to 309)	130.3 (106.1 to 153.8)
		Fungus	1210 (1070 to 1360)	1980 (1760 to 2230)	64.6 (44.8 to 85.2)	55.8 (47.7 to 63.3)	104 (86.1 to 120)	87.1 (66.3 to 109.2)
		Group B Streptococcus	1560 (1400 to 1740)	2920 (2620 to 3270)	86.9 (65.3 to 109.0)	59.5 (50.1 to 66.8)	125 (104 to 141)	110.8 (86.3 to 136.8)
		Haemophilus influenzae	6470 (5940 to 6970)	8450 (7710 to 9270)	30.7 (19.9 to 41.8)	124 (108 to 134)	128 (106 to 141)	3.1 (-5.9 to 11.0)
		Influenza	10200 (9280 to 10900)	1330 (203 to 3990)	-86.9 (-98.0 to -60.5)	449 (390 to 488)	64.6 (10.2 to 204)	-85.6 (-97.8 to -55.3)
		Klebsiella pneumoniae	3990 (3650 to 4280)	5020 (4580 to 5500)	25.9 (15.5 to 36.8)	223 (194 to 243)	323 (267 to 359)	44.9 (33.2 to 55.9)
		Legionella spp.	4760 (4250 to 5260)	7910 (7240 to 8670)	66.6 (49.1 to 85.3)	167 (144 to 186)	313 (265 to 348)	87.9 (69.0 to 108.0)
		Mycoplasma spp.	6910 (6320 to 7520)	7000 (6410 to 7630)	1.4 (-7.4 to 10.0)	80.5 (70.3 to 88.1)	90.9 (75.9 to 101)	13.0 (2.4 to 23.7)
		Polymicrobial	198 (117 to 326)	329 (183 to 562)	65.7 (43.7 to 86.9)	11.8 (6.99 to 19.8)	21.9 (12.1 to 37.7)	84.2 (59.7 to 108.5)
		Pseudomonas aeruginosa	6160 (5600 to 6740)	11600 (10500 to 12700)	88.0 (70.8 to 105.4)	241 (210 to 265)	504 (418 to 558)	108.9 (90.2 to 126.7)
		Respiratory syncytial virus	3770 (3430 to 4150)	296 (46.3 to 889)	-92.1 (-98.8 to -76.0)	46.5 (40.8 to 51.1)	4.02 (0.62 to 12.5)	-91.3 (-98.7 to -73.3)
		Staphylococcus aureus	8060 (7350 to 8680)	20300 (18600 to 22400)	152.3 (132.2 to 173.5)	681 (592 to 739)	1920 (1600 to 2130)	182.4 (161.1 to 202.8)
		Streptococcus pneumoniae	33700 (31200 to 36000)	17200 (15800 to 18800)	-49.0 (-52.6 to -45.0)	968 (847 to 1050)	597 (501 to 659)	-38.4 (-43.4 to -34.0)
		Other viruses	22200 (20600 to 23700)	24800 (22900 to 27000)	11.8 (3.8 to 20.9)	233 (205 to 252)	323 (271 to 357)	38.7 (27.6 to 49.1)
		Other bacterial pathogens	7180 (6220 to 8160)	8050 (6950 to 9380)	12.2 (0.8 to 25.1)	253 (211 to 293)	314 (248 to 371)	24.3 (11.7 to 37.7)
	Under 5	*Acinetobacter baumannii*	7.43 (4.85 to 11.1)	3.34 (2.02 to 5.23)	-55.3 (-62.6 to -48.2)	0.29 (0.16 to 0.49)	0.12 (0.06 to 0.22)	-58.5 (-66.7 to -49.3)
		Chlamydia spp.	113 (92.2 to 136)	59.8 (47.8 to 74.4)	-46.8 (-54.7 to -38.3)	0.49 (0.44 to 0.55)	0.15 (0.12 to 0.17)	-69.3 (-74.8 to -63.4)
		Enterobacter spp.	20 (14.7 to 26.6)	24.8 (18.1 to 33)	24.9 (4.3 to 47.2)	0.36 (0.27 to 0.5)	0.2 (0.14 to 0.28)	-43.8 (-54.2 to -32.9)
		Escherichia coli	54 (43.7 to 66.9)	72.2 (56.8 to 89.5)	34.0 (12.5 to 56.0)	0.93 (0.82 to 1.06)	0.52 (0.43 to 0.6)	-44.5 (-54.0 to -34.3)
		Fungus	10.3 (7.19 to 14.6)	9.93 (6.93 to 14.3)	-2.7 (-20.4 to 16.1)	0.11 (0.08 to 0.15)	0.04 (0.03 to 0.06)	-61.5 (-70.5 to -50.3)
		Group B Streptococcus	51.8 (41.9 to 64.3)	55.4 (43.6 to 69.7)	7.0 (-9.4 to 25.0)	0.76 (0.66 to 0.87)	0.38 (0.32 to 0.44)	-49.9 (-58.6 to -40.8)
		Haemophilus influenzae	512 (421 to 623)	349 (278 to 436)	-31.7 (-41.3 to -22.3)	2.53 (2.24 to 2.83)	0.41 (0.35 to 0.49)	-83.6 (-86.8 to -79.9)
		Influenza	405 (334 to 491)	23.2 (3.71 to 66.9)	-94.3 (-99.1 to -83.3)	3.74 (3.36 to 4.16)	0.08 (0.01 to 0.25)	-97.8 (-99.7 to -93.3)

		Klebsiella pneumoniae	195 (162 to 240)	101 (81.5 to 126)	-48.1 (-54.8 to -40.6)	2.55 (2.29 to 2.84)	0.66 (0.56 to 0.76)	-74.1 (-78.8 to -69.0)
		Legionella spp.	31.1 (22 to 43.6)	29.8 (19.8 to 42)	-4.3 (-18.8 to 10.7)	0.47 (0.36 to 0.59)	0.25 (0.19 to 0.32)	-47.0 (-56.6 to -36.7)
		Mycoplasma spp.	275 (224 to 339)	291 (234 to 362)	5.9 (-8.0 to 20.7)	0.91 (0.8 to 1.04)	0.39 (0.32 to 0.46)	-57.2 (-65.7 to -47.3)
		Polymicrobial	10.1 (5.51 to 16.7)	7.37 (4.03 to 12.4)	-26.8 (-37.6 to -16.4)	0.51 (0.25 to 0.88)	0.3 (0.14 to 0.55)	-41.0 (-52.1 to -28.6)
		Pseudomonas aeruginosa	177 (145 to 217)	251 (201 to 311)	41.5 (22.6 to 63.1)	1.82 (1.63 to 2.05)	1.06 (0.9 to 1.23)	-41.5 (-51.9 to -30.1)
		Respiratory syncytial virus	1460 (1220 to 1750)	91.2 (14.5 to 263)	-93.7 (-99.0 to -81.7)	6.72 (6.12 to 7.44)	0.17 (0.03 to 0.53)	-97.4 (-99.6 to -92.0)
		Staphylococcus aureus	183 (152 to 222)	250 (200 to 309)	36.3 (20.6 to 52.1)	3.35 (3.04 to 3.68)	1.87 (1.57 to 2.18)	-44.2 (-53.9 to -33.3)
		Streptococcus pneumoniae	2520 (2100 to 3050)	470 (377 to 583)	-81.4 (-83.6 to -79.3)	17.1 (15.4 to 19)	1.23 (1.02 to 1.45)	-92.8 (-94.2 to -91.2)
		Other viruses	2880 (2390 to 3480)	1430 (1150 to 1760)	-50.3 (-55.8 to -44.9)	7.84 (7.04 to 8.67)	1.75 (1.48 to 2.04)	-77.6 (-81.7 to -73.1)
		Other bacterial pathogens	56.9 (42.8 to 74.3)	38.8 (29.1 to 51.6)	-31.6 (-42.3 to -19.6)	0.49 (0.38 to 0.63)	0.16 (0.12 to 0.23)	-66.5 (-73.6 to -58.6)
	5-14 years	*Acinetobacter baumannii*	11.9 (8.65 to 16.2)	5.75 (4.16 to 7.78)	-51.5 (-58.2 to -44.5)	0.07 (0.05 to 0.08)	0.01 (0.01 to 0.02)	-78.5 (-82.2 to -74.5)
		Chlamydia spp.	82.6 (60.3 to 110)	56.3 (40.8 to 75.1)	-31.6 (-40.3 to -22.4)	0.11 (0.1 to 0.13)	0.03 (0.03 to 0.04)	-69.7 (-74.9 to -64.1)
		Enterobacter spp.	12.7 (9.08 to 17.3)	21.6 (15.3 to 28.9)	70.7 (49.1 to 93.8)	0.07 (0.06 to 0.08)	0.05 (0.04 to 0.06)	-24.4 (-37.4 to -9.7)
		Escherichia coli	26.2 (19 to 35)	41.6 (30.5 to 54.9)	59.1 (41.1 to 78.9)	0.14 (0.12 to 0.16)	0.1 (0.09 to 0.11)	-29.5 (-41.3 to -17.0)
		Fungus	31.4 (23 to 42.5)	38.7 (28.3 to 51.4)	23.7 (7.9 to 41.0)	0.17 (0.14 to 0.19)	0.09 (0.08 to 0.11)	-45.2 (-54.7 to -34.0)
		Group B Streptococcus	36.7 (26.6 to 49)	52.6 (38.3 to 69.9)	43.9 (23.9 to 65.6)	0.14 (0.12 to 0.16)	0.09 (0.08 to 0.1)	-36.3 (-46.5 to -23.9)
		Haemophilus influenzae	167 (126 to 220)	157 (115 to 204)	-6.2 (-15.5 to 3.7)	0.34 (0.31 to 0.38)	0.1 (0.08 to 0.11)	-71.9 (-76.0 to -66.7)
		Influenza	225 (168 to 295)	20.6 (3.16 to 63)	-90.8 (-98.5 to -72.0)	0.91 (0.81 to 1)	0.04 (0.01 to 0.11)	-96.1 (-99.4 to -88.0)
		Klebsiella pneumoniae	76.3 (56.8 to 100)	61.8 (45.9 to 80.3)	-18.9 (-26.6 to -10.9)	0.41 (0.37 to 0.45)	0.15 (0.13 to 0.16)	-64.1 (-69.3 to -57.7)
		Legionella spp.	112 (83.2 to 149)	140 (104 to 184)	25.4 (10.2 to 40.8)	0.41 (0.37 to 0.46)	0.23 (0.2 to 0.26)	-44.5 (-54.0 to -34.1)
		Mycoplasma spp.	353 (260 to 464)	321 (238 to 424)	-9.0 (-18.3 to 0.5)	0.5 (0.44 to 0.56)	0.2 (0.18 to 0.23)	-59.7 (-66.1 to -51.5)
		Polymicrobial	3.34 (1.87 to 5.48)	4.08 (2.12 to 6.91)	21.7 (4.7 to 40.1)	0.02 (0.01 to 0.03)	0.01 (0.01 to 0.02)	-46.1 (-55.6 to -35.3)
		Pseudomonas aeruginosa	114 (84.3 to 149)	169 (126 to 219)	48.4 (32.2 to 64.7)	0.42 (0.38 to 0.47)	0.28 (0.24 to 0.31)	-34.3 (-44.3 to -22.6)
		Respiratory syncytial virus	91.5 (68.4 to 121)	6.47 (1 to 20.1)	-92.9 (-98.9 to -78.3)	0.17 (0.15 to 0.19)	0.01 (0 to 0.02)	-97.0 (-99.5 to -90.7)

		Staphylococcus aureus	146 (109 to 191)	265 (197 to 343)	81.6 (66.2 to 98.6)	1.11 (1 to 1.22)	0.89 (0.78 to 1.01)	-19.6 (-31.3 to -6.5)
		Streptococcus pneumoniae	1210 (902 to 1570)	433 (321 to 560)	-64.1 (-67.2 to -60.7)	3.83 (3.45 to 4.19)	0.61 (0.53 to 0.68)	-84.1 (-86.3 to -81.4)
		Other viruses	834 (621 to 1100)	672 (500 to 875)	-19.3 (-26.4 to -11.8)	1.04 (0.94 to 1.14)	0.37 (0.32 to 0.42)	-64.3 (-69.3 to -58.4)
		Other bacterial pathogens	109 (79.3 to 145)	89 (64.7 to 117)	-17.9 (-27.2 to -7.9)	0.33 (0.28 to 0.38)	0.12 (0.1 to 0.14)	-63.6 (-69.7 to -57.0)
	15-49 years	*Acinetobacter baumannii*	55.2 (43.4 to 67.8)	24.4 (19.9 to 29.8)	-55.6 (-61.0 to -49.5)	0.61 (0.52 to 0.72)	0.17 (0.15 to 0.2)	-71.3 (-75.1 to -67.3)
		Chlamydia spp.	383 (323 to 454)	239 (202 to 281)	-37.5 (-44.7 to -30.1)	1.05 (0.95 to 1.16)	0.42 (0.38 to 0.48)	-59.6 (-64.7 to -53.9)
		Enterobacter spp.	58.9 (47.5 to 71.1)	91.8 (75.3 to 111)	56.3 (37.5 to 77.6)	0.62 (0.53 to 0.71)	0.62 (0.54 to 0.71)	1.0 (-12.5 to 16.2)
		Escherichia coli	122 (101 to 145)	177 (149 to 209)	45.6 (29.4 to 64.0)	1.32 (1.19 to 1.45)	1.24 (1.13 to 1.36)	-5.8 (-16.8 to 6.0)
		Fungus	146 (122 to 175)	164 (138 to 198)	13.2 (-1.4 to 28.3)	1.56 (1.39 to 1.75)	1.14 (1 to 1.29)	-26.8 (-35.7 to -16.9)
		Group B Streptococcus	170 (140 to 205)	224 (185 to 267)	31.7 (14.5 to 49.5)	1.31 (1.17 to 1.47)	1.11 (0.99 to 1.25)	-14.8 (-26.2 to -3.0)
		Haemophilus influenzae	776 (654 to 913)	666 (563 to 775)	-14.1 (-21.9 to -5.6)	3.21 (2.99 to 3.45)	1.2 (1.1 to 1.31)	-62.5 (-66.4 to -58.4)
		Influenza	1040 (889 to 1210)	88.2 (13.4 to 274)	-91.6 (-98.7 to -74.2)	8.45 (7.91 to 8.99)	0.44 (0.07 to 1.36)	-94.8 (-99.2 to -83.6)
		Klebsiella pneumoniae	354 (300 to 414)	263 (224 to 307)	-25.8 (-32.1 to -19.1)	3.82 (3.57 to 4.08)	1.83 (1.69 to 1.98)	-52.0 (-56.5 to -46.9)
		Legionella spp.	521 (438 to 611)	596 (505 to 692)	14.7 (1.4 to 27.9)	3.86 (3.51 to 4.24)	2.86 (2.62 to 3.1)	-25.8 (-35.0 to -16.6)
		Mycoplasma spp.	1640 (1390 to 1900)	1360 (1160 to 1590)	-16.7 (-24.4 to -8.5)	4.68 (4.35 to 5.05)	2.52 (2.32 to 2.73)	-46.2 (-51.9 to -39.8)
		Polymicrobial	15.5 (8.88 to 25.7)	17.4 (9.38 to 29.7)	11.4 (-2.9 to 28.9)	0.17 (0.1 to 0.28)	0.12 (0.07 to 0.21)	-28.0 (-38.3 to -17.7)
		Pseudomonas aeruginosa	528 (445 to 621)	716 (616 to 831)	35.8 (23.4 to 49.9)	3.95 (3.64 to 4.27)	3.46 (3.21 to 3.76)	-12.2 (-21.2 to -1.7)
		Respiratory syncytial virus	425 (356 to 499)	27.6 (4.21 to 85)	-93.5 (-99.0 to -79.9)	1.57 (1.44 to 1.71)	0.06 (0.01 to 0.2)	-96.0 (-99.4 to -87.6)
		Staphylococcus aureus	678 (577 to 794)	1130 (967 to 1310)	66.2 (53.6 to 79.7)	10.4 (9.77 to 11)	11.1 (10.4 to 12)	7.5 (-2.1 to 17.6)
		Streptococcus pneumoniae	5590 (4780 to 6500)	1840 (1580 to 2130)	-67.1 (-69.6 to -64.5)	35.6 (33.7 to 37.8)	7.57 (7.03 to 8.15)	-78.8 (-80.7 to -76.7)
		Other viruses	3870 (3300 to 4510)	2850 (2440 to 3310)	-26.2 (-31.6 to -20.4)	9.69 (9.16 to 10.3)	4.62 (4.3 to 4.98)	-52.3 (-56.7 to -47.9)
		Other bacterial pathogens	504 (410 to 605)	378 (309 to 457)	-24.9 (-33.1 to -16.6)	3.07 (2.69 to 3.47)	1.49 (1.28 to 1.71)	-51.4 (-57.2 to -45.5)
	50-69 years	*Acinetobacter baumannii*	202 (168 to 238)	130 (109 to 153)	-35.3 (-43.2 to -27.4)	3.04 (2.59 to 3.58)	2.01 (1.71 to 2.34)	-33.8 (-43.9 to -23.4)
		Chlamydia spp.	829 (720 to 943)	705 (613 to 802)	-14.8 (-24.7 to -3.0)	3.11 (2.76 to 3.5)	2.7 (2.35 to 3.05)	-12.9 (-26.3 to 2.1)

		Enterobacter spp.	209 (178 to 243)	457 (394 to 524)	119.3 (92.3 to 150.8)	2.99 (2.58 to 3.44)	6.68 (5.82 to 7.67)	124.3 (91.1 to 161.1)
		Escherichia coli	477 (413 to 536)	921 (824 to 1030)	93.7 (71.5 to 116.3)	7.05 (6.27 to 7.89)	13.9 (12.5 to 15.3)	98.1 (71.1 to 126.4)
		Fungus	455 (392 to 520)	726 (632 to 832)	60.2 (40.7 to 82.2)	6.63 (5.78 to 7.49)	10.8 (9.49 to 12.4)	63.9 (40.6 to 90.6)
		Group B Streptococcus	427 (371 to 488)	767 (667 to 876)	79.9 (56.3 to 105.2)	4.49 (3.95 to 5.08)	8.23 (7.22 to 9.36)	84.1 (54.8 to 114.5)
		Haemophilus influenzae	1660 (1470 to 1840)	2190 (1950 to 2440)	31.7 (19.1 to 45.3)	9.38 (8.57 to 10.3)	8.53 (7.69 to 9.38)	-8.9 (-20.1 to 3.9)
		Influenza	1930 (1710 to 2130)	245 (37.9 to 749)	-87.3 (-98.1 to -61.2)	21.3 (19.5 to 23.4)	2.65 (0.41 to 8.1)	-87.5 (-98.1 to -61.5)
		Klebsiella pneumoniae	973 (865 to 1070)	1200 (1070 to 1330)	23.1 (10.9 to 35.2)	14.3 (13.1 to 15.8)	18 (16.2 to 19.7)	26.0 (10.4 to 42.2)
		Legionella spp.	1570 (1360 to 1760)	2500 (2230 to 2800)	59.7 (41.5 to 81.6)	15.9 (14.1 to 17.7)	25.9 (23.4 to 28.6)	63.4 (40.9 to 88.2)
		Mycoplasma spp.	1610 (1420 to 1800)	1580 (1420 to 1770)	-1.6 (-12.2 to 9.1)	6.28 (5.64 to 6.98)	6.31 (5.66 to 6.98)	0.6 (-12.6 to 14.7)
		Polymicrobial	47.3 (27.6 to 77.1)	75.8 (42.3 to 130)	59.6 (38.0 to 82.3)	0.71 (0.41 to 1.21)	1.16 (0.65 to 1.97)	63.3 (38.1 to 92.0)
		Pseudomonas aeruginosa	1610 (1430 to 1790)	2830 (2530 to 3150)	76.5 (58.6 to 96.0)	16.4 (14.9 to 18.1)	29.5 (26.6 to 32.4)	80.6 (56.6 to 105.1)
		Respiratory syncytial virus	581 (507 to 657)	50.1 (7.69 to 153)	-91.4 (-98.7 to -73.5)	2.92 (2.58 to 3.32)	0.25 (0.04 to 0.76)	-91.5 (-98.7 to -74.0)
		Staphylococcus aureus	1810 (1600 to 1980)	4300 (3860 to 4780)	137.7 (117.4 to 161.4)	37.9 (34.8 to 41.4)	91.9 (83.3 to 101)	143.2 (113.4 to 174.7)
		Streptococcus pneumoniae	7390 (6550 to 8080)	3950 (3530 to 4380)	-46.4 (-50.8 to -41.5)	64.2 (59 to 70.2)	35.1 (31.7 to 38.5)	-45.2 (-51.8 to -37.8)
		Other viruses	4470 (3970 to 4900)	5480 (4920 to 6070)	22.7 (11.8 to 34.3)	15.3 (14 to 16.7)	19.1 (17.2 to 21)	25.5 (10.2 to 42.1)
		Other bacterial pathogens	1630 (1390 to 1880)	1690 (1420 to 1990)	3.9 (-7.4 to 16.3)	13.6 (11.8 to 15.5)	14.4 (12.2 to 16.9)	6.5 (-8.0 to 23.2)
	70+ years	*Acinetobacter baumannii*	451 (379 to 536)	336 (286 to 396)	-25.3 (-34.6 to -15.3)	39 (32 to 45.4)	30.6 (24.3 to 35.9)	-21.3 (-30.6 to -11.9)
		Chlamydia spp.	2210 (1930 to 2480)	2170 (1900 to 2480)	-1.6 (-13.5 to 11.8)	47.4 (40.4 to 53.1)	49.1 (40.2 to 56.3)	3.6 (-8.6 to 15.8)
		Enterobacter spp.	455 (388 to 529)	1150 (999 to 1310)	153.2 (122.2 to 189.6)	37.3 (30.6 to 43.5)	99.4 (79.3 to 114)	166.8 (137.4 to 199.4)
		Escherichia coli	1310 (1150 to 1470)	2920 (2600 to 3290)	123.7 (98.8 to 152.2)	111 (94 to 124)	261 (214 to 292)	135.6 (110.6 to 160.3)
		Fungus	566 (489 to 648)	1040 (904 to 1210)	85.1 (60.2 to 112.7)	47.3 (39.7 to 54.4)	92.2 (73.8 to 107)	94.9 (72.7 to 117.9)
		Group B Streptococcus	877 (767 to 989)	1820 (1600 to 2070)	107.8 (82.0 to 136.1)	52.8 (43.9 to 59.6)	115 (94.8 to 131)	118.9 (93.4 to 146.7)
		Haemophilus influenzae	3350 (3010 to 3690)	5090 (4540 to 5720)	52.1 (36.6 to 67.8)	108 (93.2 to 118)	117 (96.3 to 131)	8.3 (-1.6 to 16.9)
		Influenza	6560 (5900 to 7170)	956 (146 to 2870)	-85.4 (-97.7 to -56.1)	415 (356 to 453)	61.4 (9.7 to 194)	-85.2 (-97.7 to -54.2)
		Klebsiella pneumoniae	2400 (2160 to 2620)	3400 (3050 to 3820)	42.2 (28.7 to 57.4)	202 (173 to 221)	303 (248 to 337)	49.8 (37.3 to 61.3)

		Legionella spp.	2520 (2220 to 2840)	4640 (4170 to 5220)	84.5 (62.5 to 109.2)	146 (125 to 164)	284 (235 to 318)	94.3 (73.5 to 115.6)
		Mycoplasma spp.	3040 (2700 to 3390)	3450 (3080 to 3890)	13.6 (0.4 to 26.9)	68.1 (58.3 to 75.4)	81.5 (67.1 to 91.7)	19.7 (8.3 to 31.1)
		Polymicrobial	121 (68.9 to 206)	224 (121 to 392)	84.4 (59.2 to 110.8)	10.4 (5.89 to 17.7)	20.3 (11 to 35.3)	94.2 (68.6 to 119.6)
		Pseudomonas aeruginosa	3740 (3320 to 4150)	7600 (6820 to 8530)	103.9 (82.3 to 126.5)	219 (188 to 242)	470 (386 to 522)	114.7 (95.0 to 133.0)
		Respiratory syncytial virus	1220 (1070 to 1380)	121 (18.4 to 362)	-90.1 (-98.5 to -69.7)	35.1 (29.7 to 39.6)	3.54 (0.55 to 11.1)	-89.9 (-98.5 to -69.0)
		Staphylococcus aureus	5240 (4720 to 5740)	14400 (13000 to 16100)	174.6 (149.1 to 204.4)	628 (541 to 684)	1820 (1500 to 2020)	189.2 (166.5 to 210.7)
		Streptococcus pneumoniae	17000 (15400 to 18500)	10500 (9440 to 11800)	-38.1 (-44.1 to -31.5)	847 (729 to 923)	552 (457 to 612)	-34.8 (-40.2 to -30.1)
		Other viruses	10200 (9210 to 11100)	14400 (13000 to 16100)	41.7 (28.2 to 57.2)	199 (172 to 217)	297 (246 to 330)	49.3 (37.4 to 60.8)
		Other bacterial pathogens	4880 (4190 to 5600)	5850 (5010 to 6890)	20.0 (6.7 to 35.5)	235 (193 to 273)	298 (233 to 352)	26.6 (13.6 to 40.5)
Norway	All Ages	*Acinetobacter baumannii*	525 (448 to 610)	191 (166 to 220)	-63.5 (-66.8 to -59.8)	35 (29.3 to 40.5)	11.1 (8.78 to 13.1)	-68.1 (-71.7 to -64.5)
		Chlamydia spp.	2480 (2240 to 2740)	1250 (1130 to 1380)	-49.8 (-54.5 to -44.5)	40.8 (35 to 45.2)	17.6 (14.4 to 20)	-57.0 (-61.5 to -52.1)
		Enterobacter spp.	500 (438 to 566)	652 (581 to 726)	30.7 (17.3 to 44.3)	31 (25.9 to 35.7)	35.2 (28.1 to 40.4)	13.6 (1.1 to 26.7)
		Escherichia coli	1370 (1240 to 1520)	1550 (1430 to 1690)	13.6 (4.1 to 23.3)	92.8 (80.4 to 103)	91.7 (74.5 to 102)	-1.2 (-11.2 to 8.4)
		Fungus	774 (693 to 872)	734 (652 to 820)	-4.9 (-15.1 to 4.8)	41.9 (35.7 to 47.7)	33.8 (26.9 to 39.1)	-19.1 (-27.8 to -9.9)
		Group B Streptococcus	1020 (924 to 1130)	1100 (998 to 1220)	8.1 (-2.6 to 19.4)	44.7 (38 to 50)	41.2 (33.4 to 46.6)	-7.7 (-18.4 to 2.9)
		Haemophilus influenzae	4060 (3790 to 4400)	3220 (2980 to 3510)	-20.6 (-25.5 to -16.1)	91.2 (80.2 to 98.3)	41.7 (34 to 46.5)	-54.4 (-58.5 to -50.8)
		Influenza	6770 (6310 to 7280)	324 (60.4 to 1020)	-95.2 (-99.1 to -85.0)	340 (297 to 366)	13.5 (2.47 to 41.8)	-96.0 (-99.3 to -87.4)
		Klebsiella pneumoniae	2670 (2490 to 2880)	1910 (1770 to 2080)	-28.3 (-32.4 to -23.9)	170 (148 to 183)	108 (88.2 to 120)	-36.7 (-42.0 to -32.3)
		Legionella spp.	2980 (2710 to 3290)	2960 (2720 to 3220)	-0.7 (-9.8 to 9.4)	121 (105 to 135)	103 (84 to 115)	-14.9 (-23.6 to -5.9)
		Mycoplasma spp.	4150 (3830 to 4520)	2810 (2580 to 3050)	-32.3 (-37.1 to -27.7)	58.4 (50.9 to 63.7)	29.9 (24.6 to 33.4)	-48.8 (-53.5 to -44.4)
		Polymicrobial	138 (83.4 to 228)	125 (69.9 to 213)	-10.1 (-20.6 to 1.8)	9.13 (5.35 to 15.3)	7.22 (3.9 to 12.6)	-21.5 (-31.6 to -11.3)
		Pseudomonas aeruginosa	3990 (3680 to 4360)	4390 (4040 to 4770)	10.2 (2.5 to 17.7)	178 (155 to 193)	167 (137 to 186)	-5.8 (-14.3 to 1.9)
		Respiratory syncytial virus	1990 (1840 to 2170)	78.1 (14.6 to 248)	-96.1 (-99.3 to -87.8)	30.3 (26.2 to 33.4)	0.82 (0.15 to 2.62)	-97.3 (-99.5 to -91.3)
		Staphylococcus aureus	5390 (5030 to 5800)	7670 (7090 to 8340)	42.4 (35.3 to 49.4)	513 (447 to 550)	637 (522 to 705)	24.0 (14.3 to 31.8)
		Streptococcus pneumoniae	21000 (19700 to 22500)	6810 (6330 to 7370)	-67.6 (-69.1 to -66.2)	717 (630 to 767)	202 (165 to 224)	-71.8 (-74.0 to -70.0)

		Other viruses	13000 (12200 to 13800)	9780 (9120 to 10500)	-24.5 (-27.9 to -21.1)	164 (145 to 176)	108 (88.2 to 119)	-34.6 (-39.8 to -30.2)
		Other bacterial pathogens	5010 (4410 to 5700)	3070 (2650 to 3550)	-38.7 (-43.3 to -33.5)	196 (164 to 225)	106 (82.1 to 125)	-46.3 (-51.5 to -41.2)
	Under 5	*Acinetobacter baumannii*	4.3 (2.71 to 6.84)	1.68 (1.04 to 2.69)	-61.1 (-65.4 to -56.7)	0.12 (0.06 to 0.2)	0.02 (0.01 to 0.03)	-83.3 (-86.6 to -80.0)
		Chlamydia spp.	50.2 (42.5 to 59.2)	28.2 (22.8 to 34.9)	-43.7 (-51.5 to -35.8)	0.15 (0.13 to 0.17)	0.02 (0.02 to 0.03)	-85.8 (-88.3 to -83.3)
		Enterobacter spp.	8.7 (6.46 to 11.4)	11.5 (8.57 to 15.4)	32.8 (12.0 to 57.6)	0.11 (0.08 to 0.16)	0.03 (0.02 to 0.04)	-74.7 (-79.2 to -69.9)
		Escherichia coli	23.5 (19.5 to 28.5)	33.5 (26.6 to 41.3)	42.4 (22.6 to 63.6)	0.28 (0.25 to 0.32)	0.07 (0.06 to 0.08)	-75.4 (-79.8 to -70.9)
		Fungus	4.12 (2.88 to 5.76)	4.58 (3.22 to 6.45)	11.5 (-6.8 to 31.3)	0.03 (0.02 to 0.03)	0 (0 to 0.01)	-79.8 (-84.5 to -74.7)
		Group B Streptococcus	23.4 (19.5 to 28.1)	25.9 (20.7 to 32.3)	10.4 (-6.3 to 26.4)	0.24 (0.22 to 0.28)	0.05 (0.04 to 0.06)	-78.2 (-82.0 to -74.1)
		Haemophilus influenzae	207 (174 to 247)	161 (130 to 198)	-22.4 (-31.9 to -12.8)	0.63 (0.57 to 0.7)	0.05 (0.04 to 0.06)	-92.3 (-93.9 to -90.6)
		Influenza	163 (137 to 193)	6.77 (1.16 to 21.5)	-95.9 (-99.2 to -87.0)	0.91 (0.83 to 0.99)	0.01 (0 to 0.02)	-99.3 (-99.9 to -97.7)
		Klebsiella pneumoniae	80.7 (68.2 to 96)	46.8 (38.1 to 57.2)	-42.0 (-48.2 to -35.7)	0.66 (0.6 to 0.72)	0.09 (0.07 to 0.1)	-86.8 (-89.1 to -84.5)
		Legionella spp.	13.9 (10.1 to 18.5)	14 (9.56 to 19.5)	1.1 (-13.5 to 18.2)	0.15 (0.12 to 0.19)	0.04 (0.03 to 0.05)	-76.2 (-80.3 to -71.4)
		Mycoplasma spp.	111 (92.6 to 134)	138 (112 to 172)	23.9 (9.5 to 40.1)	0.22 (0.2 to 0.25)	0.05 (0.04 to 0.06)	-78.2 (-82.3 to -73.6)
		Polymicrobial	6.19 (3.3 to 10.4)	3.7 (2 to 6.13)	-39.9 (-48.5 to -29.6)	0.2 (0.1 to 0.34)	0.05 (0.02 to 0.08)	-76.2 (-80.5 to -71.2)
		Pseudomonas aeruginosa	72.3 (60.9 to 86.7)	117 (95.1 to 145)	62.1 (43.2 to 83.6)	0.49 (0.44 to 0.54)	0.14 (0.12 to 0.16)	-71.8 (-76.7 to -67.0)
		Respiratory syncytial virus	578 (500 to 677)	27.4 (4.77 to 86.4)	-95.3 (-99.1 to -85.3)	1.69 (1.55 to 1.82)	0.01 (0 to 0.05)	-99.2 (-99.8 to -97.3)
		Staphylococcus aureus	73.9 (62.5 to 87.3)	115 (93.3 to 141)	55.0 (41.6 to 68.3)	0.83 (0.76 to 0.9)	0.23 (0.19 to 0.27)	-72.3 (-77.3 to -67.3)
		Streptococcus pneumoniae	1010 (861 to 1200)	219 (179 to 270)	-78.3 (-80.2 to -76.6)	3.91 (3.59 to 4.24)	0.15 (0.12 to 0.17)	-96.2 (-96.9 to -95.5)
		Other viruses	1110 (948 to 1310)	660 (538 to 810)	-40.9 (-45.6 to -36.4)	1.76 (1.61 to 1.91)	0.22 (0.18 to 0.26)	-87.7 (-89.8 to -85.4)
		Other bacterial pathogens	23.8 (18.3 to 30.2)	18.2 (13.5 to 23.9)	-23.6 (-34.3 to -11.8)	0.14 (0.1 to 0.18)	0.02 (0.02 to 0.03)	-83.7 (-86.8 to -80.3)
	5-14 years	*Acinetobacter baumannii*	6.17 (4.49 to 8.39)	3.81 (2.83 to 5.13)	-38.1 (-44.8 to -30.8)	0.02 (0.01 to 0.02)	0 (0 to 0)	-87.7 (-89.3 to -85.7)
		Chlamydia spp.	41.4 (30.5 to 54.5)	36.8 (27.5 to 48.4)	-11.0 (-19.3 to -1.8)	0.03 (0.02 to 0.03)	0 (0 to 0.01)	-82.4 (-84.6 to -79.8)
		Enterobacter spp.	6.11 (4.43 to 8.28)	13.9 (10.2 to 18.7)	129.0 (104.3 to 154.8)	0.01 (0.01 to 0.02)	0.01 (0.01 to 0.01)	-54.7 (-60.9 to -47.4)
		Escherichia coli	12.8 (9.44 to 16.9)	26.7 (20.2 to 34.9)	108.6 (90.1 to 128.8)	0.03 (0.03 to 0.03)	0.01 (0.01 to 0.01)	-58.7 (-63.9 to -53.1)

		Fungus	15.4 (11.4 to 20.6)	25.1 (18.8 to 33.5)	63.6 (45.8 to 81.5)	0.04 (0.03 to 0.04)	0.01 (0.01 to 0.01)	-67.6 (-71.9 to -62.8)
		Group B Streptococcus	17.8 (13 to 23.4)	34 (25.5 to 44.9)	91.5 (69.4 to 113.1)	0.03 (0.03 to 0.03)	0.01 (0.01 to 0.01)	-62.1 (-67.4 to -56.4)
		Haemophilus influenzae	80.3 (60.9 to 105)	102 (76.8 to 134)	27.4 (18.8 to 36.1)	0.07 (0.07 to 0.08)	0.01 (0.01 to 0.01)	-83.1 (-84.9 to -81.1)
		Influenza	109 (82 to 140)	8.55 (1.47 to 27.9)	-92.2 (-98.6 to -75.2)	0.2 (0.18 to 0.21)	0 (0 to 0.01)	-98.5 (-99.7 to -95.3)
		Klebsiella pneumoniae	37.4 (28.2 to 48.5)	40.8 (31 to 52.7)	9.0 (2.5 to 15.6)	0.09 (0.08 to 0.1)	0.02 (0.02 to 0.02)	-78.4 (-80.6 to -75.9)
		Legionella spp.	52.5 (39.7 to 69.1)	91.1 (69.5 to 118)	73.8 (56.4 to 90.8)	0.09 (0.08 to 0.1)	0.03 (0.03 to 0.03)	-65.6 (-69.8 to -60.5)
		Mycoplasma spp.	170 (127 to 220)	208 (158 to 271)	22.1 (13.2 to 30.5)	0.11 (0.1 to 0.12)	0.03 (0.02 to 0.03)	-75.8 (-78.5 to -72.8)
		Polymicrobial	1.68 (0.95 to 2.76)	2.64 (1.39 to 4.41)	56.9 (37.1 to 78.3)	0 (0 to 0.01)	0 (0 to 0)	-69.0 (-73.4 to -63.9)
		Pseudomonas aeruginosa	53.7 (40.4 to 69.4)	109 (82.7 to 141)	103.6 (88.5 to 117.2)	0.09 (0.08 to 0.1)	0.04 (0.03 to 0.04)	-59.7 (-63.9 to -55.1)
		Respiratory syncytial virus	42.6 (32.1 to 55.8)	2.7 (0.46 to 8.82)	-93.7 (-98.9 to -80.3)	0.04 (0.03 to 0.04)	0 (0 to 0)	-98.8 (-99.8 to -96.2)
		Staphylococcus aureus	70 (53.1 to 90.8)	171 (130 to 220)	144.3 (132.4 to 156.1)	0.24 (0.22 to 0.26)	0.12 (0.11 to 0.13)	-51.7 (-56.2 to -46.3)
		Streptococcus pneumoniae	585 (443 to 756)	290 (222 to 373)	-50.4 (-52.7 to -48.2)	0.84 (0.78 to 0.89)	0.08 (0.08 to 0.09)	-90.2 (-91.1 to -89.1)
		Other viruses	391 (296 to 504)	444 (341 to 574)	13.8 (8.4 to 18.9)	0.22 (0.2 to 0.23)	0.05 (0.05 to 0.05)	-77.5 (-79.6 to -75.0)
		Other bacterial pathogens	53.4 (39.3 to 71.2)	58.1 (43.2 to 77.2)	8.8 (-0.5 to 18.6)	0.07 (0.06 to 0.08)	0.02 (0.01 to 0.02)	-78.4 (-81.0 to -75.6)
	15-49 years	*Acinetobacter baumannii*	23.9 (19.1 to 29.2)	11.3 (9.13 to 13.8)	-52.7 (-57.7 to -47.2)	0.19 (0.16 to 0.22)	0.03 (0.03 to 0.04)	-82.0 (-84.1 to -79.8)
		Chlamydia spp.	160 (136 to 189)	109 (91.1 to 129)	-32.0 (-38.4 to -25.0)	0.31 (0.28 to 0.34)	0.08 (0.07 to 0.09)	-74.1 (-77.0 to -71.0)
		Enterobacter spp.	23.6 (19.3 to 28.2)	41.3 (33.7 to 50.2)	75.1 (56.8 to 96.5)	0.17 (0.15 to 0.2)	0.12 (0.1 to 0.13)	-33.5 (-41.0 to -25.3)
		Escherichia coli	49.7 (41.9 to 58.2)	79.2 (66.9 to 92.6)	59.4 (45.2 to 74.4)	0.38 (0.34 to 0.42)	0.23 (0.21 to 0.25)	-39.4 (-45.5 to -32.9)
		Fungus	59.6 (50.2 to 70)	74.4 (62.5 to 88.7)	25.0 (12.2 to 39.1)	0.45 (0.41 to 0.5)	0.21 (0.19 to 0.24)	-52.5 (-57.8 to -46.7)
		Group B Streptococcus	68.9 (57.8 to 82.3)	101 (84.3 to 120)	46.4 (30.7 to 63.0)	0.38 (0.34 to 0.42)	0.21 (0.19 to 0.23)	-44.3 (-50.8 to -37.3)
		Haemophilus influenzae	311 (268 to 358)	303 (256 to 354)	-2.6 (-9.8 to 4.2)	0.91 (0.86 to 0.97)	0.22 (0.21 to 0.24)	-75.2 (-77.3 to -72.8)
		Influenza	421 (366 to 481)	25.3 (4.65 to 81)	-94.0 (-98.9 to -81.1)	2.41 (2.29 to 2.53)	0.05 (0.01 to 0.17)	-97.8 (-99.6 to -93.1)
		Klebsiella pneumoniae	145 (126 to 166)	121 (102 to 140)	-16.7 (-21.4 to -11.7)	1.1 (1.05 to 1.17)	0.35 (0.33 to 0.37)	-68.3 (-70.7 to -65.8)
		Legionella spp.	203 (173 to 236)	270 (230 to 313)	32.9 (19.8 to 46.4)	1.07 (0.98 to 1.16)	0.54 (0.5 to 0.58)	-49.5 (-54.7 to -44.2)

		Mycoplasma spp.	659 (568 to 755)	615 (522 to 717)	-6.7 (-13.6 to 0.1)	1.33 (1.25 to 1.42)	0.47 (0.44 to 0.51)	-64.5 (-67.5 to -61.4)
		Polymicrobial	6.51 (3.73 to 10.6)	7.85 (4.2 to 13.3)	20.0 (5.0 to 35.6)	0.05 (0.03 to 0.08)	0.02 (0.01 to 0.04)	-54.4 (-60.3 to -47.8)
		Pseudomonas aeruginosa	208 (178 to 239)	323 (276 to 376)	55.6 (44.1 to 67.2)	1.1 (1.02 to 1.18)	0.65 (0.61 to 0.7)	-40.8 (-45.9 to -35.6)
		Respiratory syncytial virus	165 (142 to 192)	7.98 (1.44 to 25.6)	-95.2 (-99.1 to -85.0)	0.43 (0.4 to 0.47)	0.01 (0 to 0.02)	-98.2 (-99.7 to -94.4)
		Staphylococcus aureus	271 (236 to 312)	506 (435 to 587)	86.8 (78.0 to 95.6)	2.94 (2.81 to 3.09)	2.08 (1.97 to 2.2)	-29.0 (-33.8 to -23.9)
		Streptococcus pneumoniae	2260 (1980 to 2580)	859 (736 to 994)	-62.1 (-63.8 to -60.4)	10.2 (9.84 to 10.7)	1.47 (1.39 to 1.56)	-85.6 (-86.5 to -84.5)
		Other viruses	1510 (1320 to 1720)	1320 (1120 to 1530)	-13.0 (-17.2 to -9.1)	2.68 (2.56 to 2.8)	0.89 (0.84 to 0.94)	-66.9 (-69.0 to -64.7)
		Other bacterial pathogens	207 (171 to 244)	172 (141 to 209)	-16.8 (-23.9 to -9.0)	0.89 (0.78 to 1)	0.28 (0.24 to 0.33)	-68.3 (-71.5 to -65.0)
	50-69 years	*Acinetobacter baumannii*	120 (102 to 139)	40.8 (34.5 to 47.6)	-66.0 (-69.4 to -62.4)	1.83 (1.59 to 2.11)	0.41 (0.36 to 0.47)	-77.6 (-80.1 to -74.9)
		Chlamydia spp.	477 (421 to 537)	218 (190 to 247)	-54.2 (-58.8 to -49.0)	1.8 (1.62 to 1.99)	0.54 (0.49 to 0.6)	-69.8 (-72.9 to -66.3)
		Enterobacter spp.	115 (99.3 to 132)	140 (120 to 160)	21.3 (8.8 to 35.1)	1.66 (1.47 to 1.87)	1.32 (1.18 to 1.47)	-20.1 (-29.2 to -10.5)
		Escherichia coli	268 (238 to 300)	281 (249 to 311)	5.0 (-4.3 to 15.0)	3.99 (3.66 to 4.35)	2.76 (2.52 to 2.96)	-30.8 (-37.9 to -23.1)
		Fungus	255 (222 to 290)	223 (193 to 254)	-12.6 (-22.0 to -2.8)	3.75 (3.35 to 4.15)	2.15 (1.91 to 2.41)	-42.5 (-48.8 to -35.6)
		Group B Streptococcus	238 (208 to 271)	235 (206 to 266)	-1.1 (-12.0 to 10.5)	2.52 (2.28 to 2.8)	1.64 (1.47 to 1.82)	-34.9 (-42.4 to -26.6)
		Haemophilus influenzae	916 (827 to 998)	672 (597 to 742)	-26.7 (-31.9 to -21.8)	5.21 (4.9 to 5.54)	1.68 (1.54 to 1.81)	-67.7 (-70.3 to -64.8)
		Influenza	1070 (964 to 1170)	47.4 (8.72 to 151)	-95.6 (-99.2 to -86.0)	11.9 (11.2 to 12.6)	0.33 (0.06 to 1.04)	-97.2 (-99.5 to -91.3)
		Klebsiella pneumoniae	547 (498 to 597)	369 (329 to 407)	-32.4 (-37.1 to -27.9)	8.1 (7.63 to 8.59)	3.6 (3.32 to 3.87)	-55.5 (-59.0 to -51.9)
		Legionella spp.	842 (749 to 938)	768 (682 to 858)	-8.6 (-17.2 to 1.3)	8.59 (7.86 to 9.32)	5.16 (4.74 to 5.57)	-39.8 (-45.8 to -33.2)
		Mycoplasma spp.	890 (797 to 985)	489 (433 to 544)	-45.0 (-49.4 to -40.6)	3.5 (3.24 to 3.78)	1.27 (1.16 to 1.38)	-63.8 (-67.0 to -60.4)
		Polymicrobial	27.2 (16.1 to 44.9)	23.2 (13 to 39.8)	-15.2 (-25.5 to -4.3)	0.41 (0.25 to 0.68)	0.23 (0.13 to 0.38)	-44.2 (-51.6 to -36.3)
		Pseudomonas aeruginosa	868 (780 to 958)	870 (775 to 963)	0.3 (-7.0 to 8.4)	8.92 (8.32 to 9.57)	5.89 (5.47 to 6.34)	-33.9 (-39.6 to -27.8)
		Respiratory syncytial virus	310 (274 to 346)	9.85 (1.79 to 31.4)	-96.8 (-99.4 to -90.1)	1.57 (1.42 to 1.73)	0.03 (0.01 to 0.1)	-98.0 (-99.6 to -93.6)
		Staphylococcus aureus	996 (905 to 1080)	1310 (1170 to 1440)	31.7 (24.4 to 39.4)	21 (19.9 to 22.1)	18.2 (16.9 to 19.5)	-13.2 (-19.4 to -6.2)
		Streptococcus pneumoniae	4110 (3740 to 4450)	1240 (1100 to 1370)	-69.7 (-71.5 to -68.0)	36 (34.3 to 37.8)	7.17 (6.66 to 7.68)	-80.1 (-81.5 to -78.5)

		Other viruses	2400 (2190 to 2610)	1700 (1510 to 1870)	-29.4 (-33.5 to -24.8)	8.27 (7.85 to 8.69)	3.85 (3.57 to 4.11)	-53.5 (-56.8 to -49.8)
		Other bacterial pathogens	920 (796 to 1060)	523 (441 to 609)	-43.1 (-47.9 to -38.0)	7.7 (6.77 to 8.68)	2.89 (2.49 to 3.33)	-62.5 (-66.2 to -58.5)
	70+ years	*Acinetobacter baumannii*	371 (314 to 436)	134 (114 to 156)	-63.9 (-67.4 to -59.8)	32.8 (27.2 to 38.1)	10.7 (8.34 to 12.6)	-67.4 (-71.1 to -63.8)
		Chlamydia spp.	1750 (1570 to 1960)	853 (762 to 978)	-51.3 (-56.2 to -45.9)	38.6 (32.8 to 42.7)	16.9 (13.8 to 19.4)	-56.1 (-60.8 to -51.1)
		Enterobacter spp.	347 (298 to 397)	446 (390 to 503)	29.0 (15.6 to 42.9)	29.1 (24.1 to 33.5)	33.8 (26.7 to 38.8)	16.2 (3.4 to 29.7)
		Escherichia coli	1010 (911 to 1140)	1130 (1020 to 1250)	11.7 (1.6 to 22.1)	88.1 (75.7 to 97.7)	88.7 (71.6 to 98.8)	0.6 (-9.8 to 10.3)
		Fungus	439 (387 to 503)	407 (355 to 463)	-7.1 (-17.3 to 3.4)	37.6 (31.5 to 43.1)	31.5 (24.8 to 36.5)	-16.3 (-25.4 to -6.8)
		Group B Streptococcus	674 (602 to 751)	707 (628 to 794)	5.2 (-5.6 to 17.4)	41.5 (34.9 to 46.5)	39.3 (31.4 to 44.6)	-5.3 (-16.0 to 5.6)
		Haemophilus influenzae	2550 (2340 to 2790)	1990 (1800 to 2200)	-22.0 (-27.6 to -16.6)	84.4 (73.6 to 91.3)	39.7 (32.1 to 44.4)	-53.0 (-57.2 to -49.3)
		Influenza	5010 (4620 to 5450)	236 (44.1 to 735)	-95.3 (-99.1 to -85.2)	325 (281 to 350)	13.1 (2.4 to 40.6)	-96.0 (-99.3 to -87.3)
		Klebsiella pneumoniae	1860 (1720 to 2030)	1340 (1210 to 1480)	-28.1 (-32.8 to -23.1)	161 (139 to 173)	104 (84.4 to 116)	-35.3 (-40.7 to -30.8)
		Legionella spp.	1870 (1680 to 2080)	1810 (1640 to 2020)	-2.8 (-12.9 to 7.6)	111 (94.8 to 124)	97.2 (78.5 to 109)	-12.5 (-21.6 to -3.1)
		Mycoplasma spp.	2320 (2100 to 2570)	1360 (1230 to 1520)	-41.5 (-46.5 to -36.8)	53.3 (45.9 to 58.3)	28.1 (22.9 to 31.6)	-47.3 (-52.1 to -42.6)
		Polymicrobial	96.3 (56.2 to 163)	87.2 (47.7 to 153)	-9.8 (-20.9 to 2.5)	8.47 (4.89 to 14.4)	6.91 (3.72 to 12.1)	-18.8 (-28.8 to -8.5)
		Pseudomonas aeruginosa	2790 (2550 to 3090)	2970 (2690 to 3300)	6.7 (-1.5 to 15.0)	167 (144 to 182)	161 (130 to 179)	-3.9 (-12.5 to 4.0)
		Respiratory syncytial virus	898 (803 to 1010)	30.2 (5.61 to 93.8)	-96.6 (-99.4 to -89.4)	26.5 (22.7 to 29.6)	0.76 (0.14 to 2.44)	-97.1 (-99.5 to -90.7)
		Staphylococcus aureus	3980 (3680 to 4340)	5570 (5060 to 6150)	40.1 (32.1 to 48.4)	488 (423 to 524)	616 (501 to 684)	26.2 (16.4 to 34.1)
		Streptococcus pneumoniae	13000 (12100 to 14200)	4200 (3810 to 4630)	-67.8 (-69.7 to -65.9)	666 (580 to 715)	193 (157 to 215)	-71.0 (-73.3 to -69.1)
		Other viruses	7540 (6970 to 8240)	5660 (5150 to 6240)	-24.9 (-29.3 to -20.4)	151 (132 to 163)	103 (83.4 to 114)	-32.3 (-37.6 to -27.8)
		Other bacterial pathogens	3800 (3330 to 4390)	2300 (1980 to 2690)	-39.5 (-44.3 to -34.1)	188 (156 to 216)	102 (79.2 to 122)	-45.5 (-50.8 to -40.3)
Portugal	All Ages	*Acinetobacter baumannii*	1300 (1130 to 1490)	977 (847 to 1130)	-24.9 (-32.8 to -15.6)	60.3 (52 to 68.8)	72.7 (57.9 to 85)	20.5 (2.8 to 37.1)
		Chlamydia spp.	5020 (4540 to 5500)	3660 (3340 to 4030)	-26.9 (-34.2 to -18.4)	56.3 (50.9 to 61.8)	67.6 (55.5 to 76.1)	20.1 (2.4 to 35.7)
		Enterobacter spp.	765 (670 to 869)	1510 (1350 to 1680)	97.6 (74.9 to 122.1)	32.7 (28.4 to 37.2)	105 (84.4 to 120)	221.8 (168.5 to 272.4)
		Escherichia coli	2250 (2020 to 2520)	3440 (3160 to 3760)	53.3 (37.1 to 72.6)	107 (95.2 to 117)	260 (212 to 291)	144.2 (105.4 to 179.0)
		Fungus	1510 (1330 to 1700)	1900 (1730 to 2110)	26.6 (12.3 to 43.2)	55 (48.9 to 61.3)	116 (95.3 to 132)	111.4 (79.7 to 139.3)

		Group B Streptococcus	1740 (1560 to 1930)	2720 (2490 to 2970)	56.2 (37.8 to 76.0)	51.6 (46.4 to 57.4)	134 (110 to 150)	159.4 (121.2 to 197.5)
		Haemophilus influenzae	6870 (6380 to 7400)	6840 (6330 to 7400)	-0.4 (-8.8 to 9.0)	102 (94.2 to 109)	118 (97.4 to 131)	15.9 (0.8 to 29.2)
		Influenza	10400 (9570 to 11200)	1180 (131 to 3690)	-88.6 (-98.7 to -64.5)	356 (328 to 382)	66.1 (7.96 to 213)	-81.4 (-97.8 to -39.1)
		Klebsiella pneumoniae	4710 (4360 to 5090)	5830 (5370 to 6320)	24.0 (12.7 to 36.4)	204 (188 to 218)	424 (347 to 471)	107.9 (79.0 to 132.7)
		Legionella spp.	4070 (3650 to 4520)	6790 (6290 to 7380)	67.2 (48.8 to 87.3)	112 (100 to 123)	309 (257 to 345)	176.2 (134.8 to 214.1)
		Mycoplasma spp.	7780 (7210 to 8470)	6980 (6520 to 7500)	-10.2 (-17.9 to -1.5)	70.6 (65.1 to 75.8)	110 (91 to 123)	55.8 (33.7 to 74.3)
		Polymicrobial	272 (175 to 422)	326 (184 to 551)	18.9 (0.7 to 39.0)	12.7 (8.2 to 19.9)	24.4 (13.7 to 42.4)	89.9 (55.6 to 124.6)
		Pseudomonas aeruginosa	5370 (4920 to 5870)	11500 (10600 to 12400)	114.0 (94.3 to 138.4)	164 (149 to 177)	571 (468 to 635)	248.7 (198.2 to 291.0)
		Respiratory syncytial virus	3700 (3380 to 4050)	310 (34.5 to 965)	-91.6 (-99.0 to -73.7)	35.7 (33.1 to 38.3)	5.55 (0.66 to 18.4)	-84.4 (-98.1 to -48.9)
		Staphylococcus aureus	7750 (7160 to 8410)	17200 (15900 to 18800)	122.5 (104.1 to 144.6)	511 (471 to 547)	1840 (1500 to 2040)	259.2 (210.3 to 298.7)
		Streptococcus pneumoniae	38500 (36100 to 41100)	25500 (23800 to 27600)	-33.5 (-38.4 to -27.8)	851 (793 to 904)	1010 (833 to 1120)	19.1 (3.1 to 31.5)
		Other viruses	20200 (18900 to 21600)	25600 (23900 to 27700)	27.0 (17.0 to 38.3)	163 (152 to 172)	387 (317 to 429)	138.1 (104.2 to 163.9)
		Other bacterial pathogens	8250 (7290 to 9310)	8570 (7490 to 9770)	4.0 (-5.8 to 15.8)	228 (200 to 256)	375 (298 to 440)	64.5 (41.7 to 85.1)
	Under 5	*Acinetobacter baumannii*	18.7 (12.7 to 27.3)	3.04 (2 to 4.65)	-83.7 (-86.6 to -80.6)	0.59 (0.36 to 0.96)	0.05 (0.03 to 0.1)	-91.1 (-93.3 to -88.6)
		Chlamydia spp.	241 (204 to 284)	41.7 (34.1 to 50.7)	-82.6 (-85.9 to -79.1)	1.15 (1.02 to 1.29)	0.08 (0.06 to 0.09)	-93.3 (-94.7 to -91.8)
		Enterobacter spp.	31 (23.5 to 40.4)	13.3 (9.9 to 17.6)	-57.0 (-65.5 to -47.5)	0.6 (0.45 to 0.79)	0.09 (0.06 to 0.12)	-85.4 (-88.6 to -81.9)
		Escherichia coli	96.3 (79.9 to 116)	37.1 (30 to 45.3)	-61.3 (-69.0 to -52.7)	1.82 (1.59 to 2.1)	0.23 (0.18 to 0.27)	-87.4 (-90.0 to -84.5)
		Fungus	18.1 (12.5 to 25.2)	5.85 (4.08 to 8.21)	-67.5 (-74.3 to -59.8)	0.26 (0.18 to 0.35)	0.03 (0.02 to 0.04)	-90.3 (-92.6 to -87.7)
		Group B Streptococcus	88.7 (73.1 to 107)	32.4 (25.8 to 39.9)	-63.4 (-70.2 to -55.7)	1.36 (1.2 to 1.54)	0.17 (0.14 to 0.2)	-87.4 (-90.0 to -84.7)
		Haemophilus influenzae	787 (660 to 921)	173 (140 to 212)	-78.0 (-82.3 to -73.3)	4.84 (4.33 to 5.36)	0.2 (0.16 to 0.24)	-95.9 (-96.8 to -94.9)
		Influenza	642 (544 to 745)	12.2 (1.43 to 37.1)	-98.1 (-99.8 to -94.0)	7.52 (6.8 to 8.25)	0.04 (0 to 0.15)	-99.4 (-99.9 to -98.0)
		Klebsiella pneumoniae	348 (293 to 405)	69.9 (56.9 to 85.3)	-79.8 (-83.4 to -76.0)	5.59 (5.06 to 6.15)	0.38 (0.31 to 0.46)	-93.2 (-94.5 to -91.7)
		Legionella spp.	41.8 (30 to 58.3)	16.3 (11.2 to 23)	-61.0 (-68.2 to -52.8)	0.66 (0.51 to 0.84)	0.1 (0.07 to 0.13)	-85.1 (-88.1 to -81.4)
		Mycoplasma spp.	438 (370 to 517)	190 (155 to 233)	-56.4 (-64.5 to -47.2)	1.84 (1.65 to 2.05)	0.24 (0.19 to 0.29)	-87.1 (-89.9 to -83.9)

		Polymicrobial	17.9 (10.5 to 28.7)	4.83 (2.63 to 8)	-73.1 (-77.7 to -67.8)	0.69 (0.38 to 1.12)	0.11 (0.05 to 0.19)	-84.4 (-88.4 to -80.1)
		Pseudomonas aeruginosa	239 (200 to 281)	153 (125 to 184)	-35.8 (-47.4 to -23.0)	2.91 (2.6 to 3.26)	0.56 (0.45 to 0.66)	-80.8 (-84.9 to -76.4)
		Respiratory syncytial virus	1790 (1530 to 2050)	66.6 (7.81 to 204)	-96.3 (-99.6 to -88.5)	10.2 (9.3 to 11.1)	0.12 (0.01 to 0.38)	-98.9 (-99.9 to -96.2)
		Staphylococcus aureus	275 (235 to 317)	127 (104 to 155)	-53.5 (-61.0 to -45.5)	6.2 (5.62 to 6.81)	0.88 (0.72 to 1.06)	-85.7 (-88.7 to -82.7)
		Streptococcus pneumoniae	4110 (3500 to 4750)	420 (346 to 513)	-89.8 (-91.4 to -88.0)	36.1 (32.8 to 39.2)	1.13 (0.9 to 1.36)	-96.9 (-97.5 to -96.2)
		Other viruses	3620 (3090 to 4180)	897 (737 to 1090)	-75.2 (-79.2 to -71.1)	12.7 (11.6 to 13.9)	1 (0.8 to 1.2)	-92.1 (-93.8 to -90.4)
		Other bacterial pathogens	104 (80.2 to 132)	24.9 (18.7 to 32.7)	-76.0 (-80.7 to -70.6)	1.06 (0.85 to 1.31)	0.09 (0.06 to 0.11)	-91.8 (-93.6 to -89.8)
	5-14 years	*Acinetobacter baumannii*	33 (24.6 to 43.2)	7.13 (5.26 to 9.57)	-78.4 (-81.0 to -75.8)	0.26 (0.21 to 0.3)	0.02 (0.02 to 0.03)	-90.4 (-92.1 to -88.3)
		Chlamydia spp.	176 (135 to 228)	39.8 (29.9 to 51.9)	-77.4 (-79.9 to -74.6)	0.34 (0.3 to 0.39)	0.03 (0.03 to 0.04)	-90.0 (-91.7 to -87.7)
		Enterobacter spp.	19.6 (14.6 to 25.9)	11.7 (8.49 to 15.6)	-40.4 (-48.1 to -32.5)	0.14 (0.12 to 0.17)	0.04 (0.03 to 0.05)	-73.6 (-78.6 to -67.1)
		Escherichia coli	46.7 (35.9 to 60.5)	20.8 (15.6 to 27.3)	-55.4 (-60.7 to -49.6)	0.35 (0.31 to 0.41)	0.07 (0.06 to 0.08)	-80.2 (-83.7 to -76.3)
		Fungus	56.2 (42.6 to 72.7)	23.7 (17.9 to 31.5)	-57.7 (-62.6 to -52.3)	0.42 (0.36 to 0.49)	0.08 (0.07 to 0.09)	-81.2 (-84.4 to -77.3)
		Group B Streptococcus	61.1 (45.7 to 79)	30.6 (22.6 to 39.7)	-49.9 (-56.0 to -42.3)	0.33 (0.28 to 0.38)	0.07 (0.06 to 0.08)	-77.8 (-82.0 to -73.3)
		Haemophilus influenzae	258 (201 to 327)	83 (62.1 to 109)	-67.8 (-71.2 to -64.3)	0.74 (0.66 to 0.83)	0.07 (0.06 to 0.08)	-90.5 (-92.1 to -88.6)
		Influenza	359 (281 to 455)	12 (1.27 to 37.6)	-96.7 (-99.6 to -89.5)	2.03 (1.82 to 2.27)	0.03 (0 to 0.1)	-98.5 (-99.8 to -95.2)
		Klebsiella pneumoniae	137 (107 to 175)	48 (36.3 to 62.3)	-64.9 (-68.3 to -60.9)	1.03 (0.92 to 1.16)	0.16 (0.14 to 0.18)	-84.4 (-87.1 to -81.3)
		Legionella spp.	142 (110 to 186)	75.3 (57.2 to 97.4)	-46.9 (-53.5 to -39.8)	0.74 (0.64 to 0.84)	0.17 (0.15 to 0.2)	-76.4 (-80.7 to -71.3)
		Mycoplasma spp.	562 (432 to 716)	192 (146 to 248)	-65.8 (-69.3 to -62.1)	1.12 (0.99 to 1.26)	0.17 (0.14 to 0.2)	-84.9 (-87.5 to -81.9)
		Polymicrobial	7.14 (4.34 to 11.3)	2.56 (1.4 to 4.24)	-64.5 (-70.3 to -59.0)	0.06 (0.03 to 0.09)	0.01 (0 to 0.01)	-84.3 (-87.3 to -80.7)
		Pseudomonas aeruginosa	152 (117 to 195)	99.5 (75 to 129)	-34.4 (-41.9 to -27.1)	0.79 (0.71 to 0.89)	0.23 (0.2 to 0.26)	-70.9 (-75.8 to -64.8)
		Respiratory syncytial virus	112 (87.6 to 144)	4.74 (0.51 to 14.8)	-95.8 (-99.5 to -87.0)	0.29 (0.26 to 0.33)	0.01 (0 to 0.02)	-98.1 (-99.8 to -93.9)
		Staphylococcus aureus	221 (172 to 279)	141 (107 to 182)	-36.2 (-42.2 to -29.5)	2.36 (2.12 to 2.63)	0.67 (0.58 to 0.77)	-71.7 (-76.3 to -66.3)
		Streptococcus pneumoniae	1990 (1560 to 2510)	448 (338 to 579)	-77.5 (-79.6 to -75.3)	8.87 (8.01 to 9.86)	0.88 (0.76 to 1.01)	-90.0 (-91.6 to -88.1)
		Other viruses	1060 (828 to 1340)	480 (365 to 620)	-54.8 (-58.8 to -50.1)	1.86 (1.67 to 2.07)	0.37 (0.32 to 0.43)	-79.9 (-83.2 to -76.1)

		Other bacterial pathogens	200 (153 to 258)	57.8 (43.1 to 76.5)	-71.1 (-74.2 to -67.8)	0.85 (0.73 to 0.97)	0.11 (0.09 to 0.13)	-87.1 (-89.3 to -84.5)
	15-49 years	*Acinetobacter baumannii*	122 (101 to 145)	43.7 (35.8 to 51.9)	-64.2 (-68.4 to -59.9)	1.84 (1.58 to 2.13)	0.49 (0.41 to 0.58)	-73.4 (-77.3 to -69.1)
		Chlamydia spp.	652 (572 to 742)	244 (212 to 279)	-62.5 (-66.5 to -58.1)	2.44 (2.18 to 2.73)	0.68 (0.6 to 0.77)	-72.2 (-75.9 to -68.0)
		Enterobacter spp.	72.7 (61.4 to 85.3)	71.6 (60.3 to 84.4)	-1.4 (-14.1 to 11.5)	1.04 (0.9 to 1.2)	0.76 (0.64 to 0.89)	-26.8 (-37.6 to -14.8)
		Escherichia coli	173 (150 to 196)	128 (111 to 146)	-26.2 (-34.5 to -17.4)	2.55 (2.28 to 2.83)	1.4 (1.24 to 1.57)	-45.2 (-52.9 to -36.5)
		Fungus	208 (178 to 240)	146 (125 to 167)	-30.0 (-38.2 to -20.8)	3.03 (2.66 to 3.42)	1.57 (1.36 to 1.79)	-48.0 (-55.3 to -39.7)
		Group B Streptococcus	226 (197 to 264)	187 (162 to 216)	-17.0 (-26.5 to -7.0)	2.37 (2.1 to 2.7)	1.46 (1.29 to 1.66)	-38.4 (-47.4 to -27.8)
		Haemophilus influenzae	955 (849 to 1060)	509 (445 to 577)	-46.7 (-51.4 to -41.1)	5.38 (4.91 to 5.86)	1.42 (1.27 to 1.58)	-73.6 (-76.9 to -69.9)
		Influenza	1330 (1190 to 1480)	73.4 (8.52 to 227)	-94.5 (-99.4 to -82.6)	14.6 (13.5 to 15.9)	0.6 (0.07 to 2)	-95.9 (-99.5 to -86.2)
		Klebsiella pneumoniae	507 (453 to 565)	295 (257 to 334)	-41.9 (-47.1 to -36.2)	7.44 (6.83 to 8.09)	3.2 (2.89 to 3.6)	-56.8 (-62.1 to -50.8)
		Legionella spp.	526 (460 to 600)	462 (403 to 524)	-12.0 (-22.4 to -1.2)	5.31 (4.73 to 5.91)	3.46 (3.1 to 3.89)	-34.7 (-44.1 to -24.1)
		Mycoplasma spp.	2080 (1860 to 2330)	1180 (1030 to 1330)	-43.4 (-49.1 to -37.5)	8.11 (7.39 to 8.9)	3.4 (3.05 to 3.78)	-58.0 (-63.5 to -51.9)
		Polymicrobial	26.5 (16.5 to 41.5)	15.7 (8.82 to 26.4)	-41.2 (-49.6 to -32.2)	0.4 (0.25 to 0.62)	0.17 (0.1 to 0.29)	-56.4 (-63.4 to -48.2)
		Pseudomonas aeruginosa	563 (500 to 635)	610 (535 to 690)	8.6 (-2.1 to 19.9)	5.73 (5.2 to 6.27)	4.61 (4.13 to 5.16)	-19.4 (-29.2 to -8.1)
		Respiratory syncytial virus	415 (365 to 467)	28.9 (3.3 to 90)	-93.0 (-99.2 to -78.1)	2.08 (1.88 to 2.3)	0.11 (0.01 to 0.36)	-94.8 (-99.4 to -82.3)
		Staphylococcus aureus	818 (735 to 905)	864 (759 to 976)	5.7 (-3.1 to 15.1)	17.1 (15.8 to 18.4)	13.4 (12 to 14.9)	-21.6 (-30.9 to -11.2)
		Streptococcus pneumoniae	7380 (6620 to 8150)	2750 (2400 to 3110)	-62.8 (-65.7 to -59.5)	64 (59.1 to 69)	17.7 (16 to 19.6)	-72.4 (-75.7 to -68.7)
		Other viruses	3930 (3510 to 4340)	2940 (2580 to 3330)	-25.1 (-31.0 to -18.5)	13.4 (12.4 to 14.4)	7.45 (6.73 to 8.27)	-44.4 (-51.0 to -37.1)
		Other bacterial pathogens	740 (633 to 848)	354 (294 to 418)	-52.1 (-56.6 to -46.9)	6.12 (5.35 to 6.87)	2.18 (1.86 to 2.56)	-64.4 (-69.2 to -59.1)
	50-69 years	*Acinetobacter baumannii*	485 (409 to 564)	231 (196 to 270)	-52.2 (-58.0 to -45.6)	9.89 (8.54 to 11.3)	4.62 (3.99 to 5.37)	-53.2 (-59.4 to -46.1)
		Chlamydia spp.	1530 (1340 to 1750)	731 (651 to 823)	-52.1 (-57.7 to -45.8)	7.75 (6.94 to 8.69)	3.63 (3.21 to 4.09)	-53.0 (-59.6 to -46.1)
		Enterobacter spp.	280 (241 to 325)	361 (317 to 412)	29.4 (11.9 to 47.7)	5.41 (4.73 to 6.19)	6.83 (5.97 to 7.75)	26.7 (7.7 to 47.3)
		Escherichia coli	736 (647 to 842)	698 (629 to 777)	-4.9 (-17.0 to 8.0)	14.7 (13.1 to 16.5)	13.7 (12.4 to 15)	-6.9 (-19.8 to 6.8)
		Fungus	704 (607 to 808)	648 (569 to 739)	-7.6 (-19.5 to 5.1)	13.9 (12.2 to 15.6)	12.5 (11 to 14.1)	-9.6 (-20.9 to 3.5)

		Group B Streptococcus	616 (538 to 709)	659 (587 to 737)	7.3 (-7.1 to 22.6)	8.75 (7.75 to 9.89)	9.16 (8.19 to 10.2)	5.0 (-9.8 to 21.9)
		Haemophilus influenzae	2220 (2000 to 2470)	1650 (1480 to 1820)	-25.7 (-34.4 to -17.2)	16.9 (15.5 to 18.6)	8.2 (7.45 to 9.03)	-51.5 (-57.7 to -45.1)
		Influenza	2660 (2410 to 2980)	196 (22.1 to 609)	-92.6 (-99.2 to -77.3)	39.7 (36.5 to 43.2)	2.84 (0.32 to 9.18)	-92.9 (-99.2 to -76.8)
		Klebsiella pneumoniae	1510 (1360 to 1680)	1260 (1140 to 1400)	-16.2 (-25.7 to -6.7)	30 (27.7 to 32.6)	24.6 (22.3 to 27.1)	-18.0 (-27.9 to -6.9)
		Legionella spp.	1720 (1510 to 1970)	1980 (1800 to 2190)	15.5 (1.0 to 30.8)	23.5 (21 to 26.3)	26.5 (24 to 29.3)	13.1 (-2.3 to 29.3)
		Mycoplasma spp.	2220 (1970 to 2490)	1540 (1400 to 1710)	-30.5 (-38.2 to -22.0)	11.7 (10.6 to 12.9)	7.95 (7.18 to 8.77)	-31.9 (-40.4 to -22.8)
		Polymicrobial	87.5 (55.2 to 138)	68.5 (39.1 to 115)	-22.1 (-34.3 to -8.8)	1.77 (1.11 to 2.8)	1.36 (0.79 to 2.26)	-23.8 (-36.3 to -10.4)
		Pseudomonas aeruginosa	1850 (1650 to 2080)	2580 (2340 to 2860)	39.5 (23.0 to 55.6)	25.6 (23.3 to 28.1)	34.9 (31.7 to 38.4)	36.6 (19.2 to 56.1)
		Respiratory syncytial virus	615 (541 to 698)	55.1 (6.43 to 173)	-91.0 (-99.0 to -73.1)	4.19 (3.7 to 4.71)	0.36 (0.04 to 1.21)	-91.3 (-99.0 to -71.4)
		Staphylococcus aureus	2370 (2140 to 2660)	3310 (3010 to 3660)	39.8 (24.2 to 54.9)	67 (61.5 to 72.7)	91.5 (83.8 to 100)	36.9 (21.1 to 55.2)
		Streptococcus pneumoniae	10600 (9560 to 11700)	5380 (4910 to 5930)	-48.9 (-54.6 to -43.4)	124 (115 to 135)	62 (56.3 to 68.1)	-50.0 (-56.0 to -43.6)
		Other viruses	4920 (4440 to 5490)	5270 (4800 to 5830)	7.2 (-4.2 to 18.4)	22.8 (21 to 24.6)	23.9 (21.7 to 26.1)	5.0 (-7.7 to 18.4)
		Other bacterial pathogens	2590 (2250 to 2960)	1630 (1390 to 1880)	-37.2 (-44.2 to -28.9)	29.1 (25.6 to 33.1)	17.9 (15.5 to 20.7)	-38.4 (-46.1 to -29.5)
	70+ years	*Acinetobacter baumannii*	643 (541 to 756)	691 (590 to 806)	7.7 (-4.0 to 22.2)	47.8 (40.6 to 55.4)	67.5 (52.9 to 79.2)	41.4 (20.3 to 62.0)
		Chlamydia spp.	2420 (2110 to 2740)	2600 (2340 to 2930)	8.0 (-4.5 to 22.2)	44.6 (39.6 to 49.8)	63.1 (51.4 to 71.5)	41.7 (20.9 to 61.5)
		Enterobacter spp.	362 (308 to 422)	1050 (918 to 1180)	191.4 (153.2 to 231.9)	25.5 (21.9 to 29.4)	97.2 (77.1 to 112)	282.5 (218.9 to 342.6)
		Escherichia coli	1200 (1050 to 1360)	2560 (2310 to 2840)	114.1 (89.6 to 144.0)	87.3 (77.2 to 96.4)	245 (197 to 275)	181.0 (136.3 to 222.1)
		Fungus	521 (443 to 598)	1080 (953 to 1220)	108.0 (83.6 to 139.9)	37.4 (32.5 to 42.5)	102 (81.8 to 117)	173.0 (131.5 to 211.7)
		Group B Streptococcus	750 (652 to 855)	1810 (1620 to 2020)	141.6 (111.6 to 176.1)	38.8 (34.2 to 43.7)	123 (99.8 to 139)	217.0 (170.5 to 264.0)
		Haemophilus influenzae	2650 (2370 to 2960)	4430 (4000 to 4860)	67.2 (49.6 to 87.4)	73.7 (66.7 to 80.3)	108 (87.8 to 121)	46.3 (27.7 to 64.3)
		Influenza	5370 (4790 to 5970)	887 (100 to 2770)	-83.4 (-98.1 to -48.4)	292 (266 to 318)	62.6 (7.55 to 202)	-78.5 (-97.5 to -29.3)
		Klebsiella pneumoniae	2210 (1970 to 2450)	4150 (3760 to 4580)	88.6 (69.8 to 111.4)	160 (145 to 174)	395 (320 to 442)	147.5 (113.0 to 180.3)
		Legionella spp.	1640 (1430 to 1860)	4250 (3840 to 4710)	160.1 (129.4 to 197.0)	81.7 (72.1 to 90.9)	279 (227 to 313)	241.3 (191.0 to 290.4)
		Mycoplasma spp.	2490 (2220 to 2790)	3890 (3510 to 4300)	56.6 (39.8 to 76.9)	47.8 (43 to 52.5)	98.2 (79.5 to 110)	105.5 (77.4 to 131.9)

		Polymicrobial	133 (81.7 to 215)	235 (129 to 397)	75.4 (49.5 to 105.1)	9.81 (6.06 to 15.5)	22.7 (12.7 to 40)	130.1 (90.1 to 169.5)
		Pseudomonas aeruginosa	2560 (2280 to 2870)	8030 (7280 to 8820)	214.2 (180.8 to 254.1)	129 (115 to 140)	530 (429 to 594)	312.3 (251.7 to 364.0)
		Respiratory syncytial virus	765 (671 to 873)	154 (16.7 to 483)	-79.8 (-97.6 to -37.6)	19 (16.9 to 21.1)	4.96 (0.59 to 16.3)	-73.7 (-96.9 to -14.3)
		Staphylococcus aureus	4070 (3640 to 4520)	12800 (11600 to 14100)	214.9 (184.9 to 252.4)	419 (380 to 453)	1730 (1400 to 1930)	313.2 (256.2 to 362.8)
		Streptococcus pneumoniae	14400 (13000 to 16000)	16500 (15100 to 18200)	15.0 (4.4 to 29.0)	617 (563 to 668)	932 (753 to 1040)	50.9 (30.2 to 68.3)
		Other viruses	6640 (5930 to 7360)	16000 (14500 to 17700)	141.5 (117.8 to 170.8)	112 (101 to 120)	355 (286 to 396)	216.9 (174.5 to 254.7)
		Other bacterial pathogens	4610 (4000 to 5280)	6510 (5630 to 7480)	41.4 (26.3 to 60.3)	191 (166 to 215)	354 (279 to 417)	86.0 (59.8 to 110.8)
San Marino	All Ages	*Acinetobacter baumannii*	1.19 (1 to 1.4)	1.86 (1.57 to 2.19)	56.9 (41.5 to 74.7)	0.05 (0.04 to 0.06)	0.04 (0.03 to 0.06)	-11.2 (-39.4 to 22.5)
		Chlamydia spp.	5.97 (5.35 to 6.69)	7.31 (6.61 to 8.15)	22.7 (11.5 to 35.6)	0.06 (0.05 to 0.07)	0.04 (0.03 to 0.06)	-29.7 (-51.3 to -3.8)
		Enterobacter spp.	1.26 (1.09 to 1.45)	3.08 (2.69 to 3.51)	144.4 (119.4 to 172.6)	0.05 (0.04 to 0.06)	0.07 (0.04 to 0.09)	39.6 (-5.2 to 90.0)
		Escherichia coli	3.29 (2.94 to 3.67)	6.7 (6.01 to 7.4)	103.9 (86.3 to 127.2)	0.14 (0.11 to 0.17)	0.16 (0.1 to 0.22)	14.1 (-22.4 to 56.5)
		Fungus	2.02 (1.79 to 2.3)	3.75 (3.33 to 4.17)	86.4 (68.2 to 106.1)	0.07 (0.05 to 0.08)	0.07 (0.05 to 0.1)	10.3 (-25.1 to 51.4)
		Group B Streptococcus	2.6 (2.34 to 2.9)	5.51 (5.01 to 6.08)	112.0 (91.7 to 136.0)	0.07 (0.06 to 0.08)	0.09 (0.06 to 0.12)	23.1 (-15.4 to 68.4)
		Haemophilus influenzae	10.8 (9.89 to 11.7)	14.5 (13.3 to 16)	34.5 (25.2 to 45.9)	0.15 (0.12 to 0.17)	0.08 (0.05 to 0.11)	-43.7 (-61.6 to -23.8)
		Influenza	16.9 (15.4 to 18.4)	2 (1.04 to 3.45)	-88.1 (-93.9 to -79.3)	0.53 (0.43 to 0.62)	0.03 (0.02 to 0.06)	-93.4 (-97.0 to -88.3)
		Klebsiella pneumoniae	6.63 (6.07 to 7.24)	12.1 (10.9 to 13.3)	82.3 (68.2 to 99.3)	0.26 (0.21 to 0.3)	0.28 (0.18 to 0.38)	6.3 (-26.7 to 44.5)
		Legionella spp.	8 (7.15 to 8.88)	15.3 (14 to 16.9)	91.7 (73.6 to 112.8)	0.2 (0.16 to 0.24)	0.22 (0.14 to 0.3)	11.6 (-22.8 to 51.5)
		Mycoplasma spp.	11.6 (10.5 to 12.6)	15.4 (14.1 to 16.7)	33.2 (23.7 to 44.2)	0.09 (0.08 to 0.11)	0.08 (0.05 to 0.1)	-19.3 (-44.0 to 11.0)
		Polymicrobial	0.32 (0.19 to 0.54)	0.61 (0.34 to 1.05)	86.2 (65.7 to 110.8)	0.01 (0.01 to 0.02)	0.01 (0.01 to 0.03)	6.4 (-27.3 to 45.4)
		Pseudomonas aeruginosa	10.3 (9.36 to 11.4)	25.6 (23.3 to 28.3)	148.6 (128.5 to 172.4)	0.28 (0.23 to 0.34)	0.4 (0.26 to 0.55)	41.6 (-3.0 to 93.5)
		Respiratory syncytial virus	6.4 (5.83 to 7.01)	0.63 (0.32 to 1.07)	-90.2 (-94.9 to -82.9)	0.06 (0.05 to 0.07)	0 (0 to 0.01)	-93.8 (-97.2 to -89.1)
		Staphylococcus aureus	13.4 (12.3 to 14.6)	35.4 (32.2 to 38.9)	163.6 (145.9 to 187.1)	0.8 (0.65 to 0.94)	1.19 (0.76 to 1.63)	48.9 (3.7 to 101.7)
		Streptococcus pneumoniae	56.2 (51.9 to 60.6)	60.2 (55.4 to 65.4)	7.2 (0.5 to 15.7)	1.13 (0.93 to 1.31)	0.75 (0.48 to 1.03)	-33.9 (-54.1 to -10.0)
		Other viruses	37.4 (34.6 to 40.2)	60.9 (55.8 to 66.1)	62.8 (52.4 to 75.9)	0.28 (0.23 to 0.32)	0.29 (0.18 to 0.4)	4.5 (-27.3 to 42.3)

		Other bacterial pathogens	11.9 (10.2 to 13.7)	17.7 (15.2 to 20.7)	49.4 (36.1 to 64.5)	0.3 (0.23 to 0.36)	0.24 (0.15 to 0.35)	-16.8 (-42.4 to 13.8)
	Under 5	*Acinetobacter baumannii*	0.01 (0.01 to 0.02)	0.01 (0 to 0.01)	-51.6 (-57.4 to -44.8)	0 (0 to 0)	0 (0 to 0)	-87.6 (-93.9 to -78.3)
		Chlamydia spp.	0.19 (0.16 to 0.23)	0.09 (0.08 to 0.12)	-50.8 (-57.1 to -44.0)	0 (0 to 0)	0 (0 to 0)	-89.8 (-94.6 to -82.7)
		Enterobacter spp.	0.03 (0.03 to 0.04)	0.03 (0.02 to 0.04)	-9.7 (-22.5 to 5.2)	0 (0 to 0)	0 (0 to 0)	-82.3 (-90.7 to -70.1)
		Escherichia coli	0.09 (0.07 to 0.11)	0.08 (0.06 to 0.1)	-14.0 (-26.0 to -1.6)	0 (0 to 0)	0 (0 to 0)	-83.4 (-91.4 to -71.4)
		Fungus	0.02 (0.01 to 0.02)	0.01 (0.01 to 0.02)	-22.5 (-33.8 to -9.2)	0 (0 to 0)	0 (0 to 0)	-85.3 (-92.5 to -74.0)
		Group B Streptococcus	0.09 (0.07 to 0.11)	0.07 (0.06 to 0.09)	-16.9 (-28.5 to -4.5)	0 (0 to 0)	0 (0 to 0)	-83.7 (-91.4 to -72.6)
		Haemophilus influenzae	0.87 (0.72 to 1.05)	0.42 (0.34 to 0.52)	-51.6 (-57.3 to -45.5)	0 (0 to 0)	0 (0 to 0)	-93.8 (-96.8 to -89.6)
		Influenza	0.69 (0.58 to 0.84)	0.03 (0.01 to 0.04)	-96.3 (-98.1 to -93.7)	0 (0 to 0.01)	0 (0 to 0)	-99.3 (-99.7 to -98.6)
		Klebsiella pneumoniae	0.33 (0.28 to 0.4)	0.17 (0.14 to 0.21)	-47.9 (-54.0 to -41.4)	0 (0 to 0)	0 (0 to 0)	-89.8 (-94.6 to -82.5)
		Legionella spp.	0.05 (0.04 to 0.07)	0.04 (0.03 to 0.06)	-24.9 (-34.1 to -14.5)	0 (0 to 0)	0 (0 to 0)	-84.3 (-91.8 to -72.8)
		Mycoplasma spp.	0.47 (0.38 to 0.58)	0.42 (0.34 to 0.52)	-9.8 (-20.6 to 1.9)	0 (0 to 0)	0 (0 to 0)	-82.9 (-91.1 to -71.1)
		Polymicrobial	0.02 (0.01 to 0.03)	0.01 (0.01 to 0.02)	-43.6 (-51.7 to -35.4)	0 (0 to 0)	0 (0 to 0)	-83.8 (-92.6 to -70.7)
		Pseudomonas aeruginosa	0.3 (0.25 to 0.37)	0.36 (0.29 to 0.44)	19.0 (5.1 to 34.6)	0 (0 to 0)	0 (0 to 0)	-77.6 (-88.1 to -61.5)
		Respiratory syncytial virus	2.51 (2.11 to 3)	0.15 (0.07 to 0.26)	-94.2 (-97.0 to -90.2)	0.01 (0.01 to 0.01)	0 (0 to 0)	-98.9 (-99.6 to -97.8)
		Staphylococcus aureus	0.31 (0.26 to 0.38)	0.3 (0.24 to 0.37)	-3.8 (-12.6 to 7.3)	0 (0 to 0.01)	0 (0 to 0)	-81.7 (-90.2 to -69.2)
		Streptococcus pneumoniae	4.3 (3.6 to 5.17)	1.3 (1.05 to 1.61)	-69.8 (-72.5 to -66.5)	0.02 (0.02 to 0.03)	0 (0 to 0)	-94.2 (-97.0 to -90.1)
		Other viruses	4.94 (4.13 to 5.88)	2.58 (2.11 to 3.18)	-47.7 (-52.2 to -42.6)	0.01 (0.01 to 0.02)	0 (0 to 0)	-89.9 (-94.7 to -83.0)
		Other bacterial pathogens	0.1 (0.07 to 0.12)	0.06 (0.04 to 0.07)	-40.8 (-48.4 to -32.3)	0 (0 to 0)	0 (0 to 0)	-88.3 (-94.0 to -79.8)
	5-14 years	*Acinetobacter baumannii*	0.02 (0.02 to 0.03)	0.02 (0.01 to 0.02)	-12.4 (-21.4 to -2.8)	0 (0 to 0)	0 (0 to 0)	-73.2 (-83.0 to -59.2)
		Chlamydia spp.	0.15 (0.11 to 0.2)	0.11 (0.08 to 0.14)	-26.8 (-33.6 to -19.2)	0 (0 to 0)	0 (0 to 0)	-77.5 (-85.8 to -66.3)
		Enterobacter spp.	0.02 (0.02 to 0.03)	0.03 (0.02 to 0.04)	41.3 (26.2 to 57.2)	0 (0 to 0)	0 (0 to 0)	-56.7 (-72.8 to -35.0)
		Escherichia coli	0.05 (0.03 to 0.06)	0.06 (0.04 to 0.07)	16.7 (5.4 to 29.1)	0 (0 to 0)	0 (0 to 0)	-64.2 (-77.1 to -45.8)
		Fungus	0.06 (0.04 to 0.08)	0.06 (0.05 to 0.09)	14.1 (3.0 to 26.2)	0 (0 to 0)	0 (0 to 0)	-65.0 (-77.5 to -47.0)

[truncated: 493,292 more chars]
