# Supplementary material for: Global, regional, and national incidence and mortality burden of non-COVID-19 lower respiratory infections and aetiologies, 1990–2021: a systematic analysis from the Global Burden of Disease Study 2021
Source: Lancet Infect Dis. 2024 Sep;24(9):974–1002. doi: 10.1016/S1473-3099(24)00176-2 (PMC11339187; doi:10.1016/S1473-3099(24)00176-2)
Supplement: Supplementary appendix 1 [file mmc1.pdf]

THE LANCET
Infectious Diseases

Supplementary appendix 1

This appendix formed part of the original submission and has been peer reviewed. We post it as supplied by the authors.

Supplement to: GBD 2021 Lower Respiratory Infections and Antimicrobial Resistance Collaborators. Global, regional, and national incidence and mortality burden of non-COVID-19 lower respiratory infections and aetiologies, 1990–2021: a systematic analysis from the Global Burden of Disease Study 2021. *Lancet Infect Dis* 2024; published online April 15. https://doi.org/10.1016/S1473-3099(24)00176-2.

Appendix 1

Methods Appendix for "Global, regional, and national incidence and mortality burden of non-COVID lower respiratory infections and aetiologies, 1990–2021: a systematic analysis from the Global Burden of Disease Study 2021"

This appendix provides further methodological detail and results for "Global Burden of Lower Respiratory Infections, 1990-2021"

All the material in the paper itself is novel although it builds off previous GBD works. However, parts of the supplemental methods appendix include sections adapted from the GBD Capstones previously published in The Lancet and previous IHME work on antimicrobial resistance.[1]

Table of Contents

Methods 4
Region Classification..4
Appendix Figure 1: GBD regions and super-regions 4
Case Definition 5
Cause of Death Input data..5
Cause of Death Modelling Strategy..6
Appendix Figure 2: Flowchart of LRI mortality estimation 6
Modelling fatal LRI...7
Appendix Table 1: Covariates used for LRI cause-of-death ensemble modelling for children under 5 years ..8
Appendix Table 2: Covariates used for LRI cause-of-death ensemble modelling for 5–95+ years 9
Non-Fatal Input data 9
Model inputs 9
Appendix Figure 3: Prisma Diagram of systematic review for LRI incidence and prevalence data 10
Bias corrections.. 10
Appendix Table 3A: MR-BRT crosswalk adjustment factors for lower respiratory infections, surveys 11
Appendix Figure 4: Meta-regression of the log ratio of community-level clinician-diagnosed LRI to clinical inpatient LRI prevalence 12
Appendix Table 3B: MR-BRT crosswalk adjustment factors for lower respiratory infections: clinical inpatient, claims, hospital-based studies, and self-reported data to the level of the reference case definition 12
Severity splits 13
Appendix Table 4: Data inputs for lower respiratory infections morbidity modeling by parameter 14
Appendix Table 5: Data inputs for lower respiratory infections morbidity modeling by parameter 14
Non-Fatal Modelling strategy.. 15
Appendix Figure 5: Flowchart of LRI non-fatal burden estimation 15
Appendix Table 6: Summary of covariates used in the LRI DisMod-MR meta-regression model................. 17

Aetiology Estimation 17
Aetiologies Input Data 17
Appendix Table 7: ICD Codes Used in Aetiology Estimation 17
Appendix Figure 6: Prisma Diagram of systematic review for PCV vaccine efficacy data............................. 19
Appendix Figure 7: Non-fatal LRI aetiology site-years.. 20
Appendix Figure 8: Fatal LRI aetiology site-years 20
Nonfatal Aetiology Modelling Strategy... 20
Appendix Table 8: Covariates used in aetiology modeling 22
Fatal Aetiology Modelling Strategy 24
COVID adjustment.. 26
Appendix Figure 9: RegMod example for influenza in Indonesia. The top panel represents cases over time; points are the observed number of reported cases and line is the interpolated number of reported cases from the RegMod model. The bottom panel represents the residual over time and the time trend. 27

Statement of GATHER Compliance 30
Appendix Table 9. Checklist of information that should be included in reports of global health estimates, with description of compliance and location of information the current study... 30

References 32

Authors' Contributions 34

Managing the overall research enterprise 34
Writing the first draft of the manuscript 34
Primary responsibility for applying analytical methods to produce estimates 34
Primary responsibility for seeking, cataloguing, extracting, or cleaning data; designing or coding figures and tables 34
Providing data or critical feedback on data sources 34
Developing methods or computational machinery 35
Providing critical feedback on methods or results 35
Drafting the work or revising it critically for important intellectual content 36
Managing the estimation or publications process 37

Methods

Region Classification

Regions and super-regions are classified as described in the GBD 2010 capstone paper, appendix, page 6.[2] A copy of Web Figure 2 from that study's appendix is provided below.

Appendix Figure 1: GBD regions and super-regions

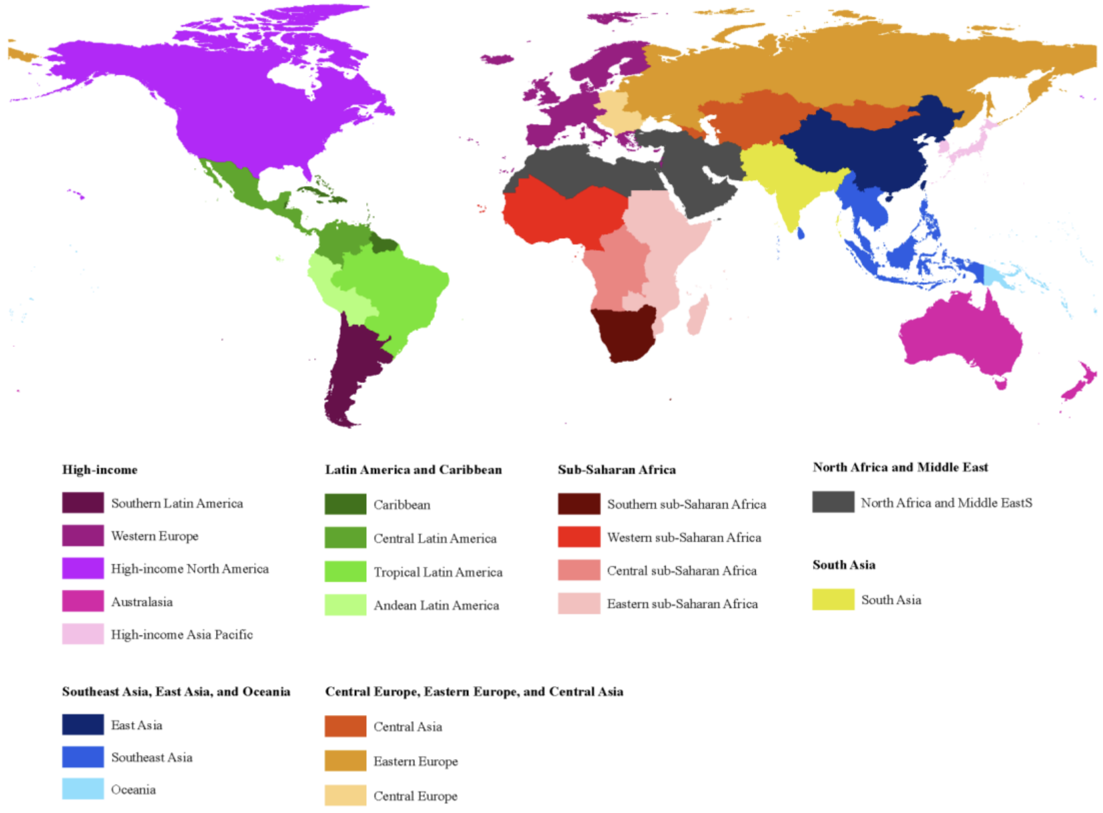

Socio-Demographic Index

SDI is a composite indicator of a country's lag-distributed income per capita, average years of schooling, and the total fertility rate (TFR) in females under the age of 25 years. SDI ranges from 0 to 1, with 0 representing the lowest income per capita, lowest educational attainment and highest fertility under age 25 years observed across all GBD geographies while 1 represents the highest income per capita, highest educational attainment and lowest fertility under 25 years observed across all GBD geographies. More information can be found within the appendix of the GBD 2021 fertility and mortality paper.[3]

The GBD 2021 SDI quintile cutoffs are:

Quintile	Lower cutoff	Upper cutoff
Low SDI	0	46.58
Low-middle SDI	46.59	61.88
Middle SDI	61.89	71.19
High-middle SDI	71.20	81.02
High SDI	81.03	100

Case Definition

Lower respiratory infections (LRI) are defined by the GBD study as pneumonia or bronchiolitis. Symptoms include cough, fever, and shortness of breath. Included in the GBD modelling were cases meeting ICD-9 diagnostics criteria for LRI (079.82, 466-469, 470.0, 480-481.9, 482.0-482.89, 483.0-483.9, 484.1-484.2, 484.6-484.7, 487-490.9, 510-511.9, 513.0-513.9) and ICD-10 diagnostic criteria for LRI (A48.1, A70, B96.0-96.1, B97.21, B97.4-B97.6, J09-J11.89, J12-J13.9, J14-J14.0, J15-J15.8, J20-J21.9, J85.1, J91.0, P23.0-P23.4, U04-U04.9).[4] In addition, the following garbage codes were redistributed entirely to LRI in ICD-9 (482, 482.9-483, 484, 484.3-484.5, 484.8-486.9, 770.0, V12.61) and ICD-10 (J15.9, J16-J19.6, J22-J22.9, P23, P23.5-P23.9).[4] The GBD case definition of LRI does not include tuberculosis or COVID-19; although these pathogens can infect the lower respiratory tract, they are modeled separately due to their individual public health significance.

Cause of Death Input data

Input data for the overall LRI model came from the cause of death (CoD) database. The CoD database contains several types of data sources, five of which are used in estimation of LRI: vital registration (VR), verbal autopsy (VA), sample vital registration (VR-S), surveillance, and minimally invasive tissue sample (MITS) diagnoses. In locations with robust VR systems, VR is the primary source of data for causes of death. In countries with incomplete or nonexistent VR systems, vital statistics for causes of death are supplemented with these other data types.[5] We outliered data that violated well-established time or age trends.

Cause of Death Modelling Strategy

Appendix Figure 2: Flowchart of LRI mortality estimation

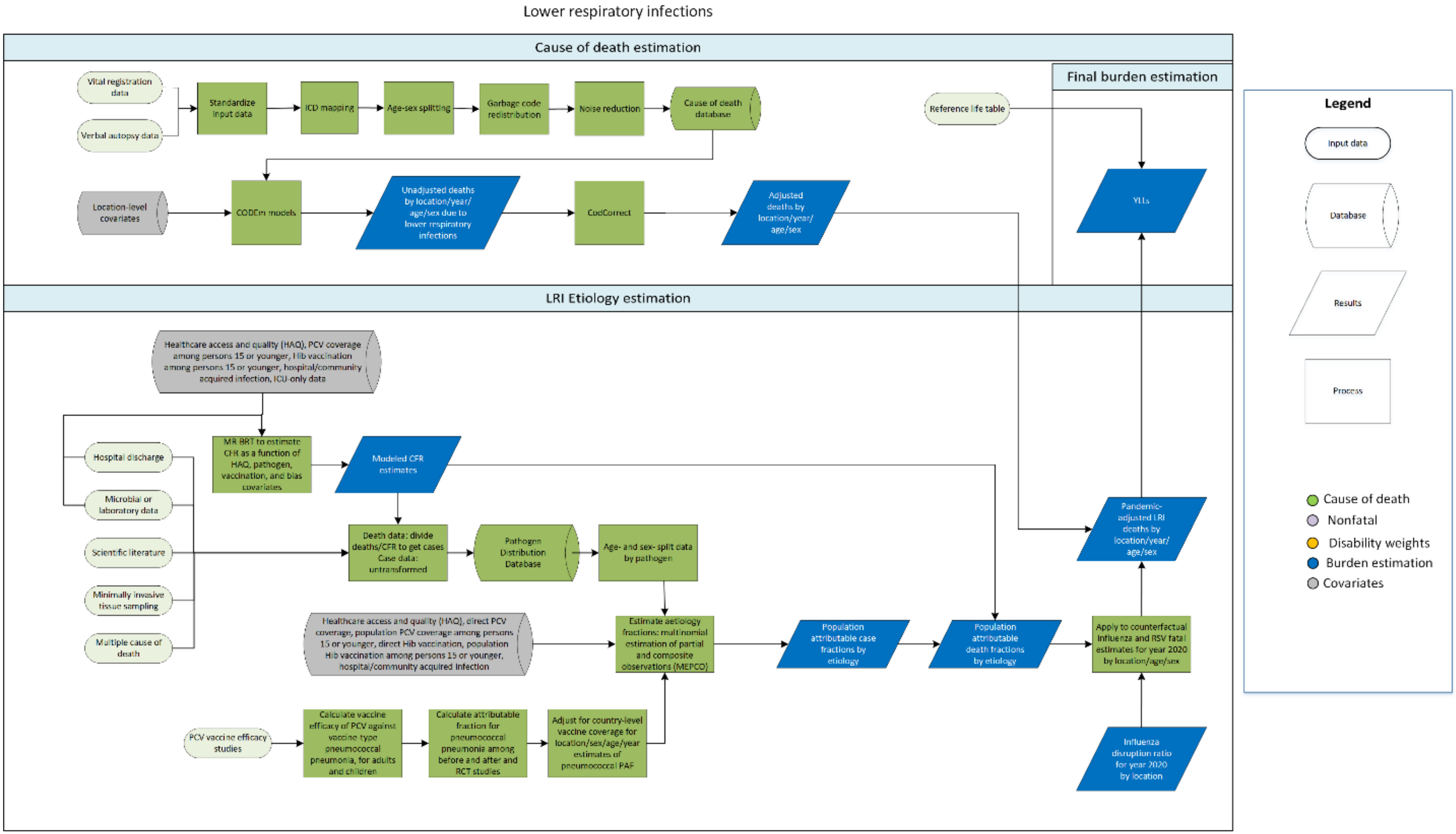

Modelling fatal LRI

We modelled deaths due to all LRI with two CODEm models, separately for each sex and two age categories (under 5 and 5 years and above), as the mortality trends differ substantially between these age groups. The final sex-specific models for deaths due to all LRI were a hybridised model of separate global and data-rich models for males and females.

In the CODEm framework, four families of statistical models are used: linear mixed effects regression (LMER) models of the natural log of the cause-specific death rate, LMER models of the logit of the cause fraction, spatiotemporal Gaussian process regression (ST-GPR) models of the natural logarithm of the cause-specific death rate, and ST-GPR models of the logit of the cause fraction (see the 2x2 table in Foreman et al).[6] For each family of models, all plausible relationships between covariates and the response variable are identified. Based on the evidence of a causal relationship with LRI mortality, covariates are ranked from 1 (proximally related) to 3 (distally related). The direction of the association between each covariate and LRI mortality is assigned as a prior based on the literature (Appendix Tables 1 and 2). Because all possible combinations of selected covariates are considered for each family of models, multi-collinearity between covariates may produce implausible signs on coefficients or unstable coefficients. Each combination is therefore tested for statistical significance (covariate coefficients must have a coefficient with p-value $< 0{\cdot}05$) and plausibility (the coefficients must have the directions expected on the basis of the literature). Only covariate combinations meeting these criteria are retained. This selection process is run for both cause fractions and death rates, then ST-GPR and LMER-only models are created for each set of covariates.

The families of models that go through ST-GPR incorporate information about data variance. The main inputs for a Gaussian process regression (GPR) are a mean function, a covariance function, and data variance for each data point. These inputs are described in detail in Foreman et al. Three components of data variance are now used in CODEm: sampling variance, non-sampling variance, and garbage code redistribution variance. The computation of sampling variance and non-sampling variance has not changed since previous iterations of the GBD and is also described in Foreman et al.[6] Garbage code redistribution variance is computed in the CoD database process. Since variance is additive, we calculate total data variance as the sum of sampling variance, non-sampling variance, and redistribution variance. Increased data variance in GPR may result in the GPR draws not following the data point as closely.

The performance of all models (individual and ensemble) is evaluated by means of out-of-sample predictive validity tests. Thirty percent of the data are randomly excluded from the initial model fits. These individual model fits are evaluated and ranked by using half of the excluded data (15% of the total), then used to construct the ensembles on the basis of their performance. Data are held out from the analysis on the basis of the cause-specific missingness patterns for ages and years across locations. Out-of-sample predictive validity testing is repeated 20 times for each model, which has been shown to produce stable results. These performance tests include the root mean square error (RMSE) for the log of LRI death rate, the direction of the predicted versus actual trend in the data, and the coverage of the predicted 95% UI.

The component models are weighted on the basis of their predictive validity rank to determine their contribution to the ensemble estimate. The relative weights are determined both by the model ranks and by a parameter ψ, whose value determines how quickly the weights taper off as rank decreases. The

distribution of ψ is described in more detail in Foreman et al. A set of ensemble models is then created by using the weights constructed from the combinations of ranks and ψ values. These ensembles are tested by using the predictive validity metrics described in the previous section on the remaining 15% of the data, and the ensemble with the best performance in out-of-sample trend and RMSE is chosen as the final model. Lastly, 1000 draws are created for the final ensemble, and the number of draws contributed by each model is proportional to its weight. The mean of the draws is used as the final estimate for the CODEm process, and a 95% UI is created from the 0·025 and 0·975 quantiles of the draws.

Similar to other models of mortality in GBD, LRI mortality models are single-cause, requiring that the sum of all mortality models must be equal to the all-cause mortality envelope. We correct LRI mortality estimates, and other causes of mortality, by rescaling them according to the uncertainty around the cause-specific mortality rate. This process is called CoDCorrect and is essential to ensure internal consistency among causes of death.

In past GBD cycles, estimates of PCV3, Hib3, and DTP3 vaccine coverage among infants in the modelled year were used as the primary covariate for this linear regression. In GBD 2021, we now use a lagged mean of PCV3, Hib3, and DTP3 vaccine coverage calculated over a rolling, five-year interval to capture population-level vaccine-derived immunity among under-5-year-olds, including coverage both in the current year and in recent years.

Appendix Table 1: Covariates used for LRI cause-of-death ensemble modelling for children under 5 years

**Level**	**Covariate**	**Direction of the association**
**1**	Childhood stunting summary exposure value (SEV)	+
	Childhood underweight SEV	+
	Childhood wasting SEV	+
	Indoor air pollution	+
	LRI SEV	+
	Antibiotics for LRI	-
	Hib3 vaccine coverage proportion, lagged	-
	PCV3 vaccine coverage proportion, lagged	-
**2**	Secondhand smoking prevalence	+
	Zinc deficiency	+
	DTP3 vaccine coverage proportion, lagged	-
	Healthcare Access and Quality Index	-
	Ambient particulate matter SEV	+
	Household air pollution SEV	+
	Outdoor air pollution ($PM_{2.5}$)	+
	Handwashing SEV	+
**3**	Sanitation SEV	+
	Population density >1000/km^2	+
	Maternal education	-
	Socio-demographic Index	-

Appendix Table 2: Covariates used for LRI cause-of-death ensemble modelling for 5–95+ years

Level	Covariate	Direction of the association
1	Indoor air pollution	+
	LRI SEV	+
	Outdoor air pollution ($PM_{2.5}$)	+
	Secondhand smoking prevalence	+
	Smoking prevalence	+
2	DTP3 vaccine coverage proportion, lagged	-
	Adult underweight	+
	Healthcare Access and Quality Index	-
	PCV3 vaccine coverage proportion, lagged	-
	Handwashing access	+
3	Education years per capita	-
	Lag distributed income per capita	-
	Socio-demographic Index	-
	Sanitation SEV	+

We adjusted overall LRI mortality estimates for 2020 and 2021 to account for the reductions in influenza and RSV mortality associated with the COVID-19 pandemic, as described on page 17 in this appendix.

Non-Fatal Input data

Model inputs

Input data included all data used in GBD 2019 and new data identified in our updated systematic review, newly acquired surveys, and new claims and inpatient data. These data measure lower respiratory infection incidence and prevalence. They come from a systematic literature review, hospital inpatient and outpatient data, claims data from the USA, and surveys. In our study, we have only included population-representative surveys. We assessed representativeness by categorizing the population studied by the survey. A population-representative survey studies the general population of a nation, province, or other geographic area. As a note, we still consider a survey representative if it only focuses on certain ages or sexes, because in those cases, we only use it as an input to the model for those ages and sexes. DHS and MICS are the gold-standard examples of representative surveys. In contrast, a non-representative survey studies only a specific subgroup of the population living in a certain area, almost always a marginalized subgroup within the greater society. Examples of non-representative surveys, which we would exclude, are those that focus only on refugees, prisoners, or people who inject drugs.

Data were outliered or excluded if we found them unreasonable when compared to regional, super-regional, and global rates.

Our search string for systematic review was constructed as follows: (("lower respiratory"[MeSH] OR pneumonia[MeSH]) AND (2019/02/07[PDat] : 2020/12/31[PDat]) AND ((incidence OR prevalence OR epidemiology) OR (etiolog*[title/abstract] OR influenza[title/abstract] OR "respiratory syncytial virus"[title/abstract])) NOT(autoimmune[title/abstract] OR COPD [title/abstract] OR "cystic fibrosis"[title/abstract] OR Review[ptyp]) NOT (animals[MeSH] NOT humans[MeSH]). This string identified 284 records as detailed in Appendix Figure 2 below.

Appendix Figure 3: Prisma Diagram of systematic review for LRI incidence and prevalence data

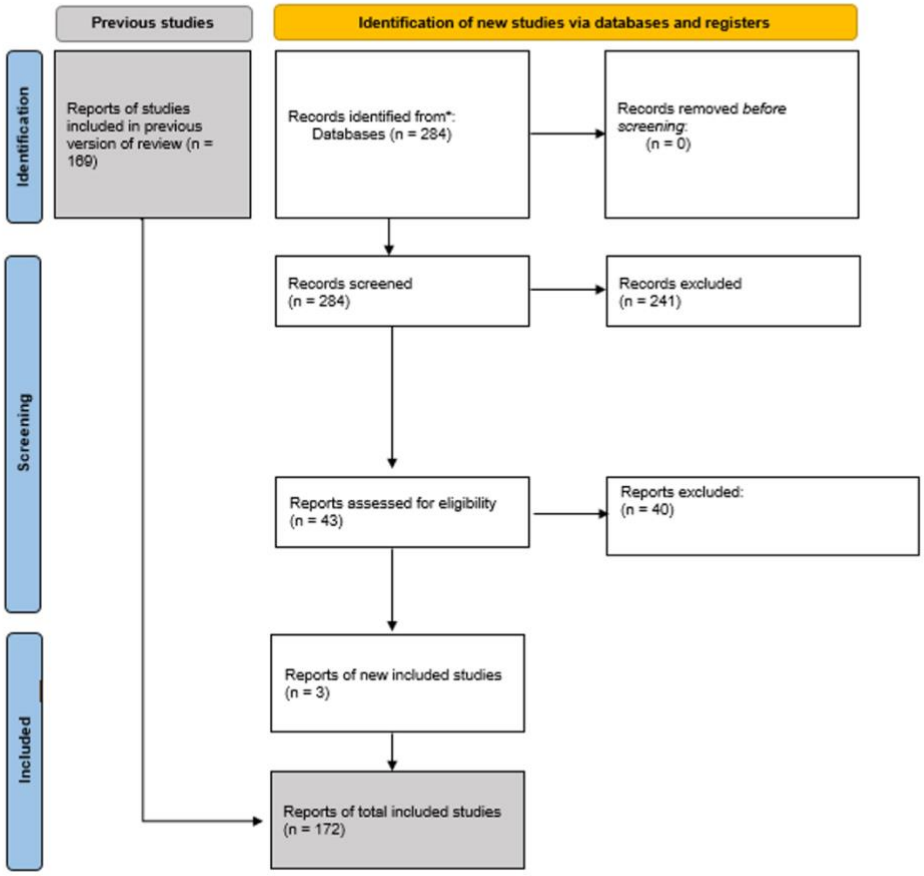

Bias corrections

To estimate the non-fatal burden of LRI, we also used self-reported prevalence of LRI symptoms from population-representative surveys, such as the Demographic and Health Survey and the Multiple Indicator Cluster Survey. We applied sampling weights to adjust for unequal probabilities of selection and non-responses to ensure representative estimates of the population. When possible, we extracted survey data by one-year age group and by sex. We converted these data from two-week period prevalence to point prevalence. The equation for this adjustment is:

$$Point\ Prevalence = \frac{Period\ Prevalence\ *\ Duration}{(Recall\ Period + Duration - 1)}$$

We accepted four survey definitions for the prevalence of symptoms of LRI: 1) Cough with difficulty breathing with symptoms in the chest with a fever was our gold standard, but we also accepted 2) Cough with difficulty breathing with symptoms in the chest *without* fever, 3) Cough with difficulty breathing with fever, and 4) Cough with difficulty breathing *without* fever. To make these definitions comparable, we identified the surveys that met the best case definition (definition 1). Within these surveys, we calculated the ratio of the prevalence of the best case definition to the prevalence of the

alternate definitions. This ratio was used as the dependent variable in a meta-regression. The results from that meta-regression were used to adjust the prevalence and uncertainty for all the surveys that reported alternate case definitions (Appendix Table 3a).

Appendix Table 3A: MR-BRT crosswalk adjustment factors for lower respiratory infections, surveys

Data Input	Reference or alternative case definition	Gamma†	Crosswalk covariate	Beta coefficient, log(95% UI)*	Adjustment Factor (95% UI)**
Cough, with difficulty breathing and fever	ref	--	--	--	--
Survey, chest without fever	alt	0.17	intercept	–0.48 (–1.28 to 0.32)	0.62 (0.28 to 1.38)
Survey, difficulty breath without fever	alt	0.51	intercept	–0.82 (–2.22 to 0.58)	0.44 (0.11 to 1.79)
Survey, difficulty breathing with fever	alt	0.22	intercept	–0.58 (–1.5 to 0.34)	0.56 (0.22 to 1.40)

**MR-BRT crosswalk adjustments can be interpreted as the factor the alternative case definition is adjusted by to reflect what it would have been had it been measured using the reference case definition. If the log/logit beta coefficient is negative, then the alternative is adjusted up to the reference. If the log/logit beta coefficient is positive, then the alternative is adjusted down to the reference.*
***The adjustment factor column is the exponentiated beta coefficient. For log beta coefficients, this is the relative rate between the two case definitions. For logit beta coefficients, this is the relative odds between the two case definitions.*
†Gamma is a measure of between-study heterogeneity and is incorporated in the calculation of variance around the beta coefficient

Survey data were adjusted for seasonality. An inclusion criterion for scientific literature is a study duration longer than one year to avoid bias in the seasonal timing of LRI. Surveys are frequently conducted over several months. To account for seasonal variation in LRI symptom prevalence, we fit a generalised additive model with an identity link function, incorporating forced periodicity for each GBD region, and assumed a normally distributed random error term with a mean of zero and a variance of σ^2.. The model is mixed-effects with random effects on each country. The model accounts for the year of the survey and the case definition used. The percent difference between the monthly model fit LRI prevalence and the corresponding regional-mean LRI prevalence is a scalar to adjust survey data by month and geography. We adjusted the self-reported survey data to the level of our reference case definition, clinician-diagnosed pneumonia or bronchiolitis, using the adjustment factor from Appendix Table 3b to enhance data comparability.In addition to survey data, hospital inpatient and US inpatient claims data were included in the LRI modelling. These data are adjusted prior to modelling for readmissions and multiple diagnoses. To make the data more consistent in the modelling process, we

converted all incidence data to prevalence. We found the ratio of the prevalence of LRI in hospitalisation records to the prevalence of LRI in our case definition (clinician-diagnosed pneumonia or bronchiolitis) for locations that contained data on both these prevalence values. We then regressed this ratio in a meta-regression to predict the adjustment factor for hospitalisation data to make them compatible with the reference case definition for our modelling. This meta-regression considered the Socio-demographic Index (SDI) as a predictor of this ratio for inpatient data, assuming that location-years with higher values of SDI are more likely to have access to health care, making this ratio smaller in those location-years (Appendix Figure 3, Table 3b). Similarly, age was considered a predictor for hospital-based studies, and data were adjusted accordingly using age midpoint (Appendix Figure 3, Table 3b).

Appendix Figure 4: Meta-regression of the log ratio of community-level clinician-diagnosed LRI to clinical inpatient LRI prevalence

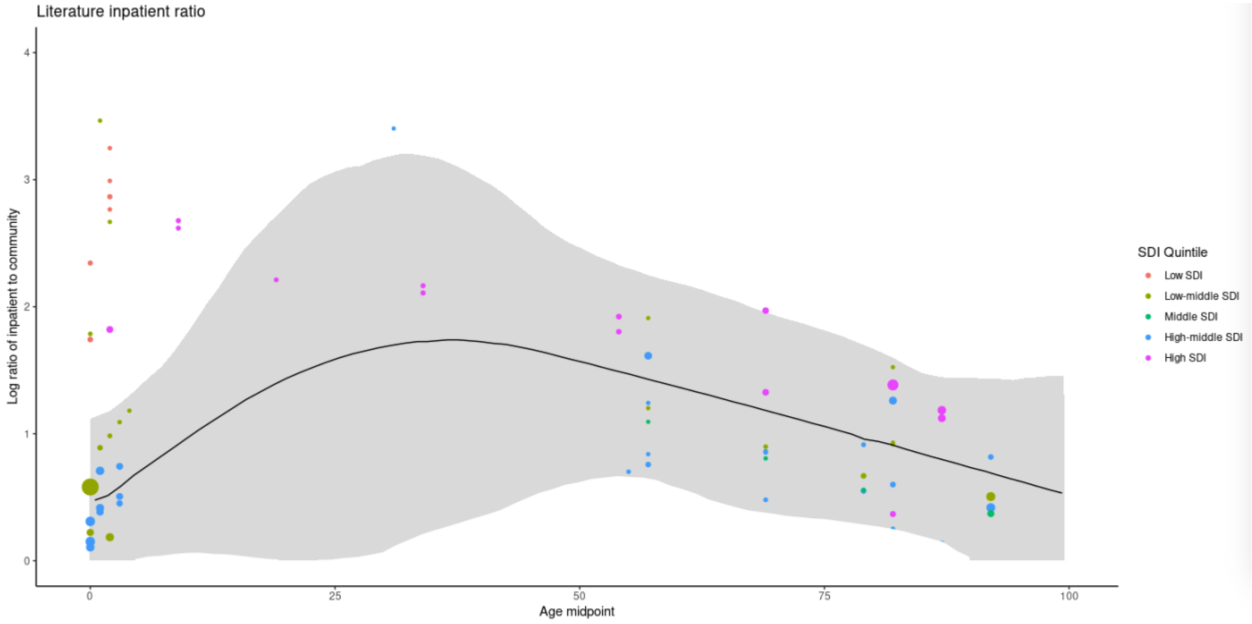

Note: The log ratios are to be interpreted as the log ratio of community level, clinician diagnosed LRI prevalence to clinical inpatient LRI prevalence. A larger ratio means that the prevalence of community level, clinician diagnosed LRI is greater than the clinical inpatient prevalence.

Claims data for GBD 2019 include MarketScan (USA), and data from Taiwan (province of China), Poland, and Russia. MarketScan data are retrieved by IHME’s Clinical Informatics Team. As with inpatient clinical data, these data are converted first to prevalence, then compared to the reference definition for LRI using a meta-regression model (Appendix Table 3b). Taiwan claims data were dropped as there were no reference data to match with and because the values there were systematically different from those in the USA.

Appendix Table 3B: MR-BRT crosswalk adjustment factors for lower respiratory infections: clinical inpatient, claims, hospital-based studies, and self-reported data to the level of the reference case definition

Data input	Reference or alternative case definition	Gamma†	Crosswalk covariate	Beta coefficient, log (95% UI)*	Adjustment factor (95% UI)**
Clinician-diagnosed	ref		--	--	--

pneumonia or bronchiolitis					
Clinical, inpatient	alt	1.43	sdi_0	2.79 (0.2 to 5.38)	16.23 (1.22 to (217.02)
Clinical, inpatient	alt		sdi_1	4.87 (2.31 to 7.43)	129.85 (10.07 to 1685.81)
Clinical, inpatient	alt		sdi_2	1.08 (–1.49 to 3.65)	2.94 (0.23 to 38.47)
Clinical, inpatient	alt		sdi_3	0.02 (–2.43 to 2.47)	1.02 (0.09 to 11.82)
Literature, hospital-based	alt	0.30	age_mid_0	1.06 (–0.31 to 2.42)	2.87 (0.73 to 11.25)
Literature, hospital-based	alt		age_mid_1	1.98 (–0.42 to 4.38)	7.23 (0.66 to 79.84)
Literature, hospital-based	alt		age_mid_2	1.31 (–0.11 to 2.74)	3.72 (0.90 to 15.49)
Literature, hospital-based	alt		age_mid_3	0.95 (–0.2 to 2.1)	2.59 (0.82 to 8.17)
Self-report	alt	0.81	Intercept	–1.19 (–2.98 to 0.6)	0.30 (0.05 to 1.82)
Claims, MarketScan	alt	0.87	intercept	1.14 (–0.69 to 2.97)	3.13 (0.5 to 19.49)

**MR-BRT crosswalk adjustments can be interpreted as the factor the alternative case definition is adjusted by to reflect what it would have been had it been measured using the reference case definition. If the log/logit beta coefficient is negative, then the alternative is adjusted up to the reference. If the log/logit beta coefficient is positive, then the alternative is adjusted down to the reference.*

***The adjustment factor column is the exponentiated beta coefficient. For log beta coefficients, this is the relative rate between the two case definitions. For logit beta coefficients, this is the relative odds between the two case definitions.*

†Gamma is a measure of between-study heterogeneity and is incorporated in the calculation of variance around the beta coefficient

We performed a systematic review of the duration of symptoms of LRI. We sought consistency with our case definition of LRI and defined our duration as the time between the onset of symptoms to the resolution of increased work of breathing. Although crucial, there were very limited data on spatial, temporal, or age-specific duration, which may vary based on severity, aetiology, and treatment. We identified 485 titles from PubMed and extracted six studies which were used in a meta-analysis (mean duration 7.79 days [6.2–9.64]). We used this as the duration of LRI in our conversions from period to point prevalence and for the conversion between incidence and prevalence.

Severity splits

The distribution of moderate (85%) and severe (15%) lower respiratory infections is determined by a meta-analysis of the ratio of severe to all LRI from studies that report the incidence of moderate and severe lower respiratory infections.

We used the health states of acute infectious disease episode, moderate and severe, with the lay descriptions and disability weight values shown in Appendix Table 4 below:

Appendix Table 4: Data inputs for lower respiratory infections morbidity modeling by parameter

Severity level	Lay description	DW (95% CI)
Moderate	Has a fever and aches and feels weak which causes some difficulty with daily activities.	0.051 (0.032 to 0.074)
Severe	Has a high fever and pain and feels very weak, which causes great difficulty with daily activities.	0.133 (0.088 to 0.19)

Appendix Table 5: Data inputs for lower respiratory infections morbidity modeling by parameter

	**Countries with data**	**Total source counts**
Incidence	162	2058
Prevalence	156	969

Non-Fatal Modelling strategy

Appendix Figure 5: Flowchart of LRI non-fatal burden estimation

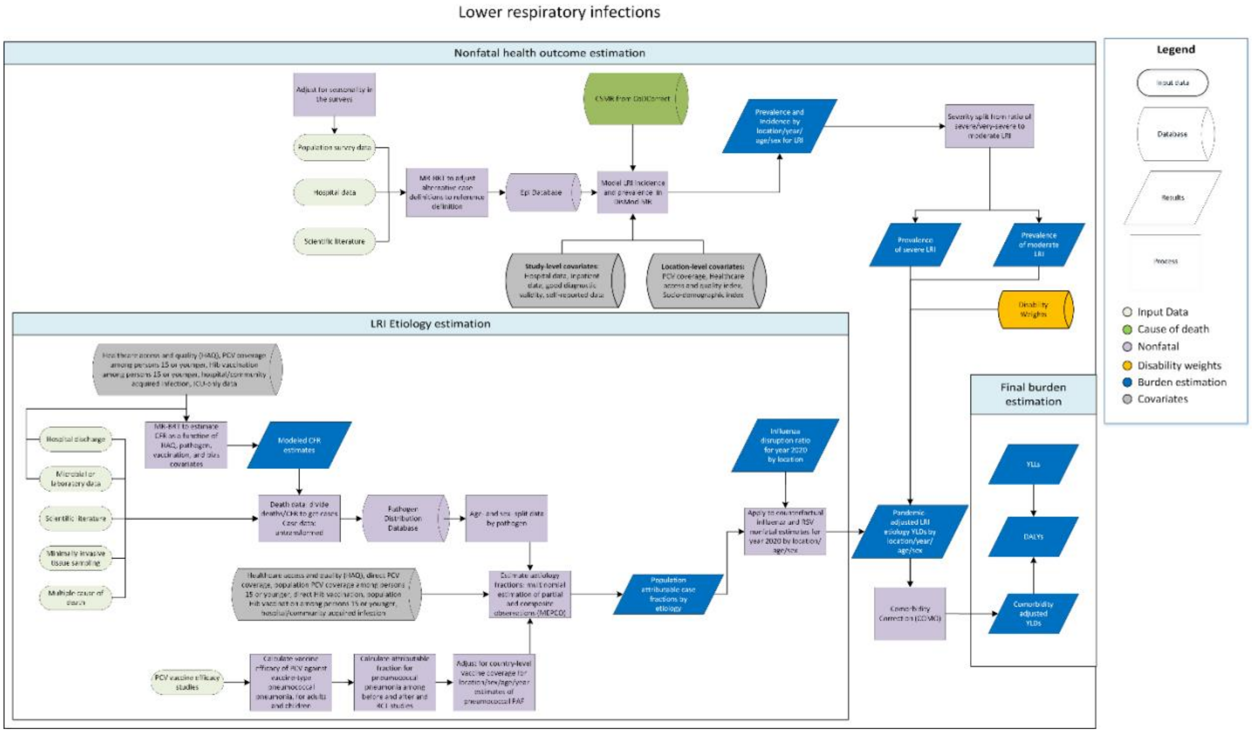

The non-fatal lower respiratory infection burden is modelled in DisMod-MR 2.1, a Bayesian meta-regression modelling framework. DisMod-MR produces estimates of the incidence, prevalence, and remission of LRI for each age, sex, geographical location, and year. We defined the time to recovery as an average of 10 days (5–15 days), which corresponds with a remission 36.5. The models are informed by country-level covariates (Appendix Table 6).

DisMod-MR 2.1 description

The sequence of estimation in DisMod MR 2.1[1] occurs at five levels: global, super-region, region, country and, where applicable, subnational location. The super-region priors are generated at the global level with mixed-effects, nonlinear regression using all available data; the super-region fit, in turn, informs the region fit, and so on down the cascade. Subnational estimation was informed by the country fit and country covariates, plus an adjustment based on the average of the residuals between the subnational location's available data and it's prior. This mimicked the impact of a random effect on estimates between subnationals. At each level of the cascade, the DisMod-MR 2.1 enforces consistency between all parameters. Analysts have the choice to branch the cascade in terms of time and sex at different levels depending on data density.[5] We used the default option to model LRI, which is to branch by sex after the global fit but to retain all years of data until the lowest level in the cascade.

The coefficients for country covariates were re-estimated at each level of the cascade. For a given location, country coefficients were calculated using both data and prior information available for that location. In GBD 2021, we generated model fits for the years 1990, 1995, 2000, 2005, 2010, 2015, 2019, 2020 and 2021, and log-linearly interpolated estimates for the intervening years. Convergence was

assessed qualitatively by visually inspecting diagnostic plots of the posterior distributions. The 95% uncertainty intervals were computed based on 1000 draws from the posterior distribution of the converged model using the 2.5th and 97.5th percentiles of the ordered 1000 values.

Analysts have the choice of using a Gaussian, log-Gaussian, Laplace or Log-Laplace likelihood function in DisMod-MR 2.1. We used the default log-Gaussian equation for the data likelihood, which is:

$$-log\left[p\left(y_j|\Phi\right)\right] = \log\left(\sqrt{2\pi}\right) + \log\left(\delta_j + s_j\right) + \frac{1}{2}\left(\frac{\log\left(a_j + \eta_j\right) - \log\left(m_j + \eta_j\right)}{\delta_j + s_j}\right)^2$$

where, y_j is a 'measurement value' (i.e., data point); Φ denotes all model random variables; η_jj is the offset value, eta, for a particular 'integrand' (prevalence, incidence, remission, excess mortality rate, cause- specific mortality rate) and a_j is the adjusted measurement for data point j, defined by:

$$a_j = e^{(-u_j - c_j)} y_j$$

where u_j is the total 'area effect' (i.e., the sum of the random effects at three levels of the cascade: super- region, region and country) and c_j is the total covariate effect (i.e., the mean combined fixed effects for sex, study level and country level covariates), defined by:

$$c_j = \sum_{k=0}^{K[I(j)]-1} \beta_{I(j),k} \hat{X}_{k,j}$$

with standard deviation

$$s_j = \sum_{l=0}^{L[I(j)]-1} \zeta_{I(j),l} \hat{Z}_{k,j}$$

where k denotes the mean value of each data point in relation to a covariate (also called x-covariate); $I(j)$ denotes a data point for a particular integrand, j; $\beta_{I(j),k}$ is the multiplier of the kth x-covariate for the ith integrand; $\hat{X}_{k,j}$ is the covariate value corresponding to the data point j for covariate k; l denotes the standard deviation of each data point in relation to a covariate (also called z-covariate); ζI(j),k is the multiplier of the lth z-covariate for the ith integrand; and δ_j is the standard deviation for adjusted measurement j, defined by:

$$\delta_j = log\left[y_j + e^{(-u_j - c_j)}\eta_j + c_j\right] - log\left[y_j + e^{(-u_j - c_j)}\eta_j\right]$$

Where m_j denotes the model for the jth measurement, not counting effects or measurement noise and defined by:

$$m_j = \frac{1}{B(j)-A(j)} \int_{A(j)}^{B(j)} I_j(\mathrm{a})\,\mathrm{da}$$

where $A(j)$ is the lower bound of the age range for a data point; $B(j)$ is the upper bound of the age range for a data point; and I_j denotes the function of age corresponding to the integrand for data point j.

The source code for DisMod-MR 2.1 as well as the wrapper code is available at the following link:

https://github.com/ihmeuw/ihmemodelling/tree/master/gbd_2017/shared_code/central_comp/nonfatal/dismod.

Appendix Table 6: Summary of covariates used in the LRI DisMod-MR meta-regression model

Covariate	Type	Parameter	Exponentiated beta (95% Uncertainty Interval)
Socio-demographic Index	Country-level	Prevalence	0.14 (0.14 to 0.14)
Healthcare Access and Quality index	Country-level	Excess mortality	0.37 (0.14 to 0.95)

We adjusted overall LRI incidence and prevalence estimates for 2020 and 2021 to account for the reductions in influenza and RSV mortality associated with the COVID-19 pandemic, as described on page 17 in this appendix.

Aetiology Estimation

Aetiologies Input Data

Input data for aetiology estimation consisted of multiple cause of death, vital registration, hospital discharge, and microbial data, as well as the PCV and Hib3 efficacy literature review shown in Appendix Figure 4, and a separate, targeted review pulling data from citations found in meta-analyses. For data sources that provided ICD codes (multiple cause of death, vital registration, hospital discharge, and some microbial data), these codes were used to identify patients with lower respiratory tract infections and the culprit pathogen, when detailed. For the microbial data that did not provide ICD codes, we identified pathogens associated with LRI based on the type of sample that was collected from the patient. Samples we deemed related to LRI included sputum, aspirates from the lower respiratory tract, and pleural fluid. We excluded samples from the eyes, ears, nose, or throat.

Appendix Table 7: ICD Codes Used in Aetiology Estimation

Type of LRI	ICD 10 code(s)	ICD9 code(s)
LRI due to Bordetella pertussis	A37-A37.9	033-033.9, 484.3
LRI due to *Legionella spp.*	A48.1-A48.2	--

LRI due to Actinomyces	--	039.1
LRI due to *Chlamydia spp.*	A70, J16.0, P23.1	073-073.9, 483.1, 484.2
LRI due to *Streptococcus pneumoniae*	J13-J13.9, J15.4, J20.2	481-481.9, 482.3
LRI due to *Haemophilus influenzae*	J14-J14.0, J20.1	482.2
LRI due to *Klebsiella pneumoniae*	J15.0	482.0
LRI due to *Pseudomonas spp.*	--	482.1
LRI due to *Pseudomonas aeruginosa*	J15.1, P23.5	--
LRI due to *Staphylococcus aureus*	J15.2, P23.2	482.4
LRI due to Group B Streptococcus	J15.3, P23.3	--
LRI due to *Escherichia coli*	J15.5, P23.4	--
LRI due to *Mycoplasma pneumoniae*	J15.7, J20.0	483.0
LRI due to *Francisella tularensis*	--	484.4
LRI due to *Bacillus anthracis*	--	484.5
LRI due to virus	--	079.6-079.7, 480-480.9, 484.0-484.1, 487-489
LRI due to Coronaviruses	B34.2, B97.2, J12.8	--
LRI due to Respiratory Syncytial Virus	B97.4, J12.1, J20.5, J21.0	--
LRI due to Influenza viruses	J09-J11.8	--
LRI due to Parainfluenza viruses	J12.2, J20.4	--
LRI due to Adenoviruses	J12.0	--
LRI due to Rhinoviruses	J20.6	--
LRI due to other virus	J12, J12.3, J12.9, J17.0, J17.2-J17.8, J20.3, J20.7-J20.8, J21.1	--

Data on pathogens cultured from human infections were solicited from a wide array of international stakeholders (representing every inhabited continent). These included research hospitals, surveillance networks, and infection databases maintained by private laboratories and medical technology companies. For a full list and details on the sources used for our estimates, please refer to the following article appendix (section 2 and section 6).[1]

Due to the documented challenge[7,8] in the microbiological identification of some LRI culprit pathogens, we supplemented these data with estimates of the PAF of pneumonia due to *Streptococcus pneumoniae* (pneumococcus), which was calculated based on vaccine efficacy data reported in 18 high-quality vaccine probe studies.

We conducted a systematic literature review of PCV efficacy studies until January 2020. For PCV studies, we extracted, if available, the distribution of S. pneumoniae *serotypes and the serotypes included in the PCV used in the study. Four new studies were identified for GBD 2021, which were all extracted only from PCV efficacy studies. PCV trial data are also frequently limited to younger age populations. To understand the contribution of* S. pneumoniae *in older populations, we also included PCV efficacy studies that used before-after approaches.*

Appendix Figure 6: Prisma Diagram of systematic review for PCV vaccine efficacy data

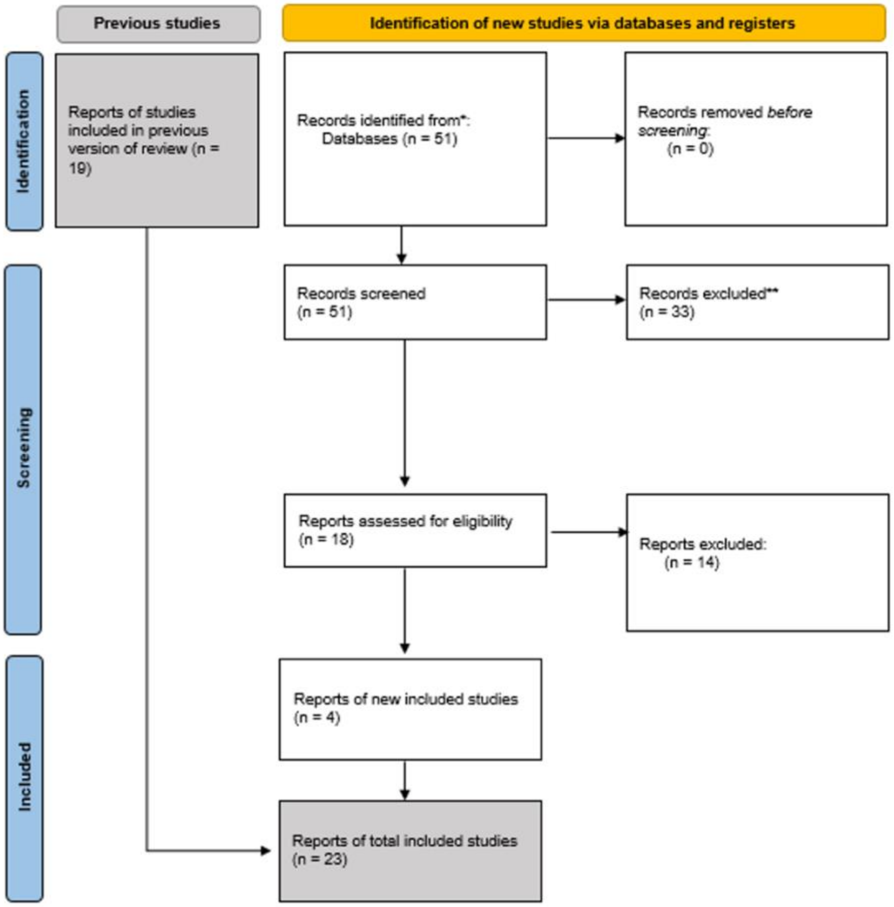

Appendix Figure 7: Non-fatal LRI aetiology site-years

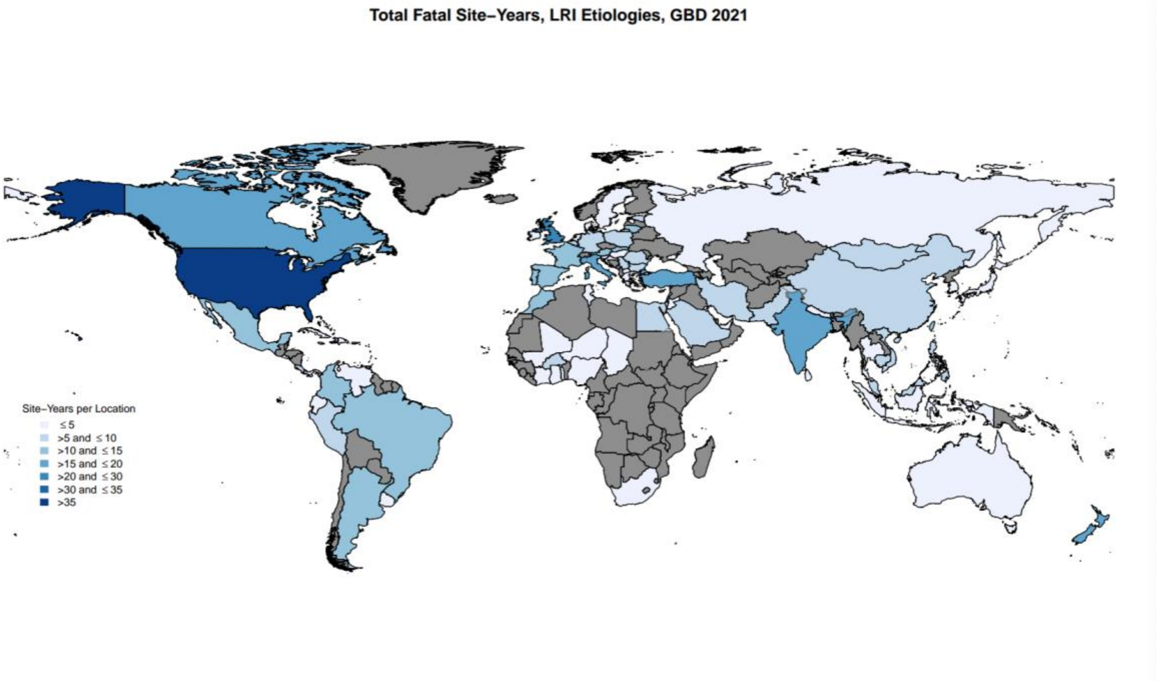

Appendix Figure 8: Fatal LRI aetiology site-years

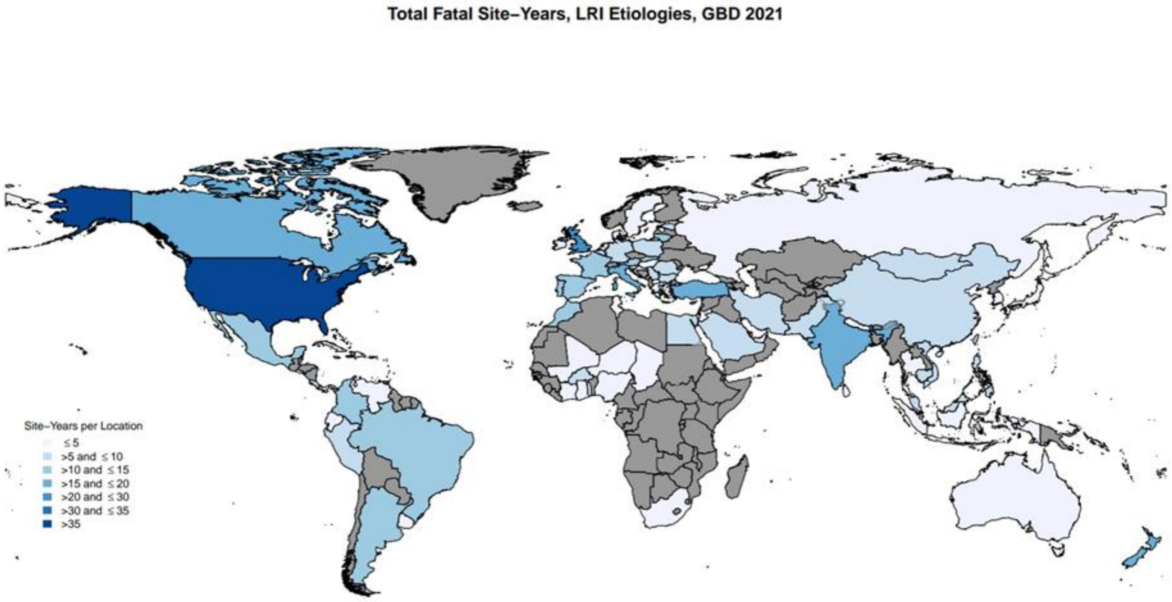

Nonfatal Aetiology Modelling Strategy

We estimated mutually-exclusive proportions of LRI cases attributable to the following set of pathogens: *Acinetobacter baumannii*, *Chlamydia* spp., *Enterobacter* spp., *Escherichia coli*, fungi, group B

Streptococcus, *Haemophilus influenzae*, influenza, *Klebsiella pneumoniae*, *Legionella* spp., *Mycoplasma* spp., polymicrobial infections, *Pseudomonas aeruginosa*, respiratory syncytial virus (RSV), *Staphylococcus aureus*, *Streptococcus pneumoniae*, and other viruses, as well as a residual, ‘other pathogen’ category. These proportions were estimated for five aggregate age groups: neonatal, post-neonatal to 5 years, 5 to 50 years, 50 to 70 years, and 70 years or older.

We estimated LRI aetiologies separately from overall LRI mortality and morbidity using two distinct counterfactual modeling strategies to estimate population attributable fractions (PAFs), described in detail below. The PAF represents the relative reduction in LRI mortality if there was no exposure to a given aetiology. We calculated uncertainty of our PAF estimates from 1000 draws of each parameter using normal distributions in log space.

Streptococcus pneumoniae

For *Streptococcus pneumoniae*, we calculated the population attributable fraction using a vaccine probe design[9] due to the documented challenge in the microbiological identification of this pathogen.[7,8] We then used these results as an input into the MEPCO pathogen distribution model. In a vaccine probe design, the ratio of vaccine efficacy against all pneumonia (non-pathogen specific) to vaccine-type, pathogen-specific disease represents the fraction of pneumonia cases attributable to each pathogen.

To estimate the PAF for *S. pneumoniae* pneumonia, we calculated study-level PAFs as the ratio of vaccine efficacy against all pneumonia to vaccine-type pathogen-specific pneumonia (Equation 1 & 2). For *S. pneumoniae* pneumonia, we used only the vaccine efficacy against vaccine-type *S. pneumoniae* pneumonia. This value was available in three studies and was calculated separately for children and adults, pooling the results of the Cutts[10] and Madhi[11] studies for children and using the Bonten[12] study for adults. Vaccine efficacy for all pneumonia was available at the study level. To estimate the PAF for *S. pneumoniae* pneumonia, we included RCTs and before and after vaccine introduction longitudinal studies.

For *S. pneumoniae* pneumonia, we adjusted the PAF by vaccine serotype coverage. Finally, we used an age distribution of PAF modelled in MR-BRT to determine the PAF by age. Because of an absence of data describing vaccine efficacy against Hib in children older than 2 years, we did not attribute Hib to episodes of LRI in ages 5 years and older.

We used a vaccine probe design to estimate the PAF for *S. pneumoniae* pneumonia and (Hib) by first calculating the ratio of vaccine efficacy against all pneumonia to pathogen-specific pneumonia at the study level (Equation 1).[1,13,14] We then adjusted this estimate by vaccine coverage and expected vaccine performance to estimate country- and year-specific PAF values (Equation 2).

$$1)\quad PneumoPAF_{Base} = \frac{VE_{all\_pneumonia}}{VE_{vt\_pneumococcal\_pneumonia} * Cov_{Serotype}}$$

$$2)\quad PAF_{Pneumo} = PneumoPAF_{Base} * \frac{(1 - Cov_{PCV} * VE_{all\_pneumonia})}{(1 - PneumoPAF_{Base} * Cov_{PCV} * VE_{all\_pneumonia})}$$

Where $VE_{all\_pneumonia}$ is the vaccine efficacy against non-specific pneumonia, $VE_{vt\_pneumococcal\_pneumonia}$ is the vaccine efficacy against vaccine-type *S. pneumoniae* pneumonia, $Cov_{serotype}$ is the serotype-specific vaccine coverage for PCV[15], and Cov_{PCV} is the PCV coverage.

We used the PAF_{Pneumo} as an input to our aetiology estimation model, described below, where it represented the proportion of LRI incidence attributable to *Streptococcus pneumoniae*. The remainder, $1 - PAF_{Pneumo}$, represented "non-pneumococcus" LRI, and was represented as a composite of all of the non- *Streptococcus pneumoniae* pathogens we estimated as well as the residual "other pathogens" category.

Other aetiologies

Aetiology proportions were calculated using an entirely new method from that applied in previous rounds of the GBD. Proportions were estimated as a function of age group, hospital/community-acquired infection, Hib and pneumococcal vaccination, and the Healthcare Access and Quality index (HAQi). These covariates vary across geography and time, creating unique predictions for each age group, location, and year. Working from the assumption that aetiologies would follow a multinomial distribution, we estimated aetiology fractions using a method previously described as multinomial estimation of partial and composite observations (MEPCO).[Error! Bookmark not defined.] Briefly, we constructed a network model with the dependent variable as the log ratio of cases between different pathogens.

Due to vastly different aetiology proportions among neonates relative to other ages, we estimated neonatal aetiologies separately. The model estimates both the proportions of hospital- and community-acquired LRI cases attributable to each aetiology. For the current GBD study, we report the distribution only amongst community-acquired disease as the pathogen distribution of LRI. This is because hospital-acquired infections occur with a non-LRI underlying cause, and they would therefore not be a part of the LRI envelope reported in the current study.

Appendix Table 8: Covariates used in aetiology modeling

Covariate	Model
Age group (neonatal, post-neonatal to 5, 5 to 50, 50 to 70, 70 plus)	Non-neonatal
Healthcare Access and Quality Index	Neonatal, Non-neonatal
Community vs. Hospital-acquired infection	Neonatal, Non-neonatal
Proportion of people who as infants were vaccinated with PCV	Non-neonatal
Proportion of population age 15 or younger vaccinated against pneumococcus	Neonatal, Non-neonatal
Proportion of people who as infants were vaccinated against *Haemophilus influenzae* type B	Non-neonatal
Proportion of population age 15 or younger vaccinated against *Haemophilus influenzae* type B	Neonatal, Non-neonatal

Due to inconsistencies in which pathogens are tested for and reported by different data sources, each data source contained partial observations of the possible outcomes of the underlying multinomial distribution. Certain data sources like the vaccine probe estimates represent compositional

observations, where pathogens like "not *S. pneumoniae*" represent aggregates of more detailed pathogens.

In order to use both partial and compositional data, we constructed a network model with the dependent variable as the log ratio of cases between different pathogens and estimated over a flexible parameterisation of multinomial parameters using a maximum likelihood approach. Consider a given infectious syndrome with a multinomial distribution of n mutually exclusive, collectively exhaustive aetiologies with probabilities $p = (p_1, \dots, p_n)$, so that each $p_j \in (0,1)$ and $\sum_j p_j = 1$. The likelihood of an observation of $c = (c_1, \dots, c_n)$, where c_j = number of cases of pathogen j in a total sample of N infections ($\sum_j c_j = N$), is:

$$P(c|p) = N! \prod_{j=1}^{n} \frac{p_j^{c_j}}{c_j!} \quad (1)$$

We modelled the probabilities using a composition of a link function with a linear predictor:

$$p_{i,j} = \exp\left(x_{i,j}^T \beta_j\right) \quad (2)$$

for observations i, a vector of covariates $x_{i,j}$, and a vector of coefficientsβ_j for each pathogen j. However, we did not observe these probabilities directly. Rather, we observed ratios between sums of these probabilities, which reduce to ratios between sums of cases within each study. These observations therefore take the form:

$$y_i = \frac{cases\ of\ pathogen\ A}{cases\ of\ pathogen\ B} = \frac{\sum_{j=1}^{n} w_{i,j}^a \exp\left(x_{i,j}^T \beta_j\right)}{\sum_{j=1}^{n} w_{i,j}^b \exp\left(x_{i,j}^T \beta_j\right)} \quad (3)$$

where $w_{i,j}^a$ is a weight of 0 or 1 that selects the mutually exclusive, collectively exhaustive most-detailed pathogens that make up observed pathogen A, which may be a composite observation. For example, for the "non *Streptococcus pneumoniae*" pathogen, $w_{i,j}$ would be 1 for *Acinetobacter baumannii*, *Chlamydia* spp., *Enterobacter* spp., *Escherichia coli*, fungi, group B *Streptococcus*, *Haemophilus influenzae*, influenza, *Klebsiella pneumoniae*, *Legionella* spp., *Mycoplasma* spp., polymicrobial infections, *Pseudomonas aeruginosa*, respiratory syncytial virus (RSV), *Staphylococcus aureus*, other viruses, and the residual, 'other pathogen' category and 0 for *Streptococcus pneumoniae*. We dropped all observations where either the numerator or denominator had 0 observed cases to make this calculation and a forthcoming log transform possible. This may bias the model towards overestimating less common pathogens.

It is not possible to infer all coefficients β_j from the observations, since they are all relative. However, if we fix all of the coefficients for one pathogen to 0 as a reference group, then we obtain a well-posed inverse problem, as long as there is enough data to estimate the remaining coefficients. Without loss of generality, we assumed $\beta_1 = 0$ for all elements and obtain estimates of the remaining $\beta_2, \dots, \beta_n$ by minimising the sum of the residuals between log-transformed observations y and corresponding log-transformed predictions from equation 3:

$$\min_{\beta_2,\dots,\beta_n} f(\beta) := \sum_i \frac{1}{\sigma_i^2}\left[\ln(y_i) - \ln\left(\sum_{j=1}^{n} w_{i,j}^{a}\, exp\left(x_{i,j}^{T}\beta_j\right)\right) + \ln\left(\sum_{j=1}^{n} w_{i,j}^{b}\, exp\left(x_{i,j}^{T}\beta_j\right)\right)\right]^2 \qquad (4)$$

where σ_i^2 are variances corresponding to the data points. Equation 4 is a nonlinear likelihood minimisation problem that that we optimised using a standard implementation of the Gauss-Newton method.[16] We then re-normalised the optimal coefficients to obtain final predictions of the probabilities of each pathogen:

$$p_{i,j} = \frac{\exp\left(x_{i,j}^{T}\beta_j\right)}{\sum_{\hat{j}} \exp\left(x_{i,\hat{j}}^{T}\beta_{\hat{j}}\right)} \qquad (5)$$

To quantify the uncertainty of this estimate, we used asymptotic statistics to obtain the posterior distribution of $(\beta_2, \dots, \beta_n)$. Specifically, using the Gauss-Newton Hessian approximation gave us the asymptotic information matrix for all β_j except for the reference pathogen, allowing us to sample draws of $\beta = (\beta_1 = 0, \beta_2, \dots, \beta_n)$. For each β draw and given feature x, we obtained a corresponding draw of p using equation 6.3.1.5.

This network regression with covariates framework allowed us to use partial and composite data that reported on one or only a few pathogens, or that reported multiple pathogens aggregated together. Networks, however, can be unstable with sparse data and stable estimates have in some cases required the use of Bayesian priors in these models. In particular, we imposed Gaussian priors with mean 0 and non-zero variance on all coefficients except intercepts, to bias the model away from spurious effects driven by data sparsity. For the neonatal model, a prior standard deviation of 0.2 was used. For the non-neonatal model, we used a standard deviation of 0.1. The standard deviation values of the priors were determined based on expert review and out-of-sample cross-validation.

Fatal Aetiology Modelling Strategy

To generate aetiology fraction estimates for fatal lower respiratory infections, we took our aetiology fractions estimated for nonfatal LRI and multiplied them by a set of pathogen-specific case fatality rates (CFRs). CFRs were estimated using ICD-coded hospital data and microbial data with patient discharge status using a cases-offset Poisson regression model.[17] We predicted CFRs as a function of pathogen, crude age (neonatal, post neonatal-5 years, 5-50 years, 50-70 years, and 70 years and older), an interaction term between pathogen and the proportions of the population age 15 or younger that had received PCV and *Haemophilus influenzae* type B vaccinations[18], Healthcare Access and Quality Index (HAQ Index), and bias covariates for data source (for the largest data sources). Separate models were run for CFRs associated with hospital-acquired and community-acquired LRI, and for the aetiology results reported here, only community-acquired CFRs were used. We additionally controlled for data provided from ICU-only sources (which would be biased towards higher CFRs) and data with "unknown" setting of infection origin (which was included in the community-acquired models to supplement input data). Of note, in using CFR data from a hospital setting, we assume that the ratio between the hospitalized CFRs for pathogen X and pathogen Y is the same as the ratio between the non-hospitalized CFRs for pathogens X and Y. CFRs in relation to one another drive estimation, rather than absolute CFR

values. This is the best available assumption given the sparsity of data concerning non-hospitalized patients.

The CFR model was run using the RegMod python package. The RegMod package implements and extends the Generalized Linear Modeling (GLM) framework. In particular, it allows:

User-specified likelihoods, capturing standard model family examples such as linear, Poisson, and binomial, as well as quasi-likelihoods, and other user-defined extensions.

- User-specified models for predicting parameters, based on link functions, covariates, and splines.
- Priors, constraints, and trimming.

We utilized a Poisson family model, encoding the number of deaths as our Y variable. The Poisson probability distribution takes the form

$$P(y_i|\lambda_i) = \frac{1}{y_i!}\exp(-\lambda_i)\,\lambda_i^{y_i} = \frac{1}{y_i!}\exp\left(-\lambda_i + y_i \log(\lambda_i)\right)$$

which suggest a parameterization

$$\log(\lambda_i) = c_i + \langle x_i, \beta\rangle.$$

Here, the link function is the exponential map, and $\langle x_i, \beta\rangle$ is a linear predictor that uses direct covariates. The quantity c_i is an offset, log(# of cases), which we use for observation-specific normalization of the number of cases, thereby allowing our model to estimate rates.

The negative log likelihood estimation problem for β becomes

$$\min_{\beta} \sum_i \exp\left(c_i + \langle x_i, \beta\rangle\right) - y_i(c_i + \langle x_i, \beta\rangle)$$

Where we can place constraints and priors on the β coefficients. The following priors were used:

- Prior on β for pathogen:vaccination interaction: We assumed vaccination would have no impact on CFRs of unrelated pathogens, and for all combinations of the pathogen:vaccination interaction that were not *Streptococcus pneumoniae*:PCV vaccination or *Haemophilus influenzae*:Hib vaccination we coerced the βs to 0 using model priors. For the *Streptococcus pneumoniae*:PCV vaccination and *Haemophilus influenzae*:Hib vaccination interaction terms, we employed a negativity prior to enforce case-fatality rates for these pathogens to decrease as vaccination was introduced.
- Prior on β for large data source dummy-variables: data source was included to account for source heterogeneity, however many input data sources covered only a single country, leading to low variability in HAQ Index within each data source. Such collinearity adversely influenced the accuracy of the estimated effect of HAQ Index, which was instrumental in extrapolating trends from the input data to global results. To emphasise the contribution of HAQ Index over data-source in the modelled estimates, we implemented a Gaussian prior (mean 0, standard error 0.1) on the βs for data source variables.[1]

Nonfatal pathogen proportions $p_{i,j}$ for a given demographic group i and pathogen j were then converted to deaths using the CFRs estimates for demographic group i as follows:

$$p_{i,j}^{deaths} = \frac{p_{i,j} \times CFR_i}{\sum_{\hat{j}} p_{i,\hat{j}} \times CFR_i}$$

We assumed inverse linear associations between the HAQ index and pathogen-specific CFRs, between PCV vaccination and *Streptococcus pneumoniae* CFR, and between Hib vaccination and *Haemophilus influenzae* CFR. We did not investigate other types of relationships, such as quadratic associations.

A separate, simplified model was used to estimate the case-fatality rate for "other bacteria." For this model, we withheld all non-bacterial pathogens from the input data and pooled the remaining pathogens together to get an all-bacteria-aggregate estimate.

We adjusted influenza and RSV mortality estimates for 2020 and 2021 to account for the reductions in influenza and RSV cases associated with the COVID-19 pandemic, as described below. A more thorough account of these methods, including model validation, has been described previously elsewhere.[1]

COVID adjustment

We reviewed national-level case notification data from ministry of health websites, media reports, and published literature for measles, pertussis, diphtheria, tetanus, varicella, diarrheal disease, influenza, respiratory syncytial virus, and infections due to *S. pneumoniae*, *H. influenzae*, and *N. meningitidis* to look for evidence of disruption. For measles and influenza, we relied on case notifications reported directly by countries to WHO regional offices; these causes had the most complete geographic and temporal coverage. Because of this completeness in reporting, we utilized them as indicator causes for further modelling, as described below. Only the influenza data were used for adjustments of LRI infections.

Modelling

We began by evaluating a select set of reportable infections for evidence of disruption. For each cause, to determine whether a disruption occurred in 2020, we conducted a random effect meta-analysis with restricted maximum likelihood estimation using the metafor package in R. Each point was the ratio of cases observed in 2020 to the cases observed over the average of 2017-2019. Given the relative completeness of influenza data, we developed a primary model for it and then, for infections other than influenza, evaluated whether the reduction modelled for influenza could be applied directly to the other infection. To do this, we examined the change in case notifications between 2020 and previous years for a cause relative to the change in case notifications between 2020 and previous years for influenza. When determining whether to adjust each cause, we considered the size and statistical significance of the observed effect, the consistency and quality of the available data, and epidemiological plausibility. At the time of estimation, these factors supported adjustment of only RSV, using estimates of disruption derived from the influenza disruption model results (see below). As we receive more data, we plan to reexamine additional causes and etiologies to apply disruption if warranted.

We developed a multi-step modelling process to estimate the effect of NPIs associated with the COVID-19 pandemic on the incidence of influenza and RSV in 2020 and 2021. First, we interpolated the number

of reported cases of influenza in 2020 and 2021, by month. We leveraged the RegMod framework, a Poisson model that estimates the underlying rate of infection in each month as a function of a seasonal pattern and an underlying temporal trend. The temporal trend was reflected as a piecewise linear spline with knots at the start of each year. We placed the last knot of the underlying time trend in January 2021 for influenza. We used monthly data through March 2022 (the last month of available data at the time of modeling) to fit the model, starting in January 2010 for influenza. The RegMod model results are 1000 sets of estimates of the number reported cases in each month and inputs to the next phase of modelling. We excluded from this modeling process any country missing at least 6 months of data in any year within 2017-2021 to reduce the risk of outbreaks occurring and subsiding during the periods of missing data.

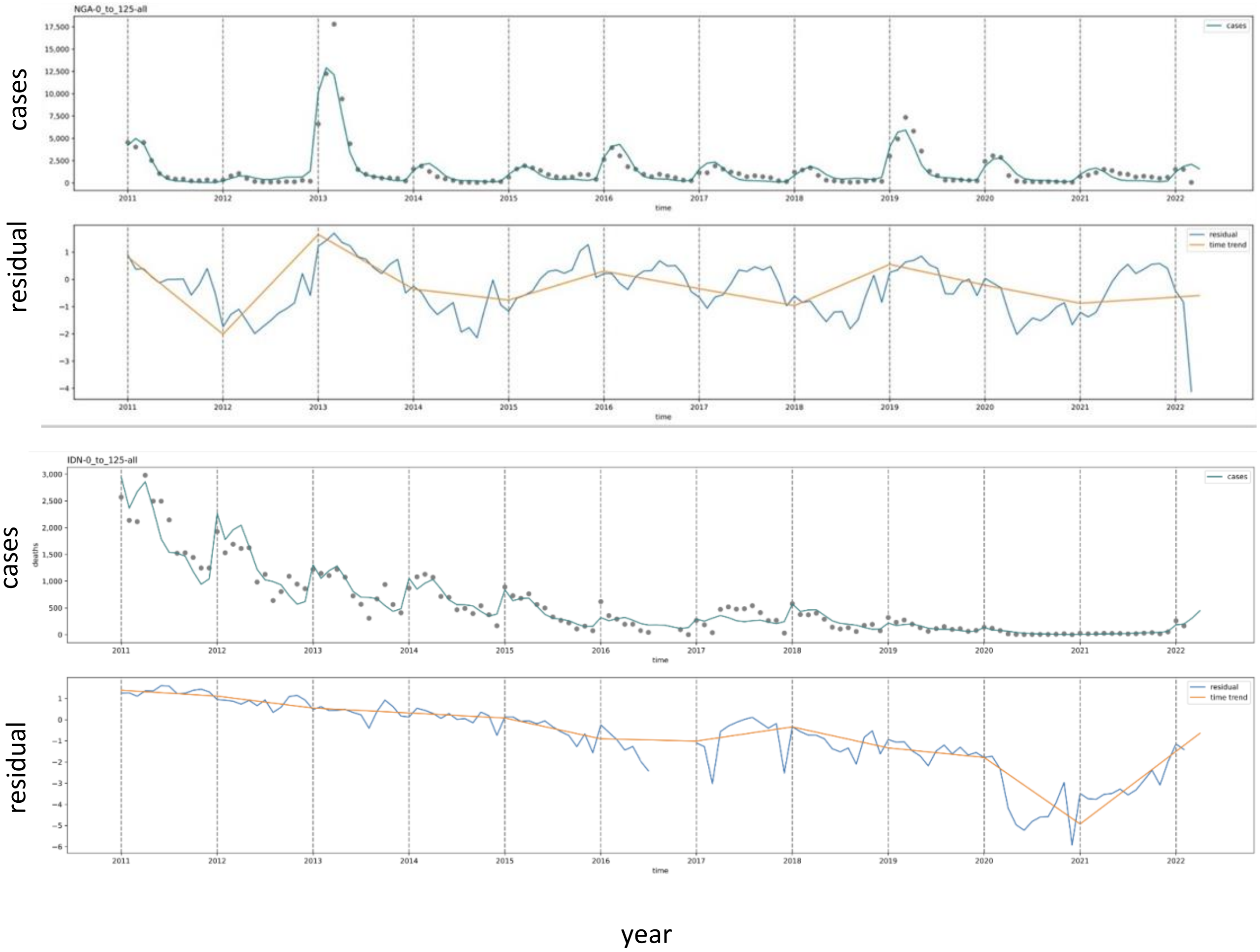

Appendix Figure 9: RegMod example for influenza in Indonesia. The top panel represents cases over time; points are the observed number of reported cases and line is the interpolated number of reported cases from the RegMod model. The bottom panel represents the residual over time and the time trend.

In the second step of the modelling process, we calculated the underreporting ratio (URR) in the pre-pandemic reference period 2017-2019, for each location, by dividing the interpolated number of reported cases from RegMod by the GBD estimated number of cases of LRI due to influenza. We used a reference period of 2017-2019 when calculating the URR. Third, we estimated the pandemic free

counterfactual number of reported cases, meaning, the number of reported cases we would have expected during 2020 and 2021 in the hypothetical pandemic-free scenario. We did this by multiplying the URR by the estimated number of cases of LRI due to influenza, for 2020 and 2021, that GBD models would have estimated in a pandemic-free scenario. Fourth, we calculated a disruption influenza scalar for each location for 2020 and 2021. This scalar was computed by dividing the interpolated number of reported cases from RegMod (result of first step) by the counterfactual disruption-free number of reported cases (result of third step). For countries with no data, the median disruption scalar in the region was used. All operations were performed at the 1000 draw level.

LRI Adjustment

We conducted a meta-analysis to compare location-specific disruptions for RSV to influenza. To inform the meta-analysis, we first created matched pairs of the percentage change in RSV to the percentage change in influenza by country with available data and calculated the ratio of these two percentage changes. More specifically, the ratio was computed by dividing the RSV percentage change in 2020 relative to the average from 2017–2019 by the corresponding influenza percentage change. We then conducted a meta-analysis to generate a pooled ratio of these percentage changes (1.41, 95% confidence interval 0.86 to 1.96), which was not statistically significant as the confidence interval includes 1. Consequently, we applied the influenza reduction percentages directly to RSV. For each location/age/sex for which LRI is estimated, influenza and RSV cases were scaled using the annualized ratios as calculated for influenza. Other aetiology-attributed cases of LRI were not scaled at this time.

Next, we calculated how the disruption scalars for influenza and RSV would apply to the overall LRI estimates. Because the etiological fraction of LRI due to RSV and influenza varies by age and sex, this calculation was performed by sex at the most granular age group level, for each country and year. It was also performed separately for deaths and cases since the etiological fraction of LRI due to RSV and influenza is different for deaths and cases. For a given country-year, the influenza disruption scalar was multiplied by the number of LRI influenza and RSV case/death counts, as pulled from GBD disruption-free counterfactual estimates, to get adjusted flu and RSV counts. GBD disruption-free counterfactual estimates are defined as the number of cases and deaths of LRI due to influenza and RSV that would be estimated by GBD models using standard methods[1] (as described under the Nonfatal Aetiology Modelling Strategy section in the methods appendix), run for 2020 and 2021, in the absence of any pandemic disruption adjustment or pandemic year data input. Then, we calculated the number of LRI cases/deaths to “remove” from the counterfactual number of LRI cases/deaths in the adjusted scenario as: the sum of counterfactual flu count and RSV count, minus the sum of COVID-adjusted flu count and RSV count. Finally, we calculate the LRI scalar for each country-age-sex-year as the LRI cases/deaths count from GBD counterfactual estimates, minus the number of LRI cases/deaths to “remove”, all divided by the counterfactual LRI cases/deaths count.

To adjust incidence and prevalence estimates for a given cause, we simply multiplied these estimates by the annual disruption ratio for that cause, calculated as described above. To adjust mortality estimates for a given cause, scalars are applied to an intermediate set of mortality results (counterfactual LRI death count) to create a count of LRI deaths to subtract using the formula below:

$$\text{LRI deaths to subtract} = (\text{Counterfactual LRI death count} * (\text{LRI scalar} - 1))$$

These values are subtracted from counterfactual LRI deaths to get adjusted LRI deaths. This operation is performed at the 1000 draw level for each location, age, sex, and year. This process is applied to final estimates the same way as other causes known in the GBD framework as fatal discontinuities.

Statement of GATHER Compliance

Appendix Table 9. Checklist of information that should be included in reports of global health estimates, with description of compliance and location of information the current study

#	GATHER checklist item	Description of compliance	Reference
**Objectives and funding**			
**1**	Define the indicator(s), populations (including age, sex, and geographic entities), and time period(s) for which estimates were made.	Narrative provided in paper and appendix describing indicators, definitions, populations, and time periods	Main text (Methods) and Appendix (Methods)
**2**	List the funding sources for the work.	Funding sources listed in paper	Summary (Funding)
**Data Inputs**			
For all data inputs from multiple sources that are synthesized as part of the study:			
**3**	Describe how the data were identified and how the data were accessed.	Narrative description of data seeking methods provided	Main text (Methods) and Appendix (Methods)
**4**	Specify the inclusion and exclusion criteria. Identify all ad-hoc exclusions.	Narrative about inclusion and exclusion criteria provided; ad hoc exclusions in appendix supplementary methods	Main text (Methods) and Appendix (Methods)
**5**	Provide information on all included data sources and their main characteristics. For each data source used, report reference information or contact name/institution, population represented, data collection method, year(s) of data collection, sex and age range, diagnostic criteria or measurement method, and sample size, as relevant.	An interactive, online data source tool that provides metadata for data sources by component, geography, cause, risk, or impairment has been developed, and data source citations provided	Appendix (Methods) with additional information about these sources available at https://ghdx.healthdata.org/record/ihme-data/global-burden-disease-study-2021-lower-respiratory-incidence-mortality-estimates-1990-2021
**6**	Identify and describe any categories of input data that have potentially important biases (e.g., based on characteristics listed in item 5).	Summary of known biases included in appendix supplementary methods	Appendix (Methods)
For data inputs that contribute to the analysis but were not synthesized as part of the study:			
**7**	Describe and give sources for any other data inputs.	Included in online data source tool	Global Health Data Exchange https://ghdx.healthdata.org/record/ihme-data/global-burden-disease-study-2021-lower-respiratory-incidence-mortality-estimates-1990-2021
For all data inputs:			
**8**	Provide all data inputs in a file format from which data can be efficiently extracted (e.g., a spreadsheet rather than a PDF), including all relevant meta-data listed in item 5. For any data inputs that cannot be shared because of ethical or legal reasons, such as third-party ownership, provide a contact name or the name of the institution that retains the right to the data.	Downloads of input data available through online data tools; input data not available in tools will be made available upon request	Global Health Data Exchange https://ghdx.healthdata.org/record/ihme-data/global-burden-disease-study-2021-lower-respiratory-incidence-mortality-estimates-1990-2021

**Data analysis**			
**9**	Provide a conceptual overview of the data analysis method. A diagram may be helpful.	Flow diagram of methodological process provided, as well as narrative descriptions of modelling process	Main text (Methods) and Appendix (Methods)
**10**	Provide a detailed description of all steps of the analysis, including mathematical formulae. This description should cover, as relevant, data cleaning, data pre-processing, data adjustments and weighting of data sources, and mathematical or statistical model(s).	Flow diagram and detailed methods write-up covering all data extraction, processing, and modelling processes provided	Main text (Methods) and Appendix (Methods)
**11**	Describe how candidate models were evaluated and how the final model(s) were selected.	Provided in methodological write-up	Appendix (Methods)
**12**	Provide the results of an evaluation of model performance, if done, as well as the results of any relevant sensitivity analysis.	Provided in methodological write-up	Appendix (Methods)
**13**	Describe methods for calculating uncertainty of the estimates. State which sources of uncertainty were, and were not, accounted for in the uncertainty analysis.	Provided in main text methods narrative description and appendix methodological writeup	Main text (Methods) and Appendix (Methods)
**14**	State how analytic or statistical source code used to generate estimates can be accessed.	Remote code repository for access to analytic code provided	Remote code repository https://ghdx.healthdata.org/record/ihme-data/global-burden-disease-study-2021-lower-respiratory-incidence-mortality-estimates-1990-2021
**Results and Discussion**			
**15**	Provide published estimates in a file format from which data can be efficiently extracted.	Tables in appendices and online results tool	Appendix Results and https://ghdx.healthdata.org/record/ihme-data/global-burden-disease-study-2021-lower-respiratory-incidence-mortality-estimates-1990-2021
**16**	Report a quantitative measure of the uncertainty of the estimates (e.g. uncertainty intervals).	Uncertainty provided with all results	Main text (Results), Appendix Results
**17**	Interpret results in light of existing evidence. If updating a previous set of estimates, describe the reasons for changes in estimates.	Discussion of results and methodological changes between GBD rounds provided in manuscript narrative and appendix	Main text (Methods, Results and Discussion) and Appendix (Methods)
**18**	Discuss limitations of the estimates. Include a discussion of any modelling assumptions or data limitations that affect interpretation of the estimates.	Discussion of limitations, including modelling assumptions and data limitations, included in manuscript narrative and appendix	Main text (Methods and Discussion) and Appendix (Methods)

Note: A full set of granular estimates can be found in the GBD Results Tool here, https://ghdx.healthdata.org/record/ihme-data/global-burden-disease-study-2021-lower-respiratory-incidence-mortality-estimates-1990-2021

References

1 Murray CJ, Ikuta KS, Sharara F, *et al.* Global burden of bacterial antimicrobial resistance in 2019: a systematic analysis. *The Lancet* 2022; **399**: 629–55.

2 Murray CJ, Ezzati M, Flaxman AD, *et al.* GBD 2010: design, definitions, and metrics. *The Lancet* 2012; **380**: 2063–6.

3 Schumacher AE, Kyu HH, Aali A, *et al.* Global age-sex-specific mortality, life expectancy, and population estimates in 204 countries and territories and 811 subnational locations, 1950–2021, and the impact of the COVID-19 pandemic: a comprehensive demographic analysis for the Global Burden of Disease Study 2021. *The Lancet* 2024; **0**. DOI:10.1016/S0140-6736(24)00476-8.

4 Johnson SC, Cunningham M, Dippenaar IN, *et al.* Public health utility of cause of death data: applying empirical algorithms to improve data quality. *BMC Med Inform Decis Mak* 2021; **21**: 175.

5 Vos T, Lim SS, Abbafati C, *et al.* Global burden of 369 diseases and injuries in 204 countries and territories, 1990–2019: a systematic analysis for the Global Burden of Disease Study 2019. *The Lancet* 2020; **396**: 1204–22.

6 Foreman KJ, Lozano R, Lopez AD, Murray CJ. Modeling causes of death: an integrated approach using CODEm. *Popul Health Metr* 2012; **10**: 1.

7 Ewig S, Schlochtermeier M, Göke N, Niederman MS. Applying sputum as a diagnostic tool in pneumonia: limited yield, minimal impact on treatment decisions. *Chest* 2002; **121**: 1486–92.

8 Ogawa H, Kitsios GD, Iwata M, Terasawa T. Sputum Gram Stain for Bacterial Pathogen Diagnosis in Community-acquired Pneumonia: A Systematic Review and Bayesian Meta-analysis of Diagnostic Accuracy and Yield. *Clin Infect Dis Off Publ Infect Dis Soc Am* 2020; **71**: 499–513.

9 Feikin DR, Scott JAG, Gessner BD. Use of vaccines as probes to define disease burden. *Lancet Lond Engl* 2014; **383**: 1762–70.

10 Cutts FT, Zaman SMA, Enwere G, *et al.* Efficacy of nine-valent pneumococcal conjugate vaccine against pneumonia and invasive pneumococcal disease in The Gambia: randomised, double-blind, placebo-controlled trial. *Lancet Lond Engl* 2005; **365**: 1139–46.

11 Madhi SA, Kuwanda L, Cutland C, Klugman KP. The impact of a 9-valent pneumococcal conjugate vaccine on the public health burden of pneumonia in HIV-infected and -uninfected children. *Clin Infect Dis Off Publ Infect Dis Soc Am* 2005; **40**: 1511–8.

12 Bonten MJM, Huijts SM, Bolkenbaas M, *et al.* Polysaccharide conjugate vaccine against pneumococcal pneumonia in adults. *N Engl J Med* 2015; **372**: 1114–25.

13 O'Brien KL, Wolfson LJ, Watt JP, *et al.* Burden of disease caused by Streptococcus pneumoniae in children younger than 5 years: global estimates. *Lancet Lond Engl* 2009; **374**: 893–902.

14 Watt JP, Wolfson LJ, O'Brien KL, *et al.* Burden of disease caused by Haemophilus influenzae type b in children younger than 5 years: global estimates. *Lancet Lond Engl* 2009; **374**: 903–11.

15 Johnson HL, Deloria-Knoll M, Levine OS, *et al.* Systematic evaluation of serotypes causing invasive pneumococcal disease among children under five: the pneumococcal global serotype project. *PLoS Med* 2010; **7**: e1000348.

16 Numerical Optimization. Springer New York, 2006 DOI:10.1007/978-0-387-40065-5.

17 Zheng P, Barber R, Sorensen RJD, Murray CJL, Aravkin AY. Trimmed Constrained Mixed Effects Models: Formulations and Algorithms. *J Comput Graph Stat* 2021; **30**: 544–56.

18 Galles NC, Liu PY, Updike RL, *et al.* Measuring routine childhood vaccination coverage in 204 countries and territories, 1980–2019: a systematic analysis for the Global Burden of Disease Study 2020, Release 1. *The Lancet* 2021; **398**: 503–21.

Authors' Contributions

Managing the overall research enterprise

Amanda Novotney, Peng Zheng, Aleksandr Y Aravkin, Theo Vos, Simon I Hay, Jonathan F Mosser, Stephen S Lim, Mohsen Naghavi, Christopher J L Murray, Hmwe Hmwe Kyu

Writing the first draft of the manuscript

Rose Grace Bender, Sarah Brooke Sirota, Lucien R Swetschinski, Regina-Mae Villanueva Dominguez, Hmwe Hmwe Kyu

Primary responsibility for applying analytical methods to produce estimates

Rose Grace Bender, Sarah Brooke Sirota, Lucien Swetschinski, Kevin S Ikuta, Emma Lynn Best Rogowski, Matthew C Doxey, Christopher E Troeger, Jianing Ma, Jiawei He, Kelsey Lynn Maass

Primary responsibility for seeking, cataloguing, extracting, or cleaning data; designing or coding figures and tables

Sarah Brooke Sirota, Regina-Mae Villaneuva Dominguez, Avina Vongpradith, Samuel B Albertson

Providing data or critical feedback on data sources

Meriem Abdoun, Jeza Muhamad Abdul Aziz, Salahdein Aburuz, Abiola Victor Adepoju, Rishan Adha, Antonella Agodi, Ayman Ahmed, Haroon Ahmed, Karolina Akinosoglou, Mohammed Albashtawy, Mohammad T. AlBataineh, Hediyeh Alemi, Abid Ali, Syed Shujait Shujait Ali, Edward Kwabena Ameyaw, John H Amuasi, Reza Arefnezhad, Ahmed Y Azzam, Stephen Baker, Martina Barchitta, Ravi Batra, Nebiyou Simegnew Bayileyegn, Apostolos Beloukas, Rose Grace Bender, James A Berkley, Julia A Bielicki, Katrin Burkart, Vijay Kumar Chattu, Hitesh Chopra, Eunice Chung, Xiaochen Dai, Lalit Dandona, Rakhi Dandona, Denise Myriam Dekker, Vinoth Gnana Chellaiyan Devanbu, Thao Huynh Phuong Do, Klara Georgieva Dokova, Christiane Dolecek, Regina-Mae Villanueva Dominguez, Aziz Eftekharimehrabad, David William Eyre, Alireza Feizkhah, Santosh Gaihre, Brhane Gebremariam, Kazem Ghaffari, Pouya Goleij, Mesay Dechasa Gudeta, Wase Benti Hailu, Arvin Haj-Mirzaian, Sebastian Haller, Mohammad Hamiduzzaman, Jan Hansel, Johannes Haubold, Simon I Hay, Nguyen Quoc Hoan, Hong-Han Huynh, Mahsa Jalili, Charity Ehimwenma Joshua, Md. Awal Kabir, Zul Kamal, Rami S. Kantar, Harkiran Kaur, Faham Khamesipour, M Nuruzzaman Khan, Mahammed Ziauddin Khan suheb, Khaled Khatab, Mahalaqua Nazli Khatib, Grace Kim, Kewal Krishan, Ralf Krumkamp, Hmwe Hmwe Kyu, Chandrakant Lahariya, Kaveh Latifinaibin, Nhi Huu Hanh Le, Thao Thi Thu Le, Trang Diep Thanh Le, Seung Won Lee, Stephen S Lim, Kashish Malhotra, Tauqeer Hussain Mallhi, Anand Manoharan, Bernardo Alfonso Martinez-Guerra, Alexander G. Mathioudakis, Rita Mattiello, Jürgen May, Barney McManigal, Le Huu Nhat Minh, Awoke Misganaw, Arup Kumar Misra, Mustapha Mohammed, Ali H Mokdad, Lorenzo Monasta, Jonathan F. Mosser, Vincent Mougin, Francesk Mulita, Christopher J L Murray, Mohsen Naghavi, Ganesh R Naik, Shumaila Nargus, Dang H Nguyen, Van Thanh Nguyen, Hau Thi Hien Nguyen, Taxiarchis Konstantinos Nikolouzakis, Amanda Novotney, Ismail A. Odetokun, Edgar Ortiz-Brizuela, Amel Ouyahia, Jagadish Rao Padubidri, Anton Pak, Anamika Pandey, Romil R Parikh, Ashwaghosha Parthasarathi, Prince Peprah, Hoang Tran Pham, Alfredo Ponce-De-Leon, Peralam Yegneswaran Prakash, Elton Junio Sady Prates, Nguyen Khoi Quan, Fakher Rahim, Shakthi Kumaran Ramasamy, Shubham Ranjan, Ahmed Mustafa Rashid, Sayaphet Rattanavong, Nakul Ravikumar, Luis Felipe Reyes, Tamalee Roberts, Mónica Rodrigues, Victor Daniel Rosenthal, Priyanka Roy, Tilleye Runghien, Umar Saeed, Narjes

Saheb Sharif-Askari, Joseph W Sakshaug, Afeez Abolarinwa Salami, Malik Sallam, Sara Samadzadeh, Sunder Sham, Rajesh P. Shastry, Aminu Shittu, Sarah Brooke Sirota, Andy Stergachis, Temenuga Zhekova Stoeva, Chandan Kumar Swain, Lukasz Szarpak, Mohamad-Hani Temsah, Pugazhenthan Thangaraju, Ngoc-Ha Tran, Christopher E Troeger, Munkhtuya Tumurkhuu, Sree Sudha Ty, Tungki Pratama Umar, Jef Van den Eynde, Avina Vongpradith, Theo Vos, Judd L Walson, Galal Yahya, Dong Keon Yon, Chunxia Zhai, Magdalena Zielińska.

Developing methods or computational machinery

Jeza Muhamad Abdul Aziz, Aleksandr Y Aravkin, Ahmed Y Azzam, Rose Grace Bender, Eunice Chung, Xiaochen Dai, Kazem Ghaffari, Shi-Yang Guan, Simon I Hay, Hong-Han Huynh, Kevin S Ikuta, Mahsa Jalili, Md. Awal Kabir, M Nuruzzaman Khan, Mahalaqua Nazli Khatib, Nhi Huu Hanh Le, Thao Thi Thu Le, Kelsey Lynn Maass, Le Huu Nhat Minh, Ali H Mokdad, Francesk Mulita, Christopher J L Murray, Mohsen Naghavi, Dang H Nguyen, Van Thanh Nguyen, Amanda Novotney, Michal Ordak, Hoang Tran Pham, Mónica Rodrigues, Emma Lynn Best Rogowski, Victor Daniel Rosenthal, Umar Saeed, Austin E Schumacher, Sarah Brooke Sirota, Reed J D Sorensen, Chandan Kumar Swain, Lucien R Swetschinski, Christopher E Troeger, Theo Vos, Dong Keon Yon, Peng Zheng

Providing critical feedback on methods or results

Meriem Abdoun, Jeza Muhamad Abdul Aziz, Deldar Morad Abdulah, Samir Abu Rumeileh, Hasan Abualruz, Salahdein Aburuz, Abiola Victor Adepoju, Rishan Adha, Wirawan Adikusuma, Saryia Adra, Shahin Aghamiri, Antonella Agodi, Amir Mahmoud Ahmadzade, Ayman Ahmed, Haroon Ahmed, Karolina Akinosoglou, Rasmieh Mustafa Al-amer, Mohammed Albashtawy, Mohammad T. AlBataineh, Hediyeh Alemi, Adel Ali Saeed Al-Gheethi, Abid Ali, Syed Shujait Shujait Ali, Jaber S Alqahtani, Mohammad AlQudah, Jaffar A. Al-Tawfiq, Yaser Mohammed Al-Worafi, Karem H Alzoubi, Reza Amani, Prince M Amegbor, Edward Kwabena Ameyaw, John H Amuasi, Philip Emeka Anyanwu, Mosab Arafat, Damelash Areda, Kendalem Asmare Atalell, Firayad Ayele, Ahmed Y Azzam, Hassan Babamohamadi, Yogesh Bahurupi, Biswajit Banik, Martina Barchitta, Hiba Jawdat Barqawi, Zarrin Basharat, Pritish Baskaran, Kavita Batra, Ravi Batra, Nebiyou Simegnew Bayileyegn, Apostolos Beloukas, Rose Grace Bender, Ashish Bhargava, Priyadarshini Bhattacharjee, Julia A Bielicki, Mariah Malak Bilalaga, Veera R Bitra, Colin Stewart Brown, Katrin Burkart, Yasser Bustanji, Yaacoub Chahine, Vijay Kumar Chattu, Hitesh Chopra, Isaac Sunday Chukwu, Eunice Chung, Xiaochen Dai, Lalit Dandona, Rakhi Dandona, Isaac Darban, Nihar Ranjan Dash, Mohsen Dashti, Mohadese Dashtkoohi, Ivan Delgado-Enciso, Vinoth Gnana Chellaiyan Devanbu, Kuldeep Dhama, Nancy Diao, Thao Huynh Phuong Do, Klara Georgieva Dokova, Christiane Dolecek, Arkadiusz Marian Dziedzic, Abdelaziz Ed-Dra, Ferry Efendi, Aziz Eftekharimehrabad, Ayesha Fahim, Alireza Feizkhah, Timothy William Felton, Luisa S Flor, Santosh Gaihre, Miglas W Gebregergis, Mesfin Gebrehiwot, Brhane Gebremariam, Urge Gerema, Kazem Ghaffari, Shi-Yang Guan, Mesay Dechasa Gudeta, Cui Guo, Veer Bala Gupta, Farrokh Habibzadeh, Najah R Hadi, Wase Benti Hailu, Ramtin Hajibeygi, Arvin Haj-Mirzaian, Sebastian Haller, Mohammad Hamiduzzaman, Md Saquib Hasnain, Johannes Haubold, Simon I Hay, Nguyen Quoc Hoan, Hong-Han Huynh, Kevin S Ikuta, Kenneth Chukwuemeka Iregbu, Md. Rabiul Islam, Ammar Abdulrahman Jairoun, Mahsa Jalili, Nabi Jomehzadeh, Charity Ehimwenma Joshua, Md. Awal Kabir, Zul Kamal, Kehinde Kazeem Kanmodi, Rami S. Kantar, Arman Karimi Behnagh, Navjot Kaur, Harkiran Kaur, Faham Khamesipour, M Nuruzzaman Khan,

Mahammed Ziauddin Khan suheb, Vishnu Khanal, Khaled Khatab, Mahalaqua Nazli Khatib, Grace Kim, Kwanghyun Kim, Aiggan Tamene Tamene Kitila, Somayeh Komaki, Kewal Krishan, Md Abdul Kuddus, Maria Dyah Kurniasari, Hmwe Hmwe Kyu, Chandrakant Lahariya, Kaveh Latifinaibin, Nhi Huu Hanh Le, Thao Thi Thu Le, Trang Diep Thanh Le, Seung Won Lee, Temesgen L. Lerango, Ming-Chieh Li, Stephen S Lim, Amir Ali Mahboobipour, Kashish Malhotra, Tauqeer Hussain Mallhi, Bernardo Alfonso Martinez-Guerra, Alexander G. Mathioudakis, Sazan Qadir Maulud, Steven M McPhail, Tesfahun Mekene Meto, Max Alberto Mendez Mendez-Lopez, Sultan Ayoub Meo, Mohsen Merati, Tomislav Mestrovic, Laurette Mhlanga, Le Huu Nhat Minh, Awoke Misganaw, Vinaytosh Mishra, Arup Kumar Misra, Nouh Saad Mohamed, Esmaeil Mohammadi, Mustapha Mohammed, Mesud Mohammed, Ali H Mokdad, Catrin E Moore, Jonathan F. Mosser, Rohith Motappa, Francesk Mulita, Atsedemariam, Andualem Mulu, Christopher J L Murray, Pirouz Naghavi, Ganesh R Naik, Firzan Nainu, Tapas Sadasivan Nair, Shumaila Nargus, Mohammad Negaresh, Dang H Nguyen, Van Thanh Nguyen, Taxiarchis Konstantinos Nikolouzakis, Efaq Ali Noman, Amanda Novotney, Chisom Adaobi Nri-Ezedi, Ismail A. Odetokun, Matifan Dereje Olana, Omotola O. Olasupo, Antonio Olivas-Martinez, Michal Ordak, Edgar Ortiz-Brizuela, Amel Ouyahia, Jagadish Rao Padubidri, Anton Pak, Anamika Pandey, Ioannis Pantazopoulos, Pragyan Paramita Parija, Romil R Parikh, Seoyeon Park, Ashwaghosha Parthasarathi, Ava Pashaei, Prince Peprah, Hoang Tran Pham, Dimitri Poddighe, Peralam Yegneswaran Prakash, Elton Junio Sady Prates, Nguyen Khoi Quan, Pourya Raee, Fakher Rahim, Mosiur Rahman, Masoud Rahmati, Shakthi Kumaran Ramasamy, Shubham Ranjan, Indu Ramachandra Rao, Ahmed Mustafa Rashid, Murali Mohan Rama Krishna Reddy, Elrashdy Moustafa Mohamed Redwan, Robert C Reiner Jr., Luis Felipe Reyes, Mónica Rodrigues, Emma Lynn Best Rogowski, Victor Daniel Rosenthal, Priyanka Roy, Umar Saeed, Amene Saghazadeh, Narjes Saheb Sharif-Askari, Fatemeh Saheb Sharif-Askari, Soumya Swaroop Sahoo, Joseph W Sakshaug, Afeez Abolarinwa Salami, Mohamed A. Saleh, Hossein Salehi omran, Malik Sallam, Sara Samadzadeh, Yoseph Leonardo Samodra, Rama Krishna Sanjeev, Benn Sartorius, Jennifer Saulam, Austin E Schumacher, Seyed Arsalan Seyedi, Mahan Shafie, Samiah Shahid, Muhammad Aaqib Shamim, Mohammad Ali Shamshirgaran, Rajesh P. Shastry, Samendra P Sherchan, Desalegn Shiferaw, Aminu Shittu, Emmanuel Edwar Siddig, Robert Sinto, Sarah Brooke Sirota, Aayushi Sood, Reed J D Sorensen, Chandan Kumar Swain, Lucien R Swetschinski, Lukasz Szarpak, Jacques Lukenze Tamuzi, Mohamad-Hani Temsah, Melkamu B Tessema Tessema, Pugazhenthan Thangaraju, Nghia Minh Tran, Ngoc-Ha Tran, Christopher E Troeger, Munkhtuya Tumurkhuu, Sree Sudha Ty, Aniefiok John Udoakang, Inam Ulhaq, Tungki Pratama Umar, Abdurezak Adem Umer, Seyed Mohammad Vahabi, Asokan Govindaraj Vaithinathan, Jef Van den Eynde, Theo Vos, Judd L Walson, Muhammad Waqas, Yuhan Xing, Mukesh Kumar Yadav, Galal Yahya, Dong Keon Yon, Abed Zahedi Bialvaei, Fathiah Zakham, Abyalew Mamuye Zeleke, Chunxia Zhai, Haijun Zhang, Zhaofeng Zhang, Magdalena Zielińska.

Drafting the work or revising it critically for important intellectual content

Jeza Muhamad Abdul Aziz, Samir Abu Rumeileh, Hasan Abualruz, Salahdein Aburuz, Rishan Adha, Saryia Adra, Ali Afraz, Antonella Agodi, Ayman Ahmed, Haroon Ahmed, Tareq Mohammed Ali AL-Ahdal, Rasmieh Mustafa Al-amer, Mohammed Albashtawy, Mohammad T. AlBataineh, Hediyeh Alemi, Abid Ali, Syed Shujait Shujait Ali, Jaber S Alqahtani, Mohammad AlQudah, Jaffar A. Al-Tawfiq, Yaser Mohammed Al-Worafi, Karem H Alzoubi, Reza Amani, Prince M Amegbor, Abhishek Anil, Philip Emeka Anyanwu, Mosab Arafat, Damelash Areda, Kendalem Asmare Atalell, Firayad Ayele, Ahmed Y Azzam, Yogesh Bahurupi, Martina Barchitta, Hiba Jawdat Barqawi, Pritish Baskaran, Apostolos Beloukas, Rose Grace Bender, James A Berkley, Kebede A Beyene, Ashish Bhargava, Priyadarshini Bhattacharjee, Mariah Malak

Bilalaga, Veera R Bitra, Colin Stewart Brown, Yasser Bustanji, Sinclair Carr, Yaacoub Chahine, Vijay Kumar Chattu, Fatemeh Chichagi, Hitesh Chopra, Sriharsha Dadana, Nihar Ranjan Dash, Mohsen Dashti, Mohadese Dashtkoohi, Ivan Delgado-Enciso, Nancy Diao, Regina-Mae Villanueva Dominguez, Arkadiusz Marian Dziedzic, Abdelaziz Ed-Dra, Aziz Eftekharimehrabad, Ayesha Fahim, Timothy William Felton, Nuno Ferreira, Santosh Gaihre, Miglas W Gebregergis, Brhane Gebremariam, Urge Gerema, Kazem Ghaffari, Mohamad Goldust, Shi-Yang Guan, Mesay Dechasa Gudeta, Cui Guo, Veer Bala Gupta, Farrokh Habibzadeh, Najah R Hadi, Emily Haeuser, Wase Benti Hailu, Ramtin Hajibeygi, Arvin Haj-Mirzaian, Mohammad Hamiduzzaman, Nasrin Hanifi, Jan Hansel, Md Saquib Hasnain, Johannes Haubold, Simon I Hay, Nguyen Quoc Hoan, Hong-Han Huynh, Kenneth Chukwuemeka Iregbu, Md. Rabiul Islam, Abdollah Jafarzadeh, Mahsa Jalili, Nabi Jomehzadeh, Charity Ehimwenma Joshua, Md. Awal Kabir, Zul Kamal, Kehinde Kazeem Kanmodi, Rami S. Kantar, Navjot Kaur, M Nuruzzaman Khan, Mahammed Ziauddin Khan suheb, Vishnu Khanal, Khaled Khatab, Mahalaqua Nazli Khatib, Grace Kim, Aiggan Tamene Tamene Kitila, Somayeh Komaki, Kewal Krishan, Md Abdul Kuddus, Hmwe Hmwe Kyu, Chandrakant Lahariya, Kaveh Latifinaibin, Nhi Huu Hanh Le, Thao Thi Thu Le, Kashish Malhotra, Tauqeer Hussain Mallhi, Bernardo Alfonso Martinez-Guerra, Alexander G. Mathioudakis, Sazan Qadir Maulud, Jürgen May, Steven M McPhail, Tesfahun Mekene Meto, Max Alberto Mendez Mendez-Lopez, Sultan Ayoub Meo, Mohsen Merati, Tomislav Mestrovic, Le Huu Nhat Minh, Awoke Misganaw, Nouh Saad Mohamed, Esmaeil Mohammadi, Mustapha Mohammed, Mesud Mohammed, Ali H Mokdad, Lorenzo Monasta, Catrin E Moore, Rohith Motappa, Parsa Mousavi, Atsedemariam, Andualem Mulu, Christopher J L Murray, Mohsen Naghavi, Firzan Nainu, Shumaila Nargus, Mohammad Negaresh, Dang H Nguyen, Van Thanh Nguyen, Hau Thi Hien Nguyen, Taxiarchis Konstantinos Nikolouzakis, Amanda Novotney, Chisom Adaobi Nri-Ezedi, Ismail A. Odetokun, Patrick Godwin Okwute, Matifan Dereje Olana, Titilope O Olanipekun, Antonio Olivas-Martinez, Michal Ordak, Edgar Ortiz-Brizuela, Jagadish Rao Padubidri, Anton Pak, Ioannis Pantazopoulos, Pragyan Paramita Parija, Romil R Parikh, Ashwaghosha Parthasarathi, Ava Pashaei, Hoang Tran Pham, Dimitri Poddighe, Peralam Yegneswaran Prakash, Elton Junio Sady Prates, Nguyen Khoi Quan, Fakher Rahim, Masoud Rahmati, Shakthi Kumaran Ramasamy, Shubham Ranjan, Ahmed Mustafa Rashid, Nakul Ravikumar, Elrashdy Moustafa Mohamed Redwan, Luis Felipe Reyes, Mónica Rodrigues, Emma Lynn Best Rogowski, Victor Daniel Rosenthal, Umar Saeed, Fatemeh Saheb Sharif-Askari, Soumya Swaroop Sahoo, Monalisha Sahu, Joseph W Sakshaug, Afeez Abolarinwa Salami, Hossein Salehi omran, Malik Sallam, Sara Samadzadeh, Made Ary Sarasmita, Aswini Saravanan, Mahan Shafie, Samiah Shahid, Muhammad Aaqib Shamim, Rajesh P. Shastry, Aminu Shittu, Emmanuel Edwar Siddig, Robert Sinto, Sarah Brooke Sirota, Aayushi Sood, Chandan Kumar Swain, Lucien R Swetschinski, Lukasz Szarpak, Jacques Lukenze Tamuzi, Mohamad-Hani Temsah, Pugazhenthan Thangaraju, Nghia Minh Tran, Ngoc-Ha Tran, Sree Sudha Ty, Aniefiok John Udoakang, Tungki Pratama Umar, Abdurezak Adem Umer, Asokan Govindaraj Vaithinathan, Jef Van den Eynde, Mukesh Kumar Yadav, Galal Yahya, Dong Keon Yon, Abed Zahedi Bialvaei, Haijun Zhang, Magdalena Zielińska.

Managing the estimation or publications process

Jeza Muhamad Abdul Aziz, Ahmed Y Azzam, Rose Grace Bender, Ayesha Fahim, Kazem Ghaffari, Simon I Hay, Hong-Han Huynh, Mahsa Jalili, M Nuruzzaman Khan, Mahalaqua Nazli Khatib, Hmwe Hmwe Kyu, Chandrakant Lahariya, Nhi Huu Hanh Le, Thao Thi Thu Le, Le Huu Nhat Minh, Ali H Mokdad, Atsedemariam, Andualem Mulu, Christopher J L Murray, Mohsen Naghavi, Van Thanh Nguyen, Amanda Novotney, Hoang Tran Pham, Mónica Rodrigues, Sarah Brooke Sirota, Lucien R Swetschinski, Eve E Wool.
